# Origin and Deep Evolution of Human Endogenous Retroviruses in Pan-Primates

**DOI:** 10.3390/v14071370

**Published:** 2022-06-23

**Authors:** Yian Li, Guojie Zhang, Jie Cui

**Affiliations:** 1 CAS Key Laboratory of Molecular Virology & Immunology, Institute Pasteur of Shanghai, Center for Biosafety Mega-Science, Chinese Academy of Sciences, Shanghai 200031, China; yali@ips.ac.cn; 2 University of Chinese Academy of Sciences, Beijing 100049, China; 3 Section for Ecology and Evolution, Department of Biology, University of Copenhagen, DK-1353 Copenhagen, Denmark; guojie.zhang@bio.ku.dk; 4 State Key Laboratory of Genetic Resources and Evolution, Kunming Institute of Zoology, Chinese Academy of Sciences, Kunming 650201, China

**Keywords:** human endogenous retroviruses, origin, evolution, vertical transmission, genomic rearrangement, pan-primates

## Abstract

Human endogenous retroviruses (HERVs) are viral “fossils” in the human genome that originated from the ancient integration of exogenous retroviruses. Although HERVs have sporadically been reported in nonhuman primate genomes, their deep origination in pan-primates remains to be explored. Hence, based on the in silico genomic mining of full-length HERVs in 49 primates, we performed the largest systematic survey to date of the distribution, phylogeny, and functional predictions of HERVs. Most importantly, we obtained conclusive evidence of nonhuman origin for most contemporary HERVs. We found that various supergroups, including HERVW9, HUERSP, HSERVIII, HERVIPADP, HERVK, and HERVHF, were widely distributed in *Strepsirrhini*, *Platyrrhini* (New World monkeys) and *Catarrhini* (Old World monkeys and apes). We found that numerous HERVHFs are spread by vertical transmission within *Catarrhini* and one HERVHF was traced in 17 species, indicating its ancient nature. We also discovered that 164 HERVs were likely involved in genomic rearrangement and 107 HERVs were potentially coopted in the form of noncoding RNAs (ncRNAs) in humans. In summary, we provided comprehensive data on the deep origination of modern HERVs in pan-primates.

## 1. Introduction

Human endogenous retroviruses (HERVs) are retroviral remnants in the human genome derived from the ancient integration of exogenous retroviruses and occupy approximately 8% of the human genomic DNA [1,2]. HERVs are mainly formed via two major mechanisms: (1) horizontal transmission, in which exogenous retroviral RNA is integrated into the host genome, thus becoming a provirus that will produce infectious virus; (2) vertical transmission, in which the past retroviral infection of germline cells results in a provirus with Mendelian heritability [1,3].

A typical HERV contains two long terminal repeats (LTRs) and four major genes: *gag* (containing matrix (MA), capsid (CA) and nucleocapsid (NC) domains), *pro* (containing protease (PR) and dUTPase (DU) domains), *pol* (containing reverse transcriptase (RT), RNAse H (RH) and integrase (IN) domains) and *env* (containing surface (SU) and transmembrane (TM) domains) [4,5]. Since the first HERV was identified in the 1980s [6], over 3000 classifiable HERVs have been identified, and they can be divided into 3 classes (Class I, Class II and Class III) and 11 supergroups based on the phylogeny of *pol* genes: Class I: MLLV, HERVERI, HERVFRDLIKE, HEPSI, HUERSP, HERVW9, HERVIPADP, MER50like, and HERVHF; Class II: HERVK/HML; and Class III: HSERVIII [7,8].

Some evidence has shown that HERVs also appear in nonhuman primates [9,10,11]. Furthermore, LTR dating indicated that some HERVs could have entered the genomes of primate ancestors 25 million years ago (MYA) [12], although such dating results should be considered cautiously [9]. To decode the deep origination of HERVs, we performed the systematically in silico mining survey of HERVs in 49 primate genomes covering all major families of the order Primates. Based on these extensive data, we present the full landscape of evolutionary pathways leading to the generation of modern HERVs.

## 2. Materials and Methods

### 2.1. Genome Screening and Identification of HERVs

For the strict identification of full-length HERVs, we first used the RT (reverse transcriptase) sequences from the human genome available in the gEVE database [13] and performed TBLASTN searches [14] to screen the 49 primate genomes with a cutoff e-value of 0.00001. All genome assemblies were accessed from National Center for Biotechnology Information (NCBI) Assembly Database (https://www.ncbi.nlm.nih.gov/assembly/ accessed on 30 November 2021) and the Sequence Read Archive (SRA) Database (https://www.ncbi.nlm.nih.gov/sra/ accessed on 30 November 2021) under an accessible BioProject accession code: PRJNA785018. Then, the data generated from the TBLASTN analysis were transformed into gene transfer format (GTF), and repetitive data were removed according to the contig names, start positions, and end positions. BEDTools [15] was employed to merge locations with distances of less than 1000 base pairs (bps).

To reduce false positive results, the merged sequences were extracted, and DIAMOND BLASTX searches [16] were performed against all primate (taxonomy ID 9443) alone sequences and viruses (taxonomy ID 10239) in the Reference Sequence (RefSeq) database [17] with an e-value of 0.00001. The best alignment results were extracted and screened based on query names and bit scores, and phylogenetic analysis (see below for details) was performed with reference RT sequences of HERVs from a previous publication [18] to confirm whether the screened results represented true RT domains of HERVs. The recognized sequences were extended to a length of 10,000 bp from both ends for further LTR (long terminal repeat) identification.

LTR-Harvest [19] with the default parameters was utilized to determine the boundaries of each LTR of HERVs. The internal sequences were searched against HERV proteins in the gEVE database by using DIAMOND BLASTX with an e-value of 0.00001. The results were transformed into browser extensible data (BED) files, merged, and extracted as previously described. Another round of DIAMOND BLASTX searches was performed to ascertain the nature of HERV genes. The results were manually checked, genomic fragments were merged based on the orders and locations in primate genomes to reduce repeatability, and only translations of at least 100 amino acids in the length of HERV genes were retained. For classification, phylogenetic reconstruction was performed using *pol* sequences aligned by MAFFT [20] with the parameter “--auto”, and the alignments were trimmed by trimAl [21] with the parameter “-gt 0.1” or “-gt 0.5”. IQ-TREE2 [22] was applied to construct the maximum likelihood (ML) trees with the parameters “-B 1000 -alrt 1000”.

### 2.2. Vertical Transmission Identification

To identify vertical transmission events, BLASTN searches [14] were performed with the full-length HERVs with both flanking regions (~2000 bp in length). Hits that met the following three conditions were extracted: (1) two HERV sequences showed 90% coverage and identity; (2) two HERV LTR-flanking sequences were matched with an identity over 90%; and (3) the BLASTN results of both LTR-flanking sequences showed over 25% coverage, with at least one result showing 80% coverage. Only candidate HERVs that simultaneously met all three conditions were selected for further analysis. Vertical transmission-associated paired HERVs were analyzed with the igraph package [23], and vertical transmission events were estimated based on the species that contained paired HERVs. The species tree of primates was generated using TimeTree [24], and the bubble pie chart was created for visualization with the scatterpie package [25].

### 2.3. Genomic Rearrangement Analysis

Homologous recombination (i.e., between two similar HERVs in different genomic locations in a given species) leading to genomic rearrangement might have occurred during primate genome evolution [26]. We first attempted to detect the rearrangement signals by performing phylogenetic LTR reconstructions according to different HERV categories and primate species based on the 5′ and 3′LTR sequences of full-length HERVs. We collected sequences that did not cluster in pairs (i.e., the 5′LTR and 3′LTR of a single HERV) in the phylogenetic trees. We next tested their coverage and identity by performing BLASTN searches with the same query and subject. We selected the paired LTRs with higher bit scores from different HERV sources (e.g., the bit score of 5′ LTR-1 vs. 5′ LTR-2 was higher than that of 5′ LTR-1 vs. 3′ LTR-1). Then, we checked whether these paired LTRs matched other LTRs (e.g., 5′LTR-1 matched 5′/3′LTR-2 and 3′LTR-1 matched 3′/5′LTR-2). We reasoned that the HERVs with matching LTRs may be subjected to genomic rearrangement.

### 2.4. HERVs-Derived ncRNA Verification in the Human Genome

We employed the coordinates of all known human transcripts from Ensembl database version 104 and ncRNAs [27] from the NONCODE database [28] in Genome Reference Consortium Human Build 38 (GRCh38/hg38) and retained those that intersected with the coordinates of HERVs in the human genome by using BEDTools with the parameter “intersect -wo -s”. We only selected the results for which the coverage of at least one feature in a pair of features was equal to 100% and predicted the possible HERV-derived ncRNA molecules based on these results.

## 3. Results

### 3.1. HERVs Are Widely Dispersed in the Genomes of Old World Monkeys and Apes

**Figure 1:**
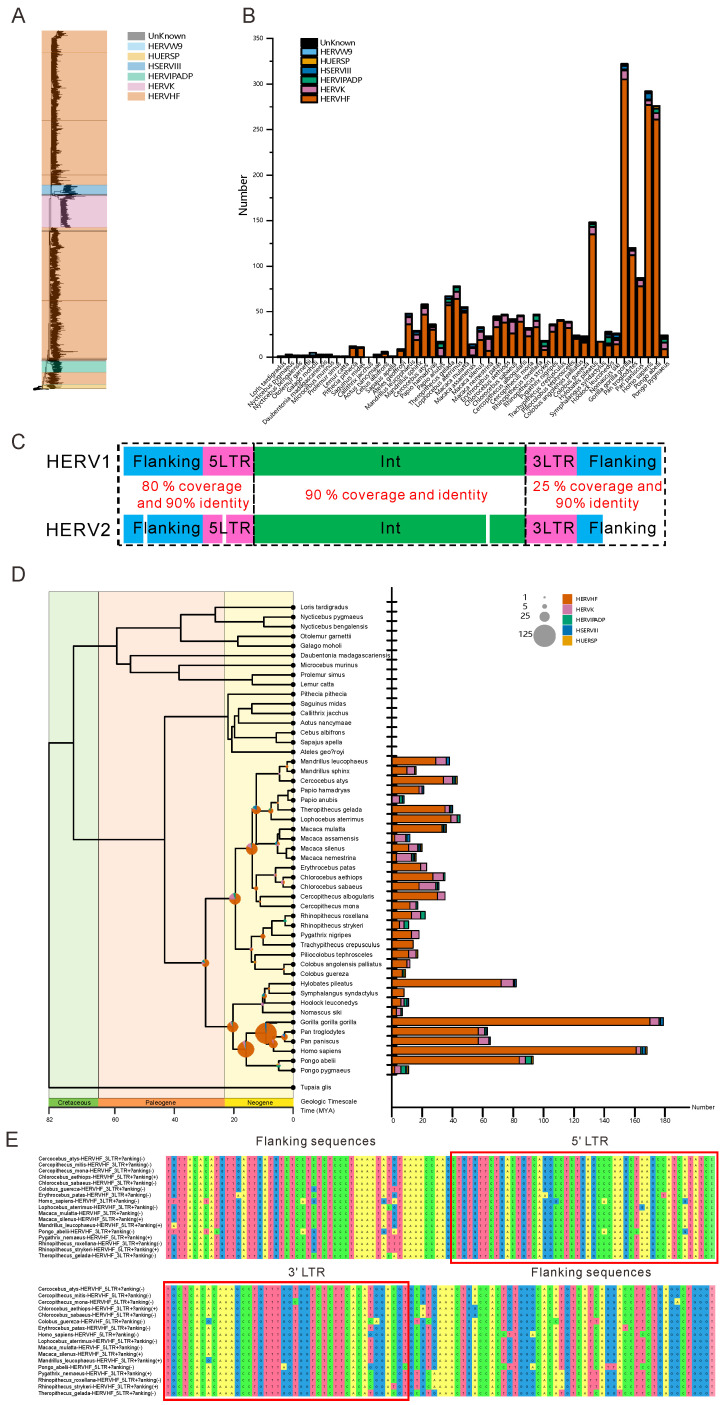
The distribution, classification, and vertical transmission of F-HERVs in 49 primates. (**A**) The phylogenetic tree of all F-HERVs identified. The classifications of F-HERVs are indicated in different colors, and “Unknown” represents F-HERVs that cannot be classified according to their phylogenetic relationships with reference sequences or the similarity of their RT domains with reference RT sequences. Ultrafast bootstrap approximation (UFBoot) values over 95 are provided beside the nodes. (**B**) Histogram showing the number of F-HERVs in each species, and the classifications of HERVs are indicated in different colors. (**C**) The diagram shows the standards that were used to screen vertical transmission events. The blue boxes, pink boxes, and green boxes represent the flanking sequences (Flanking), LTR sequences (5LTR or 3LTR), and internal sequences (Int) of F-HERVs, respectively. The dashed boxes indicate the BLASTN searches we performed and the thresholds of each search (in red). The white segments represent the mismatch or gap in BLASTN. The detailed procedure of vertical transmission identification is provided in the Materials and Methods. (**D**) The left panel shows the rooted phylogenetic tree and the divergence times of 50 primates (*Tupaia glis* was used as the root), and the numbers of possible vertical transmission events are indicated on the corresponding nodes by the area of pies. The right panel shows the numbers of vertical transmission-related F-HERVs in each species we studied. The classifications of the F-HERVs are indicated in different colors. (**E**) The image shows the alignments of the 5′LTR and flanking sequences (upper) and the 3′LTR and flanking sequences (lower) of a widely vertically transmitted F-HERV-H in 17 species. The names of the species are listed on the left, and red boxes indicate the LTR sequences of F-HERVH. The full alignments of the LTR and flanking sequences are provided in the Supplementary Materials.

To identify HERVs, we first analyzed the genomes of 49 species of primates to identify the RT domains of reference HERVs because the RT domains of HERVs are often used to distinguish HERVs and other retroviruses [18,29,30]. Briefly, we performed a first round of TBLASTN analysis to search the HERV RT domains of primate genomes, and a second round of DIAMOND BLASTX analysis was then performed to exclude those RT domains that were better aligned with host proteins or other viral proteins. Next, we performed phylogenetic analysis to verify whether these RT sequences belonged to HERVs. We extended the length of the verified sequences and identified their LTR boundaries. We subsequently estimated the internal sequences between two LTRs with DIAMOND BLASTX, and only sequences that contained RT domains and showed the correct ordering of other genes (e.g., *gag*-*pro*-*pol*-*env*) were identified as classifiable HERVs, and hence defined as “full-length HERVs” (F-HERVs). We reconstructed the *pol* gene of each HERV and performed phylogenetic analysis to classify the HERVs (Figure 1A). The HERVs were classified according to their phylogenetic relationships with reference sequences and the similarity of their RT domains with reference RT sequences.

In total, we identified 2301 classifiable F-HERV copies (Figure 1A,B & Supplementary Data S2), with most of them found in *Catarrhini*, (Figure 1B). The limited numbers identified in this study reflected our rigorous search methodology and the limitations of using full-length HERVs. The greatest number of the identified F-HERVs belonged to the HERVHF supergroup, whose members reportedly integrated into *Catarrhini* genomes at least 30–45 million years ago [31,32,33,34]. It is worth noting that we found HERVH sequences not only in the species in which they have been reported previously (*Homo sapiens*, *Gorilla gorilla gorilla*, *Pongo abelii*, *Papio anubis*, *Chlorocebus aethiops*, *Callithrix jacchus*, *Pan troglodytes*, *Nomascus siki* and *Aotus nancymaae*) but also in some new genera of *Catarrhini*, such as *Mandrillus*, *Rhinopithecus,* and *Colobus* (Figure 1B), further confirming the widespread and ancient nature of HERVHF. We also found other types of F-HERVs, such as HERVW9, HERVIPADP, HERVK, and HSERVIII members (Figure 1B), in primates, which was consistent with previous studies [35,36,37,38] but with hosts expanded in this study. Together, these results demonstrated that F-HERVs are ancient, and humans inherited such elements via vertical transmission from nonhuman primates.

### 3.2. Numerous HERVHFs Are Spread by Vertical Transmission within Catarrhini

If vertical transmission events occurred, the sequences of the two viruses and their flanking sequences should be the same in different primate genomes. However, over a long evolutionary history, many mutations accumulate in HERVs, which makes it difficult to identify vertical transmission events. Therefore, we set the following strict criteria for identifying possible vertical transmission events: (1) two HERVs must show high identity and coverage; (2) the flanking sequences of the two HERVs must show high identity; and (3) at least one of the flanking sequences must show high coverage (Figure 1C).

In total, we discovered 1226 F-HERVs that may participate in vertical transmission and identified 408 vertical transmission events (Figure 1D). All of the vertical transmission events were identified within *Catarrhini*, and more than half of these (222 of 408) were found in apes. According to HERV classification, most of the vertically transmitted F-HERVs belonged to the HERVHF group, which was consistent with the distribution of HERVs (Figure 1B). Interestingly, we found that several F-HERVs may have infiltrated the common ancestor of Old-World monkeys and apes, including 10 HERVHF, 2 HERVK, 1 HERVIPADP, 1 HSERVIII, and 1 HUERSP members (Figure 1D). Strikingly, one HERVHF was vertically transmitted from Old World monkeys to apes, and the pathway of its vertical transmission was traced in 17 species (Figure 1E & Supplementary Data S4–S6). In addition, we estimated the time of F-HERV integration based on the time tree of these 49 primates and the vertical transmission events detected within them (Figure 1D). We speculated that detectable vertical transmission of F-HERVs occurred from 0 to 29.4 MYA and that nearly 25% of vertical transmission events (118 in 468) occurred at 9.1 MYA, when *Gorilla gorilla gorilla* separated from *Homo sapiens*.

### 3.3. Some F-HERVs May Be Involved in Genomic Rearrangement

HERVs are not only molecular ‘fossils’ of ancient retroviruses but are also functional in host genomes under certain circumstances [39,40,41]. One of the functions of HERVs is mediating host genomic recombination, leading to potential genomic rearrangement [26,42,43]. When a HERV is integrated into a host genome, the two LTRs of that one element should be more similar to each other than to the LTRs of any other element, although they accumulate mutations after integration and residence in the germ line [26]. Therefore, if a HERV has two similar but different LTRs, genomic recombination may occur within that HERV.

**Figure 2:**
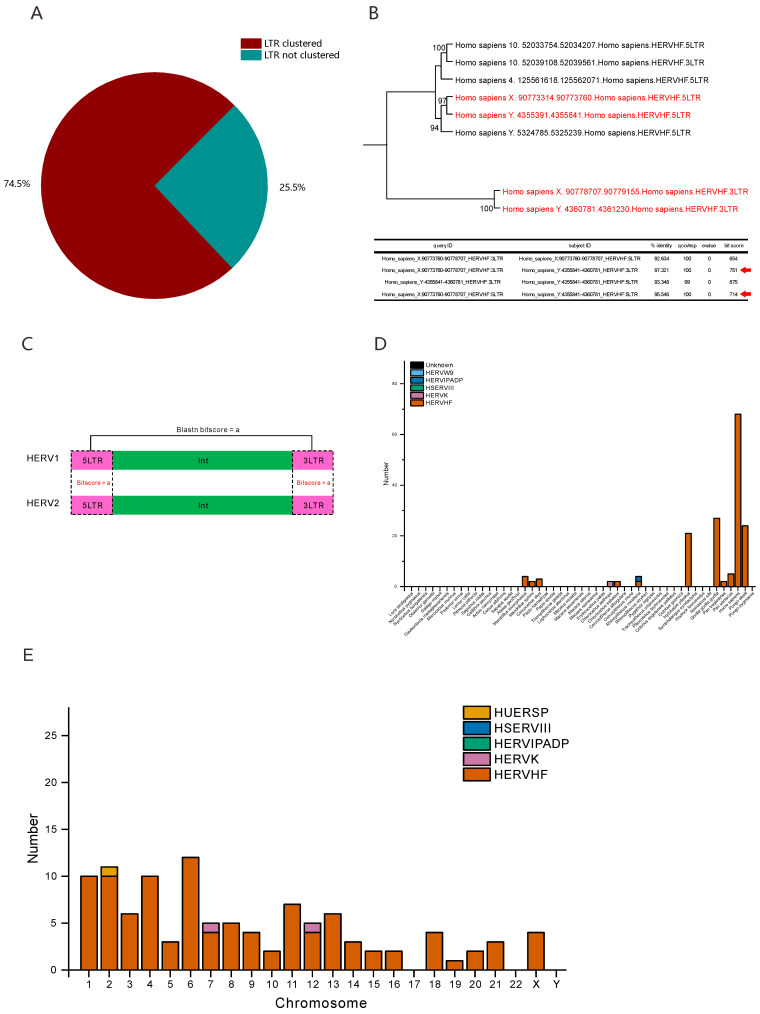
F-HERVs may be involved in host genomic recombination or transcribed into ncRNAs. (**A**) The pie chart shows the number of F-HERVs with clustered LTRs (dark red) and HERVs with non-clustered LTRs (blue green). (**B**) The phylogenetic tree (upper) shows an example of LTR separation in one F-HERV-H of humans. The separation of the two LTRs is indicated in red, and ultrafast bootstrap approximation (UFBoot) values over 95 are provided beside the nodes. The BLASTN results of these two pairs of LTRs are shown in the table (lower) together with the BLASTN results. “qcovhsp” indicates the coverage of the query, and the red arrows indicate better alignments. The detailed alignments are shown in the Supplementary Data. (**C**) The diagram shows the standards used to screen F-HERV-related host genomic recombination events. The pink boxes and green boxes represent the LTR sequences (5LTR or 3LTR) or internal sequences (int) of F-HERVs, respectively. The dashed boxes show the BLASTN searches that we performed and the thresholds of each search (in red). The details of the genomic rearrangement analysis are provided in the Materials and Methods. (**D**) Histogram showing the number of recombination-related F-HERVs in each species, and the classifications of the F-HERVs are indicated in different colors. “Unknown” represents F-HERVs that cannot be classified according to their phylogenetic relationships with reference sequences or the similarity of their RT domains with reference RT sequences. (**E**) Histogram showing the number of F-HERVs that share the same locations and orientations with known ncRNAs on each human chromosome. The classifications of the F-HERVs are indicated in different colors.

We predicted F-HERVs that may be involved in host genomic recombination based on this hypothesis and found that 25.5% (586 of 2301) of F-HERVs had a pair of nonclustered LTRs, indicating that these F-HERVs may be related to host genomic recombination (Figure 2A). We performed BLASTN searches to identify the “mismatches” of LTRs (LTRs showing better alignment with other HERV LTRs) (Figure 2B,C) and counted the number of different types of recombination-related HERVs in each species (Figure 2D & Supplementary Data S7–S9). The results showed that most of the recombination-related F-HERVs (147 of 164) located in the genomes of apes belonged to the HERVHF group (Figure 2D), which was consistent with the total distribution of F-HERVs (Figure 1B). Overall, our results suggested that some of the F-HERVs that we identified were associated with the recombination of primate genomes.

### 3.4. Some F-HERVs in Human Genomes Are Likely Transcribed into ncRNAs

Another function of HERVs may involve their transcription into ncRNAs that then regulate host genes [44,45,46]. Because the location information of human ncRNAs has been well annotated in human genomes [28], we used our HERV coordinates to merge the known human ncRNAs. We attempted to identify ncRNAs derived from F-HERVs or ncRNAs containing F-HERVs. We finally identified 107 F-HERVs and ncRNAs that showed the same locations and orientations (Table 1 and Supplementary Data S10). We calculated the statistics of the F-HERV distribution and classification on each human chromosome and found that most of the ncRNA-correlated F-HERVs belonged to the HERVHF group (104 of 107), and the three the human chromosomes that possessed the greatest numbers of ncRNA-correlated F-HERVs were chromosomes 6, 2 and 1, with 12, 11 and 10 of these F-HERVs, respectively (Figure 2E). In short, these data showed that some F-HERVs in human genomes may be involved in evolutionary co-option with primates and function in the form of ncRNAs.

## 4. Discussion

In the past 30 years, many HERVs have been identified in human genomes, but there has been little systematic research on HERVs in other nonhuman primates. In this study, we used all known HERV sequences to determine the classifiable HERVs in 49 species of primates and annotated their specific loci in the host genomes (Supplementary Data S2). We only discovered 292 F-HERVs in humans, which was much lower than the number indicated by previous research [8,47]. One major reason for this difference was that we used only the RT sequences of HERVs in the human genomes available from gEVE, rather than using exogenous retroviral *pol* sequences to search HERVs, because we were focused on tracing the origin of different types of HERVs in human genomes and the RT domain is the most conserved domain that can be used to distinguish retroviruses [29,48]. Indeed, many HERVs accumulate mutations or are even lost during long-term evolution, and phylogenetic analysis based on these proteins sometimes cannot rebuild their phylogenetic relationships. Although we identified fewer F-HERVs in the human genome through our pipeline, these F-HERVs showed a relatively intact genomic structure and covered 5 superclasses of HERVs (Figure 1A,B). In addition, further investigation revealed that some of these F-HERVs were involved in vertical transmission. Thus, these F-HERVs could help us to effectively pursue the origin of HERVs.

Vertical transmission events of HERVs provide strong evidence that could indicate the origin of HERVs. One of the most ancient HERVs (HERVLs) reported to date, which integrated into an ancestor of all extant placental mammals at more than 100 MYA, was identified based on this line of reasoning [49]. We found that most vertical transmission-related F-HERVs in human genomes were derived from those present in *Hominidae*, and the others came from *Hylobates* and Old World monkeys (Supplementary Data S3). We estimated that the integration times of these F-HERVs, which ranged from 9.1 MYA to 29.4 MYA (Figure 1C and Supplementary Data S3) depended on the time of separation, and this result was consistent with previous reports [50,51]. Vertical transmission events spanning long periods are difficult to track because of the strict definition of vertical transmission, which requires very high sequence similarity and coverage of HERVs and their flanking sequences. Mutations in HERVs show a positive correlation with time, and we were, therefore, unable to identify vertical transmission that may have occurred in the ancestors of NWM or *Strepsirrhini*. Another reason for the unsuccessful detection of vertical transmission was that the total number of F-HERVs found in the first step was small. If we were to consider the HERVs that have lost their RT domains, different results might be obtained.

HERVs are capable of causing homologous recombination due to their high sequence similarities. Many studies have analyzed HERV-related gene recombination by comparing the genomes of different individuals [52,53,54]. However, the available genomes from the different individuals of the same species are insufficient. We assume that homologous recombination takes place between two HERVs of the same type (e.g., HERVHF) and that they share highly similar sequences but show differences in their LTRs. When homologous recombination occurs, such HERVs will exchange their internal sequences, leading to a pair of analogous but different LTRs. We conjecture that homologous recombination takes place on the basis of this assumption (Figure 2B,C & Supplementary Data S7), and the results should be treated with caution because recombination of endogenous retroviruses which had microhomologic sequences has also been reported in other mammals [55].

Recently, HERVs have been reported to be associated with many human diseases, including cancer and infectious and autoimmune diseases, and the mechanisms underlying the functions of HERVs in these illnesses also vary (e.g., acting as promoters or enhancers to regulate gene expression or encoding peptides that participate in immune regulation) [39,40,56,57,58]. Therefore, it is important to consider HERVs that have the potential to be expressed. To identify potentially expressed F-HERVs in humans, we intersected the coordinates of all human F-HERVs and all known transcripts from other public databases and found that some HERVs may be transcribed into ncRNAs and functional under certain conditions, such as viral infections [40]. We also performed searches of F-HERVs from other nonhuman primates in the RefSeq database and the nucleotide sequence (nt) database of NCBI with BLASTN, and we did not find any credible transcripts strongly related to these HERVs. However, some of these F-HERVs showed high similarities with F-HERVs from humans, so we surmised they may have homologous functions.

In conclusion, we traced the F-HERVs present in the human genome back to nonhuman primates and found that some HERVs originated before the speciation of Hominidae, Hylobates, and Old-World monkeys. In addition, some of the F-HERVs that we identified were possibly functional from the perspective of genomics or transcriptomics, likely indicating long-term co-option. Together, these findings could help us to better understand the deep origin and evolution of modern HERVs.

## Figures and Tables

**Table 1:** The annotation of HERV related ncRNAs in human.

Chromosome	Start	End	HERVname	Strand	Related-ncRNA
1	22997488	23002547	Homo_sapiens_1_23000272-23002212-HERVHF	−	NONHSAG057580.1
1	43087974	43091095	Homo_sapiens_1_43087972-43089561-HERVHF	+	NONHSAG056389.1
1	68386003	68391994	Homo_sapiens_1_68388791-68390135-HERVHF	+	NONHSAG001773.3
1	82354581	82360561	Homo_sapiens_1_82356924-82357985-HERVHF	−	ENSG00000233290
1	82955299	82961592	Homo_sapiens_1_82956772-82959208-HERVHF	+	ENSG00000230817
1	209451675	209454012	Homo_sapiens_1_209451456-209452546-HERVHF	+	NONHSAG057153.1
1	224840009	224846095	Homo_sapiens_1_224842067-224843662-HERVHF	−	ENSG00000286719
1	228942542	228942868	Homo_sapiens_1_228948757-228950104-HERVHF	+	NONHSAG057288.1
1	232120241	232123943	Homo_sapiens_1_232120704-232121847-HERVHF	+	NONHSAG004630.2
1	241433890	241439885	Homo_sapiens_1_241436435-241438237-HERVHF	+	ENSG00000287516
2	5000768	5003355	Homo_sapiens_2_5003505-5005037-HERVHF	+	NONHSAG077068.1
2	16791950	16797713	Homo_sapiens_2_16793801-16795145-HERVHF	−	NONHSAG078497.1
2	34789818	34796058	Homo_sapiens_2_34792231-34793755-HERVHF	+	NONHSAG077257.1
2	38080800	38086513	Homo_sapiens_2_38082627-38083973-HERVHF	−	ENSG00000138061
2	67333734	67337603	Homo_sapiens_2_67334137-67335887-HERVHF	+	NONHSAG028042.3
2	69789900	69795859	Homo_sapiens_2_69792965-69796021-HUERSP	−	NONHSAG028084.2
2	77965137	77970868	Homo_sapiens_2_77967627-77968976-HERVHF	+	NONHSAG077496.1
2	192506078	192513184	Homo_sapiens_2_192509946-192511342-HERVHF	+	NONHSAG030125.2
2	215922303	215928129	Homo_sapiens_2_215924899-215926434-HERVHF	+	NONHSAG078151.1
2	224225331	224230988	Homo_sapiens_2_224227814-224229160-HERVHF	+	NONHSAG078214.1
2	237606784	237611630	Homo_sapiens_2_237609479-237610820-HERVHF	+	NONHSAG110040.1
3	21189031	21194139	Homo_sapiens_3_21190643-21192440-HERVHF	−	ENSG00000282987
3	54634482	54638068	Homo_sapiens_3_54636349-54637755-HERVHF	−	ENSG00000265992
3	112418410	112423366	Homo_sapiens_3_112419312-112420768-HERVHF	−	NONHSAG035734.2
3	115798715	115799166	Homo_sapiens_3_115795176-115796709-HERVHF	−	NONHSAG085690.1
3	155274423	155278762	Homo_sapiens_3_155276457-155278448-HERVHF	−	NONHSAG036456.2
3	186660747	186663692	Homo_sapiens_3_186660542-186661888-HERVHF	+	ENSG00000113905
4	3927445	3930682	Homo_sapiens_4_3929901-3931242-HERVHF	+	NONHSAG087348.2
4	17000545	17003928	Homo_sapiens_4_17000127-17001778-HERVHF	+	NONHSAG037572.2
4	24500975	24501427	Homo_sapiens_4_24503534-24505060-HERVHF	+	NONHSAG037630.2
4	27974874	27981319	Homo_sapiens_4_27976550-27977552-HERVHF	+	NONHSAG037691.2
4	92271492	92275299	Homo_sapiens_4_92273363-92274770-HERVHF	−	ENSG00000249152
4	103553770	103557353	Homo_sapiens_4_103555460-103556971-HERVHF	−	ENSG00000250920
4	128640901	128644450	Homo_sapiens_4_128642726-128644003-HERVHF	−	NONHSAG088517.2
4	145698823	145703505	Homo_sapiens_4_145701612-145702617-HERVHF	+	ENSG00000237136
4	152741345	152747172	Homo_sapiens_4_152743196-152744540-HERVHF	−	NONHSAG039129.2
4	175461163	175467003	Homo_sapiens_4_175463047-175464647-HERVHF	−	ENSG00000249945
5	92826033	92829706	Homo_sapiens_5_92826486-92827829-HERVHF	+	ENSG00000248588
5	136303790	136307028	Homo_sapiens_5_136303833-136305180-HERVHF	+	ENSG00000250947
5	161245405	161254586	Homo_sapiens_5_161251016-161252646-HERVHF	+	NONHSAG090654.1
6	16259010	16264893	Homo_sapiens_6_16260854-16262201-HERVHF	−	ENSG00000282024
6	18754142	18756902	Homo_sapiens_6_18755932-18757277-HERVHF	−	NONHSAG043117.2
6	80509795	80515805	Homo_sapiens_6_80511941-80513513-HERVHF	−	NONHSAG113295.1
6	94553917	94559610	Homo_sapiens_6_94555806-94557152-HERVHF	−	NONHSAG044390.2
6	97779489	97785327	Homo_sapiens_6_97782122-97783636-HERVHF	+	ENSG00000271860
6	123582333	123588007	Homo_sapiens_6_123584156-123585562-HERVHF	−	ENSG00000186439
6	125701846	125707764	Homo_sapiens_6_125703727-125705069-HERVHF	−	ENSG00000237742
6	126851224	126854456	Homo_sapiens_6_126851273-126852794-HERVHF	+	NONHSAG044785.3
6	131295347	131301206	Homo_sapiens_6_131297975-131299503-HERVHF	+	NONHSAG093612.2
6	131338799	131344566	Homo_sapiens_6_131340338-131342739-HERVHF	+	NONHSAG093612.2
6	131903830	131907420	Homo_sapiens_6_131904209-131905555-HERVHF	+	ENSG00000236673
6	144923164	144928866	Homo_sapiens_6_144925698-144927040-HERVHF	+	NONHSAG095837.2
7	26024199	26029809	Homo_sapiens_7_26026061-26027405-HERVHF	−	NONHSAG047156.2
7	34300132	34300573	Homo_sapiens_7_34301985-34303122-HERVHF	−	NONHSAG047318.2
7	102975230	102978736	Homo_sapiens_7_102976263-102977254-HERVHF	+	ENSG00000230257
7	125920130	125924112	Homo_sapiens_7_125920071-125921895-HERVHF	+	ENSG00000197462
7	155238821	155244070	Homo_sapiens_7_155240740-155241657-HERVK	−	NONHSAG049243.2
8	71676972	71680514	Homo_sapiens_8_71677433-71678968-HERVHF	+	ENSG00000254277
8	90090224	90093794	Homo_sapiens_8_90091914-90093348-HERVHF	−	ENSG00000104327
8	97200769	97206658	Homo_sapiens_8_97202388-97204973-HERVHF	+	NONHSAG098987.1
8	114284546	114287727	Homo_sapiens_8_114284508-114286044-HERVHF	+	ENSG00000254339
8	132080235	132086002	Homo_sapiens_8_132081909-132083445-HERVHF	−	ENSG00000132297
9	12950832	12954130	Homo_sapiens_9_12950845-12952399-HERVHF	+	NONHSAG101172.2
9	80137297	80143055	Homo_sapiens_9_80139873-80141469-HERVHF	+	NONHSAG052646.2
9	85461120	85466955	Homo_sapiens_9_85461166-85462902-HERVHF	+	NONHSAG052703.2
9	115475420	115478923	Homo_sapiens_9_115475976-115477349-HERVHF	+	NONHSAG053288.3
10	6797081	6802954	Homo_sapiens_10_6798770-6800364-HERVHF	−	NONHSAG005151.3
10	25716420	25722928	Homo_sapiens_10_25718978-25720776-HERVHF	+	ENSG00000280809
11	6366039	6371662	Homo_sapiens_11_6368276-6369885-HERVHF	+	NONHSAG007525.2
11	27629072	27632889	Homo_sapiens_11_27630864-27632291-HERVHF	−	ENSG00000254934
11	94641661	94647315	Homo_sapiens_11_94644134-94645475-HERVHF	+	ENSG00000255666
11	96499960	96506627	Homo_sapiens_11_96501724-96503654-HERVHF	+	ENSG00000183340
11	96590439	96593677	Homo_sapiens_11_96590449-96591982-HERVHF	+	ENSG00000254587
11	130565609	130570121	Homo_sapiens_11_130566060-130567548-HERVHF	−	NONHSAG010050.2
11	130753494	130756702	Homo_sapiens_11_130755373-130756704-HERVHF	−	NONHSAG010050.2
12	4018623	4023691	Homo_sapiens_12_4021109-4022208-HERVHF	+	ENSG00000256969
12	11462168	11468022	Homo_sapiens_12_11463877-11465381-HERVHF	−	ENSG00000121335
12	34269097	34274242	Homo_sapiens_12_34268101-34269869-HERVHF	+	NONHSAG010874.2
12	70444894	70450107	Homo_sapiens_12_70446553-70447732-HERVK	−	NONHSAG011664.2
12	86941530	86944748	Homo_sapiens_12_86941432-86943069-HERVHF	+	NONHSAG064903.1
13	42868001	42871007	Homo_sapiens_13_42869513-42870545-HERVHF	−	NONHSAG013351.2
13	48866771	48872457	Homo_sapiens_13_48868391-48870330-HERVHF	−	NONHSAG067525.1
13	51169866	51175008	Homo_sapiens_13_51172521-51173517-HERVHF	+	NONHSAG013541.3
13	54127417	54133159	Homo_sapiens_13_54129305-54130960-HERVHF	−	ENSG00000234787
13	66142250	66147037	Homo_sapiens_13_66143157-66144503-HERVHF	−	NONHSAG013698.2
13	79276611	79279830	Homo_sapiens_13_79276654-79278001-HERVHF	+	NONHSAG067153.1
14	38193319	38196529	Homo_sapiens_14_38193317-38194783-HERVHF	+	ENSG00000258649
14	41521426	41521883	Homo_sapiens_14_41518469-41520184-HERVHF	+	NONHSAG014802.2
14	48262389	48263146	Homo_sapiens_14_48256895-48258584-HERVHF	−	ENSG00000287492
15	74354141	74359786	Homo_sapiens_15_74355867-74357936-HERVHF	+	ENSG00000260266
15	87831107	87837024	Homo_sapiens_15_87833731-87835137-HERVHF	+	NONHSAG017784.2
16	60078536	60084582	Homo_sapiens_16_60081354-60082700-HERVHF	+	NONHSAG071739.1
16	65229803	65233421	Homo_sapiens_16_65231504-65233039-HERVHF	−	ENSG00000260834
18	28693028	28696068	Homo_sapiens_18_28694974-28696314-HERVHF	−	NONHSAG075074.1
18	56417745	56421344	Homo_sapiens_18_56418118-56419466-HERVHF	+	NONHSAG074828.1
18	57064647	57070296	Homo_sapiens_18_57068491-57069834-HERVHF	−	ENSG00000258609
18	73327171	73330369	Homo_sapiens_18_73327166-73328509-HERVHF	+	ENSG00000261780
19	22568269	22575022	Homo_sapiens_19_22570768-22572352-HERVHF	+	NONHSAG025320.2
20	12756027	12759632	Homo_sapiens_20_12756400-12757916-HERVHF	+	NONHSAG031288.2
20	40269047	40274769	Homo_sapiens_20_40271576-40272881-HERVHF	+	NONHSAG081519.1
21	17124024	17127764	Homo_sapiens_21_17123959-17125734-HERVHF	+	NONHSAG110806.1
21	26227947	26233485	Homo_sapiens_21_26229594-26231104-HERVHF	−	NONHSAG032575.2
21	42800845	42803999	Homo_sapiens_21_42802518-42804296-HERVHF	−	NONHSAG083198.1
X	71264372	71272628	Homo_sapiens_X_71266645-71268493-HERVHF	+	ENSG00000147140
X	94698818	94701832	Homo_sapiens_X_94700559-94702091-HERVHF	−	NONHSAG054922.2
X	111543806	111549675	Homo_sapiens_X_111546380-111547978-HERVHF	+	NONHSAG055109.3
X	122227333	122227787	Homo_sapiens_X_122224556-122226109-HERVHF	+	NONHSAG055239.2

## Data Availability

The data presented in this study are available in the article and the Supplementary Materials here. Additional data related to this article may be acquired from the authors.

## Supplementary Materials

The following supporting information can be downloaded at: https://www.mdpi.com/article/10.3390/v14071370/s1, Data S1: The common name, scientific name and classification of each primate we analyzed in this research; Data S2: The loci of each HERV we identified in the genomes of 49 primates; Data S3: The vertical transmission events in human genomes; Data S4: The alignments of the 5LTR and flanking sequences of a widely vertical transmitted HERV-H in 17 species; Data S5: The alignments of the 3LTR and flanking sequences of a widely vertical transmitted HERV-H in 17 species; Data S6: The alignments of the internal sequences of a widely vertical transmitted HERV-H in 17 species; Data S7: The LTRs’ blastn results of possible HERVs which were involved in host gene recombination; Data S8: The alignments of the 5LTR sequences of HERV in Figure 2B; Data S9: The alignments of the 3LTR sequences of HERV in Figure 2B; Data S10: The relationship between HERVs in human genomes and Human ncRNA.
