# Supplementary material for: Origin and Deep Evolution of Human Endogenous Retroviruses in Pan-Primates
Source: Viruses. 2022 Jun 23;14(7):1370. doi: 10.3390/v14071370 (PMC9323773; doi:10.3390/v14071370)
Supplement: Supplementary file 1 [file viruses-14-01370-s001.zip › viruses-1764260-supplementary.pdf]

Supplementary data of "Origin and deep evolution of human endogenous retroviruses in pan-primates"

Supplementary data of "Origin and deep evolution of human endogenous retroviruses in pan-primates" 1

Supplementary data S1 The common name, scientific name and classification of each primate we analyzed in this research 2

Supplementary data S2 The loci of each HERV we identified in the genomes of 49 primates 4

Supplementary data S3 The vertical transmission events in human genomes 247

Supplementary data S4 The alignments of the 5LTR and flanking sequences of a widely vertical transmitted HERV-H in 17 species 252

Supplementary data S5 The alignments of the 3LTR and flanking sequences of a widely vertical transmitted HERV-H in 17 species 267

Supplementary data S6 The alignments of the internal sequences of a widely vertical transmitted HERV-H in 17 species 282

Supplementary data S7 The LTRs' blastn results of possible HERVs which were involved in host gene recombination 316

Supplementary data S8 The alignments of the 5LTR sequences of HERV in Figure 2B 346

Supplementary data S9 The alignments of the 3LTR sequences of HERV in Figure 2B 347

Supplementary data S10 The relationship between HERVs in human genomes and Human ncRNA . 348

Supplementary data S1 The common name, scientific name and classification of each primate we analyzed in this research

Common name	Scientific name	Classification
Slender Loris	Loris tardigradus	prosimii
Pygmy Slow Loris	Nycticebus pygmaeus	prosimii
Bengal Slow Loris	Nycticebus bengalensis	prosimii
Small-Eared Galago	Otolemur garnettii	prosimii
Moholi Bushbaby	Galago moholi	prosimii
Aye-Aye	Daubentonia madagascariensis	prosimii
Gray Mouse Lemur	Microcebus murinus	prosimii
Greater Bamboo Lemur	Prolemur simus	prosimii
Ring-Tailed Lemur	Lemur catta	prosimii
White-Faced Saki	Pithecia pithecia	new world monkey
Midas Tamarin	Saguinus midas	new world monkey
White-Tufted-Ear Marmoset	Callithrix jacchus	new world monkey
Ma'S Night Monkey	Aotus nancymaae	new world monkey
White-Fronted Capuchin	Cebus albifrons	new world monkey
Tufted Capuchin	Sapajus apella	new world monkey
Black-Handed Spider Monkey	Ateles geoffroyi	new world monkey
Drill	Mandrillus leucophaeus	old world monkey
Mandrill	Mandrillus sphinx	old world monkey
Sooty Mangabey	Cercocebus atys	old world monkey
Hamadryas Baboon	Papio hamadryas	old world monkey
Olive Baboon	Papio anubis	old world monkey
Gelada	Theropithecus gelada	old world monkey
Black Crested Mangabey	Lophocebus aterrimus	old world monkey
Rhesus Monkey	Macaca mulatta	old world monkey
Assam Macaque	Macaca assamensis	old world monkey
Liontail Macaque	Macaca silenus	old world monkey
Pig-Tailed Macaque	Macaca nemestrina	old world monkey
Red Guenon	Erythrocebus patas	old world monkey
Grivet	Chlorocebus aethiops	old world monkey
Green Monkey	Chlorocebus sabaeus	old world monkey
Sykes' Monkey	Cercopithecus albogularis	old world monkey
Mona Monkey	Cercopithecus mona	old world monkey
Golden Snub-Nosed Monkey	Rhinopithecus roxellana	old world monkey
Burmese Snub-Nosed Monkey	Rhinopithecus strykeri	old world monkey
Black-Shanked Douc Langur	Pygathrix nigripes	old world monkey
Phayre'S Leaf Monkey	Trachypithecus crepusculus	old world monkey
Ugandan Red Colobus	Piliocolobus tephrosceles	old world monkey
Angolan colobus	Colobus angolensis palliatus	old world monkey
Mantled Guereza	Colobus guereza	old world monkey

Pileated Gibbon	Hylobates pileatus	ape
Siamang	Symphalangus syndactylus	ape
Eastern Hoolock Gibbon	Hoolock leuconedys	ape
Southern White-Cheeked Gibbon	Nomascus siki	ape
Western Lowland Gorilla	Gorilla gorilla gorilla	ape
Chimpanzee	Pan troglodytes	ape
Pygmy Chimpanzee	Pan paniscus	ape
Human	Homo sapiens	ape
Sumatran Orangutan	Pongo abelii	ape
Bornean Orangutan	Pongo pygmaeus	ape

Supplementary data S2 The loci of each HERV we identified in the genomes of 49 primates

Chr/contig	start	end	HERV&domanin	strand
Aotus_nancymaae_KZ196745.1	6462506	6462809	HERVIPADP_5LTR	+
Aotus_nancymaae_KZ196745.1	6465555	6468407	HERVIPADP_pol	+
Aotus_nancymaae_KZ196745.1	6470666	6471049	HERVIPADP_env	+
Aotus_nancymaae_KZ196745.1	6471355	6471661	HERVIPADP_3LTR	+
Aotus_nancymaae_KZ203894.1	2822626	2822960	HERVHF_5LTR	-
Aotus_nancymaae_KZ203894.1	2823470	2824845	HERVHF_pol	-
Aotus_nancymaae_KZ203894.1	2825275	2825961	HERVHF_gag	-
Aotus_nancymaae_KZ203894.1	2830884	2831226	HERVHF_3LTR	-
Aotus_nancymaae_KZ204317.1	11552841	11553166	HERVHF_5LTR	+
Aotus_nancymaae_KZ204317.1	11556883	11558726	HERVHF_pol	+
Aotus_nancymaae_KZ204317.1	11561301	11561639	HERVHF_3LTR	+
Atele_fuscicepstarseq_144	8885967	8886442	HERVHF_5LTR	+
Atele_fuscicepstarseq_144	8888365	8888913	HERVHF_gag	+
Atele_fuscicepstarseq_144	8889823	8891698	HERVHF_pol	+
Atele_fuscicepstarseq_144	8892536	8893022	HERVHF_3LTR	+
Atele_fuscicepstarseq_148	3665123	3665631	HERVHF_5LTR	-
Atele_fuscicepstarseq_148	3667488	3670180	HERVHF_pol	-
Atele_fuscicepstarseq_148	3671284	3671622	HERVHF_gag	-
Atele_fuscicepstarseq_148	3672683	3673201	HERVHF_3LTR	-
Atele_fuscicepstarseq_2063	47185985	47186354	HERVHF_5LTR	-
Atele_fuscicepstarseq_2063	47186645	47187378	HERVHF_env	-
Atele_fuscicepstarseq_2063	47188382	47189005	HERVHF_pol	-
Atele_fuscicepstarseq_2063	47189430	47189810	HERVHF_pro	-
Atele_fuscicepstarseq_2063	47189844	47190265	HERVHF_gag	-
Atele_fuscicepstarseq_2063	47191563	47191917	HERVHF_3LTR	-
Atele_fuscicepstarseq_2082	23074918	23075313	HERVHF_5LTR	+
Atele_fuscicepstarseq_2082	23078691	23079410	HERVHF_pol	+
Atele_fuscicepstarseq_2082	23080247	23080629	HERVHF_3LTR	+
Atele_fuscicepstarseq_2722	7528869	7529338	HERVHF_5LTR	-
Atele_fuscicepstarseq_2722	7533118	7535167	HERVHF_pol	-
Atele_fuscicepstarseq_2722	7535170	7535694	HERVHF_pro	-
Atele_fuscicepstarseq_2722	7536024	7536617	HERVHF_gag	-
Atele_fuscicepstarseq_2722	7538067	7538535	HERVHF_3LTR	-
Atele_fuscicepstarseq_577	37646577	37646921	Unknown_HERV_5LTR	+
Atele_fuscicepstarseq_577	37654083	37654670	Unknown_HERV_gag	+
Atele_fuscicepstarseq_577	37654884	37655444	Unknown_HERV_pro	+
Atele_fuscicepstarseq_577	37654998	37656101	Unknown_HERV_pol	+
Atele_fuscicepstarseq_577	37658162	37658521	Unknown_HERV_3LTR	+
Atele_fuscicepstarseq_588	2045392	2045706	HERVHF_5LTR	+
Atele_fuscicepstarseq_588	2046909	2047505	HERVHF_gag	+
Atele_fuscicepstarseq_588	2048103	2050228	HERVHF_pol	+

Atele_fuscicepstarseq_588	2051013	2052102	HERVHF_env	+
Atele_fuscicepstarseq_588	2052567	2052884	HERVHF_3LTR	+
Atele_fuscicepstarseq_6	53390673	53391027	Unknown_HERV_5LTR	+
Atele_fuscicepstarseq_6	53392738	53395088	Unknown_HERV_pol	+
Atele_fuscicepstarseq_6	53395581	53395945	Unknown_HERV_3LTR	+
Atele_fuscicepstarseq_857	13665434	13665774	HERVHF_5LTR	-
Atele_fuscicepstarseq_857	13668205	13669126	HERVHF_env	-
Atele_fuscicepstarseq_857	13669197	13669772	HERVHF_pol	-
Atele_fuscicepstarseq_857	13670232	13670789	HERVHF_pro	-
Atele_fuscicepstarseq_857	13670879	13671214	HERVHF_gag	-
Atele_fuscicepstarseq_857	13675042	13675393	HERVHF_3LTR	-
Cebus_albifrons_Contig13	33676470	33676810	HERVHF_5LTR	+
Cebus_albifrons_Contig13	33679764	33681718	HERVHF_pol	+
Cebus_albifrons_Contig13	33684029	33684371	HERVHF_3LTR	+
Cebus_albifrons_Contig14	1051783	1052334	HERVHF_5LTR	+
Cebus_albifrons_Contig14	1054942	1055250	HERVHF_gag	+
Cebus_albifrons_Contig14	1055450	1056190	HERVHF_pro	+
Cebus_albifrons_Contig14	1055798	1056852	HERVHF_pol	+
Cebus_albifrons_Contig14	1058627	1059185	HERVHF_3LTR	+
Cebus_albifrons_Contig2	16808811	16809455	HSERVIII_5LTR	-
Cebus_albifrons_Contig2	16813252	16814124	HSERVIII_pol	-
Cebus_albifrons_Contig2	16814851	16815495	HSERVIII_3LTR	-
Cebus_albifrons_Contig2	52232669	52232973	HERVHF_5LTR	+
Cebus_albifrons_Contig2	52235146	52235511	HERVHF_gag	+
Cebus_albifrons_Contig2	52236088	52236690	HERVHF_pro	+
Cebus_albifrons_Contig2	52236307	52238691	HERVHF_pol	+
Cebus_albifrons_Contig2	52239880	52240189	HERVHF_3LTR	+
Cebus_albifrons_Contig323	498717	499055	HERVHF_5LTR	-
Cebus_albifrons_Contig323	500759	503896	HERVHF_pol	-
Cebus_albifrons_Contig323	506453	506791	HERVHF_3LTR	-
Cebus_albifrons_Contig64	4161196	4161500	HERVK_5LTR	-
Cebus_albifrons_Contig64	4166588	4168631	HERVK_pol	-
Cebus_albifrons_Contig64	4170062	4170366	HERVK_3LTR	-
Cercocebus_atys_KQ009612.1	4196	4700	HERVK_5LTR	+
Cercocebus_atys_KQ009612.1	5620	6186	HERVK_gag	+
Cercocebus_atys_KQ009612.1	6203	7029	HERVK_pro	+
Cercocebus_atys_KQ009612.1	6885	8559	HERVK_pol	+
Cercocebus_atys_KQ009612.1	9082	9408	HERVK_env	+
Cercocebus_atys_KQ009612.1	9664	10168	HERVK_3LTR	+
Cercocebus_atys_KQ009636.1	285669	286009	HERVK_5LTR	+
Cercocebus_atys_KQ009636.1	287520	288212	HERVK_pro	+
Cercocebus_atys_KQ009636.1	288223	290132	HERVK_pol	+
Cercocebus_atys_KQ009636.1	290619	290954	HERVK_env	+
Cercocebus_atys_KQ009636.1	291500	291848	HERVK_3LTR	+

Cercocebus_atys_KQ009831.1	6997557	6997877	HSERVIII_5LTR	-
Cercocebus_atys_KQ009831.1	6999122	7001999	HSERVIII_pol	-
Cercocebus_atys_KQ009831.1	7004066	7004384	HSERVIII_3LTR	-
Cercocebus_atys_KQ009832.1	2600850	2601300	HERVHF_5LTR	-
Cercocebus_atys_KQ009832.1	2601770	2603458	HERVHF_pol	-
Cercocebus_atys_KQ009832.1	2603457	2603798	HERVHF_pro	-
Cercocebus_atys_KQ009832.1	2605865	2606319	HERVHF_3LTR	-
Cercocebus_atys_KQ009853.1	833045	833374	HERVHF_5LTR	-
Cercocebus_atys_KQ009853.1	834396	836409	HERVHF_pol	-
Cercocebus_atys_KQ009853.1	835852	836652	HERVHF_pro	-
Cercocebus_atys_KQ009853.1	836705	837046	HERVHF_gag	-
Cercocebus_atys_KQ009853.1	838494	838813	HERVHF_3LTR	-
Cercocebus_atys_KQ009918.1	6809356	6809797	HERVHF_5LTR	-
Cercocebus_atys_KQ009918.1	6810346	6810729	HERVHF_env	-
Cercocebus_atys_KQ009918.1	6810730	6812378	HERVHF_pol	-
Cercocebus_atys_KQ009918.1	6811740	6812525	HERVHF_pro	-
Cercocebus_atys_KQ009918.1	6812575	6812892	HERVHF_gag	-
Cercocebus_atys_KQ009918.1	6814537	6814982	HERVHF_3LTR	-
Cercocebus_atys_KQ010004.1	3852813	3853153	HERVHF_5LTR	-
Cercocebus_atys_KQ010004.1	3853631	3855575	HERVHF_pol	-
Cercocebus_atys_KQ010004.1	3855886	3856206	HERVHF_gag	-
Cercocebus_atys_KQ010004.1	3859140	3859487	HERVHF_3LTR	-
Cercocebus_atys_KQ010004.1	8289803	8290204	HERVHF_5LTR	-
Cercocebus_atys_KQ010004.1	8291090	8293012	HERVHF_pol	-
Cercocebus_atys_KQ010004.1	8292903	8293412	HERVHF_pro	-
Cercocebus_atys_KQ010004.1	8293473	8293778	HERVHF_gag	-
Cercocebus_atys_KQ010004.1	8294490	8294877	HERVHF_3LTR	-
Cercocebus_atys_KQ010015.1	969093	969447	HERVHF_5LTR	+
Cercocebus_atys_KQ010015.1	970825	971166	HERVHF_gag	+
Cercocebus_atys_KQ010015.1	971305	972396	HERVHF_pro	+
Cercocebus_atys_KQ010015.1	972037	974384	HERVHF_pol	+
Cercocebus_atys_KQ010015.1	976005	976370	HERVHF_3LTR	+
Cercocebus_atys_KQ010089.1	3585511	3585893	HERVHF_5LTR	-
Cercocebus_atys_KQ010089.1	3590420	3592958	HERVHF_pol	-
Cercocebus_atys_KQ010089.1	3593546	3594256	HERVHF_gag	-
Cercocebus_atys_KQ010089.1	3595669	3596056	HERVHF_3LTR	-
Cercocebus_atys_KQ010415.1	1927089	1927498	HERVHF_5LTR	-
Cercocebus_atys_KQ010415.1	1927664	1928014	HERVHF_env	-
Cercocebus_atys_KQ010415.1	1928331	1930837	HERVHF_pol	-
Cercocebus_atys_KQ010415.1	1930561	1931022	HERVHF_pro	-
Cercocebus_atys_KQ010415.1	1931086	1931547	HERVHF_gag	-
Cercocebus_atys_KQ010415.1	1933137	1933538	HERVHF_3LTR	-
Cercocebus_atys_KQ010443.1	5394267	5394635	HERVHF_5LTR	+
Cercocebus_atys_KQ010443.1	5396669	5397277	HERVHF_pro	+

Cercocebus_atys_KQ010443.1	5397151	5399070	HERVHF_pol	+
Cercocebus_atys_KQ010443.1	5399940	5400298	HERVHF_3LTR	+
Cercocebus_atys_KQ010523.1	1433937	1434260	HERVHF_5LTR	-
Cercocebus_atys_KQ010523.1	1434881	1437319	HERVHF_pol	-
Cercocebus_atys_KQ010523.1	1437537	1438118	HERVHF_pro	-
Cercocebus_atys_KQ010523.1	1439857	1440188	HERVHF_3LTR	-
Cercocebus_atys_KQ010541.1	2149201	2149603	HERVHF_5LTR	+
Cercocebus_atys_KQ010541.1	2151721	2152470	HERVHF_pro	+
Cercocebus_atys_KQ010541.1	2151973	2152641	HERVHF_pol	+
Cercocebus_atys_KQ010541.1	2153565	2154327	HERVHF_env	+
Cercocebus_atys_KQ010541.1	2154512	2154909	HERVHF_3LTR	+
Cercocebus_atys_KQ010562.1	1772858	1773218	HERVHF_5LTR	-
Cercocebus_atys_KQ010562.1	1774587	1777089	HERVHF_pol	-
Cercocebus_atys_KQ010562.1	1776550	1777089	HERVHF_pro	-
Cercocebus_atys_KQ010562.1	1778217	1778588	HERVHF_3LTR	-
Cercocebus_atys_KQ010602.1	3428960	3429399	HERVK_5LTR	+
Cercocebus_atys_KQ010602.1	3430200	3431426	HERVK_pro	+
Cercocebus_atys_KQ010602.1	3431019	3431750	HERVK_pol	+
Cercocebus_atys_KQ010602.1	3433032	3433467	HERVK_3LTR	+
Cercocebus_atys_KQ010662.1	6715481	6715804	HERVHF_5LTR	+
Cercocebus_atys_KQ010662.1	6718179	6718502	HERVHF_gag	+
Cercocebus_atys_KQ010662.1	6718519	6719181	HERVHF_pro	+
Cercocebus_atys_KQ010662.1	6718780	6720829	HERVHF_pol	+
Cercocebus_atys_KQ010662.1	6721277	6721604	HERVHF_3LTR	+
Cercocebus_atys_KQ010662.1	9059382	9059788	HERVHF_5LTR	-
Cercocebus_atys_KQ010662.1	9059954	9067526	HERVHF_env	-
Cercocebus_atys_KQ010662.1	9067973	9070399	HERVHF_pol	-
Cercocebus_atys_KQ010662.1	9069878	9070678	HERVHF_pro	-
Cercocebus_atys_KQ010662.1	9073317	9073723	HERVHF_3LTR	-
Cercocebus_atys_KQ010752.1	19978952	19979329	HERVHF_5LTR	-
Cercocebus_atys_KQ010752.1	19980196	19982388	HERVHF_pol	-
Cercocebus_atys_KQ010752.1	19981813	19982652	HERVHF_pro	-
Cercocebus_atys_KQ010752.1	19982696	19983016	HERVHF_gag	-
Cercocebus_atys_KQ010752.1	19984496	19984872	HERVHF_3LTR	-
Cercocebus_atys_KQ010796.1	3522236	3522598	HERVHF_5LTR	-
Cercocebus_atys_KQ010796.1	3523635	3524075	HERVHF_pol	-
Cercocebus_atys_KQ010796.1	3524222	3524629	HERVHF_pro	-
Cercocebus_atys_KQ010796.1	3524622	3525227	HERVHF_gag	-
Cercocebus_atys_KQ010796.1	3526491	3526840	HERVHF_3LTR	-
Cercocebus_atys_KQ010807.1	387332	387691	HERVHF_5LTR	+
Cercocebus_atys_KQ010807.1	389745	390494	HERVHF_pro	+
Cercocebus_atys_KQ010807.1	390363	392313	HERVHF_pol	+
Cercocebus_atys_KQ010807.1	392788	393141	HERVHF_3LTR	+
Cercocebus_atys_KQ010817.1	876464	876794	HERVHF_5LTR	+

Cercocebus_atys_KQ010817.1	879206	879904	HERVHF_pol	+
Cercocebus_atys_KQ010817.1	880184	880558	HERVHF_env	+
Cercocebus_atys_KQ010817.1	880725	881056	HERVHF_3LTR	+
Cercocebus_atys_KQ010939.1	22340397	22340708	HERVHF_5LTR	-
Cercocebus_atys_KQ010939.1	22341498	22343271	HERVHF_pol	-
Cercocebus_atys_KQ010939.1	22343375	22343695	HERVHF_pro	-
Cercocebus_atys_KQ010939.1	22345592	22345900	HERVHF_3LTR	-
Cercocebus_atys_KQ011029.1	12402692	12403052	HERVHF_5LTR	-
Cercocebus_atys_KQ011029.1	12403939	12405532	HERVHF_pol	-
Cercocebus_atys_KQ011029.1	12404990	12405739	HERVHF_pro	-
Cercocebus_atys_KQ011029.1	12407910	12408277	HERVHF_3LTR	-
Cercocebus_atys_KQ011124.1	1915138	1915438	HERVHF_5LTR	-
Cercocebus_atys_KQ011124.1	1916279	1917823	HERVHF_pol	-
Cercocebus_atys_KQ011124.1	1918329	1918706	HERVHF_gag	-
Cercocebus_atys_KQ011124.1	1920624	1920926	HERVHF_3LTR	-
Cercocebus_atys_KQ011124.1	8891546	8891877	HERVHF_5LTR	-
Cercocebus_atys_KQ011124.1	8893164	8893745	HERVHF_pol	-
Cercocebus_atys_KQ011124.1	8893355	8893957	HERVHF_pro	-
Cercocebus_atys_KQ011124.1	8894168	8894491	HERVHF_gag	-
Cercocebus_atys_KQ011124.1	8896235	8896550	HERVHF_3LTR	-
Cercocebus_atys_KQ011181.1	2671711	2672018	HERVHF_5LTR	-
Cercocebus_atys_KQ011181.1	2672193	2672613	HERVHF_env	-
Cercocebus_atys_KQ011181.1	2673038	2673806	HERVHF_pol	-
Cercocebus_atys_KQ011181.1	2674001	2674756	HERVHF_pro	-
Cercocebus_atys_KQ011181.1	2674902	2675261	HERVHF_gag	-
Cercocebus_atys_KQ011181.1	2676857	2677173	HERVHF_3LTR	-
Cercocebus_atys_KQ011219.1	17637751	17638182	HERVHF_5LTR	-
Cercocebus_atys_KQ011219.1	17639406	17641306	HERVHF_pol	-
Cercocebus_atys_KQ011219.1	17640668	17641453	HERVHF_pro	-
Cercocebus_atys_KQ011219.1	17643650	17644082	HERVHF_3LTR	-
Cercocebus_atys_KQ011258.1	379972	380430	HERVK_5LTR	-
Cercocebus_atys_KQ011258.1	381965	383438	HERVK_pol	-
Cercocebus_atys_KQ011258.1	383348	384007	HERVK_pro	-
Cercocebus_atys_KQ011258.1	385745	386217	HERVK_3LTR	-
Cercocebus_atys_KQ011346.1	1808369	1808769	HERVHF_5LTR	-
Cercocebus_atys_KQ011346.1	1809610	1811855	HERVHF_pol	-
Cercocebus_atys_KQ011346.1	1811217	1811948	HERVHF_pro	-
Cercocebus_atys_KQ011346.1	1812047	1812388	HERVHF_gag	-
Cercocebus_atys_KQ011346.1	1813963	1814354	HERVHF_3LTR	-
Cercocebus_atys_KQ011503.1	8041399	8041846	HERVHF_5LTR	-
Cercocebus_atys_KQ011503.1	8041993	8042346	HERVHF_env	-
Cercocebus_atys_KQ011503.1	8043423	8044978	HERVHF_pol	-
Cercocebus_atys_KQ011503.1	8045761	8047055	HERVHF_gag	-
Cercocebus_atys_KQ011503.1	8047766	8048218	HERVHF_3LTR	-

Cercocebus_atys_KQ011593.1	491621	492071	HERVHF_5LTR	+
Cercocebus_atys_KQ011593.1	493766	494086	HERVHF_gag	+
Cercocebus_atys_KQ011593.1	494267	495819	HERVHF_pol	+
Cercocebus_atys_KQ011593.1	496880	497331	HERVHF_3LTR	+
Cercocebus_atys_KQ011788.1	52420	52735	HERVIPADP_5LTR	+
Cercocebus_atys_KQ011788.1	54429	54923	HERVIPADP_gag	+
Cercocebus_atys_KQ011788.1	55709	56464	HERVIPADP_pol	+
Cercocebus_atys_KQ011788.1	57799	58587	HERVIPADP_env	+
Cercocebus_atys_KQ011788.1	58764	59084	HERVIPADP_3LTR	+
Cercocebus_atys_KQ011789.1	858385	858805	HERVHF_5LTR	+
Cercocebus_atys_KQ011789.1	860280	860588	HERVHF_gag	+
Cercocebus_atys_KQ011789.1	860908	861543	HERVHF_pol	+
Cercocebus_atys_KQ011789.1	868411	868835	HERVHF_3LTR	+
Cercocebus_atys_KQ011854.1	551713	552091	HERVHF_5LTR	-
Cercocebus_atys_KQ011854.1	552241	552597	HERVHF_env	-
Cercocebus_atys_KQ011854.1	553926	555494	HERVHF_pol	-
Cercocebus_atys_KQ011854.1	555708	556019	HERVHF_gag	-
Cercocebus_atys_KQ011854.1	557372	557750	HERVHF_3LTR	-
Cercocebus_atys_KQ011882.1	3324979	3325390	HERVHF_5LTR	+
Cercocebus_atys_KQ011882.1	3326875	3327231	HERVHF_gag	+
Cercocebus_atys_KQ011882.1	3327236	3328189	HERVHF_pro	+
Cercocebus_atys_KQ011882.1	3327521	3330311	HERVHF_pol	+
Cercocebus_atys_KQ011882.1	3330953	3331370	HERVHF_3LTR	+
Cercocebus_atys_KQ012375.1	28905162	28905502	HERVK_5LTR	-
Cercocebus_atys_KQ012375.1	28905736	28906064	HERVK_env	-
Cercocebus_atys_KQ012375.1	28906748	28908504	HERVK_pol	-
Cercocebus_atys_KQ012375.1	28908396	28909298	HERVK_pro	-
Cercocebus_atys_KQ012375.1	28910246	28910598	HERVK_3LTR	-
Cercocebus_atys_KQ012397.1	8911896	8912347	HERVHF_5LTR	-
Cercocebus_atys_KQ012397.1	8912793	8915033	HERVHF_pol	-
Cercocebus_atys_KQ012397.1	8914680	8915198	HERVHF_pro	-
Cercocebus_atys_KQ012397.1	8918253	8918706	HERVHF_3LTR	-
Cercocebus_atys_KQ012397.1	16731686	16732056	HUERSP_5LTR	-
Cercocebus_atys_KQ012397.1	16732321	16735258	HUERSP_env	-
Cercocebus_atys_KQ012397.1	16737081	16737715	HUERSP_pol	-
Cercocebus_atys_KQ012397.1	16738413	16739045	HUERSP_gag	-
Cercocebus_atys_KQ012397.1	16741061	16741426	HUERSP_3LTR	-
Cercocebus_atys_KQ012408.1	13474040	13474470	HERVHF_5LTR	+
Cercocebus_atys_KQ012408.1	13476677	13478824	HERVHF_pol	+
Cercocebus_atys_KQ012408.1	13479649	13480080	HERVHF_3LTR	+
Cercocebus_atys_KQ012419.1	1780545	1780940	HERVHF_5LTR	+
Cercocebus_atys_KQ012419.1	1782546	1783124	HERVHF_gag	+
Cercocebus_atys_KQ012419.1	1783306	1784097	HERVHF_pro	+
Cercocebus_atys_KQ012419.1	1783617	1785833	HERVHF_pol	+

Cercocebus_atys_KQ012419.1	1785930	1786283	HERVHF_env	+
Cercocebus_atys_KQ012419.1	1786450	1786846	HERVHF_3LTR	+
Cercocebus_atys_KQ012430.1	6748237	6748668	HERVHF_5LTR	-
Cercocebus_atys_KQ012430.1	6749640	6751926	HERVHF_pol	-
Cercocebus_atys_KQ012430.1	6752868	6754076	HERVHF_gag	-
Cercocebus_atys_KQ012430.1	6755702	6756147	HERVHF_3LTR	-
Cercocebus_atys_KQ012453.1	14623272	14623660	HERVHF_5LTR	-
Cercocebus_atys_KQ012453.1	14623869	14624360	HERVHF_env	-
Cercocebus_atys_KQ012453.1	14625906	14627159	HERVHF_pol	-
Cercocebus_atys_KQ012453.1	14627293	14628109	HERVHF_pro	-
Cercocebus_atys_KQ012453.1	14627852	14628337	HERVHF_gag	-
Cercocebus_atys_KQ012453.1	14629569	14629956	HERVHF_3LTR	-
Cercocebus_atys_KQ012484.1	248694	249013	HERVHF_5LTR	-
Cercocebus_atys_KQ012484.1	250093	252634	HERVHF_pol	-
Cercocebus_atys_KQ012484.1	253079	253657	HERVHF_gag	-
Cercocebus_atys_KQ012484.1	254904	255236	HERVHF_3LTR	-
Cercocebus_atys_KQ012552.1	42753369	42753695	HERVHF_5LTR	+
Cercocebus_atys_KQ012552.1	42755589	42756401	HERVHF_pro	+
Cercocebus_atys_KQ012552.1	42755826	42758252	HERVHF_pol	+
Cercocebus_atys_KQ012552.1	42758867	42759194	HERVHF_3LTR	+
Cercocebus_atys_KQ012575.1	13739872	13740278	HERVHF_5LTR	+
Cercocebus_atys_KQ012575.1	13742534	13744221	HERVHF_pol	+
Cercocebus_atys_KQ012575.1	13745055	13745450	HERVHF_3LTR	+
Cercocebus_atys_KQ012597.1	2481050	2481542	HERVK_5LTR	+
Cercocebus_atys_KQ012597.1	2483072	2483938	HERVK_pro	+
Cercocebus_atys_KQ012597.1	2483851	2485576	HERVK_pol	+
Cercocebus_atys_KQ012597.1	2486116	2486430	HERVK_env	+
Cercocebus_atys_KQ012597.1	2486528	2487041	HERVK_3LTR	+
Cercocebus_atys_KQ012597.1	7381822	7382123	HERVHF_5LTR	-
Cercocebus_atys_KQ012597.1	7382131	7384534	HERVHF_pol	-
Cercocebus_atys_KQ012597.1	7384575	7384904	HERVHF_pro	-
Cercocebus_atys_KQ012597.1	7386868	7387177	HERVHF_3LTR	-
Cercocebus_atys_KQ012619.1	9201550	9202007	HERVHF_5LTR	-
Cercocebus_atys_KQ012619.1	9202935	9204537	HERVHF_pol	-
Cercocebus_atys_KQ012619.1	9203893	9204765	HERVHF_pro	-
Cercocebus_atys_KQ012619.1	9206621	9207076	HERVHF_3LTR	-
Cercocebus_atys_KQ012641.1	14684709	14685060	HERVHF_5LTR	-
Cercocebus_atys_KQ012641.1	14685666	14688219	HERVHF_pol	-
Cercocebus_atys_KQ012641.1	14687833	14688534	HERVHF_pro	-
Cercocebus_atys_KQ012641.1	14688717	14689316	HERVHF_gag	-
Cercocebus_atys_KQ012641.1	14690840	14691189	HERVHF_3LTR	-
Cercocebus_atys_KQ012652.1	2177523	2178008	HERVK_5LTR	+
Cercocebus_atys_KQ012652.1	2178913	2179452	HERVK_gag	+
Cercocebus_atys_KQ012652.1	2179757	2180464	HERVK_pro	+

Cercocebus_atys_KQ012652.1	2180463	2181883	HERVK_pol	+
Cercocebus_atys_KQ012652.1	2182300	2182731	HERVK_env	+
Cercocebus_atys_KQ012652.1	2182828	2183301	HERVK_3LTR	+
Cercocebus_atys_KQ012741.1	10253175	10253533	HERVHF_5LTR	+
Cercocebus_atys_KQ012741.1	10255941	10258178	HERVHF_pol	+
Cercocebus_atys_KQ012741.1	10258911	10259272	HERVHF_3LTR	+
Cercocebus_atys_KQ012774.1	22835127	22835618	HERVIPADP_5LTR	-
Cercocebus_atys_KQ012774.1	22838224	22840888	HERVIPADP_pol	-
Cercocebus_atys_KQ012774.1	22841314	22841670	HERVIPADP_gag	-
Cercocebus_atys_KQ012774.1	22843446	22843939	HERVIPADP_3LTR	-
Cercocebus_atys_KQ012786.1	12672936	12673380	HERVHF_5LTR	+
Cercocebus_atys_KQ012786.1	12675180	12675488	HERVHF_gag	+
Cercocebus_atys_KQ012786.1	12675541	12675963	HERVHF_pro	+
Cercocebus_atys_KQ012786.1	12675912	12677449	HERVHF_pol	+
Cercocebus_atys_KQ012786.1	12678119	12678560	HERVHF_3LTR	+
Cercocebus_atys_KQ012885.1	26400329	26400757	HERVHF_5LTR	-
Cercocebus_atys_KQ012885.1	26401208	26403651	HERVHF_pol	-
Cercocebus_atys_KQ012885.1	26403519	26403977	HERVHF_pro	-
Cercocebus_atys_KQ012885.1	26405972	26406408	HERVHF_3LTR	-
Cercocebus_atys_KQ012929.1	1266037	1266468	HERVHF_5LTR	-
Cercocebus_atys_KQ012929.1	1267371	1269027	HERVHF_pol	-
Cercocebus_atys_KQ012929.1	1268401	1269168	HERVHF_pro	-
Cercocebus_atys_KQ012929.1	1271240	1271692	HERVHF_3LTR	-
Cercocebus_atys_KQ012961.1	4255195	4255665	HERVHF_5LTR	+
Cercocebus_atys_KQ012961.1	4257811	4258452	HERVHF_pro	+
Cercocebus_atys_KQ012961.1	4257919	4259630	HERVHF_pol	+
Cercocebus_atys_KQ012961.1	4260784	4261254	HERVHF_3LTR	+
Cercocebus_atys_KQ012983.1	7733074	7733424	HERVHF_5LTR	+
Cercocebus_atys_KQ012983.1	7735076	7735549	HERVHF_gag	+
Cercocebus_atys_KQ012983.1	7735978	7738069	HERVHF_pol	+
Cercocebus_atys_KQ012983.1	7739518	7740027	HERVHF_env	+
Cercocebus_atys_KQ012983.1	7740255	7740617	HERVHF_3LTR	+
Cercopithecus_mitis_ctg1	24122119	24122573	HERVK_5LTR	-
Cercopithecus_mitis_ctg1	24123530	24125443	HERVK_pol	-
Cercopithecus_mitis_ctg1	24125508	24126221	HERVK_pro	-
Cercopithecus_mitis_ctg1	24127205	24127654	HERVK_3LTR	-
Cercopithecus_mitis_ctg102	8091606	8092063	HERVHF_5LTR	+
Cercopithecus_mitis_ctg102	8093529	8093882	HERVHF_gag	+
Cercopithecus_mitis_ctg102	8094493	8096975	HERVHF_pol	+
Cercopithecus_mitis_ctg102	8098744	8099462	HERVHF_env	+
Cercopithecus_mitis_ctg102	8099673	8100147	HERVHF_3LTR	+
Cercopithecus_mitis_ctg104	96059	96358	HERVHF_5LTR	+
Cercopithecus_mitis_ctg104	98043	98420	HERVHF_gag	+
Cercopithecus_mitis_ctg104	98536	99288	HERVHF_pro	+

Cercopithecus_mitis_ctg104	98728	100188	HERVHF_pol	+
Cercopithecus_mitis_ctg104	100300	100818	HERVHF_env	+
Cercopithecus_mitis_ctg104	101250	101554	HERVHF_3LTR	+
Cercopithecus_mitis_ctg111	6692497	6692872	HERVHF_5LTR	-
Cercopithecus_mitis_ctg111	6694199	6696808	HERVHF_pol	-
Cercopithecus_mitis_ctg111	6696765	6697205	HERVHF_pro	-
Cercopithecus_mitis_ctg111	6697325	6697651	HERVHF_gag	-
Cercopithecus_mitis_ctg111	6699108	6699471	HERVHF_3LTR	-
Cercopithecus_mitis_ctg112	5216784	5217147	HERVHF_5LTR	-
Cercopithecus_mitis_ctg112	5217312	5217886	HERVHF_env	-
Cercopithecus_mitis_ctg112	5218588	5220686	HERVHF_pol	-
Cercopithecus_mitis_ctg112	5220410	5220871	HERVHF_pro	-
Cercopithecus_mitis_ctg112	5220912	5221220	HERVHF_gag	-
Cercopithecus_mitis_ctg112	5222700	5223060	HERVHF_3LTR	-
Cercopithecus_mitis_ctg114	26663217	26663528	HERVHF_5LTR	+
Cercopithecus_mitis_ctg114	26666278	26666640	HERVHF_gag	+
Cercopithecus_mitis_ctg114	26667342	26668010	HERVHF_pro	+
Cercopithecus_mitis_ctg114	26667447	26669341	HERVHF_pol	+
Cercopithecus_mitis_ctg114	26675797	26676118	HERVHF_3LTR	+
Cercopithecus_mitis_ctg125	8302176	8302602	HERVHF_5LTR	-
Cercopithecus_mitis_ctg125	8302790	8304211	HERVHF_pol	-
Cercopithecus_mitis_ctg125	8304005	8304710	HERVHF_pro	-
Cercopithecus_mitis_ctg125	8304943	8305369	HERVHF_3LTR	-
Cercopithecus_mitis_ctg129	8726531	8726940	HERVHF_5LTR	-
Cercopithecus_mitis_ctg129	8727528	8730066	HERVHF_pol	-
Cercopithecus_mitis_ctg129	8729410	8730252	HERVHF_pro	-
Cercopithecus_mitis_ctg129	8730282	8730644	HERVHF_gag	-
Cercopithecus_mitis_ctg129	8732173	8732586	HERVHF_3LTR	-
Cercopithecus_mitis_ctg13	8839949	8840357	HERVHF_5LTR	-
Cercopithecus_mitis_ctg13	8840958	8842920	HERVHF_pol	-
Cercopithecus_mitis_ctg13	8842674	8843387	HERVHF_pro	-
Cercopithecus_mitis_ctg13	8843421	8843864	HERVHF_gag	-
Cercopithecus_mitis_ctg13	8845236	8845633	HERVHF_3LTR	-
Cercopithecus_mitis_ctg132	9457461	9457790	HERVHF_5LTR	+
Cercopithecus_mitis_ctg132	9459942	9460316	HERVHF_pro	+
Cercopithecus_mitis_ctg132	9460221	9462539	HERVHF_pol	+
Cercopithecus_mitis_ctg132	9462924	9463310	HERVHF_env	+
Cercopithecus_mitis_ctg132	9463395	9463739	HERVHF_3LTR	+
Cercopithecus_mitis_ctg156	1280365	1280817	HERVHF_5LTR	+
Cercopithecus_mitis_ctg156	1282505	1282810	HERVHF_gag	+
Cercopithecus_mitis_ctg156	1282893	1283615	HERVHF_pro	+
Cercopithecus_mitis_ctg156	1283061	1285133	HERVHF_pol	+
Cercopithecus_mitis_ctg156	1285569	1286025	HERVHF_3LTR	+
Cercopithecus_mitis_ctg158	3705244	3705626	HERVHF_5LTR	-

Cercopithecus_mitis_ctg158	3705809	3706530	HERVHF_env	-
Cercopithecus_mitis_ctg158	3712322	3714636	HERVHF_pol	-
Cercopithecus_mitis_ctg158	3714695	3715141	HERVHF_pro	-
Cercopithecus_mitis_ctg158	3717321	3717708	HERVHF_3LTR	-
Cercopithecus_mitis_ctg16	44551454	44551904	HERVHF_5LTR	-
Cercopithecus_mitis_ctg16	44552757	44554398	HERVHF_pol	-
Cercopithecus_mitis_ctg16	44554696	44555037	HERVHF_gag	-
Cercopithecus_mitis_ctg16	44556704	44557154	HERVHF_3LTR	-
Cercopithecus_mitis_ctg161	2806018	2806389	HERVHF_5LTR	+
Cercopithecus_mitis_ctg161	2807025	2807744	HERVHF_pro	+
Cercopithecus_mitis_ctg161	2807268	2808898	HERVHF_pol	+
Cercopithecus_mitis_ctg161	2810558	2810921	HERVHF_3LTR	+
Cercopithecus_mitis_ctg165	6190827	6191260	HERVHF_5LTR	+
Cercopithecus_mitis_ctg165	6192164	6193062	HERVHF_gag	+
Cercopithecus_mitis_ctg165	6193428	6195818	HERVHF_pol	+
Cercopithecus_mitis_ctg165	6196363	6196798	HERVHF_3LTR	+
Cercopithecus_mitis_ctg184	1332441	1332837	HERVHF_5LTR	+
Cercopithecus_mitis_ctg184	1334671	1335687	HERVHF_pro	+
Cercopithecus_mitis_ctg184	1335007	1337303	HERVHF_pol	+
Cercopithecus_mitis_ctg184	1337671	1338068	HERVHF_3LTR	+
Cercopithecus_mitis_ctg194	3317430	3317870	HERVHF_5LTR	+
Cercopithecus_mitis_ctg194	3320257	3321741	HERVHF_pol	+
Cercopithecus_mitis_ctg194	3322422	3322876	HERVHF_3LTR	+
Cercopithecus_mitis_ctg197	2316921	2317289	HERVHF_5LTR	+
Cercopithecus_mitis_ctg197	2320063	2320518	HERVHF_gag	+
Cercopithecus_mitis_ctg197	2320537	2321010	HERVHF_pro	+
Cercopithecus_mitis_ctg197	2321057	2322599	HERVHF_pol	+
Cercopithecus_mitis_ctg197	2323067	2323439	HERVHF_3LTR	+
Cercopithecus_mitis_ctg2	19933394	19933842	HERVHF_5LTR	-
Cercopithecus_mitis_ctg2	19935154	19936737	HERVHF_pol	-
Cercopithecus_mitis_ctg2	19936195	19936980	HERVHF_pro	-
Cercopithecus_mitis_ctg2	19938987	19939440	HERVHF_3LTR	-
Cercopithecus_mitis_ctg21	964768	965285	HERVK_5LTR	+
Cercopithecus_mitis_ctg21	965662	966646	HERVK_gag	+
Cercopithecus_mitis_ctg21	966709	967591	HERVK_pro	+
Cercopithecus_mitis_ctg21	967450	968716	HERVK_pol	+
Cercopithecus_mitis_ctg21	969208	969519	HERVK_env	+
Cercopithecus_mitis_ctg21	969596	970110	HERVK_3LTR	+
Cercopithecus_mitis_ctg247	1454353	1454746	HERVHF_5LTR	-
Cercopithecus_mitis_ctg247	1455434	1457600	HERVHF_pol	-
Cercopithecus_mitis_ctg247	1457196	1457924	HERVHF_pro	-
Cercopithecus_mitis_ctg247	1458023	1458364	HERVHF_gag	-
Cercopithecus_mitis_ctg247	1459939	1460330	HERVHF_3LTR	-
Cercopithecus_mitis_ctg26	15585634	15586066	HERVHF_5LTR	+

Cercopithecus_mitis_ctg26	15587895	15588326	HERVHF_pro	+
Cercopithecus_mitis_ctg26	15588229	15590400	HERVHF_pol	+
Cercopithecus_mitis_ctg26	15590720	15591040	HERVHF_env	+
Cercopithecus_mitis_ctg26	15591202	15591640	HERVHF_3LTR	+
Cercopithecus_mitis_ctg26	37440988	37441311	HERVHF_5LTR	-
Cercopithecus_mitis_ctg26	37441882	37443612	HERVHF_pol	-
Cercopithecus_mitis_ctg26	37446450	37446781	HERVHF_3LTR	-
Cercopithecus_mitis_ctg282	163058	163498	HERVHF_5LTR	-
Cercopithecus_mitis_ctg282	164587	166460	HERVHF_pol	-
Cercopithecus_mitis_ctg282	166787	167086	HERVHF_gag	-
Cercopithecus_mitis_ctg282	168665	169097	HERVHF_3LTR	-
Cercopithecus_mitis_ctg29	7170370	7170817	HERVHF_5LTR	+
Cercopithecus_mitis_ctg29	7172503	7172856	HERVHF_gag	+
Cercopithecus_mitis_ctg29	7172846	7173673	HERVHF_pro	+
Cercopithecus_mitis_ctg29	7173134	7174766	HERVHF_pol	+
Cercopithecus_mitis_ctg29	7175635	7176068	HERVHF_3LTR	+
Cercopithecus_mitis_ctg30	16391519	16391931	HERVHF_5LTR	-
Cercopithecus_mitis_ctg30	16392651	16395374	HERVHF_pol	-
Cercopithecus_mitis_ctg30	16394736	16395440	HERVHF_pro	-
Cercopithecus_mitis_ctg30	16397653	16398065	HERVHF_3LTR	-
Cercopithecus_mitis_ctg393	404262	404594	HERVHF_5LTR	+
Cercopithecus_mitis_ctg393	405840	406418	HERVHF_gag	+
Cercopithecus_mitis_ctg393	406606	407436	HERVHF_pro	+
Cercopithecus_mitis_ctg393	406624	410167	HERVHF_pol	+
Cercopithecus_mitis_ctg393	410482	410803	HERVHF_3LTR	+
Cercopithecus_mitis_ctg42	10341181	10341533	HERVHF_5LTR	-
Cercopithecus_mitis_ctg42	10342257	10343967	HERVHF_pol	-
Cercopithecus_mitis_ctg42	10343925	10344359	HERVHF_pro	-
Cercopithecus_mitis_ctg42	10344440	10344873	HERVHF_gag	-
Cercopithecus_mitis_ctg42	10346383	10346731	HERVHF_3LTR	-
Cercopithecus_mitis_ctg43	11649635	11649984	HERVK_5LTR	+
Cercopithecus_mitis_ctg43	11650496	11651447	HERVK_gag	+
Cercopithecus_mitis_ctg43	11651707	11652270	HERVK_pro	+
Cercopithecus_mitis_ctg43	11652189	11654085	HERVK_pol	+
Cercopithecus_mitis_ctg43	11655062	11655430	HERVK_3LTR	+
Cercopithecus_mitis_ctg44	29614624	29614950	HERVHF_5LTR	-
Cercopithecus_mitis_ctg44	29615240	29615581	HERVHF_env	-
Cercopithecus_mitis_ctg44	29615878	29616582	HERVHF_pol	-
Cercopithecus_mitis_ctg44	29616896	29617306	HERVHF_gag	-
Cercopithecus_mitis_ctg44	29618888	29619206	HERVHF_3LTR	-
Cercopithecus_mitis_ctg47	1733475	1733791	HERVHF_5LTR	+
Cercopithecus_mitis_ctg47	1735327	1735779	HERVHF_gag	+
Cercopithecus_mitis_ctg47	1735897	1736742	HERVHF_pro	+
Cercopithecus_mitis_ctg47	1736362	1738505	HERVHF_pol	+

Cercopithecus_mitis_ctg47	1739369	1739700	HERVHF_3LTR	+
Cercopithecus_mitis_ctg49	6281887	6282336	HERVHF_5LTR	+
Cercopithecus_mitis_ctg49	6283906	6284259	HERVHF_gag	+
Cercopithecus_mitis_ctg49	6284428	6285177	HERVHF_pro	+
Cercopithecus_mitis_ctg49	6284533	6286075	HERVHF_pol	+
Cercopithecus_mitis_ctg49	6286890	6287337	HERVHF_3LTR	+
Cercopithecus_mitis_ctg5	11880088	11880395	HERVHF_5LTR	+
Cercopithecus_mitis_ctg5	11881837	11882289	HERVHF_gag	+
Cercopithecus_mitis_ctg5	11882347	11883123	HERVHF_pro	+
Cercopithecus_mitis_ctg5	11882509	11883411	HERVHF_pol	+
Cercopithecus_mitis_ctg5	11885268	11885579	HERVHF_3LTR	+
Cercopithecus_mitis_ctg55	186473	186833	HERVHF_5LTR	-
Cercopithecus_mitis_ctg55	187061	187381	HERVHF_env	-
Cercopithecus_mitis_ctg55	187318	188368	HERVHF_pol	-
Cercopithecus_mitis_ctg55	188265	189131	HERVHF_pro	-
Cercopithecus_mitis_ctg55	189284	190126	HERVHF_gag	-
Cercopithecus_mitis_ctg55	190866	191233	HERVHF_3LTR	-
Cercopithecus_mitis_ctg58	9304446	9304905	HERVHF_5LTR	+
Cercopithecus_mitis_ctg58	9307258	9307956	HERVHF_pro	+
Cercopithecus_mitis_ctg58	9307288	9309215	HERVHF_pol	+
Cercopithecus_mitis_ctg58	9309714	9310181	HERVHF_3LTR	+
Cercopithecus_mitis_ctg62	8248149	8248508	HERVHF_5LTR	-
Cercopithecus_mitis_ctg62	8249049	8250964	HERVHF_pol	-
Cercopithecus_mitis_ctg62	8250608	8251129	HERVHF_pro	-
Cercopithecus_mitis_ctg62	8251438	8251995	HERVHF_gag	-
Cercopithecus_mitis_ctg62	8253408	8253751	HERVHF_3LTR	-
Cercopithecus_mitis_ctg63	11506896	11507340	HERVK_5LTR	+
Cercopithecus_mitis_ctg63	11508972	11509592	HERVK_pro	+
Cercopithecus_mitis_ctg63	11509454	11511247	HERVK_pol	+
Cercopithecus_mitis_ctg63	11511922	11512641	HERVK_env	+
Cercopithecus_mitis_ctg63	11512733	11513162	HERVK_3LTR	+
Cercopithecus_mitis_ctg63	18295973	18296291	HSERVIII_5LTR	-
Cercopithecus_mitis_ctg63	18300864	18303349	HSERVIII_pol	-
Cercopithecus_mitis_ctg63	18309129	18309458	HSERVIII_3LTR	-
Cercopithecus_mitis_ctg7	8155376	8155887	HERVK_5LTR	+
Cercopithecus_mitis_ctg7	8156758	8157570	HERVK_pro	+
Cercopithecus_mitis_ctg7	8157465	8158793	HERVK_pol	+
Cercopithecus_mitis_ctg7	8159691	8160212	HERVK_env	+
Cercopithecus_mitis_ctg7	8160284	8160805	HERVK_3LTR	+
Cercopithecus_mitis_ctg7	13695227	13695617	HERVHF_5LTR	+
Cercopithecus_mitis_ctg7	13697309	13697641	HERVHF_gag	+
Cercopithecus_mitis_ctg7	13697910	13698518	HERVHF_pro	+
Cercopithecus_mitis_ctg7	13698195	13698785	HERVHF_pol	+
Cercopithecus_mitis_ctg7	13700404	13700779	HERVHF_3LTR	+

Cercopithecus_mitis_ctg73	1939556	1939871	HERVHF_5LTR	-
Cercopithecus_mitis_ctg73	1940630	1942603	HERVHF_pol	-
Cercopithecus_mitis_ctg73	1942097	1942795	HERVHF_pro	-
Cercopithecus_mitis_ctg73	1942854	1943294	HERVHF_gag	-
Cercopithecus_mitis_ctg73	1945134	1945447	HERVHF_3LTR	-
Cercopithecus_mitis_ctg76	8461018	8461346	HERVK_5LTR	+
Cercopithecus_mitis_ctg76	8461766	8462317	HERVK_gag	+
Cercopithecus_mitis_ctg76	8463535	8464820	HERVK_pro	+
Cercopithecus_mitis_ctg76	8465996	8468012	HERVK_pol	+
Cercopithecus_mitis_ctg76	8468556	8468861	HERVK_env	+
Cercopithecus_mitis_ctg76	8469441	8469776	HERVK_3LTR	+
Cercopithecus_mitis_ctg9	4561251	4561654	HERVHF_5LTR	-
Cercopithecus_mitis_ctg9	4562439	4564007	HERVHF_pol	-
Cercopithecus_mitis_ctg9	4566396	4566794	HERVHF_3LTR	-
Cercopithecus_mitis_ctg90	2091941	2092298	HERVHF_5LTR	-
Cercopithecus_mitis_ctg90	2092886	2095095	HERVHF_pol	-
Cercopithecus_mitis_ctg90	2095565	2095921	HERVHF_gag	-
Cercopithecus_mitis_ctg90	2097364	2097724	HERVHF_3LTR	-
Cercopithecus_mitis_ctg91	8884233	8884634	HERVHF_5LTR	+
Cercopithecus_mitis_ctg91	8885369	8886690	HERVHF_gag	+
Cercopithecus_mitis_ctg91	8886739	8887329	HERVHF_pro	+
Cercopithecus_mitis_ctg91	8887159	8889857	HERVHF_pol	+
Cercopithecus_mitis_ctg91	8891253	8891638	HERVHF_3LTR	+
Cercopithecus_mitis_ctg94	1825288	1825742	HERVHF_5LTR	+
Cercopithecus_mitis_ctg94	1827249	1828532	HERVHF_pol	+
Cercopithecus_mitis_ctg94	1828719	1829172	HERVHF_3LTR	+
Cercopithecus_mona.fasta_Contig1	63229211	63229571	HERVHF_5LTR	-
Cercopithecus_mona.fasta_Contig1	63230016	63231647	HERVHF_pol	-
Cercopithecus_mona.fasta_Contig1	63231286	63232110	HERVHF_pro	-
Cercopithecus_mona.fasta_Contig1	63234222	63234597	HERVHF_3LTR	-
Cercopithecus_mona.fasta_Contig1	71286507	71286826	HERVHF_5LTR	-
Cercopithecus_mona.fasta_Contig1	71287602	71289318	HERVHF_pol	-
Cercopithecus_mona.fasta_Contig1	71291742	71292056	HERVHF_3LTR	-
Cercopithecus_mona.fasta_Contig107	3200774	3201269	HERVK_5LTR	-
Cercopithecus_mona.fasta_Contig107	3202024	3203956	HERVK_pol	-
Cercopithecus_mona.fasta_Contig107	3203851	3204792	HERVK_pro	-
Cercopithecus_mona.fasta_Contig107	3204668	3206117	HERVK_gag	-
Cercopithecus_mona.fasta_Contig107	3206579	3207052	HERVK_3LTR	-
Cercopithecus_mona.fasta_Contig12	79341	79640	HERVHF_5LTR	+
Cercopithecus_mona.fasta_Contig12	81325	81762	HERVHF_gag	+
Cercopithecus_mona.fasta_Contig12	81845	82591	HERVHF_pro	+
Cercopithecus_mona.fasta_Contig12	82010	83605	HERVHF_pol	+
Cercopithecus_mona.fasta_Contig12	83606	84100	HERVHF_env	+
Cercopithecus_mona.fasta_Contig12	84532	84836	HERVHF_3LTR	+

Cercopithecus_mona.fasta_Contig12	8348008	8348461	HERVHF_5LTR	-
Cercopithecus_mona.fasta_Contig12	8348910	8351184	HERVHF_pol	-
Cercopithecus_mona.fasta_Contig12	8350753	8351328	HERVHF_pro	-
Cercopithecus_mona.fasta_Contig12	8353630	8354082	HERVHF_3LTR	-
Cercopithecus_mona.fasta_Contig12	45163377	45163732	HERVHF_5LTR	+
Cercopithecus_mona.fasta_Contig12	45165096	45165623	HERVHF_gag	+
Cercopithecus_mona.fasta_Contig12	45165747	45167774	HERVHF_pol	+
Cercopithecus_mona.fasta_Contig12	45168700	45169042	HERVHF_3LTR	+
Cercopithecus_mona.fasta_Contig13	35739844	35740242	HERVHF_5LTR	+
Cercopithecus_mona.fasta_Contig13	35742421	35744322	HERVHF_pol	+
Cercopithecus_mona.fasta_Contig13	35744989	35745392	HERVHF_3LTR	+
Cercopithecus_mona.fasta_Contig14	7740641	7741158	HERVK_5LTR	-
Cercopithecus_mona.fasta_Contig14	7741232	7741564	HERVK_env	-
Cercopithecus_mona.fasta_Contig14	7742757	7744016	HERVK_pol	-
Cercopithecus_mona.fasta_Contig14	7743884	7744823	HERVK_pro	-
Cercopithecus_mona.fasta_Contig14	7745574	7746080	HERVK_3LTR	-
Cercopithecus_mona.fasta_Contig14	17200144	17200661	HERVK_5LTR	+
Cercopithecus_mona.fasta_Contig14	17202199	17202966	HERVK_pro	+
Cercopithecus_mona.fasta_Contig14	17202861	17204127	HERVK_pol	+
Cercopithecus_mona.fasta_Contig14	17204619	17204930	HERVK_env	+
Cercopithecus_mona.fasta_Contig14	17205007	17205521	HERVK_3LTR	+
Cercopithecus_mona.fasta_Contig18	9483412	9483738	HERVHF_5LTR	-
Cercopithecus_mona.fasta_Contig18	9484026	9484364	HERVHF_env	-
Cercopithecus_mona.fasta_Contig18	9484672	9485430	HERVHF_pol	-
Cercopithecus_mona.fasta_Contig18	9485690	9486100	HERVHF_gag	-
Cercopithecus_mona.fasta_Contig18	9487655	9487973	HERVHF_3LTR	-
Cercopithecus_mona.fasta_Contig18	29499307	29499666	HERVHF_5LTR	-
Cercopithecus_mona.fasta_Contig18	29500606	29502118	HERVHF_pol	-
Cercopithecus_mona.fasta_Contig18	29501762	29502283	HERVHF_pro	-
Cercopithecus_mona.fasta_Contig18	29502592	29502897	HERVHF_gag	-
Cercopithecus_mona.fasta_Contig18	29504534	29504874	HERVHF_3LTR	-
Cercopithecus_mona.fasta_Contig185	1113487	1113919	HERVHF_5LTR	+
Cercopithecus_mona.fasta_Contig185	1116094	1116609	HERVHF_pro	+
Cercopithecus_mona.fasta_Contig185	1116202	1118273	HERVHF_pol	+
Cercopithecus_mona.fasta_Contig185	1118884	1119240	HERVHF_env	+
Cercopithecus_mona.fasta_Contig185	1119395	1119835	HERVHF_3LTR	+
Cercopithecus_mona.fasta_Contig19	1004738	1005190	HERVHF_5LTR	+
Cercopithecus_mona.fasta_Contig19	1007242	1008024	HERVHF_pro	+
Cercopithecus_mona.fasta_Contig19	1007452	1009072	HERVHF_pol	+
Cercopithecus_mona.fasta_Contig19	1010071	1010527	HERVHF_3LTR	+
Cercopithecus_mona.fasta_Contig23	29600574	29600974	HERVHF_5LTR	-
Cercopithecus_mona.fasta_Contig23	29601564	29603986	HERVHF_pol	-
Cercopithecus_mona.fasta_Contig23	29603444	29604286	HERVHF_pro	-
Cercopithecus_mona.fasta_Contig23	29606112	29606504	HERVHF_3LTR	-

Cercopithecus_mona.fasta_Contig24	19922673	19923178	HERVIPADP_5LTR	+
Cercopithecus_mona.fasta_Contig24	19924751	19925101	HERVIPADP_gag	+
Cercopithecus_mona.fasta_Contig24	19925716	19928523	HERVIPADP_pol	+
Cercopithecus_mona.fasta_Contig24	19930377	19930805	HERVIPADP_env	+
Cercopithecus_mona.fasta_Contig24	19930981	19931486	HERVIPADP_3LTR	+
Cercopithecus_mona.fasta_Contig26	23225035	23225374	HERVK_5LTR	+
Cercopithecus_mona.fasta_Contig26	23226455	23227090	HERVK_gag	+
Cercopithecus_mona.fasta_Contig26	23226958	23227837	HERVK_pro	+
Cercopithecus_mona.fasta_Contig26	23227818	23228537	HERVK_pol	+
Cercopithecus_mona.fasta_Contig26	23229555	23229901	HERVK_3LTR	+
Cercopithecus_mona.fasta_Contig28	3795864	3796532	HERVHF_5LTR	-
Cercopithecus_mona.fasta_Contig28	3796596	3798721	HERVHF_pol	-
Cercopithecus_mona.fasta_Contig28	3798481	3799482	HERVHF_pro	-
Cercopithecus_mona.fasta_Contig28	3801741	3802405	HERVHF_3LTR	-
Cercopithecus_mona.fasta_Contig28	6945127	6945906	HUERSP_5LTR	+
Cercopithecus_mona.fasta_Contig28	6947801	6948268	HUERSP_gag	+
Cercopithecus_mona.fasta_Contig28	6948433	6949083	HUERSP_pro	+
Cercopithecus_mona.fasta_Contig28	6948610	6949236	HUERSP_pol	+
Cercopithecus_mona.fasta_Contig28	6950009	6951727	HUERSP_env	+
Cercopithecus_mona.fasta_Contig28	6955204	6955974	HUERSP_3LTR	+
Cercopithecus_mona.fasta_Contig3	58173002	58173462	HERVHF_5LTR	+
Cercopithecus_mona.fasta_Contig3	58174579	58175007	HERVHF_gag	+
Cercopithecus_mona.fasta_Contig3	58176328	58177227	HERVHF_pol	+
Cercopithecus_mona.fasta_Contig3	58180274	58180870	HERVHF_env	+
Cercopithecus_mona.fasta_Contig3	58181031	58181493	HERVHF_3LTR	+
Cercopithecus_mona.fasta_Contig304	233274	233666	HERVK_5LTR	+
Cercopithecus_mona.fasta_Contig304	235172	236113	HERVK_pro	+
Cercopithecus_mona.fasta_Contig304	236008	238246	HERVK_pol	+
Cercopithecus_mona.fasta_Contig304	239635	240043	HERVK_3LTR	+
Cercopithecus_mona.fasta_Contig36	10555555	10555879	HERVK_5LTR	-
Cercopithecus_mona.fasta_Contig36	10556019	10556933	HERVK_env	-
Cercopithecus_mona.fasta_Contig36	10557272	10559137	HERVK_pol	-
Cercopithecus_mona.fasta_Contig36	10559035	10559811	HERVK_pro	-
Cercopithecus_mona.fasta_Contig36	10561390	10561718	HERVK_3LTR	-
Cercopithecus_mona.fasta_Contig37	4677372	4677779	HERVHF_5LTR	+
Cercopithecus_mona.fasta_Contig37	4679192	4679548	HERVHF_gag	+
Cercopithecus_mona.fasta_Contig37	4681398	4682198	HERVHF_pro	+
Cercopithecus_mona.fasta_Contig37	4681635	4684055	HERVHF_pol	+
Cercopithecus_mona.fasta_Contig37	4684227	4684574	HERVHF_env	+
Cercopithecus_mona.fasta_Contig37	4687787	4688187	HERVHF_3LTR	+
Cercopithecus_mona.fasta_Contig4	22294757	22295210	HERVHF_5LTR	-
Cercopithecus_mona.fasta_Contig4	22295374	22296692	HERVHF_pol	-
Cercopithecus_mona.fasta_Contig4	22298197	22298652	HERVHF_3LTR	-
Cercopithecus_mona.fasta_Contig42	13013620	13014074	HERVHF_5LTR	+

Cercopithecus_mona.fasta_Contig42	13015554	13016159	HERVHF_gag	+
Cercopithecus_mona.fasta_Contig42	13016410	13018756	HERVHF_pol	+
Cercopithecus_mona.fasta_Contig42	13019626	13020079	HERVHF_3LTR	+
Cercopithecus_mona.fasta_Contig47	486885	487335	HERVHF_5LTR	+
Cercopithecus_mona.fasta_Contig47	489008	489349	HERVHF_gag	+
Cercopithecus_mona.fasta_Contig47	489587	490363	HERVHF_pro	+
Cercopithecus_mona.fasta_Contig47	489659	491081	HERVHF_pol	+
Cercopithecus_mona.fasta_Contig47	492138	492589	HERVHF_3LTR	+
Cercopithecus_mona.fasta_Contig49	164199	165028	HERVK_5LTR	+
Cercopithecus_mona.fasta_Contig49	166493	167047	HERVK_pro	+
Cercopithecus_mona.fasta_Contig49	167234	168513	HERVK_pol	+
Cercopithecus_mona.fasta_Contig49	169703	170542	HERVK_3LTR	+
Cercopithecus_mona.fasta_Contig50	7165085	7165392	HERVHF_5LTR	+
Cercopithecus_mona.fasta_Contig50	7165513	7165908	HERVHF_gag	+
Cercopithecus_mona.fasta_Contig50	7166047	7166619	HERVHF_pro	+
Cercopithecus_mona.fasta_Contig50	7166305	7168731	HERVHF_pol	+
Cercopithecus_mona.fasta_Contig50	7168978	7169346	HERVHF_env	+
Cercopithecus_mona.fasta_Contig50	7169696	7170000	HERVHF_3LTR	+
Cercopithecus_mona.fasta_Contig51	3260046	3260448	HERVHF_5LTR	-
Cercopithecus_mona.fasta_Contig51	3261492	3263408	HERVHF_pol	-
Cercopithecus_mona.fasta_Contig51	3263067	3263651	HERVHF_pro	-
Cercopithecus_mona.fasta_Contig51	3263766	3264113	HERVHF_gag	-
Cercopithecus_mona.fasta_Contig51	3265554	3265956	HERVHF_3LTR	-
Cercopithecus_mona.fasta_Contig697	13968	14387	HERVHF_5LTR	-
Cercopithecus_mona.fasta_Contig697	19215	21140	HERVHF_pol	-
Cercopithecus_mona.fasta_Contig697	21258	21914	HERVHF_gag	-
Cercopithecus_mona.fasta_Contig697	26398	26820	HERVHF_3LTR	-
Cercopithecus_mona.fasta_Contig89	3714241	3714623	HERVHF_5LTR	+
Cercopithecus_mona.fasta_Contig89	3716064	3716381	HERVHF_gag	+
Cercopithecus_mona.fasta_Contig89	3717659	3720376	HERVHF_pol	+
Cercopithecus_mona.fasta_Contig89	3720815	3721191	HERVHF_3LTR	+
Cercopithecus_mona.fasta_Contig9	9863128	9863563	HERVHF_5LTR	-
Cercopithecus_mona.fasta_Contig9	9864321	9864899	HERVHF_pol	-
Cercopithecus_mona.fasta_Contig9	9864914	9865246	HERVHF_pro	-
Cercopithecus_mona.fasta_Contig9	9865366	9865814	HERVHF_3LTR	-
Cercopithecus_mona.fasta_Contig94	9312470	9312899	HERVHF_5LTR	+
Cercopithecus_mona.fasta_Contig94	9314462	9314869	HERVHF_gag	+
Cercopithecus_mona.fasta_Contig94	9315298	9317309	HERVHF_pol	+
Cercopithecus_mona.fasta_Contig94	9317671	9319344	HERVHF_env	+
Cercopithecus_mona.fasta_Contig94	9319638	9320084	HERVHF_3LTR	+
Chlorocebus_aethiops.fasta_ctg102	3569049	3569474	HERVHF_5LTR	-
Chlorocebus_aethiops.fasta_ctg102	3570362	3572373	HERVHF_pol	-
Chlorocebus_aethiops.fasta_ctg102	3571840	3572616	HERVHF_pro	-
Chlorocebus_aethiops.fasta_ctg102	3572669	3572989	HERVHF_gag	-

Chlorocebus_aethiops.fasta_ctg102	3574459	3574877	HERVHF_3LTR	-
Chlorocebus_aethiops.fasta_ctg104	2616456	2616964	HERVK_5LTR	+
Chlorocebus_aethiops.fasta_ctg104	2617978	2618481	HERVK_gag	+
Chlorocebus_aethiops.fasta_ctg104	2618608	2619213	HERVK_pro	+
Chlorocebus_aethiops.fasta_ctg104	2619078	2620653	HERVK_pol	+
Chlorocebus_aethiops.fasta_ctg104	2622344	2622840	HERVK_3LTR	+
Chlorocebus_aethiops.fasta_ctg11	9321016	9321342	HERVHF_5LTR	+
Chlorocebus_aethiops.fasta_ctg11	9323664	9325763	HERVHF_pol	+
Chlorocebus_aethiops.fasta_ctg11	9326071	9326392	HERVHF_3LTR	+
Chlorocebus_aethiops.fasta_ctg132	3927254	3927643	HERVHF_5LTR	+
Chlorocebus_aethiops.fasta_ctg132	3929118	3929420	HERVHF_gag	+
Chlorocebus_aethiops.fasta_ctg132	3929452	3930294	HERVHF_pro	+
Chlorocebus_aethiops.fasta_ctg132	3929752	3932173	HERVHF_pol	+
Chlorocebus_aethiops.fasta_ctg132	3932761	3933161	HERVHF_3LTR	+
Chlorocebus_aethiops.fasta_ctg139	487210	487629	HERVHF_5LTR	+
Chlorocebus_aethiops.fasta_ctg139	489110	489442	HERVHF_gag	+
Chlorocebus_aethiops.fasta_ctg139	489456	490265	HERVHF_pro	+
Chlorocebus_aethiops.fasta_ctg139	489636	491364	HERVHF_pol	+
Chlorocebus_aethiops.fasta_ctg139	492188	492613	HERVHF_3LTR	+
Chlorocebus_aethiops.fasta_ctg139	4349838	4350800	HERVK_5LTR	+
Chlorocebus_aethiops.fasta_ctg139	4351499	4352032	HERVK_gag	+
Chlorocebus_aethiops.fasta_ctg139	4352098	4353133	HERVK_pro	+
Chlorocebus_aethiops.fasta_ctg139	4353103	4353723	HERVK_pol	+
Chlorocebus_aethiops.fasta_ctg139	4354177	4355297	HERVK_env	+
Chlorocebus_aethiops.fasta_ctg139	4356223	4357188	HERVK_3LTR	+
Chlorocebus_aethiops.fasta_ctg145	1571057	1571376	HERVHF_5LTR	+
Chlorocebus_aethiops.fasta_ctg145	1572487	1573311	HERVHF_gag	+
Chlorocebus_aethiops.fasta_ctg145	1573715	1574527	HERVHF_pro	+
Chlorocebus_aethiops.fasta_ctg145	1574084	1576236	HERVHF_pol	+
Chlorocebus_aethiops.fasta_ctg145	1578039	1578788	HERVHF_env	+
Chlorocebus_aethiops.fasta_ctg145	1579007	1579327	HERVHF_3LTR	+
Chlorocebus_aethiops.fasta_ctg15	6104075	6104397	HERVHF_5LTR	-
Chlorocebus_aethiops.fasta_ctg15	6105018	6107460	HERVHF_pol	-
Chlorocebus_aethiops.fasta_ctg15	6107678	6108304	HERVHF_pro	-
Chlorocebus_aethiops.fasta_ctg15	6108294	6108629	HERVHF_gag	-
Chlorocebus_aethiops.fasta_ctg15	6109987	6110318	HERVHF_3LTR	-
Chlorocebus_aethiops.fasta_ctg159	2460744	2461194	HERVHF_5LTR	-
Chlorocebus_aethiops.fasta_ctg159	2462074	2463580	HERVHF_pol	-
Chlorocebus_aethiops.fasta_ctg159	2466067	2466519	HERVHF_3LTR	-
Chlorocebus_aethiops.fasta_ctg164	3248402	3248853	HERVHF_5LTR	-
Chlorocebus_aethiops.fasta_ctg164	3249706	3251212	HERVHF_pol	-
Chlorocebus_aethiops.fasta_ctg164	3250805	3251401	HERVHF_pro	-
Chlorocebus_aethiops.fasta_ctg164	3251645	3251974	HERVHF_gag	-
Chlorocebus_aethiops.fasta_ctg164	3253658	3254110	HERVHF_3LTR	-

Chlorocebus_aethiops.fasta_ctg165	2928619	2929482	HERVHF_5LTR	+
Chlorocebus_aethiops.fasta_ctg165	2931212	2931901	HERVHF_pro	+
Chlorocebus_aethiops.fasta_ctg165	2931242	2933561	HERVHF_pol	+
Chlorocebus_aethiops.fasta_ctg165	2939216	2940095	HERVHF_3LTR	+
Chlorocebus_aethiops.fasta_ctg173	4157982	4158356	HERVHF_5LTR	+
Chlorocebus_aethiops.fasta_ctg173	4159781	4160110	HERVHF_gag	+
Chlorocebus_aethiops.fasta_ctg173	4161275	4164154	HERVHF_pol	+
Chlorocebus_aethiops.fasta_ctg173	4164592	4164970	HERVHF_3LTR	+
Chlorocebus_aethiops.fasta_ctg18	6403973	6404371	HERVHF_5LTR	+
Chlorocebus_aethiops.fasta_ctg18	6406380	6406997	HERVHF_pro	+
Chlorocebus_aethiops.fasta_ctg18	6406823	6408432	HERVHF_pol	+
Chlorocebus_aethiops.fasta_ctg18	6409123	6409527	HERVHF_3LTR	+
Chlorocebus_aethiops.fasta_ctg19	18927737	18928118	HERVHF_5LTR	-
Chlorocebus_aethiops.fasta_ctg19	18928338	18929621	HERVHF_pol	-
Chlorocebus_aethiops.fasta_ctg19	18931241	18931609	HERVHF_3LTR	-
Chlorocebus_aethiops.fasta_ctg2	5078446	5078746	HERVHF_5LTR	-
Chlorocebus_aethiops.fasta_ctg2	5086948	5088628	HERVHF_pol	-
Chlorocebus_aethiops.fasta_ctg2	5088338	5089000	HERVHF_pro	-
Chlorocebus_aethiops.fasta_ctg2	5089167	5089634	HERVHF_gag	-
Chlorocebus_aethiops.fasta_ctg2	5091180	5091484	HERVHF_3LTR	-
Chlorocebus_aethiops.fasta_ctg20	11231407	11231857	HERVHF_5LTR	+
Chlorocebus_aethiops.fasta_ctg20	11233545	11233865	HERVHF_gag	+
Chlorocebus_aethiops.fasta_ctg20	11233921	11234697	HERVHF_pro	+
Chlorocebus_aethiops.fasta_ctg20	11234053	11235562	HERVHF_pol	+
Chlorocebus_aethiops.fasta_ctg20	11236414	11236865	HERVHF_3LTR	+
Chlorocebus_aethiops.fasta_ctg202	4661633	4662087	HERVHF_5LTR	-
Chlorocebus_aethiops.fasta_ctg202	4662771	4664679	HERVHF_pol	-
Chlorocebus_aethiops.fasta_ctg202	4666887	4667327	HERVHF_3LTR	-
Chlorocebus_aethiops.fasta_ctg210	1810756	1811075	HERVK_5LTR	+
Chlorocebus_aethiops.fasta_ctg210	1811680	1812228	HERVK_gag	+
Chlorocebus_aethiops.fasta_ctg210	1812957	1813400	HERVK_pro	+
Chlorocebus_aethiops.fasta_ctg210	1813513	1815420	HERVK_pol	+
Chlorocebus_aethiops.fasta_ctg210	1816667	1816982	HERVK_3LTR	+
Chlorocebus_aethiops.fasta_ctg227	2079627	2080004	HERVHF_5LTR	+
Chlorocebus_aethiops.fasta_ctg227	2082194	2082949	HERVHF_pro	+
Chlorocebus_aethiops.fasta_ctg227	2082332	2084085	HERVHF_pol	+
Chlorocebus_aethiops.fasta_ctg227	2084766	2085138	HERVHF_3LTR	+
Chlorocebus_aethiops.fasta_ctg233	3044711	3045174	HERVHF_5LTR	+
Chlorocebus_aethiops.fasta_ctg233	3045922	3047115	HERVHF_gag	+
Chlorocebus_aethiops.fasta_ctg233	3047598	3050190	HERVHF_pol	+
Chlorocebus_aethiops.fasta_ctg233	3050907	3051385	HERVHF_3LTR	+
Chlorocebus_aethiops.fasta_ctg234	2817986	2818387	HERVHF_5LTR	-
Chlorocebus_aethiops.fasta_ctg234	2818624	2818986	HERVHF_env	-
Chlorocebus_aethiops.fasta_ctg234	2819796	2822414	HERVHF_pol	-

Chlorocebus_aethiops.fasta_ctg234	2825131	2825531	HERVHF_3LTR	-
Chlorocebus_aethiops.fasta_ctg26	15520778	15521433	HERVHF_5LTR	-
Chlorocebus_aethiops.fasta_ctg26	15525068	15527486	HERVHF_pol	-
Chlorocebus_aethiops.fasta_ctg26	15526989	15527621	HERVHF_pro	-
Chlorocebus_aethiops.fasta_ctg26	15529489	15530145	HERVHF_3LTR	-
Chlorocebus_aethiops.fasta_ctg279	888263	888642	HERVHF_5LTR	-
Chlorocebus_aethiops.fasta_ctg279	889728	892455	HERVHF_pol	-
Chlorocebus_aethiops.fasta_ctg279	891817	892617	HERVHF_pro	-
Chlorocebus_aethiops.fasta_ctg279	894820	895197	HERVHF_3LTR	-
Chlorocebus_aethiops.fasta_ctg296	1422758	1423151	HERVHF_5LTR	-
Chlorocebus_aethiops.fasta_ctg296	1423839	1426228	HERVHF_pol	-
Chlorocebus_aethiops.fasta_ctg296	1425590	1426321	HERVHF_pro	-
Chlorocebus_aethiops.fasta_ctg296	1426420	1426761	HERVHF_gag	-
Chlorocebus_aethiops.fasta_ctg296	1428350	1428728	HERVHF_3LTR	-
Chlorocebus_aethiops.fasta_ctg311	1397349	1397820	HERVK_5LTR	-
Chlorocebus_aethiops.fasta_ctg311	1399094	1400008	HERVK_pol	-
Chlorocebus_aethiops.fasta_ctg311	1399867	1400544	HERVK_pro	-
Chlorocebus_aethiops.fasta_ctg311	1401634	1402108	HERVK_3LTR	-
Chlorocebus_aethiops.fasta_ctg33	13788944	13789415	HERVK_5LTR	+
Chlorocebus_aethiops.fasta_ctg33	13790363	13791010	HERVK_pro	+
Chlorocebus_aethiops.fasta_ctg33	13791170	13791913	HERVK_pol	+
Chlorocebus_aethiops.fasta_ctg33	13792948	13793337	HERVK_env	+
Chlorocebus_aethiops.fasta_ctg33	13793341	13793821	HERVK_3LTR	+
Chlorocebus_aethiops.fasta_ctg351	1287394	1287716	HERVIPADP_5LTR	-
Chlorocebus_aethiops.fasta_ctg351	1288929	1289432	HERVIPADP_env	-
Chlorocebus_aethiops.fasta_ctg351	1290289	1293948	HERVIPADP_pol	-
Chlorocebus_aethiops.fasta_ctg351	1294437	1294754	HERVIPADP_gag	-
Chlorocebus_aethiops.fasta_ctg351	1296251	1296578	HERVIPADP_3LTR	-
Chlorocebus_aethiops.fasta_ctg365	491533	491966	HERVHF_5LTR	+
Chlorocebus_aethiops.fasta_ctg365	494130	494993	HERVHF_pro	+
Chlorocebus_aethiops.fasta_ctg365	494325	496767	HERVHF_pol	+
Chlorocebus_aethiops.fasta_ctg365	496989	497342	HERVHF_env	+
Chlorocebus_aethiops.fasta_ctg365	497606	498038	HERVHF_3LTR	+
Chlorocebus_aethiops.fasta_ctg37	6210373	6210806	HERVHF_5LTR	+
Chlorocebus_aethiops.fasta_ctg37	6212161	6212484	HERVHF_gag	+
Chlorocebus_aethiops.fasta_ctg37	6212981	6215573	HERVHF_pol	+
Chlorocebus_aethiops.fasta_ctg37	6216168	6216598	HERVHF_3LTR	+
Chlorocebus_aethiops.fasta_ctg370	1320946	1321412	HERVK_5LTR	+
Chlorocebus_aethiops.fasta_ctg370	1321958	1322572	HERVK_gag	+
Chlorocebus_aethiops.fasta_ctg370	1323152	1323811	HERVK_pro	+
Chlorocebus_aethiops.fasta_ctg370	1323691	1325224	HERVK_pol	+
Chlorocebus_aethiops.fasta_ctg370	1326733	1327201	HERVK_3LTR	+
Chlorocebus_aethiops.fasta_ctg392	297736	298130	HERVK_5LTR	+
Chlorocebus_aethiops.fasta_ctg392	299075	299836	HERVK_pro	+

Chlorocebus_aethiops.fasta_ctg392	299889	301805	HERVK_pol	+
Chlorocebus_aethiops.fasta_ctg392	302816	303225	HERVK_3LTR	+
Chlorocebus_aethiops.fasta_ctg4	24809421	24809931	HERVK_5LTR	-
Chlorocebus_aethiops.fasta_ctg4	24810010	24810360	HERVK_env	-
Chlorocebus_aethiops.fasta_ctg4	24810794	24812077	HERVK_pol	-
Chlorocebus_aethiops.fasta_ctg4	24811981	24812737	HERVK_pro	-
Chlorocebus_aethiops.fasta_ctg4	24812856	24813314	HERVK_gag	-
Chlorocebus_aethiops.fasta_ctg4	24814277	24814798	HERVK_3LTR	-
Chlorocebus_aethiops.fasta_ctg4	42159023	42159433	HERVHF_5LTR	+
Chlorocebus_aethiops.fasta_ctg4	42161876	42162469	HERVHF_pro	+
Chlorocebus_aethiops.fasta_ctg4	42161969	42163051	HERVHF_pol	+
Chlorocebus_aethiops.fasta_ctg4	42165310	42165721	HERVHF_3LTR	+
Chlorocebus_aethiops.fasta_ctg40	5910859	5911183	HERVHF_5LTR	-
Chlorocebus_aethiops.fasta_ctg40	5911329	5911691	HERVHF_env	-
Chlorocebus_aethiops.fasta_ctg40	5912974	5914698	HERVHF_pol	-
Chlorocebus_aethiops.fasta_ctg40	5915323	5916432	HERVHF_gag	-
Chlorocebus_aethiops.fasta_ctg40	5917193	5917514	HERVHF_3LTR	-
Chlorocebus_aethiops.fasta_ctg43	4252657	4253089	HERVHF_5LTR	+
Chlorocebus_aethiops.fasta_ctg43	4254601	4254927	HERVHF_gag	+
Chlorocebus_aethiops.fasta_ctg43	4254917	4255402	HERVHF_pro	+
Chlorocebus_aethiops.fasta_ctg43	4255582	4257422	HERVHF_pol	+
Chlorocebus_aethiops.fasta_ctg43	4258224	4258662	HERVHF_3LTR	+
Chlorocebus_aethiops.fasta_ctg49	8657806	8658240	HERVHF_5LTR	-
Chlorocebus_aethiops.fasta_ctg49	8658721	8661189	HERVHF_pol	-
Chlorocebus_aethiops.fasta_ctg49	8661498	8662456	HERVHF_gag	-
Chlorocebus_aethiops.fasta_ctg49	8663392	8663828	HERVHF_3LTR	-
Chlorocebus_aethiops.fasta_ctg51	14094052	14094416	HERVHF_5LTR	-
Chlorocebus_aethiops.fasta_ctg51	14095297	14097789	HERVHF_pol	-
Chlorocebus_aethiops.fasta_ctg51	14097037	14097936	HERVHF_pro	-
Chlorocebus_aethiops.fasta_ctg51	14098580	14098950	HERVHF_3LTR	-
Chlorocebus_aethiops.fasta_ctg51	44688797	44689115	HERVHF_5LTR	+
Chlorocebus_aethiops.fasta_ctg51	44690699	44691046	HERVHF_gag	+
Chlorocebus_aethiops.fasta_ctg51	44691348	44692106	HERVHF_pol	+
Chlorocebus_aethiops.fasta_ctg51	44692414	44692755	HERVHF_env	+
Chlorocebus_aethiops.fasta_ctg51	44693040	44693364	HERVHF_3LTR	+
Chlorocebus_aethiops.fasta_ctg54	1221026	1221458	HERVHF_5LTR	+
Chlorocebus_aethiops.fasta_ctg54	1223519	1223845	HERVHF_gag	+
Chlorocebus_aethiops.fasta_ctg54	1223851	1224363	HERVHF_pro	+
Chlorocebus_aethiops.fasta_ctg54	1226339	1228006	HERVHF_pol	+
Chlorocebus_aethiops.fasta_ctg54	1228835	1229262	HERVHF_3LTR	+
Chlorocebus_aethiops.fasta_ctg58	6848909	6849275	HERVHF_5LTR	+
Chlorocebus_aethiops.fasta_ctg58	6851157	6851927	HERVHF_pro	+
Chlorocebus_aethiops.fasta_ctg58	6851364	6853577	HERVHF_pol	+
Chlorocebus_aethiops.fasta_ctg58	6854481	6854832	HERVHF_3LTR	+

Chlorocebus_aethiops.fasta_ctg58	9677674	9678022	HERVHF_5LTR	-
Chlorocebus_aethiops.fasta_ctg58	9678769	9680650	HERVHF_pol	-
Chlorocebus_aethiops.fasta_ctg58	9680078	9680863	HERVHF_pro	-
Chlorocebus_aethiops.fasta_ctg58	9680915	9681397	HERVHF_gag	-
Chlorocebus_aethiops.fasta_ctg58	9682908	9683263	HERVHF_3LTR	-
Chlorocebus_aethiops.fasta_ctg64	4755886	4756273	HERVHF_5LTR	+
Chlorocebus_aethiops.fasta_ctg64	4757939	4758256	HERVHF_gag	+
Chlorocebus_aethiops.fasta_ctg64	4758273	4758902	HERVHF_pro	+
Chlorocebus_aethiops.fasta_ctg64	4758669	4760141	HERVHF_pol	+
Chlorocebus_aethiops.fasta_ctg64	4761033	4761430	HERVHF_3LTR	+
Chlorocebus_aethiops.fasta_ctg71	22910460	22910890	HERVHF_5LTR	+
Chlorocebus_aethiops.fasta_ctg71	22912243	22912644	HERVHF_gag	+
Chlorocebus_aethiops.fasta_ctg71	22913568	22916242	HERVHF_pol	+
Chlorocebus_aethiops.fasta_ctg71	22915952	22916302	HERVHF_env	+
Chlorocebus_aethiops.fasta_ctg71	22916466	22916892	HERVHF_3LTR	+
Chlorocebus_aethiops.fasta_ctg75	13760343	13760703	HERVHF_5LTR	+
Chlorocebus_aethiops.fasta_ctg75	13762467	13762832	HERVHF_gag	+
Chlorocebus_aethiops.fasta_ctg75	13762822	13763205	HERVHF_pro	+
Chlorocebus_aethiops.fasta_ctg75	13763185	13763838	HERVHF_pol	+
Chlorocebus_aethiops.fasta_ctg75	13764838	13765242	HERVHF_env	+
Chlorocebus_aethiops.fasta_ctg75	13765408	13765783	HERVHF_3LTR	+
Chlorocebus_aethiops.fasta_ctg796	39143	39475	HERVHF_5LTR	+
Chlorocebus_aethiops.fasta_ctg796	40861	41280	HERVHF_gag	+
Chlorocebus_aethiops.fasta_ctg796	41743	43967	HERVHF_pol	+
Chlorocebus_aethiops.fasta_ctg796	45357	45678	HERVHF_3LTR	+
Chlorocebus_aethiops.fasta_ctg88	3482807	3483177	HERVHF_5LTR	-
Chlorocebus_aethiops.fasta_ctg88	3483338	3483721	HERVHF_env	-
Chlorocebus_aethiops.fasta_ctg88	3484204	3486048	HERVHF_pol	-
Chlorocebus_aethiops.fasta_ctg88	3485911	3486783	HERVHF_pro	-
Chlorocebus_aethiops.fasta_ctg88	3486987	3487407	HERVHF_gag	-
Chlorocebus_aethiops.fasta_ctg88	3488744	3489127	HERVHF_3LTR	-
Chlorocebus_aethiops.fasta_ctg88	16714757	16715196	HERVHF_5LTR	-
Chlorocebus_aethiops.fasta_ctg88	16715746	16716129	HERVHF_env	-
Chlorocebus_aethiops.fasta_ctg88	16716130	16717781	HERVHF_pol	-
Chlorocebus_aethiops.fasta_ctg88	16717170	16717928	HERVHF_pro	-
Chlorocebus_aethiops.fasta_ctg88	16719959	16720400	HERVHF_3LTR	-
Chlorocebus_sabaeus_CM001941.2	124554238	124554610	HERVHF_5LTR	+
Chlorocebus_sabaeus_CM001941.2	124556251	124556730	HERVHF_gag	+
Chlorocebus_sabaeus_CM001941.2	124556763	124557185	HERVHF_pro	+
Chlorocebus_sabaeus_CM001941.2	124557097	124558558	HERVHF_pol	+
Chlorocebus_sabaeus_CM001941.2	124559835	124560222	HERVHF_3LTR	+
Chlorocebus_sabaeus_CM001942.1	26759264	26759615	HERVHF_5LTR	-

Chlorocebus_sabaeus_CM001942.1 26760653 26762554 HERVHF_pol -
Chlorocebus_sabaeus_CM001942.1 26762491 26762964 HERVHF_pro -
Chlorocebus_sabaeus_CM001942.1 26764827 26765193 HERVHF_3LTR -
Chlorocebus_sabaeus_CM001942.1 51295834 51296180 HERVHF_5LTR -
Chlorocebus_sabaeus_CM001942.1 51296929 51298810 HERVHF_pol -
Chlorocebus_sabaeus_CM001942.1 51298238 51299023 HERVHF_pro -
Chlorocebus_sabaeus_CM001942.1 51299079 51299494 HERVHF_gag -
Chlorocebus_sabaeus_CM001942.1 51301069 51301424 HERVHF_3LTR -
Chlorocebus_sabaeus_CM001943.2 26067073 26067510 HERVHF_5LTR -
Chlorocebus_sabaeus_CM001943.2 26068101 26070151 HERVHF_pol -
Chlorocebus_sabaeus_CM001943.2 26069980 26070816 HERVHF_pro -
Chlorocebus_sabaeus_CM001943.2 26070809 26071129 HERVHF_gag -
Chlorocebus_sabaeus_CM001943.2 26072618 26073051 HERVHF_3LTR -
Chlorocebus_sabaeus_CM001943.2 61191237 61191573 HERVK_5LTR +
Chlorocebus_sabaeus_CM001943.2 61193346 61194098 HERVK_pro +
Chlorocebus_sabaeus_CM001943.2 61193993 61194778 HERVK_pol +
Chlorocebus_sabaeus_CM001943.2 61196025 61196360 HERVK_3LTR +
Chlorocebus_sabaeus_CM001943.2 65314867 65315331 HERVHF_5LTR -
Chlorocebus_sabaeus_CM001943.2 65315516 65316316 HERVHF_env -
Chlorocebus_sabaeus_CM001943.2 65316969 65317598 HERVHF_pol -
Chlorocebus_sabaeus_CM001943.2 65317032 65317658 HERVHF_pro -
Chlorocebus_sabaeus_CM001943.2 65318382 65318831 HERVHF_gag -
Chlorocebus_sabaeus_CM001943.2 65319826 65320292 HERVHF_3LTR -
Chlorocebus_sabaeus_CM001946.1 49129441 49129755 HERVK_5LTR -
Chlorocebus_sabaeus_CM001946.1 49130993 49132870 HERVK_pol -
Chlorocebus_sabaeus_CM001946.1 49133027 49133455 HERVK_pro -
Chlorocebus_sabaeus_CM001946.1 49134049 49134528 HERVK_gag -
Chlorocebus_sabaeus_CM001946.1 49135335 49135656 HERVK_3LTR -
Chlorocebus_sabaeus_CM001947.1 22397295 22397609 HERVK_5LTR +
Chlorocebus_sabaeus_CM001947.1 22399875 22402393 HERVK_pol +
Chlorocebus_sabaeus_CM001947.1 22403889 22404509 HERVK_env +
Chlorocebus_sabaeus_CM001947.1 22404565 22404872 HERVK_3LTR +
Chlorocebus_sabaeus_CM001947.1 79412161 79412579 HERVK_5LTR -
Chlorocebus_sabaeus_CM001947.1 79413507 79415407 HERVK_pol -
Chlorocebus_sabaeus_CM001947.1 79415387 79416241 HERVK_pro -
Chlorocebus_sabaeus_CM001947.1 79417718 79418139 HERVK_3LTR -
Chlorocebus_sabaeus_CM001949.2 32139244 32139609 HERVHF_5LTR +
Chlorocebus_sabaeus_CM001949.2 32140288 32141444 HERVHF_gag +
Chlorocebus_sabaeus_CM001949.2 32141979 32143066 HERVHF_pol +
Chlorocebus_sabaeus_CM001949.2 32143498 32143861 HERVHF_3LTR +
Chlorocebus_sabaeus_CM001950.2 10649785 10650281 HERVK_5LTR -
Chlorocebus_sabaeus_CM001950.2 10650288 10650626 HERVK_env -
Chlorocebus_sabaeus_CM001950.2 10651737 10653357 HERVK_pol -
Chlorocebus_sabaeus_CM001950.2 10653435 10653983 HERVK_pro -

Chlorocebus_sabaeus_CM001950.2	10655618	10656126	HERVK_3LTR	-
Chlorocebus_sabaeus_CM001950.2	52581101	52581510	HERVK_5LTR	-
Chlorocebus_sabaeus_CM001950.2	52581666	52582412	HERVK_env	-
Chlorocebus_sabaeus_CM001950.2	52582523	52584456	HERVK_pol	-
Chlorocebus_sabaeus_CM001950.2	52584351	52585271	HERVK_pro	-
Chlorocebus_sabaeus_CM001950.2	52586213	52586607	HERVK_3LTR	-
Chlorocebus_sabaeus_CM001951.2	59688168	59688513	HSERVIII_5LTR	-
Chlorocebus_sabaeus_CM001951.2	59688940	59692107	HSERVIII_pol	-
Chlorocebus_sabaeus_CM001951.2	59694139	59694484	HSERVIII_3LTR	-
Chlorocebus_sabaeus_CM001951.2	77116245	77116587	HERVHF_5LTR	+
Chlorocebus_sabaeus_CM001951.2	77118630	77119624	HERVHF_pro	+
Chlorocebus_sabaeus_CM001951.2	77118815	77121868	HERVHF_pol	+
Chlorocebus_sabaeus_CM001951.2	77123183	77123788	HERVHF_env	+
Chlorocebus_sabaeus_CM001951.2	77124099	77124451	HERVHF_3LTR	+
Chlorocebus_sabaeus_CM001952.2	35808120	35808472	HERVHF_5LTR	+
Chlorocebus_sabaeus_CM001952.2	35810252	35810581	HERVHF_gag	+
Chlorocebus_sabaeus_CM001952.2	35810879	35812520	HERVHF_pol	+
Chlorocebus_sabaeus_CM001952.2	35813371	35813735	HERVHF_3LTR	+
Chlorocebus_sabaeus_CM001952.2	71846023	71846344	HERVHF_5LTR	-
Chlorocebus_sabaeus_CM001952.2	71846654	71848753	HERVHF_pol	-
Chlorocebus_sabaeus_CM001952.2	71851073	71851399	HERVHF_3LTR	-
Chlorocebus_sabaeus_CM001953.2	4824692	4825093	HERVHF_5LTR	-
Chlorocebus_sabaeus_CM001953.2	4825378	4825899	HERVHF_env	-
Chlorocebus_sabaeus_CM001953.2	4825991	4829203	HERVHF_pol	-
Chlorocebus_sabaeus_CM001953.2	4828844	4829269	HERVHF_pro	-
Chlorocebus_sabaeus_CM001953.2	4829955	4830296	HERVHF_gag	-
Chlorocebus_sabaeus_CM001953.2	4831915	4832315	HERVHF_3LTR	-
Chlorocebus_sabaeus_CM001954.1	50219255	50219679	HERVHF_5LTR	-
Chlorocebus_sabaeus_CM001954.1	50220095	50222232	HERVHF_pol	-
Chlorocebus_sabaeus_CM001954.1	50221603	50222412	HERVHF_pro	-
Chlorocebus_sabaeus_CM001954.1	50222494	50222832	HERVHF_gag	-
Chlorocebus_sabaeus_CM001954.1	50224232	50224651	HERVHF_3LTR	-
Chlorocebus_sabaeus_CM001954.1	61922288	61922648	HERVHF_5LTR	+
Chlorocebus_sabaeus_CM001954.1	61924720	61925481	HERVHF_pro	+
Chlorocebus_sabaeus_CM001954.1	61924900	61926691	HERVHF_pol	+
Chlorocebus_sabaeus_CM001954.1	61927297	61927659	HERVHF_3LTR	+
Chlorocebus_sabaeus_CM001955.2	1848999	1849361	HERVHF_5LTR	-
Chlorocebus_sabaeus_CM001955.2	1850054	1851820	HERVHF_pol	-
Chlorocebus_sabaeus_CM001955.2	1851239	1852018	HERVHF_pro	-
Chlorocebus_sabaeus_CM001955.2	1854198	1854561	HERVHF_3LTR	-
Chlorocebus_sabaeus_CM001955.2	34015812	34016174	HERVHF_5LTR	-
Chlorocebus_sabaeus_CM001955.2	34017073	34019508	HERVHF_pol	-
Chlorocebus_sabaeus_CM001955.2	34018966	34019706	HERVHF_pro	-
Chlorocebus_sabaeus_CM001955.2	34020338	34020708	HERVHF_3LTR	-

Chlorocebus_sabaeus_CM001955.2	64275207	64275525	HERVHF_5LTR	+
Chlorocebus_sabaeus_CM001955.2	64277290	64277657	HERVHF_gag	+
Chlorocebus_sabaeus_CM001955.2	64277939	64278697	HERVHF_pol	+
Chlorocebus_sabaeus_CM001955.2	64279005	64279343	HERVHF_env	+
Chlorocebus_sabaeus_CM001955.2	64279635	64279959	HERVHF_3LTR	+
Chlorocebus_sabaeus_CM001956.2	75902540	75902990	HERVHF_5LTR	-
Chlorocebus_sabaeus_CM001956.2	75903650	75905377	HERVHF_pol	-
Chlorocebus_sabaeus_CM001956.2	75905696	75906016	HERVHF_gag	-
Chlorocebus_sabaeus_CM001956.2	75908022	75908474	HERVHF_3LTR	-
Chlorocebus_sabaeus_CM001958.2	8203185	8203603	HERVHF_5LTR	+
Chlorocebus_sabaeus_CM001958.2	8204950	8205393	HERVHF_gag	+
Chlorocebus_sabaeus_CM001958.2	8205449	8206225	HERVHF_pro	+
Chlorocebus_sabaeus_CM001958.2	8205689	8207748	HERVHF_pol	+
Chlorocebus_sabaeus_CM001958.2	8208620	8209045	HERVHF_3LTR	+
Chlorocebus_sabaeus_CM001959.2	45510761	45511215	HERVHF_5LTR	-
Chlorocebus_sabaeus_CM001959.2	45511899	45513717	HERVHF_pol	-
Chlorocebus_sabaeus_CM001959.2	45513874	45514227	HERVHF_pro	-
Chlorocebus_sabaeus_CM001959.2	45516073	45516513	HERVHF_3LTR	-
Chlorocebus_sabaeus_CM001961.2	55726990	55727340	HERVK_5LTR	+
Chlorocebus_sabaeus_CM001961.2	55727724	55728251	HERVK_gag	+
Chlorocebus_sabaeus_CM001961.2	55728758	55729494	HERVK_pro	+
Chlorocebus_sabaeus_CM001961.2	55729583	55731488	HERVK_pol	+
Chlorocebus_sabaeus_CM001961.2	55732465	55732800	HERVK_3LTR	+
Chlorocebus_sabaeus_CM001961.2	119078011	119078327	HERVIPADP_5LTR	+
Chlorocebus_sabaeus_CM001961.2	119085638	119085955	HERVIPADP_gag	+
Chlorocebus_sabaeus_CM001961.2	119086444	119090092	HERVIPADP_pol	+
Chlorocebus_sabaeus_CM001961.2	119090985	119091464	HERVIPADP_env	+
Chlorocebus_sabaeus_CM001961.2	119091860	119092169	HERVIPADP_3LTR	+
Chlorocebus_sabaeus_CM001962.2	2129070	2129536	HERVK_5LTR	+
Chlorocebus_sabaeus_CM001962.2	2130082	2130696	HERVK_gag	+
Chlorocebus_sabaeus_CM001962.2	2131276	2131935	HERVK_pro	+
Chlorocebus_sabaeus_CM001962.2	2131896	2133375	HERVK_pol	+
Chlorocebus_sabaeus_CM001962.2	2134853	2135321	HERVK_3LTR	+
Chlorocebus_sabaeus_CM001962.2	61959408	61959819	HERVHF_5LTR	-
Chlorocebus_sabaeus_CM001962.2	61961229	61963119	HERVHF_pol	-
Chlorocebus_sabaeus_CM001962.2	61962637	61963257	HERVHF_pro	-
Chlorocebus_sabaeus_CM001962.2	61965740	61966150	HERVHF_3LTR	-
Chlorocebus_sabaeus_CM001962.2	64630992	64631446	HERVHF_5LTR	-
Chlorocebus_sabaeus_CM001962.2	64631887	64633650	HERVHF_pol	-

Chlorocebus_sabaeus_CM001962.2	64633357	64634004	HERVHF_pro	-
Chlorocebus_sabaeus_CM001962.2	64634273	64634605	HERVHF_gag	-
Chlorocebus_sabaeus_CM001962.2	64636282	64636736	HERVHF_3LTR	-
Chlorocebus_sabaeus_CM001962.2	78895927	78896448	HERVK_5LTR	+
Chlorocebus_sabaeus_CM001962.2	78896606	78897870	HERVK_gag	+
Chlorocebus_sabaeus_CM001962.2	78897986	78898405	HERVK_pro	+
Chlorocebus_sabaeus_CM001962.2	78898620	78900002	HERVK_pol	+
Chlorocebus_sabaeus_CM001962.2	78900085	78900714	HERVK_env	+
Chlorocebus_sabaeus_CM001962.2	78900790	78901301	HERVK_3LTR	+
Chlorocebus_sabaeus_CM001963.2	7782589	7783100	HERVK_5LTR	-
Chlorocebus_sabaeus_CM001963.2	7783181	7783615	HERVK_env	-
Chlorocebus_sabaeus_CM001963.2	7784603	7785958	HERVK_pol	-
Chlorocebus_sabaeus_CM001963.2	7785853	7786866	HERVK_pro	-
Chlorocebus_sabaeus_CM001963.2	7788206	7788731	HERVK_3LTR	-
Chlorocebus_sabaeus_CM001964.1	64596469	64596949	HERVK_5LTR	-
Chlorocebus_sabaeus_CM001964.1	64598415	64599125	HERVK_pol	-
Chlorocebus_sabaeus_CM001964.1	64599267	64599929	HERVK_pro	-
Chlorocebus_sabaeus_CM001964.1	64599564	64600106	HERVK_gag	-
Chlorocebus_sabaeus_CM001964.1	64600873	64601344	HERVK_3LTR	-
Chlorocebus_sabaeus_CM001965.2	70373149	70373539	HERVIPADP_5LTR	-
Chlorocebus_sabaeus_CM001965.2	70374006	70374632	HERVIPADP_env	-
Chlorocebus_sabaeus_CM001965.2	70376824	70378861	HERVIPADP_pol	-
Chlorocebus_sabaeus_CM001965.2	70381991	70382372	HERVIPADP_3LTR	-
Chlorocebus_sabaeus_CM001965.2	79276322	79276722	HERVHF_5LTR	-
Chlorocebus_sabaeus_CM001965.2	79277313	79279734	HERVHF_pol	-
Chlorocebus_sabaeus_CM001965.2	79279192	79280034	HERVHF_pro	-
Chlorocebus_sabaeus_CM001965.2	79280066	79280594	HERVHF_gag	-
Chlorocebus_sabaeus_CM001965.2	79281841	79282230	HERVHF_3LTR	-
Chlorocebus_sabaeus_CM001966.2	5193014	5193681	HERVHF_5LTR	+
Chlorocebus_sabaeus_CM001966.2	5195676	5196281	HERVHF_pro	+
Chlorocebus_sabaeus_CM001966.2	5195679	5198143	HERVHF_pol	+
Chlorocebus_sabaeus_CM001966.2	5201733	5202406	HERVHF_3LTR	+
Chlorocebus_sabaeus_CM001966.2	23450404	23450922	HERVK_5LTR	-
Chlorocebus_sabaeus_CM001966.2	23452026	23453114	HERVK_pol	-
Chlorocebus_sabaeus_CM001966.2	23452976	23453945	HERVK_pro	-
Chlorocebus_sabaeus_CM001966.2	23454394	23454882	HERVK_gag	-
Chlorocebus_sabaeus_CM001966.2	23455361	23455875	HERVK_3LTR	-
Chlorocebus_sabaeus_CM001966.2	32753171	32753575	HERVHF_5LTR	-
Chlorocebus_sabaeus_CM001966.2	32754241	32755878	HERVHF_pol	-
Chlorocebus_sabaeus_CM001966.2	32755704	32756321	HERVHF_pro	-
Chlorocebus_sabaeus_CM001966.2	32758328	32758727	HERVHF_3LTR	-
Chlorocebus_sabaeus_CM001966.2	54000152	54000539	HERVK_5LTR	+
Chlorocebus_sabaeus_CM001966.2	54000794	54002067	HERVK_gag	+
Chlorocebus_sabaeus_CM001966.2	54002440	54002952	HERVK_pro	+

Chlorocebus_sabaeus_CM001966.2	54002847	54004789	HERVK_pol	+
Chlorocebus_sabaeus_CM001966.2	54005247	54005590	HERVK_env	+
Chlorocebus_sabaeus_CM001966.2	54005667	54006048	HERVK_3LTR	+
Chlorocebus_sabaeus_CM001967.1	9129471	9129795	HERVHF_5LTR	-
Chlorocebus_sabaeus_CM001967.1	9130398	9132337	HERVHF_pol	-
Chlorocebus_sabaeus_CM001967.1	9132887	9134033	HERVHF_gag	-
Chlorocebus_sabaeus_CM001967.1	9134807	9135145	HERVHF_3LTR	-
Chlorocebus_sabaeus_CM001970.2	2945325	2945719	HERVHF_5LTR	-
Chlorocebus_sabaeus_CM001970.2	2946560	2948712	HERVHF_pol	-
Chlorocebus_sabaeus_CM001970.2	2948170	2948901	HERVHF_pro	-
Chlorocebus_sabaeus_CM001970.2	2949000	2949341	HERVHF_gag	-
Chlorocebus_sabaeus_CM001970.2	2950915	2951308	HERVHF_3LTR	-
Chlorocebus_sabaeus_KE148142.1	17949	18373	HERVHF_5LTR	+
Chlorocebus_sabaeus_KE148142.1	19666	20157	HERVHF_gag	+
Chlorocebus_sabaeus_KE148142.1	20520	21341	HERVHF_pro	+
Chlorocebus_sabaeus_KE148142.1	20805	22683	HERVHF_pol	+
Chlorocebus_sabaeus_KE148142.1	23709	24124	HERVHF_3LTR	+
Colobus_angolensis_palliatus_KN979419.1	6617190	6617504	HERVK_5LTR	+
Colobus_angolensis_palliatus_KN979419.1	6618819	6619604	HERVK_pro	+
Colobus_angolensis_palliatus_KN979419.1	6619499	6620236	HERVK_pol	+
Colobus_angolensis_palliatus_KN979419.1	6621109	6621459	HERVK_env	+
Colobus_angolensis_palliatus_KN979419.1	6621564	6621872	HERVK_3LTR	+
Colobus_angolensis_palliatus_KN979619.1	5072265	5072679	HERVHF_5LTR	+
Colobus_angolensis_palliatus_KN979619.1	5074730	5075146	HERVHF_gag	+
Colobus_angolensis_palliatus_KN979619.1	5075230	5076009	HERVHF_pro	+
Colobus_angolensis_palliatus_KN979619.1	5075341	5077256	HERVHF_pol	+
Colobus_angolensis_palliatus_KN979619.1	5077914	5078492	HERVHF_env	+
Colobus_angolensis_palliatus_KN979619.1	5078643	5079048	HERVHF_3LTR	+
Colobus_angolensis_palliatus_KN979652.1	4988609	4988932	HERVHF_5LTR	+
Colobus_angolensis_palliatus_KN979652.1	4990391	4990993	HERVHF_gag	+
Colobus_angolensis_palliatus_KN979652.1	4991030	4991488	HERVHF_pro	+
Colobus_angolensis_palliatus_KN979652.1	4991571	4993799	HERVHF_pol	+
Colobus_angolensis_palliatus_KN979652.1	4994771	4995163	HERVHF_env	+
Colobus_angolensis_palliatus_KN979652.1	4995408	4995734	HERVHF_3LTR	+
Colobus_angolensis_palliatus_KN979885.1	7515557	7516011	HERVHF_5LTR	-
Colobus_angolensis_palliatus_KN979885.1	7516435	7518248	HERVHF_pol	-
Colobus_angolensis_palliatus_KN979885.1	7521124	7521585	HERVHF_3LTR	-
Colobus_angolensis_palliatus_KN979940.1	6231554	6231884	HERVHF_5LTR	+

Colobus_angolensis_palliatus_KN979940.1	6233662	6233985	HERVHF_gag	+
Colobus_angolensis_palliatus_KN979940.1	6234282	6234809	HERVHF_pol	+
Colobus_angolensis_palliatus_KN979940.1	6235195	6235557	HERVHF_env	+
Colobus_angolensis_palliatus_KN979940.1	6235807	6236139	HERVHF_3LTR	+
Colobus_angolensis_palliatus_KN980051.1	8647365	8647775	HERVHF_5LTR	+
Colobus_angolensis_palliatus_KN980051.1	8649262	8649585	HERVHF_gag	+
Colobus_angolensis_palliatus_KN980051.1	8649636	8650058	HERVHF_pro	+
Colobus_angolensis_palliatus_KN980051.1	8650069	8652691	HERVHF_pol	+
Colobus_angolensis_palliatus_KN980051.1	8652789	8653144	HERVHF_env	+
Colobus_angolensis_palliatus_KN980051.1	8653308	8653726	HERVHF_3LTR	+
Colobus_angolensis_palliatus_KN980519.1	1997855	1998185	HERVHF_5LTR	+
Colobus_angolensis_palliatus_KN980519.1	1999088	1999684	HERVHF_pro	+
Colobus_angolensis_palliatus_KN980519.1	1999594	2001072	HERVHF_pol	+
Colobus_angolensis_palliatus_KN980519.1	2002118	2002487	HERVHF_env	+
Colobus_angolensis_palliatus_KN980519.1	2002580	2002906	HERVHF_3LTR	+
Colobus_angolensis_palliatus_KN980818.1	3164069	3164474	HERVHF_5LTR	+
Colobus_angolensis_palliatus_KN980818.1	3165785	3166273	HERVHF_gag	+
Colobus_angolensis_palliatus_KN980818.1	3166775	3169125	HERVHF_pol	+
Colobus_angolensis_palliatus_KN980818.1	3169724	3170126	HERVHF_3LTR	+
Colobus_angolensis_palliatus_KN980841.1	2205788	2206243	HERVHF_5LTR	+
Colobus_angolensis_palliatus_KN980841.1	2208401	2210464	HERVHF_pol	+
Colobus_angolensis_palliatus_KN980841.1	2210874	2211310	HERVHF_3LTR	+
Colobus_angolensis_palliatus_KN981104.1	2271492	2271892	HERVHF_5LTR	-
Colobus_angolensis_palliatus_KN981104.1	2272103	2273362	HERVHF_pol	-
Colobus_angolensis_palliatus_KN981104.1	2275980	2276384	HERVHF_3LTR	-
Colobus_angolensis_palliatus_KN981247.1	2898111	2898509	HERVHF_5LTR	+
Colobus_angolensis_palliatus_KN981247.1	2899885	2900619	HERVHF_gag	+
Colobus_angolensis_palliatus_KN981247.1	2900637	2901284	HERVHF_pro	+
Colobus_angolensis_palliatus_KN981247.1	2900682	2902657	HERVHF_pol	+
Colobus_angolensis_palliatus_KN981247.1	2903193	2903587	HERVHF_3LTR	+

Colobus_angolensis_palliatus_KN981446.1	1416715	1417208	HERVIPADP_5LTR	-
Colobus_angolensis_palliatus_KN981446.1	1417323	1417760	HERVIPADP_env	-
Colobus_angolensis_palliatus_KN981446.1	1419470	1422116	HERVIPADP_pol	-
Colobus_angolensis_palliatus_KN981446.1	1422859	1423260	HERVIPADP_gag	-
Colobus_angolensis_palliatus_KN981446.1	1424891	1425384	HERVIPADP_3LTR	-
Colobus_angolensis_palliatus_KN981891.1	2834817	2835147	HERVHF_5LTR	+
Colobus_angolensis_palliatus_KN981891.1	2836507	2837156	HERVHF_gag	+
Colobus_angolensis_palliatus_KN981891.1	2837631	2838389	HERVHF_pro	+
Colobus_angolensis_palliatus_KN981891.1	2837763	2840116	HERVHF_pol	+
Colobus_angolensis_palliatus_KN981891.1	2840835	2841166	HERVHF_3LTR	+
Colobus_angolensis_palliatus_KN982682.1	419603	420002	HERVHF_5LTR	-
Colobus_angolensis_palliatus_KN982682.1	420649	421230	HERVHF_pol	-
Colobus_angolensis_palliatus_KN982682.1	420847	421647	HERVHF_pro	-
Colobus_angolensis_palliatus_KN982682.1	421844	422290	HERVHF_gag	-
Colobus_angolensis_palliatus_KN982682.1	423100	423506	HERVHF_3LTR	-
Colobus_angolensis_palliatus_KN983036.1	529720	530152	HERVHF_5LTR	+
Colobus_angolensis_palliatus_KN983036.1	531537	531839	HERVHF_gag	+
Colobus_angolensis_palliatus_KN983036.1	532002	532802	HERVHF_pro	+
Colobus_angolensis_palliatus_KN983036.1	532245	533730	HERVHF_pol	+
Colobus_angolensis_palliatus_KN983036.1	534817	535254	HERVHF_3LTR	+
Colobus_angolensis_palliatus_KN983046.1	1378365	1378773	HERVHF_5LTR	+
Colobus_angolensis_palliatus_KN983046.1	1380184	1380534	HERVHF_gag	+
Colobus_angolensis_palliatus_KN983046.1	1380683	1381459	HERVHF_pro	+
Colobus_angolensis_palliatus_KN983046.1	1380920	1383354	HERVHF_pol	+
Colobus_angolensis_palliatus_KN983046.1	1383800	1384220	HERVHF_3LTR	+
Colobus_angolensis_palliatus_KN984364.1	7370958	7371365	HERVHF_5LTR	-
Colobus_angolensis_palliatus_KN984364.1	7371847	7373816	HERVHF_pol	-
Colobus_angolensis_palliatus_KN984364.1	7374368	7374841	HERVHF_gag	-
Colobus_angolensis_palliatus_KN984364.1	7376188	7376587	HERVHF_3LTR	-

Colobus_angolensis_palliatus_KN984673.1	3655456	3655820	HERVHF_5LTR	-
Colobus_angolensis_palliatus_KN984673.1	3657023	3658580	HERVHF_pol	-
Colobus_angolensis_palliatus_KN984673.1	3658185	3658811	HERVHF_pro	-
Colobus_angolensis_palliatus_KN984673.1	3659006	3659326	HERVHF_gag	-
Colobus_angolensis_palliatus_KN984673.1	3661192	3661559	HERVHF_3LTR	-
Colobus_angolensis_palliatus_KN985382.1	527	959	HERVK_5LTR	-
Colobus_angolensis_palliatus_KN985382.1	2471	3920	HERVK_pol	-
Colobus_angolensis_palliatus_KN985382.1	3911	4834	HERVK_pro	-
Colobus_angolensis_palliatus_KN985382.1	6323	6756	HERVK_3LTR	-
Colobus_angolensis_palliatus_KN985685.1	300794	301251	HERVHF_5LTR	+
Colobus_angolensis_palliatus_KN985685.1	303799	304380	HERVHF_pro	+
Colobus_angolensis_palliatus_KN985685.1	303856	305531	HERVHF_pol	+
Colobus_angolensis_palliatus_KN985685.1	306341	306796	HERVHF_3LTR	+
Colobus_angolensis_palliatus_KN985700.1	85869	86282	HERVHF_5LTR	-
Colobus_angolensis_palliatus_KN985700.1	86982	89410	HERVHF_pol	-
Colobus_angolensis_palliatus_KN985700.1	88772	89476	HERVHF_pro	-
Colobus_angolensis_palliatus_KN985700.1	89715	90179	HERVHF_gag	-
Colobus_angolensis_palliatus_KN985700.1	91492	91905	HERVHF_3LTR	-
Colobus_angolensis_palliatus_KN985711.1	8836491	8836926	HERVHF_5LTR	+
Colobus_angolensis_palliatus_KN985711.1	8838437	8838796	HERVHF_gag	+
Colobus_angolensis_palliatus_KN985711.1	8839170	8841578	HERVHF_pol	+
Colobus_angolensis_palliatus_KN985711.1	8842039	8842472	HERVHF_3LTR	+
Colobus_angolensis_palliatus_KN985737.1	11540	11890	HERVIPADP_5LTR	-
Colobus_angolensis_palliatus_KN985737.1	12172	12474	HERVIPADP_env	-
Colobus_angolensis_palliatus_KN985737.1	16113	19009	HERVIPADP_pol	-
Colobus_angolensis_palliatus_KN985737.1	20421	20999	HERVIPADP_gag	-
Colobus_angolensis_palliatus_KN985737.1	22153	22509	HERVIPADP_3LTR	-
Colobus_angolensis_palliatus_KN985934.1	384429	384788	HERVHF_5LTR	+
Colobus_angolensis_palliatus_KN985934.1	387291	389066	HERVHF_pol	+
Colobus_angolensis_palliatus_KN985934.1	389686	391262	HERVHF_env	+

Colobus_angolensis_palliatus_KN985934.1	391552	391932	HERVHF_3LTR	+
Colobus_guereza_ContigUN	2254126	2254435	HERVHF_5LTR	+
Colobus_guereza_ContigUN	2256065	2256535	HERVHF_gag	+
Colobus_guereza_ContigUN	2256717	2257442	HERVHF_pro	+
Colobus_guereza_ContigUN	2256927	2258264	HERVHF_pol	+
Colobus_guereza_ContigUN	2258836	2259150	HERVHF_3LTR	+
Colobus_guereza_LG01	177203219	177203552	HERVHF_5LTR	+
Colobus_guereza_LG01	177205725	177206522	HERVHF_pro	+
Colobus_guereza_LG01	177205959	177208337	HERVHF_pol	+
Colobus_guereza_LG01	177209387	177209730	HERVHF_3LTR	+
Colobus_guereza_LG02	22064195	22064945	HSERVIII_5LTR	+
Colobus_guereza_LG02	22066858	22067553	HSERVIII_pol	+
Colobus_guereza_LG02	22072181	22072939	HSERVIII_3LTR	+
Colobus_guereza_LG03	168855231	168855631	HERVHF_5LTR	-
Colobus_guereza_LG03	168856221	168858460	HERVHF_pol	-
Colobus_guereza_LG03	168860770	168861166	HERVHF_3LTR	-
Colobus_guereza_LG04	24358834	24359270	HERVHF_5LTR	-
Colobus_guereza_LG04	24359971	24362043	HERVHF_pol	-
Colobus_guereza_LG04	24364190	24364645	HERVHF_3LTR	-
Colobus_guereza_LG04	30649087	30649397	Unknown_HERV_5LTR	-
Colobus_guereza_LG04	30650044	30656434	Unknown_HERV_pol	-
Colobus_guereza_LG04	30660744	30661078	Unknown_HERV_3LTR	-
Colobus_guereza_LG04	45132011	45132470	HERVHF_5LTR	-
Colobus_guereza_LG04	45132899	45134746	HERVHF_pol	-
Colobus_guereza_LG04	45134695	45135210	HERVHF_pro	-
Colobus_guereza_LG04	45137174	45137639	HERVHF_3LTR	-
Colobus_guereza_LG04	141627966	141628375	HERVIPADP_5LTR	+
Colobus_guereza_LG04	141630830	141633954	HERVIPADP_pol	+
Colobus_guereza_LG04	141636129	141636626	HERVIPADP_env	+
Colobus_guereza_LG04	141636866	141637268	HERVIPADP_3LTR	+
Colobus_guereza_LG07	30551128	30551449	HERVHF_5LTR	-
Colobus_guereza_LG07	30552142	30554356	HERVHF_pol	-
Colobus_guereza_LG07	30554165	30554647	HERVHF_pro	-
Colobus_guereza_LG07	30556713	30557020	HERVHF_3LTR	-
Colobus_guereza_LG07	58247766	58248224	HERVHF_5LTR	-
Colobus_guereza_LG07	58250624	58252550	HERVHF_pol	-
Colobus_guereza_LG07	58251915	58252691	HERVHF_pro	-
Colobus_guereza_LG07	58255186	58255572	HERVHF_gag	-
Colobus_guereza_LG07	58256922	58257379	HERVHF_3LTR	-
Colobus_guereza_LG07	65917767	65918754	HSERVIII_5LTR	+
Colobus_guereza_LG07	65919280	65919747	HSERVIII_pol	+
Colobus_guereza_LG07	65920215	65921207	HSERVIII_3LTR	+
Colobus_guereza_LG07	94959881	94960224	HERVHF_5LTR	+

Colobus_guereza_LG07	94966045	94966392	HERVHF_gag	+
Colobus_guereza_LG07	94966634	94967413	HERVHF_pro	+
Colobus_guereza_LG07	94966871	94969114	HERVHF_pol	+
Colobus_guereza_LG07	94969189	94969527	HERVHF_3LTR	+
Colobus_guereza_LG09	93831066	93831523	Unknown_HERV_5LTR	+
Colobus_guereza_LG09	93833573	93834424	Unknown_HERV_pro	+
Colobus_guereza_LG09	93833765	93836059	Unknown_HERV_pol	+
Colobus_guereza_LG09	93836584	93837039	Unknown_HERV_3LTR	+
Colobus_guereza_LG09	100538305	100538710	HERVHF_5LTR	-
Colobus_guereza_LG09	100538863	100539441	HERVHF_env	-
Colobus_guereza_LG09	100540051	100541983	HERVHF_pol	-
Colobus_guereza_LG09	100541384	100542115	HERVHF_pro	-
Colobus_guereza_LG09	100542630	100543043	HERVHF_gag	-
Colobus_guereza_LG09	100544351	100544765	HERVHF_3LTR	-
Colobus_guereza_LG11	129720258	129720656	HERVHF_5LTR	-
Colobus_guereza_LG11	129721303	129722076	HERVHF_pol	-
Colobus_guereza_LG11	129721477	129722100	HERVHF_pro	-
Colobus_guereza_LG11	129723751	129724151	HERVHF_3LTR	-
Colobus_guereza_LG12	54427286	54427681	HERVHF_5LTR	+
Colobus_guereza_LG12	54429639	54430109	HERVHF_pro	+
Colobus_guereza_LG12	54430598	54431867	HERVHF_pol	+
Colobus_guereza_LG12	54432776	54433154	HERVHF_3LTR	+
Colobus_guereza_LG13	102125204	102125680	HERVHF_5LTR	-
Colobus_guereza_LG13	102126690	102128557	HERVHF_pol	-
Colobus_guereza_LG13	102127991	102128800	HERVHF_pro	-
Colobus_guereza_LG13	102128906	102129268	HERVHF_gag	-
Colobus_guereza_LG13	102130776	102131249	HERVHF_3LTR	-
Colobus_guereza_LG15	70287811	70288435	HERVHF_5LTR	-
Colobus_guereza_LG15	70288628	70289463	HERVHF_pol	-
Colobus_guereza_LG15	70290289	70290921	HERVHF_3LTR	-
Colobus_guereza_LG16	81677337	81677670	HUERSP_5LTR	-
Colobus_guereza_LG16	81683001	81683659	HUERSP_pol	-
Colobus_guereza_LG16	81683336	81683644	HUERSP_pro	-
Colobus_guereza_LG16	81684356	81685012	HUERSP_gag	-
Colobus_guereza_LG16	81687006	81687327	HUERSP_3LTR	-
Colobus_guereza_LG17	12937785	12938129	HERVHF_5LTR	-
Colobus_guereza_LG17	12938795	12940689	HERVHF_pol	-
Colobus_guereza_LG17	12940141	12940920	HERVHF_pro	-
Colobus_guereza_LG17	12941009	12941314	HERVHF_gag	-
Colobus_guereza_LG17	12943128	12943471	HERVHF_3LTR	-
Colobus_guereza_LG18	36587215	36587664	HERVIPADP_5LTR	+
Colobus_guereza_LG18	36589854	36590216	HERVIPADP_gag	+
Colobus_guereza_LG18	36590604	36594237	HERVIPADP_pol	+
Colobus_guereza_LG18	36595735	36596187	HERVIPADP_env	+

Colobus_guereza_LG18	36596275	36596718	HERVIPADP_3LTR	+
Colobus_guereza_LG18	92302394	92302849	HERVHF_5LTR	-
Colobus_guereza_LG18	92303466	92305328	HERVHF_pol	-
Colobus_guereza_LG18	92305619	92305942	HERVHF_gag	-
Colobus_guereza_LG18	92307651	92308105	HERVHF_3LTR	-
Colobus_guereza_LG19	42896800	42897109	HERVHF_5LTR	-
Colobus_guereza_LG19	42900157	42906591	HERVHF_pol	-
Colobus_guereza_LG19	42906118	42906627	HERVHF_pro	-
Colobus_guereza_LG19	42911195	42911499	HERVHF_3LTR	-
Daubentonia_madagascariensis.fasta_ctg1	19222278	19222597	HERVHF_5LTR	-
Daubentonia_madagascariensis.fasta_ctg1	19224528	19227600	HERVHF_pol	-
Daubentonia_madagascariensis.fasta_ctg1	19228205	19228699	HERVHF_gag	-
Daubentonia_madagascariensis.fasta_ctg1	19230102	19230412	HERVHF_3LTR	-
Daubentonia_madagascariensis.fasta_ctg12	989558	989887	HSERVIII_5LTR	+
Daubentonia_madagascariensis.fasta_ctg12	991660	994038	HSERVIII_pol	+
Daubentonia_madagascariensis.fasta_ctg12	995497	995824	HSERVIII_3LTR	+
Daubentonia_madagascariensis.fasta_ctg128	2000620	2001037	Unknown_HERV_5LTR	-
Daubentonia_madagascariensis.fasta_ctg128	2004010	2006046	Unknown_HERV_pol	-
Daubentonia_madagascariensis.fasta_ctg128	2007572	2007990	Unknown_HERV_3LTR	-
Erythrocebus_patas_Contig1	31483142	31483463	HERVHF_5LTR	-
Erythrocebus_patas_Contig1	31483912	31485901	HERVHF_pol	-
Erythrocebus_patas_Contig1	31488184	31488510	HERVHF_3LTR	-
Erythrocebus_patas_Contig10	24937773	24938197	HERVHF_5LTR	-
Erythrocebus_patas_Contig10	24938628	24940482	HERVHF_pol	-
Erythrocebus_patas_Contig10	24940174	24940950	HERVHF_pro	-
Erythrocebus_patas_Contig10	24941086	24941403	HERVHF_gag	-
Erythrocebus_patas_Contig10	24942804	24943224	HERVHF_3LTR	-
Erythrocebus_patas_Contig10	33877933	33878346	HERVHF_5LTR	+
Erythrocebus_patas_Contig10	33880376	33881125	HERVHF_pro	+
Erythrocebus_patas_Contig10	33880835	33882533	HERVHF_pol	+
Erythrocebus_patas_Contig10	33883048	33883460	HERVHF_3LTR	+
Erythrocebus_patas_Contig104	3226968	3227419	HERVK_5LTR	-
Erythrocebus_patas_Contig104	3228465	3230244	HERVK_pol	-
Erythrocebus_patas_Contig104	3230139	3231047	HERVK_pro	-
Erythrocebus_patas_Contig104	3230493	3231212	HERVK_gag	-
Erythrocebus_patas_Contig104	3232816	3233264	HERVK_3LTR	-
Erythrocebus_patas_Contig11	467636	468109	HERVK_5LTR	+

Erythrocebus_patas_Contig11	468919	469850	HERVK_pro	+
Erythrocebus_patas_Contig11	469682	470912	HERVK_pol	+
Erythrocebus_patas_Contig11	471880	472352	HERVK_3LTR	+
Erythrocebus_patas_Contig11	5757035	5757348	HERVHF_5LTR	+
Erythrocebus_patas_Contig11	5759169	5759600	HERVHF_pro	+
Erythrocebus_patas_Contig11	5759642	5762337	HERVHF_pol	+
Erythrocebus_patas_Contig11	5762870	5763186	HERVHF_3LTR	+
Erythrocebus_patas_Contig11	21739397	21739786	HERVHF_5LTR	+
Erythrocebus_patas_Contig11	21741406	21741891	HERVHF_gag	+
Erythrocebus_patas_Contig11	21742079	21742456	HERVHF_pro	+
Erythrocebus_patas_Contig11	21742347	21745382	HERVHF_pol	+
Erythrocebus_patas_Contig11	21745706	21746080	HERVHF_env	+
Erythrocebus_patas_Contig11	21746315	21746699	HERVHF_3LTR	+
Erythrocebus_patas_Contig115	6203133	6203584	HERVHF_5LTR	-
Erythrocebus_patas_Contig115	6204647	6206048	HERVHF_pol	-
Erythrocebus_patas_Contig115	6206358	6206699	HERVHF_gag	-
Erythrocebus_patas_Contig115	6208331	6208779	HERVHF_3LTR	-
Erythrocebus_patas_Contig120	2410720	2411221	HERVHF_5LTR	+
Erythrocebus_patas_Contig120	2413067	2413858	HERVHF_pro	+
Erythrocebus_patas_Contig120	2413553	2414637	HERVHF_pol	+
Erythrocebus_patas_Contig120	2414934	2415439	HERVHF_3LTR	+
Erythrocebus_patas_Contig121	1503711	1504070	HERVHF_5LTR	-
Erythrocebus_patas_Contig121	1504214	1504516	HERVHF_env	-
Erythrocebus_patas_Contig121	1504673	1506478	HERVHF_pol	-
Erythrocebus_patas_Contig121	1506498	1506923	HERVHF_pro	-
Erythrocebus_patas_Contig121	1509014	1509368	HERVHF_3LTR	-
Erythrocebus_patas_Contig13	29365738	29366082	HERVHF_5LTR	-
Erythrocebus_patas_Contig13	29366388	29367417	HERVHF_env	-
Erythrocebus_patas_Contig13	29369040	29371403	HERVHF_pol	-
Erythrocebus_patas_Contig13	29370816	29371532	HERVHF_pro	-
Erythrocebus_patas_Contig13	29371992	29372414	HERVHF_gag	-
Erythrocebus_patas_Contig13	29373994	29374347	HERVHF_3LTR	-
Erythrocebus_patas_Contig138	4130673	4131033	HERVHF_5LTR	+
Erythrocebus_patas_Contig138	4133322	4135150	HERVHF_pol	+
Erythrocebus_patas_Contig138	4135873	4136235	HERVHF_3LTR	+
Erythrocebus_patas_Contig14	9175294	9175625	HERVHF_5LTR	-
Erythrocebus_patas_Contig14	9175913	9176251	HERVHF_env	-
Erythrocebus_patas_Contig14	9176559	9177263	HERVHF_pol	-
Erythrocebus_patas_Contig14	9179547	9179868	HERVHF_3LTR	-
Erythrocebus_patas_Contig159	2942345	2942716	HERVHF_5LTR	+
Erythrocebus_patas_Contig159	2943407	2944087	HERVHF_pro	+
Erythrocebus_patas_Contig159	2943680	2945998	HERVHF_pol	+
Erythrocebus_patas_Contig159	2946874	2947237	HERVHF_3LTR	+
Erythrocebus_patas_Contig189	15905	16305	HERVHF_5LTR	+

Erythrocebus_patas_Contig189	18963	21683	HERVHF_pol	+
Erythrocebus_patas_Contig189	22349	22801	HERVHF_env	+
Erythrocebus_patas_Contig189	23009	23410	HERVHF_3LTR	+
Erythrocebus_patas_Contig19	30085947	30086348	HERVHF_5LTR	-
Erythrocebus_patas_Contig19	30086954	30089358	HERVHF_pol	-
Erythrocebus_patas_Contig19	30088816	30089658	HERVHF_pro	-
Erythrocebus_patas_Contig19	30089865	30090218	HERVHF_gag	-
Erythrocebus_patas_Contig19	30091465	30091857	HERVHF_3LTR	-
Erythrocebus_patas_Contig196	1670592	1671102	HERVK_5LTR	-
Erythrocebus_patas_Contig196	1672029	1674465	HERVK_pol	-
Erythrocebus_patas_Contig196	1674333	1675283	HERVK_pro	-
Erythrocebus_patas_Contig196	1675229	1676279	HERVK_gag	-
Erythrocebus_patas_Contig196	1676701	1677214	HERVK_3LTR	-
Erythrocebus_patas_Contig2	24330326	24330759	HERVHF_5LTR	+
Erythrocebus_patas_Contig2	24331588	24334342	HERVHF_gag	+
Erythrocebus_patas_Contig2	24334630	24335235	HERVHF_pro	+
Erythrocebus_patas_Contig2	24334660	24337124	HERVHF_pol	+
Erythrocebus_patas_Contig2	24337663	24338098	HERVHF_3LTR	+
Erythrocebus_patas_Contig224	384977	385289	HERVHF_5LTR	+
Erythrocebus_patas_Contig224	390067	390732	HERVHF_gag	+
Erythrocebus_patas_Contig224	391222	393534	HERVHF_pol	+
Erythrocebus_patas_Contig224	396242	396550	HERVHF_3LTR	+
Erythrocebus_patas_Contig27	12912397	12912908	HERVK_5LTR	+
Erythrocebus_patas_Contig27	12913888	12914664	HERVK_gag	+
Erythrocebus_patas_Contig27	12914273	12914986	HERVK_pro	+
Erythrocebus_patas_Contig27	12915033	12915815	HERVK_pol	+
Erythrocebus_patas_Contig27	12917812	12918177	HERVK_env	+
Erythrocebus_patas_Contig27	12918179	12918676	HERVK_3LTR	+
Erythrocebus_patas_Contig3	9282418	9282918	HERVIPADP_5LTR	+
Erythrocebus_patas_Contig3	9283173	9283544	HERVIPADP_gag	+
Erythrocebus_patas_Contig3	9284120	9284605	HERVIPADP_pol	+
Erythrocebus_patas_Contig3	9294544	9295034	HERVIPADP_3LTR	+
Erythrocebus_patas_Contig33	4709180	4709847	HERVHF_5LTR	+
Erythrocebus_patas_Contig33	4711502	4711930	HERVHF_pro	+
Erythrocebus_patas_Contig33	4711965	4714272	HERVHF_pol	+
Erythrocebus_patas_Contig33	4717907	4718580	HERVHF_3LTR	+
Erythrocebus_patas_Contig356	11195	11694	HERVK_5LTR	+
Erythrocebus_patas_Contig356	12496	13416	HERVK_pro	+
Erythrocebus_patas_Contig356	13326	15223	HERVK_pol	+
Erythrocebus_patas_Contig356	15644	16051	HERVK_env	+
Erythrocebus_patas_Contig356	16128	16625	HERVK_3LTR	+
Erythrocebus_patas_Contig363	96294	96702	HERVHF_5LTR	-
Erythrocebus_patas_Contig363	96868	97230	HERVHF_env	-
Erythrocebus_patas_Contig363	99436	100182	HERVHF_pol	-

Erythrocebus_patas_Contig363	99598	100296	HERVHF_pro	-
Erythrocebus_patas_Contig363	100419	100754	HERVHF_gag	-
Erythrocebus_patas_Contig363	102191	102608	HERVHF_3LTR	-
Erythrocebus_patas_Contig39	5603570	5603997	HERVHF_5LTR	-
Erythrocebus_patas_Contig39	5604539	5606884	HERVHF_pol	-
Erythrocebus_patas_Contig39	5609152	5609588	HERVHF_3LTR	-
Erythrocebus_patas_Contig4	9194026	9194430	HERVHF_5LTR	-
Erythrocebus_patas_Contig4	9194958	9196701	HERVHF_pol	-
Erythrocebus_patas_Contig4	9196701	9197138	HERVHF_pro	-
Erythrocebus_patas_Contig4	9199167	9199565	HERVHF_3LTR	-
Erythrocebus_patas_Contig4	39357295	39357718	HERVHF_5LTR	-
Erythrocebus_patas_Contig4	39357930	39358324	HERVHF_env	-
Erythrocebus_patas_Contig4	39359321	39361155	HERVHF_pol	-
Erythrocebus_patas_Contig4	39360844	39361662	HERVHF_pro	-
Erythrocebus_patas_Contig4	39362019	39362477	HERVHF_gag	-
Erythrocebus_patas_Contig4	39363733	39364155	HERVHF_3LTR	-
Erythrocebus_patas_Contig40	8027211	8027590	HERVHF_5LTR	-
Erythrocebus_patas_Contig40	8028132	8030395	HERVHF_pol	-
Erythrocebus_patas_Contig40	8030119	8030595	HERVHF_pro	-
Erythrocebus_patas_Contig40	8032399	8032777	HERVHF_3LTR	-
Erythrocebus_patas_Contig45	12422394	12422883	HERVK_5LTR	+
Erythrocebus_patas_Contig45	12424319	12425275	HERVK_pro	+
Erythrocebus_patas_Contig45	12425131	12426781	HERVK_pol	+
Erythrocebus_patas_Contig45	12427573	12427905	HERVK_env	+
Erythrocebus_patas_Contig45	12427984	12428474	HERVK_3LTR	+
Erythrocebus_patas_Contig456	276188	276540	HERVHF_5LTR	-
Erythrocebus_patas_Contig456	277137	279348	HERVHF_pol	-
Erythrocebus_patas_Contig456	279782	280138	HERVHF_gag	-
Erythrocebus_patas_Contig456	281583	281941	HERVHF_3LTR	-
Erythrocebus_patas_Contig46	2951790	2952140	HERVHF_5LTR	+
Erythrocebus_patas_Contig46	2954772	2956398	HERVHF_pol	+
Erythrocebus_patas_Contig46	2957169	2957528	HERVHF_3LTR	+
Erythrocebus_patas_Contig47	11341387	11341696	HERVHF_5LTR	-
Erythrocebus_patas_Contig47	11341983	11342348	HERVHF_env	-
Erythrocebus_patas_Contig47	11343483	11345219	HERVHF_pol	-
Erythrocebus_patas_Contig47	11344614	11345372	HERVHF_pro	-
Erythrocebus_patas_Contig47	11345527	11345862	HERVHF_gag	-
Erythrocebus_patas_Contig47	11346133	11346436	HERVHF_3LTR	-
Erythrocebus_patas_Contig49	14668064	14668383	HERVHF_5LTR	-
Erythrocebus_patas_Contig49	14668560	14669152	HERVHF_env	-
Erythrocebus_patas_Contig49	14671446	14673707	HERVHF_pol	-
Erythrocebus_patas_Contig49	14676052	14676429	HERVHF_pro	-
Erythrocebus_patas_Contig49	14676556	14677131	HERVHF_gag	-
Erythrocebus_patas_Contig49	14678718	14679033	HERVHF_3LTR	-

Erythrocebus_patas_Contig5	37534626	37535079	HERVHF_5LTR	-
Erythrocebus_patas_Contig5	37535760	37537398	HERVHF_pol	-
Erythrocebus_patas_Contig5	37537067	37537861	HERVHF_pro	-
Erythrocebus_patas_Contig5	37539912	37540374	HERVHF_3LTR	-
Erythrocebus_patas_Contig5	45771813	45772197	HERVHF_5LTR	-
Erythrocebus_patas_Contig5	45773092	45774583	HERVHF_pol	-
Erythrocebus_patas_Contig5	45774350	45774979	HERVHF_pro	-
Erythrocebus_patas_Contig5	45777384	45777762	HERVHF_3LTR	-
Erythrocebus_patas_Contig50	12223567	12223881	HUERSP_5LTR	-
Erythrocebus_patas_Contig50	12228019	12230913	HUERSP_pol	-
Erythrocebus_patas_Contig50	12231268	12231624	HUERSP_gag	-
Erythrocebus_patas_Contig50	12233936	12234251	HUERSP_3LTR	-
Erythrocebus_patas_Contig52	11423773	11424762	HSERVIII_5LTR	+
Erythrocebus_patas_Contig52	11425608	11425967	HSERVIII_pol	+
Erythrocebus_patas_Contig52	11426766	11427755	HSERVIII_3LTR	+
Erythrocebus_patas_Contig55	519149	519601	HERVHF_5LTR	+
Erythrocebus_patas_Contig55	521086	521454	HERVHF_gag	+
Erythrocebus_patas_Contig55	521224	522368	HERVHF_pro	+
Erythrocebus_patas_Contig55	521832	523669	HERVHF_pol	+
Erythrocebus_patas_Contig55	524269	524716	HERVHF_3LTR	+
Erythrocebus_patas_Contig58	12503297	12503729	HERVHF_5LTR	+
Erythrocebus_patas_Contig58	12505322	12505624	HERVHF_gag	+
Erythrocebus_patas_Contig58	12506322	12508210	HERVHF_pol	+
Erythrocebus_patas_Contig58	12509327	12510463	HERVHF_env	+
Erythrocebus_patas_Contig58	12510682	12511118	HERVHF_3LTR	+
Erythrocebus_patas_Contig6	34901948	34902459	HERVK_5LTR	-
Erythrocebus_patas_Contig6	34902538	34903110	HERVK_env	-
Erythrocebus_patas_Contig6	34903322	34904752	HERVK_pol	-
Erythrocebus_patas_Contig6	34904500	34905268	HERVK_pro	-
Erythrocebus_patas_Contig6	34905390	34906040	HERVK_gag	-
Erythrocebus_patas_Contig6	34906801	34907321	HERVK_3LTR	-
Erythrocebus_patas_Contig6	44812281	44812648	HERVK_5LTR	+
Erythrocebus_patas_Contig6	44813759	44814451	HERVK_pro	+
Erythrocebus_patas_Contig6	44814352	44815418	HERVK_pol	+
Erythrocebus_patas_Contig6	44816539	44817075	HERVK_env	+
Erythrocebus_patas_Contig6	44817310	44817676	HERVK_3LTR	+
Erythrocebus_patas_Contig67	11334371	11334746	HERVHF_5LTR	+
Erythrocebus_patas_Contig67	11337194	11338958	HERVHF_pol	+
Erythrocebus_patas_Contig67	11339626	11340000	HERVHF_3LTR	+
Erythrocebus_patas_Contig7	902955	903335	HERVHF_5LTR	-
Erythrocebus_patas_Contig7	903546	903938	HERVHF_env	-
Erythrocebus_patas_Contig7	904943	906647	HERVHF_pol	-
Erythrocebus_patas_Contig7	907642	908019	HERVHF_3LTR	-
Erythrocebus_patas_Contig73	790714	791166	HERVHF_5LTR	+

Erythrocebus_patas_Contig73	792830	793153	HERVHF_gag	+
Erythrocebus_patas_Contig73	793342	793818	HERVHF_pro	+
Erythrocebus_patas_Contig73	793567	795004	HERVHF_pol	+
Erythrocebus_patas_Contig73	795317	795802	HERVHF_env	+
Erythrocebus_patas_Contig73	795912	796370	HERVHF_3LTR	+
Erythrocebus_patas_Contig90	8519320	8519891	HSERVIII_5LTR	+
Erythrocebus_patas_Contig90	8520682	8521239	HSERVIII_pol	+
Erythrocebus_patas_Contig90	8521773	8522350	HSERVIII_3LTR	+
Galago_moholi_ctg1909	396175	396571	HERVW9_5LTR	+
Galago_moholi_ctg1909	398387	399049	HERVW9_gag	+
Galago_moholi_ctg1909	399346	400491	HERVW9_pro	+
Galago_moholi_ctg1909	400213	402310	HERVW9_pol	+
Galago_moholi_ctg1909	403323	403723	HERVW9_3LTR	+
Galago_moholi_ctg3130	45350	46092	HERVHF_5LTR	-
Galago_moholi_ctg3130	47364	50286	HERVHF_pol	-
Galago_moholi_ctg3130	51296	51958	HERVHF_gag	-
Galago_moholi_ctg3130	58357	59103	HERVHF_3LTR	-
Galago_moholi_ctg6283	48549	49464	HERVHF_5LTR	-
Galago_moholi_ctg6283	55524	56548	HERVHF_pol	-
Galago_moholi_ctg6283	60065	60972	HERVHF_3LTR	-
Galago_moholi_ctg7351	21674	22071	HERVW9_5LTR	-
Galago_moholi_ctg7351	23251	25812	HERVW9_pol	-
Galago_moholi_ctg7351	26530	27114	HERVW9_gag	-
Galago_moholi_ctg7351	29094	29480	HERVW9_3LTR	-
Galago_moholi_ctg7630	8022	8490	HERVW9_5LTR	+
Galago_moholi_ctg7630	15242	17368	HERVW9_pol	+
Galago_moholi_ctg7630	18028	18503	HERVW9_3LTR	+
Gorilla_gorilla_gorilla_002198F_quiver_patched	14017	14352	HERVHF_5LTR	-
Gorilla_gorilla_gorilla_002198F_quiver_patched	15594	17211	HERVHF_pol	-
Gorilla_gorilla_gorilla_002198F_quiver_patched	16807	17631	HERVHF_pro	-
Gorilla_gorilla_gorilla_002198F_quiver_patched	17706	18188	HERVHF_gag	-
Gorilla_gorilla_gorilla_002198F_quiver_patched	18741	19069	HERVHF_3LTR	-
Gorilla_gorilla_gorilla_chr1	4452990	4453326	HERVHF_5LTR	+
Gorilla_gorilla_gorilla_chr1	4455933	4458184	HERVHF_pol	+
Gorilla_gorilla_gorilla_chr1	4460558	4461302	HERVHF_env	+
Gorilla_gorilla_gorilla_chr1	4461527	4461879	HERVHF_3LTR	+
Gorilla_gorilla_gorilla_chr1	12150798	12151209	HERVHF_5LTR	+
Gorilla_gorilla_gorilla_chr1	12152142	12154315	HERVHF_gag	+
Gorilla_gorilla_gorilla_chr1	12154305	12155282	HERVHF_pro	+

Gorilla_gorilla_gorilla_chr1	12154656	12157074	HERVHF_pol	+
Gorilla_gorilla_gorilla_chr1	12157557	12157968	HERVHF_3LTR	+
Gorilla_gorilla_gorilla_chr1	37351251	37351663	HERVHF_5LTR	+
Gorilla_gorilla_gorilla_chr1	37353277	37353663	HERVHF_gag	+
Gorilla_gorilla_gorilla_chr1	37353735	37354490	HERVHF_pro	+
Gorilla_gorilla_gorilla_chr1	37354110	37356110	HERVHF_pol	+
Gorilla_gorilla_gorilla_chr1	37356503	37356916	HERVHF_3LTR	+
Gorilla_gorilla_gorilla_chr1	44741319	44741676	HERVIPADP_5LTR	-
Gorilla_gorilla_gorilla_chr1	44741837	44742397	HERVIPADP_env	-
Gorilla_gorilla_gorilla_chr1	44743823	44747122	HERVIPADP_pol	-
Gorilla_gorilla_gorilla_chr1	44747762	44748073	HERVIPADP_gag	-
Gorilla_gorilla_gorilla_chr1	44750145	44750502	HERVIPADP_3LTR	-
Gorilla_gorilla_gorilla_chr1	47164062	47164480	HERVHF_5LTR	-
Gorilla_gorilla_gorilla_chr1	47165055	47166878	HERVHF_pol	-
Gorilla_gorilla_gorilla_chr1	47167314	47167832	HERVHF_gag	-
Gorilla_gorilla_gorilla_chr1	47169393	47169821	HERVHF_3LTR	-
Gorilla_gorilla_gorilla_chr1	52372687	52373132	HERVHF_5LTR	+
Gorilla_gorilla_gorilla_chr1	52374693	52375064	HERVHF_gag	+
Gorilla_gorilla_gorilla_chr1	52375126	52375968	HERVHF_pro	+
Gorilla_gorilla_gorilla_chr1	52375330	52377551	HERVHF_pol	+
Gorilla_gorilla_gorilla_chr1	52377942	52378387	HERVHF_3LTR	+
Gorilla_gorilla_gorilla_chr1	75857095	75857513	HERVHF_5LTR	-
Gorilla_gorilla_gorilla_chr1	75858104	75860019	HERVHF_pol	-
Gorilla_gorilla_gorilla_chr1	75859450	75860307	HERVHF_pro	-
Gorilla_gorilla_gorilla_chr1	75862249	75862674	HERVHF_3LTR	-
Gorilla_gorilla_gorilla_chr1	78505033	78505430	HERVHF_5LTR	-
Gorilla_gorilla_gorilla_chr1	78505838	78508003	HERVHF_pol	-
Gorilla_gorilla_gorilla_chr1	78510266	78510673	HERVHF_3LTR	-
Gorilla_gorilla_gorilla_chr1	78710572	78711008	HERVHF_5LTR	-
Gorilla_gorilla_gorilla_chr1	78711415	78713458	HERVHF_pol	-
Gorilla_gorilla_gorilla_chr1	78713018	78713659	HERVHF_pro	-
Gorilla_gorilla_gorilla_chr1	78715992	78716436	HERVHF_3LTR	-
Gorilla_gorilla_gorilla_chr1	93644387	93644696	HERVHF_5LTR	-
Gorilla_gorilla_gorilla_chr1	93645428	93647740	HERVHF_pol	-
Gorilla_gorilla_gorilla_chr1	93647177	93647902	HERVHF_pro	-
Gorilla_gorilla_gorilla_chr1	93648065	93648996	HERVHF_gag	-
Gorilla_gorilla_gorilla_chr1	93650289	93650595	HERVHF_3LTR	-
Gorilla_gorilla_gorilla_chr1	97871087	97871527	HERVHF_5LTR	-
Gorilla_gorilla_gorilla_chr1	97871939	97874253	HERVHF_pol	-
Gorilla_gorilla_gorilla_chr1	97873828	97874427	HERVHF_pro	-
Gorilla_gorilla_gorilla_chr1	97874713	97875191	HERVHF_gag	-
Gorilla_gorilla_gorilla_chr1	97876598	97877031	HERVHF_3LTR	-
Gorilla_gorilla_gorilla_chr1	98581622	98582065	HERVHF_5LTR	+
Gorilla_gorilla_gorilla_chr1	98583874	98584290	HERVHF_gag	+

Gorilla_gorilla_gorilla_chr1	98584327	98585100	HERVHF_pro	+
Gorilla_gorilla_gorilla_chr1	98584489	98586466	HERVHF_pol	+
Gorilla_gorilla_gorilla_chr1	98587112	98587564	HERVHF_3LTR	+
Gorilla_gorilla_gorilla_chr1	157023619	157024057	HERVHF_5LTR	+
Gorilla_gorilla_gorilla_chr1	157026002	157026331	HERVHF_gag	+
Gorilla_gorilla_gorilla_chr1	157026760	157028750	HERVHF_pol	+
Gorilla_gorilla_gorilla_chr1	157029181	157029615	HERVHF_3LTR	+
Gorilla_gorilla_gorilla_chr1	159360967	159361422	HERVHF_5LTR	-
Gorilla_gorilla_gorilla_chr1	159361877	159363908	HERVHF_pol	-
Gorilla_gorilla_gorilla_chr1	159364246	159364569	HERVHF_gag	-
Gorilla_gorilla_gorilla_chr1	159366411	159366869	HERVHF_3LTR	-
Gorilla_gorilla_gorilla_chr1	171483819	171484165	HERVHF_5LTR	+
Gorilla_gorilla_gorilla_chr1	171486462	171486905	HERVHF_pro	+
Gorilla_gorilla_gorilla_chr1	171486845	171488732	HERVHF_pol	+
Gorilla_gorilla_gorilla_chr1	171489749	171490097	HERVHF_3LTR	+
Gorilla_gorilla_gorilla_chr1	179342772	179343217	HERVK_5LTR	+
Gorilla_gorilla_gorilla_chr1	179344692	179345629	HERVK_pro	+
Gorilla_gorilla_gorilla_chr1	179345524	179346793	HERVK_pol	+
Gorilla_gorilla_gorilla_chr1	179347287	179347638	HERVK_env	+
Gorilla_gorilla_gorilla_chr1	179347724	179348161	HERVK_3LTR	+
Gorilla_gorilla_gorilla_chr1	182903392	182903859	HERVHF_5LTR	+
Gorilla_gorilla_gorilla_chr1	182906398	182907583	HERVHF_pol	+
Gorilla_gorilla_gorilla_chr1	182908211	182908671	HERVHF_3LTR	+
Gorilla_gorilla_gorilla_chr1	189475210	189475622	HERVHF_5LTR	+
Gorilla_gorilla_gorilla_chr1	189477701	189478480	HERVHF_pro	+
Gorilla_gorilla_gorilla_chr1	189477866	189479907	HERVHF_pol	+
Gorilla_gorilla_gorilla_chr1	189480466	189480878	HERVHF_3LTR	+
Gorilla_gorilla_gorilla_chr1	189613665	189613998	HERVHF_5LTR	+
Gorilla_gorilla_gorilla_chr1	189616175	189617005	HERVHF_pro	+
Gorilla_gorilla_gorilla_chr1	189616424	189617182	HERVHF_pol	+
Gorilla_gorilla_gorilla_chr1	189619316	189620197	HERVHF_env	+
Gorilla_gorilla_gorilla_chr1	189620370	189620695	HERVHF_3LTR	+
Gorilla_gorilla_gorilla_chr1	195406518	195406983	HERVHF_5LTR	+
Gorilla_gorilla_gorilla_chr1	195409233	195410123	HERVHF_pro	+
Gorilla_gorilla_gorilla_chr1	195409575	195410749	HERVHF_pol	+
Gorilla_gorilla_gorilla_chr1	195416915	195417382	HERVHF_3LTR	+
Gorilla_gorilla_gorilla_chr1	202015628	202016017	HERVHF_5LTR	+
Gorilla_gorilla_gorilla_chr1	202017802	202018122	HERVHF_gag	+
Gorilla_gorilla_gorilla_chr1	202018210	202018959	HERVHF_pro	+
Gorilla_gorilla_gorilla_chr1	202018417	202020535	HERVHF_pol	+
Gorilla_gorilla_gorilla_chr1	202021023	202021395	HERVHF_3LTR	+
Gorilla_gorilla_gorilla_chr1	202870854	202871257	HERVHF_5LTR	+
Gorilla_gorilla_gorilla_chr1	202873290	202873877	HERVHF_pro	+
Gorilla_gorilla_gorilla_chr1	202873810	202875561	HERVHF_pol	+

Gorilla_gorilla_gorilla_chr1	202876143	202876554	HERVHF_3LTR	+
Gorilla_gorilla_gorilla_chr1	204710155	204710585	HERVHF_5LTR	-
Gorilla_gorilla_gorilla_chr1	204711024	204713652	HERVHF_pol	-
Gorilla_gorilla_gorilla_chr1	204713110	204713859	HERVHF_pro	-
Gorilla_gorilla_gorilla_chr1	204714049	204714390	HERVHF_gag	-
Gorilla_gorilla_gorilla_chr1	204715720	204716150	HERVHF_3LTR	-
Gorilla_gorilla_gorilla_chr1	205107945	205108377	HERVHF_5LTR	-
Gorilla_gorilla_gorilla_chr1	205109212	205110907	HERVHF_pol	-
Gorilla_gorilla_gorilla_chr1	205111201	205111530	HERVHF_gag	-
Gorilla_gorilla_gorilla_chr1	205113198	205113634	HERVHF_3LTR	-
Gorilla_gorilla_gorilla_chr10	5578876	5579290	HERVHF_5LTR	+
Gorilla_gorilla_gorilla_chr10	5581351	5581650	HERVHF_gag	+
Gorilla_gorilla_gorilla_chr10	5581947	5582477	HERVHF_pro	+
Gorilla_gorilla_gorilla_chr10	5582070	5584223	HERVHF_pol	+
Gorilla_gorilla_gorilla_chr10	5584672	5585084	HERVHF_3LTR	+
Gorilla_gorilla_gorilla_chr10	6987370	6987823	HERVHF_5LTR	-
Gorilla_gorilla_gorilla_chr10	6988276	6990204	HERVHF_pol	-
Gorilla_gorilla_gorilla_chr10	6992836	6993290	HERVHF_3LTR	-
Gorilla_gorilla_gorilla_chr10	26375925	26376260	HERVHF_5LTR	+
Gorilla_gorilla_gorilla_chr10	26377734	26378312	HERVHF_gag	+
Gorilla_gorilla_gorilla_chr10	26378309	26379148	HERVHF_pro	+
Gorilla_gorilla_gorilla_chr10	26378510	26381455	HERVHF_pol	+
Gorilla_gorilla_gorilla_chr10	26381969	26382313	HERVHF_3LTR	+
Gorilla_gorilla_gorilla_chr10	26428862	26429239	HERVHF_5LTR	-
Gorilla_gorilla_gorilla_chr10	26430361	26431649	HERVHF_pol	-
Gorilla_gorilla_gorilla_chr10	26431053	26431937	HERVHF_pro	-
Gorilla_gorilla_gorilla_chr10	26432048	26432955	HERVHF_gag	-
Gorilla_gorilla_gorilla_chr10	26434426	26434808	HERVHF_3LTR	-
Gorilla_gorilla_gorilla_chr10	30583504	30583945	HERVHF_5LTR	-
Gorilla_gorilla_gorilla_chr10	30584401	30585893	HERVHF_pol	-
Gorilla_gorilla_gorilla_chr10	30585381	30586109	HERVHF_pro	-
Gorilla_gorilla_gorilla_chr10	30586187	30586507	HERVHF_gag	-
Gorilla_gorilla_gorilla_chr10	30588537	30588978	HERVHF_3LTR	-
Gorilla_gorilla_gorilla_chr10	48559730	48560137	HERVHF_5LTR	+
Gorilla_gorilla_gorilla_chr10	48560915	48561721	HERVHF_pro	+
Gorilla_gorilla_gorilla_chr10	48561206	48563155	HERVHF_pol	+
Gorilla_gorilla_gorilla_chr10	48563743	48564159	HERVHF_3LTR	+
Gorilla_gorilla_gorilla_chr10	53148954	53149402	HERVHF_5LTR	-
Gorilla_gorilla_gorilla_chr10	53149868	53151948	HERVHF_pol	-
Gorilla_gorilla_gorilla_chr10	53151406	53152209	HERVHF_pro	-
Gorilla_gorilla_gorilla_chr10	53154431	53154883	HERVHF_3LTR	-
Gorilla_gorilla_gorilla_chr10	53875975	53876426	HERVHF_5LTR	+
Gorilla_gorilla_gorilla_chr10	53878208	53878537	HERVHF_gag	+
Gorilla_gorilla_gorilla_chr10	53878966	53887254	HERVHF_pol	+

Gorilla_gorilla_gorilla_chr10	53887646	53888103	HERVHF_3LTR	+
Gorilla_gorilla_gorilla_chr10	91237709	91238161	HERVHF_5LTR	+
Gorilla_gorilla_gorilla_chr10	91240039	91240362	HERVHF_gag	+
Gorilla_gorilla_gorilla_chr10	91240414	91241178	HERVHF_pro	+
Gorilla_gorilla_gorilla_chr10	91240618	91242438	HERVHF_pol	+
Gorilla_gorilla_gorilla_chr10	91243039	91243491	HERVHF_3LTR	+
Gorilla_gorilla_gorilla_chr10	92756976	92757424	HERVHF_5LTR	+
Gorilla_gorilla_gorilla_chr10	92758381	92758704	HERVHF_gag	+
Gorilla_gorilla_gorilla_chr10	92758741	92759514	HERVHF_pro	+
Gorilla_gorilla_gorilla_chr10	92758957	92759670	HERVHF_pol	+
Gorilla_gorilla_gorilla_chr10	92760183	92760625	HERVHF_3LTR	+
Gorilla_gorilla_gorilla_chr10	97206282	97206649	HERVHF_5LTR	-
Gorilla_gorilla_gorilla_chr10	97206813	97207387	HERVHF_env	-
Gorilla_gorilla_gorilla_chr10	97208422	97210561	HERVHF_pol	-
Gorilla_gorilla_gorilla_chr10	97211301	97211642	HERVHF_gag	-
Gorilla_gorilla_gorilla_chr10	97212895	97213262	HERVHF_3LTR	-
Gorilla_gorilla_gorilla_chr10	116900714	116901039	HERVHF_5LTR	+
Gorilla_gorilla_gorilla_chr10	116903046	116903876	HERVHF_pro	+
Gorilla_gorilla_gorilla_chr10	116903334	116905257	HERVHF_pol	+
Gorilla_gorilla_gorilla_chr10	116905970	116906291	HERVHF_3LTR	+
Gorilla_gorilla_gorilla_chr11	4862098	4862512	HERVHF_5LTR	-
Gorilla_gorilla_gorilla_chr11	4862906	4864889	HERVHF_pol	-
Gorilla_gorilla_gorilla_chr11	4865347	4865649	HERVHF_gag	-
Gorilla_gorilla_gorilla_chr11	4867489	4867903	HERVHF_3LTR	-
Gorilla_gorilla_gorilla_chr11	6106414	6106734	HERVHF_5LTR	+
Gorilla_gorilla_gorilla_chr11	6108336	6108644	HERVHF_gag	+
Gorilla_gorilla_gorilla_chr11	6108962	6111203	HERVHF_pol	+
Gorilla_gorilla_gorilla_chr11	6111652	6111962	HERVHF_3LTR	+
Gorilla_gorilla_gorilla_chr11	7739811	7740264	HERVHF_5LTR	-
Gorilla_gorilla_gorilla_chr11	7740661	7742773	HERVHF_pol	-
Gorilla_gorilla_gorilla_chr11	7742231	7743061	HERVHF_pro	-
Gorilla_gorilla_gorilla_chr11	7743068	7743397	HERVHF_gag	-
Gorilla_gorilla_gorilla_chr11	7745214	7745670	HERVHF_3LTR	-
Gorilla_gorilla_gorilla_chr11	10029822	10030252	HERVHF_5LTR	+
Gorilla_gorilla_gorilla_chr11	10030740	10031429	HERVHF_pro	+
Gorilla_gorilla_gorilla_chr11	10030800	10033437	HERVHF_pol	+
Gorilla_gorilla_gorilla_chr11	10033663	10034010	HERVHF_env	+
Gorilla_gorilla_gorilla_chr11	10034187	10034619	HERVHF_3LTR	+
Gorilla_gorilla_gorilla_chr11	18695776	18696231	HERVHF_5LTR	-
Gorilla_gorilla_gorilla_chr11	18697119	18698611	HERVHF_pol	-
Gorilla_gorilla_gorilla_chr11	18699045	18699362	HERVHF_gag	-
Gorilla_gorilla_gorilla_chr11	18701100	18701553	HERVHF_3LTR	-
Gorilla_gorilla_gorilla_chr11	23159792	23160246	HERVHF_5LTR	-
Gorilla_gorilla_gorilla_chr11	23160718	23162659	HERVHF_pol	-

Gorilla_gorilla_gorilla_chr11	23162193	23162993	HERVHF_pro	-
Gorilla_gorilla_gorilla_chr11	23163051	23163380	HERVHF_gag	-
Gorilla_gorilla_gorilla_chr11	23165183	23165643	HERVHF_3LTR	-
Gorilla_gorilla_gorilla_chr11	59788579	59788990	HERVHF_5LTR	+
Gorilla_gorilla_gorilla_chr11	59790726	59791025	HERVHF_gag	+
Gorilla_gorilla_gorilla_chr11	59791536	59793287	HERVHF_pol	+
Gorilla_gorilla_gorilla_chr11	59793859	59794272	HERVHF_3LTR	+
Gorilla_gorilla_gorilla_chr11	60639676	60639996	HERVHF_5LTR	+
Gorilla_gorilla_gorilla_chr11	60643150	60643512	HERVHF_gag	+
Gorilla_gorilla_gorilla_chr11	60643849	60645977	HERVHF_pol	+
Gorilla_gorilla_gorilla_chr11	60646324	60646642	HERVHF_3LTR	+
Gorilla_gorilla_gorilla_chr11	73776377	73776709	HERVHF_5LTR	+
Gorilla_gorilla_gorilla_chr11	73778675	73779487	HERVHF_pro	+
Gorilla_gorilla_gorilla_chr11	73778966	73780299	HERVHF_pol	+
Gorilla_gorilla_gorilla_chr11	73781542	73781857	HERVHF_3LTR	+
Gorilla_gorilla_gorilla_chr11	86023589	86023982	HERVHF_5LTR	+
Gorilla_gorilla_gorilla_chr11	86025773	86026195	HERVHF_gag	+
Gorilla_gorilla_gorilla_chr11	86026424	86027008	HERVHF_pro	+
Gorilla_gorilla_gorilla_chr11	86026466	86028408	HERVHF_pol	+
Gorilla_gorilla_gorilla_chr11	86028998	86029383	HERVHF_3LTR	+
Gorilla_gorilla_gorilla_chr11	86697233	86697696	HERVHF_5LTR	+
Gorilla_gorilla_gorilla_chr11	86699280	86699660	HERVHF_gag	+
Gorilla_gorilla_gorilla_chr11	86699974	86702056	HERVHF_pol	+
Gorilla_gorilla_gorilla_chr11	86704257	86704856	HERVHF_env	+
Gorilla_gorilla_gorilla_chr11	86705021	86705495	HERVHF_3LTR	+
Gorilla_gorilla_gorilla_chr11	122079792	122080158	HERVHF_5LTR	-
Gorilla_gorilla_gorilla_chr11	122080724	122082585	HERVHF_pol	-
Gorilla_gorilla_gorilla_chr11	122082231	122082941	HERVHF_pro	-
Gorilla_gorilla_gorilla_chr11	122084922	122085279	HERVHF_3LTR	-
Gorilla_gorilla_gorilla_chr11	124976603	124977015	HERVHF_5LTR	+
Gorilla_gorilla_gorilla_chr11	124979275	124980000	HERVHF_pro	+
Gorilla_gorilla_gorilla_chr11	124979617	124981709	HERVHF_pol	+
Gorilla_gorilla_gorilla_chr11	124982220	124982632	HERVHF_3LTR	+
Gorilla_gorilla_gorilla_chr12	4024039	4024502	HERVHF_5LTR	+
Gorilla_gorilla_gorilla_chr12	4026809	4027342	HERVHF_pro	+
Gorilla_gorilla_gorilla_chr12	4026932	4028059	HERVHF_pol	+
Gorilla_gorilla_gorilla_chr12	4028716	4029177	HERVHF_3LTR	+
Gorilla_gorilla_gorilla_chr12	11043531	11043995	HERVHF_5LTR	-
Gorilla_gorilla_gorilla_chr12	11044563	11046513	HERVHF_pol	-
Gorilla_gorilla_gorilla_chr12	11046917	11047228	HERVHF_gag	-
Gorilla_gorilla_gorilla_chr12	11048986	11049458	HERVHF_3LTR	-
Gorilla_gorilla_gorilla_chr12	11189667	11190143	HERVHF_5LTR	-
Gorilla_gorilla_gorilla_chr12	11190506	11192467	HERVHF_pol	-
Gorilla_gorilla_gorilla_chr12	11192060	11192641	HERVHF_pro	-

Gorilla_gorilla_gorilla_chr12	11192913	11193296	HERVHF_gag	-
Gorilla_gorilla_gorilla_chr12	11194926	11195398	HERVHF_3LTR	-
Gorilla_gorilla_gorilla_chr12	16591088	16591523	HERVHF_5LTR	-
Gorilla_gorilla_gorilla_chr12	16591910	16594081	HERVHF_pol	-
Gorilla_gorilla_gorilla_chr12	16593500	16594306	HERVHF_pro	-
Gorilla_gorilla_gorilla_chr12	16596261	16596694	HERVHF_3LTR	-
Gorilla_gorilla_gorilla_chr12	26294360	26294852	HERVHF_5LTR	+
Gorilla_gorilla_gorilla_chr12	26296487	26296855	HERVHF_gag	+
Gorilla_gorilla_gorilla_chr12	26296902	26297378	HERVHF_pro	+
Gorilla_gorilla_gorilla_chr12	26297389	26298881	HERVHF_pol	+
Gorilla_gorilla_gorilla_chr12	26299698	26300184	HERVHF_3LTR	+
Gorilla_gorilla_gorilla_chr12	52784859	52785270	HERVHF_5LTR	-
Gorilla_gorilla_gorilla_chr12	52785724	52788430	HERVHF_pol	-
Gorilla_gorilla_gorilla_chr12	52788861	52789193	HERVHF_gag	-
Gorilla_gorilla_gorilla_chr12	52790805	52791217	HERVHF_3LTR	-
Gorilla_gorilla_gorilla_chr12	53712495	53712943	HERVHF_5LTR	+
Gorilla_gorilla_gorilla_chr12	53715234	53717788	HERVHF_pol	+
Gorilla_gorilla_gorilla_chr12	53718223	53718671	HERVHF_3LTR	+
Gorilla_gorilla_gorilla_chr12	57693411	57693763	HERVHF_5LTR	-
Gorilla_gorilla_gorilla_chr12	57694043	57694959	HERVHF_env	-
Gorilla_gorilla_gorilla_chr12	57695794	57697081	HERVHF_pol	-
Gorilla_gorilla_gorilla_chr12	57696566	57697258	HERVHF_pro	-
Gorilla_gorilla_gorilla_chr12	57699414	57699770	HERVHF_3LTR	-
Gorilla_gorilla_gorilla_chr12	64632329	64632779	HERVHF_5LTR	-
Gorilla_gorilla_gorilla_chr12	64633191	64634950	HERVHF_pol	-
Gorilla_gorilla_gorilla_chr12	64637582	64638034	HERVHF_3LTR	-
Gorilla_gorilla_gorilla_chr12	69884716	69885145	HERVHF_5LTR	-
Gorilla_gorilla_gorilla_chr12	69886353	69887751	HERVHF_pol	-
Gorilla_gorilla_gorilla_chr12	69890187	69890613	HERVHF_3LTR	-
Gorilla_gorilla_gorilla_chr12	76446860	76447313	HERVHF_5LTR	+
Gorilla_gorilla_gorilla_chr12	76448937	76449260	HERVHF_gag	+
Gorilla_gorilla_gorilla_chr12	76449383	76449775	HERVHF_pro	+
Gorilla_gorilla_gorilla_chr12	76449846	76451646	HERVHF_pol	+
Gorilla_gorilla_gorilla_chr12	76452177	76452633	HERVHF_3LTR	+
Gorilla_gorilla_gorilla_chr12	83965502	83965877	HERVHF_5LTR	+
Gorilla_gorilla_gorilla_chr12	83967694	83968023	HERVHF_gag	+
Gorilla_gorilla_gorilla_chr12	83968465	83970464	HERVHF_pol	+
Gorilla_gorilla_gorilla_chr12	83971091	83971481	HERVHF_3LTR	+
Gorilla_gorilla_gorilla_chr12	84938033	84938378	HERVHF_5LTR	-
Gorilla_gorilla_gorilla_chr12	84938874	84940974	HERVHF_pol	-
Gorilla_gorilla_gorilla_chr12	84943216	84943558	HERVHF_3LTR	-
Gorilla_gorilla_gorilla_chr12	96772687	96773106	HERVHF_5LTR	+
Gorilla_gorilla_gorilla_chr12	96774877	96775203	HERVHF_gag	+
Gorilla_gorilla_gorilla_chr12	96775207	96776040	HERVHF_pro	+

Gorilla_gorilla_gorilla_chr12	96775498	96777509	HERVHF_pol	+
Gorilla_gorilla_gorilla_chr12	96778558	96778968	HERVHF_3LTR	+
Gorilla_gorilla_gorilla_chr12	100288162	100289024	HERVHF_5LTR	-
Gorilla_gorilla_gorilla_chr12	100289625	100291819	HERVHF_pol	-
Gorilla_gorilla_gorilla_chr12	100291983	100292303	HERVHF_gag	-
Gorilla_gorilla_gorilla_chr12	100294139	100295020	HERVHF_3LTR	-
Gorilla_gorilla_gorilla_chr12	111113999	111114420	HERVHF_5LTR	+
Gorilla_gorilla_gorilla_chr12	111115524	111116027	HERVHF_gag	+
Gorilla_gorilla_gorilla_chr12	111116752	111120279	HERVHF_pol	+
Gorilla_gorilla_gorilla_chr12	111121480	111122052	HERVHF_env	+
Gorilla_gorilla_gorilla_chr12	111122225	111122654	HERVHF_3LTR	+
Gorilla_gorilla_gorilla_chr13	841418	841916	HERVK_5LTR	+
Gorilla_gorilla_gorilla_chr13	842802	843722	HERVK_pro	+
Gorilla_gorilla_gorilla_chr13	843590	845017	HERVK_pol	+
Gorilla_gorilla_gorilla_chr13	845812	846318	HERVK_3LTR	+
Gorilla_gorilla_gorilla_chr13	17605338	17605805	HERVHF_5LTR	+
Gorilla_gorilla_gorilla_chr13	17607533	17607916	HERVHF_gag	+
Gorilla_gorilla_gorilla_chr13	17608238	17610357	HERVHF_pol	+
Gorilla_gorilla_gorilla_chr13	17610749	17611211	HERVHF_3LTR	+
Gorilla_gorilla_gorilla_chr13	30136242	30136577	HERVHF_5LTR	-
Gorilla_gorilla_gorilla_chr13	30137006	30139468	HERVHF_pol	-
Gorilla_gorilla_gorilla_chr13	30138926	30139801	HERVHF_pro	-
Gorilla_gorilla_gorilla_chr13	30141767	30142105	HERVHF_3LTR	-
Gorilla_gorilla_gorilla_chr13	35432656	35432956	HERVHF_5LTR	-
Gorilla_gorilla_gorilla_chr13	35433545	35435861	HERVHF_pol	-
Gorilla_gorilla_gorilla_chr13	35435223	35435966	HERVHF_pro	-
Gorilla_gorilla_gorilla_chr13	35436064	35436402	HERVHF_gag	-
Gorilla_gorilla_gorilla_chr13	35437969	35438280	HERVHF_3LTR	-
Gorilla_gorilla_gorilla_chr13	36883032	36883484	HERVHF_5LTR	-
Gorilla_gorilla_gorilla_chr13	36884198	36885259	HERVHF_pol	-
Gorilla_gorilla_gorilla_chr13	36885151	36885672	HERVHF_pro	-
Gorilla_gorilla_gorilla_chr13	36885676	36886089	HERVHF_gag	-
Gorilla_gorilla_gorilla_chr13	36887806	36888257	HERVHF_3LTR	-
Gorilla_gorilla_gorilla_chr13	36959170	36959576	HERVHF_5LTR	-
Gorilla_gorilla_gorilla_chr13	36960045	36962271	HERVHF_pol	-
Gorilla_gorilla_gorilla_chr13	36962475	36962798	HERVHF_gag	-
Gorilla_gorilla_gorilla_chr13	36964653	36965050	HERVHF_3LTR	-
Gorilla_gorilla_gorilla_chr13	42397629	42398033	HERVHF_5LTR	+
Gorilla_gorilla_gorilla_chr13	42398770	42399108	HERVHF_gag	+
Gorilla_gorilla_gorilla_chr13	42399460	42401791	HERVHF_pol	+
Gorilla_gorilla_gorilla_chr13	42402824	42403229	HERVHF_3LTR	+
Gorilla_gorilla_gorilla_chr13	47358436	47358763	HERVHF_5LTR	-
Gorilla_gorilla_gorilla_chr13	47360115	47361249	HERVHF_pol	-
Gorilla_gorilla_gorilla_chr13	47361704	47362024	HERVHF_gag	-

Gorilla_gorilla_gorilla_chr13	47364958	47365291	HERVHF_3LTR	-
Gorilla_gorilla_gorilla_chr13	59194189	59194601	HERVHF_5LTR	-
Gorilla_gorilla_gorilla_chr13	59195153	59197289	HERVHF_pol	-
Gorilla_gorilla_gorilla_chr13	59196684	59197484	HERVHF_pro	-
Gorilla_gorilla_gorilla_chr13	59197931	59198434	HERVHF_gag	-
Gorilla_gorilla_gorilla_chr13	59199928	59200343	HERVHF_3LTR	-
Gorilla_gorilla_gorilla_chr13	65156577	65157007	HERVHF_5LTR	-
Gorilla_gorilla_gorilla_chr13	65157453	65159614	HERVHF_pol	-
Gorilla_gorilla_gorilla_chr13	65158997	65159809	HERVHF_pro	-
Gorilla_gorilla_gorilla_chr13	65159813	65160136	HERVHF_gag	-
Gorilla_gorilla_gorilla_chr13	65161747	65162175	HERVHF_3LTR	-
Gorilla_gorilla_gorilla_chr13	67550320	67550775	HERVHF_5LTR	+
Gorilla_gorilla_gorilla_chr13	67552704	67553027	HERVHF_gag	+
Gorilla_gorilla_gorilla_chr13	67553040	67553873	HERVHF_pro	+
Gorilla_gorilla_gorilla_chr13	67553322	67555406	HERVHF_pol	+
Gorilla_gorilla_gorilla_chr13	67555842	67556298	HERVHF_3LTR	+
Gorilla_gorilla_gorilla_chr13	72029474	72029846	HERVHF_5LTR	+
Gorilla_gorilla_gorilla_chr13	72030642	72031424	HERVHF_pro	+
Gorilla_gorilla_gorilla_chr13	72030903	72032796	HERVHF_pol	+
Gorilla_gorilla_gorilla_chr13	72033419	72033785	HERVHF_3LTR	+
Gorilla_gorilla_gorilla_chr14	3806580	3807033	HERVHF_5LTR	-
Gorilla_gorilla_gorilla_chr14	3807442	3809618	HERVHF_pol	-
Gorilla_gorilla_gorilla_chr14	3809036	3810043	HERVHF_pro	-
Gorilla_gorilla_gorilla_chr14	3810047	3810346	HERVHF_gag	-
Gorilla_gorilla_gorilla_chr14	3812064	3812522	HERVHF_3LTR	-
Gorilla_gorilla_gorilla_chr14	27592793	27593095	HERVHF_5LTR	+
Gorilla_gorilla_gorilla_chr14	27595412	27595735	HERVHF_gag	+
Gorilla_gorilla_gorilla_chr14	27595792	27596211	HERVHF_pro	+
Gorilla_gorilla_gorilla_chr14	27596171	27596788	HERVHF_pol	+
Gorilla_gorilla_gorilla_chr14	27597380	27597679	HERVHF_3LTR	+
Gorilla_gorilla_gorilla_chr14	51555848	51556258	HERVHF_5LTR	+
Gorilla_gorilla_gorilla_chr14	51558108	51558446	HERVHF_gag	+
Gorilla_gorilla_gorilla_chr14	51558453	51559070	HERVHF_pro	+
Gorilla_gorilla_gorilla_chr14	51558644	51560648	HERVHF_pol	+
Gorilla_gorilla_gorilla_chr14	51561253	51561662	HERVHF_3LTR	+
Gorilla_gorilla_gorilla_chr14	53559947	53560294	HERVHF_5LTR	-
Gorilla_gorilla_gorilla_chr14	53560460	53560834	HERVHF_env	-
Gorilla_gorilla_gorilla_chr14	53561258	53562751	HERVHF_pol	-
Gorilla_gorilla_gorilla_chr14	53563090	53563545	HERVHF_gag	-
Gorilla_gorilla_gorilla_chr14	53565144	53565513	HERVHF_3LTR	-
Gorilla_gorilla_gorilla_chr14	54111465	54111897	HERVHF_5LTR	+
Gorilla_gorilla_gorilla_chr14	54113708	54114118	HERVHF_gag	+
Gorilla_gorilla_gorilla_chr14	54114125	54114955	HERVHF_pro	+
Gorilla_gorilla_gorilla_chr14	54114317	54116488	HERVHF_pol	+

Gorilla_gorilla_gorilla_chr14	54116939	54117372	HERVHF_3LTR	+
Gorilla_gorilla_gorilla_chr14	64972207	64972639	HERVHF_5LTR	-
Gorilla_gorilla_gorilla_chr14	64973644	64974974	HERVHF_pol	-
Gorilla_gorilla_gorilla_chr14	64974588	64975409	HERVHF_pro	-
Gorilla_gorilla_gorilla_chr14	64975422	64975805	HERVHF_gag	-
Gorilla_gorilla_gorilla_chr14	64977470	64977891	HERVHF_3LTR	-
Gorilla_gorilla_gorilla_chr14	67738635	67739045	HERVHF_5LTR	-
Gorilla_gorilla_gorilla_chr14	67739444	67741981	HERVHF_pol	-
Gorilla_gorilla_gorilla_chr14	67741421	67742230	HERVHF_pro	-
Gorilla_gorilla_gorilla_chr14	67744309	67744719	HERVHF_3LTR	-
Gorilla_gorilla_gorilla_chr14	76490445	76490846	HERVHF_5LTR	+
Gorilla_gorilla_gorilla_chr14	76492506	76492817	HERVHF_gag	+
Gorilla_gorilla_gorilla_chr14	76492850	76493662	HERVHF_pro	+
Gorilla_gorilla_gorilla_chr14	76493045	76495432	HERVHF_pol	+
Gorilla_gorilla_gorilla_chr14	76495884	76496285	HERVHF_3LTR	+
Gorilla_gorilla_gorilla_chr15	10556642	10557093	HERVHF_5LTR	+
Gorilla_gorilla_gorilla_chr15	10558885	10559433	HERVHF_pro	+
Gorilla_gorilla_gorilla_chr15	10560034	10561495	HERVHF_pol	+
Gorilla_gorilla_gorilla_chr15	10561897	10562352	HERVHF_3LTR	+
Gorilla_gorilla_gorilla_chr15	11295066	11295511	HERVHF_5LTR	+
Gorilla_gorilla_gorilla_chr15	11297789	11298427	HERVHF_pro	+
Gorilla_gorilla_gorilla_chr15	11297828	11298601	HERVHF_pol	+
Gorilla_gorilla_gorilla_chr15	11299742	11300175	HERVHF_3LTR	+
Gorilla_gorilla_gorilla_chr15	25624744	25625156	HERVHF_5LTR	-
Gorilla_gorilla_gorilla_chr15	25625324	25626228	HERVHF_env	-
Gorilla_gorilla_gorilla_chr15	25627043	25628349	HERVHF_pol	-
Gorilla_gorilla_gorilla_chr15	25627831	25628349	HERVHF_pro	-
Gorilla_gorilla_gorilla_chr15	25628650	25629147	HERVHF_gag	-
Gorilla_gorilla_gorilla_chr15	25630557	25630960	HERVHF_3LTR	-
Gorilla_gorilla_gorilla_chr15	29324992	29325376	HERVHF_5LTR	-
Gorilla_gorilla_gorilla_chr15	29325826	29327805	HERVHF_pol	-
Gorilla_gorilla_gorilla_chr15	29327590	29328177	HERVHF_pro	-
Gorilla_gorilla_gorilla_chr15	29328255	29328554	HERVHF_gag	-
Gorilla_gorilla_gorilla_chr15	29330158	29330536	HERVHF_3LTR	-
Gorilla_gorilla_gorilla_chr15	50474934	50475332	HERVHF_5LTR	+
Gorilla_gorilla_gorilla_chr15	50476623	50477198	HERVHF_gag	+
Gorilla_gorilla_gorilla_chr15	50477251	50478153	HERVHF_pro	+
Gorilla_gorilla_gorilla_chr15	50477452	50479740	HERVHF_pol	+
Gorilla_gorilla_gorilla_chr15	50480175	50480578	HERVHF_3LTR	+
Gorilla_gorilla_gorilla_chr15	64266971	64267272	HERVHF_5LTR	-
Gorilla_gorilla_gorilla_chr15	64268650	64270162	HERVHF_pol	-
Gorilla_gorilla_gorilla_chr15	64277317	64277620	HERVHF_3LTR	-
Gorilla_gorilla_gorilla_chr15	76234075	76234503	HERVHF_5LTR	+
Gorilla_gorilla_gorilla_chr15	76236338	76236694	HERVHF_gag	+

Gorilla_gorilla_gorilla_chr15	76237545	76238573	HERVHF_pol	+
Gorilla_gorilla_gorilla_chr15	76239709	76240134	HERVHF_3LTR	+
Gorilla_gorilla_gorilla_chr16	9917509	9917960	HERVHF_5LTR	-
Gorilla_gorilla_gorilla_chr16	9918725	9920996	HERVHF_pol	-
Gorilla_gorilla_gorilla_chr16	9921516	9922043	HERVHF_gag	-
Gorilla_gorilla_gorilla_chr16	9923697	9924148	HERVHF_3LTR	-
Gorilla_gorilla_gorilla_chr16	45460378	45460830	HERVHF_5LTR	+
Gorilla_gorilla_gorilla_chr16	45462633	45463013	HERVHF_gag	+
Gorilla_gorilla_gorilla_chr16	45463060	45463932	HERVHF_pro	+
Gorilla_gorilla_gorilla_chr16	45463297	45465430	HERVHF_pol	+
Gorilla_gorilla_gorilla_chr16	45465872	45466324	HERVHF_3LTR	+
Gorilla_gorilla_gorilla_chr16	50623004	50623460	HERVHF_5LTR	-
Gorilla_gorilla_gorilla_chr16	50623916	50625778	HERVHF_pol	-
Gorilla_gorilla_gorilla_chr16	50625461	50626288	HERVHF_pro	-
Gorilla_gorilla_gorilla_chr16	50628109	50628562	HERVHF_3LTR	-
Gorilla_gorilla_gorilla_chr16	55864374	55865228	HERVHF_5LTR	-
Gorilla_gorilla_gorilla_chr16	55867090	55868749	HERVHF_pol	-
Gorilla_gorilla_gorilla_chr16	55869005	55869862	HERVHF_3LTR	-
Gorilla_gorilla_gorilla_chr17	11893030	11893463	HERVHF_5LTR	+
Gorilla_gorilla_gorilla_chr17	11895768	11896484	HERVHF_pro	+
Gorilla_gorilla_gorilla_chr17	11895975	11897245	HERVHF_pol	+
Gorilla_gorilla_gorilla_chr17	11897837	11898270	HERVHF_3LTR	+
Gorilla_gorilla_gorilla_chr17	19653560	19653987	HERVHF_5LTR	+
Gorilla_gorilla_gorilla_chr17	19656015	19656908	HERVHF_gag	+
Gorilla_gorilla_gorilla_chr17	19657107	19659365	HERVHF_pol	+
Gorilla_gorilla_gorilla_chr17	19659707	19660051	HERVHF_env	+
Gorilla_gorilla_gorilla_chr17	19660217	19660633	HERVHF_3LTR	+
Gorilla_gorilla_gorilla_chr17	20180531	20180982	HERVHF_5LTR	-
Gorilla_gorilla_gorilla_chr17	20181434	20183581	HERVHF_pol	-
Gorilla_gorilla_gorilla_chr17	20182970	20183788	HERVHF_pro	-
Gorilla_gorilla_gorilla_chr17	20183778	20184194	HERVHF_gag	-
Gorilla_gorilla_gorilla_chr17	20185925	20186376	HERVHF_3LTR	-
Gorilla_gorilla_gorilla_chr17	42851916	42852378	HERVK_5LTR	-
Gorilla_gorilla_gorilla_chr17	42853382	42854851	HERVK_pol	-
Gorilla_gorilla_gorilla_chr17	42854909	42855688	HERVK_pro	-
Gorilla_gorilla_gorilla_chr17	42857133	42857604	HERVK_3LTR	-
Gorilla_gorilla_gorilla_chr17	73803524	73803971	HERVHF_5LTR	+
Gorilla_gorilla_gorilla_chr17	73805631	73806047	HERVHF_gag	+
Gorilla_gorilla_gorilla_chr17	73806054	73806905	HERVHF_pro	+
Gorilla_gorilla_gorilla_chr17	73806246	73808282	HERVHF_pol	+
Gorilla_gorilla_gorilla_chr17	73808873	73809326	HERVHF_3LTR	+
Gorilla_gorilla_gorilla_chr17	83438784	83439281	HERVHF_5LTR	+
Gorilla_gorilla_gorilla_chr17	83439286	83440048	HERVHF_gag	+
Gorilla_gorilla_gorilla_chr17	83440038	83440886	HERVHF_pro	+

Gorilla_gorilla_gorilla_chr17	83440248	83442677	HERVHF_pol	+
Gorilla_gorilla_gorilla_chr17	83451346	83451849	HERVHF_3LTR	+
Gorilla_gorilla_gorilla_chr17	91671514	91671943	HERVHF_5LTR	-
Gorilla_gorilla_gorilla_chr17	91672353	91674509	HERVHF_pol	-
Gorilla_gorilla_gorilla_chr17	91673928	91674758	HERVHF_pro	-
Gorilla_gorilla_gorilla_chr17	91674768	91675094	HERVHF_gag	-
Gorilla_gorilla_gorilla_chr17	91676917	91677347	HERVHF_3LTR	-
Gorilla_gorilla_gorilla_chr18	28266107	28266562	HERVHF_5LTR	+
Gorilla_gorilla_gorilla_chr18	28267732	28268971	HERVHF_gag	+
Gorilla_gorilla_gorilla_chr18	28269457	28270268	HERVHF_pol	+
Gorilla_gorilla_gorilla_chr18	28271860	28272581	HERVHF_env	+
Gorilla_gorilla_gorilla_chr18	28273255	28273727	HERVHF_3LTR	+
Gorilla_gorilla_gorilla_chr18	28891086	28891439	HERVHF_5LTR	+
Gorilla_gorilla_gorilla_chr18	28892710	28893282	HERVHF_gag	+
Gorilla_gorilla_gorilla_chr18	28893455	28894309	HERVHF_pro	+
Gorilla_gorilla_gorilla_chr18	28893569	28895611	HERVHF_pol	+
Gorilla_gorilla_gorilla_chr18	28897013	28897360	HERVHF_3LTR	+
Gorilla_gorilla_gorilla_chr18	35770933	35771328	HERVHF_5LTR	+
Gorilla_gorilla_gorilla_chr18	35773097	35773852	HERVHF_pro	+
Gorilla_gorilla_gorilla_chr18	35773331	35775270	HERVHF_pol	+
Gorilla_gorilla_gorilla_chr18	35775848	35776245	HERVHF_3LTR	+
Gorilla_gorilla_gorilla_chr18	36027624	36027983	HERVHF_5LTR	+
Gorilla_gorilla_gorilla_chr18	36028936	36029259	HERVHF_gag	+
Gorilla_gorilla_gorilla_chr18	36029026	36029778	HERVHF_pro	+
Gorilla_gorilla_gorilla_chr18	36029678	36031947	HERVHF_pol	+
Gorilla_gorilla_gorilla_chr18	36032458	36032805	HERVHF_3LTR	+
Gorilla_gorilla_gorilla_chr18	49426111	49426562	HERVHF_5LTR	+
Gorilla_gorilla_gorilla_chr18	49428334	49428738	HERVHF_gag	+
Gorilla_gorilla_gorilla_chr18	49428839	49429555	HERVHF_pro	+
Gorilla_gorilla_gorilla_chr18	49429040	49431162	HERVHF_pol	+
Gorilla_gorilla_gorilla_chr18	49431566	49432017	HERVHF_3LTR	+
Gorilla_gorilla_gorilla_chr18	66350160	66350593	HERVHF_5LTR	+
Gorilla_gorilla_gorilla_chr18	66352334	66352654	HERVHF_gag	+
Gorilla_gorilla_gorilla_chr18	66352825	66353508	HERVHF_pro	+
Gorilla_gorilla_gorilla_chr18	66352909	66354880	HERVHF_pol	+
Gorilla_gorilla_gorilla_chr18	66355472	66355905	HERVHF_3LTR	+
Gorilla_gorilla_gorilla_chr19	23324258	23324576	HERVK_5LTR	-
Gorilla_gorilla_gorilla_chr19	23325486	23326751	HERVK_pol	-
Gorilla_gorilla_gorilla_chr19	23326646	23327424	HERVK_pro	-
Gorilla_gorilla_gorilla_chr19	23328482	23328816	HERVK_3LTR	-
Gorilla_gorilla_gorilla_chr19	39699377	39699815	HERVHF_5LTR	-
Gorilla_gorilla_gorilla_chr19	39700403	39702230	HERVHF_pol	-
Gorilla_gorilla_gorilla_chr19	39702651	39702950	HERVHF_gag	-
Gorilla_gorilla_gorilla_chr19	39705027	39705461	HERVHF_3LTR	-

Gorilla_gorilla_gorilla_chr19	42499350	42499695	HERVHF_5LTR	-
Gorilla_gorilla_gorilla_chr19	42499964	42500302	HERVHF_env	-
Gorilla_gorilla_gorilla_chr19	42500800	42504061	HERVHF_pol	-
Gorilla_gorilla_gorilla_chr19	42503423	42504178	HERVHF_pro	-
Gorilla_gorilla_gorilla_chr19	42506558	42506896	HERVHF_3LTR	-
Gorilla_gorilla_gorilla_chr19	42667809	42668225	HERVHF_5LTR	-
Gorilla_gorilla_gorilla_chr19	42668827	42671014	HERVHF_pol	-
Gorilla_gorilla_gorilla_chr19	42670376	42671155	HERVHF_pro	-
Gorilla_gorilla_gorilla_chr19	42673045	42673465	HERVHF_3LTR	-
Gorilla_gorilla_gorilla_chr19	43459960	43460390	HERVHF_5LTR	-
Gorilla_gorilla_gorilla_chr19	43460780	43462769	HERVHF_pol	-
Gorilla_gorilla_gorilla_chr19	43462320	43462853	HERVHF_pro	-
Gorilla_gorilla_gorilla_chr19	43463124	43463423	HERVHF_gag	-
Gorilla_gorilla_gorilla_chr19	43465062	43465492	HERVHF_3LTR	-
Gorilla_gorilla_gorilla_chr19	43807862	43808305	HERVHF_5LTR	+
Gorilla_gorilla_gorilla_chr19	43809709	43810119	HERVHF_gag	+
Gorilla_gorilla_gorilla_chr19	43810801	43812978	HERVHF_pol	+
Gorilla_gorilla_gorilla_chr19	43813376	43813819	HERVHF_3LTR	+
Gorilla_gorilla_gorilla_chr20	12290208	12290537	HERVHF_5LTR	-
Gorilla_gorilla_gorilla_chr20	12290990	12292946	HERVHF_pol	-
Gorilla_gorilla_gorilla_chr20	12293364	12293687	HERVHF_gag	-
Gorilla_gorilla_gorilla_chr20	12295740	12296062	HERVHF_3LTR	-
Gorilla_gorilla_gorilla_chr20	12706987	12707397	HERVHF_5LTR	+
Gorilla_gorilla_gorilla_chr20	12709090	12709800	HERVHF_pro	+
Gorilla_gorilla_gorilla_chr20	12709288	12711228	HERVHF_pol	+
Gorilla_gorilla_gorilla_chr20	12711866	12712280	HERVHF_3LTR	+
Gorilla_gorilla_gorilla_chr20	13312056	13312509	HERVHF_5LTR	+
Gorilla_gorilla_gorilla_chr20	13314211	13314534	HERVHF_gag	+
Gorilla_gorilla_gorilla_chr20	13314963	13316935	HERVHF_pol	+
Gorilla_gorilla_gorilla_chr20	13317340	13317792	HERVHF_3LTR	+
Gorilla_gorilla_gorilla_chr20	19743841	19744276	HERVHF_5LTR	-
Gorilla_gorilla_gorilla_chr20	19744693	19745874	HERVHF_pol	-
Gorilla_gorilla_gorilla_chr20	19745383	19746207	HERVHF_pro	-
Gorilla_gorilla_gorilla_chr20	19748276	19748705	HERVHF_3LTR	-
Gorilla_gorilla_gorilla_chr20	36017199	36017655	HERVHF_5LTR	+
Gorilla_gorilla_gorilla_chr20	36019963	36020715	HERVHF_pro	+
Gorilla_gorilla_gorilla_chr20	36020380	36022266	HERVHF_pol	+
Gorilla_gorilla_gorilla_chr20	36022675	36023126	HERVHF_3LTR	+
Gorilla_gorilla_gorilla_chr20	36198983	36199445	HERVHF_5LTR	+
Gorilla_gorilla_gorilla_chr20	36201336	36201635	HERVHF_gag	+
Gorilla_gorilla_gorilla_chr20	36201662	36202492	HERVHF_pro	+
Gorilla_gorilla_gorilla_chr20	36201950	36204047	HERVHF_pol	+
Gorilla_gorilla_gorilla_chr20	36204457	36204914	HERVHF_3LTR	+
Gorilla_gorilla_gorilla_chr21	3216438	3216752	HERVHF_5LTR	+

Gorilla_gorilla_gorilla_chr21	3218125	3218559	HERVHF_gag	+
Gorilla_gorilla_gorilla_chr21	3218754	3219482	HERVHF_pro	+
Gorilla_gorilla_gorilla_chr21	3219102	3221425	HERVHF_pol	+
Gorilla_gorilla_gorilla_chr21	3221868	3222195	HERVHF_3LTR	+
Gorilla_gorilla_gorilla_chr21	12740363	12740780	HERVHF_5LTR	-
Gorilla_gorilla_gorilla_chr21	12741178	12743548	HERVHF_pol	-
Gorilla_gorilla_gorilla_chr21	12743884	12744234	HERVHF_gag	-
Gorilla_gorilla_gorilla_chr21	12745598	12746010	HERVHF_3LTR	-
Gorilla_gorilla_gorilla_chr21	27458488	27458939	HERVHF_5LTR	+
Gorilla_gorilla_gorilla_chr21	27460685	27461095	HERVHF_gag	+
Gorilla_gorilla_gorilla_chr21	27461101	27461637	HERVHF_pro	+
Gorilla_gorilla_gorilla_chr21	27461574	27463510	HERVHF_pol	+
Gorilla_gorilla_gorilla_chr21	27463540	27463901	HERVHF_env	+
Gorilla_gorilla_gorilla_chr21	27463913	27464365	HERVHF_3LTR	+
Gorilla_gorilla_gorilla_chr21	29296549	29296995	HERVHF_5LTR	-
Gorilla_gorilla_gorilla_chr21	29297416	29299778	HERVHF_pol	-
Gorilla_gorilla_gorilla_chr21	29301852	29302299	HERVHF_3LTR	-
Gorilla_gorilla_gorilla_chr22	16021673	16022106	HERVHF_5LTR	+
Gorilla_gorilla_gorilla_chr22	16023929	16024228	HERVHF_gag	+
Gorilla_gorilla_gorilla_chr22	16024719	16026233	HERVHF_pol	+
Gorilla_gorilla_gorilla_chr22	16027060	16027492	HERVHF_3LTR	+
Gorilla_gorilla_gorilla_chr2a	28001034	28001463	HERVHF_5LTR	-
Gorilla_gorilla_gorilla_chr2a	28001915	28004046	HERVHF_pol	-
Gorilla_gorilla_gorilla_chr2a	28003818	28004264	HERVHF_pro	-
Gorilla_gorilla_gorilla_chr2a	28004298	28004702	HERVHF_gag	-
Gorilla_gorilla_gorilla_chr2a	28006282	28006713	HERVHF_3LTR	-
Gorilla_gorilla_gorilla_chr2a	34762117	34762546	HERVHF_5LTR	+
Gorilla_gorilla_gorilla_chr2a	34764594	34764974	HERVHF_gag	+
Gorilla_gorilla_gorilla_chr2a	34764993	34765814	HERVHF_pro	+
Gorilla_gorilla_gorilla_chr2a	34765257	34767539	HERVHF_pol	+
Gorilla_gorilla_gorilla_chr2a	34767987	34768417	HERVHF_3LTR	+
Gorilla_gorilla_gorilla_chr2a	34835979	34836310	HERVHF_5LTR	+
Gorilla_gorilla_gorilla_chr2a	34837988	34838812	HERVHF_pro	+
Gorilla_gorilla_gorilla_chr2a	34838195	34838962	HERVHF_pol	+
Gorilla_gorilla_gorilla_chr2a	34842390	34842717	HERVHF_3LTR	+
Gorilla_gorilla_gorilla_chr2a	35276403	35276809	HERVHF_5LTR	-
Gorilla_gorilla_gorilla_chr2a	35277258	35279286	HERVHF_pol	-
Gorilla_gorilla_gorilla_chr2a	35278882	35279457	HERVHF_pro	-
Gorilla_gorilla_gorilla_chr2a	35279826	35280125	HERVHF_gag	-
Gorilla_gorilla_gorilla_chr2a	35281869	35282273	HERVHF_3LTR	-
Gorilla_gorilla_gorilla_chr2a	36119166	36119536	HUERSP_5LTR	+
Gorilla_gorilla_gorilla_chr2a	36122602	36123012	HUERSP_pro	+
Gorilla_gorilla_gorilla_chr2a	36122629	36126140	HUERSP_pol	+
Gorilla_gorilla_gorilla_chr2a	36129236	36129612	HUERSP_3LTR	+

Gorilla_gorilla_gorilla_chr2a	39725352	39725795	HERVHF_5LTR	-
Gorilla_gorilla_gorilla_chr2a	39726679	39728642	HERVHF_pol	-
Gorilla_gorilla_gorilla_chr2a	39728082	39728894	HERVHF_pro	-
Gorilla_gorilla_gorilla_chr2a	39728918	39729223	HERVHF_gag	-
Gorilla_gorilla_gorilla_chr2a	39731722	39732174	HERVHF_3LTR	-
Gorilla_gorilla_gorilla_chr2a	45628265	45628737	HERVHF_5LTR	-
Gorilla_gorilla_gorilla_chr2a	45629154	45630432	HERVHF_pol	-
Gorilla_gorilla_gorilla_chr2a	45629851	45630681	HERVHF_pro	-
Gorilla_gorilla_gorilla_chr2a	45630708	45631007	HERVHF_gag	-
Gorilla_gorilla_gorilla_chr2a	45632756	45633229	HERVHF_3LTR	-
Gorilla_gorilla_gorilla_chr2a	47902222	47902579	HERVHF_5LTR	+
Gorilla_gorilla_gorilla_chr2a	47904633	47905409	HERVHF_pro	+
Gorilla_gorilla_gorilla_chr2a	47904852	47907012	HERVHF_pol	+
Gorilla_gorilla_gorilla_chr2a	47907489	47907846	HERVHF_3LTR	+
Gorilla_gorilla_gorilla_chr2a	49308854	49309327	HERVK_5LTR	-
Gorilla_gorilla_gorilla_chr2a	49309491	49309844	HERVK_env	-
Gorilla_gorilla_gorilla_chr2a	49310449	49311249	HERVK_pol	-
Gorilla_gorilla_gorilla_chr2a	49311037	49312311	HERVK_pro	-
Gorilla_gorilla_gorilla_chr2a	49312085	49313126	HERVK_gag	-
Gorilla_gorilla_gorilla_chr2a	49313550	49314013	HERVK_3LTR	-
Gorilla_gorilla_gorilla_chr2a	64759050	64759480	HERVHF_5LTR	-
Gorilla_gorilla_gorilla_chr2a	64759649	64760092	HERVHF_env	-
Gorilla_gorilla_gorilla_chr2a	64760410	64763483	HERVHF_pol	-
Gorilla_gorilla_gorilla_chr2a	64763530	64764336	HERVHF_pro	-
Gorilla_gorilla_gorilla_chr2a	64764737	64765708	HERVHF_gag	-
Gorilla_gorilla_gorilla_chr2a	64766488	64766919	HERVHF_3LTR	-
Gorilla_gorilla_gorilla_chr2a	69646045	69646475	HERVHF_5LTR	-
Gorilla_gorilla_gorilla_chr2a	69647079	69649117	HERVHF_pol	-
Gorilla_gorilla_gorilla_chr2a	69648479	69649228	HERVHF_pro	-
Gorilla_gorilla_gorilla_chr2a	69649315	69649728	HERVHF_gag	-
Gorilla_gorilla_gorilla_chr2a	69651522	69651952	HERVHF_3LTR	-
Gorilla_gorilla_gorilla_chr2a	70840472	70840907	HERVHF_5LTR	+
Gorilla_gorilla_gorilla_chr2a	70842986	70843588	HERVHF_pro	+
Gorilla_gorilla_gorilla_chr2a	70842989	70845109	HERVHF_pol	+
Gorilla_gorilla_gorilla_chr2a	70845737	70846165	HERVHF_3LTR	+
Gorilla_gorilla_gorilla_chr2a	86196870	86197279	HERVHF_5LTR	-
Gorilla_gorilla_gorilla_chr2a	86197730	86199755	HERVHF_pol	-
Gorilla_gorilla_gorilla_chr2a	86199776	86200231	HERVHF_pro	-
Gorilla_gorilla_gorilla_chr2a	86200191	86200520	HERVHF_gag	-
Gorilla_gorilla_gorilla_chr2a	86201975	86202389	HERVHF_3LTR	-
Gorilla_gorilla_gorilla_chr2a	93101844	93102308	HERVHF_5LTR	+
Gorilla_gorilla_gorilla_chr2a	93103978	93104349	HERVHF_gag	+
Gorilla_gorilla_gorilla_chr2a	93104488	93105228	HERVHF_pro	+
Gorilla_gorilla_gorilla_chr2a	93104584	93107028	HERVHF_pol	+

Gorilla_gorilla_gorilla_chr2a	93107418	93107899	HERVHF_3LTR	+
Gorilla_gorilla_gorilla_chr2a	93638163	93638615	HERVHF_5LTR	-
Gorilla_gorilla_gorilla_chr2a	93639130	93641003	HERVHF_pol	-
Gorilla_gorilla_gorilla_chr2a	93640596	93641126	HERVHF_pro	-
Gorilla_gorilla_gorilla_chr2a	93641458	93641838	HERVHF_gag	-
Gorilla_gorilla_gorilla_chr2a	93643764	93644214	HERVHF_3LTR	-
Gorilla_gorilla_gorilla_chr2a	98424328	98424729	HERVHF_5LTR	+
Gorilla_gorilla_gorilla_chr2a	98426945	98427883	HERVHF_pro	+
Gorilla_gorilla_gorilla_chr2a	98427155	98429293	HERVHF_pol	+
Gorilla_gorilla_gorilla_chr2a	98429262	98429573	HERVHF_env	+
Gorilla_gorilla_gorilla_chr2a	98429784	98430174	HERVHF_3LTR	+
Gorilla_gorilla_gorilla_chr2b	36404606	36405072	HERVHF_5LTR	+
Gorilla_gorilla_gorilla_chr2b	36407185	36407748	HERVHF_pro	+
Gorilla_gorilla_gorilla_chr2b	36407215	36409540	HERVHF_pol	+
Gorilla_gorilla_gorilla_chr2b	36409928	36410369	HERVHF_3LTR	+
Gorilla_gorilla_gorilla_chr2b	41145474	41145975	HERVHF_5LTR	+
Gorilla_gorilla_gorilla_chr2b	41148463	41149652	HERVHF_pol	+
Gorilla_gorilla_gorilla_chr2b	41151300	41152112	HERVHF_env	+
Gorilla_gorilla_gorilla_chr2b	41152339	41152846	HERVHF_3LTR	+
Gorilla_gorilla_gorilla_chr2b	50559274	50559724	HERVHF_5LTR	+
Gorilla_gorilla_gorilla_chr2b	50561795	50562628	HERVHF_pro	+
Gorilla_gorilla_gorilla_chr2b	50561990	50564167	HERVHF_pol	+
Gorilla_gorilla_gorilla_chr2b	50564582	50565037	HERVHF_3LTR	+
Gorilla_gorilla_gorilla_chr2b	73942677	73943058	HERVHF_5LTR	-
Gorilla_gorilla_gorilla_chr2b	73943378	73944202	HERVHF_env	-
Gorilla_gorilla_gorilla_chr2b	73946048	73948218	HERVHF_pol	-
Gorilla_gorilla_gorilla_chr2b	73947637	73948269	HERVHF_pro	-
Gorilla_gorilla_gorilla_chr2b	73948496	73948876	HERVHF_gag	-
Gorilla_gorilla_gorilla_chr2b	73950453	73950844	HERVHF_3LTR	-
Gorilla_gorilla_gorilla_chr2b	74577720	74578132	HERVHF_5LTR	-
Gorilla_gorilla_gorilla_chr2b	74579132	74581295	HERVHF_pol	-
Gorilla_gorilla_gorilla_chr2b	74580678	74581490	HERVHF_pro	-
Gorilla_gorilla_gorilla_chr2b	74583366	74583780	HERVHF_3LTR	-
Gorilla_gorilla_gorilla_chr2b	78541154	78541587	HERVHF_5LTR	+
Gorilla_gorilla_gorilla_chr2b	78542615	78543022	HERVHF_gag	+
Gorilla_gorilla_gorilla_chr2b	78542840	78543986	HERVHF_pro	+
Gorilla_gorilla_gorilla_chr2b	78543447	78545350	HERVHF_pol	+
Gorilla_gorilla_gorilla_chr2b	78545923	78546370	HERVHF_3LTR	+
Gorilla_gorilla_gorilla_chr2b	78912263	78912678	HERVHF_5LTR	+
Gorilla_gorilla_gorilla_chr2b	78913609	78916076	HERVHF_gag	+
Gorilla_gorilla_gorilla_chr2b	78916390	78916941	HERVHF_pro	+
Gorilla_gorilla_gorilla_chr2b	78916564	78918504	HERVHF_pol	+
Gorilla_gorilla_gorilla_chr2b	78918955	78919370	HERVHF_3LTR	+
Gorilla_gorilla_gorilla_chr2b	83899982	83900345	HERVHF_5LTR	+

Gorilla_gorilla_gorilla_chr2b	83902218	83902688	HERVHF_pro	+
Gorilla_gorilla_gorilla_chr2b	83903006	83905024	HERVHF_pol	+
Gorilla_gorilla_gorilla_chr2b	83907599	83908732	HERVHF_env	+
Gorilla_gorilla_gorilla_chr2b	83908959	83909315	HERVHF_3LTR	+
Gorilla_gorilla_gorilla_chr2b	93529319	93529730	HERVHF_5LTR	+
Gorilla_gorilla_gorilla_chr2b	93532177	93533927	HERVHF_pol	+
Gorilla_gorilla_gorilla_chr2b	93534968	93535381	HERVHF_3LTR	+
Gorilla_gorilla_gorilla_chr2b	95607061	95607458	HERVHF_5LTR	+
Gorilla_gorilla_gorilla_chr2b	95609230	95609550	HERVHF_gag	+
Gorilla_gorilla_gorilla_chr2b	95609974	95612090	HERVHF_pol	+
Gorilla_gorilla_gorilla_chr2b	95612926	95613310	HERVHF_3LTR	+
Gorilla_gorilla_gorilla_chr2b	95816830	95817130	HERVHF_5LTR	+
Gorilla_gorilla_gorilla_chr2b	95819442	95820260	HERVHF_pro	+
Gorilla_gorilla_gorilla_chr2b	95819739	95821800	HERVHF_pol	+
Gorilla_gorilla_gorilla_chr2b	95822222	95822526	HERVHF_3LTR	+
Gorilla_gorilla_gorilla_chr2b	101393468	101393941	HERVHF_5LTR	-
Gorilla_gorilla_gorilla_chr2b	101394352	101396909	HERVHF_pol	-
Gorilla_gorilla_gorilla_chr2b	101396346	101397152	HERVHF_pro	-
Gorilla_gorilla_gorilla_chr2b	101397207	101397616	HERVHF_gag	-
Gorilla_gorilla_gorilla_chr2b	101399420	101399888	HERVHF_3LTR	-
Gorilla_gorilla_gorilla_chr2b	101651096	101651432	HERVHF_5LTR	+
Gorilla_gorilla_gorilla_chr2b	101652839	101653195	HERVHF_gag	+
Gorilla_gorilla_gorilla_chr2b	101653499	101655661	HERVHF_pol	+
Gorilla_gorilla_gorilla_chr2b	101656815	101657155	HERVHF_3LTR	+
Gorilla_gorilla_gorilla_chr2b	102494801	102495258	HERVHF_5LTR	+
Gorilla_gorilla_gorilla_chr2b	102497012	102497332	HERVHF_gag	+
Gorilla_gorilla_gorilla_chr2b	102497420	102498169	HERVHF_pro	+
Gorilla_gorilla_gorilla_chr2b	102497627	102499709	HERVHF_pol	+
Gorilla_gorilla_gorilla_chr2b	102500161	102500614	HERVHF_3LTR	+
Gorilla_gorilla_gorilla_chr2b	110917816	110918228	HERVHF_5LTR	+
Gorilla_gorilla_gorilla_chr2b	110919912	110920319	HERVHF_gag	+
Gorilla_gorilla_gorilla_chr2b	110920828	110922724	HERVHF_pol	+
Gorilla_gorilla_gorilla_chr2b	110923107	110923532	HERVHF_3LTR	+
Gorilla_gorilla_gorilla_chr3	941721	942135	HERVHF_5LTR	-
Gorilla_gorilla_gorilla_chr3	942589	944763	HERVHF_pol	-
Gorilla_gorilla_gorilla_chr3	944125	944958	HERVHF_pro	-
Gorilla_gorilla_gorilla_chr3	944991	945500	HERVHF_gag	-
Gorilla_gorilla_gorilla_chr3	947026	947440	HERVHF_3LTR	-
Gorilla_gorilla_gorilla_chr3	8235459	8235871	HERVHF_5LTR	+
Gorilla_gorilla_gorilla_chr3	8237514	8237897	HERVHF_gag	+
Gorilla_gorilla_gorilla_chr3	8238171	8238752	HERVHF_pro	+
Gorilla_gorilla_gorilla_chr3	8238312	8240162	HERVHF_pol	+
Gorilla_gorilla_gorilla_chr3	8240734	8241145	HERVHF_3LTR	+
Gorilla_gorilla_gorilla_chr3	21293393	21293800	HERVHF_5LTR	-

Gorilla_gorilla_gorilla_chr3	21294236	21296411	HERVHF_pol	-
Gorilla_gorilla_gorilla_chr3	21296825	21297166	HERVHF_gag	-
Gorilla_gorilla_gorilla_chr3	21298167	21298570	HERVHF_3LTR	-
Gorilla_gorilla_gorilla_chr3	32590515	32590859	HERVHF_5LTR	-
Gorilla_gorilla_gorilla_chr3	32591682	32593997	HERVHF_pol	-
Gorilla_gorilla_gorilla_chr3	32593359	32594201	HERVHF_pro	-
Gorilla_gorilla_gorilla_chr3	32594191	32594631	HERVHF_gag	-
Gorilla_gorilla_gorilla_chr3	32596053	32596393	HERVHF_3LTR	-
Gorilla_gorilla_gorilla_chr3	36049346	36049678	HERVHF_5LTR	-
Gorilla_gorilla_gorilla_chr3	36050294	36052009	HERVHF_pol	-
Gorilla_gorilla_gorilla_chr3	36051692	36052471	HERVHF_pro	-
Gorilla_gorilla_gorilla_chr3	36052538	36052957	HERVHF_gag	-
Gorilla_gorilla_gorilla_chr3	36054559	36054892	HERVHF_3LTR	-
Gorilla_gorilla_gorilla_chr3	46413391	46413847	HERVHF_5LTR	-
Gorilla_gorilla_gorilla_chr3	46414378	46416393	HERVHF_pol	-
Gorilla_gorilla_gorilla_chr3	46418893	46419347	HERVHF_3LTR	-
Gorilla_gorilla_gorilla_chr3	46554275	46554715	HERVHF_5LTR	-
Gorilla_gorilla_gorilla_chr3	46554884	46555258	HERVHF_env	-
Gorilla_gorilla_gorilla_chr3	46556180	46557453	HERVHF_pol	-
Gorilla_gorilla_gorilla_chr3	46559554	46559995	HERVHF_3LTR	-
Gorilla_gorilla_gorilla_chr3	78395143	78395594	HERVHF_5LTR	+
Gorilla_gorilla_gorilla_chr3	78398102	78398683	HERVHF_pro	+
Gorilla_gorilla_gorilla_chr3	78398141	78400274	HERVHF_pol	+
Gorilla_gorilla_gorilla_chr3	78400678	78401130	HERVHF_3LTR	+
Gorilla_gorilla_gorilla_chr3	87672090	87672411	HERVHF_5LTR	+
Gorilla_gorilla_gorilla_chr3	87674251	87674694	HERVHF_pro	+
Gorilla_gorilla_gorilla_chr3	87674643	87676801	HERVHF_pol	+
Gorilla_gorilla_gorilla_chr3	87677235	87677579	HERVHF_env	+
Gorilla_gorilla_gorilla_chr3	87677823	87678127	HERVHF_3LTR	+
Gorilla_gorilla_gorilla_chr3	91536924	91537306	HERVHF_5LTR	+
Gorilla_gorilla_gorilla_chr3	91538813	91539154	HERVHF_gag	+
Gorilla_gorilla_gorilla_chr3	91539161	91539952	HERVHF_pro	+
Gorilla_gorilla_gorilla_chr3	91539455	91541825	HERVHF_pol	+
Gorilla_gorilla_gorilla_chr3	91542539	91542933	HERVHF_3LTR	+
Gorilla_gorilla_gorilla_chr3	102058053	102058488	HERVHF_5LTR	-
Gorilla_gorilla_gorilla_chr3	102058899	102061011	HERVHF_pol	-
Gorilla_gorilla_gorilla_chr3	102060469	102061290	HERVHF_pro	-
Gorilla_gorilla_gorilla_chr3	102061297	102061710	HERVHF_gag	-
Gorilla_gorilla_gorilla_chr3	102063378	102063812	HERVHF_3LTR	-
Gorilla_gorilla_gorilla_chr3	103219442	103219856	HSERVIII_5LTR	-
Gorilla_gorilla_gorilla_chr3	103220525	103223475	HSERVIII_pol	-
Gorilla_gorilla_gorilla_chr3	103225903	103226314	HSERVIII_3LTR	-
Gorilla_gorilla_gorilla_chr3	110062920	110063374	HERVHF_5LTR	-
Gorilla_gorilla_gorilla_chr3	110063705	110065036	HERVHF_pol	-

Gorilla_gorilla_gorilla_chr3	110064521	110065243	HERVHF_pro	-
Gorilla_gorilla_gorilla_chr3	110067372	110067828	HERVHF_3LTR	-
Gorilla_gorilla_gorilla_chr3	113446881	113447334	HERVHF_5LTR	-
Gorilla_gorilla_gorilla_chr3	113447928	113449868	HERVHF_pol	-
Gorilla_gorilla_gorilla_chr3	113449305	113450075	HERVHF_pro	-
Gorilla_gorilla_gorilla_chr3	113450163	113450483	HERVHF_gag	-
Gorilla_gorilla_gorilla_chr3	113452222	113452674	HERVHF_3LTR	-
Gorilla_gorilla_gorilla_chr3	122391329	122391662	HERVHF_5LTR	-
Gorilla_gorilla_gorilla_chr3	122392072	122394236	HERVHF_pol	-
Gorilla_gorilla_gorilla_chr3	122393634	122394347	HERVHF_pro	-
Gorilla_gorilla_gorilla_chr3	122396607	122396945	HERVHF_3LTR	-
Gorilla_gorilla_gorilla_chr3	126316470	126316829	HERVHF_5LTR	-
Gorilla_gorilla_gorilla_chr3	126317370	126319079	HERVHF_pol	-
Gorilla_gorilla_gorilla_chr3	126318654	126319253	HERVHF_pro	-
Gorilla_gorilla_gorilla_chr3	126323212	126323570	HERVHF_3LTR	-
Gorilla_gorilla_gorilla_chr3	127536111	127536539	HERVHF_5LTR	+
Gorilla_gorilla_gorilla_chr3	127539129	127541106	HERVHF_pol	+
Gorilla_gorilla_gorilla_chr3	127541525	127541948	HERVHF_3LTR	+
Gorilla_gorilla_gorilla_chr3	135024275	135024737	HERVHF_5LTR	+
Gorilla_gorilla_gorilla_chr3	135025985	135026586	HERVHF_gag	+
Gorilla_gorilla_gorilla_chr3	135026598	135027386	HERVHF_pro	+
Gorilla_gorilla_gorilla_chr3	135026889	135029092	HERVHF_pol	+
Gorilla_gorilla_gorilla_chr3	135029350	135029807	HERVHF_3LTR	+
Gorilla_gorilla_gorilla_chr3	141242173	141242522	HERVHF_5LTR	+
Gorilla_gorilla_gorilla_chr3	141244054	141244353	HERVHF_gag	+
Gorilla_gorilla_gorilla_chr3	141244700	141248314	HERVHF_pol	+
Gorilla_gorilla_gorilla_chr3	141248736	141249884	HERVHF_env	+
Gorilla_gorilla_gorilla_chr3	141250145	141250490	HERVHF_3LTR	+
Gorilla_gorilla_gorilla_chr3	145849261	145849717	HERVHF_5LTR	+
Gorilla_gorilla_gorilla_chr3	145851887	145852693	HERVHF_pro	+
Gorilla_gorilla_gorilla_chr3	145852178	145854119	HERVHF_pol	+
Gorilla_gorilla_gorilla_chr3	145854706	145855158	HERVHF_3LTR	+
Gorilla_gorilla_gorilla_chr3	150041098	150041530	HERVHF_5LTR	+
Gorilla_gorilla_gorilla_chr3	150043054	150043578	HERVHF_gag	+
Gorilla_gorilla_gorilla_chr3	150044026	150044910	HERVHF_pro	+
Gorilla_gorilla_gorilla_chr3	150044314	150046435	HERVHF_pol	+
Gorilla_gorilla_gorilla_chr3	150046823	150047244	HERVHF_3LTR	+
Gorilla_gorilla_gorilla_chr3	152714060	152714476	HERVHF_5LTR	-
Gorilla_gorilla_gorilla_chr3	152715562	152717779	HERVHF_pol	-
Gorilla_gorilla_gorilla_chr3	152717237	152718016	HERVHF_pro	-
Gorilla_gorilla_gorilla_chr3	152718069	152718647	HERVHF_gag	-
Gorilla_gorilla_gorilla_chr3	152720129	152720538	HERVHF_3LTR	-
Gorilla_gorilla_gorilla_chr3	164816066	164816375	HERVHF_5LTR	+
Gorilla_gorilla_gorilla_chr3	164818131	164818442	HERVHF_gag	+

Gorilla_gorilla_gorilla_chr3	164818892	164820258	HERVHF_pol	+
Gorilla_gorilla_gorilla_chr3	164821492	164821798	HERVHF_3LTR	+
Gorilla_gorilla_gorilla_chr3	175062278	175062734	HERVHF_5LTR	-
Gorilla_gorilla_gorilla_chr3	175063190	175065361	HERVHF_pol	-
Gorilla_gorilla_gorilla_chr3	175064726	175065553	HERVHF_pro	-
Gorilla_gorilla_gorilla_chr3	175065580	175065879	HERVHF_gag	-
Gorilla_gorilla_gorilla_chr3	175067289	175067743	HERVHF_3LTR	-
Gorilla_gorilla_gorilla_chr3	175602405	175602825	HERVHF_5LTR	+
Gorilla_gorilla_gorilla_chr3	175604720	175605061	HERVHF_gag	+
Gorilla_gorilla_gorilla_chr3	175605133	175605909	HERVHF_pro	+
Gorilla_gorilla_gorilla_chr3	175605355	175607358	HERVHF_pol	+
Gorilla_gorilla_gorilla_chr3	175608196	175609061	HERVHF_env	+
Gorilla_gorilla_gorilla_chr3	175609285	175609704	HERVHF_3LTR	+
Gorilla_gorilla_gorilla_chr3	176903649	176904060	HERVHF_5LTR	+
Gorilla_gorilla_gorilla_chr3	176905915	176906628	HERVHF_pro	+
Gorilla_gorilla_gorilla_chr3	176906204	176907495	HERVHF_pol	+
Gorilla_gorilla_gorilla_chr3	176908661	176909073	HERVHF_3LTR	+
Gorilla_gorilla_gorilla_chr3	184046467	184046867	HERVHF_5LTR	+
Gorilla_gorilla_gorilla_chr3	184048512	184049072	HERVHF_gag	+
Gorilla_gorilla_gorilla_chr3	184049480	184051199	HERVHF_pol	+
Gorilla_gorilla_gorilla_chr3	184051999	184052405	HERVHF_3LTR	+
Gorilla_gorilla_gorilla_chr3	190436813	190437227	HERVHF_5LTR	+
Gorilla_gorilla_gorilla_chr3	190438652	190439206	HERVHF_gag	+
Gorilla_gorilla_gorilla_chr3	190439681	190441604	HERVHF_pol	+
Gorilla_gorilla_gorilla_chr3	190442053	190442463	HERVHF_3LTR	+
Gorilla_gorilla_gorilla_chr4	8486734	8487186	HERVHF_5LTR	-
Gorilla_gorilla_gorilla_chr4	8488422	8489849	HERVHF_pol	-
Gorilla_gorilla_gorilla_chr4	8489828	8490364	HERVHF_pro	-
Gorilla_gorilla_gorilla_chr4	8490377	8490694	HERVHF_gag	-
Gorilla_gorilla_gorilla_chr4	8492546	8492997	HERVHF_3LTR	-
Gorilla_gorilla_gorilla_chr4	11504681	11505032	HERVHF_5LTR	+
Gorilla_gorilla_gorilla_chr4	11506587	11506982	HERVHF_gag	+
Gorilla_gorilla_gorilla_chr4	11507032	11507865	HERVHF_pro	+
Gorilla_gorilla_gorilla_chr4	11507371	11509447	HERVHF_pol	+
Gorilla_gorilla_gorilla_chr4	11509919	11510262	HERVHF_3LTR	+
Gorilla_gorilla_gorilla_chr4	12058174	12058561	HERVHF_5LTR	+
Gorilla_gorilla_gorilla_chr4	12060188	12060499	HERVHF_gag	+
Gorilla_gorilla_gorilla_chr4	12060536	12061387	HERVHF_pro	+
Gorilla_gorilla_gorilla_chr4	12060728	12062976	HERVHF_pol	+
Gorilla_gorilla_gorilla_chr4	12063588	12063959	HERVHF_3LTR	+
Gorilla_gorilla_gorilla_chr4	13868398	13868849	HERVHF_5LTR	-
Gorilla_gorilla_gorilla_chr4	13869807	13871801	HERVHF_pol	-
Gorilla_gorilla_gorilla_chr4	13871803	13872543	HERVHF_pro	-
Gorilla_gorilla_gorilla_chr4	13872328	13872627	HERVHF_gag	-

Gorilla_gorilla_gorilla_chr4	13873952	13874403	HERVHF_3LTR	-
Gorilla_gorilla_gorilla_chr4	23378988	23379439	HERVHF_5LTR	-
Gorilla_gorilla_gorilla_chr4	23379773	23380966	HERVHF_pol	-
Gorilla_gorilla_gorilla_chr4	23380541	23381332	HERVHF_pro	-
Gorilla_gorilla_gorilla_chr4	23381395	23381724	HERVHF_gag	-
Gorilla_gorilla_gorilla_chr4	23383590	23384040	HERVHF_3LTR	-
Gorilla_gorilla_gorilla_chr4	24155929	24156382	HERVHF_5LTR	+
Gorilla_gorilla_gorilla_chr4	24157955	24158269	HERVHF_gag	+
Gorilla_gorilla_gorilla_chr4	24158691	24160698	HERVHF_pol	+
Gorilla_gorilla_gorilla_chr4	24161182	24161634	HERVHF_3LTR	+
Gorilla_gorilla_gorilla_chr4	49774836	49775204	HERVHF_5LTR	-
Gorilla_gorilla_gorilla_chr4	49775354	49775719	HERVHF_env	-
Gorilla_gorilla_gorilla_chr4	49777300	49779121	HERVHF_pol	-
Gorilla_gorilla_gorilla_chr4	49778791	49779267	HERVHF_pro	-
Gorilla_gorilla_gorilla_chr4	49779323	49779649	HERVHF_gag	-
Gorilla_gorilla_gorilla_chr4	49781102	49781474	HERVHF_3LTR	-
Gorilla_gorilla_gorilla_chr4	51038544	51038962	HERVHF_5LTR	+
Gorilla_gorilla_gorilla_chr4	51040735	51041196	HERVHF_pro	+
Gorilla_gorilla_gorilla_chr4	51041214	51042699	HERVHF_pol	+
Gorilla_gorilla_gorilla_chr4	51042902	51043309	HERVHF_3LTR	+
Gorilla_gorilla_gorilla_chr4	55993188	55993643	HERVHF_5LTR	+
Gorilla_gorilla_gorilla_chr4	55995536	55995919	HERVHF_gag	+
Gorilla_gorilla_gorilla_chr4	55995996	55996526	HERVHF_pro	+
Gorilla_gorilla_gorilla_chr4	55996355	55998380	HERVHF_pol	+
Gorilla_gorilla_gorilla_chr4	55998778	55999232	HERVHF_3LTR	+
Gorilla_gorilla_gorilla_chr4	66028792	66029120	HERVK_5LTR	-
Gorilla_gorilla_gorilla_chr4	66031753	66032899	HERVK_pol	-
Gorilla_gorilla_gorilla_chr4	66032534	66033754	HERVK_pro	-
Gorilla_gorilla_gorilla_chr4	66038684	66039011	HERVK_3LTR	-
Gorilla_gorilla_gorilla_chr4	75981822	75982275	HERVHF_5LTR	-
Gorilla_gorilla_gorilla_chr4	75982687	75984675	HERVHF_pol	-
Gorilla_gorilla_gorilla_chr4	75984268	75984900	HERVHF_pro	-
Gorilla_gorilla_gorilla_chr4	75985103	75985432	HERVHF_gag	-
Gorilla_gorilla_gorilla_chr4	75987316	75987767	HERVHF_3LTR	-
Gorilla_gorilla_gorilla_chr4	76818745	76819145	HERVHF_5LTR	-
Gorilla_gorilla_gorilla_chr4	76819547	76821666	HERVHF_pol	-
Gorilla_gorilla_gorilla_chr4	76821124	76821957	HERVHF_pro	-
Gorilla_gorilla_gorilla_chr4	76821991	76822374	HERVHF_gag	-
Gorilla_gorilla_gorilla_chr4	76824043	76824442	HERVHF_3LTR	-
Gorilla_gorilla_gorilla_chr4	85226656	85227121	HERVHF_5LTR	+
Gorilla_gorilla_gorilla_chr4	85229203	85230030	HERVHF_pro	+
Gorilla_gorilla_gorilla_chr4	85229488	85231617	HERVHF_pol	+
Gorilla_gorilla_gorilla_chr4	85232023	85232479	HERVHF_3LTR	+
Gorilla_gorilla_gorilla_chr4	87272243	87272653	HERVHF_5LTR	-

Gorilla_gorilla_gorilla_chr4	87273102	87275216	HERVHF_pol	-
Gorilla_gorilla_gorilla_chr4	87275538	87275918	HERVHF_gag	-
Gorilla_gorilla_gorilla_chr4	87277523	87277927	HERVHF_3LTR	-
Gorilla_gorilla_gorilla_chr4	88336675	88337037	HERVHF_5LTR	-
Gorilla_gorilla_gorilla_chr4	88337602	88340008	HERVHF_pol	-
Gorilla_gorilla_gorilla_chr4	88339995	88340561	HERVHF_pro	-
Gorilla_gorilla_gorilla_chr4	88342504	88342862	HERVHF_3LTR	-
Gorilla_gorilla_gorilla_chr4	88916911	88917361	HERVHF_5LTR	+
Gorilla_gorilla_gorilla_chr4	88919419	88919742	HERVHF_gag	+
Gorilla_gorilla_gorilla_chr4	88919753	88920565	HERVHF_pro	+
Gorilla_gorilla_gorilla_chr4	88920026	88922104	HERVHF_pol	+
Gorilla_gorilla_gorilla_chr4	88922424	88922878	HERVHF_3LTR	+
Gorilla_gorilla_gorilla_chr4	91773362	91773765	HERVHF_5LTR	-
Gorilla_gorilla_gorilla_chr4	91774633	91776919	HERVHF_pol	-
Gorilla_gorilla_gorilla_chr4	91776338	91777171	HERVHF_pro	-
Gorilla_gorilla_gorilla_chr4	91777175	91777504	HERVHF_gag	-
Gorilla_gorilla_gorilla_chr4	91779378	91779792	HERVHF_3LTR	-
Gorilla_gorilla_gorilla_chr4	101386317	101386732	HERVHF_5LTR	+
Gorilla_gorilla_gorilla_chr4	101388237	101388641	HERVHF_gag	+
Gorilla_gorilla_gorilla_chr4	101388879	101390482	HERVHF_pol	+
Gorilla_gorilla_gorilla_chr4	101391698	101392113	HERVHF_3LTR	+
Gorilla_gorilla_gorilla_chr4	117132923	117133236	HERVHF_5LTR	+
Gorilla_gorilla_gorilla_chr4	117135285	117135605	HERVHF_gag	+
Gorilla_gorilla_gorilla_chr4	117135728	117136441	HERVHF_pro	+
Gorilla_gorilla_gorilla_chr4	117135803	117138080	HERVHF_pol	+
Gorilla_gorilla_gorilla_chr4	117138629	117138939	HERVHF_3LTR	+
Gorilla_gorilla_gorilla_chr4	122739193	122739646	HERVHF_5LTR	-
Gorilla_gorilla_gorilla_chr4	122740234	122742174	HERVHF_pol	-
Gorilla_gorilla_gorilla_chr4	122741632	122742381	HERVHF_pro	-
Gorilla_gorilla_gorilla_chr4	122744620	122745071	HERVHF_3LTR	-
Gorilla_gorilla_gorilla_chr4	125904738	125905152	HERVHF_5LTR	-
Gorilla_gorilla_gorilla_chr4	125905552	125907674	HERVHF_pol	-
Gorilla_gorilla_gorilla_chr4	125907078	125907806	HERVHF_pro	-
Gorilla_gorilla_gorilla_chr4	125907953	125908380	HERVHF_gag	-
Gorilla_gorilla_gorilla_chr4	125910074	125910483	HERVHF_3LTR	-
Gorilla_gorilla_gorilla_chr4	131101772	131102177	HERVHF_5LTR	+
Gorilla_gorilla_gorilla_chr4	131103900	131104217	HERVHF_gag	+
Gorilla_gorilla_gorilla_chr4	131104269	131105054	HERVHF_pro	+
Gorilla_gorilla_gorilla_chr4	131104425	131107040	HERVHF_pol	+
Gorilla_gorilla_gorilla_chr4	131107547	131107949	HERVHF_3LTR	+
Gorilla_gorilla_gorilla_chr4	149542802	149543256	HERVHF_5LTR	-
Gorilla_gorilla_gorilla_chr4	149543684	149545716	HERVHF_pol	-
Gorilla_gorilla_gorilla_chr4	149548033	149548503	HERVHF_3LTR	-
Gorilla_gorilla_gorilla_chr4	153425345	153425785	HERVHF_5LTR	+

Gorilla_gorilla_gorilla_chr4	153427596	153427978	HERVHF_gag	+
Gorilla_gorilla_gorilla_chr4	153428486	153430428	HERVHF_pol	+
Gorilla_gorilla_gorilla_chr4	153430835	153431284	HERVHF_3LTR	+
Gorilla_gorilla_gorilla_chr4	163406241	163406701	HERVHF_5LTR	+
Gorilla_gorilla_gorilla_chr4	163408637	163408960	HERVHF_gag	+
Gorilla_gorilla_gorilla_chr4	163408994	163409374	HERVHF_pro	+
Gorilla_gorilla_gorilla_chr4	163409323	163411378	HERVHF_pol	+
Gorilla_gorilla_gorilla_chr4	163411785	163412237	HERVHF_3LTR	+
Gorilla_gorilla_gorilla_chr4	164578401	164578798	HERVHF_5LTR	+
Gorilla_gorilla_gorilla_chr4	164580470	164580790	HERVHF_gag	+
Gorilla_gorilla_gorilla_chr4	164580842	164581408	HERVHF_pro	+
Gorilla_gorilla_gorilla_chr4	164581499	164582462	HERVHF_pol	+
Gorilla_gorilla_gorilla_chr4	164583689	164584087	HERVHF_3LTR	+
Gorilla_gorilla_gorilla_chr4	164626432	164626901	HERVHF_5LTR	+
Gorilla_gorilla_gorilla_chr4	164628399	164628914	HERVHF_gag	+
Gorilla_gorilla_gorilla_chr4	164629237	164631197	HERVHF_pol	+
Gorilla_gorilla_gorilla_chr4	164631784	164632252	HERVHF_3LTR	+
Gorilla_gorilla_gorilla_chr4	166825995	166826409	HERVHF_5LTR	+
Gorilla_gorilla_gorilla_chr4	166828862	166830810	HERVHF_pol	+
Gorilla_gorilla_gorilla_chr4	166831205	166831622	HERVHF_3LTR	+
Gorilla_gorilla_gorilla_chr4	169738947	169739266	HERVHF_5LTR	-
Gorilla_gorilla_gorilla_chr4	169740311	169742575	HERVHF_pol	-
Gorilla_gorilla_gorilla_chr4	169742075	169742575	HERVHF_pro	-
Gorilla_gorilla_gorilla_chr4	169742830	169743171	HERVHF_gag	-
Gorilla_gorilla_gorilla_chr4	169744970	169745270	HERVHF_3LTR	-
Gorilla_gorilla_gorilla_chr4	179640580	179641013	HERVHF_5LTR	+
Gorilla_gorilla_gorilla_chr4	179643253	179643582	HERVHF_gag	+
Gorilla_gorilla_gorilla_chr4	179643589	179644365	HERVHF_pro	+
Gorilla_gorilla_gorilla_chr4	179643742	179644500	HERVHF_pol	+
Gorilla_gorilla_gorilla_chr4	179645038	179645470	HERVHF_3LTR	+
Gorilla_gorilla_gorilla_chr4	182874643	182874946	HERVHF_5LTR	-
Gorilla_gorilla_gorilla_chr4	182875397	182877599	HERVHF_pol	-
Gorilla_gorilla_gorilla_chr4	182877952	182878293	HERVHF_gag	-
Gorilla_gorilla_gorilla_chr4	182879762	182880073	HERVHF_3LTR	-
Gorilla_gorilla_gorilla_chr4	186263168	186263618	HERVHF_5LTR	-
Gorilla_gorilla_gorilla_chr4	186264281	186266951	HERVHF_pol	-
Gorilla_gorilla_gorilla_chr4	186267441	186267944	HERVHF_gag	-
Gorilla_gorilla_gorilla_chr4	186269657	186270120	HERVHF_3LTR	-
Gorilla_gorilla_gorilla_chr4	186633813	186634215	HERVHF_5LTR	+
Gorilla_gorilla_gorilla_chr4	186636277	186637101	HERVHF_pro	+
Gorilla_gorilla_gorilla_chr4	186636436	186638922	HERVHF_pol	+
Gorilla_gorilla_gorilla_chr4	186639375	186639777	HERVHF_3LTR	+
Gorilla_gorilla_gorilla_chr5	48681701	48682134	HERVHF_5LTR	-
Gorilla_gorilla_gorilla_chr5	48683369	48684767	HERVHF_pol	-

Gorilla_gorilla_gorilla_chr5	48684153	48684962	HERVHF_pro	-
Gorilla_gorilla_gorilla_chr5	48684965	48685294	HERVHF_gag	-
Gorilla_gorilla_gorilla_chr5	48687089	48687522	HERVHF_3LTR	-
Gorilla_gorilla_gorilla_chr5	62640482	62640791	HERVHF_5LTR	+
Gorilla_gorilla_gorilla_chr5	62642537	62642842	HERVHF_gag	+
Gorilla_gorilla_gorilla_chr5	62643088	62643678	HERVHF_pro	+
Gorilla_gorilla_gorilla_chr5	62643097	62644698	HERVHF_pol	+
Gorilla_gorilla_gorilla_chr5	62646055	62646361	HERVHF_3LTR	+
Gorilla_gorilla_gorilla_chr5	81530683	81531035	HERVHF_5LTR	-
Gorilla_gorilla_gorilla_chr5	81531624	81533531	HERVHF_pol	-
Gorilla_gorilla_gorilla_chr5	81532968	81533840	HERVHF_pro	-
Gorilla_gorilla_gorilla_chr5	81536265	81536614	HERVHF_3LTR	-
Gorilla_gorilla_gorilla_chr5	84578414	84578826	HERVHF_5LTR	+
Gorilla_gorilla_gorilla_chr5	84580464	84580763	HERVHF_gag	+
Gorilla_gorilla_gorilla_chr5	84580841	84581590	HERVHF_pro	+
Gorilla_gorilla_gorilla_chr5	84581129	84583200	HERVHF_pol	+
Gorilla_gorilla_gorilla_chr5	84583214	84583581	HERVHF_env	+
Gorilla_gorilla_gorilla_chr5	84583595	84584005	HERVHF_3LTR	+
Gorilla_gorilla_gorilla_chr5	84608907	84609374	HERVHF_5LTR	-
Gorilla_gorilla_gorilla_chr5	84609766	84612429	HERVHF_pol	-
Gorilla_gorilla_gorilla_chr5	84611887	84612633	HERVHF_pro	-
Gorilla_gorilla_gorilla_chr5	84613036	84614077	HERVHF_gag	-
Gorilla_gorilla_gorilla_chr5	84615286	84615765	HERVHF_3LTR	-
Gorilla_gorilla_gorilla_chr5	87513941	87514250	HERVHF_5LTR	+
Gorilla_gorilla_gorilla_chr5	87516190	87516870	HERVHF_pro	+
Gorilla_gorilla_gorilla_chr5	87516448	87518497	HERVHF_pol	+
Gorilla_gorilla_gorilla_chr5	87518978	87519291	HERVHF_3LTR	+
Gorilla_gorilla_gorilla_chr5	89092939	89093395	HERVHF_5LTR	+
Gorilla_gorilla_gorilla_chr5	89094832	89095155	HERVHF_gag	+
Gorilla_gorilla_gorilla_chr5	89095278	89095724	HERVHF_pro	+
Gorilla_gorilla_gorilla_chr5	89095685	89097539	HERVHF_pol	+
Gorilla_gorilla_gorilla_chr5	89098028	89098460	HERVHF_3LTR	+
Gorilla_gorilla_gorilla_chr5	94238766	94239219	HERVK_5LTR	+
Gorilla_gorilla_gorilla_chr5	94239997	94240937	HERVK_pro	+
Gorilla_gorilla_gorilla_chr5	94240832	94241626	HERVK_pol	+
Gorilla_gorilla_gorilla_chr5	94242947	94243402	HERVK_3LTR	+
Gorilla_gorilla_gorilla_chr5	101253948	101254400	HERVHF_5LTR	-
Gorilla_gorilla_gorilla_chr5	101254851	101256893	HERVHF_pol	-
Gorilla_gorilla_gorilla_chr5	101256351	101257184	HERVHF_pro	-
Gorilla_gorilla_gorilla_chr5	101257188	101257511	HERVHF_gag	-
Gorilla_gorilla_gorilla_chr5	101259339	101259793	HERVHF_3LTR	-
Gorilla_gorilla_gorilla_chr5	104944699	104945042	HERVHF_5LTR	+
Gorilla_gorilla_gorilla_chr5	104947281	104948030	HERVHF_pro	+
Gorilla_gorilla_gorilla_chr5	104947392	104949567	HERVHF_pol	+

Gorilla_gorilla_gorilla_chr5	104950067	104950412	HERVHF_3LTR	+
Gorilla_gorilla_gorilla_chr5	116992967	116993429	HERVHF_5LTR	+
Gorilla_gorilla_gorilla_chr5	116994800	116995336	HERVHF_gag	+
Gorilla_gorilla_gorilla_chr5	116995410	116995949	HERVHF_pro	+
Gorilla_gorilla_gorilla_chr5	116995806	116997310	HERVHF_pol	+
Gorilla_gorilla_gorilla_chr5	116998186	116998638	HERVHF_3LTR	+
Gorilla_gorilla_gorilla_chr5	117232733	117233154	HERVHF_5LTR	-
Gorilla_gorilla_gorilla_chr5	117233607	117234314	HERVHF_pol	-
Gorilla_gorilla_gorilla_chr5	117234650	117234973	HERVHF_gag	-
Gorilla_gorilla_gorilla_chr5	117236673	117237095	HERVHF_3LTR	-
Gorilla_gorilla_gorilla_chr5	121537799	121538242	HSERVIII_5LTR	-
Gorilla_gorilla_gorilla_chr5	121538774	121539430	HSERVIII_pol	-
Gorilla_gorilla_gorilla_chr5	121541621	121542066	HSERVIII_3LTR	-
Gorilla_gorilla_gorilla_chr5	134135303	134135760	HERVHF_5LTR	-
Gorilla_gorilla_gorilla_chr5	134136286	134138157	HERVHF_pol	-
Gorilla_gorilla_gorilla_chr5	134140581	134141034	HERVHF_3LTR	-
Gorilla_gorilla_gorilla_chr6	16811240	16811650	HERVHF_5LTR	+
Gorilla_gorilla_gorilla_chr6	16813981	16815924	HERVHF_pol	+
Gorilla_gorilla_gorilla_chr6	16816496	16816905	HERVHF_3LTR	+
Gorilla_gorilla_gorilla_chr6	18687877	18688280	HERVHF_5LTR	-
Gorilla_gorilla_gorilla_chr6	18688731	18690781	HERVHF_pol	-
Gorilla_gorilla_gorilla_chr6	18693221	18693624	HERVHF_3LTR	-
Gorilla_gorilla_gorilla_chr6	29194077	29194436	HERVHF_5LTR	-
Gorilla_gorilla_gorilla_chr6	29194607	29195116	HERVHF_env	-
Gorilla_gorilla_gorilla_chr6	29195498	29197749	HERVHF_pol	-
Gorilla_gorilla_gorilla_chr6	29198168	29198956	HERVHF_pro	-
Gorilla_gorilla_gorilla_chr6	29199013	29199369	HERVHF_gag	-
Gorilla_gorilla_gorilla_chr6	29200990	29201345	HERVHF_3LTR	-
Gorilla_gorilla_gorilla_chr6	41025422	41025855	HERVHF_5LTR	-
Gorilla_gorilla_gorilla_chr6	41026016	41027200	HERVHF_env	-
Gorilla_gorilla_gorilla_chr6	41028043	41031062	HERVHF_pol	-
Gorilla_gorilla_gorilla_chr6	41031490	41032080	HERVHF_gag	-
Gorilla_gorilla_gorilla_chr6	41032343	41032776	HERVHF_3LTR	-
Gorilla_gorilla_gorilla_chr6	52128444	52128891	HERVHF_5LTR	+
Gorilla_gorilla_gorilla_chr6	52130533	52130859	HERVHF_gag	+
Gorilla_gorilla_gorilla_chr6	52131285	52133217	HERVHF_pol	+
Gorilla_gorilla_gorilla_chr6	52133847	52134298	HERVHF_3LTR	+
Gorilla_gorilla_gorilla_chr6	61837828	61838194	HERVHF_5LTR	+
Gorilla_gorilla_gorilla_chr6	61839566	61839895	HERVHF_gag	+
Gorilla_gorilla_gorilla_chr6	61840114	61840908	HERVHF_pro	+
Gorilla_gorilla_gorilla_chr6	61840177	61842343	HERVHF_pol	+
Gorilla_gorilla_gorilla_chr6	61843265	61843627	HERVHF_env	+
Gorilla_gorilla_gorilla_chr6	61843786	61844151	HERVHF_3LTR	+
Gorilla_gorilla_gorilla_chr6	65452004	65452440	HERVHF_5LTR	-

Gorilla_gorilla_gorilla_chr6	65452610	65453288	HERVHF_env	-
Gorilla_gorilla_gorilla_chr6	65453970	65455937	HERVHF_pol	-
Gorilla_gorilla_gorilla_chr6	65455353	65456186	HERVHF_pro	-
Gorilla_gorilla_gorilla_chr6	65457914	65458351	HERVHF_3LTR	-
Gorilla_gorilla_gorilla_chr6	78124554	78124967	HERVHF_5LTR	-
Gorilla_gorilla_gorilla_chr6	78126019	78127908	HERVHF_pol	-
Gorilla_gorilla_gorilla_chr6	78128367	78128678	HERVHF_gag	-
Gorilla_gorilla_gorilla_chr6	78130163	78130575	HERVHF_3LTR	-
Gorilla_gorilla_gorilla_chr6	78943687	78944015	HERVHF_5LTR	-
Gorilla_gorilla_gorilla_chr6	78952833	78954731	HERVHF_pol	-
Gorilla_gorilla_gorilla_chr6	78954168	78955022	HERVHF_pro	-
Gorilla_gorilla_gorilla_chr6	78957745	78958078	HERVHF_3LTR	-
Gorilla_gorilla_gorilla_chr6	88291679	88292134	HERVHF_5LTR	-
Gorilla_gorilla_gorilla_chr6	88292939	88294709	HERVHF_pol	-
Gorilla_gorilla_gorilla_chr6	88294953	88295282	HERVHF_gag	-
Gorilla_gorilla_gorilla_chr6	88296523	88296976	HERVHF_3LTR	-
Gorilla_gorilla_gorilla_chr6	91464034	91464495	HERVHF_5LTR	-
Gorilla_gorilla_gorilla_chr6	91465273	91467076	HERVHF_pol	-
Gorilla_gorilla_gorilla_chr6	91466438	91467190	HERVHF_pro	-
Gorilla_gorilla_gorilla_chr6	91467274	91467603	HERVHF_gag	-
Gorilla_gorilla_gorilla_chr6	91469335	91469800	HERVHF_3LTR	-
Gorilla_gorilla_gorilla_chr6	109945792	109946258	HERVHF_5LTR	-
Gorilla_gorilla_gorilla_chr6	109947115	109948801	HERVHF_pol	-
Gorilla_gorilla_gorilla_chr6	109948259	109949089	HERVHF_pro	-
Gorilla_gorilla_gorilla_chr6	109949154	109949507	HERVHF_gag	-
Gorilla_gorilla_gorilla_chr6	109951426	109951876	HERVHF_3LTR	-
Gorilla_gorilla_gorilla_chr6	110555472	110555930	HERVHF_5LTR	+
Gorilla_gorilla_gorilla_chr6	110558040	110558870	HERVHF_pro	+
Gorilla_gorilla_gorilla_chr6	110558235	110560394	HERVHF_pol	+
Gorilla_gorilla_gorilla_chr6	110560849	110561308	HERVHF_3LTR	+
Gorilla_gorilla_gorilla_chr6	119088595	119088997	HERVHF_5LTR	-
Gorilla_gorilla_gorilla_chr6	119089396	119091390	HERVHF_pol	-
Gorilla_gorilla_gorilla_chr6	119091097	119091555	HERVHF_pro	-
Gorilla_gorilla_gorilla_chr6	119091818	119092132	HERVHF_gag	-
Gorilla_gorilla_gorilla_chr6	119093867	119094270	HERVHF_3LTR	-
Gorilla_gorilla_gorilla_chr6	122356339	122356769	HERVHF_5LTR	+
Gorilla_gorilla_gorilla_chr6	122358841	122359590	HERVHF_pro	+
Gorilla_gorilla_gorilla_chr6	122359048	122361151	HERVHF_pol	+
Gorilla_gorilla_gorilla_chr6	122361560	122361990	HERVHF_3LTR	+
Gorilla_gorilla_gorilla_chr6	126825408	126825864	HERVHF_5LTR	+
Gorilla_gorilla_gorilla_chr6	126827875	126828651	HERVHF_pro	+
Gorilla_gorilla_gorilla_chr6	126828136	126830076	HERVHF_pol	+
Gorilla_gorilla_gorilla_chr6	126830661	126831105	HERVHF_3LTR	+
Gorilla_gorilla_gorilla_chr6	126862980	126863433	HERVHF_5LTR	+

Gorilla_gorilla_gorilla_chr6	126865142	126865441	HERVHF_gag	+
Gorilla_gorilla_gorilla_chr6	126865858	126867442	HERVHF_pol	+
Gorilla_gorilla_gorilla_chr6	126868223	126868677	HERVHF_3LTR	+
Gorilla_gorilla_gorilla_chr6	126929469	126929826	HERVHF_5LTR	+
Gorilla_gorilla_gorilla_chr6	126931275	126931715	HERVHF_gag	+
Gorilla_gorilla_gorilla_chr6	126932320	126934064	HERVHF_pol	+
Gorilla_gorilla_gorilla_chr6	126934655	126935020	HERVHF_3LTR	+
Gorilla_gorilla_gorilla_chr6	137492333	137492787	HERVHF_5LTR	+
Gorilla_gorilla_gorilla_chr6	137494828	137495373	HERVHF_pro	+
Gorilla_gorilla_gorilla_chr6	137495375	137497455	HERVHF_pol	+
Gorilla_gorilla_gorilla_chr6	137497849	137498318	HERVHF_3LTR	+
Gorilla_gorilla_gorilla_chr6	140387656	140388089	HERVHF_5LTR	+
Gorilla_gorilla_gorilla_chr6	140389933	140390256	HERVHF_gag	+
Gorilla_gorilla_gorilla_chr6	140390693	140392439	HERVHF_pol	+
Gorilla_gorilla_gorilla_chr6	140393018	140393429	HERVHF_3LTR	+
Gorilla_gorilla_gorilla_chr6	140569274	140569683	HERVHF_5LTR	-
Gorilla_gorilla_gorilla_chr6	140570266	140572070	HERVHF_pol	-
Gorilla_gorilla_gorilla_chr6	140571690	140572418	HERVHF_pro	-
Gorilla_gorilla_gorilla_chr6	140574538	140574949	HERVHF_3LTR	-
Gorilla_gorilla_gorilla_chr6	155751602	155752005	HERVHF_5LTR	-
Gorilla_gorilla_gorilla_chr6	155752780	155754399	HERVHF_pol	-
Gorilla_gorilla_gorilla_chr6	155754022	155754573	HERVHF_pro	-
Gorilla_gorilla_gorilla_chr6	155756778	155757183	HERVHF_3LTR	-
Gorilla_gorilla_gorilla_chr6	159780417	159780831	HERVHF_5LTR	-
Gorilla_gorilla_gorilla_chr6	159781403	159783289	HERVHF_pol	-
Gorilla_gorilla_gorilla_chr6	159786090	159786516	HERVHF_3LTR	-
Gorilla_gorilla_gorilla_chr7	6846895	6847325	HERVHF_5LTR	+
Gorilla_gorilla_gorilla_chr7	6849771	6851221	HERVHF_pol	+
Gorilla_gorilla_gorilla_chr7	6851670	6852094	HERVHF_3LTR	+
Gorilla_gorilla_gorilla_chr7	9336511	9336901	HERVHF_5LTR	-
Gorilla_gorilla_gorilla_chr7	9338129	9339441	HERVHF_pol	-
Gorilla_gorilla_gorilla_chr7	9339729	9340052	HERVHF_gag	-
Gorilla_gorilla_gorilla_chr7	9341859	9342250	HERVHF_3LTR	-
Gorilla_gorilla_gorilla_chr7	9435955	9436405	HERVHF_5LTR	-
Gorilla_gorilla_gorilla_chr7	9437282	9439596	HERVHF_pol	-
Gorilla_gorilla_gorilla_chr7	9439177	9439785	HERVHF_pro	-
Gorilla_gorilla_gorilla_chr7	9441953	9442406	HERVHF_3LTR	-
Gorilla_gorilla_gorilla_chr7	20144693	20145153	HERVHF_5LTR	+
Gorilla_gorilla_gorilla_chr7	20146585	20146935	HERVHF_gag	+
Gorilla_gorilla_gorilla_chr7	20147275	20147976	HERVHF_pro	+
Gorilla_gorilla_gorilla_chr7	20147314	20149377	HERVHF_pol	+
Gorilla_gorilla_gorilla_chr7	20150028	20150486	HERVHF_3LTR	+
Gorilla_gorilla_gorilla_chr7	26021328	26021675	HERVHF_5LTR	-
Gorilla_gorilla_gorilla_chr7	26022519	26024200	HERVHF_pol	-

Gorilla_gorilla_gorilla_chr7	26023661	26024461	HERVHF_pro	-
Gorilla_gorilla_gorilla_chr7	26024515	26024898	HERVHF_gag	-
Gorilla_gorilla_gorilla_chr7	26026434	26026765	HERVHF_3LTR	-
Gorilla_gorilla_gorilla_chr7	31479119	31479523	HERVHF_5LTR	-
Gorilla_gorilla_gorilla_chr7	31479973	31482095	HERVHF_pol	-
Gorilla_gorilla_gorilla_chr7	31482588	31482971	HERVHF_gag	-
Gorilla_gorilla_gorilla_chr7	31484211	31484620	HERVHF_3LTR	-
Gorilla_gorilla_gorilla_chr7	34350281	34350723	HERVHF_5LTR	-
Gorilla_gorilla_gorilla_chr7	34351128	34353186	HERVHF_pol	-
Gorilla_gorilla_gorilla_chr7	34355279	34355720	HERVHF_3LTR	-
Gorilla_gorilla_gorilla_chr7	89665060	89665509	HERVHF_5LTR	-
Gorilla_gorilla_gorilla_chr7	89666306	89667977	HERVHF_pol	-
Gorilla_gorilla_gorilla_chr7	89667477	89668307	HERVHF_pro	-
Gorilla_gorilla_gorilla_chr7	89668314	89668724	HERVHF_gag	-
Gorilla_gorilla_gorilla_chr7	89670547	89671002	HERVHF_3LTR	-
Gorilla_gorilla_gorilla_chr7	96224196	96224611	HERVHF_5LTR	+
Gorilla_gorilla_gorilla_chr7	96226477	96226896	HERVHF_gag	+
Gorilla_gorilla_gorilla_chr7	96227621	96228034	HERVHF_pol	+
Gorilla_gorilla_gorilla_chr7	96228216	96228611	HERVHF_env	+
Gorilla_gorilla_gorilla_chr7	96229314	96229728	HERVHF_3LTR	+
Gorilla_gorilla_gorilla_chr7	114150411	114150816	HERVHF_5LTR	-
Gorilla_gorilla_gorilla_chr7	114151846	114153760	HERVHF_pol	-
Gorilla_gorilla_gorilla_chr7	114153353	114154183	HERVHF_pro	-
Gorilla_gorilla_gorilla_chr7	114154279	114154731	HERVHF_gag	-
Gorilla_gorilla_gorilla_chr7	114156382	114156787	HERVHF_3LTR	-
Gorilla_gorilla_gorilla_chr7	119338059	119338537	HERVHF_5LTR	+
Gorilla_gorilla_gorilla_chr7	119340259	119340579	HERVHF_gag	+
Gorilla_gorilla_gorilla_chr7	119340631	119341416	HERVHF_pro	+
Gorilla_gorilla_gorilla_chr7	119340874	119342955	HERVHF_pol	+
Gorilla_gorilla_gorilla_chr7	119343388	119343864	HERVHF_3LTR	+
Gorilla_gorilla_gorilla_chr7	119345675	119346083	HERVHF_5LTR	+
Gorilla_gorilla_gorilla_chr7	119348279	119348959	HERVHF_pro	+
Gorilla_gorilla_gorilla_chr7	119348486	119351209	HERVHF_pol	+
Gorilla_gorilla_gorilla_chr7	119351803	119352213	HERVHF_3LTR	+
Gorilla_gorilla_gorilla_chr7	141697888	141698302	HERVHF_5LTR	-
Gorilla_gorilla_gorilla_chr7	141698537	141699273	HERVHF_env	-
Gorilla_gorilla_gorilla_chr7	141700165	141701376	HERVHF_pol	-
Gorilla_gorilla_gorilla_chr7	141703666	141704082	HERVHF_3LTR	-
Gorilla_gorilla_gorilla_chr8	35092140	35092573	HERVHF_5LTR	+
Gorilla_gorilla_gorilla_chr8	35094400	35094720	HERVHF_gag	+
Gorilla_gorilla_gorilla_chr8	35094713	35095555	HERVHF_pro	+
Gorilla_gorilla_gorilla_chr8	35094917	35096933	HERVHF_pol	+
Gorilla_gorilla_gorilla_chr8	35097539	35097973	HERVHF_3LTR	+
Gorilla_gorilla_gorilla_chr8	42882558	42882858	HERVHF_5LTR	-

Gorilla_gorilla_gorilla_chr8	42883448	42885383	HERVHF_pol	-
Gorilla_gorilla_gorilla_chr8	42884868	42885668	HERVHF_pro	-
Gorilla_gorilla_gorilla_chr8	42885717	42886088	HERVHF_gag	-
Gorilla_gorilla_gorilla_chr8	42887941	42888247	HERVHF_3LTR	-
Gorilla_gorilla_gorilla_chr8	45582327	45582744	HERVHF_5LTR	-
Gorilla_gorilla_gorilla_chr8	45583091	45585059	HERVHF_pol	-
Gorilla_gorilla_gorilla_chr8	45584535	45585482	HERVHF_pro	-
Gorilla_gorilla_gorilla_chr8	45585489	45585809	HERVHF_gag	-
Gorilla_gorilla_gorilla_chr8	45587806	45588209	HERVHF_3LTR	-
Gorilla_gorilla_gorilla_chr8	65759603	65760116	HERVK_5LTR	-
Gorilla_gorilla_gorilla_chr8	65761194	65762567	HERVK_pol	-
Gorilla_gorilla_gorilla_chr8	65762465	65763223	HERVK_pro	-
Gorilla_gorilla_gorilla_chr8	65763447	65764121	HERVK_gag	-
Gorilla_gorilla_gorilla_chr8	65764809	65765321	HERVK_3LTR	-
Gorilla_gorilla_gorilla_chr8	86128748	86129202	HERVHF_5LTR	-
Gorilla_gorilla_gorilla_chr8	86129612	86131695	HERVHF_pol	-
Gorilla_gorilla_gorilla_chr8	86134039	86134470	HERVHF_3LTR	-
Gorilla_gorilla_gorilla_chr8	95975821	95976177	HERVHF_5LTR	+
Gorilla_gorilla_gorilla_chr8	95977979	95978392	HERVHF_gag	+
Gorilla_gorilla_gorilla_chr8	95978401	95979228	HERVHF_pro	+
Gorilla_gorilla_gorilla_chr8	95978686	95979970	HERVHF_pol	+
Gorilla_gorilla_gorilla_chr8	95981297	95981659	HERVHF_3LTR	+
Gorilla_gorilla_gorilla_chr8	110323921	110324395	HERVHF_5LTR	+
Gorilla_gorilla_gorilla_chr8	110326158	110326481	HERVHF_gag	+
Gorilla_gorilla_gorilla_chr8	110326574	110327407	HERVHF_pro	+
Gorilla_gorilla_gorilla_chr8	110326865	110328999	HERVHF_pol	+
Gorilla_gorilla_gorilla_chr8	110329380	110329846	HERVHF_3LTR	+
Gorilla_gorilla_gorilla_chr8	124671168	124671634	HERVHF_5LTR	-
Gorilla_gorilla_gorilla_chr8	124672072	124674018	HERVHF_pol	-
Gorilla_gorilla_gorilla_chr8	124676509	124676969	HERVHF_3LTR	-
Gorilla_gorilla_gorilla_chr8	124963103	124963526	HERVHF_5LTR	+
Gorilla_gorilla_gorilla_chr8	124966025	124968184	HERVHF_pol	+
Gorilla_gorilla_gorilla_chr8	124968597	124969020	HERVHF_3LTR	+
Gorilla_gorilla_gorilla_chr8	128154923	128155375	HERVHF_5LTR	-
Gorilla_gorilla_gorilla_chr8	128155990	128157895	HERVHF_pol	-
Gorilla_gorilla_gorilla_chr8	128158191	128158514	HERVHF_gag	-
Gorilla_gorilla_gorilla_chr8	128160107	128160565	HERVHF_3LTR	-
Gorilla_gorilla_gorilla_chr9	9907563	9908017	HERVHF_5LTR	-
Gorilla_gorilla_gorilla_chr9	9908180	9908779	HERVHF_env	-
Gorilla_gorilla_gorilla_chr9	9910819	9912948	HERVHF_pol	-
Gorilla_gorilla_gorilla_chr9	9915474	9915922	HERVHF_3LTR	-
Gorilla_gorilla_gorilla_chr9	15747122	15747525	HERVHF_5LTR	-
Gorilla_gorilla_gorilla_chr9	15748113	15750548	HERVHF_pol	-
Gorilla_gorilla_gorilla_chr9	15752956	15753358	HERVHF_3LTR	-

Gorilla_gorilla_gorilla_chr9	18805294	18805629	HERVHF_5LTR	+
Gorilla_gorilla_gorilla_chr9	18807187	18807492	HERVHF_gag	+
Gorilla_gorilla_gorilla_chr9	18807754	18808359	HERVHF_pro	+
Gorilla_gorilla_gorilla_chr9	18807763	18810002	HERVHF_pol	+
Gorilla_gorilla_gorilla_chr9	18810531	18810871	HERVHF_3LTR	+
Gorilla_gorilla_gorilla_chr9	28522641	28523124	HERVHF_5LTR	-
Gorilla_gorilla_gorilla_chr9	28523574	28525531	HERVHF_pol	-
Gorilla_gorilla_gorilla_chr9	28525340	28525927	HERVHF_pro	-
Gorilla_gorilla_gorilla_chr9	28525990	28526370	HERVHF_gag	-
Gorilla_gorilla_gorilla_chr9	28528005	28528488	HERVHF_3LTR	-
Gorilla_gorilla_gorilla_chr9	39422466	39422812	HERVHF_5LTR	-
Gorilla_gorilla_gorilla_chr9	39423362	39425648	HERVHF_pol	-
Gorilla_gorilla_gorilla_chr9	39425085	39425867	HERVHF_pro	-
Gorilla_gorilla_gorilla_chr9	39427923	39428277	HERVHF_3LTR	-
Gorilla_gorilla_gorilla_chr9	47441470	47441853	HERVHF_5LTR	+
Gorilla_gorilla_gorilla_chr9	47443710	47444150	HERVHF_gag	+
Gorilla_gorilla_gorilla_chr9	47444638	47446525	HERVHF_pol	+
Gorilla_gorilla_gorilla_chr9	47447030	47447400	HERVHF_3LTR	+
Gorilla_gorilla_gorilla_chr9	48707230	48707643	HERVHF_5LTR	-
Gorilla_gorilla_gorilla_chr9	48707927	48708390	HERVHF_env	-
Gorilla_gorilla_gorilla_chr9	48709382	48710834	HERVHF_pol	-
Gorilla_gorilla_gorilla_chr9	48710271	48711011	HERVHF_pro	-
Gorilla_gorilla_gorilla_chr9	48713261	48713666	HERVHF_3LTR	-
Gorilla_gorilla_gorilla_chr9	53509145	53509489	HERVHF_5LTR	+
Gorilla_gorilla_gorilla_chr9	53511176	53511628	HERVHF_gag	+
Gorilla_gorilla_gorilla_chr9	53511734	53512567	HERVHF_pro	+
Gorilla_gorilla_gorilla_chr9	53512025	53514155	HERVHF_pol	+
Gorilla_gorilla_gorilla_chr9	53514564	53514909	HERVHF_3LTR	+
Gorilla_gorilla_gorilla_chr9	56075765	56076207	HERVHF_5LTR	-
Gorilla_gorilla_gorilla_chr9	56077347	56078003	HERVHF_pol	-
Gorilla_gorilla_gorilla_chr9	56077386	56078168	HERVHF_pro	-
Gorilla_gorilla_gorilla_chr9	56080195	56080639	HERVHF_3LTR	-
Gorilla_gorilla_gorilla_chr9	56265057	56265406	HERVHF_5LTR	+
Gorilla_gorilla_gorilla_chr9	56267488	56268234	HERVHF_pro	+
Gorilla_gorilla_gorilla_chr9	56267602	56269773	HERVHF_pol	+
Gorilla_gorilla_gorilla_chr9	56270332	56270676	HERVHF_3LTR	+
Gorilla_gorilla_gorilla_chr9	58886903	58887356	HERVHF_5LTR	+
Gorilla_gorilla_gorilla_chr9	58889333	58889653	HERVHF_gag	+
Gorilla_gorilla_gorilla_chr9	58889657	58890460	HERVHF_pro	+
Gorilla_gorilla_gorilla_chr9	58889948	58892793	HERVHF_pol	+
Gorilla_gorilla_gorilla_chr9	58894133	58894800	HERVHF_env	+
Gorilla_gorilla_gorilla_chr9	58895030	58895487	HERVHF_3LTR	+
Gorilla_gorilla_gorilla_chr9	60838099	60838551	HERVHF_5LTR	+
Gorilla_gorilla_gorilla_chr9	60840677	60841507	HERVHF_pro	+

Gorilla_gorilla_gorilla_chr9	60840965	60842834	HERVHF_pol	+
Gorilla_gorilla_gorilla_chr9	60843495	60843950	HERVHF_3LTR	+
Gorilla_gorilla_gorilla_chr9	67209654	67210165	HERVK_5LTR	-
Gorilla_gorilla_gorilla_chr9	67211091	67212158	HERVK_pol	-
Gorilla_gorilla_gorilla_chr9	67212100	67212986	HERVK_pro	-
Gorilla_gorilla_gorilla_chr9	67213876	67214391	HERVK_3LTR	-
Gorilla_gorilla_gorilla_chr9	68015197	68015612	HERVHF_5LTR	+
Gorilla_gorilla_gorilla_chr9	68017652	68018454	HERVHF_pro	+
Gorilla_gorilla_gorilla_chr9	68018044	68019927	HERVHF_pol	+
Gorilla_gorilla_gorilla_chr9	68020435	68020857	HERVHF_3LTR	+
Gorilla_gorilla_gorilla_chr9	73488052	73488478	HERVHF_5LTR	-
Gorilla_gorilla_gorilla_chr9	73489061	73491531	HERVHF_pol	-
Gorilla_gorilla_gorilla_chr9	73491010	73491822	HERVHF_pro	-
Gorilla_gorilla_gorilla_chr9	73491826	73492155	HERVHF_gag	-
Gorilla_gorilla_gorilla_chr9	73494024	73494453	HERVHF_3LTR	-
Gorilla_gorilla_gorilla_chr9	89278656	89279106	HERVHF_5LTR	-
Gorilla_gorilla_gorilla_chr9	89279505	89281644	HERVHF_pol	-
Gorilla_gorilla_gorilla_chr9	89281102	89281935	HERVHF_pro	-
Gorilla_gorilla_gorilla_chr9	89281939	89282352	HERVHF_gag	-
Gorilla_gorilla_gorilla_chr9	89284212	89284654	HERVHF_3LTR	-
Gorilla_gorilla_gorilla_chr9	95243427	95243879	HERVHF_5LTR	+
Gorilla_gorilla_gorilla_chr9	95245558	95246160	HERVHF_gag	+
Gorilla_gorilla_gorilla_chr9	95246352	95247086	HERVHF_pro	+
Gorilla_gorilla_gorilla_chr9	95246427	95248579	HERVHF_pol	+
Gorilla_gorilla_gorilla_chr9	95248766	95249577	HERVHF_env	+
Gorilla_gorilla_gorilla_chr9	95249741	95250204	HERVHF_3LTR	+
Gorilla_gorilla_gorilla_chr9	95841616	95842006	HERVHF_5LTR	-
Gorilla_gorilla_gorilla_chr9	95842349	95844534	HERVHF_pol	-
Gorilla_gorilla_gorilla_chr9	95846580	95846948	HERVHF_3LTR	-
Gorilla_gorilla_gorilla_chr9	99393015	99393415	HERVHF_5LTR	-
Gorilla_gorilla_gorilla_chr9	99394614	99396157	HERVHF_pol	-
Gorilla_gorilla_gorilla_chr9	99395597	99396196	HERVHF_pro	-
Gorilla_gorilla_gorilla_chr9	99396451	99396771	HERVHF_gag	-
Gorilla_gorilla_gorilla_chr9	99398577	99398970	HERVHF_3LTR	-
Gorilla_gorilla_gorilla_chrX	2802708	2803394	HERVHF_5LTR	+
Gorilla_gorilla_gorilla_chrX	2805560	2805859	HERVHF_gag	+
Gorilla_gorilla_gorilla_chrX	2806308	2808251	HERVHF_pol	+
Gorilla_gorilla_gorilla_chrX	2809139	2809835	HERVHF_3LTR	+
Gorilla_gorilla_gorilla_chrX	10841316	10841737	HERVHF_5LTR	+
Gorilla_gorilla_gorilla_chrX	10844022	10844471	HERVHF_pro	+
Gorilla_gorilla_gorilla_chrX	10844061	10844774	HERVHF_pol	+
Gorilla_gorilla_gorilla_chrX	10846580	10847003	HERVHF_3LTR	+
Gorilla_gorilla_gorilla_chrX	28429616	28430030	HERVHF_5LTR	-
Gorilla_gorilla_gorilla_chrX	28430613	28432803	HERVHF_pol	-

Gorilla_gorilla_gorilla_chrX	28432204	28432809	HERVHF_pro	-
Gorilla_gorilla_gorilla_chrX	28433030	28433359	HERVHF_gag	-
Gorilla_gorilla_gorilla_chrX	28435074	28435490	HERVHF_3LTR	-
Gorilla_gorilla_gorilla_chrX	58437860	58438193	HERVHF_5LTR	-
Gorilla_gorilla_gorilla_chrX	58438692	58440950	HERVHF_pol	-
Gorilla_gorilla_gorilla_chrX	58440651	58441409	HERVHF_pro	-
Gorilla_gorilla_gorilla_chrX	58443271	58443595	HERVHF_3LTR	-
Gorilla_gorilla_gorilla_chrX	58753854	58754213	HERVK_5LTR	-
Gorilla_gorilla_gorilla_chrX	58754997	58756487	HERVK_pol	-
Gorilla_gorilla_gorilla_chrX	58756356	58757207	HERVK_pro	-
Gorilla_gorilla_gorilla_chrX	58758757	58759110	HERVK_3LTR	-
Gorilla_gorilla_gorilla_chrX	63119094	63119435	HERVHF_5LTR	-
Gorilla_gorilla_gorilla_chrX	63120204	63122742	HERVHF_pol	-
Gorilla_gorilla_gorilla_chrX	63122314	63123099	HERVHF_pro	-
Gorilla_gorilla_gorilla_chrX	63123609	63123989	HERVHF_gag	-
Gorilla_gorilla_gorilla_chrX	63125455	63125801	HERVHF_3LTR	-
Gorilla_gorilla_gorilla_chrX	76046816	76047247	HERVHF_5LTR	+
Gorilla_gorilla_gorilla_chrX	76047961	76049347	HERVHF_gag	+
Gorilla_gorilla_gorilla_chrX	76049821	76052310	HERVHF_pol	+
Gorilla_gorilla_gorilla_chrX	76052327	76052707	HERVHF_env	+
Gorilla_gorilla_gorilla_chrX	76052934	76053365	HERVHF_3LTR	+
Gorilla_gorilla_gorilla_chrX	81105182	81105621	HERVHF_5LTR	+
Gorilla_gorilla_gorilla_chrX	81107753	81108076	HERVHF_gag	+
Gorilla_gorilla_gorilla_chrX	81108083	81108913	HERVHF_pro	+
Gorilla_gorilla_gorilla_chrX	81108371	81110492	HERVHF_pol	+
Gorilla_gorilla_gorilla_chrX	81110902	81111335	HERVHF_3LTR	+
Gorilla_gorilla_gorilla_chrX	85504827	85505260	HERVHF_5LTR	-
Gorilla_gorilla_gorilla_chrX	85505749	85507821	HERVHF_pol	-
Gorilla_gorilla_gorilla_chrX	85507279	85507917	HERVHF_pro	-
Gorilla_gorilla_gorilla_chrX	85510114	85510547	HERVHF_3LTR	-
Gorilla_gorilla_gorilla_chrX	85675818	85676251	HERVHF_5LTR	-
Gorilla_gorilla_gorilla_chrX	85676658	85678774	HERVHF_pol	-
Gorilla_gorilla_gorilla_chrX	85678268	85678981	HERVHF_pro	-
Gorilla_gorilla_gorilla_chrX	85681157	85681596	HERVHF_3LTR	-
Gorilla_gorilla_gorilla_chrX	88109517	88109970	HERVHF_5LTR	-
Gorilla_gorilla_gorilla_chrX	88110359	88112387	HERVHF_pol	-
Gorilla_gorilla_gorilla_chrX	88111974	88112702	HERVHF_pro	-
Gorilla_gorilla_gorilla_chrX	88115344	88115794	HERVHF_3LTR	-
Gorilla_gorilla_gorilla_chrX	102485662	102486104	HERVHF_5LTR	+
Gorilla_gorilla_gorilla_chrX	102487787	102488158	HERVHF_gag	+
Gorilla_gorilla_gorilla_chrX	102488646	102490589	HERVHF_pol	+
Gorilla_gorilla_gorilla_chrX	102491035	102491476	HERVHF_3LTR	+
Gorilla_gorilla_gorilla_chrX	103181782	103182107	HERVHF_5LTR	-
Gorilla_gorilla_gorilla_chrX	103182676	103183914	HERVHF_pol	-

Gorilla_gorilla_gorilla_chrX	103183372	103184205	HERVHF_pro	-
Gorilla_gorilla_gorilla_chrX	103184292	103184615	HERVHF_gag	-
Gorilla_gorilla_gorilla_chrX	103186162	103186494	HERVHF_3LTR	-
Gorilla_gorilla_gorilla_chrX	107821522	107821824	HERVHF_5LTR	+
Gorilla_gorilla_gorilla_chrX	107823648	107824061	HERVHF_gag	+
Gorilla_gorilla_gorilla_chrX	107824271	107824774	HERVHF_pro	+
Gorilla_gorilla_gorilla_chrX	107824364	107826069	HERVHF_pol	+
Gorilla_gorilla_gorilla_chrX	107827046	107827350	HERVHF_3LTR	+
Gorilla_gorilla_gorilla_chrX	107950558	107950953	HERVHF_5LTR	-
Gorilla_gorilla_gorilla_chrX	107951784	107953646	HERVHF_pol	-
Gorilla_gorilla_gorilla_chrX	107953011	107953859	HERVHF_pro	-
Gorilla_gorilla_gorilla_chrX	107953849	107954262	HERVHF_gag	-
Gorilla_gorilla_gorilla_chrX	107956083	107956459	HERVHF_3LTR	-
Gorilla_gorilla_gorilla_chrX	109673555	109673910	HERVHF_5LTR	+
Gorilla_gorilla_gorilla_chrX	109675065	109675643	HERVHF_pro	+
Gorilla_gorilla_gorilla_chrX	109675068	109677419	HERVHF_pol	+
Gorilla_gorilla_gorilla_chrX	109677990	109678360	HERVHF_3LTR	+
Gorilla_gorilla_gorilla_chrX	110022728	110023142	HERVHF_5LTR	-
Gorilla_gorilla_gorilla_chrX	110023377	110024166	HERVHF_env	-
Gorilla_gorilla_gorilla_chrX	110024998	110026374	HERVHF_pol	-
Gorilla_gorilla_gorilla_chrX	110025910	110026539	HERVHF_pro	-
Gorilla_gorilla_gorilla_chrX	110026622	110027083	HERVHF_gag	-
Gorilla_gorilla_gorilla_chrX	110028702	110029116	HERVHF_3LTR	-
Gorilla_gorilla_gorilla_chrX	115464505	115464956	HERVHF_5LTR	+
Gorilla_gorilla_gorilla_chrX	115466680	115467051	HERVHF_gag	+
Gorilla_gorilla_gorilla_chrX	115467427	115469318	HERVHF_pol	+
Gorilla_gorilla_gorilla_chrX	115469906	115470360	HERVHF_3LTR	+
Gorilla_gorilla_gorilla_chrX	120150015	120150447	HERVHF_5LTR	+
Gorilla_gorilla_gorilla_chrX	120152762	120153412	HERVHF_pro	+
Gorilla_gorilla_gorilla_chrX	120153005	120155139	HERVHF_pol	+
Gorilla_gorilla_gorilla_chrX	120155558	120155994	HERVHF_3LTR	+
Gorilla_gorilla_gorilla_chrX	138492261	138492603	HERVHF_5LTR	-
Gorilla_gorilla_gorilla_chrX	138495350	138497218	HERVHF_pol	-
Gorilla_gorilla_gorilla_chrX	138497980	138498492	HERVHF_gag	-
Gorilla_gorilla_gorilla_chrX	138499619	138499960	HERVHF_3LTR	-
Gorilla_gorilla_gorilla_chrX	142307645	142307963	HSERVIII_5LTR	+
Gorilla_gorilla_gorilla_chrX	142309896	142312265	HSERVIII_pol	+
Gorilla_gorilla_gorilla_chrX	142314007	142314327	HSERVIII_3LTR	+
Gorilla_gorilla_gorilla_chrX	142542405	142542836	HERVHF_5LTR	+
Gorilla_gorilla_gorilla_chrX	142545177	142545779	HERVHF_pro	+
Gorilla_gorilla_gorilla_chrX	142545351	142547294	HERVHF_pol	+
Gorilla_gorilla_gorilla_chrX	142547743	142548174	HERVHF_3LTR	+
Gorilla_gorilla_gorilla_chrX	142884484	142884913	HSERVIII_5LTR	-
Gorilla_gorilla_gorilla_chrX	142886002	142886837	HSERVIII_pol	-

Gorilla_gorilla_gorilla_chrX	142888936	142889370	HSERVIII_3LTR	-
Gorilla_gorilla_gorilla_chrX	146251396	146251837	HERVHF_5LTR	-
Gorilla_gorilla_gorilla_chrX	146252006	146252464	HERVHF_env	-
Gorilla_gorilla_gorilla_chrX	146253013	146255671	HERVHF_pol	-
Gorilla_gorilla_gorilla_chrX	146254916	146256052	HERVHF_pro	-
Gorilla_gorilla_gorilla_chrX	146256110	146256553	HERVHF_gag	-
Gorilla_gorilla_gorilla_chrX	146257883	146258326	HERVHF_3LTR	-
Homo_sapiens_1	5044808	5045144	HERVHF_5LTR	+
Homo_sapiens_1	5046910	5047209	HERVHF_gag	+
Homo_sapiens_1	5047327	5048160	HERVHF_pro	+
Homo_sapiens_1	5047618	5050462	HERVHF_pol	+
Homo_sapiens_1	5051308	5052420	HERVHF_env	+
Homo_sapiens_1	5052648	5053000	HERVHF_3LTR	+
Homo_sapiens_1	22997488	22997912	HERVHF_5LTR	-
Homo_sapiens_1	22998530	23001867	HERVHF_pol	-
Homo_sapiens_1	23002236	23002547	HERVHF_gag	-
Homo_sapiens_1	23003988	23004403	HERVHF_3LTR	-
Homo_sapiens_1	29794213	29794559	HSERVIII_5LTR	+
Homo_sapiens_1	29796614	29799221	HSERVIII_pol	+
Homo_sapiens_1	29799585	29799942	HSERVIII_3LTR	+
Homo_sapiens_1	43085533	43085862	HERVHF_5LTR	+
Homo_sapiens_1	43087974	43088405	HERVHF_pro	+
Homo_sapiens_1	43088444	43090183	HERVHF_pol	+
Homo_sapiens_1	43090771	43091095	HERVHF_3LTR	+
Homo_sapiens_1	45753339	45753696	HERVIPADP_5LTR	-
Homo_sapiens_1	45753845	45754408	HERVIPADP_env	-
Homo_sapiens_1	45755745	45759131	HERVIPADP_pol	-
Homo_sapiens_1	45759618	45760088	HERVIPADP_gag	-
Homo_sapiens_1	45762104	45762461	HERVIPADP_3LTR	-
Homo_sapiens_1	48208994	48209412	HERVHF_5LTR	-
Homo_sapiens_1	48210005	48211811	HERVHF_pol	-
Homo_sapiens_1	48211404	48211934	HERVHF_pro	-
Homo_sapiens_1	48212350	48212661	HERVHF_gag	-
Homo_sapiens_1	48214397	48214824	HERVHF_3LTR	-
Homo_sapiens_1	53424323	53424768	HERVHF_5LTR	+
Homo_sapiens_1	53426329	53426772	HERVHF_gag	+
Homo_sapiens_1	53426865	53427605	HERVHF_pro	+
Homo_sapiens_1	53427063	53429190	HERVHF_pol	+
Homo_sapiens_1	53429582	53430027	HERVHF_3LTR	+
Homo_sapiens_1	64349934	64350335	HERVHF_5LTR	-
Homo_sapiens_1	64350908	64352931	HERVHF_pol	-
Homo_sapiens_1	64352389	64353219	HERVHF_pro	-
Homo_sapiens_1	64353226	64353549	HERVHF_gag	-
Homo_sapiens_1	64355311	64355699	HERVHF_3LTR	-

Homo_sapiens_1	65643205	65643638	HERVHF_5LTR	-
Homo_sapiens_1	65644171	65644827	HERVHF_pol	-
Homo_sapiens_1	65647601	65648034	HERVHF_3LTR	-
Homo_sapiens_1	68386003	68386439	HERVHF_5LTR	+
Homo_sapiens_1	68388421	68388741	HERVHF_gag	+
Homo_sapiens_1	68389169	68391111	HERVHF_pol	+
Homo_sapiens_1	68391558	68391994	HERVHF_3LTR	+
Homo_sapiens_1	72980444	72980898	HERVHF_5LTR	-
Homo_sapiens_1	72981316	72982805	HERVHF_pol	-
Homo_sapiens_1	72982263	72983108	HERVHF_pro	-
Homo_sapiens_1	72983118	72983504	HERVHF_gag	-
Homo_sapiens_1	72985037	72985491	HERVHF_3LTR	-
Homo_sapiens_1	82354581	82354965	HERVHF_5LTR	-
Homo_sapiens_1	82355816	82357821	HERVHF_pol	-
Homo_sapiens_1	82357279	82358037	HERVHF_pro	-
Homo_sapiens_1	82360176	82360561	HERVHF_3LTR	-
Homo_sapiens_1	82955299	82955725	HERVHF_5LTR	+
Homo_sapiens_1	82957379	82957759	HERVHF_gag	+
Homo_sapiens_1	82957868	82958632	HERVHF_pro	+
Homo_sapiens_1	82958120	82960341	HERVHF_pol	+
Homo_sapiens_1	82961176	82961592	HERVHF_3LTR	+
Homo_sapiens_1	99509593	99509965	HERVHF_5LTR	+
Homo_sapiens_1	99511673	99512059	HERVHF_gag	+
Homo_sapiens_1	99512102	99512932	HERVHF_pro	+
Homo_sapiens_1	99512390	99514304	HERVHF_pol	+
Homo_sapiens_1	99515002	99515364	HERVHF_3LTR	+
Homo_sapiens_1	185948276	185948729	HERVHF_5LTR	-
Homo_sapiens_1	185949184	185951263	HERVHF_pol	-
Homo_sapiens_1	185950721	185951554	HERVHF_pro	-
Homo_sapiens_1	185951558	185951887	HERVHF_gag	-
Homo_sapiens_1	185953756	185954216	HERVHF_3LTR	-
Homo_sapiens_1	209448799	209449267	HERVHF_5LTR	+
Homo_sapiens_1	209451675	209453142	HERVHF_pol	+
Homo_sapiens_1	209453552	209454012	HERVHF_3LTR	+
Homo_sapiens_1	216008143	216008555	HERVHF_5LTR	+
Homo_sapiens_1	216010479	216011309	HERVHF_pro	+
Homo_sapiens_1	216010728	216012869	HERVHF_pol	+
Homo_sapiens_1	216013459	216013871	HERVHF_3LTR	+
Homo_sapiens_1	224840009	224840329	HERVHF_5LTR	-
Homo_sapiens_1	224841379	224843462	HERVHF_pol	-
Homo_sapiens_1	224842881	224843657	HERVHF_pro	-
Homo_sapiens_1	224845764	224846095	HERVHF_3LTR	-
Homo_sapiens_1	228942542	228942868	HERVHF_5LTR	+
Homo_sapiens_1	228948298	228948678	HERVHF_gag	+

Homo_sapiens_1	228948714	228949544	HERVHF_pro	+
Homo_sapiens_1	228948906	228950910	HERVHF_pol	+
Homo_sapiens_1	228953920	228954247	HERVHF_3LTR	+
Homo_sapiens_1	232118097	232118574	HERVHF_5LTR	+
Homo_sapiens_1	232120241	232120654	HERVHF_gag	+
Homo_sapiens_1	232120661	232121512	HERVHF_pro	+
Homo_sapiens_1	232120994	232122891	HERVHF_pol	+
Homo_sapiens_1	232123477	232123943	HERVHF_3LTR	+
Homo_sapiens_1	233683447	233683884	HERVHF_5LTR	-
Homo_sapiens_1	233684292	233686399	HERVHF_pol	-
Homo_sapiens_1	233685836	233686690	HERVHF_pro	-
Homo_sapiens_1	233686694	233687089	HERVHF_gag	-
Homo_sapiens_1	233688771	233689205	HERVHF_3LTR	-
Homo_sapiens_1	236932314	236932745	HERVHF_5LTR	-
Homo_sapiens_1	236933317	236935221	HERVHF_pol	-
Homo_sapiens_1	236937486	236937922	HERVHF_3LTR	-
Homo_sapiens_1	241433890	241434298	HERVHF_5LTR	+
Homo_sapiens_1	241436142	241436447	HERVHF_gag	+
Homo_sapiens_1	241436737	241439030	HERVHF_pol	+
Homo_sapiens_1	241439482	241439885	HERVHF_3LTR	+
Homo_sapiens_10	5470767	5471180	HERVHF_5LTR	+
Homo_sapiens_10	5473577	5474398	HERVHF_pro	+
Homo_sapiens_10	5473877	5476210	HERVHF_pol	+
Homo_sapiens_10	5476605	5477017	HERVHF_3LTR	+
Homo_sapiens_10	6629574	6629975	HERVHF_5LTR	-
Homo_sapiens_10	6630451	6632563	HERVHF_pol	-
Homo_sapiens_10	6632024	6632854	HERVHF_pro	-
Homo_sapiens_10	6632887	6633267	HERVHF_gag	-
Homo_sapiens_10	6635084	6635498	HERVHF_3LTR	-
Homo_sapiens_10	6797081	6797535	HERVHF_5LTR	-
Homo_sapiens_10	6798366	6799928	HERVHF_pol	-
Homo_sapiens_10	6800444	6800767	HERVHF_gag	-
Homo_sapiens_10	6802501	6802954	HERVHF_3LTR	-
Homo_sapiens_10	20119202	20119613	HERVHF_5LTR	-
Homo_sapiens_10	20119622	20119983	HERVHF_env	-
Homo_sapiens_10	20120056	20122028	HERVHF_pol	-
Homo_sapiens_10	20121588	20122169	HERVHF_pro	-
Homo_sapiens_10	20124529	20124935	HERVHF_3LTR	-
Homo_sapiens_10	25716420	25716834	HERVHF_5LTR	+
Homo_sapiens_10	25718477	25718863	HERVHF_gag	+
Homo_sapiens_10	25718959	25719759	HERVHF_pro	+
Homo_sapiens_10	25719121	25721515	HERVHF_pol	+
Homo_sapiens_10	25722171	25722503	HERVHF_env	+
Homo_sapiens_10	25722512	25722928	HERVHF_3LTR	+

Homo_sapiens_10	52033754	52034207	HERVHF_5LTR	-
Homo_sapiens_10	52035036	52036733	HERVHF_pol	-
Homo_sapiens_10	52036137	52036940	HERVHF_pro	-
Homo_sapiens_10	52039108	52039561	HERVHF_3LTR	-
Homo_sapiens_10	53492722	53493177	HERVHF_5LTR	-
Homo_sapiens_10	53496219	53502000	HERVHF_pol	-
Homo_sapiens_10	53501458	53502291	HERVHF_pro	-
Homo_sapiens_10	53502295	53502840	HERVHF_gag	-
Homo_sapiens_10	53504276	53504729	HERVHF_3LTR	-
Homo_sapiens_10	54166675	54167112	HERVHF_5LTR	+
Homo_sapiens_10	54168818	54169180	HERVHF_gag	+
Homo_sapiens_10	54169315	54170037	HERVHF_pro	+
Homo_sapiens_10	54169522	54170855	HERVHF_pol	+
Homo_sapiens_10	54172055	54172503	HERVHF_3LTR	+
Homo_sapiens_10	58860993	58861449	HERVHF_5LTR	-
Homo_sapiens_10	58861899	58863758	HERVHF_pol	-
Homo_sapiens_10	58863441	58864226	HERVHF_pro	-
Homo_sapiens_10	58864277	58864600	HERVHF_gag	-
Homo_sapiens_10	58866879	58867332	HERVHF_3LTR	-
Homo_sapiens_10	83971226	83971628	HSERVIII_5LTR	-
Homo_sapiens_10	83972540	83975072	HSERVIII_pol	-
Homo_sapiens_10	83977372	83977778	HSERVIII_3LTR	-
Homo_sapiens_10	90807289	90807739	HERVHF_5LTR	+
Homo_sapiens_10	90809488	90809808	HERVHF_gag	+
Homo_sapiens_10	90809949	90810713	HERVHF_pro	+
Homo_sapiens_10	90810192	90812217	HERVHF_pol	+
Homo_sapiens_10	90812810	90813260	HERVHF_3LTR	+
Homo_sapiens_10	96732986	96733309	HERVHF_5LTR	+
Homo_sapiens_10	96734365	96734688	HERVHF_gag	+
Homo_sapiens_10	96734725	96735498	HERVHF_pro	+
Homo_sapiens_10	96735169	96735654	HERVHF_pol	+
Homo_sapiens_10	96736169	96736493	HERVHF_3LTR	+
Homo_sapiens_11	5160295	5160710	HERVHF_5LTR	-
Homo_sapiens_11	5161282	5163250	HERVHF_pol	-
Homo_sapiens_11	5163544	5163885	HERVHF_gag	-
Homo_sapiens_11	5165704	5166118	HERVHF_3LTR	-
Homo_sapiens_11	6366039	6366440	HERVHF_5LTR	+
Homo_sapiens_11	6367846	6368226	HERVHF_gag	+
Homo_sapiens_11	6368676	6370836	HERVHF_pol	+
Homo_sapiens_11	6371260	6371662	HERVHF_3LTR	+
Homo_sapiens_11	20567867	20568314	HERVHF_5LTR	+
Homo_sapiens_11	20569935	20570264	HERVHF_gag	+
Homo_sapiens_11	20570594	20571798	HERVHF_pol	+
Homo_sapiens_11	20572198	20572650	HERVHF_3LTR	+

Homo_sapiens_11	23183932	23184385	HERVHF_5LTR	-
Homo_sapiens_11	23184617	23186805	HERVHF_pol	-
Homo_sapiens_11	23187199	23187528	HERVHF_gag	-
Homo_sapiens_11	23189291	23189744	HERVHF_3LTR	-
Homo_sapiens_11	23887274	23887605	HERVHF_5LTR	-
Homo_sapiens_11	23888260	23890252	HERVHF_pol	-
Homo_sapiens_11	23889644	23890357	HERVHF_pro	-
Homo_sapiens_11	23890442	23890987	HERVHF_gag	-
Homo_sapiens_11	23892534	23892864	HERVHF_3LTR	-
Homo_sapiens_11	27629072	27629528	HERVHF_5LTR	-
Homo_sapiens_11	27630027	27632049	HERVHF_pol	-
Homo_sapiens_11	27631507	27632340	HERVHF_pro	-
Homo_sapiens_11	27632416	27632889	HERVHF_gag	-
Homo_sapiens_11	27634477	27634938	HERVHF_3LTR	-
Homo_sapiens_11	94641661	94642011	HERVHF_5LTR	+
Homo_sapiens_11	94644136	94644915	HERVHF_pro	+
Homo_sapiens_11	94644373	94646313	HERVHF_pol	+
Homo_sapiens_11	94646973	94647315	HERVHF_3LTR	+
Homo_sapiens_11	95309405	95309899	HERVHF_5LTR	+
Homo_sapiens_11	95312049	95312846	HERVHF_pro	+
Homo_sapiens_11	95312283	95315121	HERVHF_pol	+
Homo_sapiens_11	95315995	95317167	HERVHF_env	+
Homo_sapiens_11	95317333	95317806	HERVHF_3LTR	+
Homo_sapiens_11	96499960	96500271	HERVHF_5LTR	+
Homo_sapiens_11	96501014	96501424	HERVHF_gag	+
Homo_sapiens_11	96501726	96502403	HERVHF_pro	+
Homo_sapiens_11	96501900	96504947	HERVHF_pol	+
Homo_sapiens_11	96506073	96506627	HERVHF_env	+
Homo_sapiens_11	96507611	96507918	HERVHF_3LTR	+
Homo_sapiens_11	96587635	96588088	HERVHF_5LTR	+
Homo_sapiens_11	96590439	96591233	HERVHF_pro	+
Homo_sapiens_11	96590598	96592771	HERVHF_pol	+
Homo_sapiens_11	96593224	96593677	HERVHF_3LTR	+
Homo_sapiens_11	121637355	121637761	HERVHF_5LTR	-
Homo_sapiens_11	121638255	121640933	HERVHF_pol	-
Homo_sapiens_11	121640352	121641194	HERVHF_pro	-
Homo_sapiens_11	121643108	121643516	HERVHF_3LTR	-
Homo_sapiens_11	130564387	130564827	HERVHF_5LTR	-
Homo_sapiens_11	130565609	130567330	HERVHF_pol	-
Homo_sapiens_11	130566809	130567627	HERVHF_pro	-
Homo_sapiens_11	130569681	130570121	HERVHF_3LTR	-
Homo_sapiens_11	130753494	130753946	HERVHF_5LTR	-
Homo_sapiens_11	130754355	130756582	HERVHF_pol	-
Homo_sapiens_11	130755941	130756702	HERVHF_pro	-

Homo_sapiens_11	130758681	130759137	HERVHF_3LTR	-
Homo_sapiens_12	4018623	4018993	HERVHF_5LTR	+
Homo_sapiens_12	4020593	4021006	HERVHF_gag	+
Homo_sapiens_12	4021003	4021665	HERVHF_pro	+
Homo_sapiens_12	4021511	4022836	HERVHF_pol	+
Homo_sapiens_12	4023325	4023691	HERVHF_3LTR	+
Homo_sapiens_12	11462168	11462625	HERVHF_5LTR	-
Homo_sapiens_12	11463071	11465137	HERVHF_pol	-
Homo_sapiens_12	11467569	11468022	HERVHF_3LTR	-
Homo_sapiens_12	14705418	14705902	HERVHF_5LTR	-
Homo_sapiens_12	14706356	14707614	HERVHF_pol	-
Homo_sapiens_12	14707324	14708130	HERVHF_pro	-
Homo_sapiens_12	14710153	14710640	HERVHF_3LTR	-
Homo_sapiens_12	17021894	17022331	HERVHF_5LTR	-
Homo_sapiens_12	17022907	17024592	HERVHF_pol	-
Homo_sapiens_12	17024305	17024766	HERVHF_pro	-
Homo_sapiens_12	17024994	17025293	HERVHF_gag	-
Homo_sapiens_12	17026933	17027366	HERVHF_3LTR	-
Homo_sapiens_12	25163213	25163629	HERVHF_5LTR	+
Homo_sapiens_12	25165679	25166455	HERVHF_pro	+
Homo_sapiens_12	25165913	25167195	HERVHF_pol	+
Homo_sapiens_12	25168018	25168876	HERVHF_env	+
Homo_sapiens_12	25169104	25169518	HERVHF_3LTR	+
Homo_sapiens_12	34265635	34266029	HERVHF_5LTR	+
Homo_sapiens_12	34267793	34268113	HERVHF_gag	+
Homo_sapiens_12	34268166	34268759	HERVHF_pro	+
Homo_sapiens_12	34269097	34271546	HERVHF_pol	+
Homo_sapiens_12	34272421	34273596	HERVHF_env	+
Homo_sapiens_12	34273840	34274242	HERVHF_3LTR	+
Homo_sapiens_12	47443338	47443848	HERVK_5LTR	-
Homo_sapiens_12	47443928	47444233	HERVK_env	-
Homo_sapiens_12	47445250	47446577	HERVK_pol	-
Homo_sapiens_12	47446574	47447479	HERVK_pro	-
Homo_sapiens_12	47447380	47448135	HERVK_gag	-
Homo_sapiens_12	47448256	47448766	HERVK_3LTR	-
Homo_sapiens_12	67766803	67767256	HERVHF_5LTR	-
Homo_sapiens_12	67767662	67769614	HERVHF_pol	-
Homo_sapiens_12	67769075	67769905	HERVHF_pro	-
Homo_sapiens_12	67769949	67770458	HERVHF_gag	-
Homo_sapiens_12	67771894	67772346	HERVHF_3LTR	-
Homo_sapiens_12	70444894	70445309	HERVK_5LTR	-
Homo_sapiens_12	70445322	70447607	HERVK_pol	-
Homo_sapiens_12	70448002	70448352	HERVK_pro	-
Homo_sapiens_12	70448376	70448912	HERVK_gag	-

Homo_sapiens_12	70449682	70450107	HERVK_3LTR	-
Homo_sapiens_12	72703213	72703674	HERVHF_5LTR	+
Homo_sapiens_12	72705496	72705825	HERVHF_gag	+
Homo_sapiens_12	72705877	72706662	HERVHF_pro	+
Homo_sapiens_12	72706120	72708178	HERVHF_pol	+
Homo_sapiens_12	72708522	72708968	HERVHF_3LTR	+
Homo_sapiens_12	80783118	80783525	HERVHF_5LTR	-
Homo_sapiens_12	80784110	80786049	HERVHF_pol	-
Homo_sapiens_12	80788486	80788889	HERVHF_3LTR	-
Homo_sapiens_12	86938732	86939134	HERVHF_5LTR	+
Homo_sapiens_12	86940952	86941275	HERVHF_gag	+
Homo_sapiens_12	86941530	86943723	HERVHF_pol	+
Homo_sapiens_12	86944355	86944748	HERVHF_3LTR	+
Homo_sapiens_12	87907283	87907629	HERVHF_5LTR	-
Homo_sapiens_12	87908125	87910418	HERVHF_pol	-
Homo_sapiens_12	87909852	87910454	HERVHF_pro	-
Homo_sapiens_12	87912483	87912826	HERVHF_3LTR	-
Homo_sapiens_12	99715184	99715598	HERVHF_5LTR	+
Homo_sapiens_12	99717322	99717633	HERVHF_gag	+
Homo_sapiens_12	99718271	99720738	HERVHF_pol	+
Homo_sapiens_12	99721328	99721742	HERVHF_3LTR	+
Homo_sapiens_13	36316404	36316772	HERVHF_5LTR	+
Homo_sapiens_13	36318589	36318918	HERVHF_gag	+
Homo_sapiens_13	36319082	36321288	HERVHF_pol	+
Homo_sapiens_13	36321823	36322191	HERVHF_3LTR	+
Homo_sapiens_13	42868001	42868452	HERVHF_5LTR	-
Homo_sapiens_13	42869058	42870339	HERVHF_pol	-
Homo_sapiens_13	42869842	42870594	HERVHF_pro	-
Homo_sapiens_13	42870678	42871007	HERVHF_gag	-
Homo_sapiens_13	42872701	42873158	HERVHF_3LTR	-
Homo_sapiens_13	48866771	48867093	HERVHF_5LTR	-
Homo_sapiens_13	48868555	48870047	HERVHF_pol	-
Homo_sapiens_13	48869643	48870272	HERVHF_pro	-
Homo_sapiens_13	48872132	48872457	HERVHF_3LTR	-
Homo_sapiens_13	51169866	51170315	HERVHF_5LTR	+
Homo_sapiens_13	51172014	51172343	HERVHF_gag	+
Homo_sapiens_13	51172529	51173197	HERVHF_pro	+
Homo_sapiens_13	51172592	51174102	HERVHF_pol	+
Homo_sapiens_13	51174556	51175008	HERVHF_3LTR	+
Homo_sapiens_13	54127417	54127817	HERVHF_5LTR	-
Homo_sapiens_13	54128407	54130751	HERVHF_pol	-
Homo_sapiens_13	54130113	54130865	HERVHF_pro	-
Homo_sapiens_13	54130954	54131292	HERVHF_gag	-
Homo_sapiens_13	54132759	54133159	HERVHF_3LTR	-

Homo_sapiens_13	61205681	61206085	HERVHF_5LTR	+
Homo_sapiens_13	61207480	61208130	HERVHF_pro	+
Homo_sapiens_13	61207483	61210430	HERVHF_pol	+
Homo_sapiens_13	61210874	61211281	HERVHF_3LTR	+
Homo_sapiens_13	66141327	66141765	HERVHF_5LTR	-
Homo_sapiens_13	66142250	66144110	HERVHF_pol	-
Homo_sapiens_13	66144597	66144917	HERVHF_gag	-
Homo_sapiens_13	66146605	66147037	HERVHF_3LTR	-
Homo_sapiens_13	79274081	79274498	HERVHF_5LTR	+
Homo_sapiens_13	79276195	79276509	HERVHF_gag	+
Homo_sapiens_13	79276611	79277441	HERVHF_pro	+
Homo_sapiens_13	79276860	79278376	HERVHF_pol	+
Homo_sapiens_13	79279414	79279830	HERVHF_3LTR	+
Homo_sapiens_13	81866091	81866506	HERVHF_5LTR	+
Homo_sapiens_13	81868327	81868650	HERVHF_gag	+
Homo_sapiens_13	81868974	81871097	HERVHF_pol	+
Homo_sapiens_13	81872008	81872439	HERVHF_3LTR	+
Homo_sapiens_13	83908302	83908634	HERVHF_5LTR	-
Homo_sapiens_13	83909136	83911198	HERVHF_pol	-
Homo_sapiens_13	83910707	83911537	HERVHF_pro	-
Homo_sapiens_13	83911541	83911954	HERVHF_gag	-
Homo_sapiens_13	83913411	83913744	HERVHF_3LTR	-
Homo_sapiens_13	86358167	86358624	HERVHF_5LTR	+
Homo_sapiens_13	86361185	86363252	HERVHF_pol	+
Homo_sapiens_13	86363686	86364142	HERVHF_3LTR	+
Homo_sapiens_13	87186451	87186859	HERVHF_5LTR	-
Homo_sapiens_13	87187274	87189334	HERVHF_pol	-
Homo_sapiens_13	87189687	87190013	HERVHF_gag	-
Homo_sapiens_13	87191714	87192109	HERVHF_3LTR	-
Homo_sapiens_14	38190635	38191085	HERVHF_5LTR	+
Homo_sapiens_14	38193319	38194104	HERVHF_pro	+
Homo_sapiens_14	38193562	38195486	HERVHF_pol	+
Homo_sapiens_14	38196077	38196529	HERVHF_3LTR	+
Homo_sapiens_14	41515866	41516322	HERVHF_5LTR	+
Homo_sapiens_14	41518090	41519277	HERVHF_pro	+
Homo_sapiens_14	41518714	41520810	HERVHF_pol	+
Homo_sapiens_14	41521426	41521883	HERVHF_3LTR	+
Homo_sapiens_14	46544005	46544467	HERVHF_5LTR	+
Homo_sapiens_14	46546394	46547224	HERVHF_pro	+
Homo_sapiens_14	46546685	46548638	HERVHF_pol	+
Homo_sapiens_14	46549199	46549670	HERVHF_3LTR	+
Homo_sapiens_14	48255191	48255959	HERVHF_5LTR	-
Homo_sapiens_14	48256057	48258390	HERVHF_pol	-
Homo_sapiens_14	48257788	48258639	HERVHF_pro	-

Homo_sapiens_14	48262389	48263146	HERVHF_3LTR	-
Homo_sapiens_14	61133252	61133644	HERVHF_5LTR	-
Homo_sapiens_14	61134097	61136254	HERVHF_pol	-
Homo_sapiens_14	61136685	61137125	HERVHF_gag	-
Homo_sapiens_14	61138770	61139158	HERVHF_3LTR	-
Homo_sapiens_14	73703338	73703770	HERVHF_5LTR	+
Homo_sapiens_14	73705638	73705967	HERVHF_gag	+
Homo_sapiens_14	73705971	73706774	HERVHF_pro	+
Homo_sapiens_14	73706169	73708378	HERVHF_pol	+
Homo_sapiens_14	73708783	73709215	HERVHF_3LTR	+
Homo_sapiens_14	84500245	84500672	HERVHF_5LTR	-
Homo_sapiens_14	84501242	84503182	HERVHF_pol	-
Homo_sapiens_14	84503495	84503878	HERVHF_gag	-
Homo_sapiens_14	84505560	84505980	HERVHF_3LTR	-
Homo_sapiens_14	85732429	85732842	HERVHF_5LTR	-
Homo_sapiens_14	85733748	85735805	HERVHF_pol	-
Homo_sapiens_14	85736263	85736574	HERVHF_gag	-
Homo_sapiens_14	85738185	85738601	HERVHF_3LTR	-
Homo_sapiens_14	98985205	98985687	HERVHF_5LTR	+
Homo_sapiens_14	98987259	98987642	HERVHF_gag	+
Homo_sapiens_14	98987678	98988508	HERVHF_pro	+
Homo_sapiens_14	98987966	98990051	HERVHF_pol	+
Homo_sapiens_14	98991701	98992300	HERVHF_env	+
Homo_sapiens_14	98992460	98992942	HERVHF_3LTR	+
Homo_sapiens_14	101305252	101305655	HERVHF_5LTR	-
Homo_sapiens_14	101306514	101308678	HERVHF_pol	-
Homo_sapiens_14	101308139	101308915	HERVHF_pro	-
Homo_sapiens_14	101310847	101311248	HERVHF_3LTR	-
Homo_sapiens_15	35238617	35239062	HERVHF_5LTR	+
Homo_sapiens_15	35240917	35241246	HERVHF_gag	+
Homo_sapiens_15	35241328	35242059	HERVHF_pro	+
Homo_sapiens_15	35241445	35242812	HERVHF_pol	+
Homo_sapiens_15	35243404	35243837	HERVHF_3LTR	+
Homo_sapiens_15	49698516	49698929	HERVHF_5LTR	-
Homo_sapiens_15	49699097	49699860	HERVHF_env	-
Homo_sapiens_15	49700775	49702106	HERVHF_pol	-
Homo_sapiens_15	49704343	49704756	HERVHF_3LTR	-
Homo_sapiens_15	53426844	53427278	HERVHF_5LTR	-
Homo_sapiens_15	53427887	53429671	HERVHF_pol	-
Homo_sapiens_15	53432030	53432463	HERVHF_3LTR	-
Homo_sapiens_15	74354141	74354539	HERVHF_5LTR	+
Homo_sapiens_15	74355965	74356405	HERVHF_gag	+
Homo_sapiens_15	74356488	74357237	HERVHF_pro	+
Homo_sapiens_15	74356599	74358842	HERVHF_pol	+

Homo_sapiens_15	74359383	74359786	HERVHF_3LTR	+
Homo_sapiens_15	87831107	87831557	HERVHF_5LTR	+
Homo_sapiens_15	87833733	87834578	HERVHF_pro	+
Homo_sapiens_15	87833994	87835340	HERVHF_pol	+
Homo_sapiens_15	87836573	87837024	HERVHF_3LTR	+
Homo_sapiens_15	94755298	94755669	HERVHF_5LTR	+
Homo_sapiens_15	94756514	94756834	HERVHF_gag	+
Homo_sapiens_15	94756948	94757781	HERVHF_pro	+
Homo_sapiens_15	94757239	94759185	HERVHF_pol	+
Homo_sapiens_15	94759210	94759572	HERVHF_3LTR	+
Homo_sapiens_16	60078536	60078989	HERVHF_5LTR	+
Homo_sapiens_16	60080893	60081216	HERVHF_gag	+
Homo_sapiens_16	60081323	60082141	HERVHF_pro	+
Homo_sapiens_16	60081599	60083447	HERVHF_pol	+
Homo_sapiens_16	60084128	60084582	HERVHF_3LTR	+
Homo_sapiens_16	65229803	65230258	HERVHF_5LTR	-
Homo_sapiens_16	65231100	65232797	HERVHF_pol	-
Homo_sapiens_16	65232255	65233088	HERVHF_pro	-
Homo_sapiens_16	65233092	65233421	HERVHF_gag	-
Homo_sapiens_16	65234898	65235350	HERVHF_3LTR	-
Homo_sapiens_16	86278096	86278535	HERVHF_5LTR	+
Homo_sapiens_16	86279128	86279901	HERVHF_pro	+
Homo_sapiens_16	86279209	86279865	HERVHF_pol	+
Homo_sapiens_16	86280230	86280676	HERVHF_env	+
Homo_sapiens_16	86280838	86281288	HERVHF_3LTR	+
Homo_sapiens_17	34183197	34183525	HERVHF_5LTR	+
Homo_sapiens_17	34185335	34185751	HERVHF_gag	+
Homo_sapiens_17	34185803	34186567	HERVHF_pro	+
Homo_sapiens_17	34185950	34187742	HERVHF_pol	+
Homo_sapiens_17	34188574	34188906	HERVHF_3LTR	+
Homo_sapiens_18	28693028	28693465	HERVHF_5LTR	-
Homo_sapiens_18	28694048	28696068	HERVHF_pol	-
Homo_sapiens_18	28696387	28696686	HERVHF_gag	-
Homo_sapiens_18	28698449	28698882	HERVHF_3LTR	-
Homo_sapiens_18	56415436	56415887	HERVHF_5LTR	+
Homo_sapiens_18	56417745	56418068	HERVHF_gag	+
Homo_sapiens_18	56418362	56420489	HERVHF_pol	+
Homo_sapiens_18	56420893	56421344	HERVHF_3LTR	+
Homo_sapiens_18	57064647	57065099	HERVHF_5LTR	-
Homo_sapiens_18	57065267	57065866	HERVHF_env	-
Homo_sapiens_18	57067308	57069458	HERVHF_pol	-
Homo_sapiens_18	57069033	57069632	HERVHF_pro	-
Homo_sapiens_18	57069916	57070296	HERVHF_gag	-
Homo_sapiens_18	57072050	57072502	HERVHF_3LTR	-

Homo_sapiens_18	73324612	73325047	HERVHF_5LTR	+
Homo_sapiens_18	73327171	73327950	HERVHF_pro	+
Homo_sapiens_18	73327408	73329488	HERVHF_pol	+
Homo_sapiens_18	73329931	73330369	HERVHF_3LTR	+
Homo_sapiens_19	22568269	22568753	HERVHF_5LTR	+
Homo_sapiens_19	22571012	22573853	HERVHF_pol	+
Homo_sapiens_19	22574536	22575022	HERVHF_3LTR	+
Homo_sapiens_19	47049861	47050298	HERVHF_5LTR	-
Homo_sapiens_19	47051098	47052722	HERVHF_pol	-
Homo_sapiens_19	47052243	47052776	HERVHF_pro	-
Homo_sapiens_19	47053047	47053558	HERVHF_gag	-
Homo_sapiens_19	47054979	47055419	HERVHF_3LTR	-
Homo_sapiens_19	47392829	47393271	HERVHF_5LTR	+
Homo_sapiens_19	47395198	47395506	HERVHF_gag	+
Homo_sapiens_19	47395759	47396322	HERVHF_pro	+
Homo_sapiens_19	47395765	47397689	HERVHF_pol	+
Homo_sapiens_19	47398316	47398759	HERVHF_3LTR	+
Homo_sapiens_19	53831404	53831857	HERVHF_5LTR	+
Homo_sapiens_19	53833245	53833670	HERVHF_gag	+
Homo_sapiens_19	53834067	53834912	HERVHF_pro	+
Homo_sapiens_19	53834274	53836445	HERVHF_pol	+
Homo_sapiens_19	53836889	53837351	HERVHF_3LTR	+
Homo_sapiens_19	54894295	54894715	HERVHF_5LTR	-
Homo_sapiens_19	54895547	54897591	HERVHF_pol	-
Homo_sapiens_19	54897602	54898156	HERVHF_pro	-
Homo_sapiens_19	54900183	54900598	HERVHF_3LTR	-
Homo_sapiens_2	5000768	5001218	HERVHF_5LTR	+
Homo_sapiens_2	5003044	5003355	HERVHF_gag	+
Homo_sapiens_2	5003462	5004271	HERVHF_pro	+
Homo_sapiens_2	5003750	5005875	HERVHF_pol	+
Homo_sapiens_2	5006279	5006732	HERVHF_3LTR	+
Homo_sapiens_2	16791950	16792379	HERVHF_5LTR	-
Homo_sapiens_2	16792786	16794855	HERVHF_pol	-
Homo_sapiens_2	16794364	16795194	HERVHF_pro	-
Homo_sapiens_2	16795201	16795527	HERVHF_gag	-
Homo_sapiens_2	16797278	16797713	HERVHF_3LTR	-
Homo_sapiens_2	34789818	34790229	HERVHF_5LTR	+
Homo_sapiens_2	34791769	34792152	HERVHF_gag	+
Homo_sapiens_2	34792609	34793756	HERVHF_pol	+
Homo_sapiens_2	34794693	34795526	HERVHF_env	+
Homo_sapiens_2	34795641	34796058	HERVHF_3LTR	+
Homo_sapiens_2	36599100	36599527	HERVHF_5LTR	+
Homo_sapiens_2	36601097	36601510	HERVHF_gag	+
Homo_sapiens_2	36601597	36602346	HERVHF_pro	+

Homo_sapiens_2	36601708	36603923	HERVHF_pol	+
Homo_sapiens_2	36604333	36604763	HERVHF_3LTR	+
Homo_sapiens_2	38080800	38081210	HERVHF_5LTR	-
Homo_sapiens_2	38081220	38081575	HERVHF_env	-
Homo_sapiens_2	38081793	38083597	HERVHF_pol	-
Homo_sapiens_2	38083220	38083771	HERVHF_pro	-
Homo_sapiens_2	38086112	38086513	HERVHF_3LTR	-
Homo_sapiens_2	58113434	58113791	HERVHF_5LTR	-
Homo_sapiens_2	58113879	58114195	HERVHF_env	-
Homo_sapiens_2	58114467	58116483	HERVHF_pol	-
Homo_sapiens_2	58115845	58116678	HERVHF_pro	-
Homo_sapiens_2	58118698	58119055	HERVHF_3LTR	-
Homo_sapiens_2	67331517	67331916	HERVHF_5LTR	+
Homo_sapiens_2	67333734	67334075	HERVHF_gag	+
Homo_sapiens_2	67334330	67335070	HERVHF_pro	+
Homo_sapiens_2	67334504	67336274	HERVHF_pol	+
Homo_sapiens_2	67337203	67337603	HERVHF_3LTR	+
Homo_sapiens_2	69789900	69790233	HUERSP_5LTR	-
Homo_sapiens_2	69794775	69795859	HUERSP_pol	-
Homo_sapiens_2	69795440	69795853	HUERSP_pro	-
Homo_sapiens_2	69799273	69799618	HUERSP_3LTR	-
Homo_sapiens_2	71086358	71086810	HERVHF_5LTR	-
Homo_sapiens_2	71087296	71088009	HERVHF_pol	-
Homo_sapiens_2	71087467	71088297	HERVHF_pro	-
Homo_sapiens_2	71090545	71090998	HERVHF_3LTR	-
Homo_sapiens_2	76573623	76574075	HERVHF_5LTR	-
Homo_sapiens_2	76574528	76576681	HERVHF_pol	-
Homo_sapiens_2	76577110	76577433	HERVHF_gag	-
Homo_sapiens_2	76579252	76579704	HERVHF_3LTR	-
Homo_sapiens_2	77088248	77088587	HERVHF_5LTR	+
Homo_sapiens_2	77090896	77091681	HERVHF_pro	+
Homo_sapiens_2	77091043	77093257	HERVHF_pol	+
Homo_sapiens_2	77093690	77094041	HERVHF_3LTR	+
Homo_sapiens_2	77965137	77965570	HERVHF_5LTR	+
Homo_sapiens_2	77967584	77968414	HERVHF_pro	+
Homo_sapiens_2	77967872	77970012	HERVHF_pol	+
Homo_sapiens_2	77970418	77970868	HERVHF_3LTR	+
Homo_sapiens_2	116183751	116184169	HERVHF_5LTR	-
Homo_sapiens_2	116184795	116187097	HERVHF_pol	-
Homo_sapiens_2	116186690	116187325	HERVHF_pro	-
Homo_sapiens_2	116187528	116187854	HERVHF_gag	-
Homo_sapiens_2	116189298	116189711	HERVHF_3LTR	-
Homo_sapiens_2	140426394	140426788	HERVHF_5LTR	+
Homo_sapiens_2	140428048	140428524	HERVHF_gag	+

Homo_sapiens_2	140428698	140429447	HERVHF_pro	+
Homo_sapiens_2	140428950	140431540	HERVHF_pol	+
Homo_sapiens_2	140431994	140432382	HERVHF_3LTR	+
Homo_sapiens_2	150112716	150113171	HERVHF_5LTR	+
Homo_sapiens_2	150114585	150115055	HERVHF_gag	+
Homo_sapiens_2	150115415	150117524	HERVHF_pol	+
Homo_sapiens_2	150118100	150118566	HERVHF_3LTR	+
Homo_sapiens_2	154867350	154867726	HERVHF_5LTR	+
Homo_sapiens_2	154870083	154871371	HERVHF_pol	+
Homo_sapiens_2	154873073	154873885	HERVHF_env	+
Homo_sapiens_2	154874196	154874560	HERVHF_3LTR	+
Homo_sapiens_2	156720089	156720504	HERVHF_5LTR	-
Homo_sapiens_2	156720949	156723097	HERVHF_pol	-
Homo_sapiens_2	156723325	156723648	HERVHF_gag	-
Homo_sapiens_2	156725439	156725860	HERVHF_3LTR	-
Homo_sapiens_2	164295810	164296142	HERVHF_5LTR	+
Homo_sapiens_2	164297998	164298327	HERVHF_gag	+
Homo_sapiens_2	164298324	164299157	HERVHF_pro	+
Homo_sapiens_2	164298615	164300513	HERVHF_pol	+
Homo_sapiens_2	164301116	164301451	HERVHF_3LTR	+
Homo_sapiens_2	170977457	170977864	HERVHF_5LTR	-
Homo_sapiens_2	170978313	170980466	HERVHF_pol	-
Homo_sapiens_2	170980086	170980841	HERVHF_pro	-
Homo_sapiens_2	170981009	170981464	HERVHF_gag	-
Homo_sapiens_2	170982984	170983388	HERVHF_3LTR	-
Homo_sapiens_2	192130350	192130819	HERVHF_5LTR	+
Homo_sapiens_2	192132978	192133724	HERVHF_pro	+
Homo_sapiens_2	192133182	192135273	HERVHF_pol	+
Homo_sapiens_2	192135644	192136107	HERVHF_3LTR	+
Homo_sapiens_2	192506078	192506492	HERVHF_5LTR	+
Homo_sapiens_2	192507423	192509865	HERVHF_gag	+
Homo_sapiens_2	192510149	192510781	HERVHF_pro	+
Homo_sapiens_2	192510377	192512317	HERVHF_pol	+
Homo_sapiens_2	192512768	192513184	HERVHF_3LTR	+
Homo_sapiens_2	192918287	192918656	HERVHF_5LTR	+
Homo_sapiens_2	192920211	192920537	HERVHF_gag	+
Homo_sapiens_2	192920620	192921390	HERVHF_pro	+
Homo_sapiens_2	192920827	192921543	HERVHF_pol	+
Homo_sapiens_2	192923669	192924253	HERVHF_env	+
Homo_sapiens_2	192924402	192924763	HERVHF_3LTR	+
Homo_sapiens_2	194130688	194131111	HERVHF_5LTR	-
Homo_sapiens_2	194131526	194133889	HERVHF_pol	-
Homo_sapiens_2	194133188	194134122	HERVHF_pro	-
Homo_sapiens_2	194136134	194136553	HERVHF_3LTR	-

Homo_sapiens_2	215922303	215922762	HERVHF_5LTR	+
Homo_sapiens_2	215924520	215924849	HERVHF_gag	+
Homo_sapiens_2	215924901	215925665	HERVHF_pro	+
Homo_sapiens_2	215925144	215927087	HERVHF_pol	+
Homo_sapiens_2	215927674	215928129	HERVHF_3LTR	+
Homo_sapiens_2	218174020	218174458	HERVHF_5LTR	-
Homo_sapiens_2	218175024	218176780	HERVHF_pol	-
Homo_sapiens_2	218176500	218177294	HERVHF_pro	-
Homo_sapiens_2	218177318	218177701	HERVHF_gag	-
Homo_sapiens_2	218179456	218179903	HERVHF_3LTR	-
Homo_sapiens_2	224225331	224225730	HERVHF_5LTR	+
Homo_sapiens_2	224228022	224228600	HERVHF_pro	+
Homo_sapiens_2	224228193	224229742	HERVHF_pol	+
Homo_sapiens_2	224230587	224230988	HERVHF_3LTR	+
Homo_sapiens_2	224296632	224297045	HERVHF_5LTR	+
Homo_sapiens_2	224299201	224299959	HERVHF_pro	+
Homo_sapiens_2	224299579	224301326	HERVHF_pol	+
Homo_sapiens_2	224301940	224302365	HERVHF_3LTR	+
Homo_sapiens_2	237606784	237607197	HERVHF_5LTR	+
Homo_sapiens_2	237609020	237609400	HERVHF_gag	+
Homo_sapiens_2	237609517	237610233	HERVHF_pro	+
Homo_sapiens_2	237609622	237611630	HERVHF_pol	+
Homo_sapiens_2	237612243	237612656	HERVHF_3LTR	+
Homo_sapiens_20	12340267	12340705	HERVHF_5LTR	-
Homo_sapiens_20	12341115	12343098	HERVHF_pol	-
Homo_sapiens_20	12343106	12343498	HERVHF_pro	-
Homo_sapiens_20	12343532	12343852	HERVHF_gag	-
Homo_sapiens_20	12345506	12345940	HERVHF_3LTR	-
Homo_sapiens_20	12753802	12754276	HERVHF_5LTR	+
Homo_sapiens_20	12756027	12756350	HERVHF_gag	+
Homo_sapiens_20	12756779	12758770	HERVHF_pol	+
Homo_sapiens_20	12759162	12759632	HERVHF_3LTR	+
Homo_sapiens_20	19752046	19752476	HERVHF_5LTR	-
Homo_sapiens_20	19752891	19753827	HERVHF_pol	-
Homo_sapiens_20	19753824	19754375	HERVHF_pro	-
Homo_sapiens_20	19754465	19754815	HERVHF_gag	-
Homo_sapiens_20	19756340	19756776	HERVHF_3LTR	-
Homo_sapiens_20	40269047	40269508	HERVHF_5LTR	+
Homo_sapiens_20	40271560	40272339	HERVHF_pro	+
Homo_sapiens_20	40271758	40273720	HERVHF_pol	+
Homo_sapiens_20	40274312	40274769	HERVHF_3LTR	+
Homo_sapiens_21	16914128	16914536	HERVHF_5LTR	+
Homo_sapiens_21	16916125	16916436	HERVHF_gag	+
Homo_sapiens_21	16916717	16917301	HERVHF_pro	+

Homo_sapiens_21	16916894	16918726	HERVHF_pol	+
Homo_sapiens_21	16919493	16919901	HERVHF_3LTR	+
Homo_sapiens_21	17121415	17121851	HERVHF_5LTR	+
Homo_sapiens_21	17123651	17123971	HERVHF_gag	+
Homo_sapiens_21	17124024	17124968	HERVHF_pro	+
Homo_sapiens_21	17124222	17126293	HERVHF_pol	+
Homo_sapiens_21	17127325	17127764	HERVHF_3LTR	+
Homo_sapiens_21	26227947	26228284	HERVHF_5LTR	-
Homo_sapiens_21	26228683	26231015	HERVHF_pol	-
Homo_sapiens_21	26230473	26231054	HERVHF_pro	-
Homo_sapiens_21	26231389	26231739	HERVHF_gag	-
Homo_sapiens_21	26233157	26233485	HERVHF_3LTR	-
Homo_sapiens_21	42800845	42801294	HERVHF_5LTR	-
Homo_sapiens_21	42801909	42803999	HERVHF_pol	-
Homo_sapiens_21	42806130	42806578	HERVHF_3LTR	-
Homo_sapiens_3	890691	891105	HERVHF_5LTR	-
Homo_sapiens_3	891555	893583	HERVHF_pol	-
Homo_sapiens_3	893071	893922	HERVHF_pro	-
Homo_sapiens_3	895996	896413	HERVHF_3LTR	-
Homo_sapiens_3	8177035	8177443	HERVHF_5LTR	+
Homo_sapiens_3	8179571	8180296	HERVHF_pro	+
Homo_sapiens_3	8179778	8181790	HERVHF_pol	+
Homo_sapiens_3	8182306	8182722	HERVHF_3LTR	+
Homo_sapiens_3	21189031	21189353	HERVHF_5LTR	-
Homo_sapiens_3	21189872	21191978	HERVHF_pol	-
Homo_sapiens_3	21192518	21192895	HERVHF_gag	-
Homo_sapiens_3	21193803	21194139	HERVHF_3LTR	-
Homo_sapiens_3	35874404	35874736	HERVHF_5LTR	-
Homo_sapiens_3	35875354	35877250	HERVHF_pol	-
Homo_sapiens_3	35876771	35877529	HERVHF_pro	-
Homo_sapiens_3	35877674	35878018	HERVHF_gag	-
Homo_sapiens_3	35879665	35879998	HERVHF_3LTR	-
Homo_sapiens_3	38075877	38076624	HERVHF_5LTR	-
Homo_sapiens_3	38077539	38079107	HERVHF_pol	-
Homo_sapiens_3	38078700	38079281	HERVHF_pro	-
Homo_sapiens_3	38079536	38079865	HERVHF_gag	-
Homo_sapiens_3	38082755	38083512	HERVHF_3LTR	-
Homo_sapiens_3	54634482	54634938	HERVHF_5LTR	-
Homo_sapiens_3	54635330	54637456	HERVHF_pol	-
Homo_sapiens_3	54636914	54637756	HERVHF_pro	-
Homo_sapiens_3	54637769	54638068	HERVHF_gag	-
Homo_sapiens_3	54639751	54640205	HERVHF_3LTR	-
Homo_sapiens_3	72023124	72023534	HERVHF_5LTR	+
Homo_sapiens_3	72025143	72025466	HERVHF_gag	+

Homo_sapiens_3	72025538	72026323	HERVHF_pro	+
Homo_sapiens_3	72025664	72028047	HERVHF_pol	+
Homo_sapiens_3	72028493	72028898	HERVHF_3LTR	+
Homo_sapiens_3	104439927	104440361	HERVHF_5LTR	-
Homo_sapiens_3	104440832	104442835	HERVHF_pol	-
Homo_sapiens_3	104442338	104443171	HERVHF_pro	-
Homo_sapiens_3	104443175	104443588	HERVHF_gag	-
Homo_sapiens_3	104445340	104445775	HERVHF_3LTR	-
Homo_sapiens_3	105601130	105601544	HSERVIII_5LTR	-
Homo_sapiens_3	105602260	105604076	HSERVIII_pol	-
Homo_sapiens_3	105607168	105607579	HSERVIII_3LTR	-
Homo_sapiens_3	112418410	112418865	HERVHF_5LTR	-
Homo_sapiens_3	112419048	112420617	HERVHF_pol	-
Homo_sapiens_3	112420824	112421368	HERVHF_gag	-
Homo_sapiens_3	112422911	112423366	HERVHF_3LTR	-
Homo_sapiens_3	115793481	115793932	HERVHF_5LTR	-
Homo_sapiens_3	115794526	115796364	HERVHF_pol	-
Homo_sapiens_3	115796762	115797091	HERVHF_gag	-
Homo_sapiens_3	115798715	115799166	HERVHF_3LTR	-
Homo_sapiens_3	128959817	128960144	HERVHF_5LTR	+
Homo_sapiens_3	128962177	128963010	HERVHF_pro	+
Homo_sapiens_3	128962468	128964143	HERVHF_pol	+
Homo_sapiens_3	128964958	128965264	HERVHF_3LTR	+
Homo_sapiens_3	130059222	130059571	HERVHF_5LTR	+
Homo_sapiens_3	130062082	130064170	HERVHF_pol	+
Homo_sapiens_3	130064629	130064980	HERVHF_3LTR	+
Homo_sapiens_3	137595594	137596055	HERVHF_5LTR	+
Homo_sapiens_3	137597610	137597921	HERVHF_gag	+
Homo_sapiens_3	137598245	137600583	HERVHF_pol	+
Homo_sapiens_3	137600742	137601199	HERVHF_3LTR	+
Homo_sapiens_3	148414244	148414700	HERVHF_5LTR	+
Homo_sapiens_3	148416409	148416780	HERVHF_gag	+
Homo_sapiens_3	148416909	148417658	HERVHF_pro	+
Homo_sapiens_3	148417290	148419056	HERVHF_pol	+
Homo_sapiens_3	148419646	148420099	HERVHF_3LTR	+
Homo_sapiens_3	153226145	153226572	HERVHF_5LTR	-
Homo_sapiens_3	153228373	153229655	HERVHF_pol	-
Homo_sapiens_3	153229116	153229943	HERVHF_pro	-
Homo_sapiens_3	153229950	153230288	HERVHF_gag	-
Homo_sapiens_3	153232110	153232531	HERVHF_3LTR	-
Homo_sapiens_3	155274423	155274841	HERVHF_5LTR	-
Homo_sapiens_3	155275702	155278149	HERVHF_pol	-
Homo_sapiens_3	155277628	155278386	HERVHF_pro	-
Homo_sapiens_3	155278439	155278762	HERVHF_gag	-

Homo_sapiens_3	155280382	155280792	HERVHF_3LTR	-
Homo_sapiens_3	161721912	161722322	HERVHF_5LTR	-
Homo_sapiens_3	161722774	161724851	HERVHF_pol	-
Homo_sapiens_3	161724288	161725067	HERVHF_pro	-
Homo_sapiens_3	161725118	161725534	HERVHF_gag	-
Homo_sapiens_3	161727366	161727777	HERVHF_3LTR	-
Homo_sapiens_3	166500682	166501004	HERVHF_5LTR	-
Homo_sapiens_3	166501414	166503748	HERVHF_pol	-
Homo_sapiens_3	166503206	166503985	HERVHF_pro	-
Homo_sapiens_3	166506099	166506419	HERVHF_3LTR	-
Homo_sapiens_3	170681756	170682257	HERVHF_5LTR	-
Homo_sapiens_3	170682420	170683196	HERVHF_env	-
Homo_sapiens_3	170684340	170686398	HERVHF_pol	-
Homo_sapiens_3	170685754	170686533	HERVHF_pro	-
Homo_sapiens_3	170688805	170689306	HERVHF_3LTR	-
Homo_sapiens_3	178207407	178207830	HERVHF_5LTR	+
Homo_sapiens_3	178209668	178209979	HERVHF_gag	+
Homo_sapiens_3	178210068	178210847	HERVHF_pro	+
Homo_sapiens_3	178210203	178212316	HERVHF_pol	+
Homo_sapiens_3	178213056	178214020	HERVHF_env	+
Homo_sapiens_3	178214244	178214674	HERVHF_3LTR	+
Homo_sapiens_3	186657773	186658172	HERVHF_5LTR	+
Homo_sapiens_3	186659818	186660423	HERVHF_gag	+
Homo_sapiens_3	186660747	186661298	HERVHF_pro	+
Homo_sapiens_3	186660786	186662849	HERVHF_pol	+
Homo_sapiens_3	186663286	186663692	HERVHF_3LTR	+
Homo_sapiens_3	190920164	190920619	HERVHF_5LTR	+
Homo_sapiens_3	190923015	190924819	HERVHF_pol	+
Homo_sapiens_3	190925408	190925863	HERVHF_3LTR	+
Homo_sapiens_3	191666258	191666713	HERVHF_5LTR	-
Homo_sapiens_3	191667212	191669450	HERVHF_pol	-
Homo_sapiens_3	191668908	191669738	HERVHF_pro	-
Homo_sapiens_3	191669745	191670077	HERVHF_gag	-
Homo_sapiens_3	191671926	191672379	HERVHF_3LTR	-
Homo_sapiens_3	191895458	191895798	HERVHF_5LTR	-
Homo_sapiens_3	191896350	191898124	HERVHF_pol	-
Homo_sapiens_3	191900805	191901132	HERVHF_3LTR	-
Homo_sapiens_3	192071107	192071527	HERVHF_5LTR	-
Homo_sapiens_3	192072114	192074052	HERVHF_pol	-
Homo_sapiens_3	192073516	192074289	HERVHF_pro	-
Homo_sapiens_3	192074342	192074671	HERVHF_gag	-
Homo_sapiens_3	192076443	192076859	HERVHF_3LTR	-
Homo_sapiens_4	3927445	3927854	HERVHF_5LTR	+
Homo_sapiens_4	3929526	3929825	HERVHF_gag	+

Homo_sapiens_4	3929852	3930682	HERVHF_pro	+
Homo_sapiens_4	3930140	3932053	HERVHF_pol	+
Homo_sapiens_4	3932671	3933082	HERVHF_3LTR	+
Homo_sapiens_4	14169946	14170399	HERVHF_5LTR	-
Homo_sapiens_4	14171508	14173531	HERVHF_pol	-
Homo_sapiens_4	14172947	14173783	HERVHF_pro	-
Homo_sapiens_4	14173823	14174227	HERVHF_gag	-
Homo_sapiens_4	14175524	14175976	HERVHF_3LTR	-
Homo_sapiens_4	16997808	16998200	HERVHF_5LTR	+
Homo_sapiens_4	17000545	17002680	HERVHF_pol	+
Homo_sapiens_4	17003527	17003928	HERVHF_3LTR	+
Homo_sapiens_4	24500975	24501427	HERVHF_5LTR	+
Homo_sapiens_4	24503155	24503454	HERVHF_gag	+
Homo_sapiens_4	24503488	24504321	HERVHF_pro	+
Homo_sapiens_4	24503779	24505852	HERVHF_pol	+
Homo_sapiens_4	24506300	24506752	HERVHF_3LTR	+
Homo_sapiens_4	27974874	27975246	HERVHF_5LTR	+
Homo_sapiens_4	27975998	27976339	HERVHF_gag	+
Homo_sapiens_4	27976612	27977274	HERVHF_pro	+
Homo_sapiens_4	27976735	27980331	HERVHF_pol	+
Homo_sapiens_4	27980964	27981319	HERVHF_3LTR	+
Homo_sapiens_4	61764274	61764699	HERVHF_5LTR	-
Homo_sapiens_4	61765149	61767131	HERVHF_pol	-
Homo_sapiens_4	61766724	61767359	HERVHF_pro	-
Homo_sapiens_4	61767632	61768099	HERVHF_gag	-
Homo_sapiens_4	61769601	61770008	HERVHF_3LTR	-
Homo_sapiens_4	78713840	78714293	HERVHF_5LTR	-
Homo_sapiens_4	78714700	78716826	HERVHF_pol	-
Homo_sapiens_4	78716284	78716919	HERVHF_pro	-
Homo_sapiens_4	78717122	78717439	HERVHF_gag	-
Homo_sapiens_4	78719270	78719721	HERVHF_3LTR	-
Homo_sapiens_4	79554023	79554421	HERVHF_5LTR	-
Homo_sapiens_4	79555128	79556943	HERVHF_pol	-
Homo_sapiens_4	79556428	79557150	HERVHF_pro	-
Homo_sapiens_4	79557241	79557651	HERVHF_gag	-
Homo_sapiens_4	79559319	79559718	HERVHF_3LTR	-
Homo_sapiens_4	91623641	91624091	HERVHF_5LTR	+
Homo_sapiens_4	91626641	91627363	HERVHF_pro	+
Homo_sapiens_4	91626752	91628928	HERVHF_pol	+
Homo_sapiens_4	91629248	91629703	HERVHF_3LTR	+
Homo_sapiens_4	92271492	92271860	HERVHF_5LTR	-
Homo_sapiens_4	92272378	92274561	HERVHF_pol	-
Homo_sapiens_4	92273926	92274771	HERVHF_pro	-
Homo_sapiens_4	92274781	92275299	HERVHF_gag	-

Homo_sapiens_4	92277196	92277564	HERVHF_3LTR	-
Homo_sapiens_4	94382917	94383320	HERVHF_5LTR	-
Homo_sapiens_4	94384188	94386301	HERVHF_pol	-
Homo_sapiens_4	94386732	94387058	HERVHF_gag	-
Homo_sapiens_4	94388862	94389273	HERVHF_3LTR	-
Homo_sapiens_4	103553770	103554203	HERVHF_5LTR	-
Homo_sapiens_4	103554640	103556729	HERVHF_pol	-
Homo_sapiens_4	103556187	103557020	HERVHF_pro	-
Homo_sapiens_4	103557024	103557353	HERVHF_gag	-
Homo_sapiens_4	103559041	103559475	HERVHF_3LTR	-
Homo_sapiens_4	119993716	119994118	HERVHF_5LTR	+
Homo_sapiens_4	119995847	119996302	HERVHF_gag	+
Homo_sapiens_4	119996399	119997211	HERVHF_pro	+
Homo_sapiens_4	119996690	119997978	HERVHF_pol	+
Homo_sapiens_4	119999268	119999651	HERVHF_3LTR	+
Homo_sapiens_4	125561618	125562071	HERVHF_5LTR	-
Homo_sapiens_4	125562473	125564689	HERVHF_pol	-
Homo_sapiens_4	125564897	125565226	HERVHF_gag	-
Homo_sapiens_4	125567006	125567457	HERVHF_3LTR	-
Homo_sapiens_4	128640901	128641313	HERVHF_5LTR	-
Homo_sapiens_4	128641891	128643929	HERVHF_pol	-
Homo_sapiens_4	128643291	128643971	HERVHF_pro	-
Homo_sapiens_4	128644112	128644450	HERVHF_gag	-
Homo_sapiens_4	128646206	128646616	HERVHF_3LTR	-
Homo_sapiens_4	130767409	130767812	HERVHF_5LTR	+
Homo_sapiens_4	130769391	130769807	HERVHF_gag	+
Homo_sapiens_4	130770133	130772462	HERVHF_pol	+
Homo_sapiens_4	130772870	130773270	HERVHF_3LTR	+
Homo_sapiens_4	133263584	133263969	HERVHF_5LTR	-
Homo_sapiens_4	133265166	133266533	HERVHF_pol	-
Homo_sapiens_4	133266407	133266946	HERVHF_pro	-
Homo_sapiens_4	133266953	133267396	HERVHF_gag	-
Homo_sapiens_4	133268524	133268917	HERVHF_3LTR	-
Homo_sapiens_4	134012205	134012609	HERVHF_5LTR	+
Homo_sapiens_4	134014655	134015497	HERVHF_pro	+
Homo_sapiens_4	134014943	134017565	HERVHF_pol	+
Homo_sapiens_4	134017974	134018374	HERVHF_3LTR	+
Homo_sapiens_4	135167433	135167868	HERVHF_5LTR	-
Homo_sapiens_4	135168278	135170266	HERVHF_pol	-
Homo_sapiens_4	135169703	135170473	HERVHF_pro	-
Homo_sapiens_4	135172687	135173119	HERVHF_3LTR	-
Homo_sapiens_4	138927837	138928239	HERVHF_5LTR	+
Homo_sapiens_4	138930431	138931174	HERVHF_pro	+
Homo_sapiens_4	138930632	138932695	HERVHF_pol	+

Homo_sapiens_4	138933142	138933547	HERVHF_3LTR	+
Homo_sapiens_4	145698823	145699274	HERVHF_5LTR	+
Homo_sapiens_4	145701596	145702378	HERVHF_pro	+
Homo_sapiens_4	145701734	145702528	HERVHF_pol	+
Homo_sapiens_4	145703056	145703505	HERVHF_3LTR	+
Homo_sapiens_4	152741345	152741806	HERVHF_5LTR	-
Homo_sapiens_4	152742614	152744164	HERVHF_pol	-
Homo_sapiens_4	152743760	152744263	HERVHF_pro	-
Homo_sapiens_4	152746701	152747172	HERVHF_3LTR	-
Homo_sapiens_4	156600197	156600651	HERVHF_5LTR	+
Homo_sapiens_4	156602433	156602756	HERVHF_gag	+
Homo_sapiens_4	156603049	156605073	HERVHF_pol	+
Homo_sapiens_4	156605584	156606038	HERVHF_3LTR	+
Homo_sapiens_4	166716125	166716585	HERVHF_5LTR	+
Homo_sapiens_4	166718361	166718774	HERVHF_gag	+
Homo_sapiens_4	166718781	166719614	HERVHF_pro	+
Homo_sapiens_4	166719069	166721085	HERVHF_pol	+
Homo_sapiens_4	166721604	166722057	HERVHF_3LTR	+
Homo_sapiens_4	167922959	167923319	HERVHF_5LTR	+
Homo_sapiens_4	167925658	167927423	HERVHF_pol	+
Homo_sapiens_4	167928277	167928630	HERVHF_3LTR	+
Homo_sapiens_4	170116433	170116847	HERVHF_5LTR	+
Homo_sapiens_4	170119176	170119760	HERVHF_pro	+
Homo_sapiens_4	170119353	170121157	HERVHF_pol	+
Homo_sapiens_4	170121747	170122164	HERVHF_3LTR	+
Homo_sapiens_4	175461163	175461498	HERVHF_5LTR	-
Homo_sapiens_4	175462086	175464209	HERVHF_pol	-
Homo_sapiens_4	175464527	175464889	HERVHF_pro	-
Homo_sapiens_4	175466677	175467003	HERVHF_3LTR	-
Homo_sapiens_4	178207119	178207533	HERVHF_5LTR	+
Homo_sapiens_4	178209276	178209596	HERVHF_gag	+
Homo_sapiens_4	178209862	178212501	HERVHF_pol	+
Homo_sapiens_4	178212550	178212918	HERVHF_env	+
Homo_sapiens_4	178212931	178213344	HERVHF_3LTR	+
Homo_sapiens_5	76357256	76357707	HERVHF_5LTR	+
Homo_sapiens_5	76359499	76359828	HERVHF_gag	+
Homo_sapiens_5	76359835	76360665	HERVHF_pro	+
Homo_sapiens_5	76360297	76361794	HERVHF_pol	+
Homo_sapiens_5	76362626	76363073	HERVHF_3LTR	+
Homo_sapiens_5	92823742	92824175	HERVHF_5LTR	+
Homo_sapiens_5	92826033	92826407	HERVHF_gag	+
Homo_sapiens_5	92826862	92828213	HERVHF_pol	+
Homo_sapiens_5	92829273	92829706	HERVHF_3LTR	+
Homo_sapiens_5	99010833	99011226	HERVHF_5LTR	-

Homo_sapiens_5	99011420	99011785	HERVHF_env	-
Homo_sapiens_5	99011881	99014153	HERVHF_pol	-
Homo_sapiens_5	99013746	99014375	HERVHF_pro	-
Homo_sapiens_5	99014627	99015070	HERVHF_gag	-
Homo_sapiens_5	99016533	99016911	HERVHF_3LTR	-
Homo_sapiens_5	100984293	100984733	HERVHF_5LTR	-
Homo_sapiens_5	100985518	100987320	HERVHF_pol	-
Homo_sapiens_5	100986703	100987425	HERVHF_pro	-
Homo_sapiens_5	100989791	100990240	HERVHF_3LTR	-
Homo_sapiens_5	104031710	104032120	HERVHF_5LTR	+
Homo_sapiens_5	104033762	104034061	HERVHF_gag	+
Homo_sapiens_5	104034512	104036512	HERVHF_pol	+
Homo_sapiens_5	104036554	104036891	HERVHF_env	+
Homo_sapiens_5	104036905	104037315	HERVHF_3LTR	+
Homo_sapiens_5	104063637	104064105	HERVHF_5LTR	-
Homo_sapiens_5	104064759	104067168	HERVHF_pol	-
Homo_sapiens_5	104066572	104067411	HERVHF_pro	-
Homo_sapiens_5	104070029	104070507	HERVHF_3LTR	-
Homo_sapiens_5	113711920	113712431	HERVK_5LTR	+
Homo_sapiens_5	113713169	113714110	HERVK_pro	+
Homo_sapiens_5	113714005	113714688	HERVK_pol	+
Homo_sapiens_5	113715448	113716020	HERVK_env	+
Homo_sapiens_5	113716118	113716618	HERVK_3LTR	+
Homo_sapiens_5	136301377	136301842	HERVHF_5LTR	+
Homo_sapiens_5	136303446	136303754	HERVHF_gag	+
Homo_sapiens_5	136303790	136304620	HERVHF_pro	+
Homo_sapiens_5	136304078	136305769	HERVHF_pol	+
Homo_sapiens_5	136306563	136307028	HERVHF_3LTR	+
Homo_sapiens_5	136565340	136565792	HERVHF_5LTR	-
Homo_sapiens_5	136566178	136568317	HERVHF_pol	-
Homo_sapiens_5	136567754	136568518	HERVHF_pro	-
Homo_sapiens_5	136568608	136568928	HERVHF_gag	-
Homo_sapiens_5	136570795	136571248	HERVHF_3LTR	-
Homo_sapiens_5	161245405	161245783	HERVHF_5LTR	+
Homo_sapiens_5	161250625	161250936	HERVHF_gag	+
Homo_sapiens_5	161251362	161253786	HERVHF_pol	+
Homo_sapiens_5	161254200	161254586	HERVHF_3LTR	+
Homo_sapiens_6	6027805	6028220	HERVHF_5LTR	-
Homo_sapiens_6	6028648	6030758	HERVHF_pol	-
Homo_sapiens_6	6030216	6031049	HERVHF_pro	-
Homo_sapiens_6	6031136	6031459	HERVHF_gag	-
Homo_sapiens_6	6033181	6033592	HERVHF_3LTR	-
Homo_sapiens_6	16259010	16259427	HERVHF_5LTR	-
Homo_sapiens_6	16260019	16261731	HERVHF_pol	-

Homo_sapiens_6 16261417 16262241 HERVHF_pro -
Homo_sapiens_6 16264480 16264893 HERVHF_3LTR -
Homo_sapiens_6 18754142 18754535 HERVHF_5LTR -
Homo_sapiens_6 18754988 18756902 HERVHF_pol -
Homo_sapiens_6 18759478 18759871 HERVHF_3LTR -
Homo_sapiens_6 51938231 51938689 HERVHF_5LTR +
Homo_sapiens_6 51940647 51941819 HERVHF_pro +
Homo_sapiens_6 51941547 51943125 HERVHF_pol +
Homo_sapiens_6 51943978 51944430 HERVHF_3LTR +
Homo_sapiens_6 77658019 77658462 HERVHF_5LTR -
Homo_sapiens_6 77658624 77659796 HERVHF_env -
Homo_sapiens_6 77660674 77663521 HERVHF_pol -
Homo_sapiens_6 77662979 77663782 HERVHF_pro -
Homo_sapiens_6 77665998 77666441 HERVHF_3LTR -
Homo_sapiens_6 80509795 80510208 HERVHF_5LTR -
Homo_sapiens_6 80510376 80513272 HERVHF_pol -
Homo_sapiens_6 80512745 80513302 HERVHF_pro -
Homo_sapiens_6 80513596 80513907 HERVHF_gag -
Homo_sapiens_6 80515391 80515805 HERVHF_3LTR -
Homo_sapiens_6 81337017 81337345 HERVHF_5LTR -
Homo_sapiens_6 81346215 81348209 HERVHF_pol -
Homo_sapiens_6 81347574 81348401 HERVHF_pro -
Homo_sapiens_6 81348431 81348814 HERVHF_gag -
Homo_sapiens_6 81351161 81351494 HERVHF_3LTR -
Homo_sapiens_6 90670268 90670654 HERVHF_5LTR -
Homo_sapiens_6 90671341 90673178 HERVHF_pol -
Homo_sapiens_6 90672639 90673466 HERVHF_pro -
Homo_sapiens_6 90673473 90673802 HERVHF_gag -
Homo_sapiens_6 90675059 90675449 HERVHF_3LTR -
Homo_sapiens_6 93830155 93830563 HERVHF_5LTR -
Homo_sapiens_6 93831028 93833044 HERVHF_pol -
Homo_sapiens_6 93832505 93833332 HERVHF_pro -
Homo_sapiens_6 93833339 93833659 HERVHF_gag -
Homo_sapiens_6 93835343 93835759 HERVHF_3LTR -
Homo_sapiens_6 94553917 94554368 HERVHF_5LTR -
Homo_sapiens_6 94554823 94556910 HERVHF_pol -
Homo_sapiens_6 94556395 94557201 HERVHF_pro -
Homo_sapiens_6 94559159 94559610 HERVHF_3LTR -
Homo_sapiens_6 97779489 97779949 HERVHF_5LTR +
Homo_sapiens_6 97782076 97782870 HERVHF_pro +
Homo_sapiens_6 97782271 97784463 HERVHF_pol +
Homo_sapiens_6 97784874 97785327 HERVHF_3LTR +
Homo_sapiens_6 109855762 109856184 HERVHF_5LTR -
Homo_sapiens_6 109856778 109858731 HERVHF_pol -

Homo_sapiens_6	109858192	109859019	HERVHF_pro	-
Homo_sapiens_6	109859109	109859429	HERVHF_gag	-
Homo_sapiens_6	109861156	109861571	HERVHF_3LTR	-
Homo_sapiens_6	115031787	115032245	HERVHF_5LTR	+
Homo_sapiens_6	115033944	115034261	HERVHF_gag	+
Homo_sapiens_6	115034360	115035187	HERVHF_pro	+
Homo_sapiens_6	115034648	115036338	HERVHF_pol	+
Homo_sapiens_6	115037164	115037622	HERVHF_3LTR	+
Homo_sapiens_6	121620508	121620869	HERVHF_5LTR	-
Homo_sapiens_6	121621463	121623501	HERVHF_pol	-
Homo_sapiens_6	121622890	121623696	HERVHF_pro	-
Homo_sapiens_6	121623700	121624113	HERVHF_gag	-
Homo_sapiens_6	121625942	121626294	HERVHF_3LTR	-
Homo_sapiens_6	123582333	123582734	HERVHF_5LTR	-
Homo_sapiens_6	123584586	123585260	HERVHF_pol	-
Homo_sapiens_6	123584721	123585290	HERVHF_pro	-
Homo_sapiens_6	123587604	123588007	HERVHF_3LTR	-
Homo_sapiens_6	124890407	124890844	HERVHF_5LTR	+
Homo_sapiens_6	124894190	124894510	HERVHF_gag	+
Homo_sapiens_6	124894940	124896785	HERVHF_pol	+
Homo_sapiens_6	124897330	124897764	HERVHF_3LTR	+
Homo_sapiens_6	125701846	125702300	HERVHF_5LTR	-
Homo_sapiens_6	125702812	125704819	HERVHF_pol	-
Homo_sapiens_6	125705125	125705535	HERVHF_gag	-
Homo_sapiens_6	125707310	125707764	HERVHF_3LTR	-
Homo_sapiens_6	126848846	126849276	HERVHF_5LTR	+
Homo_sapiens_6	126850727	126851197	HERVHF_gag	+
Homo_sapiens_6	126851224	126852054	HERVHF_pro	+
Homo_sapiens_6	126851557	126853403	HERVHF_pol	+
Homo_sapiens_6	126854026	126854456	HERVHF_3LTR	+
Homo_sapiens_6	131295347	131295808	HERVHF_5LTR	+
Homo_sapiens_6	131298228	131299742	HERVHF_pol	+
Homo_sapiens_6	131300740	131301206	HERVHF_3LTR	+
Homo_sapiens_6	131338799	131339252	HERVHF_5LTR	+
Homo_sapiens_6	131340935	131341324	HERVHF_gag	+
Homo_sapiens_6	131341378	131342177	HERVHF_pro	+
Homo_sapiens_6	131341770	131343672	HERVHF_pol	+
Homo_sapiens_6	131344112	131344566	HERVHF_3LTR	+
Homo_sapiens_6	131901550	131902010	HERVHF_5LTR	+
Homo_sapiens_6	131903830	131904159	HERVHF_gag	+
Homo_sapiens_6	131904166	131904993	HERVHF_pro	+
Homo_sapiens_6	131904451	131906364	HERVHF_pol	+
Homo_sapiens_6	131906969	131907420	HERVHF_3LTR	+
Homo_sapiens_6	144923164	144923609	HERVHF_5LTR	+

Homo_sapiens_6	144925945	144927834	HERVHF_pol	+
Homo_sapiens_6	144928414	144928866	HERVHF_3LTR	+
Homo_sapiens_6	148639773	148640185	HERVHF_5LTR	+
Homo_sapiens_6	148642529	148643080	HERVHF_pro	+
Homo_sapiens_6	148642703	148644508	HERVHF_pol	+
Homo_sapiens_6	148645099	148645512	HERVHF_3LTR	+
Homo_sapiens_6	160286609	160287012	HERVHF_5LTR	-
Homo_sapiens_6	160287604	160289407	HERVHF_pol	-
Homo_sapiens_6	160288982	160289581	HERVHF_pro	-
Homo_sapiens_6	160289925	160290245	HERVHF_gag	-
Homo_sapiens_6	160291906	160292315	HERVHF_3LTR	-
Homo_sapiens_6	164338761	164339178	HERVHF_5LTR	-
Homo_sapiens_6	164339767	164341641	HERVHF_pol	-
Homo_sapiens_6	164344350	164344780	HERVHF_3LTR	-
Homo_sapiens_7	9457701	9458151	HERVHF_5LTR	-
Homo_sapiens_7	9458606	9461567	HERVHF_pol	-
Homo_sapiens_7	9460893	9461804	HERVHF_pro	-
Homo_sapiens_7	9463767	9464220	HERVHF_3LTR	-
Homo_sapiens_7	26024199	26024639	HERVHF_5LTR	-
Homo_sapiens_7	26025479	26027163	HERVHF_pol	-
Homo_sapiens_7	26026567	26027451	HERVHF_pro	-
Homo_sapiens_7	26029364	26029809	HERVHF_3LTR	-
Homo_sapiens_7	31466857	31467260	HERVHF_5LTR	-
Homo_sapiens_7	31467710	31469839	HERVHF_pol	-
Homo_sapiens_7	31471973	31472383	HERVHF_3LTR	-
Homo_sapiens_7	32838745	32839187	HERVHF_5LTR	-
Homo_sapiens_7	32840141	32840968	HERVHF_pol	-
Homo_sapiens_7	32840339	32841040	HERVHF_pro	-
Homo_sapiens_7	32841194	32841577	HERVHF_gag	-
Homo_sapiens_7	32843099	32843543	HERVHF_3LTR	-
Homo_sapiens_7	34300132	34300573	HERVHF_5LTR	-
Homo_sapiens_7	34300978	34303075	HERVHF_pol	-
Homo_sapiens_7	34303183	34303482	HERVHF_gag	-
Homo_sapiens_7	34305132	34305573	HERVHF_3LTR	-
Homo_sapiens_7	56144904	56145221	HERVK_5LTR	+
Homo_sapiens_7	56146786	56147721	HERVK_pro	+
Homo_sapiens_7	56147616	56149111	HERVK_pol	+
Homo_sapiens_7	56149952	56152862	HERVK_env	+
Homo_sapiens_7	56152939	56153268	HERVK_3LTR	+
Homo_sapiens_7	83459667	83460120	HERVHF_5LTR	+
Homo_sapiens_7	83462141	83462971	HERVHF_pro	+
Homo_sapiens_7	83462429	83464125	HERVHF_pol	+
Homo_sapiens_7	83464935	83465385	HERVHF_3LTR	+
Homo_sapiens_7	88540114	88540553	HUERSP_5LTR	-

Homo_sapiens_7	88542213	88544382	HUERSP_pol	-
Homo_sapiens_7	88547818	88548264	HUERSP_3LTR	-
Homo_sapiens_7	102973207	102973622	HERVHF_5LTR	+
Homo_sapiens_7	102975230	102975898	HERVHF_gag	+
Homo_sapiens_7	102976371	102977021	HERVHF_pro	+
Homo_sapiens_7	102976449	102977042	HERVHF_pol	+
Homo_sapiens_7	102977224	102977607	HERVHF_env	+
Homo_sapiens_7	102978322	102978736	HERVHF_3LTR	+
Homo_sapiens_7	113234431	113234903	HERVHF_5LTR	-
Homo_sapiens_7	113235994	113236650	HERVHF_pol	-
Homo_sapiens_7	113236012	113236845	HERVHF_pro	-
Homo_sapiens_7	113236869	113237168	HERVHF_gag	-
Homo_sapiens_7	113238838	113239309	HERVHF_3LTR	-
Homo_sapiens_7	120776620	120777025	HERVHF_5LTR	-
Homo_sapiens_7	120777481	120780152	HERVHF_pol	-
Homo_sapiens_7	120779568	120780401	HERVHF_pro	-
Homo_sapiens_7	120782483	120782888	HERVHF_3LTR	-
Homo_sapiens_7	125917557	125917967	HERVHF_5LTR	+
Homo_sapiens_7	125920130	125920885	HERVHF_pro	+
Homo_sapiens_7	125920475	125922154	HERVHF_pol	+
Homo_sapiens_7	125923701	125924112	HERVHF_3LTR	+
Homo_sapiens_7	141578060	141578418	HERVHF_5LTR	-
Homo_sapiens_7	141579378	141581884	HERVHF_pol	-
Homo_sapiens_7	141581123	141581836	HERVHF_pro	-
Homo_sapiens_7	141582139	141582486	HERVHF_gag	-
Homo_sapiens_7	141584031	141584376	HERVHF_3LTR	-
Homo_sapiens_7	148309409	148309795	HERVHF_5LTR	-
Homo_sapiens_7	148310061	148310791	HERVHF_env	-
Homo_sapiens_7	148311723	148312985	HERVHF_pol	-
Homo_sapiens_7	148313374	148313883	HERVHF_gag	-
Homo_sapiens_7	148315201	148315584	HERVHF_3LTR	-
Homo_sapiens_7	155238209	155238582	HERVK_5LTR	-
Homo_sapiens_7	155238821	155239129	HERVK_env	-
Homo_sapiens_7	155239444	155241440	HERVK_pol	-
Homo_sapiens_7	155241335	155242276	HERVK_pro	-
Homo_sapiens_7	155243682	155244070	HERVK_3LTR	-
Homo_sapiens_8	71674934	71675240	HERVHF_5LTR	+
Homo_sapiens_8	71676972	71677343	HERVHF_gag	+
Homo_sapiens_8	71677387	71678241	HERVHF_pro	+
Homo_sapiens_8	71677678	71679621	HERVHF_pol	+
Homo_sapiens_8	71680214	71680514	HERVHF_3LTR	+
Homo_sapiens_8	79386104	79386539	HERVHF_5LTR	-
Homo_sapiens_8	79387308	79389168	HERVHF_pol	-
Homo_sapiens_8	79388557	79389695	HERVHF_pro	-

Homo_sapiens_8	79389480	79389920	HERVHF_gag	-
Homo_sapiens_8	79391251	79391685	HERVHF_3LTR	-
Homo_sapiens_8	90090224	90090661	HERVHF_5LTR	-
Homo_sapiens_8	90091063	90093283	HERVHF_pol	-
Homo_sapiens_8	90093495	90093794	HERVHF_gag	-
Homo_sapiens_8	90095435	90095869	HERVHF_3LTR	-
Homo_sapiens_8	97200769	97201222	HERVHF_5LTR	+
Homo_sapiens_8	97202976	97203299	HERVHF_gag	+
Homo_sapiens_8	97203392	97204225	HERVHF_pro	+
Homo_sapiens_8	97203683	97205588	HERVHF_pol	+
Homo_sapiens_8	97206202	97206658	HERVHF_3LTR	+
Homo_sapiens_8	99943774	99944145	HERVHF_5LTR	+
Homo_sapiens_8	99946395	99947180	HERVHF_pro	+
Homo_sapiens_8	99946638	99948330	HERVHF_pol	+
Homo_sapiens_8	99949257	99949628	HERVHF_3LTR	+
Homo_sapiens_8	104285074	104285911	HERVHF_5LTR	+
Homo_sapiens_8	104287994	104288407	HERVHF_gag	+
Homo_sapiens_8	104288703	104290422	HERVHF_pol	+
Homo_sapiens_8	104292094	104292933	HERVHF_3LTR	+
Homo_sapiens_8	114241603	114242056	HERVHF_5LTR	+
Homo_sapiens_8	114243394	114243717	HERVHF_gag	+
Homo_sapiens_8	114243814	114244647	HERVHF_pro	+
Homo_sapiens_8	114244279	114245799	HERVHF_pol	+
Homo_sapiens_8	114246631	114247085	HERVHF_3LTR	+
Homo_sapiens_8	114281683	114282133	HERVHF_5LTR	+
Homo_sapiens_8	114284546	114285295	HERVHF_pro	+
Homo_sapiens_8	114284753	114286697	HERVHF_pol	+
Homo_sapiens_8	114287268	114287727	HERVHF_3LTR	+
Homo_sapiens_8	128611900	128612367	HERVHF_5LTR	-
Homo_sapiens_8	128612758	128614884	HERVHF_pol	-
Homo_sapiens_8	128614363	128615175	HERVHF_pro	-
Homo_sapiens_8	128615199	128615585	HERVHF_gag	-
Homo_sapiens_8	128617178	128617638	HERVHF_3LTR	-
Homo_sapiens_8	131740750	131741164	HERVHF_5LTR	+
Homo_sapiens_8	131742853	131743164	HERVHF_gag	+
Homo_sapiens_8	131743449	131744030	HERVHF_pro	+
Homo_sapiens_8	131743623	131745774	HERVHF_pol	+
Homo_sapiens_8	131746225	131746637	HERVHF_3LTR	+
Homo_sapiens_8	132080235	132080688	HERVHF_5LTR	-
Homo_sapiens_8	132081082	132083067	HERVHF_pol	-
Homo_sapiens_8	132083498	132083827	HERVHF_gag	-
Homo_sapiens_8	132085544	132086002	HERVHF_3LTR	-
Homo_sapiens_9	6095047	6095357	HSERVIII_5LTR	+
Homo_sapiens_9	6101427	6104024	HSERVIII_pol	+

Homo_sapiens_9	6107815	6108127	HSERVIII_3LTR	+
Homo_sapiens_9	12948345	12948827	HERVHF_5LTR	+
Homo_sapiens_9	12950832	12951653	HERVHF_pro	+
Homo_sapiens_9	12951135	12953200	HERVHF_pol	+
Homo_sapiens_9	12953648	12954130	HERVHF_3LTR	+
Homo_sapiens_9	22658612	22658930	HERVHF_5LTR	-
Homo_sapiens_9	22659379	22661576	HERVHF_pol	-
Homo_sapiens_9	22661019	22661723	HERVHF_pro	-
Homo_sapiens_9	22663746	22664056	HERVHF_3LTR	-
Homo_sapiens_9	75351143	75351558	HERVHF_5LTR	-
Homo_sapiens_9	75351790	75352304	HERVHF_env	-
Homo_sapiens_9	75353426	75354751	HERVHF_pol	-
Homo_sapiens_9	75354209	75354994	HERVHF_pro	-
Homo_sapiens_9	75355041	75355397	HERVHF_gag	-
Homo_sapiens_9	75357173	75357576	HERVHF_3LTR	-
Homo_sapiens_9	78180694	78181106	HERVHF_5LTR	+
Homo_sapiens_9	78183315	78184118	HERVHF_pro	+
Homo_sapiens_9	78183576	78185683	HERVHF_pol	+
Homo_sapiens_9	78186409	78186822	HERVHF_3LTR	+
Homo_sapiens_9	80137297	80137640	HERVHF_5LTR	+
Homo_sapiens_9	80139559	80139885	HERVHF_gag	+
Homo_sapiens_9	80140314	80142260	HERVHF_pol	+
Homo_sapiens_9	80142712	80143055	HERVHF_3LTR	+
Homo_sapiens_9	82701562	82702004	HERVHF_5LTR	-
Homo_sapiens_9	82702400	82703783	HERVHF_pol	-
Homo_sapiens_9	82703148	82703894	HERVHF_pro	-
Homo_sapiens_9	82703981	82704388	HERVHF_gag	-
Homo_sapiens_9	82705845	82706287	HERVHF_3LTR	-
Homo_sapiens_9	82883732	82884185	HERVHF_5LTR	+
Homo_sapiens_9	82886223	82887056	HERVHF_pro	+
Homo_sapiens_9	82886514	82888590	HERVHF_pol	+
Homo_sapiens_9	82889038	82889491	HERVHF_3LTR	+
Homo_sapiens_9	85458329	85458782	HERVHF_5LTR	+
Homo_sapiens_9	85460704	85461087	HERVHF_gag	+
Homo_sapiens_9	85461120	85461926	HERVHF_pro	+
Homo_sapiens_9	85461315	85463744	HERVHF_pol	+
Homo_sapiens_9	85465130	85466268	HERVHF_env	+
Homo_sapiens_9	85466509	85466955	HERVHF_3LTR	+
Homo_sapiens_9	93347586	93347887	HERVHF_5LTR	+
Homo_sapiens_9	93349762	93350079	HERVHF_gag	+
Homo_sapiens_9	93350086	93350916	HERVHF_pro	+
Homo_sapiens_9	93350374	93352298	HERVHF_pol	+
Homo_sapiens_9	93352520	93352833	HERVHF_env	+
Homo_sapiens_9	93352901	93353212	HERVHF_3LTR	+

Homo_sapiens_9	94539872	94540300	HERVHF_5LTR	+
Homo_sapiens_9	94541972	94542271	HERVHF_gag	+
Homo_sapiens_9	94542717	94544296	HERVHF_pol	+
Homo_sapiens_9	94545091	94545512	HERVHF_3LTR	+
Homo_sapiens_9	99669781	99670236	HERVHF_5LTR	+
Homo_sapiens_9	99672085	99672528	HERVHF_gag	+
Homo_sapiens_9	99672639	99673226	HERVHF_pro	+
Homo_sapiens_9	99672836	99675041	HERVHF_pol	+
Homo_sapiens_9	99675451	99675904	HERVHF_3LTR	+
Homo_sapiens_9	100140183	100140620	HERVHF_5LTR	-
Homo_sapiens_9	100141584	100143661	HERVHF_pol	-
Homo_sapiens_9	100143990	100144394	HERVHF_gag	-
Homo_sapiens_9	100146114	100146555	HERVHF_3LTR	-
Homo_sapiens_9	115174661	115175062	HERVHF_5LTR	-
Homo_sapiens_9	115175687	115177590	HERVHF_pol	-
Homo_sapiens_9	115177069	115177890	HERVHF_pro	-
Homo_sapiens_9	115179935	115180342	HERVHF_3LTR	-
Homo_sapiens_9	115473181	115473637	HERVHF_5LTR	+
Homo_sapiens_9	115475420	115475800	HERVHF_gag	+
Homo_sapiens_9	115476023	115476676	HERVHF_pro	+
Homo_sapiens_9	115476248	115478150	HERVHF_pol	+
Homo_sapiens_9	115478470	115478923	HERVHF_3LTR	+
Homo_sapiens_9	115839176	115839628	HERVHF_5LTR	-
Homo_sapiens_9	115840035	115842170	HERVHF_pol	-
Homo_sapiens_9	115841628	115842458	HERVHF_pro	-
Homo_sapiens_9	115842465	115842794	HERVHF_gag	-
Homo_sapiens_9	115844477	115844929	HERVHF_3LTR	-
Homo_sapiens_9	121790001	121790452	HERVHF_5LTR	+
Homo_sapiens_9	121792163	121792696	HERVHF_gag	+
Homo_sapiens_9	121792802	121793656	HERVHF_pro	+
Homo_sapiens_9	121792997	121794402	HERVHF_pol	+
Homo_sapiens_9	121795640	121796155	HERVHF_env	+
Homo_sapiens_9	121796319	121796773	HERVHF_3LTR	+
Homo_sapiens_X	16043840	16044422	HERVHF_5LTR	-
Homo_sapiens_X	16045580	16047522	HERVHF_pol	-
Homo_sapiens_X	16047133	16047696	HERVHF_pro	-
Homo_sapiens_X	16050413	16050985	HERVHF_3LTR	-
Homo_sapiens_X	69697081	69697422	HERVHF_5LTR	-
Homo_sapiens_X	69697717	69698226	HERVHF_env	-
Homo_sapiens_X	69698607	69700744	HERVHF_pol	-
Homo_sapiens_X	69701629	69702009	HERVHF_gag	-
Homo_sapiens_X	69703482	69703828	HERVHF_3LTR	-
Homo_sapiens_X	71264372	71264714	HERVHF_5LTR	+
Homo_sapiens_X	71266244	71266657	HERVHF_gag	+

Homo_sapiens_X	71266704	71267651	HERVHF_pro	+
Homo_sapiens_X	71267253	71269614	HERVHF_pol	+
Homo_sapiens_X	71271762	71272124	HERVHF_env	+
Homo_sapiens_X	71272292	71272628	HERVHF_3LTR	+
Homo_sapiens_X	87681680	87682131	HERVHF_5LTR	+
Homo_sapiens_X	87684041	87684364	HERVHF_gag	+
Homo_sapiens_X	87684371	87685201	HERVHF_pro	+
Homo_sapiens_X	87684659	87686461	HERVHF_pol	+
Homo_sapiens_X	87687194	87687646	HERVHF_3LTR	+
Homo_sapiens_X	90773314	90773760	HERVHF_5LTR	-
Homo_sapiens_X	90774538	90776292	HERVHF_pol	-
Homo_sapiens_X	90775783	90776499	HERVHF_pro	-
Homo_sapiens_X	90776607	90776906	HERVHF_gag	-
Homo_sapiens_X	90778707	90779155	HERVHF_3LTR	-
Homo_sapiens_X	92273087	92273521	HERVHF_5LTR	-
Homo_sapiens_X	92273918	92276034	HERVHF_pol	-
Homo_sapiens_X	92275513	92276319	HERVHF_pro	-
Homo_sapiens_X	92278361	92278800	HERVHF_3LTR	-
Homo_sapiens_X	92824428	92824882	HERVHF_5LTR	+
Homo_sapiens_X	92826553	92826966	HERVHF_gag	+
Homo_sapiens_X	92827175	92827822	HERVHF_pro	+
Homo_sapiens_X	92827220	92828521	HERVHF_pol	+
Homo_sapiens_X	92828895	92829347	HERVHF_3LTR	+
Homo_sapiens_X	94698818	94699270	HERVHF_5LTR	-
Homo_sapiens_X	94699849	94701688	HERVHF_pol	-
Homo_sapiens_X	94701248	94701832	HERVHF_pro	-
Homo_sapiens_X	94704637	94705089	HERVHF_3LTR	-
Homo_sapiens_X	94819302	94819615	HSERVIII_5LTR	-
Homo_sapiens_X	94820732	94823586	HSERVIII_pol	-
Homo_sapiens_X	94828209	94828529	HSERVIII_3LTR	-
Homo_sapiens_X	98524240	98524704	HERVHF_5LTR	-
Homo_sapiens_X	98525292	98527235	HERVHF_pol	-
Homo_sapiens_X	98526693	98527523	HERVHF_pro	-
Homo_sapiens_X	98527530	98527850	HERVHF_gag	-
Homo_sapiens_X	98529618	98530078	HERVHF_3LTR	-
Homo_sapiens_X	109178342	109178814	HERVHF_5LTR	+
Homo_sapiens_X	109181321	109183264	HERVHF_pol	+
Homo_sapiens_X	109183707	109184176	HERVHF_3LTR	+
Homo_sapiens_X	111543806	111544260	HERVHF_5LTR	+
Homo_sapiens_X	111546072	111546392	HERVHF_gag	+
Homo_sapiens_X	111546382	111547230	HERVHF_pro	+
Homo_sapiens_X	111546688	111548769	HERVHF_pol	+
Homo_sapiens_X	111549221	111549675	HERVHF_3LTR	+
Homo_sapiens_X	112530380	112530836	HERVHF_5LTR	+

Homo_sapiens_X	112533516	112535451	HERVHF_pol	+
Homo_sapiens_X	112535906	112536360	HERVHF_3LTR	+
Homo_sapiens_X	114601269	114601663	HERVHF_5LTR	-
Homo_sapiens_X	114602179	114604363	HERVHF_pol	-
Homo_sapiens_X	114603725	114604477	HERVHF_pro	-
Homo_sapiens_X	114604561	114604974	HERVHF_gag	-
Homo_sapiens_X	114606792	114607167	HERVHF_3LTR	-
Homo_sapiens_X	122222003	122222455	HERVHF_5LTR	+
Homo_sapiens_X	122224097	122224477	HERVHF_gag	+
Homo_sapiens_X	122224510	122225313	HERVHF_pro	+
Homo_sapiens_X	122224705	122226744	HERVHF_pol	+
Homo_sapiens_X	122227333	122227787	HERVHF_3LTR	+
Homo_sapiens_X	142423374	142423787	HERVHF_5LTR	-
Homo_sapiens_X	142424523	142426496	HERVHF_pol	-
Homo_sapiens_X	142425975	142426775	HERVHF_pro	-
Homo_sapiens_X	142426911	142427264	HERVHF_gag	-
Homo_sapiens_X	142428547	142428961	HERVHF_3LTR	-
Homo_sapiens_X	149073591	149073909	HSERVIII_5LTR	+
Homo_sapiens_X	149075763	149076530	HSERVIII_pol	+
Homo_sapiens_X	149079939	149080259	HSERVIII_3LTR	+
Homo_sapiens_X	149749529	149750009	HSERVIII_5LTR	-
Homo_sapiens_X	149751046	149751830	HSERVIII_pol	-
Homo_sapiens_X	149753983	149754464	HSERVIII_3LTR	-
Homo_sapiens_Y	4355391	4355841	HERVHF_5LTR	-
Homo_sapiens_Y	4356610	4358372	HERVHF_pol	-
Homo_sapiens_Y	4357809	4358462	HERVHF_pro	-
Homo_sapiens_Y	4358686	4358994	HERVHF_gag	-
Homo_sapiens_Y	4360781	4361230	HERVHF_3LTR	-
Homo_sapiens_Y	5324785	5325239	HERVHF_5LTR	-
Homo_sapiens_Y	5325640	5327757	HERVHF_pol	-
Homo_sapiens_Y	5327215	5327853	HERVHF_pro	-
Homo_sapiens_Y	5328124	5328591	HERVHF_gag	-
Homo_sapiens_Y	5329976	5330428	HERVHF_3LTR	-
Homo_sapiens_Y	5941111	5941562	HERVHF_5LTR	+
Homo_sapiens_Y	5943321	5943656	HERVHF_gag	+
Homo_sapiens_Y	5943693	5944472	HERVHF_pro	+
Homo_sapiens_Y	5943951	5945213	HERVHF_pol	+
Homo_sapiens_Y	5945587	5946038	HERVHF_3LTR	+
Hoolock_hoolock_ctg1	10341805	10342247	Unknown_HERV_5LTR	-
Hoolock_hoolock_ctg1	10342812	10344544	Unknown_HERV_pol	-
Hoolock_hoolock_ctg1	10344866	10345165	Unknown_HERV_gag	-
Hoolock_hoolock_ctg1	10346729	10347169	Unknown_HERV_3LTR	-
Hoolock_hoolock_ctg1	50709621	50710034	HERVHF_5LTR	+
Hoolock_hoolock_ctg1	50711555	50711878	HERVHF_gag	+

Hoolock_hoolock_ctg1	50712064	50712819	HERVHF_pro	+
Hoolock_hoolock_ctg1	50712469	50714426	HERVHF_pol	+
Hoolock_hoolock_ctg1	50714858	50715274	HERVHF_3LTR	+
Hoolock_hoolock_ctg14	33704346	33704773	HERVHF_5LTR	+
Hoolock_hoolock_ctg14	33706804	33707625	HERVHF_pro	+
Hoolock_hoolock_ctg14	33707224	33709113	HERVHF_pol	+
Hoolock_hoolock_ctg14	33709544	33709958	HERVHF_3LTR	+
Hoolock_hoolock_ctg15	4271648	4271952	Unknown_HERV_5LTR	+
Hoolock_hoolock_ctg15	4276107	4278233	Unknown_HERV_pol	+
Hoolock_hoolock_ctg15	4281939	4282243	Unknown_HERV_3LTR	+
Hoolock_hoolock_ctg15	29232753	29233149	HERVIPADP_5LTR	-
Hoolock_hoolock_ctg15	29233696	29234016	HERVIPADP_env	-
Hoolock_hoolock_ctg15	29235123	29238328	HERVIPADP_pol	-
Hoolock_hoolock_ctg15	29241048	29241421	HERVIPADP_3LTR	-
Hoolock_hoolock_ctg15	37505496	37505919	HERVIPADP_5LTR	+
Hoolock_hoolock_ctg15	37507593	37508000	HERVIPADP_gag	+
Hoolock_hoolock_ctg15	37508423	37511031	HERVIPADP_pol	+
Hoolock_hoolock_ctg15	37513225	37513818	HERVIPADP_env	+
Hoolock_hoolock_ctg15	37513902	37514321	HERVIPADP_3LTR	+
Hoolock_hoolock_ctg150	537827	538222	HERVHF_5LTR	+
Hoolock_hoolock_ctg150	539873	540202	HERVHF_gag	+
Hoolock_hoolock_ctg150	540322	541164	HERVHF_pro	+
Hoolock_hoolock_ctg150	540789	542258	HERVHF_pol	+
Hoolock_hoolock_ctg150	543059	543459	HERVHF_3LTR	+
Hoolock_hoolock_ctg16	5375495	5375941	HERVIPADP_5LTR	+
Hoolock_hoolock_ctg16	5377561	5379294	HERVIPADP_pol	+
Hoolock_hoolock_ctg16	5381870	5382307	HERVIPADP_env	+
Hoolock_hoolock_ctg16	5382417	5382863	HERVIPADP_3LTR	+
Hoolock_hoolock_ctg16	21920015	21920518	HERVIPADP_5LTR	+
Hoolock_hoolock_ctg16	21922067	21922447	HERVIPADP_gag	+
Hoolock_hoolock_ctg16	21923383	21925878	HERVIPADP_pol	+
Hoolock_hoolock_ctg16	21927769	21928248	HERVIPADP_env	+
Hoolock_hoolock_ctg16	21928269	21928761	HERVIPADP_3LTR	+
Hoolock_hoolock_ctg20	6657273	6657722	HERVK_5LTR	+
Hoolock_hoolock_ctg20	6659104	6660108	HERVK_pro	+
Hoolock_hoolock_ctg20	6661254	6663057	HERVK_pol	+
Hoolock_hoolock_ctg20	6663629	6663962	HERVK_env	+
Hoolock_hoolock_ctg20	6664112	6664565	HERVK_3LTR	+
Hoolock_hoolock_ctg22	17835722	17836148	HERVIPADP_5LTR	+
Hoolock_hoolock_ctg22	17838480	17842482	HERVIPADP_pol	+
Hoolock_hoolock_ctg22	17844569	17844982	HERVIPADP_env	+
Hoolock_hoolock_ctg22	17845095	17845540	HERVIPADP_3LTR	+
Hoolock_hoolock_ctg24	23640291	23640704	HERVHF_5LTR	-
Hoolock_hoolock_ctg24	23641295	23643100	HERVHF_pol	-

Hoolock_hoolock_ctg24	23643123	23643521	HERVHF_pro	-
Hoolock_hoolock_ctg24	23643618	23643917	HERVHF_gag	-
Hoolock_hoolock_ctg24	23645553	23645967	HERVHF_3LTR	-
Hoolock_hoolock_ctg3	60881913	60882364	HERVHF_5LTR	-
Hoolock_hoolock_ctg3	60882450	60883309	HERVHF_env	-
Hoolock_hoolock_ctg3	60884633	60886466	HERVHF_pol	-
Hoolock_hoolock_ctg3	60886128	60886949	HERVHF_pro	-
Hoolock_hoolock_ctg3	60887003	60887383	HERVHF_gag	-
Hoolock_hoolock_ctg3	60888639	60889095	HERVHF_3LTR	-
Hoolock_hoolock_ctg317	103857	104239	HSERVIII_5LTR	-
Hoolock_hoolock_ctg317	104384	104965	HSERVIII_pol	-
Hoolock_hoolock_ctg317	106159	106539	HSERVIII_3LTR	-
Hoolock_hoolock_ctg38	4317241	4317701	HERVIPADP_5LTR	+
Hoolock_hoolock_ctg38	4319880	4322528	HERVIPADP_pol	+
Hoolock_hoolock_ctg38	4324284	4324592	HERVIPADP_env	+
Hoolock_hoolock_ctg38	4325240	4325697	HERVIPADP_3LTR	+
Hoolock_hoolock_ctg4	22621914	22622269	HSERVIII_5LTR	-
Hoolock_hoolock_ctg4	22622918	22625438	HSERVIII_pol	-
Hoolock_hoolock_ctg4	22627593	22627943	HSERVIII_3LTR	-
Hoolock_hoolock_ctg4	44805229	44805646	HERVHF_5LTR	+
Hoolock_hoolock_ctg4	44806130	44808928	HERVHF_pol	+
Hoolock_hoolock_ctg4	44809110	44809508	HERVHF_env	+
Hoolock_hoolock_ctg4	44810213	44810627	HERVHF_3LTR	+
Hoolock_hoolock_ctg4	66853672	66854081	HERVHF_5LTR	+
Hoolock_hoolock_ctg4	66856680	66857261	HERVHF_pro	+
Hoolock_hoolock_ctg4	66856854	66858672	HERVHF_pol	+
Hoolock_hoolock_ctg4	66859258	66859678	HERVHF_3LTR	+
Hoolock_hoolock_ctg40	10723619	10723931	HERVHF_5LTR	+
Hoolock_hoolock_ctg40	10724984	10725838	HERVHF_pol	+
Hoolock_hoolock_ctg40	10727833	10728273	HERVHF_env	+
Hoolock_hoolock_ctg40	10728579	10728891	HERVHF_3LTR	+
Hoolock_hoolock_ctg45	14796138	14796476	HERVHF_5LTR	-
Hoolock_hoolock_ctg45	14797920	14799208	HERVHF_pol	-
Hoolock_hoolock_ctg45	14798648	14799448	HERVHF_pro	-
Hoolock_hoolock_ctg45	14799545	14800039	HERVHF_gag	-
Hoolock_hoolock_ctg45	14801542	14801865	HERVHF_3LTR	-
Hoolock_hoolock_ctg64	4730052	4730380	Unknown_HERV_5LTR	-
Hoolock_hoolock_ctg64	4732169	4733372	Unknown_HERV_pol	-
Hoolock_hoolock_ctg64	4733492	4733824	Unknown_HERV_pro	-
Hoolock_hoolock_ctg64	4733919	4734260	Unknown_HERV_gag	-
Hoolock_hoolock_ctg64	4735872	4736190	Unknown_HERV_3LTR	-
Hoolock_hoolock_ctg75	2738005	2738365	HSERVIII_5LTR	+
Hoolock_hoolock_ctg75	2747283	2748600	HSERVIII_pol	+
Hoolock_hoolock_ctg75	2752651	2752995	HSERVIII_3LTR	+

Hoolock_hoolock_ctg76	4784530	4785017	HERVK_5LTR	-
Hoolock_hoolock_ctg76	4786255	4787157	HERVK_pol	-
Hoolock_hoolock_ctg76	4786902	4787843	HERVK_pro	-
Hoolock_hoolock_ctg76	4788340	4788807	HERVK_gag	-
Hoolock_hoolock_ctg76	4789251	4789735	HERVK_3LTR	-
Hoolock_hoolock_ctg78	7191727	7192137	HERVHF_5LTR	-
Hoolock_hoolock_ctg78	7192549	7194507	HERVHF_pol	-
Hoolock_hoolock_ctg78	7194999	7195322	HERVHF_gag	-
Hoolock_hoolock_ctg78	7196972	7197384	HERVHF_3LTR	-
Hoolock_hoolock_ctg80	3413298	3413729	HERVHF_5LTR	-
Hoolock_hoolock_ctg80	3414184	3415963	HERVHF_pol	-
Hoolock_hoolock_ctg80	3415591	3416349	HERVHF_pro	-
Hoolock_hoolock_ctg80	3416567	3416950	HERVHF_gag	-
Hoolock_hoolock_ctg80	3418549	3418973	HERVHF_3LTR	-
Hoolock_hoolock_ctg83	16891941	16892353	HERVHF_5LTR	+
Hoolock_hoolock_ctg83	16894354	16894866	HERVHF_pro	+
Hoolock_hoolock_ctg83	16894821	16896325	HERVHF_pol	+
Hoolock_hoolock_ctg83	16897143	16897554	HERVHF_3LTR	+
Hoolock_hoolock_ctg86	6360412	6360805	HERVIPADP_5LTR	-
Hoolock_hoolock_ctg86	6361062	6361721	HERVIPADP_env	-
Hoolock_hoolock_ctg86	6362894	6366003	HERVIPADP_pol	-
Hoolock_hoolock_ctg86	6368978	6369378	HERVIPADP_3LTR	-
Hoolock_hoolock_ctg94	7292051	7292362	HERVHF_5LTR	-
Hoolock_hoolock_ctg94	7294637	7295335	HERVHF_pol	-
Hoolock_hoolock_ctg94	7295730	7296335	HERVHF_gag	-
Hoolock_hoolock_ctg94	7297042	7297354	HERVHF_3LTR	-
Hylobates_lar_ContigUN	22320383	22320721	HERVHF_5LTR	+
Hylobates_lar_ContigUN	22322151	22322480	HERVHF_gag	+
Hylobates_lar_ContigUN	22322969	22324627	HERVHF_pol	+
Hylobates_lar_ContigUN	22325812	22326186	HERVHF_env	+
Hylobates_lar_ContigUN	22326901	22327248	HERVHF_3LTR	+
Hylobates_lar_LG01	18360753	18361129	HERVHF_5LTR	-
Hylobates_lar_LG01	18361562	18363526	HERVHF_pol	-
Hylobates_lar_LG01	18363119	18363700	HERVHF_pro	-
Hylobates_lar_LG01	18366061	18366427	HERVHF_3LTR	-
Hylobates_lar_LG01	19436675	19437067	HERVHF_5LTR	+
Hylobates_lar_LG01	19438679	19439020	HERVHF_gag	+
Hylobates_lar_LG01	19439128	19439952	HERVHF_pro	+
Hylobates_lar_LG01	19439419	19442930	HERVHF_pol	+
Hylobates_lar_LG01	19443389	19443787	HERVHF_3LTR	+
Hylobates_lar_LG01	34388720	34389174	HERVHF_5LTR	-
Hylobates_lar_LG01	34390190	34391933	HERVHF_pol	-
Hylobates_lar_LG01	34392142	34392555	HERVHF_pro	-
Hylobates_lar_LG01	34392545	34392868	HERVHF_gag	-

Hylobates_lar_LG01	34394862	34395317	HERVHF_3LTR	-
Hylobates_lar_LG01	57005269	57005578	HERVHF_5LTR	-
Hylobates_lar_LG01	57006257	57008302	HERVHF_pol	-
Hylobates_lar_LG01	57007760	57008539	HERVHF_pro	-
Hylobates_lar_LG01	57008668	57009030	HERVHF_gag	-
Hylobates_lar_LG01	57010485	57010810	HERVHF_3LTR	-
Hylobates_lar_LG01	79013517	79013946	HERVHF_5LTR	-
Hylobates_lar_LG01	79014567	79016466	HERVHF_pol	-
Hylobates_lar_LG01	79015870	79016754	HERVHF_pro	-
Hylobates_lar_LG01	79016761	79017084	HERVHF_gag	-
Hylobates_lar_LG01	79018792	79019222	HERVHF_3LTR	-
Hylobates_lar_LG01	88551318	88551758	HERVHF_5LTR	+
Hylobates_lar_LG01	88554055	88555984	HERVHF_pol	+
Hylobates_lar_LG01	88558604	88559045	HERVHF_3LTR	+
Hylobates_lar_LG01	107779924	107780381	HERVHF_5LTR	+
Hylobates_lar_LG01	107781820	107782119	HERVHF_gag	+
Hylobates_lar_LG01	107782176	107782691	HERVHF_pro	+
Hylobates_lar_LG01	107782642	107785364	HERVHF_pol	+
Hylobates_lar_LG01	107785773	107786235	HERVHF_3LTR	+
Hylobates_lar_LG01	158668585	158668997	HERVHF_5LTR	-
Hylobates_lar_LG01	158670054	158672141	HERVHF_pol	-
Hylobates_lar_LG01	158671566	158672246	HERVHF_pro	-
Hylobates_lar_LG01	158674472	158674898	HERVHF_3LTR	-
Hylobates_lar_LG01	200427659	200428001	HERVHF_5LTR	+
Hylobates_lar_LG01	200429293	200429676	HERVHF_gag	+
Hylobates_lar_LG01	200430299	200431003	HERVHF_pro	+
Hylobates_lar_LG01	200430422	200433957	HERVHF_pol	+
Hylobates_lar_LG01	200434748	200435872	HERVHF_env	+
Hylobates_lar_LG01	200436085	200436438	HERVHF_3LTR	+
Hylobates_lar_LG01	214541123	214541584	HERVHF_5LTR	+
Hylobates_lar_LG01	214543353	214544132	HERVHF_pro	+
Hylobates_lar_LG01	214543590	214545859	HERVHF_pol	+
Hylobates_lar_LG01	214546531	214546999	HERVHF_3LTR	+
Hylobates_lar_LG01	215486824	215487245	HERVHF_5LTR	-
Hylobates_lar_LG01	215487626	215489613	HERVHF_pol	-
Hylobates_lar_LG01	215492137	215492578	HERVHF_3LTR	-
Hylobates_lar_LG01	218208061	218208504	HERVHF_5LTR	+
Hylobates_lar_LG01	218210090	218210473	HERVHF_gag	+
Hylobates_lar_LG01	218211668	218212219	HERVHF_pro	+
Hylobates_lar_LG01	218211707	218213628	HERVHF_pol	+
Hylobates_lar_LG01	218214198	218214645	HERVHF_3LTR	+
Hylobates_lar_LG01	219604541	219604953	HERVHF_5LTR	+
Hylobates_lar_LG01	219606906	219607229	HERVHF_gag	+
Hylobates_lar_LG01	219607558	219608154	HERVHF_pro	+

Hylobates_lar_LG01	219607564	219609409	HERVHF_pol	+
Hylobates_lar_LG01	219610142	219610558	HERVHF_3LTR	+
Hylobates_lar_LG01	244298782	244299162	HERVHF_5LTR	-
Hylobates_lar_LG01	244299409	244300002	HERVHF_env	-
Hylobates_lar_LG01	244301102	244302429	HERVHF_pol	-
Hylobates_lar_LG01	244302782	244303258	HERVHF_gag	-
Hylobates_lar_LG01	244304632	244305011	HERVHF_3LTR	-
Hylobates_lar_LG01	247602658	247602979	HERVK_5LTR	-
Hylobates_lar_LG01	247603235	247603546	HERVK_env	-
Hylobates_lar_LG01	247604075	247607241	HERVK_pol	-
Hylobates_lar_LG01	247607318	247607937	HERVK_pro	-
Hylobates_lar_LG01	247608612	247608913	HERVK_3LTR	-
Hylobates_lar_LG02	27595104	27595545	HERVHF_5LTR	+
Hylobates_lar_LG02	27595808	27597612	HERVHF_pol	+
Hylobates_lar_LG02	27598466	27598852	HERVHF_env	+
Hylobates_lar_LG02	27599080	27599515	HERVHF_3LTR	+
Hylobates_lar_LG02	51473116	51473517	HERVHF_5LTR	-
Hylobates_lar_LG02	51474083	51475906	HERVHF_pol	-
Hylobates_lar_LG02	51475499	51476083	HERVHF_pro	-
Hylobates_lar_LG02	51478207	51478608	HERVHF_3LTR	-
Hylobates_lar_LG02	63783625	63784032	HERVHF_5LTR	+
Hylobates_lar_LG02	63786263	63786724	HERVHF_pro	+
Hylobates_lar_LG02	63786594	63788700	HERVHF_pol	+
Hylobates_lar_LG02	63789148	63789554	HERVHF_3LTR	+
Hylobates_lar_LG02	71306090	71306550	HERVHF_5LTR	-
Hylobates_lar_LG02	71306568	71306929	HERVHF_env	-
Hylobates_lar_LG02	71307169	71308953	HERVHF_pol	-
Hylobates_lar_LG02	71309358	71309678	HERVHF_gag	-
Hylobates_lar_LG02	71311312	71311772	HERVHF_3LTR	-
Hylobates_lar_LG02	85866533	85866947	HERVHF_5LTR	+
Hylobates_lar_LG02	85868685	85869005	HERVHF_gag	+
Hylobates_lar_LG02	85869041	85869856	HERVHF_pro	+
Hylobates_lar_LG02	85869431	85871648	HERVHF_pol	+
Hylobates_lar_LG02	85872594	85873012	HERVHF_3LTR	+
Hylobates_lar_LG02	145693977	145694430	HERVHF_5LTR	+
Hylobates_lar_LG02	145695929	145696426	HERVHF_gag	+
Hylobates_lar_LG02	145696867	145698674	HERVHF_pol	+
Hylobates_lar_LG02	145699266	145699719	HERVHF_3LTR	+
Hylobates_lar_LG02	177856038	177856774	HERVHF_5LTR	+
Hylobates_lar_LG02	177857753	177858166	HERVHF_gag	+
Hylobates_lar_LG02	177858219	177858995	HERVHF_pro	+
Hylobates_lar_LG02	177858453	177859169	HERVHF_pol	+
Hylobates_lar_LG02	177859607	177860344	HERVHF_3LTR	+
Hylobates_lar_LG03	26827389	26827809	HERVIPADP_5LTR	-

Hylobates_lar_LG03	26827916	26828371	HERVIPADP_env	-
Hylobates_lar_LG03	26829862	26833128	HERVIPADP_pol	-
Hylobates_lar_LG03	26833653	26834054	HERVIPADP_gag	-
Hylobates_lar_LG03	26835954	26836377	HERVIPADP_3LTR	-
Hylobates_lar_LG03	28198303	28198677	HERVIPADP_5LTR	+
Hylobates_lar_LG03	28201395	28204574	HERVIPADP_pol	+
Hylobates_lar_LG03	28205450	28205941	HERVIPADP_env	+
Hylobates_lar_LG03	28206598	28206994	HERVIPADP_3LTR	+
Hylobates_lar_LG03	44751704	44752109	HERVHF_5LTR	-
Hylobates_lar_LG03	44752339	44753057	HERVHF_env	-
Hylobates_lar_LG03	44754240	44755440	HERVHF_pol	-
Hylobates_lar_LG03	44754919	44755773	HERVHF_pro	-
Hylobates_lar_LG03	44755891	44756217	HERVHF_gag	-
Hylobates_lar_LG03	44757706	44758110	HERVHF_3LTR	-
Hylobates_lar_LG03	138127328	138127743	HERVHF_5LTR	+
Hylobates_lar_LG03	138128551	138128970	HERVHF_gag	+
Hylobates_lar_LG03	138129461	138130213	HERVHF_pro	+
Hylobates_lar_LG03	138129701	138131633	HERVHF_pol	+
Hylobates_lar_LG03	138132209	138132623	HERVHF_3LTR	+
Hylobates_lar_LG03	155209346	155209757	HERVHF_5LTR	-
Hylobates_lar_LG03	155210808	155213992	HERVHF_pol	-
Hylobates_lar_LG03	155214299	155214739	HERVHF_gag	-
Hylobates_lar_LG03	155216392	155216804	HERVHF_3LTR	-
Hylobates_lar_LG03	158489220	158489633	HERVHF_5LTR	-
Hylobates_lar_LG03	158490277	158492190	HERVHF_pol	-
Hylobates_lar_LG03	158491615	158492385	HERVHF_pro	-
Hylobates_lar_LG03	158492462	158492974	HERVHF_gag	-
Hylobates_lar_LG03	158494538	158494954	HERVHF_3LTR	-
Hylobates_lar_LG03	163302661	163303076	HERVHF_5LTR	-
Hylobates_lar_LG03	163303662	163305432	HERVHF_pol	-
Hylobates_lar_LG03	163307990	163308403	HERVHF_3LTR	-
Hylobates_lar_LG03	163559167	163559576	HERVHF_5LTR	-
Hylobates_lar_LG03	163560162	163561877	HERVHF_pol	-
Hylobates_lar_LG03	163561509	163562390	HERVHF_pro	-
Hylobates_lar_LG03	163562432	163562806	HERVHF_gag	-
Hylobates_lar_LG03	163564418	163564832	HERVHF_3LTR	-
Hylobates_lar_LG03	176837019	176837428	HERVHF_5LTR	-
Hylobates_lar_LG03	176837937	176840018	HERVHF_pol	-
Hylobates_lar_LG03	176839380	176840132	HERVHF_pro	-
Hylobates_lar_LG03	176840211	176840534	HERVHF_gag	-
Hylobates_lar_LG03	176842252	176842649	HERVHF_3LTR	-
Hylobates_lar_LG04	6326972	6327523	HERVHF_5LTR	+
Hylobates_lar_LG04	6332529	6334620	HERVHF_pol	+
Hylobates_lar_LG04	6339607	6340170	HERVHF_3LTR	+

Hylobates_lar_LG04	6384778	6385251	HERVHF_5LTR	+
Hylobates_lar_LG04	6386994	6387494	HERVHF_gag	+
Hylobates_lar_LG04	6387852	6388373	HERVHF_pro	+
Hylobates_lar_LG04	6387966	6389921	HERVHF_pol	+
Hylobates_lar_LG04	6390307	6390788	HERVHF_3LTR	+
Hylobates_lar_LG04	13565554	13565967	HERVHF_5LTR	-
Hylobates_lar_LG04	13566421	13569952	HERVHF_pol	-
Hylobates_lar_LG04	13569545	13570126	HERVHF_pro	-
Hylobates_lar_LG04	13570406	13570813	HERVHF_gag	-
Hylobates_lar_LG04	13572328	13572743	HERVHF_3LTR	-
Hylobates_lar_LG04	26177053	26177466	HERVHF_5LTR	-
Hylobates_lar_LG04	26178057	26179875	HERVHF_pol	-
Hylobates_lar_LG04	26179695	26180189	HERVHF_pro	-
Hylobates_lar_LG04	26180407	26180706	HERVHF_gag	-
Hylobates_lar_LG04	26182427	26182841	HERVHF_3LTR	-
Hylobates_lar_LG04	27939100	27939531	HERVHF_5LTR	+
Hylobates_lar_LG04	27941114	27941419	HERVHF_gag	+
Hylobates_lar_LG04	27942147	27943677	HERVHF_pol	+
Hylobates_lar_LG04	27944335	27944771	HERVHF_3LTR	+
Hylobates_lar_LG04	111353628	111354041	HERVHF_5LTR	+
Hylobates_lar_LG04	111355672	111356097	HERVHF_gag	+
Hylobates_lar_LG04	111356506	111358787	HERVHF_pol	+
Hylobates_lar_LG04	111359203	111359614	HERVHF_3LTR	+
Hylobates_lar_LG04	129164563	129164988	HERVHF_5LTR	+
Hylobates_lar_LG04	129165874	129167117	HERVHF_gag	+
Hylobates_lar_LG04	129167175	129167573	HERVHF_pro	+
Hylobates_lar_LG04	129167644	129168579	HERVHF_pol	+
Hylobates_lar_LG04	129169021	129169449	HERVHF_3LTR	+
Hylobates_lar_LG04	133443945	133444289	HERVHF_5LTR	-
Hylobates_lar_LG04	133444796	133446739	HERVHF_pol	-
Hylobates_lar_LG04	133447163	133447585	HERVHF_gag	-
Hylobates_lar_LG04	133449196	133449547	HERVHF_3LTR	-
Hylobates_lar_LG04	137269096	137269444	HERVHF_5LTR	-
Hylobates_lar_LG04	137269854	137272016	HERVHF_pol	-
Hylobates_lar_LG04	137272300	137272689	HERVHF_gag	-
Hylobates_lar_LG04	137274236	137274588	HERVHF_3LTR	-
Hylobates_lar_LG04	173411111	173411515	HERVHF_5LTR	+
Hylobates_lar_LG04	173413852	173414394	HERVHF_pro	+
Hylobates_lar_LG04	173413891	173415963	HERVHF_pol	+
Hylobates_lar_LG04	173416410	173416802	HERVHF_3LTR	+
Hylobates_lar_LG05	14609709	14610162	HERVHF_5LTR	+
Hylobates_lar_LG05	14612293	14612946	HERVHF_pro	+
Hylobates_lar_LG05	14612498	14614414	HERVHF_pol	+
Hylobates_lar_LG05	14614867	14615317	HERVHF_3LTR	+

Hylobates_lar_LG05	15801625	15802017	HERVHF_5LTR	-
Hylobates_lar_LG05	15803130	15804722	HERVHF_pol	-
Hylobates_lar_LG05	15804351	15805130	HERVHF_pro	-
Hylobates_lar_LG05	15805218	15805529	HERVHF_gag	-
Hylobates_lar_LG05	15807234	15807630	HERVHF_3LTR	-
Hylobates_lar_LG05	23752341	23752690	HERVHF_5LTR	-
Hylobates_lar_LG05	23753259	23755074	HERVHF_pol	-
Hylobates_lar_LG05	23754694	23755422	HERVHF_pro	-
Hylobates_lar_LG05	23755537	23756267	HERVHF_gag	-
Hylobates_lar_LG05	23757604	23757955	HERVHF_3LTR	-
Hylobates_lar_LG05	28288476	28288785	HERVHF_5LTR	-
Hylobates_lar_LG05	28289582	28291451	HERVHF_pol	-
Hylobates_lar_LG05	28291343	28291969	HERVHF_pro	-
Hylobates_lar_LG05	28292140	28292646	HERVHF_gag	-
Hylobates_lar_LG05	28292834	28293140	HERVHF_3LTR	-
Hylobates_lar_LG05	32613289	32613736	HERVHF_5LTR	-
Hylobates_lar_LG05	32614708	32617039	HERVHF_pol	-
Hylobates_lar_LG05	32617398	32617865	HERVHF_gag	-
Hylobates_lar_LG05	32619193	32619639	HERVHF_3LTR	-
Hylobates_lar_LG05	81465795	81466219	HERVK_5LTR	+
Hylobates_lar_LG05	81468859	81469839	HERVK_pro	+
Hylobates_lar_LG05	81469806	81472549	HERVK_pol	+
Hylobates_lar_LG05	81478941	81479357	HERVK_3LTR	+
Hylobates_lar_LG05	115275938	115276341	HERVHF_5LTR	+
Hylobates_lar_LG05	115278830	115279684	HERVHF_pro	+
Hylobates_lar_LG05	115279565	115282750	HERVHF_pol	+
Hylobates_lar_LG05	115283339	115283743	HERVHF_3LTR	+
Hylobates_lar_LG05	119559476	119559924	HERVHF_5LTR	+
Hylobates_lar_LG05	119561753	119562577	HERVHF_pro	+
Hylobates_lar_LG05	119561978	119564277	HERVHF_pol	+
Hylobates_lar_LG05	119565318	119565766	HERVHF_3LTR	+
Hylobates_lar_LG05	141886424	141886874	HERVHF_5LTR	-
Hylobates_lar_LG05	141887002	141887850	HERVHF_env	-
Hylobates_lar_LG05	141889547	141894244	HERVHF_pol	-
Hylobates_lar_LG05	141897583	141898035	HERVHF_3LTR	-
Hylobates_lar_LG06	36049931	36050527	HERVHF_5LTR	-
Hylobates_lar_LG06	36052175	36053329	HERVHF_env	-
Hylobates_lar_LG06	36054845	36056661	HERVHF_pol	-
Hylobates_lar_LG06	36057534	36058133	HERVHF_3LTR	-
Hylobates_lar_LG06	38831518	38831828	HERVHF_5LTR	+
Hylobates_lar_LG06	38833322	38833663	HERVHF_gag	+
Hylobates_lar_LG06	38834072	38837056	HERVHF_pol	+
Hylobates_lar_LG06	38837745	38838060	HERVHF_3LTR	+
Hylobates_lar_LG06	64123185	64123588	HERVHF_5LTR	-

Hylobates_lar_LG06	64124006	64126193	HERVHF_pol	-
Hylobates_lar_LG06	64126620	64126940	HERVHF_gag	-
Hylobates_lar_LG06	64128645	64129049	HERVHF_3LTR	-
Hylobates_lar_LG07	21098754	21099589	HERVHF_5LTR	+
Hylobates_lar_LG07	21100064	21102380	HERVHF_pol	+
Hylobates_lar_LG07	21103353	21104191	HERVHF_3LTR	+
Hylobates_lar_LG07	27547945	27548270	HERVHF_5LTR	+
Hylobates_lar_LG07	27550536	27551479	HERVHF_pol	+
Hylobates_lar_LG07	27552457	27553392	HERVHF_env	+
Hylobates_lar_LG07	27553688	27554027	HERVHF_3LTR	+
Hylobates_lar_LG07	46243819	46244265	HSERVIII_5LTR	+
Hylobates_lar_LG07	46247684	46248421	HSERVIII_pol	+
Hylobates_lar_LG07	46250042	46250493	HSERVIII_3LTR	+
Hylobates_lar_LG07	53204409	53204833	HERVHF_5LTR	+
Hylobates_lar_LG07	53206223	53206594	HERVHF_gag	+
Hylobates_lar_LG07	53207090	53210700	HERVHF_pol	+
Hylobates_lar_LG07	53210906	53211268	HERVHF_env	+
Hylobates_lar_LG07	53211437	53211878	HERVHF_3LTR	+
Hylobates_lar_LG07	69447739	69448136	HERVHF_5LTR	+
Hylobates_lar_LG07	69449949	69450317	HERVHF_gag	+
Hylobates_lar_LG07	69450368	69451219	HERVHF_pro	+
Hylobates_lar_LG07	69450656	69452653	HERVHF_pol	+
Hylobates_lar_LG07	69453186	69453599	HERVHF_3LTR	+
Hylobates_lar_LG07	76861595	76862098	HERVHF_5LTR	-
Hylobates_lar_LG07	76862324	76863434	HERVHF_env	-
Hylobates_lar_LG07	76864308	76867082	HERVHF_pol	-
Hylobates_lar_LG07	76867440	76867757	HERVHF_gag	-
Hylobates_lar_LG07	76869005	76869510	HERVHF_3LTR	-
Hylobates_lar_LG07	125048676	125048991	HERVHF_5LTR	+
Hylobates_lar_LG07	125050478	125050777	HERVHF_gag	+
Hylobates_lar_LG07	125050767	125051216	HERVHF_pro	+
Hylobates_lar_LG07	125051111	125053218	HERVHF_pol	+
Hylobates_lar_LG07	125053948	125054310	HERVHF_env	+
Hylobates_lar_LG07	125054536	125054854	HERVHF_3LTR	+
Hylobates_lar_LG07	130700840	130701272	HERVHF_5LTR	-
Hylobates_lar_LG07	130701680	130703777	HERVHF_pol	-
Hylobates_lar_LG07	130703235	130704008	HERVHF_pro	-
Hylobates_lar_LG07	130704142	130704465	HERVHF_gag	-
Hylobates_lar_LG07	130705980	130706410	HERVHF_3LTR	-
Hylobates_lar_LG08	11545787	11546265	HERVHF_5LTR	-
Hylobates_lar_LG08	11547019	11548779	HERVHF_pol	-
Hylobates_lar_LG08	11548189	11548770	HERVHF_pro	-
Hylobates_lar_LG08	11548990	11549421	HERVHF_gag	-
Hylobates_lar_LG08	11551057	11551534	HERVHF_3LTR	-

Hylobates_lar_LG08	60495252	60495571	HERVHF_5LTR	+
Hylobates_lar_LG08	60498225	60498929	HERVHF_pro	+
Hylobates_lar_LG08	60498678	60499996	HERVHF_pol	+
Hylobates_lar_LG08	60501413	60501723	HERVHF_3LTR	+
Hylobates_lar_LG08	89631815	89632223	HERVK_5LTR	+
Hylobates_lar_LG08	89633645	89634567	HERVK_pro	+
Hylobates_lar_LG08	89634462	89636387	HERVK_pol	+
Hylobates_lar_LG08	89637426	89637833	HERVK_3LTR	+
Hylobates_lar_LG08	96760303	96760714	HERVHF_5LTR	+
Hylobates_lar_LG08	96762321	96762782	HERVHF_gag	+
Hylobates_lar_LG08	96763225	96765204	HERVHF_pol	+
Hylobates_lar_LG08	96766571	96766982	HERVHF_3LTR	+
Hylobates_lar_LG08	116578799	116579236	HERVHF_5LTR	+
Hylobates_lar_LG08	116580945	116581253	HERVHF_gag	+
Hylobates_lar_LG08	116581348	116582097	HERVHF_pro	+
Hylobates_lar_LG08	116581537	116583340	HERVHF_pol	+
Hylobates_lar_LG08	116584584	116585007	HERVHF_3LTR	+
Hylobates_lar_LG08	120078823	120079574	HERVK_5LTR	+
Hylobates_lar_LG08	120081310	120081693	HERVK_gag	+
Hylobates_lar_LG08	120081630	120082585	HERVK_pro	+
Hylobates_lar_LG08	120082564	120087006	HERVK_pol	+
Hylobates_lar_LG08	120087475	120088218	HERVK_3LTR	+
Hylobates_lar_LG08	125538840	125539291	HERVHF_5LTR	-
Hylobates_lar_LG08	125539704	125541841	HERVHF_pol	-
Hylobates_lar_LG08	125541242	125541907	HERVHF_pro	-
Hylobates_lar_LG08	125542101	125542424	HERVHF_gag	-
Hylobates_lar_LG08	125544237	125544689	HERVHF_3LTR	-
Hylobates_lar_LG08	125777738	125778193	HERVHF_5LTR	-
Hylobates_lar_LG08	125778316	125778623	HERVHF_env	-
Hylobates_lar_LG08	125779232	125781328	HERVHF_pol	-
Hylobates_lar_LG08	125780711	125781454	HERVHF_pro	-
Hylobates_lar_LG08	125781558	125781941	HERVHF_gag	-
Hylobates_lar_LG08	125783483	125783936	HERVHF_3LTR	-
Hylobates_lar_LG08	128737307	128737715	HERVHF_5LTR	+
Hylobates_lar_LG08	128739296	128739622	HERVHF_gag	+
Hylobates_lar_LG08	128739815	128740564	HERVHF_pro	+
Hylobates_lar_LG08	128740022	128742134	HERVHF_pol	+
Hylobates_lar_LG08	128743136	128743541	HERVHF_3LTR	+
Hylobates_lar_LG08	131511032	131511462	HERVHF_5LTR	-
Hylobates_lar_LG08	131512431	131514541	HERVHF_pol	-
Hylobates_lar_LG08	131514029	131514571	HERVHF_pro	-
Hylobates_lar_LG08	131515888	131516313	HERVHF_gag	-
Hylobates_lar_LG08	131523408	131523835	HERVHF_3LTR	-
Hylobates_lar_LG09	52597434	52597904	HERVIPADP_5LTR	-

Hylobates_lar_LG09	52601197	52603789	HERVIPADP_pol	-
Hylobates_lar_LG09	52608210	52608688	HERVIPADP_3LTR	-
Hylobates_lar_LG09	75671665	75672181	HERVK_5LTR	+
Hylobates_lar_LG09	75672689	75673650	HERVK_gag	+
Hylobates_lar_LG09	75673894	75674340	HERVK_pro	+
Hylobates_lar_LG09	75674579	75676357	HERVK_pol	+
Hylobates_lar_LG09	75676858	75677196	HERVK_env	+
Hylobates_lar_LG09	75677267	75677782	HERVK_3LTR	+
Hylobates_lar_LG09	95717041	95717472	HERVHF_5LTR	+
Hylobates_lar_LG09	95718755	95719075	HERVHF_gag	+
Hylobates_lar_LG09	95719133	95719930	HERVHF_pro	+
Hylobates_lar_LG09	95719367	95721243	HERVHF_pol	+
Hylobates_lar_LG09	95722136	95722571	HERVHF_3LTR	+
Hylobates_lar_LG09	112447122	112447534	HERVHF_5LTR	+
Hylobates_lar_LG09	112449184	112449567	HERVHF_gag	+
Hylobates_lar_LG09	112449835	112450383	HERVHF_pro	+
Hylobates_lar_LG09	112450006	112451940	HERVHF_pol	+
Hylobates_lar_LG09	112452391	112452802	HERVHF_3LTR	+
Hylobates_lar_LG10	16670763	16671150	HERVHF_5LTR	-
Hylobates_lar_LG10	16671791	16673728	HERVHF_pol	-
Hylobates_lar_LG10	16673219	16673941	HERVHF_pro	-
Hylobates_lar_LG10	16676069	16676447	HERVHF_3LTR	-
Hylobates_lar_LG10	21036537	21036944	HERVHF_5LTR	-
Hylobates_lar_LG10	21037367	21039253	HERVHF_pol	-
Hylobates_lar_LG10	21039204	21039752	HERVHF_pro	-
Hylobates_lar_LG10	21041822	21042233	HERVHF_3LTR	-
Hylobates_lar_LG10	43326438	43326802	HERVHF_5LTR	-
Hylobates_lar_LG10	43327422	43329350	HERVHF_pol	-
Hylobates_lar_LG10	43328775	43329350	HERVHF_pro	-
Hylobates_lar_LG10	43329612	43330022	HERVHF_gag	-
Hylobates_lar_LG10	43331720	43332089	HERVHF_3LTR	-
Hylobates_lar_LG10	61679942	61680243	HERVHF_5LTR	-
Hylobates_lar_LG10	61681343	61683559	HERVHF_pol	-
Hylobates_lar_LG10	61683427	61683870	HERVHF_pro	-
Hylobates_lar_LG10	61685442	61685743	HERVHF_3LTR	-
Hylobates_lar_LG10	64538442	64538844	HERVHF_5LTR	+
Hylobates_lar_LG10	64540064	64540393	HERVHF_gag	+
Hylobates_lar_LG10	64540631	64541494	HERVHF_pro	+
Hylobates_lar_LG10	64540973	64543053	HERVHF_pol	+
Hylobates_lar_LG10	64543454	64543860	HERVHF_3LTR	+
Hylobates_lar_LG11	15215377	15215974	HERVHF_5LTR	-
Hylobates_lar_LG11	15216147	15218330	HERVHF_pol	-
Hylobates_lar_LG11	15217815	15218561	HERVHF_pro	-
Hylobates_lar_LG11	15219695	15220302	HERVHF_3LTR	-

Hylobates_lar_LG11	40022159	40022573	HERVHF_5LTR	+
Hylobates_lar_LG11	40024630	40024965	HERVHF_pro	+
Hylobates_lar_LG11	40025070	40027196	HERVHF_pol	+
Hylobates_lar_LG11	40027648	40028056	HERVHF_3LTR	+
Hylobates_lar_LG11	40971565	40972002	HERVHF_5LTR	-
Hylobates_lar_LG11	40972245	40973107	HERVHF_env	-
Hylobates_lar_LG11	40974421	40976913	HERVHF_pol	-
Hylobates_lar_LG11	40979377	40979809	HERVHF_3LTR	-
Hylobates_lar_LG11	47045116	47045448	HERVHF_5LTR	-
Hylobates_lar_LG11	47045931	47049251	HERVHF_pol	-
Hylobates_lar_LG11	47048619	47049251	HERVHF_pro	-
Hylobates_lar_LG11	47049499	47049888	HERVHF_gag	-
Hylobates_lar_LG11	47051338	47051675	HERVHF_3LTR	-
Hylobates_lar_LG11	53438183	53438574	HERVHF_5LTR	-
Hylobates_lar_LG11	53438807	53439596	HERVHF_env	-
Hylobates_lar_LG11	53440423	53441564	HERVHF_pol	-
Hylobates_lar_LG11	53441561	53441986	HERVHF_pro	-
Hylobates_lar_LG11	53442069	53442380	HERVHF_gag	-
Hylobates_lar_LG11	53444128	53444507	HERVHF_3LTR	-
Hylobates_lar_LG11	58455472	58455932	HERVHF_5LTR	-
Hylobates_lar_LG11	58456387	58458485	HERVHF_pol	-
Hylobates_lar_LG11	58458033	58458728	HERVHF_pro	-
Hylobates_lar_LG11	58458738	58459064	HERVHF_gag	-
Hylobates_lar_LG11	58460613	58461084	HERVHF_3LTR	-
Hylobates_lar_LG11	72024100	72025095	HERVHF_5LTR	-
Hylobates_lar_LG11	72025995	72026753	HERVHF_pol	-
Hylobates_lar_LG11	72026148	72026942	HERVHF_pro	-
Hylobates_lar_LG11	72027642	72028638	HERVHF_3LTR	-
Hylobates_lar_LG11	92145503	92145803	HERVHF_5LTR	-
Hylobates_lar_LG11	92145503	92145803	HERVHF_5LTR	-
Hylobates_lar_LG11	92147130	92148451	HERVHF_pol	-
Hylobates_lar_LG11	92150879	92152941	HERVHF_pol	+
Hylobates_lar_LG11	92159268	92159581	HERVHF_3LTR	+
Hylobates_lar_LG11	92159268	92159581	HERVHF_3LTR	+
Hylobates_lar_LG11	96908094	96908534	HERVHF_5LTR	-
Hylobates_lar_LG11	96908997	96912051	HERVHF_pol	-
Hylobates_lar_LG11	96912357	96912767	HERVHF_gag	-
Hylobates_lar_LG11	96914317	96914742	HERVHF_3LTR	-
Hylobates_lar_LG11	102156968	102157308	HERVHF_5LTR	-
Hylobates_lar_LG11	102157730	102159557	HERVHF_pol	-
Hylobates_lar_LG11	102159243	102159962	HERVHF_pro	-
Hylobates_lar_LG11	102162020	102162371	HERVHF_3LTR	-
Hylobates_lar_LG11	110189578	110189891	HERVHF_5LTR	-
Hylobates_lar_LG11	110192272	110193033	HERVHF_pol	-

Hylobates_lar_LG11	110194196	110194660	HERVHF_gag	-
Hylobates_lar_LG11	110195315	110195626	HERVHF_3LTR	-
Hylobates_lar_LG12	10110069	10110479	HERVHF_5LTR	-
Hylobates_lar_LG12	10112774	10114579	HERVHF_pol	-
Hylobates_lar_LG12	10116384	10116734	HERVHF_gag	-
Hylobates_lar_LG12	10117222	10117620	HERVHF_3LTR	-
Hylobates_lar_LG12	13345419	13345884	HERVHF_5LTR	-
Hylobates_lar_LG12	13346053	13347214	HERVHF_env	-
Hylobates_lar_LG12	13348939	13351178	HERVHF_pol	-
Hylobates_lar_LG12	13352066	13352428	HERVHF_gag	-
Hylobates_lar_LG12	13354033	13354486	HERVHF_3LTR	-
Hylobates_lar_LG12	18230292	18230705	HERVHF_5LTR	-
Hylobates_lar_LG12	18231159	18233196	HERVHF_pol	-
Hylobates_lar_LG12	18232675	18233556	HERVHF_pro	-
Hylobates_lar_LG12	18235492	18235906	HERVHF_3LTR	-
Hylobates_lar_LG12	55320627	55320953	HERVHF_5LTR	+
Hylobates_lar_LG12	55321786	55322112	HERVHF_gag	+
Hylobates_lar_LG12	55322229	55322780	HERVHF_pro	+
Hylobates_lar_LG12	55322633	55325313	HERVHF_pol	+
Hylobates_lar_LG12	55325892	55326215	HERVHF_3LTR	+
Hylobates_lar_LG12	55436183	55436505	HERVHF_5LTR	+
Hylobates_lar_LG12	55441298	55441645	HERVHF_gag	+
Hylobates_lar_LG12	55441740	55442558	HERVHF_pro	+
Hylobates_lar_LG12	55441977	55444131	HERVHF_pol	+
Hylobates_lar_LG12	55445611	55445918	HERVHF_3LTR	+
Hylobates_lar_LG12	56854725	56855080	HERVHF_5LTR	-
Hylobates_lar_LG12	56856567	56857911	HERVHF_pol	-
Hylobates_lar_LG12	56857357	56857986	HERVHF_pro	-
Hylobates_lar_LG12	56860102	56860440	HERVHF_3LTR	-
Hylobates_lar_LG12	69220852	69221266	HERVHF_5LTR	-
Hylobates_lar_LG12	69221851	69223886	HERVHF_pol	-
Hylobates_lar_LG12	69223269	69223997	HERVHF_pro	-
Hylobates_lar_LG12	69224149	69224520	HERVHF_gag	-
Hylobates_lar_LG12	69226231	69226645	HERVHF_3LTR	-
Hylobates_lar_LG12	70572684	70572995	HERVHF_5LTR	+
Hylobates_lar_LG12	70574566	70574907	HERVHF_gag	+
Hylobates_lar_LG12	70575052	70575858	HERVHF_pro	+
Hylobates_lar_LG12	70575295	70577530	HERVHF_pol	+
Hylobates_lar_LG12	70577980	70578300	HERVHF_3LTR	+
Hylobates_lar_LG13	37374131	37374493	HERVK_5LTR	+
Hylobates_lar_LG13	37375377	37375847	HERVK_gag	+
Hylobates_lar_LG13	37375919	37376230	HERVK_pro	+
Hylobates_lar_LG13	37376649	37378903	HERVK_pol	+
Hylobates_lar_LG13	37378911	37379274	HERVK_3LTR	+

Hylobates_lar_LG13	38704974	38705280	HERVHF_5LTR	-
Hylobates_lar_LG13	38706420	38708789	HERVHF_pol	-
Hylobates_lar_LG13	38708136	38708990	HERVHF_pro	-
Hylobates_lar_LG13	38709028	38709399	HERVHF_gag	-
Hylobates_lar_LG13	38711064	38711370	HERVHF_3LTR	-
Hylobates_lar_LG13	67375723	67376156	HERVHF_5LTR	+
Hylobates_lar_LG13	67377836	67378249	HERVHF_gag	+
Hylobates_lar_LG13	67378283	67378729	HERVHF_pro	+
Hylobates_lar_LG13	67378729	67380620	HERVHF_pol	+
Hylobates_lar_LG13	67381384	67381815	HERVHF_3LTR	+
Hylobates_lar_LG13	70285602	70286015	HERVHF_5LTR	-
Hylobates_lar_LG13	70286174	70286527	HERVHF_env	-
Hylobates_lar_LG13	70286911	70290346	HERVHF_pol	-
Hylobates_lar_LG13	70290785	70291183	HERVHF_gag	-
Hylobates_lar_LG13	70292480	70292890	HERVHF_3LTR	-
Hylobates_lar_LG13	92627065	92627426	HERVHF_5LTR	+
Hylobates_lar_LG13	92628586	92629053	HERVHF_gag	+
Hylobates_lar_LG13	92630091	92632297	HERVHF_pol	+
Hylobates_lar_LG13	92634488	92635291	HERVHF_env	+
Hylobates_lar_LG13	92635580	92635948	HERVHF_3LTR	+
Hylobates_lar_LG13	106681956	106682293	HERVHF_5LTR	+
Hylobates_lar_LG13	106683573	106684064	HERVHF_gag	+
Hylobates_lar_LG13	106684571	106685236	HERVHF_pro	+
Hylobates_lar_LG13	106684619	106686590	HERVHF_pol	+
Hylobates_lar_LG13	106687880	106688223	HERVHF_3LTR	+
Hylobates_lar_LG14	2246248	2246731	HERVHF_5LTR	-
Hylobates_lar_LG14	2247627	2248925	HERVHF_pol	-
Hylobates_lar_LG14	2248925	2249401	HERVHF_pro	-
Hylobates_lar_LG14	2249534	2249944	HERVHF_gag	-
Hylobates_lar_LG14	2260349	2260840	HERVHF_3LTR	-
Hylobates_lar_LG14	9027142	9027554	HERVHF_5LTR	+
Hylobates_lar_LG14	9030836	9031156	HERVHF_gag	+
Hylobates_lar_LG14	9031607	9033362	HERVHF_pol	+
Hylobates_lar_LG14	9034478	9034901	HERVHF_3LTR	+
Hylobates_lar_LG14	16825624	16826020	HERVHF_5LTR	-
Hylobates_lar_LG14	16826469	16828642	HERVHF_pol	-
Hylobates_lar_LG14	16830896	16831292	HERVHF_3LTR	-
Hylobates_lar_LG14	29705688	29706095	HERVHF_5LTR	-
Hylobates_lar_LG14	29706543	29708880	HERVHF_pol	-
Hylobates_lar_LG14	29711000	29711405	HERVHF_3LTR	-
Hylobates_lar_LG14	33401399	33401791	HERVHF_5LTR	-
Hylobates_lar_LG14	33401872	33402223	HERVHF_env	-
Hylobates_lar_LG14	33402748	33404067	HERVHF_pol	-
Hylobates_lar_LG14	33404066	33404455	HERVHF_pro	-

Hylobates_lar_LG14	33406638	33407032	HERVHF_3LTR	-
Hylobates_lar_LG14	36203552	36203979	HERVHF_5LTR	-
Hylobates_lar_LG14	36204384	36206457	HERVHF_pol	-
Hylobates_lar_LG14	36205915	36206553	HERVHF_pro	-
Hylobates_lar_LG14	36206771	36207291	HERVHF_gag	-
Hylobates_lar_LG14	36208786	36209216	HERVHF_3LTR	-
Hylobates_lar_LG15	14023600	14024039	HERVHF_5LTR	+
Hylobates_lar_LG15	14025744	14026043	HERVHF_gag	+
Hylobates_lar_LG15	14026088	14026678	HERVHF_pro	+
Hylobates_lar_LG15	14026280	14028200	HERVHF_pol	+
Hylobates_lar_LG15	14028850	14029280	HERVHF_3LTR	+
Hylobates_lar_LG15	29446119	29446539	HERVHF_5LTR	-
Hylobates_lar_LG15	29447290	29449670	HERVHF_pol	-
Hylobates_lar_LG15	29449781	29450329	HERVHF_pro	-
Hylobates_lar_LG15	29450686	29451129	HERVHF_gag	-
Hylobates_lar_LG15	29452490	29452912	HERVHF_3LTR	-
Hylobates_lar_LG15	65578351	65578764	HERVHF_5LTR	-
Hylobates_lar_LG15	65579172	65581145	HERVHF_pol	-
Hylobates_lar_LG15	65580768	65581319	HERVHF_pro	-
Hylobates_lar_LG15	65583951	65584356	HERVHF_3LTR	-
Hylobates_lar_LG15	66832563	66832958	HERVHF_5LTR	-
Hylobates_lar_LG15	66833369	66836128	HERVHF_pol	-
Hylobates_lar_LG15	66838355	66838759	HERVHF_3LTR	-
Hylobates_lar_LG15	92682839	92683277	HERVHF_5LTR	-
Hylobates_lar_LG15	92684103	92685792	HERVHF_pol	-
Hylobates_lar_LG15	92685250	92685831	HERVHF_pro	-
Hylobates_lar_LG15	92688077	92688519	HERVHF_3LTR	-
Hylobates_lar_LG15	94653876	94654287	HERVHF_5LTR	+
Hylobates_lar_LG15	94656178	94656624	HERVHF_pro	+
Hylobates_lar_LG15	94656348	94658487	HERVHF_pol	+
Hylobates_lar_LG15	94658901	94659313	HERVHF_3LTR	+
Hylobates_lar_LG15	102172849	102173251	HERVHF_5LTR	+
Hylobates_lar_LG15	102174992	102175585	HERVHF_gag	+
Hylobates_lar_LG15	102175601	102176425	HERVHF_pro	+
Hylobates_lar_LG15	102175883	102178879	HERVHF_pol	+
Hylobates_lar_LG15	102179281	102179688	HERVHF_3LTR	+
Hylobates_lar_LG15	102721328	102721765	HERVHF_5LTR	-
Hylobates_lar_LG15	102722204	102724279	HERVHF_pol	-
Hylobates_lar_LG15	102723746	102724588	HERVHF_pro	-
Hylobates_lar_LG15	102724581	102724991	HERVHF_gag	-
Hylobates_lar_LG15	102728396	102728834	HERVHF_3LTR	-
Hylobates_lar_LG15	107635720	107636128	HERVHF_5LTR	-
Hylobates_lar_LG15	107636583	107638461	HERVHF_pol	-
Hylobates_lar_LG15	107639235	107639555	HERVHF_gag	-

Hylobates_lar_LG15	107643571	107643982	HERVHF_3LTR	-
Hylobates_lar_LG16	2540790	2541098	HERVHF_5LTR	-
Hylobates_lar_LG16	2541650	2542327	HERVHF_env	-
Hylobates_lar_LG16	2543156	2544318	HERVHF_pol	-
Hylobates_lar_LG16	2545076	2545723	HERVHF_gag	-
Hylobates_lar_LG16	2546383	2546693	HERVHF_3LTR	-
Hylobates_lar_LG16	58484169	58484819	HERVHF_5LTR	-
Hylobates_lar_LG16	58485941	58488162	HERVHF_pol	-
Hylobates_lar_LG16	58487455	58488327	HERVHF_pro	-
Hylobates_lar_LG16	58488399	58488899	HERVHF_gag	-
Hylobates_lar_LG16	58491969	58492610	HERVHF_3LTR	-
Hylobates_lar_LG16	64028049	64028441	HERVHF_5LTR	-
Hylobates_lar_LG16	64028889	64031051	HERVHF_pol	-
Hylobates_lar_LG16	64030509	64031339	HERVHF_pro	-
Hylobates_lar_LG16	64031349	64031759	HERVHF_gag	-
Hylobates_lar_LG16	64033422	64033811	HERVHF_3LTR	-
Hylobates_lar_LG16	71067430	71067838	HERVHF_5LTR	+
Hylobates_lar_LG16	71069907	71072973	HERVHF_pro	+
Hylobates_lar_LG16	71072413	71074267	HERVHF_pol	+
Hylobates_lar_LG16	71076271	71076680	HERVHF_3LTR	+
Hylobates_lar_LG16	79100903	79101276	HERVK_5LTR	-
Hylobates_lar_LG16	79101383	79101715	HERVK_env	-
Hylobates_lar_LG16	79102421	79103266	HERVK_pol	-
Hylobates_lar_LG16	79103170	79104103	HERVK_pro	-
Hylobates_lar_LG16	79104176	79105043	HERVK_gag	-
Hylobates_lar_LG16	79105625	79105994	HERVK_3LTR	-
Hylobates_lar_LG16	84145811	84146191	HERVHF_5LTR	+
Hylobates_lar_LG16	84146666	84147262	HERVHF_gag	+
Hylobates_lar_LG16	84147199	84147657	HERVHF_pro	+
Hylobates_lar_LG16	84147710	84148051	HERVHF_pol	+
Hylobates_lar_LG16	84150247	84150627	HERVHF_3LTR	+
Hylobates_lar_LG16	101610179	101610592	HERVHF_5LTR	-
Hylobates_lar_LG16	101610612	101610917	HERVHF_env	-
Hylobates_lar_LG16	101611042	101613041	HERVHF_pol	-
Hylobates_lar_LG16	101613414	101613857	HERVHF_gag	-
Hylobates_lar_LG16	101617187	101617608	HERVHF_3LTR	-
Hylobates_lar_LG17	10646256	10646670	HERVHF_5LTR	+
Hylobates_lar_LG17	10648792	10650214	HERVHF_pro	+
Hylobates_lar_LG17	10649699	10651637	HERVHF_pol	+
Hylobates_lar_LG17	10652247	10652662	HERVHF_3LTR	+
Hylobates_lar_LG17	19325665	19326070	HERVHF_5LTR	-
Hylobates_lar_LG17	19326502	19328658	HERVHF_pol	-
Hylobates_lar_LG17	19328131	19328718	HERVHF_pro	-
Hylobates_lar_LG17	19331199	19331598	HERVHF_3LTR	-

Hylobates_lar_LG17	34124424	34124837	HERVK_5LTR	+
Hylobates_lar_LG17	34125635	34126315	HERVK_gag	+
Hylobates_lar_LG17	34126565	34127290	HERVK_pro	+
Hylobates_lar_LG17	34127185	34128445	HERVK_pol	+
Hylobates_lar_LG17	34128943	34129293	HERVK_env	+
Hylobates_lar_LG17	34129370	34129788	HERVK_3LTR	+
Hylobates_lar_LG17	76674478	76674886	HERVHF_5LTR	-
Hylobates_lar_LG17	76675481	76677198	HERVHF_pol	-
Hylobates_lar_LG17	76677074	76677679	HERVHF_pro	-
Hylobates_lar_LG17	76677775	76678089	HERVHF_gag	-
Hylobates_lar_LG17	76679638	76680048	HERVHF_3LTR	-
Hylobates_lar_LG17	81172729	81173180	HERVHF_5LTR	-
Hylobates_lar_LG17	81174337	81176262	HERVHF_pol	-
Hylobates_lar_LG17	81175858	81176685	HERVHF_pro	-
Hylobates_lar_LG17	81176721	81177101	HERVHF_gag	-
Hylobates_lar_LG17	81178674	81179125	HERVHF_3LTR	-
Hylobates_lar_LG17	92049961	92050389	HERVHF_5LTR	-
Hylobates_lar_LG17	92050970	92052927	HERVHF_pol	-
Hylobates_lar_LG17	92052325	92053179	HERVHF_pro	-
Hylobates_lar_LG17	92053272	92053595	HERVHF_gag	-
Hylobates_lar_LG17	92055146	92055576	HERVHF_3LTR	-
Hylobates_lar_LG18	1601711	1602090	HERVHF_5LTR	+
Hylobates_lar_LG18	1602714	1603256	HERVHF_gag	+
Hylobates_lar_LG18	1603290	1604195	HERVHF_pro	+
Hylobates_lar_LG18	1603578	1607120	HERVHF_pol	+
Hylobates_lar_LG18	1607566	1607953	HERVHF_3LTR	+
Hylobates_lar_LG18	30372297	30372700	HERVHF_5LTR	+
Hylobates_lar_LG18	30374413	30374823	HERVHF_gag	+
Hylobates_lar_LG18	30374984	30375688	HERVHF_pro	+
Hylobates_lar_LG18	30375050	30377391	HERVHF_pol	+
Hylobates_lar_LG18	30377982	30378384	HERVHF_3LTR	+
Hylobates_lar_LG18	53587930	53588343	HERVHF_5LTR	-
Hylobates_lar_LG18	53588932	53590738	HERVHF_pol	-
Hylobates_lar_LG18	53590358	53591086	HERVHF_pro	-
Hylobates_lar_LG18	53593122	53593519	HERVHF_3LTR	-
Hylobates_lar_LG18	65507183	65507595	HERVHF_5LTR	-
Hylobates_lar_LG18	65508791	65512768	HERVHF_pol	-
Hylobates_lar_LG18	65512226	65513011	HERVHF_pro	-
Hylobates_lar_LG18	65513058	65513381	HERVHF_gag	-
Hylobates_lar_LG18	65514025	65514436	HERVHF_3LTR	-
Hylobates_lar_LG18	86843147	86843570	HERVHF_5LTR	-
Hylobates_lar_LG18	86843978	86845855	HERVHF_pol	-
Hylobates_lar_LG18	86845881	86846291	HERVHF_pro	-
Hylobates_lar_LG18	86849231	86849648	HERVHF_3LTR	-

Hylobates_lar_LG19	14607424	14607723	HERVHF_5LTR	-
Hylobates_lar_LG19	14608316	14616594	HERVHF_pol	-
Hylobates_lar_LG19	14620095	14620403	HERVHF_3LTR	-
Hylobates_lar_LG19	75559544	75559956	HERVHF_5LTR	-
Hylobates_lar_LG19	75560734	75564569	HERVHF_pol	-
Hylobates_lar_LG19	75564027	75564617	HERVHF_pro	-
Hylobates_lar_LG19	75564863	75565186	HERVHF_gag	-
Hylobates_lar_LG19	75567011	75567428	HERVHF_3LTR	-
Hylobates_lar_LG19	81156815	81157314	HERVHF_5LTR	+
Hylobates_lar_LG19	81159559	81161877	HERVHF_pol	+
Hylobates_lar_LG19	81162950	81164613	HERVHF_env	+
Hylobates_lar_LG19	81164800	81165301	HERVHF_3LTR	+
Hylobates_lar_LG20	16902615	16903045	HERVHF_5LTR	+
Hylobates_lar_LG20	16904617	16904937	HERVHF_gag	+
Hylobates_lar_LG20	16904944	16905774	HERVHF_pro	+
Hylobates_lar_LG20	16905175	16907320	HERVHF_pol	+
Hylobates_lar_LG20	16908295	16908725	HERVHF_3LTR	+
Hylobates_lar_LG20	64777776	64778164	HSERVIII_5LTR	-
Hylobates_lar_LG20	64782157	64783498	HSERVIII_pol	-
Hylobates_lar_LG20	64792407	64792792	HSERVIII_3LTR	-
Hylobates_lar_LG22	3053011	3053314	HERVHF_5LTR	+
Hylobates_lar_LG22	3054732	3055121	HERVHF_gag	+
Hylobates_lar_LG22	3055773	3056372	HERVHF_pro	+
Hylobates_lar_LG22	3055965	3057344	HERVHF_pol	+
Hylobates_lar_LG22	3060659	3060979	HERVHF_3LTR	+
Hylobates_lar_LG22	42116914	42117367	HERVHF_5LTR	-
Hylobates_lar_LG22	42118990	42121355	HERVHF_pol	-
Hylobates_lar_LG22	42120954	42121610	HERVHF_pro	-
Hylobates_lar_LG22	42122480	42122926	HERVHF_3LTR	-
Hylobates_lar_LG22	51329506	51329841	HERVHF_5LTR	-
Hylobates_lar_LG22	51332305	51333264	HERVHF_pol	-
Hylobates_lar_LG22	51335101	51335449	HERVHF_3LTR	-
Lemur_catta_Contig101	2984787	2985144	HSERVIII_5LTR	+
Lemur_catta_Contig101	2986434	2988038	HSERVIII_pol	+
Lemur_catta_Contig101	2994365	2994703	HSERVIII_3LTR	+
Lophocebus_aterrimus_ctg10	5726120	5726450	HERVHF_5LTR	+
Lophocebus_aterrimus_ctg10	5727921	5728250	HERVHF_gag	+
Lophocebus_aterrimus_ctg10	5729008	5732653	HERVHF_pol	+
Lophocebus_aterrimus_ctg10	5733467	5733798	HERVHF_3LTR	+
Lophocebus_aterrimus_ctg105	333776	334168	HERVHF_5LTR	-
Lophocebus_aterrimus_ctg105	335810	337752	HERVHF_pol	-
Lophocebus_aterrimus_ctg105	340520	340901	HERVHF_3LTR	-
Lophocebus_aterrimus_ctg109	4184369	4184669	HERVHF_5LTR	-
Lophocebus_aterrimus_ctg109	4185281	4187682	HERVHF_pol	-

Lophocebus_aterrimus_ctg109	4187212	4188417	HERVHF_pro	-
Lophocebus_aterrimus_ctg109	4188494	4188793	HERVHF_gag	-
Lophocebus_aterrimus_ctg109	4190282	4190584	HERVHF_3LTR	-
Lophocebus_aterrimus_ctg11	2418964	2419314	HERVHF_5LTR	-
Lophocebus_aterrimus_ctg11	2419482	2420681	HERVHF_env	-
Lophocebus_aterrimus_ctg11	2421920	2423778	HERVHF_pol	-
Lophocebus_aterrimus_ctg11	2424211	2424756	HERVHF_gag	-
Lophocebus_aterrimus_ctg11	2426336	2426692	HERVHF_3LTR	-
Lophocebus_aterrimus_ctg111	3208381	3208793	HERVHF_5LTR	+
Lophocebus_aterrimus_ctg111	3209823	3213300	HERVHF_pol	+
Lophocebus_aterrimus_ctg111	3214651	3215062	HERVHF_3LTR	+
Lophocebus_aterrimus_ctg1184	89154	89585	HERVHF_5LTR	-
Lophocebus_aterrimus_ctg1184	94177	95043	HERVHF_env	-
Lophocebus_aterrimus_ctg1184	98121	100092	HERVHF_pol	-
Lophocebus_aterrimus_ctg1184	99595	100485	HERVHF_pro	-
Lophocebus_aterrimus_ctg1184	100917	101420	HERVHF_gag	-
Lophocebus_aterrimus_ctg1184	101900	102330	HERVHF_3LTR	-
Lophocebus_aterrimus_ctg119	2335576	2335888	HERVHF_5LTR	-
Lophocebus_aterrimus_ctg119	2337596	2340185	HERVHF_pol	-
Lophocebus_aterrimus_ctg119	2340622	2345000	HERVHF_gag	-
Lophocebus_aterrimus_ctg119	2345341	2345653	HERVHF_3LTR	-
Lophocebus_aterrimus_ctg13	8305251	8305563	HERVHF_5LTR	-
Lophocebus_aterrimus_ctg13	8306353	8308135	HERVHF_pol	-
Lophocebus_aterrimus_ctg13	8308227	8308559	HERVHF_pro	-
Lophocebus_aterrimus_ctg13	8308566	8309024	HERVHF_gag	-
Lophocebus_aterrimus_ctg13	8310458	8310766	HERVHF_3LTR	-
Lophocebus_aterrimus_ctg14	23438834	23439266	HERVHF_5LTR	+
Lophocebus_aterrimus_ctg14	23441120	23441476	HERVHF_gag	+
Lophocebus_aterrimus_ctg14	23441572	23442246	HERVHF_pro	+
Lophocebus_aterrimus_ctg14	23441833	23444344	HERVHF_pol	+
Lophocebus_aterrimus_ctg14	23444728	23445161	HERVHF_3LTR	+
Lophocebus_aterrimus_ctg149	2422262	2422574	HSERVIII_5LTR	-
Lophocebus_aterrimus_ctg149	2429066	2434910	HSERVIII_pol	-
Lophocebus_aterrimus_ctg149	2436837	2437143	HSERVIII_3LTR	-
Lophocebus_aterrimus_ctg149	10624085	10624395	HERVHF_5LTR	+
Lophocebus_aterrimus_ctg149	10625977	10626297	HERVHF_gag	+
Lophocebus_aterrimus_ctg149	10626290	10626622	HERVHF_pro	+
Lophocebus_aterrimus_ctg149	10626690	10629043	HERVHF_pol	+
Lophocebus_aterrimus_ctg149	10629070	10629371	HERVHF_3LTR	+
Lophocebus_aterrimus_ctg152	3913318	3913692	HERVHF_5LTR	-
Lophocebus_aterrimus_ctg152	3913858	3914428	HERVHF_env	-
Lophocebus_aterrimus_ctg152	3914773	3916601	HERVHF_pol	-
Lophocebus_aterrimus_ctg152	3916164	3917012	HERVHF_pro	-
Lophocebus_aterrimus_ctg152	3916994	3917437	HERVHF_gag	-

Lophocebus_aterrimus_ctg152	3918790	3919162	HERVHF_3LTR	-
Lophocebus_aterrimus_ctg160	11712400	11712829	HERVHF_5LTR	-
Lophocebus_aterrimus_ctg160	11713425	11716021	HERVHF_pol	-
Lophocebus_aterrimus_ctg160	11718195	11718624	HERVHF_3LTR	-
Lophocebus_aterrimus_ctg160	12512527	12512853	HERVHF_5LTR	+
Lophocebus_aterrimus_ctg160	12515132	12515446	HERVHF_gag	+
Lophocebus_aterrimus_ctg160	12515849	12517427	HERVHF_pol	+
Lophocebus_aterrimus_ctg160	12518616	12518943	HERVHF_3LTR	+
Lophocebus_aterrimus_ctg1634	10417	10785	HERVHF_5LTR	+
Lophocebus_aterrimus_ctg1634	12863	13705	HERVHF_pro	+
Lophocebus_aterrimus_ctg1634	13163	14756	HERVHF_pol	+
Lophocebus_aterrimus_ctg1634	15642	16002	HERVHF_3LTR	+
Lophocebus_aterrimus_ctg167	8272728	8273179	HERVHF_5LTR	-
Lophocebus_aterrimus_ctg167	8274036	8275662	HERVHF_pol	-
Lophocebus_aterrimus_ctg167	8275159	8275896	HERVHF_pro	-
Lophocebus_aterrimus_ctg167	8275972	8276313	HERVHF_gag	-
Lophocebus_aterrimus_ctg167	8277903	8278354	HERVHF_3LTR	-
Lophocebus_aterrimus_ctg17	981767	982128	HERVHF_5LTR	+
Lophocebus_aterrimus_ctg17	984069	984494	HERVHF_pro	+
Lophocebus_aterrimus_ctg17	984520	985858	HERVHF_pol	+
Lophocebus_aterrimus_ctg17	987008	987379	HERVHF_3LTR	+
Lophocebus_aterrimus_ctg17	1710312	1710624	HERVHF_5LTR	+
Lophocebus_aterrimus_ctg17	1712128	1712448	HERVHF_gag	+
Lophocebus_aterrimus_ctg17	1712725	1714708	HERVHF_pol	+
Lophocebus_aterrimus_ctg17	1715837	1716157	HERVHF_3LTR	+
Lophocebus_aterrimus_ctg170	3902444	3902813	HERVHF_5LTR	-
Lophocebus_aterrimus_ctg170	3903045	3903713	HERVHF_env	-
Lophocebus_aterrimus_ctg170	3903919	3905231	HERVHF_pol	-
Lophocebus_aterrimus_ctg170	3904893	3905480	HERVHF_pro	-
Lophocebus_aterrimus_ctg170	3905568	3905891	HERVHF_gag	-
Lophocebus_aterrimus_ctg170	3907553	3907949	HERVHF_3LTR	-
Lophocebus_aterrimus_ctg172	1102922	1103340	HERVHF_5LTR	-
Lophocebus_aterrimus_ctg172	1103506	1103892	HERVHF_env	-
Lophocebus_aterrimus_ctg172	1103967	1106888	HERVHF_pol	-
Lophocebus_aterrimus_ctg172	1106502	1107188	HERVHF_pro	-
Lophocebus_aterrimus_ctg172	1107314	1107754	HERVHF_gag	-
Lophocebus_aterrimus_ctg172	1109152	1109563	HERVHF_3LTR	-
Lophocebus_aterrimus_ctg177	7507376	7507776	HERVHF_5LTR	-
Lophocebus_aterrimus_ctg177	7508662	7510869	HERVHF_pol	-
Lophocebus_aterrimus_ctg177	7510222	7510950	HERVHF_pro	-
Lophocebus_aterrimus_ctg177	7512083	7512471	HERVHF_3LTR	-
Lophocebus_aterrimus_ctg183	23303	23794	HERVHF_5LTR	+
Lophocebus_aterrimus_ctg183	24521	25557	HERVHF_gag	+
Lophocebus_aterrimus_ctg183	25366	26007	HERVHF_pro	+

Lophocebus_aterrimus_ctg183	26020	27834	HERVHF_pol	+
Lophocebus_aterrimus_ctg183	29236	29727	HERVHF_3LTR	+
Lophocebus_aterrimus_ctg19	1227564	1227970	HERVHF_5LTR	+
Lophocebus_aterrimus_ctg19	1229757	1230191	HERVHF_gag	+
Lophocebus_aterrimus_ctg19	1230181	1230723	HERVHF_pro	+
Lophocebus_aterrimus_ctg19	1230641	1232765	HERVHF_pol	+
Lophocebus_aterrimus_ctg19	1233238	1233762	HERVHF_env	+
Lophocebus_aterrimus_ctg19	1234101	1234522	HERVHF_3LTR	+
Lophocebus_aterrimus_ctg19	1791150	1791582	HERVHF_5LTR	-
Lophocebus_aterrimus_ctg19	1791899	1792414	HERVHF_env	-
Lophocebus_aterrimus_ctg19	1792726	1794618	HERVHF_pol	-
Lophocebus_aterrimus_ctg19	1794951	1795250	HERVHF_gag	-
Lophocebus_aterrimus_ctg19	1796800	1797230	HERVHF_3LTR	-
Lophocebus_aterrimus_ctg193	618107	618559	HERVHF_5LTR	+
Lophocebus_aterrimus_ctg193	621253	621675	HERVHF_pro	+
Lophocebus_aterrimus_ctg193	621475	623134	HERVHF_pol	+
Lophocebus_aterrimus_ctg193	623433	624107	HERVHF_env	+
Lophocebus_aterrimus_ctg193	624268	624726	HERVHF_3LTR	+
Lophocebus_aterrimus_ctg193	1902562	1902953	HERVHF_5LTR	-
Lophocebus_aterrimus_ctg193	1905111	1908573	HERVHF_pol	-
Lophocebus_aterrimus_ctg193	1907971	1908864	HERVHF_pro	-
Lophocebus_aterrimus_ctg193	1909304	1909711	HERVHF_gag	-
Lophocebus_aterrimus_ctg193	1911022	1911422	HERVHF_3LTR	-
Lophocebus_aterrimus_ctg207	5923382	5923801	HERVHF_5LTR	+
Lophocebus_aterrimus_ctg207	5925088	5925507	HERVHF_gag	+
Lophocebus_aterrimus_ctg207	5925818	5926444	HERVHF_pro	+
Lophocebus_aterrimus_ctg207	5925866	5927464	HERVHF_pol	+
Lophocebus_aterrimus_ctg207	5928775	5929199	HERVHF_3LTR	+
Lophocebus_aterrimus_ctg208	2193111	2193565	HERVHF_5LTR	-
Lophocebus_aterrimus_ctg208	2194250	2196189	HERVHF_pol	-
Lophocebus_aterrimus_ctg208	2198483	2198924	HERVHF_3LTR	-
Lophocebus_aterrimus_ctg233	2305824	2306175	HERVHF_5LTR	+
Lophocebus_aterrimus_ctg233	2307748	2308047	HERVHF_gag	+
Lophocebus_aterrimus_ctg233	2308061	2308450	HERVHF_pro	+
Lophocebus_aterrimus_ctg233	2308594	2309034	HERVHF_pol	+
Lophocebus_aterrimus_ctg233	2310071	2310435	HERVHF_3LTR	+
Lophocebus_aterrimus_ctg24	23661915	23662322	HERVHF_5LTR	+
Lophocebus_aterrimus_ctg24	23663674	23664108	HERVHF_gag	+
Lophocebus_aterrimus_ctg24	23664681	23665331	HERVHF_pro	+
Lophocebus_aterrimus_ctg24	23664690	23667014	HERVHF_pol	+
Lophocebus_aterrimus_ctg24	23667332	23667643	HERVHF_env	+
Lophocebus_aterrimus_ctg24	23667806	23668217	HERVHF_3LTR	+
Lophocebus_aterrimus_ctg246	8256288	8256658	HERVHF_5LTR	+
Lophocebus_aterrimus_ctg246	8258683	8259510	HERVHF_pro	+

Lophocebus_aterrimus_ctg246	8258968	8260871	HERVHF_pol	+
Lophocebus_aterrimus_ctg246	8261305	8261681	HERVHF_3LTR	+
Lophocebus_aterrimus_ctg252	3508946	3509349	HERVIPADP_5LTR	+
Lophocebus_aterrimus_ctg252	3510828	3511160	HERVIPADP_gag	+
Lophocebus_aterrimus_ctg252	3512230	3514739	HERVIPADP_pol	+
Lophocebus_aterrimus_ctg252	3516026	3516592	HERVIPADP_env	+
Lophocebus_aterrimus_ctg252	3516793	3517214	HERVIPADP_3LTR	+
Lophocebus_aterrimus_ctg257	3564382	3564778	HERVHF_5LTR	-
Lophocebus_aterrimus_ctg257	3565756	3567541	HERVHF_pol	-
Lophocebus_aterrimus_ctg257	3566948	3567733	HERVHF_pro	-
Lophocebus_aterrimus_ctg257	3569586	3569983	HERVHF_3LTR	-
Lophocebus_aterrimus_ctg268	2172688	2173116	HERVHF_5LTR	-
Lophocebus_aterrimus_ctg268	2173380	2174780	HERVHF_pol	-
Lophocebus_aterrimus_ctg268	2175436	2175861	HERVHF_3LTR	-
Lophocebus_aterrimus_ctg282	104104	104449	HERVHF_5LTR	+
Lophocebus_aterrimus_ctg282	105568	106137	HERVHF_gag	+
Lophocebus_aterrimus_ctg282	107106	115305	HERVHF_pol	+
Lophocebus_aterrimus_ctg282	117987	118580	HERVHF_env	+
Lophocebus_aterrimus_ctg282	118863	119212	HERVHF_3LTR	+
Lophocebus_aterrimus_ctg297	1650212	1650531	HERVHF_5LTR	-
Lophocebus_aterrimus_ctg297	1656548	1658719	HERVHF_pol	-
Lophocebus_aterrimus_ctg297	1662076	1662391	HERVHF_3LTR	-
Lophocebus_aterrimus_ctg35	6373193	6373533	HERVHF_5LTR	-
Lophocebus_aterrimus_ctg35	6374540	6376211	HERVHF_pol	-
Lophocebus_aterrimus_ctg35	6376288	6376707	HERVHF_pro	-
Lophocebus_aterrimus_ctg35	6378487	6378823	HERVHF_3LTR	-
Lophocebus_aterrimus_ctg360	1269569	1269936	HERVHF_5LTR	-
Lophocebus_aterrimus_ctg360	1270705	1272765	HERVHF_pol	-
Lophocebus_aterrimus_ctg360	1272208	1273026	HERVHF_pro	-
Lophocebus_aterrimus_ctg360	1273016	1273357	HERVHF_gag	-
Lophocebus_aterrimus_ctg360	1274807	1275175	HERVHF_3LTR	-
Lophocebus_aterrimus_ctg37	15247110	15247425	HERVK_5LTR	+
Lophocebus_aterrimus_ctg37	15247823	15248380	HERVK_gag	+
Lophocebus_aterrimus_ctg37	15248864	15249994	HERVK_pro	+
Lophocebus_aterrimus_ctg37	15249976	15251869	HERVK_pol	+
Lophocebus_aterrimus_ctg37	15252340	15252657	HERVK_env	+
Lophocebus_aterrimus_ctg37	15253233	15253567	HERVK_3LTR	+
Lophocebus_aterrimus_ctg370	2430413	2430723	HERVHF_5LTR	-
Lophocebus_aterrimus_ctg370	2431544	2433298	HERVHF_pol	-
Lophocebus_aterrimus_ctg370	2433193	2433732	HERVHF_pro	-
Lophocebus_aterrimus_ctg370	2433785	2434228	HERVHF_gag	-
Lophocebus_aterrimus_ctg370	2435613	2435915	HERVHF_3LTR	-
Lophocebus_aterrimus_ctg373	3593846	3594170	HERVK_5LTR	-
Lophocebus_aterrimus_ctg373	3595000	3596302	HERVK_pol	-

Lophocebus_aterrimus_ctg373	3597560	3598360	HERVK_pro	-
Lophocebus_aterrimus_ctg373	3599568	3599892	HERVK_3LTR	-
Lophocebus_aterrimus_ctg38	13176080	13176535	HERVHF_5LTR	-
Lophocebus_aterrimus_ctg38	13177131	13179469	HERVHF_pol	-
Lophocebus_aterrimus_ctg38	13179037	13180629	HERVHF_pro	-
Lophocebus_aterrimus_ctg38	13180387	13180719	HERVHF_gag	-
Lophocebus_aterrimus_ctg38	13182264	13182725	HERVHF_3LTR	-
Lophocebus_aterrimus_ctg403	1096939	1097263	HERVHF_5LTR	-
Lophocebus_aterrimus_ctg403	1097868	1100018	HERVHF_pol	-
Lophocebus_aterrimus_ctg403	1099506	1100315	HERVHF_pro	-
Lophocebus_aterrimus_ctg403	1100308	1100880	HERVHF_gag	-
Lophocebus_aterrimus_ctg403	1102193	1102533	HERVHF_3LTR	-
Lophocebus_aterrimus_ctg436	790011	790380	HERVHF_5LTR	+
Lophocebus_aterrimus_ctg436	792493	794663	HERVHF_pol	+
Lophocebus_aterrimus_ctg436	795211	795583	HERVHF_3LTR	+
Lophocebus_aterrimus_ctg46	5642080	5642520	HERVHF_5LTR	-
Lophocebus_aterrimus_ctg46	5643061	5645339	HERVHF_pol	-
Lophocebus_aterrimus_ctg46	5646193	5647277	HERVHF_gag	-
Lophocebus_aterrimus_ctg46	5648083	5648515	HERVHF_3LTR	-
Lophocebus_aterrimus_ctg46	22414998	22415328	HERVK_5LTR	-
Lophocebus_aterrimus_ctg46	22415602	22415946	HERVK_env	-
Lophocebus_aterrimus_ctg46	22416556	22418035	HERVK_pol	-
Lophocebus_aterrimus_ctg46	22417948	22418698	HERVK_pro	-
Lophocebus_aterrimus_ctg46	22418810	22419343	HERVK_gag	-
Lophocebus_aterrimus_ctg46	22420236	22420562	HERVK_3LTR	-
Lophocebus_aterrimus_ctg47	8752589	8752987	HERVHF_5LTR	+
Lophocebus_aterrimus_ctg47	8754313	8754663	HERVHF_gag	+
Lophocebus_aterrimus_ctg47	8754830	8755243	HERVHF_pro	+
Lophocebus_aterrimus_ctg47	8755219	8757015	HERVHF_pol	+
Lophocebus_aterrimus_ctg47	8758835	8759176	HERVHF_env	+
Lophocebus_aterrimus_ctg47	8759342	8759732	HERVHF_3LTR	+
Lophocebus_aterrimus_ctg48	6152496	6152965	HERVHF_5LTR	+
Lophocebus_aterrimus_ctg48	6155459	6155896	HERVHF_pro	+
Lophocebus_aterrimus_ctg48	6155483	6157199	HERVHF_pol	+
Lophocebus_aterrimus_ctg48	6158392	6158862	HERVHF_3LTR	+
Lophocebus_aterrimus_ctg516	2182644	2183040	HERVHF_5LTR	+
Lophocebus_aterrimus_ctg516	2185785	2186378	HERVHF_pro	+
Lophocebus_aterrimus_ctg516	2185971	2187957	HERVHF_pol	+
Lophocebus_aterrimus_ctg516	2188400	2188799	HERVHF_3LTR	+
Lophocebus_aterrimus_ctg520	1471706	1472251	HERVHF_5LTR	+
Lophocebus_aterrimus_ctg520	1477641	1478411	HERVHF_pro	+
Lophocebus_aterrimus_ctg520	1477773	1479866	HERVHF_pol	+
Lophocebus_aterrimus_ctg520	1486385	1486920	HERVHF_3LTR	+
Lophocebus_aterrimus_ctg54	905476	905867	HERVIPADP_5LTR	-

Lophocebus_aterrimus_ctg54	906417	907049	HERVIPADP_env	-
Lophocebus_aterrimus_ctg54	908707	911536	HERVIPADP_pol	-
Lophocebus_aterrimus_ctg54	912137	912667	HERVIPADP_gag	-
Lophocebus_aterrimus_ctg54	914338	914719	HERVIPADP_3LTR	-
Lophocebus_aterrimus_ctg54	9744379	9744781	HERVHF_5LTR	-
Lophocebus_aterrimus_ctg54	9745366	9747785	HERVHF_pol	-
Lophocebus_aterrimus_ctg54	9747225	9748085	HERVHF_pro	-
Lophocebus_aterrimus_ctg54	9748087	9748449	HERVHF_gag	-
Lophocebus_aterrimus_ctg54	9749924	9750313	HERVHF_3LTR	-
Lophocebus_aterrimus_ctg55	18409799	18410150	HERVHF_5LTR	+
Lophocebus_aterrimus_ctg55	18412122	18412544	HERVHF_pro	+
Lophocebus_aterrimus_ctg55	18412493	18414729	HERVHF_pol	+
Lophocebus_aterrimus_ctg55	18415672	18416004	HERVHF_3LTR	+
Lophocebus_aterrimus_ctg575	88937	89315	HERVHF_5LTR	+
Lophocebus_aterrimus_ctg575	90777	91076	HERVHF_gag	+
Lophocebus_aterrimus_ctg575	91099	91479	HERVHF_pro	+
Lophocebus_aterrimus_ctg575	91408	92528	HERVHF_pol	+
Lophocebus_aterrimus_ctg575	93668	94044	HERVHF_3LTR	+
Lophocebus_aterrimus_ctg598	31852	32179	HERVHF_5LTR	-
Lophocebus_aterrimus_ctg598	32360	33060	HERVHF_env	-
Lophocebus_aterrimus_ctg598	33181	34153	HERVHF_pol	-
Lophocebus_aterrimus_ctg598	34347	34982	HERVHF_pro	-
Lophocebus_aterrimus_ctg598	37009	37333	HERVHF_3LTR	-
Lophocebus_aterrimus_ctg60	3840210	3840521	HERVHF_5LTR	-
Lophocebus_aterrimus_ctg60	3841357	3843128	HERVHF_pol	-
Lophocebus_aterrimus_ctg60	3842943	3843875	HERVHF_pro	-
Lophocebus_aterrimus_ctg60	3846788	3847105	HERVHF_3LTR	-
Lophocebus_aterrimus_ctg601	279886	280264	HERVHF_5LTR	-
Lophocebus_aterrimus_ctg601	280415	280771	HERVHF_env	-
Lophocebus_aterrimus_ctg601	280881	283887	HERVHF_pol	-
Lophocebus_aterrimus_ctg601	283878	284189	HERVHF_gag	-
Lophocebus_aterrimus_ctg601	285542	285920	HERVHF_3LTR	-
Lophocebus_aterrimus_ctg61	10495144	10495623	HERVK_5LTR	+
Lophocebus_aterrimus_ctg61	10497063	10498004	HERVK_pro	+
Lophocebus_aterrimus_ctg61	10497929	10499480	HERVK_pol	+
Lophocebus_aterrimus_ctg61	10500167	10500481	HERVK_env	+
Lophocebus_aterrimus_ctg61	10500579	10501046	HERVK_3LTR	+
Lophocebus_aterrimus_ctg620	136412	136730	HERVHF_5LTR	+
Lophocebus_aterrimus_ctg620	138315	138698	HERVHF_gag	+
Lophocebus_aterrimus_ctg620	138749	139554	HERVHF_pro	+
Lophocebus_aterrimus_ctg620	139261	139755	HERVHF_pol	+
Lophocebus_aterrimus_ctg620	140047	140409	HERVHF_env	+
Lophocebus_aterrimus_ctg620	140697	141023	HERVHF_3LTR	+
Lophocebus_aterrimus_ctg630	655652	656087	HERVK_5LTR	-

Lophocebus_aterrimus_ctg630	656158	656478	HERVK_env	-
Lophocebus_aterrimus_ctg630	657758	658621	HERVK_pol	-
Lophocebus_aterrimus_ctg630	658082	659329	HERVK_pro	-
Lophocebus_aterrimus_ctg630	660107	660550	HERVK_3LTR	-
Lophocebus_aterrimus_ctg64	11221376	11221807	HERVHF_5LTR	-
Lophocebus_aterrimus_ctg64	11223025	11224871	HERVHF_pol	-
Lophocebus_aterrimus_ctg64	11224287	11225072	HERVHF_pro	-
Lophocebus_aterrimus_ctg64	11225122	11225558	HERVHF_gag	-
Lophocebus_aterrimus_ctg64	11227154	11227587	HERVHF_3LTR	-
Lophocebus_aterrimus_ctg649	691584	691984	HERVHF_5LTR	-
Lophocebus_aterrimus_ctg649	692803	695049	HERVHF_pol	-
Lophocebus_aterrimus_ctg649	694411	695142	HERVHF_pro	-
Lophocebus_aterrimus_ctg649	695241	696988	HERVHF_gag	-
Lophocebus_aterrimus_ctg649	698546	698937	HERVHF_3LTR	-
Lophocebus_aterrimus_ctg65	5249192	5249503	HERVHF_5LTR	-
Lophocebus_aterrimus_ctg65	5250333	5252382	HERVHF_pol	-
Lophocebus_aterrimus_ctg65	5252331	5252792	HERVHF_pro	-
Lophocebus_aterrimus_ctg65	5254790	5255105	HERVHF_3LTR	-
Lophocebus_aterrimus_ctg671	607784	608240	HERVK_5LTR	-
Lophocebus_aterrimus_ctg671	609748	611287	HERVK_pol	-
Lophocebus_aterrimus_ctg671	611206	611826	HERVK_pro	-
Lophocebus_aterrimus_ctg671	612433	613020	HERVK_gag	-
Lophocebus_aterrimus_ctg671	613564	614036	HERVK_3LTR	-
Lophocebus_aterrimus_ctg7	7438691	7439161	HERVK_5LTR	+
Lophocebus_aterrimus_ctg7	7440107	7441006	HERVK_pro	+
Lophocebus_aterrimus_ctg7	7440901	7442648	HERVK_pol	+
Lophocebus_aterrimus_ctg7	7443332	7443648	HERVK_env	+
Lophocebus_aterrimus_ctg7	7443766	7444231	HERVK_3LTR	+
Lophocebus_aterrimus_ctg706	339993	340294	HERVIPADP_5LTR	+
Lophocebus_aterrimus_ctg706	345220	346359	HERVIPADP_pol	+
Lophocebus_aterrimus_ctg706	350457	350761	HERVIPADP_3LTR	+
Lophocebus_aterrimus_ctg706	602520	602989	HERVHF_5LTR	-
Lophocebus_aterrimus_ctg706	603543	604100	HERVHF_env	-
Lophocebus_aterrimus_ctg706	606969	607664	HERVHF_pol	-
Lophocebus_aterrimus_ctg706	610974	611433	HERVHF_3LTR	-
Lophocebus_aterrimus_ctg71	9396930	9397291	HERVHF_5LTR	-
Lophocebus_aterrimus_ctg71	9397762	9399630	HERVHF_pol	-
Lophocebus_aterrimus_ctg71	9399645	9399977	HERVHF_pro	-
Lophocebus_aterrimus_ctg71	9402192	9402557	HERVHF_3LTR	-
Lophocebus_aterrimus_ctg727	432656	433017	HERVHF_5LTR	-
Lophocebus_aterrimus_ctg727	435175	437723	HERVHF_pol	-
Lophocebus_aterrimus_ctg727	438848	439220	HERVHF_3LTR	-
Lophocebus_aterrimus_ctg734	280417	280845	HERVK_5LTR	-
Lophocebus_aterrimus_ctg734	281155	282013	HERVK_env	-

Lophocebus_aterrimus_ctg734	283168	284082	HERVK_pol	-
Lophocebus_aterrimus_ctg734	283807	285054	HERVK_pro	-
Lophocebus_aterrimus_ctg734	287858	288284	HERVK_3LTR	-
Lophocebus_aterrimus_ctg78	4820872	4821573	HERVHF_5LTR	-
Lophocebus_aterrimus_ctg78	4821578	4822236	HERVHF_env	-
Lophocebus_aterrimus_ctg78	4823634	4826243	HERVHF_pol	-
Lophocebus_aterrimus_ctg78	4827357	4828130	HERVHF_gag	-
Lophocebus_aterrimus_ctg78	4828935	4829648	HERVHF_3LTR	-
Lophocebus_aterrimus_ctg8	4834017	4834473	HERVHF_5LTR	-
Lophocebus_aterrimus_ctg8	4836045	4837537	HERVHF_pol	-
Lophocebus_aterrimus_ctg8	4837461	4838000	HERVHF_pro	-
Lophocebus_aterrimus_ctg8	4837996	4838319	HERVHF_gag	-
Lophocebus_aterrimus_ctg8	4840063	4840519	HERVHF_3LTR	-
Lophocebus_aterrimus_ctg828	240217	240617	HERVHF_5LTR	+
Lophocebus_aterrimus_ctg828	242215	242670	HERVHF_gag	+
Lophocebus_aterrimus_ctg828	242680	243195	HERVHF_pro	+
Lophocebus_aterrimus_ctg828	243075	245243	HERVHF_pol	+
Lophocebus_aterrimus_ctg828	245467	245847	HERVHF_env	+
Lophocebus_aterrimus_ctg828	246011	246418	HERVHF_3LTR	+
Lophocebus_aterrimus_ctg833	262362	262781	HERVHF_5LTR	+
Lophocebus_aterrimus_ctg833	264012	264611	HERVHF_gag	+
Lophocebus_aterrimus_ctg833	264635	265615	HERVHF_pro	+
Lophocebus_aterrimus_ctg833	265031	267567	HERVHF_pol	+
Lophocebus_aterrimus_ctg833	268580	269013	HERVHF_3LTR	+
Lophocebus_aterrimus_ctg84	5066670	5067088	HERVHF_5LTR	+
Lophocebus_aterrimus_ctg84	5070532	5070855	HERVHF_gag	+
Lophocebus_aterrimus_ctg84	5071032	5071658	HERVHF_pro	+
Lophocebus_aterrimus_ctg84	5071137	5073638	HERVHF_pol	+
Lophocebus_aterrimus_ctg84	5074095	5074524	HERVHF_3LTR	+
Lophocebus_aterrimus_ctg872	52807	53127	HERVHF_5LTR	-
Lophocebus_aterrimus_ctg872	60557	61885	HERVHF_pol	-
Lophocebus_aterrimus_ctg872	61376	62095	HERVHF_pro	-
Lophocebus_aterrimus_ctg872	62176	62499	HERVHF_gag	-
Lophocebus_aterrimus_ctg872	67621	67949	HERVHF_3LTR	-
Lophocebus_aterrimus_ctg97	250019	250470	HERVHF_5LTR	-
Lophocebus_aterrimus_ctg97	250915	252803	HERVHF_pol	-
Lophocebus_aterrimus_ctg97	252787	253278	HERVHF_pro	-
Lophocebus_aterrimus_ctg97	253274	253594	HERVHF_gag	-
Lophocebus_aterrimus_ctg97	255305	255759	HERVHF_3LTR	-
Lophocebus_aterrimus_ctg97	12048706	12049290	HERVIPADP_5LTR	+
Lophocebus_aterrimus_ctg97	12050069	12050554	HERVIPADP_gag	+
Lophocebus_aterrimus_ctg97	12051255	12054937	HERVIPADP_pol	+
Lophocebus_aterrimus_ctg97	12057014	12057596	HERVIPADP_3LTR	+
Loris_tardigradus_ctg538	584098	584650	Unknown_HERV_5LTR	-

Loris_tardigradus_ctg538	588730	590297	Unknown_HERV_pol	-
Loris_tardigradus_ctg538	589977	590988	Unknown_HERV_pro	-
Loris_tardigradus_ctg538	593040	593594	Unknown_HERV_3LTR	-
Macaca_assamensis_Contig1	29597833	29598173	HERVK_5LTR	-
Macaca_assamensis_Contig1	29598406	29598837	HERVK_env	-
Macaca_assamensis_Contig1	29599406	29601162	HERVK_pol	-
Macaca_assamensis_Contig1	29601021	29601851	HERVK_pro	-
Macaca_assamensis_Contig1	29602892	29603248	HERVK_3LTR	-
Macaca_assamensis_Contig1	32206558	32206875	HSERVIII_5LTR	-
Macaca_assamensis_Contig1	32210533	32210958	HSERVIII_pol	-
Macaca_assamensis_Contig1	32215272	32215603	HSERVIII_3LTR	-
Macaca_assamensis_Contig128	321722	322169	HERVK_5LTR	-
Macaca_assamensis_Contig128	322249	322611	HERVK_env	-
Macaca_assamensis_Contig128	323432	324238	HERVK_pol	-
Macaca_assamensis_Contig128	324231	325070	HERVK_pro	-
Macaca_assamensis_Contig128	325873	326314	HERVK_3LTR	-
Macaca_assamensis_Contig14	20277002	20277319	HERVIPADP_5LTR	+
Macaca_assamensis_Contig14	20279095	20279451	HERVIPADP_gag	+
Macaca_assamensis_Contig14	20280051	20284627	HERVIPADP_pol	+
Macaca_assamensis_Contig14	20290934	20291247	HERVIPADP_3LTR	+
Macaca_assamensis_Contig177	1327343	1327642	HSERVIII_5LTR	+
Macaca_assamensis_Contig177	1336281	1336712	HSERVIII_pol	+
Macaca_assamensis_Contig177	1341536	1341838	HSERVIII_3LTR	+
Macaca_assamensis_Contig19	23147483	23147802	HSERVIII_5LTR	+
Macaca_assamensis_Contig19	23149806	23152541	HSERVIII_pol	+
Macaca_assamensis_Contig19	23153491	23153804	HSERVIII_3LTR	+
Macaca_assamensis_Contig196	872615	872949	HERVK_5LTR	-
Macaca_assamensis_Contig196	873048	873422	HERVK_env	-
Macaca_assamensis_Contig196	874079	875560	HERVK_pol	-
Macaca_assamensis_Contig196	875857	876396	HERVK_pro	-
Macaca_assamensis_Contig196	876261	877685	HERVK_gag	-
Macaca_assamensis_Contig196	878025	878349	HERVK_3LTR	-
Macaca_assamensis_Contig20	32205658	32205982	HERVHF_5LTR	+
Macaca_assamensis_Contig20	32208199	32208840	HERVHF_pro	+
Macaca_assamensis_Contig20	32208220	32209493	HERVHF_pol	+
Macaca_assamensis_Contig20	32209831	32210444	HERVHF_env	+
Macaca_assamensis_Contig20	32210809	32211136	HERVHF_3LTR	+
Macaca_assamensis_Contig3	21430222	21430733	HERVK_5LTR	-
Macaca_assamensis_Contig3	21431660	21432929	HERVK_pol	-
Macaca_assamensis_Contig3	21432824	21433765	HERVK_pro	-
Macaca_assamensis_Contig3	21435193	21435702	HERVK_3LTR	-
Macaca_assamensis_Contig3	52160800	52161187	HERVK_5LTR	+
Macaca_assamensis_Contig3	52161703	52162191	HERVK_gag	+
Macaca_assamensis_Contig3	52162843	52163600	HERVK_pro	+

Macaca_assamensis_Contig3	52163495	52165019	HERVK_pol	+
Macaca_assamensis_Contig3	52165677	52166272	HERVK_env	+
Macaca_assamensis_Contig3	52166274	52166655	HERVK_3LTR	+
Macaca_assamensis_Contig37	13368774	13369115	HERVK_5LTR	-
Macaca_assamensis_Contig37	13370087	13371977	HERVK_pol	-
Macaca_assamensis_Contig37	13372042	13372809	HERVK_pro	-
Macaca_assamensis_Contig37	13374229	13374582	HERVK_3LTR	-
Macaca_assamensis_Contig5	56349283	56349650	HERVHF_5LTR	-
Macaca_assamensis_Contig5	56350419	56352479	HERVHF_pol	-
Macaca_assamensis_Contig5	56351880	56352671	HERVHF_pro	-
Macaca_assamensis_Contig5	56352760	56353071	HERVHF_gag	-
Macaca_assamensis_Contig5	56354519	56354886	HERVHF_3LTR	-
Macaca_assamensis_Contig6	43969422	43969929	HERVK_5LTR	+
Macaca_assamensis_Contig6	43970782	43971480	HERVK_pro	+
Macaca_assamensis_Contig6	43971669	43973216	HERVK_pol	+
Macaca_assamensis_Contig6	43973960	43974417	HERVK_env	+
Macaca_assamensis_Contig6	43974438	43974955	HERVK_3LTR	+
Macaca_assamensis_Contig67	12584223	12584739	HERVK_5LTR	-
Macaca_assamensis_Contig67	12585637	12587198	HERVK_pol	-
Macaca_assamensis_Contig67	12587314	12587823	HERVK_pro	-
Macaca_assamensis_Contig67	12589386	12589900	HERVK_3LTR	-
Macaca_mulatta_chr1	74634679	74635023	HERVHF_5LTR	+
Macaca_mulatta_chr1	74636800	74637198	HERVHF_pro	+
Macaca_mulatta_chr1	74637188	74640162	HERVHF_pol	+
Macaca_mulatta_chr1	74640847	74641205	HERVHF_3LTR	+
Macaca_mulatta_chr1	111474064	111474387	HERVHF_5LTR	-
Macaca_mulatta_chr1	111475008	111477446	HERVHF_pol	-
Macaca_mulatta_chr1	111477547	111478254	HERVHF_pro	-
Macaca_mulatta_chr1	111478304	111478636	HERVHF_gag	-
Macaca_mulatta_chr1	111479993	111480324	HERVHF_3LTR	-
Macaca_mulatta_chr1	124030176	124030489	HSERVIII_5LTR	+
Macaca_mulatta_chr1	124039199	124039570	HSERVIII_pol	+
Macaca_mulatta_chr1	124044396	124044714	HSERVIII_3LTR	+
Macaca_mulatta_chr1	146606373	146606826	HERVHF_5LTR	-
Macaca_mulatta_chr1	146607271	146610074	HERVHF_pol	-
Macaca_mulatta_chr1	146609670	146610413	HERVHF_pro	-
Macaca_mulatta_chr1	146610469	146611978	HERVHF_gag	-
Macaca_mulatta_chr1	146613496	146613953	HERVHF_3LTR	-
Macaca_mulatta_chr11	39976417	39976867	HERVHF_5LTR	+
Macaca_mulatta_chr11	39978766	39979107	HERVHF_gag	+
Macaca_mulatta_chr11	39979306	39980869	HERVHF_pol	+
Macaca_mulatta_chr11	39981896	39982347	HERVHF_3LTR	+
Macaca_mulatta_chr11	66702670	66703405	HERVHF_5LTR	-
Macaca_mulatta_chr11	66703741	66704520	HERVHF_pol	-

Macaca_mulatta_chr11	66703978	66704763	HERVHF_pro	-
Macaca_mulatta_chr11	66704813	66705112	HERVHF_gag	-
Macaca_mulatta_chr11	66711089	66711818	HERVHF_3LTR	-
Macaca_mulatta_chr11	78193774	78194075	HERVHF_5LTR	-
Macaca_mulatta_chr11	78194535	78196530	HERVHF_pol	-
Macaca_mulatta_chr11	78196538	78196870	HERVHF_pro	-
Macaca_mulatta_chr11	78198818	78199127	HERVHF_3LTR	-
Macaca_mulatta_chr12	75697924	75698289	HERVHF_5LTR	-
Macaca_mulatta_chr12	75698800	75700875	HERVHF_pol	-
Macaca_mulatta_chr12	75701112	75701621	HERVHF_pro	-
Macaca_mulatta_chr12	75701665	75703110	HERVHF_gag	-
Macaca_mulatta_chr12	75703840	75704206	HERVHF_3LTR	-
Macaca_mulatta_chr12	115036855	115037223	HERVHF_5LTR	-
Macaca_mulatta_chr12	115037390	115038289	HERVHF_env	-
Macaca_mulatta_chr12	115039141	115041517	HERVHF_pol	-
Macaca_mulatta_chr12	115043939	115044296	HERVHF_3LTR	-
Macaca_mulatta_chr13	43308557	43308884	HERVHF_5LTR	-
Macaca_mulatta_chr13	43309193	43309555	HERVHF_env	-
Macaca_mulatta_chr13	43309848	43310549	HERVHF_pol	-
Macaca_mulatta_chr13	43312841	43313159	HERVHF_3LTR	-
Macaca_mulatta_chr13	52634808	52635209	HERVHF_5LTR	-
Macaca_mulatta_chr13	52635692	52637878	HERVHF_pol	-
Macaca_mulatta_chr13	52637910	52638446	HERVHF_pro	-
Macaca_mulatta_chr13	52639500	52639889	HERVHF_3LTR	-
Macaca_mulatta_chr13	70374180	70374551	HUERSP_5LTR	-
Macaca_mulatta_chr13	70379567	70380249	HUERSP_pol	-
Macaca_mulatta_chr13	70380920	70381615	HUERSP_gag	-
Macaca_mulatta_chr13	70383568	70383934	HUERSP_3LTR	-
Macaca_mulatta_chr13	73754919	73755289	HERVHF_5LTR	+
Macaca_mulatta_chr13	73756122	73757663	HERVHF_pol	+
Macaca_mulatta_chr13	73759451	73759814	HERVHF_3LTR	+
Macaca_mulatta_chr13	90648541	90648900	HERVHF_5LTR	-
Macaca_mulatta_chr13	90649365	90651831	HERVHF_pol	-
Macaca_mulatta_chr13	90654263	90654623	HERVHF_3LTR	-
Macaca_mulatta_chr14	34470733	34471209	HERVHF_5LTR	-
Macaca_mulatta_chr14	34472363	34474082	HERVHF_pol	-
Macaca_mulatta_chr14	34473540	34474259	HERVHF_pro	-
Macaca_mulatta_chr14	34476326	34476799	HERVHF_3LTR	-
Macaca_mulatta_chr14	75125086	75125438	HERVHF_5LTR	+
Macaca_mulatta_chr14	75127345	75127905	HERVHF_gag	+
Macaca_mulatta_chr14	75128097	75128963	HERVHF_pro	+
Macaca_mulatta_chr14	75128475	75128960	HERVHF_pol	+
Macaca_mulatta_chr14	75129894	75130253	HERVHF_env	+
Macaca_mulatta_chr14	75131251	75131599	HERVHF_3LTR	+

Macaca_mulatta_chr14	97528012	97528429	HERVHF_5LTR	-
Macaca_mulatta_chr14	97529522	97533187	HERVHF_pol	-
Macaca_mulatta_chr14	97532609	97533382	HERVHF_pro	-
Macaca_mulatta_chr14	97533485	97534090	HERVHF_gag	-
Macaca_mulatta_chr14	97535543	97535961	HERVHF_3LTR	-
Macaca_mulatta_chr14	132484120	132484571	HERVHF_5LTR	+
Macaca_mulatta_chr14	132486668	132487405	HERVHF_pro	+
Macaca_mulatta_chr14	132487163	132488878	HERVHF_pol	+
Macaca_mulatta_chr14	132489377	132489836	HERVHF_3LTR	+
Macaca_mulatta_chr14	133267686	133267991	HERVHF_5LTR	-
Macaca_mulatta_chr14	133270590	133271423	HERVHF_pol	-
Macaca_mulatta_chr14	133270788	133271540	HERVHF_pro	-
Macaca_mulatta_chr14	133281071	133281389	HERVHF_3LTR	-
Macaca_mulatta_chr15	52258968	52259398	HERVHF_5LTR	+
Macaca_mulatta_chr15	52261602	52262561	HERVHF_pro	+
Macaca_mulatta_chr15	52261809	52263932	HERVHF_pol	+
Macaca_mulatta_chr15	52265189	52265616	HERVHF_3LTR	+
Macaca_mulatta_chr16	77862358	77862783	HERVHF_5LTR	+
Macaca_mulatta_chr16	77864270	77864680	HERVHF_gag	+
Macaca_mulatta_chr16	77865248	77867478	HERVHF_pol	+
Macaca_mulatta_chr16	77868786	77869598	HERVHF_env	+
Macaca_mulatta_chr16	77869782	77870181	HERVHF_3LTR	+
Macaca_mulatta_chr17	5215681	5216133	HERVHF_5LTR	+
Macaca_mulatta_chr17	5217602	5217988	HERVHF_gag	+
Macaca_mulatta_chr17	5218033	5219175	HERVHF_pro	+
Macaca_mulatta_chr17	5218630	5220320	HERVHF_pol	+
Macaca_mulatta_chr17	5221476	5221924	HERVHF_3LTR	+
Macaca_mulatta_chr17	27963861	27964297	HERVHF_5LTR	-
Macaca_mulatta_chr17	27964506	27964868	HERVHF_env	-
Macaca_mulatta_chr17	27965514	27967464	HERVHF_pol	-
Macaca_mulatta_chr17	27967063	27967641	HERVHF_pro	-
Macaca_mulatta_chr17	27967882	27968199	HERVHF_gag	-
Macaca_mulatta_chr17	27969702	27970134	HERVHF_3LTR	-
Macaca_mulatta_chr19	20690057	20690367	HERVIPADP_5LTR	+
Macaca_mulatta_chr19	20695184	20696105	HERVIPADP_pol	+
Macaca_mulatta_chr19	20704772	20705083	HERVIPADP_3LTR	+
Macaca_mulatta_chr19	42517110	42517469	HERVHF_5LTR	-
Macaca_mulatta_chr19	42518985	42522191	HERVHF_pol	-
Macaca_mulatta_chr19	42521652	42522191	HERVHF_pro	-
Macaca_mulatta_chr19	42523318	42523668	HERVHF_3LTR	-
Macaca_mulatta_chr2	36255765	36256214	HERVHF_5LTR	+
Macaca_mulatta_chr2	36257936	36258259	HERVHF_gag	+
Macaca_mulatta_chr2	36258419	36259084	HERVHF_pro	+
Macaca_mulatta_chr2	36258452	36260196	HERVHF_pol	+

Macaca_mulatta_chr2	36260891	36261343	HERVHF_3LTR	+
Macaca_mulatta_chr2	62284717	62285151	HERVHF_5LTR	+
Macaca_mulatta_chr2	62287550	62289778	HERVHF_pol	+
Macaca_mulatta_chr2	62290219	62290653	HERVHF_3LTR	+
Macaca_mulatta_chr2	125044762	125045148	HERVHF_5LTR	-
Macaca_mulatta_chr2	125045322	125046495	HERVHF_env	-
Macaca_mulatta_chr2	125047345	125050247	HERVHF_pol	-
Macaca_mulatta_chr2	125050303	125050827	HERVHF_pro	-
Macaca_mulatta_chr2	125051000	125051491	HERVHF_gag	-
Macaca_mulatta_chr2	125053176	125053572	HERVHF_3LTR	-
Macaca_mulatta_chr2	180984912	180985220	HERVHF_5LTR	+
Macaca_mulatta_chr2	180986630	180987064	HERVHF_gag	+
Macaca_mulatta_chr2	180987368	180989567	HERVHF_pol	+
Macaca_mulatta_chr2	180989684	180990250	HERVHF_env	+
Macaca_mulatta_chr2	180990541	180990852	HERVHF_3LTR	+
Macaca_mulatta_chr3	119955655	119956070	HERVHF_5LTR	-
Macaca_mulatta_chr3	119957284	119959762	HERVHF_pol	-
Macaca_mulatta_chr3	119959220	119959924	HERVHF_pro	-
Macaca_mulatta_chr3	119962321	119962727	HERVHF_3LTR	-
Macaca_mulatta_chr3	181806972	181807314	HERVHF_5LTR	-
Macaca_mulatta_chr3	181807582	181810089	HERVHF_pol	-
Macaca_mulatta_chr3	181809807	181810406	HERVHF_pro	-
Macaca_mulatta_chr3	181810520	181810867	HERVHF_gag	-
Macaca_mulatta_chr3	181812251	181812586	HERVHF_3LTR	-
Macaca_mulatta_chr4	2919612	2919912	HERVHF_5LTR	+
Macaca_mulatta_chr4	2924631	2926753	HERVHF_pol	+
Macaca_mulatta_chr4	2933364	2933671	HERVHF_3LTR	+
Macaca_mulatta_chr4	21281230	21281635	HERVIPADP_5LTR	-
Macaca_mulatta_chr4	21281983	21282369	HERVIPADP_env	-
Macaca_mulatta_chr4	21284300	21287455	HERVIPADP_pol	-
Macaca_mulatta_chr4	21288306	21288683	HERVIPADP_gag	-
Macaca_mulatta_chr4	21290134	21290544	HERVIPADP_3LTR	-
Macaca_mulatta_chr4	67466998	67467362	HERVHF_5LTR	-
Macaca_mulatta_chr4	67467509	67468023	HERVHF_env	-
Macaca_mulatta_chr4	67469835	67472270	HERVHF_pol	-
Macaca_mulatta_chr4	67473165	67473947	HERVHF_gag	-
Macaca_mulatta_chr4	67475073	67475449	HERVHF_3LTR	-
Macaca_mulatta_chr5	11335481	11335887	HERVHF_5LTR	-
Macaca_mulatta_chr5	11336531	11338226	HERVHF_pol	-
Macaca_mulatta_chr5	11338655	11338972	HERVHF_gag	-
Macaca_mulatta_chr5	11340593	11341006	HERVHF_3LTR	-
Macaca_mulatta_chr5	42988418	42988808	HERVHF_5LTR	+
Macaca_mulatta_chr5	42989994	42990308	HERVHF_gag	+
Macaca_mulatta_chr5	42990684	42991388	HERVHF_pro	+

Macaca_mulatta_chr5	42991017	42993118	HERVHF_pol	+
Macaca_mulatta_chr5	42993913	42994304	HERVHF_3LTR	+
Macaca_mulatta_chr5	141172261	141172733	HERVHF_5LTR	-
Macaca_mulatta_chr5	141173564	141175185	HERVHF_pol	-
Macaca_mulatta_chr5	141177452	141177923	HERVHF_3LTR	-
Macaca_mulatta_chr5	186492672	186493039	HERVHF_5LTR	-
Macaca_mulatta_chr5	186493469	186495697	HERVHF_pol	-
Macaca_mulatta_chr5	186495269	186496063	HERVHF_pro	-
Macaca_mulatta_chr5	186496149	186496460	HERVHF_gag	-
Macaca_mulatta_chr5	186497908	186498275	HERVHF_3LTR	-
Macaca_mulatta_chr6	86308447	86308892	HERVHF_5LTR	+
Macaca_mulatta_chr6	86311031	86311363	HERVHF_pro	+
Macaca_mulatta_chr6	86311378	86313106	HERVHF_pol	+
Macaca_mulatta_chr6	86313719	86314159	HERVHF_3LTR	+
Macaca_mulatta_chr6	93303323	93303627	HERVHF_5LTR	+
Macaca_mulatta_chr6	93306241	93307806	HERVHF_pol	+
Macaca_mulatta_chr6	93308645	93308955	HERVHF_3LTR	+
Macaca_mulatta_chr6	118193296	118193624	HERVHF_5LTR	-
Macaca_mulatta_chr6	118193790	118194221	HERVHF_env	-
Macaca_mulatta_chr6	118195248	118197529	HERVHF_pol	-
Macaca_mulatta_chr6	118198462	118198947	HERVHF_gag	-
Macaca_mulatta_chr6	118200214	118200548	HERVHF_3LTR	-
Macaca_mulatta_chr6	137597040	137597491	HERVHF_5LTR	-
Macaca_mulatta_chr6	137598716	137600388	HERVHF_pol	-
Macaca_mulatta_chr6	137599960	137600631	HERVHF_pro	-
Macaca_mulatta_chr6	137600835	137601137	HERVHF_gag	-
Macaca_mulatta_chr6	137602761	137603208	HERVHF_3LTR	-
Macaca_mulatta_chr6	171984233	171984584	HERVHF_5LTR	-
Macaca_mulatta_chr6	171986152	171987840	HERVHF_pol	-
Macaca_mulatta_chr6	171987617	171988033	HERVHF_pro	-
Macaca_mulatta_chr6	171988165	171988755	HERVHF_gag	-
Macaca_mulatta_chr6	171990359	171990708	HERVHF_3LTR	-
Macaca_mulatta_chr7	63140288	63140679	HERVHF_5LTR	+
Macaca_mulatta_chr7	63142269	63142592	HERVHF_gag	+
Macaca_mulatta_chr7	63142703	63143443	HERVHF_pro	+
Macaca_mulatta_chr7	63142784	63145047	HERVHF_pol	+
Macaca_mulatta_chr7	63145868	63146268	HERVHF_3LTR	+
Macaca_mulatta_chr7	100629550	100630001	HERVHF_5LTR	-
Macaca_mulatta_chr7	100630472	100632200	HERVHF_pol	-
Macaca_mulatta_chr7	100631658	100632449	HERVHF_pro	-
Macaca_mulatta_chr7	100632546	100633010	HERVHF_gag	-
Macaca_mulatta_chr7	100634531	100634985	HERVHF_3LTR	-
Macaca_mulatta_chr7	139543044	139543355	HERVHF_5LTR	-
Macaca_mulatta_chr7	139544033	139546099	HERVHF_pol	-

Macaca_mulatta_chr7	139545567	139546412	HERVHF_pro	-
Macaca_mulatta_chr7	139546773	139547297	HERVHF_gag	-
Macaca_mulatta_chr7	139548665	139548972	HERVHF_3LTR	-
Macaca_mulatta_chr7	150331994	150332395	HERVHF_5LTR	-
Macaca_mulatta_chr7	150332985	150335407	HERVHF_pol	-
Macaca_mulatta_chr7	150334865	150335707	HERVHF_pro	-
Macaca_mulatta_chr7	150335709	150336071	HERVHF_gag	-
Macaca_mulatta_chr7	150337545	150337936	HERVHF_3LTR	-
Macaca_mulatta_chr8	54671394	54671707	HSERVIII_5LTR	-
Macaca_mulatta_chr8	54677195	54677794	HSERVIII_pol	-
Macaca_mulatta_chr8	54680263	54680568	HSERVIII_3LTR	-
Macaca_mulatta_chr8	55621170	55621559	HSERVIII_5LTR	+
Macaca_mulatta_chr8	55623411	55623827	HSERVIII_pol	+
Macaca_mulatta_chr8	55626655	55627047	HSERVIII_3LTR	+
Macaca_mulatta_chr8	90801928	90802346	HERVHF_5LTR	-
Macaca_mulatta_chr8	90802983	90805564	HERVHF_pol	-
Macaca_mulatta_chr8	90805255	90806061	HERVHF_pro	-
Macaca_mulatta_chr8	90806066	90806422	HERVHF_gag	-
Macaca_mulatta_chr8	90807904	90808315	HERVHF_3LTR	-
Macaca_mulatta_chr9	36667215	36667578	HERVHF_5LTR	+
Macaca_mulatta_chr9	36669139	36669438	HERVHF_gag	+
Macaca_mulatta_chr9	36669912	36670618	HERVHF_pol	+
Macaca_mulatta_chr9	36671457	36671819	HERVHF_3LTR	+
Macaca_mulatta_chrX	37880884	37881317	HERVHF_5LTR	-
Macaca_mulatta_chrX	37882754	37885029	HERVHF_pol	-
Macaca_mulatta_chrX	37885376	37885810	HERVHF_gag	-
Macaca_mulatta_chrX	37887170	37887592	HERVHF_3LTR	-
Macaca_mulatta_chrX	71555472	71555850	HERVHF_5LTR	-
Macaca_mulatta_chrX	71556015	71556356	HERVHF_env	-
Macaca_mulatta_chrX	71557751	71559238	HERVHF_pol	-
Macaca_mulatta_chrX	71559463	71559774	HERVHF_gag	-
Macaca_mulatta_chrX	71561127	71561505	HERVHF_3LTR	-
Macaca_mulatta_chrX	99079355	99079748	HERVHF_5LTR	-
Macaca_mulatta_chrX	99079913	99080491	HERVHF_env	-
Macaca_mulatta_chrX	99081153	99083276	HERVHF_pol	-
Macaca_mulatta_chrX	99082632	99083411	HERVHF_pro	-
Macaca_mulatta_chrX	99083441	99083827	HERVHF_gag	-
Macaca_mulatta_chrX	99085273	99085648	HERVHF_3LTR	-
Macaca_mulatta_chrY	6381051	6381443	HERVHF_5LTR	-
Macaca_mulatta_chrY	6382969	6384479	HERVHF_pol	-
Macaca_mulatta_chrY	6384488	6384919	HERVHF_pro	-
Macaca_mulatta_chrY	6387355	6387749	HERVHF_3LTR	-
Macaca_nemestrina_KQ004642.1	3511742	3512161	HERVK_5LTR	+
Macaca_nemestrina_KQ004642.1	3512935	3513564	HERVK_pro	+

Macaca_nemestrina_KQ004642.1	3513654	3514523	HERVK_pol	+
Macaca_nemestrina_KQ004642.1	3515418	3515729	HERVK_env	+
Macaca_nemestrina_KQ004642.1	3515804	3516223	HERVK_3LTR	+
Macaca_nemestrina_KQ004731.1	4552548	4552871	HERVK_5LTR	-
Macaca_nemestrina_KQ004731.1	4553722	4555021	HERVK_pol	-
Macaca_nemestrina_KQ004731.1	4554889	4555689	HERVK_pro	-
Macaca_nemestrina_KQ004731.1	4556897	4557221	HERVK_3LTR	-
Macaca_nemestrina_KQ004775.1	364425	364939	HERVK_5LTR	+
Macaca_nemestrina_KQ004775.1	366504	367013	HERVK_pro	+
Macaca_nemestrina_KQ004775.1	367156	368690	HERVK_pol	+
Macaca_nemestrina_KQ004775.1	369586	370102	HERVK_3LTR	+
Macaca_nemestrina_KQ004830.1	4877838	4878352	HERVK_5LTR	-
Macaca_nemestrina_KQ004830.1	4878431	4878936	HERVK_env	-
Macaca_nemestrina_KQ004830.1	4880150	4881283	HERVK_pol	-
Macaca_nemestrina_KQ004830.1	4881037	4881784	HERVK_pro	-
Macaca_nemestrina_KQ004830.1	4882424	4882924	HERVK_gag	-
Macaca_nemestrina_KQ004830.1	4883310	4883818	HERVK_3LTR	-
Macaca_nemestrina_KQ004830.1	36416926	36417436	HERVK_5LTR	+
Macaca_nemestrina_KQ004830.1	36418893	36419807	HERVK_pro	+
Macaca_nemestrina_KQ004830.1	36419702	36420971	HERVK_pol	+
Macaca_nemestrina_KQ004830.1	36421896	36422409	HERVK_3LTR	+
Macaca_nemestrina_KQ005050.1	16516198	16516539	HERVK_5LTR	+
Macaca_nemestrina_KQ005050.1	16517525	16518064	HERVK_gag	+
Macaca_nemestrina_KQ005050.1	16518139	16518804	HERVK_pro	+
Macaca_nemestrina_KQ005050.1	16518896	16519690	HERVK_pol	+
Macaca_nemestrina_KQ005050.1	16520708	16521036	HERVK_3LTR	+
Macaca_nemestrina_KQ005701.1	25097569	25097980	HERVHF_5LTR	+
Macaca_nemestrina_KQ005701.1	25099022	25099534	HERVHF_gag	+
Macaca_nemestrina_KQ005701.1	25099797	25102537	HERVHF_pol	+
Macaca_nemestrina_KQ005701.1	25103905	25104955	HERVHF_env	+
Macaca_nemestrina_KQ005701.1	25105310	25105713	HERVHF_3LTR	+
Macaca_nemestrina_KQ005769.1	740973	741430	HERVK_5LTR	-
Macaca_nemestrina_KQ005769.1	742917	744477	HERVK_pol	-
Macaca_nemestrina_KQ005769.1	744396	745016	HERVK_pro	-
Macaca_nemestrina_KQ005769.1	745171	745926	HERVK_gag	-
Macaca_nemestrina_KQ005769.1	746755	747226	HERVK_3LTR	-
Macaca_nemestrina_KQ005879.1	2802914	2803431	HERVK_5LTR	-
Macaca_nemestrina_KQ005879.1	2803436	2803911	HERVK_env	-
Macaca_nemestrina_KQ005879.1	2804595	2806202	HERVK_pol	-
Macaca_nemestrina_KQ005879.1	2806391	2807068	HERVK_pro	-
Macaca_nemestrina_KQ005879.1	2807940	2808447	HERVK_3LTR	-
Macaca_nemestrina_KQ005912.1	1957251	1957629	HERVHF_5LTR	+
Macaca_nemestrina_KQ005912.1	1958841	1959284	HERVHF_gag	+
Macaca_nemestrina_KQ005912.1	1959521	1961014	HERVHF_pol	+

Macaca_nemestrina_KQ005912.1	1962400	1962750	HERVHF_env	+
Macaca_nemestrina_KQ005912.1	1962913	1963291	HERVHF_3LTR	+
Macaca_nemestrina_KQ006137.1	362956	363349	HERVIPADP_5LTR	-
Macaca_nemestrina_KQ006137.1	365952	368613	HERVIPADP_pol	-
Macaca_nemestrina_KQ006137.1	369039	369395	HERVIPADP_gag	-
Macaca_nemestrina_KQ006137.1	371271	371668	HERVIPADP_3LTR	-
Macaca_nemestrina_KQ006343.1	9916420	9916748	HERVHF_5LTR	-
Macaca_nemestrina_KQ006343.1	9916914	9917414	HERVHF_env	-
Macaca_nemestrina_KQ006343.1	9918372	9920869	HERVHF_pol	-
Macaca_nemestrina_KQ006343.1	9921929	9922390	HERVHF_gag	-
Macaca_nemestrina_KQ006343.1	9923657	9923991	HERVHF_3LTR	-
Macaca_nemestrina_KQ006601.1	11491	11805	HERVHF_5LTR	+
Macaca_nemestrina_KQ006601.1	13277	13618	HERVHF_gag	+
Macaca_nemestrina_KQ006601.1	13652	14365	HERVHF_pro	+
Macaca_nemestrina_KQ006601.1	13816	16168	HERVHF_pol	+
Macaca_nemestrina_KQ006601.1	16735	17041	HERVHF_3LTR	+
Macaca_nemestrina_KQ006730.1	38302652	38303022	HUERSP_5LTR	-
Macaca_nemestrina_KQ006730.1	38308005	38308705	HUERSP_pol	-
Macaca_nemestrina_KQ006730.1	38312051	38312416	HUERSP_3LTR	-
Macaca_nemestrina_KQ007533.1	4314931	4315256	HERVHF_5LTR	-
Macaca_nemestrina_KQ007533.1	4316007	4318339	HERVHF_pol	-
Macaca_nemestrina_KQ007533.1	4317791	4318711	HERVHF_pro	-
Macaca_nemestrina_KQ007533.1	4318776	4319584	HERVHF_gag	-
Macaca_nemestrina_KQ007533.1	4320945	4321282	HERVHF_3LTR	-
Macaca_nemestrina_KQ009049.1	13204873	13205182	HSERVIII_5LTR	-
Macaca_nemestrina_KQ009049.1	13206149	13208867	HSERVIII_pol	-
Macaca_nemestrina_KQ009049.1	13210870	13211179	HSERVIII_3LTR	-
Macaca_nemestrina_KQ009060.1	3896265	3896776	HERVK_5LTR	-
Macaca_nemestrina_KQ009060.1	3896855	3897187	HERVK_env	-
Macaca_nemestrina_KQ009060.1	3898068	3898922	HERVK_pol	-
Macaca_nemestrina_KQ009060.1	3898817	3899582	HERVK_pro	-
Macaca_nemestrina_KQ009060.1	3899703	3900644	HERVK_gag	-
Macaca_nemestrina_KQ009060.1	3901127	3901647	HERVK_3LTR	-
Macaca_nemestrina_KQ009060.1	13107717	13108172	HERVK_5LTR	+
Macaca_nemestrina_KQ009060.1	13108925	13110391	HERVK_pro	+
Macaca_nemestrina_KQ009060.1	13110286	13112034	HERVK_pol	+
Macaca_nemestrina_KQ009060.1	13112725	13113036	HERVK_env	+
Macaca_nemestrina_KQ009060.1	13113151	13113623	HERVK_3LTR	+
Macaca_nemestrina_KQ009172.1	10328302	10328773	HERVK_5LTR	-
Macaca_nemestrina_KQ009172.1	10328872	10329219	HERVK_env	-
Macaca_nemestrina_KQ009172.1	10329719	10331020	HERVK_pol	-
Macaca_nemestrina_KQ009172.1	10330891	10331912	HERVK_pro	-
Macaca_nemestrina_KQ009172.1	10333877	10334415	HERVK_gag	-
Macaca_nemestrina_KQ009172.1	10334828	10335304	HERVK_3LTR	-

Macaca_nemestrina_KQ009183.1	1944392	1944751	HERVHF_5LTR	-
Macaca_nemestrina_KQ009183.1	1946578	1949547	HERVHF_pol	-
Macaca_nemestrina_KQ009183.1	1949008	1949547	HERVHF_pro	-
Macaca_nemestrina_KQ009183.1	1950675	1951025	HERVHF_3LTR	-
Macaca_nemestrina_KQ009282.1	19313992	19314302	HERVHF_5LTR	-
Macaca_nemestrina_KQ009282.1	19319328	19322359	HERVHF_pol	-
Macaca_nemestrina_KQ009282.1	19322030	19323328	HERVHF_pro	-
Macaca_nemestrina_KQ009282.1	19323697	19324464	HERVHF_gag	-
Macaca_nemestrina_KQ009282.1	19324746	19325049	HERVHF_3LTR	-
Macaca_nemestrina_KQ009403.1	1801844	1802176	HERVK_5LTR	-
Macaca_nemestrina_KQ009403.1	1806312	1806791	HERVK_pol	-
Macaca_nemestrina_KQ009403.1	1806791	1807765	HERVK_pro	-
Macaca_nemestrina_KQ009403.1	1808954	1809283	HERVK_3LTR	-
Macaca_nemestrina_KQ009425.1	11080775	11081256	HERVK_5LTR	+
Macaca_nemestrina_KQ009425.1	11082202	11083101	HERVK_pro	+
Macaca_nemestrina_KQ009425.1	11083002	11084908	HERVK_pol	+
Macaca_nemestrina_KQ009425.1	11085850	11086327	HERVK_3LTR	+
Macaca_silenus_Contig10	2262868	2263178	HERVHF_5LTR	-
Macaca_silenus_Contig10	2264019	2265584	HERVHF_pol	-
Macaca_silenus_Contig10	2268029	2268335	HERVHF_3LTR	-
Macaca_silenus_Contig10	22453192	22453502	HSERVIII_5LTR	-
Macaca_silenus_Contig10	22454483	22457090	HSERVIII_pol	-
Macaca_silenus_Contig10	22459248	22459557	HSERVIII_3LTR	-
Macaca_silenus_Contig11	12182542	12183045	HERVK_5LTR	-
Macaca_silenus_Contig11	12183124	12183474	HERVK_env	-
Macaca_silenus_Contig11	12184302	12185976	HERVK_pol	-
Macaca_silenus_Contig11	12186085	12186477	HERVK_pro	-
Macaca_silenus_Contig11	12187129	12187590	HERVK_gag	-
Macaca_silenus_Contig11	12188009	12188512	HERVK_3LTR	-
Macaca_silenus_Contig11	43157129	43157639	HERVK_5LTR	+
Macaca_silenus_Contig11	43159096	43160010	HERVK_pro	+
Macaca_silenus_Contig11	43159905	43161174	HERVK_pol	+
Macaca_silenus_Contig11	43162099	43162610	HERVK_3LTR	+
Macaca_silenus_Contig110	3133639	3134001	HERVHF_5LTR	-
Macaca_silenus_Contig110	3135039	3135554	HERVHF_pol	-
Macaca_silenus_Contig110	3136028	3136633	HERVHF_gag	-
Macaca_silenus_Contig110	3137886	3138249	HERVHF_3LTR	-
Macaca_silenus_Contig142	1256819	1257260	HERVHF_5LTR	+
Macaca_silenus_Contig142	1258752	1259072	HERVHF_gag	+
Macaca_silenus_Contig142	1259062	1259598	HERVHF_pro	+
Macaca_silenus_Contig142	1259484	1261247	HERVHF_pol	+
Macaca_silenus_Contig142	1261982	1262427	HERVHF_3LTR	+
Macaca_silenus_Contig15	19035196	19035500	HERVIPADP_5LTR	-
Macaca_silenus_Contig15	19038076	19038621	HERVIPADP_env	-

Macaca_silenus_Contig15	19043180	19046385	HERVIPADP_pol	-
Macaca_silenus_Contig15	19047034	19047390	HERVIPADP_gag	-
Macaca_silenus_Contig15	19049171	19049478	HERVIPADP_3LTR	-
Macaca_silenus_Contig155	2302348	2302671	HERVK_5LTR	-
Macaca_silenus_Contig155	2303587	2304886	HERVK_pol	-
Macaca_silenus_Contig155	2304754	2305554	HERVK_pro	-
Macaca_silenus_Contig155	2306762	2307086	HERVK_3LTR	-
Macaca_silenus_Contig16	8080658	8081075	HERVHF_5LTR	-
Macaca_silenus_Contig16	8081934	8086360	HERVHF_pol	-
Macaca_silenus_Contig16	8085755	8086555	HERVHF_pro	-
Macaca_silenus_Contig16	8086658	8087191	HERVHF_gag	-
Macaca_silenus_Contig16	8088716	8089133	HERVHF_3LTR	-
Macaca_silenus_Contig18	5888165	5888469	HSERVIII_5LTR	-
Macaca_silenus_Contig18	5893989	5894588	HSERVIII_pol	-
Macaca_silenus_Contig18	5901352	5901666	HSERVIII_3LTR	-
Macaca_silenus_Contig188	269639	269942	HSERVIII_5LTR	-
Macaca_silenus_Contig188	274767	275432	HSERVIII_pol	-
Macaca_silenus_Contig188	283861	284172	HSERVIII_3LTR	-
Macaca_silenus_Contig2	566008	566372	HERVHF_5LTR	-
Macaca_silenus_Contig2	567256	569705	HERVHF_pol	-
Macaca_silenus_Contig2	569163	569903	HERVHF_pro	-
Macaca_silenus_Contig2	570536	570906	HERVHF_3LTR	-
Macaca_silenus_Contig2	3974013	3974377	HUERSP_5LTR	+
Macaca_silenus_Contig2	3976371	3977027	HUERSP_gag	+
Macaca_silenus_Contig2	3977698	3978425	HUERSP_pol	+
Macaca_silenus_Contig2	3983385	3983756	HUERSP_3LTR	+
Macaca_silenus_Contig2	11818749	11819508	HERVHF_5LTR	+
Macaca_silenus_Contig2	11821403	11821708	HERVHF_gag	+
Macaca_silenus_Contig2	11821957	11822538	HERVHF_pro	+
Macaca_silenus_Contig2	11822182	11824046	HERVHF_pol	+
Macaca_silenus_Contig2	11824319	11825079	HERVHF_3LTR	+
Macaca_silenus_Contig2	30989672	30989990	HERVHF_5LTR	+
Macaca_silenus_Contig2	30992230	30992988	HERVHF_pol	+
Macaca_silenus_Contig2	30993277	30993639	HERVHF_env	+
Macaca_silenus_Contig2	30993928	30994254	HERVHF_3LTR	+
Macaca_silenus_Contig20	3858886	3859277	HERVHF_5LTR	+
Macaca_silenus_Contig20	3860754	3861074	HERVHF_gag	+
Macaca_silenus_Contig20	3861118	3861957	HERVHF_pro	+
Macaca_silenus_Contig20	3861583	3863837	HERVHF_pol	+
Macaca_silenus_Contig20	3864425	3864826	HERVHF_3LTR	+
Macaca_silenus_Contig225	701486	701886	HERVHF_5LTR	-
Macaca_silenus_Contig225	702726	704971	HERVHF_pol	-
Macaca_silenus_Contig225	704333	705064	HERVHF_pro	-
Macaca_silenus_Contig225	705163	705504	HERVHF_gag	-

Macaca_silenus_Contig225	707054	707445	HERVHF_3LTR	-
Macaca_silenus_Contig263	617428	617769	HERVK_5LTR	-
Macaca_silenus_Contig263	618038	618622	HERVK_env	-
Macaca_silenus_Contig263	618728	619994	HERVK_pol	-
Macaca_silenus_Contig263	619889	620826	HERVK_pro	-
Macaca_silenus_Contig263	622296	622640	HERVK_3LTR	-
Macaca_silenus_Contig27	10436659	10436968	HERVHF_5LTR	+
Macaca_silenus_Contig27	10438291	10438722	HERVHF_gag	+
Macaca_silenus_Contig27	10438771	10439614	HERVHF_pro	+
Macaca_silenus_Contig27	10439207	10441422	HERVHF_pol	+
Macaca_silenus_Contig27	10441554	10441855	HERVHF_3LTR	+
Macaca_silenus_Contig27	22006808	22007537	HERVHF_5LTR	+
Macaca_silenus_Contig27	22013504	22013824	HERVHF_gag	+
Macaca_silenus_Contig27	22013877	22014656	HERVHF_pro	+
Macaca_silenus_Contig27	22014075	22014827	HERVHF_pol	+
Macaca_silenus_Contig27	22015227	22015962	HERVHF_3LTR	+
Macaca_silenus_Contig35	9485530	9486041	HERVK_5LTR	-
Macaca_silenus_Contig35	9486120	9486431	HERVK_env	-
Macaca_silenus_Contig35	9487453	9488187	HERVK_pol	-
Macaca_silenus_Contig35	9488091	9488847	HERVK_pro	-
Macaca_silenus_Contig35	9489142	9490017	HERVK_gag	-
Macaca_silenus_Contig35	9490392	9490912	HERVK_3LTR	-
Macaca_silenus_Contig35	18445883	18446360	HERVK_5LTR	+
Macaca_silenus_Contig35	18447113	18448054	HERVK_pro	+
Macaca_silenus_Contig35	18447952	18449865	HERVK_pol	+
Macaca_silenus_Contig35	18450388	18450699	HERVK_env	+
Macaca_silenus_Contig35	18450797	18451287	HERVK_3LTR	+
Macaca_silenus_Contig42	370360	370825	HERVHF_5LTR	+
Macaca_silenus_Contig42	372940	374698	HERVHF_pol	+
Macaca_silenus_Contig42	375529	376000	HERVHF_3LTR	+
Macaca_silenus_Contig46	9533334	9533713	HERVHF_5LTR	+
Macaca_silenus_Contig46	9535966	9537999	HERVHF_pol	+
Macaca_silenus_Contig46	9538359	9538724	HERVHF_env	+
Macaca_silenus_Contig46	9538942	9539315	HERVHF_3LTR	+
Macaca_silenus_Contig5	29571001	29571341	HERVK_5LTR	-
Macaca_silenus_Contig5	29572422	29574334	HERVK_pol	-
Macaca_silenus_Contig5	29574241	29575128	HERVK_pro	-
Macaca_silenus_Contig5	29576072	29576428	HERVK_3LTR	-
Macaca_silenus_Contig56	13526073	13526503	HERVHF_5LTR	+
Macaca_silenus_Contig56	13528656	13530865	HERVHF_pol	+
Macaca_silenus_Contig56	13531625	13532056	HERVHF_3LTR	+
Macaca_silenus_Contig57	2852834	2853135	HERVHF_5LTR	-
Macaca_silenus_Contig57	2853431	2853928	HERVHF_env	-
Macaca_silenus_Contig57	2855545	2858055	HERVHF_pol	-

Macaca_silenus_Contig57	2857498	2858298	HERVHF_pro	-
Macaca_silenus_Contig57	2858708	2859286	HERVHF_gag	-
Macaca_silenus_Contig57	2860508	2860807	HERVHF_3LTR	-
Macaca_silenus_Contig64	6682435	6683283	HERVHF_5LTR	+
Macaca_silenus_Contig64	6683388	6684185	HERVHF_pro	+
Macaca_silenus_Contig64	6683586	6685081	HERVHF_pol	+
Macaca_silenus_Contig64	6687631	6688484	HERVHF_3LTR	+
Macaca_silenus_Contig67	997897	998303	HERVHF_5LTR	+
Macaca_silenus_Contig67	1000144	1000524	HERVHF_gag	+
Macaca_silenus_Contig67	1000708	1001412	HERVHF_pro	+
Macaca_silenus_Contig67	1000774	1004112	HERVHF_pol	+
Macaca_silenus_Contig67	1004741	1005155	HERVHF_3LTR	+
Macaca_silenus_Contig69	10321193	10321503	HERVHF_5LTR	+
Macaca_silenus_Contig69	10323736	10324089	HERVHF_gag	+
Macaca_silenus_Contig69	10324571	10330336	HERVHF_pol	+
Macaca_silenus_Contig69	10330657	10330958	HERVHF_3LTR	+
Macaca_silenus_Contig71	10617782	10618299	HERVK_5LTR	-
Macaca_silenus_Contig71	10618304	10618779	HERVK_env	-
Macaca_silenus_Contig71	10619463	10621070	HERVK_pol	-
Macaca_silenus_Contig71	10621259	10621957	HERVK_pro	-
Macaca_silenus_Contig71	10622808	10623315	HERVK_3LTR	-
Macaca_silenus_Contig78	6966859	6967223	HERVK_5LTR	-
Macaca_silenus_Contig78	6967320	6967664	HERVK_env	-
Macaca_silenus_Contig78	6968171	6970355	HERVK_pol	-
Macaca_silenus_Contig78	6970241	6971182	HERVK_pro	-
Macaca_silenus_Contig78	6972763	6973133	HERVK_3LTR	-
Macaca_silenus_Contig8	22296362	22296719	HERVHF_5LTR	+
Macaca_silenus_Contig8	22299119	22301213	HERVHF_pol	+
Macaca_silenus_Contig8	22302647	22303246	HERVHF_env	+
Macaca_silenus_Contig8	22303411	22303779	HERVHF_3LTR	+
Mandrillus_leucophaeus_scaffold115	9160213	9160652	HERVHF_5LTR	-
Mandrillus_leucophaeus_scaffold115	9161403	9163062	HERVHF_pol	-
Mandrillus_leucophaeus_scaffold115	9163419	9163736	HERVHF_gag	-
Mandrillus_leucophaeus_scaffold115	9165412	9165857	HERVHF_3LTR	-
Mandrillus_leucophaeus_scaffold115	22066850	22067163	HSERVIII_5LTR	-
Mandrillus_leucophaeus_scaffold115	22068018	22070844	HSERVIII_pol	-
Mandrillus_leucophaeus_scaffold115	22072849	22073168	HSERVIII_3LTR	-
Mandrillus_leucophaeus_scaffold125	43477125	43477566	HERVHF_5LTR	-
Mandrillus_leucophaeus_scaffold125	43478004	43478498	HERVHF_env	-
Mandrillus_leucophaeus_scaffold125	43478637	43480090	HERVHF_pol	-
Mandrillus_leucophaeus_scaffold125	43479509	43480294	HERVHF_pro	-
Mandrillus_leucophaeus_scaffold125	43482311	43482756	HERVHF_3LTR	-
Mandrillus_leucophaeus_scaffold138	4278695	4279086	HERVHF_5LTR	+
Mandrillus_leucophaeus_scaffold138	4280563	4280862	HERVHF_gag	+

Mandrillus_leucophaeus_scaffold138	4280927	4281766	HERVHF_pro	+
Mandrillus_leucophaeus_scaffold138	4281191	4283649	HERVHF_pol	+
Mandrillus_leucophaeus_scaffold138	4284237	4284638	HERVHF_3LTR	+
Mandrillus_leucophaeus_scaffold138	32323790	32324103	HERVHF_5LTR	+
Mandrillus_leucophaeus_scaffold138	32326193	32326948	HERVHF_pro	+
Mandrillus_leucophaeus_scaffold138	32327143	32327911	HERVHF_pol	+
Mandrillus_leucophaeus_scaffold138	32328336	32328743	HERVHF_env	+
Mandrillus_leucophaeus_scaffold138	32328929	32329249	HERVHF_3LTR	+
Mandrillus_leucophaeus_scaffold139	19013117	19013506	HERVHF_5LTR	+
Mandrillus_leucophaeus_scaffold139	19016156	19019170	HERVHF_pol	+
Mandrillus_leucophaeus_scaffold139	19019224	19019699	HERVHF_env	+
Mandrillus_leucophaeus_scaffold139	19019982	19020367	HERVHF_3LTR	+
Mandrillus_leucophaeus_scaffold141	20900989	20901363	HERVHF_5LTR	-
Mandrillus_leucophaeus_scaffold141	20901529	20902097	HERVHF_env	-
Mandrillus_leucophaeus_scaffold141	20902191	20904268	HERVHF_pol	-
Mandrillus_leucophaeus_scaffold141	20903831	20904679	HERVHF_pro	-
Mandrillus_leucophaeus_scaffold141	20904751	20905107	HERVHF_gag	-
Mandrillus_leucophaeus_scaffold141	20906455	20906827	HERVHF_3LTR	-
Mandrillus_leucophaeus_scaffold141	24954560	24955014	HERVHF_5LTR	+
Mandrillus_leucophaeus_scaffold141	24956578	24957853	HERVHF_pol	+
Mandrillus_leucophaeus_scaffold141	24958081	24958532	HERVHF_3LTR	+
Mandrillus_leucophaeus_scaffold142	12991458	12991909	HERVHF_5LTR	-
Mandrillus_leucophaeus_scaffold142	12992376	12994200	HERVHF_pol	-
Mandrillus_leucophaeus_scaffold142	12993580	12994344	HERVHF_pro	-
Mandrillus_leucophaeus_scaffold142	12996486	12996940	HERVHF_3LTR	-
Mandrillus_leucophaeus_scaffold147	45430264	45430675	HERVHF_5LTR	+
Mandrillus_leucophaeus_scaffold147	45432860	45433612	HERVHF_pro	+
Mandrillus_leucophaeus_scaffold147	45432965	45434677	HERVHF_pol	+
Mandrillus_leucophaeus_scaffold147	45435542	45435949	HERVHF_3LTR	+
Mandrillus_leucophaeus_scaffold153820	6449417	6449826	HERVHF_5LTR	-
Mandrillus_leucophaeus_scaffold153820	6449992	6450360	HERVHF_env	-
Mandrillus_leucophaeus_scaffold153820	6450453	6453149	HERVHF_pol	-
Mandrillus_leucophaeus_scaffold153820	6452873	6453388	HERVHF_pro	-
Mandrillus_leucophaeus_scaffold153820	6453425	6454435	HERVHF_gag	-
Mandrillus_leucophaeus_scaffold153820	6455450	6455851	HERVHF_3LTR	-
Mandrillus_leucophaeus_scaffold153923	6272318	6272753	HERVK_5LTR	-
Mandrillus_leucophaeus_scaffold153923	6272824	6273135	HERVK_env	-
Mandrillus_leucophaeus_scaffold153923	6273614	6274807	HERVK_pol	-
Mandrillus_leucophaeus_scaffold153923	6274364	6275584	HERVK_pro	-
Mandrillus_leucophaeus_scaffold153923	6276389	6276832	HERVK_3LTR	-
Mandrillus_leucophaeus_scaffold154115	4046790	4047151	HERVHF_5LTR	-
Mandrillus_leucophaeus_scaffold154115	4048192	4048704	HERVHF_pol	-
Mandrillus_leucophaeus_scaffold154115	4049179	4049478	HERVHF_gag	-
Mandrillus_leucophaeus_scaffold154115	4051051	4051400	HERVHF_3LTR	-

Mandrillus_leucophaeus_scaffold154151	4046908	4047269	HERVHF_5LTR	-
Mandrillus_leucophaeus_scaffold154151	4048310	4048822	HERVHF_pol	-
Mandrillus_leucophaeus_scaffold154151	4049297	4049596	HERVHF_gag	-
Mandrillus_leucophaeus_scaffold154151	4051169	4051518	HERVHF_3LTR	-
Mandrillus_leucophaeus_scaffold178	5651103	5651551	HERVIPADP_5LTR	-
Mandrillus_leucophaeus_scaffold178	5652034	5652465	HERVIPADP_env	-
Mandrillus_leucophaeus_scaffold178	5653472	5656775	HERVIPADP_pol	-
Mandrillus_leucophaeus_scaffold178	5659445	5659897	HERVIPADP_3LTR	-
Mandrillus_leucophaeus_scaffold178	10056672	10056972	HERVHF_5LTR	-
Mandrillus_leucophaeus_scaffold178	10057587	10060123	HERVHF_pol	-
Mandrillus_leucophaeus_scaffold178	10059617	10060240	HERVHF_pro	-
Mandrillus_leucophaeus_scaffold178	10060775	10061074	HERVHF_gag	-
Mandrillus_leucophaeus_scaffold178	10062648	10062950	HERVHF_3LTR	-
Mandrillus_leucophaeus_scaffold178	13885893	13886411	HERVHF_5LTR	-
Mandrillus_leucophaeus_scaffold178	13887644	13889346	HERVHF_pol	-
Mandrillus_leucophaeus_scaffold178	13889603	13890034	HERVHF_pro	-
Mandrillus_leucophaeus_scaffold178	13890096	13890539	HERVHF_gag	-
Mandrillus_leucophaeus_scaffold178	13892255	13892777	HERVHF_3LTR	-
Mandrillus_leucophaeus_scaffold210	8662420	8663188	HERVHF_5LTR	-
Mandrillus_leucophaeus_scaffold210	8663191	8665294	HERVHF_pol	-
Mandrillus_leucophaeus_scaffold210	8668353	8669104	HERVHF_3LTR	-
Mandrillus_leucophaeus_scaffold211	1778883	1779204	HERVHF_5LTR	-
Mandrillus_leucophaeus_scaffold211	1780030	1782141	HERVHF_pol	-
Mandrillus_leucophaeus_scaffold211	1781683	1782198	HERVHF_pro	-
Mandrillus_leucophaeus_scaffold211	1782507	1783006	HERVHF_gag	-
Mandrillus_leucophaeus_scaffold211	1784639	1784944	HERVHF_3LTR	-
Mandrillus_leucophaeus_scaffold213	9001900	9002345	HERVHF_5LTR	+
Mandrillus_leucophaeus_scaffold213	9003974	9004279	HERVHF_gag	+
Mandrillus_leucophaeus_scaffold213	9004525	9005103	HERVHF_pro	+
Mandrillus_leucophaeus_scaffold213	9004750	9006896	HERVHF_pol	+
Mandrillus_leucophaeus_scaffold213	9007424	9007878	HERVHF_3LTR	+
Mandrillus_leucophaeus_scaffold226	11534543	11534893	HERVK_5LTR	+
Mandrillus_leucophaeus_scaffold226	11535277	11535807	HERVK_gag	+
Mandrillus_leucophaeus_scaffold226	11536309	11537087	HERVK_pro	+
Mandrillus_leucophaeus_scaffold226	11537137	11539030	HERVK_pol	+
Mandrillus_leucophaeus_scaffold226	11540004	11540372	HERVK_3LTR	+
Mandrillus_leucophaeus_scaffold227	25013307	25013736	HERVHF_5LTR	-
Mandrillus_leucophaeus_scaffold227	25014579	25016860	HERVHF_pol	-
Mandrillus_leucophaeus_scaffold227	25016222	25016965	HERVHF_pro	-
Mandrillus_leucophaeus_scaffold227	25019206	25019638	HERVHF_3LTR	-
Mandrillus_leucophaeus_scaffold245	12872119	12872571	HERVHF_5LTR	+
Mandrillus_leucophaeus_scaffold245	12874562	12875350	HERVHF_pro	+
Mandrillus_leucophaeus_scaffold245	12874766	12876317	HERVHF_pol	+
Mandrillus_leucophaeus_scaffold245	12877377	12877829	HERVHF_3LTR	+

Mandrillus_leucophaeus_scaffold245	35324356	35324809	HERVHF_5LTR	-
Mandrillus_leucophaeus_scaffold245	35325810	35327520	HERVHF_pol	-
Mandrillus_leucophaeus_scaffold245	35327510	35328766	HERVHF_pro	-
Mandrillus_leucophaeus_scaffold245	35330542	35330994	HERVHF_3LTR	-
Mandrillus_leucophaeus_scaffold270	10391416	10391886	HERVK_5LTR	+
Mandrillus_leucophaeus_scaffold270	10392832	10393731	HERVK_pro	+
Mandrillus_leucophaeus_scaffold270	10393629	10395382	HERVK_pol	+
Mandrillus_leucophaeus_scaffold270	10396492	10396958	HERVK_3LTR	+
Mandrillus_leucophaeus_scaffold280	3616099	3616463	HERVHF_5LTR	+
Mandrillus_leucophaeus_scaffold280	3617915	3618226	HERVHF_gag	+
Mandrillus_leucophaeus_scaffold280	3618289	3619110	HERVHF_pro	+
Mandrillus_leucophaeus_scaffold280	3618778	3620691	HERVHF_pol	+
Mandrillus_leucophaeus_scaffold280	3621337	3621705	HERVHF_3LTR	+
Mandrillus_leucophaeus_scaffold284	16645734	16646050	HERVHF_5LTR	+
Mandrillus_leucophaeus_scaffold284	16647438	16647785	HERVHF_gag	+
Mandrillus_leucophaeus_scaffold284	16647955	16648704	HERVHF_pro	+
Mandrillus_leucophaeus_scaffold284	16648300	16651860	HERVHF_pol	+
Mandrillus_leucophaeus_scaffold284	16651931	16652317	HERVHF_env	+
Mandrillus_leucophaeus_scaffold284	16652483	16652807	HERVHF_3LTR	+
Mandrillus_leucophaeus_scaffold284	24840944	24841252	HERVHF_5LTR	+
Mandrillus_leucophaeus_scaffold284	24842692	24843030	HERVHF_gag	+
Mandrillus_leucophaeus_scaffold284	24843151	24843474	HERVHF_pro	+
Mandrillus_leucophaeus_scaffold284	24843575	24845349	HERVHF_pol	+
Mandrillus_leucophaeus_scaffold284	24846138	24846450	HERVHF_3LTR	+
Mandrillus_leucophaeus_scaffold330	10184710	10185038	HERVHF_5LTR	+
Mandrillus_leucophaeus_scaffold330	10186415	10186762	HERVHF_gag	+
Mandrillus_leucophaeus_scaffold330	10186826	10187671	HERVHF_pro	+
Mandrillus_leucophaeus_scaffold330	10187193	10189003	HERVHF_pol	+
Mandrillus_leucophaeus_scaffold330	10190001	10190340	HERVHF_3LTR	+
Mandrillus_leucophaeus_scaffold332	3647806	3648128	HSERVIII_5LTR	-
Mandrillus_leucophaeus_scaffold332	3653930	3656776	HSERVIII_pol	-
Mandrillus_leucophaeus_scaffold332	3661897	3662232	HSERVIII_3LTR	-
Mandrillus_leucophaeus_scaffold341	2132120	2132568	HERVK_5LTR	+
Mandrillus_leucophaeus_scaffold341	2134417	2134743	HERVK_gag	+
Mandrillus_leucophaeus_scaffold341	2134893	2135824	HERVK_pro	+
Mandrillus_leucophaeus_scaffold341	2135806	2136339	HERVK_pol	+
Mandrillus_leucophaeus_scaffold341	2138014	2138779	HERVK_env	+
Mandrillus_leucophaeus_scaffold341	2139090	2139560	HERVK_3LTR	+
Mandrillus_leucophaeus_scaffold357	2504643	2505013	HERVHF_5LTR	-
Mandrillus_leucophaeus_scaffold357	2505429	2508312	HERVHF_pol	-
Mandrillus_leucophaeus_scaffold357	2507749	2508501	HERVHF_pro	-
Mandrillus_leucophaeus_scaffold357	2508600	2508941	HERVHF_gag	-
Mandrillus_leucophaeus_scaffold357	2510531	2510907	HERVHF_3LTR	-
Mandrillus_leucophaeus_scaffold403	10703023	10703341	HSERVIII_5LTR	+

Mandrillus_leucophaeus_scaffold403	10705410	10708104	HSERVIII_pol	+
Mandrillus_leucophaeus_scaffold403	10709527	10709847	HSERVIII_3LTR	+
Mandrillus_leucophaeus_scaffold403	11233085	11233575	HERVHF_5LTR	-
Mandrillus_leucophaeus_scaffold403	11234978	11236583	HERVHF_pol	-
Mandrillus_leucophaeus_scaffold403	11236800	11237441	HERVHF_pro	-
Mandrillus_leucophaeus_scaffold403	11237280	11238288	HERVHF_gag	-
Mandrillus_leucophaeus_scaffold403	11239063	11239554	HERVHF_3LTR	-
Mandrillus_leucophaeus_scaffold475	1120743	1121175	HERVHF_5LTR	+
Mandrillus_leucophaeus_scaffold475	1122490	1122840	HERVHF_gag	+
Mandrillus_leucophaeus_scaffold475	1123699	1124646	HERVHF_pro	+
Mandrillus_leucophaeus_scaffold475	1124013	1125904	HERVHF_pol	+
Mandrillus_leucophaeus_scaffold475	1126949	1127382	HERVHF_3LTR	+
Mandrillus_leucophaeus_scaffold536	113595	113919	HERVK_5LTR	+
Mandrillus_leucophaeus_scaffold536	115161	115928	HERVK_pro	+
Mandrillus_leucophaeus_scaffold536	115823	117098	HERVK_pol	+
Mandrillus_leucophaeus_scaffold536	117948	118272	HERVK_3LTR	+
Mandrillus_leucophaeus_scaffold706	9742488	9742865	HERVHF_5LTR	+
Mandrillus_leucophaeus_scaffold706	9744888	9745694	HERVHF_pro	+
Mandrillus_leucophaeus_scaffold706	9745173	9747074	HERVHF_pol	+
Mandrillus_leucophaeus_scaffold706	9747509	9747886	HERVHF_3LTR	+
Mandrillus_leucophaeus_scaffold714	372739	373153	HERVK_5LTR	-
Mandrillus_leucophaeus_scaffold714	374753	376226	HERVK_pol	-
Mandrillus_leucophaeus_scaffold714	376136	376795	HERVK_pro	-
Mandrillus_leucophaeus_scaffold714	378533	378937	HERVK_3LTR	-
Mandrillus_leucophaeus_scaffold76	56549167	56549476	HERVHF_5LTR	+
Mandrillus_leucophaeus_scaffold76	56551435	56551770	HERVHF_pro	+
Mandrillus_leucophaeus_scaffold76	56551802	56554148	HERVHF_pol	+
Mandrillus_leucophaeus_scaffold76	56554217	56554518	HERVHF_3LTR	+
Mandrillus_leucophaeus_scaffold76	67876368	67877202	HERVHF_5LTR	+
Mandrillus_leucophaeus_scaffold76	67883709	67884509	HERVHF_pro	+
Mandrillus_leucophaeus_scaffold76	67884162	67884701	HERVHF_pol	+
Mandrillus_leucophaeus_scaffold76	67885101	67885929	HERVHF_3LTR	+
Mandrillus_leucophaeus_scaffold78	34774903	34775299	HERVHF_5LTR	-
Mandrillus_leucophaeus_scaffold78	34775677	34777815	HERVHF_pol	-
Mandrillus_leucophaeus_scaffold78	34777341	34778303	HERVHF_pro	-
Mandrillus_leucophaeus_scaffold78	34780101	34780498	HERVHF_3LTR	-
Mandrillus_leucophaeus_scaffold78	52975255	52975607	HERVHF_5LTR	-
Mandrillus_leucophaeus_scaffold78	52978879	52981577	HERVHF_pol	-
Mandrillus_leucophaeus_scaffold78	52981170	52981955	HERVHF_pro	-
Mandrillus_leucophaeus_scaffold78	52982044	52982442	HERVHF_gag	-
Mandrillus_leucophaeus_scaffold78	52983976	52984327	HERVHF_3LTR	-
Mandrillus_leucophaeus_scaffold79	33064551	33065030	HERVK_5LTR	+
Mandrillus_leucophaeus_scaffold79	33065927	33066736	HERVK_pro	+
Mandrillus_leucophaeus_scaffold79	33066637	33068379	HERVK_pol	+

Mandrillus_leucophaeus_scaffold79	33069081	33069392	HERVK_env	+
Mandrillus_leucophaeus_scaffold79	33069491	33069981	HERVK_3LTR	+
Mandrillus_leucophaeus_scaffold96	42536009	42536335	HERVHF_5LTR	-
Mandrillus_leucophaeus_scaffold96	42536625	42536987	HERVHF_env	-
Mandrillus_leucophaeus_scaffold96	42537276	42537980	HERVHF_pol	-
Mandrillus_leucophaeus_scaffold96	42540209	42540527	HERVHF_3LTR	-
Mandrillus_leucophaeus_scaffold96	47203157	47203541	HERVHF_5LTR	-
Mandrillus_leucophaeus_scaffold96	47203996	47205961	HERVHF_pol	-
Mandrillus_leucophaeus_scaffold96	47206272	47206610	HERVHF_gag	-
Mandrillus_leucophaeus_scaffold96	47209505	47209868	HERVHF_3LTR	-
Mandrillus_leucophaeus_scaffold97	2248627	2248964	HERVK_5LTR	-
Mandrillus_leucophaeus_scaffold97	2250339	2252246	HERVK_pol	-
Mandrillus_leucophaeus_scaffold97	2253006	2253338	HERVK_pro	-
Mandrillus_leucophaeus_scaffold97	2253801	2254310	HERVK_gag	-
Mandrillus_leucophaeus_scaffold97	2254788	2255131	HERVK_3LTR	-
Mandrillus_leucophaeus_scaffold97	4244945	4245407	HERVHF_5LTR	-
Mandrillus_leucophaeus_scaffold97	4245636	4246442	HERVHF_env	-
Mandrillus_leucophaeus_scaffold97	4247384	4249864	HERVHF_pol	-
Mandrillus_leucophaeus_scaffold97	4249166	4250107	HERVHF_pro	-
Mandrillus_leucophaeus_scaffold97	4250193	4250714	HERVHF_gag	-
Mandrillus_leucophaeus_scaffold97	4252194	4252656	HERVHF_3LTR	-
Mandrillus_leucophaeus_scaffold97	55810199	55810610	HERVHF_5LTR	+
Mandrillus_leucophaeus_scaffold97	55812011	55812451	HERVHF_gag	+
Mandrillus_leucophaeus_scaffold97	55812456	55813403	HERVHF_pro	+
Mandrillus_leucophaeus_scaffold97	55812938	55815276	HERVHF_pol	+
Mandrillus_leucophaeus_scaffold97	55815404	55816059	HERVHF_env	+
Mandrillus_leucophaeus_scaffold97	55816170	55816588	HERVHF_3LTR	+
Mandrillus_sphinx_Contig1	74336656	74337008	HERVK_5LTR	+
Mandrillus_sphinx_Contig1	74337956	74338855	HERVK_pro	+
Mandrillus_sphinx_Contig1	74338750	74340497	HERVK_pol	+
Mandrillus_sphinx_Contig1	74341742	74342082	HERVK_3LTR	+
Mandrillus_sphinx_Contig10	26850493	26851226	HERVHF_5LTR	-
Mandrillus_sphinx_Contig10	26851625	26852206	HERVHF_pol	-
Mandrillus_sphinx_Contig10	26859196	26859924	HERVHF_3LTR	-
Mandrillus_sphinx_Contig10	38608225	38608526	HERVHF_5LTR	-
Mandrillus_sphinx_Contig10	38609316	38610941	HERVHF_pol	-
Mandrillus_sphinx_Contig10	38613269	38613577	HERVHF_3LTR	-
Mandrillus_sphinx_Contig101	2332955	2333254	HERVHF_5LTR	-
Mandrillus_sphinx_Contig101	2334092	2335605	HERVHF_pol	-
Mandrillus_sphinx_Contig101	2336144	2336521	HERVHF_gag	-
Mandrillus_sphinx_Contig101	2338273	2338575	HERVHF_3LTR	-
Mandrillus_sphinx_Contig105	3020251	3020711	HERVHF_5LTR	+
Mandrillus_sphinx_Contig105	3022759	3023688	HERVHF_pro	+
Mandrillus_sphinx_Contig105	3022936	3024673	HERVHF_pol	+

Mandrillus_sphinx_Contig105	3025471	3025922	HERVHF_3LTR	+
Mandrillus_sphinx_Contig113	3513826	3514136	HSERVIII_5LTR	-
Mandrillus_sphinx_Contig113	3520248	3523093	HSERVIII_pol	-
Mandrillus_sphinx_Contig113	3528234	3528540	HSERVIII_3LTR	-
Mandrillus_sphinx_Contig122	4604486	4604884	HERVHF_5LTR	-
Mandrillus_sphinx_Contig122	4605358	4607301	HERVHF_pol	-
Mandrillus_sphinx_Contig122	4606897	4607655	HERVHF_pro	-
Mandrillus_sphinx_Contig122	4610567	4610962	HERVHF_3LTR	-
Mandrillus_sphinx_Contig156	1449025	1449343	HSERVIII_5LTR	+
Mandrillus_sphinx_Contig156	1452159	1454762	HSERVIII_pol	+
Mandrillus_sphinx_Contig156	1456191	1456511	HSERVIII_3LTR	+
Mandrillus_sphinx_Contig19	28245875	28246286	HERVHF_5LTR	-
Mandrillus_sphinx_Contig19	28249080	28251934	HERVHF_pol	-
Mandrillus_sphinx_Contig19	28252396	28252956	HERVHF_gag	-
Mandrillus_sphinx_Contig19	28254682	28255095	HERVHF_3LTR	-
Mandrillus_sphinx_Contig27	21017197	21017529	HERVHF_5LTR	-
Mandrillus_sphinx_Contig27	21017593	21017916	HERVHF_env	-
Mandrillus_sphinx_Contig27	21019375	21020264	HERVHF_pol	-
Mandrillus_sphinx_Contig27	21019905	21020723	HERVHF_pro	-
Mandrillus_sphinx_Contig27	21020823	21021332	HERVHF_gag	-
Mandrillus_sphinx_Contig27	21023287	21023614	HERVHF_3LTR	-
Mandrillus_sphinx_Contig3	43917860	43918186	HERVHF_5LTR	-
Mandrillus_sphinx_Contig3	43918476	43918838	HERVHF_env	-
Mandrillus_sphinx_Contig3	43919127	43919831	HERVHF_pol	-
Mandrillus_sphinx_Contig3	43922103	43922421	HERVHF_3LTR	-
Mandrillus_sphinx_Contig3	48694894	48695229	HERVHF_5LTR	-
Mandrillus_sphinx_Contig3	48695793	48697887	HERVHF_pol	-
Mandrillus_sphinx_Contig3	48698045	48698383	HERVHF_gag	-
Mandrillus_sphinx_Contig3	48701282	48701626	HERVHF_3LTR	-
Mandrillus_sphinx_Contig3	63399060	63399513	HERVHF_5LTR	-
Mandrillus_sphinx_Contig3	63400622	63402183	HERVHF_pol	-
Mandrillus_sphinx_Contig3	63401830	63402348	HERVHF_pro	-
Mandrillus_sphinx_Contig3	63402654	63402959	HERVHF_gag	-
Mandrillus_sphinx_Contig3	63404586	63405031	HERVHF_3LTR	-
Mandrillus_sphinx_Contig3	71365212	71365553	HUERSP_5LTR	-
Mandrillus_sphinx_Contig3	71370571	71371220	HUERSP_pol	-
Mandrillus_sphinx_Contig3	71371918	71372616	HUERSP_gag	-
Mandrillus_sphinx_Contig3	71374592	71374936	HUERSP_3LTR	-
Mandrillus_sphinx_Contig35	3491117	3491463	HERVHF_5LTR	+
Mandrillus_sphinx_Contig35	3493775	3495655	HERVHF_pol	+
Mandrillus_sphinx_Contig35	3496343	3496694	HERVHF_3LTR	+
Mandrillus_sphinx_Contig5	28445271	28445651	HERVHF_5LTR	+
Mandrillus_sphinx_Contig5	28447371	28447691	HERVHF_gag	+
Mandrillus_sphinx_Contig5	28447681	28448160	HERVHF_pro	+

Mandrillus_sphinx_Contig5	28448179	28449400	HERVHF_pol	+
Mandrillus_sphinx_Contig5	28450516	28450906	HERVHF_3LTR	+
Mandrillus_sphinx_Contig51	10878947	10879419	HERVHF_5LTR	-
Mandrillus_sphinx_Contig51	10879850	10881873	HERVHF_pol	-
Mandrillus_sphinx_Contig51	10881292	10882071	HERVHF_pro	-
Mandrillus_sphinx_Contig51	10884072	10884539	HERVHF_3LTR	-
Mandrillus_sphinx_Contig55	1818322	1818643	HERVHF_5LTR	-
Mandrillus_sphinx_Contig55	1819467	1821578	HERVHF_pol	-
Mandrillus_sphinx_Contig55	1821120	1821635	HERVHF_pro	-
Mandrillus_sphinx_Contig55	1824245	1824550	HERVHF_3LTR	-
Mandrillus_sphinx_Contig6	20583725	20584245	HSERVIII_5LTR	-
Mandrillus_sphinx_Contig6	20584448	20585083	HSERVIII_pol	-
Mandrillus_sphinx_Contig6	20585671	20586194	HSERVIII_3LTR	-
Mandrillus_sphinx_Contig6	48124440	48124854	HERVHF_5LTR	+
Mandrillus_sphinx_Contig6	48126204	48126533	HERVHF_gag	+
Mandrillus_sphinx_Contig6	48126704	48127480	HERVHF_pro	+
Mandrillus_sphinx_Contig6	48126944	48128957	HERVHF_pol	+
Mandrillus_sphinx_Contig6	48129881	48130306	HERVHF_3LTR	+
Mandrillus_sphinx_Contig69	3048648	3049084	HERVHF_5LTR	+
Mandrillus_sphinx_Contig69	3050412	3051077	HERVHF_gag	+
Mandrillus_sphinx_Contig69	3050937	3051731	HERVHF_pro	+
Mandrillus_sphinx_Contig69	3051141	3053621	HERVHF_pol	+
Mandrillus_sphinx_Contig69	3053957	3054325	HERVHF_env	+
Mandrillus_sphinx_Contig69	3054485	3054914	HERVHF_3LTR	+
Mandrillus_sphinx_Contig7	7414455	7414767	HERVHF_5LTR	-
Mandrillus_sphinx_Contig7	7415518	7417331	HERVHF_pol	-
Mandrillus_sphinx_Contig7	7417804	7418205	HERVHF_gag	-
Mandrillus_sphinx_Contig7	7419637	7419945	HERVHF_3LTR	-
Mandrillus_sphinx_Contig70	3296547	3296943	HERVHF_5LTR	-
Mandrillus_sphinx_Contig70	3297597	3299940	HERVHF_pol	-
Mandrillus_sphinx_Contig70	3299368	3300231	HERVHF_pro	-
Mandrillus_sphinx_Contig70	3302434	3302831	HERVHF_3LTR	-
Mandrillus_sphinx_Contig74	5907771	5908156	HERVHF_5LTR	-
Mandrillus_sphinx_Contig74	5908447	5908916	HERVHF_env	-
Mandrillus_sphinx_Contig74	5908925	5911984	HERVHF_pol	-
Mandrillus_sphinx_Contig74	5912590	5913075	HERVHF_gag	-
Mandrillus_sphinx_Contig74	5914640	5915029	HERVHF_3LTR	-
Mandrillus_sphinx_Contig8	7451183	7451673	HERVK_5LTR	-
Mandrillus_sphinx_Contig8	7451774	7452112	HERVK_env	-
Mandrillus_sphinx_Contig8	7452619	7454529	HERVK_pol	-
Mandrillus_sphinx_Contig8	7454430	7455239	HERVK_pro	-
Mandrillus_sphinx_Contig8	7456134	7456613	HERVK_3LTR	-
Mandrillus_sphinx_Contig8	16678059	16678576	HERVK_5LTR	+
Mandrillus_sphinx_Contig8	16679075	16679998	HERVK_gag	+

Mandrillus_sphinx_Contig8	16679976	16680885	HERVK_pro	+
Mandrillus_sphinx_Contig8	16680780	16682066	HERVK_pol	+
Mandrillus_sphinx_Contig8	16682497	16682847	HERVK_env	+
Mandrillus_sphinx_Contig8	16682924	16683438	HERVK_3LTR	+
Mandrillus_sphinx_Contig86	6594449	6594906	HERVK_5LTR	-
Mandrillus_sphinx_Contig86	6594977	6595288	HERVK_env	-
Mandrillus_sphinx_Contig86	6595824	6596885	HERVK_pol	-
Mandrillus_sphinx_Contig86	6596950	6597822	HERVK_pro	-
Mandrillus_sphinx_Contig86	6598600	6599065	HERVK_3LTR	-
Mandrillus_sphinx_Contig88	2717701	2718071	HERVK_5LTR	+
Mandrillus_sphinx_Contig88	2719481	2720038	HERVK_gag	+
Mandrillus_sphinx_Contig88	2719655	2720554	HERVK_pro	+
Mandrillus_sphinx_Contig88	2720449	2721708	HERVK_pol	+
Mandrillus_sphinx_Contig88	2723283	2723661	HERVK_3LTR	+
Mandrillus_sphinx_Contig99	8316616	8316964	HERVK_5LTR	-
Mandrillus_sphinx_Contig99	8318346	8320252	HERVK_pol	-
Mandrillus_sphinx_Contig99	8320263	8320955	HERVK_pro	-
Mandrillus_sphinx_Contig99	8322466	8322804	HERVK_3LTR	-
Microcebus_murinus_CM007676.1	53132794	53133099	Unknown_HERV_5LTR	-
Microcebus_murinus_CM007676.1	53135607	53136293	Unknown_HERV_pol	-
Microcebus_murinus_CM007676.1	53145546	53145853	Unknown_HERV_3LTR	-
Microcebus_murinus_CM007693.1	20082129	20082469	Unknown_HERV_5LTR	-
Microcebus_murinus_CM007693.1	20085607	20086996	Unknown_HERV_pol	-
Microcebus_murinus_CM007693.1	20092654	20092993	Unknown_HERV_3LTR	-
Microcebus_murinus_CM007693.1	67280892	67281471	HERVIPADP_5LTR	+
Microcebus_murinus_CM007693.1	67282948	67285059	HERVIPADP_pol	+
Microcebus_murinus_CM007693.1	67288770	67289351	HERVIPADP_3LTR	+
Nomascus_leucogenys_tarseq_0	10127461	10127902	HUERSP_5LTR	-
Nomascus_leucogenys_tarseq_0	10129505	10131689	HUERSP_pol	-
Nomascus_leucogenys_tarseq_0	10132637	10133293	HUERSP_gag	-
Nomascus_leucogenys_tarseq_0	10135453	10135899	HUERSP_3LTR	-
Nomascus_leucogenys_tarseq_1	13138765	13139179	HERVHF_5LTR	+
Nomascus_leucogenys_tarseq_1	13140493	13141350	HERVHF_gag	+
Nomascus_leucogenys_tarseq_1	13142189	13142596	HERVHF_pol	+
Nomascus_leucogenys_tarseq_1	13142778	13143161	HERVHF_env	+
Nomascus_leucogenys_tarseq_1	13143883	13144297	HERVHF_3LTR	+
Nomascus_leucogenys_tarseq_103	98094	98532	HERVIPADP_5LTR	+
Nomascus_leucogenys_tarseq_103	100851	105113	HERVIPADP_pol	+
Nomascus_leucogenys_tarseq_103	107020	107388	HERVIPADP_env	+
Nomascus_leucogenys_tarseq_103	107621	108056	HERVIPADP_3LTR	+
Nomascus_leucogenys_tarseq_11	22159819	22160130	HERVHF_5LTR	-
Nomascus_leucogenys_tarseq_11	22162619	22163383	HERVHF_pol	-
Nomascus_leucogenys_tarseq_11	22163904	22164521	HERVHF_gag	-
Nomascus_leucogenys_tarseq_11	22165401	22165711	HERVHF_3LTR	-

Nomascus_leucogenys_tarseq_112	20931278	20931679	HSERVIII_5LTR	-
Nomascus_leucogenys_tarseq_112	20932434	20934771	HSERVIII_pol	-
Nomascus_leucogenys_tarseq_112	20936783	20937183	HSERVIII_3LTR	-
Nomascus_leucogenys_tarseq_12	6851265	6851714	HERVHF_5LTR	+
Nomascus_leucogenys_tarseq_12	6853712	6854728	HERVHF_pro	+
Nomascus_leucogenys_tarseq_12	6853967	6855889	HERVHF_pol	+
Nomascus_leucogenys_tarseq_12	6856257	6856595	HERVHF_env	+
Nomascus_leucogenys_tarseq_12	6856760	6857211	HERVHF_3LTR	+
Nomascus_leucogenys_tarseq_138	3850784	3851456	HSERVIII_5LTR	-
Nomascus_leucogenys_tarseq_138	3856533	3859338	HSERVIII_pol	-
Nomascus_leucogenys_tarseq_138	3863272	3863945	HSERVIII_3LTR	-
Nomascus_leucogenys_tarseq_140	2518488	2518899	HERVHF_5LTR	+
Nomascus_leucogenys_tarseq_140	2521301	2522984	HERVHF_pol	+
Nomascus_leucogenys_tarseq_140	2523628	2524039	HERVHF_3LTR	+
Nomascus_leucogenys_tarseq_147	724363	724751	HERVK_5LTR	-
Nomascus_leucogenys_tarseq_147	724921	725392	HERVK_env	-
Nomascus_leucogenys_tarseq_147	726350	727632	HERVK_pol	-
Nomascus_leucogenys_tarseq_147	728781	729686	HERVK_pro	-
Nomascus_leucogenys_tarseq_147	729754	730434	HERVK_gag	-
Nomascus_leucogenys_tarseq_147	731247	731626	HERVK_3LTR	-
Nomascus_leucogenys_tarseq_154	1830457	1830901	HSERVIII_5LTR	+
Nomascus_leucogenys_tarseq_154	1837612	1838151	HSERVIII_pol	+
Nomascus_leucogenys_tarseq_154	1839032	1839480	HSERVIII_3LTR	+
Nomascus_leucogenys_tarseq_16	2498582	2498994	HERVHF_5LTR	+
Nomascus_leucogenys_tarseq_16	2500972	2501352	HERVHF_gag	+
Nomascus_leucogenys_tarseq_16	2501385	2503461	HERVHF_pro	+
Nomascus_leucogenys_tarseq_16	2502919	2504868	HERVHF_pol	+
Nomascus_leucogenys_tarseq_16	2505439	2505853	HERVHF_3LTR	+
Nomascus_leucogenys_tarseq_25	6837678	6838064	HERVIPADP_5LTR	-
Nomascus_leucogenys_tarseq_25	6838250	6839104	HERVIPADP_env	-
Nomascus_leucogenys_tarseq_25	6840464	6843279	HERVIPADP_pol	-
Nomascus_leucogenys_tarseq_25	6843980	6844504	HERVIPADP_gag	-
Nomascus_leucogenys_tarseq_25	6845946	6846330	HERVIPADP_3LTR	-
Nomascus_leucogenys_tarseq_260	7390828	7391281	HERVHF_5LTR	+
Nomascus_leucogenys_tarseq_260	7392694	7392993	HERVHF_gag	+
Nomascus_leucogenys_tarseq_260	7393071	7393841	HERVHF_pro	+
Nomascus_leucogenys_tarseq_260	7393263	7394402	HERVHF_pol	+
Nomascus_leucogenys_tarseq_260	7396195	7397184	HERVHF_env	+
Nomascus_leucogenys_tarseq_260	7397349	7397800	HERVHF_3LTR	+
Nomascus_leucogenys_tarseq_3	2102209	2102551	HERVHF_5LTR	+
Nomascus_leucogenys_tarseq_3	2105144	2105725	HERVHF_pol	+
Nomascus_leucogenys_tarseq_3	2107388	2107731	HERVHF_3LTR	+
Nomascus_leucogenys_tarseq_3	21415972	21416385	HERVHF_5LTR	-
Nomascus_leucogenys_tarseq_3	21416975	21418914	HERVHF_pol	-

Nomascus_leucogenys_tarseq_3	21418372	21419190	HERVHF_pro	-
Nomascus_leucogenys_tarseq_3	21420583	21420998	HERVHF_3LTR	-
Nomascus_leucogenys_tarseq_31	15346712	15347162	HERVHF_5LTR	+
Nomascus_leucogenys_tarseq_31	15349108	15349431	HERVHF_gag	+
Nomascus_leucogenys_tarseq_31	15349757	15351396	HERVHF_pol	+
Nomascus_leucogenys_tarseq_31	15352192	15352644	HERVHF_3LTR	+
Nomascus_leucogenys_tarseq_33	6011756	6012109	HERVK_5LTR	-
Nomascus_leucogenys_tarseq_33	6012182	6012520	HERVK_env	-
Nomascus_leucogenys_tarseq_33	6013020	6014794	HERVK_pol	-
Nomascus_leucogenys_tarseq_33	6014904	6015674	HERVK_pro	-
Nomascus_leucogenys_tarseq_33	6017361	6017704	HERVK_3LTR	-
Nomascus_leucogenys_tarseq_37	3719348	3719832	HERVK_5LTR	+
Nomascus_leucogenys_tarseq_37	3720706	3721170	HERVK_gag	+
Nomascus_leucogenys_tarseq_37	3721273	3722176	HERVK_pro	+
Nomascus_leucogenys_tarseq_37	3722080	3722832	HERVK_pol	+
Nomascus_leucogenys_tarseq_37	3724068	3724556	HERVK_3LTR	+
Nomascus_leucogenys_tarseq_41	11852484	11852895	HERVHF_5LTR	+
Nomascus_leucogenys_tarseq_41	11854291	11854839	HERVHF_gag	+
Nomascus_leucogenys_tarseq_41	11854999	11855703	HERVHF_pro	+
Nomascus_leucogenys_tarseq_41	11855065	11857276	HERVHF_pol	+
Nomascus_leucogenys_tarseq_41	11857339	11857872	HERVHF_env	+
Nomascus_leucogenys_tarseq_41	11858035	11858448	HERVHF_3LTR	+
Nomascus_leucogenys_tarseq_41	12515322	12515678	HERVHF_5LTR	+
Nomascus_leucogenys_tarseq_41	12517407	12517706	HERVHF_gag	+
Nomascus_leucogenys_tarseq_41	12518231	12520189	HERVHF_pol	+
Nomascus_leucogenys_tarseq_41	12520739	12521088	HERVHF_3LTR	+
Nomascus_leucogenys_tarseq_430	3167345	3167660	HERVK_5LTR	-
Nomascus_leucogenys_tarseq_430	3167667	3168072	HERVK_env	-
Nomascus_leucogenys_tarseq_430	3169009	3170484	HERVK_pol	-
Nomascus_leucogenys_tarseq_430	3170423	3171348	HERVK_pro	-
Nomascus_leucogenys_tarseq_430	3171723	3172283	HERVK_gag	-
Nomascus_leucogenys_tarseq_430	3172917	3173233	HERVK_3LTR	-
Nomascus_leucogenys_tarseq_44	14090159	14090461	HERVHF_5LTR	+
Nomascus_leucogenys_tarseq_44	14092590	14093147	HERVHF_pro	+
Nomascus_leucogenys_tarseq_44	14093164	14095821	HERVHF_pol	+
Nomascus_leucogenys_tarseq_44	14096364	14096668	HERVHF_3LTR	+
Nomascus_leucogenys_tarseq_44	22523800	22524208	HERVK_5LTR	+
Nomascus_leucogenys_tarseq_44	22525636	22526564	HERVK_pro	+
Nomascus_leucogenys_tarseq_44	22526459	22527772	HERVK_pol	+
Nomascus_leucogenys_tarseq_44	22529569	22529976	HERVK_3LTR	+
Nomascus_leucogenys_tarseq_51	7176387	7176697	HERVHF_5LTR	+
Nomascus_leucogenys_tarseq_51	7178050	7178478	HERVHF_pro	+
Nomascus_leucogenys_tarseq_51	7178657	7179277	HERVHF_pol	+
Nomascus_leucogenys_tarseq_51	7182126	7182436	HERVHF_3LTR	+

Nomascus_leucogenys_tarseq_9 7417553 7417965 HERVHF_5LTR -
Nomascus_leucogenys_tarseq_9 7418590 7420176 HERVHF_pol -
Nomascus_leucogenys_tarseq_9 7419898 7420713 HERVHF_pro -
Nomascus_leucogenys_tarseq_9 7420823 7421206 HERVHF_gag -
Nomascus_leucogenys_tarseq_9 7422728 7423147 HERVHF_3LTR -
Nomascus_leucogenys_tarseq_9 21485266 21485565 HSERVIII_5LTR +
Nomascus_leucogenys_tarseq_9 21487357 21487830 HSERVIII_pol +
Nomascus_leucogenys_tarseq_9 21490874 21491178 HSERVIII_3LTR +
Nycticebus_bengalensis_LG02 100642868 100643579 Unknown_HERV_5LTR -

Nycticebus_bengalensis_LG02 100645912 100649249 Unknown_HERV_pol -

Nycticebus_bengalensis_LG02 100649115 100650137 Unknown_HERV_pro -

Nycticebus_bengalensis_LG02 100652088 100652802 Unknown_HERV_3LTR -

Nycticebus_bengalensis_LG02 102672581 102672897 HERVHF_5LTR -
Nycticebus_bengalensis_LG02 102674383 102676483 HERVHF_pol -
Nycticebus_bengalensis_LG02 102677458 102677958 HERVHF_gag -
Nycticebus_bengalensis_LG02 102679344 102679654 HERVHF_3LTR -
Nycticebus_pygmaeus_lh.2rd_polish_hic_scaffold_20 41529627 41530007 Unknown_HERV_5LTR +
Nycticebus_pygmaeus_lh.2rd_polish_hic_scaffold_20 41531444 41532874 Unknown_HERV_pol +
Nycticebus_pygmaeus_lh.2rd_polish_hic_scaffold_20 41533507 41533857 Unknown_HERV_env +
Nycticebus_pygmaeus_lh.2rd_polish_hic_scaffold_20 41534089 41534469 Unknown_HERV_3LTR +
Nycticebus_pygmaeus_lh.2rd_polish_hic_scaffold_3 34740973 34741848 HERVHF_5LTR -

Nycticebus_pygmaeus_lh.2rd_polish_hic_scaffold_3 34742729 34745132 HERVHF_pol -

Nycticebus_pygmaeus_lh.2rd_polish_hic_scaffold_3 34744952 34745503 HERVHF_pro -

Nycticebus_pygmaeus_lh.2rd_polish_hic_scaffold_3 34751113 34751996 HERVHF_3LTR -

Nycticebus_pygmaeus_lh.2rd_polish_hic_scaffold_9 29265859 29266248 Unknown_HERV_5LTR +
Nycticebus_pygmaeus_lh.2rd_polish_hic_scaffold_9 29269000 29270731 Unknown_HERV_gag +
Nycticebus_pygmaeus_lh.2rd_polish_hic_scaffold_9 29270948 29271274 Unknown_HERV_pro +
Nycticebus_pygmaeus_lh.2rd_polish_hic_scaffold_9 29271618 29273634 Unknown_HERV_pol +

Nycticebus_pygmaeus_lh.2rd_polish_hic_scaffold_9 29274373 29274762 Unknown_HERV_3LTR +

Otolemur_garnettii_GL873590.1 6207717 6208066 HERVW9_5LTR -

Otolemur_garnettii_GL873590.1 6208618 6211341 HERVW9_pol -

Otolemur_garnettii_GL873590.1 6211350 6212042 HERVW9_pro -

Otolemur_garnettii_GL873590.1 6212285 6212881 HERVW9_gag -

Otolemur_garnettii_GL873590.1 6215297 6215648 HERVW9_3LTR -

Otolemur_garnettii_GL873777.1 1217236 1217546 HERVHF_5LTR -

Otolemur_garnettii_GL873777.1 1218437 1220680 HERVHF_pol -

Otolemur_garnettii_GL873777.1 1219952 1220929 HERVHF_pro -

Otolemur_garnettii_GL873777.1 1223463 1223775 HERVHF_3LTR -

Pan_paniscus_CM003383.1 4912654 4912982 HERVHF_5LTR +

Pan_paniscus_CM003383.1 4913544 4914049 HERVHF_gag +

Pan_paniscus_CM003383.1 4915301 4917102 HERVHF_pol +

Pan_paniscus_CM003383.1 4918175 4918511 HERVHF_3LTR +

Pan_paniscus_CM003383.1 16233726 16234240 HERVK_5LTR -

Pan_paniscus_CM003383.1 16235456 16236956 HERVK_pol -

Pan_paniscus_CM003383.1 16236854 16237795 HERVK_pro -

Pan_paniscus_CM003383.1 16239199 16239715 HERVK_3LTR -

Pan_paniscus_CM003383.1 23130769 23131193 HERVHF_5LTR -

Pan_paniscus_CM003383.1 23131361 23131711 HERVHF_env -

Pan_paniscus_CM003383.1 23132773 23135184 HERVHF_pol -

Pan_paniscus_CM003383.1 23134754 23135449 HERVHF_pro -

Pan_paniscus_CM003383.1 23135523 23135846 HERVHF_gag -

Pan_paniscus_CM003383.1 23137303 23137718 HERVHF_3LTR -

Pan_paniscus_CM003383.1 71586511 71586893 HERVHF_5LTR +

Pan_paniscus_CM003383.1 71588315 71588749 HERVHF_gag +

Pan_paniscus_CM003383.1 71588798 71589322 HERVHF_pro +

Pan_paniscus_CM003383.1 71589213 71591833 HERVHF_pol +

Pan_paniscus_CM003383.1 71591934 71592290 HERVHF_env +

Pan_paniscus_CM003383.1 71592454 71592827 HERVHF_3LTR +

Pan_paniscus_CM003383.1 78195859 78196266 HERVHF_5LTR -

Pan_paniscus_CM003383.1 78196827 78198597 HERVHF_pol -

Pan_paniscus_CM003383.1 78198170 78199030 HERVHF_pro -

Pan_paniscus_CM003383.1 78199037 78199357 HERVHF_gag -

Pan_paniscus_CM003383.1 78200970 78201393 HERVHF_3LTR -

Pan_paniscus_CM003383.1 80218595 80218929 HERVHF_5LTR +

Pan_paniscus_CM003383.1 80220477 80221073 HERVHF_gag +

Pan_paniscus_CM003383.1 80221125 80221889 HERVHF_pro +

Pan_paniscus_CM003383.1 80221368 80223777 HERVHF_pol +

Pan_paniscus_CM003383.1 80225856 80226571 HERVHF_env +

Pan_paniscus_CM003383.1 80226889 80227225 HERVHF_3LTR +

Pan_paniscus_CM003383.1 83676068 83676465 HERVHF_5LTR -

Pan_paniscus_CM003383.1 83677532 83679328 HERVHF_pol -

Pan_paniscus_CM003383.1	83678786	83679451	HERVHF_pro	-
Pan_paniscus_CM003383.1	83679625	83679981	HERVHF_gag	-
Pan_paniscus_CM003383.1	83681664	83682061	HERVHF_3LTR	-
Pan_paniscus_CM003383.1	95956544	95956851	HERVHF_5LTR	-
Pan_paniscus_CM003383.1	95957587	95959898	HERVHF_pol	-
Pan_paniscus_CM003383.1	95959356	95960060	HERVHF_pro	-
Pan_paniscus_CM003383.1	95962448	95962754	HERVHF_3LTR	-
Pan_paniscus_CM003383.1	185783185	185783630	HERVK_5LTR	+
Pan_paniscus_CM003383.1	185785129	185786045	HERVK_pro	+
Pan_paniscus_CM003383.1	185785940	185787208	HERVK_pol	+
Pan_paniscus_CM003383.1	185788130	185788582	HERVK_3LTR	+
Pan_paniscus_CM003383.1	195835107	195835555	HERVHF_5LTR	-
Pan_paniscus_CM003383.1	195836160	195837782	HERVHF_pol	-
Pan_paniscus_CM003383.1	195837739	195838197	HERVHF_pro	-
Pan_paniscus_CM003383.1	195840269	195840718	HERVHF_3LTR	-
Pan_paniscus_CM003383.1	209751527	209751973	HERVHF_5LTR	+
Pan_paniscus_CM003383.1	209754539	209754943	HERVHF_gag	+
Pan_paniscus_CM003383.1	209755179	209755751	HERVHF_pro	+
Pan_paniscus_CM003383.1	209755233	209757367	HERVHF_pol	+
Pan_paniscus_CM003383.1	209757889	209758329	HERVHF_3LTR	+
Pan_paniscus_CM003383.1	221977301	221977703	HERVHF_5LTR	+
Pan_paniscus_CM003383.1	221979552	221979857	HERVHF_gag	+
Pan_paniscus_CM003383.1	221980290	221982472	HERVHF_pol	+
Pan_paniscus_CM003383.1	221982891	221983293	HERVHF_3LTR	+
Pan_paniscus_CM003384.1	20184708	20185141	HERVHF_5LTR	+
Pan_paniscus_CM003384.1	20186622	20186951	HERVHF_gag	+
Pan_paniscus_CM003384.1	20186944	20187702	HERVHF_pro	+
Pan_paniscus_CM003384.1	20187443	20189388	HERVHF_pol	+
Pan_paniscus_CM003384.1	20189798	20190209	HERVHF_3LTR	+
Pan_paniscus_CM003384.1	38864068	38864479	HERVHF_5LTR	-
Pan_paniscus_CM003384.1	38864489	38864850	HERVHF_env	-
Pan_paniscus_CM003384.1	38865020	38867001	HERVHF_pol	-
Pan_paniscus_CM003384.1	38866465	38867250	HERVHF_pro	-
Pan_paniscus_CM003384.1	38867391	38867711	HERVHF_gag	-
Pan_paniscus_CM003384.1	38869412	38869813	HERVHF_3LTR	-
Pan_paniscus_CM003384.1	42334505	42334936	HERVHF_5LTR	+
Pan_paniscus_CM003384.1	42335712	42337075	HERVHF_gag	+
Pan_paniscus_CM003384.1	42337085	42337615	HERVHF_pro	+
Pan_paniscus_CM003384.1	42338083	42341031	HERVHF_pol	+
Pan_paniscus_CM003384.1	42341198	42341521	HERVHF_env	+
Pan_paniscus_CM003384.1	42341956	42342387	HERVHF_3LTR	+
Pan_paniscus_CM003384.1	68515613	68516012	HERVHF_5LTR	+
Pan_paniscus_CM003384.1	68517853	68518164	HERVHF_gag	+
Pan_paniscus_CM003384.1	68518449	68519189	HERVHF_pro	+

Pan_paniscus_CM003384.1	68518623	68520145	HERVHF_pol	+
Pan_paniscus_CM003384.1	68521323	68521723	HERVHF_3LTR	+
Pan_paniscus_CM003384.1	72705308	72705748	HERVHF_5LTR	-
Pan_paniscus_CM003384.1	72706646	72708165	HERVHF_pol	-
Pan_paniscus_CM003384.1	72707575	72708159	HERVHF_pro	-
Pan_paniscus_CM003384.1	72710418	72710856	HERVHF_3LTR	-
Pan_paniscus_CM003385.1	161223748	161224162	HERVHF_5LTR	-
Pan_paniscus_CM003385.1	161224768	161226350	HERVHF_pol	-
Pan_paniscus_CM003385.1	161226349	161226945	HERVHF_pro	-
Pan_paniscus_CM003385.1	161226979	161227299	HERVHF_gag	-
Pan_paniscus_CM003385.1	161229114	161229534	HERVHF_3LTR	-
Pan_paniscus_CM003385.1	175718812	175719218	HERVHF_5LTR	-
Pan_paniscus_CM003385.1	175719667	175721823	HERVHF_pol	-
Pan_paniscus_CM003385.1	175721449	175722168	HERVHF_pro	-
Pan_paniscus_CM003385.1	175724297	175724700	HERVHF_3LTR	-
Pan_paniscus_CM003385.1	213578704	213579008	HERVHF_5LTR	-
Pan_paniscus_CM003385.1	213579460	213581967	HERVHF_pol	-
Pan_paniscus_CM003385.1	213581353	213582033	HERVHF_pro	-
Pan_paniscus_CM003385.1	213584302	213584608	HERVHF_3LTR	-
Pan_paniscus_CM003385.1	214554812	214555124	HERVHF_5LTR	+
Pan_paniscus_CM003385.1	214563815	214564351	HERVHF_pro	+
Pan_paniscus_CM003385.1	214564422	214566629	HERVHF_pol	+
Pan_paniscus_CM003385.1	214567071	214567379	HERVHF_3LTR	+
Pan_paniscus_CM003385.1	220569612	220570071	HERVHF_5LTR	-
Pan_paniscus_CM003385.1	220570320	220572735	HERVHF_pol	-
Pan_paniscus_CM003385.1	220572770	220573285	HERVHF_pro	-
Pan_paniscus_CM003385.1	220575543	220575996	HERVHF_3LTR	-
Pan_paniscus_CM003385.1	220825791	220826128	HERVHF_5LTR	+
Pan_paniscus_CM003385.1	220828123	220828734	HERVHF_pro	+
Pan_paniscus_CM003385.1	220828306	220830058	HERVHF_pol	+
Pan_paniscus_CM003385.1	220831515	220831855	HERVHF_3LTR	+
Pan_paniscus_CM003386.1	931490	931904	HERVHF_5LTR	-
Pan_paniscus_CM003386.1	932331	934435	HERVHF_pol	-
Pan_paniscus_CM003386.1	933893	934726	HERVHF_pro	-
Pan_paniscus_CM003386.1	934730	935059	HERVHF_gag	-
Pan_paniscus_CM003386.1	936800	937217	HERVHF_3LTR	-
Pan_paniscus_CM003386.1	21438814	21439220	HERVHF_5LTR	-
Pan_paniscus_CM003386.1	21439667	21441813	HERVHF_pol	-
Pan_paniscus_CM003386.1	21441247	21442170	HERVHF_pro	-
Pan_paniscus_CM003386.1	21442227	21442550	HERVHF_gag	-
Pan_paniscus_CM003386.1	21443575	21443978	HERVHF_3LTR	-
Pan_paniscus_CM003386.1	71735709	71736121	HERVHF_5LTR	-
Pan_paniscus_CM003386.1	71736577	71738555	HERVHF_pol	-
Pan_paniscus_CM003386.1	71738304	71739128	HERVHF_pro	-

Pan_paniscus_CM003386.1	71739251	71739601	HERVHF_gag	-
Pan_paniscus_CM003386.1	71741117	71741535	HERVHF_3LTR	-
Pan_paniscus_CM003386.1	73317427	73317840	HERVHF_5LTR	+
Pan_paniscus_CM003386.1	73319292	73319735	HERVHF_gag	+
Pan_paniscus_CM003386.1	73319822	73320592	HERVHF_pro	+
Pan_paniscus_CM003386.1	73320029	73322469	HERVHF_pol	+
Pan_paniscus_CM003386.1	73322864	73323269	HERVHF_3LTR	+
Pan_paniscus_CM003386.1	92331191	92331609	HERVHF_5LTR	+
Pan_paniscus_CM003386.1	92331739	92332161	HERVHF_pro	+
Pan_paniscus_CM003386.1	92332223	92333948	HERVHF_pol	+
Pan_paniscus_CM003386.1	92334558	92334985	HERVHF_3LTR	+
Pan_paniscus_CM003386.1	171570100	171570422	HERVHF_5LTR	-
Pan_paniscus_CM003386.1	171570832	171573166	HERVHF_pol	-
Pan_paniscus_CM003386.1	171572624	171573409	HERVHF_pro	-
Pan_paniscus_CM003386.1	171573505	171573903	HERVHF_gag	-
Pan_paniscus_CM003386.1	171575516	171575840	HERVHF_3LTR	-
Pan_paniscus_CM003386.1	172503098	172503500	HERVHF_5LTR	+
Pan_paniscus_CM003386.1	172505463	172506347	HERVHF_pro	+
Pan_paniscus_CM003386.1	172505754	172508079	HERVHF_pol	+
Pan_paniscus_CM003386.1	172508495	172508900	HERVHF_3LTR	+
Pan_paniscus_CM003386.1	181554968	181555435	HERVHF_5LTR	-
Pan_paniscus_CM003386.1	181556034	181557890	HERVHF_pol	-
Pan_paniscus_CM003386.1	181557420	181557911	HERVHF_pro	-
Pan_paniscus_CM003386.1	181558475	181558807	HERVHF_gag	-
Pan_paniscus_CM003386.1	181560252	181560706	HERVHF_3LTR	-
Pan_paniscus_CM003387.1	16711924	16712236	HERVHF_5LTR	+
Pan_paniscus_CM003387.1	16713670	16714071	HERVHF_pro	+
Pan_paniscus_CM003387.1	16714350	16716565	HERVHF_pol	+
Pan_paniscus_CM003387.1	16717070	16717372	HERVHF_3LTR	+
Pan_paniscus_CM003387.1	89507156	89507618	HERVHF_5LTR	+
Pan_paniscus_CM003387.1	89509074	89509409	HERVHF_gag	+
Pan_paniscus_CM003387.1	89509564	89509977	HERVHF_pro	+
Pan_paniscus_CM003387.1	89509710	89512097	HERVHF_pol	+
Pan_paniscus_CM003387.1	89512503	89512964	HERVHF_3LTR	+
Pan_paniscus_CM003387.1	134221735	134222156	HERVHF_5LTR	+
Pan_paniscus_CM003387.1	134223738	134224148	HERVHF_gag	+
Pan_paniscus_CM003387.1	134224474	134226537	HERVHF_pol	+
Pan_paniscus_CM003387.1	134227195	134227611	HERVHF_3LTR	+
Pan_paniscus_CM003387.1	170818349	170818751	HERVHF_5LTR	+
Pan_paniscus_CM003387.1	170820324	170820647	HERVHF_gag	+
Pan_paniscus_CM003387.1	170820441	170821427	HERVHF_pro	+
Pan_paniscus_CM003387.1	170820942	170823174	HERVHF_pol	+
Pan_paniscus_CM003387.1	170823626	170824030	HERVHF_3LTR	+
Pan_paniscus_CM003387.1	174409788	174410202	HERVHF_5LTR	+

Pan_paniscus_CM003387.1	174412491	174413093	HERVHF_pro	+
Pan_paniscus_CM003387.1	174412665	174414588	HERVHF_pol	+
Pan_paniscus_CM003387.1	174415052	174415470	HERVHF_3LTR	+
Pan_paniscus_CM003387.1	177422094	177422413	HERVHF_5LTR	-
Pan_paniscus_CM003387.1	177423453	177425542	HERVHF_pol	-
Pan_paniscus_CM003387.1	177425135	177425716	HERVHF_pro	-
Pan_paniscus_CM003387.1	177426021	177426410	HERVHF_gag	-
Pan_paniscus_CM003387.1	177428007	177428307	HERVHF_3LTR	-
Pan_paniscus_CM003387.1	190618163	190618530	HERVHF_5LTR	-
Pan_paniscus_CM003387.1	190619077	190621270	HERVHF_pol	-
Pan_paniscus_CM003387.1	190621574	190621903	HERVHF_gag	-
Pan_paniscus_CM003387.1	190623406	190623775	HERVHF_3LTR	-
Pan_paniscus_CM003388.1	9120093	9120404	HERVHF_5LTR	-
Pan_paniscus_CM003388.1	9126494	9129236	HERVHF_env	-
Pan_paniscus_CM003388.1	9129946	9131597	HERVHF_pol	-
Pan_paniscus_CM003388.1	9130962	9131828	HERVHF_pro	-
Pan_paniscus_CM003388.1	9131921	9132232	HERVHF_gag	-
Pan_paniscus_CM003388.1	9132831	9133157	HERVHF_3LTR	-
Pan_paniscus_CM003388.1	38957065	38957485	HERVHF_5LTR	+
Pan_paniscus_CM003388.1	38959500	38960077	HERVHF_gag	+
Pan_paniscus_CM003388.1	38960309	38960923	HERVHF_pro	+
Pan_paniscus_CM003388.1	38960366	38962471	HERVHF_pol	+
Pan_paniscus_CM003388.1	38962870	38963226	HERVHF_env	+
Pan_paniscus_CM003388.1	38963391	38963805	HERVHF_3LTR	+
Pan_paniscus_CM003388.1	63355162	63355627	HERVK_5LTR	-
Pan_paniscus_CM003388.1	63356196	63358233	HERVK_pol	-
Pan_paniscus_CM003388.1	63357691	63358941	HERVK_pro	-
Pan_paniscus_CM003388.1	63360391	63360861	HERVK_3LTR	-
Pan_paniscus_CM003388.1	99873896	99874304	HERVHF_5LTR	-
Pan_paniscus_CM003388.1	99874498	99874890	HERVHF_env	-
Pan_paniscus_CM003388.1	99874953	99877220	HERVHF_pol	-
Pan_paniscus_CM003388.1	99876813	99877442	HERVHF_pro	-
Pan_paniscus_CM003388.1	99879566	99879970	HERVHF_3LTR	-
Pan_paniscus_CM003388.1	114871205	114871716	HERVK_5LTR	+
Pan_paniscus_CM003388.1	114872485	114873393	HERVK_pro	+
Pan_paniscus_CM003388.1	114873288	114874091	HERVK_pol	+
Pan_paniscus_CM003388.1	114874934	114875305	HERVK_env	+
Pan_paniscus_CM003388.1	114875401	114875901	HERVK_3LTR	+
Pan_paniscus_CM003388.1	138266311	138266643	HERVHF_5LTR	+
Pan_paniscus_CM003388.1	138268214	138268879	HERVHF_gag	+
Pan_paniscus_CM003388.1	138269385	138272908	HERVHF_pol	+
Pan_paniscus_CM003388.1	138273312	138274508	HERVHF_env	+
Pan_paniscus_CM003388.1	138274724	138275037	HERVHF_3LTR	+
Pan_paniscus_CM003389.1	6043543	6043973	HERVHF_5LTR	-

Pan_paniscus_CM003389.1	6044582	6046582	HERVHF_pol	-
Pan_paniscus_CM003389.1	6045944	6046693	HERVHF_pro	-
Pan_paniscus_CM003389.1	6046801	6047190	HERVHF_gag	-
Pan_paniscus_CM003389.1	6048829	6049264	HERVHF_3LTR	-
Pan_paniscus_CM003389.1	31971373	31971776	HERVIPADP_5LTR	-
Pan_paniscus_CM003389.1	31972106	31972857	HERVIPADP_env	-
Pan_paniscus_CM003389.1	31974314	31977462	HERVIPADP_pol	-
Pan_paniscus_CM003389.1	31978067	31978510	HERVIPADP_gag	-
Pan_paniscus_CM003389.1	31979893	31980304	HERVIPADP_3LTR	-
Pan_paniscus_CM003389.1	52755394	52755838	HERVHF_5LTR	+
Pan_paniscus_CM003389.1	52758066	52759940	HERVHF_pol	+
Pan_paniscus_CM003389.1	52760743	52761193	HERVHF_3LTR	+
Pan_paniscus_CM003389.1	64544332	64544782	HERVHF_5LTR	+
Pan_paniscus_CM003389.1	64546400	64546720	HERVHF_gag	+
Pan_paniscus_CM003389.1	64546730	64547578	HERVHF_pro	+
Pan_paniscus_CM003389.1	64546979	64549037	HERVHF_pol	+
Pan_paniscus_CM003389.1	64549970	64550421	HERVHF_3LTR	+
Pan_paniscus_CM003389.1	66893105	66893471	HERVHF_5LTR	+
Pan_paniscus_CM003389.1	66894929	66895234	HERVHF_gag	+
Pan_paniscus_CM003389.1	66895224	66895649	HERVHF_pro	+
Pan_paniscus_CM003389.1	66895688	66898434	HERVHF_pol	+
Pan_paniscus_CM003389.1	66898515	66898877	HERVHF_env	+
Pan_paniscus_CM003389.1	66899036	66899401	HERVHF_3LTR	+
Pan_paniscus_CM003389.1	78789775	78790238	HERVHF_5LTR	-
Pan_paniscus_CM003389.1	78790406	78791573	HERVHF_env	-
Pan_paniscus_CM003389.1	78792592	78794935	HERVHF_pol	-
Pan_paniscus_CM003389.1	78794925	78795392	HERVHF_pro	-
Pan_paniscus_CM003389.1	78795453	78795773	HERVHF_gag	-
Pan_paniscus_CM003389.1	78797546	78798007	HERVHF_3LTR	-
Pan_paniscus_CM003389.1	111702202	111702625	HERVHF_5LTR	-
Pan_paniscus_CM003389.1	111703174	111705032	HERVHF_pol	-
Pan_paniscus_CM003389.1	111704625	111705206	HERVHF_pro	-
Pan_paniscus_CM003389.1	111707526	111707940	HERVHF_3LTR	-
Pan_paniscus_CM003389.1	116899530	116899890	HERVHF_5LTR	+
Pan_paniscus_CM003389.1	116902184	116902525	HERVHF_pro	+
Pan_paniscus_CM003389.1	116902587	116904377	HERVHF_pol	+
Pan_paniscus_CM003389.1	116904804	116905160	HERVHF_3LTR	+
Pan_paniscus_CM003389.1	133310916	133311273	HERVHF_5LTR	+
Pan_paniscus_CM003389.1	133312865	133313278	HERVHF_gag	+
Pan_paniscus_CM003389.1	133313285	133314118	HERVHF_pro	+
Pan_paniscus_CM003389.1	133313576	133314881	HERVHF_pol	+
Pan_paniscus_CM003389.1	133316106	133316471	HERVHF_3LTR	+
Pan_paniscus_CM003390.1	111230566	111231025	HERVHF_5LTR	-
Pan_paniscus_CM003390.1	111231976	111234050	HERVHF_pol	-

Pan_paniscus_CM003390.1	111233786	111234451	HERVHF_pro	-
Pan_paniscus_CM003390.1	111236037	111236497	HERVHF_3LTR	-
Pan_paniscus_CM003390.1	127510252	127510704	HERVHF_5LTR	+
Pan_paniscus_CM003390.1	127512614	127512934	HERVHF_gag	+
Pan_paniscus_CM003390.1	127512941	127513525	HERVHF_pro	+
Pan_paniscus_CM003390.1	127513521	127515346	HERVHF_pol	+
Pan_paniscus_CM003390.1	127515768	127516220	HERVHF_3LTR	+
Pan_paniscus_CM003391.1	64069914	64070301	HERVHF_5LTR	+
Pan_paniscus_CM003391.1	64071974	64072306	HERVHF_gag	+
Pan_paniscus_CM003391.1	64072662	64073252	HERVHF_pro	+
Pan_paniscus_CM003391.1	64072863	64075524	HERVHF_pol	+
Pan_paniscus_CM003391.1	64076067	64076459	HERVHF_env	+
Pan_paniscus_CM003391.1	64076738	64077118	HERVHF_3LTR	+
Pan_paniscus_CM003391.1	131373330	131373744	HERVHF_5LTR	+
Pan_paniscus_CM003391.1	131375432	131375743	HERVHF_gag	+
Pan_paniscus_CM003391.1	131375886	131376596	HERVHF_pro	+
Pan_paniscus_CM003391.1	131376027	131378357	HERVHF_pol	+
Pan_paniscus_CM003391.1	131378808	131379220	HERVHF_3LTR	+
Pan_paniscus_CM003393.1	50814798	50815253	HERVHF_5LTR	-
Pan_paniscus_CM003393.1	50815675	50817615	HERVHF_pol	-
Pan_paniscus_CM003393.1	50820254	50820708	HERVHF_3LTR	-
Pan_paniscus_CM003393.1	54800482	54800818	HERVHF_5LTR	+
Pan_paniscus_CM003393.1	54802268	54802567	HERVHF_gag	+
Pan_paniscus_CM003393.1	54802620	54803396	HERVHF_pro	+
Pan_paniscus_CM003393.1	54803082	54804599	HERVHF_pol	+
Pan_paniscus_CM003393.1	54805495	54805832	HERVHF_3LTR	+
Pan_paniscus_CM003393.1	116502393	116502805	HERVHF_5LTR	+
Pan_paniscus_CM003393.1	116504480	116504860	HERVHF_gag	+
Pan_paniscus_CM003393.1	116504896	116505702	HERVHF_pro	+
Pan_paniscus_CM003393.1	116505181	116507425	HERVHF_pol	+
Pan_paniscus_CM003393.1	116507821	116508232	HERVHF_3LTR	+
Pan_paniscus_CM003394.1	14318115	14318551	HERVHF_5LTR	+
Pan_paniscus_CM003394.1	14319102	14321651	HERVHF_pol	+
Pan_paniscus_CM003394.1	14321968	14322327	HERVHF_env	+
Pan_paniscus_CM003394.1	14322493	14322919	HERVHF_3LTR	+
Pan_paniscus_CM003395.1	42157331	42157841	HERVK_5LTR	+
Pan_paniscus_CM003395.1	42158590	42159510	HERVK_pro	+
Pan_paniscus_CM003395.1	42159452	42161330	HERVK_pol	+
Pan_paniscus_CM003395.1	42161877	42162179	HERVK_env	+
Pan_paniscus_CM003395.1	42162257	42162768	HERVK_3LTR	+
Pan_paniscus_CM003395.1	60115240	60115546	HSERVIII_5LTR	-
Pan_paniscus_CM003395.1	60118592	60119209	HSERVIII_pol	-
Pan_paniscus_CM003395.1	60120289	60120590	HSERVIII_3LTR	-
Pan_paniscus_CM003395.1	70722833	70723247	HERVK_5LTR	-

Pan_paniscus_CM003395.1	70725079	70725549	HERVK_pol	-
Pan_paniscus_CM003395.1	70725917	70726300	HERVK_pro	-
Pan_paniscus_CM003395.1	70727621	70728046	HERVK_3LTR	-
Pan_paniscus_CM003395.1	81413805	81414212	HERVHF_5LTR	-
Pan_paniscus_CM003395.1	81414666	81416605	HERVHF_pol	-
Pan_paniscus_CM003395.1	81416228	81416779	HERVHF_pro	-
Pan_paniscus_CM003395.1	81419089	81419489	HERVHF_3LTR	-
Pan_paniscus_CM003395.1	104196467	104197380	HERVHF_5LTR	-
Pan_paniscus_CM003395.1	104198903	104200268	HERVHF_pol	-
Pan_paniscus_CM003395.1	104200183	104200851	HERVHF_pro	-
Pan_paniscus_CM003395.1	104200885	104201208	HERVHF_gag	-
Pan_paniscus_CM003395.1	104202964	104203881	HERVHF_3LTR	-
Pan_paniscus_CM003395.1	115036283	115036712	HERVHF_5LTR	+
Pan_paniscus_CM003395.1	115037925	115038479	HERVHF_gag	+
Pan_paniscus_CM003395.1	115038986	115039690	HERVHF_pro	+
Pan_paniscus_CM003395.1	115039034	115042346	HERVHF_pol	+
Pan_paniscus_CM003395.1	115044015	115044353	HERVHF_env	+
Pan_paniscus_CM003395.1	115044526	115044955	HERVHF_3LTR	+
Pan_paniscus_CM003395.1	125949316	125949754	HERVHF_5LTR	+
Pan_paniscus_CM003395.1	125950980	125951402	HERVHF_gag	+
Pan_paniscus_CM003395.1	125951987	125952625	HERVHF_pro	+
Pan_paniscus_CM003395.1	125952218	125954704	HERVHF_pol	+
Pan_paniscus_CM003395.1	125954993	125955694	HERVHF_env	+
Pan_paniscus_CM003395.1	125955858	125956291	HERVHF_3LTR	+
Pan_paniscus_CM003396.1	48711478	48711832	HERVHF_5LTR	-
Pan_paniscus_CM003396.1	48712381	48714932	HERVHF_pol	-
Pan_paniscus_CM003396.1	48714390	48715019	HERVHF_pro	-
Pan_paniscus_CM003396.1	48717000	48717345	HERVHF_3LTR	-
Pan_paniscus_CM003396.1	60984609	60984917	HERVHF_5LTR	+
Pan_paniscus_CM003396.1	60986475	60986807	HERVHF_gag	+
Pan_paniscus_CM003396.1	60987009	60987728	HERVHF_pro	+
Pan_paniscus_CM003396.1	60987525	60989707	HERVHF_pol	+
Pan_paniscus_CM003396.1	60990188	60990495	HERVHF_3LTR	+
Pan_paniscus_CM003396.1	79539507	79539924	HERVHF_5LTR	+
Pan_paniscus_CM003396.1	79541597	79541983	HERVHF_gag	+
Pan_paniscus_CM003396.1	79542285	79542836	HERVHF_pro	+
Pan_paniscus_CM003396.1	79542459	79544207	HERVHF_pol	+
Pan_paniscus_CM003396.1	79544833	79545247	HERVHF_3LTR	+
Pan_paniscus_CM003397.1	19622222	19622633	HERVHF_5LTR	+
Pan_paniscus_CM003397.1	19624000	19624428	HERVHF_gag	+
Pan_paniscus_CM003397.1	19624684	19625259	HERVHF_pro	+
Pan_paniscus_CM003397.1	19624864	19626693	HERVHF_pol	+
Pan_paniscus_CM003397.1	19627563	19627980	HERVHF_3LTR	+
Pan_paniscus_CM003397.1	69756333	69756678	HERVHF_5LTR	-

Pan_paniscus_CM003397.1	69756849	69757445	HERVHF_env	-
Pan_paniscus_CM003397.1	69758896	69760681	HERVHF_pol	-
Pan_paniscus_CM003397.1	69761031	69761834	HERVHF_pro	-
Pan_paniscus_CM003397.1	69762243	69762770	HERVHF_gag	-
Pan_paniscus_CM003397.1	69764403	69764737	HERVHF_3LTR	-
Pan_paniscus_CM003397.1	87207849	87208260	HERVHF_5LTR	-
Pan_paniscus_CM003397.1	87209459	87210622	HERVHF_pol	-
Pan_paniscus_CM003397.1	87210171	87211406	HERVHF_pro	-
Pan_paniscus_CM003397.1	87211492	87211872	HERVHF_gag	-
Pan_paniscus_CM003397.1	87213535	87213944	HERVHF_3LTR	-
Pan_paniscus_CM003397.1	98396543	98396956	HERVHF_5LTR	-
Pan_paniscus_CM003397.1	98398165	98399805	HERVHF_pol	-
Pan_paniscus_CM003397.1	98401962	98402376	HERVHF_3LTR	-
Pan_paniscus_CM003398.1	98460432	98460879	HERVHF_5LTR	+
Pan_paniscus_CM003398.1	98462680	98463003	HERVHF_gag	+
Pan_paniscus_CM003398.1	98463010	98463861	HERVHF_pro	+
Pan_paniscus_CM003398.1	98463298	98465590	HERVHF_pol	+
Pan_paniscus_CM003398.1	98465999	98466446	HERVHF_3LTR	+
Pan_paniscus_CM003402.1	20517739	20518189	HERVHF_5LTR	-
Pan_paniscus_CM003402.1	20518355	20519530	HERVHF_env	-
Pan_paniscus_CM003402.1	20521208	20523502	HERVHF_pol	-
Pan_paniscus_CM003402.1	20523933	20524514	HERVHF_gag	-
Pan_paniscus_CM003402.1	20526390	20526847	HERVHF_3LTR	-
Pan_paniscus_CM003402.1	23543185	23543571	HERVK_5LTR	-
Pan_paniscus_CM003402.1	23544480	23545745	HERVK_pol	-
Pan_paniscus_CM003402.1	23545640	23546497	HERVK_pro	-
Pan_paniscus_CM003402.1	23546159	23546671	HERVK_gag	-
Pan_paniscus_CM003402.1	23547428	23547815	HERVK_3LTR	-
Pan_paniscus_CM003402.1	51654384	51654729	HERVHF_5LTR	-
Pan_paniscus_CM003402.1	51655812	51664386	HERVHF_pol	-
Pan_paniscus_CM003402.1	51664317	51664817	HERVHF_pro	-
Pan_paniscus_CM003402.1	51667167	51667506	HERVHF_3LTR	-
Pan_paniscus_CM003402.1	52566336	52566768	HERVHF_5LTR	-
Pan_paniscus_CM003402.1	52567187	52569244	HERVHF_pol	-
Pan_paniscus_CM003402.1	52568717	52569235	HERVHF_pro	-
Pan_paniscus_CM003402.1	52569591	52570031	HERVHF_gag	-
Pan_paniscus_CM003402.1	52571447	52571877	HERVHF_3LTR	-
Pan_paniscus_CM003403.1	13303113	13303566	HERVHF_5LTR	+
Pan_paniscus_CM003403.1	13305138	13305518	HERVHF_gag	+
Pan_paniscus_CM003403.1	13305581	13305973	HERVHF_pro	+
Pan_paniscus_CM003403.1	13306053	13307119	HERVHF_pol	+
Pan_paniscus_CM003403.1	13308359	13308812	HERVHF_3LTR	+
Pan_paniscus_CM003404.1	16736640	16736970	HERVHF_5LTR	+
Pan_paniscus_CM003404.1	16738558	16738887	HERVHF_gag	+

Pan_paniscus_CM003404.1	16739142	16739723	HERVHF_pro	+
Pan_paniscus_CM003404.1	16739316	16741635	HERVHF_pol	+
Pan_paniscus_CM003404.1	16742060	16742381	HERVHF_3LTR	+
Pan_paniscus_CM003404.1	17002274	17002689	HERVHF_5LTR	+
Pan_paniscus_CM003404.1	17004399	17004719	HERVHF_gag	+
Pan_paniscus_CM003404.1	17004961	17005542	HERVHF_pro	+
Pan_paniscus_CM003404.1	17005300	17006454	HERVHF_pol	+
Pan_paniscus_CM003404.1	17007906	17008323	HERVHF_3LTR	+
Pan_paniscus_CM003404.1	40629858	40630310	HERVHF_5LTR	+
Pan_paniscus_CM003404.1	40631881	40632210	HERVHF_gag	+
Pan_paniscus_CM003404.1	40632322	40632702	HERVHF_pro	+
Pan_paniscus_CM003404.1	40632728	40634702	HERVHF_pol	+
Pan_paniscus_CM003404.1	40635111	40635563	HERVHF_3LTR	+
Pan_paniscus_CM003406.1	70583813	70584155	HERVHF_5LTR	+
Pan_paniscus_CM003406.1	70585780	70586097	HERVHF_gag	+
Pan_paniscus_CM003406.1	70586676	70587026	HERVHF_pro	+
Pan_paniscus_CM003406.1	70587028	70589078	HERVHF_pol	+
Pan_paniscus_CM003406.1	70590761	70591602	HERVHF_env	+
Pan_paniscus_CM003406.1	70591766	70592102	HERVHF_3LTR	+
Pan_paniscus_CM003406.1	98525053	98525456	HERVHF_5LTR	-
Pan_paniscus_CM003406.1	98525972	98527847	HERVHF_pol	-
Pan_paniscus_CM003406.1	98530365	98530767	HERVHF_3LTR	-
Pan_paniscus_CM003406.1	144706531	144706839	HERVHF_5LTR	-
Pan_paniscus_CM003406.1	144709237	144711610	HERVHF_pol	-
Pan_paniscus_CM003406.1	144712192	144712674	HERVHF_gag	-
Pan_paniscus_CM003406.1	144713882	144714196	HERVHF_3LTR	-
Pan_troglodytes_CM009238.2	14750808	14751323	HERVK_5LTR	-
Pan_troglodytes_CM009238.2	14752521	14754048	HERVK_pol	-
Pan_troglodytes_CM009238.2	14753946	14754887	HERVK_pro	-
Pan_troglodytes_CM009238.2	14756291	14756807	HERVK_3LTR	-
Pan_troglodytes_CM009238.2	21566932	21567356	HERVHF_5LTR	-
Pan_troglodytes_CM009238.2	21567530	21567874	HERVHF_env	-
Pan_troglodytes_CM009238.2	21569005	21571344	HERVHF_pol	-
Pan_troglodytes_CM009238.2	21571731	21572261	HERVHF_gag	-
Pan_troglodytes_CM009238.2	21573462	21573876	HERVHF_3LTR	-
Pan_troglodytes_CM009238.2	37209253	37209665	HERVHF_5LTR	+
Pan_troglodytes_CM009238.2	37211368	37211667	HERVHF_gag	+
Pan_troglodytes_CM009238.2	37211694	37212575	HERVHF_pro	+
Pan_troglodytes_CM009238.2	37211979	37214031	HERVHF_pol	+
Pan_troglodytes_CM009238.2	37214507	37214919	HERVHF_3LTR	+
Pan_troglodytes_CM009238.2	44560116	44560473	HERVIPADP_5LTR	-
Pan_troglodytes_CM009238.2	44560634	44561194	HERVIPADP_env	-
Pan_troglodytes_CM009238.2	44563558	44565982	HERVIPADP_pol	-
Pan_troglodytes_CM009238.2	44566559	44566870	HERVIPADP_gag	-

Pan_troglodytes_CM009238.2 44568939 44569296 HERVIPADP_3LTR -
Pan_troglodytes_CM009238.2 77834279 77834613 HERVHF_5LTR +
Pan_troglodytes_CM009238.2 77836161 77836757 HERVHF_gag +
Pan_troglodytes_CM009238.2 77836764 77837594 HERVHF_pro +
Pan_troglodytes_CM009238.2 77837052 77839419 HERVHF_pol +
Pan_troglodytes_CM009238.2 77841528 77842255 HERVHF_env +
Pan_troglodytes_CM009238.2 77842573 77842909 HERVHF_3LTR +
Pan_troglodytes_CM009238.2 80198633 80199085 HERVHF_5LTR +
Pan_troglodytes_CM009238.2 80200976 80201299 HERVHF_gag +
Pan_troglodytes_CM009238.2 80201392 80202135 HERVHF_pro +
Pan_troglodytes_CM009238.2 80201638 80203538 HERVHF_pol +
Pan_troglodytes_CM009238.2 80204131 80204583 HERVHF_3LTR +
Pan_troglodytes_CM009238.2 115352053 115352403 HERVHF_5LTR -
Pan_troglodytes_CM009238.2 115352619 115353056 HERVHF_env -
Pan_troglodytes_CM009238.2 115354201 115356290 HERVHF_pol -
Pan_troglodytes_CM009238.2 115356783 115357319 HERVHF_gag -
Pan_troglodytes_CM009238.2 115357939 115358279 HERVHF_3LTR -
Pan_troglodytes_CM009238.2 138623261 138623662 HERVHF_5LTR +
Pan_troglodytes_CM009238.2 138625119 138625448 HERVHF_gag +
Pan_troglodytes_CM009238.2 138625536 138626339 HERVHF_pro +
Pan_troglodytes_CM009238.2 138625638 138627708 HERVHF_pol +
Pan_troglodytes_CM009238.2 138628086 138628486 HERVHF_3LTR +
Pan_troglodytes_CM009238.2 144716421 144716834 HERVHF_5LTR +
Pan_troglodytes_CM009238.2 144718307 144718648 HERVHF_gag +
Pan_troglodytes_CM009238.2 144718740 144719405 HERVHF_pro +
Pan_troglodytes_CM009238.2 144719141 144720185 HERVHF_pol +
Pan_troglodytes_CM009238.2 144721608 144722033 HERVHF_3LTR +
Pan_troglodytes_CM009238.2 162872146 162872518 HERVHF_5LTR -
Pan_troglodytes_CM009238.2 162873012 162875262 HERVHF_pol -
Pan_troglodytes_CM009238.2 162874624 162875406 HERVHF_pro -
Pan_troglodytes_CM009238.2 162875504 162875989 HERVHF_gag -
Pan_troglodytes_CM009238.2 162877448 162877821 HERVHF_3LTR -
Pan_troglodytes_CM009238.2 190610019 190610467 HERVHF_5LTR -
Pan_troglodytes_CM009238.2 190610866 190612864 HERVHF_pol -
Pan_troglodytes_CM009238.2 190612633 190613058 HERVHF_pro -
Pan_troglodytes_CM009238.2 190613110 190613439 HERVHF_gag -
Pan_troglodytes_CM009238.2 190615141 190615591 HERVHF_3LTR -
Pan_troglodytes_CM009239.2 12525044 12525476 HERVHF_5LTR +
Pan_troglodytes_CM009239.2 12527185 12527598 HERVHF_gag +
Pan_troglodytes_CM009239.2 12527641 12528174 HERVHF_pro +
Pan_troglodytes_CM009239.2 12528086 12529941 HERVHF_pol +
Pan_troglodytes_CM009239.2 12530386 12530819 HERVHF_3LTR +
Pan_troglodytes_CM009239.2 13113032 13113497 HERVHF_5LTR -
Pan_troglodytes_CM009239.2 13114119 13116277 HERVHF_pol -

Pan_troglodytes_CM009239.2 13115684 13116433 HERVHF_pro -
Pan_troglodytes_CM009239.2 13116552 13116932 HERVHF_gag -
Pan_troglodytes_CM009239.2 13118548 13119025 HERVHF_3LTR -
Pan_troglodytes_CM009239.2 35429108 35429534 HERVHF_5LTR -
Pan_troglodytes_CM009239.2 35429923 35432244 HERVHF_pol -
Pan_troglodytes_CM009239.2 35431705 35432286 HERVHF_pro -
Pan_troglodytes_CM009239.2 35432569 35432877 HERVHF_gag -
Pan_troglodytes_CM009239.2 35434361 35434797 HERVHF_3LTR -
Pan_troglodytes_CM009239.2 41523267 41523698 HERVHF_5LTR +
Pan_troglodytes_CM009239.2 41524480 41525843 HERVHF_gag +
Pan_troglodytes_CM009239.2 41525853 41526383 HERVHF_pro +
Pan_troglodytes_CM009239.2 41526833 41529798 HERVHF_pol +
Pan_troglodytes_CM009239.2 41530046 41530498 HERVHF_env +
Pan_troglodytes_CM009239.2 41530725 41531156 HERVHF_3LTR +
Pan_troglodytes_CM009239.2 64388229 64388715 HERVHF_5LTR +
Pan_troglodytes_CM009239.2 64390800 64391561 HERVHF_pro +
Pan_troglodytes_CM009239.2 64391253 64392498 HERVHF_pol +
Pan_troglodytes_CM009239.2 64392993 64393479 HERVHF_3LTR +
Pan_troglodytes_CM009239.2 71315914 71316344 HERVHF_5LTR -
Pan_troglodytes_CM009239.2 71316799 71319091 HERVHF_pol -
Pan_troglodytes_CM009239.2 71318504 71319082 HERVHF_pro -
Pan_troglodytes_CM009239.2 71319466 71319774 HERVHF_gag -
Pan_troglodytes_CM009239.2 71321635 71322063 HERVHF_3LTR -
Pan_troglodytes_CM009239.2 75368224 75368696 HERVHF_5LTR -
Pan_troglodytes_CM009239.2 75369521 75371112 HERVHF_pol -
Pan_troglodytes_CM009239.2 75373676 75374140 HERVHF_3LTR -
Pan_troglodytes_CM009240.2 29064450 29064795 HERVHF_5LTR -
Pan_troglodytes_CM009240.2 29065247 29067146 HERVHF_pol -
Pan_troglodytes_CM009240.2 29066817 29067767 HERVHF_pro -
Pan_troglodytes_CM009240.2 29067909 29068253 HERVHF_gag -
Pan_troglodytes_CM009240.2 29070003 29070347 HERVHF_3LTR -
Pan_troglodytes_CM009240.2 52263079 52263565 HERVHF_5LTR -
Pan_troglodytes_CM009240.2 52263790 52264899 HERVHF_env -
Pan_troglodytes_CM009240.2 52266088 52268861 HERVHF_pol -
Pan_troglodytes_CM009240.2 52268313 52269155 HERVHF_pro -
Pan_troglodytes_CM009240.2 52271238 52271719 HERVHF_3LTR -
Pan_troglodytes_CM009240.2 74234862 74235332 HERVHF_5LTR -
Pan_troglodytes_CM009240.2 74235725 74238201 HERVHF_pol -
Pan_troglodytes_CM009240.2 74240594 74241046 HERVHF_3LTR -
Pan_troglodytes_CM009240.2 74757458 74757872 HERVHF_5LTR -
Pan_troglodytes_CM009240.2 74758705 74760769 HERVHF_pol -
Pan_troglodytes_CM009240.2 74760398 74761201 HERVHF_pro -
Pan_troglodytes_CM009240.2 74763063 74763476 HERVHF_3LTR -
Pan_troglodytes_CM009240.2 78750171 78750603 HERVHF_5LTR +

Pan_troglodytes_CM009240.2	78752798	78753361	HERVHF_pro	+
Pan_troglodytes_CM009240.2	78752837	78755147	HERVHF_pol	+
Pan_troglodytes_CM009240.2	78755359	78755804	HERVHF_3LTR	+
Pan_troglodytes_CM009240.2	80728121	80728535	HERVHF_5LTR	-
Pan_troglodytes_CM009240.2	80728930	80731275	HERVHF_pol	-
Pan_troglodytes_CM009240.2	80730607	80731449	HERVHF_pro	-
Pan_troglodytes_CM009240.2	80733561	80733973	HERVHF_3LTR	-
Pan_troglodytes_CM009240.2	95493254	95493566	HERVHF_5LTR	+
Pan_troglodytes_CM009240.2	95501288	95501893	HERVHF_pro	+
Pan_troglodytes_CM009240.2	95501627	95503719	HERVHF_pol	+
Pan_troglodytes_CM009240.2	95504545	95504853	HERVHF_3LTR	+
Pan_troglodytes_CM009240.2	101742731	101743068	HERVHF_5LTR	+
Pan_troglodytes_CM009240.2	101744502	101744825	HERVHF_gag	+
Pan_troglodytes_CM009240.2	101745246	101747100	HERVHF_pol	+
Pan_troglodytes_CM009240.2	101748456	101748796	HERVHF_3LTR	+
Pan_troglodytes_CM009241.2	1462834	1463307	HERVHF_5LTR	-
Pan_troglodytes_CM009241.2	1463607	1463921	HERVHF_env	-
Pan_troglodytes_CM009241.2	1464126	1464938	HERVHF_pol	-
Pan_troglodytes_CM009241.2	1464303	1465049	HERVHF_pro	-
Pan_troglodytes_CM009241.2	1465136	1465549	HERVHF_gag	-
Pan_troglodytes_CM009241.2	1466558	1467032	HERVHF_3LTR	-
Pan_troglodytes_CM009241.2	32279764	32280095	HERVHF_5LTR	+
Pan_troglodytes_CM009241.2	32280748	32282367	HERVHF_gag	+
Pan_troglodytes_CM009241.2	32282871	32285200	HERVHF_pol	+
Pan_troglodytes_CM009241.2	32287195	32287905	HERVHF_env	+
Pan_troglodytes_CM009241.2	32288072	32288414	HERVHF_3LTR	+
Pan_troglodytes_CM009241.2	46625604	46626044	HERVHF_5LTR	-
Pan_troglodytes_CM009241.2	46626213	46626641	HERVHF_env	-
Pan_troglodytes_CM009241.2	46627433	46628563	HERVHF_pol	-
Pan_troglodytes_CM009241.2	46628962	46629952	HERVHF_gag	-
Pan_troglodytes_CM009241.2	46630870	46631311	HERVHF_3LTR	-
Pan_troglodytes_CM009241.2	90690741	90691165	HERVHF_5LTR	+
Pan_troglodytes_CM009241.2	90691295	90691717	HERVHF_pro	+
Pan_troglodytes_CM009241.2	90691779	90693071	HERVHF_pol	+
Pan_troglodytes_CM009241.2	90694114	90694544	HERVHF_3LTR	+
Pan_troglodytes_CM009241.2	92080444	92080826	HERVHF_5LTR	+
Pan_troglodytes_CM009241.2	92082093	92082671	HERVHF_gag	+
Pan_troglodytes_CM009241.2	92082741	92083469	HERVHF_pro	+
Pan_troglodytes_CM009241.2	92083463	92085319	HERVHF_pol	+
Pan_troglodytes_CM009241.2	92086057	92086451	HERVHF_3LTR	+
Pan_troglodytes_CM009241.2	139295989	139296396	HERVHF_5LTR	+
Pan_troglodytes_CM009241.2	139298062	139298382	HERVHF_gag	+
Pan_troglodytes_CM009241.2	139298793	139300043	HERVHF_pol	+
Pan_troglodytes_CM009241.2	139301284	139302096	HERVHF_env	+

Pan_troglodytes_CM009241.2	139302265	139302672	HERVHF_3LTR	+
Pan_troglodytes_CM009241.2	141883502	141883963	HERVHF_5LTR	+
Pan_troglodytes_CM009241.2	141886141	141889768	HERVHF_pol	+
Pan_troglodytes_CM009241.2	141890631	141891299	HERVHF_env	+
Pan_troglodytes_CM009241.2	141891515	141891966	HERVHF_3LTR	+
Pan_troglodytes_CM009241.2	143633585	143633996	HERVHF_5LTR	-
Pan_troglodytes_CM009241.2	143634391	143636452	HERVHF_pol	-
Pan_troglodytes_CM009241.2	143636835	143637221	HERVHF_gag	-
Pan_troglodytes_CM009241.2	143638884	143639296	HERVHF_3LTR	-
Pan_troglodytes_CM009241.2	164861687	164862106	HERVHF_5LTR	-
Pan_troglodytes_CM009241.2	164862272	164863087	HERVHF_env	-
Pan_troglodytes_CM009241.2	164864221	164865997	HERVHF_pol	-
Pan_troglodytes_CM009241.2	164865919	164866437	HERVHF_pro	-
Pan_troglodytes_CM009241.2	164866491	164866874	HERVHF_gag	-
Pan_troglodytes_CM009241.2	164868413	164868832	HERVHF_3LTR	-
Pan_troglodytes_CM009241.2	173911128	173911570	HERVHF_5LTR	+
Pan_troglodytes_CM009241.2	173913307	173913627	HERVHF_gag	+
Pan_troglodytes_CM009241.2	173913634	173914380	HERVHF_pro	+
Pan_troglodytes_CM009241.2	173913814	173916975	HERVHF_pol	+
Pan_troglodytes_CM009241.2	173918105	173919043	HERVHF_env	+
Pan_troglodytes_CM009241.2	173919209	173919660	HERVHF_3LTR	+
Pan_troglodytes_CM009241.2	174415624	174416091	HERVHF_5LTR	-
Pan_troglodytes_CM009241.2	174416705	174418573	HERVHF_pol	-
Pan_troglodytes_CM009241.2	174418910	174419209	HERVHF_gag	-
Pan_troglodytes_CM009241.2	174420913	174421367	HERVHF_3LTR	-
Pan_troglodytes_CM009241.2	190137979	190138376	HERVHF_5LTR	-
Pan_troglodytes_CM009241.2	190138835	190140793	HERVHF_pol	-
Pan_troglodytes_CM009241.2	190140572	190141165	HERVHF_pro	-
Pan_troglodytes_CM009241.2	190141253	190141657	HERVHF_gag	-
Pan_troglodytes_CM009241.2	190143452	190143827	HERVHF_3LTR	-
Pan_troglodytes_CM009242.2	16486852	16487164	HERVHF_5LTR	+
Pan_troglodytes_CM009242.2	16488606	16489007	HERVHF_pro	+
Pan_troglodytes_CM009242.2	16489286	16491501	HERVHF_pol	+
Pan_troglodytes_CM009242.2	16492006	16492308	HERVHF_3LTR	+
Pan_troglodytes_CM009242.2	20105392	20105805	HERVHF_5LTR	-
Pan_troglodytes_CM009242.2	20106639	20108829	HERVHF_pol	-
Pan_troglodytes_CM009242.2	20108709	20109155	HERVHF_pro	-
Pan_troglodytes_CM009242.2	20109189	20109737	HERVHF_gag	-
Pan_troglodytes_CM009242.2	20111198	20111602	HERVHF_3LTR	-
Pan_troglodytes_CM009242.2	22947188	22947531	HSERVIII_5LTR	-
Pan_troglodytes_CM009242.2	22948533	22951311	HSERVIII_pol	-
Pan_troglodytes_CM009242.2	22953257	22953611	HSERVIII_3LTR	-
Pan_troglodytes_CM009242.2	45372134	45372619	HERVHF_5LTR	+
Pan_troglodytes_CM009242.2	45374030	45374503	HERVHF_gag	+

Pan_troglodytes_CM009242.2	45374904	45376158	HERVHF_pol	+
Pan_troglodytes_CM009242.2	45376527	45377014	HERVHF_3LTR	+
Pan_troglodytes_CM009242.2	83939130	83939592	HERVHF_5LTR	+
Pan_troglodytes_CM009242.2	83941046	83941486	HERVHF_gag	+
Pan_troglodytes_CM009242.2	83941476	83941907	HERVHF_pro	+
Pan_troglodytes_CM009242.2	83941952	83943679	HERVHF_pol	+
Pan_troglodytes_CM009242.2	83944481	83944942	HERVHF_3LTR	+
Pan_troglodytes_CM009242.2	85376352	85376704	HERVHF_5LTR	+
Pan_troglodytes_CM009242.2	85378472	85378795	HERVHF_gag	+
Pan_troglodytes_CM009242.2	85378918	85379364	HERVHF_pro	+
Pan_troglodytes_CM009242.2	85379313	85381306	HERVHF_pol	+
Pan_troglodytes_CM009242.2	85381710	85382072	HERVHF_3LTR	+
Pan_troglodytes_CM009242.2	114886748	114887164	HERVHF_5LTR	+
Pan_troglodytes_CM009242.2	114888937	114889236	HERVHF_gag	+
Pan_troglodytes_CM009242.2	114889315	114889707	HERVHF_pro	+
Pan_troglodytes_CM009242.2	114889686	114891237	HERVHF_pol	+
Pan_troglodytes_CM009242.2	114891703	114892067	HERVHF_env	+
Pan_troglodytes_CM009242.2	114892074	114892486	HERVHF_3LTR	+
Pan_troglodytes_CM009242.2	126022104	126022516	HERVHF_5LTR	-
Pan_troglodytes_CM009242.2	126022956	126024838	HERVHF_pol	-
Pan_troglodytes_CM009242.2	126024625	126025320	HERVHF_pro	-
Pan_troglodytes_CM009242.2	126027388	126027798	HERVHF_3LTR	-
Pan_troglodytes_CM009242.2	153180271	153180724	HERVHF_5LTR	-
Pan_troglodytes_CM009242.2	153181172	153183297	HERVHF_pol	-
Pan_troglodytes_CM009242.2	153183752	153184132	HERVHF_gag	-
Pan_troglodytes_CM009242.2	153185802	153186257	HERVHF_3LTR	-
Pan_troglodytes_CM009242.2	160681352	160681753	HERVHF_5LTR	+
Pan_troglodytes_CM009242.2	160683210	160683692	HERVHF_gag	+
Pan_troglodytes_CM009242.2	160683799	160684122	HERVHF_pro	+
Pan_troglodytes_CM009242.2	160684139	160686050	HERVHF_pol	+
Pan_troglodytes_CM009242.2	160686495	160686898	HERVHF_3LTR	+
Pan_troglodytes_CM009242.2	161071575	161071945	HERVHF_5LTR	-
Pan_troglodytes_CM009242.2	161073416	161075415	HERVHF_pol	-
Pan_troglodytes_CM009242.2	161075534	161075909	HERVHF_3LTR	-
Pan_troglodytes_CM009242.2	177656988	177657403	HERVHF_5LTR	+
Pan_troglodytes_CM009242.2	177659045	177659356	HERVHF_gag	+
Pan_troglodytes_CM009242.2	177659474	177660397	HERVHF_pro	+
Pan_troglodytes_CM009242.2	177659642	177662143	HERVHF_pol	+
Pan_troglodytes_CM009242.2	177662961	177663373	HERVHF_3LTR	+
Pan_troglodytes_CM009242.2	180788890	180789320	HERVHF_5LTR	+
Pan_troglodytes_CM009242.2	180791231	180791626	HERVHF_gag	+
Pan_troglodytes_CM009242.2	180791895	180792458	HERVHF_pro	+
Pan_troglodytes_CM009242.2	180792069	180792596	HERVHF_pol	+
Pan_troglodytes_CM009242.2	180793134	180793563	HERVHF_3LTR	+

Pan_troglodytes_CM009242.2	184193964	184194331	HERVHF_5LTR	-
Pan_troglodytes_CM009242.2	184194878	184197017	HERVHF_pol	-
Pan_troglodytes_CM009242.2	184197375	184197812	HERVHF_gag	-
Pan_troglodytes_CM009242.2	184199208	184199577	HERVHF_3LTR	-
Pan_troglodytes_CM009243.2	6962082	6962467	HERVHF_5LTR	-
Pan_troglodytes_CM009243.2	6962971	6963393	HERVHF_env	-
Pan_troglodytes_CM009243.2	6963726	6966789	HERVHF_pol	-
Pan_troglodytes_CM009243.2	6966289	6967161	HERVHF_pro	-
Pan_troglodytes_CM009243.2	6969019	6969412	HERVHF_3LTR	-
Pan_troglodytes_CM009243.2	37942039	37942464	HERVHF_5LTR	+
Pan_troglodytes_CM009243.2	37944955	37945532	HERVHF_gag	+
Pan_troglodytes_CM009243.2	37945821	37947833	HERVHF_pol	+
Pan_troglodytes_CM009243.2	37948334	37948681	HERVHF_env	+
Pan_troglodytes_CM009243.2	37948846	37949262	HERVHF_3LTR	+
Pan_troglodytes_CM009243.2	91835789	91836300	HERVK_5LTR	+
Pan_troglodytes_CM009243.2	91837069	91837923	HERVK_pro	+
Pan_troglodytes_CM009243.2	91837872	91839182	HERVK_pol	+
Pan_troglodytes_CM009243.2	91839480	91839860	HERVK_env	+
Pan_troglodytes_CM009243.2	91839979	91840482	HERVK_3LTR	+
Pan_troglodytes_CM009243.2	114924886	114925218	HERVHF_5LTR	+
Pan_troglodytes_CM009243.2	114926830	114927414	HERVHF_gag	+
Pan_troglodytes_CM009243.2	114927956	114931478	HERVHF_pol	+
Pan_troglodytes_CM009243.2	114931882	114933078	HERVHF_env	+
Pan_troglodytes_CM009243.2	114933297	114933610	HERVHF_3LTR	+
Pan_troglodytes_CM009243.2	139386807	139387232	HERVHF_5LTR	+
Pan_troglodytes_CM009243.2	139387773	139388093	HERVHF_gag	+
Pan_troglodytes_CM009243.2	139388334	139388936	HERVHF_pro	+
Pan_troglodytes_CM009243.2	139388349	139390844	HERVHF_pol	+
Pan_troglodytes_CM009243.2	139395579	139396000	HERVHF_3LTR	+
Pan_troglodytes_CM009243.2	140400986	140401399	HERVHF_5LTR	-
Pan_troglodytes_CM009243.2	140401992	140403731	HERVHF_pol	-
Pan_troglodytes_CM009243.2	140403395	140404171	HERVHF_pro	-
Pan_troglodytes_CM009243.2	140406305	140406718	HERVHF_3LTR	-
Pan_troglodytes_CM009243.2	141465093	141465466	HERVHF_5LTR	+
Pan_troglodytes_CM009243.2	141467288	141468154	HERVHF_pro	+
Pan_troglodytes_CM009243.2	141467693	141469407	HERVHF_pol	+
Pan_troglodytes_CM009243.2	141470344	141470769	HERVHF_env	+
Pan_troglodytes_CM009243.2	141470951	141471318	HERVHF_3LTR	+
Pan_troglodytes_CM009243.2	149142400	149142774	HERVHF_5LTR	-
Pan_troglodytes_CM009243.2	149143666	149146155	HERVHF_pol	-
Pan_troglodytes_CM009243.2	149145610	149146335	HERVHF_pro	-
Pan_troglodytes_CM009243.2	149146451	149146852	HERVHF_gag	-
Pan_troglodytes_CM009243.2	149150185	149150573	HERVHF_3LTR	-
Pan_troglodytes_CM009244.2	40382429	40382862	HERVHF_5LTR	-

Pan_troglodytes_CM009244.2	40383023	40384206	HERVHF_env	-
Pan_troglodytes_CM009244.2	40384601	40388132	HERVHF_pol	-
Pan_troglodytes_CM009244.2	40388500	40389090	HERVHF_gag	-
Pan_troglodytes_CM009244.2	40389352	40389785	HERVHF_3LTR	-
Pan_troglodytes_CM009244.2	61336194	61336560	HERVHF_5LTR	+
Pan_troglodytes_CM009244.2	61338020	61338325	HERVHF_gag	+
Pan_troglodytes_CM009244.2	61338788	61341522	HERVHF_pol	+
Pan_troglodytes_CM009244.2	61341631	61341966	HERVHF_env	+
Pan_troglodytes_CM009244.2	61342125	61342490	HERVHF_3LTR	+
Pan_troglodytes_CM009244.2	110656184	110656522	HERVK_5LTR	-
Pan_troglodytes_CM009244.2	110657893	110659377	HERVK_pol	-
Pan_troglodytes_CM009244.2	110659272	110660198	HERVK_pro	-
Pan_troglodytes_CM009244.2	110660763	110661087	HERVK_3LTR	-
Pan_troglodytes_CM009244.2	115036889	115037328	HERVHF_5LTR	-
Pan_troglodytes_CM009244.2	115038199	115040877	HERVHF_pol	-
Pan_troglodytes_CM009244.2	115040083	115041012	HERVHF_pro	-
Pan_troglodytes_CM009244.2	115042852	115043289	HERVHF_3LTR	-
Pan_troglodytes_CM009244.2	128701675	128702132	HERVHF_5LTR	+
Pan_troglodytes_CM009244.2	128704313	128705143	HERVHF_pro	+
Pan_troglodytes_CM009244.2	128704601	128706678	HERVHF_pol	+
Pan_troglodytes_CM009244.2	128707127	128707578	HERVHF_3LTR	+
Pan_troglodytes_CM009244.2	134387840	134388217	HERVHF_5LTR	-
Pan_troglodytes_CM009244.2	134388666	134391538	HERVHF_pol	-
Pan_troglodytes_CM009244.2	134390951	134391730	HERVHF_pro	-
Pan_troglodytes_CM009244.2	134393987	134394359	HERVHF_3LTR	-
Pan_troglodytes_CM009245.2	12697826	12698209	HERVHF_5LTR	-
Pan_troglodytes_CM009245.2	12699835	12701404	HERVHF_pol	-
Pan_troglodytes_CM009245.2	12701621	12702064	HERVHF_pro	-
Pan_troglodytes_CM009245.2	12702808	12703797	HERVHF_gag	-
Pan_troglodytes_CM009245.2	12704233	12704602	HERVHF_3LTR	-
Pan_troglodytes_CM009245.2	20653816	20654225	HERVHF_5LTR	+
Pan_troglodytes_CM009245.2	20655282	20657533	HERVHF_pol	+
Pan_troglodytes_CM009245.2	20658403	20658816	HERVHF_3LTR	+
Pan_troglodytes_CM009245.2	29890095	29890498	HERVHF_5LTR	+
Pan_troglodytes_CM009245.2	29892100	29892399	HERVHF_gag	+
Pan_troglodytes_CM009245.2	29892429	29893256	HERVHF_pro	+
Pan_troglodytes_CM009245.2	29892675	29894988	HERVHF_pol	+
Pan_troglodytes_CM009245.2	29896524	29896976	HERVHF_env	+
Pan_troglodytes_CM009245.2	29897242	29897642	HERVHF_3LTR	+
Pan_troglodytes_CM009245.2	32920766	32921207	HERVHF_5LTR	-
Pan_troglodytes_CM009245.2	32922185	32922849	HERVHF_pol	-
Pan_troglodytes_CM009245.2	32922481	32923059	HERVHF_pro	-
Pan_troglodytes_CM009245.2	32923213	32923596	HERVHF_gag	-
Pan_troglodytes_CM009245.2	32925114	32925558	HERVHF_3LTR	-

Pan_troglodytes_CM009245.2	73374589	73374893	HERVHF_5LTR	+
Pan_troglodytes_CM009245.2	73376824	73377546	HERVHF_pro	+
Pan_troglodytes_CM009245.2	73376983	73378855	HERVHF_pol	+
Pan_troglodytes_CM009245.2	73380060	73380384	HERVHF_3LTR	+
Pan_troglodytes_CM009245.2	138459300	138459703	HERVHF_5LTR	-
Pan_troglodytes_CM009245.2	138460555	138463011	HERVHF_pol	-
Pan_troglodytes_CM009245.2	138462472	138463056	HERVHF_pro	-
Pan_troglodytes_CM009245.2	138463353	138463709	HERVHF_gag	-
Pan_troglodytes_CM009245.2	138465271	138465658	HERVHF_3LTR	-
Pan_troglodytes_CM009246.2	63934436	63934798	HERVHF_5LTR	+
Pan_troglodytes_CM009246.2	63936516	63936848	HERVHF_gag	+
Pan_troglodytes_CM009246.2	63937204	63937794	HERVHF_pro	+
Pan_troglodytes_CM009246.2	63937405	63940066	HERVHF_pol	+
Pan_troglodytes_CM009246.2	63940554	63941003	HERVHF_env	+
Pan_troglodytes_CM009246.2	63941283	63941648	HERVHF_3LTR	+
Pan_troglodytes_CM009246.2	80012945	80013323	HERVHF_5LTR	-
Pan_troglodytes_CM009246.2	80013565	80013930	HERVHF_env	-
Pan_troglodytes_CM009246.2	80014307	80016295	HERVHF_pol	-
Pan_troglodytes_CM009246.2	80015969	80016523	HERVHF_pro	-
Pan_troglodytes_CM009246.2	80016721	80017047	HERVHF_gag	-
Pan_troglodytes_CM009246.2	80018488	80018863	HERVHF_3LTR	-
Pan_troglodytes_CM009246.2	113203990	113204442	HERVHF_5LTR	+
Pan_troglodytes_CM009246.2	113206133	113206453	HERVHF_gag	+
Pan_troglodytes_CM009246.2	113206505	113207260	HERVHF_pro	+
Pan_troglodytes_CM009246.2	113206703	113208872	HERVHF_pol	+
Pan_troglodytes_CM009246.2	113209282	113209734	HERVHF_3LTR	+
Pan_troglodytes_CM009247.2	90767505	90767981	HERVHF_5LTR	-
Pan_troglodytes_CM009247.2	90768567	90770607	HERVHF_pol	-
Pan_troglodytes_CM009247.2	90770604	90771011	HERVHF_pro	-
Pan_troglodytes_CM009247.2	90771054	90771563	HERVHF_gag	-
Pan_troglodytes_CM009247.2	90773151	90773627	HERVHF_3LTR	-
Pan_troglodytes_CM009247.2	94068547	94068999	HERVHF_5LTR	+
Pan_troglodytes_CM009247.2	94070890	94071447	HERVHF_gag	+
Pan_troglodytes_CM009247.2	94071568	94072398	HERVHF_pro	+
Pan_troglodytes_CM009247.2	94071856	94073916	HERVHF_pol	+
Pan_troglodytes_CM009247.2	94074401	94074916	HERVHF_env	+
Pan_troglodytes_CM009247.2	94075080	94075541	HERVHF_3LTR	+
Pan_troglodytes_CM009248.2	51834005	51834341	HERVHF_5LTR	+
Pan_troglodytes_CM009248.2	51835791	51836090	HERVHF_gag	+
Pan_troglodytes_CM009248.2	51836143	51836919	HERVHF_pro	+
Pan_troglodytes_CM009248.2	51836515	51838122	HERVHF_pol	+
Pan_troglodytes_CM009248.2	51839020	51839357	HERVHF_3LTR	+
Pan_troglodytes_CM009248.2	86261866	86262283	HERVHF_5LTR	+
Pan_troglodytes_CM009248.2	86264792	86265265	HERVHF_pro	+

Pan_troglodytes_CM009248.2 86264843 86266781 HERVHF_pol +
Pan_troglodytes_CM009248.2 86267373 86267795 HERVHF_3LTR +
Pan_troglodytes_CM009248.2 129096374 129096811 HERVHF_5LTR +
Pan_troglodytes_CM009248.2 129098732 129099061 HERVHF_gag +
Pan_troglodytes_CM009248.2 129099202 129099978 HERVHF_pro +
Pan_troglodytes_CM009248.2 129099400 129100677 HERVHF_pol +
Pan_troglodytes_CM009248.2 129101086 129101533 HERVHF_3LTR +
Pan_troglodytes_CM009249.2 4160623 4161616 HERVHF_5LTR -
Pan_troglodytes_CM009249.2 4165183 4167126 HERVHF_pol -
Pan_troglodytes_CM009249.2 4167117 4167497 HERVHF_pro -
Pan_troglodytes_CM009249.2 4167827 4168285 HERVHF_gag -
Pan_troglodytes_CM009249.2 4169381 4170379 HERVHF_3LTR -
Pan_troglodytes_CM009249.2 14344260 14344693 HERVHF_5LTR +
Pan_troglodytes_CM009249.2 14345242 14347791 HERVHF_pol +
Pan_troglodytes_CM009249.2 14348106 14348468 HERVHF_env +
Pan_troglodytes_CM009249.2 14348634 14349067 HERVHF_3LTR +
Pan_troglodytes_CM009249.2 18393876 18394316 HERVHF_5LTR +
Pan_troglodytes_CM009249.2 18396182 18397120 HERVHF_pro +
Pan_troglodytes_CM009249.2 18396368 18398858 HERVHF_pol +
Pan_troglodytes_CM009249.2 18399176 18399538 HERVHF_env +
Pan_troglodytes_CM009249.2 18399692 18400132 HERVHF_3LTR +
Pan_troglodytes_CM009249.2 39459006 39459438 HERVHF_5LTR +
Pan_troglodytes_CM009249.2 39461073 39461675 HERVHF_gag +
Pan_troglodytes_CM009249.2 39462112 39464436 HERVHF_pol +
Pan_troglodytes_CM009249.2 39464979 39465326 HERVHF_env +
Pan_troglodytes_CM009249.2 39465499 39465933 HERVHF_3LTR +
Pan_troglodytes_CM009250.2 17701048 17701408 HERVHF_5LTR +
Pan_troglodytes_CM009250.2 17702706 17703056 HERVHF_gag +
Pan_troglodytes_CM009250.2 17703702 17704376 HERVHF_pro +
Pan_troglodytes_CM009250.2 17703720 17708320 HERVHF_pol +
Pan_troglodytes_CM009250.2 17708652 17709002 HERVHF_env +
Pan_troglodytes_CM009250.2 17709235 17709588 HERVHF_3LTR +
Pan_troglodytes_CM009250.2 21002720 21003172 HERVHF_5LTR +
Pan_troglodytes_CM009250.2 21004890 21005276 HERVHF_gag +
Pan_troglodytes_CM009250.2 21005357 21005866 HERVHF_pro +
Pan_troglodytes_CM009250.2 21005881 21007606 HERVHF_pol +
Pan_troglodytes_CM009250.2 21008061 21008512 HERVHF_3LTR +
Pan_troglodytes_CM009250.2 41609740 41610250 HERVK_5LTR +
Pan_troglodytes_CM009250.2 41611007 41611885 HERVK_pro +
Pan_troglodytes_CM009250.2 41611860 41613722 HERVK_pol +
Pan_troglodytes_CM009250.2 41614269 41614571 HERVK_env +
Pan_troglodytes_CM009250.2 41614649 41615160 HERVK_3LTR +
Pan_troglodytes_CM009250.2 51968835 51969328 HERVHF_5LTR -
Pan_troglodytes_CM009250.2 51969585 51970693 HERVHF_env -

Pan_troglodytes_CM009250.2	51972231	51974711	HERVHF_pol	-
Pan_troglodytes_CM009250.2	51974085	51974804	HERVHF_pro	-
Pan_troglodytes_CM009250.2	51975005	51975322	HERVHF_gag	-
Pan_troglodytes_CM009250.2	51977066	51977559	HERVHF_3LTR	-
Pan_troglodytes_CM009250.2	57499033	57499339	HSERVIII_5LTR	-
Pan_troglodytes_CM009250.2	57502385	57503002	HSERVIII_pol	-
Pan_troglodytes_CM009250.2	57504082	57504383	HSERVIII_3LTR	-
Pan_troglodytes_CM009250.2	70541368	70541796	HERVHF_5LTR	-
Pan_troglodytes_CM009250.2	70542533	70544452	HERVHF_pol	-
Pan_troglodytes_CM009250.2	70544766	70545302	HERVHF_gag	-
Pan_troglodytes_CM009250.2	70546824	70547250	HERVHF_3LTR	-
Pan_troglodytes_CM009250.2	111719729	111720158	HERVHF_5LTR	+
Pan_troglodytes_CM009250.2	111721371	111721925	HERVHF_gag	+
Pan_troglodytes_CM009250.2	111722405	111723157	HERVHF_pro	+
Pan_troglodytes_CM009250.2	111722480	111725792	HERVHF_pol	+
Pan_troglodytes_CM009250.2	111727461	111727799	HERVHF_env	+
Pan_troglodytes_CM009250.2	111727972	111728401	HERVHF_3LTR	+
Pan_troglodytes_CM009250.2	124834031	124834393	HERVHF_5LTR	+
Pan_troglodytes_CM009250.2	124836078	124836527	HERVHF_gag	+
Pan_troglodytes_CM009250.2	124836698	124837351	HERVHF_pro	+
Pan_troglodytes_CM009250.2	124836902	124837807	HERVHF_pol	+
Pan_troglodytes_CM009250.2	124839942	124840307	HERVHF_3LTR	+
Pan_troglodytes_CM009251.2	29856729	29857085	HERVHF_5LTR	-
Pan_troglodytes_CM009251.2	29857632	29860144	HERVHF_pol	-
Pan_troglodytes_CM009251.2	29859665	29860321	HERVHF_pro	-
Pan_troglodytes_CM009251.2	29862272	29862618	HERVHF_3LTR	-
Pan_troglodytes_CM009252.2	1672542	1672952	HERVHF_5LTR	+
Pan_troglodytes_CM009252.2	1674318	1674731	HERVHF_gag	+
Pan_troglodytes_CM009252.2	1674987	1675550	HERVHF_pro	+
Pan_troglodytes_CM009252.2	1675167	1676996	HERVHF_pol	+
Pan_troglodytes_CM009252.2	1677866	1678283	HERVHF_3LTR	+
Pan_troglodytes_CM009252.2	68061096	68061507	HERVHF_5LTR	-
Pan_troglodytes_CM009252.2	68062077	68064164	HERVHF_pol	-
Pan_troglodytes_CM009252.2	68063874	68064590	HERVHF_pro	-
Pan_troglodytes_CM009252.2	68066794	68067194	HERVHF_3LTR	-
Pan_troglodytes_CM009254.2	9151162	9151589	HERVHF_5LTR	-
Pan_troglodytes_CM009254.2	9151820	9153464	HERVHF_env	-
Pan_troglodytes_CM009254.2	9153797	9156252	HERVHF_pol	-
Pan_troglodytes_CM009254.2	9155716	9156513	HERVHF_pro	-
Pan_troglodytes_CM009254.2	9156626	9156949	HERVHF_gag	-
Pan_troglodytes_CM009254.2	9158485	9158915	HERVHF_3LTR	-
Pan_troglodytes_CM009254.2	30583371	30583746	HERVHF_5LTR	+
Pan_troglodytes_CM009254.2	30584336	30585843	HERVHF_gag	+
Pan_troglodytes_CM009254.2	30586043	30586378	HERVHF_pro	+

Pan_troglodytes_CM009254.2	30586172	30587980	HERVHF_pol	+
Pan_troglodytes_CM009254.2	30589776	30591204	HERVHF_env	+
Pan_troglodytes_CM009254.2	30592252	30592623	HERVHF_3LTR	+
Pan_troglodytes_CM009255.2	38824354	38824761	HERVHF_5LTR	-
Pan_troglodytes_CM009255.2	38825383	38826694	HERVHF_pol	-
Pan_troglodytes_CM009255.2	38826113	38826946	HERVHF_pro	-
Pan_troglodytes_CM009255.2	38829076	38829472	HERVHF_3LTR	-
Pan_troglodytes_CM009255.2	68359237	68359584	HERVHF_5LTR	+
Pan_troglodytes_CM009255.2	68361192	68361500	HERVHF_gag	+
Pan_troglodytes_CM009255.2	68361628	68362338	HERVHF_pro	+
Pan_troglodytes_CM009255.2	68361871	68363993	HERVHF_pol	+
Pan_troglodytes_CM009255.2	68364524	68364869	HERVHF_3LTR	+
Pan_troglodytes_CM009256.2	67547095	67547528	HERVHF_5LTR	+
Pan_troglodytes_CM009256.2	67550054	67551932	HERVHF_pol	+
Pan_troglodytes_CM009256.2	67552526	67552959	HERVHF_3LTR	+
Pan_troglodytes_CM009257.2	5471150	5471621	HERVHF_5LTR	+
Pan_troglodytes_CM009257.2	5473153	5473452	HERVHF_gag	+
Pan_troglodytes_CM009257.2	5473592	5474389	HERVHF_pro	+
Pan_troglodytes_CM009257.2	5473853	5474641	HERVHF_pol	+
Pan_troglodytes_CM009257.2	5474855	5475193	HERVHF_env	+
Pan_troglodytes_CM009257.2	5475385	5475855	HERVHF_3LTR	+
Pan_troglodytes_CM009257.2	44057131	44057476	HERVHF_5LTR	-
Pan_troglodytes_CM009257.2	44058559	44067137	HERVHF_pol	-
Pan_troglodytes_CM009257.2	44067068	44067568	HERVHF_pro	-
Pan_troglodytes_CM009257.2	44069919	44070258	HERVHF_3LTR	-
Pan_troglodytes_CM009258.2	15454444	15454797	HERVHF_5LTR	-
Pan_troglodytes_CM009258.2	15455254	15457546	HERVHF_pol	-
Pan_troglodytes_CM009258.2	15457004	15457837	HERVHF_pro	-
Pan_troglodytes_CM009258.2	15458007	15458417	HERVHF_gag	-
Pan_troglodytes_CM009258.2	15459892	15460249	HERVHF_3LTR	-
Pan_troglodytes_CM009258.2	39667596	39668051	HERVHF_5LTR	+
Pan_troglodytes_CM009258.2	39670322	39670702	HERVHF_pro	+
Pan_troglodytes_CM009258.2	39670717	39672616	HERVHF_pol	+
Pan_troglodytes_CM009258.2	39673052	39673504	HERVHF_3LTR	+
Pan_troglodytes_CM009259.2	3539036	3539366	HERVHF_5LTR	+
Pan_troglodytes_CM009259.2	3540960	3541283	HERVHF_gag	+
Pan_troglodytes_CM009259.2	3541538	3542119	HERVHF_pro	+
Pan_troglodytes_CM009259.2	3541712	3544031	HERVHF_pol	+
Pan_troglodytes_CM009259.2	3544456	3544777	HERVHF_3LTR	+
Pan_troglodytes_CM009259.2	3804981	3805396	HERVHF_5LTR	+
Pan_troglodytes_CM009259.2	3807106	3807426	HERVHF_gag	+
Pan_troglodytes_CM009259.2	3807668	3810164	HERVHF_pol	+
Pan_troglodytes_CM009259.2	3807668	3808249	HERVHF_pro	+
Pan_troglodytes_CM009259.2	3810614	3811031	HERVHF_3LTR	+

Pan_troglodytes_CM009260.2	17767838	17768258	HERVHF_5LTR	+
Pan_troglodytes_CM009260.2	17769604	17770017	HERVHF_gag	+
Pan_troglodytes_CM009260.2	17770070	17770846	HERVHF_pro	+
Pan_troglodytes_CM009260.2	17770534	17772332	HERVHF_pol	+
Pan_troglodytes_CM009260.2	17772827	17773248	HERVHF_3LTR	+
Pan_troglodytes_CM009261.2	28448170	28448496	HERVHF_5LTR	-
Pan_troglodytes_CM009261.2	28448659	28449009	HERVHF_env	-
Pan_troglodytes_CM009261.2	28449904	28450953	HERVHF_pol	-
Pan_troglodytes_CM009261.2	28451155	28452244	HERVHF_gag	-
Pan_troglodytes_CM009261.2	28453039	28453382	HERVHF_3LTR	-
Pan_troglodytes_CM009261.2	65097825	65098166	HERVHF_5LTR	-
Pan_troglodytes_CM009261.2	65098293	65098715	HERVHF_env	-
Pan_troglodytes_CM009261.2	65099348	65101742	HERVHF_pol	-
Pan_troglodytes_CM009261.2	65101098	65101949	HERVHF_pro	-
Pan_troglodytes_CM009261.2	65102189	65102725	HERVHF_gag	-
Pan_troglodytes_CM009261.2	65104172	65104518	HERVHF_3LTR	-
Pan_troglodytes_CM009261.2	82404929	82405372	HERVHF_5LTR	+
Pan_troglodytes_CM009261.2	82407081	82407380	HERVHF_gag	+
Pan_troglodytes_CM009261.2	82407437	82408237	HERVHF_pro	+
Pan_troglodytes_CM009261.2	82407620	82409591	HERVHF_pol	+
Pan_troglodytes_CM009261.2	82410172	82410611	HERVHF_3LTR	+
Pan_troglodytes_CM009261.2	87540440	87540897	HERVHF_5LTR	-
Pan_troglodytes_CM009261.2	87541719	87543411	HERVHF_pol	-
Pan_troglodytes_CM009261.2	87542887	87543507	HERVHF_pro	-
Pan_troglodytes_CM009261.2	87545640	87546094	HERVHF_3LTR	-
Pan_troglodytes_CM009261.2	116546206	116546515	HERVHF_5LTR	-
Pan_troglodytes_CM009261.2	116546795	116547166	HERVHF_env	-
Pan_troglodytes_CM009261.2	116547211	116548372	HERVHF_pol	-
Pan_troglodytes_CM009261.2	116547994	116548581	HERVHF_pro	-
Pan_troglodytes_CM009261.2	116548661	116549137	HERVHF_gag	-
Pan_troglodytes_CM009261.2	116550583	116550887	HERVHF_3LTR	-
Pan_troglodytes_CM009261.2	140421820	140422128	HERVHF_5LTR	-
Pan_troglodytes_CM009261.2	140424526	140426900	HERVHF_pol	-
Pan_troglodytes_CM009261.2	140427185	140427670	HERVHF_gag	-
Pan_troglodytes_CM009261.2	140429177	140429491	HERVHF_3LTR	-
Pan_troglodytes_KZ622834.1	5394343	5394716	HERVHF_5LTR	+
Pan_troglodytes_KZ622834.1	5401874	5403349	HERVHF_gag	+
Pan_troglodytes_KZ622834.1	5404015	5406611	HERVHF_pol	+
Pan_troglodytes_KZ622834.1	5408170	5408813	HERVHF_env	+
Pan_troglodytes_KZ622834.1	5409232	5409589	HERVHF_3LTR	+
Pan_troglodytes_KZ622835.1	669576	669900	HERVHF_5LTR	+
Pan_troglodytes_KZ622835.1	671281	671861	HERVHF_gag	+
Pan_troglodytes_KZ622835.1	671918	672706	HERVHF_pro	+
Pan_troglodytes_KZ622835.1	672471	674425	HERVHF_pol	+

Pan_troglodytes_KZ622835.1	676066	677192	HERVHF_env	+
Pan_troglodytes_KZ622835.1	677417	677732	HERVHF_3LTR	+
Pan_troglodytes_NBAG03000060.1	38461	38765	HERVHF_5LTR	+
Pan_troglodytes_NBAG03000060.1	45678	45995	HERVHF_gag	+
Pan_troglodytes_NBAG03000060.1	46104	46781	HERVHF_pro	+
Pan_troglodytes_NBAG03000060.1	46287	48111	HERVHF_pol	+
Pan_troglodytes_NBAG03000060.1	49367	49681	HERVHF_3LTR	+
Pan_troglodytes_NBAG03000384.1	1709669	1710134	HERVK_5LTR	-
Pan_troglodytes_NBAG03000384.1	1710703	1712743	HERVK_pol	-
Pan_troglodytes_NBAG03000384.1	1712201	1713451	HERVK_pro	-
Pan_troglodytes_NBAG03000384.1	1714901	1715371	HERVK_3LTR	-
Pan_troglodytes_NBAG03001518.1	45869	46211	HERVHF_5LTR	+
Pan_troglodytes_NBAG03001518.1	47825	48154	HERVHF_gag	+
Pan_troglodytes_NBAG03001518.1	48204	49205	HERVHF_pro	+
Pan_troglodytes_NBAG03001518.1	48750	51093	HERVHF_pol	+
Pan_troglodytes_NBAG03001518.1	52771	53546	HERVHF_env	+
Pan_troglodytes_NBAG03001518.1	54094	54430	HERVHF_3LTR	+
Pan_troglodytes_NBAG03002951.1	15507	15908	HERVHF_5LTR	+
Pan_troglodytes_NBAG03002951.1	17365	17847	HERVHF_gag	+
Pan_troglodytes_NBAG03002951.1	17954	18277	HERVHF_pro	+
Pan_troglodytes_NBAG03002951.1	18294	20205	HERVHF_pol	+
Pan_troglodytes_NBAG03002951.1	20650	21053	HERVHF_3LTR	+
Pan_troglodytes_NBAG03002955.1	15483	15884	HERVHF_5LTR	+
Pan_troglodytes_NBAG03002955.1	17341	17823	HERVHF_gag	+
Pan_troglodytes_NBAG03002955.1	17930	18253	HERVHF_pro	+
Pan_troglodytes_NBAG03002955.1	18270	20181	HERVHF_pol	+
Pan_troglodytes_NBAG03002955.1	20626	21029	HERVHF_3LTR	+
Papio_anubis_CM001491.2	11599737	11600051	HERVIPADP_5LTR	-
Papio_anubis_CM001491.2	11600075	11600730	HERVIPADP_env	-
Papio_anubis_CM001491.2	11602962	11605782	HERVIPADP_pol	-
Papio_anubis_CM001491.2	11606342	11606659	HERVIPADP_gag	-
Papio_anubis_CM001491.2	11614561	11614880	HERVIPADP_3LTR	-
Papio_anubis_CM001491.2	75806746	75807112	HERVK_5LTR	-
Papio_anubis_CM001491.2	75808252	75809910	HERVK_pol	-
Papio_anubis_CM001491.2	75810016	75810743	HERVK_pro	-
Papio_anubis_CM001491.2	75811242	75811682	HERVK_gag	-
Papio_anubis_CM001491.2	75812151	75812501	HERVK_3LTR	-
Papio_anubis_CM001492.2	10733785	10734160	HERVK_5LTR	-
Papio_anubis_CM001492.2	10734819	10736011	HERVK_env	-
Papio_anubis_CM001492.2	10737347	10738505	HERVK_pol	-
Papio_anubis_CM001492.2	10738346	10738852	HERVK_pro	-
Papio_anubis_CM001492.2	10740496	10740856	HERVK_3LTR	-
Papio_anubis_CM001493.2	123829370	123829857	HERVK_5LTR	-
Papio_anubis_CM001493.2	123829965	123830273	HERVK_env	-

Papio_anubis_CM001493.2	123830810	123832720	HERVK_pol	-
Papio_anubis_CM001493.2	123832621	123833430	HERVK_pro	-
Papio_anubis_CM001493.2	123834312	123834789	HERVK_3LTR	-
Papio_anubis_CM001493.2	132810664	132811108	HERVK_5LTR	+
Papio_anubis_CM001493.2	132812809	132813672	HERVK_gag	+
Papio_anubis_CM001493.2	132813832	132814699	HERVK_pro	+
Papio_anubis_CM001493.2	132814585	132815712	HERVK_pol	+
Papio_anubis_CM001493.2	132816366	132816677	HERVK_env	+
Papio_anubis_CM001493.2	132816832	132817271	HERVK_3LTR	+
Papio_anubis_CM001494.2	110884803	110885121	HSERVIII_5LTR	+
Papio_anubis_CM001494.2	110886994	110889874	HSERVIII_pol	+
Papio_anubis_CM001494.2	110891302	110891623	HSERVIII_3LTR	+
Papio_anubis_CM001495.2	127306340	127306682	HERVK_5LTR	-
Papio_anubis_CM001495.2	127307612	127309406	HERVK_pol	-
Papio_anubis_CM001495.2	127309474	127310321	HERVK_pro	-
Papio_anubis_CM001495.2	127310756	127311244	HERVK_gag	-
Papio_anubis_CM001495.2	127311894	127312225	HERVK_3LTR	-
Papio_anubis_CM001498.2	38252091	38252400	HERVIPADP_5LTR	-
Papio_anubis_CM001498.2	38256053	38257678	HERVIPADP_pol	-
Papio_anubis_CM001498.2	38258347	38258652	HERVIPADP_gag	-
Papio_anubis_CM001498.2	38260544	38260856	HERVIPADP_3LTR	-
Papio_anubis_CM001501.2	52308345	52308650	HSERVIII_5LTR	+
Papio_anubis_CM001501.2	52315530	52318428	HSERVIII_pol	+
Papio_anubis_CM001501.2	52322846	52323156	HSERVIII_3LTR	+
Papio_anubis_CM001502.2	52818856	52819322	HERVK_5LTR	-
Papio_anubis_CM001502.2	52819430	52819737	HERVK_env	-
Papio_anubis_CM001502.2	52820442	52822187	HERVK_pol	-
Papio_anubis_CM001502.2	52822082	52822861	HERVK_pro	-
Papio_anubis_CM001502.2	52823923	52824393	HERVK_3LTR	-
Papio_anubis_CM001502.2	117391642	117391994	HERVHF_5LTR	+
Papio_anubis_CM001502.2	117393870	117394169	HERVHF_gag	+
Papio_anubis_CM001502.2	117394159	117395001	HERVHF_pro	+
Papio_anubis_CM001502.2	117394357	117397467	HERVHF_pol	+
Papio_anubis_CM001502.2	117398556	117398914	HERVHF_3LTR	+
Papio_anubis_CM001504.2	64105283	64105595	HERVK_5LTR	-
Papio_anubis_CM001504.2	64105856	64107292	HERVK_env	-
Papio_anubis_CM001504.2	64110395	64111132	HERVK_pol	-
Papio_anubis_CM001504.2	64111132	64111887	HERVK_pro	-
Papio_anubis_CM001504.2	64116506	64116823	HERVK_3LTR	-
Papio_anubis_CM001505.2	77153764	77154255	HERVIPADP_5LTR	-
Papio_anubis_CM001505.2	77156433	77159542	HERVIPADP_pol	-
Papio_anubis_CM001505.2	77159938	77160297	HERVIPADP_gag	-
Papio_anubis_CM001505.2	77162075	77162568	HERVIPADP_3LTR	-
Papio_anubis_CM001506.2	23934342	23934664	HERVK_5LTR	-

Papio_anubis_CM001506.2	23935518	23936820	HERVK_pol	-
Papio_anubis_CM001506.2	23936688	23937455	HERVK_pro	-
Papio_anubis_CM001506.2	23938697	23939018	HERVK_3LTR	-
Papio_anubis_CM001507.2	88416681	88416995	HERVIPADP_5LTR	+
Papio_anubis_CM001507.2	88422755	88425194	HERVIPADP_pol	+
Papio_anubis_CM001507.2	88427255	88427561	HERVIPADP_3LTR	+
Papio_anubis_KZ097202.1	3518	3922	HERVIPADP_5LTR	-
Papio_anubis_KZ097202.1	4270	4674	HERVIPADP_env	-
Papio_anubis_KZ097202.1	6592	10063	HERVIPADP_pol	-
Papio_anubis_KZ097202.1	10588	10929	HERVIPADP_gag	-
Papio_anubis_KZ097202.1	12688	13098	HERVIPADP_3LTR	-
Papio_anubis_KZ098017.1	48506	49020	HERVK_5LTR	-
Papio_anubis_KZ098017.1	49096	49428	HERVK_env	-
Papio_anubis_KZ098017.1	49947	51833	HERVK_pol	-
Papio_anubis_KZ098017.1	51795	52604	HERVK_pro	-
Papio_anubis_KZ098017.1	52699	54037	HERVK_gag	-
Papio_anubis_KZ098017.1	55050	55545	HERVK_3LTR	-
Papio_hamadryas_Contig1	56473625	56474091	HERVK_5LTR	-
Papio_hamadryas_Contig1	56474199	56474506	HERVK_env	-
Papio_hamadryas_Contig1	56475211	56476956	HERVK_pol	-
Papio_hamadryas_Contig1	56476851	56477630	HERVK_pro	-
Papio_hamadryas_Contig1	56478692	56479162	HERVK_3LTR	-
Papio_hamadryas_Contig1	99329013	99329464	HERVHF_5LTR	-
Papio_hamadryas_Contig1	99329694	99330959	HERVHF_pol	-
Papio_hamadryas_Contig1	99333104	99333558	HERVHF_3LTR	-
Papio_hamadryas_Contig1	113198831	113199192	HERVHF_5LTR	-
Papio_hamadryas_Contig1	113199501	113200558	HERVHF_env	-
Papio_hamadryas_Contig1	113201618	113203476	HERVHF_pol	-
Papio_hamadryas_Contig1	113205995	113206346	HERVHF_3LTR	-
Papio_hamadryas_Contig10	11644360	11644669	HERVHF_5LTR	+
Papio_hamadryas_Contig10	11646314	11646637	HERVHF_gag	+
Papio_hamadryas_Contig10	11646627	11646962	HERVHF_pro	+
Papio_hamadryas_Contig10	11646967	11649386	HERVHF_pol	+
Papio_hamadryas_Contig10	11649410	11649710	HERVHF_3LTR	+
Papio_hamadryas_Contig10	17080119	17080633	HERVK_5LTR	-
Papio_hamadryas_Contig10	17080733	17081047	HERVK_env	-
Papio_hamadryas_Contig10	17081572	17083313	HERVK_pol	-
Papio_hamadryas_Contig10	17083214	17084155	HERVK_pro	-
Papio_hamadryas_Contig10	17085570	17086084	HERVK_3LTR	-
Papio_hamadryas_Contig10	52221913	52222366	HERVHF_5LTR	-
Papio_hamadryas_Contig10	52222891	52224843	HERVHF_pol	-
Papio_hamadryas_Contig10	52225153	52225494	HERVHF_gag	-
Papio_hamadryas_Contig10	52227105	52227562	HERVHF_3LTR	-
Papio_hamadryas_Contig11	36892214	36892669	HERVHF_5LTR	-

Papio_hamadryas_Contig11	36893396	36895050	HERVHF_pol	-
Papio_hamadryas_Contig11	36894665	36895462	HERVHF_pro	-
Papio_hamadryas_Contig11	36897576	36898033	HERVHF_3LTR	-
Papio_hamadryas_Contig115	814422	814760	HERVK_5LTR	+
Papio_hamadryas_Contig115	822399	823465	HERVK_pro	+
Papio_hamadryas_Contig115	823419	825143	HERVK_pol	+
Papio_hamadryas_Contig115	829096	829428	HERVK_3LTR	+
Papio_hamadryas_Contig119	459284	459909	HERVHF_5LTR	-
Papio_hamadryas_Contig119	460076	460605	HERVHF_env	-
Papio_hamadryas_Contig119	463068	465026	HERVHF_pol	-
Papio_hamadryas_Contig119	465704	466316	HERVHF_3LTR	-
Papio_hamadryas_Contig125	3565270	3565650	HERVHF_5LTR	+
Papio_hamadryas_Contig125	3567145	3567672	HERVHF_gag	+
Papio_hamadryas_Contig125	3567975	3571590	HERVHF_pol	+
Papio_hamadryas_Contig125	3572282	3572644	HERVHF_3LTR	+
Papio_hamadryas_Contig13	40156861	40157226	HERVHF_5LTR	+
Papio_hamadryas_Contig13	40159495	40160148	HERVHF_pro	+
Papio_hamadryas_Contig13	40159525	40161468	HERVHF_pol	+
Papio_hamadryas_Contig13	40162079	40162440	HERVHF_3LTR	+
Papio_hamadryas_Contig13	47820668	47820970	HERVHF_5LTR	+
Papio_hamadryas_Contig13	47823528	47825093	HERVHF_pol	+
Papio_hamadryas_Contig13	47825932	47826231	HERVHF_3LTR	+
Papio_hamadryas_Contig131	1623864	1624166	HERVHF_5LTR	-
Papio_hamadryas_Contig131	1625340	1625675	HERVHF_env	-
Papio_hamadryas_Contig131	1625595	1628249	HERVHF_pol	-
Papio_hamadryas_Contig131	1629398	1634949	HERVHF_gag	-
Papio_hamadryas_Contig131	1636516	1636815	HERVHF_3LTR	-
Papio_hamadryas_Contig16	14072570	14072870	HUERSP_5LTR	-
Papio_hamadryas_Contig16	14077256	14078779	HUERSP_env	-
Papio_hamadryas_Contig16	14082411	14083460	HUERSP_pol	-
Papio_hamadryas_Contig16	14083830	14084159	HUERSP_gag	-
Papio_hamadryas_Contig16	14085357	14085671	HUERSP_3LTR	-
Papio_hamadryas_Contig16	18400404	18400868	HERVHF_5LTR	-
Papio_hamadryas_Contig16	18401033	18401410	HERVHF_env	-
Papio_hamadryas_Contig16	18401750	18403354	HERVHF_pol	-
Papio_hamadryas_Contig16	18403234	18403680	HERVHF_pro	-
Papio_hamadryas_Contig16	18404148	18404534	HERVHF_gag	-
Papio_hamadryas_Contig16	18405799	18406267	HERVHF_3LTR	-
Papio_hamadryas_Contig17	18882275	18882695	HERVHF_5LTR	+
Papio_hamadryas_Contig17	18884176	18884493	HERVHF_gag	+
Papio_hamadryas_Contig17	18884717	18885349	HERVHF_pro	+
Papio_hamadryas_Contig17	18884948	18886437	HERVHF_pol	+
Papio_hamadryas_Contig17	18887307	18887731	HERVHF_3LTR	+
Papio_hamadryas_Contig20	16178332	16178783	HERVHF_5LTR	-

Papio_hamadryas_Contig20	16179207	16181113	HERVHF_pol	-
Papio_hamadryas_Contig20	16181082	16181573	HERVHF_pro	-
Papio_hamadryas_Contig20	16183593	16184046	HERVHF_3LTR	-
Papio_hamadryas_Contig20	23349222	23349579	HERVHF_5LTR	+
Papio_hamadryas_Contig20	23350903	23351412	HERVHF_gag	+
Papio_hamadryas_Contig20	23351799	23352626	HERVHF_pro	+
Papio_hamadryas_Contig20	23351973	23354931	HERVHF_pol	+
Papio_hamadryas_Contig20	23356133	23357450	HERVHF_env	+
Papio_hamadryas_Contig20	23357529	23357892	HERVHF_3LTR	+
Papio_hamadryas_Contig3	25486506	25486842	HERVHF_5LTR	+
Papio_hamadryas_Contig3	25488519	25489154	HERVHF_gag	+
Papio_hamadryas_Contig3	25490861	25491589	HERVHF_pro	+
Papio_hamadryas_Contig3	25490972	25494010	HERVHF_pol	+
Papio_hamadryas_Contig3	25495411	25495950	HERVHF_env	+
Papio_hamadryas_Contig3	25496468	25496812	HERVHF_3LTR	+
Papio_hamadryas_Contig3	46288106	46288542	HERVHF_5LTR	+
Papio_hamadryas_Contig3	46289993	46290598	HERVHF_gag	+
Papio_hamadryas_Contig3	46291079	46294370	HERVHF_pol	+
Papio_hamadryas_Contig3	46294900	46295347	HERVHF_3LTR	+
Papio_hamadryas_Contig30	8985262	8985589	HERVHF_5LTR	-
Papio_hamadryas_Contig30	8986033	8987962	HERVHF_pol	-
Papio_hamadryas_Contig30	8988365	8988679	HERVHF_gag	-
Papio_hamadryas_Contig30	8991065	8991391	HERVHF_3LTR	-
Papio_hamadryas_Contig32	16412755	16413212	HERVHF_5LTR	-
Papio_hamadryas_Contig32	16414368	16416081	HERVHF_pol	-
Papio_hamadryas_Contig32	16415518	16416318	HERVHF_pro	-
Papio_hamadryas_Contig32	16418336	16418800	HERVHF_3LTR	-
Papio_hamadryas_Contig36	16340289	16340679	HERVHF_5LTR	-
Papio_hamadryas_Contig36	16340847	16341425	HERVHF_env	-
Papio_hamadryas_Contig36	16343092	16344741	HERVHF_pol	-
Papio_hamadryas_Contig36	16344506	16345261	HERVHF_pro	-
Papio_hamadryas_Contig36	16345353	16345703	HERVHF_gag	-
Papio_hamadryas_Contig36	16347027	16347425	HERVHF_3LTR	-
Papio_hamadryas_Contig39	17058061	17058428	HERVHF_5LTR	-
Papio_hamadryas_Contig39	17058702	17061290	HERVHF_pol	-
Papio_hamadryas_Contig39	17060691	17061485	HERVHF_pro	-
Papio_hamadryas_Contig39	17061541	17061873	HERVHF_gag	-
Papio_hamadryas_Contig39	17063334	17063701	HERVHF_3LTR	-
Papio_hamadryas_Contig44	2942610	2943045	HERVHF_5LTR	+
Papio_hamadryas_Contig44	2944712	2945035	HERVHF_gag	+
Papio_hamadryas_Contig44	2945252	2945863	HERVHF_pro	+
Papio_hamadryas_Contig44	2945324	2947010	HERVHF_pol	+
Papio_hamadryas_Contig44	2947834	2948271	HERVHF_3LTR	+
Papio_hamadryas_Contig45	14504224	14504581	HERVHF_5LTR	+

Papio_hamadryas_Contig45	14506047	14506361	HERVHF_gag	+
Papio_hamadryas_Contig45	14506444	14506947	HERVHF_pro	+
Papio_hamadryas_Contig45	14506590	14509615	HERVHF_pol	+
Papio_hamadryas_Contig45	14510511	14511113	HERVHF_env	+
Papio_hamadryas_Contig45	14511279	14511643	HERVHF_3LTR	+
Papio_hamadryas_Contig5	27610986	27611417	HERVHF_5LTR	-
Papio_hamadryas_Contig5	27611760	27614524	HERVHF_pol	-
Papio_hamadryas_Contig5	27613889	27614671	HERVHF_pro	-
Papio_hamadryas_Contig5	27614718	27615157	HERVHF_gag	-
Papio_hamadryas_Contig5	27616865	27617297	HERVHF_3LTR	-
Papio_hamadryas_Contig5	36113634	36114015	HERVHF_5LTR	+
Papio_hamadryas_Contig5	36115734	36116054	HERVHF_gag	+
Papio_hamadryas_Contig5	36116253	36116885	HERVHF_pro	+
Papio_hamadryas_Contig5	36116304	36117773	HERVHF_pol	+
Papio_hamadryas_Contig5	36117986	36118609	HERVHF_env	+
Papio_hamadryas_Contig5	36118902	36119292	HERVHF_3LTR	+
Papio_hamadryas_Contig50	15503311	15503742	HERVHF_5LTR	+
Papio_hamadryas_Contig50	15505200	15505631	HERVHF_gag	+
Papio_hamadryas_Contig50	15506017	15507567	HERVHF_pol	+
Papio_hamadryas_Contig50	15508991	15509422	HERVHF_3LTR	+
Papio_hamadryas_Contig51	16108191	16108608	HERVHF_5LTR	+
Papio_hamadryas_Contig51	16110658	16111431	HERVHF_pro	+
Papio_hamadryas_Contig51	16110850	16113386	HERVHF_pol	+
Papio_hamadryas_Contig51	16113927	16114356	HERVHF_3LTR	+
Papio_hamadryas_Contig6	49462200	49462630	HERVHF_5LTR	+
Papio_hamadryas_Contig6	49464076	49464405	HERVHF_gag	+
Papio_hamadryas_Contig6	49465326	49467656	HERVHF_pol	+
Papio_hamadryas_Contig6	49468194	49468634	HERVHF_3LTR	+
Papio_hamadryas_Contig71	8401262	8401633	HERVHF_5LTR	-
Papio_hamadryas_Contig71	8402192	8404323	HERVHF_pol	-
Papio_hamadryas_Contig71	8403751	8404527	HERVHF_pro	-
Papio_hamadryas_Contig71	8404574	8404894	HERVHF_gag	-
Papio_hamadryas_Contig71	8406495	8406856	HERVHF_3LTR	-
Papio_hamadryas_Contig8	58882520	58882838	HSERVIII_5LTR	+
Papio_hamadryas_Contig8	58884711	58887591	HSERVIII_pol	+
Papio_hamadryas_Contig8	58889019	58889340	HSERVIII_3LTR	+
Papio_hamadryas_Contig81	1783953	1784952	HERVHF_5LTR	+
Papio_hamadryas_Contig81	1787164	1795487	HERVHF_pol	+
Papio_hamadryas_Contig81	1797211	1798203	HERVHF_3LTR	+
Papio_hamadryas_Contig9	22826528	22826924	HERVHF_5LTR	+
Papio_hamadryas_Contig9	22828757	22829506	HERVHF_pro	+
Papio_hamadryas_Contig9	22829219	22831338	HERVHF_pol	+
Papio_hamadryas_Contig9	22831757	22832154	HERVHF_3LTR	+
Papio_hamadryas_Contig93	3391924	3392282	HERVHF_5LTR	+

Papio_hamadryas_Contig93 3393784 3394293 HERVHF_gag +
Papio_hamadryas_Contig93 3395068 3396989 HERVHF_pol +
Papio_hamadryas_Contig93 3397405 3397757 HERVHF_3LTR +
Piliocolobus_tephrosceles_PDMG02000017.1 1675943 1676340 HERVHF_5LTR +

Piliocolobus_tephrosceles_PDMG02000017.1 1678196 1679343 HERVHF_pro +
Piliocolobus_tephrosceles_PDMG02000017.1 1678423 1680972 HERVHF_pol +
Piliocolobus_tephrosceles_PDMG02000017.1 1681824 1682226 HERVHF_3LTR +

Piliocolobus_tephrosceles_PDMG02000017.1 27368488 27368801 HERVHF_5LTR +

Piliocolobus_tephrosceles_PDMG02000017.1 27370270 27370713 HERVHF_gag +
Piliocolobus_tephrosceles_PDMG02000017.1 27371133 27371807 HERVHF_pro +
Piliocolobus_tephrosceles_PDMG02000017.1 27371283 27373290 HERVHF_pol +
Piliocolobus_tephrosceles_PDMG02000017.1 27374042 27374357 HERVHF_3LTR +

Piliocolobus_tephrosceles_PDMG02000027.1 4925586 4926027 HERVHF_5LTR +

Piliocolobus_tephrosceles_PDMG02000027.1 4928008 4928832 HERVHF_pro +
Piliocolobus_tephrosceles_PDMG02000027.1 4928332 4929003 HERVHF_pol +
Piliocolobus_tephrosceles_PDMG02000027.1 4929898 4930275 HERVHF_env +
Piliocolobus_tephrosceles_PDMG02000027.1 4930770 4931212 HERVHF_3LTR +

Piliocolobus_tephrosceles_PDMG02000045.1 13103273 13103672 HERVHF_5LTR +

Piliocolobus_tephrosceles_PDMG02000045.1 13106307 13108345 HERVHF_pol +
Piliocolobus_tephrosceles_PDMG02000045.1 13108830 13109238 HERVHF_3LTR +

Piliocolobus_tephrosceles_PDMG02000045.1 26058991 26059494 HERVK_5LTR +
Piliocolobus_tephrosceles_PDMG02000045.1 26060998 26061780 HERVK_pro +
Piliocolobus_tephrosceles_PDMG02000045.1 26061703 26063360 HERVK_pol +
Piliocolobus_tephrosceles_PDMG02000045.1 26064045 26064385 HERVK_env +
Piliocolobus_tephrosceles_PDMG02000045.1 26064466 26064966 HERVK_3LTR +
Piliocolobus_tephrosceles_PDMG02000046.1 9519389 9519801 HERVHF_5LTR +

Piliocolobus_tephrosceles_PDMG02000046.1 9521177 9521641 HERVHF_gag +
Piliocolobus_tephrosceles_PDMG02000046.1 9522449 9523150 HERVHF_pro +
Piliocolobus_tephrosceles_PDMG02000046.1 9522515 9525025 HERVHF_pol +
Piliocolobus_tephrosceles_PDMG02000046.1 9525656 9526069 HERVHF_3LTR +

Piliocolobus_tephrosceles_PDMG02000046.1 34488419 34488822 HERVHF_5LTR +

Piliocolobus_tephrosceles_PDMG02000046.1 34490136 34490600 HERVHF_gag +
Piliocolobus_tephrosceles_PDMG02000046.1 34490520 34490876 HERVHF_pro +

Piliocolobus_tephrosceles_PDMG02000046.1	34491050	34492999	HERVHF_pol	+
Piliocolobus_tephrosceles_PDMG02000046.1	34494006	34494410	HERVHF_3LTR	+
Piliocolobus_tephrosceles_PDMG02000070.1	36695123	36695541	HERVHF_5LTR	+
Piliocolobus_tephrosceles_PDMG02000070.1	36697650	36698741	HERVHF_pro	+
Piliocolobus_tephrosceles_PDMG02000070.1	36698115	36700225	HERVHF_pol	+
Piliocolobus_tephrosceles_PDMG02000070.1	36700696	36701106	HERVHF_3LTR	+
Piliocolobus_tephrosceles_PDMG02000082.1	3316242	3316684	HERVHF_5LTR	+
Piliocolobus_tephrosceles_PDMG02000082.1	3318191	3318490	HERVHF_gag	+
Piliocolobus_tephrosceles_PDMG02000082.1	3319148	3319834	HERVHF_pro	+
Piliocolobus_tephrosceles_PDMG02000082.1	3319331	3321708	HERVHF_pol	+
Piliocolobus_tephrosceles_PDMG02000082.1	3322282	3322734	HERVHF_3LTR	+
Piliocolobus_tephrosceles_PDMG02000090.1	2738526	2738842	HERVHF_5LTR	-
Piliocolobus_tephrosceles_PDMG02000090.1	2739128	2740237	HERVHF_env	-
Piliocolobus_tephrosceles_PDMG02000090.1	2740663	2744320	HERVHF_pol	-
Piliocolobus_tephrosceles_PDMG02000090.1	2743637	2744590	HERVHF_pro	-
Piliocolobus_tephrosceles_PDMG02000090.1	2744618	2745193	HERVHF_gag	-
Piliocolobus_tephrosceles_PDMG02000090.1	2746877	2747193	HERVHF_3LTR	-
Piliocolobus_tephrosceles_PDMG02000091.1	5507238	5507626	HERVHF_5LTR	+
Piliocolobus_tephrosceles_PDMG02000091.1	5509712	5510143	HERVHF_pro	+
Piliocolobus_tephrosceles_PDMG02000091.1	5510302	5512917	HERVHF_pol	+
Piliocolobus_tephrosceles_PDMG02000091.1	5513583	5513962	HERVHF_3LTR	+
Piliocolobus_tephrosceles_PDMG02000098.1	18752699	18753158	HERVHF_5LTR	-
Piliocolobus_tephrosceles_PDMG02000098.1	18754176	18756218	HERVHF_pol	-
Piliocolobus_tephrosceles_PDMG02000098.1	18756521	18756877	HERVHF_gag	-
Piliocolobus_tephrosceles_PDMG02000098.1	18758359	18758802	HERVHF_3LTR	-
Piliocolobus_tephrosceles_PDMG02000108.1	2542050	2542456	HERVHF_5LTR	-
Piliocolobus_tephrosceles_PDMG02000108.1	2542612	2543196	HERVHF_env	-
Piliocolobus_tephrosceles_PDMG02000108.1	2543806	2545738	HERVHF_pol	-
Piliocolobus_tephrosceles_PDMG02000108.1	2545124	2545861	HERVHF_pro	-
Piliocolobus_tephrosceles_PDMG02000108.1	2545957	2546376	HERVHF_gag	-
Piliocolobus_tephrosceles_PDMG02000108.1	2548109	2548525	HERVHF_3LTR	-

Piliocolobus_tephrosceles_PDMG02000132.1	6638262	6638781	HERVK_5LTR	-
Piliocolobus_tephrosceles_PDMG02000132.1	6639541	6641623	HERVK_pol	-
Piliocolobus_tephrosceles_PDMG02000132.1	6641371	6642316	HERVK_pro	-
Piliocolobus_tephrosceles_PDMG02000132.1	6644031	6644555	HERVK_3LTR	-
Piliocolobus_tephrosceles_PDMG02000136.1	2749885	2750222	HERVHF_5LTR	-
Piliocolobus_tephrosceles_PDMG02000136.1	2750701	2753382	HERVHF_env	-
Piliocolobus_tephrosceles_PDMG02000136.1	2754160	2756389	HERVHF_pol	-
Piliocolobus_tephrosceles_PDMG02000136.1	2755811	2756650	HERVHF_pro	-
Piliocolobus_tephrosceles_PDMG02000136.1	2756945	2757259	HERVHF_gag	-
Piliocolobus_tephrosceles_PDMG02000136.1	2761261	2761583	HERVHF_3LTR	-
Piliocolobus_tephrosceles_PDMG02000163.1	2170393	2170854	HERVHF_5LTR	-
Piliocolobus_tephrosceles_PDMG02000163.1	2171009	2171374	HERVHF_env	-
Piliocolobus_tephrosceles_PDMG02000163.1	2171738	2173742	HERVHF_pol	-
Piliocolobus_tephrosceles_PDMG02000163.1	2173407	2174045	HERVHF_pro	-
Piliocolobus_tephrosceles_PDMG02000163.1	2174150	2174473	HERVHF_gag	-
Piliocolobus_tephrosceles_PDMG02000163.1	2176393	2176838	HERVHF_3LTR	-
Piliocolobus_tephrosceles_PDMG02000167.1	2774572	2774933	HERVHF_5LTR	-
Piliocolobus_tephrosceles_PDMG02000167.1	2775109	2775498	HERVHF_env	-
Piliocolobus_tephrosceles_PDMG02000167.1	2778922	2780866	HERVHF_pol	-
Piliocolobus_tephrosceles_PDMG02000167.1	2780231	2780866	HERVHF_pro	-
Piliocolobus_tephrosceles_PDMG02000167.1	2781617	2782261	HERVHF_gag	-
Piliocolobus_tephrosceles_PDMG02000167.1	2783329	2783678	HERVHF_3LTR	-
Piliocolobus_tephrosceles_PDMG02000167.1	5854881	5855191	HERVHF_5LTR	+
Piliocolobus_tephrosceles_PDMG02000167.1	5863929	5864756	HERVHF_pro	+
Piliocolobus_tephrosceles_PDMG02000167.1	5864175	5865868	HERVHF_pol	+
Piliocolobus_tephrosceles_PDMG02000167.1	5866557	5866861	HERVHF_3LTR	+
Piliocolobus_tephrosceles_PDMG02000174.1	1870054	1870406	HERVK_5LTR	-
Piliocolobus_tephrosceles_PDMG02000174.1	1871310	1873218	HERVK_pol	-
Piliocolobus_tephrosceles_PDMG02000174.1	1873125	1874057	HERVK_pro	-
Piliocolobus_tephrosceles_PDMG02000174.1	1875556	1875909	HERVK_3LTR	-
Piliocolobus_tephrosceles_PDMG02000175.1	13496879	13497239	HERVHF_5LTR	-
Piliocolobus_tephrosceles_PDMG02000175.1	13497464	13498006	HERVHF_env	-
Piliocolobus_tephrosceles_PDMG02000175.1	13499019	13502461	HERVHF_pol	-
Piliocolobus_tephrosceles_PDMG02000175.1	13505042	13505410	HERVHF_3LTR	-

Piliocolobus_tephrosceles_PDMG02000180.1	4787822	4788334	HERVK_5LTR	+
Piliocolobus_tephrosceles_PDMG02000180.1	4789744	4790685	HERVK_pro	+
Piliocolobus_tephrosceles_PDMG02000180.1	4790682	4791368	HERVK_pol	+
Piliocolobus_tephrosceles_PDMG02000180.1	4792273	4792575	HERVK_env	+
Piliocolobus_tephrosceles_PDMG02000180.1	4792782	4793293	HERVK_3LTR	+
Piliocolobus_tephrosceles_PDMG02000180.1	4981295	4981639	HERVHF_5LTR	-
Piliocolobus_tephrosceles_PDMG02000180.1	4982118	4984218	HERVHF_pol	-
Piliocolobus_tephrosceles_PDMG02000180.1	4983687	4984697	HERVHF_pro	-
Piliocolobus_tephrosceles_PDMG02000180.1	4986511	4986852	HERVHF_3LTR	-
Piliocolobus_tephrosceles_PDMG02000183.1	6252580	6252906	HERVHF_5LTR	-
Piliocolobus_tephrosceles_PDMG02000183.1	6253153	6253545	HERVHF_env	-
Piliocolobus_tephrosceles_PDMG02000183.1	6254457	6257089	HERVHF_pol	-
Piliocolobus_tephrosceles_PDMG02000183.1	6256873	6257253	HERVHF_pro	-
Piliocolobus_tephrosceles_PDMG02000183.1	6257323	6257925	HERVHF_gag	-
Piliocolobus_tephrosceles_PDMG02000183.1	6259385	6259708	HERVHF_3LTR	-
Piliocolobus_tephrosceles_PDMG02000190.1	1820923	1821368	HERVHF_5LTR	-
Piliocolobus_tephrosceles_PDMG02000190.1	1826027	1828176	HERVHF_pol	-
Piliocolobus_tephrosceles_PDMG02000190.1	1828731	1830133	HERVHF_gag	-
Piliocolobus_tephrosceles_PDMG02000190.1	1830830	1831279	HERVHF_3LTR	-
Piliocolobus_tephrosceles_PDMG02000192.1	3809365	3809730	HERVHF_5LTR	-
Piliocolobus_tephrosceles_PDMG02000192.1	3810597	3812261	HERVHF_pol	-
Piliocolobus_tephrosceles_PDMG02000192.1	3811767	3812300	HERVHF_pro	-
Piliocolobus_tephrosceles_PDMG02000192.1	3814528	3814892	HERVHF_3LTR	-
Piliocolobus_tephrosceles_PDMG02000200.1	1384317	1384673	HERVHF_5LTR	-
Piliocolobus_tephrosceles_PDMG02000200.1	1386561	1388299	HERVHF_pol	-
Piliocolobus_tephrosceles_PDMG02000200.1	1387763	1388299	HERVHF_pro	-
Piliocolobus_tephrosceles_PDMG02000200.1	1389423	1389776	HERVHF_3LTR	-
Piliocolobus_tephrosceles_PDMG02000239.1	5349316	5349679	HERVHF_5LTR	-
Piliocolobus_tephrosceles_PDMG02000239.1	5350112	5351160	HERVHF_pol	-
Piliocolobus_tephrosceles_PDMG02000239.1	5353562	5353925	HERVHF_3LTR	-
Piliocolobus_tephrosceles_PDMG02000261.1	3351440	3351764	HERVK_5LTR	-
Piliocolobus_tephrosceles_PDMG02000261.1	3352614	3354039	HERVK_pol	-

Piliocolobus_tephrosceles_PDMG02000261.1	3353784	3354722	HERVK_pro	-
Piliocolobus_tephrosceles_PDMG02000261.1	3355774	3356098	HERVK_3LTR	-
Piliocolobus_tephrosceles_PDMG02000276.1	966776	967177	HERVHF_5LTR	-
Piliocolobus_tephrosceles_PDMG02000276.1	967603	969514	HERVHF_pol	-
Piliocolobus_tephrosceles_PDMG02000276.1	969939	970241	HERVHF_gag	-
Piliocolobus_tephrosceles_PDMG02000276.1	972494	972895	HERVHF_3LTR	-
Piliocolobus_tephrosceles_PDMG02000298.1	1273022	1273387	HUERSP_5LTR	+
Piliocolobus_tephrosceles_PDMG02000298.1	1275764	1276156	HUERSP_gag	+
Piliocolobus_tephrosceles_PDMG02000298.1	1276746	1277054	HUERSP_pro	+
Piliocolobus_tephrosceles_PDMG02000298.1	1277057	1277422	HUERSP_pol	+
Piliocolobus_tephrosceles_PDMG02000298.1	1282646	1283019	HUERSP_3LTR	+
Piliocolobus_tephrosceles_PDMG02000303.1	1590178	1590544	HERVHF_5LTR	+
Piliocolobus_tephrosceles_PDMG02000303.1	1592083	1592547	HERVHF_gag	+
Piliocolobus_tephrosceles_PDMG02000303.1	1592953	1593711	HERVHF_pro	+
Piliocolobus_tephrosceles_PDMG02000303.1	1593085	1595640	HERVHF_pol	+
Piliocolobus_tephrosceles_PDMG02000303.1	1596062	1596385	HERVHF_env	+
Piliocolobus_tephrosceles_PDMG02000303.1	1596647	1597033	HERVHF_3LTR	+
Piliocolobus_tephrosceles_PDMG02000376.1	6220079	6220473	HERVHF_5LTR	+
Piliocolobus_tephrosceles_PDMG02000376.1	6222061	6222369	HERVHF_gag	+
Piliocolobus_tephrosceles_PDMG02000376.1	6222506	6223228	HERVHF_pro	+
Piliocolobus_tephrosceles_PDMG02000376.1	6222590	6224837	HERVHF_pol	+
Piliocolobus_tephrosceles_PDMG02000376.1	6225676	6226074	HERVHF_3LTR	+
Piliocolobus_tephrosceles_PDMG02029865.1	53484	53864	HERVHF_5LTR	+
Piliocolobus_tephrosceles_PDMG02029865.1	55216	55527	HERVHF_gag	+
Piliocolobus_tephrosceles_PDMG02029865.1	55737	57346	HERVHF_pol	+
Piliocolobus_tephrosceles_PDMG02029865.1	58636	59019	HERVHF_env	+
Piliocolobus_tephrosceles_PDMG02029865.1	59149	59535	HERVHF_3LTR	+
Piliocolobus_tephrosceles_PDMG02030611.1	169967	170425	HERVHF_5LTR	-
Piliocolobus_tephrosceles_PDMG02030611.1	171078	174789	HERVHF_pol	-
Piliocolobus_tephrosceles_PDMG02030611.1	174136	174912	HERVHF_pro	-
Piliocolobus_tephrosceles_PDMG02030611.1	175395	175814	HERVHF_gag	-
Piliocolobus_tephrosceles_PDMG02030611.1	177113	177564	HERVHF_3LTR	-

Piliocolobus_tephrosceles_PDMG02031256.1	309761	310158	HERVK_5LTR	+
Piliocolobus_tephrosceles_PDMG02031256.1	311095	311793	HERVK_pro	+
Piliocolobus_tephrosceles_PDMG02031256.1	311728	312498	HERVK_pol	+
Piliocolobus_tephrosceles_PDMG02031256.1	313806	314216	HERVK_3LTR	+
Piliocolobus_tephrosceles_PDMG02031489.1	1770002	1770560	HERVHF_5LTR	+
Piliocolobus_tephrosceles_PDMG02031489.1	1773378	1773734	HERVHF_gag	+
Piliocolobus_tephrosceles_PDMG02031489.1	1773860	1774576	HERVHF_pro	+
Piliocolobus_tephrosceles_PDMG02031489.1	1773938	1776277	HERVHF_pol	+
Piliocolobus_tephrosceles_PDMG02031489.1	1783573	1784138	HERVHF_3LTR	+
Piliocolobus_tephrosceles_PDMG02031618.1	6436991	6437348	HERVHF_5LTR	-
Piliocolobus_tephrosceles_PDMG02031618.1	6438353	6440055	HERVHF_pol	-
Piliocolobus_tephrosceles_PDMG02031618.1	6439399	6440184	HERVHF_pro	-
Piliocolobus_tephrosceles_PDMG02031618.1	6442213	6442574	HERVHF_3LTR	-
Piliocolobus_tephrosceles_PDMG02032196.1	5073863	5074254	HERVHF_5LTR	+
Piliocolobus_tephrosceles_PDMG02032196.1	5076061	5076516	HERVHF_gag	+
Piliocolobus_tephrosceles_PDMG02032196.1	5076523	5077443	HERVHF_pro	+
Piliocolobus_tephrosceles_PDMG02032196.1	5076946	5079569	HERVHF_pol	+
Piliocolobus_tephrosceles_PDMG02032196.1	5080995	5081670	HERVHF_env	+
Piliocolobus_tephrosceles_PDMG02032196.1	5082001	5082380	HERVHF_3LTR	+
Piliocolobus_tephrosceles_PDMG02032196.1	24765062	24765455	HERVHF_5LTR	-
Piliocolobus_tephrosceles_PDMG02032196.1	24765625	24766202	HERVHF_env	-
Piliocolobus_tephrosceles_PDMG02032196.1	24766679	24769026	HERVHF_pol	-
Piliocolobus_tephrosceles_PDMG02032196.1	24768349	24769155	HERVHF_pro	-
Piliocolobus_tephrosceles_PDMG02032196.1	24769208	24769537	HERVHF_gag	-
Piliocolobus_tephrosceles_PDMG02032196.1	24771337	24771712	HERVHF_3LTR	-
Pithecia_pithecia_tarseq_1	39782320	39782707	HERVHF_5LTR	+
Pithecia_pithecia_tarseq_1	39784743	39785075	HERVHF_gag	+
Pithecia_pithecia_tarseq_1	39785582	39786301	HERVHF_pol	+
Pithecia_pithecia_tarseq_1	39788521	39788913	HERVHF_3LTR	+
Pithecia_pithecia_tarseq_113	91836	92176	HERVHF_5LTR	-
Pithecia_pithecia_tarseq_113	92400	92702	HERVHF_env	-
Pithecia_pithecia_tarseq_113	92993	93740	HERVHF_pol	-
Pithecia_pithecia_tarseq_113	94218	94745	HERVHF_gag	-
Pithecia_pithecia_tarseq_113	95950	96299	HERVHF_3LTR	-
Pithecia_pithecia_tarseq_16	9127813	9128180	HERVHF_5LTR	-
Pithecia_pithecia_tarseq_16	9128756	9130152	HERVHF_pol	-

Pithecia_pithecia_tarseq_16	9131035	9131538	HERVHF_gag	-
Pithecia_pithecia_tarseq_16	9133344	9133705	HERVHF_3LTR	-
Pithecia_pithecia_tarseq_21	55910	56529	HERVHF_5LTR	-
Pithecia_pithecia_tarseq_21	58300	59157	HERVHF_pol	-
Pithecia_pithecia_tarseq_21	59153	59764	HERVHF_pro	-
Pithecia_pithecia_tarseq_21	60326	60709	HERVHF_gag	-
Pithecia_pithecia_tarseq_21	62030	62647	HERVHF_3LTR	-
Pithecia_pithecia_tarseq_22	10724611	10725107	HERVHF_5LTR	+
Pithecia_pithecia_tarseq_22	10728630	10729634	HERVHF_pol	+
Pithecia_pithecia_tarseq_22	10730924	10731435	HERVHF_3LTR	+
Pithecia_pithecia_tarseq_289	1132978	1133506	HERVHF_5LTR	-
Pithecia_pithecia_tarseq_289	1135593	1136303	HERVHF_pol	-
Pithecia_pithecia_tarseq_289	1139294	1139833	HERVHF_3LTR	-
Pithecia_pithecia_tarseq_309	541887	542287	HERVHF_5LTR	+
Pithecia_pithecia_tarseq_309	543513	543818	HERVHF_pro	+
Pithecia_pithecia_tarseq_309	543844	546178	HERVHF_pol	+
Pithecia_pithecia_tarseq_309	548227	548627	HERVHF_3LTR	+
Pithecia_pithecia_tarseq_60	443111	443568	HERVHF_5LTR	-
Pithecia_pithecia_tarseq_60	444406	445748	HERVHF_pol	-
Pithecia_pithecia_tarseq_60	446336	446788	HERVHF_gag	-
Pithecia_pithecia_tarseq_60	448725	449186	HERVHF_3LTR	-
Pithecia_pithecia_tarseq_62	478764	479096	HSERVIII_5LTR	+
Pithecia_pithecia_tarseq_62	481494	483886	HSERVIII_pol	+
Pithecia_pithecia_tarseq_62	484771	485105	HSERVIII_3LTR	+
Pithecia_pithecia_tarseq_647	29477	29963	HERVHF_5LTR	+
Pithecia_pithecia_tarseq_647	35431	36687	HERVHF_pol	+
Pithecia_pithecia_tarseq_647	38602	39098	HERVHF_3LTR	+
Pithecia_pithecia_tarseq_72	1916066	1916385	HERVHF_5LTR	-
Pithecia_pithecia_tarseq_72	1916802	1917116	HERVHF_env	-
Pithecia_pithecia_tarseq_72	1917501	1917974	HERVHF_pol	-
Pithecia_pithecia_tarseq_72	1918101	1918703	HERVHF_pro	-
Pithecia_pithecia_tarseq_72	1920454	1920766	HERVHF_3LTR	-
Pithecia_pithecia_tarseq_98	1488724	1489190	HERVHF_5LTR	-
Pithecia_pithecia_tarseq_98	1491472	1493910	HERVHF_pol	-
Pithecia_pithecia_tarseq_98	1496488	1496959	HERVHF_3LTR	-
Pongo_abelii_CM009262.2	2031053	2031430	HERVK_5LTR	-
Pongo_abelii_CM009262.2	2032354	2034243	HERVK_pol	-
Pongo_abelii_CM009262.2	2034162	2035071	HERVK_pro	-
Pongo_abelii_CM009262.2	2036055	2036436	HERVK_3LTR	-
Pongo_abelii_CM009262.2	7686928	7687328	HERVHF_5LTR	-
Pongo_abelii_CM009262.2	7687782	7689865	HERVHF_pol	-
Pongo_abelii_CM009262.2	7692490	7692896	HERVHF_3LTR	-
Pongo_abelii_CM009262.2	12381425	12381864	HERVHF_5LTR	+
Pongo_abelii_CM009262.2	12383513	12383812	HERVHF_gag	+

Pongo_abelii_CM009262.2	12383913	12384641	HERVHF_pro	+
Pongo_abelii_CM009262.2	12384066	12384689	HERVHF_pol	+
Pongo_abelii_CM009262.2	12386603	12387034	HERVHF_3LTR	+
Pongo_abelii_CM009262.2	17574035	17574466	HERVHF_5LTR	+
Pongo_abelii_CM009262.2	17575984	17576385	HERVHF_gag	+
Pongo_abelii_CM009262.2	17576558	17577262	HERVHF_pro	+
Pongo_abelii_CM009262.2	17576624	17578431	HERVHF_pol	+
Pongo_abelii_CM009262.2	17579812	17580242	HERVHF_3LTR	+
Pongo_abelii_CM009262.2	20045039	20045492	HERVHF_5LTR	-
Pongo_abelii_CM009262.2	20046528	20048526	HERVHF_pol	-
Pongo_abelii_CM009262.2	20048029	20048778	HERVHF_pro	-
Pongo_abelii_CM009262.2	20048861	20049277	HERVHF_gag	-
Pongo_abelii_CM009262.2	20050895	20051348	HERVHF_3LTR	-
Pongo_abelii_CM009262.2	20267865	20268319	HERVHF_5LTR	-
Pongo_abelii_CM009262.2	20268919	20270532	HERVHF_pol	-
Pongo_abelii_CM009262.2	20269969	20270820	HERVHF_pro	-
Pongo_abelii_CM009262.2	20270896	20271381	HERVHF_gag	-
Pongo_abelii_CM009262.2	20272884	20273336	HERVHF_3LTR	-
Pongo_abelii_CM009262.2	24787365	24787722	HERVHF_5LTR	+
Pongo_abelii_CM009262.2	24789781	24790716	HERVHF_pro	+
Pongo_abelii_CM009262.2	24789988	24792275	HERVHF_pol	+
Pongo_abelii_CM009262.2	24793156	24793511	HERVHF_3LTR	+
Pongo_abelii_CM009262.2	27414830	27415264	HERVHF_5LTR	+
Pongo_abelii_CM009262.2	27416775	27417098	HERVHF_gag	+
Pongo_abelii_CM009262.2	27417512	27419597	HERVHF_pol	+
Pongo_abelii_CM009262.2	27419981	27420420	HERVHF_3LTR	+
Pongo_abelii_CM009262.2	33492013	33492358	HERVHF_5LTR	-
Pongo_abelii_CM009262.2	33492817	33494992	HERVHF_pol	-
Pongo_abelii_CM009262.2	33494432	33495190	HERVHF_pro	-
Pongo_abelii_CM009262.2	33495268	33495651	HERVHF_gag	-
Pongo_abelii_CM009262.2	33497235	33497583	HERVHF_3LTR	-
Pongo_abelii_CM009262.2	66363526	66363978	HERVHF_5LTR	-
Pongo_abelii_CM009262.2	66364390	66366522	HERVHF_pol	-
Pongo_abelii_CM009262.2	66365980	66366810	HERVHF_pro	-
Pongo_abelii_CM009262.2	66366817	66367134	HERVHF_gag	-
Pongo_abelii_CM009262.2	66368888	66369340	HERVHF_3LTR	-
Pongo_abelii_CM009262.2	128252916	128253317	HERVHF_5LTR	+
Pongo_abelii_CM009262.2	128254629	128255099	HERVHF_gag	+
Pongo_abelii_CM009262.2	128255220	128256065	HERVHF_pro	+
Pongo_abelii_CM009262.2	128255463	128257775	HERVHF_pol	+
Pongo_abelii_CM009262.2	128258702	128259098	HERVHF_3LTR	+
Pongo_abelii_CM009262.2	137147267	137147669	HERVHF_5LTR	+
Pongo_abelii_CM009262.2	137149297	137149626	HERVHF_gag	+
Pongo_abelii_CM009262.2	137149663	137150481	HERVHF_pro	+

Pongo_abelii_CM009262.2	137149921	137151995	HERVHF_pol	+
Pongo_abelii_CM009262.2	137152502	137152909	HERVHF_3LTR	+
Pongo_abelii_CM009262.2	143077354	143077811	HERVHF_5LTR	-
Pongo_abelii_CM009262.2	143078260	143080150	HERVHF_pol	-
Pongo_abelii_CM009262.2	143080629	143081045	HERVHF_gag	-
Pongo_abelii_CM009262.2	143082726	143083184	HERVHF_3LTR	-
Pongo_abelii_CM009262.2	143775240	143775691	HERVHF_5LTR	-
Pongo_abelii_CM009262.2	143776147	143778235	HERVHF_pol	-
Pongo_abelii_CM009262.2	143777693	143778478	HERVHF_pro	-
Pongo_abelii_CM009262.2	143778594	143779078	HERVHF_gag	-
Pongo_abelii_CM009262.2	143780632	143781073	HERVHF_3LTR	-
Pongo_abelii_CM009262.2	150433010	150433372	HERVHF_5LTR	+
Pongo_abelii_CM009262.2	150434984	150435310	HERVHF_gag	+
Pongo_abelii_CM009262.2	150435746	150437537	HERVHF_pol	+
Pongo_abelii_CM009262.2	150438172	150438520	HERVHF_3LTR	+
Pongo_abelii_CM009262.2	154405136	154405575	HERVHF_5LTR	+
Pongo_abelii_CM009262.2	154407414	154407779	HERVHF_gag	+
Pongo_abelii_CM009262.2	154407931	154408638	HERVHF_pro	+
Pongo_abelii_CM009262.2	154407997	154410233	HERVHF_pol	+
Pongo_abelii_CM009262.2	154410630	154411078	HERVHF_3LTR	+
Pongo_abelii_CM009262.2	156880879	156881292	HERVHF_5LTR	-
Pongo_abelii_CM009262.2	156881773	156883855	HERVHF_pol	-
Pongo_abelii_CM009262.2	156883292	156884092	HERVHF_pro	-
Pongo_abelii_CM009262.2	156884159	156884467	HERVHF_gag	-
Pongo_abelii_CM009262.2	156886127	156886538	HERVHF_3LTR	-
Pongo_abelii_CM009262.2	157112803	157113221	HERVHF_5LTR	-
Pongo_abelii_CM009262.2	157113388	157113744	HERVHF_env	-
Pongo_abelii_CM009262.2	157114689	157116507	HERVHF_pol	-
Pongo_abelii_CM009262.2	157116136	157116906	HERVHF_pro	-
Pongo_abelii_CM009262.2	157116965	157117399	HERVHF_gag	-
Pongo_abelii_CM009262.2	157118789	157119207	HERVHF_3LTR	-
Pongo_abelii_CM009262.2	181919184	181919537	HERVIPADP_5LTR	+
Pongo_abelii_CM009262.2	181921264	181921641	HERVIPADP_gag	+
Pongo_abelii_CM009262.2	181922152	181925613	HERVIPADP_pol	+
Pongo_abelii_CM009262.2	181926869	181927477	HERVIPADP_env	+
Pongo_abelii_CM009262.2	181927650	181928012	HERVIPADP_3LTR	+
Pongo_abelii_CM009262.2	190775617	190776075	HERVHF_5LTR	-
Pongo_abelii_CM009262.2	190776662	190778551	HERVHF_pol	-
Pongo_abelii_CM009262.2	190778009	190778794	HERVHF_pro	-
Pongo_abelii_CM009262.2	190778882	190779307	HERVHF_gag	-
Pongo_abelii_CM009262.2	190781214	190781668	HERVHF_3LTR	-
Pongo_abelii_CM009263.2	25995419	25995830	HERVHF_5LTR	+
Pongo_abelii_CM009263.2	25998083	25998658	HERVHF_pro	+
Pongo_abelii_CM009263.2	25998308	26000009	HERVHF_pol	+

Pongo_abelii_CM009263.2	26000631	26001041	HERVHF_3LTR	+
Pongo_abelii_CM009263.2	27356039	27356470	HERVHF_5LTR	-
Pongo_abelii_CM009263.2	27357461	27358991	HERVHF_pol	-
Pongo_abelii_CM009263.2	27358410	27359048	HERVHF_pro	-
Pongo_abelii_CM009263.2	27359265	27359564	HERVHF_gag	-
Pongo_abelii_CM009263.2	27361467	27361898	HERVHF_3LTR	-
Pongo_abelii_CM009263.2	37915349	37915740	HERVHF_5LTR	-
Pongo_abelii_CM009263.2	37916326	37918447	HERVHF_pol	-
Pongo_abelii_CM009263.2	37917809	37918639	HERVHF_pro	-
Pongo_abelii_CM009263.2	37921067	37921455	HERVHF_3LTR	-
Pongo_abelii_CM009263.2	41220858	41221259	HERVHF_5LTR	-
Pongo_abelii_CM009263.2	41221722	41223874	HERVHF_pol	-
Pongo_abelii_CM009263.2	41223473	41224225	HERVHF_pro	-
Pongo_abelii_CM009263.2	41224343	41224654	HERVHF_gag	-
Pongo_abelii_CM009263.2	41226425	41226824	HERVHF_3LTR	-
Pongo_abelii_CM009263.2	44516283	44516653	HERVHF_5LTR	+
Pongo_abelii_CM009263.2	44518177	44518713	HERVHF_gag	+
Pongo_abelii_CM009263.2	44519229	44520941	HERVHF_pol	+
Pongo_abelii_CM009263.2	44521999	44522377	HERVHF_3LTR	+
Pongo_abelii_CM009263.2	50233905	50234315	HERVHF_5LTR	-
Pongo_abelii_CM009263.2	50234706	50236925	HERVHF_pol	-
Pongo_abelii_CM009263.2	50237230	50237580	HERVHF_gag	-
Pongo_abelii_CM009263.2	50239222	50239627	HERVHF_3LTR	-
Pongo_abelii_CM009263.2	70793958	70794283	HERVHF_5LTR	+
Pongo_abelii_CM009263.2	70796112	70796492	HERVHF_gag	+
Pongo_abelii_CM009263.2	70796767	70798755	HERVHF_pol	+
Pongo_abelii_CM009263.2	70799331	70799658	HERVHF_3LTR	+
Pongo_abelii_CM009263.2	88996285	88996717	HERVHF_5LTR	-
Pongo_abelii_CM009263.2	88997252	88999176	HERVHF_pol	-
Pongo_abelii_CM009263.2	88998637	88999416	HERVHF_pro	-
Pongo_abelii_CM009263.2	89001260	89001684	HERVHF_3LTR	-
Pongo_abelii_CM009263.2	89157535	89157872	HERVK_5LTR	-
Pongo_abelii_CM009263.2	89158136	89158456	HERVK_env	-
Pongo_abelii_CM009263.2	89159528	89160298	HERVK_pol	-
Pongo_abelii_CM009263.2	89160682	89161107	HERVK_pro	-
Pongo_abelii_CM009263.2	89160972	89162362	HERVK_gag	-
Pongo_abelii_CM009263.2	89162821	89163154	HERVK_3LTR	-
Pongo_abelii_CM009263.2	96038159	96038623	HERVHF_5LTR	+
Pongo_abelii_CM009263.2	96040802	96041680	HERVHF_pro	+
Pongo_abelii_CM009263.2	96041087	96043181	HERVHF_pol	+
Pongo_abelii_CM009263.2	96043584	96044065	HERVHF_3LTR	+
Pongo_abelii_CM009263.2	104708825	104709278	HERVHF_5LTR	-
Pongo_abelii_CM009263.2	104709853	104711862	HERVHF_pol	-
Pongo_abelii_CM009263.2	104711302	104711853	HERVHF_pro	-

Pongo_abelii_CM009263.2	104714293	104714750	HERVHF_3LTR	-
Pongo_abelii_CM009264.2	26196467	26196882	HERVHF_5LTR	+
Pongo_abelii_CM009264.2	26198230	26198607	HERVHF_gag	+
Pongo_abelii_CM009264.2	26198672	26199475	HERVHF_pro	+
Pongo_abelii_CM009264.2	26199170	26200782	HERVHF_pol	+
Pongo_abelii_CM009264.2	26202041	26202456	HERVHF_3LTR	+
Pongo_abelii_CM009264.2	66878044	66878498	HERVHF_5LTR	-
Pongo_abelii_CM009264.2	66878951	66881064	HERVHF_pol	-
Pongo_abelii_CM009264.2	66881314	66881730	HERVHF_gag	-
Pongo_abelii_CM009264.2	66883270	66883722	HERVHF_3LTR	-
Pongo_abelii_CM009264.2	73870221	73870535	HERVHF_5LTR	+
Pongo_abelii_CM009264.2	73872537	73872836	HERVHF_gag	+
Pongo_abelii_CM009264.2	73872860	73873693	HERVHF_pro	+
Pongo_abelii_CM009264.2	73873151	73875071	HERVHF_pol	+
Pongo_abelii_CM009264.2	73875623	73875956	HERVHF_3LTR	+
Pongo_abelii_CM009264.2	74122165	74122614	HERVHF_5LTR	-
Pongo_abelii_CM009264.2	74123062	74124963	HERVHF_pol	-
Pongo_abelii_CM009264.2	74125055	74125378	HERVHF_pro	-
Pongo_abelii_CM009264.2	74127528	74127976	HERVHF_3LTR	-
Pongo_abelii_CM009264.2	78648303	78648736	HERVHF_5LTR	+
Pongo_abelii_CM009264.2	78650461	78650760	HERVHF_gag	+
Pongo_abelii_CM009264.2	78651007	78651609	HERVHF_pro	+
Pongo_abelii_CM009264.2	78651172	78653155	HERVHF_pol	+
Pongo_abelii_CM009264.2	78653553	78653996	HERVHF_3LTR	+
Pongo_abelii_CM009264.2	79441068	79441410	HERVHF_5LTR	+
Pongo_abelii_CM009264.2	79442793	79443173	HERVHF_gag	+
Pongo_abelii_CM009264.2	79443285	79444064	HERVHF_pro	+
Pongo_abelii_CM009264.2	79443522	79445021	HERVHF_pol	+
Pongo_abelii_CM009264.2	79446370	79446954	HERVHF_env	+
Pongo_abelii_CM009264.2	79447167	79447521	HERVHF_3LTR	+
Pongo_abelii_CM009264.2	93734580	93735033	HERVHF_5LTR	-
Pongo_abelii_CM009264.2	93735445	93737567	HERVHF_pol	-
Pongo_abelii_CM009264.2	93736971	93737831	HERVHF_pro	-
Pongo_abelii_CM009264.2	93737859	93738182	HERVHF_gag	-
Pongo_abelii_CM009264.2	93740006	93740462	HERVHF_3LTR	-
Pongo_abelii_CM009264.2	93890568	93890939	HERVHF_5LTR	+
Pongo_abelii_CM009264.2	93892630	93893033	HERVHF_gag	+
Pongo_abelii_CM009264.2	93893126	93893713	HERVHF_pro	+
Pongo_abelii_CM009264.2	93893663	93895177	HERVHF_pol	+
Pongo_abelii_CM009264.2	93896219	93896590	HERVHF_3LTR	+
Pongo_abelii_CM009264.2	95972195	95972648	HERVHF_5LTR	+
Pongo_abelii_CM009264.2	95974700	95975713	HERVHF_pro	+
Pongo_abelii_CM009264.2	95975000	95977063	HERVHF_pol	+
Pongo_abelii_CM009264.2	95978086	95978536	HERVHF_3LTR	+

Pongo_abelii_CM009264.2	105266424	105266857	HERVHF_5LTR	-
Pongo_abelii_CM009264.2	105267257	105269197	HERVHF_pol	-
Pongo_abelii_CM009264.2	105269128	105269673	HERVHF_pro	-
Pongo_abelii_CM009264.2	105271831	105272262	HERVHF_3LTR	-
Pongo_abelii_CM009264.2	111215680	111216081	HERVHF_5LTR	-
Pongo_abelii_CM009264.2	111216517	111218978	HERVHF_pol	-
Pongo_abelii_CM009264.2	111219017	111219409	HERVHF_pro	-
Pongo_abelii_CM009264.2	111219442	111219741	HERVHF_gag	-
Pongo_abelii_CM009264.2	111221430	111221829	HERVHF_3LTR	-
Pongo_abelii_CM009264.2	111354527	111354926	HERVHF_5LTR	+
Pongo_abelii_CM009264.2	111356580	111356948	HERVHF_gag	+
Pongo_abelii_CM009264.2	111357283	111359223	HERVHF_pol	+
Pongo_abelii_CM009264.2	111359816	111360218	HERVHF_3LTR	+
Pongo_abelii_CM009264.2	111425407	111425820	HERVHF_5LTR	+
Pongo_abelii_CM009264.2	111428178	111430252	HERVHF_pol	+
Pongo_abelii_CM009264.2	111430691	111431115	HERVHF_3LTR	+
Pongo_abelii_CM009264.2	124887811	124888223	HERVHF_5LTR	+
Pongo_abelii_CM009264.2	124889933	124890253	HERVHF_gag	+
Pongo_abelii_CM009264.2	124890805	124892760	HERVHF_pol	+
Pongo_abelii_CM009264.2	124893169	124893582	HERVHF_3LTR	+
Pongo_abelii_CM009265.2	5077589	5077907	HERVHF_5LTR	-
Pongo_abelii_CM009265.2	5078432	5080844	HERVHF_pol	-
Pongo_abelii_CM009265.2	5080859	5081638	HERVHF_pro	-
Pongo_abelii_CM009265.2	5081297	5081737	HERVHF_gag	-
Pongo_abelii_CM009265.2	5083219	5083535	HERVHF_3LTR	-
Pongo_abelii_CM009265.2	22546483	22546834	HERVHF_5LTR	+
Pongo_abelii_CM009265.2	22549075	22549857	HERVHF_pro	+
Pongo_abelii_CM009265.2	22549534	22551244	HERVHF_pol	+
Pongo_abelii_CM009265.2	22551856	22552217	HERVHF_3LTR	+
Pongo_abelii_CM009265.2	57867850	57868262	HERVHF_5LTR	-
Pongo_abelii_CM009265.2	57868831	57870793	HERVHF_pol	-
Pongo_abelii_CM009265.2	57870254	57871084	HERVHF_pro	-
Pongo_abelii_CM009265.2	57871108	57871407	HERVHF_gag	-
Pongo_abelii_CM009265.2	57873083	57873495	HERVHF_3LTR	-
Pongo_abelii_CM009265.2	59657004	59657392	HERVHF_5LTR	-
Pongo_abelii_CM009265.2	59658039	59659935	HERVHF_pol	-
Pongo_abelii_CM009265.2	59659393	59660226	HERVHF_pro	-
Pongo_abelii_CM009265.2	59662389	59662779	HERVHF_3LTR	-
Pongo_abelii_CM009265.2	60942563	60942896	HERVIPADP_5LTR	+
Pongo_abelii_CM009265.2	60944420	60944743	HERVIPADP_gag	+
Pongo_abelii_CM009265.2	60945870	60948814	HERVIPADP_pol	+
Pongo_abelii_CM009265.2	60950949	60951288	HERVIPADP_3LTR	+
Pongo_abelii_CM009265.2	64970361	64970813	HERVHF_5LTR	+
Pongo_abelii_CM009265.2	64972431	64972754	HERVHF_gag	+

Pongo_abelii_CM009265.2	64972810	64973586	HERVHF_pro	+
Pongo_abelii_CM009265.2	64972948	64975126	HERVHF_pol	+
Pongo_abelii_CM009265.2	64976136	64976588	HERVHF_3LTR	+
Pongo_abelii_CM009265.2	68466585	68467038	HERVHF_5LTR	+
Pongo_abelii_CM009265.2	68468654	68471028	HERVHF_gag	+
Pongo_abelii_CM009265.2	68471080	68471844	HERVHF_pro	+
Pongo_abelii_CM009265.2	68471323	68473254	HERVHF_pol	+
Pongo_abelii_CM009265.2	68473859	68474306	HERVHF_3LTR	+
Pongo_abelii_CM009265.2	69536064	69536489	HERVHF_5LTR	-
Pongo_abelii_CM009265.2	69536908	69539271	HERVHF_pol	-
Pongo_abelii_CM009265.2	69538669	69539523	HERVHF_pro	-
Pongo_abelii_CM009265.2	69539527	69539850	HERVHF_gag	-
Pongo_abelii_CM009265.2	69541419	69541842	HERVHF_3LTR	-
Pongo_abelii_CM009265.2	71126290	71126704	HERVHF_5LTR	+
Pongo_abelii_CM009265.2	71128035	71128403	HERVHF_gag	+
Pongo_abelii_CM009265.2	71128693	71129490	HERVHF_pro	+
Pongo_abelii_CM009265.2	71129086	71131244	HERVHF_pol	+
Pongo_abelii_CM009265.2	71131694	71132108	HERVHF_3LTR	+
Pongo_abelii_CM009265.2	95252236	95252688	HERVHF_5LTR	+
Pongo_abelii_CM009265.2	95255177	95257110	HERVHF_pol	+
Pongo_abelii_CM009265.2	95257564	95258013	HERVHF_3LTR	+
Pongo_abelii_CM009265.2	95561153	95561617	HERVK_5LTR	+
Pongo_abelii_CM009265.2	95563108	95564049	HERVK_pro	+
Pongo_abelii_CM009265.2	95563908	95565485	HERVK_pol	+
Pongo_abelii_CM009265.2	95566765	95567235	HERVK_3LTR	+
Pongo_abelii_CM009265.2	102346464	102346874	HERVHF_5LTR	+
Pongo_abelii_CM009265.2	102348523	102348846	HERVHF_gag	+
Pongo_abelii_CM009265.2	102348972	102349754	HERVHF_pro	+
Pongo_abelii_CM009265.2	102349167	102351170	HERVHF_pol	+
Pongo_abelii_CM009265.2	102353314	102353727	HERVHF_3LTR	+
Pongo_abelii_CM009265.2	106108830	106109193	HERVHF_5LTR	+
Pongo_abelii_CM009265.2	106111247	106111837	HERVHF_gag	+
Pongo_abelii_CM009265.2	106112011	106112673	HERVHF_pro	+
Pongo_abelii_CM009265.2	106112092	106114041	HERVHF_pol	+
Pongo_abelii_CM009265.2	106114612	106114987	HERVHF_3LTR	+
Pongo_abelii_CM009265.2	125489170	125489531	HERVHF_5LTR	-
Pongo_abelii_CM009265.2	125490212	125492152	HERVHF_pol	-
Pongo_abelii_CM009265.2	125492475	125492855	HERVHF_gag	-
Pongo_abelii_CM009265.2	125494560	125494923	HERVHF_3LTR	-
Pongo_abelii_CM009265.2	126226306	126226666	HERVIPADP_5LTR	+
Pongo_abelii_CM009265.2	126229093	126232393	HERVIPADP_pol	+
Pongo_abelii_CM009265.2	126233474	126233851	HERVIPADP_env	+
Pongo_abelii_CM009265.2	126234589	126234946	HERVIPADP_3LTR	+
Pongo_abelii_CM009265.2	131583781	131584217	HERVHF_5LTR	-

Pongo_abelii_CM009265.2	131584445	131586841	HERVHF_pol	-
Pongo_abelii_CM009265.2	131586227	131587006	HERVHF_pro	-
Pongo_abelii_CM009265.2	131587129	131587452	HERVHF_gag	-
Pongo_abelii_CM009265.2	131589218	131589652	HERVHF_3LTR	-
Pongo_abelii_CM009265.2	134640111	134640468	HERVHF_5LTR	+
Pongo_abelii_CM009265.2	134642158	134642481	HERVHF_gag	+
Pongo_abelii_CM009265.2	134642912	134645063	HERVHF_pol	+
Pongo_abelii_CM009265.2	134645269	134645600	HERVHF_3LTR	+
Pongo_abelii_CM009265.2	149922444	149922746	HERVHF_5LTR	+
Pongo_abelii_CM009265.2	149924512	149924964	HERVHF_gag	+
Pongo_abelii_CM009265.2	149925311	149926060	HERVHF_pro	+
Pongo_abelii_CM009265.2	149925518	149927629	HERVHF_pol	+
Pongo_abelii_CM009265.2	149928157	149928470	HERVHF_3LTR	+
Pongo_abelii_CM009265.2	150568454	150568879	HERVHF_5LTR	-
Pongo_abelii_CM009265.2	150570132	150571960	HERVHF_pol	-
Pongo_abelii_CM009265.2	150571397	150572173	HERVHF_pro	-
Pongo_abelii_CM009265.2	150572226	150572546	HERVHF_gag	-
Pongo_abelii_CM009265.2	150574222	150574637	HERVHF_3LTR	-
Pongo_abelii_CM009265.2	159126733	159127143	HERVHF_5LTR	-
Pongo_abelii_CM009265.2	159127716	159129540	HERVHF_pol	-
Pongo_abelii_CM009265.2	159129961	159130290	HERVHF_gag	-
Pongo_abelii_CM009265.2	159132003	159132416	HERVHF_3LTR	-
Pongo_abelii_CM009265.2	161366408	161366772	HERVHF_5LTR	+
Pongo_abelii_CM009265.2	161368978	161369829	HERVHF_pro	+
Pongo_abelii_CM009265.2	161369266	161372078	HERVHF_pol	+
Pongo_abelii_CM009265.2	161372391	161372767	HERVHF_3LTR	+
Pongo_abelii_CM009265.2	164031233	164031568	HERVHF_5LTR	-
Pongo_abelii_CM009265.2	164031833	164034197	HERVHF_pol	-
Pongo_abelii_CM009265.2	164034598	164034909	HERVHF_gag	-
Pongo_abelii_CM009265.2	164036627	164036967	HERVHF_3LTR	-
Pongo_abelii_CM009265.2	164965549	164965952	HERVHF_5LTR	+
Pongo_abelii_CM009265.2	164967522	164967842	HERVHF_gag	+
Pongo_abelii_CM009265.2	164967894	164968658	HERVHF_pro	+
Pongo_abelii_CM009265.2	164968137	164970462	HERVHF_pol	+
Pongo_abelii_CM009265.2	164970870	164971273	HERVHF_3LTR	+
Pongo_abelii_CM009265.2	168268014	168268452	HERVHF_5LTR	-
Pongo_abelii_CM009265.2	168268785	168269738	HERVHF_env	-
Pongo_abelii_CM009265.2	168270546	168272524	HERVHF_pol	-
Pongo_abelii_CM009265.2	168272950	168273390	HERVHF_gag	-
Pongo_abelii_CM009265.2	168275169	168275606	HERVHF_3LTR	-
Pongo_abelii_CM009265.2	170758375	170758792	HERVHF_5LTR	-
Pongo_abelii_CM009265.2	170759462	170761785	HERVHF_pol	-
Pongo_abelii_CM009265.2	170761543	170762296	HERVHF_pro	-
Pongo_abelii_CM009265.2	170762401	170762709	HERVHF_gag	-

Pongo_abelii_CM009265.2	170764327	170764749	HERVHF_3LTR	-
Pongo_abelii_CM009265.2	177183147	177183557	HERVHF_5LTR	+
Pongo_abelii_CM009265.2	177184971	177185369	HERVHF_gag	+
Pongo_abelii_CM009265.2	177185462	177185890	HERVHF_pro	+
Pongo_abelii_CM009265.2	177185614	177187563	HERVHF_pol	+
Pongo_abelii_CM009265.2	177188159	177188571	HERVHF_3LTR	+
Pongo_abelii_CM009265.2	180408131	180408489	HUERSP_5LTR	-
Pongo_abelii_CM009265.2	180414658	180415380	HUERSP_pol	-
Pongo_abelii_CM009265.2	180418703	180419062	HUERSP_3LTR	-
Pongo_abelii_CM009265.2	184338932	184339333	HERVHF_5LTR	+
Pongo_abelii_CM009265.2	184340913	184341479	HERVHF_gag	+
Pongo_abelii_CM009265.2	184341630	184342148	HERVHF_pro	+
Pongo_abelii_CM009265.2	184342052	184343602	HERVHF_pol	+
Pongo_abelii_CM009265.2	184344431	184344839	HERVHF_3LTR	+
Pongo_abelii_CM009265.2	187825732	187826143	HERVHF_5LTR	+
Pongo_abelii_CM009265.2	187827770	187828090	HERVHF_gag	+
Pongo_abelii_CM009265.2	187828437	187828853	HERVHF_pro	+
Pongo_abelii_CM009265.2	187828670	187830608	HERVHF_pol	+
Pongo_abelii_CM009265.2	187831004	187831414	HERVHF_3LTR	+
Pongo_abelii_CM009265.2	189585797	189586109	HERVHF_5LTR	-
Pongo_abelii_CM009265.2	189586796	189588739	HERVHF_pol	-
Pongo_abelii_CM009265.2	189589146	189589571	HERVHF_gag	-
Pongo_abelii_CM009265.2	189591152	189591456	HERVHF_3LTR	-
Pongo_abelii_CM009265.2	189762877	189763222	HERVHF_5LTR	-
Pongo_abelii_CM009265.2	189763701	189765779	HERVHF_pol	-
Pongo_abelii_CM009265.2	189765237	189766016	HERVHF_pro	-
Pongo_abelii_CM009265.2	189768502	189768859	HERVHF_3LTR	-
Pongo_abelii_CM009265.2	190675039	190675454	HERVHF_5LTR	+
Pongo_abelii_CM009265.2	190678073	190679859	HERVHF_pol	+
Pongo_abelii_CM009265.2	190680432	190680844	HERVHF_3LTR	+
Pongo_abelii_CM009266.2	7215224	7215656	HERVHF_5LTR	-
Pongo_abelii_CM009266.2	7215677	7216022	HERVHF_env	-
Pongo_abelii_CM009266.2	7216273	7218291	HERVHF_pol	-
Pongo_abelii_CM009266.2	7217656	7218483	HERVHF_pro	-
Pongo_abelii_CM009266.2	7218600	7218926	HERVHF_gag	-
Pongo_abelii_CM009266.2	7221194	7221630	HERVHF_3LTR	-
Pongo_abelii_CM009266.2	10736407	10736811	HERVHF_5LTR	-
Pongo_abelii_CM009266.2	10737401	10739416	HERVHF_pol	-
Pongo_abelii_CM009266.2	10741702	10742112	HERVHF_3LTR	-
Pongo_abelii_CM009266.2	12735502	12735843	HERVHF_5LTR	-
Pongo_abelii_CM009266.2	12736569	12738462	HERVHF_pol	-
Pongo_abelii_CM009266.2	12737950	12738501	HERVHF_pro	-
Pongo_abelii_CM009266.2	12738777	12739076	HERVHF_gag	-
Pongo_abelii_CM009266.2	12740975	12741312	HERVHF_3LTR	-

Pongo_abelii_CM009266.2	16134562	16135016	HERVHF_5LTR	+
Pongo_abelii_CM009266.2	16136677	16136997	HERVHF_gag	+
Pongo_abelii_CM009266.2	16137420	16139350	HERVHF_pol	+
Pongo_abelii_CM009266.2	16140334	16140788	HERVHF_3LTR	+
Pongo_abelii_CM009266.2	18089721	18090177	HERVHF_5LTR	+
Pongo_abelii_CM009266.2	18092032	18092355	HERVHF_gag	+
Pongo_abelii_CM009266.2	18092504	18093301	HERVHF_pro	+
Pongo_abelii_CM009266.2	18092738	18094863	HERVHF_pol	+
Pongo_abelii_CM009266.2	18095268	18095721	HERVHF_3LTR	+
Pongo_abelii_CM009266.2	26776833	26777284	HERVHF_5LTR	-
Pongo_abelii_CM009266.2	26777876	26779815	HERVHF_pol	-
Pongo_abelii_CM009266.2	26779255	26779914	HERVHF_pro	-
Pongo_abelii_CM009266.2	26780127	26780426	HERVHF_gag	-
Pongo_abelii_CM009266.2	26782315	26782768	HERVHF_3LTR	-
Pongo_abelii_CM009266.2	27318163	27318575	HERVHF_5LTR	+
Pongo_abelii_CM009266.2	27320165	27320521	HERVHF_gag	+
Pongo_abelii_CM009266.2	27320767	27321366	HERVHF_pro	+
Pongo_abelii_CM009266.2	27320959	27323242	HERVHF_pol	+
Pongo_abelii_CM009266.2	27323661	27324072	HERVHF_3LTR	+
Pongo_abelii_CM009266.2	69966562	69967015	HERVHF_5LTR	+
Pongo_abelii_CM009266.2	69969375	69970124	HERVHF_pro	+
Pongo_abelii_CM009266.2	69969585	69971519	HERVHF_pol	+
Pongo_abelii_CM009266.2	69972105	69972563	HERVHF_3LTR	+
Pongo_abelii_CM009266.2	84860878	84861341	HERVHF_5LTR	+
Pongo_abelii_CM009266.2	84862791	84863192	HERVHF_gag	+
Pongo_abelii_CM009266.2	84863551	84865803	HERVHF_pol	+
Pongo_abelii_CM009266.2	84866219	84866678	HERVHF_3LTR	+
Pongo_abelii_CM009266.2	88393712	88394117	HERVHF_5LTR	-
Pongo_abelii_CM009266.2	88394518	88396502	HERVHF_pol	-
Pongo_abelii_CM009266.2	88398953	88399357	HERVHF_3LTR	-
Pongo_abelii_CM009266.2	92853532	92853868	HERVHF_5LTR	-
Pongo_abelii_CM009266.2	92854823	92856895	HERVHF_pol	-
Pongo_abelii_CM009266.2	92856473	92857006	HERVHF_pro	-
Pongo_abelii_CM009266.2	92857323	92857646	HERVHF_gag	-
Pongo_abelii_CM009266.2	92859472	92859825	HERVHF_3LTR	-
Pongo_abelii_CM009266.2	107500687	107501087	HERVHF_5LTR	+
Pongo_abelii_CM009266.2	107502633	107503139	HERVHF_gag	+
Pongo_abelii_CM009266.2	107503566	107504147	HERVHF_pro	+
Pongo_abelii_CM009266.2	107503740	107505906	HERVHF_pol	+
Pongo_abelii_CM009266.2	107506136	107506539	HERVHF_3LTR	+
Pongo_abelii_CM009266.2	114896178	114896582	HERVHF_5LTR	-
Pongo_abelii_CM009266.2	114897157	114898968	HERVHF_pol	-
Pongo_abelii_CM009266.2	114898573	114899289	HERVHF_pro	-
Pongo_abelii_CM009266.2	114899436	114899956	HERVHF_gag	-

Pongo_abelii_CM009266.2	114901546	114901961	HERVHF_3LTR	-
Pongo_abelii_CM009266.2	116229016	116229328	HERVHF_5LTR	+
Pongo_abelii_CM009266.2	116231133	116231453	HERVHF_gag	+
Pongo_abelii_CM009266.2	116231883	116233681	HERVHF_pol	+
Pongo_abelii_CM009266.2	116234274	116234592	HERVHF_3LTR	+
Pongo_abelii_CM009266.2	126100401	126100804	HERVHF_5LTR	-
Pongo_abelii_CM009266.2	126101256	126103818	HERVHF_pol	-
Pongo_abelii_CM009266.2	126106157	126106561	HERVHF_3LTR	-
Pongo_abelii_CM009266.2	129368859	129369262	HERVHF_5LTR	+
Pongo_abelii_CM009266.2	129370689	129371000	HERVHF_gag	+
Pongo_abelii_CM009266.2	129371178	129371849	HERVHF_pro	+
Pongo_abelii_CM009266.2	129371250	129372807	HERVHF_pol	+
Pongo_abelii_CM009266.2	129374046	129374446	HERVHF_3LTR	+
Pongo_abelii_CM009266.2	130771291	130771712	HERVHF_5LTR	+
Pongo_abelii_CM009266.2	130773798	130774526	HERVHF_pro	+
Pongo_abelii_CM009266.2	130774146	130775653	HERVHF_pol	+
Pongo_abelii_CM009266.2	130776280	130776715	HERVHF_3LTR	+
Pongo_abelii_CM009266.2	130784551	130784985	HERVHF_5LTR	-
Pongo_abelii_CM009266.2	130785422	130787587	HERVHF_pol	-
Pongo_abelii_CM009266.2	130789829	130790269	HERVHF_3LTR	-
Pongo_abelii_CM009266.2	132357455	132357901	HERVHF_5LTR	-
Pongo_abelii_CM009266.2	132358295	132360154	HERVHF_pol	-
Pongo_abelii_CM009266.2	132362925	132363361	HERVHF_3LTR	-
Pongo_abelii_CM009266.2	132734739	132735157	HERVHF_5LTR	+
Pongo_abelii_CM009266.2	132736894	132737217	HERVHF_gag	+
Pongo_abelii_CM009266.2	132737305	132738054	HERVHF_pro	+
Pongo_abelii_CM009266.2	132737512	132739982	HERVHF_pol	+
Pongo_abelii_CM009266.2	132740552	132740964	HERVHF_3LTR	+
Pongo_abelii_CM009266.2	133454189	133454601	HERVHF_5LTR	-
Pongo_abelii_CM009266.2	133454996	133457125	HERVHF_pol	-
Pongo_abelii_CM009266.2	133456565	133457188	HERVHF_pro	-
Pongo_abelii_CM009266.2	133457454	133457828	HERVHF_gag	-
Pongo_abelii_CM009266.2	133459516	133459927	HERVHF_3LTR	-
Pongo_abelii_CM009266.2	133967372	133967807	HERVHF_5LTR	-
Pongo_abelii_CM009266.2	133968216	133970191	HERVHF_pol	-
Pongo_abelii_CM009266.2	133969823	133970428	HERVHF_pro	-
Pongo_abelii_CM009266.2	133972637	133973071	HERVHF_3LTR	-
Pongo_abelii_CM009266.2	133993124	133993424	HERVHF_5LTR	+
Pongo_abelii_CM009266.2	133995496	133995873	HERVHF_pro	+
Pongo_abelii_CM009266.2	133995893	133997890	HERVHF_pol	+
Pongo_abelii_CM009266.2	133998349	133998656	HERVHF_3LTR	+
Pongo_abelii_CM009266.2	137846817	137847220	HERVHF_5LTR	+
Pongo_abelii_CM009266.2	137848908	137849291	HERVHF_gag	+
Pongo_abelii_CM009266.2	137849327	137850157	HERVHF_pro	+

Pongo_abelii_CM009266.2	137849615	137851501	HERVHF_pol	+
Pongo_abelii_CM009266.2	137852125	137852528	HERVHF_3LTR	+
Pongo_abelii_CM009266.2	140584925	140585357	HERVHF_5LTR	-
Pongo_abelii_CM009266.2	140585767	140587940	HERVHF_pol	-
Pongo_abelii_CM009266.2	140587323	140588087	HERVHF_pro	-
Pongo_abelii_CM009266.2	140590268	140590699	HERVHF_3LTR	-
Pongo_abelii_CM009266.2	151598011	151598442	HERVHF_5LTR	+
Pongo_abelii_CM009266.2	151600632	151601396	HERVHF_pro	+
Pongo_abelii_CM009266.2	151600818	151602802	HERVHF_pol	+
Pongo_abelii_CM009266.2	151603017	151604704	HERVHF_env	+
Pongo_abelii_CM009266.2	151604865	151605299	HERVHF_3LTR	+
Pongo_abelii_CM009266.2	161870226	161870678	HERVHF_5LTR	+
Pongo_abelii_CM009266.2	161872495	161872818	HERVHF_gag	+
Pongo_abelii_CM009266.2	161873247	161875122	HERVHF_pol	+
Pongo_abelii_CM009266.2	161875632	161876085	HERVHF_3LTR	+
Pongo_abelii_CM009266.2	162055672	162056093	HERVHF_5LTR	-
Pongo_abelii_CM009266.2	162056260	162056598	HERVHF_env	-
Pongo_abelii_CM009266.2	162057435	162059453	HERVHF_pol	-
Pongo_abelii_CM009266.2	162059607	162060035	HERVHF_3LTR	-
Pongo_abelii_CM009266.2	165564750	165565154	HERVHF_5LTR	+
Pongo_abelii_CM009266.2	165566645	165567001	HERVHF_gag	+
Pongo_abelii_CM009266.2	165567294	165569619	HERVHF_pol	+
Pongo_abelii_CM009266.2	165570025	165570425	HERVHF_3LTR	+
Pongo_abelii_CM009266.2	165770442	165770892	HERVHF_5LTR	-
Pongo_abelii_CM009266.2	165771426	165773312	HERVHF_pol	-
Pongo_abelii_CM009266.2	165773725	165774138	HERVHF_gag	-
Pongo_abelii_CM009266.2	165776232	165776681	HERVHF_3LTR	-
Pongo_abelii_CM009266.2	166936186	166936585	HERVHF_5LTR	+
Pongo_abelii_CM009266.2	166938032	166938535	HERVHF_gag	+
Pongo_abelii_CM009266.2	166939017	166941065	HERVHF_pol	+
Pongo_abelii_CM009266.2	166941496	166941897	HERVHF_3LTR	+
Pongo_abelii_CM009266.2	166982199	166982668	HERVHF_5LTR	+
Pongo_abelii_CM009266.2	166984260	166984640	HERVHF_gag	+
Pongo_abelii_CM009266.2	166984921	166985505	HERVHF_pro	+
Pongo_abelii_CM009266.2	166985098	166987095	HERVHF_pol	+
Pongo_abelii_CM009266.2	166987489	166987959	HERVHF_3LTR	+
Pongo_abelii_CM009266.2	169289042	169289456	HERVHF_5LTR	+
Pongo_abelii_CM009266.2	169291741	169293757	HERVHF_pol	+
Pongo_abelii_CM009266.2	169294266	169294683	HERVHF_3LTR	+
Pongo_abelii_CM009266.2	170334983	170335462	HERVHF_5LTR	+
Pongo_abelii_CM009266.2	170337333	170337656	HERVHF_gag	+
Pongo_abelii_CM009266.2	170337711	170338388	HERVHF_pro	+
Pongo_abelii_CM009266.2	170338258	170339859	HERVHF_pol	+
Pongo_abelii_CM009266.2	170340466	170340935	HERVHF_3LTR	+

Pongo_abelii_CM009266.2	170888032	170888490	HERVHF_5LTR	-
Pongo_abelii_CM009266.2	170888880	170891090	HERVHF_pol	-
Pongo_abelii_CM009266.2	170890452	170891069	HERVHF_pro	-
Pongo_abelii_CM009266.2	170893531	170893981	HERVHF_3LTR	-
Pongo_abelii_CM009266.2	171237974	171238363	HERVHF_5LTR	-
Pongo_abelii_CM009266.2	171238956	171240874	HERVHF_pol	-
Pongo_abelii_CM009266.2	171241177	171241503	HERVHF_gag	-
Pongo_abelii_CM009266.2	171243178	171243576	HERVHF_3LTR	-
Pongo_abelii_CM009266.2	172281866	172282186	HERVHF_5LTR	-
Pongo_abelii_CM009266.2	172283015	172285353	HERVHF_pol	-
Pongo_abelii_CM009266.2	172284940	172285524	HERVHF_pro	-
Pongo_abelii_CM009266.2	172287916	172288217	HERVHF_3LTR	-
Pongo_abelii_CM009266.2	174803879	174804330	HERVHF_5LTR	-
Pongo_abelii_CM009266.2	174804786	174806747	HERVHF_pol	-
Pongo_abelii_CM009266.2	174806592	174807155	HERVHF_pro	-
Pongo_abelii_CM009266.2	174807438	174807818	HERVHF_gag	-
Pongo_abelii_CM009266.2	174809366	174809819	HERVHF_3LTR	-
Pongo_abelii_CM009266.2	185436290	185436695	HERVHF_5LTR	-
Pongo_abelii_CM009266.2	185437100	185439407	HERVHF_pol	-
Pongo_abelii_CM009266.2	185438844	185439695	HERVHF_pro	-
Pongo_abelii_CM009266.2	185439740	185440138	HERVHF_gag	-
Pongo_abelii_CM009266.2	185441506	185441910	HERVHF_3LTR	-
Pongo_abelii_CM009266.2	188363554	188363883	HERVHF_5LTR	-
Pongo_abelii_CM009266.2	188364664	188366365	HERVHF_pol	-
Pongo_abelii_CM009266.2	188366048	188366890	HERVHF_pro	-
Pongo_abelii_CM009266.2	188366883	188367278	HERVHF_gag	-
Pongo_abelii_CM009266.2	188368902	188369217	HERVHF_3LTR	-
Pongo_abelii_CM009267.2	7223598	7223947	HERVHF_5LTR	+
Pongo_abelii_CM009267.2	7225021	7227132	HERVHF_pol	+
Pongo_abelii_CM009267.2	7227585	7227939	HERVHF_3LTR	+
Pongo_abelii_CM009267.2	9055806	9056479	HERVHF_5LTR	-
Pongo_abelii_CM009267.2	9062461	9064786	HERVHF_pol	-
Pongo_abelii_CM009267.2	9064151	9064918	HERVHF_pro	-
Pongo_abelii_CM009267.2	9065080	9065523	HERVHF_gag	-
Pongo_abelii_CM009267.2	9066604	9067268	HERVHF_3LTR	-
Pongo_abelii_CM009267.2	15032921	15033324	HERVHF_5LTR	-
Pongo_abelii_CM009267.2	15033720	15035900	HERVHF_pol	-
Pongo_abelii_CM009267.2	15035292	15036113	HERVHF_pro	-
Pongo_abelii_CM009267.2	15037717	15038121	HERVHF_3LTR	-
Pongo_abelii_CM009267.2	72614960	72615390	HERVHF_5LTR	+
Pongo_abelii_CM009267.2	72623610	72624440	HERVHF_pro	+
Pongo_abelii_CM009267.2	72623802	72625943	HERVHF_pol	+
Pongo_abelii_CM009267.2	72626418	72626848	HERVHF_3LTR	+
Pongo_abelii_CM009267.2	78332302	78332722	HERVHF_5LTR	+

Pongo_abelii_CM009267.2	78334365	78334736	HERVHF_gag	+
Pongo_abelii_CM009267.2	78335208	78337240	HERVHF_pol	+
Pongo_abelii_CM009267.2	78337829	78338254	HERVHF_3LTR	+
Pongo_abelii_CM009267.2	100802925	100803308	HERVHF_5LTR	+
Pongo_abelii_CM009267.2	100804912	100805307	HERVHF_gag	+
Pongo_abelii_CM009267.2	100805365	100806123	HERVHF_pro	+
Pongo_abelii_CM009267.2	100805506	100807735	HERVHF_pol	+
Pongo_abelii_CM009267.2	100808153	100808523	HERVHF_3LTR	+
Pongo_abelii_CM009267.2	101667561	101667904	HUERSP_5LTR	-
Pongo_abelii_CM009267.2	101677144	101679204	HUERSP_pol	-
Pongo_abelii_CM009267.2	101679612	101680037	HUERSP_gag	-
Pongo_abelii_CM009267.2	101682213	101682568	HUERSP_3LTR	-
Pongo_abelii_CM009267.2	105399059	105399411	HERVHF_5LTR	-
Pongo_abelii_CM009267.2	105399797	105400949	HERVHF_pol	-
Pongo_abelii_CM009267.2	105400696	105401271	HERVHF_pro	-
Pongo_abelii_CM009267.2	105401301	105401684	HERVHF_gag	-
Pongo_abelii_CM009267.2	105403500	105403846	HERVHF_3LTR	-
Pongo_abelii_CM009267.2	110652445	110652956	HERVK_5LTR	+
Pongo_abelii_CM009267.2	110653694	110654635	HERVK_pro	+
Pongo_abelii_CM009267.2	110654530	110655369	HERVK_pol	+
Pongo_abelii_CM009267.2	110656642	110657142	HERVK_3LTR	+
Pongo_abelii_CM009267.2	114083675	114084111	HERVHF_5LTR	-
Pongo_abelii_CM009267.2	114084752	114086403	HERVHF_pol	-
Pongo_abelii_CM009267.2	114086005	114086604	HERVHF_pro	-
Pongo_abelii_CM009267.2	114086917	114087243	HERVHF_gag	-
Pongo_abelii_CM009267.2	114088873	114089305	HERVHF_3LTR	-
Pongo_abelii_CM009267.2	117782682	117783136	HERVHF_5LTR	-
Pongo_abelii_CM009267.2	117783973	117785626	HERVHF_pol	-
Pongo_abelii_CM009267.2	117785138	117785962	HERVHF_pro	-
Pongo_abelii_CM009267.2	117785969	117786289	HERVHF_gag	-
Pongo_abelii_CM009267.2	117788261	117788714	HERVHF_3LTR	-
Pongo_abelii_CM009267.2	117824580	117825034	HERVHF_5LTR	-
Pongo_abelii_CM009267.2	117825810	117827667	HERVHF_pol	-
Pongo_abelii_CM009267.2	117827008	117827859	HERVHF_pro	-
Pongo_abelii_CM009267.2	117827869	117828186	HERVHF_gag	-
Pongo_abelii_CM009267.2	117830173	117830626	HERVHF_3LTR	-
Pongo_abelii_CM009267.2	145205578	145206008	HERVHF_5LTR	-
Pongo_abelii_CM009267.2	145206638	145208527	HERVHF_pol	-
Pongo_abelii_CM009267.2	145207964	145208818	HERVHF_pro	-
Pongo_abelii_CM009267.2	145210844	145211274	HERVHF_3LTR	-
Pongo_abelii_CM009267.2	158869735	158870656	HERVHF_5LTR	+
Pongo_abelii_CM009267.2	158875584	158876423	HERVHF_pro	+
Pongo_abelii_CM009267.2	158875794	158878173	HERVHF_pol	+
Pongo_abelii_CM009267.2	158878517	158879448	HERVHF_3LTR	+

Pongo_abelii_CM009267.2	159908796	159909207	HERVHF_5LTR	-
Pongo_abelii_CM009267.2	159909804	159911741	HERVHF_pol	-
Pongo_abelii_CM009267.2	159911220	159911978	HERVHF_pro	-
Pongo_abelii_CM009267.2	159912063	159912560	HERVHF_gag	-
Pongo_abelii_CM009267.2	159914126	159914529	HERVHF_3LTR	-
Pongo_abelii_CM009267.2	164445998	164446410	HERVHF_5LTR	-
Pongo_abelii_CM009267.2	164446420	164446829	HERVHF_env	-
Pongo_abelii_CM009267.2	164447031	164447884	HERVHF_pol	-
Pongo_abelii_CM009267.2	164448337	164448660	HERVHF_gag	-
Pongo_abelii_CM009267.2	164450237	164450646	HERVHF_3LTR	-
Pongo_abelii_CM009268.2	5878788	5879239	HERVHF_5LTR	+
Pongo_abelii_CM009268.2	5881663	5882232	HERVHF_pro	+
Pongo_abelii_CM009268.2	5882225	5883821	HERVHF_pol	+
Pongo_abelii_CM009268.2	5884412	5884865	HERVHF_3LTR	+
Pongo_abelii_CM009268.2	12525764	12526170	HERVHF_5LTR	+
Pongo_abelii_CM009268.2	12527567	12527914	HERVHF_gag	+
Pongo_abelii_CM009268.2	12528164	12528706	HERVHF_pro	+
Pongo_abelii_CM009268.2	12528586	12530531	HERVHF_pol	+
Pongo_abelii_CM009268.2	12530976	12531376	HERVHF_3LTR	+
Pongo_abelii_CM009268.2	16768448	16768850	HERVHF_5LTR	+
Pongo_abelii_CM009268.2	16771024	16771740	HERVHF_pro	+
Pongo_abelii_CM009268.2	16771105	16773198	HERVHF_pol	+
Pongo_abelii_CM009268.2	16773732	16774134	HERVHF_3LTR	+
Pongo_abelii_CM009268.2	31453916	31454291	HERVIPADP_5LTR	-
Pongo_abelii_CM009268.2	31455521	31455865	HERVIPADP_env	-
Pongo_abelii_CM009268.2	31458701	31460242	HERVIPADP_pol	-
Pongo_abelii_CM009268.2	31460574	31462913	HERVIPADP_gag	-
Pongo_abelii_CM009268.2	31464449	31464828	HERVIPADP_3LTR	-
Pongo_abelii_CM009268.2	52421278	52421725	HERVHF_5LTR	+
Pongo_abelii_CM009268.2	52423595	52423930	HERVHF_pro	+
Pongo_abelii_CM009268.2	52424031	52426206	HERVHF_pol	+
Pongo_abelii_CM009268.2	52426613	52427066	HERVHF_3LTR	+
Pongo_abelii_CM009268.2	58809699	58810143	HERVHF_5LTR	+
Pongo_abelii_CM009268.2	58811771	58812094	HERVHF_gag	+
Pongo_abelii_CM009268.2	58812101	58812910	HERVHF_pro	+
Pongo_abelii_CM009268.2	58812431	58814422	HERVHF_pol	+
Pongo_abelii_CM009268.2	58815360	58815805	HERVHF_3LTR	+
Pongo_abelii_CM009268.2	65380141	65380529	HERVHF_5LTR	+
Pongo_abelii_CM009268.2	65381829	65382308	HERVHF_gag	+
Pongo_abelii_CM009268.2	65382361	65383119	HERVHF_pro	+
Pongo_abelii_CM009268.2	65382502	65384747	HERVHF_pol	+
Pongo_abelii_CM009268.2	65385294	65385692	HERVHF_3LTR	+
Pongo_abelii_CM009268.2	93530311	93530753	HERVHF_5LTR	+
Pongo_abelii_CM009268.2	93532540	93532863	HERVHF_gag	+

Pongo_abelii_CM009268.2	93533386	93535182	HERVHF_pol	+
Pongo_abelii_CM009268.2	93535787	93536239	HERVHF_3LTR	+
Pongo_abelii_CM009268.2	112584315	112584740	HERVHF_5LTR	-
Pongo_abelii_CM009268.2	112585303	112587081	HERVHF_pol	-
Pongo_abelii_CM009268.2	112586764	112587534	HERVHF_pro	-
Pongo_abelii_CM009268.2	112587619	112587990	HERVHF_gag	-
Pongo_abelii_CM009268.2	112589676	112590086	HERVHF_3LTR	-
Pongo_abelii_CM009268.2	121897573	121897887	HERVHF_5LTR	-
Pongo_abelii_CM009268.2	121898477	121900411	HERVHF_pol	-
Pongo_abelii_CM009268.2	121902793	121903100	HERVHF_3LTR	-
Pongo_abelii_CM009268.2	129868978	129869412	HERVHF_5LTR	+
Pongo_abelii_CM009268.2	129871029	129871355	HERVHF_gag	+
Pongo_abelii_CM009268.2	129871741	129873841	HERVHF_pol	+
Pongo_abelii_CM009268.2	129874250	129874681	HERVHF_3LTR	+
Pongo_abelii_CM009268.2	130124072	130124505	HERVHF_5LTR	+
Pongo_abelii_CM009268.2	130126232	130126540	HERVHF_gag	+
Pongo_abelii_CM009268.2	130126666	130127253	HERVHF_pro	+
Pongo_abelii_CM009268.2	130127124	130128998	HERVHF_pol	+
Pongo_abelii_CM009268.2	130129402	130129833	HERVHF_3LTR	+
Pongo_abelii_CM009268.2	130355375	130355829	HERVHF_5LTR	+
Pongo_abelii_CM009268.2	130357349	130357663	HERVHF_gag	+
Pongo_abelii_CM009268.2	130357439	130358590	HERVHF_pro	+
Pongo_abelii_CM009268.2	130358222	130360336	HERVHF_pol	+
Pongo_abelii_CM009268.2	130361126	130361580	HERVHF_3LTR	+
Pongo_abelii_CM009268.2	139999496	139999900	HERVHF_5LTR	+
Pongo_abelii_CM009268.2	140001622	140001942	HERVHF_gag	+
Pongo_abelii_CM009268.2	140002023	140002751	HERVHF_pro	+
Pongo_abelii_CM009268.2	140002146	140004717	HERVHF_pol	+
Pongo_abelii_CM009268.2	140005943	140006706	HERVHF_env	+
Pongo_abelii_CM009268.2	140006933	140007335	HERVHF_3LTR	+
Pongo_abelii_CM009268.2	143456405	143456849	HERVHF_5LTR	+
Pongo_abelii_CM009268.2	143459078	143459650	HERVHF_pro	+
Pongo_abelii_CM009268.2	143459243	143461000	HERVHF_pol	+
Pongo_abelii_CM009268.2	143461583	143462032	HERVHF_3LTR	+
Pongo_abelii_CM009268.2	143546012	143546456	HERVHF_5LTR	-
Pongo_abelii_CM009268.2	143546857	143548955	HERVHF_pol	-
Pongo_abelii_CM009268.2	143548416	143549243	HERVHF_pro	-
Pongo_abelii_CM009268.2	143549250	143549570	HERVHF_gag	-
Pongo_abelii_CM009268.2	143550885	143551344	HERVHF_3LTR	-
Pongo_abelii_CM009268.2	147199349	147199655	HERVHF_5LTR	+
Pongo_abelii_CM009268.2	147201419	147201751	HERVHF_gag	+
Pongo_abelii_CM009268.2	147201908	147202636	HERVHF_pro	+
Pongo_abelii_CM009268.2	147202256	147204107	HERVHF_pol	+
Pongo_abelii_CM009268.2	147204653	147204967	HERVHF_3LTR	+

Pongo_abelii_CM009268.2	152586931	152587357	HERVHF_5LTR	+
Pongo_abelii_CM009268.2	152588987	152589310	HERVHF_gag	+
Pongo_abelii_CM009268.2	152589314	152590126	HERVHF_pro	+
Pongo_abelii_CM009268.2	152589509	152591689	HERVHF_pol	+
Pongo_abelii_CM009268.2	152592127	152592548	HERVHF_3LTR	+
Pongo_abelii_CM009268.2	159002207	159002561	HERVHF_5LTR	-
Pongo_abelii_CM009268.2	159003014	159005114	HERVHF_pol	-
Pongo_abelii_CM009268.2	159005448	159005870	HERVHF_gag	-
Pongo_abelii_CM009268.2	159007532	159007891	HERVHF_3LTR	-
Pongo_abelii_CM009268.2	163203469	163203883	HERVHF_5LTR	-
Pongo_abelii_CM009268.2	163204659	163206326	HERVHF_pol	-
Pongo_abelii_CM009268.2	163206775	163207161	HERVHF_gag	-
Pongo_abelii_CM009268.2	163208935	163209367	HERVHF_3LTR	-
Pongo_abelii_CM009268.2	163522980	163523296	HERVHF_5LTR	-
Pongo_abelii_CM009268.2	163523871	163525972	HERVHF_pol	-
Pongo_abelii_CM009268.2	163525355	163526107	HERVHF_pro	-
Pongo_abelii_CM009268.2	163526164	163526493	HERVHF_gag	-
Pongo_abelii_CM009268.2	163528368	163528686	HERVHF_3LTR	-
Pongo_abelii_CM009269.2	22939485	22939934	HERVHF_5LTR	-
Pongo_abelii_CM009269.2	22940330	22942437	HERVHF_pol	-
Pongo_abelii_CM009269.2	22942763	22943086	HERVHF_gag	-
Pongo_abelii_CM009269.2	22944822	22945275	HERVHF_3LTR	-
Pongo_abelii_CM009269.2	41868789	41869195	HERVHF_5LTR	+
Pongo_abelii_CM009269.2	41870360	41870884	HERVHF_gag	+
Pongo_abelii_CM009269.2	41871336	41873546	HERVHF_pol	+
Pongo_abelii_CM009269.2	41873999	41874402	HERVHF_3LTR	+
Pongo_abelii_CM009269.2	49136885	49137324	HERVHF_5LTR	-
Pongo_abelii_CM009269.2	49137770	49139618	HERVHF_pol	-
Pongo_abelii_CM009269.2	49139545	49140075	HERVHF_pro	-
Pongo_abelii_CM009269.2	49142107	49142546	HERVHF_3LTR	-
Pongo_abelii_CM009269.2	60853813	60854253	HERVHF_5LTR	+
Pongo_abelii_CM009269.2	60856081	60856467	HERVHF_gag	+
Pongo_abelii_CM009269.2	60856733	60858862	HERVHF_pol	+
Pongo_abelii_CM009269.2	60859315	60859756	HERVHF_3LTR	+
Pongo_abelii_CM009269.2	71369924	71370347	HERVHF_5LTR	-
Pongo_abelii_CM009269.2	71370364	71370718	HERVHF_env	-
Pongo_abelii_CM009269.2	71372137	71372718	HERVHF_pol	-
Pongo_abelii_CM009269.2	71372341	71372892	HERVHF_pro	-
Pongo_abelii_CM009269.2	71373174	71373554	HERVHF_gag	-
Pongo_abelii_CM009269.2	71375211	71375620	HERVHF_3LTR	-
Pongo_abelii_CM009269.2	76349932	76350315	HUERSP_5LTR	+
Pongo_abelii_CM009269.2	76352534	76353232	HUERSP_gag	+
Pongo_abelii_CM009269.2	76353935	76356185	HUERSP_pol	+
Pongo_abelii_CM009269.2	76357734	76358117	HUERSP_3LTR	+

Pongo_abelii_CM009269.2	81495448	81495901	HERVHF_5LTR	-
Pongo_abelii_CM009269.2	81496473	81498278	HERVHF_pol	-
Pongo_abelii_CM009269.2	81497871	81498632	HERVHF_pro	-
Pongo_abelii_CM009269.2	81498706	81499032	HERVHF_gag	-
Pongo_abelii_CM009269.2	81500897	81501350	HERVHF_3LTR	-
Pongo_abelii_CM009269.2	91932807	91933269	HERVHF_5LTR	-
Pongo_abelii_CM009269.2	91933437	91933998	HERVHF_env	-
Pongo_abelii_CM009269.2	91934151	91936500	HERVHF_pol	-
Pongo_abelii_CM009269.2	91935949	91936734	HERVHF_pro	-
Pongo_abelii_CM009269.2	91936816	91937169	HERVHF_gag	-
Pongo_abelii_CM009269.2	91938265	91938726	HERVHF_3LTR	-
Pongo_abelii_CM009269.2	95095256	95095590	HERVHF_5LTR	-
Pongo_abelii_CM009269.2	95095957	95098502	HERVHF_pol	-
Pongo_abelii_CM009269.2	95097921	95098733	HERVHF_pro	-
Pongo_abelii_CM009269.2	95098815	95099186	HERVHF_gag	-
Pongo_abelii_CM009269.2	95100613	95100946	HERVHF_3LTR	-
Pongo_abelii_CM009269.2	104542453	104542862	HERVHF_5LTR	-
Pongo_abelii_CM009269.2	104543271	104545244	HERVHF_pol	-
Pongo_abelii_CM009269.2	104545784	104546083	HERVHF_gag	-
Pongo_abelii_CM009269.2	104547631	104548038	HERVHF_3LTR	-
Pongo_abelii_CM009269.2	105873096	105873478	HERVHF_5LTR	+
Pongo_abelii_CM009269.2	105874851	105875213	HERVHF_gag	+
Pongo_abelii_CM009269.2	105875333	105876121	HERVHF_pro	+
Pongo_abelii_CM009269.2	105875579	105877866	HERVHF_pol	+
Pongo_abelii_CM009269.2	105878307	105878677	HERVHF_3LTR	+
Pongo_abelii_CM009269.2	106653506	106653910	HERVHF_5LTR	-
Pongo_abelii_CM009269.2	106654926	106656985	HERVHF_pol	-
Pongo_abelii_CM009269.2	106656272	106657276	HERVHF_pro	-
Pongo_abelii_CM009269.2	106657280	106657609	HERVHF_gag	-
Pongo_abelii_CM009269.2	106659341	106659746	HERVHF_3LTR	-
Pongo_abelii_CM009270.2	678349	678761	HERVHF_5LTR	-
Pongo_abelii_CM009270.2	679161	681290	HERVHF_pol	-
Pongo_abelii_CM009270.2	681694	682128	HERVHF_gag	-
Pongo_abelii_CM009270.2	683551	683960	HERVHF_3LTR	-
Pongo_abelii_CM009270.2	9099254	9099670	HERVHF_5LTR	-
Pongo_abelii_CM009270.2	9100731	9102305	HERVHF_pol	-
Pongo_abelii_CM009270.2	9101901	9102731	HERVHF_pro	-
Pongo_abelii_CM009270.2	9102765	9103304	HERVHF_gag	-
Pongo_abelii_CM009270.2	9104886	9105318	HERVHF_3LTR	-
Pongo_abelii_CM009270.2	46592912	46593330	HERVHF_5LTR	+
Pongo_abelii_CM009270.2	46594702	46595052	HERVHF_gag	+
Pongo_abelii_CM009270.2	46595645	46597621	HERVHF_pol	+
Pongo_abelii_CM009270.2	46598590	46599021	HERVHF_env	+
Pongo_abelii_CM009270.2	46599143	46599550	HERVHF_3LTR	+

Pongo_abelii_CM009270.2	57769102	57769528	HERVHF_5LTR	-
Pongo_abelii_CM009270.2	57769804	57770121	HERVHF_env	-
Pongo_abelii_CM009270.2	57771076	57773246	HERVHF_pol	-
Pongo_abelii_CM009270.2	57772521	57773408	HERVHF_pro	-
Pongo_abelii_CM009270.2	57775315	57775747	HERVHF_3LTR	-
Pongo_abelii_CM009270.2	63427139	63427528	HERVHF_5LTR	+
Pongo_abelii_CM009270.2	63428754	63429677	HERVHF_gag	+
Pongo_abelii_CM009270.2	63430144	63432441	HERVHF_pol	+
Pongo_abelii_CM009270.2	63433224	63433775	HERVHF_env	+
Pongo_abelii_CM009270.2	63433979	63434364	HERVHF_3LTR	+
Pongo_abelii_CM009270.2	79743080	79743407	HERVHF_5LTR	-
Pongo_abelii_CM009270.2	79744377	79746570	HERVHF_pol	-
Pongo_abelii_CM009270.2	79746109	79746807	HERVHF_pro	-
Pongo_abelii_CM009270.2	79746860	79747189	HERVHF_gag	-
Pongo_abelii_CM009270.2	79748637	79748974	HERVHF_3LTR	-
Pongo_abelii_CM009270.2	127090804	127091190	HERVHF_5LTR	-
Pongo_abelii_CM009270.2	127091654	127093619	HERVHF_pol	-
Pongo_abelii_CM009270.2	127093080	127093907	HERVHF_pro	-
Pongo_abelii_CM009270.2	127094006	127094323	HERVHF_gag	-
Pongo_abelii_CM009270.2	127095947	127096331	HERVHF_3LTR	-
Pongo_abelii_CM009270.2	127485811	127486172	HERVHF_5LTR	+
Pongo_abelii_CM009270.2	127486759	127487154	HERVHF_gag	+
Pongo_abelii_CM009270.2	127487629	127489819	HERVHF_pol	+
Pongo_abelii_CM009270.2	127496471	127496842	HERVHF_3LTR	+
Pongo_abelii_CM009270.2	136504079	136504487	HERVHF_5LTR	-
Pongo_abelii_CM009270.2	136504943	136507308	HERVHF_pol	-
Pongo_abelii_CM009270.2	136506766	136507569	HERVHF_pro	-
Pongo_abelii_CM009270.2	136507603	136508040	HERVHF_gag	-
Pongo_abelii_CM009270.2	136509612	136510002	HERVHF_3LTR	-
Pongo_abelii_CM009271.2	17952713	17953116	HERVHF_5LTR	+
Pongo_abelii_CM009271.2	17955263	17957646	HERVHF_pol	+
Pongo_abelii_CM009271.2	17955263	17955814	HERVHF_pro	+
Pongo_abelii_CM009271.2	17958053	17958458	HERVHF_3LTR	+
Pongo_abelii_CM009271.2	48306384	48306705	HERVHF_5LTR	-
Pongo_abelii_CM009271.2	48306938	48307750	HERVHF_env	-
Pongo_abelii_CM009271.2	48308612	48309760	HERVHF_pol	-
Pongo_abelii_CM009271.2	48309353	48309883	HERVHF_pro	-
Pongo_abelii_CM009271.2	48310181	48310504	HERVHF_gag	-
Pongo_abelii_CM009271.2	48312285	48312597	HERVHF_3LTR	-
Pongo_abelii_CM009271.2	49516710	49517163	HERVHF_5LTR	-
Pongo_abelii_CM009271.2	49517552	49519642	HERVHF_pol	-
Pongo_abelii_CM009271.2	49519540	49520130	HERVHF_pro	-
Pongo_abelii_CM009271.2	49521897	49522350	HERVHF_3LTR	-
Pongo_abelii_CM009271.2	57373562	57373885	HERVHF_5LTR	-

Pongo_abelii_CM009271.2	57374658	57376499	HERVHF_pol	-
Pongo_abelii_CM009271.2	57377024	57377507	HERVHF_gag	-
Pongo_abelii_CM009271.2	57378763	57379087	HERVHF_3LTR	-
Pongo_abelii_CM009271.2	63274812	63275275	HERVHF_5LTR	-
Pongo_abelii_CM009271.2	63276496	63277891	HERVHF_pol	-
Pongo_abelii_CM009271.2	63278138	63278485	HERVHF_gag	-
Pongo_abelii_CM009271.2	63280377	63280832	HERVHF_3LTR	-
Pongo_abelii_CM009271.2	67296955	67297463	HERVK_5LTR	-
Pongo_abelii_CM009271.2	67297546	67298273	HERVK_env	-
Pongo_abelii_CM009271.2	67298392	67299458	HERVK_pol	-
Pongo_abelii_CM009271.2	67299400	67300319	HERVK_pro	-
Pongo_abelii_CM009271.2	67301180	67301693	HERVK_3LTR	-
Pongo_abelii_CM009271.2	71924930	71925910	HERVHF_5LTR	-
Pongo_abelii_CM009271.2	71925918	71927208	HERVHF_pol	-
Pongo_abelii_CM009271.2	71927625	71928062	HERVHF_gag	-
Pongo_abelii_CM009271.2	71932996	71933994	HERVHF_3LTR	-
Pongo_abelii_CM009271.2	92434354	92434833	HERVK_5LTR	-
Pongo_abelii_CM009271.2	92434883	92435245	HERVK_env	-
Pongo_abelii_CM009271.2	92435938	92437060	HERVK_pol	-
Pongo_abelii_CM009271.2	92436955	92437893	HERVK_pro	-
Pongo_abelii_CM009271.2	92438758	92439230	HERVK_3LTR	-
Pongo_abelii_CM009271.2	100985097	100985548	HERVHF_5LTR	-
Pongo_abelii_CM009271.2	100986002	100988082	HERVHF_pol	-
Pongo_abelii_CM009271.2	100987540	100988370	HERVHF_pro	-
Pongo_abelii_CM009271.2	100988377	100988697	HERVHF_gag	-
Pongo_abelii_CM009271.2	100990741	100991193	HERVHF_3LTR	-
Pongo_abelii_CM009272.2	6492718	6493171	HERVHF_5LTR	-
Pongo_abelii_CM009272.2	6493570	6495553	HERVHF_pol	-
Pongo_abelii_CM009272.2	6496024	6496515	HERVHF_gag	-
Pongo_abelii_CM009272.2	6497993	6498450	HERVHF_3LTR	-
Pongo_abelii_CM009272.2	20245221	20245630	HERVHF_5LTR	-
Pongo_abelii_CM009272.2	20246031	20248014	HERVHF_pol	-
Pongo_abelii_CM009272.2	20247607	20248191	HERVHF_pro	-
Pongo_abelii_CM009272.2	20250548	20250954	HERVHF_3LTR	-
Pongo_abelii_CM009272.2	85259613	85260044	HERVHF_5LTR	+
Pongo_abelii_CM009272.2	85261765	85262088	HERVHF_gag	+
Pongo_abelii_CM009272.2	85262205	85262987	HERVHF_pro	+
Pongo_abelii_CM009272.2	85262370	85264569	HERVHF_pol	+
Pongo_abelii_CM009272.2	85264979	85265410	HERVHF_3LTR	+
Pongo_abelii_CM009272.2	87427659	87428051	HERVHF_5LTR	+
Pongo_abelii_CM009272.2	87429568	87430302	HERVHF_gag	+
Pongo_abelii_CM009272.2	87430198	87431085	HERVHF_pro	+
Pongo_abelii_CM009272.2	87430827	87432257	HERVHF_pol	+
Pongo_abelii_CM009272.2	87432933	87433298	HERVHF_env	+

Pongo_abelii_CM009272.2	87433430	87433831	HERVHF_3LTR	+
Pongo_abelii_CM009272.2	93320764	93321199	HERVHF_5LTR	+
Pongo_abelii_CM009272.2	93323988	93325109	HERVHF_pro	+
Pongo_abelii_CM009272.2	93324573	93326705	HERVHF_pol	+
Pongo_abelii_CM009272.2	93327102	93327533	HERVHF_3LTR	+
Pongo_abelii_CM009272.2	103239701	103240154	HERVHF_5LTR	-
Pongo_abelii_CM009272.2	103240731	103242516	HERVHF_pol	-
Pongo_abelii_CM009272.2	103242148	103242927	HERVHF_pro	-
Pongo_abelii_CM009272.2	103242968	103243489	HERVHF_gag	-
Pongo_abelii_CM009272.2	103245063	103245514	HERVHF_3LTR	-
Pongo_abelii_CM009272.2	104383652	104384097	HERVHF_5LTR	-
Pongo_abelii_CM009272.2	104384340	104385141	HERVHF_env	-
Pongo_abelii_CM009272.2	104384764	104387575	HERVHF_pol	-
Pongo_abelii_CM009272.2	104387790	104388200	HERVHF_gag	-
Pongo_abelii_CM009272.2	104389553	104389999	HERVHF_3LTR	-
Pongo_abelii_CM009273.2	4287257	4287673	HERVHF_5LTR	+
Pongo_abelii_CM009273.2	4290035	4292242	HERVHF_pol	+
Pongo_abelii_CM009273.2	4292939	4293356	HERVHF_3LTR	+
Pongo_abelii_CM009273.2	44038119	44038587	HERVIPADP_5LTR	+
Pongo_abelii_CM009273.2	44040092	44040502	HERVIPADP_gag	+
Pongo_abelii_CM009273.2	44040733	44042818	HERVIPADP_pol	+
Pongo_abelii_CM009273.2	44044585	44045444	HERVIPADP_env	+
Pongo_abelii_CM009273.2	44045737	44046192	HERVIPADP_3LTR	+
Pongo_abelii_CM009273.2	48408898	48409271	HERVHF_5LTR	-
Pongo_abelii_CM009273.2	48409506	48409868	HERVHF_env	-
Pongo_abelii_CM009273.2	48409957	48412595	HERVHF_pol	-
Pongo_abelii_CM009273.2	48412134	48412832	HERVHF_pro	-
Pongo_abelii_CM009273.2	48412890	48413213	HERVHF_gag	-
Pongo_abelii_CM009273.2	48414719	48415084	HERVHF_3LTR	-
Pongo_abelii_CM009273.2	61970785	61971146	HERVHF_5LTR	+
Pongo_abelii_CM009273.2	61973712	61975654	HERVHF_pol	+
Pongo_abelii_CM009273.2	61976102	61976465	HERVHF_3LTR	+
Pongo_abelii_CM009273.2	62100150	62100478	HERVHF_5LTR	-
Pongo_abelii_CM009273.2	62101021	62102813	HERVHF_pol	-
Pongo_abelii_CM009273.2	62102520	62103299	HERVHF_pro	-
Pongo_abelii_CM009273.2	62103362	62103877	HERVHF_gag	-
Pongo_abelii_CM009273.2	62105447	62105771	HERVHF_3LTR	-
Pongo_abelii_CM009273.2	63825876	63826303	HERVHF_5LTR	-
Pongo_abelii_CM009273.2	63826735	63828744	HERVHF_pol	-
Pongo_abelii_CM009273.2	63829081	63829491	HERVHF_gag	-
Pongo_abelii_CM009273.2	63831189	63831618	HERVHF_3LTR	-
Pongo_abelii_CM009273.2	74108403	74108853	HERVHF_5LTR	+
Pongo_abelii_CM009273.2	74110354	74110665	HERVHF_gag	+
Pongo_abelii_CM009273.2	74111120	74112319	HERVHF_pol	+

Pongo_abelii_CM009273.2	74113557	74114011	HERVHF_3LTR	+
Pongo_abelii_CM009273.2	75556682	75557137	HERVHF_5LTR	-
Pongo_abelii_CM009273.2	75558213	75560449	HERVHF_pol	-
Pongo_abelii_CM009273.2	75559907	75560620	HERVHF_pro	-
Pongo_abelii_CM009273.2	75560771	75561169	HERVHF_gag	-
Pongo_abelii_CM009273.2	75562003	75562459	HERVHF_3LTR	-
Pongo_abelii_CM009273.2	86930222	86930613	HERVHF_5LTR	+
Pongo_abelii_CM009273.2	86931516	86931827	HERVHF_gag	+
Pongo_abelii_CM009273.2	86931915	86932670	HERVHF_pro	+
Pongo_abelii_CM009273.2	86932326	86934301	HERVHF_pol	+
Pongo_abelii_CM009273.2	86934369	86934766	HERVHF_3LTR	+
Pongo_abelii_CM009273.2	111572361	111572777	HERVHF_5LTR	-
Pongo_abelii_CM009273.2	111573359	111575140	HERVHF_pol	-
Pongo_abelii_CM009273.2	111577594	111578022	HERVHF_3LTR	-
Pongo_abelii_CM009273.2	114487493	114487944	HERVHF_5LTR	-
Pongo_abelii_CM009273.2	114488993	114491034	HERVHF_pol	-
Pongo_abelii_CM009273.2	114490471	114491247	HERVHF_pro	-
Pongo_abelii_CM009273.2	114493381	114493835	HERVHF_3LTR	-
Pongo_abelii_CM009273.2	115670776	115671187	HERVHF_5LTR	+
Pongo_abelii_CM009273.2	115673064	115673387	HERVHF_gag	+
Pongo_abelii_CM009273.2	115673757	115675628	HERVHF_pol	+
Pongo_abelii_CM009273.2	115676204	115676613	HERVHF_3LTR	+
Pongo_abelii_CM009273.2	116729033	116729371	HERVHF_5LTR	-
Pongo_abelii_CM009273.2	116729903	116732193	HERVHF_pol	-
Pongo_abelii_CM009273.2	116731534	116732481	HERVHF_pro	-
Pongo_abelii_CM009273.2	116732488	116732829	HERVHF_gag	-
Pongo_abelii_CM009273.2	116734283	116734620	HERVHF_3LTR	-
Pongo_abelii_CM009274.2	1806073	1806413	HERVHF_5LTR	+
Pongo_abelii_CM009274.2	1808131	1808517	HERVHF_gag	+
Pongo_abelii_CM009274.2	1808781	1810860	HERVHF_pol	+
Pongo_abelii_CM009274.2	1811364	1811713	HERVHF_3LTR	+
Pongo_abelii_CM009274.2	5144028	5144499	HERVHF_5LTR	-
Pongo_abelii_CM009274.2	5144664	5145110	HERVHF_env	-
Pongo_abelii_CM009274.2	5146740	5148956	HERVHF_pol	-
Pongo_abelii_CM009274.2	5148459	5149289	HERVHF_pro	-
Pongo_abelii_CM009274.2	5149421	5149753	HERVHF_gag	-
Pongo_abelii_CM009274.2	5150927	5151399	HERVHF_3LTR	-
Pongo_abelii_CM009274.2	21236331	21236781	HERVHF_5LTR	+
Pongo_abelii_CM009274.2	21238314	21238865	HERVHF_gag	+
Pongo_abelii_CM009274.2	21239080	21239469	HERVHF_pro	+
Pongo_abelii_CM009274.2	21239438	21241332	HERVHF_pol	+
Pongo_abelii_CM009274.2	21241609	21242060	HERVHF_3LTR	+
Pongo_abelii_CM009274.2	35165551	35165924	HERVHF_5LTR	+
Pongo_abelii_CM009274.2	35167995	35168810	HERVHF_pro	+

Pongo_abelii_CM009274.2	35168964	35170886	HERVHF_pol	+
Pongo_abelii_CM009274.2	35172355	35173447	HERVHF_env	+
Pongo_abelii_CM009274.2	35173760	35174123	HERVHF_3LTR	+
Pongo_abelii_CM009274.2	36434009	36434456	HERVHF_5LTR	-
Pongo_abelii_CM009274.2	36435068	36437037	HERVHF_pol	-
Pongo_abelii_CM009274.2	36436609	36437202	HERVHF_pro	-
Pongo_abelii_CM009274.2	36439463	36439905	HERVHF_3LTR	-
Pongo_abelii_CM009274.2	67855955	67856374	HERVHF_5LTR	-
Pongo_abelii_CM009274.2	67856977	67858918	HERVHF_pol	-
Pongo_abelii_CM009274.2	67858355	67859155	HERVHF_pro	-
Pongo_abelii_CM009274.2	67861401	67861831	HERVHF_3LTR	-
Pongo_abelii_CM009274.2	68995162	68995496	HERVHF_5LTR	-
Pongo_abelii_CM009274.2	68996009	68998088	HERVHF_pol	-
Pongo_abelii_CM009274.2	68997600	68998424	HERVHF_pro	-
Pongo_abelii_CM009274.2	68998448	68998968	HERVHF_gag	-
Pongo_abelii_CM009274.2	69000761	69001098	HERVHF_3LTR	-
Pongo_abelii_CM009274.2	70942203	70942596	HERVHF_5LTR	-
Pongo_abelii_CM009274.2	70943184	70945156	HERVHF_pol	-
Pongo_abelii_CM009274.2	70945592	70946707	HERVHF_gag	-
Pongo_abelii_CM009274.2	70947680	70948060	HERVHF_3LTR	-
Pongo_abelii_CM009274.2	72560242	72560662	HERVHF_5LTR	+
Pongo_abelii_CM009274.2	72561696	72562529	HERVHF_pro	+
Pongo_abelii_CM009274.2	72561984	72564095	HERVHF_pol	+
Pongo_abelii_CM009274.2	72564500	72564921	HERVHF_3LTR	+
Pongo_abelii_CM009274.2	74302517	74302820	HERVHF_5LTR	+
Pongo_abelii_CM009274.2	74304404	74304715	HERVHF_gag	+
Pongo_abelii_CM009274.2	74304792	74305409	HERVHF_pro	+
Pongo_abelii_CM009274.2	74305035	74306263	HERVHF_pol	+
Pongo_abelii_CM009274.2	74308205	74308515	HERVHF_3LTR	+
Pongo_abelii_CM009274.2	86201720	86202080	HERVHF_5LTR	-
Pongo_abelii_CM009274.2	86202559	86204793	HERVHF_pol	-
Pongo_abelii_CM009274.2	86204278	86205006	HERVHF_pro	-
Pongo_abelii_CM009274.2	86206953	86207307	HERVHF_3LTR	-
Pongo_abelii_CM009274.2	125920249	125920590	HERVHF_5LTR	+
Pongo_abelii_CM009274.2	125921806	125922417	HERVHF_gag	+
Pongo_abelii_CM009274.2	125923176	125924891	HERVHF_pol	+
Pongo_abelii_CM009274.2	125926170	125926512	HERVHF_3LTR	+
Pongo_abelii_CM009275.2	30829822	30830134	HERVHF_5LTR	+
Pongo_abelii_CM009275.2	30831859	30832314	HERVHF_gag	+
Pongo_abelii_CM009275.2	30832612	30834866	HERVHF_pol	+
Pongo_abelii_CM009275.2	30835415	30835720	HERVHF_3LTR	+
Pongo_abelii_CM009275.2	37845571	37845960	HERVHF_5LTR	+
Pongo_abelii_CM009275.2	37847916	37848287	HERVHF_gag	+
Pongo_abelii_CM009275.2	37848404	37849189	HERVHF_pro	+

Pongo_abelii_CM009275.2	37848875	37850553	HERVHF_pol	+
Pongo_abelii_CM009275.2	37851197	37851579	HERVHF_3LTR	+
Pongo_abelii_CM009275.2	44373754	44374156	HERVHF_5LTR	+
Pongo_abelii_CM009275.2	44375013	44375333	HERVHF_gag	+
Pongo_abelii_CM009275.2	44375340	44376188	HERVHF_pro	+
Pongo_abelii_CM009275.2	44375628	44377866	HERVHF_pol	+
Pongo_abelii_CM009275.2	44378934	44379341	HERVHF_3LTR	+
Pongo_abelii_CM009275.2	49568632	49569087	HERVHF_5LTR	-
Pongo_abelii_CM009275.2	49569902	49571719	HERVHF_pol	-
Pongo_abelii_CM009275.2	49571084	49571914	HERVHF_pro	-
Pongo_abelii_CM009275.2	49573878	49574332	HERVHF_3LTR	-
Pongo_abelii_CM009275.2	61534765	61535151	HERVHF_5LTR	-
Pongo_abelii_CM009275.2	61535910	61537908	HERVHF_pol	-
Pongo_abelii_CM009275.2	61537267	61538013	HERVHF_pro	-
Pongo_abelii_CM009275.2	61540647	61541045	HERVHF_3LTR	-
Pongo_abelii_CM009275.2	63817792	63818145	HERVHF_5LTR	+
Pongo_abelii_CM009275.2	63819930	63820256	HERVHF_gag	+
Pongo_abelii_CM009275.2	63820416	63822550	HERVHF_pol	+
Pongo_abelii_CM009275.2	63823713	63824062	HERVHF_3LTR	+
Pongo_abelii_CM009275.2	64744222	64744675	HERVHF_5LTR	+
Pongo_abelii_CM009275.2	64746711	64747538	HERVHF_pro	+
Pongo_abelii_CM009275.2	64746900	64748826	HERVHF_pol	+
Pongo_abelii_CM009275.2	64749263	64749717	HERVHF_3LTR	+
Pongo_abelii_CM009275.2	65555266	65555623	HERVHF_5LTR	+
Pongo_abelii_CM009275.2	65558098	65560694	HERVHF_pol	+
Pongo_abelii_CM009275.2	65561194	65561550	HERVHF_3LTR	+
Pongo_abelii_CM009275.2	71069004	71069429	HERVHF_5LTR	-
Pongo_abelii_CM009275.2	71069808	71071915	HERVHF_pol	-
Pongo_abelii_CM009275.2	71072257	71072589	HERVHF_gag	-
Pongo_abelii_CM009275.2	71074296	71074704	HERVHF_3LTR	-
Pongo_abelii_CM009275.2	74897075	74897510	HERVHF_5LTR	+
Pongo_abelii_CM009275.2	74899197	74899517	HERVHF_gag	+
Pongo_abelii_CM009275.2	74899524	74900357	HERVHF_pro	+
Pongo_abelii_CM009275.2	74899815	74901722	HERVHF_pol	+
Pongo_abelii_CM009275.2	74902347	74902780	HERVHF_3LTR	+
Pongo_abelii_CM009275.2	80130626	80131079	HERVHF_5LTR	+
Pongo_abelii_CM009275.2	80132729	80133070	HERVHF_gag	+
Pongo_abelii_CM009275.2	80133176	80133739	HERVHF_pro	+
Pongo_abelii_CM009275.2	80133482	80134989	HERVHF_pol	+
Pongo_abelii_CM009275.2	80136357	80136810	HERVHF_3LTR	+
Pongo_abelii_CM009275.2	81802032	81802441	HERVHF_5LTR	+
Pongo_abelii_CM009275.2	81804185	81804514	HERVHF_gag	+
Pongo_abelii_CM009275.2	81804940	81806950	HERVHF_pol	+
Pongo_abelii_CM009275.2	81807539	81807962	HERVHF_3LTR	+

Pongo_abelii_CM009275.2	93489520	93489951	HERVHF_5LTR	+
Pongo_abelii_CM009275.2	93491805	93492104	HERVHF_gag	+
Pongo_abelii_CM009275.2	93492303	93492968	HERVHF_pro	+
Pongo_abelii_CM009275.2	93492414	93494325	HERVHF_pol	+
Pongo_abelii_CM009275.2	93494765	93495197	HERVHF_3LTR	+
Pongo_abelii_CM009276.2	11131924	11132382	HERVHF_5LTR	-
Pongo_abelii_CM009276.2	11132770	11134727	HERVHF_pol	-
Pongo_abelii_CM009276.2	11134478	11135212	HERVHF_pro	-
Pongo_abelii_CM009276.2	11135219	11135548	HERVHF_gag	-
Pongo_abelii_CM009276.2	11137455	11137918	HERVHF_3LTR	-
Pongo_abelii_CM009276.2	19073091	19073535	HERVHF_5LTR	+
Pongo_abelii_CM009276.2	19074987	19075977	HERVHF_gag	+
Pongo_abelii_CM009276.2	19076168	19076839	HERVHF_pro	+
Pongo_abelii_CM009276.2	19076195	19078199	HERVHF_pol	+
Pongo_abelii_CM009276.2	19078640	19079007	HERVHF_env	+
Pongo_abelii_CM009276.2	19079019	19079470	HERVHF_3LTR	+
Pongo_abelii_CM009276.2	29507902	29508305	HERVHF_5LTR	-
Pongo_abelii_CM009276.2	29508723	29511035	HERVHF_pol	-
Pongo_abelii_CM009276.2	29510505	29511035	HERVHF_pro	-
Pongo_abelii_CM009276.2	29511338	29511907	HERVHF_gag	-
Pongo_abelii_CM009276.2	29513221	29513626	HERVHF_3LTR	-
Pongo_abelii_CM009276.2	77695751	77696152	HERVHF_5LTR	+
Pongo_abelii_CM009276.2	77697791	77698117	HERVHF_gag	+
Pongo_abelii_CM009276.2	77698151	77699071	HERVHF_pro	+
Pongo_abelii_CM009276.2	77698586	77700690	HERVHF_pol	+
Pongo_abelii_CM009276.2	77701135	77701536	HERVHF_3LTR	+
Pongo_abelii_CM009276.2	83062340	83062690	HERVHF_5LTR	-
Pongo_abelii_CM009276.2	83063547	83065801	HERVHF_pol	-
Pongo_abelii_CM009276.2	83065166	83066005	HERVHF_pro	-
Pongo_abelii_CM009276.2	83066057	83066371	HERVHF_gag	-
Pongo_abelii_CM009276.2	83067941	83068280	HERVHF_3LTR	-
Pongo_abelii_CM009277.2	29193303	29193745	HERVHF_5LTR	-
Pongo_abelii_CM009277.2	29194155	29196141	HERVHF_pol	-
Pongo_abelii_CM009277.2	29195716	29196312	HERVHF_pro	-
Pongo_abelii_CM009277.2	29196587	29196886	HERVHF_gag	-
Pongo_abelii_CM009277.2	29198874	29199315	HERVHF_3LTR	-
Pongo_abelii_CM009277.2	29496004	29496323	HERVHF_5LTR	+
Pongo_abelii_CM009277.2	29498058	29498387	HERVHF_gag	+
Pongo_abelii_CM009277.2	29498397	29499245	HERVHF_pro	+
Pongo_abelii_CM009277.2	29498682	29500643	HERVHF_pol	+
Pongo_abelii_CM009277.2	29501347	29501677	HERVHF_3LTR	+
Pongo_abelii_CM009277.2	68881615	68882063	HERVHF_5LTR	-
Pongo_abelii_CM009277.2	68882670	68884679	HERVHF_pol	-
Pongo_abelii_CM009277.2	68884422	68885051	HERVHF_pro	-

Pongo_abelii_CM009277.2	68887295	68887734	HERVHF_3LTR	-
Pongo_abelii_CM009277.2	72871317	72871743	HERVHF_5LTR	+
Pongo_abelii_CM009277.2	72873429	72873869	HERVHF_gag	+
Pongo_abelii_CM009277.2	72873916	72874719	HERVHF_pro	+
Pongo_abelii_CM009277.2	72874831	72876472	HERVHF_pol	+
Pongo_abelii_CM009277.2	72876864	72877300	HERVHF_3LTR	+
Pongo_abelii_CM009277.2	80883269	80883698	HERVHF_5LTR	+
Pongo_abelii_CM009277.2	80885274	80885717	HERVHF_gag	+
Pongo_abelii_CM009277.2	80886146	80888339	HERVHF_pol	+
Pongo_abelii_CM009277.2	80888716	80889141	HERVHF_3LTR	+
Pongo_abelii_CM009277.2	81513881	81514254	HERVHF_5LTR	+
Pongo_abelii_CM009277.2	81515847	81516251	HERVHF_gag	+
Pongo_abelii_CM009277.2	81516397	81517083	HERVHF_pro	+
Pongo_abelii_CM009277.2	81516574	81518677	HERVHF_pol	+
Pongo_abelii_CM009277.2	81519215	81519575	HERVHF_3LTR	+
Pongo_abelii_CM009278.2	10567617	10568068	HERVHF_5LTR	-
Pongo_abelii_CM009278.2	10569056	10571160	HERVHF_pol	-
Pongo_abelii_CM009278.2	10570561	10571364	HERVHF_pro	-
Pongo_abelii_CM009278.2	10571797	10572324	HERVHF_gag	-
Pongo_abelii_CM009278.2	10573932	10574382	HERVHF_3LTR	-
Pongo_abelii_CM009278.2	37406037	37406381	HERVHF_5LTR	-
Pongo_abelii_CM009278.2	37407042	37408956	HERVHF_pol	-
Pongo_abelii_CM009278.2	37408414	37409220	HERVHF_pro	-
Pongo_abelii_CM009278.2	37409274	37409573	HERVHF_gag	-
Pongo_abelii_CM009278.2	37411281	37411630	HERVHF_3LTR	-
Pongo_abelii_CM009279.2	51338168	51338627	HERVHF_5LTR	-
Pongo_abelii_CM009279.2	51339404	51340927	HERVHF_pol	-
Pongo_abelii_CM009279.2	51340610	51341443	HERVHF_pro	-
Pongo_abelii_CM009279.2	51341476	51341784	HERVHF_gag	-
Pongo_abelii_CM009279.2	51343913	51344376	HERVHF_3LTR	-
Pongo_abelii_CM009279.2	60300149	60300616	HERVHF_5LTR	-
Pongo_abelii_CM009279.2	60301145	60303173	HERVHF_pol	-
Pongo_abelii_CM009279.2	60302577	60303425	HERVHF_pro	-
Pongo_abelii_CM009279.2	60303493	60303840	HERVHF_gag	-
Pongo_abelii_CM009279.2	60305573	60306036	HERVHF_3LTR	-
Pongo_abelii_CM009279.2	67400065	67400499	HERVHF_5LTR	-
Pongo_abelii_CM009279.2	67400658	67401220	HERVHF_env	-
Pongo_abelii_CM009279.2	67401511	67403384	HERVHF_pol	-
Pongo_abelii_CM009279.2	67402856	67403818	HERVHF_pro	-
Pongo_abelii_CM009279.2	67405862	67406295	HERVHF_3LTR	-
Pongo_abelii_CM009280.2	917546	918060	HERVK_5LTR	-
Pongo_abelii_CM009280.2	918148	918465	HERVK_env	-
Pongo_abelii_CM009280.2	918933	920066	HERVK_pol	-
Pongo_abelii_CM009280.2	920003	920935	HERVK_pro	-

Pongo_abelii_CM009280.2	922334	922849	HERVK_3LTR	-
Pongo_abelii_CM009280.2	29899546	29900098	HERVHF_5LTR	+
Pongo_abelii_CM009280.2	29902117	29902416	HERVHF_gag	+
Pongo_abelii_CM009280.2	29902463	29903242	HERVHF_pro	+
Pongo_abelii_CM009280.2	29902631	29904379	HERVHF_pol	+
Pongo_abelii_CM009280.2	29905207	29905959	HERVHF_env	+
Pongo_abelii_CM009280.2	29906711	29907248	HERVHF_3LTR	+
Pongo_abelii_CM009280.2	30039027	30039456	HERVHF_5LTR	-
Pongo_abelii_CM009280.2	30039898	30042032	HERVHF_pol	-
Pongo_abelii_CM009280.2	30042034	30042465	HERVHF_pro	-
Pongo_abelii_CM009280.2	30042465	30042890	HERVHF_gag	-
Pongo_abelii_CM009280.2	30044264	30044671	HERVHF_3LTR	-
Pongo_abelii_CM009280.2	37467767	37468176	HERVHF_5LTR	+
Pongo_abelii_CM009280.2	37469884	37470207	HERVHF_gag	+
Pongo_abelii_CM009280.2	37470496	37472574	HERVHF_pol	+
Pongo_abelii_CM009280.2	37473028	37473433	HERVHF_3LTR	+
Pongo_abelii_CM009280.2	52242989	52243438	HERVHF_5LTR	-
Pongo_abelii_CM009280.2	52243606	52245336	HERVHF_env	-
Pongo_abelii_CM009280.2	52245648	52247984	HERVHF_pol	-
Pongo_abelii_CM009280.2	52247394	52247975	HERVHF_pro	-
Pongo_abelii_CM009280.2	52250390	52250842	HERVHF_3LTR	-
Pongo_abelii_CM009281.2	45697098	45697522	HERVHF_5LTR	+
Pongo_abelii_CM009281.2	45699123	45699776	HERVHF_gag	+
Pongo_abelii_CM009281.2	45699886	45700662	HERVHF_pro	+
Pongo_abelii_CM009281.2	45700234	45702480	HERVHF_pol	+
Pongo_abelii_CM009281.2	45702887	45703304	HERVHF_3LTR	+
Pongo_abelii_CM009282.2	27520021	27520390	HERVHF_5LTR	-
Pongo_abelii_CM009282.2	27520565	27521698	HERVHF_env	-
Pongo_abelii_CM009282.2	27522999	27523796	HERVHF_pol	-
Pongo_abelii_CM009282.2	27523260	27523970	HERVHF_pro	-
Pongo_abelii_CM009282.2	27525628	27525987	HERVHF_3LTR	-
Pongo_abelii_CM009282.2	52314002	52314442	HERVHF_5LTR	-
Pongo_abelii_CM009282.2	52314851	52317012	HERVHF_pol	-
Pongo_abelii_CM009282.2	52316422	52317003	HERVHF_pro	-
Pongo_abelii_CM009282.2	52317278	52317577	HERVHF_gag	-
Pongo_abelii_CM009282.2	52319227	52319670	HERVHF_3LTR	-
Pongo_abelii_CM009283.2	3070204	3070669	HERVHF_5LTR	-
Pongo_abelii_CM009283.2	3071078	3073240	HERVHF_pol	-
Pongo_abelii_CM009283.2	3073651	3073950	HERVHF_gag	-
Pongo_abelii_CM009283.2	3075499	3075955	HERVHF_3LTR	-
Pongo_abelii_CM009283.2	4008829	4009233	HERVHF_5LTR	+
Pongo_abelii_CM009283.2	4011016	4011357	HERVHF_gag	+
Pongo_abelii_CM009283.2	4011415	4012287	HERVHF_pro	+
Pongo_abelii_CM009283.2	4011577	4014109	HERVHF_pol	+

Pongo_abelii_CM009283.2	4014561	4014966	HERVHF_3LTR	+
Pongo_abelii_CM009283.2	13770177	13770595	HERVHF_5LTR	-
Pongo_abelii_CM009283.2	13771015	13773267	HERVHF_pol	-
Pongo_abelii_CM009283.2	13775495	13775908	HERVHF_3LTR	-
Pongo_abelii_CM009283.2	15734079	15734494	HERVHF_5LTR	+
Pongo_abelii_CM009283.2	15736570	15737151	HERVHF_pro	+
Pongo_abelii_CM009283.2	15736609	15738548	HERVHF_pol	+
Pongo_abelii_CM009283.2	15739133	15739561	HERVHF_3LTR	+
Pongo_abelii_CM009285.2	26779043	26779454	HERVHF_5LTR	+
Pongo_abelii_CM009285.2	26781343	26782092	HERVHF_pro	+
Pongo_abelii_CM009285.2	26781508	26783397	HERVHF_pol	+
Pongo_abelii_CM009285.2	26784293	26784705	HERVHF_3LTR	+
Pongo_abelii_CM009285.2	27145831	27146251	HERVHF_5LTR	-
Pongo_abelii_CM009285.2	27146709	27148922	HERVHF_pol	-
Pongo_abelii_CM009285.2	27149220	27149549	HERVHF_gag	-
Pongo_abelii_CM009285.2	27150987	27151406	HERVHF_3LTR	-
Pongo_abelii_CM009285.2	28083761	28084074	HERVHF_5LTR	+
Pongo_abelii_CM009285.2	28086244	28088492	HERVHF_pol	+
Pongo_abelii_CM009285.2	28089122	28089437	HERVHF_3LTR	+
Pongo_abelii_CM009285.2	69972225	69972648	HERVHF_5LTR	-
Pongo_abelii_CM009285.2	69973231	69974984	HERVHF_pol	-
Pongo_abelii_CM009285.2	69974577	69975323	HERVHF_pro	-
Pongo_abelii_CM009285.2	69975434	69975733	HERVHF_gag	-
Pongo_abelii_CM009285.2	69977375	69977791	HERVHF_3LTR	-
Pongo_abelii_CM009285.2	76768998	76769429	HERVHF_5LTR	+
Pongo_abelii_CM009285.2	76770143	76771114	HERVHF_gag	+
Pongo_abelii_CM009285.2	76771952	76774108	HERVHF_pol	+
Pongo_abelii_CM009285.2	76774614	76774964	HERVHF_env	+
Pongo_abelii_CM009285.2	76775128	76775559	HERVHF_3LTR	+
Pongo_abelii_CM009285.2	85207773	85208092	HERVHF_5LTR	+
Pongo_abelii_CM009285.2	85209738	85210496	HERVHF_pro	+
Pongo_abelii_CM009285.2	85209879	85211901	HERVHF_pol	+
Pongo_abelii_CM009285.2	85212475	85212790	HERVHF_3LTR	+
Pongo_abelii_CM009285.2	103701996	103702447	HERVHF_5LTR	+
Pongo_abelii_CM009285.2	103704982	103706913	HERVHF_pol	+
Pongo_abelii_CM009285.2	103706876	103707193	HERVHF_env	+
Pongo_abelii_CM009285.2	103707356	103707808	HERVHF_3LTR	+
Pongo_abelii_CM009285.2	108632360	108632768	HERVHF_5LTR	+
Pongo_abelii_CM009285.2	108634194	108634703	HERVHF_gag	+
Pongo_abelii_CM009285.2	108634830	108635588	HERVHF_pro	+
Pongo_abelii_CM009285.2	108635067	108637372	HERVHF_pol	+
Pongo_abelii_CM009285.2	108643495	108643887	HERVHF_3LTR	+
Pongo_abelii_CM009285.2	109151223	109151656	HERVHF_5LTR	-
Pongo_abelii_CM009285.2	109152250	109154197	HERVHF_pol	-

Pongo_abelii_CM009285.2	109153619	109154320	HERVHF_pro	-
Pongo_abelii_CM009285.2	109154472	109154855	HERVHF_gag	-
Pongo_abelii_CM009285.2	109156701	109157133	HERVHF_3LTR	-
Pongo_abelii_CM009285.2	119911835	119912269	HERVHF_5LTR	-
Pongo_abelii_CM009285.2	119912889	119914876	HERVHF_pol	-
Pongo_abelii_CM009285.2	119914238	119915368	HERVHF_pro	-
Pongo_abelii_CM009285.2	119915072	119915455	HERVHF_gag	-
Pongo_abelii_CM009285.2	119917000	119917432	HERVHF_3LTR	-
Pongo_abelii_CM009285.2	137292269	137292681	HERVHF_5LTR	-
Pongo_abelii_CM009285.2	137293591	137295380	HERVHF_pol	-
Pongo_abelii_CM009285.2	137295812	137296153	HERVHF_gag	-
Pongo_abelii_CM009285.2	137297528	137297941	HERVHF_3LTR	-
Pongo_abelii_KZ622955.1	849918	850328	HERVHF_5LTR	+
Pongo_abelii_KZ622955.1	852894	854411	HERVHF_pol	+
Pongo_abelii_KZ622955.1	855504	855910	HERVHF_3LTR	+
Pongo_abelii_NDHI03000125.1	27007	27343	HERVHF_5LTR	-
Pongo_abelii_NDHI03000125.1	27984	29932	HERVHF_pol	-
Pongo_abelii_NDHI03000125.1	29360	30178	HERVHF_pro	-
Pongo_abelii_NDHI03000125.1	32383	32721	HERVHF_3LTR	-
Pongo_abelii_NDHI03000614.1	3494	3948	HERVHF_5LTR	+
Pongo_abelii_NDHI03000614.1	6012	6857	HERVHF_pro	+
Pongo_abelii_NDHI03000614.1	6315	7609	HERVHF_pol	+
Pongo_abelii_NDHI03000614.1	8832	9284	HERVHF_3LTR	+
Pongo_abelii_NDHI03003893.1	83150	83604	HERVHF_5LTR	+
Pongo_abelii_NDHI03003893.1	85515	85844	HERVHF_gag	+
Pongo_abelii_NDHI03003893.1	85848	86681	HERVHF_pro	+
Pongo_abelii_NDHI03003893.1	86184	88245	HERVHF_pol	+
Pongo_abelii_NDHI03003893.1	88655	89110	HERVHF_3LTR	+
Pongo_abelii_NDHI03003980.1	132789	133113	HERVHF_5LTR	+
Pongo_abelii_NDHI03003980.1	134725	135165	HERVHF_gag	+
Pongo_abelii_NDHI03003980.1	135275	135880	HERVHF_pro	+
Pongo_abelii_NDHI03003980.1	135469	137665	HERVHF_pol	+
Pongo_abelii_NDHI03003980.1	137729	138053	HERVHF_env	+
Pongo_abelii_NDHI03003980.1	138071	138390	HERVHF_3LTR	+
Pongo_abelii_NDHI03004321.1	1864	2311	HERVHF_5LTR	+
Pongo_abelii_NDHI03004321.1	3763	4221	HERVHF_gag	+
Pongo_abelii_NDHI03004321.1	4551	5408	HERVHF_pro	+
Pongo_abelii_NDHI03004321.1	4755	6740	HERVHF_pol	+
Pongo_abelii_NDHI03004321.1	7353	7799	HERVHF_3LTR	+
Pongo_abelii_NDHI03004652.1	52934	53243	HERVHF_5LTR	+
Pongo_abelii_NDHI03004652.1	55043	55366	HERVHF_gag	+
Pongo_abelii_NDHI03004652.1	55507	56307	HERVHF_pro	+
Pongo_abelii_NDHI03004652.1	55744	57435	HERVHF_pol	+
Pongo_abelii_NDHI03004652.1	58258	58571	HERVHF_3LTR	+

Pongo_abelii_NDHI03004823.1	27589	28043	HERVHF_5LTR	+
Pongo_abelii_NDHI03004823.1	29778	30191	HERVHF_gag	+
Pongo_abelii_NDHI03004823.1	30503	30970	HERVHF_pro	+
Pongo_abelii_NDHI03004823.1	30623	32612	HERVHF_pol	+
Pongo_abelii_NDHI03004823.1	33021	33472	HERVHF_3LTR	+
Pongo_pygmaeus_ctg10	5187447	5187785	HERVIPADP_5LTR	+
Pongo_pygmaeus_ctg10	5190547	5193035	HERVIPADP_pol	+
Pongo_pygmaeus_ctg10	5194432	5195139	HERVIPADP_env	+
Pongo_pygmaeus_ctg10	5195396	5195721	HERVIPADP_3LTR	+
Pongo_pygmaeus_ctg11	41236857	41237225	HERVHF_5LTR	-
Pongo_pygmaeus_ctg11	41237930	41241626	HERVHF_pol	-
Pongo_pygmaeus_ctg11	41241996	41243018	HERVHF_gag	-
Pongo_pygmaeus_ctg11	41244400	41244779	HERVHF_3LTR	-
Pongo_pygmaeus_ctg118	8510861	8511285	HERVHF_5LTR	-
Pongo_pygmaeus_ctg118	8511717	8512880	HERVHF_pol	-
Pongo_pygmaeus_ctg118	8512416	8513219	HERVHF_pro	-
Pongo_pygmaeus_ctg118	8513253	8513693	HERVHF_gag	-
Pongo_pygmaeus_ctg118	8515404	8515845	HERVHF_3LTR	-
Pongo_pygmaeus_ctg127	6274998	6275462	HERVK_5LTR	+
Pongo_pygmaeus_ctg127	6276952	6277857	HERVK_pro	+
Pongo_pygmaeus_ctg127	6277781	6279679	HERVK_pol	+
Pongo_pygmaeus_ctg127	6280608	6281078	HERVK_3LTR	+
Pongo_pygmaeus_ctg17	10560545	10560926	HERVHF_5LTR	+
Pongo_pygmaeus_ctg17	10562353	10562826	HERVHF_gag	+
Pongo_pygmaeus_ctg17	10563408	10566219	HERVHF_pol	+
Pongo_pygmaeus_ctg17	10567102	10567480	HERVHF_3LTR	+
Pongo_pygmaeus_ctg17	25978851	25979234	HUERSP_5LTR	+
Pongo_pygmaeus_ctg17	25981520	25982164	HUERSP_gag	+
Pongo_pygmaeus_ctg17	25982891	25985866	HUERSP_pol	+
Pongo_pygmaeus_ctg17	25986660	25987043	HUERSP_3LTR	+
Pongo_pygmaeus_ctg182	1739756	1740075	HERVK_5LTR	-
Pongo_pygmaeus_ctg182	1741648	1743386	HERVK_pol	-
Pongo_pygmaeus_ctg182	1743643	1744218	HERVK_pro	-
Pongo_pygmaeus_ctg182	1744272	1745227	HERVK_gag	-
Pongo_pygmaeus_ctg182	1753496	1753805	HERVK_3LTR	-
Pongo_pygmaeus_ctg186	3174171	3174507	HERVHF_5LTR	-
Pongo_pygmaeus_ctg186	3175182	3175748	HERVHF_env	-
Pongo_pygmaeus_ctg186	3175788	3176327	HERVHF_pol	-
Pongo_pygmaeus_ctg186	3177037	3177744	HERVHF_gag	-
Pongo_pygmaeus_ctg186	3179260	3179598	HERVHF_3LTR	-
Pongo_pygmaeus_ctg2	20244924	20245257	HERVK_5LTR	+
Pongo_pygmaeus_ctg2	20245778	20247027	HERVK_gag	+
Pongo_pygmaeus_ctg2	20246973	20247617	HERVK_pro	+
Pongo_pygmaeus_ctg2	20247808	20248954	HERVK_pol	+

Pongo_pygmaeus_ctg2	20249583	20249948	HERVK_env	+
Pongo_pygmaeus_ctg2	20250153	20250490	HERVK_3LTR	+
Pongo_pygmaeus_ctg24	13655583	13655993	HERVIPADP_5LTR	+
Pongo_pygmaeus_ctg24	13660315	13662914	HERVIPADP_pol	+
Pongo_pygmaeus_ctg24	13664666	13665904	HERVIPADP_env	+
Pongo_pygmaeus_ctg24	13666146	13666554	HERVIPADP_3LTR	+
Pongo_pygmaeus_ctg25	24997643	24997999	HUERSP_5LTR	-
Pongo_pygmaeus_ctg25	25005589	25007415	HUERSP_pol	-
Pongo_pygmaeus_ctg25	25008057	25008482	HUERSP_gag	-
Pongo_pygmaeus_ctg25	25012310	25012661	HUERSP_3LTR	-
Pongo_pygmaeus_ctg27	48366463	48366866	HERVHF_5LTR	+
Pongo_pygmaeus_ctg27	48367678	48370644	HERVHF_pol	+
Pongo_pygmaeus_ctg27	48371096	48371504	HERVHF_3LTR	+
Pongo_pygmaeus_ctg27	63644205	63644705	HERVK_5LTR	-
Pongo_pygmaeus_ctg27	63644712	63645236	HERVK_env	-
Pongo_pygmaeus_ctg27	63645980	63646819	HERVK_pol	-
Pongo_pygmaeus_ctg27	63646714	63647655	HERVK_pro	-
Pongo_pygmaeus_ctg27	63648391	63648903	HERVK_3LTR	-
Pongo_pygmaeus_ctg270	1002231	1002664	HERVK_5LTR	-
Pongo_pygmaeus_ctg270	1004274	1005491	HERVK_pol	-
Pongo_pygmaeus_ctg270	1005410	1006322	HERVK_pro	-
Pongo_pygmaeus_ctg270	1007237	1007685	HERVK_3LTR	-
Pongo_pygmaeus_ctg3	1782684	1783026	HERVHF_5LTR	-
Pongo_pygmaeus_ctg3	1783258	1784092	HERVHF_env	-
Pongo_pygmaeus_ctg3	1784797	1788228	HERVHF_pol	-
Pongo_pygmaeus_ctg3	1788224	1788694	HERVHF_pro	-
Pongo_pygmaeus_ctg3	1788911	1789333	HERVHF_gag	-
Pongo_pygmaeus_ctg3	1797036	1797366	HERVHF_3LTR	-
Pongo_pygmaeus_ctg3	18122978	18123338	HERVIPADP_5LTR	+
Pongo_pygmaeus_ctg3	18126011	18129065	HERVIPADP_pol	+
Pongo_pygmaeus_ctg3	18130146	18130523	HERVIPADP_env	+
Pongo_pygmaeus_ctg3	18131262	18131619	HERVIPADP_3LTR	+
Pongo_pygmaeus_ctg334	1015094	1015481	HERVK_5LTR	+
Pongo_pygmaeus_ctg334	1016483	1017262	HERVK_pro	+
Pongo_pygmaeus_ctg334	1017157	1018423	HERVK_pol	+
Pongo_pygmaeus_ctg334	1019333	1019722	HERVK_3LTR	+
Pongo_pygmaeus_ctg35	38505333	38505735	HERVHF_5LTR	+
Pongo_pygmaeus_ctg35	38507509	38507814	HERVHF_gag	+
Pongo_pygmaeus_ctg35	38507850	38508680	HERVHF_pro	+
Pongo_pygmaeus_ctg35	38508039	38510265	HERVHF_pol	+
Pongo_pygmaeus_ctg35	38510661	38511060	HERVHF_3LTR	+
Pongo_pygmaeus_ctg53	1530975	1531297	HERVHF_5LTR	+
Pongo_pygmaeus_ctg53	1533777	1534703	HERVHF_pro	+
Pongo_pygmaeus_ctg53	1534203	1536514	HERVHF_pol	+

Pongo_pygmaeus_ctg53	1537092	1537410	HERVHF_3LTR	+
Pongo_pygmaeus_ctg6	16858518	16858908	HERVHF_5LTR	+
Pongo_pygmaeus_ctg6	16863611	16864042	HERVHF_gag	+
Pongo_pygmaeus_ctg6	16864888	16865721	HERVHF_pro	+
Pongo_pygmaeus_ctg6	16865377	16866320	HERVHF_pol	+
Pongo_pygmaeus_ctg6	16872426	16872820	HERVHF_3LTR	+
Pongo_pygmaeus_ctg7	13982397	13982794	HSERVIII_5LTR	-
Pongo_pygmaeus_ctg7	13983587	13986530	HSERVIII_pol	-
Pongo_pygmaeus_ctg7	13988926	13989311	HSERVIII_3LTR	-
Pongo_pygmaeus_ctg8	56807488	56807996	HERVK_5LTR	+
Pongo_pygmaeus_ctg8	56809916	56810674	HERVK_pro	+
Pongo_pygmaeus_ctg8	56810569	56812486	HERVK_pol	+
Pongo_pygmaeus_ctg8	56813290	56813795	HERVK_3LTR	+
Pongo_pygmaeus_ctg87	7422280	7422637	HSERVIII_5LTR	-
Pongo_pygmaeus_ctg87	7423226	7426259	HSERVIII_pol	-
Pongo_pygmaeus_ctg87	7428304	7428667	HSERVIII_3LTR	-
Pongo_pygmaeus_ctg9	669003	669458	HERVIPADP_5LTR	-
Pongo_pygmaeus_ctg9	669753	670232	HERVIPADP_env	-
Pongo_pygmaeus_ctg9	672259	674464	HERVIPADP_pol	-
Pongo_pygmaeus_ctg9	674695	675105	HERVIPADP_gag	-
Pongo_pygmaeus_ctg9	676608	677076	HERVIPADP_3LTR	-
Prolemur_simus_MPIZ01001670.1	146461	146923	HERVHF_5LTR	+
Prolemur_simus_MPIZ01001670.1	147945	148361	HERVHF_gag	+
Prolemur_simus_MPIZ01001670.1	148389	148955	HERVHF_pro	+
Prolemur_simus_MPIZ01001670.1	148961	149584	HERVHF_pol	+
Prolemur_simus_MPIZ01001670.1	151333	151795	HERVHF_3LTR	+
Pygathrix_nemaeus_ctg1	13462522	13463026	HERVK_5LTR	-
Pygathrix_nemaeus_ctg1	13464106	13465833	HERVK_pol	-
Pygathrix_nemaeus_ctg1	13465883	13466669	HERVK_pro	-
Pygathrix_nemaeus_ctg1	13467540	13468045	HERVK_3LTR	-
Pygathrix_nemaeus_ctg1	19745114	19745513	HERVHF_5LTR	-
Pygathrix_nemaeus_ctg1	19746040	19748412	HERVHF_pol	-
Pygathrix_nemaeus_ctg1	19749794	19750184	HERVHF_3LTR	-
Pygathrix_nemaeus_ctg1	28998574	28998946	HERVHF_5LTR	+
Pygathrix_nemaeus_ctg1	29001077	29001619	HERVHF_pro	+
Pygathrix_nemaeus_ctg1	29004813	29008975	HERVHF_pol	+
Pygathrix_nemaeus_ctg1	29010039	29010416	HERVHF_3LTR	+
Pygathrix_nemaeus_ctg108	5489385	5489773	HERVHF_5LTR	-
Pygathrix_nemaeus_ctg108	5490356	5492108	HERVHF_pol	-
Pygathrix_nemaeus_ctg108	5494850	5495244	HERVHF_3LTR	-
Pygathrix_nemaeus_ctg110	28428	28772	HERVHF_5LTR	+
Pygathrix_nemaeus_ctg110	30374	30697	HERVHF_gag	+
Pygathrix_nemaeus_ctg110	30756	31523	HERVHF_pro	+
Pygathrix_nemaeus_ctg110	30897	32934	HERVHF_pol	+

Pygathrix_nemaeus_ctg110	33494	33828	HERVHF_3LTR	+
Pygathrix_nemaeus_ctg113	462081	462384	HERVHF_5LTR	+
Pygathrix_nemaeus_ctg113	463939	464646	HERVHF_gag	+
Pygathrix_nemaeus_ctg113	465185	467494	HERVHF_pol	+
Pygathrix_nemaeus_ctg113	471163	471469	HERVHF_3LTR	+
Pygathrix_nemaeus_ctg115	1805092	1805561	HERVK_5LTR	+
Pygathrix_nemaeus_ctg115	1805741	1806568	HERVK_gag	+
Pygathrix_nemaeus_ctg115	1807234	1807916	HERVK_pro	+
Pygathrix_nemaeus_ctg115	1807779	1809762	HERVK_pol	+
Pygathrix_nemaeus_ctg115	1810986	1811431	HERVK_3LTR	+
Pygathrix_nemaeus_ctg16	2394005	2394454	HERVHF_5LTR	-
Pygathrix_nemaeus_ctg16	2395144	2396763	HERVHF_pol	-
Pygathrix_nemaeus_ctg16	2398946	2399397	HERVHF_3LTR	-
Pygathrix_nemaeus_ctg163	4572964	4573342	HERVHF_5LTR	-
Pygathrix_nemaeus_ctg163	4573783	4577453	HERVHF_pol	-
Pygathrix_nemaeus_ctg163	4578760	4579059	HERVHF_gag	-
Pygathrix_nemaeus_ctg163	4580462	4580833	HERVHF_3LTR	-
Pygathrix_nemaeus_ctg17	8238923	8239306	HERVHF_5LTR	-
Pygathrix_nemaeus_ctg17	8240406	8241630	HERVHF_pol	-
Pygathrix_nemaeus_ctg17	8242077	8242400	HERVHF_gag	-
Pygathrix_nemaeus_ctg17	8244527	8244923	HERVHF_3LTR	-
Pygathrix_nemaeus_ctg2	25877365	25877676	HERVHF_5LTR	-
Pygathrix_nemaeus_ctg2	25878367	25880604	HERVHF_pol	-
Pygathrix_nemaeus_ctg2	25880044	25880805	HERVHF_pro	-
Pygathrix_nemaeus_ctg2	25880877	25881200	HERVHF_gag	-
Pygathrix_nemaeus_ctg2	25882894	25883213	HERVHF_3LTR	-
Pygathrix_nemaeus_ctg21	23576327	23576837	HERVK_5LTR	-
Pygathrix_nemaeus_ctg21	23577046	23577348	HERVK_env	-
Pygathrix_nemaeus_ctg21	23578160	23579041	HERVK_pol	-
Pygathrix_nemaeus_ctg21	23578936	23579877	HERVK_pro	-
Pygathrix_nemaeus_ctg21	23581294	23581806	HERVK_3LTR	-
Pygathrix_nemaeus_ctg211	1610604	1610910	HERVHF_5LTR	+
Pygathrix_nemaeus_ctg211	1615873	1616232	HERVHF_gag	+
Pygathrix_nemaeus_ctg211	1616878	1618937	HERVHF_pol	+
Pygathrix_nemaeus_ctg211	1619550	1619857	HERVHF_3LTR	+
Pygathrix_nemaeus_ctg226	1472473	1472835	HERVHF_5LTR	-
Pygathrix_nemaeus_ctg226	1473718	1475434	HERVHF_pol	-
Pygathrix_nemaeus_ctg226	1474778	1475611	HERVHF_pro	-
Pygathrix_nemaeus_ctg226	1477667	1478038	HERVHF_3LTR	-
Pygathrix_nemaeus_ctg23	25316741	25317066	HERVHF_5LTR	-
Pygathrix_nemaeus_ctg23	25317512	25319397	HERVHF_pol	-
Pygathrix_nemaeus_ctg23	25321879	25322201	HERVHF_3LTR	-
Pygathrix_nemaeus_ctg23	30320113	30320464	HERVK_5LTR	-
Pygathrix_nemaeus_ctg23	30320599	30321029	HERVK_env	-

Pygathrix_nemaeus_ctg23	30321446	30323336	HERVK_pol	-
Pygathrix_nemaeus_ctg23	30323255	30324168	HERVK_pro	-
Pygathrix_nemaeus_ctg23	30324158	30325362	HERVK_gag	-
Pygathrix_nemaeus_ctg23	30325903	30326248	HERVK_3LTR	-
Pygathrix_nemaeus_ctg241	1556338	1556673	HERVHF_5LTR	+
Pygathrix_nemaeus_ctg241	1557899	1558420	HERVHF_gag	+
Pygathrix_nemaeus_ctg241	1558663	1561140	HERVHF_pol	+
Pygathrix_nemaeus_ctg241	1561725	1562070	HERVHF_3LTR	+
Pygathrix_nemaeus_ctg30	1939142	1939581	HERVHF_5LTR	+
Pygathrix_nemaeus_ctg30	1940897	1941298	HERVHF_gag	+
Pygathrix_nemaeus_ctg30	1941638	1942156	HERVHF_pro	+
Pygathrix_nemaeus_ctg30	1942110	1944118	HERVHF_pol	+
Pygathrix_nemaeus_ctg30	1944671	1945111	HERVHF_3LTR	+
Pygathrix_nemaeus_ctg305	869606	870000	HERVHF_5LTR	+
Pygathrix_nemaeus_ctg305	871562	871885	HERVHF_gag	+
Pygathrix_nemaeus_ctg305	872016	872765	HERVHF_pro	+
Pygathrix_nemaeus_ctg305	872106	874353	HERVHF_pol	+
Pygathrix_nemaeus_ctg305	875192	875590	HERVHF_3LTR	+
Pygathrix_nemaeus_ctg31	3692842	3693199	HERVHF_5LTR	+
Pygathrix_nemaeus_ctg31	3695136	3697085	HERVHF_gag	+
Pygathrix_nemaeus_ctg31	3697476	3699367	HERVHF_pol	+
Pygathrix_nemaeus_ctg31	3699868	3700242	HERVHF_3LTR	+
Pygathrix_nemaeus_ctg339	454897	455221	HERVK_5LTR	+
Pygathrix_nemaeus_ctg339	456276	457217	HERVK_pro	+
Pygathrix_nemaeus_ctg339	457115	458387	HERVK_pol	+
Pygathrix_nemaeus_ctg339	459233	459557	HERVK_3LTR	+
Pygathrix_nemaeus_ctg35	16092793	16093180	HERVK_5LTR	-
Pygathrix_nemaeus_ctg35	16094799	16095941	HERVK_pol	-
Pygathrix_nemaeus_ctg35	16095925	16096788	HERVK_pro	-
Pygathrix_nemaeus_ctg35	16098229	16098608	HERVK_3LTR	-
Pygathrix_nemaeus_ctg37	782433	782863	HERVHF_5LTR	-
Pygathrix_nemaeus_ctg37	783678	786044	HERVHF_pol	-
Pygathrix_nemaeus_ctg37	785634	786683	HERVHF_pro	-
Pygathrix_nemaeus_ctg37	786804	787151	HERVHF_gag	-
Pygathrix_nemaeus_ctg37	788625	789061	HERVHF_3LTR	-
Pygathrix_nemaeus_ctg38	3876301	3876755	HERVHF_5LTR	+
Pygathrix_nemaeus_ctg38	3884412	3884920	HERVHF_gag	+
Pygathrix_nemaeus_ctg38	3885037	3885429	HERVHF_pro	+
Pygathrix_nemaeus_ctg38	3885211	3887338	HERVHF_pol	+
Pygathrix_nemaeus_ctg38	3887994	3888450	HERVHF_3LTR	+
Pygathrix_nemaeus_ctg39	21192755	21193190	HERVHF_5LTR	-
Pygathrix_nemaeus_ctg39	21194184	21196108	HERVHF_pol	-
Pygathrix_nemaeus_ctg39	21196941	21197363	HERVHF_pro	-
Pygathrix_nemaeus_ctg39	21199232	21199665	HERVHF_3LTR	-

Pygathrix_nemaeus_ctg48	4365541	4366052	HERVK_5LTR	+
Pygathrix_nemaeus_ctg48	4366933	4367682	HERVK_pro	+
Pygathrix_nemaeus_ctg48	4367669	4369243	HERVK_pol	+
Pygathrix_nemaeus_ctg48	4370000	4370401	HERVK_env	+
Pygathrix_nemaeus_ctg48	4370473	4370982	HERVK_3LTR	+
Pygathrix_nemaeus_ctg48	12780885	12781300	HERVHF_5LTR	+
Pygathrix_nemaeus_ctg48	12783611	12785951	HERVHF_pol	+
Pygathrix_nemaeus_ctg48	12786504	12786916	HERVHF_3LTR	+
Pygathrix_nemaeus_ctg5	20242040	20242511	HERVHF_5LTR	-
Pygathrix_nemaeus_ctg5	20243184	20244680	HERVHF_pol	-
Pygathrix_nemaeus_ctg5	20247311	20247783	HERVHF_3LTR	-
Pygathrix_nemaeus_ctg5	37143667	37144124	HERVHF_5LTR	-
Pygathrix_nemaeus_ctg5	37145349	37147374	HERVHF_pol	-
Pygathrix_nemaeus_ctg5	37146772	37147542	HERVHF_pro	-
Pygathrix_nemaeus_ctg5	37149511	37149966	HERVHF_3LTR	-
Pygathrix_nemaeus_ctg6	45892642	45893020	HERVHF_5LTR	+
Pygathrix_nemaeus_ctg6	45895423	45897494	HERVHF_pol	+
Pygathrix_nemaeus_ctg6	45898399	45898770	HERVHF_3LTR	+
Pygathrix_nemaeus_ctg66	786716	787114	HERVHF_5LTR	-
Pygathrix_nemaeus_ctg66	788625	789278	HERVHF_pol	-
Pygathrix_nemaeus_ctg66	788643	789380	HERVHF_pro	-
Pygathrix_nemaeus_ctg66	791494	791902	HERVHF_3LTR	-
Pygathrix_nemaeus_ctg68	401930	402357	HERVHF_5LTR	-
Pygathrix_nemaeus_ctg68	402526	403087	HERVHF_env	-
Pygathrix_nemaeus_ctg68	403113	405335	HERVHF_pol	-
Pygathrix_nemaeus_ctg68	407833	408263	HERVHF_3LTR	-
Pygathrix_nemaeus_ctg69	2350823	2351153	HERVHF_5LTR	-
Pygathrix_nemaeus_ctg69	2351953	2353494	HERVHF_pol	-
Pygathrix_nemaeus_ctg69	2354075	2354407	HERVHF_gag	-
Pygathrix_nemaeus_ctg69	2355943	2356286	HERVHF_3LTR	-
Pygathrix_nemaeus_ctg69	9563253	9563704	HERVHF_5LTR	-
Pygathrix_nemaeus_ctg69	9564303	9566097	HERVHF_pol	-
Pygathrix_nemaeus_ctg69	9565879	9566337	HERVHF_pro	-
Pygathrix_nemaeus_ctg69	9566566	9567105	HERVHF_gag	-
Pygathrix_nemaeus_ctg69	9568529	9568980	HERVHF_3LTR	-
Pygathrix_nemaeus_ctg7	3060425	3060830	HERVHF_5LTR	+
Pygathrix_nemaeus_ctg7	3062750	3063481	HERVHF_pro	+
Pygathrix_nemaeus_ctg7	3062915	3065385	HERVHF_pol	+
Pygathrix_nemaeus_ctg7	3065969	3066373	HERVHF_3LTR	+
Pygathrix_nemaeus_ctg96	13417564	13417973	HERVHF_5LTR	+
Pygathrix_nemaeus_ctg96	13420203	13421303	HERVHF_pro	+
Pygathrix_nemaeus_ctg96	13420326	13422523	HERVHF_pol	+
Pygathrix_nemaeus_ctg96	13423796	13424814	HERVHF_env	+
Pygathrix_nemaeus_ctg96	13425067	13425482	HERVHF_3LTR	+

Rhinopithecus_roxellana_CM017351.1	37053370	37053787	HERVHF_5LTR	+
Rhinopithecus_roxellana_CM017351.1	37062369	37062749	HERVHF_pro	+
Rhinopithecus_roxellana_CM017351.1	37063202	37064673	HERVHF_pol	+
Rhinopithecus_roxellana_CM017351.1	37065366	37065779	HERVHF_3LTR	+
Rhinopithecus_roxellana_CM017351.1	166950693	166951126	HERVHF_5LTR	-
Rhinopithecus_roxellana_CM017351.1	166951294	166951656	HERVHF_env	-
Rhinopithecus_roxellana_CM017351.1	166952042	166954042	HERVHF_pol	-
Rhinopithecus_roxellana_CM017351.1	166954471	166954908	HERVHF_gag	-
Rhinopithecus_roxellana_CM017351.1	166956456	166956887	HERVHF_3LTR	-
Rhinopithecus_roxellana_CM017351.1	199982959	199983452	HERVK_5LTR	-
Rhinopithecus_roxellana_CM017351.1	199983524	199984129	HERVK_env	-
Rhinopithecus_roxellana_CM017351.1	199984385	199986311	HERVK_pol	-
Rhinopithecus_roxellana_CM017351.1	199986209	199987135	HERVK_pro	-
Rhinopithecus_roxellana_CM017351.1	199987548	199988201	HERVK_gag	-
Rhinopithecus_roxellana_CM017351.1	199988598	199989070	HERVK_3LTR	-
Rhinopithecus_roxellana_CM017352.1	19640491	19640890	HERVIPADP_5LTR	+
Rhinopithecus_roxellana_CM017352.1	19642811	19643155	HERVIPADP_gag	+
Rhinopithecus_roxellana_CM017352.1	19643834	19646467	HERVIPADP_pol	+
Rhinopithecus_roxellana_CM017352.1	19648550	19649005	HERVIPADP_env	+
Rhinopithecus_roxellana_CM017352.1	19649093	19649501	HERVIPADP_3LTR	+
Rhinopithecus_roxellana_CM017352.1	145959206	145959580	HERVHF_5LTR	+
Rhinopithecus_roxellana_CM017352.1	145961505	145961840	HERVHF_pro	+
Rhinopithecus_roxellana_CM017352.1	145961965	145963824	HERVHF_pol	+
Rhinopithecus_roxellana_CM017352.1	145964712	145965097	HERVHF_3LTR	+
Rhinopithecus_roxellana_CM017353.1	130789883	130790213	HERVHF_5LTR	-
Rhinopithecus_roxellana_CM017353.1	130790820	130792769	HERVHF_pol	-
Rhinopithecus_roxellana_CM017353.1	130793124	130793459	HERVHF_gag	-

Rhinopithecus_roxellana_CM017353.1	130795071	130795413	HERVHF_3LTR	-
Rhinopithecus_roxellana_CM017353.1	153331383	153331729	HERVHF_5LTR	+
Rhinopithecus_roxellana_CM017353.1	153333314	153333982	HERVHF_gag	+
Rhinopithecus_roxellana_CM017353.1	153334016	153334960	HERVHF_pro	+
Rhinopithecus_roxellana_CM017353.1	153334289	153336328	HERVHF_pol	+
Rhinopithecus_roxellana_CM017353.1	153338362	153339123	HERVHF_env	+
Rhinopithecus_roxellana_CM017353.1	153339396	153339722	HERVHF_3LTR	+
Rhinopithecus_roxellana_CM017354.1	24650323	24650719	HERVIPADP_5LTR	-
Rhinopithecus_roxellana_CM017354.1	24650982	24651651	HERVIPADP_env	-
Rhinopithecus_roxellana_CM017354.1	24653443	24656674	HERVIPADP_pol	-
Rhinopithecus_roxellana_CM017354.1	24657295	24657657	HERVIPADP_gag	-
Rhinopithecus_roxellana_CM017354.1	24659249	24659650	HERVIPADP_3LTR	-
Rhinopithecus_roxellana_CM017354.1	24740192	24740588	HERVIPADP_5LTR	-
Rhinopithecus_roxellana_CM017354.1	24740897	24741220	HERVIPADP_env	-
Rhinopithecus_roxellana_CM017354.1	24743137	24746464	HERVIPADP_pol	-
Rhinopithecus_roxellana_CM017354.1	24747304	24747672	HERVIPADP_gag	-
Rhinopithecus_roxellana_CM017354.1	24749134	24749533	HERVIPADP_3LTR	-
Rhinopithecus_roxellana_CM017354.1	24820325	24820666	HERVIPADP_5LTR	-
Rhinopithecus_roxellana_CM017354.1	24820903	24821427	HERVIPADP_env	-
Rhinopithecus_roxellana_CM017354.1	24823268	24826905	HERVIPADP_pol	-
Rhinopithecus_roxellana_CM017354.1	24827424	24827729	HERVIPADP_gag	-
Rhinopithecus_roxellana_CM017354.1	24829279	24829616	HERVIPADP_3LTR	-
Rhinopithecus_roxellana_CM017354.1	24902177	24902598	HERVIPADP_5LTR	+
Rhinopithecus_roxellana_CM017354.1	24905720	24908767	HERVIPADP_pol	+
Rhinopithecus_roxellana_CM017354.1	24909987	24910520	HERVIPADP_env	+
Rhinopithecus_roxellana_CM017354.1	24911158	24911586	HERVIPADP_3LTR	+
Rhinopithecus_roxellana_CM017354.1	142088755	142089134	HERVHF_5LTR	-
Rhinopithecus_roxellana_CM017354.1	142089977	142091931	HERVHF_pol	-
Rhinopithecus_roxellana_CM017354.1	142092099	142092527	HERVHF_pro	-
Rhinopithecus_roxellana_CM017354.1	142094615	142094999	HERVHF_3LTR	-
Rhinopithecus_roxellana_CM017355.1	102052399	102052736	HERVHF_5LTR	+

Rhinopithecus_roxellana_CM017355.1	102054371	102054885	HERVHF_gag	+
Rhinopithecus_roxellana_CM017355.1	102054899	102055306	HERVHF_pro	+
Rhinopithecus_roxellana_CM017355.1	102055321	102056820	HERVHF_pol	+
Rhinopithecus_roxellana_CM017355.1	102057473	102057816	HERVHF_3LTR	+
Rhinopithecus_roxellana_CM017355.1	151120513	151120900	HERVIPADP_5LTR	-
Rhinopithecus_roxellana_CM017355.1	151121366	151122556	HERVIPADP_env	-
Rhinopithecus_roxellana_CM017355.1	151123291	151126546	HERVIPADP_pol	-
Rhinopithecus_roxellana_CM017355.1	151127469	151127783	HERVIPADP_gag	-
Rhinopithecus_roxellana_CM017355.1	151129804	151130181	HERVIPADP_3LTR	-
Rhinopithecus_roxellana_CM017355.1	159902419	159902821	HERVHF_5LTR	-
Rhinopithecus_roxellana_CM017355.1	159903410	159905832	HERVHF_pol	-
Rhinopithecus_roxellana_CM017355.1	159905314	159906030	HERVHF_pro	-
Rhinopithecus_roxellana_CM017355.1	159906087	159906497	HERVHF_gag	-
Rhinopithecus_roxellana_CM017355.1	159907963	159908363	HERVHF_3LTR	-
Rhinopithecus_roxellana_CM017356.1	136003041	136003542	HERVHF_5LTR	-
Rhinopithecus_roxellana_CM017356.1	136004616	136006201	HERVHF_pol	-
Rhinopithecus_roxellana_CM017356.1	136005659	136006315	HERVHF_pro	-
Rhinopithecus_roxellana_CM017356.1	136006506	136006892	HERVHF_gag	-
Rhinopithecus_roxellana_CM017356.1	136008454	136008954	HERVHF_3LTR	-
Rhinopithecus_roxellana_CM017357.1	9496050	9496409	HERVHF_5LTR	+
Rhinopithecus_roxellana_CM017357.1	9497876	9498208	HERVHF_gag	+
Rhinopithecus_roxellana_CM017357.1	9498638	9500859	HERVHF_pol	+
Rhinopithecus_roxellana_CM017357.1	9501446	9501808	HERVHF_3LTR	+
Rhinopithecus_roxellana_CM017357.1	21852345	21852762	HERVHF_5LTR	-
Rhinopithecus_roxellana_CM017357.1	21853511	21855498	HERVHF_pol	-

Rhinopithecus_roxellana_CM017357.1 21854956 21855735 HERVHF_pro -
Rhinopithecus_roxellana_CM017357.1 21857764 21858176 HERVHF_3LTR -
Rhinopithecus_roxellana_CM017357.1 101445362 101445820 HERVHF_5LTR -

Rhinopithecus_roxellana_CM017357.1 101448617 101450838 HERVHF_pol -

Rhinopithecus_roxellana_CM017357.1 101451445 101451777 HERVHF_gag -

Rhinopithecus_roxellana_CM017357.1 101453149 101453600 HERVHF_3LTR -

Rhinopithecus_roxellana_CM017358.1 77075684 77076195 HERVK_5LTR -
Rhinopithecus_roxellana_CM017358.1 77076357 77076683 HERVK_env -
Rhinopithecus_roxellana_CM017358.1 77077026 77078399 HERVK_pol -
Rhinopithecus_roxellana_CM017358.1 77078294 77079235 HERVK_pro -
Rhinopithecus_roxellana_CM017358.1 77080653 77081164 HERVK_3LTR -
Rhinopithecus_roxellana_CM017358.1 87682269 87682617 HERVHF_5LTR -
Rhinopithecus_roxellana_CM017358.1 87685950 87686261 HERVHF_env -
Rhinopithecus_roxellana_CM017358.1 87686542 87688929 HERVHF_pol -
Rhinopithecus_roxellana_CM017358.1 87689350 87689709 HERVHF_gag -
Rhinopithecus_roxellana_CM017358.1 87691248 87691582 HERVHF_3LTR -
Rhinopithecus_roxellana_CM017358.1 125216180 125216685 HERVK_5LTR -

Rhinopithecus_roxellana_CM017358.1 125216766 125217109 HERVK_env -

Rhinopithecus_roxellana_CM017358.1 125217787 125219438 HERVK_pol -

Rhinopithecus_roxellana_CM017358.1 125219377 125220174 HERVK_pro -

Rhinopithecus_roxellana_CM017358.1 125221652 125222155 HERVK_3LTR -

Rhinopithecus_roxellana_CM017359.1 12300365 12300681 HERVHF_5LTR -
Rhinopithecus_roxellana_CM017359.1 12301252 12303888 HERVHF_pol -
Rhinopithecus_roxellana_CM017359.1 12303961 12304392 HERVHF_pro -
Rhinopithecus_roxellana_CM017359.1 12304382 12304945 HERVHF_gag -
Rhinopithecus_roxellana_CM017359.1 12306389 12306702 HERVHF_3LTR -
Rhinopithecus_roxellana_CM017359.1 24633285 24633644 HERVHF_5LTR +
Rhinopithecus_roxellana_CM017359.1 24635053 24635592 HERVHF_gag +
Rhinopithecus_roxellana_CM017359.1 24636051 24636614 HERVHF_pol +
Rhinopithecus_roxellana_CM017359.1 24637292 24637807 HERVHF_env +
Rhinopithecus_roxellana_CM017359.1 24638062 24638422 HERVHF_3LTR +
Rhinopithecus_roxellana_CM017359.1 66897537 66897943 HERVHF_5LTR -
Rhinopithecus_roxellana_CM017359.1 66898098 66898703 HERVHF_env -
Rhinopithecus_roxellana_CM017359.1 66898848 66901059 HERVHF_pol -
Rhinopithecus_roxellana_CM017359.1 66901830 66902255 HERVHF_gag -

Rhinopithecus_roxellana_CM017359.1	66903626	66904040	HERVHF_3LTR	-
Rhinopithecus_roxellana_CM017359.1	126642271	126642581	HERVHF_5LTR	+
Rhinopithecus_roxellana_CM017359.1	126645770	126646504	HERVHF_pro	+
Rhinopithecus_roxellana_CM017359.1	126645980	126647315	HERVHF_pol	+
Rhinopithecus_roxellana_CM017359.1	126647888	126648202	HERVHF_3LTR	+
Rhinopithecus_roxellana_CM017361.1	9027166	9027497	HERVHF_5LTR	-
Rhinopithecus_roxellana_CM017361.1	9028554	9030637	HERVHF_pol	-
Rhinopithecus_roxellana_CM017361.1	9033686	9034011	HERVHF_3LTR	-
Rhinopithecus_roxellana_CM017361.1	62205482	62205907	HERVHF_5LTR	-
Rhinopithecus_roxellana_CM017361.1	62206076	62206471	HERVHF_env	-
Rhinopithecus_roxellana_CM017361.1	62206749	62210279	HERVHF_pol	-
Rhinopithecus_roxellana_CM017361.1	62209982	62210782	HERVHF_pro	-
Rhinopithecus_roxellana_CM017361.1	62210846	62211175	HERVHF_gag	-
Rhinopithecus_roxellana_CM017361.1	62213257	62213685	HERVHF_3LTR	-
Rhinopithecus_roxellana_CM017362.1	94016823	94017346	HERVK_5LTR	-
Rhinopithecus_roxellana_CM017362.1	94018104	94020047	HERVK_pol	-
Rhinopithecus_roxellana_CM017362.1	94019942	94020884	HERVK_pro	-
Rhinopithecus_roxellana_CM017362.1	94021259	94021912	HERVK_gag	-
Rhinopithecus_roxellana_CM017362.1	94022288	94022812	HERVK_3LTR	-
Rhinopithecus_roxellana_CM017362.1	106138084	106138444	HERVHF_5LTR	-
Rhinopithecus_roxellana_CM017362.1	106138671	106139066	HERVHF_env	-
Rhinopithecus_roxellana_CM017362.1	106139112	106141673	HERVHF_pol	-
Rhinopithecus_roxellana_CM017362.1	106143180	106143533	HERVHF_3LTR	-
Rhinopithecus_roxellana_CM017363.1	80905082	80905534	HERVHF_5LTR	+
Rhinopithecus_roxellana_CM017363.1	80907220	80907543	HERVHF_gag	+
Rhinopithecus_roxellana_CM017363.1	80907533	80908390	HERVHF_pro	+
Rhinopithecus_roxellana_CM017363.1	80907737	80909478	HERVHF_pol	+
Rhinopithecus_roxellana_CM017363.1	80910307	80910764	HERVHF_3LTR	+
Rhinopithecus_roxellana_CM017364.1	43303717	43304033	HERVHF_5LTR	-
Rhinopithecus_roxellana_CM017364.1	43306724	43308400	HERVHF_pol	-
Rhinopithecus_roxellana_CM017364.1	43309018	43309317	HERVHF_gag	-
Rhinopithecus_roxellana_CM017364.1	43315327	43315645	HERVHF_3LTR	-
Rhinopithecus_roxellana_CM017364.1	52883002	52883366	HERVHF_5LTR	+
Rhinopithecus_roxellana_CM017364.1	52884056	52885543	HERVHF_gag	+
Rhinopithecus_roxellana_CM017364.1	52885559	52886053	HERVHF_pro	+
Rhinopithecus_roxellana_CM017364.1	52885960	52888006	HERVHF_pol	+

Rhinopithecus_roxellana_CM017364.1	52888880	52889260	HERVHF_3LTR	+
Rhinopithecus_roxellana_CM017364.1	73018178	73018538	HERVHF_5LTR	-
Rhinopithecus_roxellana_CM017364.1	73019210	73020929	HERVHF_pol	-
Rhinopithecus_roxellana_CM017364.1	73020390	73021139	HERVHF_pro	-
Rhinopithecus_roxellana_CM017364.1	73021222	73021545	HERVHF_gag	-
Rhinopithecus_roxellana_CM017364.1	73023848	73024189	HERVHF_3LTR	-
Rhinopithecus_roxellana_CM017364.1	134810665	134811134	HERVK_5LTR	+
Rhinopithecus_roxellana_CM017364.1	134811656	134812132	HERVK_gag	+
Rhinopithecus_roxellana_CM017364.1	134812613	134813689	HERVK_pro	+
Rhinopithecus_roxellana_CM017364.1	134813351	134815334	HERVK_pol	+
Rhinopithecus_roxellana_CM017364.1	134816562	134817007	HERVK_3LTR	+
Rhinopithecus_roxellana_CM017365.1	24854630	24855030	HERVHF_5LTR	-
Rhinopithecus_roxellana_CM017365.1	24855640	24858020	HERVHF_pol	-
Rhinopithecus_roxellana_CM017365.1	24859394	24859784	HERVHF_3LTR	-
Rhinopithecus_roxellana_CM017366.1	82577437	82577879	HERVHF_5LTR	-
Rhinopithecus_roxellana_CM017366.1	82578433	82580464	HERVHF_pol	-
Rhinopithecus_roxellana_CM017366.1	82580395	82580916	HERVHF_pro	-
Rhinopithecus_roxellana_CM017366.1	82581319	82581693	HERVHF_gag	-
Rhinopithecus_roxellana_CM017366.1	82582964	82583401	HERVHF_3LTR	-
Rhinopithecus_roxellana_CM017366.1	86961154	86961488	HERVHF_5LTR	+
Rhinopithecus_roxellana_CM017366.1	86963568	86964140	HERVHF_pro	+
Rhinopithecus_roxellana_CM017366.1	86964288	86967329	HERVHF_pol	+
Rhinopithecus_roxellana_CM017366.1	86968368	86968796	HERVHF_env	+
Rhinopithecus_roxellana_CM017366.1	86969599	86969930	HERVHF_3LTR	+
Rhinopithecus_roxellana_CM017367.1	39471830	39472205	HERVHF_5LTR	+
Rhinopithecus_roxellana_CM017367.1	39473103	39475026	HERVHF_pol	+
Rhinopithecus_roxellana_CM017367.1	39475687	39476067	HERVHF_3LTR	+
Rhinopithecus_roxellana_CM017367.1	100255651	100256167	HERVHF_5LTR	+
Rhinopithecus_roxellana_CM017367.1	100257364	100258298	HERVHF_gag	+
Rhinopithecus_roxellana_CM017367.1	100260176	100260799	HERVHF_pro	+
Rhinopithecus_roxellana_CM017367.1	100260224	100262814	HERVHF_pol	+
Rhinopithecus_roxellana_CM017367.1	100264623	100265054	HERVHF_env	+
Rhinopithecus_roxellana_CM017367.1	100265600	100266122	HERVHF_3LTR	+

Rhinopithecus_roxellana_CM017367.1	111245993	111246358	HERVHF_5LTR	+
Rhinopithecus_roxellana_CM017367.1	111251156	111251557	HERVHF_gag	+
Rhinopithecus_roxellana_CM017367.1	111251631	111252695	HERVHF_pro	+
Rhinopithecus_roxellana_CM017367.1	111252153	111254388	HERVHF_pol	+
Rhinopithecus_roxellana_CM017367.1	111254827	111255187	HERVHF_3LTR	+
Rhinopithecus_roxellana_CM017368.1	43723454	43723812	HERVHF_5LTR	+
Rhinopithecus_roxellana_CM017368.1	43725359	43725700	HERVHF_gag	+
Rhinopithecus_roxellana_CM017368.1	43725849	43726598	HERVHF_pro	+
Rhinopithecus_roxellana_CM017368.1	43726038	43727568	HERVHF_pol	+
Rhinopithecus_roxellana_CM017368.1	43728715	43729073	HERVHF_3LTR	+
Rhinopithecus_roxellana_CM017369.1	20543195	20543519	HERVK_5LTR	-
Rhinopithecus_roxellana_CM017369.1	20544369	20545640	HERVK_pol	-
Rhinopithecus_roxellana_CM017369.1	20545535	20546476	HERVK_pro	-
Rhinopithecus_roxellana_CM017369.1	20547532	20547856	HERVK_3LTR	-
Rhinopithecus_roxellana_CM017369.1	41016707	41017121	HERVHF_5LTR	-
Rhinopithecus_roxellana_CM017369.1	41017845	41018971	HERVHF_env	-
Rhinopithecus_roxellana_CM017369.1	41020352	41021359	HERVHF_pol	-
Rhinopithecus_roxellana_CM017369.1	41021137	41022351	HERVHF_pro	-
Rhinopithecus_roxellana_CM017369.1	41024586	41024996	HERVHF_3LTR	-
Rhinopithecus_roxellana_CM017371.1	16215279	16215730	HERVHF_5LTR	+
Rhinopithecus_roxellana_CM017371.1	16218266	16219759	HERVHF_pol	+
Rhinopithecus_roxellana_CM017371.1	16220446	16220897	HERVHF_3LTR	+
Rhinopithecus_roxellana_CM017372.1	8724440	8725085	HERVK_5LTR	+
Rhinopithecus_roxellana_CM017372.1	8725973	8727214	HERVK_gag	+
Rhinopithecus_roxellana_CM017372.1	8727096	8727686	HERVK_pro	+
Rhinopithecus_roxellana_CM017372.1	8727942	8730517	HERVK_pol	+
Rhinopithecus_roxellana_CM017372.1	8731377	8732288	HERVK_env	+
Rhinopithecus_roxellana_CM017372.1	8732793	8733436	HERVK_3LTR	+
Rhinopithecus_roxellana_VMRL01001680.1	27861	28450	HERVHF_5LTR	-
Rhinopithecus_roxellana_VMRL01001680.1	33276	33722	HERVHF_env	-
Rhinopithecus_roxellana_VMRL01001680.1	35511	37395	HERVHF_pol	-
Rhinopithecus_roxellana_VMRL01001680.1	41626	42204	HERVHF_3LTR	-
Rhinopithecus_strykeri_ctg10	19645487	19645886	HERVIPADP_5LTR	+
Rhinopithecus_strykeri_ctg10	19647804	19648148	HERVIPADP_gag	+
Rhinopithecus_strykeri_ctg10	19648824	19651337	HERVIPADP_pol	+
Rhinopithecus_strykeri_ctg10	19653543	19653998	HERVIPADP_env	+
Rhinopithecus_strykeri_ctg10	19654084	19654492	HERVIPADP_3LTR	+

Rhinopithecus_strykeri_ctg10414790009 14790315 HERVHF_5LTR +
Rhinopithecus_strykeri_ctg10414793089 14793469 HERVHF_gag +
Rhinopithecus_strykeri_ctg10414793583 14794347 HERVHF_pro +
Rhinopithecus_strykeri_ctg10414793817 14795531 HERVHF_pol +
Rhinopithecus_strykeri_ctg10414798490 14798796 HERVHF_3LTR +
Rhinopithecus_strykeri_ctg11054169 54581 HERVIPADP_5LTR +
Rhinopithecus_strykeri_ctg11057436 61059 HERVIPADP_pol +
Rhinopithecus_strykeri_ctg11062252 62785 HERVIPADP_env +
Rhinopithecus_strykeri_ctg11063439 63856 HERVIPADP_3LTR +
Rhinopithecus_strykeri_ctg12 4528512 4528865 HERVHF_5LTR +
Rhinopithecus_strykeri_ctg12 4530394 4530726 HERVHF_gag +
Rhinopithecus_strykeri_ctg12 4531059 4533481 HERVHF_pol +
Rhinopithecus_strykeri_ctg12 4534105 4534459 HERVHF_3LTR +
Rhinopithecus_strykeri_ctg12 13363594 13363970 HERVIPADP_5LTR +
Rhinopithecus_strykeri_ctg12 13365993 13366307 HERVIPADP_gag +
Rhinopithecus_strykeri_ctg12 13367265 13370484 HERVIPADP_pol +
Rhinopithecus_strykeri_ctg12 13371774 13372316 HERVIPADP_env +
Rhinopithecus_strykeri_ctg12 13372864 13373253 HERVIPADP_3LTR +
Rhinopithecus_strykeri_ctg12111087871 11088195 HERVK_5LTR +
Rhinopithecus_strykeri_ctg12111089290 11090204 HERVK_pro +
Rhinopithecus_strykeri_ctg12111090099 11091374 HERVK_pol +
Rhinopithecus_strykeri_ctg12111092221 11092545 HERVK_3LTR +
Rhinopithecus_strykeri_ctg1398171654 8171988 HUERSP_5LTR -
Rhinopithecus_strykeri_ctg1398177915 8179051 HUERSP_pol -
Rhinopithecus_strykeri_ctg1398182242 8182579 HUERSP_3LTR -
Rhinopithecus_strykeri_ctg1532100665 2101137 HERVK_5LTR +
Rhinopithecus_strykeri_ctg1532101549 2102202 HERVK_gag +
Rhinopithecus_strykeri_ctg1532102615 2103544 HERVK_pro +
Rhinopithecus_strykeri_ctg1532103439 2105365 HERVK_pol +
Rhinopithecus_strykeri_ctg1532105621 2106226 HERVK_env +
Rhinopithecus_strykeri_ctg1532106296 2106789 HERVK_3LTR +
Rhinopithecus_strykeri_ctg17 2230803 2231314 HERVK_5LTR +
Rhinopithecus_strykeri_ctg17 2232739 2233674 HERVK_pro +
Rhinopithecus_strykeri_ctg17 2233572 2234450 HERVK_pol +
Rhinopithecus_strykeri_ctg17 2235256 2235618 HERVK_env +
Rhinopithecus_strykeri_ctg17 2235772 2236283 HERVK_3LTR +
Rhinopithecus_strykeri_ctg187508685 508999 HERVIPADP_5LTR +
Rhinopithecus_strykeri_ctg187510830 511243 HERVIPADP_gag +
Rhinopithecus_strykeri_ctg187512031 514890 HERVIPADP_pol +
Rhinopithecus_strykeri_ctg187516118 516543 HERVIPADP_env +
Rhinopithecus_strykeri_ctg187517125 517432 HERVIPADP_3LTR +
Rhinopithecus_strykeri_ctg2861589829 1590131 HERVK_5LTR -
Rhinopithecus_strykeri_ctg2861590379 1590948 HERVK_env -
Rhinopithecus_strykeri_ctg2861590974 1591801 HERVK_pol -

Rhinopithecus_strykeri_ctg286	1591696	1592625	HERVK_pro	-
Rhinopithecus_strykeri_ctg286	1594068	1594373	HERVK_3LTR	-
Rhinopithecus_strykeri_ctg291	22411	22828	HERVHF_5LTR	-
Rhinopithecus_strykeri_ctg291	23768	25602	HERVHF_pol	-
Rhinopithecus_strykeri_ctg291	25021	25800	HERVHF_pro	-
Rhinopithecus_strykeri_ctg291	28415	28828	HERVHF_3LTR	-
Rhinopithecus_strykeri_ctg43	10408784	10409237	HERVHF_5LTR	+
Rhinopithecus_strykeri_ctg43	10410181	10412348	HERVHF_gag	+
Rhinopithecus_strykeri_ctg43	10412820	10414349	HERVHF_pol	+
Rhinopithecus_strykeri_ctg43	10415199	10415653	HERVHF_3LTR	+
Rhinopithecus_strykeri_ctg47	12017831	12018152	HERVIPADP_5LTR	-
Rhinopithecus_strykeri_ctg47	12018381	12019330	HERVIPADP_env	-
Rhinopithecus_strykeri_ctg47	12020363	12024057	HERVIPADP_pol	-
Rhinopithecus_strykeri_ctg47	12024543	12024860	HERVIPADP_gag	-
Rhinopithecus_strykeri_ctg47	12026360	12026687	HERVIPADP_3LTR	-
Rhinopithecus_strykeri_ctg6	20338313	20338770	HERVHF_5LTR	-
Rhinopithecus_strykeri_ctg6	20339559	20341975	HERVHF_pol	-
Rhinopithecus_strykeri_ctg6	20341412	20342212	HERVHF_pro	-
Rhinopithecus_strykeri_ctg6	20344307	20344762	HERVHF_3LTR	-
Rhinopithecus_strykeri_ctg7	35211119	35211550	HERVHF_5LTR	+
Rhinopithecus_strykeri_ctg7	35213100	35213540	HERVHF_gag	+
Rhinopithecus_strykeri_ctg7	35213966	35215964	HERVHF_pol	+
Rhinopithecus_strykeri_ctg7	35216327	35216710	HERVHF_env	+
Rhinopithecus_strykeri_ctg7	35216876	35217309	HERVHF_3LTR	+
Rhinopithecus_strykeri_ctg72	1420067	1420427	HERVHF_5LTR	-
Rhinopithecus_strykeri_ctg72	1421011	1422959	HERVHF_pol	-
Rhinopithecus_strykeri_ctg72	1422579	1423205	HERVHF_pro	-
Rhinopithecus_strykeri_ctg72	1423658	1424617	HERVHF_gag	-
Rhinopithecus_strykeri_ctg72	1428864	1429229	HERVHF_3LTR	-
Rhinopithecus_strykeri_ctg9	14146116	14146527	HERVHF_5LTR	-
Rhinopithecus_strykeri_ctg9	14147160	14149676	HERVHF_pol	-
Rhinopithecus_strykeri_ctg9	14149017	14149718	HERVHF_pro	-
Rhinopithecus_strykeri_ctg9	14149885	14150283	HERVHF_gag	-
Rhinopithecus_strykeri_ctg9	14151615	14152027	HERVHF_3LTR	-
Saguinus_midas_LG02	71916510	71916833	HUERSP_5LTR	+
Saguinus_midas_LG02	71919127	71919558	HUERSP_gag	+
Saguinus_midas_LG02	71920084	71920716	HUERSP_pro	+
Saguinus_midas_LG02	71920135	71922669	HUERSP_pol	+
Saguinus_midas_LG02	71923503	71923907	HUERSP_env	+
Saguinus_midas_LG02	71924435	71924758	HUERSP_3LTR	+
Saguinus_midas_LG02	139088051	139088399	HERVHF_5LTR	+
Saguinus_midas_LG02	139090100	139090513	HERVHF_gag	+
Saguinus_midas_LG02	139091389	139092408	HERVHF_pro	+
Saguinus_midas_LG02	139091767	139094333	HERVHF_pol	+

Saguinus_midas_LG02	139095784	139096542	HERVHF_env	+
Saguinus_midas_LG02	139097049	139097415	HERVHF_3LTR	+
Saguinus_midas_LG03	38985829	38986385	HERVHF_5LTR	-
Saguinus_midas_LG03	38988434	38990856	HERVHF_pol	-
Saguinus_midas_LG03	38991560	38992549	HERVHF_gag	-
Saguinus_midas_LG03	38993560	38994107	HERVHF_3LTR	-
Saguinus_midas_LG06	46422988	46423408	HERVHF_5LTR	-
Saguinus_midas_LG06	46426382	46428188	HERVHF_pol	-
Saguinus_midas_LG06	46427863	46428414	HERVHF_pro	-
Saguinus_midas_LG06	46428775	46430254	HERVHF_gag	-
Saguinus_midas_LG06	46430670	46431082	HERVHF_3LTR	-
Saguinus_midas_LG08	65080925	65081250	HERVHF_5LTR	+
Saguinus_midas_LG08	65082955	65083377	HERVHF_pro	+
Saguinus_midas_LG08	65083418	65084724	HERVHF_pol	+
Saguinus_midas_LG08	65086299	65086898	HERVHF_env	+
Saguinus_midas_LG08	65087121	65087444	HERVHF_3LTR	+
Saguinus_midas_LG09	88042686	88043122	HERVHF_5LTR	+
Saguinus_midas_LG09	88044356	88044748	HERVHF_gag	+
Saguinus_midas_LG09	88045536	88045981	HERVHF_pol	+
Saguinus_midas_LG09	88046648	88047067	HERVHF_3LTR	+
Saguinus_midas_LG10	40013594	40013976	HERVHF_5LTR	+
Saguinus_midas_LG10	40016142	40018277	HERVHF_pol	+
Saguinus_midas_LG10	40019309	40019992	HERVHF_env	+
Saguinus_midas_LG10	40020370	40020741	HERVHF_3LTR	+
Saguinus_midas_LG11	23650294	23650652	HERVHF_5LTR	+
Saguinus_midas_LG11	23651577	23652164	HERVHF_gag	+
Saguinus_midas_LG11	23653247	23654098	HERVHF_pol	+
Saguinus_midas_LG11	23657929	23658286	HERVHF_3LTR	+
Saguinus_midas_LG11	126306948	126307292	HERVHF_5LTR	-
Saguinus_midas_LG11	126310312	126312165	HERVHF_pol	-
Saguinus_midas_LG11	126314615	126314971	HERVHF_3LTR	-
Saguinus_midas_LG19	39682326	39682643	HERVHF_5LTR	+
Saguinus_midas_LG19	39684065	39684586	HERVHF_gag	+
Saguinus_midas_LG19	39685018	39687876	HERVHF_pol	+
Saguinus_midas_LG19	39685018	39685614	HERVHF_pro	+
Saguinus_midas_LG19	39690069	39690386	HERVHF_3LTR	+
Saguinus_midas_LG20	26428222	26428650	HERVHF_5LTR	-
Saguinus_midas_LG20	26430428	26433467	HERVHF_pol	-
Saguinus_midas_LG20	26433099	26433437	HERVHF_pro	-
Saguinus_midas_LG20	26435901	26436319	HERVHF_3LTR	-
Sapajus_apella_ctg70	6148497	6148922	HERVHF_5LTR	-
Sapajus_apella_ctg70	6150357	6151376	HERVHF_pol	-
Sapajus_apella_ctg70	6152122	6152775	HERVHF_gag	-
Sapajus_apella_ctg70	6154384	6154811	HERVHF_3LTR	-

Symphalangus_syndactylus_tarseq_0	13518898	13519207	HERVHF_5LTR	-
Symphalangus_syndactylus_tarseq_0	13522442	13523296	HERVHF_pol	-
Symphalangus_syndactylus_tarseq_0	13525778	13526088	HERVHF_3LTR	-
Symphalangus_syndactylus_tarseq_101	5859844	5860253	HERVHF_5LTR	+
Symphalangus_syndactylus_tarseq_101	5862769	5864644	HERVHF_pol	+
Symphalangus_syndactylus_tarseq_101	5865079	5865490	HERVHF_3LTR	+
Symphalangus_syndactylus_tarseq_12	5637548	5637877	HERVHF_5LTR	+
Symphalangus_syndactylus_tarseq_12	5639646	5640179	HERVHF_gag	+
Symphalangus_syndactylus_tarseq_12	5640272	5640691	HERVHF_pro	+
Symphalangus_syndactylus_tarseq_12	5640517	5643060	HERVHF_pol	+
Symphalangus_syndactylus_tarseq_12	5643722	5644066	HERVHF_3LTR	+
Symphalangus_syndactylus_tarseq_1272	20257892	20258265	HERVHF_5LTR	+
Symphalangus_syndactylus_tarseq_1272	20260004	20260312	HERVHF_gag	+
Symphalangus_syndactylus_tarseq_1272	20260316	20261155	HERVHF_pro	+
Symphalangus_syndactylus_tarseq_1272	20260520	20261959	HERVHF_pol	+
Symphalangus_syndactylus_tarseq_1272	20263142	20263515	HERVHF_3LTR	+
Symphalangus_syndactylus_tarseq_1273	9619974	9620387	HERVHF_5LTR	-
Symphalangus_syndactylus_tarseq_1273	9620805	9622948	HERVHF_pol	-
Symphalangus_syndactylus_tarseq_1273	9622337	9623113	HERVHF_pro	-
Symphalangus_syndactylus_tarseq_1273	9625265	9625681	HERVHF_3LTR	-
Symphalangus_syndactylus_tarseq_1274	5037850	5038263	HERVHF_5LTR	-
Symphalangus_syndactylus_tarseq_1274	5039035	5041871	HERVHF_pol	-
Symphalangus_syndactylus_tarseq_1274	5041332	5041919	HERVHF_pro	-
Symphalangus_syndactylus_tarseq_1274	5042164	5042493	HERVHF_gag	-
Symphalangus_syndactylus_tarseq_1274	5044181	5044598	HERVHF_3LTR	-
Symphalangus_syndactylus_tarseq_1278	11350225	11350657	HERVHF_5LTR	+
Symphalangus_syndactylus_tarseq_1278	11352590	11353417	HERVHF_pro	+
Symphalangus_syndactylus_tarseq_1278	11353055	11353594	HERVHF_pol	+
Symphalangus_syndactylus_tarseq_1278	11355697	11356128	HERVHF_3LTR	+
Symphalangus_syndactylus_tarseq_1280	5604859	5605309	HERVHF_5LTR	-
Symphalangus_syndactylus_tarseq_1280	5605478	5606326	HERVHF_env	-
Symphalangus_syndactylus_tarseq_1280	5607141	5607797	HERVHF_pol	-
Symphalangus_syndactylus_tarseq_1280	5607162	5607932	HERVHF_pro	-
Symphalangus_syndactylus_tarseq_1280	5608104	5608532	HERVHF_gag	-
Symphalangus_syndactylus_tarseq_1280	5610045	5610497	HERVHF_3LTR	-
Symphalangus_syndactylus_tarseq_1287	1158436	1158849	HERVHF_5LTR	-
Symphalangus_syndactylus_tarseq_1287	1159302	1161216	HERVHF_pol	-
Symphalangus_syndactylus_tarseq_1287	1161053	1161643	HERVHF_pro	-
Symphalangus_syndactylus_tarseq_1287	1163733	1164147	HERVHF_3LTR	-
Symphalangus_syndactylus_tarseq_1288	866193	866636	HERVHF_5LTR	+
Symphalangus_syndactylus_tarseq_1288	874219	874902	HERVHF_gag	+
Symphalangus_syndactylus_tarseq_1288	875151	875489	HERVHF_pro	+
Symphalangus_syndactylus_tarseq_1288	875522	877274	HERVHF_pol	+
Symphalangus_syndactylus_tarseq_1288	879281	879727	HERVHF_3LTR	+

Symphalangus_syndactylus_tarseq_132	3239291	3239762	HERVHF_5LTR	-
Symphalangus_syndactylus_tarseq_132	3240182	3241905	HERVHF_pol	-
Symphalangus_syndactylus_tarseq_132	3241841	3242479	HERVHF_pro	-
Symphalangus_syndactylus_tarseq_132	3244483	3244960	HERVHF_3LTR	-
Symphalangus_syndactylus_tarseq_17	10246148	10246469	HERVHF_5LTR	+
Symphalangus_syndactylus_tarseq_17	10248483	10249214	HERVHF_pro	+
Symphalangus_syndactylus_tarseq_17	10248600	10250484	HERVHF_pol	+
Symphalangus_syndactylus_tarseq_17	10251240	10251569	HERVHF_3LTR	+
Symphalangus_syndactylus_tarseq_3	29354817	29355230	HERVHF_5LTR	+
Symphalangus_syndactylus_tarseq_3	29356802	29357128	HERVHF_gag	+
Symphalangus_syndactylus_tarseq_3	29357182	29357994	HERVHF_pro	+
Symphalangus_syndactylus_tarseq_3	29357353	29359621	HERVHF_pol	+
Symphalangus_syndactylus_tarseq_3	29360023	29360436	HERVHF_3LTR	+
Symphalangus_syndactylus_tarseq_43	26722756	26723187	HERVHF_5LTR	+
Symphalangus_syndactylus_tarseq_43	26724741	26725040	HERVHF_gag	+
Symphalangus_syndactylus_tarseq_43	26725157	26725957	HERVHF_pro	+
Symphalangus_syndactylus_tarseq_43	26725583	26727492	HERVHF_pol	+
Symphalangus_syndactylus_tarseq_43	26727946	26728377	HERVHF_3LTR	+
Symphalangus_syndactylus_tarseq_619	764916	765266	HERVHF_5LTR	+
Symphalangus_syndactylus_tarseq_619	766839	767159	HERVHF_gag	+
Symphalangus_syndactylus_tarseq_619	767270	768055	HERVHF_pro	+
Symphalangus_syndactylus_tarseq_619	767807	769636	HERVHF_pol	+
Symphalangus_syndactylus_tarseq_619	770163	770498	HERVHF_3LTR	+
Symphalangus_syndactylus_tarseq_72	32122859	32123259	HERVHF_5LTR	-
Symphalangus_syndactylus_tarseq_72	32124045	32125822	HERVHF_pol	-
Symphalangus_syndactylus_tarseq_72	32125250	32126083	HERVHF_pro	-
Symphalangus_syndactylus_tarseq_72	32126230	32126685	HERVHF_gag	-
Symphalangus_syndactylus_tarseq_72	32128066	32128452	HERVHF_3LTR	-
Symphalangus_syndactylus_tarseq_80	24593444	24593889	HERVHF_5LTR	+
Symphalangus_syndactylus_tarseq_80	24595940	24596758	HERVHF_pro	+
Symphalangus_syndactylus_tarseq_80	24596186	24598271	HERVHF_pol	+
Symphalangus_syndactylus_tarseq_80	24598691	24599133	HERVHF_3LTR	+
Theropithecus_gelada_CM009950.1	7728166	7728540	HERVHF_5LTR	-
Theropithecus_gelada_CM009950.1	7728755	7729099	HERVHF_env	-
Theropithecus_gelada_CM009950.1	7730157	7732392	HERVHF_pol	-
Theropithecus_gelada_CM009950.1	7732116	7732796	HERVHF_pro	-
Theropithecus_gelada_CM009950.1	7733120	7733518	HERVHF_gag	-
Theropithecus_gelada_CM009950.1	7734978	7735342	HERVHF_3LTR	-
Theropithecus_gelada_CM009950.1	41410562	41410878	HERVIPADP_5LTR	-
Theropithecus_gelada_CM009950.1	41411267	41411608	HERVIPADP_env	-
Theropithecus_gelada_CM009950.1	41412977	41416009	HERVIPADP_pol	-
Theropithecus_gelada_CM009950.1	41416818	41417135	HERVIPADP_gag	-
Theropithecus_gelada_CM009950.1	41424887	41425206	HERVIPADP_3LTR	-
Theropithecus_gelada_CM009950.1	57725622	57726011	HERVHF_5LTR	+

Theropithecus_gelada_CM009950.1	57727451	57727774	HERVHF_gag	+
Theropithecus_gelada_CM009950.1	57727971	57729441	HERVHF_pol	+
Theropithecus_gelada_CM009950.1	57729882	57730229	HERVHF_env	+
Theropithecus_gelada_CM009950.1	57730395	57730779	HERVHF_3LTR	+
Theropithecus_gelada_CM009950.1	98233908	98234333	HERVHF_5LTR	+
Theropithecus_gelada_CM009950.1	98236466	98236819	HERVHF_gag	+
Theropithecus_gelada_CM009950.1	98236951	98237754	HERVHF_pro	+
Theropithecus_gelada_CM009950.1	98237191	98239659	HERVHF_pol	+
Theropithecus_gelada_CM009950.1	98240102	98240467	HERVHF_env	+
Theropithecus_gelada_CM009950.1	98240631	98241077	HERVHF_3LTR	+
Theropithecus_gelada_CM009950.1	100600973	100601300	HERVHF_5LTR	-
Theropithecus_gelada_CM009950.1	100601744	100603673	HERVHF_pol	-
Theropithecus_gelada_CM009950.1	100606728	100607054	HERVHF_3LTR	-
Theropithecus_gelada_CM009950.1	122869945	122870399	HERVHF_5LTR	+
Theropithecus_gelada_CM009950.1	122871086	122872320	HERVHF_gag	+
Theropithecus_gelada_CM009950.1	122873050	122873463	HERVHF_pro	+
Theropithecus_gelada_CM009950.1	122875830	122879159	HERVHF_pol	+
Theropithecus_gelada_CM009950.1	122880570	122880980	HERVHF_env	+
Theropithecus_gelada_CM009950.1	122881142	122881605	HERVHF_3LTR	+
Theropithecus_gelada_CM009950.1	126864665	126865119	HERVHF_5LTR	-
Theropithecus_gelada_CM009950.1	126865991	126867546	HERVHF_pol	-
Theropithecus_gelada_CM009950.1	126867208	126867894	HERVHF_pro	-
Theropithecus_gelada_CM009950.1	126868000	126868455	HERVHF_gag	-
Theropithecus_gelada_CM009950.1	126869898	126870346	HERVHF_3LTR	-
Theropithecus_gelada_CM009950.1	132663389	132663746	HERVHF_5LTR	+
Theropithecus_gelada_CM009950.1	132665211	132665555	HERVHF_gag	+
Theropithecus_gelada_CM009950.1	132665545	132666138	HERVHF_pro	+
Theropithecus_gelada_CM009950.1	132665845	132668099	HERVHF_pol	+
Theropithecus_gelada_CM009950.1	132668789	132669391	HERVHF_env	+
Theropithecus_gelada_CM009950.1	132669557	132669919	HERVHF_3LTR	+
Theropithecus_gelada_CM009950.1	143562450	143562926	HERVHF_5LTR	-
Theropithecus_gelada_CM009950.1	143565211	143570466	HERVHF_pol	-
Theropithecus_gelada_CM009950.1	143569855	143570601	HERVHF_pro	-
Theropithecus_gelada_CM009950.1	143571331	143571795	HERVHF_gag	-
Theropithecus_gelada_CM009950.1	143573144	143573601	HERVHF_3LTR	-

Theropithecus_gelada_CM009950.1 212391696 212392022 HERVHF_5LTR -

Theropithecus_gelada_CM009950.1 212392714 212393385 HERVHF_pol -
Theropithecus_gelada_CM009950.1 212395688 212396015 HERVHF_3LTR -

Theropithecus_gelada_CM009951.1 16354273 16354592 HERVHF_5LTR +
Theropithecus_gelada_CM009951.1 16360412 16360753 HERVHF_gag +
Theropithecus_gelada_CM009951.1 16361042 16362981 HERVHF_pol +
Theropithecus_gelada_CM009951.1 16365431 16365746 HERVHF_3LTR +
Theropithecus_gelada_CM009951.1 91470257 91470579 HERVHF_5LTR +
Theropithecus_gelada_CM009951.1 91471040 91471934 HERVHF_gag +
Theropithecus_gelada_CM009951.1 91471938 91473086 HERVHF_pro +
Theropithecus_gelada_CM009951.1 91472154 91474625 HERVHF_pol +
Theropithecus_gelada_CM009951.1 91484313 91484619 HERVHF_3LTR +
Theropithecus_gelada_CM009951.1 104674653 104675046 HERVHF_5LTR -

Theropithecus_gelada_CM009951.1 104675321 104675857 HERVHF_pol -
Theropithecus_gelada_CM009951.1 104675345 104675941 HERVHF_pro -
Theropithecus_gelada_CM009951.1 104678647 104679042 HERVHF_3LTR -

Theropithecus_gelada_CM009952.1 10021776 10022172 HERVHF_5LTR +
Theropithecus_gelada_CM009952.1 10025004 10027063 HERVHF_pol +
Theropithecus_gelada_CM009952.1 10027505 10027903 HERVHF_3LTR +
Theropithecus_gelada_CM009952.1 117049151 117049562 HERVHF_5LTR -

Theropithecus_gelada_CM009952.1 117050654 117052974 HERVHF_pol -
Theropithecus_gelada_CM009952.1 117052339 117053040 HERVHF_pro -
Theropithecus_gelada_CM009952.1 117055432 117055844 HERVHF_3LTR -

Theropithecus_gelada_CM009952.1 125386588 125387078 HERVK_5LTR -
Theropithecus_gelada_CM009952.1 125387179 125387517 HERVK_env -
Theropithecus_gelada_CM009952.1 125388192 125389934 HERVK_pol -
Theropithecus_gelada_CM009952.1 125389835 125390644 HERVK_pro -
Theropithecus_gelada_CM009952.1 125391526 125392003 HERVK_3LTR -
Theropithecus_gelada_CM009953.1 33893778 33894209 HERVIPADP_5LTR +
Theropithecus_gelada_CM009953.1 33897376 33900422 HERVIPADP_pol +
Theropithecus_gelada_CM009953.1 33901458 33902045 HERVIPADP_env +
Theropithecus_gelada_CM009953.1 33902765 33903210 HERVIPADP_3LTR +
Theropithecus_gelada_CM009953.1 66944180 66944656 HERVHF_5LTR -
Theropithecus_gelada_CM009953.1 66944843 66945537 HERVHF_env -
Theropithecus_gelada_CM009953.1 66947280 66949786 HERVHF_pol -
Theropithecus_gelada_CM009953.1 66950669 66951439 HERVHF_gag -
Theropithecus_gelada_CM009953.1 66952245 66952715 HERVHF_3LTR -

Theropithecus_gelada_CM009953.1 140240846 140241259 HERVHF_5LTR -

Theropithecus_gelada_CM009953.1 140241732 140243468 HERVHF_pol -

Theropithecus_gelada_CM009953.1 140243154 140243936 HERVHF_pro -

Theropithecus_gelada_CM009953.1 140245989 140246398 HERVHF_3LTR -

Theropithecus_gelada_CM009953.1 148105410 148105829 HERVHF_5LTR +

Theropithecus_gelada_CM009953.1 148107311 148107619 HERVHF_gag +

Theropithecus_gelada_CM009953.1 148107611 148108496 HERVHF_pro +

Theropithecus_gelada_CM009953.1 148107897 148109753 HERVHF_pol +

Theropithecus_gelada_CM009953.1 148110431 148110855 HERVHF_3LTR +

Theropithecus_gelada_CM009954.1 10625991 10626436 HERVHF_5LTR -

Theropithecus_gelada_CM009954.1 10627497 10628992 HERVHF_pol -

Theropithecus_gelada_CM009954.1 10628480 10629232 HERVHF_pro -

Theropithecus_gelada_CM009954.1 10629242 10629559 HERVHF_gag -

Theropithecus_gelada_CM009954.1 10631155 10631605 HERVHF_3LTR -

Theropithecus_gelada_CM009954.1 50618131 50618538 HERVHF_5LTR +

Theropithecus_gelada_CM009954.1 50619914 50620300 HERVHF_gag +

Theropithecus_gelada_CM009954.1 50620915 50621712 HERVHF_pro +

Theropithecus_gelada_CM009954.1 50621032 50623266 HERVHF_pol +

Theropithecus_gelada_CM009954.1 50624039 50624449 HERVHF_3LTR +

Theropithecus_gelada_CM009954.1 62342556 62343009 HERVHF_5LTR -

Theropithecus_gelada_CM009954.1 62343618 62345448 HERVHF_pol -

Theropithecus_gelada_CM009954.1 62346678 62347131 HERVHF_3LTR -

Theropithecus_gelada_CM009954.1 101466854 101467190 HERVHF_5LTR +

Theropithecus_gelada_CM009954.1 101468735 101469067 HERVHF_gag +

Theropithecus_gelada_CM009954.1 101469983 101472447 HERVHF_pol +

Theropithecus_gelada_CM009954.1 101473011 101473348 HERVHF_3LTR +

Theropithecus_gelada_CM009954.1 151001645 151002043 HERVHF_5LTR +

Theropithecus_gelada_CM009954.1 151003369 151003719 HERVHF_gag +

Theropithecus_gelada_CM009954.1 151003886 151004299 HERVHF_pro +

Theropithecus_gelada_CM009954.1 151004343 151006184 HERVHF_pol +

Theropithecus_gelada_CM009954.1 151007882 151008223 HERVHF_env +

Theropithecus_gelada_CM009954.1 151008389 151008779 HERVHF_3LTR +

Theropithecus_gelada_CM009954.1 159340919 159341227 HERVHF_5LTR +

Theropithecus_gelada_CM009954.1 159343193 159343645 HERVHF_gag +

Theropithecus_gelada_CM009954.1 159344076 159345850 HERVHF_pol +

Theropithecus_gelada_CM009954.1 159346638 159346950 HERVHF_3LTR +

Theropithecus_gelada_CM009954.1 183113597 183113964 HERVHF_5LTR -

Theropithecus_gelada_CM009954.1 183114411 183116791 HERVHF_pol -
Theropithecus_gelada_CM009954.1 183116120 183116986 HERVHF_pro -
Theropithecus_gelada_CM009954.1 183117042 183117383 HERVHF_gag -
Theropithecus_gelada_CM009954.1 183118831 183119198 HERVHF_3LTR -

Theropithecus_gelada_CM009955.1 70273329 70273648 HSERVIII_5LTR +
Theropithecus_gelada_CM009955.1 70275658 70278467 HSERVIII_pol +
Theropithecus_gelada_CM009955.1 70279319 70279632 HSERVIII_3LTR +
Theropithecus_gelada_CM009955.1 83407512 83407957 HERVHF_5LTR +
Theropithecus_gelada_CM009955.1 83409682 83410005 HERVHF_gag +
Theropithecus_gelada_CM009955.1 83410081 83410413 HERVHF_pro +
Theropithecus_gelada_CM009955.1 83410428 83412156 HERVHF_pol +
Theropithecus_gelada_CM009955.1 83412769 83413208 HERVHF_3LTR +
Theropithecus_gelada_CM009955.1 90413310 90413612 HERVHF_5LTR +
Theropithecus_gelada_CM009955.1 90415412 90415729 HERVHF_gag +
Theropithecus_gelada_CM009955.1 90416316 90417827 HERVHF_pol +
Theropithecus_gelada_CM009955.1 90418666 90418965 HERVHF_3LTR +
Theropithecus_gelada_CM009955.1 115490310 115490675 HERVHF_5LTR -

Theropithecus_gelada_CM009955.1 115490841 115491230 HERVHF_env -
Theropithecus_gelada_CM009955.1 115492299 115494652 HERVHF_pol -
Theropithecus_gelada_CM009955.1 115495528 115495935 HERVHF_gag -
Theropithecus_gelada_CM009955.1 115497252 115497606 HERVHF_3LTR -

Theropithecus_gelada_CM009955.1 123181587 123181893 HERVHF_5LTR +

Theropithecus_gelada_CM009955.1 123190411 123190953 HERVHF_gag +
Theropithecus_gelada_CM009955.1 123191137 123191604 HERVHF_pro +
Theropithecus_gelada_CM009955.1 123191290 123193605 HERVHF_pol +
Theropithecus_gelada_CM009955.1 123196561 123196861 HERVHF_3LTR +

Theropithecus_gelada_CM009955.1 134186982 134187433 HERVHF_5LTR -

Theropithecus_gelada_CM009955.1 134188235 134190420 HERVHF_pol -
Theropithecus_gelada_CM009955.1 134189923 134190705 HERVHF_pro -
Theropithecus_gelada_CM009955.1 134192888 134193335 HERVHF_3LTR -

Theropithecus_gelada_CM009955.1 142109365 142109690 HERVHF_5LTR +

Theropithecus_gelada_CM009955.1 142111936 142112412 HERVHF_pro +

Theropithecus_gelada_CM009955.1	142112074	142113383	HERVHF_pol	+
Theropithecus_gelada_CM009955.1	142113592	142114206	HERVHF_env	+
Theropithecus_gelada_CM009955.1	142114490	142114825	HERVHF_3LTR	+
Theropithecus_gelada_CM009955.1	167181207	167181558	HERVHF_5LTR	-
Theropithecus_gelada_CM009955.1	167182229	167184926	HERVHF_pol	-
Theropithecus_gelada_CM009955.1	167184324	167185025	HERVHF_pro	-
Theropithecus_gelada_CM009955.1	167185376	167185948	HERVHF_gag	-
Theropithecus_gelada_CM009955.1	167187333	167187682	HERVHF_3LTR	-
Theropithecus_gelada_CM009956.1	49997931	49998246	HERVHF_5LTR	+
Theropithecus_gelada_CM009956.1	50002663	50003124	HERVHF_gag	+
Theropithecus_gelada_CM009956.1	50003314	50004033	HERVHF_pro	+
Theropithecus_gelada_CM009956.1	50003521	50005673	HERVHF_pol	+
Theropithecus_gelada_CM009956.1	50006670	50006973	HERVHF_3LTR	+
Theropithecus_gelada_CM009957.1	2561723	2562123	HERVHF_5LTR	-
Theropithecus_gelada_CM009957.1	2562964	2565113	HERVHF_pol	-
Theropithecus_gelada_CM009957.1	2564571	2565206	HERVHF_pro	-
Theropithecus_gelada_CM009957.1	2565402	2565743	HERVHF_gag	-
Theropithecus_gelada_CM009957.1	2567318	2567709	HERVHF_3LTR	-
Theropithecus_gelada_CM009957.1	38171703	38172154	HERVHF_5LTR	-
Theropithecus_gelada_CM009957.1	38172599	38174179	HERVHF_pol	-
Theropithecus_gelada_CM009957.1	38174208	38174636	HERVHF_pro	-
Theropithecus_gelada_CM009957.1	38176722	38177176	HERVHF_3LTR	-
Theropithecus_gelada_CM009957.1	102814932	102815333	HERVHF_5LTR	-
Theropithecus_gelada_CM009957.1	102816188	102818182	HERVHF_pol	-
Theropithecus_gelada_CM009957.1	102817805	102818617	HERVHF_pro	-
Theropithecus_gelada_CM009957.1	102818688	102819008	HERVHF_gag	-
Theropithecus_gelada_CM009957.1	102820484	102820875	HERVHF_3LTR	-
Theropithecus_gelada_CM009958.1	142957091	142957554	HERVHF_5LTR	+
Theropithecus_gelada_CM009958.1	142959270	142959584	HERVHF_gag	+
Theropithecus_gelada_CM009958.1	142959948	142962395	HERVHF_pol	+
Theropithecus_gelada_CM009958.1	142962475	142964128	HERVHF_env	+
Theropithecus_gelada_CM009958.1	142964352	142964815	HERVHF_3LTR	+
Theropithecus_gelada_CM009959.1	16950704	16951068	HERVHF_5LTR	-
Theropithecus_gelada_CM009959.1	16952107	16952586	HERVHF_pol	-
Theropithecus_gelada_CM009959.1	16952796	16953104	HERVHF_pro	-
Theropithecus_gelada_CM009959.1	16954965	16955327	HERVHF_3LTR	-
Theropithecus_gelada_CM009959.1	84097791	84098217	HERVHF_5LTR	-

Theropithecus_gelada_CM009959.1 84099123 84100643 HERVHF_pol -
Theropithecus_gelada_CM009959.1 84102326 84103180 HERVHF_pro -
Theropithecus_gelada_CM009959.1 84103190 84104185 HERVHF_gag -
Theropithecus_gelada_CM009959.1 84105542 84105966 HERVHF_3LTR -
Theropithecus_gelada_CM009959.1 97735385 97735697 HERVHF_5LTR -
Theropithecus_gelada_CM009959.1 97735863 97736195 HERVHF_env -
Theropithecus_gelada_CM009959.1 97736949 97737860 HERVHF_pol -
Theropithecus_gelada_CM009959.1 97737405 97738193 HERVHF_pro -
Theropithecus_gelada_CM009959.1 97738327 97738683 HERVHF_gag -
Theropithecus_gelada_CM009959.1 97740105 97740433 HERVHF_3LTR -
Theropithecus_gelada_CM009959.1 107040929 107041361 HERVHF_5LTR -

Theropithecus_gelada_CM009959.1 107042190 107044300 HERVHF_pol -
Theropithecus_gelada_CM009959.1 107043839 107044330 HERVHF_pro -
Theropithecus_gelada_CM009959.1 107044609 107044965 HERVHF_gag -
Theropithecus_gelada_CM009959.1 107046780 107047213 HERVHF_3LTR -

Theropithecus_gelada_CM009961.1 905988 906439 HERVHF_5LTR +
Theropithecus_gelada_CM009961.1 908052 908393 HERVHF_gag +
Theropithecus_gelada_CM009961.1 908592 910657 HERVHF_pol +
Theropithecus_gelada_CM009961.1 911184 911635 HERVHF_3LTR +
Theropithecus_gelada_CM009961.1 23139296 23139671 HSERVIII_5LTR -
Theropithecus_gelada_CM009961.1 23140517 23143650 HSERVIII_pol -
Theropithecus_gelada_CM009961.1 23145945 23146313 HSERVIII_3LTR -
Theropithecus_gelada_CM009961.1 35395506 35395807 HERVHF_5LTR -
Theropithecus_gelada_CM009961.1 35396071 35398219 HERVHF_pol -
Theropithecus_gelada_CM009961.1 35398260 35398592 HERVHF_pro -
Theropithecus_gelada_CM009961.1 35400554 35400863 HERVHF_3LTR -
Theropithecus_gelada_CM009961.1 83305352 83305788 HERVHF_5LTR +
Theropithecus_gelada_CM009961.1 83307531 83308022 HERVHF_gag +
Theropithecus_gelada_CM009961.1 83308297 83311174 HERVHF_pol +
Theropithecus_gelada_CM009961.1 83311283 83311633 HERVHF_env +
Theropithecus_gelada_CM009961.1 83311792 83312221 HERVHF_3LTR +
Theropithecus_gelada_CM009962.1 43518967 43519420 HERVK_5LTR -
Theropithecus_gelada_CM009962.1 43520372 43522284 HERVK_pol -
Theropithecus_gelada_CM009962.1 43522179 43523078 HERVK_pro -
Theropithecus_gelada_CM009962.1 43524045 43524494 HERVK_3LTR -
Theropithecus_gelada_CM009962.1 96915122 96915473 HERVHF_5LTR -
Theropithecus_gelada_CM009962.1 96915640 96916839 HERVHF_env -
Theropithecus_gelada_CM009962.1 96918121 96919979 HERVHF_pol -
Theropithecus_gelada_CM009962.1 96920470 96920934 HERVHF_gag -
Theropithecus_gelada_CM009962.1 96922527 96922881 HERVHF_3LTR -
Theropithecus_gelada_CM009963.1 68072041 68072385 HERVHF_5LTR -
Theropithecus_gelada_CM009963.1 68072798 68073328 HERVHF_env -

Theropithecus_gelada_CM009963.1 68074848 68077886 HERVHF_pol -
Theropithecus_gelada_CM009963.1 68077248 68077997 HERVHF_pro -
Theropithecus_gelada_CM009963.1 68079391 68080026 HERVHF_gag -
Theropithecus_gelada_CM009963.1 68081676 68082012 HERVHF_3LTR -
Theropithecus_gelada_CM009963.1 68175155 68175456 HERVHF_5LTR -
Theropithecus_gelada_CM009963.1 68179982 68182079 HERVHF_pol -
Theropithecus_gelada_CM009963.1 68182237 68182680 HERVHF_gag -
Theropithecus_gelada_CM009963.1 68183671 68183984 HERVHF_3LTR -
Theropithecus_gelada_CM009963.1 72650152 72650553 HERVHF_5LTR -
Theropithecus_gelada_CM009963.1 72651494 72653551 HERVHF_pol -
Theropithecus_gelada_CM009963.1 72652928 72653800 HERVHF_pro -
Theropithecus_gelada_CM009963.1 72654860 72655248 HERVHF_3LTR -
Theropithecus_gelada_CM009964.1 33042798 33043254 HERVHF_5LTR -
Theropithecus_gelada_CM009964.1 33044449 33046123 HERVHF_pol -
Theropithecus_gelada_CM009964.1 33045581 33046366 HERVHF_pro -
Theropithecus_gelada_CM009964.1 33048506 33048970 HERVHF_3LTR -
Theropithecus_gelada_CM009964.1 124624097 124624547 HERVHF_5LTR +

Theropithecus_gelada_CM009964.1 124626904 124628604 HERVHF_pol +
Theropithecus_gelada_CM009964.1 124629404 124629859 HERVHF_3LTR +

Theropithecus_gelada_CM009966.1 4074346 4074670 HERVK_5LTR -
Theropithecus_gelada_CM009966.1 4075517 4076819 HERVK_pol -
Theropithecus_gelada_CM009966.1 4076687 4077487 HERVK_pro -
Theropithecus_gelada_CM009966.1 4078697 4079021 HERVK_3LTR -
Theropithecus_gelada_CM009967.1 79102108 79102427 HERVHF_5LTR -
Theropithecus_gelada_CM009967.1 79102831 79103260 HERVHF_env -
Theropithecus_gelada_CM009967.1 79103462 79105619 HERVHF_pol -
Theropithecus_gelada_CM009967.1 79105083 79105580 HERVHF_pro -
Theropithecus_gelada_CM009967.1 79107636 79107948 HERVHF_3LTR -
Theropithecus_gelada_CM009969.1 19546419 19546727 HERVIPADP_5LTR -
Theropithecus_gelada_CM009969.1 19550888 19553145 HERVIPADP_pol -
Theropithecus_gelada_CM009969.1 19560579 19560900 HERVIPADP_3LTR -
Theropithecus_gelada_CM009969.1 20050828 20051299 HERVIPADP_5LTR -
Theropithecus_gelada_CM009969.1 20051852 20052433 HERVIPADP_env -
Theropithecus_gelada_CM009969.1 20053409 20056010 HERVIPADP_pol -
Theropithecus_gelada_CM009969.1 20059261 20059720 HERVIPADP_3LTR -
Theropithecus_gelada_CM009969.1 23393424 23393747 HERVHF_5LTR -
Theropithecus_gelada_CM009969.1 23394581 23397538 HERVHF_pol -
Theropithecus_gelada_CM009969.1 23397002 23397538 HERVHF_pro -
Theropithecus_gelada_CM009969.1 23404774 23405091 HERVHF_3LTR -
Theropithecus_gelada_CM009970.1 57096520 57096884 HERVHF_5LTR +
Theropithecus_gelada_CM009970.1 57098655 57098954 HERVHF_gag +
Theropithecus_gelada_CM009970.1 57099999 57100694 HERVHF_pro +

Theropithecus_gelada_CM009970.1 57100275 57103142 HERVHF_pol +
Theropithecus_gelada_CM009970.1 57103429 57103803 HERVHF_3LTR +
Theropithecus_gelada_CM009971.1 21725626 21725955 HERVHF_5LTR +
Theropithecus_gelada_CM009971.1 21727207 21727785 HERVHF_gag +
Theropithecus_gelada_CM009971.1 21727812 21728789 HERVHF_pro +
Theropithecus_gelada_CM009971.1 21728205 21730765 HERVHF_pol +
Theropithecus_gelada_CM009971.1 21731843 21732161 HERVHF_3LTR +
Theropithecus_gelada_CM009971.1 71854357 71854790 HERVHF_5LTR -
Theropithecus_gelada_CM009971.1 71859766 71860611 HERVHF_env -
Theropithecus_gelada_CM009971.1 71862735 71864709 HERVHF_pol -
Theropithecus_gelada_CM009971.1 71864281 71865078 HERVHF_pro -
Theropithecus_gelada_CM009971.1 71865507 71866043 HERVHF_gag -
Theropithecus_gelada_CM009971.1 71866519 71866949 HERVHF_3LTR -
Theropithecus_gelada_CM009971.1 80203330 80203706 HERVHF_5LTR +
Theropithecus_gelada_CM009971.1 80205061 80205372 HERVHF_gag +
Theropithecus_gelada_CM009971.1 80205363 80207336 HERVHF_pol +
Theropithecus_gelada_CM009971.1 80208090 80209152 HERVHF_env +
Theropithecus_gelada_CM009971.1 80209295 80209672 HERVHF_3LTR +
Theropithecus_gelada_CM009971.1 86976723 86977267 HERVHF_5LTR +
Theropithecus_gelada_CM009971.1 86982660 86983430 HERVHF_pro +
Theropithecus_gelada_CM009971.1 86982792 86984885 HERVHF_pol +
Theropithecus_gelada_CM009971.1 86991417 86991952 HERVHF_3LTR +
Theropithecus_gelada_CM009971.1 118380766 118381237 HERVHF_5LTR -

Theropithecus_gelada_CM009971.1 118382123 118383465 HERVHF_pol -
Theropithecus_gelada_CM009971.1 118383046 118383831 HERVHF_pro -
Theropithecus_gelada_CM009971.1 118386003 118386480 HERVHF_3LTR -

Theropithecus_gelada_CM009971.1 147187140 147187696 HSERVIII_5LTR +

Theropithecus_gelada_CM009971.1 147197253 147197795 HSERVIII_pol +
Theropithecus_gelada_CM009971.1 147198198 147198761 HSERVIII_3LTR +

Trachypithecus_phayrei_ctg1 36867080 36867537 HERVHF_5LTR -
Trachypithecus_phayrei_ctg1 36868395 36870742 HERVHF_pol -
Trachypithecus_phayrei_ctg1 36870179 36870979 HERVHF_pro -
Trachypithecus_phayrei_ctg1 36873007 36873462 HERVHF_3LTR -
Trachypithecus_phayrei_ctg11 37747240 37747690 HERVHF_5LTR +
Trachypithecus_phayrei_ctg11 37749374 37749697 HERVHF_gag +
Trachypithecus_phayrei_ctg11 37750282 37751334 HERVHF_pro +
Trachypithecus_phayrei_ctg11 37750531 37752377 HERVHF_pol +
Trachypithecus_phayrei_ctg11 37753284 37753735 HERVHF_3LTR +
Trachypithecus_phayrei_ctg117 1073782 1074205 HERVHF_5LTR -
Trachypithecus_phayrei_ctg117 1075133 1077172 HERVHF_pol -

Trachypithecus_phayrei_ctg117	1077506	1077922	HERVHF_pro	-
Trachypithecus_phayrei_ctg117	1078183	1078527	HERVHF_gag	-
Trachypithecus_phayrei_ctg117	1080063	1080484	HERVHF_3LTR	-
Trachypithecus_phayrei_ctg125	351744	352075	HERVHF_5LTR	-
Trachypithecus_phayrei_ctg125	352195	352597	HERVHF_env	-
Trachypithecus_phayrei_ctg125	352623	354757	HERVHF_pol	-
Trachypithecus_phayrei_ctg125	354539	354865	HERVHF_pro	-
Trachypithecus_phayrei_ctg125	355171	355470	HERVHF_gag	-
Trachypithecus_phayrei_ctg125	357442	357767	HERVHF_3LTR	-
Trachypithecus_phayrei_ctg15	9593176	9593482	HERVHF_5LTR	-
Trachypithecus_phayrei_ctg15	9596949	9597518	HERVHF_pol	-
Trachypithecus_phayrei_ctg15	9597060	9597818	HERVHF_pro	-
Trachypithecus_phayrei_ctg15	9604399	9604701	HERVHF_3LTR	-
Trachypithecus_phayrei_ctg159	7602136	7602483	HERVHF_5LTR	-
Trachypithecus_phayrei_ctg159	7603763	7605744	HERVHF_pol	-
Trachypithecus_phayrei_ctg159	7606463	7606894	HERVHF_gag	-
Trachypithecus_phayrei_ctg159	7608542	7608883	HERVHF_3LTR	-
Trachypithecus_phayrei_ctg16	3889536	3889951	HERVHF_5LTR	-
Trachypithecus_phayrei_ctg16	3890102	3890443	HERVHF_env	-
Trachypithecus_phayrei_ctg16	3890655	3894708	HERVHF_pol	-
Trachypithecus_phayrei_ctg16	3894043	3894801	HERVHF_pro	-
Trachypithecus_phayrei_ctg16	3895027	3895542	HERVHF_gag	-
Trachypithecus_phayrei_ctg16	3896990	3897405	HERVHF_3LTR	-
Trachypithecus_phayrei_ctg167	1862438	1862941	HERVHF_5LTR	-
Trachypithecus_phayrei_ctg167	1866814	1869070	HERVHF_pol	-
Trachypithecus_phayrei_ctg167	1868513	1869232	HERVHF_pro	-
Trachypithecus_phayrei_ctg167	1871277	1871781	HERVHF_3LTR	-
Trachypithecus_phayrei_ctg17	40160244	40161198	HERVHF_5LTR	-
Trachypithecus_phayrei_ctg17	40161237	40163090	HERVHF_pol	-
Trachypithecus_phayrei_ctg17	40165858	40166809	HERVHF_3LTR	-
Trachypithecus_phayrei_ctg18	13308	13665	HERVHF_5LTR	-
Trachypithecus_phayrei_ctg18	13897	14325	HERVHF_env	-
Trachypithecus_phayrei_ctg18	14424	17000	HERVHF_pol	-
Trachypithecus_phayrei_ctg18	16473	17036	HERVHF_pro	-
Trachypithecus_phayrei_ctg18	17248	17553	HERVHF_gag	-
Trachypithecus_phayrei_ctg18	19038	19403	HERVHF_3LTR	-
Trachypithecus_phayrei_ctg19	8184114	8184515	HERVHF_5LTR	+
Trachypithecus_phayrei_ctg19	8186082	8186834	HERVHF_pro	+
Trachypithecus_phayrei_ctg19	8186214	8187691	HERVHF_pol	+
Trachypithecus_phayrei_ctg19	8187857	8188260	HERVHF_3LTR	+
Trachypithecus_phayrei_ctg190	1456164	1456468	HERVHF_5LTR	-
Trachypithecus_phayrei_ctg190	1456974	1458957	HERVHF_pol	-
Trachypithecus_phayrei_ctg190	1458616	1459188	HERVHF_pro	-
Trachypithecus_phayrei_ctg190	1459783	1460136	HERVHF_gag	-

Trachypithecus_phayrei_ctg190	1464821	1465121	HERVHF_3LTR	-
Trachypithecus_phayrei_ctg199	1856413	1856786	HERVHF_5LTR	-
Trachypithecus_phayrei_ctg199	1857014	1857654	HERVHF_env	-
Trachypithecus_phayrei_ctg199	1858456	1859136	HERVHF_pol	-
Trachypithecus_phayrei_ctg199	1858582	1859304	HERVHF_pro	-
Trachypithecus_phayrei_ctg199	1859445	1861015	HERVHF_gag	-
Trachypithecus_phayrei_ctg199	1861744	1862116	HERVHF_3LTR	-
Trachypithecus_phayrei_ctg2	3135737	3136140	HERVHF_5LTR	-
Trachypithecus_phayrei_ctg2	3137060	3137374	HERVHF_env	-
Trachypithecus_phayrei_ctg2	3138423	3141324	HERVHF_pol	-
Trachypithecus_phayrei_ctg2	3141111	3141440	HERVHF_pro	-
Trachypithecus_phayrei_ctg2	3142029	3142418	HERVHF_gag	-
Trachypithecus_phayrei_ctg2	3148950	3149364	HERVHF_3LTR	-
Trachypithecus_phayrei_ctg203	2921305	2921629	HERVHF_5LTR	+
Trachypithecus_phayrei_ctg203	2922969	2923361	HERVHF_gag	+
Trachypithecus_phayrei_ctg203	2924635	2926762	HERVHF_pol	+
Trachypithecus_phayrei_ctg203	2927267	2927629	HERVHF_env	+
Trachypithecus_phayrei_ctg203	2927821	2928150	HERVHF_3LTR	+
Trachypithecus_phayrei_ctg21	1181086	1181395	HERVHF_5LTR	+
Trachypithecus_phayrei_ctg21	1184318	1184620	HERVHF_gag	+
Trachypithecus_phayrei_ctg21	1184706	1185512	HERVHF_pro	+
Trachypithecus_phayrei_ctg21	1184982	1186677	HERVHF_pol	+
Trachypithecus_phayrei_ctg21	1187351	1187674	HERVHF_3LTR	+
Trachypithecus_phayrei_ctg216	654655	654970	HERVHF_5LTR	-
Trachypithecus_phayrei_ctg216	655664	657064	HERVHF_pol	-
Trachypithecus_phayrei_ctg216	657152	657919	HERVHF_pro	-
Trachypithecus_phayrei_ctg216	657953	658621	HERVHF_gag	-
Trachypithecus_phayrei_ctg216	660204	660517	HERVHF_3LTR	-
Trachypithecus_phayrei_ctg222	1839646	1840060	HERVHF_5LTR	-
Trachypithecus_phayrei_ctg222	1841085	1842628	HERVHF_pol	-
Trachypithecus_phayrei_ctg222	1842116	1842829	HERVHF_pro	-
Trachypithecus_phayrei_ctg222	1842917	1843351	HERVHF_gag	-
Trachypithecus_phayrei_ctg222	1844730	1845148	HERVHF_3LTR	-
Trachypithecus_phayrei_ctg31	5193946	5194375	HERVHF_5LTR	-
Trachypithecus_phayrei_ctg31	5195182	5197574	HERVHF_pol	-
Trachypithecus_phayrei_ctg31	5197549	5197932	HERVHF_pro	-
Trachypithecus_phayrei_ctg31	5200621	5201054	HERVHF_3LTR	-
Trachypithecus_phayrei_ctg31	5993919	5994240	HERVHF_5LTR	+
Trachypithecus_phayrei_ctg31	5996259	5996582	HERVHF_gag	+
Trachypithecus_phayrei_ctg31	5996869	5997378	HERVHF_pol	+
Trachypithecus_phayrei_ctg31	5998578	5998901	HERVHF_3LTR	+
Trachypithecus_phayrei_ctg35	4550211	4550641	HERVHF_5LTR	-
Trachypithecus_phayrei_ctg35	4551928	4553380	HERVHF_pol	-
Trachypithecus_phayrei_ctg35	4555657	4556074	HERVHF_3LTR	-

Trachypithecus_phayrei_ctg354	592806	593241	HERVHF_5LTR	-
Trachypithecus_phayrei_ctg354	593617	594505	HERVHF_env	-
Trachypithecus_phayrei_ctg354	595497	597531	HERVHF_pol	-
Trachypithecus_phayrei_ctg354	597776	598216	HERVHF_pro	-
Trachypithecus_phayrei_ctg354	600504	600941	HERVHF_3LTR	-
Trachypithecus_phayrei_ctg36	19713954	19714430	HERVHF_5LTR	+
Trachypithecus_phayrei_ctg36	19716646	19717038	HERVHF_pro	+
Trachypithecus_phayrei_ctg36	19716987	19718372	HERVHF_pol	+
Trachypithecus_phayrei_ctg36	19719196	19719667	HERVHF_3LTR	+
Trachypithecus_phayrei_ctg409	272346	272798	HERVHF_5LTR	+
Trachypithecus_phayrei_ctg409	275127	276562	HERVHF_pol	+
Trachypithecus_phayrei_ctg409	277397	277849	HERVHF_3LTR	+
Trachypithecus_phayrei_ctg45	20076807	20077247	HERVHF_5LTR	-
Trachypithecus_phayrei_ctg45	20077802	20079819	HERVHF_pol	-
Trachypithecus_phayrei_ctg45	20079827	20080234	HERVHF_pro	-
Trachypithecus_phayrei_ctg45	20080333	20081062	HERVHF_gag	-
Trachypithecus_phayrei_ctg45	20082334	20082773	HERVHF_3LTR	-
Trachypithecus_phayrei_ctg46	8677890	8678351	HERVHF_5LTR	-
Trachypithecus_phayrei_ctg46	8679088	8681484	HERVHF_pol	-
Trachypithecus_phayrei_ctg46	8680720	8681553	HERVHF_pro	-
Trachypithecus_phayrei_ctg46	8683627	8684099	HERVHF_3LTR	-
Trachypithecus_phayrei_ctg49	2589775	2590208	HERVHF_5LTR	+
Trachypithecus_phayrei_ctg49	2592010	2592309	HERVHF_gag	+
Trachypithecus_phayrei_ctg49	2592675	2594341	HERVHF_pol	+
Trachypithecus_phayrei_ctg49	2595591	2596024	HERVHF_3LTR	+
Trachypithecus_phayrei_ctg497	286426	286860	HERVHF_5LTR	-
Trachypithecus_phayrei_ctg497	287968	290767	HERVHF_pol	-
Trachypithecus_phayrei_ctg497	291126	291545	HERVHF_gag	-
Trachypithecus_phayrei_ctg497	292927	293349	HERVHF_3LTR	-
Trachypithecus_phayrei_ctg500	106782	107202	HERVHF_5LTR	-
Trachypithecus_phayrei_ctg500	107713	108207	HERVHF_env	-
Trachypithecus_phayrei_ctg500	110460	112513	HERVHF_pol	-
Trachypithecus_phayrei_ctg500	113497	113829	HERVHF_gag	-
Trachypithecus_phayrei_ctg500	115754	116178	HERVHF_3LTR	-
Trachypithecus_phayrei_ctg51	2961614	2962026	HERVHF_5LTR	-
Trachypithecus_phayrei_ctg51	2962390	2964810	HERVHF_pol	-
Trachypithecus_phayrei_ctg51	2964899	2965309	HERVHF_pro	-
Trachypithecus_phayrei_ctg51	2965362	2966918	HERVHF_gag	-
Trachypithecus_phayrei_ctg51	2967511	2967923	HERVHF_3LTR	-
Trachypithecus_phayrei_ctg61	9364625	9364924	Unknown_HERV_5LTR	-
Trachypithecus_phayrei_ctg61	9367245	9375219	Unknown_HERV_pol	-
Trachypithecus_phayrei_ctg61	9374575	9375480	Unknown_HERV_pro	-
Trachypithecus_phayrei_ctg61	9375612	9375947	Unknown_HERV_gag	-
Trachypithecus_phayrei_ctg61	9379603	9379902	Unknown_HERV_3LTR	-

Trachypithecus_phayrei_ctg65	3633442	3633790	HERVHF_5LTR	-
Trachypithecus_phayrei_ctg65	3634806	3636812	HERVHF_pol	-
Trachypithecus_phayrei_ctg65	3636291	3637055	HERVHF_pro	-
Trachypithecus_phayrei_ctg65	3637108	3637464	HERVHF_gag	-
Trachypithecus_phayrei_ctg65	3638964	3639294	HERVHF_3LTR	-
Trachypithecus_phayrei_ctg66	1547521	1547932	HERVHF_5LTR	+
Trachypithecus_phayrei_ctg66	1550375	1551094	HERVHF_pro	+
Trachypithecus_phayrei_ctg66	1550579	1552281	HERVHF_pol	+
Trachypithecus_phayrei_ctg66	1553092	1553506	HERVHF_3LTR	+
Trachypithecus_phayrei_ctg69	14883471	14883778	HERVHF_5LTR	-
Trachypithecus_phayrei_ctg69	14887540	14887872	HERVHF_env	-
Trachypithecus_phayrei_ctg69	14888212	14890160	HERVHF_pol	-
Trachypithecus_phayrei_ctg69	14889612	14890241	HERVHF_pro	-
Trachypithecus_phayrei_ctg69	14890500	14890943	HERVHF_gag	-
Trachypithecus_phayrei_ctg69	14893019	14893324	HERVHF_3LTR	-
Trachypithecus_phayrei_ctg7	10861045	10861429	HERVHF_5LTR	+
Trachypithecus_phayrei_ctg7	10862961	10863530	HERVHF_gag	+
Trachypithecus_phayrei_ctg7	10863586	10864140	HERVHF_pro	+
Trachypithecus_phayrei_ctg7	10863888	10865414	HERVHF_pol	+
Trachypithecus_phayrei_ctg7	10867377	10867727	HERVHF_env	+
Trachypithecus_phayrei_ctg7	10867870	10868261	HERVHF_3LTR	+
Trachypithecus_phayrei_ctg73	27317232	27317587	HERVHF_5LTR	+
Trachypithecus_phayrei_ctg73	27320688	27321173	HERVHF_gag	+
Trachypithecus_phayrei_ctg73	27321627	27322395	HERVHF_pro	+
Trachypithecus_phayrei_ctg73	27322069	27324633	HERVHF_pol	+
Trachypithecus_phayrei_ctg73	27328537	27328896	HERVHF_3LTR	+
Trachypithecus_phayrei_ctg80	4204779	4205227	HERVHF_5LTR	+
Trachypithecus_phayrei_ctg80	4207783	4208157	HERVHF_pro	+
Trachypithecus_phayrei_ctg80	4208309	4208869	HERVHF_pol	+
Trachypithecus_phayrei_ctg80	4214182	4214583	HERVHF_env	+
Trachypithecus_phayrei_ctg80	4214999	4215445	HERVHF_3LTR	+
Trachypithecus_phayrei_ctg85	13677443	13677881	HERVHF_5LTR	-
Trachypithecus_phayrei_ctg85	13678403	13680366	HERVHF_pol	-
Trachypithecus_phayrei_ctg85	13679905	13680982	HERVHF_pro	-
Trachypithecus_phayrei_ctg85	13682940	13683382	HERVHF_3LTR	-
Trachypithecus_phayrei_ctg87	30673	31071	HERVHF_5LTR	+
Trachypithecus_phayrei_ctg87	33258	35290	HERVHF_pol	+
Trachypithecus_phayrei_ctg87	35783	36190	HERVHF_3LTR	+
Trachypithecus_phayrei_ctg95	7827629	7827966	HERVHF_5LTR	+
Trachypithecus_phayrei_ctg95	7829939	7830697	HERVHF_pro	+
Trachypithecus_phayrei_ctg95	7830074	7831924	HERVHF_pol	+
Trachypithecus_phayrei_ctg95	7832478	7832821	HERVHF_3LTR	+
Trachypithecus_phayrei_ctg96	9098975	9099341	HERVHF_5LTR	-
Trachypithecus_phayrei_ctg96	9100026	9101698	HERVHF_pol	-

Trachypithecus_phayrei_ctg96	9101363	9102055	HERVHF_pro	-
Trachypithecus_phayrei_ctg96	9102169	9102603	HERVHF_gag	-
Trachypithecus_phayrei_ctg96	9105415	9105778	HERVHF_3LTR	-

Supplementary data S3 The vertical transmission events in human genomes

HERV-class&num	Distribution
HERVHF1	"Homo sapiens,Gorilla gorilla gorilla"
HERVHF2	"Homo sapiens,Gorilla gorilla gorilla"
HERVHF3	"Homo sapiens,Gorilla gorilla gorilla"
HERVHF4	"Homo sapiens,Gorilla gorilla gorilla"
HERVHF5	"Homo sapiens,Gorilla gorilla gorilla"
HERVHF6	"Homo sapiens,Gorilla gorilla gorilla"
HERVHF7	"Homo sapiens,Gorilla gorilla gorilla"
HERVHF8	"Homo sapiens,Gorilla gorilla gorilla"
HERVHF9	"Homo sapiens,Gorilla gorilla gorilla"
HERVHF10	"Homo sapiens,Gorilla gorilla gorilla"
HERVHF11	"Homo sapiens,Gorilla gorilla gorilla"
HERVHF12	"Homo sapiens,Gorilla gorilla gorilla"
HERVHF13	"Homo sapiens,Gorilla gorilla gorilla"
HERVHF14	"Homo sapiens,Gorilla gorilla gorilla"
HERVHF15	"Homo sapiens,Gorilla gorilla gorilla"
HERVHF16	"Homo sapiens,Gorilla gorilla gorilla"
HERVHF17	"Homo sapiens,Gorilla gorilla gorilla"
HERVHF18	"Homo sapiens,Gorilla gorilla gorilla"
HERVHF19	"Homo sapiens,Gorilla gorilla gorilla"
HERVHF20	"Homo sapiens,Gorilla gorilla gorilla"
HERVHF21	"Homo sapiens,Gorilla gorilla gorilla"
HERVHF22	"Homo sapiens,Gorilla gorilla gorilla"
HERVHF23	"Homo sapiens,Gorilla gorilla gorilla"
HERVHF24	"Homo sapiens,Gorilla gorilla gorilla"
HERVHF25	"Homo sapiens,Gorilla gorilla gorilla"
HERVHF26	"Homo sapiens,Gorilla gorilla gorilla"
HERVHF27	"Homo sapiens,Gorilla gorilla gorilla"
HERVHF28	"Homo sapiens,Gorilla gorilla gorilla"
HERVHF29	"Homo sapiens,Gorilla gorilla gorilla"
HERVHF30	"Homo sapiens,Gorilla gorilla gorilla"
HERVHF31	"Homo sapiens,Gorilla gorilla gorilla"
HERVHF32	"Homo sapiens,Gorilla gorilla gorilla"
HERVHF33	"Homo sapiens,Gorilla gorilla gorilla"
HERVHF34	"Homo sapiens,Gorilla gorilla gorilla"
HERVHF35	"Homo sapiens,Gorilla gorilla gorilla"
HERVHF36	"Homo sapiens,Gorilla gorilla gorilla"
HERVHF37	"Homo sapiens,Gorilla gorilla gorilla"
HERVHF38	"Homo sapiens,Gorilla gorilla gorilla"
HERVHF39	"Homo sapiens,Gorilla gorilla gorilla"
HERVHF40	"Homo sapiens,Gorilla gorilla gorilla"
HERVHF41	"Homo sapiens,Gorilla gorilla gorilla"

HERVHF42	"Homo sapiens,Gorilla gorilla gorilla"
HERVHF43	"Homo sapiens,Gorilla gorilla gorilla"
HERVHF44	"Homo sapiens,Gorilla gorilla gorilla"
HERVHF45	"Homo sapiens,Gorilla gorilla gorilla"
HERVHF46	"Homo sapiens,Gorilla gorilla gorilla"
HERVHF47	"Homo sapiens,Gorilla gorilla gorilla"
HERVHF48	"Homo sapiens,Gorilla gorilla gorilla"
HERVHF49	"Homo sapiens,Gorilla gorilla gorilla"
HERVHF50	"Homo sapiens,Gorilla gorilla gorilla"
HERVHF51	"Homo sapiens,Gorilla gorilla gorilla"
HERVHF52	"Homo sapiens,Gorilla gorilla gorilla"
HERVHF53	"Homo sapiens,Gorilla gorilla gorilla"
HERVHF54	"Homo sapiens,Gorilla gorilla gorilla"
HERVHF55	"Homo sapiens,Gorilla gorilla gorilla"
HERVHF56	"Homo sapiens,Gorilla gorilla gorilla"
HERVHF57	"Homo sapiens,Gorilla gorilla gorilla"
HERVHF58	"Homo sapiens,Gorilla gorilla gorilla"
HERVHF59	"Homo sapiens,Gorilla gorilla gorilla"
HERVHF60	"Homo sapiens,Gorilla gorilla gorilla"
HERVHF61	"Homo sapiens,Gorilla gorilla gorilla"
HERVHF62	"Homo sapiens,Gorilla gorilla gorilla"
HERVHF63	"Homo sapiens,Gorilla gorilla gorilla"
HERVHF64	"Homo sapiens,Gorilla gorilla gorilla"
HERVHF65	"Homo sapiens,Gorilla gorilla gorilla"
HERVHF66	"Homo sapiens,Gorilla gorilla gorilla"
HERVHF67	"Homo sapiens,Gorilla gorilla gorilla"
HERVHF68	"Homo sapiens,Gorilla gorilla gorilla"
HERVHF69	"Homo sapiens,Gorilla gorilla gorilla"
HERVHF70	"Homo sapiens,Gorilla gorilla gorilla"
HERVHF71	"Homo sapiens,Gorilla gorilla gorilla"
HERVHF72	"Homo sapiens,Gorilla gorilla gorilla"
HERVHF73	"Homo sapiens,Gorilla gorilla gorilla"
HERVHF74	"Homo sapiens,Gorilla gorilla gorilla"
HERVHF75	"Homo sapiens,Gorilla gorilla gorilla"
HERVHF76	"Homo sapiens,Gorilla gorilla gorilla"
HERVHF77	"Homo sapiens,Gorilla gorilla gorilla"
HERVHF78	"Homo sapiens,Gorilla gorilla gorilla"
HERVHF79	"Homo sapiens,Gorilla gorilla gorilla"
HERVHF80	"Homo sapiens,Gorilla gorilla gorilla"
HERVHF81	"Homo sapiens,Gorilla gorilla gorilla"
HERVHF82	"Homo sapiens,Gorilla gorilla gorilla"
HERVHF83	"Homo sapiens,Gorilla gorilla gorilla"
HERVHF84	"Homo sapiens,Gorilla gorilla gorilla"
HERVHF85	"Homo sapiens,Gorilla gorilla gorilla"

HERVHF86	"Homo sapiens,Hylobates pileatus"
HERVHF87	"Homo sapiens,Pan paniscus"
HERVHF88	"Homo sapiens,Pan paniscus"
HERVHF89	"Homo sapiens,Pan paniscus"
HERVHF90	"Homo sapiens,Pan paniscus"
HERVHF91	"Homo sapiens,Pan paniscus"
HERVHF92	"Homo sapiens,Pan paniscus"
HERVHF93	"Homo sapiens,Pan paniscus"
HERVHF94	"Homo sapiens,Pan paniscus"
HERVHF95	"Homo sapiens,Pan paniscus"
HERVHF96	"Homo sapiens,Pan troglodytes"
HERVHF97	"Homo sapiens,Pan troglodytes"
HERVHF98	"Homo sapiens,Pan troglodytes"
HERVHF99	"Homo sapiens,Pan troglodytes"
HERVHF100	"Homo sapiens,Pan troglodytes"
HERVHF101	"Homo sapiens,Pongo abelii"
HERVHF102	"Homo sapiens,Pongo abelii"
HERVHF103	"Homo sapiens,Pongo abelii"
HERVHF104	"Homo sapiens,Pongo abelii"
HERVHF105	"Homo sapiens,Pongo abelii"
HERVHF106	"Homo sapiens,Pongo abelii"
HERVHF107	"Homo sapiens,Pongo abelii"
HERVHF108	"Homo sapiens,Pongo abelii"
HERVHF109	"Homo sapiens,Pongo abelii"
HERVHF110	"Homo sapiens,Pongo abelii"
HERVHF111	"Homo sapiens,Pongo abelii"
HERVHF112	"Homo sapiens,Pongo abelii"
HERVHF113	"Homo sapiens,Pongo abelii"
HERVHF114	"Homo sapiens,Pongo abelii"
HERVHF115	"Homo sapiens,Pongo abelii"
HERVHF116	"Homo sapiens,Pongo abelii"
HERVHF117	"Homo sapiens,Pongo abelii"
HERVHF118	"Homo sapiens,Gorilla gorilla gorilla"
HERVHF119	"Homo sapiens,Gorilla gorilla gorilla"
HERVHF120	"Homo sapiens,Gorilla gorilla gorilla"
HERVHF121	"Homo sapiens,Pan paniscus,Gorilla gorilla gorilla"
HERVHF122	"Homo sapiens,Pan paniscus,Gorilla gorilla gorilla"
HERVHF123	"Homo sapiens,Pan paniscus,Gorilla gorilla gorilla"
HERVHF124	"Homo sapiens,Pan troglodytes,Gorilla gorilla gorilla"
HERVHF125	"Homo sapiens,Pan troglodytes,Gorilla gorilla gorilla"
HERVHF126	"Homo sapiens,Pan troglodytes,Gorilla gorilla gorilla"
HERVHF127	"Homo sapiens,Pongo abelii,Gorilla gorilla gorilla"
HERVHF128	"Homo sapiens,Pongo abelii,Gorilla gorilla gorilla"
HERVHF129	"Homo sapiens,Pongo abelii,Gorilla gorilla gorilla"

HERVHF130	"Homo sapiens,Pongo abelii,Gorilla gorilla gorilla"
HERVHF131	"Homo sapiens,Pongo abelii,Gorilla gorilla gorilla"
HERVHF132	"Homo sapiens,Pongo abelii,Gorilla gorilla gorilla"
HERVHF133	"Homo sapiens,Pongo abelii,Gorilla gorilla gorilla"
HERVHF134	"Homo sapiens,Pongo abelii,Gorilla gorilla gorilla"
HERVHF135	"Homo sapiens,Pongo abelii,Gorilla gorilla gorilla"
HERVHF136	"Homo sapiens,Pongo abelii,Gorilla gorilla gorilla"
HERVHF137	"Homo sapiens,Pongo abelii,Gorilla gorilla gorilla"
HERVHF138	"Homo sapiens,Pongo abelii,Gorilla gorilla gorilla"
HERVHF139	"Homo sapiens,Pongo abelii,Gorilla gorilla gorilla"
HERVHF140	"Homo sapiens,Pongo abelii,Gorilla gorilla gorilla"
HERVHF141	"Homo sapiens,Hylobates pileatus,Pongo abelii"
HERVHF142	"Homo sapiens,Pan troglodytes"
HERVHF143	"Homo sapiens,Pan troglodytes"
HERVHF144	"Homo sapiens,Pan paniscus,Pan troglodytes"
HERVHF145	"Homo sapiens,Pan paniscus,Pongo abelii"
HERVHF146	"Homo sapiens,Pan paniscus,Pongo abelii"
HERVHF147	"Homo sapiens,Pan paniscus,Pongo abelii"
HERVHF148	"Homo sapiens,Hylobates pileatus,Gorilla gorilla gorilla"
HERVHF149	"Homo sapiens,Hylobates pileatus,Gorilla gorilla gorilla"
HERVHF150	"Homo sapiens,Hylobates pileatus,Gorilla gorilla gorilla"
HERVHF151	"Homo sapiens,Pan paniscus,Pongo abelii,Gorilla gorilla gorilla"
HERVHF152	"Homo sapiens,Pan troglodytes,Trachypithecus crepusculus,Gorilla gorilla gorilla"
HERVHF153	"Homo sapiens,Hylobates pileatus,Pan paniscus"
HERVHF154	"Homo sapiens,Hylobates pileatus,Pongo abelii"
HERVHF155	"Homo sapiens,Pan paniscus,Pongo abelii"
HERVHF156	"Homo sapiens,Pan paniscus,Pan troglodytes,Pongo abelii"
HERVHF157	"Homo sapiens,Hoolock leuconedys,Nomascus siki,Pongo pygmaeus,Gorilla gorilla gorilla"
HERVHF158	"Homo sapiens,Hylobates pileatus,Pongo abelii,Gorilla gorilla gorilla"
HERVHF159	"Homo sapiens,Hylobates pileatus,Pongo abelii,Gorilla gorilla gorilla"
HERVHF160	"Homo sapiens,Cercopithecus albogularis,Cercocebus atys,Lophocebus aterrimus,Macaca silenus,Mandrillus leucophaeus,Pan paniscus,Pygathrix nigripes,Papio hamadryas"
HERVHF161	"Homo sapiens,Cercocebus atys,Cercopithecus albogularis,Cercopithecus mona,Chlorocebus aethiops,Chlorocebus sabaeus,Colobus guereza,Erythrocebus patas,Lophocebus aterrimus,Macaca mulatta,Macaca silenus,Mandrillus leucophaeus,Pongo abelii,Pygathrix nigripes,Rhinopithecus roxellana,Rhinopithecus strykeri,Theropithecus gelada"
HERVIPADP1	"Homo sapiens,Pan troglodytes,Pongo abelii,Gorilla gorilla gorilla"
HERVK1	"Homo sapiens,Pan paniscus,Pan troglodytes"
HERVK2	"Homo sapiens,Hylobates pileatus,Pan paniscus"
HERVK3	"Homo sapiens,Pan paniscus,Pan troglodytes,Pongo abelii,Pongo pygmaeus,Gorilla gorilla gorilla"

HSERVIII1	"Homo sapiens,Gorilla gorilla gorilla"
HSERVIII2	"Homo sapiens,Gorilla gorilla gorilla"
HUERSP1	"Homo sapiens,Cercocebus atys,Macaca mulatta,Macaca nemestrina,Macaca silenus,Mandrillus sphinx"

Supplementary data S4 The alignments of the 5LTR and flanking sequences of a widely vertical transmitted HERV-H in 17 species

>Cercocebus_atys-HERVHF_3LTR+flanking(-)

---ATA
TGTGGCTTGTCTTTTCATCCCTTA-----ACAGTACCTCTTACAACCAGATGTGATGAATTTTAAT
GAAGCCAAACTTACCAATTTTTCT----------TTCATGGATCATGCTTTTGGTGTCGTATCTAAA
AAGTCATTGCCAAACCCTTAGGCTTTCTCTTATGTTATCCTTAAAAGTTTAATAGGTGTGTGG
TTTACACTTAGGTTTATCATCCCGTTTTGACTTA-TTTTTTGTGAAGCGTGTAAGTCTGTGTCT
ATATGCATTTGTTGGCATGTGTATCTGGTCATCACAGCACTGTTTGTTGAAAAGACTGTATTT
CTCCATTAGGCTGCCTTTGCTCTTTTGTCAAAGATCAGTCGATTACATTTGTAGAGTCTATTT
CTGGGCTCTCTGATCTGGAGGAAAAAGGTCTGTGATTGCCAGGGATTTGGAGGGACAAAA
GGCATGATCTTGGCTCACTGCAACCTCCACCT-CCCAGGTTCAAGGGATTCTCCTGCCTCAG
CCTCCTGAGTAGCTAGGATTTCAGGTGTGTACCACCATGCCCTGATAA-TTTTTGTATTTTGT
CTATTATTTTTCTAATACCACACTGTCTGTTACTGCAGCTTTGTAGCAAGTCTTGAAGTCCTC
TAAGTTTTTCTCCTAGATGTTATATTAACTATTCTGAGCCTTGCCTTTCAATCTACGTTTGAGA
ATGTGATTGTCAATATTCACAAAATAACTTGCTGGGACTTTGATTGGGATTGCACCGAATCT
GTGGATCAAATTGGGAAGAACTGATAGCAATATCGTGTCTTTCTATCCATAAACATGGAATAT
CTTTCCATTTATTTAGATCTTTTGTTTATTTCATTAGAGTTTTGTAGTTCTCCTCACATAGATTT
TGAACATGTGTTGTTAGATTTGTACCTAAGTATTTCATTTTTCTTGGTGCTAATATAAATAGTA
TTATCTTTTTAATATCGAATTTCAACTAGTCATTATCGATATATAGAAAAGCAATGACTTGTGT
ATATTAATCTTGCATCTTGCAACCTTGTTATAATCATTTATTAGTCTCAGGAGGG-TTTTTTGTT
GATGTGGGGGGATTTCTACGGATACAATCATGTCATCTT-AAAAAAAAGACG--TTCATTTCT
TCCTTTCTAATCTGTGTGCCTTTTATTTCTTTCCTTGTCTTATTGCATTCGCTAGGACTTTCA
GTACAATGTTAAAAATCAGTGGTATAAATGTAAATAATTACTATTTATTGTGAAAGAAAAATA
TCTTGGGCCCCCAACATCACTAAGCTAAAGGGAAAAGTCAAGCTGGGAACTGCTTAGGGTA
AACCTGCCTCCCATTCTATTCAAAGTCATCCCTCTGCTCACTGAGATAAATGCATATCTGATT
GCCTCCTTTGGAGAGGCTCATCAGAAATGCAGAAGAATGCAACCATTTGTCTCTCACCTGC
TTGTGACCTGGAAGCTCCCTCCCCCCTTCAAGTTGTGACCTGGAAGCCTCCTCCCCCCTTC
ACGTTGTCCTGACTTTGCATAGATTGTGGACCAAACCAATGTTCATGTTACACATGTTGATT
GATGTCTCCTCTCTCCCTAAAATATGTAAAACCAAGCTGTGTTCTGACTGTCAGGCCTCTGA
GCCCAAGCTAAGCCATCATATCCCCTGTGACTTGCACATACACATCCAGATGGCCTGAAGTA
ACTGAAG-GATTACAAAAGAAGTGAAAATGGCCTGTTCCTGCCTTAAC--TGATGACATTACC
TTATG---AAATTCCTTCTCCTGGCTCATCCTGGCTCAAAAGTTCCCCCACTGAGCACCTTGTG
ACCCCCACCCCTGCCAGCCAGAAAACAACCCCCTTTGACTGTAATTTTCCACCACCCACCC
AAATCTTATAAAACAGCCCCA-CCCTATCTCCCTTCGCTGACTCTCTTTTCGGATCCAGCCCG
CCTGCACTCAGGTGAAATAAACAGCCTTGTTGCTCACACAAAGCCTGTTTGGTGGTCTCTT
CACACAGAC---------------
>Cercopithecus_mitis-HERVHF_3LTR+flanking(-)

--ATCTTTTTCTT
CCAATATGTGGCTTGTCTTTTCATCCCTTA-----ACAGTACCTCTCACAACCAGATGTGATGAA

TTTTAATGAAGCCAAACTTTTTGTTTTTCT----------TTCATGGATCATGCTTTTGGTGTCGTA
TCTAAAAAGTCATTGCCAAACCCTTAGGCTTTCTCTTATGTTATCCTTAAAAGTTTAATAGGT
TTGTGGTTTACACTTAGGTTTATGATCCCCTTTTGACTTA-TTTTTTGTGAAGCGTGTAAGTCT
GTGTCTATATGCATTTGTTGGCATGTGTATCTGGTCATCACAGCACTGTTTGTTGAAAAGACT
ATATTTCTCCATTAGGCTGCCTTTGCTCTTTTGTCAAAGATCAGTCGATTATAATTGTAGAGTC
TATTTCTGGGCTTTCTGATCTGGAGGAAAAAGATCTGTGGTTGCCAGGGATTTGGAGGGAC
AAAAGGCATGATCTTGGCTCACTGCAACCTCCACCTCCCCGGGTTCAAGGGATTCTCCTGC
CTCAGCCTCCTGAGTAGCTAGGATTTCAGGTGCGTACCACCATGCCCTGCTAATTTTTTGTAT
TTTGTCTATTATTTTTCTAATACCACACTGTCTGTTACTGCAGCTTTGTAGCAAGTCTTGAAG
TCCTCTAAGTTTTTCTCCTAGATGTTATATTAACTATTCTGAGCTTTGCCTTTCAATCTACGTT
TGAGAATGTGATTGTCAATATTCACAAAATAACTTGCTGGGATTTTGATTGTGATTGCACCG
AATCTGTGGATCAAATTGGGAAGAACTGATAATAATATCGTGTCTTTCTATCCATAAACATGG
AATATCTTTCCATTTATTTAGATCTTTTGTTTATTTCATTAGAGTTTTGTAGTTCTCCTCACATA
GATTTTGAACATGTGTTGTTAGATTTGTACCTAAGTATTTCATTTTTCTTGGTGCTAATATAAA
TAGTATTATCTTTTTAATATCGAATTTCAACTAGTCATTATCGATATATAGAAAAGCAATGACT
TGTGTATATTAATCTTGCATCTTGCAACCTTGTTATAATCATTTATTAGTCTCAGGAGGG-TTTT
TTGTTGATGTGGGGGGATTTTCTACGGATACAATCATGTCATCTT-AAAAAAAAGACG--TTCA
TTTCTTCCTTTCTAATCTGTGTGCCTTTTATTTTCTTTCCTTGTCTTATTGCATTCGCTAGGACT
TTCAGTACAATGTTAAAAATCAGTGGTATAAATGTAAATAATTACTATTTCTTGTGAAAGAAA
AATATCTTGGGCCCCCAACATCACTAAGCTAAAGGGAAAAGTCAAGCTGGGAACTGCTTAG
GGTAAACCTGCCTCCCATTCTATTCAAAGTCATCCCTCTGCTCACTGAGATAAATGCATATCT
GATTGCCTCCTTTGGAGAGGCTTATCAGAAATGCAGAAGAATGCAACCATTTGTCTCTCACC
TACTTGTGACCTGGAAG--------------------------------CCCCCTCCCCCCTTCAAGTTGTCCTGACT
TTGCATAGATTGTGGACCAAACCAATGTTCATGTTACACATGTTGATTGATGTCTCCTCTCTC
CCTAAAATATGTAAAACCAAGCTGTGTTCTGACTGTCAGGCCTCTGAGCCCAAGCTAAGCC
ATCATATCCCCTGTGACCTGCACATATACATCCAGATGGCCTGAAGTAACTGAAG-GATCACA
AAAGAAGTGAAAATGGCCTGTTCCTGCCTTAAC--TGATGACATTACCTTATG---AAATTCCTT
CTCCTGTCTCATCCTGGCTCAAAAGCTCTCCCACTGAGCACCTCGTGACCCCCACCCATGCC
AGCCAGAAAACAACCCCCTTTGACTGTAATTTTCCTCTACCTACCCAAATATTATAAAACAG
CCCCACCCCTATCTCCCTTCGCTGACTCTCTTTTCGGATCCAGCCCGCCTGCACCCAGGTGA
AATAAACAGCCTTGTTGCTCACACAAAGCCTGTTTGGTGGTCTCTTCACACAGATGGTGTG
ACTCAGATC
>Cercopithecus_mona-HERVHF_3LTR+flanking(-)

--TTATCACACGTGTGGTTTGAA
AATCTTTTTCTTCCAATATGTGGCTTGTCTTTTCATCCCTTA-----ACAGTACCTCTCACAACCA
GATGTGATGAATTTTAATGAAGCCAAACTTACCAAGTTTTTCT----------TTCATGGATCATGCT
TTTGGTGTCGTATCTAAAAAGTCATTGCCAAACCCTTAGGCTTTCTCTTATGTTATCCTTAAA
AGTTTAATAGGTTTGTGGTTTACACTTAGGTTTATGATCCCGTTTTGACTTA-TTTTTTGTGAA
GCGTGTAAGTCTGTGTCTATATGCATTTGTTGGCAAGTGTATCTGGTCATCATAGCACTGTTT
GTTGAAAAGACTATATTTCTCCATTAGGCTGCCTTTGCTCTTTTGTCAAAGATCAGTGGATTA
TATTTGTAGAGTCTATTTCTGGGCTCTCTGATCTGGAGGAAAAAGATCTGTGGTTGCCAGGG
ATTTGGAGGGACAAAAGGCATGATCTTGGCTCACTGCAACCTCCACCT-CCCGGGTTCAAG
GGATTCTCCTGCCTCAGCCTCCTGAGTAGCTAGGATTTCAGGTGCGTACCACCATGCCCTGC

TAA-TTTTTGTATTTTGTCTATTATTTTTCTAATACCACACTGTCTGTTACTGCAGCTTTGTAGC
AAGTCTTGAAGTCCTCTAAGTTTTTCTCCTAGATGTTATATTAACTATTCTGAGCCTTGCCTTT
CAATCTACGTTTGAGAATGTGATTGTCAATATTCACAAAATAACTTGCTGGGATTTTGATTGG
AATTGCACCGAATCTGTGGATCAAACTGGGAAGAACTGGTAATAATATCGTGTCTTTCTATC
CATAAACATGGAATATCTTTCCATTTATTTAGATCTTTTGTTTATTTCATTAGAGTTTTGTAGTT
CTCCTCACATAGATTTTGAACATGTGTTGTTAGATTTGTACCTAAGTATTTCATTTTTCTTGGT
GCTAATATAAATAGTATTATCTTTTTAATATCGAATTTCAACTAGTCATTATCGATATATAGAAA
AGCAATGACTTGTGTATATTAATCTTGCATCTTGCAACCTTGTTATAATCATTTATTAGTCTCA
GGAGGG-TTTTTTGTTGATGTGGGGGGATTTCTACGGATACAATCATGTCATCTT-AAAAAA
AAGACG--TTCATTTCTTCCTTTCTAATCTGTGTGCCTTTTATTTT-----CTTGTCTTATTGCATTC
GCTAGGACTTTCAGTACAATGTTAAAAATCAGTGGTATAAATGTAAATAATTACTATTTATTGT
GAAATAAAAATATCTTGGGCCCCCAACATCACTAAGCTAAAGGGAAAAGTCAAGCTGGGAA
CTGCTTAGGGTAAACCTGCCTCCCATTCTATTCAAAGTCATCCCTCTGCTCACTGAGATAAAT
GCAGATCTGATTGCCTCCTTTGGAGAGGCTCATCAGAAATGCAGAAGAATGCAACCATTTG
TCTCTCACCTACTTGTGACCTGGAAG------------------------------CCCCCTCCCCCCTTCAAGTT
GTCCTGACTTTGCATAGATTGTGGACCAAACCAATGTTCATGTTACACATGTTGATTGATGTC
TCCTCTCTCCCTAAAATATGTAAAACCAAGCTGTGTTCTGACTGTCAGGCCTCTGAGCCCAA
GCTAAGCCATCATATCCCCTGTGACCTGCACATATACATCCAGATGGCCTGAAGTAACTGAA
G-GATCACAAAAGAAGTGAAAATGGCCTGTTCCTGCCTTAAC--TGATGACATTACCTTATG---
AAATTCCTTCTCCTGTCTCATCCTGGCTCAAAAGCTCTCCCACTGAGCACTTCGTGACCCCC
ACCCCTGCCAGCCGGAAAACAACCCCCTTTGACTGTAATTTTCCACTACCTACCCAAATATT
ATAAAACAGCCCCACCCCTATCTCCCTTCGCTGACTCTCTTTTCGGATCCAGCCCGCCTGCA
CCCAGGTGAAATAAACAGCCTTGTTGCTCACACAAAGCCTGTTTGGTGGTCTCTTCACACA
GAC--------------
>Chlorocebus_aethiops-HERVHF_5LTR+flanking(+)

---CTTTATCACACGTGTGGTTTGA
AAATCTTTTTCTTCCAATATGTGGCTTGTCTTTTCATCCCTTA-----ACAGTACCTCTCACAACC
AGATGTGATGAATTTTAATGAAGCCAAACTTACCAATTTTTTCT---------TTCATGGATCATGC
TTTTGGTGTCGTACCTAAAAAGTCACTGCCAAACCCTTAGGCTTTCTCTTATGTTATCCTTAA
AAGTTTAATAGGTTTGTGGTTTACACTTAGGTTTATGATCCCGTTTTGATTTA-TTTTTTGTGA
AGCGTGTAAGTCTGTGTCTATGTACATTTGTTGGCATGTGTATCTGGTCATCACAGCGCTGTT
TGTTGAAAAGACTATATTTCTCCATTAGGCTGCCTTTGCTCTTTTGTCAAAGATCAGTCGATT
ATATTTGTAGAGTCTATTTCTGGGCTCTCTGATCTGGAGGAAAAAGATCTGTGGTTGCCAGG
GATTTGGAGGGACACAAGGCATGATCTTGGCTCACTGCAACCTCCACCT-CCCGGGTTCAAG
GGATTCTCCTCCCTCAGCCTCCTGAGTAGCTAGGATTTCAGGTGCATACCACCATGCCCTGC
TAA-TTTTT-TATTTTGTCTATTATTTTTCTAATACCACACTGTCTGTTACTGC-------TAGCAAGT
CTTGAAGTCCTCTAAGTTTTTCTCCTAGATGTTATATTAACTATTCTGAGCCTTGCCTTTCAAT
CTACGTTTGAGAATGTGATTGTCAATATTCACAAAATAACTTGCTGGGATTTTGATTGGGATT
GCATCGAATCTGTGGATCAAATTGGGAAGAACTGATAATAATATCGTGTCTTTCTATCCATAA
ACATGGAATATCTTTCCATTTATTTAGATCTTTTGCTTATTTCATTAGAGTTTGGTAGTTCTCCT
CACATAGATTTTGAACATGTGTTGTTAGATTTGTACCTAAGTATTTCATTTTTCTTGGTGCTAA
TATAAATAGTATTATCTTTTTAATATCGAATTTCAACTAGTCATTATCGATATATAGAAAAGCAA
TGACTTGTGTATATTAATCTTGCATCTTGCAACCTTGTTATAATCATTTATTAGTCTCAGGAGG

G-TTTTTTGTTGATGTGGGGGGATTTTCTACGGATACAATCATGTCATCTT-TAAAAAAAGACG
--TTCATTTCTTCCTTTCTAATCTGTGTGCCTTTTATTTTCTTTCCTTGTCTTATTGCATTCACTA
GGACTTTCAGTACAATGTTAAAAATCAGTGGTATAAATGTAAATAATTACTATTTATTGTGAA
AGAAAAATATCTTGGGCCCCCAACATCACTAAGCTAAAGGGAAAAGTCAAGCTGGGAACT
GCTTAGGGTAAACCTGCCTCCCATTCTATTCAAAGTCATCCCTCTGCTGGCTGAGATAAATG
CATATCTGATTGCCTCCTTTAGAGACGCTCATCAGAAATGCAGAAGAATGCAACCATTTGTC
TCTCACCTACTTGTGACCTGGAAG-------------------------------------ACGCCTCCCCTCTTCAAGTTGT
CCTGACTTTGCATAGATTGTGGACCAAACCAATGTTCATGTTACACATGTTGATTGATGTCTC
CTCTCTCCCTAAAATATGTAAAACCAAGCTGTGTTCTGACTGTCAGGCCTCTGAGCCCAAGC
TAAGCCATCATATCCCCTGTGACCTGCACATATACATCCAGATGGCCTGAAGTAACTGAAG-G
ATCACAAAAGAAGTGAAAATGGCCTGTTCCTGCATTAAC--TGATGACATTACCTTATG---AA
ATTCCTTCTCCTGTCTCATCCTGGCTCAAAAGCTCTCCCACTGAGCACCTCGTGACCCCCAC
CCCTGCCAGCCGGAAAACAACCCCTTTTGACTGTAATTTTCCACTACCTACCCAAATATTATA
AAACAACCCCACCCCTATCTCCCTTCGCTGACTCTCTTTTCGGATCCAGCCCGCCTCCACCT
AGGTGAAATAAACAGCCTTGTTGCTCCCACAAAGCCTGTTTGGTGGTCTCTTCACACAGAC
G--------------
>Chlorocebus_sabaeus-HERVHF_3LTR+flanking(-)

--CTTTATCACACGTGTGGTTTGA
AAATCTTTTTCTTCCAATATGTGGCTTGTCTTTTCATCCCTTA-----ACAGTACCTCTCACAACC
AGATGTGATGAATTTTAATGAAGCCAAACTTACCAATTTTTTCT----------TTCATGGATCATGC
TTTTGGTGTCGTACCTAAAAAGTCACTGCCAAACCCTTAGGCTTTCTCTTATGTTATCCTTAA
AAGTTTAATAGGTTTGTGGTTTACACTTAGGTTTATGATCCCGTTTTGATTTATTTTTTTGTGA
AGCGTGTAAGTCTGTGTCTATGTGCATTTGTTGGCATGTGTATCTGGTCATCACAGCGCTGTT
TGTTGAAAAGACTATATTTCTCCATTAGGCTGCCTTTGCTCTTTTGTCAAAGATCAGTCGATT
ATATTTGTAGAGTCTATTTCTGGGCTCTCTGATCTGGAGGAAAAAGATCTGTGGTTGCCAGG
GATTTGGAGGGACACAAGGCATGATCTTGGCTCACTGCAACCTCCACCT-CCCGGGTTCAAG
GGATTCTCCTCCCTCAGCCTCCTGAGTAGCTAGGATTTCAGGTGCATACCACCATGCCCTGC
TAA-TTTTT-TATTTTGTCTATTATTTTTCTAATACCACACTGTCTGTTACTGC-------TAGCAAGT
CTTGAAGTCCTCTAAGTTTTCTCCTAGATGTTATATTAACTATTCTGAGCCTTGCCTTTCAAT
CTACGTTTGAGAATGTGATTGTCAATATTCACAAAATAACTTGCTGGGATTTTGATTGGGATT
GCATCGAATCTGTGGATCAAATTGGGAAGAACTGATAATAATATCGTGTCTTTCTATCCATAA
ACATGGAATATCTTTCCATTTATTTAGATCTTTTGCTTATTTCATTAGAGTTTTGTAGTTCTCCT
CACATAGATTTTGAACATGTGTTGTTAGATTTGTACCTAAGTATTTCATTTTTCTTGGTGCTAA
TATAAATAGTATTATCTTTTTAATATCGAATTTCAACTAGTCATTATCGATATATAGAAAAGCAA
TGACTTGTGTATATTAATCTTGCATCTTGCAACCTTGTTATAATCATTTATTAGTCTCAGGAGG
G-TTTTTTGTTGATGTGGGGGGATTTTCTACGGATACAATCATGTCATCTT-AAAAAAAAGAC
G--TTCATTTCTTCCTTTCTAATCTGTGTGCCTTTTATTTTCTTTCCTTGTCTTATTGCATTCACT
AGGACTTTCAGTACAATGTTAAAAATCAGTGGTATAAATGTAAATAATTACTATTTATTGTGA
AAGAAAAATATCTTGGGCCCCCAACATCACTAAGCTAAAGGGAAAAGTCAAGCTGGGAAC
TGCTTAGGGTAAACCTGCCTCCCATTCTATTCAAAGTCATCCCTCTGCTGGCTGAGATAAAT
GCATATCTGATTGCCTCCTTTAGAGACGCTCATCAGAAATGCAGAAGAATGCAACCATTTGT
CTCTCACCTACTTGTGACCTGGAAG-------------------------------------ACGCCTCCCCTCTTCAAGTTG
TCCTGACTTTGCATAGATTGTGGACCAAACCAATGTTCATGTTACACATGTTGATTGATGTCT

CCTCTCTCCCTAAAATATGTAAAACCAAGCTGTGTTCTGACTGTCAGGCCTCTGAGCCCAAG
CTAAGCCATCATATCCCCTGTGACCTGCACATATACATCCAGATGGCCTGAAGTAACTGAAG-
GATCACAAAAGAAGTGAAAATGGCCTGTTCCTGCATTAAC--TGATGACATTACCTTATG---A
AATTCCTTCTCCTGTCTCATCCTGGCTCAAAAGCTCTCCCACTGAGCACCTCGTGACCCCCA
CCCCTGCCAGCCGGAAAACAACCCCCTTTGACTGTAATTTCCACTACCTACCCAAATATTAT
AAAACAACCCCACCCCTATCTCCCTTCGCTGACTCTCTTTTCGGATCCAGCCCGCCTCCACC
CAGGTGAAATAAACAGCCTTGTTGCTCCCACAAAGCCTGTTTGGTGGTCTCTTCACACAGA
C--------------
>Colobus_guereza-HERVHF_3LTR+flanking(-)

--GAACCTCACCCGAGAGCGCAGCGTG
ACTTC------------------------CCTGGCTCCCCACACCTGAGGACCAGAGAACCTCACC-GAGAGT
GTGTGCATATTTGCAATAA----AAGGCTGCCGCTTTCTTATGTACTTTGGCCTCATGTTTAATT
ACTTAGCTCTCCTAAATTAAGTTACATTAAATTAAATTAAGA----CACTTACGTTATCCTTAAA
AGTTTAATAGTTTTGTGGTTTACACTTAGGTTTATCATCCCGTTTTGACTTA-TTTTTTGTGAA
GTGTGTAAGTCTGTGTCTATATGCATTTGTTGGCAGGTGTATCTGGTCATCACAGCACCGTTT
GTTGAAAAGACTATCTTTCTCCATTAGATTGCCTTTGCTCTTTTGTCAAAGATCAGTCAATTG
TATTTGTAGAGTCTATTTCTGGGCTCTCTGATCTGGAGGAAAAAGTTTTGTGGTTGCCAGAG
ATTTGGAGGGACACAAGGCATGATCTTCACTCACTGCAACCTCCACTT-CCCAGGTTCAAGG
GATTCTCCTGCCTCAGCCTCCTGAGTAGCTAGGATTTCAGGTGCATACCACCATGCCCTGCT
AA-TTTTTGTATTTTGTCTATTATTTTTCTAATACCACACTGTCTGTTACTGCAGCTTTGTAGCA
AGTCTTGAAGTCCTCCAAGTTTTTCTCCTGGATGTTATATTAACTATTCTGAGCCATACCTTT
CAATCTACGTTTGAGAATGTGATTGTCAATATTCACAAAATAACTTGCTGGGATTTTGATTGG
GATTGCACTGAATCTGTGGATCAAATTGGGAAGAACTGATAACAATATTGTGTCTTTCTATCC
ATAAACATGGAATATCTTTCCATTTATTTAGATCTTTTATTTATTTCATTAGAATTTTGTAGTTC
TCCTCACATAGATTTTGAACATATGTTGTTAGATTTGTACCTAAGTATTTCATTTTTCTTGGTG
CTAATATAAATAGCATTATCTTTTTAATACCAAATTTCAACTATTCATTATCGACATATAGAAAA
GCAATGACTTGTATATATTAATCTTGCATCTTGCAACCTTGTTATAATCATTTATTAGTCTCAG
GAGGG-TTTTTTGTTGATGTGGAGGGATTTTCTATGGACACAATCATGTCATCTT-AAAAAAA
AGACG--TTCATTTCTTCCTTTCTAATCTGTGTGCCTTTTATTTTCTTTCCTTGTCTTATTGCATT
AGCTAGGACTTTCAGTACAATGTTAAAAATCAGTGGTATAAATGTAAATAATTACTATTTATT
GTGAAAGAAAAATATCTTGGGCCCCCAACATCACTAAGCTAAAGGGAAAAGTCAAGCTGG
GAACTGCTTAGGGTAAACCTGCCTCCCATTCTATTCAAAGTCATCCCTGTGCTCACTGAGAT
AAATGCATATCTGATTGCCTCTTTTGGAGAGGCTCATCAGAAATGCAGAAGAATGCAACCAT
TTTTCTCTCACCTACTTGTGACCTGGAAG----------------------------------CCCCTCCCCGCTTCAA
GTTGTCCTGAATTTGCTTAGATTGTGGACCAAACCAATGTTCATGTTACACATGTTGATTGAT
GTCTCCTGTCTCCCTAAAATATTTAAAACCAAGCTGTGTTCTGACTGTCAGGCCTCTGAGCC
CAAGCTAAGCCATCATATCCCCTGTGACCTGCAC-TATACATCCAGATGGCCTGAAGTAACTG
AAG-GATCACAAAAGAAGTGAAAATGACCTGTTCCTGCCTTAAC--TGATGACATTACCTTGT
G---AAATTCCTTCTCCTGGCTCATCCTGGCTCAAAAGCTCTCCCACTGAGCACCTTGTGAAC
CCCACCCCTGCCAGCCAGGAAACAACCCCCTTTGACTGTAATTTCCACTACCTACCCAAAT
ATTATAAAACAGCCCCGCCCCTATCTCCCTT-GCTGACTCTCTTCTCAGATTCAGCCTGCCTG
CACCCAGGTGAAATAAACAGCCTTGTTGCTCACACAAAGTCTGTTTGGTGGTCTCTTCACA
CAGATGCTAGT---------

>Erythrocebus_patas-HERVHF_3LTR+flanking(-)

--CCTTTATCACACGTGTGGTTTGA
AAATCTTTTTCTTCCAATATGTGGCTTGTCTTTTCATCCCTTA-----ACAGTACCTCTCACAACC
AGATGTGATGAATTTTAATGAAGCCAAACTTACCAATTTTTTCT----------TTCATGGATCATGC
TTTTGGTGTCGTATCTAAAAAGTCATTGCCAAACCCTTAGGCTTTCTCTTATGTTATCCTTAA
AAGTTTAATAGGTTTGTGGTTTACACTTAGGTTTATGATCCCGTTTTGACTTA-TTTTTTGTGA
AGCGTGTAAGTCTGTGTCTATATGCATTTGTTGGCATGTGTATCTGGTCATCACAGCACTGTT
TGTTGAAAAGACTATATTTCTCCATTAGGCTGCCTTTGCTCTTTTGTCAAAGATCAGTCGATT
ATATTTGTAGAGTCTATTTCTGGGCTCTCTGATCTGGAGGAAAAAGATCTGTGGTTGCCAGG
GATTTGGAGGGACACAAGGCATGATCTTGGCTCACTGCAACCTCCACCT-CCCGGGTTCAAG
GGATTCTCCTCCCTCAGCCTCCTGAGTAGCTAGGATTTCAGGTGCGTACCACCATGCCCTGC
TAA-TTTTTGTATTTTGTCTATTATTTTTCTAATACCACACTGTCTGTTACTGC-------TAGCAAG
TCTTGAAGTCCTCTAAGTTTTTCTCCTAGATGTTATATTAACTATTCTGAGCCTTGCCTTTCAA
TCTACGTTTGAGAATGTGATTGTCAATATTCACAAAATAACTTGCTGGGATTTTGATTGGGAT
TGCACTGAATCTGTGGATCAAATTGGGAAGAACTGATAATAATATCGTGTCTTTCTATCCATA
AACATGGAATATCTTTCCATTTATTTAGATCTTTTGCTTATTTCATTAGAGTTTTGTAGTTCTCC
TCACATAGATTTTGAACATGTGTTGTTAGATTTGTACCTAAGTATTTCATTTTTCTTGGTGCTA
ATATAAATAGTATTATCTTTTTAATATCGAATTTCAACTAGTCATTATC-ATATATAGAAAAGCA
ATGACTTGTGTATATTAATCTTGCATCTTGCAACCTTGTTATAATCATTTATTAGTCTCAGGAG
GG-TTTTTTGTTGATGTGGGGGGATTTCTACGGATACAATCATGTCATCTT-AAAAAAAAGA
CG--TTCATTTCTTCCTTTCTAATCTGTGTGCCTTTTATTTTCTTTCCTTGTCTTATTGCATTCAC
TAGGACTTTCAGTACAATGTTAAAAATCAGTGGTATAAATGTAAATAATTACTATTTATTGTG
AAAGAAAAATATCTTGGGCCCCCAACATCACTAAGCTAAAGGGAAAAGTCAAGCTGGGAA
CTGCTTAGGGTAAACCTGCCTCCCATTCTATTCAAAGTCATACCTCTGCTGGCTGAGATAAAT
GCATGTCTGATTGCCTCCTTTAGAGATGCTCATCAGAAATGCAGAAGAATGCAACCATTTGT
CTCTCACCTACTTGTGACCTGGAAG-------------------------------------ACCCCTCCCCCCTTCAAGTTG
TCCTGACTTTGCATAGATTGTGGACCAAACCAATGTTCATGTTACACATGTTAATTGATGTCT
CCTCTCTCCCTAAAATATGTAAAACCAAGCTGTGTTCTGACTGTCAAGCCTCTGAGCCCAAG
CTAAGCTATCATATCCCCTGTGACCTGCACATATACATCCAGATGGCCTGAAGTAACTGAAG-
GATCACAAAAGAAGTGAAAATGGCCTGTTCCTGCATTAAC--TGATGACATTACCTTATG---A
AATTCCTTCTCCTGTCTCATCCTGGCTCAAAAGCTCTCCCACTGAGCACCTTGTGACCCCCA
CCCCTGCCAGCCGGAAAACAACCCCCCTTTGACTGTAATTTTCCACTACCTACCCAAATATTAT
AAAACAACCCCACCCCTATCTCCCTTCGCTGACTCTCTTTTCGGATCCAGCCCGCCTCCACC
CAGGTGAAATAAACAGCCTTGTTGCTCCCACAAAGCCTGTTTGGTGGTCTCTTCACACAGA
C---------------
>Homo_sapiens-HERVHF_3LTR+flanking(-)
--TATGCATGTACCATAGTTTATTAATCCATCTCT
GCTTTGTATATCTTCTTTGACGAGGTGTCTGTCCAGATCTCTTGACCATTTTTAATTGGGTTG
TTTTCTTATCGTTGAGTTTTAGTTTTTTGTGTTTTAGACACGAGTCCTTTATCAGACGTGTGG
TTTGAACATCTTTTTACCCCAGTACGTGGCTTGTCTTTTCATCCCTTA-----GCAGTAACTCTC
ACAACCAGATGTGATGAATTTTAATGAAGCCAAACTTACCAATTTTTTCT----------TTCATGGA
TCATGCTTTTGGTGTCGTATCTAAAAAGTCATCACCAAACACTTAGGCTTTCTCTTATGTTAT
CCTTACAAGTTTAATAGTTTTGTGGTTTACACTTAGGTTTATGATCCCATTTTGACTTA-TTTTT

TGTGAAGGGTGTAAGTCTGTGTCTATATGCATTTGTTGGCATGTGTGTCTGGTCATCACAGC
ACCACTTATTGAAAAGACTATCTTTCTCCATTAGGTTGCCTTTGCTCTTTTGTCAAAGATCAG
TCGATTATATTTATAGAGCCTATTCCTGGGCTCTCTGATCTG---
---TTCCATTGCTCTG---
-------TTTGTCTATTATTTTGCCAATACCACACTGTCTGTTACTGCAGCTTTGTAGCAAGTCTTC
AGGTCCTCTAAGTTTTCCTCCTGGATGTTATATTAACTATTCTGAGCCTTGCCTTTCAGTCTA
AGTTGAAGAATGTGATTGTCAATATTCACCAAGTAACTTGCTGAGATTTTTATTGGGATTGC
ACTGAATCTATAGATCAAATTGGGAAGAACTGATAACAATATCATGTCTTTCTATCCATAAAC
ATGGAATATCTTTCCATTTATTTAGATCTTTTATTTATTTCATTAGAGTTTTGTAGTTCTTCTCA
CATAGATTTTGAACATGTGTTGTTAGATTTGTACCTAAGTATTTCATTTTTCTTGGTGCTAAT---
-----TATTATCTTTTTAATATCAAATTTCAACTATTCATTATTGATATATAGAAAAGCAATGACTT
GTATGTATTAATCTTGCATCTTGCAACCTTGTTACAATCATTTATTAGTCTCAGGAGGG-TTTT
TTGATG----TGGGGGATTTTCTACGGACACAATCACGTCATCGT-AAAAAAAAGGCACTTTAA
TTTCTTCCTTTCTAATCTGTATGCCTTTTATTTTCTTTCCTTGTCTTATTGCATTAGCTAGGACT
TTCAGTACAATGTTAAAAATCAGTTGTATAAATGTAAATAATTATTATTTATTGCGAAAGAAA
AATATCTTGGGCCCCCAAAATCACTAAGCGAAAGGGAAAAGTCAAACTGGGAACTGCTTAC
GGCAAACCTGCCTCCCATTCTATTCAAAGTCATCCCTCTGCTCACTGAAATAAATGCATATCT
GATTGCCTCCTTCGGAGAGGCTCATCAGAAACCCAGAAGAATGCAACCGTTTGTCTCTCAC
CTACTTGTGACCTGGAAG--------------------------------TAGCCTCCCCGCTTAGAGTTGTCCCACT
TTTGCTTAGATTCTGGACCAAACCAATGTTCATTTTACATATGTTGATTGATGTCTCATGTCTC
CCTAAAATGTATAAAACCAAGCTGTGTTCTGACTGTCAGGGCTCTGAGCACAAGCTAAGCC
ATCGTATCCCATGTGACCTGGAAGTATATATCCAGATGGCCTAAAGTAACTGAAG-AATCACA
AAAGAAGTGAAAATAGTCTGGTCCTGCCTTAAC--TGATGATATTACCTTGTG---AAATTCCTT
CTCCTGGCTCATCCTGGCTCAAAAGCTCCCCCACTGAGCACCTTGTGACCCCCACCCCTGC
CAGCCATAAAACAACCCCCTTTGACTGTAATTTTCCACTACCTACCTAAATCCTATAAAACG
GCCCCACCCCTATCTCCCTTCCCTGACTCTCTTTTCGGATTCAGCCCGCCTGCACCCAGGTG
AAATAAACAGCCTTGTTGCTCACACAAAGCCTGTTTGGTGGTCTCTTCATACAGACGCGAG
TGAA------
>Lophocebus_aterrimus-HERVHF_3LTR+flanking(-)

---ATCACACGTGTGGTTTGAAA
ATCTTTTTCTTCCAATATGTGGCTTGTCTTTTCATCCCTTA-----ACAGTACCTCTCACAACCAG
ATGTGATGAATTTTAATGAAGCCAAACTTACCAATTTTTTCT---------TTCATGGATCATGCTTT
TGGTGTCGTATCTAAAAAGTCATTGCCAAACCCTTAGGCTTTCTCTTATGTTATCCTTAAAAG
TTTAATAGGTGTGTGGTTTACACTTAGGTTTATGATCCCGTTTTGACTTA-TTTTTTGTGAAGC
GTGTAAGTCTGTGTCTATATGCATTTGTTGGCATGTGTATCTGGTCATCACAGCACTGTTTGT
TGAAAAGACTATATTTCTCCATTAGGCTGCCTTTGCTCTTTTGTCAAAGATCAGTCGATTATA
TTTGTAGAGTCTATTTCTGGGCTCTCTGATCTGGAGGAAAAAGGTCTGTGATTGCCAGGGAT
TTGGAGGGACAAAAGGCATGATCTTGGCTCACTGCAACCTCCACCT-CCCGGGTTCAAGGG
ATTCTCCTGCCTCAGCCTCCTGAGTAGCTAGGATTTCAAGTGCGTACCACCATGCCCTGATA
A-TTTTTGTATTTTGTCTATTATTTTTCTAATACCACACTGTCTGTTACTGCAGCTTTGTAGCAA
GTCTTGAAGTCCTCTAAGTTTTTCTCCTAGATGTTATATTAACTATTCTGAGCCTTGCCTTTCA
ATCTACATTTGAGAATGTGATTGTCAATATTCACAAAATAACTTGCTGGGATTTTGATTGGGA
TTGTACCGAATCTGTGGATCAAATTGGGAAGAACTGATAGCAATATCGTCTCTTTCTATCCAT

AAACATGGAATATCTTTCCATTTATTTAGATCTTTTGTTTATTTCATTAGAGTTTTGTAGTTCTC
CTCACATAGATTTTGAACATGTGTTGTTAGATTTGTACCTAAGTATTTCATTTTTCTTGGTGCT
AATATAAATAGTATTAGCTTTTTAATATCGAATTTCAACTAGTCATTATCGATATATAGAAAAG
CAATGACTTGTGTATATTAATCTTGTATCTTGCAACCTTGTTATAATCATTTATTAGTCTCAGG
AGGG-TTTTTTGTTGATGTGGGGGGATTTTCTACGGATGCAATCATGTCATCTT-AAAAAAAA
GACG--TTCATTTCTTCCTTTCTAATCTGTGTGCCTTTTATTTTCTTTCCTTGTCTTATTGCATTC
GCTAGGACTTTCAGTACAATGTTAAAAATCAGTGGTATAAATGTAAATAATTACTATTTATTGT
GAAAGAAAAATATCTTGGGCCCCCAACATCACTAAGCTAAAGGGAAAAGTCAAGCTGGGA
ACTGCTCAGGGCAAACCTGCCTCCCATTCTATTCAAAGTCATCCCTCTGCTCACTGAGATAA
ATGCATATCTGATTGCCTCCTTTGGAGAGGCTCATCAGAAATGCAGAAGAATGCAACCATTT
GTCTCTCACCTGCTTGTGACCTGGAAG-------------------------------CTCCCTCCCCGCTTCAAGT
TGTCCTGACTTTGCATAGATTGTGGACCAAACCAATGTTCATGTTACACATGTTGATTGATGT
CTCCTCTCTCCCTAAAATACGTAAAACCAAGCTGTGTTCTGACTGTCAGGCCTCTGAGCCCA
AGCTGAGCCATCATATCCCCTGTGACCTGCACATATACATCCAGATGGCCTGAAGTAACTGA
AG-GATTACAAAAGAAGTGAAAATGGCCTGTTCCTGCCTTAAC--TGATGACATTACCTTATG-
--AAATTCCTTCTCCTGGCTCATCCTGGCTCAAAAGCTCCCCCACTGAGCACCTTATGACTCC
CACCCCTGCCAGCCAGAAAACAACCCCCCTTTGACTGTAATTTTCCACCACCCACCCAAATC
TTATAAAACAGCCCCA-CCCTATCTCCCTTCGCTGACTCTCTTTTAGGATCCAGCCAGCCTGC
ACCCAGGTGAAATAAACAGCCTTGTTGCTCACACAAAGCCTGTCTGGTGGTCTCTTCACAG
AC-----------------
>Macaca_mulatta-HERVHF_3LTR+flanking(-)

---AATA
TGTGGCTTGTCTTTTCATCCCTTA-----ACAGTACCTCTCACAACCAGATGTGATGAATTTTAA
TGAAGCCAAACTTACCAATTTTTTCT----------TTCATGGATCATGCTTTTGGTGTGGTATCTAA
AAAGTCATTGCCAAACCCTTAGGCTTTCTCTTATGTTATCCTTAAAAGTTTAATAGGTGTGTG
GTTTACACTTAGGTTTATGATCCCGTTTTGACTTA-TTTTTTGTGAAGCGTGTAAGTCTGTGTC
TATATGCATTTGTTGGCATGTGTATCTGGTCATCACAGCACTGTTTGTTGAAAAGACTATATT
TCTCCATTAGGCTGCCTTTGCTCTTTTGTCAAAGATCAGTCGATTATATTTGTAGAGTCTATTT
CTGGGCTCTCTGATCTGGAGGAAAAAGATCTGTGGTTGCCAGGGATTTGGAGGGACAAAA
GGCATGATCTTGGCTCACTGCAACCTCCACCT-CCCGGGTTCAAGGGATTCTCCTGCCTCAG
CCTCCTGAGTAGCTAGGATTTCAGGTGTGTACCACCATGCCCTGATAA-TTTTGTATTTTGT
CTATTATTTTTCTAATACCACACTGTCTGTTACTGCAGCTTTGTAGCAAGTCTTGAAGTCCTC
TAAGTTTTTCTCCTAGATGTTATATTAACTATTCTGAGCCTTGCCTTTCAATCTACGTTTGAGA
ATGTGATTGTCAATATTCACAAAATAACTTGCTGGGATTTTGATTGGGATTGTCCCTAATCTG
TGGATCAAATTGGGAAGAACTGATAGCAATATCGTGTCTTTCTATCCATAAACATGGAATATC
TTTCCATTTATTTAGATCTTTTGTTTATTTCATTAGAGTTTTGTAGTTCTCCTCACATAGATTTT
GAACATGTGTTGTTAGATTTGTACCTAAGTATTTCATTTTTCTTGGTGCTAATATAAATAGTAT
TATCTTTTTAATATCGAATTTCAACTAGTCATTATCGATATATAGAAAAGCAATGACTTGTGTA
TATTAGTCTTGTATCTTGCAACCTTGTTATAATCATTTATTAGTCTCAGGAGGG-TTTTTTGTTG
ATGTGGGGGGATTTTCTACGGATACAATCATGTCATCTT-AAAAAAAAGACG--TTCATTTCTT
CCTTTCTAATCTGTGTGCCTTTTATTTTCTTTCCTTGTCTTATTGCATTTGCTAGGACTTTCAG
TACAATGTTAAAAATCAGTGGTATAAATGTAAATAATTACTATTTATTGTGAAAGAAAAATAT
CTTGGGCCCCCAACATCACTAAGCTAAAGGGAAAAGTCAAGCTGGGAACTGCTTAGGGTA

AACCTGCCTCCCATTCTATTCAAAGTCATCCCTCTGCTCACTGAGATAAATGCATATCTGATT
GCCTCCTTTGGAGAGGCTCATCAGAAATGCAGAAGAATGCAACCATTTGTCTCTCACCTGC
TTGTGACCTGGAAGCTCCCT-CCCCCTTCAAGTTGTGACCTGGAAGCCTCCTCCCCCCTTCA
AGTTGTCCTGACTTTGCATAGATTGTGGACCAAACCAATGTTCATGTTACACATGTTGATTGA
TGTCTCCTCTCTCCCTAAAATATGTAAAACCAAGCTGTGTTCTGACTGTCAGGCCTCTGAGC
CCAAGCTAAGCCATCATATCCCCTGTGACCTGCACATATACATCCAGATGGCCTGAAGTAAC
TGAAG-GATTACAAAAGAAGTGAAAATGGCCTGTTCCTGCCTTAAC--TCATGACATTACCTT
ATG---AAATTCCTTCTCCTGGCAAACCTTGGCTCAAAAGCTCCCCCACTGAGCACCTTGTGA
CCCCCACCCCTGCCAGCCAGAAAACAACCCCCTTTGACTGTAATTTCCACCACCCACCCA
AATCTTATAAAACAGCCCCA-CCCAATCTCCCTTCACTGACTCTCTTTTCGGATCCAGCCCGC
CTGCACCCAGGTGAAATAAACAGCCTTGTTGCTCACACAAAGCCTGTTTGGTGGTCTCTTC
ACACAGAC---------------
>Macaca_silenus-HERVHF_5LTR+flanking(+)

--ATA
TGTGGCTTGTCTTTTCATCCCTTA-----ACAGTACCTCTCACAACCAGATGTGATGAATTTTAA
TGAAGCCAAACTTACCAATTTTTTCT----------TTCATGGATCATGCTTTTGGTGTGGTATCTAA
AAAGTCATTGCCAAACCCTTAGGCTTTCTCTTATGTTATCCTTAAAAGTTTAATAGGTGTGTG
GTTTACACTTAGGTTTATGATCCCGTTTTGACTTA-TTTTTTGTGAAGCGTGTAAGTCTGTGTC
TATATGCATTTGTTGGCATGTGTATCTGGTCATCACAGCACTGTTTGTTGAAAAGACTATATT
TCTCCATTAGGCTGCCTTTGCTCTTTTGTCAAAGATCAGTCGATTATATTTGTAGAGTCTATTT
CTGGGCTCTCTGATCTGGAGGAAAAAGATCTGTGGTTGCCAGGGATTTGGAGGGACAAAA
GGCATGATCTTGGCTCACTGCAACCTCCACCT-CCCGGGTTCAAGGGATTCTCCTGCCTCAG
CCTCCTGAGTAGCTAGGATTTCAGGTGTGTACCACCATGCCCTGATAA-TTTTTGTATTTTGT
CTATTATTTTTCTAATACCACACTGTCTGTTACTGCAGCTTTGTAGCAAGTCTTGAAGTCCTC
TAAGTTTTTCTCCTAGATGTTATATTAACTATTCTGAGCCTTGCCTTTCAATCTACGTTTGAGA
ATGTGATTGTCAATATTCACAAAATAACTTGCTGGGATTTTGATTGGGATTGTCCCTAATCTG
TGGATCAAATTGGGAAGAACTGATAGCAATATTGTGTCTTTCTATCCATAAACATGGACTATC
TTTCCATTTATTTAGATCTTTTGTTTATTTCATTAGAGTTTTGTAGTTCTCCTCACATAGATTTT
GAACATGTGTTGTTAGATTTGTACCTAAGTATTTCATTTTTCTTGGTGCTAATATAAATAGTAT
TATCTTTTCAATATCGAATTTCAACTAGTCATTATCGATATATAGAAAAGCAATGACTTGCGTA
TATTAATCTTGTATCTTGCAACCTTGTTATAATCATTTATTAGTCTCAGGAGGG-TTTTTTGTTG
ATGTGGGGGGACTTTCTACGGATACAATCATGTCATCTT-AAAAAAAAGACG--TTCATTTCTT
CCTTTCTAATCTGTGTGCCTTTTATTTTCTTTCCTTGTCTTATTGCATTTGCTAGGACTTTCAG
TACAATGTTAAAAATCAGTGGTATAAATGTAAATAATTACTATTTATTGTGAAAGAAAAATAT
CTTGGGCCCCCAACATCACTAAGCTAAAGGGAAAAGTCAAGCTGGGAACTGCTTAGGGTA
AACCTGCCTCCCATTCTATTCAAAGTCATCCCTCTGCTCACTGAGATAAATGCATATCTGATT
GCCTCCTTTGGAGAGGCTCATCAGAAATGCAGAAGAATGCAACCATTTGTCTCTCACCTGC
TTGTGACCTGGAAGCTCCCT-CCCCCTTCAAGTTGTGACCTGGAAGCCTCCTCCCCCCTTCA
AGTTGTCCTGACTTTGCATAGATTGTGGACCAAACCAATGTTCATGTTACACATGTTGATTGA
TGTCTCCTCTCTCCCTAAAATATGTAAAACCAAGCTGTGTTCTGACTGTCAGGCCTCTGAGC
CCAAGCTAAGCCATCATATCCCCTGTGACCTGCACATATACATCCAGATGGCCTGAAGTAAC
TGAAG-GATTACAAAAGAAGTGAAAATGGCCTGTTCCTGCCTTAAC--TGATGACATTACCTT
ATG---AAATTCCTTCTCCTGGCTCATCCTGGCTCAAAAGCTCCCCCACTGAGCACCTTGTGA

CCCCCACCCCTGCCAGCCAGAAAACAACCCCCTTTGACTGTAATTTTCCACCACCCACCCA
AATCTTATAAAACAGCCCCA-CCCTATCTCCCTTCACTGACTCTCTTTTCGGATCCAGCCCGC
CTGCACCCAGGTGAAATAAACAGTCTTGTTGCTCACACAAAGCCTGTTTGGTGGTCTCTTC
ACACAGACG--------------
>Mandrillus_leucophaeus-HERVHF_5LTR+flanking(+)

---GACACAAGTCCTTTATCACACGTGTGG
TTTGAAAATCTTTTTCTTCCAATATGTGGCTTGTCTTTTCATCCCTTA-----ACAGTACCTCTCA
CAACCAGATGTGATGAATTTTAATGAAGCCAAACTTACCAATTTTTTCT----------TTCATGGAT
CATGCTTTTGGTGTCGTATCTAAAAAGTCATTGCCAAACCCTTAGGCTTTCTCTTATGTTATC
CTTAAAAGTTTAATAGGTGTGTGGTTTACACTTAGGTTTATGATCCCGTTTTGACTTA-TTTTT
TGTGAAGCGTGTAAGTCTGTGTCTATATGCATTTGTTGGCATGTGTATCTGGTCATCACAGCA
CTGTTTGTTGAAAAGACTATATTTCTCCATTAGGCTGCCTTCGCTCTTTTGTCAAAGATCAGT
CGATTATATTTGTAGAGTCTATTTCTGGGCTCTCTGATCTGGAGGAAAAAGGTCTGTGATTGC
CAGGGATTTGGAGGGACAAAAGGCATGATCTTGGCTCACTGCAACCTCCACCT-CCCGGGT
TCAAGGGATTCTCCTGCCTCAGCCTCCTGAGTAGCTAGGATTTCAGGTGCGTACCACCATGC
CCTGATAA-TTTTTGTATTTTGTCTATTATTTTTCTAATACCACACTGTCTGTTACTGCAGCTTT
GTAGCAAGTCTTGAAGTCCTCTAAGTTTTTCTCCTAGATGTTATATTAACTATTCTGAGCCTT
GCCTTTCAATCTACATTTGAGAATGTGATTGTCAATATTCACAAAATAACTTGCTGGGATTTT
GATTGGGATTGTACCGAATCTGTGGATCAAATTGGGAAGAACTGATAGCAATATCGTGTTTT
TCTATCCATAAACATGGAATATCTTTCCATTTATTTAGATCTTTTGTTTATTTCATTAGAGTTTT
GTAGTTCTCCTCACATA-----------------GTTAGATTTGTACCTAAGTATTTCATTTTTCTTGGTGC
TAATATAAATAGTATTATCTTTTTAATATCGAATTTCAACTAGTCATTATCGATATATAGAAAAG
CAATGACTTGTGTATATTAATCTTGTATCTTGCAACCTTGTTATAATCATTTATTAGTCTCAGG
AGGG-TTTTTTGTTGATGTGGGGGGATTTCTACGGATACAATCATGTCATCTT-AAAAAAAA
GACG--TTCATTTCTTCCTTTCTAATCTGTGTGCCTTTTATTTTCTTTCCTTGTCTTATTGCATTC
GCTAGGACTTTCAGTACAATGTTAAAAATCAGTGGTATAAATGTAAACAATTACTATTTATTG
TGAAAGAAAAATATCTTGGGCCCCCAACATCACTAAGCTAAAGGGAAAAGTCAAGCTGGG
AACTGCTTAGGGCAAACCTGCCTCCCATTCTATTCAAAGTCATCCCTCTGCTCACTGAGATA
AATGCATATCTGATTGCCTCCTTTGGAGAGGCTCATCAGAAATGCAGAAGAATGCAACCATT
TGTCTCTCACCTGCTTGTGACCTGGAAG-------------------------------CCCCCTCCCCCCTTCAAG
TTTTCCTGACTTTGCATAGATTGTGGACCAAACCAGTGTTCGTATTACACATGTTGATTGATG
TCTCCTCTCTCCCTAAAATATGTAAAACCAAGCTGTGTTCTGACTGTCAGGCCTCTGAGCCC
AAGCTAAGCCATCATATCCCCTGTGACCTGCACATATACATCCAGATGGCCTGAAGTAACTG
AAG-GATCACAAAAGAAGTGAAAATGGCCTGTTCCTGCCTTAAT--TGATGACATTACCTTAC
G---AAATTCCTTCTCCTGGCTCATCCTGGCTCAAAAGTTCCCCCACTGAGCACCTTGTGACT
CCCACCCCTGCCTGCCAGAAAACAACCCCCTTTGACTGTAATTTCCCACTGCCCACCCAAAT
ATTATAAAACAGCCCCA-CCCCATCTCCCTTCGCTGACTCTCTTTTCGGACTCAGCCTGCCTG
CACCCAGGTGAAATAAACAGCCTTGTTGCTCACACAAAGCCTGTTTGGTGGTCTCTTCACA
CAGACG--------------
>Pongo_abelii-HERVHF_3LTR+flanking(-)
TTAATATATCTTTTCATGGCTTAATAGCTCAATTCTTTTCATTGCTAAATAATATTCCACTGTAT
GCATGTACCATAGTTTATTAATCCATCTCTGCTTTGTATATCTTCTTTGATGAGGTGTCTATCC
AGATCTCTTGACCATTTTTAATTGGGTTGTTTTCTTATCGTTGAGTTTTAGTTTTTTGTGTTTT

AGACACGAGTCCTTTATCAGACGTGTGGTTTGAACATCTTTTCCCCCAGTATGCGGCTTGT
CTTTTCATCCCTTA-----ACAGTAACTCTCACAACCAGATGTGATGAATTTAATGAAGCCAAA
CTTACCAATTTTTTCT----------TTCATGGATCATGCTTTTGGTGTCATATCTAAAAAGTCATCAC
CAAACACTTAGGCTTTCTCTTATGGTATCCTTACAAGTTTAATAGCTTTGTGGTTTACACTTA
GGTTTATGATCCCGTTTTGACTTA-TTTTTTGTGAAGGGTGTAAGTCTGTGTCTATATGCATTT
GTTGGCATGTATGTCTGGTCATCAGAGCACCACTTGTTGAAAAGACTATCTTTCTCCATTAG
ATTGCCTTTGCTCTTTTGTCAAAGATCAGTCGATTATATTTGTAGAGCCTATTCCTGGGCTCT
CTGATCTG--TTCCATTGC
TCTG---TTTGTCTGTTATTTTGCCAATACCACACT
GTCTGTTACTGCAGCTTTGTAGCAAGCCTTCAGGTCCTCTAAGTTTTTCTCCTGGATGTTATA
TTAACTATTCTGAGCCTTGCCTTTCAGTATAAATTTAAGAATGTGATTGTCAATATTCACAAA
GTAACTTGCTGGGATTTTTATTGGGATTGCACTGAATCTATAGATCAAATTGGGAAGAACTG
ATAACAATATCATGCCTTTCTATCCATAAACATGGAATATCTTTCCATTTATTTAGATCTTTTAT
TTATTTCATTAGAGTTTTGTAGTTCTTCTCACATAGATTTTGAACATGTGTTGTTAGATTTGTA
CCTAAGTATTTCATTTTTCTTAGTGCTAAT--------TATTACCTTTTTAATATTAAATTTCAACTAT
TCATTATTGATATATAGAAAAGCAATGACTTGTATATATTAATCTTGCATCTTGCAACCTTGTTA
TAATCATTTATTAGTCTCAGGAGGG-TTTTTTGTTGATGTGGGGGGATTGTCTACGGACATGA
TCACGTCATCTT-AAAAAAAAGACACTTTCATTTCTTCCTTTCTAATCTGTGTGCCTTTTATTT
TCTTTCCTTGTCTTATTGCATTAGCTAGGACTTTCAGTACAATGTTAAAAATCAGTCGTATAA
ATGTAAATAATTATTATTTATTGTGAAAGAAAAATATCTTGGGCCCCCAAAATCACTAAGCTA
AAGGGAAAAGTCAAACTGGGAACTGCTTAGGGCAAACCTGCCTCCCATTCTATTCAAAGTC
ATCCCTCTGCTCACTGAGATAAATGCATATCTGATCGCCTGCTTCAGAGAGGCTCATCAGAA
ACTCAAAAGAATGCAACTGTTTGTCTCTCACCTACTTGTGACCTGGAAG----------------------------
-------CACCCTCCCTACTTAGAGTTGTCCCGCTTTTGCTTAGATTCTGGACGAAACCAATGTTC
ATTTTACATACGTTGATTGATGTCTCATGTCTCCCTAAAATGTATAAAACCAAGCTGTGTTCT
GACTGTCAGGCCTCTGAGCACAAGCCAAGCCATCGTATCCCATGTAACCTGCACGTACACA
TCCAGATGTCCTAAAGTAACTGAAG-AATCACAAAAGAAGTGAAAACGGCCTGTTCCTGCC
TTAAC--TGATGATATTACCTTGTG---AAATTCCTTCTCCTGGCTCATCCTGGCTCAAAAGCTC
CCCAACTGAGCACCTTGTGACCCCCACCCCTGCCAGCCAGAAAACAACCCCCTTTGACTGT
AATTTTCCACTACCTACCCAAATCCTATAAAACGGCCCCACCCCTATCTCCCTTCCCTGACTC
TCTTTTCGGACTCAGCCCGCCTGCACCCAGGT---

>Pygathrix_nemaeus-HERVHF_5LTR+flanking(+)

---GAGCGCCGGCGTGACTTC----
---------------------CCTGGGCCCCCACACCTGAGGACCAGAGAACCTCACCTGAGAGTGTGTGT
ATATTTGTAATAA----AAGGCTGCCGCTTTCTTATGTATTTTGGCCTCATGTTTAATTACTTAGC
TCTCCTAAATTAAGTTACATTAAATTAAATTAAGA----CACTTATGTTATCCTTAAAAGTTTAAC
AGTTTTGTGGTTCACACTTAGGTTTATGATCCCGTTTTGACTTA-TTTTTTGTGAAGCGTGTA
AGTCTGTGTCTATATGCATTTGTTGGCATGTGTATCTGGTCATCACAGCACCGTTTGTTGAAA
AGACTATCTTTCTCCGTTAGGTTGCCTTTGCTCTTTTGTCAAAGATCAGTCGATTATATTTGTA
GAGTCTATTTCTGGGCTCTCTGATCTGGAGGAAAAAGGTTTGTGGTTGCCAGGGATTTGGA
GGGACGAAAGGCATGATCTTCGCTCACTGCAACCTCCACCT-CCCAGGTTCAACGGATTCTC
CTGCCTCAGCCTCATGAGTAGCTAGGATTTCAGGTGCGTACCACCATGCCCTGCTAA-TTTTT

GTATTTTGTCTATTATTTTTCTAATACCACACTGTCTGTTACTGCAGCTTTGTAGCAAGTCTTG
AAGTCCTCTAAGTTTTTCTCCTGGATGTTATATTAACTGTTCTGAGCCTTGCCTTTCAATCTAT
GTTTGAGAATATGATTGTCAATATTCACAAAATAACTTGCTGGGATTTTGATTGGGATTGCAC
CGAATCTGTGGATCAAATTGGGAAGAACTGATAACAATATCGTGTCTTTCTATCCATAAACAT
GGAATATCTTTCCATTTATTTAGATCTTTTTTTTATTTCATTAGAGTTTTGTAGTTCTCCTCACA
CAGATTTTGAACATATGTTGTTAGATTTGTACCTAAGTATTTCATTTTTCTTGGTGCTAATATA
AATAGTATTATCTTTTTAATATCAAATTTCAACTATTCATTATCGATATATAGAAAAGCAATGAC
TTGTATATATTAATCTTGCATCTTGCAACTTTGTTATAATCATTTATTAGTCTCAGGAGGG-TTT
TTTGTTGATGTGGAGGGATTTCTATGGACACAATCATGTCATCTTAAAAAAAAAGGCG--TT
CATTTCTTCCTTTCTAATCTGTGTGCCTTTTATTTTCTTTCCTTATCTTATTGCATTAGCTAGGA
CTTTCAGTACAATGTTAAACATCAGTGGTATAAATGTAAATAATCACTATTTATTGTGACAGA
AAAATATCTCGGGCCCCCAACATCACTAAGCTAAAGGGAAAAGTCAAGCTGGGAACTGCTT
AGGGTAAACCTGCCTCCCATTCTATTCAACGTCATCCCTCTGCTCACTGAGATAAATGCATAT
CTGATTGCCTCCCTTGGAGAGGCTCATCAGAAATGCAGAAGAATGCAACCATTTGTCTCTC
ACCTACTTGTGACCTGGAAG----------------------------------CCCCTCCCTGCTTCAAGTTGTCCTG
ACATTGCTTAGATTGTGGACCAAACCAATGTTCATGTTACACATGTTGATTGATGTCTCCTGT
CTCCCTAAAATATTTAAAACCAAGCTGTGTTCTGACTGTCAGGCCTCTGAGCCCAAGCTAAG
CCATCATATCCCCTGTGACCTGCACATATACATCCAGATGGCCTGAAGTAACTGAAG-GATCA
CAAAAGAAGTGAAAATGGCTTGTTCCTGCCTTAACAGCAGTGACATTGCACCTAGCGAGAA
TTCCTTCTCCTGGCTCATCCTGCCTCAAAAGCTCTCCCACTGAGCACCTTGTGACCCCCACC
CCTGCCAGCCAGAAAACAACCCCCTCTGACTGTAATTTTCCACTACCTACCCAAATATTATA
ATACAGCCCCACCCCTATCTCCCTT-GCTGACTCTCTTTTCGGATTCAGCCCACCTGCACCCA
GGTGAAATAAACAGCCTTGTTGCTCACACAAAGCCTGTTTGGTGGTCTCTTCACACAGACG
CTAGTGAAA-----
>Rhinopithecus_roxellana-HERVHF_3LTR+flanking(-)

---ACCAAGAGCACCGGCGTGACTT
C----------------------------CCTGGGCCCCCACACCTGAGGACCAGAGAACCTCGCCTGAGAGTGTG
TGCATATTTGTAATAA----AAGACTGCTGCTTTCTTATGTATTTTGGCCTCATGTTTAATTACTT
AGCTCTCCTAAATTAAGTTACATTAAATTAAATTAAGA----CACTTATGTTATCCTTAAAAGTTT
AATAGTTTTGTGGTTCACACTTAGGTTTATGATCCCGTTTTGACTTA-TTTTTTGTGAAGCGTG
TAAGTCTGTGTCTATATGCATTTGTTGGCATGTGTATCTGGTCATCACAGCACCGTTTGTTGG
AAAGACTATCTTTCTCCATTAGGTTGCCTTTGCTCTTTTGTCAAAGATCAGTCGATTATATTT
GTAGAGTCTAGTTCTGGGCTCTCTGATCTGGAGGAAAAAGGTTTGTGGTTGCCAGGGATTT
GGAGGGACGAAAGGCATGATCTTCGCTCACTGCAACCTCCACCT-CCCAGGTTCAAGGGAT
TCTCCTGCCTCAGCCTCATGAGTAGCTAGGATTTCAGGTGCGTACCACCATGCCCTGCTAA-T
TTTTGTATTTTGTCTTTTATTTTTCTAATACCACACTGTCTGTTACTGCAGCTTTGTAGCAAGT
CTTGAAGTCCTCTAAGTTTTTCTCCTGGATGTTATATTAACTATTCTGAGCCTTGCCTTTCAAT
CTATGTTTGAGAATGTGATTGTCAATATTCACAAAATAACTTGCTGGGATTTTGATTGGGATT
GCACCGAATCTGTGGATCAAATTGGGAAGAACTGATAACAATATCGTGTCTTTCTATCCATA
AACATGGAATATCTTTCCATTTATTTAGATCTTTTATTTATTTCATTAGAGTTTTGTAGTTCTCC
TCACATAGATTTTGAACATAGGTTGTTAGATTTGTACCTAAGTATTTCATTTTTCTTGGTGCTA
ATATAAATAGTATTATCTTTTTAATATCAAATTTCAACTATTCATTATCGATATATAGAAAAGCA
ATGACTTGTATATATTAATCTTGCATCTTGCAACTTTGTTATAATCATTTATTAGTCTCAGGAG

GGTTTTTTTGTTGATGTGGAGGGATTTCTACGGACACAATCATGTCATCTTAAAAAAAAAG
GCG--TTCATTTCTCCCTTTCTAATCTGTGTGCCTTTTATTTTCGTTCCTTGTCTTATTGCATTAG
CTAGGACTTTCAGTACAATGTTAAACATCAGTGGTATAAATGTAAATAATCACTATTTATTGT
GACAGAAAAATATCTCGGGCCCCCAACATCACTAAGCTAAAGGGAAAAGTCAAGCTGGGA
ACTGCTTAGGGTAAACCTGCCTCCCATTCTATTCAAAGTCATCTCTCTGCTCACTGAGATAAA
TGCATATCTGATTGCCTCCTTTGGAGAGGCTCATCAGAAATGCAGAAGAATGCAACCATTTG
TCTCTCACCTACTTGTGACCTGGAAG--------------------------------CCCCTCCCTGCTTCAAGTT
GTCCTGACATTGCTTAGACTGTGGACCAAACCAATGTTCATGTTACACATGTTGATTGATGT
CTCCTGTCTCCCTAAAATATTTAAAACCAAGCTGTGTTCTGACTGTCAGGCCTCTGAGCCCA
AGCTAAGCCATCATATCCCCTGTGACCTGCACATATACATCCAGATGGCCTGAAGTAACTGA
AG-AATCACAAAAGAAGTAAAAATGGCCTGTTCCTGCCTTAAC--TGATGACATTACCTTGTG
---AAATTCCTTCTCCTGGCTCATCCTGCCTCAAAAGCTCTCCCACTGAGCACCTTGTGACCC
CCACCCCTGCCAGCCAGAAAACAACCCCCTCTGACTGTAATTTTCCACTACCTACCCAAATA
TTATAATACAGCCCCACCCCTATCTCCCTT-GCTGACTCTCTTTTCGGATTCAGCCCACCTGCA
CCCAGGTGAAATAAACAACCTTGTTGCTCACACAAAGCCTGTTTGGTGGTCTCTTCACACA
GACGCTAGTGAA------
>Rhinopithecus_strykeri-HERVHF_5LTR+flanking(+)

--ACCGAGAGCACCGGCGTGACTT
C-------------------------CCTGGGCCCCCACACCTGAGGACCAGAGAACCTCGCCTGAGAGTGTG
TGCATATTTGTAATAA----AAGGCTGCTGCTTTCTTATGTATTTTGGCCTCATGTTTAATTACTT
AGCTCTCCTAAATTAAGTTACATTAAATTAAATTAAGA----CACTTATGTTATCCTTAAAAGTTT
AATAGTTTTGTGGTTCACACTTAGGTTTATGATCCCGTTTTGACTTA-TTTTTTGTGAAGCGTG
TAAGTCTGTGTCTATATGCATTTGTTGGCATGTGTATCTGGTCATCACAGCACCGTTTGTTGG
AAAGACTATCTTTCTCCGTTAGGTTGCCTTTGCTCTTTTGTCAAAGATCAGTCGATTATATTT
GTAGAGTCTAGTTCTGGGCTCTCTGATCTGGAGGAAAAAGGTTTGTGGTTGCCAGGGATTT
GGAGGGACGAAAGGCATGATCTTCGCTCACTGCAACCTCCACCT-CCCAGGTTCAAGGGAT
TCTCCTGCCTCAGCCTCATGAGTAGCTAGGATTTCAGGTGCGTACCACCATGCCCTGCTAA-T
TTTTGTATTTTGTCTTTTATTTTTCTAATACCACACTGTCTGTTACTGCAGCTTTGTAGCAAGT
CTTGAAGTCCTCTAAGTTTTTCTCCTGGATGTTATATTAACTATTCTGAGCCTTGCCTTTCAAT
CTATGTTTGAGAATGTGATTGTCAATATTCACAAAATAACTTGCTGGGATTTTGATTGGGATT
GCACCGAATCTGTGGATCAAATTGGGAAGAACTGATAACAATATCGTGTCTTTCTATCCATA
AACATGGAATATCTTTCCATTTATTTAGATCTTTTATTTATTTCATTAGAGTTTTGTAGTTCTCC
TCACATAGATTTTGAACATAGGTTGTTAGATTTGTACCTAAGTATTTCATTTTTCTTGGTGCTA
ATATAAATAGTATTATCTTTTTAATATCAAATTTCAACTATTCATTATCGATATATAGAAAAGCA
ATGACTTGTATATATTAATCTTGCATCTTGCAACTTTGTTATAATCATTTATTAGTCTCAGGAG
GG-TTTTTTGTTGATGTGGAGGGATTTCTACGGACACAATCATGTCATCTTAAAAAAAAAG
GCG--TTCATTTCTCCCTTTCTAATCTGTGTGCCTTTTATTTTCATTCCTTGTCTTATTGCATTAG
CTAGGACTTTCAGTACAATGTTAAACATCAGTGGTATAAATGTAAATAATCACTATTTATTGT
GACAGAAAAATATCTCGGGCCCCCAACATCACTAAGCTAAAGGAAAAAGTCAAGCTGGGA
ACTGCTTAGGGTAAACCTACCTCCCATTCTATTCAAAGTCATCCCTCTGCTCACTGAGATAAA
TGCATATCTGATTGCCTCCTTTGGAGAGGCTCATCAGAAATGCAGAAGAATGCAACCATTTG
TCTCTCACCTACTTGTGACCTGGAAG--------------------------------CCCCTCCCTGCTTCAAGTT
GTCCTGACATTGCTTAGACTGTGGACCAAACCAATGTTCATGTTACACATGTTGATTGATGT

CTCCTGTCTCCCTAAAATATTTAAAACCAAGCTGTGTTCTGACTGTCAGGCCTCTGAGCCCA
AGCTAAGCCATCATATCCCCTGTGACCTGCACATATACATCCAGATGGCCTGAAGTAACTGA
AG-AATCACAAAAGAAGTGAAAATGGCTTGTTCCTGCCTTAAC--TGATGACATTACCTTGTG
---AAATTCCTTCTCCTGGCTCATCCTGCCTCAAAAGCTCTCCCACTGAGCACCTTGTGACCC
CCACCCCTGCCAGCCAGAAAACAACCCCCTCTGACTGTAATTTCCACTACCTACCCAAATA
TTATAATACAGCCCCACCCCTATCTCCCTT-GCTGACTCTCTTTTCGGATTCAGCCCACCTGCA
CCCAGGTGAAATAAACAACCTTGTTGCTCACACAAAGCCTGTTTGGTGGTCTCTTCACACA
GACGCTAGTGAAA-----
>Theropithecus_gelada-HERVHF_3LTR+flanking(-)

---ATA
TGTGGCTTGTCTTTTCATCCCTTA-----ACAGTACCTCTCACAACCAGATGTGATGAATTTTAA
TGAAGCCAAACTTACCAATTTTTTCT----------TTCATGGATCATGCTTTTGGTGTCGTATCTAA
AAAGTCATTGCCAAACCCTTAGGCTTTCTCTTATGTTATCCTTAAAAGTTTAATAGGTGTGTG
GTTTACACTTAGGTTTATGATCCCGTTTTGACTTA-TTTTTTGTGAAGCGTGTAAGTCTGTGTC
TATATGCATTTGTTGGCATGTGTATCTGGTCATCACAGCACTGTTTGTTGAAAAGACTATATT
TCTCCATTAGGCTGCCTTTGCTCTTTTGTCAAAGATCAGTCGATTATATTTGTAGAGTCTATTT
CTGGGCTCTCTGATCTGGAGGAAAAAGGTCTGTGATTGCCAGGGATTTGGAGGGACAAAA
GGCATGATCTTGGCTCACTGCAACCTCCACCT-CCCGGGTTCAAGGGATTCTCCTGCCTCAG
CCTCCTGAGTAGCTAGGATTTCAAGTGCGTACCACCATGCCCTGATAA-TTTTTGTATTTTGT
CTATTATTTTTCTAATACCACACTGTCTGTTACTGCAGCTTTGTAGCAAGTCTTGAAGTCCTC
TAAGTTTTTCTCCTAGATGTTATATTAACTATTCTGAGCCTTGCCTTTCAATCTACATTTGAGA
ATGTGATTGTCACTATTCACAAAATAACTTGCTGGGATTTTGATTGGGATTGTACCGAATCTG
TGGATCAAATTGGGAAGAACTGATAGCAATATCGTGTCTTTCTATCCATAAACATGGAATATC
TTTCCATTTATTTAGATCTTTTGTTTATTTCATTAGAGTTTTGTAGTTCTCCTCACATAGATTTT
GAACATGTGTTGTTAGATTTGTACCTAAGTATTTCATTTTTCTTGGTGCTAATATAAATAGTAT
TATCTTTTTAATATCGAATTTCAACTAGTCATTATCGATATATAGAAAAGCAATGACTTGTGTA
TATTAATCTTGTATCTTGCAACCTTGTTATAATCATTTATTAGTCTCAGGAGGG-TTTTTTGTTG
ATGTGGGGGGATTTTCTACGGATGCAATCATGTCATCTT-AAAAAAAAGACG--TTCATTTCTT
CCTTTCTAATCTGTGTGCCTTTTATTTTCTTTCCTTGTCTTATTGCATTCGCTAGGACTTTCAG
TACAATGTTAAAAATCAGTGGTATAAATGTAAATAATTACTATTTATTGTGAAAGAAAAATAT
CTTGGGCCCCCAACATCACTAAGCTAAAGGGAAAAGTCAAGCTGGGAACTGCTTAGGGCA
AACCTGCCTCCCATTCTATTCAAAGTCATCCCTCTGCTCACTGAGATAAATGCATATCTGATT
GCCTCCTTTGGAGAGGCTCATCAGAAATGCAGAAGAATGCAACCATTTGTCTCTCACCTGC
TTGTGACCTGGAAGCTCCCTCCCCCCTTCAAGTTGTGACCTGGAAGCCTCCTCCCCCCTTC
AAGTTGTCCTGACTTTGCATAGATTGTGGACCAAACCAATGTTCATGTTACACATGTTGATT
GATGTCTCCTCTCTCCCTAAAATACATAAAACCAAGCTGTGTTCTGACTGTCAGGCCTCTGA
GCCCAAGCTAAGCCATCATATCCCCTGTGACCTGCACATATACATCCAGATGGCCTGAAGTA
ACTGAAG-GATTACAAAAGAAGTGAAAATGGCCTGTTCCTGCCTTAAC--TGATGACATTACC
TTATG---AAATTCCTTCTCCTGGCTCATCCTGGCTCAAAAGCTCCCCCACTGAGCACCTTGTG
ACTCCCACCCCTGCCAGCCAGAAAACAACCCCCTTTGACTGTAATTTCCACCACCCACCC
AAATCTTATAAAACAGCCCCA-CCCTATCTCCCTTCGCTGACTCTCTTTTCGGATCCAGCCCG
CCTGCACCCAGGTGAAATAAACAGCCTTGTTGCTCACACAAAGCCTGTCTGGTGGTCTCTT
CACACAGAC---------------

Supplementary data S5 The alignments of the 3LTR and flanking sequences of a widely vertical transmitted HERV-H in 17 species

>Cercocebus_atys-HERVHF_5LTR+flanking(-)
-------------------------CTAAGCCATCATATCCCCTGTGACCTGCAC-TATACATCCAGATGGCCTG
AAGTAACTGAAGAATCACAAAAGAAGTGAAAGTGGCCTGTTCCTGCCTTAACTGATGACAT
TAATTTGTGAAATTCCTTCTCCTGGCTCATCCTGGCTCAAAAGTTCCCCCACTGAGCACCTT
GTGA-CCCCCACCCCTGCCAGCCAAAA-ATAACCCCCTTTGACTGT-AATTTTCCACTACCTA
CCCAAATCCTATAAAACGACCCCACCCCTATCTCCCTTCACTGACTCTCTTTTCGGACTCAG
CCCACCTGCACCCAGGTGAAATAAACAGTCTTGTTGCTCACACAAAGCCTGTTTGGTGGTC
TCTTCACATGGACGTGCGTGAAACTGACCACTGTGGGCACATGTCATCAGGACCTTCTGAG
GCTGGGTCACAGCCGTGTGTCCTCAAACTTGGCAAAATAAACTTTCTAAATTAACTAAGAC
TTGTCTCAGATTTTCGGGGTTCATATTATCAATTAAAAAACAAAATTTAAATT-TAAAAAAAG
AGGAAAGCAGTGTTGAGGGGGGCATCCTGACCTTGCTCCTGATCTTAGCAGGGAAGCTTCA
AGTTTCTCACCATTAAGTGTGACCACTGTTGGCTTTTTGTC-GAGTTTTGACAGCTGCAGGC
TCCTTATCAAGTTCCCCTCTAGTCCTCATTTGCTGAGAGCGTTTATCATGAATGGGTGTTGGA
TTTTGTTAGATGATTTTTCTGAATCTGC---TATGATCATGTGACTTTTCTTCTTTAGCCTGTTG
AGGTGATGGATTACATGACTAGATTTTCAAATGCTGAACCAGTTTTGTGTACCTGAGATAAA
TCCCACTCTGTT---

--GTGGTATATAATTC
TTTTTATGCATTGTTTGGATTTGACTTGCTAATTTTTCGTTGAAGATTTTTGCATCTATGTTTAT
GAAAGCTATTGGCTTATGGCTTTCCCTTCTTGTGTCTTTGTCTGGTTTTGTTTTTAGGGTAAT
GCTGGCTTTATAGAATGCGTTAGAAAACATTCCTTTTGCTTCTATTATCTGGAAGACATCATA
GAAAATTACTATCATTTCTTCCTTAAATGTTTGGCAGAATTCATCAGCAAACCCGTTCAGAC
CTGGTGCTTCCTATTTGGAAGGTAATTATTTATTGATTCAATTCCTTTAATGCATACATACCTAT
TCATATTATCTATTCTTTCTTGTGTAAGTTTTGGTAGATTGCATCTTTAAGAAATTGATCCATTT
CATCTAGGTTATCATATTTCTGGGCATAGAGTTGTTATGGTAATAATATTCTATTATTATCCTTT
ACAATATTCCTCTAGGATCAGTAGCAGTGG-CACCTATTTTATTTCTAATGCTAATAAATTGTG
TCTCATTTCTTTCTTTGTTAACTTGGCCAGAGGTTTATCAATTTCATTCC------TTTATTTTTT
CAAAGAACCAGCTTTTGGTT---TTGATTTTTCTCTAGTAGGTTCCTGATTTCAGTTTCACTGA
TGTCTGCTTTAGTTAATTTTTTCTGTCTACTATGTTTTTAATTAGTTCTTCTTTTCTAGTTTCTT
GAGGTGGAAACTTAGATGATTTGTTTCTGATCTTTCTTCTTTTCTAAAATGCCTTCTTTCAAT
GCCATAAACTTTCCTCTAAGCACTGCTTTTGTTGCATCTCACAAATTTTGATAAGTTGTATTT
TCATTTTCATTTATTTCAAAATATTGCTTAATTTCTCTGGAGACCTTTTCCTTTGACTGATGTG
TTATTTAGAAGTGTTTTGTTTAATCTCCAGGTATTTTTTAATTTTCCAATTGTCTTTCTGTTATT
GATTTCTAGTTTTATTGTGTTCAGAAAGCATATTTTATTTTTTAGTTCTTTTAAACTTGTTGAG
GTGTGTCAGGGCTTAGAATCTGGTCTGTCTTGGTGAATGTTCCACATAAGCATGAGAAGAAT
GTATTTTGCTGTTGCTTCACGAAATATTCCATAGCTATCCAGTAAAT---------------------------------
-----
>Cercopithecus_mitis-HERVHF_5LTR+flanking(-)
--GAATGTCAGGCCTCTGAGCCCAAGCTAAGCCATCATATCCCCTGTGACCTGCGC-TATACAT

CCAGATGGCCTGAAGTAACTGAAGAATCACAAAAGAAGTGAAAGTGGCCTGTTCCTGCCTT
AACTGATGACATTACCTTGTGAAATTCCTTCTCCTGGCTCATCCTGGCTCAAAAGTTCCCCC
ACTGAGCACCTTGTGA-CCCCCACCCCTGCCAGCCAAAA-ATAACCCCCTTTGACTGTGAAT
TTTCCACTACCTACCCAAATCCTATAAAACGACCCCACCCCTATCTCCCTTCACTGACTCTCT
TTTCGGACTCAGCCCACCTGCACCCAGGTGAAATAAACAGTCTTGTTGCTCACACAAAGCC
TGTTTGGTGGTCTCTTCACATGGACGTGCGTGAAACTGACCACTGTGGGCACATGTCATCA
GGACCTTCTGAGGCTGGGTCACAGCCGTGTGTCCTCAAACTTGGCAAAATAAACTTTCTAA
ATTAACTAAGACTTGTCTCAGATTTTCGGGGTTCATGTTATCAATTAAAAACCAAAATTTAAA
TT-AAAAAAAAGAGGAAAGCAGTGTTGAGGGGGGCATCCTTACCTTGCTCCTGATCTTAGC
AGGGAAGCTTCAAGTTTCTCACCATTAAGTGTGACCACTGTTGGCTTTTTGTCAGAGTTTTG
ACAGCTGCAGGCTCCTTATCAAGTTCCCCTCTAGTCCTCATTTGCTGAGAGCGTTTATCATG
AATGGGTGTTGGATTTTGTTAGATGATTTTTCTGAATCTGC---TATGATCATGTGACTTTTCTT
CTTTAGCCTGTTGAGGTGATGGATTACATGACTAGATTTTCAAATGCTGAACCAGTTTTGTGT
ACCTGAGATAAATCCCACTCTGTT------------------------------------

---G
TGGTATATAATTCTTTTTATGCATTGTTTGGATTTGACTTGCTAATTTTTCGTTGAAGATTTTTG
CATCTATGTTTATGAAAGCTATTGGCTTATGGCTTTCCCTTCTTGTGTCTTTGTCTGGTTTTCT
TTTTAGGGTAATGCTGGCTTTATAGAATGCATTAGAAAACATTCTTTTTGCTTCTATTATCTGG
AAGACATCATAGAAAATTACTATCGTTTCTTCCTTAAATGTTTGGCAGAATTCATCAGCAAAC
CTGTTCAGACCTGGTGCTTCCTATTTGGAAGGTAATTATTTATTGATTCAATTCCTTTAATGCA
TACATACCTATTCATATTATCTATTCTTTTTTGTGTAAGTTTTGGTAGATTGCATCTTTAAGAAA
TTGATCCATTTCATCTAGGTTATCATATTTCTGGGCATAGAATTGTTATGGTAATAATATTCTAT
TATTATCCTTTACAATATTCCTCTAGGATCAGTAGCAGTAG-CCCCTATTTTGTTTCTAATGCTA
ACAATTTGTGTCTCATTTCTTTCTTTGTTAACTTGCCTAGAGGTTTATCAATTTCATTCC-TTTT
TTTTTTTTTTTTAAAAGAACCAGCTTTTGGTT---TTGATTTTTCTCTAGTAGGTTCCTGATTTC
AGTTTCACTGATGTCTGCTTTAGTTAATTTTTTCTGTCTACTATGTTTTTAATTAGTTCTTCTTT
TCTAGTTTCTTGAGGTGGAAACTTAGATGATTTGTTTCTGATCTTTCTTCTTTTCTAAAATGC
CTTCTTTCAATGCCATAAACTTTCCTCTAAGCACTGCTTTTGTTGCATCTCACAAATTTTGAT
AAGTTGTATTTTCATTTTCATTTATTTCAAAATATTGCTTAATTTCTCTGGAGACCTTTTCCTTT
GACTGATGTGTTATTTAGAAGTGTTTTGTTTAATCTCCAGGTATTTTTTAATTTTCCAATTGTC
TTTCTGTTATTGATTTCTAGTTTTGTTGTGTTCAGAGAGCATATTTTATTTTTTAGTTCTTTTAA
ACTTGTTGAGGTGTGCCAGGACTTAGAATTTGGTCTGTCTTGGTGAATGTTCCACATAAGCA
TGAGAAGAATGTATTTTGCTGTTGCT----------------------------------
>Cercopithecus_mona-HERVHF_5LTR+flanking(-)
--GAATGTCAGGCCTCTGAGCCCAAGCTAAGCCATCATATCCCCTGTGACCTGCGC-TATACAT
CCAGATGGCCTGAAGTAACTGAAGAATCACAAAAGAAGTGAAAGTGGCCTGTTCCTGCCTT
AACTGATGACATTACCTTGTGAAATTCCTTCTCCTGGCTCATCCTGGCTCAAAAGTTCCCCC
ACTGAGCACCTTGTGA-CCCCCACCCCTGCCAGCCAAAA-ATAACCCCCTTTGACTGT-AATT
TTCCACTACCTACCCAAATCCTATAAAACGACCCCACCCC-ATCTCCCTTCACTGACTCTCTT
TTCCGACTCAGCCCACCTGCACCCAGGTGAAATAAACAGTCTTGTTGCTCACACAAAGCCT
GTTTGGCGGTCTCTTCACATGGACGTGCGTGAAACTGACCACTGTGGGCACATGTCATCAG
GACCTTCTGAGACTGGGTCACAGCCGTGTGTCCTCAAACTTGGCAAAATAAACTTTCTAAA

TTAACTAAGACTTGTCTCAGATTTTCGGGGTTCATGTTATCAATTAAAAACCAAAATTTAAAT
T-AAAAAAAAGAGGAAAGCAGTGTTGAGGGGGGCATCCTTACCTTGCTCCTGATCTTAGCA
GGGAAGCTTCAAGTTTCTCACCATTAAGTGTGACCACTGTTGGCTTTTTGTCAGAGTTTTGA
CAGCTGCAGGCTCCTTATCAAGTTCCCCTCTAGTCCTCATTTGCTGAGAGCGTTTATCATGAA
TGGGTGTTGGATTTTGTTAGATGATTTTTCTGAATCTGC---TATGATCATGTGACTTTTCTTCT
TTAGCCTGTTGAGGTGATGGATTACATGACTAGATTTCAAATGCTGAACCAGTTTTGTGTA
CCTGAGATAAATCCCACTCTGTT--

--GT
GGTATATAATTCTTTTTATGCATTGTTTGGATTTGACTTGCTAATTTTTCGTTGAAGATTTTTG
CATCTATGTTTATGAAAGCTATTGGCTTATGGCTTTCCCTTCTTGTGTCTTTGTCTGGTTTTCT
TTTTAGGGTAATGCTGGCTTTATAGAATGCATTAGAAAACATTCCTTTTGCTTCTATTATCTGG
AAGACATCATAGAAAATTACTATCGTTTCTTCCTTAAATGTTTGGCAGAATTCATCAGCAAAC
CTGTTCAGACCTGGTGCTTCCTATTTGGAAGGTAATTATTTATTGATTCAATTCCTTTAATGCA
TACATACCTATTCATATTATCTATTCTTTCTTGTGTAAGTTTTGGTAGATTGCATCTTTAAGAAA
TTGATCCATTTCATCTAGGTTATCATATTTCTGGGCATAGAGTTGTTATGGTAATAATATTCTAT
TATTATCCTTTACAATATTCCTCTAGGATCAGTAGCAGTAG-CCCCTATTTTATTTCTAATGCTA
ACAATTTGTGTCTCATTTCTTTCTTTGTTAACTTGCCTAGAGGTTTATCAATTTCATTCC------T
TTTTTTTTTAAAAAGAACCAGCTTTTGGTT---TTGATTTTTCTCTAGTAGGTTCCTGATTTCAG
TTTCACTGATGTCTGCTTTAGTTAATTTTTTCTGTCTACTATGTTTTTAATTAGTTCTTCTTTTC
TAGTTTCTTGAGGCGGAAACTTAGATGATTTGTTTCTGATCTTTCTTCTTTTCTAAAATGCCT
TCTTTCAATGCCATAAACTTTCCTCTAAGCACTGCTTTTGTTGCATCTCACAAATTTTGATAA
GTTGTATTTTCATTTTCATTTATTTCAAAATATTGCTTAATTTCTCTGGAGACCTTTTCCTTTGA
CTGATGTGTTATTTAGAAGTGTTTTGTTTAATCTCCAGGTATTTTTTAATTTTCCAATTGTCTT
TCTGTTATTGATTTCTAGTTTTGTTGTGTTCAGAGAGCATATTTTATTTTTTAGTTCTTTTAAA
CTTGTTGAGGTGTGCCAGGGCTTAGAATTTGGTCTGTCTTGGTGAATGTTCCACATAAGCAT
GAGAAGAATGTATTTTGCTGTTGCTTCACAAA---
--
>Chlorocebus_aethiops-HERVHF_3LTR+flanking(+)
---AATGTCAGGCCTCTGAGCCCAAGCTAAGCCATCATATCCCCTGTGACTTGCAC-TATACAT
CCAGATGGCCTGAAGTAACTGAAGAATCACAAAAGAAGTGAAAGTGGCCTATTCCTGCCTT
AACTGATGACATTACCTTGTGAAATTCCTTCTCCTGGCTCATCCTAGCTCAAAAGTTCCCCC
ACTGAGCACCTCGTGA-CCCCCACCCCTGCCAGCCAAAA-ATAACCCCCTTTGACTGT-AATT
TTCCACTACCTACCCAAATCCTATAAAACGACCCCACCCCTATCTCCCTTCACTGACTCTCTT
TTCGGACTCAGCCCACCTGCACCCAGGTGAAATAAACAGTCTTGTTGCTCACACAAAGCCT
GTTTGGTGGTCTCTTCACATGGACGTGCATGAAACTGGCCACTGTGGGCACATGTCATCAG
GACCTTCTGAGGCTGGGTCACAGCCGTGTGTCCTCAAACTTGGCAAAATAAACTTTCTAAA
TTAACTAAGACTTGTCTCAGATTTTCGGAGTTCATATTATCAATTAAAAACCAAAATTTAAAT
T--AAAAAAAGAGGAAAGCAGTGTTGAGGGGGGCATCCTTACCTTGCTCCTGATCTTAGCAG
GGAAGCTTCAAGTTTCTCACCATTAAGTGTGACCACTGTTGGCTTTTTGTC-GAGTTTTGAC
AGCTGCAGGCTCCTTATCAAGTTCCCCTCTAGTCCTCATTTGCTGAGAGCGTTTATCATGAAT
GGGTGTTGGATTTTGTTAGATGATTTTTCTGAATCTGC---TATGATCATGTGACTTTTCTTCTT
TAGCCTGTTGAGGTGATGGATTACATGACTAGATTTCAAATGCTGAACCAGTTTTGTGTAC

CTGAGGTAGTATATAATTCTTTTTATGCATTGTTTGGATTTGACTTGCTAATTTTTCGTTGAAG
ATTTTTGCATCTATGTTTATGAAAGCTATTGGCTTATGACTTTCCCTTCTTGTGTCTTTGTCTG
GTTTTCTTTTTAGGGTAATGCTGGCTTTATAGAATGCATTAGAAAACATTCCTTTTGCTTCTAT
TATCTGGAAGACATCATAGAAAATTACTATCGTTTCTTCCTTAAATGTTTGGCAGAATTCATC
AGCAAACCTGTTCAGACCTGGTGCTTCCTATTTGGAAGGTAATTATTTATTGATTCAATTCCT
TTAATGCATACATACCTATTCATATTATCTATTCTTTCTTGTGTAAGTTTTGGTAGATTGCATCT
TTAAGAAATTTTCTTCTTTAGCCTGTTGAGGTGATGTATTACATGACTAGATTTTCAAATGCT
GAACCAGTTTTGTGTACCTGAGGTAGTATATAATTCTTTTTATGCATTGTTTGGATTTGACTTG
CTAATTTTTCGTTGAAGATTTTTGCATCTATGTTTATGAAAGCTATTGGCTTATGACTTTCCCT
TCTTGTGTCTTTGTCTGGTTTTCTTTTTAGGGTAATGCTGGCTTTATAGAATGCATTAGAAAA
CATTCCTTTTGCTTCTATTATCTGGAAGACATCATAGAAAATTACTATCGTTTCTTCCTTAAAT
GTTTGGCAGAATTCATCAGCAAACCTGTTCAGACCTGGTGCTTCCTATTTGGAAGGTAATTA
TTTATTGATTCAATTCCTTTAATGCATACATACCTATTCATATTATCTATTCTTTCTTGTGTAAGT
TTTGGTAGATTGCATCTTTAAGAAATTGATCCATTTCATCTAGGTTATCATATTTCTGGGCATA
GAGTTGTTATGGTAATAATATTCTATTATTATCCTTTACAATATTCCTCTAGGATCAGTAGCAGT
GG-CCCCTATTTTATTTCTAATGCTAACAATTTGTGTCTCATTTCTTTCTTTGTTAACTTGGCTA
GAGGTTTATCAATTTCATTCC--TTT-TTTTTTTTTTTTAAAGAACCAGCTTTTGGTT---TTGATT
TTTCTCTAGTAGGTTCCTGATTTCAGTTTCACTGATGTCTGCTTTAGTTAATTTTTT-------------
----

>Chlorocebus_sabaeus-HERVHF_5LTR+flanking(-)

--GAATGTCAGGCCTCTGAGCCCAAGCTAAGCCATCATATCCCCTGTGACTTGCAC-TATACAT
CCAGATGGCCTGAAGTAACTGAAGAATCACAAAAGAAGTGAAAGTGGCCTATTCCTGCCTT
AACTGATGACATTACCTTGTGAAATTCCTTCTCCTGGCTCATCCTAGCTCAAAAGTTCCCCC
ACTGAGCACCTCGTGA-CCCCCACCCCTGCCAGCCAAAA-ATAACCCCCTTTGACTGT-AATT
TTCCACTACCTACCCAAATCCTATAAAACGACCCCACCCCTATCTCCCTTCACTGACTCTCTT
TTCGGACTCAGCCCACCTGCACCCAGGTGAAATAAATAGTCTTGTTGCTCACACAAAGCCT
GTTTGGTGGTCTCTTCACATGGACGTGCATGAAACTGGCCACTGTGGGCACATGTCATCAG
GACCTTCTGAGGCTGGGTCACAGCCGTGTGTCCTCAAACTTGGCAAAATAAACTTTCTAAA
TTAACTAAGACTTGTCTCAGATTTTCGGAGTTCATATTATCAATTAAAAACCAAAATTTAAAT
T--AAAAAAAGAGGAAAGCAGTGTTGAGGGGGGCATCCTTACCTTGCTCCTGATCTTAGCAG
GGAAGCTTCAAGTTTCTCACCATTAAGTGTGACCACTGTTGGCTTTTTGTC-GAGTTTTGAC
AGCTGCAGGCTCCTTATCAAGTTCCCCTCTAGTCCTCATTTGCTGAGAGCGTTTATCATGAAT
GGGTGTTGGATTTTGTTAGATGATTTTTCTGAATCTGC---TATGATCATGTGACTTTTCTTCTT
TAGCCTGTTGAGGTGATGGATTACATGACTAGATTTTCAAATGCTGAACCAGTTTTGTGTAC
CTGAG--

---GTAGTATATAAT
TCTTTTTATGCATTGTTTGGATTTGACTTGCTAATTTTTCGTTGAAGATTTTTGCATCTATGTTT
ATGAAAGCTATTGGCTTATGACTTTCCCTTCTTGTGTCTTTGTCTGGTTTTCTTTTTAGGGTAA
TGCTGGCTTTATAGAATGCATTAGAAAACATTCCTTTTGCTTCTATTATCTGGAAGACATCAT

AGAAAATTACTATCGTTTCTTCCTTAAATGTTTGGCAGAATTCATCAGCAAACCTGTTCAGA
CCTGGTGCTTCCTATTTGGAAGGTAATTATTTATTGATTCAATTCCTTTAATGCATACATACCTA
TTCATATTATCTATTCTTTCTTGTGTAAGTTTTGGTAGATTGCATCTTTAAGAAATTGATCCATT
TCATCTAGGTTATCATATTTCTGGGCATAGAGTTGTTATGGTAATAATATTCTATTATTATCCTTT
ACAATATTCCTCTAGGATCAGTAGCAGTGG-CCCCTATTTTATTTCTAATGCTAACAATTTGTG
TCTCATTTCTTTCTTTGTTAACTTGGCTAGAGGTTTATCAATTTCATTCC-TTTT-TTTTTTTTT
TTAAAGAACCAGCTTTTGGTT---TTGATTTTTCTCTAGTAGGTTCCTGATTTCAGTTTCACTGA
TGTCTGCTTTAGTTAATTTTTTCTGTCTACTATGTTTTTAATTAGTTCTTCTTTTCTAGTTTCTT
GAGGTGGAAACTTAGATGATTTGTTTCTGATCTTTCTTCTTTTCTAAAATGCCTTCTTTCAAT
GCCATAAACTTTCCTCTAAGCACTGCTTTTGTTGCATCTCACAAATTTTGATAAGTTGTATTT
TCATTTTCATTTATTTCAAAATATTGCTTAATTTCTCTGGAGACCTTTTCCTTTGACTGATGTG
TTATTTAGAAGTGTTTTGTTTGATCTCCAGGTATTTTTTAATTTTCTAATTGTCTTCCTGTTATT
GATTTCTAGTTTTGTTGTGTTCAGAGAGCATATTTTATTTTTTAGTTCTTTTAAACTTGTTGAG
GTGTGTCATGGCTTAGAATTTGGTCTGTCTTGGTGAATGTTCCACATAAGCATGAGAAGAAT
GTATTTTGCTGTTGCTTCACAAAATATTCCATAGATA--
>Colobus_guereza-HERVHF_5LTR+flanking(-)
--GAATGTCAGGCCTCTGAGCCCAAGCTAAGCCATCATATCCCCTGTGACCTGCAC-TATACAT
CCAGATGGCCTGAAGTAACTGAAGAATCACAAAAGAAGTGAAAATGGCCTATTCCTGCCTT
AACTGATGACATTACCTTGTGAAATTCCTTCTCCTGGCTCATCCTGGCTCGAAAGCTCCCCC
ACTGAGCACCTTGTGA-CCCCCACCCC-GCCAGCCAAAAGACAACCCCCCTTTGACTGT-AAT
TTTCCACTACCTACCCAAATATTATAAAACGGCCCCACCCT-ATCTCCCTTCACTGACTCTCTT
TTTGGACTCAGCCCACCTGCACCCAGGTGAAGTAAACAGTCTTGTTGCTCACGCAAAGCCT
GTTTGGTGGTCTCTTCACACAGACGTGTGCGAAACTGACCACTGTGGGCACCTGTCATTAG
GACCTTCTGAGGCTGGGTCCCAGCCGTGTGTCCTCAAACTTGGCACAATAAACTTTCTAAA
CTAACTGAGACCTGTCTCAGATTTTTAGGGTTCATATTATCAATTAAAAACCAAAATTTACAT
T----AAAAAAAGGAAAGCAGTGTTGAGGGGGGCATCCTTACCTTGCTCCTGATCTTAGCAGG
GACACTTCAAGTTTCTCACCATTAAGTGTGACCACTGTGGGCTTTACATC-CGGT-----------CG
GGCTCCTTACCAAGTTCCCCTCTAGTCCTCATTTGCTGAGAGTGTTTATCATGAATGGGTGTT
GGATTTTGTTAGATGCTTTTTCTGAATCTGC---TATGATCATGTGACTTTTCTTCTTCAGCCTA
TTGTGGTGATGGATTACATGACTAGATTTTCATATGCTGAACCAGTTTTGTGTACCTGAGATA
AGTCCCACTCTGTT---

---GTGGTATATAAT
TCTTTTTATGCATTGTTTGGATTTGACTTGCTAATTTTTCGTTGAAGATTTTTGCATCTATGTTT
ATGAAAGCTATTGGCTTATGGCTTTCCCTTCTTGTGTCTTTGTCTGGTTTTGTTTTTAGGGTA
ACGCTGGCTTTATAGAATGAGTCAGAAAACATTCCTTTTGCTTCTATTATCTGGAAGACATCA
TAGAAAATTACTGTCATTTCTTCCTTAAATGTTTGGTAGAATTCATCAGCAAACCCGTTCAGA
CCTGGTGTTTCCTATTTGGAAGGTAATTATTTATTGATTCAATTCCTTTAATGCATACATACCTA
TTCATATTATCTATTCTTTCTTGTGTAAGTTTTGGTAGATTGCATCTTTCAGAAATTGATCCATT
TCATCTAGGTTATCATATTGCTGGGCATAGAGTTGTTATGGTAATAATATTCTATTATTATCCTT
TACAACATTCCTTTAGGATCAGTAGCAGTGG-CCCCTATTTTATTTCTAATGCTAATAATTTGT
GTCTCATTTCTTTCTTTGTTAACTTGGCTAGAGGTTTATCAATTTCATTGC---------TTTTTTTTC
AAAGATCCAGCTTTCGGTTTTGTTGATTTTTCTCTAGTAGGTTCCTGATTTCAGTTTCACTGA

TGTCTGCTTTAGTTAATTTTTTCTGTCTACTATGTTTTAATTAGTTCTTCTTTTCTAGTTTCTT
GAGGTGGAAACTTAGATGATTTGTTTCTGATCTTTCTTCTTTTCTAAAATGCCTTCTTTCAAT
GCCATAAACTTTCCTCTAAGCACTGCTTTTGTTGCATCTCACAAATTTTGATAAGTTGTATTT
TCATTTTCATTTATTTCAAAATATTGCTTAATTTCTCTGGAGACCTTTTCCTTTGACTGATGTG
TTATTTAGAAGTGTTTTGTTTAATCTCCAGGTATTTTTTAATTTTCCAGTTGTCTTTCTGTTATT
GATTTCTAGTTTTATTGTATTCAGAGAGCATATTTTATTTTTTAGTTCTTTTAAACCTGTTGAG
GTGTGTCAGGGCTTAGAATTTGGTCTATCTTGGTGAATGTTCCACATGAGCGTGAGAAGAAT
GTATTTTGCTGTTGCTTCACAAAATATTCTACAGATAT---
>Erythrocebus_patas-HERVHF_5LTR+flanking(-)
--GAATGTCAGGCCTCTGAGCCCAAGCTAAGCCATCATATCCCCTGTGACCTGCAC-TATACAT
CCAGATGGCCTGAAGTAACTGAAGAATCACAAAAGAAGTGAAGGTGGCCTATTCCTGCCTT
AACTGATGACATTACCTTGTGAAATTCCTTCTCCTGGCTCATCCTGGCTCAAAAGTTCCCCC
ACTGAGCACCTTGTGA-CCCCCACCCCTGCCAGCCAAAA-ATAACCCCCTTTGACTGT-AATT
TCCCACTACCTACCCAAATCCTATAAAACGACCCCACCCCTATCTCCCTTCACTGACTCTCTT
TTCGGACTCAGCCCACCTGCACCCAGGTGAAATAAACAGTCTTGTTGCTCACACAAAGCCT
GTTTGGTGGTCTCTTCACATGGACGTGCGTGAAACTGACCACTGTGGGCACATGTCATCAG
GATCTTCTGAGGCTGGGTCACAGCCGTGTGTCCTCAAACTTGGCAAAATAAACTTTCTAAAT
TAACTAAGACTTGTCTCAGATTTTCGGGGTTCATATTATCAATTAAAAACCAAAATTTAAATT-
AAAAAAAAGAGGAAAGCAGTGTTGAGGGGGGCGTCCTTACCTTGCTCCTGATCTTAGCAG
GGAAGCTTCAAGTTTCTCACCATTAAGTGTGACCACTGTTGGCTTTTTGTC-GAGTTTTGAC
AGCTGCAGGCTCCTTATCAAGTTCCCCTCTAGTCCTCATTTGCTGAGAGCGTTTATCATGAAT
GGGTGTTGGATTTTGTTAGATGATTTTTCTGAATCTGC---TATGATCATGTGACTTTTCTTCTT
TAGCCTGTTGAGGTGATGGATTACATGACTAGATTTTCAAATGCTGAACTAGTTTTGTGTACC
TGAG---

---GTGGTATATAATT
CTTTTTATGCATTGTTTGGATTTGACTTGCTAATTTTTCGTTGAAGATTTTTGCATCTATGTTTA
TGAAAGCTACTGGCTTATGATTTTCCCTTCTTGTGTCTTTGTCTGGTTTTCTTTTTAGGGTAAT
GCTGGCTTTATAGAATGCATTAGAAAACATTCCTTTTGCTTCTATTATCTGGAAGACATCATA
GAAAATTACTATCGTTTCTTCCTTAAATGTTTGGCAGAATTCATCAGCAAACCTGTTCAGAC
CTGGTGCTTCCTATTTGGAAGGTAATTATTTATTGATTCAATTCCTTTAATGCATACATACCTAT
TCATATTATCTATTCTTTCTTGTGTAAGTTTTGGTAGATTGCATCTTTAAGAAATTGATCCATTT
CATCTAGGTTATCATATTTCTGGGCATAGAGTTGTTATGGCAATAATATTCTATTATTATCCTTT
ACAATATTCCTCTAGGATCAGTAGCAGTGG-CCCCTATTTTATTTCTAATGCTAACAATTTGTG
TCTCATTTCTTTCTTTGTTAACTTGGCTAGAGGTTTATCAATTTCATTCC-TTTT-TTTTTTTTT
TTTAAGAACCAGCTTTTGGTT---TTGATTTTTCTCTAGTAGGTTCCTGATTTCAGTTTCACTGA
TGTCTGCTCTAGTTAATTTTTTCTGTCTACTATGTTTTAATTAGTTCTTCTTTTCTAGTTTCTT
GAGGTGAAAACTTAGATGATTTGTTTCTGATCTTTCTTCTTTTCTAAAATGCCTTCTTTCAAT
GCCATAAACGTTCCTCTAAGCACTGCTTTTGTTGCATCTCACAAATTTTGATAAGTTGTATTT
TCATTTTCATTTATTTCAAAATATTGCTTAATTTCTCTGGAGACCTTTTCCTTTGACTGATGTG
TTATTTAGAAGTGTTTTGTTTGATCTCCAGGTATTTTTTAATTTTCCAATTGTCTTCCTGTTATT
GATTTCTAGTTTTGTTGTGTTCAGAGAGCATATTTTATTTTTTAGTTCTTTTAAACTTGTTGAG
GTGTGTCACGGCTTAGAATTTGGTCTGTCTTGGTGAATGTTCCACATAAGCATGAGAAGAAT

GTATTTTGCTGTTGCTTCACAAAATATTCCATAGAT--
>Homo_sapiens-HERVHF_5LTR+flanking(-)
--GAATGTCAGGCCTCTGAGCCCAAGCTAAGCCATCGTATCCCCTGTGACCTGCACATATACA
TCCAGATGGCCTGAAGTAACTGAAGAATCACAAAAGAAGTGAAAATGGCCTGTTCCTGCCT
TAACTGATGACATTACCTTGTGAAATTCCTTCTCCTGGCTCATCCTGGTTCAAAAGCTCCCC
AACTCAGCACCTTGTGA-CCTCCACCCCTGCCAGCCATAAAACAACCCCCTTTGACTGT-AA
TTTTCCACTACCTACCCAAATCCTATAAAATGGCCCCACCCCTATCTCCCTTCCCTGACACTG
TTTTCGGACTCAGCCCGCCTGCACCCAGGTGAAATAAACAGCCTTGTTGCTCACACAAAGC
CTGTTTGGTGGTCTCTTCACAGGGACGTGCGTGAAACTGACCACCTTGGACACATGTCATC
AGGACCTCCTGAGGCTGGGTCACAGCCTTGTGACCTCAAACTTGGCAAAATAAACTTTCTA
AATTAACTGAGACCTGTCTCAGATTTTCGAGGTTCATATTATCAATTAAAAACCAAAATTTAA
ATT--AAAAAAAAAGGAAAGCAGTGTTGAGGAGGGCATTCTTACCTTGCTCCTGATCTTAGC
AGGGAAGCTTCAAGTTTCTCAACTTTAAGTGCAACCACTGTTGGCTTTTTGTT-GAGCTTTG
ACAGCTGTGGGTTCCTTATCAAGTTCCCCTCTAGTCATC-TTTGCTGAGAGCTTTTATCATGA
ATGGGTGTTGGATTTTGTTAGATGCTTTTTCTGAATCTGCTGATATGATCATGTGATTTTTCTT
CTTAAGCCTGTTGAGGTGATGGATTACATGACTAGATTTTCAAATGCTGAACCAGTTTTGTG
TACCTGAGATAAATCCCACTCGGTT---

GTGGTATATAATTCTTTTTATGCATTG-TTGGATTTGACTTGCTAATTTTTCATCGAAGATTTTT
GCACCTATGTTTACGAAAGCTATTGGCTTATGGCTTTCCCTCCTTGTGTCTTTGTCTGGTTTT
GGTTTTAGAGTAATGCTGGCTTTATAGAATGAGTTACAAAACATTCCTTTTGCTTCTATTATCT
GGAAGACATCATAGAAAATTACTATCATTTCTTCCTTAAATGTTTGGTAGAGTTCATCAGTGA
ACCCATTCAGACCTAGTTCTTTCTGTTTGGAAGGTAGTTATTTATTGATTCAATTCCTTTAATG
CATACATACCTATTCATATTATCTATTCTTTCTTGTGTAAGTTTTGGTAGATTGCATCTTTAAGA
AATTGATCTATTTCTTCTAGGTTATTATATTTGTGGGCACAGAGTTGTTATGGTAATAACATTC
TATTATTATCCTTTACAATATTCCTTTAGAATCAGTAGCAGTGG-CCTCTATTTTATTTCTAATG
CTAATAATTTGTGTCTCATTTCTTTCTTTGTTAACTTGGCTAGAGGTTTATCAATTTCATAGG---
---TTTTTTTTTTTAAAGAACCAGCTTTTGGTTTTGTTGA-TTTTCTCTAGTAGGTTCCTGATTT
CAGTTTCATTGATGTCTGCTTTAGTTAATTTTTTCTGTCTACTCTGTTTTTAATTAG---TTCTTT
TCTAGTTTCTTGAGGTGGAAACTTAGATGATTTGTTTCAGATCTTTCTTCTTTTCTAAAATGC
CTTATTTCAATGCCATAAACTTTCTTCTAAACACTGCTTTTGTTGCATCTCACAAATTTTGATA
AGTTGTATTTTCATTTTCATTTATTTCAAAATATTGCTTAATTTCTCTGGAGACCTTTTTCTTTA
ACTGACGTGTTATTTAGAAGTGTGTTGTTTAATCTCCAGGTATTTTTTAATTTTCCAATTGTCT
TTCTGTTATTGATTTCTAGTTTTATTGTGTTCAGAGAGCATATTTTATTTTTCCATTCTTTTAAA
CTTGTTGAGGTGTGTCAGGGCTTAGAATTTGGTCTATCTTGGTGAATGTTCCACGTGAACTT
GAGAAGAATGTATTTTGCTGTCGCTTAACAA---
-
>Lophocebus_aterrimus-HERVHF_5LTR+flanking(-)
--GAATGTCAGGCCTCTGAGCCCAAGCTAAGCCATCATATCCCCTGTGACCTGCAC-TATACAT
CCAGATGGCCTGAAGTAACTGAAGAATCACAAAAGAAGTGAAAGTGGCCTATTCCTGCCTT
AACTGATGACATTAATTTGTGAAATTCCTTCTCCTGGCTCATCCTGGCTCAAAAGTTCCCCC
ACTGAGCACCTTGTGATCCCCCACCCCTGCCAGCCAAAA-ATAACCCCCTTTGACTGT-AATT

TTCCACTACCTACCCAAATCCTATAAAACGACCCCACCCCTATCTCCCTTCACTGACTCTCTT
TTCGGACTCAGCCCACCTGCACCCAGGTGAAATAAACAGTCTTGTTGCTCACACAAAGCCT
GTTTGGTGGTCTCTTCACATGGACGTGCGTGAAACTGACCACTGTGGGCACATGTCATCAG
GACCTTCTGAGGCTGGGTCACAGCCGTGTGTCCTCAAACTTGGCAAAATAAACTTTCTAAA
TTAACTAAGACTTGTCTCAGATTTTCGGGGTTCATATTATCAATTAAAAAACAAAATTTAAAT
T-TAAAAAAAGAGGAAAGCAGTGTTGAAGGGGGCATCCTTACCTTGCTCCTGATCTTAGCA
GGGAAGCTTCAAGTTTCTCACCATTAAGTGTGACCACTGTTGGCTTTTTGTC-GAGTTTTGA
CAGCTGCAGGCTCCTTATCAAATTCCCCTCTAGTCCTCATTTGCTGAGAGCGTTTATCATGAA
TGGGTGTTGGATTTTGTTAGATGATTTTTCTGAATCTGC---TATGATCATGTGACTTTTCTTCT
TTAGCCTGTTGAGGTGACGGATTACATGACTAGATTTTCAAATGCTGAACCAGTTTTGTGTA
CCTGAGATAAATCCCACTCTGTT--

---GT
GGTATATAATTCTTTTTATGCATTGTTTGGATTTGACTTGCTAATTTTTCGTTGAAGATTTTTG
CATCTATGTTTATGAAAGCTATTGGCTTATGGCTTTCCCTTCTTGTGTCTTTGTCTCGTTTTGT
TTTTAGGGTAATGCTGGCTTTATAGAATGCGTTAGAAAACATTCCTTTTGCTTCTATTATCTGG
AAGACATCATAGAAAATTACTATAGTTTCTTCCTTAAATGTTTGGCAGAATTCATCAGCAAAC
CCGTTCAGACCTGGTGCTTCCTATTTGGAAGGTAATTATTTATTGATTCAATTCCTTTAATGCA
TACGTACCTATTCATATTATCTATTCTTTCTTGTGTAAGTTTTGGTAGATTGCATCTTTAAGAAA
TTGATCCATTTCATCTAGGTTATCATATTTCTGGGCATAGAGTTGTTATGGTAATAATATTCTAT
TATTATCCTTTACAATATTCCTCTAGGATCAGTAGCAGTGG-CCCCTATTTTATTTCTAATGCTA
ATAATTTGTGTCTCATTTCTTTCTTTGTTAACTTGGCTAGAGGTTTATCAATTTCATTCC------T
TTTTTTTTTTCAAAGAACCAGCTCTTGGTT---TTGATTTTTCTCTAGTAGGTTCCTGATTTCAG
TTTCACTGATGTCTGCTTTAGTTAATTTTTTCTGTCTACTATGTTTTTAATTAGTTCTTCTTTTC
TAGTTTCTTGAGGTGGAAACTTAGATGATTTGTTTCTGATCTTTCTTCTTTTCTAAAATGCCT
TCTTTCAATGCCATAAACTTTCCTCTAAGCACTGCTTTTGTTGCATCTCACAAATTTTGATAA
GTTGTATTTTCATTTTCATTTATTTCAAAATATTGCTTAATTTCTCTGGAGACCTTTTCCTTTGA
CTGATGTGTTATTTAGAAGTGTTTTGTTTAATCTCCAGGTATTTTTTAATTTTCCAATTGTCTT
TCTGTTATTGATTTCTAGTTTTATTGTGTTCAGAGAGCATATTGTATTTTTTAGTTCTTTTAAAC
TTGTTGAGGTGTGTCAGGGCTTAGAATCTGGTCTGTCTTGGTGAATGTTCCACATAAGCATG
AGAAGAATGTATTTTGCTGTTGCTTCACGA--
>Macaca_mulatta-HERVHF_5LTR+flanking(-)
--GAATGTCAGGCCTCTGAGCCCAAGCTAAGCCATCATATCCCCTGTGACCTGCCC-TATACAT
CCAGATGGCCTGAAGTAACTGAAGAATCACAAAAGAAGTGAAAGTGGCCTGTTCCTGCCTT
AACTGATGACATTACCTTGTGAAATTCCTTCTCCTGGCTCATCCTGGCTCAAAAGTTCCCCC
ACTGAGCACCTTGTGA-CCCCCACCCCTGCTAGCCAAAA-ATAACCCCCTTTGACTGT-AATT
TTCCACTACCTACCCAAATCCTATAAAACGACCCCACCCCTATCTCCCTTCACTGACTCTCTT
TTCGGACTCAGCCCACCTGCACCCAGGTGAAATAAACAGTCTTGTTGCTCACACAAAGCCT
GTTTGGTGGTCTCTTCACATGGACGTGCGTGAAACTGACCACTGTGGGCACATGTCATCAG
GACCTTCTGAGGCTGGGTCACAGCCGTGTGTCCTCAAACTTGGCAAAATAAACTTTCTAAA
TTAACTAAGACTTGTCTCAGATTTTCGGGGTTCATATTATCAATTAAAAAACAAAATTTAAAT
T-TAAAAAAAGAGGAAAGCAGTGTTGAGGGGGGCATCCTTATCTTGCTCCTAATCTTAGCAG
GGAAGCTTCAAGTTTCTCACCATTAAGTGTGACCACTGTTGGCTTTTTGTT-GAGTTTTGAC

AGCTGCAGGCTCCTTATCAAGTTCCCCTCTAGTCCTCATTTGCTGAGAGCGTTTATCATGAAT
GGGTGTTGGATTTTGTTAGATGATTTTTCTGAATCTGC---TATGATCATGTGACTTTTTTTCTTT
AGCCTGTTGAGGTGATGGATTACATGACTAGATTTTCAAATGCTGAACCAGTTTTGTGTACC
TGAGATAAATCCCACTCTGTT--

---GTGG
TATATAATTCTTTTTATGCATTGTTTGGATTTGACTTGCTAATTTTTCATTGAAGATTTTTGCAT
CTATGTTTATGAAAGCTATTGGCTTATGGCTTTCCCTTCTTGTGTCTTTGTCTGGTTTTGTTTT
TAGGGTAATGCTGGCTTTATAGAATGTGTTAGAAAACATTCCTTTTGCTTCTATTATCTGGAA
GACATCATAGAAAATTACTATCATTTCTTCCTTAAATGTTTGGCAGAATTCATCAGCAAACCC
GTTCAGACCTGGTGCTTCCTATTTGGAAGGTAATTATTTATTGATTCAATTCCTTTAATGCATA
CATACCTATTCATATTATCTATTCTTTCTTGTGTAAGTTTTGGTAGATTGCATCTTTAAGAAATT
GATCCATTTCATCTAGGTTATCATATTTCTGGGCATAGAGTTGTTATGGTAATAATATTCTATTA
TTATCCTTTACAATATTCCTCTAGGATCAGTAGCAGTGG-CCCCTATTTTATTTCTAATGCTAAT
AATTTGTGTCTCATTTCTTTCTTTGTTAACTTGGCTAGAGGTTTATCAATTTCATTCC-------TTT
TTTTTTTCAAAAAACCAGCTTTTGGTT---TTGATTTTTCTCTAGTAGGTTCCTGATTTCAGTTT
CACTGATGTCTGCTTTAGTTAATTTTTTCTGTCTACTATGTTTTTAATTAGTTCTTCTTTTCTAG
TTTCTTGAGGTGGAAACTTAGATGATTTGTTTCTGATCTTTCTTCTTTTCTAAAATGCCTTCTT
TCAATGCCATAAACTTTCCTCTAAGCACTGCTTTTGTTGCATCTCACAAATTTTGATAAGTTG
TATTTTCATTTTCATTTATTTCAAAATATTGCTTAATTTCTCTGGAGACCTTTTCCTTTGACTGA
TGTGTTATTTAGAAGTGTTTTGTTTAATCTCCAGGTATTTTTTAATTTTCCAATTGTCTTTCTG
TTATTGATTTCTAGTTTTATTGTGTTCAGAGAGCATATTTTATTTTTTAGTTCTTTTAAACTTGT
TGAGGTGTGTCAGGGCTTAGAATCTGGTCTGTCTTGGTGAATGTTCCACATAAGCACGAGA
AGAATGTATTTTGCTGTTGCTTCATGAAA--
>Macaca_silenus-HERVHF_3LTR+flanking(+)
---AATGTCAGGCCTCTGAGCCCAAGCTAAGCCATCATATCCCCTGTGACCTGCAC-TATACAT
CTAGATGGCCTGAAGTAACTGAAGAATCACAAAAGAAGTGAAAGTGGCCTGTTCCTGCCTT
AACTGATGACATTACCTTGTGAAATTCCTTCTCCTGGCTCATCCTGGCTCAAAAGTTCCCCC
ACTGAGCACCTTGTGA-CCCCCACCCCTGCCAGCCAAAA-ATAACCCCCCTTTGACTGT-AATT
TTCCACTACCTACCCAAATCCTATAAAACGACCCCACCCCTATCTCCCTTCACTGACTCTCTT
TTCGGACTCAGCCCACCTGCACCCAGGTGAAATAAACAGTCTTGTTGCTCACACAAAGCCT
GTTTGGTGGTCTCTTCACATGGACGTGCGTGAAACTGACCACTGTGGGCACATGTCATCAG
GACCTTCTGAGGCTGGGTCACAGCCGTGTGTCCTCAAACTTGGCAAAATAAACTTTCTAAA
TTAACTAAGACTTGTCTCAGATTTTCGGGGTTCATATTATCAATTAAAAAACAAAATTTAAAT
T-TAAAAAAAGAGGAAAGCAGTGTTGAGGGGGGCATCCTTACCTTGCTCCTAATCTTAGCAG
GGAAGCTTCAAGTTTCTCACCATTAAGTGTGACCACTGTTGGCTTTTTGTA-GAGTTTTGAC
AGCTGCAGGCTCCTTATCAAGTTCCCCTCTAGTCCTCATTTGCTGAGAGCGTTTATCATGAAT
GGGTGTTGGATTTTGTTAGATGATTTTTCTGAATCTGC---TATGATCATGTGACTTTTTTTCTTT
AGCCTGTTGAGGTGATGGATTACATGACTAGATTTTCAAATGCTGAACCAGTTTTGTGTACC
TGAGATAAATCCCACTCTGTT--

---GTGG

TATATAATTCTTTTTATGCATTGTTTGGATTTGACTTGCTAATTTTTCGTTGAAGATTTTTGCAT
CTATGTTTATGAAAGCTATTGGCTTATGGCTTTCCCTTCTTGTGTCTTTGTCTGGTTTTGTTTT
TAGGGTAATGCTGGCTTTATAGAATGTGTTAGAAAACATTCCTTTTGCTTCTATTATCTGGAA
GACATCATAGAAAATTACTATCATTTCTTCCTTAAATGTTTGGCAGAATTCATCAGCAAACCC
ATTCAGACCTGGTGCTTCCTATTTGGAAGGTAATTATTTATTGATTCAATTCCTTTAATGCATA
CATACCTATTCATATTATCTATTCTTTCTTGTGTAAGTTTTGGTAGATTGCATCTTTAAGAAATT
GATCCATTTCATCTAGGTTATCATATTTCTGGGCATAGAGTTGTTATGGTAATAATATTCTATTA
TTATCCTTTACAATATTCCTCTAGGATCAGTAGCAGTGG-CCCCTATTTTATTTCTAATGCTAAT
AATTTGTGTCTCATTTCTTTCTTTGTTAACTTGGCTAGAGGTTTATCAATTTCATTCC-------TTT
TTTTTCCCAAAAAACCAGCTTTTGGTT---TTGATTTTTCTCTAGTAGGTTCCTGATTTCAGTTT
CACTGATGTCTGCTTTAGTTAATTTTTTCTGTCTACTATGTTTTTAATTAGTTCTTCTTTTCTAG
TTTCTTGAGGTGGAAACTTAGATGATTTGTTTCTGATCTTTCTTCTTTTCTAAAATGCCTTCTT
TCAATGCCATAAACTTTCCTCTAAGCACTGCTTTTGTTGCATCTCACAAATTTTGATAAGTTG
TATTTTCATTTTCATTTATTTCAAAATATTGCTTAATTTCTCTGGAGACCTTTTCCTTTGACTGA
TGTGTTATTTAGAAGTGTTTTGTTTAATCTCCAGGTATTTTTTAATTTTCCAATTGTCTTTCTG
TTATTGATTTCTAGTTTTATTGTGTTCAGAGAGCATATTTTATTTTTTAGTTCTTTTAAACTTGT
TGAGGTGTGTCAGGGCTTAGAATCTGGTCTGTCTTGGTGAATGTTCCACATAAGCATGAGA
AGAATGTATTTTGCTGTTGCTTCATGAAAT---
>Mandrillus_leucophaeus-HERVHF_3LTR+flanking(+)
---AATGTCAGGCCTCTGAGCCCAAGCTAAGCCATCATATCCCCTGTGACCTGCAC-TATACAT
CCAGATGGCCTGAAGTAACTGAAGAATCACAAAAGAAGTGAAAGTGGCCTGTTCCTGCCTT
AACTGATGACATTAATTTGTGAAATTCCTTCTCCTGGCTCATCCTGGCTCAAAAGTTCCCCC
ACTGAGCACCTTGTGA-CCCCCACCCCTGTCAGCCAAAA-ATAACCCCCTTTGACTGT-AATT
TTCCACTACCTACCCAAATCCTATAAAACGACCCCACCCCCATCTCCCTTCAGTGACTCTCTT
TTCGGACTCAGCCCGCCTGCACCCAGGTGAAATAAACAGCCTTGTTGCTCACGCAAAGCCT
GTTTGGTGGTCTCTTCACATGGACGTGCGTGAAACTGACCACTGTGGGCACATGTCATCAG
GACCTTCTGAGGCTGGGTCACAGCCGTGTGTCCTCAAACTTGGCAAAATAAACTTTCTAAA
TTAACTAAGACTTGTCTCAGATTTTCGGGGTTCATATTATCAATTAAAAAACAAAATTTAAAT
T-TAAAAAAAGAGGAAAGCAGTGTTGAGGGGGGCATCCTTACCTTGCTCCTGATCTCAGCA
GGGAAGCTTCAAGTTTCTCACCATTAAGTGTGACCACTGTTGGCTTTTTGTC-GAGTTTTGA
CAGCTGCAGGCTCCTTATCAAGTTCCCCTCTAGTCCTCATTTGCTGAGAGCGTTTATCATGAA
TGGGTGTTGGATTTTGTTAGATGATTTTTCTGAATCTGC---TATGATCATGTGACTTTTCTTCT
TTAGCCTGTTGAGGTGATGGATTACATGACTAGATTTCAAATGCTGAACCAGTTTTGTGTA
CCTGAGATAAATCCCACTCTGTT---

--GT
GGTATATAATTCTTTTTATGCATTGTTTGGATTTGACTTGCTAATTTTTCGTTGAAGATTTTTG
CATCTATGTTTATGAAAGCTATTGGCTTATGGCTTTCCCTTCTTGTGTCTTTGTCTGGTTTTGT
TTTTAGGGTAATGCTGGCTTTATAGAATGCGTTAGAAAACATTCCTTTTGCTTCTATTATCTGG
AAGACATCATAGAAAATTACTATCTTTTCTTCCTTAAATGTTTGGCAGAATTCATCAGCAAAC
CTGTTCACACCTGGTGCTTCCTATTTGGAAGGTAATTATTTATTGATTCAATTCCTTTAATGCA
TACATACCTATTCATATTATCTATTCTTTCTTGTGTAAGTTTTGGTAGATTGCATCTTTAAGAAA
TTGATCCATTTCATCTAGGTTATCATATTTCTGGGCATAGAGTTGTTATGGTAATAATATTCTAT

TATTATCCTTTACAATATTCCTCTAGGATCAGTAGCAGTGG-CCCCTATTTTATTTCTAATGCTA
ATAATTTGTGTCTCATTTCTTTCTTTGTTAACTTGGCTAGAGGTTTATCAATTTCATTCC--------
TTTTTTTTTCAAAGAACCAGCTTTTGGTT---TTGATTTTTCTCTAGTAGGTTCCTGATTTCAGT
TTCACTGATGTCTGCTTTAGTTAATTTTTTCTGTCTACTATGTTTTTAATTAGTTCTTCTTTTCT
AGTTTCTTGAGGTGGAAACTTAGATGATTTGTTTCTGATCTTTCTTCTTTTCTAAAATGCCTT
CTTTCAATGCCATAAACTTTCCTCTAAGCACTGCTTTTGTTGCATCTCACAAATTTTGATAAG
TTGTATTTTCATTTTCATTTATTTCAAAATATTGCTTAATTTCTCTGGAGACCTTTTCCTGTGA
CTGATGTGTTATTTAGAAGTGTTTTGTTAATCCCCAGGTATTTTTTAATTTTCCAGTTGTCTT
TCTGTTATTGATTTCTAGTTTTATTGTGTTCAGAGAGCATATTTTATTTTTTAGTTCTTTTAAAC
TTGTTGAGGTGTGTCAGGGCTTAGAATCTGGTCCGTCTTGGTGAATGTTCCACATAAGCATG
AGAAGAATGTATTTTGCTGTTGCTTCACGAAATA---
--

>Pongo_abelii-HERVHF_5LTR+flanking(-)
--GAATGTCAGGCCTCTGAGCCCAAGCTAAGCCATCGTATCCCATGTGACCTGCACGTACACA
TCCAGATGGCCTGAAGTAACTGAAGAATCACAAAAGAAGTGAAAATGGCCTGTTCCTGCCT
TAACTGATGATATTACCTTGTGAAATTCCTTCTCCTGGCTCATCCTGGTTCAAAAGCTCCCCA
ACTGAGCACCTTGTGA-CCCCCACCCCTGCCAGCCAGTAAACAACCCCCTTTGACTGT-AAT
TTTCCACTACCTACCCAAATCCTATAAAACGGCCCCACCCCTATCTCCCTTCCCTGACTCTCT
TTTCGGACTCAGCCCGCCTGCACCGAGG------------------TGCTCACACAAAGCCTGTTTAGTG
GTCTCTTCACAGGGACGCGCGTGAAACTGACCACCTTGGACACATGTCATCATTACCTCCT
GAGGCTGGGTCATAGCCCTGTGTCCTCAAACTTGGCAAAATAAACTTTCTAAATTAACTGAG
ACCTGTCTCAGATTTTTGAGGTTCATATTATCAATTAAAAACCAAAATTTAAATTAAAAAAA
AAAAGGAAAGCAGTGTTGAGGAGGGCATCCTTACCTTGCTCCTGATCTTAGCAGGGAAGCT
TCAAGTTTCTCAACTTTAAGTGCGACCACTGTTGGCTTTTTGTT-GAGCTTTGACAGCTGTG
GGTTCCTTATCAAGTTCCCCTCTAGTCCTCTTTTGCTGAGAGCTTTTATCGTGAATGGGTGTT
GGATTTTGTTAGATGCTTTTTCTGAATCTGCTGATATGATCATGTGATTTTTCTTCCTCAGCCT
GTTGAGGTGATGGATTACATGACTAGATTTTCAAATGCTGAACCAGTTTTGTGTACCTGAGA
TAAATCCCACTCGGTT--

---GTGGTATATA
ATCCTTTTTATGCATTG-TTGGATTTGACTTGCTAATTTTTCATTGAAGATTTTTGCACCTATGT
TTACAAAAGCTATTGGCTTATGGCTTTCCCATCTTGTGTCTTTGTCTGGTTTTGGTTTTAGAG
TAATGCTGGCTTTATAGAATGAGTTAGAAAACATTCCTTTTGCTTCTATTATCTGGAAGACAT
CATAGAAAATTACTATCATTTCTTCCTTAAATGTTTGGTAGAATTCATCAGCGAACCCATTCA
GACCTGGTGCTTCCTGTTTGGAAGGTAGTTATTTATTGATTCAATTCCTTTAATGCATACATAC
CTATTCATATTATCTATTCTTTCTTGTGTAAGTTTTGGTGGATTGCATCTTTAAGAAATTGATCC
ATTTCGTCTAGGTTATTAGATTTGTGGGCATAGAGTTGTTATGGTAATAATAGTCTATTATTAC
CCTTTACAATATTCCTTTAGAATCAGTAGCAGTGG-CCTCTATTTTATTTCTAATGCTAATAATT
TGTGTCTCATTTCTTTCTTTGTTAACTTGGCTAGAGGTTTATCAATTTCATAGA------TTTTTTT
TTTTTAAAGAACCAGCTTTTGGTTTTGTTGA-TTTTCTCTAGTAGGTTCCTGATTTCAGTTTCA
TTGATGTCTGCTTTAGTTAATTTTTTCTGTCTACTATGTTTTTAATTAG---TTCTTTTCTAGTTT
CTTGAGGTGGAAACTTAGATGATTTGTTTCAGATCTGTCTTCTTTTCTAAAATGCCTTACTTC
AATGCCATAAACTTTCTTCTAAACATTGCTTTTGTTGCATCTCACAAATTTTGATAAGTTGTAT

TTTCATTTTCATTTATTTCAAAATATTGCTTAATTTCTCTGGAGACCTTTTTCTTTGACTGATG
TGTTATTTAGAAGTGTGTTGTTTAATCTCCAGGTATTTTTTAATTTTCCAATTGTCTTTCTGTTA
TTGATTTCTAGTTTTACTGCATTCAGAGAGCATATTTTATTTTTCCATTCTTTTAAACTTGTTG
AGGTGTGTCAGGGCTTAGAATTTGGTCTATCTTGGTGAATGTTCCACGTGAGCTTGAGAAG
AATGTATTTTGCTGTTGCTTAACAAAAGATTCTATAGATA---
--
>Pygathrix_nemaeus-HERVHF_3LTR+flanking(+)
TAGAATGTCAGGCCTCTGAGCCCAAGCTAAGCCATCATATCCCCTGTGACCTGCAC-TATACA
TCCAGATGGCCTGAAGTAACTGAAGAATCACAAAAGAAGTAAAAATGGTCTGTTCCTGCCT
TAACTGATGACATTACCTTGTGAAATTCCTTCTCCTGGCTCATCCTGGCTCAAAAGCTCCCC
CACTGAGCACCTTGTAA-CCCCCACCCCTGCCAGCCAAAAGACAACCCCCCTTTGACTGT-AA
TTTTCCACTACCTACCGAAATATTATAAAACAGCCCCACCTC-ATCTCCCTTCACTGACTCTC
TTTTCGGACTCAGCCCTCCTGCACCCAGGTGAAATAAACAGTCTTGTTGCTCACACAAAGC
CTGTTTGGTGGTCTCTTCACACGGACGTGTGCAAAACTGACCACTGTGGGCACAAGTCATT
AGGACCTTCTGAGGCTGGGTCACAACCGTGTGTCCTCAAACTTGGCAAAATAAACTTTCTA
AACTAACTGAGACCTGTCTCAGATTTTTAGGGTTCATATTATCAATTAAAAATCAAAATTTAA
ATT--AAAAAAAAAGGAAAGCAGTGTTGAGGGGGGCATCCTTACCTTGCTCCTGATCTTAGC
AGGGACACTTCAAGTTTCTCACCATTAAGTGTGACCACTGTGGGCTTTATGTC-AAGT---------
--CGGGCTCCTTATCAAGTTTCCCTCTAGTCCTCATTTGCTGAGAGCATTTATCATGAATGGGT
GTTGGATTTTGTTAGATGCTTTTTCTGAATCTGC---TATGATCATGTGACTTTTCTTCTTCAGC
CTATTGTGGTGATGGATTACATGACTAGATTTTCAAATGCTGAACCAGTTTTGTGTACCTGAG
ATAAATCCCACTCTGTT---

--GTGGTATAT
AATTCTTTTTATGCATTGTTTGGATTTGACTTGCTAATTTTTCATTGAAGATTTTTGCATCTATA
TTTACGAAAGCTATTGGCTTATGGCTTTCCCTTCTTGTGTCTTTGTCTGGTTTTGTTTTTAGG
GTAACACTGGCTTTATAGAATGAGTCAGAAAACATTCCTTTTGCTTCTATTATCTGGAAGACA
TCATAGAAAATTACTATTATTTCTTCCTTAAATGTTTGGTAGAATTCATCAGCAAACCCGTTC
AGACCTGGTGCTTCCTATTTGGAAGGTAATTATTTATTGATTCAATTCCTTTAATGCATACATA
CCTATTCATATTATCTATTCTTTCTTGTGTAAGTTTTGGTAGATTGCATCTTTAAGAAATTGATC
CATTTCATCTAGGTTATCATATTTCTGGGCGTTGAGTTGTTATGGTAATAATATTCTATTATTAT
CCTTTACAATATTCCTTTAGGATCAGTAGCAGTGGCCCCCTATTTTATTTCTAATGCTAATAAT
TTGTGTCTCATTTCTTTCTTTGTTAACTTGGCTAGAGGTTTATTAATTTCATTGC----T-TTTTTT
TTTTTCAAAGAACCAGCTTTCGGTTTTGTTGATTTTTCTCTAGTAGGTTCCTGATTTCCGTTT
CATTGATGTCTGCTTTAGTTAAATTTTTCTGTCTACTATGTTTTTAATTAGTTCTTCTTTTCTAG
TCTCTTGAGGTGGAAACTTAGATGATTTATTTCTGATCTTTCTTCTTTTCTAAAATGCCTTCTT
TCAATGCCATAAACTTTCCGCTAAGCACTGCTTTTGTTGCATCTCACAAATTTTGGTAAGTTG
TATTTTCATTTTCATTTATTTCAAAATATTGCTTAATTTCTCTGGAGACCTTTTCCTTTGACTGA
TGTGTTATTTAGAAGTGTTTTGTTTAATCTCCAGGTATTTTTAAATTTTCCAATTGTCTTTCTG
TTATTGATTTCTAGTTTTATTGTGTTCAGAGAGCATATTTTATTTTTTAGTTCTTTTAAACCTGT
TGAGGTGTGTCGGGGCTTAGAATTTGGTCTGTCTTGGTGAATGTTCCACATGAGCATGAGA
AGAATGTATTTTGCTGTTGCTTCACAAAATATT---
>Rhinopithecus_roxellana-HERVHF_5LTR+flanking(-)

--GAATGTCAGGCCTCTGAGCCCGAGCTAAGCCATCATATCCCCTGTGACCTGCACGTGTACA
TCCAGATGGCCTGAAGTAACTGAAGAATCACAAAAGAAGTGAAAATGGCCTGTTCCTGCCT
TAACTGACAACGTTACCTTGTGAAATTCCTTCTCTTGGCTCATCCTGGCTCAAAAGCTCCCC
CGCTGAGCACCTTGTAA-CCCCCACCCCTGCCAGCCAAAAGACAACCCCCTTTGACTGT-AA
TTTTCCACTACCTACCCAAATATTATAAAACAGCCCCACCCC-ATCTCCCTTCACTGACTCTC
TTTTCGGACTCAGCCCTCCTGCACCCAGGTGAAATAAACAGTCTTGTTGCTCACACAAAGC
CTGTTTGGTGGTCTCTTCACACGGACGTGTGCAAAACTGACCACTGTGGGCACAAGTCATT
AGGACCTTCTGAGGCTGGGTCACAACCGTGTGTCCTCAAACTTGGCAAAATAAACTTTCTA
AACTAACTGAGACCTGTCTCAGATTTTAGGGTTCATATTATCAATTAAAAATCAAAATTTAA
ATT----AAAAAAAGGAAAGCAGTGTTGAGGGGGGCATCCTTACCTTGCTCCTGATCTTAGCA
GGGACACTTCAAGTTTCTCACCATTAAGTGTGACCACTGTGGGCTTTATGTC-AAGT----------
CGGGCTCCTTATCAAGTTTCCCTCTAGTCCTCATTTGCTGAGAGCATTTATCATGAATGGGTG
TTGGATTTTGTTAGATGCTTTTTCTGAATCTGC---TATGATCATGTGACTTTTCTTCTTCAGTCT
ATTGTGGTGATGGATTACATGACTAGATTTTCAAATGCTGAACCAGTTTTGTGTACCTGAGAT
AAATCCCACTCTGTT---

--GTGGTATATAA
TTCTTTTTATGCATTGTTTGGATTTGACTTGCTAATTTTTCGTTGAAGATTTTTGCATCTATATT
TACGAAAGCTATTGGCTTATGGCTTTCCCTTCTTGTGTCTTTGTCTGGTTTTGTTTTTAGGGT
AACACTGGCTTTATAGAATGAGTCAGAAAACATTCCTTTTGCTTCTATTATCTGGAAGACATC
ATAGAAAATTACTATCATTTCTTCCTTAAATGTTTGGTAGAATTCATCAGCAAACCTGTTCAG
ACCTGGTGCTTCCTATTTGGAAGGTAATTATTTATTGATTCAATTCCTTTAATGCATGCATACC
TATTCATATTATCTATTCTTTCTTGTGTAAGTTTTGGTAGATTGCATCTTTAAGAAATTGATCCA
TTTCATCTAGGTTATCATATTTCTGGGCATTGAGTTGTTATGGTAATAATATTCTATTATTATCCT
TTACAATATTCCTTTAGGATCAGAAGCAGTGG-CCCCTATTTTATTTCTAATGCTAATAATTTGT
GTCTCATTTCTTTCTTTGTTAACTTGGCTAGAGGTTTATCAATTTCATTGC--------TTTTTTTT
CAAAGAACCAGCTTTCGTTTTTGTTGATTTTTCTCTAGTAGGTTCCTGATTTCCGTTTCATTG
ATGTCTGCTTTAGTTAAATTTTTCTGTCTACTATGTTTTTAATTAGTTCTTCTTTTCTAGTTTCT
TGAGGTGGAAACTTAGATGATTTATTCTGATCTTTCTTCTTTTCTAAAATGCCTTCTTTCAAT
GCCATAAACTTTCCGCTACGCACTGCTTTTGTTGCATCTCACAAATTTGGTAAGTTGTATTT
TCATTTTCATTTATTTCAAAATATTGCTTAATTTCTCTGGAGACCTTTTCCTTTGACTGATGTG
TTATTTAGAAGTGTTTTGTTTAATCTCCAGGTATTTTTAAATTTTCCAATTGTCTTTCTGTTATT
GATTTCTAGTTTTATTGTGTTCAGAGAGCATATTTTATTTTTTAGTTCTTTTAAACCTGTTGAG
GTGTGCCGGGGCTTAGAATTTGGTCTGTCTTGATGAATGTTCCACATGAGCATGAGAAGAAT
GTATTTTGCTGTTGCTTCACAAAATATTCTATAGA---
>Rhinopithecus_strykeri-HERVHF_3LTR+flanking(+)
---GCACGTGTACATCCAGATGGCCTGAAGTAACTGAA
GAATCACAAAAGAAGTGAAAATGGCCTGTTCCTGCCTTAACTGACGACATTACCTTGTGAA
ATTCCTTCTCTTGGCTCATCCTGGCTCAAAAGCTCCCCCGCTGAGCACCTTGTAA-CCCCCA
CCCCTGCCAGCCAAAAGACAACCCCCTTTGACTGT-AATTTTCCACTACCTACCCAAATATTA
TAAAACAGCCCCACCCC-ATCTCCCTTCACTGACTCTCTTTTCGGACTCAGCCCTCCTGCAC
CCAGGTGAAATAAACAGTCTTGTTGCTCACACAAAGCCTGTTTGGTGGTCTCTTCACACGG
ATGTGTGCAAAACTGACCACTGTGGGCACAAGTCATTAGGACCTTCTGAGGCTGGGTCACA

ACCGTGTGTCCTCAAACTTGGCAAAATAAACTTTCTAAACTAACTGAGACCTGTCTCAGATT
TTGAGGGTTCATATTATCAATTAAAAATCAAAATTTAAATT----AAAAAAAGGAAAGCAGTGT
TGAGGGGGGCATCCTTACCTTGCTCCTGATCTTAGCAGGGACACTTCAAGTTTCTCACCATT
AAGTGTGACCACTGTGGGCTTTATGTC-AAGT-----------CGGGCTCCTTATCAAGTTTCCCTCT
AGTCCTCATTTGCTGAGAGCATTTATCATGAATGGGTGTTGGATTTTGTTAGATGCTTTTTCT
GAATCTGC---TATGATCATGTGACTTTTCTTCTTCAGTCTATTGTGGTGATGGATTACATGACT
AGATTTTCAAATGCTGAACCAGTTTTGTGTACCTGAGATAAATCCCACTCTGTT----------------
----

---GTGGTATATAATTCTTTTATGCATTGTTTGGATT
TGACTTGCTAATTTTTCGTTGAAGATTTTGCATCTATATTTACGAAAGCTATTGGCTTATGGC
TTTCCCTTCTTGTGTCTTTGTCTGGTTTTGTTTTTAGGGTAACACTGGCTTTATAGAATGAGT
CAGAAAACATTCCTTTTGCTTCTATTATCTGGAAGACATCATAGAAAATTACTATCATTTCTT
CCTTAAATGTTTGGTAGAATTCATCAGCAAACCCGTTCAGACCTGGTGCTTCCTATTTGGAA
GGTAATTATTTATTGATTCAATTCCTTTAATGCATGCATACCTATTCATATTATCTATTCTTTCTT
GTGTAAGTTTTGGTAGATTGCATCTTTAAGAAATTGATCCATTTCATCTAGGTTATCATATTTC
TGGGCATTGAGTTGTTATGGTAATAATATTCTATTATTATCCTTTACAATATTCCTTTAGGATCA
GAAGCAGTGG-CCCCTATTTTATTTCTAATGCTAATAATTTGTGTCTCATTTCTTTCTTTGTTAA
CTTGGCTAGAGGTTTATCAATTTCATTGC--------TTTTTTTTTCAAAGAACCAGCTTTCGTTTT
TGTTGATTTTTCTCTAGTAGGTTCCTGATTTCCGTTTCATTGATGTCTGCTTTAGTTAAATTTT
TCTGTCTACTATGTTTTTAATTAGTTCTTCTTTTCTAGTTTCTTGAGGTGGAAACTTAGATGAT
TTATTTCTGATCTTTCTTCTTTTCTAAAATGCCTTCTTTCAATGCCATAAACTTTCCGCTAAGC
GCTGCTTTTGTTGCATCTCACAAATTTTGGTAAGTTGTATTTTCATTTTCATTTATTTCAAAAT
ATTGCTTAATTTCTCTGGAGACCTTTTCCTTTGACTGATGTGTTATTTAGAAGTGTTTTGTTTA
ATCTCCAGGTATTTTTAAATTTTCCAATTGTCTTTCTGTTATTGATTTCTAGTTTTATTGTGTTC
AGAGAGCATATTTTATTTTTTAGTTCTTTTAAACCTGTTGAGGTGTGTCGGGGCTTAGAATTT
GGTCTGTCTTGGTGAATGTTCCACATGAGCATGAGAAGAATGTATTTTGCTGTTGCTTCACA
AAATATTCTATAGATATCCAGTAAATCAGCCGAATGACAGCGCTGTTCTGTTCAATTATGTCC
T
>Theropithecus_gelada-HERVHF_5LTR+flanking(-)
--GAATGTCAGGCCTCTGAGCCCAAGCTAAGCCATCATATCCCCTGTGACCTGCAC-TATACAT
CCAGATGGCCTGAAGTAACTGAAGAATCACAAAAGAAGTGAAAGTGGCCTGTTCCTGCCTT
AACTGATGACATTAATTTGTGAAATTCCTTCTCCTGGCTCATCCTGGCTCAAAAGCTCCCCC
ACTGAGCACCTTGTGA-CCCCCACCCCTGCCAGCCAAAA-ATAACCCCCTTTGACTGT-AATT
TTCCACTACCTACCCAAATCCTATAAAACGACCCCACCCCTATCTCCCTTCACTGACTCTCTT
TTCGGACTCAGCCCGCCTGCACCCAGATGAAATAAACAGTCTTGTTGCTCACACAAAGCCT
GTTTGGTGGTCTCTTCACATGGACGTGCGTGAAACTGACCACTGTGGGCACATGTCATCAG
GTCCTTCTGAGGCTGGGTCACAGCCGTGTGTCCTCAAACTTGGCAAAATAAACTTTCTAAA
TTAACTAAGACTTGTCTCAGATTTTCGGGGTTCATATTATCAATTAAAAAACAAAATTTAAAT
T-TAAAAAAAGAGGAAAGCAGTGTTGAGGGGGGCATCCTTACCTTGCTCCTGATCTTAGCA
GGGAAGCTTCAAGTTTCTCACCATTAAGTGTGACCACTGTTGGCTTTTTGTG-GAGTTTTGA
CAGCTGCAGGCTCCTTATCAAGTTCCCCTCTAGTCCTCATTTGCTGAGAGCGTTTATCATGAA
TGGGTGTTGGATTTTGTTAGATGATTTTTCTGAATCTGC---TATGATCATGTGACTTTTCTTCT

TTAGCCTGTTGAGGTGACGGATTACATGACTAGATTTTCAAATGCTGAACCAGTTTTGTGTA
CCTGAGATAAATCCCACTCTGTT---

--GT
GGTATATAATTCTTTTTATGCATTGTTTGGATTTGACTTGCTAATTTTTCGTTGAAGACTTTTG
CATCTATGTTTATGAAAGCTATTGGCTTATGGCTTTCCCTTCTTGTGTCTTTGTCTCGTTTTGT
TTTTAGGGTAATGCTGGCTTTATAGAATGCGTTAGAAAACATTCCTTTTGCTTCTATTATCTGG
AAGACATCATAGAAAATTACTATCGTTTCTTCCTTAAATGTTTGGCAGAATTCATCAGCAAAC
CCGTTCAGACCTGGTGCTTCCTATTTGGAAGGTAATTATTTATTGATTCAATTCCTTTAATGCA
TACATACCTATTCATATTATCTATTCTTTCTTGTGTAAGTTTTGGTAGATTGCATCTTTAAGAAA
TTGATCCATTTCATCTAGGTTATCATATTTCTGGGCATAGAGTTGTTATGGTAATAATATTCTAT
TATTATCCTTTACAATATTCCTCTAGGATCAGTAGCAGTGG-CCCCTATTTTATTTCTAATGCTA
ATAATTTGTGTCTCATTTCTTTCTTTGTTAACTTGGCTAGAGGTTTATCAATTTCATTCC--------
TTTTTTTTTCAAAGAACCAGCTCTTGGTT---TTGATTTTTCTCTAGTAGGTTCCTGATTTCAGT
TTCACTGATGTCTGCTTTAGTTAATTTTTTCTGTCTACTATGTTTTTAATTAGTTCTTCTTTTCT
AGTTTCTTGAGGTGGAAACTTAGATGATTTGTTTCTGATCTTTCTTCTTTTCTAAAATGCCTT
CTTTCAATGCCATAAACTTTCCTCTAAGCACTGCTTTTGTTGCATCTCACAAATTTTGATAAG
TTGTATTTTCATTTTCATTTATTTCAAAATATTGCTTAATTTCTCTGGAGACCTTTTCCTTTGAC
TGATGTGTTATTTAGAAGTGTTTTGTTTAATCTCCAGGTATTTTTTAATTTTCCAATTGTCTTT
CTCTTATTGATTTCTAGTTTTATTGTGTTCAGAGAGCATATTTTATTTTTTAGTTCTTTTAAACT
TGTTGAGGTGTGTCAGGGCTTAGAATCTGGTCTGTCTTGGTGAATGTTCCACATAAGCATGA
GAAGAATGTATTTTGCTGTTGCTTCATGAAAT--

Supplementary data S6 The alignments of the internal sequences of a widely vertical transmitted HERV-H in 17 species

>Cercocebus_atys-HERVHF_INT(-)
---GCTAGTGAAATTTGGTGCCGTGACTCAGACTGG
GGGACCTCCCTTGGGAGATCAATCCCCTGTCCTCCTGCTCTTTGCTCCGTGAGAAAGATCCA
CTTACGACCTCAGGTCCTCAGACCAACCAGCCCCAAGAACATCTCACCAATTTTAAATCGG
GTAAGCAGCCTCTCTTTACTCTCTTCCCCAACTTCTCTCACTATCCCTCAACCTCTTTCTCCT
TTCAATCTTGGTGCCACACTTCAATCTCTCCCTTCTCTTAATTTCAGTTCCTTTCCTTTTCTGG
TAGAGACAGAGGAGACGCGTTTTATCCGTGAACCCAAAACTCCTGCACCGGTCACCGACTC
GGGAAGACAGTCTTCCCTTGGTGTTTAATCCCTGCAGGGACGTCTGCCTGATTATTCACCCA
CATTTCATTGGTGTCTGATCACCACAGGGATGCCTGCCTTCGTCATTCACCCACAATCCCTC
GGTGGCAAGTCCATTGCAGGGACACCTGCTTTGGCTGCTCACCCACATTGCAGCCCAGGGC
TGCTCACCCCAC--CCCTTCTCTCCGTGTCT
CTACCCTCTCTTCTCTCCA-------------------------------------CTTTCTTGGGGGGCTAGCACCCCC
CACCC----------CTTCTCTCTGTGTCTCTACCC---TCTCT
TTTCTCTGCACTTGCCTCCTTCATTATGGGCAAACTTCCACACCCTCCATTCCTCCTTCTTCT
CCCTTAGCCTGTGTTCTCAAGAACTTAAAACCTCTTCAACTCACAACTGACCTAAAACCTA
AATGCCTTATTTCTTCTGCAACACGGCTTGGCCCCAAAATACAAACTCGAC-AATGGTTCC
AAATAGCCAGAAAACGGCACTTTCGATTTCTCCATCCTACAAGATCTAGATAATTCTCATCAT
AAAATGGGCAAATAGTCTGAGGTGCCTGATGTCCAGGCATTCTTTACACATGAGTCCCTCC
CTGGTCTCTGTTCCCAATGCAACTCGTCCC---AAATCTTCC
TTCTTTCCCTCACGCCTGTCCCCTCAGTCCCAACCCCAAGCGTTGCTGAGTCTTTCCAAAGT
CTTCCTTTTTTACGGACCCATCTGACCTCTCCCCTCCTCCCCAGGCTGCTCCTCACCAGGCT
GAGCCAGGTCCCAA-TTCTTCCTCAGCCTCCGTTCCCCCACCCTATAATCCTTTTATCAGCTC
CCCTCCTCACACCCAGTCCGACTTGCAGTTTCGTTTTGCAACTAGCCCTCCCTCACCTGCCC
AGCAATTATCTCTTAAAAATGTGG-------CTGGAGCCGAAGGCATAGTCAGGGTTAATGCTCC
TTTTTCTTTATCTGGCCTCTCCCAAATCAGTTAGCGTTTAGGCTCTTTTTCATCAAATATAAAA
TCCCGGCCCAGTTCATGGCTTGTTTGGCAGCAACCCTGAGATGCTTTACTGCCCTAGACCCT
GAAAGGTCAAAAGGCCGTCTTATTCTCAATATACAT---TGTATTACCCAATCCACTCCTGACA
TTAAATAAAACACCAAAAATTAAGTTCCGGCCCTCAAACCCCACAACAAGACTTAATTAAC
CTTGCCTTCAAGGTGTACAATAATAGAGAAGAGTTGCAATTACTTGCCTCCGCTGTGAGAG
AAACCCCAGCCACATCTTCAGCACACAAGAACTTCAAAATGCCTAAGCCACAGCAGCCAG
GCGTTCCTTCGGGACTTTCTCCCCCAGGATCTTGCTTCAAGTGCTGGAAATCTGGCCACTGG
GCCAAGGAATGACCGTAGCCCAGAATTCC-TCCTAAACTGTGTCCCATCTGTGCAGGACCCC
ACTGGAAATTGGACTGTCCAACTGGCCCAAGGCTCTAACTGACTCCTTCCCAGATCTTCTC
AGCTTA--GGCTGAAGACTGATGCTGCCCGATCGCCTTGGAAGCTTCCTGGACCATCCCAGA
CGCTTTGGGTAACTCTTACAGTGGAGGGTAAGTCCATCCCCTTCTTAATCAATATGGAGGCT
ACCCACTCCACATTACCTTCTTTTCAAGGGCCTGTTTCCCTTGCCTCCATAAATGTTGTGGGC
ATTGACAGCCAGGTTTCTAAACCCCTTAAAACTCCCCCAGTCTGGTGCTAACTTGGACAAC
ATTCTTTTGTGCACTCTTTTTCAGTTATCCCCACCTGCCCAGTTCCCTTATTAGGCCGAGATAT
TTTAACCAAATTGTCTGCTTCCCTGACTATTCCTGGACTACAGCCACATCTCATTGCTACCCT
GCTTCCCAACCCAAAGCCTCCTTTGCACCTTCCTCTCGTATTCCCCGAACTTAATCCACATGT

ATGGGACACCTCTA---CTCCCTCCCTGGCAACCAATCACCAGCCTATTACTATCCCATTAAAA
CCTAATCACCCTTACCCCATTCAATGCCAGTATCCCATCCCACAACAGGCTTTAAAGGGATT
GAAACCTGTTATCACTTGCCTGCTACAGTATGGGCTTCTAAAACCTGTAAACTCCCCTTATAA
TTCCCCCATTTACCTGTCCAAAAACTGGACAAGTCTTACAGGTTACTTCAGGATCTGTGCC
TTATCAACCAAATTGTTTTGCCTAT---CCTTGTGGTGCGACACCCATTTACTCTCCTATCCTCA
ATACCTCCCTGCACAACCCATTATTCTGTTCTGGGTCTCAAACATGCTTTCTTTACTATTCCTT
TGCATCCTTCATCCCAGCCTCTTTTCGCTTTCACTTGGACTGACCCTGACACCCATCAGGCT
CAGGAAATTACTTGGGCTGTACTGCTGCAAGGCTTCACGGACAGCCCCTATTACTTCAGTCA
AGCCCAAATTTCTTCCTCATCTGTTACCTATCTTGGCATAATTC-TCATAAAAACACAC--GTG
CTCTCCCTGCTGATCGTGTCTGGCTAATCTCCCAAACCCCAACCCCTTCTACAAAACAACAA
CTCCTTCCTTC-GTAGGCATGGTTAGCTACTTCCACCTTTGGATACCTAGTTTACCATCTTGA
CTAAACCACGATGTAAACTCACAAAAGGAAACCTAGCTGACCCCACAGATCCTAAATCCTT
TTGCCACTCCTTTCCATTCCTTAAAAACAGCCCTAGAAGCTACTCCCACACTAGCTCTCCCT
AACTCATCCCAATCCTTTTTCATTACACACAGTCCAAGTGCAGGGCTGTGAGGTCAAAATT
CTTACACAAGGATCGGGACCATGCCCTGTGGCCTTTTTATCCAAACAACTTGACCTTACTCT
TTTAGCTTAG-CCTCATGTCTGTGTGTGGCAGCTGCTGCTTCCTTAATACTTTCAGAGGCCCT
CAAAATCACAAACTATGCTCAAC---------TCACTCTCTAGAGTTCTCATAACTTCCAAAATCTA
TTTTCTTCCTCACACCTGATGCACATATTTCCTGCC---CCCCTCCACTACCTCTCAACAAGCC
AAACTCATTGCCTTAACTCAAGCCCTCGCTCTTGCAAAAGGATTAAGTGTCAATATTTATACT
GACTCTAAATATGCCTTCCATATCCTGCACCACCATGCTGTTATATGGGCAGAAAGAAATGTC
CTAACTATGCAAGGGTCCTCCATCATTAATGCCTCTTTAATAAAAACTCTTCTCAAAGCTGCT
TTACTTCCAAAGGAAGCTGGAATCATTCACTGCAAGGACCATCAAAAGGCATCAGATCCCA
TTGCTCAGGACAATGCTTATGCTGATACGGTAGCTAAA-AAGCAGCTAGCATTCCAACTTCTA
TCCCTCACGGCAGTTTTTCTCCTTCTCATTTGGCCACTCCCACCTACTCCCCCACTGAAACT
TCCACCTATCAGTCTCTTCCCACGCAAGGCAAATGGTTCTTGGACCAAGGAAAATATCTCCT
TCCAGCCTCACAGGCCCATTCTGTTCTGTCATCATTTCATAACCTCTTCCATGTAGGTTACAA
GCTGCTAGCCCAACTCTTAGAACCTCTCATTTCCTTTCCATCGTGGAGATCTATCCTCAAGG
AAATCACTTCTCAG-TGTTCCATCTGCTATTCTACTACTCCTCAGGGATTGTTCAGGCCCCCT
CCCTTCACTACACATCAAGCTTGGTGATTTGCCCCTGCCCAGGACTGGCAAATTGACTTTAC
TCACATGCCCCGAGTCAGGAAACTAAAATACCTCTTGGTCTAGGTAGACACTTTCACTGGAT
GGGTAGAGGCCTTTCCCACAGGGTCTGCGAAGGCCACCATGGTCATTTCTTCCCTTCTGTC
AGGTATAATTCCTCGGTTTGACCTTCCTACCTCTATACAGTCCAATAATGGACCAGCCTTTGT
TAGTTAAATCACCCAACCAGTTTCTCAGGCTCTTAG---TATTCAGTGGAACTTTCATACCCCT
TACTATCCTCAGTCTTCAGGAAAGGTA-----GAACGGGCTAATGGTCTTTTAAAAACACAACT
CACCAAGCTCAGCCACCAACTTAAAAAGGACTAGACAATACTTTTACTACTTTCCCTTCTCA
AAATTCGGGCCTGCCCTCGGGATGCTACAGGGTACAGCCCATTAGAGCTTCTGTATGAACCC
TCCTATTTATTAGGCCCCAGTCTTATTCCAGACACCAGCCCAACTTGGACTGTGCCCCAAAA
ACTTGTCATCCCTACTGTCTTCTGTCTAGTCATACTCCTATTCACCGTTCTCAAATACTCATAA
ATGCCCTGCTCTTGTTTACACTGCCGGTTTACACTGTTTCTCCAAGCCATCACAGCTGATATC
TCCTGGTGCTAT-CCCAAACTGCCACTCTTAACTCCCTCTTAAAGTAAATAAATAATCTTTGCT
GGCAAGGCTATGCTGAACCTCCTTGGGCACTCTCTAATTGGATGTCCTGGATCCTCCCAATT
CTTAGTCCTTTAATACCTGTTTTTCTCCTTCTCTTATTCAGACCTTTT-GTCTTCCGTTTAGTTT
TTCAATTCATACAAAACTGCTTCCAGGCCATCACCAATCATTCTATATGATAAATGCTCCTTCT
AACAACCCCACAATATCACCCCTTACCACAAAATCTTCTTTCAGCTTAATCTCTCCCACTCT

GGGTTTCCATGCCACCCCTAATCCTGCATGAAGCAGCCCTGAGAAACATCACCCATTATCTC
TCCATGCCACCCCCCAAAAAATTTTTGC-TGCCCCAACACTTCAATACTATTTGTGTTATTTT
TCTTATTAATATAAGAAGGCAGGAATGTCAGGCCT------------------------
>Cercopithecus_mitis-HERVHF_INT(-)
--AGGGGGACCTCTCTTGGG
AGATCACTCCCCTGTCCTCCTGCTCTTTGCTCCATGAGAAAGATCCACCTACGACCTCAGGT
CCTCAGACCAACCAGCCCCAAGAACATCTCACCAATTTTAAATCGGGTAAGCAGCCTCTCT
TTACTCTCTTCTCCAACCTCTCTCACTATCCCTCAACCTCTTTCTCCTTTCAATCCTGGTGCC
ACACTTCAATCTCTCCCTTCTCTTAATTTCAGCTCCTTTCCTTTTCTGGTAGAGACAGAGGA
GACGCGTTTTATCCGTGAACCCAAAACTCCGGCACCGGTCACCGACTCGGGAAGACAGTC
TTCCTTTGGTGTTTAATCCCTGCAGGGACGTCTGCCTGATTATTCACCCACATTTCATTGGTG
TCTGATCACCGCAGGGACGCCTGCCTTGGTCATTCACCCACAATCCCTTGGTGGCAAGTCC
ATTGCAGGGACGCCTGCTTTGGCTGCTCACCCACATTGCAGCCTAGGGCTGCTCACCCCAC
CCCTTCTCTCAGTGTCTCTACCCTCTCTTCTCTGCCACTTTCTTGGGGGGCTGGCACCCCCC
CACGCCCTTCTCTCCGTGTCTCTATCCTCTCTTCTCTCCA--------------------------------------CTTT
CTTGGGGGGCTGGCACCCCCCACCC----------CTTCTCTCCGTGTCTCTACCCTCTCTTCTCTG
CACTTTCTTGGGGGGCTGGCACCCCCCACCCCTTCTCTCTGTGTCTCTTTTCTCTGGACTTG
CCTCCTTCACTATGGGCAAACTTC--CACCCTCCATTTCTCCTTCTTCTCCCTTAGCCTGTGTT
CTCAAGAACTTAAAACCTCTTCAACTTACACCTGACCTAAAACCTTAATGCCTTATCTTCTT
CTGCAATGCCGCTTAACCCC--AATACAAACTCGAC-AATGGTTCCA--------GAAAACGGCACT
TTCGATTTCTCCATCCTACAAGATCTAGATAATTCTTGTCATAAAATGGGCAAATAGTCTGAG
GTGCCTGATGTCCAGGCATTCTTTTACATATGGGTCCCTCCCTGGTCTCTGTTCCCAATGCAA
CTCGTCCC---AAATCTTCCTTCTTTCCCTCCCGCCTGTCCC
CTCAGTCCCAACCCCAAGAGTTGCTGAGTCTTTCTAA--TCTTCCTTTTTTACGGACCCAACT
GACCTCTCCCCTCCTCCCCAGGCTGCTCCTCGCCAGGCCAAGCCAGGTCCCAA-TTCTTCCT
CAGCCTCCGTTCTCCCACCCTATAATCCTTTTATCACCTCCCCTCCTCACACCCAGTCCGACT
TGCAGTTTCGTTTTGCAACTAGCCCTCCCCCACCTGCCCAGCAATGATCTCTTAAAAATGTG
G-------CTGGAGCTGAAGGCATAGTCAGGGTTAATGCTCCTTTTTCTTTATCTGGCCTCTCCCA
AATCAGTTAGCGTTTAGGCTCTTTTTCATCAAATATAAAAACCCGGCCCAGTTCATGGCTTGT
TTGGCAGCAACCCTGAGATGCTTTACTGCCCTAGACCCTGAAAGGTCAAAAGGCTGTCTTA
TTCTCAATATACAT---TGTATTACCCAATCCACTCCTGACATTAAGTAAAACACCAAAAATTAA
GTTCCGGCCCTCAAACTCCACAACAAGACTTAATTAACCTTGCCTTGAAGGTGTACAATAAT
AGAGAAGAGTTGCAATTACTTGCCTCCACTGTGAGAGAAACCCCAGCCACATCTTCAGCAT
ACAAGAACTTCAAAATGCCTAAGCCACAGCAGCCAGGCGTTCCTTCAGGACTGTCTCCCCC
AGGATCTTGCTTCAAGTGCTGGAAATCTGGCCACTGGGCCAAGGAATGCCCGTAGCCCAGG
ATTCCTTCCTAAACTGTGTCCCATCTGTGCAGGACCCCACTGGAAATTGGACTGTCCAACTG
GCCCAAGGCTCTGACTGACTCCTTCCCAGATCTTCTCAGCTTA--GGCTGAAGACTGACGCT
GCCCGATTGCCTTGGAAGCTTCCTGGACCATCCCAGACGCTTTGGGTAACTCTTACAGTGG
AGGGTAAGTCCGTCCCCTTCTTAATCAATATGGAGGCTACCCACTCCACATTACCTTCTTTTC
AAGGGTCTGTTTCCCTTGCCTCCATAACTGTTGTGGGCATTGACAGCCAGGTTTCTAAACCC
CTTATAACTCCCCTACTCTGGTGCTAACTTGGACAATATTCTTTTATGCACTCTTTTTTAGTTC
TCCCCACCTGCCCAGTTCCCTTATTAGGCCAAGATATTTTAACCAAATTGTCTGCTTCCCTGA
CTCTTCCTGGACTACAGCCACATCTAATTGCTACCCTGCTCCCCAACCCAAAGCCTCCTTCG
CGCCTTCCTCTCGTATTCCCCGAACTTAACCCACGTGTATGGGACACCTCTA---CTCCCTCCC

TGGCAACCAATCACCAGCCTATTACTATCCCATTAAAACCTAATCACCCTTACCCCATTCAAT
GCCAGTATCCCATCCCACAACAGGCTTTAAAGGGATTGAAACCTGTTATCACTCGCCTGCTA
CAGCATGGGCTTCTAAAACCTGTAAACTCCCCTTACAATTCCCCCATTTTACCTGTCCAAAA
ACTGGACAAGTCTTACAGGTTACTTCAGGATCTGTGCCTTATCAACCAAATTGTTTTGCCTAT
CCACCTTGTGGTGCAAAACCCATTTACTCTCCTATCCTCAATACCTCCCTCCACAACCCAGTA
TTCTGTTCTGGGTCTCAAACATGCTTTCTTTACTATTCCTTTGCATCCTTCATCCCAGCCTCTT
TTCGCTTTCACTTGGACTGACCCTGACACCCATCAGGCTCAGGAAATTAC-TGGGCTGTACT
GCTGCAAGGCTTCACGGACAGCCCCTATTACTTCAGTCAAGCCCAAATTTCTTCCTCATCTG
TTACCTATCTTGGCATAATTC-TCATAAAAACACACATGTGCTCTCCCTGCTGATCATGTCTGG
CTAATCTCCCAAACCCCAACCGCTTCTACAAAACAACAACTCCTTCCTTCAGTAGGCATGGT
TAGCTACTTCCACCTTTGGATACCTAGTTTTACCATCCTGACTAAACCACGATGTAAACTCAC
AAAAGGAAACCTAGCTGACCCCACAGATCCTAAATCCTTTTGCCACTCCTTTCCATTCCTTA
AAAACAGCCCTAGAAGCTGCTCCCACACTAGCTCTCCCTAACTCATCCCAACCC-TTTTTCA
TTACACACAGTCAAAGTGTAGGGCTGTGAGGTCAAAATTCTTACACAAGGATCGGGACCAT
GCCCTGTGGCCTTTTTATCCAAACAACTTGACCTTACTGTTTTAGCTTAG-CCTCATGTCTGT
GTGTGGCAGCTGCTGCTTCCTTAATACTTTCAGAGGCCCTCAAAATCACAAACTATGCTCAA
C---------TCACTCTCTAGAGTTCTCATAACTTCCAAAATCTATTTTCTTCCTCACACCTGATGC
ACATATTTCCTGGC---CCCCTCCACTACCTCTCAACAAGCCAAACTCATTGCCTTAACTCAAG
CCCTCGCCCTTGCAAAAGGATTAAGTGTCAATATTTATACTGACTCTAAATATGCCTTCCATA
TCCTGCACCACCATGCTGTTATACGGGCTGAAAGAAATGTCCTAACTATGCAAGGGTCCTCC
ATCATTAATGCCTCTTTAATAAAAACTCTTCTCAAAGCTGCTTTACTTCCAAAGGAAGCTGG
AATCATTCACTGCAAGGGCCATCAAAAGGCATCAGATCCCATTGCTCAGGACCATGCTTATG
CTGATACGGTAGCTAAA-AAGCAGCTAGCATTCCAACTTCTATCCCTCACGGCAGTTTTTCTC
CTTCTTATTTGGCCACTCCCACCTACTCCCCCGCAGAAACTTCCACCTATCAATCTCTTCCCA
CGCAAGGCAAATGGTTCTTGGACCAAGGAAAATATCTCCTTCCAGCCTCACAGGCCCATTC
TATTCTGTCATCATTTCATAACCTCTTCCATGTAGGTTACAAGCCGCTAGCCCATCTCTTAGA
ACCTCTCATTTCCTTTCCATCGTGGAGATCTATCCTCAAGGAAATCACTTCTCAG-TGTTCCA
TCTGCTATTCTACTATTCCTCAGGGATTGTTCAGGCCCCCTCCCTTCCCTACACATCAAGCTT
CGGGATTTGCCCCTGCCCAGGACTGACAAACTGACTTTACTCACATGCCCTGAGTCAGGAA
ACTAAAATACCTCTTGGTCTAGATAGACACTTTCACTGGATGGGTAGAGGCCTTTCCCACAG
GGTCTGCGAAGGCCACCGCGGTCATTTCTTCCCTTCTGTCAGGTATAATTCCTCAGTTTGAC
CTTCCTACCTCTATACAGTCCAATAATGGACCAGCCTTTGTTAGTTAAATCACCCAACCAGTT
TCTCAGGCTCTTAG---TGTTCAGTGGAACTTTCATACCCCTTACCATCCTCAATCTTCAGGAA
AGGTA-----GAACGGGCTAATGGTCTTTTAAAAACACACCTCACCAATCTCAGCCACCAACTT
AAACAGGACTAGACAATACTTTAACCACTTTCCATTCTCAAAATTCGGGCCTGTCCTCGGGA
TGCTACAGGGTACAACCCCTTTGAGCTTCTGTATGAACCCTCCTATTTATTAGGCCCCAGTCT
TATTCCAGACACCAGCCCAACTTGGACTGTGCCCCAAAAACTTGTCATCCCTACTCTCTTCT
GTCTAGTCATACTCCTATTCACCGTTCTCAAATACTCATAAATGCCCTGCTCTTGTTTACACT
GCCGGTTTACACTGTTTCTCCAAGCCATCACAGCTGATATCTCCTGGTGCTAT-CCCAAACTG
CCACTCTTAACTCCCTCTTAAAGTAAATAAATAATCTTTGCTGGCAGGGCTATGCTGAACCTC
CTTGGACACTCTCTAATTGGATGTCCTGGATCCTCCCAATTCTTAGTCCTTTAATACCCGTTT
TTCTCCTTCTCTTATTCAGACCTTTT-GTCTTCCGTTTAGTTTTTCAGTTCATACAAAACTGCT
TCCAGGCCATCACCAATCATTCTATATGATAAATGCTCCTTCTAACAACCCCACAATATCACC
CCTTACCACAAAATCTTCCTTCAGCTTAATCTCTCCCACTCTGGGTTTCCACGCCACCCC-AA

TCCTGCATGAAGCAGCCCTGAGAAACATCACCCATTATCTCTCCATGCCTCCCCCCAAAAAA
TTTTTGCAGGCCCCAACACTTCAATACTATATTGTATTATTTTTCT--TTAATATAAGAAGGCAG

>Cercopithecus_mona-HERVHF_INT(-)

---GCTAGTGAAATTTGGTGCCGTGACTCAGATCGG
GGGACCTCTCTTGGGAGATCACTCCCCTGTCCTCCTGCTCTTTGCTGCGTGAGAAAGATCC
ACCTACGACCTCAGGTCCTCAGACCAACCGGCCCCAAGAACATCTCACCAATTTTAAATCG
GGTAAGCAGCCTCTCTTTACTCTCTTCTCCAACCTCTCTCACTATCCCTCAACCTCTTTCTCC
TTTCAATCCTGGTGCCACACTTCAATCTCTCCCTTCTCTTAATTTCAGCTCCTTTCCTTTTCTG
GTAGAGACAGAGGAGACGCGTTTTATCTGTGAACCCAAAACTCCGGCACCGGTCACCGAC
TCGGGAAGACAGTCTTCCTTTGGTGTTTAAATCCTGCAGGGACGTCTGCCTGATTATTCACC
CACATTTCATTGGTGTCTGATCACCGCAGGGACGCCTGCCTTGGTCATTCACCCACAATCCC
TTGGTGGCAAGTCCATTGCAGGGACGCCTGCTTTGGCTGCTCACCCACATTGCAGGCCAGG
GCTGCTCACCCCAC--CCCTTCTCTCCGTGT
CTCTACCCTCTCTTCTCTGCA--CTTTCTTGGGGGGCTAGCACCC
CCCACCC----------CTTCTCTCTGTG---TCTCTTT
TCTCTGGACTTGCCTCCTTCACTATGGGCAAACTTC--CACCCTCCATTTCTCCTTCTTCTCCC
TTAGCCTGTGTTCTCAAGAACTTAAAACCTCTTCAACTCACACCTGACCTAAAACCTTAATG
CCTTATCTTCTTCTGCAATGCCGCTTAACCCC--AATACAAA-TTGAC-AATGGTTCCAAATAGC
CAGAAAACGGCACTTTCGATTTCTCCATCCTACAAGATCTAGATAATTCTCGTCATAAAATG
GGCAAATAGTCTGAGGTGCCTGATGTCCAGGCATTCTTTTACACATGGGTCCCTCCCTGGTC
TCTGTTCCCAATGCAACTCGTCCC---AAATCTTCCTTCTTT
CCCTCCCGCCTGTCCCCTAAGTCCCAACCCCAAGAGTTGCTGAGTCTTTCTAA--TCTTCTTT
TTTTACGGACCCATCTGACCTCTCCCCTGCTCCGCAGGCTGCTCCTCGCCAGGCCAAGCCA
GGTCCCAA-TTCTTCCTCAGCCTCCGTTCCCCCACCCTATAATCCTTTTATCACCTCACCTCCT
CACATCCAGTCTGACTTGCAGTTTCGTTTTGCAACTAGCCCTCCCCCACCTGCCCAGCAATT
ATCTCTTTAAAATGTGG-------CTGGAGCTGAAGGCATAGTCAGCGTTAATGCTCCTTTTTCTT
TATCTGGCCTCTCCCAAATCGGTTAGCGTTTAGGCTCTTTTTCATCAAATATAAAAACCCGGC
CCAGTTCATGGCTTGTTTGGCAGCAACCCTGAGATGCTTTACTGCCCTAGACCCTGAAAGG
TCAAAAGGCCGTCTTATTCTCAATATACAT---TGTATTACCCAATCCACTCCTGACATTAAATA
AAACACCAAAAATTAAGTTCCGGCCCTCAAACCCCACAACAAGACTTAATTAACCTTGCCT
TGAAGGTGTACAATAATAGAGAAGAGTTGCAATTACTTGCCTCCGCTGTGA--GAAACCCCA
GCCACATCTTCAGCACACAAGAACTTCAAAATGCCTAAGCCACAGCAGCCAGGCGTTCCTT
CAGGACTTTCTCCCCCAGGATCTTGCTTCAAGTGCTGGAAATCTGGCCACTGGGCCAAGGA
ATGCCCGTAGCCCAGGATTCC-TCCTAAACTGTGTCCCATTTGTGCAGGACCCCACTGGAAA
TTGGACTGTCCAACTGGCCCAAGGCTCTGACTGACTCCTTCCCAGATCTTCTCAGCTTA--G
GCTGAAGACTGACGCTGCCCGATTGCCTTGGAAGCTTCCTGGACCATCCCAGACGCTTTGG
GTAACTCTTACAGTGGAGAGTAAGTCCGTCCCCTTCTTAATCAATATGGAGGCTACCCACTT
CACATTACCTTCTTTTCAAGGGTCTGTTTCCCTTGCCTCCATAACTGTTGTGGGCATTGACAG
CCAGGTTTCTAAACCCCTTATAACTCCCCCACTCTGGTGCTAACTTGGACAATATTCTTTTAT
GCACTCTTTTTTAGTTCTCCCCACCTGCCCAGTTCCCTTATTAGGCCAAGATATTTTAACCAA
ATTGTCTGCTTCCCTGACTATTCCTGGACTACAGCCACATCTCATTGCTACCCTGCTCCCCAA
CCCAAAGCCTCCTTCGCGCCTTCCTCTCGTATTCCCTGAACTTAACCCACGTGTATGGGACA
CCTCTA---CTCCCTCCCTGGCAACCAATCACCGGCCTATTACTATCCCATTAAAACCTAATCA

CCCTTACCCCATTCAATGCCAGTATCCCATCCCACAACAGGCTTTAAAGGGATTGAAACCTG
TTATCACTTGCCTGCTACAGTATGGGCTTCTAAAACCTGTAAACTCCCCTTACAATTCCCCCA
TTTTACCTGTCCAAAAACTGGACAAGTCTTACAGGTTACTTCAGGATCTGTGCCTTATCAAC
CAAATTGTTTTGCCTATCCACCTTGTGGTGCGAAACCCATGTACTCTCCTATCCTCAATACCT
CCCTCCACAACCCATTATTCTGTTGTGGATCTCAAACATGCTTTCTTTACTATTCCTTTGCAC
CCTTCATCCCAGCCTCTCTTCGCTTTCACTTGGACTGACCCTGACACCCATCAGGCTCAGGA
AATTACCTGGGCTGTACTGCTGCAAGGCTTCACGGACAGCCCCTATTACTTCAGTCAAGCCC
AAATTTCTTCCTCATCTGTTACCTATCTTGGCATAATTC-TCATAAAAACACAC--GTGCTCTCC
CTGCTGATCATGTCTGGCTAATCTCCCAAACCCCAACCCCTTCTACAAAACAACAACTCCTT
CCTTC-GTAGGCATGGTTAGCTACTTCCACCTTTGGATGCCTAGTTTTACCATCTTGACTAAA
CCACGATGTAAACTCACAAAAGGAAACCTAGCTGACCCTACAGATCCTAAATCCTTTTGCC
ACTCCTTTCCATTCCTTAAAAACGGCCCTAGAAGCTGCTCCCACACTAGCTCTCCCTAACTC
ATCCCAATCC-TTTTTCATTACACACAGTCAAAGTGCAGGGCTGTGGGGTCAAAATTCTTAC
ACAAGGATCGGGACCATGCCCTGTGGCCTTTTTATCCAAACAACTTGACCTTACTGTTTTAG
CTTAG-CCTCATGTCTGTGTGTGGCAGCTGCTGCTTCCTTAATACTTTCAGAGGCCCTCAAAA
TCACAAACTATGCTCAAC---------TCACTCTCTAGAGTTCTCATAACTTCCAAAATCTATTTTC
TTCCTCACACCTGATGCACATATTTCCTGGC---CCCCTCCACTACCTCTCAACAAGCCAAACT
CCTTGCCTTAACTCAAGCCCTCGCCCTTGCAAAAGGATTAAGTGTCAATATTTATACTGACT
CTAAATATGCCTTCCATATCCTGCACCACCATGCTGTTATATGGGCAGAAAGAAATGTCCTAA
CTATGCAAGGGTCCTCCATCATTAATGCCTCTTTAATAAAAACTCTTCTCAAAGCTGCTTTAC
TTCCAAAGGAAGCTGGAGTCATTCACTGCAAGGACCATCAAAAGGCATCAGATCCCATTGC
TCAGGACAGTGCTTATGCTGATACGGTAGCTAAA-AAGCAGCTAGCATTCCAACTTCTATCCC
TCACGGCAGTTTTTCTCCTTCTCATTTGGCCACTCCCACCTACTCCCCCACTGAAACTTCCA
CCTATCAATCTCTTCCCACGCAAGGCAAATGGTTCTTGGACCAAGGAAAATATCTCCTTCCA
GCCTCACAGGCCCATTCTATTCTGTCATCATTTCATAACCTCTTCCATGTAGGTTGCAAGCTG
CTAGCCCACCTCTTAGAACCTCTCATTTCCTTTCCATCGTGGAGATCTATCCTCAAGGAAATC
ACTTCTCAG-TGTTCCATCTGCTATTCTACTACTCCTCAGGGATTGTTCAGGCCCCCTCCCTT
CCCTACACATCAAGCTTCGGGATTTGCCCCTGCCCAGGACTGGCAAATTGACTTTACTCACA
TGCCCCGAGTCAGGAAATTAAAATACCTCTTGGTCTGGGTAGACACTTTCACTGGATGGGTA
GAGGCCTTTCCCACAGGGTCTGCGAAGGCCACCGCGGTCATTTCTTCCCTTCTGTCAGGTAT
AATTCTTCGGTTTGACCTTCCTACCTCTATATAGTCCAATAATGGACCAGCCTTTGTTAGTTA
AATCACCCAACCAGTTTCTCAGGCTCTTAG---TATTCAGTGGAACTTTCATACCCCTTACCAT
CCTCAATCTTCAGGAAAGGTA-----GAACGGGCTAATGGTCTTTTAAAAACACACCTCACCAA
TCTCAGCCACCAACTTAAAAAGGACTAGACAATACTTTTACCACTTTCCGTTCTCAAAATTC
GGGCCTGTCCTCGGGATGCTACAGGGTACAGCCCATTTGAGCATCTGTATGAACCCTCCTAT
TTATTAGGCCCCAGTCTTATTCCAGACACCAGCCCAACTTGGACTGTGCCCCAAAAACTTGT
CATCCCTACTCTCTTCTGTCTAGTCATACTTCTATTCACCGTTCTCAAATACTCATAAATGCCC
TGCTCTTGTTTACACTGCCGGTTTACACTGTTTCTCCAAGCCATCACAGCTGATATCTCCTGG
TGCTAT-CCCAAACTGCCACTCTTAACTCCCTCTTAAAGTAAATAAATAATCTTTGCTGGCAG
GGCTATGCTGAACCTCCTTGGGCACTCTCTGATTGGATGTCCTGGATCCTCCCAATTCTTAGT
CTTTTAATACCTGTTTTTCTCCTTCTCTTATTCAGACCTTTT-GTCTTCCGTTTAGTTTTTCAGT
TCATACAAAACTACTTCCAGGCCATCACCAATCATTCTATATGATAAATGCTCCTTCTGACAA
CCCCACAATATCACCCCTTACAACAAAATCTTCCTTCAGCTTAATCTCTCCCACTCTGGGTTT
CCACGCCACCCCTAATCCTGCATGAAGCAGCCCTGAGAAACATCACCCATTATCTCTCCATG

CCTCCCCACAAAAAATTTTTGC-GGCCCCAACACTTCAATACTATTTGTATTATTTTTCTTAT
TAATATAAGAAGGCAG-------------------------------------

>Chlorocebus_aethiops-HERVHF_INT(+)

---CTAGTGAAATTTGGTGCCATGACTCAGATCGGG
GGACCTCTCTTGGGAGATCACTCCCCTGTCCTCCTGCTCTTTGCTCCGTGAGAAAGATCCAC
TTACAACCTCAGGTCCTCAGACCAACCCGCCCCAAGAACATCTCACCAATTTTAAATCGGG
TAAGCAGCCTCTCTTTACTCTCTTCTCCAACTTCTCTCACTATCCCTCAACCTCTTTCTCCTT
TCAATCTTGGTGCCACACTTCAATCTTTCCCTTCTCTTAATTTCAGTTCCTTTCCTTTTCTGGT
AGAGACAGAGGAGACGCGTTTTATCCGTGAACTCAAAACTCCTGCACCGGTCACCGACTC
GGGAAGACAGTCTTTCTTTGGTATTTAATCCCTGCAGGGACGTCTGCCTGATTATTCACCCA
CATTTCACTGGTGTCTGATCACCGCAGGGACGCCTGCCTTGCTCATTCACCCACAATCCCTT
GGTGGCAAGTCCATTGCAGGGACGTCTGCTTTGGCTGTTCACCCACATTGCAGCCTAGGGC
TGCTCACCTCAC---CCCTTCTCTCCGTGTCT
CTACCCTCTCTTCTCTCCA--------------------------------------CTTTCTTGGGGGGCTAGCACCCCC
CATCC----------CTTCTCTCTGTGTCTCTACCC---TCTCT
TTTCTCTGGACTTGCCTCCTTCACTATGGGCAAACTTC--CACCCTCCATTTCTCCTTCTTCTC
CCTTAGCCTGTGTTCTCAAGAACTTAAAACCTCTTCAACTCACACCTGACCTAAAACCTTAA
TGCCTTATTTTCTTCTGCAATGCTGCTTAACCCC--AATACAAACTCGAC-AATGGTTCCAAAT
AGCCAGAAAACGGCACTTTCGATTTCTCCATCCTACAAGAGCTAGATAATTCTCGTCATAAA
ATGGGCAAATAGTCTGAGGTGCCTGATGTCCAGGCATTCTTTACACATGGGTTCCTCCCTG
GTCTCTGTTCCCAATGCAACTCGTCCC---AAATCTTCCTTC
TTTCCCTCCCGCCTGTCCCCTCAGTCCCAACCCCAAGAGTTGCTGAGTCTTTCTAA--TCTTC
CTTTTTTACGGACCCATCTGACCTCTCCCCTCCTCCCCAGGCTGCTCCTCGCCAGGCCGAGC
CAGGTCCCAA-TTCTTCCTCAGCCTCCGTTCCCCCACCCTATAATCCTTTTATCACCTCCCCTC
CTCACAC--------------------------AACTAGCCCTCCCCCACCTGCCCAGCAATTATCTCTTAAAA
AGGTGG-------CTGGAGCTGAAGGCATAGTCAGGGTTAATGCTCCTTTTTCTTTATCTGGCCTC
TCCCAAATCAGTTAGCGTTTAGGCTCTTTTTCATCAAATATAAAAACCCGGCCCAGTTCATG
GCTTGTTTGGTAGCAACCCTGAGATGCTTTACTGCCCTAGACCCTGAAAGGTCAAAAGGCT
GTCTTATTCTCAATATACAT---TGTATCACCCAATCCACTCCTGACATTAAATAAAACACCAAA
AATTAAGTTCCGGCCCTCAAACCCCACAACAAGACTTAA----CCTTGCCTTGAAGGTGTACA
ATAATAGAGAAGAGTTGCAATTACTTGCCTCTGCTGTGAGGGAAACCCCAGCCACATCTTC
AGCACACAAGAACTTCAAAATGCCTAAGCCACAGCAGCCAGGCGTTCCTTCAGGACTTTCT
CCCCCAGGATCTTGCTTCAAGTGCTGGAAATCTGGCCACTGGGCCAAGGAATGCCCGTAGC
CCAGGATTCC-TCCTAAACTGTGTCCCATCTGTGCGGGACCCCACTGGAAATTGGACTGTCC
AACTGGCCCAAGGCTCTGACTGACTCCTTCCCAGATCTTCTCAGCTTA--GGCTGAAGACTG
ACGCTGCCCGATTGCCTTGGAAGCTTCCTGGACCATCCCAGACGCTTTGGGTAACTCTTACA
GTGGAGGGTAAGTCCGTCCCCTTCTTAATCAATATGGAGGCTACCCACTCCACATTACCTTC
TTTTCAAGGGCCTGTTTCCCTTGCCTCCATAACTGTTGTGGGCATTGACAGACAGGCTTCTA
AACCCCTTATAACTCCCCCACTCTGGTGCTAACTTGGACAATATTCTTTTATGCACTCTTTTG
TAGTTATCCCCACCTGCCCAGTTCCCTTATTAGGCCGAGATATTTTAACCAAATTGTCTGCTT
CCCTGACTATTCCTGGACTACAGCCACATCTCATTGCTACCCTGCTTCCCAACCCAAAGCCT
CCTTCGAGCCTTCCTCTCGTATTCCCCGAACTTAACCCACGTGTATGGGACACCTCTA---CTC
CCTCCCTGGCAACCAATCACCAGCCTATTACCATCCCATTAAAACCTAATCACCCTTACCCCA
TTCAATGCCGGTATCCCATCCCACAAGAGGCTTTAAAGGGATTGAAACCTGTTATCACTTGC

CTGCTACAGTATGGGCTTCTAAAACCTGTAAACTCCCCTTACAATTCCCCCATTTTACCTGTC
CAAAAACTGGACAAGTCTTACAGGTTACTTCAGGATCTGTGCCTTATCAACCAAATTGTTTT
GCCTATCCACCTTGTGGTGCGAAACCCATCTACTCTCCTATCCTCAATACCTCCCTCCACAAC
CCATTATTCTGTTCTGGATCTCAAACACGCTTTCTTTACTATTCCTTTGCATCCTTCATCCCAG
CCTCTTTTCGCTTTCACTTGGACTGACCCTGACACCCATCAGGCTCAGCAAATTACCCGGGC
TGTACTGCCGCAAGGCTTCACAGACAGCCCCCATTACTTCAGTCAAGCCCAAATTTCTTCCT
CATCTGTTACCTATCTTGGCATAATTC-TCATAAAAACACAT--GTGCTCTCCCTGCTGATCATG
TCTGGCTAATCTCCCAAACCCCAACCCCTTCTACAAAACAACAACTCCTTCCTTC-GTAGGC
ATGGTTAGCTACTTCCACCTTTGGATACCTAGTTTTACCATCTTGACTAAACCACGATGTAAA
CTCACAAAAGGAAACCTAGCTGACCCCACAGATCCTAAGTCCTTTTGCCACTCCTTTCCATT
CCTTAAAAACAGCCCTAGAAGCTGCTCCCACACTAGCTCTCCCTAACTCATCCCAATCC-TTT
TTCATTACACACAGTCAAAGTGCAGGGCTGTGAGGTCAAAATTCTTACACAAGGATCGGGA
CCATGCCCTGTGGCCTTTATATCCAAACAACTTGACCTTACTGTTTTAGCTTAG-CCTCATGTC
TGTGTGTGGCAGCTGCTGCTTCCTTAATACTTTCAGAGGCCCTCAAAATCACAAACTATGCT
CAAC---------TCACTCTCTAGAGTTCTTATAACTTCCAAAATCTATTTTCTTCCTCACACCTGAT
GCACATATTTCCTGGT---CCCCTCCACTACCTCTCAGTAAGCCAAACTCATTGCCTTAACTCA
AGCCCTCGCCCTTGCAAAAGGATTAAGTGTCAATATTTATACTGACTCTAAATATGCCTTCCA
TATCCTGCACCACCATGCTGTTATATGGGCAGAAAGAAATGTCCTAACTATGCAAGGGTCCT
CCATCATTAATGCGTCTTTAATAAAAACTCTTCTCAAAGCTGCTTTACTTCCAAAGGAAGCT
GGAATCATTCACTGCAAGGGCCATCAAAAGGCAT-AGATCCCATTGCTCAGGACAATGCTTA
TGCTGATACGGTAGCTAAA-AAGCAGCTAGCATTCCAACTTCTATCCCTCATGGCAGTTTTTC
TCCTTCTCATTTGGCCACTCCCACCTACTCCCCCACTGAAACTTCCACCTATCAATCTCTTCC
CACGCAAGGCAAATGGTTCTTGGACCAAGGAAAATATCTCCTTCCAGCCTCACAGGCCCAT
TCTATTCTGTCATCATTTCATAACCTCTTCCATGTAGGTTACAAGCTGCTAGCCCATCTCTTAG
AACCTCTCATTTCCTTTCCATCGTGGAGATCTATCCTCAAGGAAATCACTTCTCAG-TGTTCC
ATCTGCTATTCTACTACTCCTCAGGGATTATTCAGGCCCCCTCCCTTCCCTACACATCAAGCT
TGGGGATTTGCCCCTGCCCAGGACTGGCAAATTGACTTTACTCACATGCCCTGAGTCAGGA
AACTAAAATACCTCTTGGTCTAGGTAGACACTTTCACTGGATGGGTAGAGGCCTTTCCCACA
GGGTCTGCAAAGGCCACTGTGGTCATTTCTTCCCTTCTGTCAGGTATAATTCCTCAGTTTGA
CCTTCCTACCTCTATACAGTCCAATAATGGACCAGCCTTTGTTAGTTAAATCACCCAACCAGT
TTCTCAGGCTCTTAG---TATTCAGTGGAACTTTCATACCCCTTACCATCCTCAATCTTCAGGA
AAGGTA-----GAACGGGCTAATGGTCTTTTAAAAACACACCTCACCAATCTCAGCCACCAAC
TTAAAAAGGACTAGACAATACTTTTACCACTTTCCCTTCTCAAAATTTGGGCCTGTCCTCGG
GATGCTACAGGGTACAGCCCATTTGAGCATCTGTATGAACCCTCCTTTTTATTAGGCCCCAGT
CTTATTCCAGACACCAGCCCAACTTGGACTGTGCCCCAAAAACTTGTCATCCCTACTCTCTT
CTGTCTAGTCATACTCCTATTCACCATTCTCAAATACTCATAAATGCCCTGCTCTTGTTTACAC
TGCCGGTTTACACTGTTTCTCCAAACCATCACAGCTGATATCTCCTGGTGCTAT-CCCAAACT
GCCACTCTTAACTCCCTCTTAAAGTAAATAAATAATCTTTGCTGGCAGGGCTATGCTGAACC
TCCTTGGGCACTCTCTAATTGGATGTCCTGGATCCTCCCAATTCTTAGTCCTTTAATACCTGT
TTTTCTCCTTCTCTTATTCAGACCTTTT-GTCTTCCATTTAGTTTTTCAATTCATACAAAACTGC
TTCCAGGCCATCACCAATCATTCTATATGATAAATGCTCCTTCTAACAACCCCACAATATCAC
CCCTTACCACAAAATCTTCCTTCAGCTTAATCTCTCCCACTCTGGGTTTCCATGCCACCCCTA
ATCCTGCATGAAGCAGCCCTGAGAAACATCACCCATTATCTCTCCATGCCACCCTCCAAAAA
ATTTTTGC-TGCCCCAACACTTCAATACTATTTTGTATTATTTTTCTTATCAATATAAGAAGGCA

GG------------------------------------
>Chlorocebus_sabaeus-HERVHF_INT(-)
---GCTAGTGAAATTGGTGCCATGACTCAGATCGG
GGGACCTCTCTTGGGAGATCACTCCCCTGTCCTCCTGCTCTTTGCTCCGTGAGAAAGATCCA
CTTACAACCTCAGGTCCTCAGACCAACCCGCCCCAAGAACATCTCACCAATTTTAAATCGG
GTAAGCAGCCTCTCTTTACTCTCTTCTCCAACTTCTCTCACTATCCCTCAACCTCTTTCTCCT
TTCAATCTTGGTGCCACACTTCAATCTTTCCCTTCTCTTAATTTCAGTTCCTTTCCTTTTCTGG
TAGAGACAGAGGAGACGCGTTTTATTCGTGAACTCAAAACTCCTGCACCGGTCACCGACTC
GGGAAGACAGTCTTTCTTTGGTATTTAATCCCTGCAGGGACGTCTGCCTGATTATTCACCCA
CATTTCACTGGTGTCTGATCACCGCAGGGACGCCTGCCTTGCTCATTCACCCACAATCCCTT
GGTGGCAAGTCCATTGCAGGGACGTCTGCTTTGGCTGCTCACCCACATTGCAGCCTAGGGC
TGCTCACCTCAC---CCCTTCTCTCCGTGTCT
CTACCCTCTCTTCTCTCCA-------------------------------------CTTTCTTGGGGGGCTAGCACCCCC
CACCC----------CTTCTCTCTGTGTCTCTACCC---TCTCT
TTTCTCTGGACTTGCCTCCTTCACTATGGGCAAACTTC--CACCCTCCATTTCTCCTTCTTCTC
CCTTAGCCTGTGTTCTCAAGAACTTAAAACCTCTTCAACTCACACCTGACCTAAAACCTTAA
TGCCTTATTTTCTTCTGCAATGCTGCTTAACCCC--AATACAAACTCGAC-AATGGTTCCAAAT
AGCCAGAAAACGGCACTTTCGATTTCTCCATCCTACAAGAGCTAGATAATTCTCGTCATAAA
ATGGGCAAATAGTCTGAGGTGCCTGATGTCCAGGCATTCTTTTACACATGGGTTCCTCCCTG
GTCTCTGTTCCCAATGCAACTCGTCCC---AAATCTTCCTTC
TTTCCCTCCCGCCTGTCCCCTCAGTCCCAACCCCAAGAGTTGCTGAGTCTTTCTAA--TCTTC
CTTTTTTACGGACCCATCTGACCTCTCCCCTCCTCCCCAGGCTGCTCCTCACCAGGCCAAGC
CAGGTCCCAA-TTCTTCCTCAGCCTCCGTTCCCCCACCCTATAATCCTTTTATCACCTCCCCTC
CTCACAC--------------------------AACTAGCCCTCCCCCACCTGCCCAGCAATTATCTCTTAAAA
AGGTGG-------CTGGAGCTGAAGGCATAGTCAGGGTTAATGCTCCTTTTTCTTTATCTGGCCTC
TCCCAAATCAGTTAGCGTTTAGGCTCTTTTTCATCAAATATAAAAACCCGGCCCAGTTCATG
GCTTGTTTGGTAGCAACCCTGAGATGCTTTACTGCCCTAGACCCTGAAAGGTCAAAAGGCT
GTCTTATTCTCAATATATAT---TGTATCACCCAATCCACTCCTGACATTAAATAAAACACCAAA
AATTAAGTTCCGGCCCTCAAACCCCACAACAAGACTTAA----CCTTGCCTTGAAGGTGTACA
ATAATAGAGAAGAGTTGCAATTACTTGCCTCTGCTGTGAGGGAAACCCCAGCCACATCTTC
AGCACACAAGAACTTCAAAATGCCTAAGCCACAGCAGCCAGGCGTTCCTTCAGGACTTTCT
CCCCCAGGATCTTGCTTCAAGTGCTGGAAATCTGGCCACTGGGCCAAGGAATGCCCGTAGC
CCAGGATTCC-TCCTAAACTGTGTCCCATCTGTGCGGGACCCCACTGGAAATTGGACTGTCC
AACTGGCCCAAGGCTCTGACTGACTCCTTCCCAGATCTTCTCAGCTTA--GGCTGAAGACTG
ACGCTGCCCGATTGCCTTGGAAGCTTCCTGGACCATCCCAGACGCTTTGGGTAACTCTTACA
GTGGAGGGTAAGTCCGTCCCCTTCTTAATCAATATGGAGGCTACCCACTCCACATTACCTTC
TTTTCAAGGGCCTGTTTCCCTTGCCTCCATAACTGTTGTGGGCATTGACAGCCAGGCTTCTA
AACCCCTTATAACTCCCCCACTCTGGTGCTAACTTGGACAATATTCTTTTATGCACTCTTTTG
TAGTTATCCCCACCTGCCCAGTTCCCTTATTAGGCCGAGATATTTTAACCAAATTGTCTGCTT
CCCTGACTATTCCTGGACTACAGCCACATCTCATTGCTACCCTGCTTCCCAACCCAAAGCCT
CCTTCGAGCCTTCCTCTCGTATTCCCCGAACTTAACCCACGTGTATGGGACACCTCTA---CTC
CCTCCCTGGCAACCAATCACCAGCCTATTACCATCCCATTAAAACCTAATCACCCTTACCCCA
TTCAATGCCGGTATCCCATCCCACAAGAGGCTTTAAAGGGATTGAAACCTGTTATCACTTGC
CTGCTACAGTATGGGCTTCTAAAACCTGTAAACTCCCCTTACAATTCCCCCATTTTACCTGTC

CAAAAACTGGACAAGTCTTACAGGTTACTTCAGGATCTGTGCCTTATCAACCAAATTGTTTT
GCCTATCCACCTTGTGGTGCGAAACCCATCTACTCTCCTATCCTCAATACCTCCCTCCACAAC
CCATTATTCTGTTCTGGATCTCAAACACGCTTTCTTTACTATTCCTTTGCATCCTTCATCCCAG
CCTCTTTTCGCTTTCACTTGGACTGACCCTGACACCCATCAGGCTCAGCAAATTACCCGGGC
TGTACTGCCGCAAGGCTTCACAGACAGCCCCCATTACTTCAGTCAAGCCCAAATTTCTTCCT
CATCTGTTACCTATCTTGGCATAATTC-TCATAAAAACACAT--GTGCTCTCCCTGCTGATCATG
TCTGGCTAATCTCCCAAACCCCAACCCCTTCTACAAAACAACAACTCCTTCCTTC-GTAGGC
ATGGTTAGCTACTTCCACCTTTGGATACCTAGTTTTACCATCTTGACTAAACCACGATGTAAA
CTCACAAAAGGAAACCTAGCTGACCCCACAGATCCTAAGTCCTTTTGCCACTCCTTTCCATT
CCTTAAAAACAGCCCTAGAAGCTGCTCCCACACTAGCTCTCCCTAACTCATCCCAATCC-TTT
TTCATTACACACAGTCAAAGTGCAGGGCTGTGAGGTCAAAATTCTTACACAAGGATCGGGA
CCATGCCCTGTGGCCTTTATATCCAAACAACTTGACCTTACTGTTTTAGCTTAG-CCTCATGTC
TGTGTGTGGCAGCTGCTGCTTCCTTAATACTTTCAGAGGCCCTCAAAATCACAAACTATGCT
CAAC---------TCACTCTCTAGAGTTCTTATAACTTCCAAAATCTATTTCTTCCTCACACCTGAT
GCACATATTTCCTGGT---CCCCTCCACTACCTCTCAGCAAGCCAAACTCATTGCCTTAACTCA
AGCCCTCGCCCTTGCAAAAGGATTAAGTGTCAATATTTATACTGACTCTAAATATGCCTTCCA
TATCCTGCACCACCATGCTGTTATATGGGCAGAAAGAAATGTCCTAACTATGCAAGGGTCCT
CCATCATTAATGCCTCTTTAATAAAAACTCTTCTCAAAGCTGCTTTACTTCCAAAGGAAGCT
GGAATCATTCACTGCAAGGGCCATCAAAAGGCAT-AGATCCCATTGCTCAGGACAAGGCTTA
TGCTGATACGGTAGCTAAA-AAGCAGCTAGCATTCCAACTTCTATCCCTCACGGCAGTTTTTC
TCCTTCTCATTTGGCCACTCCCACCTACTCCCCCACTGAAACTTCCACCTATCAATCTCTTCC
CACGCAAGGCAAATGGTTCTTGGACCAAGGAAAATATCTCCTTCCAGCCTCACAGGCCCAT
TCTATTCTGTCATCATTTCATAACCTCTTCCATGTAGGTTACAAGCTGCTAGCCCATCTCTTAG
AACCTCTCATTTCCTTTCCATCGTGGAGATCTATCCTCAAGGAAATCACTTCTCAG-TGTTCC
ATCTGCTATTCTACTACTCCTCAGGGATTGTTCAGGCCCCCTCCCTTCCCTACACATCAAGCT
TGGGGATTTGCCCCTGCCCAGGACTGGCAAATTGACTTTACTCACATGCCCCGAGTCAGGA
AACTAAAATACCTCTTGGTCTAGGTAGACACTTTCACTGGATGGGTAGAGGCCTTTCCCACA
GGGTCTGCAAAGGCCACTGTGGTCATTTCTTCCCTTCTGTCAGGTATAATTCCTCGGTTTGA
CCTTCCTACCTCTATACAGTCCAATAATGGACCAGCCTTTGTTAGTTAAATCACCCAACCAGT
TTCTCAGGCTCTTAG---TATTCAGTGGAACTTTCATATCCCTTACCATCCTCAATCTTCAGGAA
AGGTA-----GAACGGGCTAATGGTCTTTTAAAAACACACCTCACCAATCTCAGCCACCAACTT
AAAAAGGACTAGACAATACTTTTACCACTTTCCCTTCTCAAAATTTGGACCTGTCCTCGGGA
TGCTACAGGGTACAGCCCATTTGAGCATCTGTATGAACCCTCCTTTTTATTAGGCCCCAGTCT
TATTCCAGACACCAGCCCAACTTGGACTGTGCCCCAAAAACTTGTCATCCCTACTCTCTTCT
GTCTAGTCATACTCCTATTCACCATTCTCAAATACTCATAAATGCCCTGCTCTTGTTTACACTG
CCGGTTTACACTGTTTCTCCAAACCATCACAGCTGATATCTCCTGGTGCTAT-CCCAAACTGC
CACTCTTAACTCCCTCTTAAAGTAAATAAATAATCTTTGCTGGCAGGGCTATGCTGAACCTCC
TTGGGCACTCTCTAATTGGATGTCCTGGATCCTCCCAATTCTTAGTCCTTTAATACCTGTTTTT
CTCCTTCTCTTATTCAGACCTTTTGGTCTTCCATTTAGTTTTTCAATTCATACAAAACTGCTTC
CAGGCCATCACCAATCATTCTATATGATAAATGCTCCTTCTAACAACCCCACAATATCACCCC
TTACCACAAAATCTTCCTTCAGCTTAATCTCTCCCACTCTGGGTTTCCATGCCACCCCTAATC
CTGCATGAAGCAACCCTGAGAAACATCACCCATTATCTCTCCATGCCACCCTCCAAAAAATT
TTTGC-TGCCCCAACACTTCAATACTATTTGTATTATTTTTCTTATCAATATAAGAAGGCAG---
----

>Colobus_guereza-HERVHF_INT(-)
--GAAATTTGGTGCCATGACTCAGATTGGGGT
ACCTCCCTTGGGAGATCAATCCCCTGTCCTCCTGCTCTTTGCTGCATGAGAAAGATCCACTT
ACGACCTCAGGTCCTCAGACCAACCAGCCCCAAGAACATCTCACCAATTTTAAATCGGGTA
AGCAGCCTCTCTTTACTCTCTTCTCCAACCTCTCTCACTATCCCTCAACCTCTTTCTCCTTTC
AATCTTGGTGCCACACTTCAATCTCTCCCTTCTCTTAATTTCAGTTCCTTTCCTTTTCTGGTA
GAGACAGAGGAGACACGTTTTATCCGTGAATCCAAAACTCCGGCACCAGTCACCTACTCGG
GAAGACAGTCTTCCCTTGGTGTTTAATCCCTGCAGGGACGCCTGCCTGATTATTCACCCACA
-TTCATTGGTGTCTGATCACTGCAGGGACGCCTGCCTTGGTCATTCATCCACATTCCCTTGGT
GGCAAGTCAATTGCAGGGATGCCTGCTTTGGCTGCTCACCCACATTGCAGCCTAGGGCTGC
TCACCCCAC---CCCTTCTCTCCTTGTCTCTA
CCCTCTCTTCTCTCCA-------------------------------------CTTTCTTGGGGGGCAAGCA-CCCCCAC
CC----------CCTCTCTCTGTGTCTCTACCC--TCTCTTTT
CTCTGGACTTGCCTCCTTCACTATGGGCAACCTTC--CACCCTCCATGCCTCCTTCTTCTCCCT
TAGCCTGTGTTCTCAAGAACTTAAAACCTCTTCAAGTCACACCTGACCTAAAACCTAAATG
CCTTATTTTCTTCTGCAACACCACTTGGCCCC--AACACAAACTTGAC-AATGGTTCCAAATA
GCCAGAAAACGGCACTTTCGATTTCTCCATCCTACAAGATCTAGATAATTCTTGTCATAAAAT
GGGCAAATAGTCTGAGGTGCCTGATGTCCAGGCATTCTTTTACACATGGGTCCCTCCCTGGT
CTCTGTTCCCAATGCAACTCGTCCC---AATCTTCCTTCTTT
CCCTCCCACCTGTCCCCTCAGTCCCAACCCCAAGAGTTGCTGAGTCTTTCTAA--TCTTCCTT
TTTTACAGACCCATCTGACCTCTCCCCTCCTCCCCAGGCTGCTCCTCGCCAGGCCAAGCGA
GGTCCCAA-TTCTTCCTCAGCCTCCACTCCCCCACCCTATAATCCTTTTGTCACCTCCCCTCC
TCACACCTGGTCCGACTTACAGTTTCATTCTGTGACTAGCCCTCCCCCACCTGCCCAGCAAT
TACCTCTTAAAAAGGTGG-------CTGGAGCTGAAGACATAGTCAAGGTTAATGCTCCTTTTTC
TTTATCTGGCTTCTCCCAAATCAGATAGCGTTTAGGCTCTTTTTCATCAAATATAAAAACCCA
GCCCAGTTCATGGCTTGTTTGGCAACAACCCTGAGACGCTTTACTGCCCTAGACCCTGAAA
GGCCAAAAGGCTGTCTTATTCTCAATATACAT---TGTATTACCCAATCCACTCCTGACATTAAA
TAAAACACCAAAAATTAAGTTCCAGCCCTCAAACTCCACAACAGGACTTAATTAACCTTGC
CTTCAAGGTGTACAATAATAGAGAAGAGTTGCAATC-CTTGCCTCCTCTGTGAAAGAAACCC
CAGCCACATCTCCAGCACACAAGAACTTCAAAATGCCTAAGCCACAGTGGCCAGGCGTTC
CTTCAGGACTTTCTCCCCCAGGATCTTGCTTCAAGTGCTGGAAA-CTGGCCACTGGGCCAA
GGAATGCCCGTAGCCCAGGATTCC-CCCTAAGACGTATCCCATCTGTACGGGACCCCACTGG
AAATTGGACTGTCCAACTGACCCAAGGCTCTGACTGACTCCTTCCCAGATCTTTTCGGCTTA
GCGGCCGAAGACTGATGCTACCCAATCACCTTGGAAGCTTCCTGGACCATCCCAGATGCTT
TGGGTAACTCTTACAGTTGAGGGTAAGTCTGTCCCCTTCTTAATCAATACAGAGGCTACCCA
CTCCACATTATCTTCTTTTCAAGGGCCTGTTTCCCTTGCCTCCATAACTGTTGTAGGTATTGAT
GGCCAGGCTTCTAAACCCCTTAAAACTCCCGCACTCTGGTGCTAACTTGGACAACATTCTTT
TATGCACTCTTCTTTAGTTA-CCCCACCTGCCCAGTTCCCTTATTAGGCCGAGATATTTAAACC
AAATTGTCTGCTTCCCTGACTATTCCTGGACTACAGCCACATCTCATTGCTACCCTGCTTCCC
AACCCATAGCCTCCTTCGTGCCTTCCTCTCGTATCCCCCAAACTTAACCCACAAGTATGGGA
TAACTCTA---CTCCCTCCCTGGCAACTGATCACCAGCCTATTACTATCCCATT-AAACCTAATC
ACCCTTACCCCGCTCAATGCCAGTATCCCATCCCACAACAGCCTTTAAAGGGATTGAAGCCT
GTTATCACTCGCCTGCTACAGTATGGGCTTCTAAAACCTGTAAACTCCCCTTACAATTCCCCC
ATTTTACCTGTCCAAAAACCGGACAAGTCTTACAGGTTACTTCAGGATCTGTGCCTTATCAA

CCAAATTGTTTGCCTATCCACCTTGTGGTGTGAAACCCATTTACTCTCCTATCCTCGATACC
TCCCTCCACAACCCATTATTCTGTTCTGGATTGTAAACATGCTTTGTTTACTATTCCTTTGCAT
CCTTCATCCCAGCCTCTTTTCAATTCCACTTGGACTGACCCTGACACCCATCAGGCTCAGGA
AATTACCTGGGCTGTACTGCCTCAAGACTTCGTGGACAGCCCCTATTACTTCAGTCAAGCCC
AAATTTCTTCCTCATCTGTTACCTATCTTGGCATAATTC-TCATAAAAACACAC--GTGCTCTCC
CTGCTGATCATGTCTGGCTAATCTCCCAAACCCCAACCCCTTCTACAAAACAACAACTCCTT
CCTTC-ATAGGCATGGTTAGGTACTTCCACCTTTGGATACCTAGTTTTACCATCTTGACTAAAC
CATGATGTAAACTCACAAAAGGAAACCTAGCTGACCCCACAGATCCTAAATCCTTTGACCA
CTCCTTTCCATTCCTTAAAAACAGCCCTAGAAGCTGCTCCCACACTAGCTCTCCCTAACTCA
TCCCAATCC-TTTTTCATTACACACAGTCAAAGTGCAGGGCTGTGAGGTCAAAATTCTTACA
CAAGGATCGGGACCATGCCCTGTGGCCTTTTTATCCAAACAACTTGACCTTACTGTTTTAGC
TTAG-CCTCGTGTCTGTGTGTGGCAGCTGCCACTTCCTTAATACTTTTAGAGGCCCTCAAAAT
CACAAACTATGCTCAAC---------TCACTCTCTAGAGTTCTCATAACTTCCAAAATCTATTTTCT
TCCTCACACCTGATGCACATATTTTCTGCT---CCCCTCCACTACCTCTCAACAAGCCAAACTC
ATTGCCTTAACTCAAGCCCTCGCTCTTGCAAAAGGATTAAGCATCAGTATTTATACTGACTTT
AAATATGCCTTCCATATCCTGCACCACCATGCTGTTATATGGGTAGAAAGAAATTTCCTCACT
ATGCAAGGGTCCTCCATCATTAATGCCTCTTTAATAAAAACTCTCCTCAAAGCTGCTTTACTT
CCAAAGGAAGCTGGAGTCATTCACTTCAAGGGCCATCAAAAGGCATCAGATCCCATTGCTC
AGGACAATGCTTATGCTGATATGTTAGCTAAA-AAGCAGCTAGCATTCCAACTTCTGTCCCCC
ACAGCAGTTTTTCTCCTTCTCATCTGGCCACTCCCACCTACTCCCCCGCTGAAACTTCCACC
TATCAATTTCTTCCCACACAAGGCAAATGGTTCTTGGACCAAGGAAAATATCTCCTTCCAGC
CTCACGGGCCCATTCTATTCTGTCGTCATTTCATAACCTCTTCCATGTAGGTTACAAGCTGCT
TGCCCATCTCTTAGAACCTCTCATTTCCTTTCCATCGTGGAAATCTATCCTCAAGGAAATCAC
TTCTCAG-TGTTCCATCTGCTATTCTACTAATCCTCAGGGATTGTTCAGGCCCCCTCCCTTCCC
TACACATCAAGCTAGGGGATTTGCCCCTGCCCAGGACTGGCAAATTGACTTTACTCACATGC
CCCGAGTCAGGAAACTAAAATACCTCTTGGTCTGGGTAGACACTTTCACTGGATGGGTAGA
GGCCTTTCCCACAGGGTCTGCAAAGGCCACCGTGGTCATTTCTTCCCTTCTGTCAGGCATAA
TTCCTTGGTTTGACCTTCCTACCTCTATACAGTCCAATAATAGACCAGCCTTTGTTAGTTAAA
TCACCCAACCAATTTCTCAGGCTCTTAG---TATTCAGTGGAATTTTCATACCCCTTACCATCCT
CAATCTTCAGGAAAGGTA-----GAACGGGCTAATGGTCTTTTAAAATCACACCTCACCAAGCT
CAGCCACCGACTTAAAAAAGACTAGACAATACTTTTACCACTTTCCCTTCTCAAAATTCGGG
CCTGTCCTCGGGATGCTACAGGGTACAGCCCATTTGAGCTTCTGTATGAATGCTCATTTTTAT
TAGGCCCCAGTCTTATTCCAGACACGAGCCCAACTTGGACTGTGCCCCAAAAACTTGTCAT
CCCTACTCTCTTCTGTCTAGTCATACTCCTATTCACCGTTCTCAAATACTCATAAATGCCCTGC
TCTTGTTTACACTGCTGGTTTGCACTGTTTCTCCAAGCCATCACAGCTGATGTCTCCTGGTG
CTAT-CCCAAACTGCCACTCTTAACTCCCTCTTAAAGTAAATAAATACTCTTTGCTGGCAGGG
CTATGCTGAACCTCCTTGGGCACTCTCTAATTGGATGTCCTGGATCCTCCCAATTCTCAGTGC
TTTAATACCTGTTTTTCTCCTTCTCTTATTCGGACCTTTT-GTCTTCCGTTTAGTTTTTCAATTC
ATACAAAACTGCTTCCAGGCCATCACCAATCATTCTATATGATAAATGCTCCTTCTAACAACC
CCACAATAGCACCCCTTACCCCAAAATCTTTCTTCAGCTTAATCTCTCCCACTCTGGGTTTCC
ATGCCACCCCTAATCCCGCTTGAAGCAGCCCTGAGAAATATCACCCATTATCTCTCCATACCA
CCCCCCAAAAAATTTTTGC-TGCCCTAACACTTCAACACTATTTTATATTATTTTTCTTATTAAT
ATAAGAAGGCAG--------------------------------------
>Erythrocebus_patas-HERVHF_INT(-)

--GCTAGTGAAATTGGTGCCATGACTCAGATCGG
GGGACCTCTCTTGGGAGATCACTCCCCTGTCCTCCTGCTCTTTGCTCCATGAGAAAGATGCA
CTTACAACCTCAGGTCCTCAGACCAACCCGCCCCAAGAACATCTCACCAATTTTAAATCGG
GTAAGCAGCCTCTCTTTACTCTCTTCTCCAACTTCTCTCACTATCCCTCAACCTCTTTCTCCT
TTCAATCTTGGTGCCACACTTCAATCTTTCCCTTCTCTTAATTTCAGTTCCTTTCATTTTCTGG
TAGAGACAGAGGAGACGCGTTTTATCCGTGAACTCAAAACTCCTGCACCGGTCACCGACTC
GGGAAGACAGTCTTTCTTTGGTGTTTAATCCCTGCAGGGACGTCTGCCTGATTATTCACCCA
CATTTCATTGGTGTCTGATCACCGCAGGGACGCCTGCCTTGCTCATTCACCCACAATCCCTT
GGTGGCAAGTCCATTGCAGGGACGCCTGCTTTGGCTGCTCACCCACATTGCAGCCTAGGGC
TGCTCACCTCAC---CCCTTCTCTCCATGTCT
CTACCCTCTCTTCTCTCCA------------------------------------CTTTCTTGGGGGGCTAGCACCCCC
CACCC----------CTTCTCTCTGTGTCTCTACCC--TCTCT
TTTCTCTGGACTTGCCTCCTTCACTATGGGCAAACTTC--CACCCTCCATTTCTCCTTCTTCTC
CCTTAGCCTGTGTTCTCAAGAACTTAAAACCTCTTCAACTCACACCTGACCTAAAACCTTAA
TGCCTTATTTTCTTCTGCAATGCCGCTTAACCCC--AATACAAACTCGAC-AATGGTTCCAAAT
AGCCAGAAAACGGCACTTTCGATTTCTCCATCCTACAAGAGCTAGATAATTCTCGTCATAAA
ATGGGCAAATAGTCTGAGGTGCCTGATGTCCAGGCATTCTTTTACACATCGGTACCTCCCTG
GTCTCTGTTCCCAATGCAACTCATCCC---AAATCTTCCTTC
TTTCCCTCCCGCCTGTCCCCTCAGTCCCAACCCCAAGAGTTGCTGAGTCTTTCTAA--TCTTC
CTTTTTTACGGACCCATCTGACCTCTGCTCTCCTCCCCAGGCTGCTCCTCACCAGGCCAAGC
CAGGTCCCAA-TTCTTCCTCAGCCTCCGTTCCCCCACCCTATAATCCTTTTATCACCTCCCCTC
CTCACAC--------------------------AACTAGCCCTCCCCCACCTGCCCAGCAATTATCTCTTAAAA
AGGTGG-------CTGGAGCTGAAGGCATAGTCAGGGTTAATGCTCCTTTTTCTTTATCTGGCCTC
TCCCAAATCAGTTAGCGTTTAGGCTCTTTTTCATCAAATATAAAAACCCGGCCCAGTTCATG
GCTTGTTTGGTAGCAACCCTGAGATGCTTTACTGCCCTAGACCCTGAAAGGTCAAAAGGCT
GTCTTATTCTCAATATACAT---TGTATCACCCAATCCACTCCTGACATTAAATAAAACACCAAA
AATTAAGTTCCGGCCCTCAAACCCCACAACAAGACTTAA----CCTTGCCTTGAAGGTGCACA
ATAATAGAGAAGAGTTGCAATCACTTGCCTCTGCTGTGAGGGAAGCCCCAACCACATCTTC
AGCACACAAGAACTTCAAAATGCCTAAGCCACAGCAGCCAGGCGTTCCTTCAGGACTTTCT
CCCCCAGGATCTTGCTTCAAGTGCTGGAAATCTGGCCACTGGGCCAAGGAATGCCCGCAGC
CCAGGATTCC-TCCTAAATTGTGTCCCATCTGTGTGGGACCCCACTGGAAATTGGACTGTCCT
ACTGGCCCAAGGCTCTGACTGACTCCTTCCCAGATCTTCTCAGCTTA--GGCTGAAGACTGA
TGCTGCCCGATTGCCTTGGAAGCTTCCTGGACCATCCCAGACGCTTTGGGTAACTCTTACAG
TGGAGGGTAAGTCCGTCCCCTTCTTAATCAATATGGAGGCTATCCACTCCACATGACCTTCTT
TTCAAGGGCCTGTTTCCCTTGCCTCCATAACTGTTGTGGGCATTGACAGCCAGGCTTCTAAA
CCCCTTATAACTCCCCCACTCTGGTGCTAACTTGGACAATATTCTTTTATGCACTCTTTTTAG
TTATCCCCACCTGCCCAGTTCCCTTATTAGGCCGAGATATTTTAACCAAATTGTCTGCTTCCC
TGACTATTCCTGGACTACAGCCACATCTCATTGCTACCCTGCTTCCCAACCCAAAGCCTCCT
TCGAGCCTTCCTCTCGTATTCCCCAAACTTAACCCACGTGTATGGGACACCTCTA---CTCCCT
CCCTGGCAACCAATCACCAGCCTATTACCATCCCATTAAAACCTAATCACCCTTACCCCATTC
AATGCCAGTATCCCATCCCACAACAGGCTTTAAAGGGATTGAAACCTGTTATCACTTGCCTG
CTACAGTATGGGCTTCTAAAACCTGTAAACTCCCCTTACAATTCCCCCATTTTACCTGTCCAA
AAACTGGACAAGTCTTACAGGTTACTTCAGGATCTGTGCCTTATCAACCAAATTGTTTTGCC
TATCCACCTTGTGGTGCGAAACCCATCTACTCTCCTATCCTCAATACCTCCCTCCACAACCCA

TTATTCTGTTCTGGATCTCAAACACGCTTTCTTTACTATTCCTTTGCATCCTTCATCCCAGCCT
CTTTTCGCTTTCACTTGGACTGACCCTGACACCCATCAGGCTCAGCAAATTACCCGGGCTGT
ACTGCCGCAAGGCTTCACAGACAGCCCCCATTACTTCAGTCAAGCCCAAATTTCTTCCTCAT
CTGTTACCTATCTTGGCATAATTC-TCGTAAAAACACAC--GTGCTCTCCCTGCTGATCATGTC
TGGCTAATCTCCCAAACCCCAACCCCTTCTACAAAACAACAACTCCTTCCTTC-GTAGGCAT
GGTTAGCTACTTCCACCTTTGGATACCTAGTTTTACCATCTTGACTAAACCACGATGTAAACT
CACAAAAGGAAACCTAGCTGACCCCACAGATCCTAAATCCTTTTGCCACTCCTTTCCATTCC
TTAAAAACAGCCCTAGAAGCTGCTCCCACACTAGCTCTCCCTAACTCATCCCAATCC-TTTTT
CATTACACACAGTCAAAGTGCAGGGTTGTGAGGTCAAAATTCTTACACAAGGTTCGGGACC
ATGCCCTGTGGCCTTTATATCCAAACAACTTGACCTTACTGTTTTAGCTTAG-GCTCATGTCT
GTGTGTGGCAGCTGTTGCTTCCTTAATACTTTCAGAGGCCCTCAAAATCACAAACTATGCTC
AAC---------TCACCCTCTAGAGTTCTCATAACTTCCAAAATCTATTTTCTTCCTCACACCTGAT
GCACATATTTCCTGGC---CCCCTCCACTACCTCTCAACAAGCCAAACTCATTGCCTTAACTCA
AGCCCTCGCCCTTGCAAAAGCATTAAGTGTCAATATTTATACTGACTCTAAATATGCCTTCCA
TATCCTGCACCACCATGCTGTTATATGGGCAGAAAGAAATGTCCTAACTATGCAAGGGTCCT
CTATCATTAATGCCTTTTTAATAAAAACTCTTCTCAAAGCTGCTTTACTTCCAAAGGAAGCTG
GAATCATTCACTGCAAGGGCCATCAAAAGGCAT-AGATCCCATTGCTCAGGACAATGCTTAT
GCTGATACTGTAGCTAAA-AAGCAGCTAGCATTCCAACTTCTATCCCACACGGCAGTTTTTCT
CCTTCCCATTTGGCCACTCCCACCTACTCCCCCACTGAAACTTCCACCTATCAATCTCTTCCC
ACGCAAGGCAAATGGTTCTTGGACCAAGGAACATATCTCCTTCCAGCCTCACAGGCCCATT
CTATTCTGTCGTCATTTCATAACCTCTTGCATGTAGGTTACAAGCTGCTAGCCCATCTCTTAG
AACCTCTCATTTCCTTTCCATCGTGGAAATCTATCCTCAAGGAAATCACTTCTCAGTTGTTCC
CTCTGCTATTCTACTACTCCTCAGGGATTGTTCAGGCCCCCTCCCTTCCCTACACATCAAGCT
TGGGAATTTGCCCCGGCCCAGGACTGGCAAACTGACTTTACTCACATGCCCTGAGTCAGGA
AACTAAAATCCCTCTTGGTCTGGGTAGACACTTTCATTGGATGGGTAGAGGCCTTTCCCACA
GGGTCTGAGAAGGCCACCGTGGTCATTTCTTCCCTTCTGTCAGACATAATTCCTCGGTTTGA
CCTTCCTACCTCTATACAGTCCAATAATGGACCAGCCTTTGTTAGTTAAATCACCCAACCAGT
TTCTCAGGCTCTTAG---TATTCAGTGGAACTTTCATACCCCTTACCGTCCTCAATCTTCAGGA
AAGGTA-----GAACGGGCTAATGGTCTTTTAAAAACACACCTCACCAATCTCAGCCACCAAC
TTAAAAAGGACTAGACAATACTTTTACCACTTTCCCTTCTCAAAATTTGGGCCTGTCCTCGG
GATGCTACAGGGTACAGCCCATTTGAGCATCTGTATGAACTCTCCTTTTTATTAGGCCCCAGT
CTTATTCCAGACACCAGCCCAACTTGGACTGTGCCCCAAAAACTTGTCATCCCTACTCTCTT
CTGTCTAGTCATACTCCTATTTGCCATTCTCAAATACTCATAAATGCCCTGCTCTTGTTTACAC
TGCCGGTTTACACTGTTTCTCCAAACCATCACAGCTGATATCTCCTGGTGCTAT-CCCAAACT
GCCACTCTTAACTCCCTCTTAAAGTAAATAAATAATCTTTGCTGGCAGGGCTATGCTGAACC
TCCTTGGGCAC--TCTAATTGGATGTCCTGGATCCTCCCAATTCTTAGTCCTTTAATACCTGTT
TTTCTCCTTCTCTTATTCAGACCTTTT-GTCTTCCGTTTAGTTTTTCAATTCATACAAAACTGC
TTCCAGACCATCACCAATCATTCTATATGATAAATGCTCCTTCTAACAACCCCACAATATCAC
CCCTTACCACAAAATCTTCCTTCAGCTTAATCTCTCCCACTCTGGGTTTCCATGCCACCCCTA
ATCCTGCTCGAAGCAGCCCTGAGAAACATCACCCATTATCTCTCCATGCCACCCTCCAAAAA
ATTTTTGC-TGCCCCAACACTTCAATACTATTTTGTATTACTTTTCTTATCAATATAAGAAGGC
AG-------------------------------------
>Homo_sapiens-HERVHF_INT(-)
---ATTTGGTGCCGTGACTCGGATTGGGGGA

CCACCCTTGGGAGATCAATCCCCTGTCCTCCTGCTCTTTGCTCCATGAGAAAGATCCACCTA
TGACCTCGGATCCTCAGACCAACCAGCCCAAGGAACATCTCACCAATTTTAAATCCGGTAA
GTGACCTCTCTTTACTCTCTTCTCTAACTTCTCTCACTATCCCTCAACCTCTTTCTCCTTTCAA
TCTTGGTGCCACACTTCAATCTCTCCCTTCTGTTAATTTCAGTTCCTTTCCTTTCCTGGTAGA
GACAAAGGAGATGCATTTTATCCGTGGACGCAAAACTCCAGCACCGGTCACAGACTCGGG
AAGACAGTCTTCCCTTGGTGTTTAATCACTGCAGGGACGCCTGCCTGATTATTCACCCACAT
TTCATTGGTATCTGATCACCGCAGGGACGCCTGCCTTGGTCATTCACTCACATTCCCTTGGT
GGCAAGTCAATTGCAGGGACGCCTGCTTTGGCTGCTCACCCACATTGCAGCCCAGGGCTGC
TCACCCCAC---CCATTCTCTCCATGCCTCTA
CTCTCTCTTTTCTCTCCACTTTCCTGGGGGGCAAGCACCTCCCACCCCTTTTCCACTTTCCT
GGGGGACAAGCACCTCCCACCC----------CTTCTCTCTGTGTCTCTACCC----------------------------
-------------------------TCTCTTTTCTCTGAACTCGCCTCCTTCACTATAGGCAACCTTC--CACCCT
CCATTCCTCCTTCTTCTCCCTTAGCCTGTGTTCTCAAGAACTTAAAACCTCTTCGACTCACA
CCTGACCTAAAACCTAAATGCCTTATTTTCTTCTGCAACACCGCTTGACCCC--AATACAAAA
TCGAC-AATGGTTCCAAATAGCCGGAAAACAGCACTTTCTATCTCTCCATCCTATAAGATCTG
GATAATTCTTGGTGTAAAATGGGCAAATGGTCTGAGGTGCCTGATGTCCAGGCATTGTTTTA
CACATTGGTCCCTCCCTGGTCTCTGTTCCCAATGCAACTTGTCCC-----------------------------------
------------AAATCTTCCTTCTTTCCCTCCCACCTGTCCCCTCAGTCCCGACCCCAAGTGTTGCT
GAGTCTTTCCAA--TCTTCCTTTTTTATGGACCCATCTGACCTCTCCCCTCCTCCCCAGGCTGC
TCCTCGCCAGGCCAAGCCAGGTCCCAA-TTCTTCCTCGGCCTCCGCTCCCCAACCCTATAAT
CCTTTTATCACCTCCCCTCCTCACACCCAGTCCCGCTTATAGTTTTGTTCTGCCACTAGCCCT
CCCCTACCTGCCCAGCAATTTCCTCTTAAAAAGGTGG-------CTGGAGCTGAAGGCATAGTCA
AGGTGAATGCTCCTTTTTCTTTATCCGGCCTCTCCCAAATCAGTTAGCGCTTAGGCTCTTTTT-
ATCAAATATAAATACCCAGCCCAGTTCATGGCTTGTTTGGCAGCAATCCTGAGACGCTTTAC
AGCCCTAGACCCTAAAAGGTCAAAAGCCCATCTTATCCTCAAAATACAT---TTTATTACCCAA
TCTGCTCCCGACATTAAATAAAACTCCAAAAATTAAATTCCAGCCCTCAAACCCCACAACAT
GACTTAATTAACCTTGCCTTCAGGGTGTACAATAATAGAGAAGAGTTGCAATTACTTGCCTC
TGCTGTGAGAGAAACCCCAGCCACATCTCCAGCACACAAGGACTTCAAAACGCCTAAGCC
ACAGCGGCCAGGCATTCCTTCAGGACTTCCTCCCCCAGGATCTTGCTTCAAGTGCTGGAAA
TCTGGCCACCGGGCCAAGGAATGCCCGCAGCCCAGGATTCC-TCCTAAGCCGTGTCCCATCT
GTGTGGGACCCCAGTGGAAACTGGACTGTCCAACTGGCCTAAGGCTCTGACTAGCTCCTTC
CCAGATCTTCTTGGCTTAGCGGCTGAAGACTGACGCTGCCCAATCACCTTAGAAGCTTCCT
GGACCATCACAGAAGCTCTAGGTAACTCTTACAGTGGAGGGTAAGTCTGTCCCCTTCTTAAT
CAATACAGAGGCTACGTACTCCACATTACCTTCTTTTCAAGGGCCCGTTTCCCTTGCCTCCAT
AACTGTTGTGGGTATTGGCAGCCAGGCTTCTAAACCCCTTAAGACTCCCCCACTCTGGTGCT
AACTTGGACAACATTCTTTTATGCATTCTTTTTTAGTTATCCCTACCTGCCCAGTTCTCTTATT
AGGCTGAGACATTTTAACTGAATTATCTGCTTCCCTGACTATTCCTGGACTACAGCCACATCT
CATTGCCACCCTTCTTCCCAACCCAAAGCCTCCTTCATGCCTTCTTCTCATATCCCCCCACCT
TAACCCACAAGTATGGGACACCTCTA---CTCCCTCCCTGGCAACTGATCACCAGCCCATT---A
TCCCATTAAAACCTAATCAACCTTACCCTGCTCAACACTAGTATCCCATCCCACAACAGGCT
TTAAAGGGACTGAAGCCTGTGATGACTCACCTGCTACAGCATGGGCTTCTAAAACCTATAA
ACTCTCCTTACAATTCCCCCATTTTACCTGTCCAAAAACCAGACAAGCCTTACAGGTTAGTT
CAGGATCCGCACCTTATCAACCAAATTGTTTTGCCTATCCCCCCCATGGTGCCAAACCCATAT
ACTCTCCTATCCTCAATACCTCCCTCCACAACCCGTTATTCTGTTCTGGATCTCAAACATGCT

TTCTTTACTATTCCTTTGCACCCTTCATCCCAGTCTTTCTTCGCTTTCACTTGGACTGACCCT
GACACCCATCAGGCTCAGCAAATTACCTGGGCTGTACTCCCGCAAGGCTTCACAGACAGCC
CCCATTACTTCAGTCAAGCCCAAATTTCTTCCTCATGTGTTACCTATCTCGACATAGTTC-TCA
TAAAAACACAC--GTGCTCTCCCTGCTGATCGTGTCCAGCTAATCTCCCAAACCCTGACCCCT
TCTACAAAATGACAACTCCTTTCCTTCCTAGGCATGGTTAGGTACTTCCACCTTTGGATACCT
CGTTTTTCCATCCTGACTAAACCATTATATAAACTCACAAAAGCAAACCTGGCTGACCCCAC
AGATCCCAAATCCTTTCGCCACTCCTTTCCATTCCTTAAAAACAGCCCTAGAAGCTGCTCCC
ACACTAGCTCTCCCTAACTCATCCCAACCC-TTTTTCATTA-ACACAGCTGAAGTGCAGGGCT
GTGCAGTCAAAATTCTTACACAAGGACTGGGACCATGCCCTGTGGCCTCTTTATCCAAACA
ACTTGACTTTACTGTTTTAGCCTAGTCCTCATGTCTGTGTGCGGTGGCTGCCGCTGCCTTAAT
ACTTTTAGAGGTCCTAAAAATCACAAACTATGCTCAACTCTCTACAGTCACTCTCTACAGTT
CTCATAACTTCCAAAATCTATTTCTTCCTCACACCTGACACATATACTTTCTGCT---CCCCTC
CACTACCTCTCAGCAAGCCAAACTCATTTCCTTAACTCAAGCCCT-ACTCTTGCAAAAGGAC
TACTTGTCAATATTTATACTGACTCTAACTATGCCTTCCATATCCTGCCCCACCATGCTGTTATA
TGGGCAGAAAGAAATTTCTTCACTACACAAGGGTCCTCCATCATTAATGCCTCTTTAATAAA
AACTCTTCTCAAAGCCGCTTTACTTCCAAAGGAAGCTGGAGTCATTCACTGCAAGGGCCAT
CAAAAGGCATCAGATCCCATTGCTCAGGGCAACGCTTATGCTGATAAGGTAGCTAAAGAAG
CAGCTAGCGTTCCAACTTCTCTCCCTCATGGCAGTTTTTCTCCTTCTCATC-GGTCACTCCCA
CTTACTCCCCTACTGAAACTTCCACCTATCAATCTCTTCCCACACAAGGCAAATGGTTCTTG
GACCAAGGAACATATCTCCTTCCAGCCTCACAGGCCCATTCTATTCTGTCGTCATTTCATAAC
CTCTTCCATGTAGGTTATAAGCTGCTAGTCTGCCTCTTAGAACCTCTCATTTCCTTTCCATCG
TGGAAATCTATCCTCAAGGAAATCACTTCTCAG-TGTTCCATCTGCTGTTCTACTACTCCTCA
GGGATTGTTCAGGGCCCCTCCCTTCCCTACACATCAAGCTCGGGGATTTGCCCTTGCCCAGG
ACTGGCAAATTGACTTTACTCACATGCCCTGAGTCAGGAAACTAAAATACGTCTCGGTCTAG
ATAGACACTTTCACTGGATGGGTAGAGGCCTTTCCCACAGGGTCTGAGAAGGCCACCGCAG
TCATTTCTTCCCTTCTGTCAGACATAATTCCTCAGTTTGGCCTTCCTACCTCTATACCGTCCAA
TAACGGACCGGCCTTTATTAGTCAAATCACCCAAGCAGTTTCTCAGGCTCTTGG---TATTCAG
TGGAACCTTCATACCCCTTACCATCCTCAATCTTCAGGAAAGGTAGAATGGAACAGACTAAT
GGTCTTTTAAAAACACACCTCCCCGAGCTCAGCCACCAACTTAAAAAGGACTAGACAATAC
TTTTACCACTTTCCCTTCTCAAAATTCGGGCGTGTCCTTGGAATGCTACAGGGTACAGCCCA
TTTGAGCTCCTATATAGACACTCCTTTTTATTAGGCCCCAGTCTTATTCCAGACAGCAGCCCA
ACTTGGACTGTGCCCCAAAAACTTGTTATCTGTACTATCTTCTGCCTAGTCATACTCTTATTC
ACTGTTCTCAACTACTCATAAATGCCCTGCTCTTGTTTACACTGCCGGTTTACACTGTTTCTC
CAGGCCATCACAGCTGATATCTCCTGGTGCTATCCCCAAACCACCACTCTTAACTCCCTCTT
AAAGTAAATCAATAATCTTTGCTGGCAGGGCTATGCTGAACCTCCTTGGGCACTCTCTAATT
GGATGTCCTGTGTCCTCCCAATTCGTAGTCCTTTAATACCAGTTTTTCTCCTTCTCTTATTCGG
ACCTTGT-GTCTTCCGCTTAGTTTTTCAATTCATACAAAACAACATCCAGGCCATCACCAATT
ATTCTATATGACAAATGCTCCTTCTAACAACCCTGCAATATCACCCCTTACCACGAAATCTTC
CTTCAGCTGAATCTCTCCCACTCTGGGTTCCCACGCCACCCCTAATCCCGCTTGAAGCAGCC
CTGAGAAACATCGCCCATTATCTCTCCATGCCACCCCCCC-AAAATTTTCAC-TGCCCCAACA
CTTCAATACTATTTTATGTTATTTTTCTTATTAATATAAGAAGGCAG----------------------------------
---
>Lophocebus_aterrimus-HERVHF_INT(-)
---GCTAGTGAAATTTGGTGCCATGACTCAGACTGG

GGGACCTCCCTTGGGAGATCAATCCCCTGTCCTCCTGCTCTTTGCTCCATGAGAAAGATCCA
CTTACGACCTCAGGTCCTCAGACCAACCAGCCCCAAGAACATCTCACCAATTTTAAACCGG
GTAAGCAGCCTCTCTTTACTCTCTTCTCCAACTTCTCTCACTATCCCTCAACCTCTTTCTCCT
TTCAATCTTGGTGCCACACTTCAATCTCTCCCTTCTCTTAATTTCAGTTCCTTTCCTTTTCTGG
TAGAGGCAGAGGAGACGCGTTTTATCCGTGAACCCAAAACTCCTGCACCGGTCACCGACTC
AGGAAGACAGTCTTCCCTTGGTGTTTAATCCCTGCAGGGACGTCTGCCTGATTATTCACCCA
CATTTCATTGGTGTCTGATCACCACAGGGATGCCTGCCTTCGTCATTCACCCACAATCCCTT
GGTGGCAAGTCCATTGCAGGGACACCTGCTTTGGCTGCTCACCCACATTGCAGCCCAGGGC
TGCTCACTCCAC---CCCTTCTCTCCGTGTCT
CTACCCTCTCTTCTCTCCA--CTTTCTTGGGGGGCTAGCACCCCC
CACCC----------CTTCTCTCTGTGTCTCTACCC---TCTCT
TTTCTCTGGACTTGCCTCCTTCATTATGGGCAAACTTCCACACCCTCCATTCCTCCTTCTTCT
CCCTTAGCCTGTGTTCTCAAGAACTTAAAACCTCTTCAACTCACAACTGACCTAAAACCTA
AATGCCTTATTTCTTCTGCAACACGGCTTGGCCCC--AATACAAACTCTAC-AATGGTTCCAA
ATAGCCAGAAAACGGCACTTTCGATTTCTCCATCCTACAAGATCTAGATAATTCTCGTCATAA
AATGGGCAAATAGTCTGAGGTGCCTGATGTCCAGGCATTCT--------TGAGTCCCTCCCTGGTC
TCTGTTCCCAATGCAACTCGTCCC--AAATCTTCCTTCTTT
CCCTCCCGCCTGTCCCCTCAGTCCCAACCCCAAGAGTTGCTGAGTCTTTCTAA--TCTTCCTT
TTTTACGGACCCATCTGACCTCTCCCCTCCTCCCCAGGCTGCTCCTCACCAGGCCAAGCCA
GGTCCCAA-TTCTTCCTCAGCCTCCGTTCCCCCACCCTATAATCCTTTTATCACCTCCCCTCCT
CACACCCAGTCCGACTTGCAGTTTCGTTTTGCAACTAGCCCTCCCTCACCTGCCCAGCAATT
ATCTCTTAAAAATGTGGCTGGAGCCTGGAGCCGAAGGCATAGTCAGGGTTAATGCTCCTTTT
TCTTTATCTGGCCTCTCCCAAATCAGTTAGCGTTTAGGCTCTTTTTCATCAAATATAAAAACC
CGGCCCAGTTCATGACTTGTTTGGCAGCAACCCTGAGATGCTTTACTGCCCTAGACCCTGA
AAGGTCAAAAGGCCGTCTTATTCTCAATATACAT---TGTATTACCCAATCCACTCCTGACATTA
AATAAAACACCAAAAATTAAGTTCCGGCCCTCAAACCCCACAACAAGACTTAATTAACCTT
GCCTTCAAGGTGTACAATAATAGAGAAGAGTTGCAATTACTTGCCTCCGCTGTGAGAGAAA
CCCCAGCCACATCTTCAGCACACAAGAACTTCAAAATGCCTAAGCCACAGCAGCCAGGCG
TTCCTTCAGGACTTTCTCCCCCAGGATCTTGCTTCAAGTGCTGGAAATCTGGCCACTGGGCC
AAGGAATGCCCGTAGCCCAGGATTCC-TCCTAAACTGTGTCCCATCTGTGCAGGACCCCACT
GGAAATTGGACTGTCTAACTGGCCCAAGGCTCTAACTGACTCCTTCCCAGATCTTCTCAGCT
TA--GGCTGAAGACTGATGCTGCCCGATCACCTTGGAAGCTTCCTGGACCATCCTAGACGCT
TTGGGTAACTCTTACAGTGGAGGGTAAGTCCATCCCCTTCTTAATCAATATGGAGACTACCC
ACTCCACATTACCTTCTTTTCAAGGGCCTGTTTCCCTTGCCTCCATAAATGTTGTGGGCATTG
ACAGCCAGGTTTCTAAACCCCTTAAAACTCCCCCAGTCTGGTGCTAACTTGGACAACATTC
TTTTATGCACTCTTTTTCAGTTATCCCCACCTGCCCAGTTCCCTTATTAGGCCAAGATATTTTA
ACCAAATTGTCTGCTTCCCTGACTATTCCTGGACTACAGCCACATCTCATTGCTACCCTGCTT
CCCAACCCAAAGCCTCCTTCGCGCCTTCCTCTCGTATTCCCCAAACTTAACCCACGTGTATG
GGACACCTCTA---CTCCCTCCCTGGCAACCAATCACCAGCCTATTACTATCCCATTAAAACCT
AATCACCCTTACCCCATTCAATGCCAGTATCCCATCCCACAACAGGCTTTAAAGGGATTGAA
ACCTGTTATCACTTGCCTGCTACAGTATGGGCTTCTAAAACCTGTAAACTCCCCTTACAATTC
CCCCATTTTACCTGTCCAAAAACTGGACAAGTCTTACAGGTTACTTCAGGATCTGTGCCTTA
TCAACCAAATTGTTTTGCCTAT---CCTTGTGGTGCGACACCCATTTACTCTCCTATCCTCAATA
CCTCCCTCCACAACCCATTATTCTGTTCTGGATCTCAAACATGCTTTCTTTACTATTCCTTTGC

ATCCTTCATCCCAGCCTCTTTTCGCTTTCACTTGGACTGACCCTGACACCCATCAGGCTCAG
GAAATTACCTGGGCTGTACTGCTGCAAGGCTTCACGGACAGCCCCTATTACTTCAGTCAAG
CCCAAATTTCTTCCTCATCTGTTACCTATCTTGGCATAATTC-TCATAAAAACACAC--ATGCTC
TCCCTGCTGATCATGTCTGGCTAATCTCCCAAACCCCAACCCCTTCTACAAAACAACAACTC
CTTCCTTC-GTAGGCATGGTTAGCTACTTCCACCTTTGGATACCTAGTTTTACCATCTTGACTA
AACCACAATGTAAACTCACAAAAGGAAACCTAGCTGACCCCACAGATCCTAAATCCTTTTG
CCACTCCTTTCCATTCCTTAAAAACAGCCCTAGAAGCTGCTCCCACACTAGCTCTCCCTAAC
TCATCCCAATCC-TTTTTCATTACACACAGTCAAAGTGCAGGGCTGTGAGGTCAAAATTCTT
ACACAAGGATTGGGACCATGCCCTGTGGCCTTTTTATCCAAACAACTTGACCTTACTCTTTT
AGCTTAG-CCTCATGTCTGTGTGTGGCAGCTGCTGCTTCCTTAATACTTTCAGAGGCCCTCAA
AATCACAAACTATGCTCAAC---------TCACTCTCTAGAGTTCTCATAACTTTCAAAATCTATTTT
ATTCCTCACACGTGATGCACATATTTCCTGCC---CCCCTCCACTACCTCTCAACAAGCCAAAC
TCATTGCCTTAACTCAAGCCCTCGCTCTTGCAAAAGGATTACGTGTCAATATTTATACTGACT
CTAAATATGCCTTCCATATCCTGCACCACCATGCTGTTATATGGGCAGAAAGAAATGTCCTAA
CTATGCAAGGGTCCTCCATCATTAATGCCTCTTTAATAAAAACTCTTCTCAAAGCTGCTTTAC
TTCCAAAGGAAGCTGGAATCATTCACTGCAAGGGCCATCAAAAGGCATCAGATCCCATTGC
TCAGGACAATGCTTATGCTGATACGGTAGCTAAA-AAGCAGCTAGCATTCCAACTTCTATCCC
TCATGGCAGTTTTTCTCCTTCGCATTTGGCCACTCCCACCTACTCCCCCACTGAAACTTCCA
CCTATCAGTCTCTTCCCACGCAAGGCAAATGGTTCTTGGACCAAGGAAAATATCTCCTTCCA
GCCTCACAGGCCCATTCTATTCTGTCATCATTTCATAACCTCTTCCATGTAGGTTACAAGCTG
CTAGCCCATCTCTTAGAACCTCTCATTTCCTTTCCATCGTGGAGATCTATCCTCAAGGAAATC
ACTTCTCAG-TGTTCCATCTGCTATTCTACTACTCCTCAGGGATTGTTCAGGCCCCCTCCCTT
CCCTACACGTCAAGCTCGGTGATTTGCCCCTGCCCAGGACTGGCAAATTGACTTTACTCACA
TGCCCCGAGTCAGGAAACTAAAATACCTCTTGGTCTAGGTAGACACTTTCACTGGACAGGT
AGAGGCCTTTCCCACAGGGTCTGCAAAGGCCACCATGGTCATTTCTTCCCTTCTGTCAGGTA
TAATTCCTCGGTTTGACCTTCCTACCTCTATACAGTCCAATAATGGACCAGCCTTTGTTAGTT
AAATCACCCAACCAGTTTCTCAGGCTCTTAG---TATTCAGTGGAACTTTCATACCCCTTACTA
TCCTCAGTCTTCAGGAAAGGTA-----GAACGGGCTAATGGTCTTTTAAAAACACACCTCACC
AATCTCAGCCACCAACTTAAAAAGGACTAGACAATACTTTTACCACTTTCCCTTCTCAAAAT
TCGGGCCTGTCCTCGGGATGCTACAGGGTACAGCCCATTTGAGCTTCTGTATGAACCCTCCT
ATTTATTAGGCCCCAGTCTTATTCCAGACACCAGCCCAACTTGGACTGTGCCCCAAAAACTT
GTCATCCCTACTCTCTTCTGTCTAGTCATACTCCTATTCACTGTTCTCAAATACTCATAAATGC
CCTGCTCTTGTTTACACTGCCGGTTTACACTGTTTCTCCAAGCCATCACAGCTGATATCTCCC
GGTGCTAT-CCCAAACTGCCACTCTTAACTCCCTCTTAAAGTAAATAAATAATCTTTGCTGGC
AGGGCTATGCTGAATCTCCTTGGGCACTCTCTAATTGGATGTCCTGGATCCTCCCAATTCTTA
GTCCTTTAATACCTGTTTTTCTCCTTCTCTTATTCAGACCTTTT-GTCTTCCGTTTAGTTTTTCA
ATTCATACAAAACTGCTTCCAGGCCATCACCAATCATTCTATATGATAAATGCTCCTTCTAAC
AACCCCACAATAT-----CTTACCACAAAATCTTCCTTCAGCTTAATCTCTCCCACTCTGGGTTT
CCATGCCACCCCTAATCCTGCATGAAGCAGCCCTGAGAAACATCGCCCATTATCTCTCCATG
CCACCCCCCAAAAAATTTTTGC-TGCCCCAACACTTCAATACTATTTTGTATTATTTTTCTTATT
AATATAAGAAGGCAG-------------------------------------
>Macaca_mulatta-HERVHF_INT(-)
---GCTAGTGAAATTTGGTGCCGTGACTCAGACTGG
GGGACCTCCCTTGGGAGATCAATCTCCTGTCCTCCTGCTCTTTGCTCCGTGAGAAAGATCCA

CTTACGACCTCAGGTCCTCAGACCAACCAGCCCCAAGAACATCTCAGCAATTTTAAATCGG
GTAAGCAGCCTCTCTTTACTCTCTTCTCCAACTTCTCTCACTATCCCTCAACCTCTTTCTCCT
TTCAATCTTGGTGCCACACTT-AATCTCTCCCTTCTCTTAATTTCAGTTCCTTTCCTTTTCTGG
TAGAGACAGAGGAGACGCGTTTTATCCGTGAACCCAAAACTCCTGCACCGGTCACCGACTT
GGGAAGACAGTCTTCCCTTGGTGTTTAATCCCTGCAGGGACGTCTGCCTGATTATTCACCCA
CATTTCATTGGTGTCTGATCACCACAGGGATGCCTGCCTTCGTCATTCACCCACAATCCCTT
GGTGGCAAGTCCATTGCAGGGACACCTGCTTTGGCTGCTCACCCACATTGCAGCCCAGGGC
TGCTCACCCCAC--CCCTTCTCTCCGTGTCT
CTACCCTCTCTTCTCTCCA--------------------------------------CTTTCTTGGGGG-CTAGCACCCCC
CACCC----------CTTCTCTCTGTGTCTCTACCC--TCTCT
TTTCTCTGGACTTGCCTCCTTCATTATGGGCAAACTTCCACACCCTCCATTCCTCCTTCTTCT
CCCTTAGCCTGTGTTCTCAAGAACTTAAAACCTCTTCAACTCACAACTGACCTAAAACCTA
AATGCCTTATTTTCTTCTGCAACACGGCTTGGCCCC--AATACAAACTCGAC-AATGGTTCCA
AATAGCCAGAAAACAGCACTTTCGATTTCTCCATCCTACAAGATCTAGATAATTCTCGTCATA
AAATGGGCAAATAGTCTGAGGTGCCTGATGTCCAGGCATTCTTTTACACATGAGTCCCTCCC
TAGTCTCTGTTCCCAATGCAACTCGTCCC---AAATCTTCCT
TCTTTCCCTCCCGCCTGTCCCCTCAGTCCCAACCCCAAGAGGTGCTGAGTCTTTCTAA--TCT
TCCTTTTTTACGGACCCATCTGACCTCTCCCCTCTTCCCCAGGCTGCTCCTCGCCAGGCCAA
ACCAGGTCCCAA-TTCTTCCTCAGCCTCCGTTCCCCCACCCTATAATCCTTTTATCACCTCCC
CTCCTCACACCCAGTCCGACTTGCAGTTTCGTTTTGCAACTAGCCCTGCCCCACCTGCCCA
GCAATTATCTCTTAAAAATGTGG-------CTGGAGCTGAAGGCATAGTCAGGGTTAATGCTCCTT
TTTCTTTATCTGGCCTCTCCCAAATCAGTTAGCGTTTAGGCTCTTTTTCATCAAATATAAAAA
CCCAGCCCAGTTCATGGCTTGTTTGGCAGCAACCCTGAGATGCTTTACTGCCCTAGACCCTG
GAAGGTCAAAAGGCCGTCTTATTCTCAATATACAT---TGTATTACCCAATCCACTCCTGACATT
AAATAAAACACCAAAAATTAAGTTCCGGCCCTCAAACCTCACAACAAGACTTAATTAACCT
TGCCTTCAAGGTTTACAATAATAGAGAAGAGTTGCAATTACTTGCCTCCACTGTGAGAGAA
ACCCCAGCCACATCTTCAGCACACAACAACTTCAAAATGCCTAAGCCACAGCAGCCAGGT
GTTCCTTCAGGACTTTCTCCCCCAGGATCTTGCTTCAAGTGCTGGAAATCTGGCCACTGCGC
CAAGGAATGCCCGTAGCCCAGGATTCC-TCCTAAACTGTGTCCCATCTGTGCGTGACCCCAC
TGGAAACTGGACTGTCCAACTGGCCCAAGGCTGTGACTGACTCCTTCCCAGATCTTCTCAG
CTTA--GGCTGAAGACTGATGCTGCCCGATCGCCTTGGAAGCTTCCTGGACCATCCCAGACG
CTTTGGGTAACTCTTACAGTGGATGGTAAGTCCATCCCCTTCTTAATCAATATGGAGGCTACC
CACTCCACATTACCTTCTTTTCAAGGGCCTGTTTCCCTTGCCTCCAAAAATGTTGTGGGCAT
TGACAGCCAGGTTTCTAAACCCCTTAAAACTCCCCCAGTCTGGTGCTAACTTGGACAACAT
TCTTTTATGCACTCTTTTTCAGTTATCCCCACCTGCCCAGTTCCCTTATTAGGCCAAGATATTT
TAACCAAATTGTCTGCTTCCCTGACTATTCCTGGACTACAGCCACATCTCATTGCTACCCTGC
TTCCCAACCCAAAGCCTCCTTCACGCCTTCCTCTCGTATTCCCCGAACTTAACCCACGTGTA
TGGGACACCTCTACTCCTCCCTCCCTGGCAACCAATCACCAGCCTATTACTATCCCATTAAA
ACCTAATCACCCTTACCCCATTCAATGCCAGTATCCCATCCCACAACAGGCTTTAAAGGGAT
TGAAACCTGTTATCACTCGCCTGCTACAGTATGGGCTTCTAAAACCTGTAAACTCCCCTTAC
AATTCCCCCATTTTACCTGTCCAAAAACTGGACAAGTCTTACAGGGCACTTCAGGATCTGTG
CCTTATCAACCAAATTGTTTTGCCTAT---CCTTGTGGTGCGACACCCATTTATTCTCCTATCCT
CAATACCTCCCTCCACAACCCATTATTCTGTTCTGGATCTCAAACATGCTTTTTTTACTATTCC
TTTGCATCCTTCATCCCAGCCTCTTTTCGCTTTCACTTGGACTGACCCTGACACCCATCAGG

CTCAGGAAATTACCTGGGCTGTACTGCTGCAAGGCTTCACGGACAGCCCCTATTACTTCAGT
CAAGCCCAAATTTCTTCCTCATCTGTTACCTATCTTGGCATAATTC-TCATAAAAACACAC--G
TGCTCTCCCTGCTGATCATGTCTGGCTAATCTCCCAAACCCCAACCCCTTCTACAAAACAAC
AACTCCTTCCTTC-GTAGGCATGGTTAGCTACTTCCACCTTTGGATACCTAGTTTTACCATCTT
GACTAAACCACGATGTAAACTCACAAAAGGAAACCTAGCTGACCCCACAGATCCTAAATCC
TTTTGCCACTCCTTTCCATTCCTTAAAAACAGCCCTAGAAGCTGCTCCCACACTAGCTCTCC
CTAACTCATCCCAATCC-TTTTTCATTACACACAGTCAAAGTGCAGGGCTGTGAGGTCAAAA
TTCTTACACAAGGATCGGGACCATGCCCTGTGGCCTTTTTATCCAAACAACTTGACCTTACT
CTTTTAGCTTAG-CCTCATGTCTGTGTGTGGCAGCTGCTGCTTCCTTAATACTTTCAGAGGCC
CTCAAAATCACAAACTATGCTCAAC---------TCACTCTCTAGAGTTCTCATAACTTCCAAAATC
TATTTTCTTCCTCACACCTGATGCACATATTTCCTGCC---CCCCTCCACTACCTCTCAACAAG
CCAAACTCATTGCCTTAACTCAAGCCCTTGCTCTTGCAAAAGGATTAAGTGTCAATATTTATA
CTGACTCTAAATATGCCTTCCATATCCTGCACCACCATGCTGTTATATGGGCAGAAAGAAATG
TCCTAACTATGCAAGGGTCCTCCATCATTAATGCCTCTTTAATAAAAACTCTTCTCAAAGCTG
CTTTACTTCCAAAGGAAGCTGGAATCATTCACTGCAAGGGCCATCAAAAGGCATCAGATCC
CATTGCTCAGGACAATGCTTATGCTGATACAGTAGCTAAA-AAGCAGCTAGCATTCCAACTT
CTATCCCTCACGGCAGTTTTTCTCCTTTGCATTTGGCCACTCCCACCTACTCCCCCACTGAA
ACTTCCACCTATCAGTCTCTTCCAACGCAAGGCAAATGGTTCTTGGACCAAGGAAAATCTC
TCCTTCCAGCCTCACAGGCCCATTCTATTCTGTCATCATTTCATAACCTCTTCCATGTAGGTTA
CAAGCTGCTAGCCCATCTCTTAGAACCTCTCATTTCCTTTCCATCGTGGAGATCTATCCTCAA
GGAAATCACTTCTCAG-TGTTCCATCTGCTATTCTACTACTCCTCAGGGATTGTTCAGGCCCC
CTCCCTTCCCTACACATCAAGCTCGGTGATTTGCCCCTGCCCAGGACTGGCAAATTGACTTT
ACTCACATGCCCCGAGTCAGGAAACTAAAATACCTCTTGGTCTAGGTAGACACTTTCACTG
GATGGGTAGAGGCCTTTCCCACAGGGTCTGCGAAGGCCACCATGGTCATTTCTTCCCTTCTG
TCAGGTATAATTCCTCGGTTTGACCTTCCTACCTCTATACAGTCCAATAATGGACCAGCCTTT
GTTAGTTAAATCACCCAACCAGTTTCTCAGGCTCTTAG---TATTCAGTGGAACTTTCATACCC
CTTACCATCCTCAGTCTTCAGGAAAGGTA-----GAACGGGCTAATGGTCTTTTAAAAACACAC
CTCACCAATCTCAGCCACCAACTTAAAAAGGACTAGACAATACTTTTACCACTTTCCCTTCT
CAAAATTCGGGCCTGTCCTCGGGATGCTACAGGGTACAGCCCATTTGAGCTTCTGTATGAAC
CCTCCTATTTATTAGGCCCCAGTCTTATTCCAGACACCAGCCCAACTTGGACTGTGCCCCAA
AAACTTGTCATCCCTACTCTCTTCTGTCTAGTCATACTCCTATTCACCATTCTCAAATACTCAT
AAATGCCCTGCTCTTGTTTACACTGCCGGTTTACACTGTTTCTCCAAGCCATCACAGCTGAT
ATCTCCTGGTGCTAT-CCCAAACTGCCACTCTTAACTCCCTCTTAAAGTAAATAAATAATCTTT
GCTGGCAGGGCTATGCTGAACCTCCTTGGGCACTCTCTAATTGGATGTCCTGGATCCTCCCA
ATTCTTAGTCCTTTAATACCTGTTTTTCTCCTTCTCTTATTCAGACCTTTT-GTCTTCCGTTTAG
TTTTTCAATTCATACAAAACTGCTTCCAGGCCATCACCAATCATTCTATATGATAAATGCTCCT
TCTAACAACCCCACAATATCACCCCTTACCACAAAATCTTCCTTCAGCTTAATCTCTCCCACT
CTGGGTTTCCATGCCACCCCTAATCCTGCATGAAGCAGCCCTGAGAAACATCACCCATTATC
TCTCCATGCCACCCCCCAAAAAATTTTTGC-TGCCCCAACACTTCAATACTATTTTGTATTATT
TTTCTCATTAATATAAGAAGGCAG------------------------------------
>Macaca_silenus-HERVHF_INT(+)
----------------------------CTAGTGAAATTTGGTGCCGTGACTCAGACTGG
GGGACCTCCCTTGGGAGATCAATCTCCTGTCCTCCTGCTCTTTGCTCCGTGAGAAAGATCCA
CTTACGACCTCAGGTCCTCAGACCAACCAGCCCCAAGAACATCTCACCAATTTTAAATCGG

GTAAGCAGCCTCTCTTTACTCTCTTCTCCAACTTCTCTCACTATCCCTCTACCTCTTTCTCCTT
TCAATCTTGGTGCCACACTT-AATCTCTCCCTTCTCTTAATTTCAGTTCCTTTCCTTTTCTGGT
AGAGACAGAGGAGACGCGTTTTATCCGTGAACCCAAAACTCCTGCACCGGTCATCGACTC
GGGAAGACAGTCTTCCCTTGGTGTTTAATCCCTGCAGGGACGTCTGCCTGATTATTCACCCA
CATTTCATTGGTGTCTGATCACCACAGGGATGCCTGCCTTCGTCATTCACCCACAATCCCTT
GGTGGCAAGTCCATTGCAGGGACACCTGCTTTGGCTGCTCACCCACATTGCAGCCCAGGGC
TGCTCACCCCAC---CCCTTCTCTCCGTGTCT
CTACCCTCTCTTCTCTCCA-----------------------------------CTTTCTTGGGGGGCTAGCACCCCC
CACCC----------CTTCTCTCTGTGTCTCTACCC---TCTCT
TTTCTCTGGACTTGCCTCCTTCATTATGGGCAAACTTCCACACCCTCCATTCCTCCTTCTTCT
CCCTTAGCCTGTGTTCTCAAGAACTTAAAACCTCTTCAACTCACAACTGACCTAAAACCTA
AATGCCTTATTTCTTCTGCAACACGGCTTGGCCCC--AATACAAACTCGAC-AATGGTTCCA
AATAGCCAGAAAACAGCACTTTCGATTTCTCCATCCTACAAGATCTAGATAATTCTCGTCATA
AAATGGGCAAATAGTCTGAGGTGCCTGATGTCCAGGCATTCTTTTACACATGAGTCCCTCCC
TAGTCTCTGTTCCCAATGCAACTCGTCCC---AAATCTTCCT
TCTTTCCCTCCCGCCTGTCCCCTCAGTCCCAACCCCAAGAGGTGCTGAGTCTTTCTAA--TCT
TCCTTTTTTACGGACCCATCTGACCTCTCCCCTCCTCCCCAGGCTGCTCCTCGCCAGGCCAA
GCCAGGTCCCAA-TTCTTCCTCAGCCTCCGTTCCCCCACCCTATAATCCTTTTATCACCTCCC
CTCCTCACACCCAGTCCGACTTGCAGCTTCGTTTTGCAACTAGCCCTCCCCCACCTGCCCA
GCAATTATCTCTTAAAAATGTGG-------CTGGAGCTGAAGGCATAGTCAGGGTTAATGCTCCTT
TTTCTTTATCTGGCCTCTCCCAAATCAGTTAGCGTTTAGGCTCTTTTTCATCAAATATAAAAA
CCCAGCCCAGTTCATGGCTTGTTTGGCAGCAACCCTGAGATGCTTTACTGCCCTAGACCCTG
GAAGGTCAAAAGGCCATCTTATTCTCAATATACAT---TGTATTACCCAATCCACTCCTGACATT
AAATAAAACACCAAAAATTAAGTTCCGGCCCTCAAACCTCACAACAAGACTTAATTAACCT
TGCCTTCAAGGTTTACAATAATAGAGAAGAGTTGCAATTACTTGCCTCCACTGTGAGAGAA
ACCCCAGCCACATCTTCAGCACACAACAACTTCAAAATGCCTAAGCCACAGCAGCCAGGT
GTTCCTTCAGGACTTTCTCCCCCAGGATCTTGCTTCAAGTGCTGGAAATCTGGCCACTGGGC
CAAGGAATGCCCGTAGCCCAGGATTCC-TCCTAAACTGTGTCCCATCTGTGCGGGACCCCAC
TGGAAACTGGACTGTCCAACTGGCCCAAGGCTGTGACTGACTCCTTCCCAGATCTTCTCAG
CTTA--GGCTGAAGACTGATGCTGCCCGATCGCCTTGGAAGCTTCCTGGACCATCCCAGACG
CTTTGGGTAACTCTTACAGTGGATGGTAAGTCCATCCCCTTCTTAATCAATATGGAGGCTACC
CACTCCACATTACCTTCTTTTCAAGGGCCTGTTTCCCTTGCCTCCATAAATGTTGTGGGCATT
GACAGCCAGGTTTCTAAACCCCTTAAAACTCCCCCAGTCTGGTGCTAACTTGGACAACATT
CTTTTATGCACTCTTTTTCAGTTATCCCCACCTGCCCAGTTCCCTTATTAGGCCGAGATATTTT
AACCAAATTGTCTGCTTCCCTGACTATTCCTGGACTACAGCCACATCTCATTGCTACCCTGCT
TCCCAACCCAAAGCCTCCTTCGCGCCTTCCTCTCGTATTCCCCGAACTTAACCCACGTGTAT
GGGACACCTCTA---CTCCCTCCCTGGCAACCAATCACCAGCCTATTACTATCCCATTAAAACC
TAATCACCCTTACCCCATTCAATGCCAGTATCCCATCCCACAACAGGCTTTAAAGGGATTGA
AACCTGTTATCACTCACCTGCTACAGTATGGGCTTCTAAAACCTGTAAACTCCCCTTACAATT
CCCCCATTTTACCTGTCCAAAAACTGGACAAGTCTTACAGGGTACTTCAGGATCTGTGCCTT
ATCAACCAAATTGTTTTGCCTAT---CCTTGTGGTGCGACACCCATTTACTCTCCTATCCTCAAT
ACCTCCCTCCACAACCCATTATTCTGTTCTGGATCTCAAACATGCTTTCTTTACTATTCCTTTG
CATCCTTCATCCCAGCCTCTTTTCGCTTTCACTTGGACTGACCCTGACACCCATCAGGCTCA
GGAAATTACCTGGGCTGTACTGCTGCAAGGCTTCATGGACAGCCCCTATTACTTCAGTCAAG

CCCAAATTTCTTCCTCATCTGTTACCTATCTTGGCATAATTC-TCATAAAAACACAC--GTGCTC
TCCCTGCTGATCATGTCTGGCTAATCTCCCAAACCCCAACCCCTTCTACAAAACAACAACTC
CTTCCTTC-GTAGGCATGGTTAGCTACTTCCACCTTTGGATACCTAGTTTACCATCTTGACTA
AACCACGATGTAAACTCACAAAAGGAAACCTAGCTGACCCCACAGATCCTAAATCCTTTTG
CCACTCCTTTCCATTCCTTAAAAACAGCCCTAGAAGCTGCTCCCACACTAGCTCTCCCTAAC
TCATCCCAATCC-TTTTTCATTACACACAGTCAAAGTGCAGGGCTGTGAGGTCAAAATTCTT
ACACAAGGATCGGGACCATGCCCTGTGGCCTTTTTATCCAAACAACTTGACCTTACTCTTTT
AGCTTAG-CCTCATGTCTGTGTGTGGCAGCTGCTGCTTCCTTGATACTTTCAGAGGCCCTCA
AAATCACAAACTATGCTCAAC---------TCACTCTCTAGAGTTCTCATAACTTCCAAAATCTATT
TTCTTCCTCACACCTGATGCACATATTTCCTGCC---CCCCTCCACTACCTCTCAACAAGCCAA
ACTCATTGCCTTAACTCAAGCCCTCGCTCTTGCAAAAGGATTAAGTGTCAATATTTATACTGA
CTCTAAATATGCCTTCCATATCCTGCACCACCATGCTGTTATATGGGCAGAAAGAAATGTCCT
AACTATGCAAGGGTCCTCCATCATTAATGCCTCTTTAATAAAAACTCTTCTCAAAGCTGCTTT
ACTTCCAAAGGAAGCTGGAATCATTCACTGCAAGGGCCATCAAAAGGCATCAGATCCCATT
GCTCAGAACAATGCTTATGCTGATACGGTAGCTAAA-AAGCAGCTAGCATTCCAACTTCTATC
CCTCACGGCAGTTTTTCTCCTTCGCATTTGGCCACTCCCACCTACTCCCCCACTGAAACTTC
CACCTATCAGTCTCTTCCCACGCAAGGCAAATGGTTCTTGGACCAAGGAAAATCTCTCCTTC
CAGCCTCACAGGCCCATTCTATTCTGTCATCATTTCATAACCTCTTCCATGTAGGTTACAAGC
TGCTAGCCCATCTCTTAGAACCTCTCATTTCCTTTCCATCGTGGAGATCTATCCTCAAGGAAA
TCACTTCTCAG-TGTTCCATCTGCTATTCTACTACTCCTCAGGGATTGTTCAGGCCCCCTCCC
TTCCCTACACATCAAGCTCGGTGATTTGCCCCTGCCCAGGACTGGCAAATTGACTTTACTCA
CATGCCCTGAGTCAGGAAACTAAAATACCTCTTGGTCTAGGTAGACACTTTCACTGGATGG
GTAGAGGCCTTTCCCACAGGGTCTGCGAAGGCCACCATGGTCATTTCTTCCCTTCTGTCAG
GTATAATTCCTCGGTTTGACCTTCCTACCTCTATACAGTCCAATAATGGACCAGCCTTTGTTA
GTTGAATCACCCAACCAGTTTCTCAGGCTCTTAG---TATTCAGTGGAACTTTCATACCCCTTA
CCATCCTCAGTCTTCAGGAAAGGTA-----GAATGGGCTAATGGTCTTTTAAAAACACACTTCA
CCAATCTCAGCCACCAACTTAAAAAGGACTAGACAATACTTTTACCACTTTCCCTTCTCAAA
ATTCGGGCCTGTCCTCGGGATGCTACAGGGTACAGTCCATTTGAGCTTCTGTATGAACCCTC
CTATTTATTAGGCCCCAGTCTTATTCCAGACACCAGCCCAACTTGGACTGTGCCCCAAAAAC
TTGTCATCCCTACTCTCTTCTGTCTAGTCATACTCCTATTCACCGTTCTCAAATACTCATAAAT
GCCCTGCTCTTGTTTACACTGCCGGTTTACACTGTTTCTCCAAGCCATCACAGCTGATATCTC
CTGATGCTAT-CCCAAACTGCCACTCTTAACTCCCTCTTAAAGTAAATAAATAATCTTTGCTG
GCAGGGCTATGCTGAACCTCCTTGGGCACTCTCTAATTGGATGTCCTGGATCCTCCCAATTC
TTAGTCCTTTAATACCTGTTTTTCTCCTTCTCTTATTCAGACCTTTT-GTCTTCCGTTTAGTTTT
TCAATTCATACGAAACTGCTTCCAGGCCATCACCAATCATTCTATATGATAAATGCTCCTTCT
AACAACCCCACAATATCACCCCTTACCACAAAATCTTCCTTCAGCTTAATCTCTCCCACTCT
GGGTTTCCATGCCACCCCTAATCCTGCATGAAGCAGCCCTGAGAAACATCACCCATTATCTC
TCCATGCCACCCCCCAAAAAATTTTTGC-TGCCCCAACACTTCAATACTATTTTGTATTATTTT
TCTTATTAATATAAGAAGGCAGG-------------------------------------
>Mandrillus_leucophaeus-HERVHF_INT(+)
--CTAGTGAAATTTGGTGCCGTGACTCAGACTGG
GGGACCTCCCTTGGGAGATCAATCCCCTGTCCTCCTGCTCTTTGCTCCGTGAGAAAGATCCA
CTTACGACCTCAGGTCCTCAGACCAACCAGCCCTAAGAACATCTCACCAATTTTAAATTGG
GTAAGCAGCCTCTCTTTACTCTCTTCTCCAACTTCTCTCACTATCCCTCAACCTCTTTCTCCT

TTCAATCTTGGTGCCACACTTCAATCTCTCCCTTCTCTTAATTTCAGTTCCTTTCCTTTTCTGG
TAGAGACAGAGGAGACGCATTTTATCCGTGAACCCAAAACTCCTGCACCGGTCACCGACTC
GGGAAGACAGTCTTCCCTTGGTGTTTAATCCCTGCAGGGACATCTGCCTGATTATTCACCCA
CATTTCATTGGTGTCTGATCACCACAGGGATGCCTGCCTTCGTCATTCACCCACAATCCCTT
GGTGGCAAGTCCATTGCAGGGACACCTGCTTTGGCTGCTCACCCACATTGCAGCCCAGGGC
TGCTCACCCCAC--CCCTTCTCTCCGTGTCT
CTACCCTCTCTTCTCTCCA------------------------------------CTTTCTTGGGGGGCTAGCA-CCCT
CACCC----------CTTCTCTCTGTGTCTCTACCC--TCTCT
TTTCTCTGGACTTGCCTCCTTCATTATGGACAAACTTCCACACCCTCCATTCCTCCTTCTTCT
CCCTTAGCCTGTGTTCTCAAGAACTTAAAACCTCTTCAACTCACAACTGACCTAAAACCTA
AATGCCTTATTTTCTTCTGCAACACGGCTTGGCCCC--AATACAAACTCGAC-AATGGTTCCA
AATAGCCAGAAAACGGCACTTTCGATTTCTCCATCCTGCAAGATCTAGATAATTCTCGTCATA
AAATGGGCAAATAGTCTGAGGTGCCTGATGTCCAGGCATTCTTTTACACATGAGTCCCTCCC
TGGTCTCTGTTCCCAATGCAACTCGTCCC---AAATCTTCCT
TCTTTCCCTCCCGCCTGTCCCCTCAGTCCCAACCCCAAGAGTTGCTGAGTCTTTCTAA--TCT
TCCTTTTTTATGGACCCATCTGACCTCTCCCCTCCTCCCCAGGCTGCTCCTCACCAGGCTGA
GCCAGGTCCCAA-TTCTTCCTCAGCCTCCGTTCCCCCACCCTATAATCCTTTTATCACCTCCC
CTCCTCACACTCAGTCCGACTTGCAGTTTCATTTTGCAACTAGCCCTCCCTCACCTGCCCAG
CAATTATCTCTTAAAAATGTGG-------CTGGAGCCGAAGGCATAGTCAGGGTTAATGCTCCTTT
TTCTTTATCCGGCCTCTCCCAAATCAGTTAGCGTTTAGGCTCTTTTTCATCAAATATAAAAAC
CCGGCCCAGTTCATGGCTTGTTTGGCAGCAACCCTGAGATGCTTTACTGCCCTAGACCCTGA
AAGGTCAAAAGGCCGTCTTATTCTCAATATACAT---TGTATTACCCAATCCACTCCTGACATTA
AATAAAACACCAAAAATTAAGTTCCGGCCCTCAAACCCCACAACAAGACTTAATTAACCTT
GCCTTCAAGGTGTACAATAATAGAGAAGAGTTGCAATTACTTGCCTCCGCTGTGAGAGAAA
CCCCAGCCACATCTTCAGCACACAAGAACTTCAAAATGCCTAAGCCACAGCAGCCAGGCAT
TCCTTCAGGACTTTCTCCCCCAGGATCTTGCTTCAAGTGCTGGAAATCTGGCCACTGGGCC
AAGGAATGCCCGTAGCCCAGGATTCC-TCCTAAACTGTGTCCCATTTGTGCAGGACCCCACT
GGAAATTGGACTGTCCAACTGGCCCAAGGCTCTGACTGACTCCTTCCCAGATCTTCTCAGC
TTA--GGCTGAAGACTGATGCTGCCCGATCGCCTTGGAAGCTTCCTGGACCATCCCAGACGC
TTTGGGTAACTCTTACAGTGGAGGGTAAGTCCATCCCCTTCTTAATCAATATGGAGGCTACC
CACTCCACATTACCTTCTTTTCAAGGGCCTGTTTCCCTTGCCTCCATAAATGTTGTGGGCATT
GACAGCCAGGTTTCTAAACCCCTTAAAACTCCCCCAGTCTGGTGCTAACTTGGACAACATT
CTTTTATGCACTCTTTTTCAGTTATCCCCACCTGGCCAGTTCCCTTATTAGGCTGAGATATTTT
AACCAAATTGTCTGCTTCCCTGACTATTCCTGGACTACAGCCACATCTCATTGCTACCCTGCT
TCCCAACCCAAAGCCTCCTTCGTGCCTTCCTCTCGTATTCCCCGAACTTAACCCACGTGTAT
GGGACACCTCTA---CTCCCTCCCTGGCAACCAATCACCAGCCTATTACTATCCCATTAAAACC
TAATCACCCTTACCCCATTCAATGCCAGTATCCCATCCCACAACAGGCTTTAAAGGGATTGA
AACCTGTTATCACTTGCCTGCTACAGTATGGGCTTCTAAAACCTGTAAACTCCCCTTACAATT
CCCCCATTTTACCTGTCCAAAAACTGGACAAGTCTTACAGGTTACTTCAGGATCTGTGCCTT
ATCAACCAAATTGTTTTGCCTAT---CCTTGTGGTGCGACACCCATTTACTCTCCTATCCTCAAT
ACCTCCCTCCACAACCCATTATTCTGTTCTGGATCTCAAACATGCTTTCTTTACTATTCCTTTG
CATCCTTCATCCCAGCCTCTTTTCGCTTTCACTTGGACTGACCCTGACACCCATCAGGCTCA
GGAAATTACCTGGGCTGTACTGCTGCAAGGCTTCACGGACAGCCCCTATTACTTCAGTCAA
GCCCAAATTTCTTCCTCATCTGTTACCTATCTTGGCATCATTC-TCATAAAAACACAC--GTGCT

CTCCCTGCTGATCATGTCTGGCTAATCTCCCAAACCCCAACCCCTTCTACAAAACAACAACT
CCTTCCTTC-ATAGGCATGGTTAGCTACTTCCACCTTTGGATACCTAGTTTTACCATCTTGACT
AAACCACGATGTAAACTCACAAAAGGAAACCTAGCTGACCCCACAGATCCTAAATCCTTTT
GCCACTCCTTTCCATTCCTTAAAAACAGCCCTAGAAGCTGCTCCCACACTAGCTCTCCCTAA
CTCATCCCAATCC-TTTTTCATTACACACAGTCAAAGTGCAGGGCTGTGAGGTCAAAATTCTT
ACACAAGGATCGGGACCATGCCCTGTGGCCTTTTTATCCAAACAACTTGACCTTACTCTTTT
AGCTTAG-CCTCATGTCTGTGTGTGGCAGCTGCCGCTTCCTTAATACTTTCAGAGGCCCTCA
AAATCACAAACTATGCTCAAC---------TCACTCTCTAGAGTTCTCATAACTTCCAAAATCTATT
TTCTTCCTCACACCTGATGCACATATTTCCTGCC---CCCCTCCACTACCTCTCAACAAGCCAA
ACTCATTGCCTTAACTCAAGCCCTCGCTCTTGCAAAAGGATTAAGTGTCAATATTTATACTGA
CTCTAAATATGCCTTCCATATCCTGCACCACCATGCTGTTATATGGGCAGAAAGAAATGTCCT
AACTATGCAAGGGTCCTCCATCATTAATGCCTCTTTAATAAAAACTCTTCTCAAAGCTGCTTT
ACTTCCAAAGGAAGCTGGAATCATTCACTGCAAGGGCCATCAAAAGGCATCAGATCCCATT
GCTCAGGACAATGCTTATGCTGATATGGTAGCTAAA-AAGCAGCTAGCATTCTAACTTCTATC
CCTCACGGCAGTTTTTCTCCTTCGCATTTGGCCACTCCCACCTACTCCCCCACTGAAACTTC
CACCTATCAGTCACTTCCCACGCAAGGCAAATGGTTCTTGGACCAAGGAAAATATCTCCTTC
CAGCCTCACAGGCCCATTCTATTCTGTCATCATTTCATAACCTCTTCCATGTAGGTTACAAGC
TGCTAGCCCATCTCTTAGAACCTCTCATTTCCTTTCCATCGTGGAGATCTATCCTCAAGGAAA
TCACTTCTCAG-TGTTCCATCTGCTATTCTACTACTCCTCAGGGATTGTTCAGGCCCCCTCCC
TTCCCTACACATCAAGCTCGGTGATTTGCCCCTGCCCAGGACTGGCAAATTGACTTTACTCA
CATGCCCCGAGTCAGGAAACTAAAATACCTCTTGGTCTAGGTAGACACTTTCACTGGACAG
GTAGAGGCCTTTCCCACAGGGTCTGCGAAGGCCACCATGGTCATTTCTTCCCTTCTGTCAG
GTATAATTCCTCAGTTTGACCTTCCTACCTCTATACAGTCCAATAATGGACCAGCCTTTGTTA
GTTAAATCACCCAACCAGTTTCTCAGTCTCTCAGTATTATTCAGTGGAACTTTCATACCCCTT
ACTATCCTCAGTCTTCAGGAAAGGTA-----GAACGGGCTAATGGTCTTTTAAAAACACACCTC
ACCAATCTCAGCCACCAACTTAAAAAGGACTAGACAATACTTTCACCACTTTCCCTTCTCAA
ACTTCAGGCCTGTCCTTGGGATGCTACAGGGTACAGCCCATTTGAGCTTCTGTATGAACCCT
CCTATTTATTAGGCCCCAGCCTTATTCCAGACACCAGCCCAACTTGGACTGTGCCCCAAAAA
CTTGTCATCCCTACTCTCTTCTGTCTAGTCATACTCCTATTCACCGTTCTCAAATACTCATAAA
TGCCCTGCTCTTGTTTACACTGCCGGTTTACACTGTTTCTCCAAGCCATCACAGCTGATATCT
CCTGGTGCTAT-CCCAAACTGCCACTCTTAACTCCCTCTTAAAGTAAATAAATAATCTTTGCT
GGCAGGGCTATGCTGAACCTCCTTGGGCACTCTCTAATTAGATGTCCTAGGTCCTCCCAATT
CTTAGTCCTTTAATACCTGTTTTTCTCCTTCTCTTATTCAGACCTTTT-GTCTTCCGTTTAGTTT
TTCAATTCATACAAAACTGCTTCCAGGCCATCACCAATCATTCTATATGATAAATGCTCCTTCT
AACAACCCCACAATATCACCCCTTACCACAAAATCTTCCTTCAGCTTAATCTCTCCCACTCT
GGGTTTCCATGCCACCCCTAATCCTGCATGAAGCAGCCCTGAGAAACATCACCCATTATCTC
TCCATGCCACCCCCCAAAAAATGTTTGC-TGCCCCAACACTTCAATACTATTTTGTATTATTTT
TCTTATTAATATAAGAAGGCAGG------------------------------------
>Pongo_abelii-HERVHF_INT(-)
GAAATAAACAGCCTTGTTGCTCACACAAAACCTGTTTGGTGGTCTCTTCATACAGACGCGA
GTGAAATTTGGTGCCGTGACTCGGACTGGGGGACCACCCTTGGGAGATAAAGCCCCTGTCC
TCCTGCTCTTTGCTCCATGAGAAAGATCCACCTACGACCTCGGGTCCTCAGACCAACCAGC
CCAAGGAACATCTCATCAATTTTAAATCCGGTAAGTGACCTCTCTTTACTCTCTTCTCCAACC
TCTCTTACTATCCCTCAACCTCTTTCTCCTTTCAATCTTGGCGCCACACTTCAATCTCTCCCTT

CTCTTAATTTCAGTTCCTTTCCTTTCCTGGTAGAGACAAAGGAGATGCGTTTTATCCGTGGAT
GCAAAACTCCGGCACCGGTCACAGACTCGGGAAGACAGTCTTCACTTGGTGTTTAATCACT
GTGGGGACGCCTGCCTGATTATTCACCCACATTTCATTGGTGTTTGATCACCGCAGGGACAT
CTGCCTTGGTCATTCACTCACATTCCCTTGGTGGCAAGTCAATTGCAGGGATGCCTGCTTTG
GCTGCTCACCTACATCGCAGCCCAGGGCTGCTCACCCTAC---
---------------------CCATTCTGTTTGTGTCTCTACCCTCTCTTTTCTCTCCACTTTCCTGGGGGGC
AAGCACCTCCCACCCCTTTTCCACTTTCCTGGGGGACAAGCACCTCCCACTC----------CTTCT
CTCTGTGTCTCTACCC---TCTCTTTTCTCTGGACTTGC
CTCCTTCACTATGGGCAAACTTC--CACCCTCCATTCCTCCTTCTTCTCCCTTAGCCTATGTTC
TCAAGAACTTAAAACCTCTTCAACTCACACCTGACCTAAAACCTAAATGCCTTATTTTCTTC
TGCAATGTCGCTTAACCCT--AATACAAAATCGACAAATGGTTCCAAATAGCCAGAAAACAG
CACTTTTGATTTCTCCATCCTACAAGATCTAGATAATTATTGTCGTAAAATGGGCAAATGGTC
TGAGGTGCCTGACGTCCAGGTATTCTTTTACACATTGGTCCCTCCCTGGTCTCTGTTCCCAAT
GCAACTTGTCCC--AAATCTTCTTTCTTTCCCTCCCGCCTG
TCCCTTCAGTCCCGACCCCAAGTGTTGCTGAGTCTTTCCAA--TCTTCCTTTTTTATGGACCC
ATCTGACCTCTCCCCTCCTCCCCAGGCTGCTCCTCGCCAGGCCAAGCCAGGTCCCAA-TTCT
TCCTCAGCCTCCGCTCCCCCACCCTTTAATCCTTTTATCACCTCCCCTCCTCACACCCAGTCT
GACTTACAGTTTCGTTCTGTGACTAGCCCTCCCCTACCTGCCCAGCAATTTCCTCTTAAAAA
CGTGG-------CTGGAGCTGAAGGCATAGTCAAGGTTAATGCTCCTTTTTCTTTATCCGGCCTCT
CCCAAATCAGTTAGAATTTAGGCTCTTTTT-ATGAAATATAAAAACCCAGCCCAGTTCATGGC
TTGTTTGGCAGCAATCCTGAGACACTTTACAGCCCTAGACCCTAAAAGGTCAAAAGCCCAT
CTTGTTCTCAAAATACGT---TTTATTACCCAATCTGCTCCTGACATTAAATAAAACTCCAAAA
ATTAAATTCCAGCCCTCAAACCCCACAACAGGACTTAATTAACCTTGCCTTCAGGGTGTACA
ATAATAGAGAAGAGTTGCAATTACTTGCCTCTGCTGTGAGAGAAACCCCAGCCACATCTCC
AGCACACAAGAACTTCAAAACTCCTAAGCCACAGTGGCCAGGCGTTCCTTCAGGACTTCC
TCCCCAAGGATCTTGCTTCAAGTGCTGGAAATCTGGCCACTGGGCCAAGGAATGCCCGCAG
CCCAGGATTCC-TCCTAAGCTGTGTCCCATCTGTGCAGGACCTCACTGGAAACTGGACTGTC
CAACTGGCCTAAGGCTCTGACTAGCTCCTTCCCAGATCTTCTCGGCTTAGCGGCTAAAGACT
GATGCTGCCTGATCACCTCAAAAGCTTCCTGGACCATCACAGAAGCTCTAGGTAACTCTTAC
AGTGGAGGGTAAGTCTGTCCCCTTCTTAATCAATACAGAGGCTACCTACTCCACATTACCTT
CTTTTCAAGGGCCTGTTTCCCTTGCCTCCATAACTGTTGTGGGTATTGACAGCCAGGCTTCT
AAACCACTCAAAAATCCCCCACTCTGGTGCTAACTTGGACAACATTCTTTTATGCACTCTTT
TTTAGTTATCCCTACCTGCCTAGTTCCCCTATCAGGCTGAGACATTTTAACTAAATTATCTGCT
TCCCTGACTATTCCTGGACTACAGCCACATCTCATTGCCGCCCTTCTTCCCAACCCAAAGCC
TCCTTCACGCCTTCTTCTCGTATCTCTCCACCTTAACCCACAAGTATGGAACACCTCTA---CT
CCCTCCCTGGCAACTGATCACCAGCCCATT---ATCCCATTAAAACCCAATCACCCTTACCCTG
CTCAACACTAGTATCCCATCCCACAACAGGCTTTAAAGGGATTGAAGCCTGTTATCACTCGC
CTGCTACAGCATGGGCTTCTAAAACCTATAAACTCTTCTTAAAATTCCCCCATTTTACCTGTC
CAAAAACCAGACAAGCCTTACAGATTAGTTCAGGATCTGCACCTTAACAACCAAGTTGTTT
TACCTGTCCACCCCATGGTGCCAAACCCATATACTCTCCTATCCTCAATACCTCCCTCCACAA
CCCATTATTCTGTTCTGGATCTCAAACATGCTTTCTTTACTATTCCTTTGCACTCTTCATCCCA
GTCTCTCTTCACTTTCACTTGGACTGACCCTGACACCCATTAGGCTCAGCAAATTACCTGGG
CTGTACTGCTGCAAGCCTTCACAGACAGCCCCCATTACTTCAGTCAAGCCCAAATTTCTTCC
TCATGTGTTACCTATCTCGACATAATTC-TCATAAAAACACAC--GTGCTCTCCCTGCTGATCAT

GTCCAGCTAATCTCCCAAACCCTGACCCCTTCTACAAAACGACAACTCCTTTCCTTCCTACG
CATGGTTAGGTACTTCTGCCTTTGGATACCTAGTTTTACCATCCTGACTTAACCATTATATAAA
CTCACAAAAGCAAACCTAGCTGACCCCACAGATCCTAAATCCTTTTGCCACTCCTTTCCATT
CCTTAAAAACAGCCCTAGAAGCTGCTCCCACACTAGTTCTCCCTAACTCATCTCAACCC-TT
TTTCATTA-ACACAGCCAAAGTGCAAGGCTGTGCGGTCAAAATTCTTACACAAGGACCGGG
ACCACGCCCTGTGGGCTTTTTATCCAAACAACTTGACCTTACTGTTTTAGCCTAGCCCTCAT
GTCTGCGTGCAGTGGCTGCCGCTGCCTTAATACTTTTAGAGGCCCTTAAAATCACGAACTAT
GCTCAACTCTCTACAGTCACTCTCTACAGTTCTTATAACTTCCAAAATCTATTTCTTCCTCA
CACCTGACGCGTATACTTTCTGCT---CCCCTCCACTACCTCTCAGCAAGCCAAACTCATTGCC
TTAACTCGAGCCCTCACTCTTGCAAAAGGACTACGTGTCAATATTTATACTGACTCTAAATAT
GCCTTCCATATCCTGTACCACCATGCTGTTATATGGGCAGAAAGAAATTTCCTCACTATGCAA
AGGTCCTCCATCATTAATTCCTCTTTAATAAAAACTCTTCTCAAAGCTGCTTTACTTCCAAAG
GAAGCTGGAGTCGTTCACTGCAAGGGCCATCAAAAGGCATCAGATCCCATCACTCAGGGC
AATGCTTACGCTGACAAGGTAGCTAAAGAAGCAGCTAGCGTTCCAAATTCTATCC-------CAG
TTTTTCTCCTTCTTGTCTGGCCACTCCCACTTACTACCCCACTGAAACTTCCACC-GTTAATC
TCTTCCCACACAAGGCAAATGGTTCTTGGACCAAAGAAAATATCTCCTTCCAACCTCACAG
GCCCATTCTATTCTGTCATCATTTCATAACCTCTTCCATGTAGGTTACAAGCTGCTAGCCCAC
CTCTTAGAACCTCTCATTTCCTTTCCATCGTGGAAATCTATCCTCAAGGAAATCAGTTCTCAG
-TGTTCCACCTGCTATTCTACTACTTCTCAGGGATTATTCAGGCCCCCTCCCTTCCCTACACAT
CAAGCTCAGGGATTGGCCCCTGCCCAGTACTGGCAAATTGACTTTACTCACATGCCCTGAG
TCAGGAAACTAAAATACCTCTCGGTCTAGATAGACACTTTCACTGGATGGGTAGAGGCCTTT
CCCACAGGGTCTGAGAAGGCCACCAGGGTCATTTCTTCCCTTCTGTCAGACATAATTCCTCG
GTTTGGCCTTCCCACCTCTATACAGTCCGATAACGGACCAGCCTTTATTAGTCAAATCACCC
AAGCAGTTTCTCAGGCTCTTAG---TGTTCAGTGGAACCTTCATACCCCTTACTGTCCTTAGTC
TTCAGGAAAGGTAGAACGGAACGGACTAATGGTCTTTTCAAAACACACCTCACCAAGCTC
AGCCACCAACTTAAAAAGGACTAGACAATACTTTTACCACTTTCCCTTCTCAAAATTCGGGC
CTGTCCTTGGAATGCTACAGGGTACAGCCCATTTGAGCTCCTGTATAGACTCTCCTTTTTATT
AGGCCACAGCCTCATTCCAGACACCAGCCTAACTTGGACTGTGCCCCAAAAACTTGTCATC
CATACTATCTTCTGCCTAGTCATACTCTTATTCACTGTTCTC---TACTCATAAATGCCCTACTCT
TGTTTACACTGCCGGTTTACACTGTTTCTCCAGGCCATCACAGCTGATATCTCCTGGTGCTAT
CCCCAAATCACCACTCTTAACTCCCTCTTAAAGTAAATCAATAATCTTTGCTGGCAGGGCTAT
GCTGAACCTCCTTGGGCACTCTCTAATTGGATGTCCTGGGTCCTCCCAATTCTTAGTCCTTTA
ATACCTGTTTTTCTCCTTCTCTTATTCGGACCTTGT-GTCTTCCGCTTAGTTTTTCAATTCATAC
AAAACCACATCCAGGCCATCACCAATCATTCTATACGACAAATGCTCCTTCTAACAACCCCA
CAATATCACGCCTTACCACAAAATCTTCCTTCAGCTTAATCTCTCCCACTCTGGGTTCCCATG
CCACCCCTAATCCCGCTTGAAGCAGCTCTGAGAAACATTGCCCATTATCTCTCCATGTCGCC
CCCCAAAAAATTTTCGC-TGCCCCAACACTTCAATACTATTTTATGTTATTTTTCTTATTAATAT
AAGAAGGCAG--------------------------------------
>Pygathrix_nemaeus-HERVHF_INT(+)
---TTTGGTGCCGTGACTCAGATTGGGGGAC
CTCTCTTGGGAGATCAATCCCCTGTCCTCCTGCTCTTTGCTGCATGAGAAAGATCCACTTAC
GACCTCAGGTCCTCAGACCAACCAGCCCCAAGAACATCTCACCAATTTTAAATGGGGTAAG
CAGCCTCTCTTTACTCTCTTCTCTAACCTCTCTCACTATCCCTCAACCTCTTTCTCCTTTCAAT
CTTGGTGCCACACTTCAATCTCTCCCTTCTCTTAATTTCAGTTCCTTTCCTTTTCTGGTAGAG

ACAGAGGAGACGTGTTTTATCCATGAACCCAAAACTCTGGCACCGGTCACCAACTCAGGA
AGACAGTCTTCCCTTGGTGTTTAATTCCTGCAGGGATGTCTGCCTGATTATTCACCCACATTT
CATTAGTGTCTGATCACCGCAGGGATGCCTGCCTTCGTCATTCACCCACAATCCCTTGGTGG
CAAGTCTATTGCAGGGACGTCTGCTTTGGCTGCTCACCCACATTGCAGCCTAGGGCTCCTCA
CCCCAC---CTCTTCTCTTCGTGTCTCTACTCT
CTCTTCTCTCCA--------------------------------------CTTTCTTGGGGGGCAAGCACCCCTCACCC---
-------CTTCTCTCTGTGTCTCTACCC--TCTCTTTCTCT
GGACTTGCCTCCTTCACTATGGGCAACCTTC--CACCCTCCATTCCTCCTTCTTCTCTCTTAGC
CTGTGTTCTCAAGAACTTAAAACCTCTTCAACTCACACCTGACCTAAATCCTTAATGCCTTA
TTTTCTTCTGCAATGCCTCTTAACCCC--AATACAAACTCGAC-AATGGTTCCAAATAGCCAGA
AAACGGCACTTTCGATTTCTCCATCCTACAAGATCTAGATAATTCTCGTCATAAAATGGGCA
AATAGTCTGAGGTGCCTGATATCTAGGCATTCTTTTACACATGGGTCCCACCCTGGTCTCTGT
CCCCAATGCAACTCATCCC--AAATCTTCCTTCTTTCCCTC
CTGTCTGTCCCCTCAGTCCCAACCCCAAGAGTTGCTGAGTCTTTCTAA--TCTTCCTTTTTA
CGGACCCATCTGACCTCTCCCCTCCTCCCCAGGCTGCTCCTCACCAGGCCAAGCGAGGTCC
CAAGTTCTTCCTCAGCCTCCACTCCCCCACCCTATAATCCTTTTATCACCTCCCCTCCTCACA
CCCGGTCCGACTTACAGTTTCATTCTGTGACTAGCCCTCCCCCACCTGCCCAGCAATTACCT
CTTAAAAAGGTGG-------CTGGAGCTGAAGGCATAGTCAAGGTTAGTGCTCCTTTTTCTTTAT
CTGGCCTCTCCCAAATCAGTTAGCATTTAGGCTCTTTTTCATCAAATATAAAAACCCAGCCC
AGCTCATGGCTTGTTTGGCAACAACCCTGAGACGCTTTACTGCCCTAGACCCTGAAAGGCC
AAAAGGCTGTCTTATTCTCAATATACAT---TGTATTACCCAATCCACTGCTGACATTAAATAAA
ACACCAAAAATTAAGTTCCAGCCCTCAAACCCCACAACGGGACTTAATTAACCTTGCCTTC
AAGGTGTACAATAATAGAGAAGAGTTGCAATCACTTGCGTCCTCTGTGAGAGAAACCCCAG
CCACATCTCCAGCACACAAGAACTTCAAAACGCCTAAGCCACAGTGGCCAGGCGTTCCTTC
AGGACTTTCTCCCCCAGGATCTTGCTTTAAGTGCTGGAAATCTGGCCACTGGGCCAAGGAA
TGCCCGTAGCCCAGGATTCC-TCCTAAGATGTGTCCCATCTGTGCAGGACCCCACTGGAAAT
TGGACTGTCCAACTGACCCAAGGCTCTGACTGACTCCTTCCCAGATCTTCTCAGCTTAGTG
GCTGAAGACTGACACTGCCCAATTGCCTTGGAAGCCTCCTGGACCATCCCAGATGCTTTGG
GTAACTCTTACAGTTGAGGGTAAGTCTGTCCCCTTCTTAATCAATACAGAGGCTACCCACTC
CACATTATCTTCTTTTCAAGGGCCTGTTTCCCTTGCCTCCATAACTGTTGTGGGTATTGATGG
CCAGGCTTCTAAACCTCTTAAAACTCCCCCACTCTCATGCTAACTTGAACAATATTATTTTAT
GCACTCTTCTTTAGTTA-CTCCACCTGCCCAGTTCCCTTATTAGGCCGAGATATTTTAACCAAA
TTGTCTGCTTCCCTGACTATTCCTGGACTACAGCCACATCTCATTGCTACCCTGCTTCCCAAC
CCAAAGCCTCCTTCGCACCTTCCTCTCATATCCCCCAAACTTAACCCACAAGTATGGGACAC
CTCTA---CTCCCTCCCTGGCAACCGATCACCAGCCTATTACTATCCCATTAAAACCTAATCAC
CCTTATCCCATTCAACGCCAGTATCCCATCCCACAACAGGCTTTAAAGGGATTGAAGCCTGT
TATCACTCGCCTGCTACAGTATGGGCTTCTAAAACCTGTAAACTCCCCTTACAATTCCCCTGT
TTTACCTGACCAAAAACCGGACAAGTCTTACAGGTTACTTCAGGATCTGTGCCTTATCAACC
AAATTGTTTTGCCTATCCACCTTGTGGTGTGAAACCCATTTACTCTCCTATCCTTGATACCTC
CCTCCACAACCCATTATTCCGTTCTGGATCTCAAACATGCTTTGTTTACTATTCCTTTGCATCC
TTCATGCCAGCCTCTTTTCACTTTCACTTGGACTGACCCTGACACCCATCAGGCTCAGGAAA
TTACCTGGGCTGTACTGCTGCAAGACTTCGCAGACAGCCCCCATTACTTCAGTCAAGCCCA
AATTTCTTCTTCATCTGTTACCTATCTTGGCATAATTC-TCATAAAAACACAT--GTACTCTCCCT
GCTGATCATGTCTGGCTAATCTCCCAAACCCCAACCCCTTCTACAAAACAACAACTCCTTCC

TTC-GTAGGCATGGTTAGGCACTTCCACCTTTGGATACCTAGTTTTACCATCTTGACTAAACC
ATGATGTAAACTCACAAAAGGAAACCTAGCTGACCCCACAGATCCTAAATCCTTTGACCAC
TCCTTTCCATTCCTTAAAAACAGCCCTAGAAGCTGCTCCCACACTAGCTCTCCCTAACTCAT
CCCAATCC-TTTTTCATTACACACAGTCAAAGTGCAGGGCTGTGAGGTCAAAATTCTTACAC
AAGGATCAGGACCATGCCCTGTGGCCTTTTTATCCAAACAACTTGACCTTACTATTTTAGCTT
AG-CGTCATGTCTGTGTGTGGCAGCTGCCCCTTCCTTAATACTTTTAGAGGCCCTCAAAATCA
CAAACTATGCTCAAC---------TTACTCTCTAGAGTTCTCGTAACTTCCAAAATCTATTTTCTTCC
TCACACCTGATGCACATATTTTCTGCT---CCCCTCCACTACCTCTCAACAAGCCAAACTCATT
GCCTTAACTCAAGCCCTCGCTGTTGCAAAAGGATTAAGCATCAATATTTATACTGACTCTAAA
TATGCCTTCCATATCCTGCACCACCATGCTGTTATGTGGGTAGAAAGAAATTTCCTCACTATG
CAAGGGTCCTCCATCATTAATGCCTCTTTAATAAAAACTCTTCCCAAAGCTGCTTTACTTCCA
AAGGAAGCTGGAGTCATTCACTGCAAGGGCCATCAAAAGGCATCAGATCCCATTGTTCAGG
ATAATGCTTATGCTGATACGGTAGCTAAA-AAGCAGCTAGCATTCCAACTTCTGTCCCTCACT
GCAGTTTTTCTCCTTCTCATCTGGCCACTCCCACCTACTCCCCCACTGAAACTTCCACCTAG
CAATCTCTTCCCACACAAGGCAAATGGTTCTTGGACCAAGGAAAATATCTCCTTCCAGCCTC
ACAGGCCCATTCTATTCTGTCATCATTTCATAACCTCTTCCATGTAGGTTACAAGCTGCTTGC
CCGTCTCTTAGAACCTCTCATTTCCTTTCCATCGTGGAAATCTATCCTCAAGGAAATCACTTC
TCAG-TGTTCCATCTGCTATTCTACTAATCCTCAGGGATTGTTCAGGCCCCCTCCCTTCCCTAC
ACATCAAGCTTGGGGATTTGCTCCTGCCCAGGACTGGCAAATTGACTTTACTTACATGCCCC
GAGTCAGGAAACTAAAATACCTCTTGGTCTGGGTAGACACTTTCACTGGATGGGTAGAGGC
CTTTCCCACAGGGTCTGCGAAGTCCACCGTGGTCATTTCTTCCCTTCTGTTAGGCATAATTC
CTCGGTTTGACCTTCCTACCTCTATACAGTCCAATAATGGACCAGCCTTTGTTAGTTAAATCA
CCCAACCAGTTTCTCAGGCTCTTAG---TATTCAGTGGAACTTTCATACCCCTTACCATCCTCA
ATCTTCAGGAAAGGTA-----GAACGGGCTAATGGTCTTTTAAAATCACACCTCACCAAGCTCA
GCCACCAACTTAAAAAAGACTAGACAATACTTTTACCACTTTCCCTTCTCAAAATTCGGGCC
TGTCCTCGGGATGCTACAGGATACAGCCCATTTGAGCTTCTGTATGAACGCTCATTTTTATTA
GGCCCCAGTCTTATTCCAGACACGAGCCCAACTTGGACTGTGCCCCAAAAACTTGTCATCC
CTACTCTCTTCTGTCTAGTCATACTCCTATTCACTGTTCTCAAATACTCATAAATGCCCTGCTC
TTGTTTACACTGCCGGTTTACACTGTTTCTCCAAGCCATCACAGCTGATGTCTCCTGGTGCTA
T-CCCAAACTGCCACTCTTAACTCCCTCTTAAAGTAAATAAATACTCTTTGCTGGCAGGGCTA
TGCTGAACCTCCTTGGGCACTGTCTAATTGGATGTCCTGGATCCTCCCAATTCTCAGTCCTTT
AATACCTGTTTTCTCCTTCTCTTATTCGGACTTTTT-GTCTTCCGTTTAG-TTCTCAATTCATA
CAAAACTGCTTCCAGGCCATCACCAATCATTCTATATGACAAATGCTCCTTCTAACAACCCC
ACAATATCACCCCTTACCCCAAAATCTTTCTTCAGCTTAATCTCTCCCATTCTGGGTTTCCAT
GCCACCCCTAATCCTGCTTGAAGCAGCCCTGAGAAATATCACCCATTATCTCTCCATACCATC
CCCCCAAAAATTTTTGC-TGCCCTAACACTTCAGTACTATTTTATATTATTTTTCTTATTAATAT
AAGAAGGC---------------------------------------
>Rhinopithecus_roxellana-HERVHF_INT(-)
--ATTTGGTGCCGTGACTCAGATTGGGGGA
CCTCTCTTGGGAGATCAATCCCCTCTCCTCCTGCTCTTTGCTGCATGAGAAAGATCCACTTA
CGACCTCAGGTCCTCAGACCAACCAGCCCCAAGAACATCTCACCAATTTTAAATGGGGTAA
GCAGCCTCTCTTTACTCTCTTCTCCAACCTCTCTCACTATCCCTCAACCTCTTTCTCCTTTCA
ATCTTGGTGCCACACTTCATTCTCTCCCTTCTCTTAATTTCAGTTCCTTTCCTTTTCTGGTAGA
GACAGAGGAGACGTGTTTTATCCATGAACCCAAAACTCTGGCACCGGTCACCAACTCGGG

AAGACAGTCTTCCCTTGGTGTTTAATTCCTGCAGGGACGTCTGCCTGATTATTCACCCACAT
TTCGTTGGTGTCTGATCACCGCAGGGATGCCTGCTTTCGTCATTCACCCACAATCCCTTGGT
GGCAAGTCTATTGCAGGGACGCCTGCTTTGGCTGCTCACCCACATTGCAGCCTAGGGCTGC
TCACCCCAC---CCCTTCTCTTCGTGTTGCTA
CTCTCTCTTCTCTCCA--CTTTCTTGGGGGGCAAGCACCCCTCAT
CC----------CTTCTCTCTGTGTCTCTACCC---TCTCTTTT
CTCTGGACTTGCCTCCTTCACTATGGGCAACCTTC--CACCCTCCATTCCTCCTTCTTCTCTCT
TAGCCTGTGTTCTCAAGAACTTAAAACCTCTTCAACTCACACCTGACCTAAAACCTTAATGC
CTTATTTTCTTCTGCAATGCCTCTTAACCCC--AATACAAACTCGAC-AATGGTTCCAAATAGC
CAGAAAACGGCACTTTCGATTTCTCCATCCTACAAGATCTAGATAATTCTCATCATAAAATGG
GCAAATAGTCTGAGGTGCCTGATGTCTAGGCATTCTTTTACACATGGGTCCCACCCTGGTCT
CTGTCCCCAATGCAATTCATCCC--AAATCTTCCTTCTTTC
CCTCCTGTCTGTCCCCTCAGTCCCAACCCCAAGAGTTGCTGAGTCTTTCTAA--TCTTCCTTT
TTTACGGACCCATCTGACCTCTCCCCTCCTCCCCAGGCTGCTCCTCGCCAGGCCAAGCGAA
GTCCCAAGTTCTTCCTCAGCCTCCACTCCCCCACCCTCTAATCCTTTTATCACCTCCCCTCCT
CACACCCGGTCCGACTTACAGTTTCATTCTGTGACTAGCCCTCCCCCACCTGCCCAGCAATT
ACCTCTTAAAAAGGTGG-------CTGGAGCTGAAGGCACAGTCAAGGTTAATGCTCCTTTTTCT
TTATCCAGCCTCTCCCAAATCAGTTAGCATTTAGGCTCTTTTTCATCAAATATAAAAACCCAG
CCCAGTTCATGGCTTGTTTGGCAACAACCCTGAGACGCTTTACTGCCCTAGACCCTGAAAG
GCCAAAAGGCTGTCTTATTCTCAATATACAC---TGTATTACCCAATCCACTCCTGACATTAAAT
AAAACACCAAAAATTAAGTTCCAGCCCTCAAACCCCACAACAGGACTTAATTAACCTTGCC
TTCAAGGTGTACAATAATAGAGAAGACTTGCAATCACTTGCGTCCTCTGTGAGAGAAACCC
CAGCCACATCTCCAGCACACAAGAACTTCAAAACGCCTAAGCCACAGTGGCCAGGCGTTC
CTTCAGGACTTTCTCCCCCAGGATCTTGCTGTAAGTGCTGGAAATCTGGCCACTGGGCCAA
GGAATGCCCGTAGCCCAGGATTCC-TCCTAAGATGTGTCCCATCTGTGCAGGACCCCACTGG
AAATTGGACTGTCCAACTGACCCAAGGCTCTGACTGACTCCTTCCCAGATCTTCTCAGCTTA
GTGGCTGAAGACTGACGCTGCCCAATTGCCTTGGAAGCCTCCTGGACCATCCCAGATGCTT
TGGGTAACTCTTACAGTTGAGGGTAAGTCTGTCCCCTTCTTAATCAATACAGAGGCTACCCA
CTCCACATTATCTTCTTTTCAAGGGCCTGTTTCCCTTGCCTCCATAACTGTTGTGGGTATTGA
TGGCCAGGCTTCTAAACCCCTTAAAACTCCCCCACTCTCGTGCTAACTTGGACAATATTATT
TTATGCACTCTTCTTTAGTTA-CCCCACCTGCCCAGTTCCCTTATTAGGCCAAGATATTTTAAC
CAAATTGTCTGCTTCCCTGACTATTCCTGGACTACAGCCACATCTCATTGCTACCCTGCTTCC
CAACCCAAAGCCTCCTTCGCGCCTTCCTCTCATATCCCCCAAACTTAACCCACAAGTATGGG
ACACCTCTA---CTCCCTCCCTGGCAACCGATCACCAGCCTATTACTATCCCATTAAAACCTAA
TCACCCTTATCCCATTCAACGCCAGTATCCCATCCCACAACAGGCTTTAAAGGGATTGAAGC
CTGTTATCACTCGCCTGCTACAGTATGGGCTTCTAAAACCTGTAAACTCCCCTTACAATTCCC
CCATTTTACCTGTCCAAAAACCAGACAAGTCTTACAGGTTACTTCAGGATCTGTGCCTTATC
AACCAAATTGTTTTGCCTATCCACCTTGTGGTGTGAAACCCATTTACTCTCCTGTCCTCGATA
CCTCCCTCCACAACCCATTATTCCGTTCTGGATCTCAAACATGCTTTGTTTACTATTCCTTTG
CATCCTTCATGCCAGCCTCTTTCCACTTTCACTTGGACTGACCCTGACACCCATCAGGCTCA
GGAAATTACCTGGGCTGTACTGCTGCAAGACTTCGCGGACAGCCCCTATTACTTCAGTCAA
GCCCAAATTTCTTCCTCATCTGTTACCTATCTTGGCATAATTC-TCATAAAAACACAT--GTGCT
CTTCCTGCTGATCATGTCTGGCTAATCTCCCAAACCCCAACCCCTTCTACAAAACAACAACT
CCTTCCTTC-GTAGGCATGGTTAGGCACTTCCACCTTTGGATACCTAGTTTTACCATCTTGACT

AAACCATGATGTAAACTCACAAAAGGAAACCTAGCTGACCCCACAGATCCTAAATCCTTTG
ACCACTCCTTTCCATTCCTTAAAAACAGCCCTAGAAGCTGCTCCCACACTAGCTCTCCCTAA
CTCATCCCAATCC-TTTTTCATTACACACAGTCAAAGTGCAGGGCTGTGAGGTCAAAATTCTT
ACACAAGGATCGGGACCATGCCCTGTGGCCTTTTATCCAAACAACTTGACCTTACTGTTTT
AGCTTAG-CGTCATGTCTGTGTGTGGCAGCTGCCGCTTCCTTAATACTTTTAGAGGCCCTCAA
AATCACAAACTATGCTCAAC---------TTACTCTCTAGAGTTCTCATAACTTCCAAAATCTATTTT
CTTCCTCACACCTGATGCACATATTTTCTGCT---CCCCTCCACTACCTCTCAACAAGCCAAAC
TCATTGCCTTCACTCAAGCCCTTGCTCTTGCAAAAGGATTAAGCATCAATATTTATGCTGACT
CTAAATATGCCTTCCATATCCTGCACCACCATGCTGTTATGTGGGTAGAAAGAAATGTCCTCA
CTATGCAAGGGTCCTCCATCATTAATGCCTCTTTAATAAAAACTCTTCTCAAAGCTGCTTTAC
TTCCAAAGGAAGCTGGAGTCATTCACTGCAAGGGCCATCAAAAGGCATCAGATCCCATTGT
TCAGGACAATGCTTATGCTGATACGGTAGCTAAA-AAGCAGCTAGCATTCCAACTTCTGTCC
CTCACTGCAGTTTTTCTCCTTCTCATCTGGCCACTCCCACCTACTCCCCCACTGAAACTTCC
ACCTATCAATCTCTTCCCACACAAGGCAAATGGTTCTTGGACCAAGGAAAATATCTCCTTCC
AGCCTCACAGGCCCATTCTATTCTGTGGTCATTTCATAACCTCTTCCATGTAGGTTACAAGCT
CCTTGCCCGTCTCTTAGAACCTCTCATTTCCTTTCCATCGTGGAAATCTATCCTCAAGGAAAT
CACTTCTCAG-TGTTCCATCTGCTATTCTACTAATCCTCAGGGATTGTTCAGGCCCCCTCCCTT
CCCTACACATCAAGCTTGGGGATTTGCCCCTGCCCAGGACTGGCAAATTGACTTTACTCACA
TGCCCCGAGTCAGGAAACTAAAATACCTCTTGGTCTGGGTAGACACTTTCACTGGATGGGT
AGAGGCCTTTCCCACAGGGTCTGCAAAGGCCACTGTGGTCATTTCTTCACTTCTGTTAGGC
ATAATTCCTCAGTTTGACCTTCCTACCTCTATACAGTCCAATAATGGACCAGCCTTTGTTAGT
TAAATCACCCAACCAGTTTCTCAGGCTCTTAG---TATTCAGTGGAATTTTCATACCCCTTACC
ATCCTCAATCTTCAGGAAAGGTA-----GAACGGGCTAATGGTCTTTTAAAATCACACCTCACC
AAGCTCAGCCACCGACTTAAAAAAGACTAGACAATACTTTTACCACTTTCCCTTCTCAAAAT
TCGGGCCTGTCCTCGGGATGCTACAGGGTACAGCCCATTTGAGCTTCTGTATGAACGCTCAT
TTTTATTAGGCCCCAGTCTTATTCCAGACACGAGCCCAACTTGGACTGTGCCCCAAAAGCTT
GTCATCCCTACTCTCTTCTGTCTAGTCATACTCCTATTCACCGTTCTCAAATACTCATAAATGC
CCTGCTCTTGTTTACACTGCCGGTTTACACTGTTTCTCCAAGCCATCACAGCTGATGTCTCCT
GGTGCTAT-CCCAAACTGCCACTCTTAACTCCCTCTTAAAGTAAATAAATACTCTTTGCTGGC
AGGGCTATGCTGAACCTCCTTGGGCACTCTCTAATTGGATGTCCTGGATCCTCCCAATTCTC
AGTCCTTTAATACCTGTTTTTCTCCTTCTCCTATTCGGACCTTTT-GTCTTCCGTTTAG-TTTTC
AATTCATACAAAACTGCTTCCAGGCCATCACCAATCATTCTATATGATAAATGCTCCTTCTAA
CAACCCCACAATATCACCCCTTACCCCAAAATCTTTCTTCAGCTTAATCTCTCCCATTCTGGG
TTTCCATGCCACCCCTAATCCTGCTTGAAGCAGCCCTGAGAAATATCACCCATTATCTCTCCA
TACCATCTCCCCAAAAATTTTTGC-TGCCCTAACACTTCAGTACTATTTTATATTATTTTTCTTA
TTAATATAAGAAGGCAG-------------------------------------
>Rhinopithecus_strykeri-HERVHF_INT(+)
--TTTGGTGCCGCGACTCAGATTGGGGGAC
CTCTCTTGGGAGATCAATCCCCTCTCCTCCTGCTCTTTGCTGCATGAGAAAGATCCACTTAC
GACCTCAGGTCCTCAGACCAACCAGCCCCAAGAACATCTCACCAATTTTAAATGGGGTAAG
CAGCCTCTCTTTACTCTCTTCTCCAACCTCTCTCACTATCCCTCAACCTCTTTCTCCTTTCAAT
CTTGGTGCCACACTTCAATCTCTCCCTTCTCTTAATTTCAGTTCCTTTCCTTTTCTGGTAGAG
ACAGAGGAGACGTGTTTTATCCATGAACCCAAAACTCTGGCACCGGTCACCAACTCGGGA
AGACAGTCTTCCCTTGGTGTTTAATTCCTGCAGGGACGTCTGCCTGATTATTCACCCACATTT

CATTAGTGTCTGATCACTGCAGGGATGCCTGCTTTCGTCATTCACCCACAATCCCTTGGTGG
CAAGTCTATTGCAGGGACACCTGCTTTGGCTGCTCACCCACATTGCAGCCTAGGGCTGCTC
ACCC--CTTCTCTCTGTGTCTCTACCCTC
TCTTTTCTCTGGACTT------------------------------GCCTCCTTCACTATGGGCAACCTTCCACCCTC
CATTCCTCCTTCTTCTCTCTGTGTCTCTACCC---TCTC
TTTTCTCTGGACTTGCCTCCTTCACTATGGGCAACCTTC--CACCCTCCATTCCTCCTTCTTCT
CTCTTAGCCTGTGTTCTCAAGAACTTAAAACCTCTTCAACTCACACCTGACCTAAAACCTTA
ATGCCTTATTTTCTTCTGCAATGCCTCTTAACCCC--AATACAAACTCGAC-AATGGTTCCAAA
TAGCCAGAAAACGGCACTTTCGATTTCTCCATCCTATAAGATCTAGATAATTCTCGTCATAAA
ATGGGCAAATAGTCTGAGGTGCCTGATGTCTAGGCATTCTTTTACACATGGGTCCCACCCTG
GTCTCTGTCCCCAGTGCAACTCATCCCAAATCTTCCTTCTTTCCCTCCTGTCTGTCCCCAGTG
CAACTCATCCCAAATCTTCCTTCTTTCCCTCCTGTCTGTCCCCTCAGTCCCAACCCCAAGAG
TTGCTGAGTCTTTCTAA--TCTTCCTTTTTTACGGACCCATCTGACCTCTCCCCTCCTCCCCAG
GCTGCTCCTCACCAGGCCAAGCGAAGTCCCAAGTTCTTCCTCAGCCTCCGCTCCCC-ACCCT
ATAATCCTTTTATCACCTC-CCTCCTCACACCCGGTCCGACTTACAGTTTCATTCTGTGACTAG
CCCTCCCCCACCTGCCCAGCAATTACCTCTTAAAAAGGTGG-------CTGGAGCTGAAGGCATA
G-CAAGGTTAATGCTCCTTTTTCTTTATCCGGCCTCTCCCAAATCAGTTAGCATTTAGGCTCTT
TTTCATCAAATATAAAAACCCAGCCCAGTTCATGGCTTGTTTGGCAACAACCCTGAGACGCT
TTACTGCCCTAGACCCTAAAAGGCCAAAAGGCTGTCTTATTCTCAATATACATGTATGTATTA
CCCAATCCACTCCCGACATTAAATAAAACACCAAAAATTAACTTCCAGCCCTCAAACCCCA
CAACAGGACTTAATTAGCCTTGCCTTCAAGGTGTACAATAATAGAGAAGACTTGCAATCACT
TGCGTCCTCTGTGAGAGAAACCCCAGCCACATCTCCAGCACACAAGAACTTCAAAACGCC
TAAGCCACAGTGGCCAGGCGTTCCTTCAGGACTTTCTCCCCCAGGATCTTGCTGTAAGTGC
TGGAAATCTGGCCACTGGGCCAAGGAATGCCCGTAGCCCAGGATTCC-TCCTAAGATGTGTC
CCATCTGTGCAGGACCCCACTGGAAATTGGACTGTCCAACTGACCCAAGGCTCTGACTGAC
TCCTTCCCAGATCTTCTCAGCTTAGTGGCTGAAGACTGACGCTGCCCAATTGCCTTGGAAG
CCTCCTGGACCATCCCAGATGCTTTGGGTAACTCTTACAGTTGAGGGTAAGTCTGTCCCCTT
CTTAATCAATACAGAGGCTACCCACTCCACATTATCTTCTTTTCAAGGGCCTGTTTCCCTTGC
CTCCATAACTGTTGTGGGTATTGATGGCCAGGCTTCTAAACCCCTTAAAACTCCCCCACTCT
CGTGCTAACTTGGACAATATTATTTATGCACTCTTCTTTAGTTA-CCCCACCTGCCCAGTTCC
CTTATTAGGCCAAGATATTTTAACCAAATTGTCTGCTTCCCTGACTATTCCTGGACTACAGCC
ACATCTCATTGCTACCCTGCTTCCCAACCCAAAGCCTCCTTCGCGCCTTCCTCTCATATCCCC
CAAACTTAACCCACAAGTATGGGACACCTCTA---CTCCCTCCCTGGCAACCGATCACCAGCT
TATTACTATCCCATTAAAACCTAATCACCCTTATCCCATTCAATGCCAGTATCCCATCCCACAA
CAGGCTTTAAAGGGATTGAAGCCTGTTATCACTTGCCTGCTACAGTATGGGCTTCTAAAACC
TGTAAACTCCCCTTACAATTCCCCCATTTACCTGTCCAAAAACCAGACAAGTCTTACAGGT
TACTTCAGGATCTGTGCCTTATCAACCAAATTGTTTTGCCTATCCACCTTGTGGTGTGAAACC
CATTTACTCTCCTATCCTCGATACCTCCCTCCACAACCCATTATTCCGTTCTGGATCTCAAACA
TGCTTTGTTTACTATTCCTTTGCATCCTTCATGCCAGCCTCTTTCCACTTTCACTTGGACTGA
CCCTGACACCCATCAGGCTCAGGAAATTACCTGGGCTGTACTGCTGCAAGACTTCGCGGAC
AGCCCCTATTACTTCAGTCAAGCCCAAATTTCTTCCTCATCTGTTACCTATCTTGGCATAATTC
-TCATAAAAACACAT--GTGCTCTCCCTGCTGATCATGTCTGGCTAATCTCCCAAACCCCAACC
CCTTCTACAAAACAACAACTCCTTCCTTC-GTAGGCATGGTTAGGCACTTCCACCTTTGGATA
CCTAGTTTTACCATCTTGACTAAACCATGATGTAAACTCACAAAAGGAAACCTAGCTGACCC

CACAGATCCTAAATCCTTTGACCACTCCTTTCCATTCCTTAAAAACAGCCCTAGAAGCTGCT
CCCACACTAGCTCTCCCTAACTCATCCCAATCC-TTTTTCATTACACACAGTCAAAGTGCAGG
GCTGTGAGGTCAAAATTCTTACACAAGGATCGGGACCATGCCCTGTGGCCTTTTATCCAAA
CAACTTGACCTTACTGTTTTAGCTTAG-CGTCATGTCTGTGTGTGGCAGCTGCCGCTTCCTTA
ATACTTTTAGAGGCCCTCAAAATCACAAACTATGCTCAAC---------TTACTCTCTAGAGTTCTC
ATAACTTCCAAAATCTATTTTCTTCCTCACACCTGATGCACATATTTTCTGCT---CCCCTCCAC
TACCTCTCAACAAGCCAAACTCATTGCCTTCACTCAAGCCCTCGCTCTTGCAAAAGGATTA
AGCATCAATATTTATACTGACTCTAAATATGCCTTCCATATCCTGCACCACCATGCTGTTATGT
GGGTAGAAAGAAATTTCCTCACTATGCAAGGGTCCTCCATCATTAATGCCTCTTTAATAAAA
ACTCTTCTCAAAGCTGCTTTACTTCCAAAGGAAGCTGGAGTCATTCACTGCAAGGGCCATC
AAAAGGCATCAGATCCCATTGTTCAGGACAATGCTTATGCTGATACGGTAGCTAAA-AAGCA
GCTAGCATTCCAACTTCTGTCCCTCACTGCAGTTTTTCTCCTTCTCATCTGGCCACTCCCACC
TACTCCCCCACTGAAACTTCCACCTATCAATCTCTTCCCACACAAGGCAAATGGTTCTTGGA
CCAAGGAAAATATCTCCTTCCAGCCTCACAGGCCCATTCTATTCTGTCGTCATTTCATAACCT
CTTCGATGTAGGTTACAAGCTGCTTGCCCGTCTCTTAGAACCTCTCATTTCCTTTCCACCGTG
GAAATCTATCCTCAAGGAAATCACTTCTCAG-GATTCCATCTGCTATTCTACTAATCCTCAGG
GATTGTTCAGGCCCCCTCCCTTCCCTACACATCAAGCTTGGGGATTTGCCCCTGCCCAGGAC
TGGCAAATTGACTTTACTCACATGCCCTGAGTCAGGAAACTAAAATACCTCTTGGTCTGGGT
AGACACTTTCACTGGATGGGTAGAGGCCTTTCCCACAGGGTCTGCGAAGGCCACTGTGGTC
ATTTCTTCACTTCTGTTAGGCATAATTCCTCAGTTTGACCTTCCTACCTCTATACAGTCCAATA
ATGGACCAGCCTTTGTTAGTTAAATCACCCAACCAGTTTCTCAGGCTCTTAG---TATTCAGTG
GAATTTTCATACCCCTTACCATCCTCAATCTTCAGGAAAGGTA-----GAACGGGCTAATGGTCT
TTTAAAATCACACCTCACCAAGCTCAGCCACCGACTTAAAAAAGACTAGACAATACTTTTA
CCACTTTCCCTTCTCAAAATTCGGGCCTGTCCTCGGGATGCTACAGGGTACAGCCCATTTGA
GCTTCTGTATGAACGCTCATTTTTATTAGGCCCCAGTCTTATTCCAGACACGAGCCCAACTTG
GACTGTGCCCCAAAAGCTTGTCATCCCTACTCTCTTCTGTCTAGTCATACTCCTATTCACCGT
TCTCAAATACTCATAAATGCCCTGCTCTTGTTTACACTGCCGGTTTACACTGTTTCTCCAAGC
CATCACAGCTGATGTCTCCTGGTGCTAT-CCCAAACTGCCACTCTTAACTCCCTCTTAAAGTA
AATAAATACTCTTTGCTGGCAGGGCTATGCTGAACCTCCTTGGGCACTCTCTAATTGGATGT
CCTGGATCCTCCCAATTCTCAGTCCTTTAATACCTATTTTTCTCCTTCTCCTATTCGGACCTTT
T-GTCTTCCGTTTAG-TTTTCAATTCATACAAAACTGCTTCCAGGCCATCACCAATCATTCTATA
TGATAAATGCTCCTTCTAACAACCCCACAATATCACCCCTTACCCCAAAATCTTTCTTCAGCT
TAATCTCTCCCATTCTGGGTTTCCATGCCACCCCTAATCCTGCTTGAAGCAGCCCTGAGAAA
TATCACCCATTATCTCTCCATACCATCCCCCCAAAAATTTTGC-TGCCCTAACACTTCAGTAC
TATTTTATATTATTTTTCTTATTAATATAAGAAGGCAGGAATGTCAGGCCTCTGAGCCCGAGCT
AAGCCATCATAT
>Theropithecus_gelada-HERVHF_INT(-)
---GCTAGTGAAATTTGGTGCCGTGACTCAGACTGG
GGGACCTCCCTTGGGAGATCAATCCCCTGTCCTCCTGCTCTTTGCTCCATGAGAAAGATCCA
CTTACGACCTCAGGTCCTCAGACCAACCAGCCCCAAGAACATCTCACCAATTTTAAACCGG
GTAAGCAGCCTCTCTTTACTCTCTTCTCCAACTTCTCTCACTATCCCTCAACCTCTTTCTCCT
TTCAATCTTGGTGCCACACTTCAATCTCTCCCTTCTCTTAATTTCAGTTCCTCTCCTTTTCTG
GTAGAGACAGAGGAGACGTGTTTTATCCGTGAACCCAAAACTCCTGCACCGGTCACCGACT
CGGGAAGACAGTCTTCCCTTGGTGTTTAATCCCTGCAGGGACGTCTGCCTGATTATTCACCC

ACATTTCATTGGTGTCTGATCACCACAGGGATGCCTGCCTTCGTCATTCACCCACAGTCCCT
TGGTGGCAAGTCCATTGCAGGGACACCTGCTTTGGCTGCTCACCCACATTGCAGCCCAGGG
CTGCTCACCCCAC---CCCTTCTCTCCGTGTC
TCTGCCCTCTCTTCTCTCCA---------------------------------------CTTTCTTGGGGGGCTAGCACCCC
CCACCC----------CTTCTCTCTGTGTCTCTACCC---TCTC
TTTTCTCTGGACTTGCCTCCTTCATTATGGGCAAACTTCCACACCCTCCATTCCTCCTTCTTC
TCCCTTAGCCTGTGTTCTCAAGAACTTAAAACTTCTTCGACTCACAACTGACCTAAAGCCTA
AATGCCTTATTTCTTCTGCAACACCGCTTGGCCCC--AATACAAACTCGAC-AATGGTTCCAA
ATAGCCAGAAAACGGCACTTTCGATTTCTCCATCCTACAAGATCTAGATAATTCTCGTCATAA
AATGGGCAAATAGTCTGAGGTGCCTGATGTCCAGGCATTCTTTTACGCATGAGTCCCTCCCT
GGTCTTTGTTCCCAATGCAACTCGTCCC---AAATCTTCCTT
CTTTCCCTCCCGCCTGTCCCCTCAGTCCCAACCCGAAGAGTTGCTGAGTCTTTCTAA--TCTT
CCTTTTTTACGGACCCATCTGACCTCTCCCCTCCTCCCCAGGCTGCTCCTCACCAGGCCAAG
CCAGGTCCCAA-TTCTTCCTCAGCCTCCGTTCCCCCACCCTATAATCCTTTTATCACCTCCCCT
CCTCACACCCAGTCCGGCTTGCAGTTTCGTTTTGCAACTAGCCCTCCCTCACCTGCCCAGC
AATTATCTCTTAAAAATGTGG-------CTGGAGCCGAAGGCATAGTCAGGGTTAATGCTCCTTTT
TCTTTATCTGGCCTCTCCCAAATCAGTTAGCGTTTAGGCTCTTTTTCATCAAATATAAAAACC
CGGCCCAGTTCATGGCTTGTTTGGCAGCAACCCTGAGATGCTTTACTGCCCTAGACCCTGA
AAGGTCAAAAGGCCGTCTTATTCTCAATATACAT---TGTATTACCCAATCCACTCCTGACATTA
AATAAAACACCAAAAATTAAGTTCCGGCCCTCAAACCCCACAACAAGACTTAATTAACCTT
GCCTTCAAGGTGTACAATAATAGAGAAGAGTTGCAATTACTTGCCTCCGCTGTGAGAGAAA
CCCCAGCCACATCTTCAGCACACAAGAACTTCAAAATGCCTAAGCCACAGCAGCCAGGCG
TTCCTTCAGGACTTTCTCCCCCAGGATCTTGCTTCAAGTGCTGGAAATCTGGCCACTGGGCC
AAGGAATGCCCGTAGCCCAGGATTCC-TCCTAAACTGTGTCCCATCTGTGCAGGACCCCACT
GGAAATTGGACTGTCCAACTGGCCCAAGGCTCTAACTGACTCCTTCCCAGATCTTCTCAGC
TTA--GGCTGAAGACTGATGCTGCCCGATCACCTTGGAAGCTTCCTGGACCATCACAGACGC
TTTGGGTAACTCTTACAGTGGAGGGTAAGTCCATCCCCTTCTTAATCAATATGGAGGCTACC
CACTCCACATTACCTTCTTTTCAAGGGCCTGTTTCCCTTGCCTCCATAAATGTTGTGGGCATT
GACAGCCAGGTTTCTAAACCCCTTAAAACTCCCCCAGTCTGGTGCTAACTTGGACAACATT
CTTTTATGCACTCTTTTTCAGTTATCCCCACCTGCCCAGTTCCCTTATTAGGCCGAGATATTTT
AACCAAATTGTCTGCTTCCCTGACTATTCCTGGACTACAGCCACATCTCATTGCTACCCTGCT
TCCCAACCCAAAGCCTCCTTCGCGCCTTCCTCTCGTATTCCCCAAACTTAACCCACGTGTAT
GGGACACCTCTA---CTCCCTCCCTGGCAACCAATCACCAGCCTATTACTATCCCATTAAAACC
TAATCACCCTTACCCCATTCAATGCCAGTATCCCATCCCACAACAGGCTTTAAAGGGATTGA
AACCTGTTATCACTTGCCTGCTACAGTATGGGCTTCTAAAACCTGTAAACTCCCCTTACAATT
CCCCCATTTTACCTGTCCAAAAACTGGACAAGTCTTACACGTTACTTCAGGATCTGTGCCTT
ATCAACCAAATTGTTTTGCCTAT---CCTTGTGGTGCAACACCCATTTACTCTCCTATCCTCAAT
ACCTCCCTCCACAACCCATTATTCTGTTCTGGATCTCAAACATGCTTTCTTTACTATTCCTTTG
CATCCTTCATCCCAGCTTCTCTTTGCTTTCACTTGGACTGACCCTGACACCCATCAGGCTCA
GGAAATTACCTGGGCTGTACTGCTGCAAGGTTTCACGGACAGCCCCCATTACTTTGGTCAA
GCCCAAATTTCTTCCTCATCCATTACCTATCTTGGCATAATTCTTCATGAAAACATAC--ATGCT
CTCCCTGCTGATCATGTCTGGCTAATCTCCCAAACCCCAACCCCTTCTACAAAACAACAACT
CCTTCCTTC-GTAGGCATGGTTAGCTACTTCCACCTTTGGATACCTAGTTTTACCATCTTGACT
AAACCACAATGTAAACTCACAAAAGGAAACCTAGCTGACCCCACAGATCCTAAATCCTTTT

GCCACTCCTTTCCATTCCTTAAAAACAGCCCTAGAAGCTGCTCCCACACTAGCTCTCCCTAA
CTCATCCCAATCC-TTTTTCATTACACACAGTCAAAGTGCAGGGCTGTGAGGTCAAAATTCTT
ACACAAGGATCGGGACCATGCCCTGTGGCCTTTTTATCCAAACAATTTGACCTTACTCTTTT
AGCTTAG-CCTCATGTCTGTGTGTGGCAGCTGCTGCTTCCTTAATACTTTCAGAGGCCCTCAA
AATCACAAACTATGCTCAAC---------TCACTCTCTAGAGTTCTCATAACTTTCAAAATCTATTT
TCTTCCTCACACCTGATGCACATATTTCCTGCC---CCCCTCCACTACCTCTCAACAAGCCAAA
CTCATTGCCTTAACTCAAGCCCTCACTCTTGCAAAAGGATTAAGTGTCAATATTTATACTGAC
TCTAAATATGCCTTCCATATCCTGCACCACCATGCTGTTATATGGGCAGAAAGAAATGTCCTA
ACTATGCAAGGGTCCTCCATCATTAATGCCTCTTTAATAAAAACTCTTCTCAAAGCTGCTTTA
CTTCCAAAGGAAGCTGGAATCATTCACTGCAAGGGCCAGCAAAAGGCATCAGATCCCATTG
CTCAGGACAATGCTTATGCTGATACGGTAGCTAAA-AAGCAGCTAGCATTCCAACTTCTATCC
CTCATGGCAGTTTTTCTCCTTCGCATTTGGCCACTCCCACCTACCCCCCCACTGAAACTTCC
ACCTATCAGTCTCTTCCCACGCAAGGCAAATGGTTCTTGGACCAAGGAAAATATCTCCTTCC
AGCCTCACAGGCCCATTCTATTCTGTCATCATTTCATAACCTCTTCCATGTAGGTTACAAGCT
GCTAGCCCATCTCTTAGAACCTCTCATTTCCTTTCCATTGTGGAGATCTATCCTCAAGGAAAT
CACTTCTCAG-TGTTCCATCTGCTATTCTACTACTCCTCAGGGATTGTTCAGGCCCCCTCCCT
TCCCTACACATCAAGCTCGGTGATTTGCCCCTGCCCAGGACTGGCAAATTGACTTTACTCAC
ATGCCCCAAGTCAGGAAACTAAAATACCTCTTGGTCTAGGTAGACACTTTCACTGCACAGG
TAGAGGCCTTTCCCACAGGGTCTGCGAAGGCCACCATGGTCATTTCTTCCCTTCTGTCAGGT
ATAATTCCTCGGTTTGACCTTCCTACCTCTATACAGTCCAATAATGGACCAGCCTTTGTTAGT
TAAATCACCCAACCAGTTTCTCAGGCTCTTAG---TATTCAGTGGAACTTTCATACCCCTTACT
ATCCTCAGTCTTCAGGAAAGGTA----GGAACGGGCTAATGGTCTTTTAAAAACACACCTCAC
CAATCTCAGCCACCAACTTAAAAAGGACTAGACAATACTTTTACCACTTTCCCTTCTCAAAA
TTCGGGCCTGTCCTCGGGATGCTACAGGGTACAGCCCATTTGAGCTTCTGTATGAACCCTCC
TATTTATTAGGCCCCAGTCTTATTCCAGACACCAGCCCAACTTGGACTGTGCCCCAAAAACT
TGTCATCCCTACTCTCTTCTGTCTAGTCATACTCCTATTCACCGTTCTCAAATACTCATAAATG
CCCTGCTCTTGTTTACACTGCCGGTTTACACTGTTTCTCCAAGCCATCACAGCTGATATCTCC
TGGTGCTAT-CCCAAACTGCCACTCTTAACTCCCTCTTAAAGTAAATAAATAATCTTTGCTGG
CAGGGCTATGCTGAACCTCCTTGGGCACTCTCTAATTGGATGTCCTGGATCCTCCCAATTCTT
AGTCCTTTAATACCTGTTTTTCTCCTTCTCTTATTCAGACCTTTT-GTCTTCCGTTTAGTTTTTC
AATTCATACAAAACTGCTTCCAGGCCATCACCAATCATTCTATATGATAAATGCTCCTTCTAA
CAACCCCACAATATCACCCCTTATCACAAAATCTTCCTTCAGCTTAATCTCTCCCACTCTGGG
TTTCCATGCCACCCCTAATCCTGCATGAAGCAGCCCTGAGAAACATCACCCATTATCTCTGC
ATGCCACCCCCCAAAAAATTTCTGC-TGCCCCAACACTTCAATACTATTTTGTATTATTTTTCT
TATTAATATAAGAAGGCAG------------------------------------

Supplementary data S7 The LTRs' blastn results of possible HERVs which were involved in host gene recombination

Query ID qLTR-type Subject ID sLTR-type % identity alignment length mismatches gap opens q. start q. end s. start s. end evalue bit score qcovhsp

Cercocebus_atys_KQ010004.1:3853153-3859140_HERVHF 3LTR Cercocebus_atys_KQ010004.1:8290204-8294490_HERVHF 3LTR 90.448 335 27 4 15 347 10 341 8.37E-129447 96

Cercocebus_atys_KQ010004.1:3853153-3859140_HERVHF 5LTR Cercocebus_atys_KQ010004.1:8290204-8294490_HERVHF 5LTR 90.909 341 26 5 5 340 3 343 4.53E-132458 99

Cercocebus_atys_KQ010004.1:3853153-3859140_HERVHF 3LTR Cercocebus_atys_KQ012885.1:26400757-26405972_HERVHF 3LTR 92.059 340 24 2 10 347 39 377 9.54E-141486 97

Cercocebus_atys_KQ010004.1:3853153-3859140_HERVHF 5LTR Cercocebus_atys_KQ012885.1:26400757-26405972_HERVHF 5LTR 89.796 343 32 3 1 340 29 371 6.72E-130451 100

Chlorocebus_aethiops.fasta_ctg370:1321412-1326733_HERVK 5LTR Chlorocebus_aethiops.fasta_ctg4:24809931-24814277_HERVK 3LTR 87.983 466 56 0 1 466 49 514 3.75E-172589 100

Chlorocebus_aethiops.fasta_ctg370:1321412-1326733_HERVK 3LTR Chlorocebus_aethiops.fasta_ctg4:24809931-24814277_HERVK 5LTR 89.009 464 48 2 1 462 44 506 5.96E-176601 99

Chlorocebus_sabaeus_CM001959.2:45511215-45516073_HERVHF 3LTR Chlorocebus_sabaeus_CM001962.2:64631446-64636282_HERVHF 3LTR 93.679 443 24 3 1 440 13 454 0 664 100

Chlorocebus_sabaeus_CM001959.2:45511215-45516073_HERVHF 5LTR Chlorocebus_sabaeus_CM001962.2:64631446-64636282_HERVHF 5LTR 92.07 454 35 1 1 453 1 454 0 654 99

Gorilla_gorilla_gorilla_chr1:202016017-202021023_HERVHF 5LTR Gorilla_gorilla_gorilla_chr13:36959576-36964653_HERVHF 3LTR 96.561 378 12 1 1 377 20 397 2.88E-180620 97

Gorilla_gorilla_gorilla_chr1:202016017-202021023_HERVHF 3LTR Gorilla_gorilla_gorilla_chr13:36959576-36964653_HERVHF 5LTR 96.505 372 13 0 1 372 19 390 4.08E-178613 100

Gorilla_gorilla_gorilla_chr10:48560137-48563743_HERVHF 5LTR Gorilla_gorilla_gorilla_chr16:50623460-50628109_HERVHF 3LTR 95.086 407 20 0 1 407 12 418 0 645 100

Gorilla_gorilla_gorilla_chr10:48560137-48563743_HERVHF 3LTR Gorilla_gorilla_gorilla_chr16:50623460-50628109_HERVHF 5LTR 94.749 419 18 2 2 416 1 419 0 652 99

Gorilla_gorilla_gorilla_chr20:12290537-12295740_HERVHF 5LTR

Gorilla_gorilla_gorilla_chr4:179641013-179645038_HERVHF 3LTR 96.296 324 11 1 7 329 1 324 1.05E-152527 98
Gorilla_gorilla_gorilla_chr20:12290537-12295740_HERVHF 3LTR
Gorilla_gorilla_gorilla_chr4:179641013-179645038_HERVHF 5LTR 97.742 310 5 2 9 318 1 308 1.53E-150521 96
Gorilla_gorilla_gorilla_chr20:12290537-12295740_HERVHF 3LTR
Gorilla_gorilla_gorilla_chr5:81531035-81536265_HERVHF 3LTR 97.152 316 7 2 3 318 8 321 4.37E-151523 98
Gorilla_gorilla_gorilla_chr20:12290537-12295740_HERVHF 5LTR
Gorilla_gorilla_gorilla_chr5:81531035-81536265_HERVHF 5LTR 97.273 330 8 1 1 329 1 330 9.22E-160552 100
Gorilla_gorilla_gorilla_chr2b:110918228-110923107_HERVHF 3LTR
Gorilla_gorilla_gorilla_chr3:8235871-8240734_HERVHF 3LTR 93.932 412 24 1 14 425 1 411 0 627 97
Gorilla_gorilla_gorilla_chr2b:110918228-110923107_HERVHF 5LTR
Gorilla_gorilla_gorilla_chr3:8235871-8240734_HERVHF 5LTR 93.447 412 26 1 2 412 1 412 9.57E-180618 99
Gorilla_gorilla_gorilla_chr3:21293800-21298167_HERVHF 5LTR
Gorilla_gorilla_gorilla_chr3:8235871-8240734_HERVHF 3LTR 90.821 414 24 4 4 407 1 410 1.23E-165571 99
Gorilla_gorilla_gorilla_chr3:21293800-21298167_HERVHF 3LTR
Gorilla_gorilla_gorilla_chr3:8235871-8240734_HERVHF 5LTR 91.198 409 24 2 6 403 4 411 8.21E-168578 99
Gorilla_gorilla_gorilla_chr3:46413847-46418893_HERVHF 5LTR
Gorilla_gorilla_gorilla_chr4:75982275-75987316_HERVHF 3LTR 95.768 449 18 1 6 454 1 448 0 721 98
Gorilla_gorilla_gorilla_chr3:46413847-46418893_HERVHF 3LTR
Gorilla_gorilla_gorilla_chr4:75982275-75987316_HERVHF 5LTR 95.806 453 18 1 3 454 1 453 0 728 99
Gorilla_gorilla_gorilla_chr3:78395594-78400678_HERVHF 3LTR
Gorilla_gorilla_gorilla_chr4:75982275-75987316_HERVHF 3LTR 94.457 451 24 1 1 451 2 451 0 698 99
Gorilla_gorilla_gorilla_chr3:78395594-78400678_HERVHF 5LTR
Gorilla_gorilla_gorilla_chr4:75982275-75987316_HERVHF 5LTR 93.805 452 27 1 1 451 2 453 0 686 100
Gorilla_gorilla_gorilla_chr3:78395594-78400678_HERVHF 5LTR
Gorilla_gorilla_gorilla_chr4:8487186-8492546_HERVHF 3LTR 94.9 451 22 1 1 451 2 451 0 707 100
Gorilla_gorilla_gorilla_chr3:78395594-78400678_HERVHF 3LTR
Gorilla_gorilla_gorilla_chr4:8487186-8492546_HERVHF 5LTR 93.57 451 29 0 1 451 2 452 0 683 99
Gorilla_gorilla_gorilla_chr3:78395594-78400678_HERVHF 3LTR
Gorilla_gorilla_gorilla_chr4:88917361-88922424_HERVHF 3LTR 93.584 452 29 0 1 452 1 452 0 685 100

Gorilla_gorilla_gorilla_chr3:78395594-78400678_HERVHF 5LTR
Gorilla_gorilla_gorilla_chr4:88917361-88922424_HERVHF 5LTR 93.556 450 27 1
2 451 1 448 0 679 99
Gorilla_gorilla_gorilla_chr6:110555930-110560849_HERVHF 3LTR
Gorilla_gorilla_gorilla_chr6:126863433-126868223_HERVHF 3LTR 94.053 454 27
0 5 458 1 454 0 698 99
Gorilla_gorilla_gorilla_chr6:110555930-110560849_HERVHF 5LTR
Gorilla_gorilla_gorilla_chr6:126863433-126868223_HERVHF 5LTR 94.26 453 26
0 6 458 1 453 0 700 99
Gorilla_gorilla_gorilla_chr6:110555930-110560849_HERVHF 3LTR
Gorilla_gorilla_gorilla_chr9:95243879-95249741_HERVHF 3LTR 94.493 454 24 1
6 459 1 453 0 703 99
Gorilla_gorilla_gorilla_chr6:110555930-110560849_HERVHF 5LTR
Gorilla_gorilla_gorilla_chr9:95243879-95249741_HERVHF 5LTR 95.585 453 19 1
6 458 1 452 0 724 99
Gorilla_gorilla_gorilla_chr8:110324395-110329380_HERVHF 5LTR
Gorilla_gorilla_gorilla_chr9:95243879-95249741_HERVHF 3LTR 95.364 453 18 2
24 474 1 452 0 717 95
Gorilla_gorilla_gorilla_chr8:110324395-110329380_HERVHF 3LTR
Gorilla_gorilla_gorilla_chr9:95243879-95249741_HERVHF 5LTR 95.575 452 17 2
17 466 1 451 0 719 97
Gorilla_gorilla_gorilla_chr9:48707643-48713261_HERVHF 3LTR
Gorilla_gorilla_gorilla_chrX:110023142-110028702_HERVHF 3LTR 94.581 406 21
1 1 405 1 406 0 630 100
Gorilla_gorilla_gorilla_chr9:48707643-48713261_HERVHF 5LTR
Gorilla_gorilla_gorilla_chrX:110023142-110028702_HERVHF 5LTR 94.686 414 21
1 1 413 1 414 0 645 100
Gorilla_gorilla_gorilla_chr9:53509489-53514564_HERVHF 3LTR
Gorilla_gorilla_gorilla_chr9:95243879-95249741_HERVHF 3LTR 95.362 345 15 1
1 345 2 345 1.09E-158547 100
Gorilla_gorilla_gorilla_chr9:53509489-53514564_HERVHF 5LTR
Gorilla_gorilla_gorilla_chr9:95243879-95249741_HERVHF 5LTR 94.169 343 19 1
1 343 4 345 3.56E-152526 99
Gorilla_gorilla_gorilla_chr9:53509489-53514564_HERVHF 3LTR
Gorilla_gorilla_gorilla_chrX:81105621-81110902_HERVHF 3LTR 94.509 346 18 1
1 345 1 346 6.89E-155535 100
Gorilla_gorilla_gorilla_chr9:53509489-53514564_HERVHF 5LTR
Gorilla_gorilla_gorilla_chrX:81105621-81110902_HERVHF 5LTR 93.86 342 21 0
1 342 3 344 4.34E-151523 99
Gorilla_gorilla_gorilla_chr9:53509489-53514564_HERVHF 5LTR
Gorilla_gorilla_gorilla_chrX:88109970-88115344_HERVHF 3LTR 93.878 343 20 1
1 343 4 345 1.51E-150521 99
Gorilla_gorilla_gorilla_chr9:53509489-53514564_HERVHF 3LTR
Gorilla_gorilla_gorilla_chrX:88109970-88115344_HERVHF 5LTR 94.22 346 19 1

1 345 2 347 8.4E-154 531 100
Gorilla_gorilla_gorilla_chr9:60838551-60843495_HERVHF 3LTR
Gorilla_gorilla_gorilla_chr9:95243879-95249741_HERVHF 3LTR 94.69 452 23 1
1 452 2 452 0 704 99
Gorilla_gorilla_gorilla_chr9:60838551-60843495_HERVHF 5LTR
Gorilla_gorilla_gorilla_chr9:95243879-95249741_HERVHF 5LTR 95.333 450 19 2
1 449 4 452 0 710 99
Gorilla_gorilla_gorilla_chrX:107821824-107827046_HERVHF 3LTR
Gorilla_gorilla_gorilla_chrX:107950953-107956083_HERVHF 3LTR 96.026 302 11
1 2 303 76 376 7.85E-141488 99
Gorilla_gorilla_gorilla_chrX:107821824-107827046_HERVHF 5LTR
Gorilla_gorilla_gorilla_chrX:107950953-107956083_HERVHF 5LTR 95.752 306 9
3 1 302 90 395 9.5E-140 484 100
Homo_sapiens_1:5045144-5052648_HERVHF 5LTR
Homo_sapiens_13:51170315-51174556_HERVHF 3LTR 96.131 336 13 0 1 336
13 348 3.9E-158 548 100
Homo_sapiens_1:5045144-5052648_HERVHF 3LTR
Homo_sapiens_13:51170315-51174556_HERVHF 5LTR 94.857 350 18 0 3 352
1 350 3.36E-159551 99
Homo_sapiens_1:5045144-5052648_HERVHF 3LTR
Homo_sapiens_16:60078989-60084128_HERVHF 3LTR 94.571 350 19 0 3 352
1 350 1.43E-157546 99
Homo_sapiens_1:5045144-5052648_HERVHF 5LTR
Homo_sapiens_16:60078989-60084128_HERVHF 5LTR 96.131 336 13 0 1 336
12 347 3.9E-158 548 100
Homo_sapiens_1:5045144-5052648_HERVHF 3LTR
Homo_sapiens_19:53831857-53836889_HERVHF 3LTR 95.455 352 16 0 1 352
8 359 5.33E-163563 100
Homo_sapiens_1:5045144-5052648_HERVHF 5LTR
Homo_sapiens_19:53831857-53836889_HERVHF 5LTR 96.726 336 11 0 1 336
13 348 7.52E-161557 100
Homo_sapiens_1:5045144-5052648_HERVHF 5LTR
Homo_sapiens_2:215922762-215927674_HERVHF3LTR 95.858 338 12 1 1 336
13 350 4.75E-157545 100
Homo_sapiens_1:5045144-5052648_HERVHF 3LTR
Homo_sapiens_2:215922762-215927674_HERVHF5LTR 94.886 352 16 1 3 352
1 352 3.36E-159552 99
Homo_sapiens_1:5045144-5052648_HERVHF 5LTR
Homo_sapiens_4:125562071-125567006_HERVHF3LTR 96.429 336 12 0 1 336
15 350 9.17E-160553 100
Homo_sapiens_1:5045144-5052648_HERVHF 3LTR
Homo_sapiens_4:125562071-125567006_HERVHF5LTR 95.739 352 15 0 1 352
1 352 4.37E-164568 100
Homo_sapiens_1:5045144-5052648_HERVHF 3LTR

Homo_sapiens_6:90670654-90675059_HERVHF 3LTR 94.602 352 19 0 1 352
7 358 1.17E-158550 100
Homo_sapiens_1:5045144-5052648_HERVHF 5LTR
Homo_sapiens_6:90670654-90675059_HERVHF 5LTR 97.024 336 10 0 1 336
17 352 1.77E-162562 100
Homo_sapiens_1:5045144-5052648_HERVHF 3LTR
Homo_sapiens_9:121790452-121796319_HERVHF3LTR 96.011 351 13 1 2 352
1 350 4.37E-164567 99
Homo_sapiens_1:5045144-5052648_HERVHF 5LTR
Homo_sapiens_9:121790452-121796319_HERVHF5LTR 97.024 336 8 2 1 336
14 347 2.63E-160554 100
Homo_sapiens_1:5045144-5052648_HERVHF 5LTR
Homo_sapiens_X:87682131-87687194_HERVHF 3LTR 96.429 336 12 0 1 336
13 348 9.17E-160553 100
Homo_sapiens_1:5045144-5052648_HERVHF 3LTR
Homo_sapiens_X:87682131-87687194_HERVHF 5LTR 95.143 350 16 1 3 352
1 349 3.36E-159552 99
Homo_sapiens_1:99509965-99515002_HERVHF 3LTR
Homo_sapiens_10:53493177-53504276_HERVHF 3LTR 95.109 368 12 3 1 362
82 449 3.03E-166575 100
Homo_sapiens_1:99509965-99515002_HERVHF 5LTR
Homo_sapiens_10:53493177-53504276_HERVHF 5LTR 95.946 370 13 2 1 370
88 455 1.16E-171593 99
Homo_sapiens_1:99509965-99515002_HERVHF 5LTR
Homo_sapiens_10:6797535-6802501_HERVHF 3LTR 94.609 371 18 2 1 371
85 453 1.09E-165572 99
Homo_sapiens_1:99509965-99515002_HERVHF 3LTR
Homo_sapiens_10:6797535-6802501_HERVHF 5LTR 94.837 368 13 3 1 362
83 450 3.7E-165 571 100
Homo_sapiens_1:99509965-99515002_HERVHF 5LTR
Homo_sapiens_16:60078989-60084128_HERVHF 3LTR 95.699 372 14 2 1 372
83 452 1.16E-171592 100
Homo_sapiens_1:99509965-99515002_HERVHF 3LTR
Homo_sapiens_16:60078989-60084128_HERVHF 5LTR 95.38 368 11 3 1 362
79 446 7.13E-168580 100
Homo_sapiens_1:99509965-99515002_HERVHF 3LTR
Homo_sapiens_20:12754276-12759162_HERVHF 3LTR 95.652 368 10 3 1 362
102 469 5.86E-169584 100
Homo_sapiens_1:99509965-99515002_HERVHF 5LTR
Homo_sapiens_20:12754276-12759162_HERVHF 5LTR 94.624 372 18 2 1 372
105 474 3.12E-166574 100
Homo_sapiens_1:99509965-99515002_HERVHF 3LTR
Homo_sapiens_3:112418865-112422911_HERVHF3LTR 95.393 369 10 4 1 362
82 450 2.49E-167578 100

Homo_sapiens_1:99509965-99515002_HERVHF 5LTR
Homo_sapiens_3:112418865-112422911_HERVHF 5LTR 94.609 371 18 2 1 371
87 455 1.09E-165572 99
Homo_sapiens_1:99509965-99515002_HERVHF 5LTR
Homo_sapiens_6:125702300-125707310_HERVHF3LTR 94.624 372 15 3 1 372
87 453 1.09E-165572 100
Homo_sapiens_1:99509965-99515002_HERVHF 3LTR
Homo_sapiens_6:125702300-125707310_HERVHF5LTR 94.837 368 13 3 1 362
83 450 3.7E-165 571 100
Homo_sapiens_1:99509965-99515002_HERVHF 5LTR
Homo_sapiens_9:99670236-99675451_HERVHF 3LTR 95.43 372 15 2 1 372
83 452 4.95E-170588 100
Homo_sapiens_1:99509965-99515002_HERVHF 3LTR
Homo_sapiens_9:99670236-99675451_HERVHF 5LTR 95.109 368 12 3 1 362
80 447 3.03E-166575 100
Homo_sapiens_1:99509965-99515002_HERVHF 3LTR
Homo_sapiens_X:92824882-92828895_HERVHF 3LTR 94.837 368 13 3 1 362
80 447 3.7E-165 571 100
Homo_sapiens_1:99509965-99515002_HERVHF 5LTR
Homo_sapiens_X:92824882-92828895_HERVHF 5LTR 95.722 374 12 3 1 372
83 454 1.16E-171593 100
Homo_sapiens_10:6629975-6635084_HERVHF 3LTR
Homo_sapiens_10:6797535-6802501_HERVHF 3LTR 95.11 409 18 1 1 407
45 453 0 645 98
Homo_sapiens_10:6629975-6635084_HERVHF 5LTR
Homo_sapiens_10:6797535-6802501_HERVHF 5LTR 98.241 398 7 0 1 398
57 454 0 687 99
Homo_sapiens_10:6629975-6635084_HERVHF 5LTR
Homo_sapiens_16:60078989-60084128_HERVHF 3LTR 97.243 399 11 0 1 399
54 452 0 671 99
Homo_sapiens_10:6629975-6635084_HERVHF 3LTR
Homo_sapiens_16:60078989-60084128_HERVHF 5LTR 95.146 412 18 1 1 410
42 453 0 651 99
Homo_sapiens_10:6629975-6635084_HERVHF 5LTR
Homo_sapiens_6:125702300-125707310_HERVHF3LTR 96.992 399 9 1 1 399
58 453 0 664 99
Homo_sapiens_10:6629975-6635084_HERVHF 3LTR
Homo_sapiens_6:125702300-125707310_HERVHF5LTR 95.11 409 18 1 1 407
46 454 0 645 98
Homo_sapiens_10:96733309-96736169_HERVHF 3LTR
Homo_sapiens_6:144923609-144928414_HERVHF3LTR 96.865 319 10 0 6 324
1 319 2.88E-153531 98
Homo_sapiens_10:96733309-96736169_HERVHF 5LTR
Homo_sapiens_6:144923609-144928414_HERVHF5LTR 97.394 307 8 0 17 323

9 315 1.81E-149518 95

Homo_sapiens_11:23887605-23892534_HERVHF 3LTR

Homo_sapiens_2:224225730-224230587_HERVHF3LTR 93.939 330 16 2 1 326

57 386 4.98E-144500 99

Homo_sapiens_11:23887605-23892534_HERVHF 5LTR

Homo_sapiens_2:224225730-224230587_HERVHF5LTR 93.06 317 16 3 18 331

73 386 3.6E-133 465 95

Homo_sapiens_11:23887605-23892534_HERVHF 3LTR

Homo_sapiens_2:67331916-67337203_HERVHF 3LTR 94.611 334 13 3 1 330

57 389 7.89E-148513 100

Homo_sapiens_11:23887605-23892534_HERVHF 5LTR

Homo_sapiens_2:67331916-67337203_HERVHF 5LTR 93.651 315 16 3 18 330

73 385 2.95E-134469 95

Homo_sapiens_11:23887605-23892534_HERVHF 5LTR

Homo_sapiens_8:131741164-131746225_HERVHF3LTR 93.038 316 18 3 18 331

73 386 4.38E-132462 95

Homo_sapiens_11:23887605-23892534_HERVHF 3LTR

Homo_sapiens_8:131741164-131746225_HERVHF5LTR 91.94 335 18 5 1 330

62 392 2.94E-134469 100

Homo_sapiens_12:11462625-11467569_HERVHF 3LTR

Homo_sapiens_2:215922762-215927674_HERVHF3LTR 95.364 453 18 2 4 453

1 453 0 717 99

Homo_sapiens_12:11462625-11467569_HERVHF 5LTR

Homo_sapiens_2:215922762-215927674_HERVHF5LTR 96.018 452 16 1 8 457

1 452 0 732 98

Homo_sapiens_12:11462625-11467569_HERVHF 5LTR

Homo_sapiens_6:125702300-125707310_HERVHF3LTR 95.585 453 15 2 7 457

2 451 0 723 99

Homo_sapiens_12:11462625-11467569_HERVHF 3LTR

Homo_sapiens_6:125702300-125707310_HERVHF5LTR 95.796 452 18 1 3 453

3 454 0 727 99

Homo_sapiens_12:72703674-72708522_HERVHF 5LTR

Homo_sapiens_16:60078989-60084128_HERVHF 3LTR 95.575 452 20 0 10 461

1 452 0 726 98

Homo_sapiens_12:72703674-72708522_HERVHF 3LTR

Homo_sapiens_16:60078989-60084128_HERVHF 5LTR 95.982 448 15 3 2 446

1 448 0 717 99

Homo_sapiens_13:36316772-36321823_HERVHF 3LTR

Homo_sapiens_13:51170315-51174556_HERVHF 3LTR 96.739 368 12 0 1 368

83 450 4.29E-177610 100

Homo_sapiens_13:36316772-36321823_HERVHF 5LTR

Homo_sapiens_13:51170315-51174556_HERVHF 5LTR 95.676 370 13 1 1 367

80 449 3.3E-172 594 99

Homo_sapiens_13:36316772-36321823_HERVHF 3LTR

Homo_sapiens_16:60078989-60084128_HERVHF 3LTR 96.196 368 14 0 1 368
83 450 2.22E-174601 100
Homo_sapiens_13:36316772-36321823_HERVHF 5LTR
Homo_sapiens_16:60078989-60084128_HERVHF 5LTR 94.906 373 14 2 1 368
79 451 5.96E-169583 100
Homo_sapiens_13:36316772-36321823_HERVHF 5LTR
Homo_sapiens_19:53831857-53836889_HERVHF 3LTR 94.638 373 15 2 1 368
89 461 2.53E-167579 100
Homo_sapiens_13:36316772-36321823_HERVHF 3LTR
Homo_sapiens_19:53831857-53836889_HERVHF 5LTR 95.924 368 15 0 1 368
83 450 9.44E-173597 100
Homo_sapiens_13:36316772-36321823_HERVHF 5LTR
Homo_sapiens_2:215922762-215927674_HERVHF3LTR 95.174 373 13 2 1 368
82 454 4.89E-170588 100
Homo_sapiens_13:36316772-36321823_HERVHF 3LTR
Homo_sapiens_2:215922762-215927674_HERVHF5LTR 95.652 368 16 0 1 368
85 452 1.15E-171592 100
Homo_sapiens_13:36316772-36321823_HERVHF 3LTR
Homo_sapiens_2:71086810-71090545_HERVHF 3LTR 95.38 368 17 0 1 368
85 452 4.89E-170588 100
Homo_sapiens_13:36316772-36321823_HERVHF 5LTR
Homo_sapiens_2:71086810-71090545_HERVHF 5LTR 94.865 370 16 1 1 367
83 452 7.26E-168581 99
Homo_sapiens_13:36316772-36321823_HERVHF 3LTR
Homo_sapiens_2:76574075-76579252_HERVHF 3LTR 94.565 368 20 0 1 368
84 451 3.09E-166574 100
Homo_sapiens_13:36316772-36321823_HERVHF 5LTR
Homo_sapiens_2:76574075-76579252_HERVHF 5LTR 94.624 372 15 2 1 367
81 452 8.84E-167577 99
Homo_sapiens_13:36316772-36321823_HERVHF 5LTR
Homo_sapiens_20:12754276-12759162_HERVHF 3LTR 95.393 369 12 2 1 364
102 470 1.71E-169585 99
Homo_sapiens_13:36316772-36321823_HERVHF 3LTR
Homo_sapiens_20:12754276-12759162_HERVHF 5LTR 95.924 368 15 0 1 368
105 472 9.44E-173597 100
Homo_sapiens_13:36316772-36321823_HERVHF 5LTR
Homo_sapiens_3:112418865-112422911_HERVHF 3LTR 94.385 374 15 3 1 368
82 455 1.08E-165572 100
Homo_sapiens_13:36316772-36321823_HERVHF 3LTR
Homo_sapiens_3:112418865-112422911_HERVHF 5LTR 95.924 368 15 0 1 368
87 454 9.44E-173597 100
Homo_sapiens_13:36316772-36321823_HERVHF 3LTR
Homo_sapiens_6:125702300-125707310_HERVHF3LTR 95.924 368 12 1 1 368
87 451 3.3E-172 595 100

Homo_sapiens_13:36316772-36321823_HERVHF 5LTR
Homo_sapiens_6:125702300-125707310_HERVHF5LTR 95.43 372 12 2 1 367
83 454 4.01E-171590 99
Homo_sapiens_13:36316772-36321823_HERVHF 5LTR
Homo_sapiens_8:97201222-97206202_HERVHF 3LTR 94.638 373 15 2 1 368
84 456 2.53E-167579 100
Homo_sapiens_13:36316772-36321823_HERVHF 3LTR
Homo_sapiens_8:97201222-97206202_HERVHF 5LTR 95.652 368 16 0 1 368
84 451 1.15E-171592 100
Homo_sapiens_13:36316772-36321823_HERVHF 5LTR
Homo_sapiens_9:99670236-99675451_HERVHF 3LTR 94.37 373 16 2 1 368
80 452 3.09E-166574 100
Homo_sapiens_13:36316772-36321823_HERVHF 3LTR
Homo_sapiens_9:99670236-99675451_HERVHF 5LTR 95.924 368 15 0 1 368
83 450 9.44E-173597 100
Homo_sapiens_13:36316772-36321823_HERVHF 5LTR
Homo_sapiens_X:87682131-87687194_HERVHF 3LTR 94.638 373 15 2 1 368
80 452 2.53E-167579 100
Homo_sapiens_13:36316772-36321823_HERVHF 3LTR
Homo_sapiens_X:87682131-87687194_HERVHF 5LTR 95.924 368 15 0 1 368
82 449 9.44E-173597 100
Homo_sapiens_13:36316772-36321823_HERVHF 5LTR
Homo_sapiens_X:92824882-92828895_HERVHF 3LTR 94.906 373 14 2 1 368
80 452 5.96E-169583 100
Homo_sapiens_13:36316772-36321823_HERVHF 3LTR
Homo_sapiens_X:92824882-92828895_HERVHF 5LTR 96.486 370 11 1 1 368
83 452 5.22E-176607 100
Homo_sapiens_13:86358624-86363686_HERVHF 3LTR
Homo_sapiens_16:60078989-60084128_HERVHF 3LTR 96.018 452 18 0 1 452
1 452 0 735 99
Homo_sapiens_13:86358624-86363686_HERVHF 5LTR
Homo_sapiens_16:60078989-60084128_HERVHF 5LTR 95.585 453 19 1 3 454
1 453 0 724 99
Homo_sapiens_13:86358624-86363686_HERVHF 3LTR
Homo_sapiens_2:215922762-215927674_HERVHF3LTR 96.476 454 14 1 1 452
1 454 0 745 99
Homo_sapiens_13:86358624-86363686_HERVHF 5LTR
Homo_sapiens_2:215922762-215927674_HERVHF5LTR 95.604 455 17 2 2 453
1 455 0 725 99
Homo_sapiens_13:86358624-86363686_HERVHF 3LTR
Homo_sapiens_3:112418865-112422911_HERVHF3LTR 96.026 453 17 1 1 452
3 455 0 733 99
Homo_sapiens_13:86358624-86363686_HERVHF 5LTR
Homo_sapiens_3:112418865-112422911_HERVHF5LTR 95.575 452 19 1 1 451

4	455	0	722	99
Homo_sapiens_13:86358624-86363686_HERVHF	5LTR
	Homo_sapiens_3:115793932-115798715_HERVHF	3LTR	95.344	451	21	0	1	451
1	451	0	719	99
Homo_sapiens_13:86358624-86363686_HERVHF	3LTR
	Homo_sapiens_3:115793932-115798715_HERVHF	5LTR	96.231	451	16	1	1	451
2	451	0	734	99
Homo_sapiens_13:86358624-86363686_HERVHF	3LTR
	Homo_sapiens_9:121790452-121796319_HERVHF	3LTR	96.018	452	17	1	1	452
2	452	0	731	99
Homo_sapiens_13:86358624-86363686_HERVHF	5LTR
	Homo_sapiens_9:121790452-121796319_HERVHF	5LTR	95.806	453	16	3	1	452
1	451	0	721	99
Homo_sapiens_13:86358624-86363686_HERVHF	3LTR
	Homo_sapiens_9:99670236-99675451_HERVHF	3LTR	96.026	453	18	0	1	453
1	453	0	737	99
Homo_sapiens_13:86358624-86363686_HERVHF	5LTR
	Homo_sapiens_9:99670236-99675451_HERVHF	5LTR	95.385	455	20	1	2	455
1	455	0	723	99
Homo_sapiens_13:86358624-86363686_HERVHF	3LTR
	Homo_sapiens_X:98524704-98529618_HERVHF	3LTR	95.565	451	20	0	1	451
10	460	0	724	99
Homo_sapiens_13:86358624-86363686_HERVHF	5LTR
	Homo_sapiens_X:98524704-98529618_HERVHF	5LTR	95.595	454	18	2	1	452
8	461	0	722	99
Homo_sapiens_15:87831557-87836573_HERVHF	3LTR
	Homo_sapiens_16:60078989-60084128_HERVHF	3LTR	94.26	453	23	3	1	451
1	452	0	690	100
Homo_sapiens_15:87831557-87836573_HERVHF	5LTR
	Homo_sapiens_16:60078989-60084128_HERVHF	5LTR	96.231	451	15	1	2	450
1	451	0	735	99
Homo_sapiens_15:87831557-87836573_HERVHF	3LTR
	Homo_sapiens_19:53831857-53836889_HERVHF	3LTR	94.04	453	24	3	1	451
10	461	0	685	100
Homo_sapiens_15:87831557-87836573_HERVHF	5LTR
	Homo_sapiens_19:53831857-53836889_HERVHF	5LTR	95.354	452	19	1	1	450
1	452	0	719	100
Homo_sapiens_15:87831557-87836573_HERVHF	3LTR
	Homo_sapiens_2:215922762-215927674_HERVHF	3LTR	94.505	455	20	4	1	451
1	454	0	695	100
Homo_sapiens_15:87831557-87836573_HERVHF	5LTR
	Homo_sapiens_2:215922762-215927674_HERVHF	5LTR	95.595	454	16	2	1	450
1	454	0	724	100
Homo_sapiens_15:87831557-87836573_HERVHF	3LTR

Homo_sapiens_2:5001218-5006279_HERVHF 3LTR 94.027 452 25 2 1 451
3 453 0 687 100
Homo_sapiens_15:87831557-87836573_HERVHF 5LTR
Homo_sapiens_2:5001218-5006279_HERVHF 5LTR 94.248 452 21 2 1 450
2 450 0 694 100
Homo_sapiens_15:87831557-87836573_HERVHF 5LTR
Homo_sapiens_2:76574075-76579252_HERVHF 3LTR 94.235 451 24 1 1 449
2 452 0 694 99
Homo_sapiens_15:87831557-87836573_HERVHF 3LTR
Homo_sapiens_2:76574075-76579252_HERVHF 5LTR 94.027 452 24 3 1 450
2 452 0 683 99
Homo_sapiens_15:87831557-87836573_HERVHF 3LTR
Homo_sapiens_20:12754276-12759162_HERVHF 3LTR 94.702 453 17 4 1 447
19 470 0 698 99
Homo_sapiens_15:87831557-87836573_HERVHF 5LTR
Homo_sapiens_20:12754276-12759162_HERVHF 5LTR 94.737 456 18 2 1 450
19 474 0 711 100
Homo_sapiens_15:87831557-87836573_HERVHF 3LTR
Homo_sapiens_3:112418865-112422911_HERVHF 3LTR 95.364 453 19 2 1 451
3 455 0 716 100
Homo_sapiens_15:87831557-87836573_HERVHF 5LTR
Homo_sapiens_3:112418865-112422911_HERVHF 5LTR 96.231 451 15 1 1 449
5 455 0 735 99
Homo_sapiens_15:87831557-87836573_HERVHF 5LTR
Homo_sapiens_3:115793932-115798715_HERVHF 3LTR 94.678 451 21 2 1 449
2 451 0 700 99
Homo_sapiens_15:87831557-87836573_HERVHF 3LTR
Homo_sapiens_3:115793932-115798715_HERVHF 5LTR 94.9 451 21 2 1 450 2
451 0 703 99
Homo_sapiens_15:87831557-87836573_HERVHF 3LTR
Homo_sapiens_3:191666713-191671926_HERVHF 3LTR 94.69 452 21 3 1 450
3 453 0 697 99
Homo_sapiens_15:87831557-87836573_HERVHF 5LTR
Homo_sapiens_3:191666713-191671926_HERVHF 5LTR 94.678 451 21 2 1 449
6 455 0 700 99
Homo_sapiens_15:87831557-87836573_HERVHF 5LTR
Homo_sapiens_4:125562071-125567006_HERVHF 3LTR 95.078 447 18 2 5 449
7 451 0 702 99
Homo_sapiens_15:87831557-87836573_HERVHF 3LTR
Homo_sapiens_4:125562071-125567006_HERVHF 5LTR 94.469 452 22 3 1 450
3 453 0 692 99
Homo_sapiens_15:87831557-87836573_HERVHF 5LTR
Homo_sapiens_4:78714293-78719270_HERVHF 3LTR 94.9 451 20 2 1 449 2
451 0 704 99

Homo_sapiens_15:87831557-87836573_HERVHF 3LTR
Homo_sapiens_4:78714293-78719270_HERVHF 5LTR 94.912 452 21 2 1 450
2 453 0 705 99
Homo_sapiens_15:87831557-87836573_HERVHF 5LTR
Homo_sapiens_6:115032245-115037164_HERVHF3LTR 95.133 452 19 2 1 450
7 457 0 710 100
Homo_sapiens_15:87831557-87836573_HERVHF 3LTR
Homo_sapiens_6:115032245-115037164_HERVHF5LTR 94.26 453 23 3 1 451
7 458 0 690 100
Homo_sapiens_15:87831557-87836573_HERVHF 3LTR
Homo_sapiens_9:121790452-121796319_HERVHF3LTR 94.26 453 22 3 1 451
2 452 0 691 100
Homo_sapiens_15:87831557-87836573_HERVHF 5LTR
Homo_sapiens_9:121790452-121796319_HERVHF5LTR 95.796 452 15 3 1 450
2 451 0 720 100
Homo_sapiens_15:87831557-87836573_HERVHF 5LTR
Homo_sapiens_9:99670236-99675451_HERVHF 3LTR 95.796 452 17 1 1 450
1 452 0 728 100
Homo_sapiens_15:87831557-87836573_HERVHF 3LTR
Homo_sapiens_9:99670236-99675451_HERVHF 5LTR 94.923 453 20 3 1 451
1 452 0 703 100
Homo_sapiens_15:87831557-87836573_HERVHF 3LTR
Homo_sapiens_X:87682131-87687194_HERVHF 3LTR 94.481 453 22 3 1 451
1 452 0 694 100
Homo_sapiens_15:87831557-87836573_HERVHF 5LTR
Homo_sapiens_X:87682131-87687194_HERVHF 5LTR 94.69 452 21 2 1 450
1 451 0 701 100
Homo_sapiens_15:87831557-87836573_HERVHF 3LTR
Homo_sapiens_X:92824882-92828895_HERVHF 3LTR 94.481 453 22 3 1 451
1 452 0 694 100
Homo_sapiens_15:87831557-87836573_HERVHF 5LTR
Homo_sapiens_X:92824882-92828895_HERVHF 5LTR 95.815 454 15 2 1 450
1 454 0 728 100
Homo_sapiens_19:54894715-54900183_HERVHF 3LTR
Homo_sapiens_3:72023534-72028493_HERVHF 3LTR 94.074 405 23 1 2 406
2 405 9.34E-180619 98
Homo_sapiens_19:54894715-54900183_HERVHF 5LTR
Homo_sapiens_3:72023534-72028493_HERVHF 5LTR 93.237 414 22 3 7 420
2 409 1.4E-177 613 99
Homo_sapiens_19:54894715-54900183_HERVHF 5LTR
Homo_sapiens_4:178207533-178212931_HERVHF3LTR 94.458 415 19 4 7 420
1 412 0 631 99
Homo_sapiens_19:54894715-54900183_HERVHF 3LTR
Homo_sapiens_4:178207533-178212931_HERVHF5LTR 92.995 414 28 1 2 415

1 413 1.39E-177613 99
Homo_sapiens_2:5001218-5006279_HERVHF 3LTR
Homo_sapiens_6:125702300-125707310_HERVHF3LTR 95.604 455 14 3 2 453
2 453 0 723 99
Homo_sapiens_2:5001218-5006279_HERVHF 5LTR
Homo_sapiens_6:125702300-125707310_HERVHF5LTR 95.796 452 16 1 1 449
3 454 0 728 99
Homo_sapiens_2:58113791-58118698_HERVHF 3LTR
Homo_sapiens_5:100984733-100989791_HERVHF3LTR 94.693 358 18 1 1 357
92 449 8.02E-161557 100
Homo_sapiens_2:58113791-58118698_HERVHF 5LTR
Homo_sapiens_5:100984733-100989791_HERVHF5LTR 95.543 359 12 2 2 356
82 440 3.64E-165571 99
Homo_sapiens_2:58113791-58118698_HERVHF 3LTR
Homo_sapiens_6:124890844-124897330_HERVHF3LTR 96.648 358 11 1 1 357
75 432 1.36E-170589 100
Homo_sapiens_2:58113791-58118698_HERVHF 5LTR
Homo_sapiens_6:124890844-124897330_HERVHF5LTR 94.722 360 15 2 2 357
71 430 2.3E-161 559 99
Homo_sapiens_2:58113791-58118698_HERVHF 5LTR
Homo_sapiens_6:144923609-144928414_HERVHF3LTR 94.167 360 17 2 2 357
82 441 1.19E-158550 99
Homo_sapiens_2:58113791-58118698_HERVHF 3LTR
Homo_sapiens_6:144923609-144928414_HERVHF5LTR 94.693 358 18 1 1 357
86 443 8.02E-161557 100
Homo_sapiens_2:58113791-58118698_HERVHF 3LTR
Homo_sapiens_8:99944145-99949257_HERVHF 3LTR 95.726 351 13 2 9 357
1 351 2.3E-161 559 98
Homo_sapiens_2:58113791-58118698_HERVHF 5LTR
Homo_sapiens_8:99944145-99949257_HERVHF 5LTR 95.977 348 13 1 11 357
1 348 1.89E-162562 97
Homo_sapiens_2:71086810-71090545_HERVHF 3LTR
Homo_sapiens_3:112418865-112422911_HERVHF 3LTR 96.026 453 17 1 2 453
2 454 0 733 99
Homo_sapiens_2:71086810-71090545_HERVHF 5LTR
Homo_sapiens_3:112418865-112422911_HERVHF 5LTR 95.374 454 19 1 1 452
2 455 0 722 100
Homo_sapiens_2:71086810-71090545_HERVHF 3LTR
Homo_sapiens_X:87682131-87687194_HERVHF 3LTR 96.437 449 16 0 5 453
3 451 0 738 99
Homo_sapiens_2:71086810-71090545_HERVHF 5LTR
Homo_sapiens_X:87682131-87687194_HERVHF 5LTR 95.565 451 17 2 4 452
1 450 0 718 99
Homo_sapiens_2:71086810-71090545_HERVHF 3LTR

Homo_sapiens_X:92824882-92828895_HERVHF 3LTR 95.991 449 18 0 5 453
3 451 0 729 99
Homo_sapiens_2:71086810-71090545_HERVHF 5LTR
Homo_sapiens_X:92824882-92828895_HERVHF 5LTR 95.585 453 16 2 4 452
1 453 0 722 99
Homo_sapiens_2:77088587-77093690_HERVHF 3LTR
Homo_sapiens_3:112418865-112422911_HERVHF 3LTR 96.481 341 9 2 1 338
26 366 2.75E-160555 96
Homo_sapiens_2:77088587-77093690_HERVHF 5LTR
Homo_sapiens_3:112418865-112422911_HERVHF 5LTR 95.87 339 14 0 1 339
29 367 1.13E-158549 100
Homo_sapiens_2:77088587-77093690_HERVHF 3LTR
Homo_sapiens_4:125562071-125567006_HERVHF3LTR 96.471 340 10 1 1 338
26 365 7.88E-161557 96
Homo_sapiens_2:77088587-77093690_HERVHF 5LTR
Homo_sapiens_4:125562071-125567006_HERVHF5LTR 95.87 339 14 0 1 339
27 365 1.13E-158549 100
Homo_sapiens_2:77088587-77093690_HERVHF 3LTR
Homo_sapiens_6:125702300-125707310_HERVHF3LTR 95.614 342 8 3 1 338
26 364 1.74E-156543 96
Homo_sapiens_2:77088587-77093690_HERVHF 5LTR
Homo_sapiens_6:125702300-125707310_HERVHF5LTR 96.46 339 12 0 1 339
28 366 2.18E-161558 100
Homo_sapiens_2:77088587-77093690_HERVHF 5LTR
Homo_sapiens_6:90670654-90675059_HERVHF 3LTR 95.575 339 15 0 1 339
33 371 4.79E-157545 100
Homo_sapiens_2:77088587-77093690_HERVHF 3LTR
Homo_sapiens_6:90670654-90675059_HERVHF 5LTR 95.882 340 12 1 1 338
28 367 4.08E-158548 96
Homo_sapiens_2:77088587-77093690_HERVHF 5LTR
Homo_sapiens_X:87682131-87687194_HERVHF 3LTR 96.755 339 11 0 1 339
25 363 1.79E-162563 100
Homo_sapiens_2:77088587-77093690_HERVHF 3LTR
Homo_sapiens_X:87682131-87687194_HERVHF 5LTR 95.882 340 11 2 1 338
24 362 4.97E-157545 96
Homo_sapiens_2:77088587-77093690_HERVHF 3LTR
Homo_sapiens_X:98524704-98529618_HERVHF 3LTR 95.588 340 13 1 1 338
33 372 4.97E-157544 96
Homo_sapiens_2:77088587-77093690_HERVHF 5LTR
Homo_sapiens_X:98524704-98529618_HERVHF 5LTR 95.882 340 13 1 1 339
33 372 3.93E-158547 100
Homo_sapiens_3:166501004-166506099_HERVHF5LTR
Homo_sapiens_4:130767812-130772870_HERVHF3LTR 94.062 320 16 2 3 322
1 317 1.07E-139486 99

Homo_sapiens_3:166501004-166506099_HERVHF3LTR
Homo_sapiens_4:130767812-130772870_HERVHF5LTR 93.769 321 17 2 3 320
1 321 1.29E-138483 99
Homo_sapiens_3:166501004-166506099_HERVHF5LTR
Homo_sapiens_7:31467260-31471973_HERVHF 3LTR 93.789 322 19 1 2 322
5 326 3.06E-140488 99
Homo_sapiens_3:166501004-166506099_HERVHF3LTR
Homo_sapiens_7:31467260-31471973_HERVHF 5LTR 93.769 321 18 1 2 320
1 321 1.06E-139487 99
Homo_sapiens_3:190920619-190925408_HERVHF5LTR
Homo_sapiens_X:87682131-87687194_HERVHF 3LTR 94.912 452 19 2 1 448
1 452 0 707 98
Homo_sapiens_3:190920619-190925408_HERVHF3LTR
Homo_sapiens_X:87682131-87687194_HERVHF 5LTR 95.354 452 19 2 4 454
1 451 0 714 99
Homo_sapiens_3:191666713-191671926_HERVHF5LTR
Homo_sapiens_6:115032245-115037164_HERVHF3LTR 94.945 455 23 0 1 455
2 456 0 718 100
Homo_sapiens_3:191666713-191671926_HERVHF3LTR
Homo_sapiens_6:115032245-115037164_HERVHF5LTR 95.575 452 20 0 2 453
6 457 0 726 99
Homo_sapiens_3:191666713-191671926_HERVHF5LTR
Homo_sapiens_6:125702300-125707310_HERVHF3LTR 94.934 454 17 3 5 455
2 452 0 708 99
Homo_sapiens_3:191666713-191671926_HERVHF3LTR
Homo_sapiens_6:125702300-125707310_HERVHF5LTR 95.133 452 22 0 2 453
3 454 0 717 99
Homo_sapiens_3:191666713-191671926_HERVHF5LTR
Homo_sapiens_9:121790452-121796319_HERVHF3LTR 95.575 452 18 2 5 455
1 451 0 719 99
Homo_sapiens_3:191666713-191671926_HERVHF3LTR
Homo_sapiens_9:121790452-121796319_HERVHF5LTR 94.912 452 21 2 2 453
1 450 0 705 99
Homo_sapiens_3:191666713-191671926_HERVHF3LTR
Homo_sapiens_9:99670236-99675451_HERVHF 3LTR 94.9 451 23 0 3 453 1
451 0 710 99
Homo_sapiens_3:191666713-191671926_HERVHF5LTR
Homo_sapiens_9:99670236-99675451_HERVHF 5LTR 95.787 451 18 1 6 455
1 451 0 725 99
Homo_sapiens_3:191666713-191671926_HERVHF5LTR
Homo_sapiens_X:92824882-92828895_HERVHF 3LTR 95.344 451 20 1 6 455
1 451 0 716 99
Homo_sapiens_3:191666713-191671926_HERVHF3LTR
Homo_sapiens_X:92824882-92828895_HERVHF 5LTR 94.923 453 21 1 3 453

1 453 0 711 99
Homo_sapiens_3:191666713-191671926_HERVHF3LTR
Homo_sapiens_X:98524704-98529618_HERVHF 3LTR 94.69 452 24 0 2 453
9 460 0 708 99
Homo_sapiens_3:191666713-191671926_HERVHF5LTR
Homo_sapiens_X:98524704-98529618_HERVHF 5LTR 94.748 457 22 2 1 455
4 460 0 710 100
Homo_sapiens_3:21189353-21193803_HERVHF 5LTR
Homo_sapiens_7:31467260-31471973_HERVHF 3LTR 93.168 322 21 1 1 322
75 395 1.59E-137479 100
Homo_sapiens_3:21189353-21193803_HERVHF 3LTR
Homo_sapiens_7:31467260-31471973_HERVHF 5LTR 93.333 330 20 2 1 329
67 395 9.18E-141489 98
Homo_sapiens_4:16998200-17003527_HERVHF 5LTR
Homo_sapiens_6:115032245-115037164_HERVHF3LTR 96.429 392 12 2 1 392
68 457 0 637 100
Homo_sapiens_4:16998200-17003527_HERVHF 3LTR
Homo_sapiens_6:115032245-115037164_HERVHF5LTR 95.949 395 16 0 6 400
64 458 0 641 99
Homo_sapiens_4:16998200-17003527_HERVHF 3LTR
Homo_sapiens_6:125702300-125707310_HERVHF3LTR 96 400 13 1 1 400 57
453 0 648 99
Homo_sapiens_4:16998200-17003527_HERVHF 5LTR
Homo_sapiens_6:125702300-125707310_HERVHF5LTR 96.931 391 11 1 1 391
65 454 0 648 99
Homo_sapiens_4:16998200-17003527_HERVHF 3LTR
Homo_sapiens_9:121790452-121796319_HERVHF3LTR 96.709 395 12 1 6 400
59 452 0 651 99
Homo_sapiens_4:16998200-17003527_HERVHF 5LTR
Homo_sapiens_9:121790452-121796319_HERVHF5LTR 96.429 392 11 2 1 392
63 451 0 638 100
Homo_sapiens_4:16998200-17003527_HERVHF 3LTR
Homo_sapiens_9:99670236-99675451_HERVHF 3LTR 95.707 396 17 0 6 401
58 453 0 638 99
Homo_sapiens_4:16998200-17003527_HERVHF 5LTR
Homo_sapiens_9:99670236-99675451_HERVHF 5LTR 96.429 392 13 1 1 392
62 452 0 641 100
Homo_sapiens_4:175461498-175466677_HERVHF5LTR
Homo_sapiens_6:125702300-125707310_HERVHF3LTR 93.75 336 18 2 1 335
121 454 1.19E-145506 100
Homo_sapiens_4:175461498-175466677_HERVHF3LTR
Homo_sapiens_6:125702300-125707310_HERVHF5LTR 95.062 324 14 2 1 322
131 454 1.16E-145506 99
Homo_sapiens_4:24501427-24506300_HERVHF 5LTR

Homo_sapiens_6:125702300-125707310_HERVHF3LTR 95.585 453 15 2 1 451
3 452 0 723 99
Homo_sapiens_4:24501427-24506300_HERVHF 3LTR
Homo_sapiens_6:125702300-125707310_HERVHF5LTR 95.787 451 19 0 1 451
4 454 0 728 99
Homo_sapiens_4:24501427-24506300_HERVHF 3LTR
Homo_sapiens_9:99670236-99675451_HERVHF 3LTR 95.787 451 19 0 1 451
1 451 0 728 99
Homo_sapiens_4:24501427-24506300_HERVHF 5LTR
Homo_sapiens_9:99670236-99675451_HERVHF 5LTR 95.565 451 20 0 1 451
1 451 0 724 99
Homo_sapiens_4:79554421-79559319_HERVHF 3LTR
Homo_sapiens_6:115032245-115037164_HERVHF3LTR 95.385 390 17 1 10 399
7 395 8.96E-180619 98
Homo_sapiens_4:79554421-79559319_HERVHF 5LTR
Homo_sapiens_6:115032245-115037164_HERVHF5LTR 95.19 395 19 0 3 397
3 397 0 627 99
Homo_sapiens_4:79554421-79559319_HERVHF 5LTR
Homo_sapiens_6:125702300-125707310_HERVHF3LTR 95.431 394 13 2 6 397
2 392 0 626 98
Homo_sapiens_4:79554421-79559319_HERVHF 3LTR
Homo_sapiens_6:125702300-125707310_HERVHF5LTR 95.641 390 17 0 10 399
4 393 0 627 98
Homo_sapiens_4:79554421-79559319_HERVHF 3LTR
Homo_sapiens_6:93830563-93835343_HERVHF 3LTR 95.641 390 17 0 10 399
12 401 0 627 98
Homo_sapiens_4:79554421-79559319_HERVHF 5LTR
Homo_sapiens_6:93830563-93835343_HERVHF 5LTR 95.939 394 15 1 4 397
1 393 0 636 99
Homo_sapiens_4:79554421-79559319_HERVHF 5LTR
Homo_sapiens_9:99670236-99675451_HERVHF 3LTR 95.652 391 17 0 7 397
1 391 0 629 98
Homo_sapiens_4:79554421-79559319_HERVHF 3LTR
Homo_sapiens_9:99670236-99675451_HERVHF 5LTR 95.641 390 17 0 10 399
1 390 0 627 98
Homo_sapiens_4:79554421-79559319_HERVHF 3LTR
Homo_sapiens_X:87682131-87687194_HERVHF 3LTR 95.897 390 16 0 10 399
1 390 0 632 98
Homo_sapiens_4:79554421-79559319_HERVHF 5LTR
Homo_sapiens_X:87682131-87687194_HERVHF 5LTR 96.419 391 13 1 7 397
1 390 0 639 98
Homo_sapiens_4:79554421-79559319_HERVHF 5LTR
Homo_sapiens_X:98524704-98529618_HERVHF 3LTR 96.684 392 13 0 6 397
9 400 0 649 98

Homo_sapiens_4:79554421-79559319_HERVHF 3LTR
Homo_sapiens_X:98524704-98529618_HERVHF 5LTR 95.652 391 16 1 10 399 9 399 0 626 98
Homo_sapiens_5:100984733-100989791_HERVHF5LTR
Homo_sapiens_6:144923609-144928414_HERVHF3LTR 96.136 440 17 0 1 440 1 440 0 718 100
Homo_sapiens_5:100984733-100989791_HERVHF3LTR
Homo_sapiens_6:144923609-144928414_HERVHF5LTR 96.33 436 16 0 14 449 8 443 0 715 97
Homo_sapiens_5:136565792-136570795_HERVHF3LTR
Homo_sapiens_6:115032245-115037164_HERVHF3LTR 93.377 453 25 4 4 453 6 456 0 669 99
Homo_sapiens_5:136565792-136570795_HERVHF5LTR
Homo_sapiens_6:115032245-115037164_HERVHF5LTR 94.444 450 25 0 3 452 6 455 0 700 99
Homo_sapiens_5:136565792-136570795_HERVHF3LTR
Homo_sapiens_6:125702300-125707310_HERVHF3LTR 94.298 456 17 5 3 453 1 452 0 691 99
Homo_sapiens_5:136565792-136570795_HERVHF5LTR
Homo_sapiens_6:125702300-125707310_HERVHF5LTR 95.133 452 22 0 1 452 1 452 0 717 100
Homo_sapiens_5:136565792-136570795_HERVHF5LTR
Homo_sapiens_6:131902010-131906969_HERVHF3LTR 93.541 449 27 1 4 452 1 447 0 677 99
Homo_sapiens_5:136565792-136570795_HERVHF3LTR
Homo_sapiens_6:131902010-131906969_HERVHF5LTR 93.612 454 24 3 5 453 1 454 0 680 99
Homo_sapiens_5:136565792-136570795_HERVHF5LTR
Homo_sapiens_7:83460120-83464935_HERVHF 3LTR 93.792 451 24 2 2 452 1 447 0 682 99
Homo_sapiens_5:136565792-136570795_HERVHF3LTR
Homo_sapiens_7:83460120-83464935_HERVHF 5LTR 94.481 453 21 3 4 453 1 452 0 695 99
Homo_sapiens_5:136565792-136570795_HERVHF5LTR
Homo_sapiens_8:114282133-114287268_HERVHF3LTR 93.636 440 26 1 1 440 15 452 0 665 97
Homo_sapiens_5:136565792-136570795_HERVHF3LTR
Homo_sapiens_8:114282133-114287268_HERVHF5LTR 94.091 440 24 2 1 439 12 450 0 670 97
Homo_sapiens_5:136565792-136570795_HERVHF3LTR
Homo_sapiens_8:97201222-97206202_HERVHF 3LTR 95.364 453 17 3 4 453 4 455 0 713 99
Homo_sapiens_5:136565792-136570795_HERVHF5LTR
Homo_sapiens_8:97201222-97206202_HERVHF 5LTR 94 450 27 0 3 452 1

450 0 691 99
Homo_sapiens_5:136565792-136570795_HERVHF3LTR
Homo_sapiens_9:121790452-121796319_HERVHF3LTR 94.26 453 21 3 4 453
1 451 0 691 99
Homo_sapiens_5:136565792-136570795_HERVHF5LTR
Homo_sapiens_9:121790452-121796319_HERVHF5LTR 94.222 450 24 2 3 452
1 448 0 688 99
Homo_sapiens_5:136565792-136570795_HERVHF3LTR
Homo_sapiens_9:99670236-99675451_HERVHF 3LTR 94.469 452 21 3 5 453
1 451 0 693 99
Homo_sapiens_5:136565792-136570795_HERVHF5LTR
Homo_sapiens_9:99670236-99675451_HERVHF 5LTR 94.878 449 23 0 4 452
1 449 0 707 99
Homo_sapiens_5:136565792-136570795_HERVHF3LTR
Homo_sapiens_X:87682131-87687194_HERVHF 3LTR 94.912 452 19 3 5 453
1 451 0 702 99
Homo_sapiens_5:136565792-136570795_HERVHF5LTR
Homo_sapiens_X:87682131-87687194_HERVHF 5LTR 93.318 449 29 1 4 452
1 448 0 672 99
Homo_sapiens_5:136565792-136570795_HERVHF3LTR
Homo_sapiens_X:92824882-92828895_HERVHF 3LTR 95.354 452 17 3 5 453
1 451 0 711 99
Homo_sapiens_5:136565792-136570795_HERVHF5LTR
Homo_sapiens_X:92824882-92828895_HERVHF 5LTR 94.013 451 25 1 4 452
1 451 0 690 99
Homo_sapiens_5:136565792-136570795_HERVHF3LTR
Homo_sapiens_X:98524704-98529618_HERVHF 3LTR 93.202 456 27 3 1 453
6 460 0 673 100
Homo_sapiens_5:136565792-136570795_HERVHF5LTR
Homo_sapiens_X:98524704-98529618_HERVHF 5LTR 95.444 439 19 1 1 438
6 444 0 699 97
Homo_sapiens_5:136565792-136570795_HERVHF3LTR
Homo_sapiens_Y:5941562-5945587_HERVHF 3LTR 93.584 452 24 4 5 453
1 450 0 672 99
Homo_sapiens_5:136565792-136570795_HERVHF5LTR
Homo_sapiens_Y:5941562-5945587_HERVHF 5LTR 93.541 449 28 1 4 452
1 448 0 676 99
Homo_sapiens_6:115032245-115037164_HERVHF3LTR
Homo_sapiens_9:99670236-99675451_HERVHF 3LTR 95.575 452 19 1 7 457
1 452 0 722 98
Homo_sapiens_6:115032245-115037164_HERVHF5LTR
Homo_sapiens_9:99670236-99675451_HERVHF 5LTR 96.681 452 15 0 7 458
1 452 0 748 99
Homo_sapiens_6:121620869-121625942_HERVHF3LTR

Homo_sapiens_6:125702300-125707310_HERVHF3LTR 94.915 354 12 3 1 352
8 357 1.17E-158550 100
Homo_sapiens_6:121620869-121625942_HERVHF5LTR
Homo_sapiens_6:125702300-125707310_HERVHF5LTR 96.089 358 13 1 1 357
3 360 7.11E-168580 99
Homo_sapiens_6:121620869-121625942_HERVHF3LTR
Homo_sapiens_6:131339252-131344112_HERVHF3LTR 94.286 350 19 1 1 350
8 356 2.12E-155538 99
Homo_sapiens_6:121620869-121625942_HERVHF5LTR
Homo_sapiens_6:131339252-131344112_HERVHF5LTR 95.251 358 16 1 1 357
1 358 1.57E-163566 99
Homo_sapiens_6:121620869-121625942_HERVHF3LTR
Homo_sapiens_6:131902010-131906969_HERVHF3LTR 95.455 352 15 1 1 352
6 356 6.49E-162560 100
Homo_sapiens_6:121620869-121625942_HERVHF5LTR
Homo_sapiens_6:131902010-131906969_HERVHF5LTR 94.972 358 16 2 2 357
1 358 2.33E-161558 99
Homo_sapiens_6:121620869-121625942_HERVHF3LTR
Homo_sapiens_6:90670654-90675059_HERVHF 3LTR 94.886 352 17 1 1 352
14 364 3.36E-159551 100
Homo_sapiens_6:121620869-121625942_HERVHF5LTR
Homo_sapiens_6:90670654-90675059_HERVHF 5LTR 95.531 358 15 1 1 357
4 361 3.68E-165571 99
Homo_sapiens_6:121620869-121625942_HERVHF3LTR
Homo_sapiens_6:93830563-93835343_HERVHF 3LTR 94.886 352 17 1 1 352
17 367 3.36E-159551 100
Homo_sapiens_6:121620869-121625942_HERVHF5LTR
Homo_sapiens_6:93830563-93835343_HERVHF 5LTR 94.134 358 19 2 1 357
3 359 5.12E-157545 99
Homo_sapiens_6:121620869-121625942_HERVHF5LTR
Homo_sapiens_8:114242056-114246631_HERVHF3LTR 93.575 358 22 1 1 357
4 361 2.18E-155539 99
Homo_sapiens_6:121620869-121625942_HERVHF3LTR
Homo_sapiens_8:114242056-114246631_HERVHF5LTR 95.17 352 16 1 1 352
7 357 2.76E-160555 100
Homo_sapiens_6:121620869-121625942_HERVHF3LTR
Homo_sapiens_8:114282133-114287268_HERVHF3LTR 94.286 350 19 1 1 350
23 371 2.12E-155538 99
Homo_sapiens_6:121620869-121625942_HERVHF5LTR
Homo_sapiens_8:114282133-114287268_HERVHF5LTR 93.646 362 22 1 1 361
15 376 1.47E-157546 100
Homo_sapiens_6:121620869-121625942_HERVHF3LTR
Homo_sapiens_8:97201222-97206202_HERVHF 3LTR 95.739 352 14 1 1 352
10 360 5.33E-163564 100

Homo_sapiens_6:121620869-121625942_HERVHF5LTR
Homo_sapiens_8:97201222-97206202_HERVHF 5LTR 94.693 358 18 1 1 357
1 358 8.12E-161557 99
Homo_sapiens_6:121620869-121625942_HERVHF3LTR
Homo_sapiens_9:121790452-121796319_HERVHF3LTR 94.886 352 16 2 1 352
7 356 4.09E-158547 100
Homo_sapiens_6:121620869-121625942_HERVHF5LTR
Homo_sapiens_9:121790452-121796319_HERVHF5LTR 94.693 358 16 3 1 357
1 356 1.2E-158 550 99
Homo_sapiens_6:121620869-121625942_HERVHF3LTR
Homo_sapiens_9:99670236-99675451_HERVHF 3LTR 94.602 352 18 1 1 352
6 356 1.43E-157546 100
Homo_sapiens_6:121620869-121625942_HERVHF5LTR
Homo_sapiens_9:99670236-99675451_HERVHF 5LTR 94.958 357 17 1 2 357
1 357 6.66E-162560 99
Homo_sapiens_6:121620869-121625942_HERVHF3LTR
Homo_sapiens_X:87682131-87687194_HERVHF 3LTR 96.591 352 11 1 1 352
6 356 2.42E-167578 100
Homo_sapiens_6:121620869-121625942_HERVHF5LTR
Homo_sapiens_X:87682131-87687194_HERVHF 5LTR 94.678 357 17 2 2 357
1 356 3.45E-159552 99
Homo_sapiens_6:121620869-121625942_HERVHF3LTR
Homo_sapiens_X:92824882-92828895_HERVHF 3LTR 95.455 352 15 1 1 352
6 356 6.49E-162560 100
Homo_sapiens_6:121620869-121625942_HERVHF5LTR
Homo_sapiens_X:92824882-92828895_HERVHF 5LTR 94.986 359 15 2 2 357
1 359 6.66E-162561 99
Homo_sapiens_6:121620869-121625942_HERVHF3LTR
Homo_sapiens_X:98524704-98529618_HERVHF 3LTR 94.886 352 17 1 1 352
15 365 3.36E-159551 100
Homo_sapiens_6:121620869-121625942_HERVHF5LTR
Homo_sapiens_X:98524704-98529618_HERVHF 5LTR 94.986 359 16 2 1 357
8 366 6.66E-162560 99
Homo_sapiens_6:131339252-131344112_HERVHF3LTR
Homo_sapiens_8:97201222-97206202_HERVHF 3LTR 94.493 454 25 0 1 454
3 456 0 707 100
Homo_sapiens_6:131339252-131344112_HERVHF5LTR
Homo_sapiens_8:97201222-97206202_HERVHF 5LTR 95.143 453 22 0 1 453
1 453 0 719 100
Homo_sapiens_6:131339252-131344112_HERVHF3LTR
Homo_sapiens_9:121790452-121796319_HERVHF3LTR 94.702 453 23 1 2 454
1 452 0 706 99
Homo_sapiens_6:131339252-131344112_HERVHF5LTR
Homo_sapiens_9:121790452-121796319_HERVHF5LTR 95.143 453 20 2 1 453

1 451 0 711 100
Homo_sapiens_6:131339252-131344112_HERVHF3LTR
Homo_sapiens_X:92824882-92828895_HERVHF 3LTR 94.912 452 23 0 3 454
1 452 0 712 99
Homo_sapiens_6:131339252-131344112_HERVHF5LTR
Homo_sapiens_X:92824882-92828895_HERVHF 5LTR 96.035 454 16 1 2 453
1 454 0 736 99
Homo_sapiens_6:131902010-131906969_HERVHF5LTR
Homo_sapiens_8:97201222-97206202_HERVHF 3LTR 95.165 455 19 2 1 455
5 456 0 716 99
Homo_sapiens_6:131902010-131906969_HERVHF3LTR
Homo_sapiens_8:97201222-97206202_HERVHF 5LTR 95.354 452 19 1 1 450
2 453 0 719 99
Homo_sapiens_6:131902010-131906969_HERVHF3LTR
Homo_sapiens_X:87682131-87687194_HERVHF 3LTR 95.796 452 17 1 1 450
1 452 0 728 99
Homo_sapiens_6:131902010-131906969_HERVHF5LTR
Homo_sapiens_X:87682131-87687194_HERVHF 5LTR 95.385 455 17 3 1 455
1 451 0 717 99
Homo_sapiens_8:114282133-114287268_HERVHF3LTR
Homo_sapiens_8:97201222-97206202_HERVHF 3LTR 94.796 442 21 1 14 453
1 442 0 691 96
Homo_sapiens_8:114282133-114287268_HERVHF5LTR
Homo_sapiens_8:97201222-97206202_HERVHF 5LTR 94.521 438 22 1 15 450
1 438 0 680 97
Homo_sapiens_8:114282133-114287268_HERVHF3LTR
Homo_sapiens_9:121790452-121796319_HERVHF3LTR 94.977 438 19 2 17 452
1 437 0 685 95
Homo_sapiens_8:114282133-114287268_HERVHF5LTR
Homo_sapiens_9:121790452-121796319_HERVHF5LTR 95.183 436 17 3 15 448
1 434 0 682 96
Homo_sapiens_8:114282133-114287268_HERVHF3LTR
Homo_sapiens_9:99670236-99675451_HERVHF 3LTR 94.357 443 23 1 18 458
1 443 0 684 96
Homo_sapiens_8:114282133-114287268_HERVHF5LTR
Homo_sapiens_9:99670236-99675451_HERVHF 5LTR 95.195 437 19 1 16 450
1 437 0 691 97
Homo_sapiens_8:114282133-114287268_HERVHF3LTR
Homo_sapiens_X:87682131-87687194_HERVHF 3LTR 95.652 437 17 1 18 452
1 437 0 700 95
Homo_sapiens_8:114282133-114287268_HERVHF5LTR
Homo_sapiens_X:87682131-87687194_HERVHF 5LTR 94.508 437 21 2 16 450
1 436 0 674 97
Homo_sapiens_8:114282133-114287268_HERVHF3LTR

Homo_sapiens_X:92824882-92828895_HERVHF 3LTR 94.966 437 20 1 18 452
1 437 0 687 95
Homo_sapiens_8:114282133-114287268_HERVHF 5LTR
Homo_sapiens_X:92824882-92828895_HERVHF 5LTR 95.444 439 16 2 16 450
1 439 0 697 97
Homo_sapiens_8:114282133-114287268_HERVHF 3LTR
Homo_sapiens_X:98524704-98529618_HERVHF 3LTR 94.292 438 23 1 17 452
9 446 0 675 95
Homo_sapiens_8:114282133-114287268_HERVHF 5LTR
Homo_sapiens_X:98524704-98529618_HERVHF 5LTR 95.195 437 18 2 15 448
8 444 0 688 96
Homo_sapiens_8:114282133-114287268_HERVHF 5LTR
Homo_sapiens_Y:5941562-5945587_HERVHF 3LTR 94.737 437 20 2 16 450
1 436 0 679 97
Homo_sapiens_8:114282133-114287268_HERVHF 3LTR
Homo_sapiens_Y:5941562-5945587_HERVHF 5LTR 94.737 437 20 2 18 452
1 436 0 679 95
Homo_sapiens_9:80137640-80142712_HERVHF 3LTR
Homo_sapiens_X:87682131-87687194_HERVHF 3LTR 95.087 346 13 2 1 343
1 345 1.69E-156543 100
Homo_sapiens_9:80137640-80142712_HERVHF 5LTR
Homo_sapiens_X:87682131-87687194_HERVHF 5LTR 96.21 343 12 1 1 343
3 344 7.69E-161557 100
Homo_sapiens_9:80137640-80142712_HERVHF 3LTR
Homo_sapiens_X:92824882-92828895_HERVHF 3LTR 94.493 345 15 2 1 342
1 344 3.06E-153532 99
Homo_sapiens_9:80137640-80142712_HERVHF 5LTR
Homo_sapiens_X:92824882-92828895_HERVHF 5LTR 95.64 344 13 1 1 342
3 346 3.27E-159551 99
Homo_sapiens_X:90773760-90778707_HERVHF 3LTR
Homo_sapiens_X:92824882-92828895_HERVHF 3LTR 94.866 448 23 0 1 448
2 449 0 705 100
Homo_sapiens_X:90773760-90778707_HERVHF 5LTR
Homo_sapiens_X:92824882-92828895_HERVHF 5LTR 92.683 451 28 3 1 446
2 452 0 656 100
Homo_sapiens_X:90773760-90778707_HERVHF 3LTR
Homo_sapiens_Y:4355841-4360781_HERVHF 3LTR 97.321 448 11 1 1 448
1 447 0 751 100
Homo_sapiens_X:90773760-90778707_HERVHF 5LTR
Homo_sapiens_Y:4355841-4360781_HERVHF 5LTR 95.546 449 17 2 1 446
1 449 0 714 100
Homo_sapiens_X:90773760-90778707_HERVHF 3LTR
Homo_sapiens_Y:5325239-5329976_HERVHF 3LTR 94.196 448 25 1 1 448
4 450 0 688 100

Homo_sapiens_X:90773760-90778707_HERVHF 5LTR
Homo_sapiens_Y:5325239-5329976_HERVHF 5LTR 93.318 449 25 4 1 446
7 453 0 662 100
Homo_sapiens_Y:4355841-4360781_HERVHF 3LTR
Homo_sapiens_Y:5941562-5945587_HERVHF 3LTR 93.778 450 26 2 1 449
2 450 0 679 100
Homo_sapiens_Y:4355841-4360781_HERVHF 5LTR
Homo_sapiens_Y:5941562-5945587_HERVHF 5LTR 93.792 451 25 3 1 450
2 450 0 677 100
Homo_sapiens_Y:5325239-5329976_HERVHF 3LTR
Homo_sapiens_Y:5941562-5945587_HERVHF 3LTR 95.122 451 20 2 3 452
1 450 0 708 99
Homo_sapiens_Y:5325239-5329976_HERVHF 5LTR
Homo_sapiens_Y:5941562-5945587_HERVHF 5LTR 94.444 450 24 1 6 454
1 450 0 696 99
Hylobates_lar_LG01:19437067-19443389_HERVHF 5LTR
Hylobates_lar_LG14:29706095-29711000_HERVHF 3LTR 93.367 392 23 2 2
392 3 392 2.07E-169584 99
Hylobates_lar_LG01:19437067-19443389_HERVHF 3LTR
Hylobates_lar_LG14:29706095-29711000_HERVHF 5LTR 94.472 398 22 0 1
398 7 404 2.92E-180619 100
Hylobates_lar_LG01:19437067-19443389_HERVHF 3LTR
Hylobates_lar_LG18:30372700-30377982_HERVHF 3LTR 94.724 398 20 1 1
398 3 399 2.92E-180620 100
Hylobates_lar_LG01:19437067-19443389_HERVHF 5LTR
Hylobates_lar_LG18:30372700-30377982_HERVHF 5LTR 93.35 391 25 1 2
391 2 392 5.92E-170585 99
Hylobates_lar_LG01:244299162-244304632_HERVHF 5LTR
Hylobates_lar_LG20:16903045-16908295_HERVHF 3LTR 93.369 377 23 2 1
376 52 427 2.28E-162561 99
Hylobates_lar_LG01:244299162-244304632_HERVHF 3LTR
Hylobates_lar_LG20:16903045-16908295_HERVHF 5LTR 94.947 376 15 4 3
377 58 430 8.48E-168579 99
Hylobates_lar_LG03:158489633-158494538_HERVHF 3LTR
Hylobates_lar_LG17:76674886-76679638_HERVHF 3LTR 94.66 412 20 2 1
412 1 410 0 637 99
Hylobates_lar_LG03:158489633-158494538_HERVHF 5LTR
Hylobates_lar_LG17:76674886-76679638_HERVHF 5LTR 95.25 400 17 2 3
402 1 398 0 629 97
Hylobates_lar_LG04:133444289-133449196_HERVHF 3LTR
Hylobates_lar_LG04:26177466-26182427_HERVHF 3LTR 91.098 337 27 3 15
351 78 411 4.34E-133462 96
Hylobates_lar_LG04:133444289-133449196_HERVHF 5LTR
Hylobates_lar_LG04:26177466-26182427_HERVHF 5LTR 94.767 344 18 0 1

344 64 407 1.93E-156540 100
Hylobates_lar_LG04:133444289-133449196_HERVHF 3LTR
Hylobates_lar_LG08:125778193-125783483_HERVHF 3LTR 95.157 351 17 0 1
351 103 453 2.56E-161557 100
Hylobates_lar_LG04:133444289-133449196_HERVHF 5LTR
Hylobates_lar_LG08:125778193-125783483_HERVHF 5LTR 91.071 336 28 2 11
344 115 450 1.22E-133464 97
Hylobates_lar_LG04:133444289-133449196_HERVHF 3LTR
Hylobates_lar_LG08:96760714-96766571_HERVHF 3LTR 91.691 337 25 3 15
351 77 410 8.37E-136471 96
Hylobates_lar_LG04:133444289-133449196_HERVHF 5LTR
Hylobates_lar_LG08:96760714-96766571_HERVHF 5LTR 94.477 344 18 1 1
344 63 405 9.98E-154532 100
Hylobates_lar_LG04:133444289-133449196_HERVHF 3LTR
Hylobates_lar_LG09:112447534-112452391_HERVHF 3LTR 91.691 337 25 2 15
351 77 410 6.87E-137476 96
Hylobates_lar_LG04:133444289-133449196_HERVHF 5LTR
Hylobates_lar_LG09:112447534-112452391_HERVHF 5LTR 92.878 337 22 2 1
335 63 399 2.51E-142493 97
Hylobates_lar_LG04:133444289-133449196_HERVHF 3LTR
Hylobates_lar_LG16:101610592-101617187_HERVHF 3LTR 91.395 337 28 1 15
351 76 411 2.4E-136 474 96
Hylobates_lar_LG04:133444289-133449196_HERVHF 5LTR
Hylobates_lar_LG16:101610592-101617187_HERVHF 5LTR 94.783 345 17 1 1
344 64 408 6.72E-156538 100
Hylobates_lar_LG04:133444289-133449196_HERVHF 5LTR
Hylobates_lar_LG17:81173180-81178674_HERVHF 3LTR 91.239 331 28 1 15
344 116 446 4.25E-133463 96
Hylobates_lar_LG04:133444289-133449196_HERVHF 3LTR
Hylobates_lar_LG17:81173180-81178674_HERVHF 5LTR 92.857 350 24 1 2
351 103 451 7.83E-149516 99
Hylobates_lar_LG04:133444289-133449196_HERVHF 3LTR
Hylobates_lar_LG20:16903045-16908295_HERVHF 3LTR 91.743 327 25 2 15
341 92 416 1.51E-132462 93
Hylobates_lar_LG04:133444289-133449196_HERVHF 5LTR
Hylobates_lar_LG20:16903045-16908295_HERVHF 5LTR 94.428 341 19 0 1
341 81 421 3.48E-153530 99
Hylobates_lar_LG06:38831828-38837745_HERVHF 3LTR
Hylobates_lar_LG08:96760714-96766571_HERVHF 3LTR 93.311 299 17 3 1
299 116 411 4.41E-126439 95
Hylobates_lar_LG06:38831828-38837745_HERVHF 5LTR
Hylobates_lar_LG08:96760714-96766571_HERVHF 5LTR 92.977 299 18 2 2
298 114 411 4.34E-126439 96
Hylobates_lar_LG06:38831828-38837745_HERVHF 5LTR

Hylobates_lar_LG09:112447534-112452391_HERVHF 3LTR 91.639 299 21 2 2 298 115 411 1.16E-120422 96
Hylobates_lar_LG06:38831828-38837745_HERVHF 3LTR
Hylobates_lar_LG09:112447534-112452391_HERVHF 5LTR 93.793 290 16 2 1 289 116 404 5.38E-125435 92
Hylobates_lar_LG06:38831828-38837745_HERVHF 3LTR
Hylobates_lar_LG16:101610592-101617187_HERVHF 3LTR 92.208 308 23 1 1 308 115 421 1.04E-127444 98
Hylobates_lar_LG06:38831828-38837745_HERVHF 5LTR
Hylobates_lar_LG16:101610592-101617187_HERVHF 5LTR 92.617 298 20 1 2 297 116 413 5.29E-125436 95
Hylobates_lar_LG06:38831828-38837745_HERVHF 5LTR
Hylobates_lar_LG20:16903045-16908295_HERVHF 3LTR 92.642 299 20 1 2 298 129 427 1.52E-125438 96
Hylobates_lar_LG06:38831828-38837745_HERVHF 3LTR
Hylobates_lar_LG20:16903045-16908295_HERVHF 5LTR 96.321 299 9 2 1 299 134 430 4.13E-139483 95
Hylobates_lar_LG08:128737715-128743136_HERVHF 5LTR
Hylobates_lar_LG18:30372700-30377982_HERVHF 3LTR 93.035 402 27 1 1 402 2 402 3.41E-173596 99
Hylobates_lar_LG08:128737715-128743136_HERVHF 3LTR
Hylobates_lar_LG18:30372700-30377982_HERVHF 5LTR 93.797 403 25 0 1 403 1 403 1.26E-178615 99
Hylobates_lar_LG11:110189891-110195315_HERVHF 5LTR
Hylobates_lar_LG22:51329841-51335101_HERVHF 3LTR 93.949 314 18 1 1 313 33 346 1.74E-137478 100
Hylobates_lar_LG11:110189891-110195315_HERVHF 3LTR
Hylobates_lar_LG22:51329841-51335101_HERVHF 5LTR 96.141 311 12 0 1 311 20 330 1.02E-146508 100
Hylobates_lar_LG12:55320953-55325892_HERVHF 5LTR
Hylobates_lar_LG16:101610592-101617187_HERVHF 3LTR 91.026 312 27 1 1 311 85 396 1.95E-124434 95
Hylobates_lar_LG12:55320953-55325892_HERVHF 3LTR
Hylobates_lar_LG16:101610592-101617187_HERVHF 5LTR 90.123 324 30 2 1 323 91 413 1.93E-124434 100
Hylobates_lar_LG12:55320953-55325892_HERVHF 3LTR
Hylobates_lar_LG17:76674886-76679638_HERVHF 3LTR 90.402 323 28 3 1 323 91 410 6.73E-124433 100
Hylobates_lar_LG12:55320953-55325892_HERVHF 5LTR
Hylobates_lar_LG17:76674886-76679638_HERVHF 5LTR 93.865 326 19 1 1 326 84 408 6.79E-143495 100
Hylobates_lar_LG12:55320953-55325892_HERVHF 3LTR
Hylobates_lar_LG20:16903045-16908295_HERVHF 3LTR 92.284 324 22 3 1 323 105 426 1.39E-132462 100

Hylobates_lar_LG12:55320953-55325892_HERVHF 5LTR
Hylobates_lar_LG20:16903045-16908295_HERVHF 5LTR 92.661 327 22 2 1 326 104 429 6.36E-137475 100
Hylobates_lar_LG14:9027554-9034478_HERVHF 3LTR
Hylobates_lar_LG20:16903045-16908295_HERVHF 3LTR 91.981 424 28 3 1 423 8 426 2.39E-175604 100
Hylobates_lar_LG14:9027554-9034478_HERVHF 5LTR
Hylobates_lar_LG20:16903045-16908295_HERVHF 5LTR 93.689 412 24 2 2 412 18 428 3.02E-180619 99
Hylobates_lar_LG18:1602090-1607566_HERVHF 3LTR
Hylobates_lar_LG18:30372700-30377982_HERVHF 3LTR 94.832 387 18 2 1 387 1 385 7.6E-175 601 100
Hylobates_lar_LG18:1602090-1607566_HERVHF 5LTR
Hylobates_lar_LG18:30372700-30377982_HERVHF 5LTR 93.668 379 24 0 1 379 8 386 2.96E-167576 100
Mandrillus_leucophaeus_scaffold154115:4047151-4051051_HERVHF 3LTR
Mandrillus_leucophaeus_scaffold154151:4047269-4051169_HERVHF 3LTR 100 349 0 0 1 349 1 349 0 630 100
Mandrillus_leucophaeus_scaffold154115:4047151-4051051_HERVHF 5LTR
Mandrillus_leucophaeus_scaffold154151:4047269-4051169_HERVHF 5LTR 99.723 361 1 0 1 361 1 361 0 647 100
Mandrillus_leucophaeus_scaffold245:12872571-12877377_HERVHF 3LTR
Mandrillus_leucophaeus_scaffold245:35324809-35330542_HERVHF 3LTR 92.986 442 28 2 2 440 1 442 0 652 97
Mandrillus_leucophaeus_scaffold245:12872571-12877377_HERVHF 5LTR
Mandrillus_leucophaeus_scaffold245:35324809-35330542_HERVHF 5LTR 93.363 452 29 1 1 451 2 453 0 677 99
Mandrillus_sphinx_Contig122:4604884-4610567_HERVHF 3LTR
Mandrillus_sphinx_Contig70:3296943-3302434_HERVHF 3LTR 91.729 399 26 4 1 395 1 396 2.96E-163560 100
Mandrillus_sphinx_Contig122:4604884-4610567_HERVHF 5LTR
Mandrillus_sphinx_Contig70:3296943-3302434_HERVHF 5LTR 92.5 400 24 5 1 398 1 396 5.76E-166570 100
Pan_paniscus_CM003383.1:80218929-80226889_HERVHF 3LTR
Pan_paniscus_CM003395.1:125949754-125955858_HERVHF 3LTR 92.216 334 25 1 1 334 99 431 1.84E-139482 99
Pan_paniscus_CM003383.1:80218929-80226889_HERVHF 5LTR
Pan_paniscus_CM003395.1:125949754-125955858_HERVHF 5LTR 92.216 334 25 1 1 334 98 430 1.82E-139482 100
Pan_paniscus_CM003386.1:21439220-21443575_HERVHF 3LTR
Pan_paniscus_CM003404.1:17002689-17007906_HERVHF 3LTR 92.804 403 26 2 4 403 2 404 5.95E-172590 99
Pan_paniscus_CM003386.1:21439220-21443575_HERVHF 5LTR
Pan_paniscus_CM003404.1:17002689-17007906_HERVHF 5LTR 94.059 404 23 1

3	406	3	405	4.31E-180618	99

Pan_paniscus_CM003388.1:38957485-38963391_HERVHF	5LTR	Pan_paniscus_CM003395.1:125949754-125955858_HERVHF	3LTR	94.699	415	21	1	7	420	2	416	0	646	99

Pan_paniscus_CM003388.1:38957485-38963391_HERVHF	3LTR	Pan_paniscus_CM003395.1:125949754-125955858_HERVHF	5LTR	94.444	414	23	0	1	414	2	415	0	644	100

Pan_troglodytes_NBAG03002951.1:15908-20650_HERVHF	3LTR	Pan_troglodytes_NBAG03002955.1:15884-20626_HERVHF	3LTR	100	403	0	0	1	403	1	403	0	728	100

Pan_troglodytes_NBAG03002951.1:15908-20650_HERVHF	5LTR	Pan_troglodytes_NBAG03002955.1:15884-20626_HERVHF	5LTR	100	401	0	0	1	401	1	401	0	724	100

Pongo_abelii_CM009262.2:150433372-150438172_HERVHF5LTR	Pongo_abelii_CM009265.2:69536489-69541419_HERVHF	3LTR	92.394	355	26	1	1	354	69	423	4.09E-149516	98

Pongo_abelii_CM009262.2:150433372-150438172_HERVHF3LTR	Pongo_abelii_CM009265.2:69536489-69541419_HERVHF	5LTR	93.902	328	17	2	13	337	90	417	3.68E-143496	93

Pongo_abelii_CM009262.2:20268319-20272884_HERVHF	3LTR	Pongo_abelii_CM009263.2:104709278-104714293_HERVHF3LTR	94.248	452	25	1	2	452	1	452	0	695	99

Pongo_abelii_CM009262.2:20268319-20272884_HERVHF	5LTR	Pongo_abelii_CM009263.2:104709278-104714293_HERVHF5LTR	95.354	452	20	1	4	454	1	452	0	718	99

Pongo_abelii_CM009262.2:20268319-20272884_HERVHF	3LTR	Pongo_abelii_CM009264.2:93735033-93740006_HERVHF	3LTR	94.027	452	26	1	2	452	1	452	0	691	99

Pongo_abelii_CM009262.2:20268319-20272884_HERVHF	5LTR	Pongo_abelii_CM009264.2:93735033-93740006_HERVHF	5LTR	94.469	452	24	1	4	454	1	452	0	700	99

Pongo_abelii_CM009265.2:189763222-189768502_HERVHF3LTR	Pongo_abelii_CM009266.2:114896582-114901546_HERVHF3LTR	92.219	347	25	2	11	357	40	384	2.68E-143498	97

Pongo_abelii_CM009265.2:189763222-189768502_HERVHF5LTR	Pongo_abelii_CM009266.2:114896582-114901546_HERVHF5LTR	91.908	346	25	3	1	345	28	371	1.34E-140488	100

Pongo_abelii_CM009265.2:189763222-189768502_HERVHF3LTR	Pongo_abelii_CM009266.2:130784985-130789829_HERVHF3LTR	91.185	363	20	6	1	357	52	408	9.34E-143495	100

Pongo_abelii_CM009265.2:189763222-189768502_HERVHF5LTR	Pongo_abelii_CM009266.2:130784985-130789829_HERVHF5LTR	92.045	352	20	4	1	345	41	391	7.39E-144499	100

Pongo_abelii_CM009265.2:189763222-189768502_HERVHF3LTR

Pongo_abelii_CM009266.2:137847220-137852125_HERVHF 3LTR 91.036 357 19 4
1 357 28 371 3.26E-142494 100
Pongo_abelii_CM009265.2:189763222-189768502_HERVHF 5LTR
Pongo_abelii_CM009266.2:137847220-137852125_HERVHF 5LTR 91.066 347 19 3
1 345 27 363 1.63E-139484 100
Pongo_abelii_CM009265.2:189763222-189768502_HERVHF 3LTR
Pongo_abelii_CM009270.2:678761-683551_HERVHF 3LTR 91.877 357 22 5 1
357 29 378 2.68E-143498 100
Pongo_abelii_CM009265.2:189763222-189768502_HERVHF 5LTR
Pongo_abelii_CM009270.2:678761-683551_HERVHF 5LTR 92.754 345 22 3 1
343 28 371 7.39E-144499 99
Pongo_abelii_CM009266.2:116229328-116234274_HERVHF 5LTR
Pongo_abelii_CM009266.2:137847220-137852125_HERVHF 3LTR 96.078 306 12 0
7 312 1 306 6.65E-144499 98
Pongo_abelii_CM009266.2:116229328-116234274_HERVHF 3LTR
Pongo_abelii_CM009266.2:137847220-137852125_HERVHF 5LTR 94.855 311 14 2
8 317 1 310 5.22E-139482 97
Pongo_abelii_CM009266.2:133993424-133998349_HERVHF 3LTR
Pongo_abelii_CM009266.2:137847220-137852125_HERVHF 3LTR 94.118 289 15 2
1 287 115 403 1.87E-125438 93
Pongo_abelii_CM009266.2:133993424-133998349_HERVHF 5LTR
Pongo_abelii_CM009266.2:137847220-137852125_HERVHF 5LTR 95.374 281 11 2
1 279 123 403 1.5E-126 442 93
Pongo_abelii_CM009266.2:166936585-166941496_HERVHF 5LTR
Pongo_abelii_CM009268.2:139999900-140006933_HERVHF 3LTR 92.768 401 26 2
1 399 1 400 2.16E-170587 100
Pongo_abelii_CM009266.2:166936585-166941496_HERVHF 3LTR
Pongo_abelii_CM009268.2:139999900-140006933_HERVHF 5LTR 93.97 398 23 1
2 399 1 397 2.32E-176607 99
Pongo_abelii_CM009266.2:170888490-170893531_HERVHF 3LTR
Pongo_abelii_CM009266.2:26777284-26782315_HERVHF 3LTR 92.257 452 32 3
1 450 2 452 0 647 100
Pongo_abelii_CM009266.2:170888490-170893531_HERVHF 5LTR
Pongo_abelii_CM009266.2:26777284-26782315_HERVHF 5LTR 92.494 453 30 3
7 457 1 451 0 655 98
Pongo_abelii_CM009266.2:170888490-170893531_HERVHF 3LTR
Pongo_abelii_CM009267.2:117783136-117788261_HERVHF 3LTR 92.035 452 32 4
2 450 2 452 0 639 99
Pongo_abelii_CM009266.2:170888490-170893531_HERVHF 5LTR
Pongo_abelii_CM009267.2:117783136-117788261_HERVHF 5LTR 92.07 454 33 2
8 458 1 454 0 651 98
Pongo_abelii_CM009266.2:170888490-170893531_HERVHF 5LTR
Pongo_abelii_CM009269.2:81495901-81500897_HERVHF 3LTR 93.85 439 25 1
8 444 1 439 0 668 95

Pongo_abelii_CM009266.2:170888490-170893531_HERVHF 3LTR
Pongo_abelii_CM009269.2:81495901-81500897_HERVHF 5LTR 92.239 451 33 2 2 450 2 452 0 649 99
Pongo_abelii_CM009269.2:49137324-49142107_HERVHF 3LTR
Pongo_abelii_CM009269.2:60854253-60859315_HERVHF 3LTR 96.11 437 17 0 3 439 1 437 0 712 99
Pongo_abelii_CM009269.2:49137324-49142107_HERVHF 5LTR
Pongo_abelii_CM009269.2:60854253-60859315_HERVHF 5LTR 96.339 437 14 2 4 439 1 436 0 710 99
Pongo_abelii_CM009271.2:100985548-100990741_HERVHF 5LTR
Pongo_abelii_CM009275.2:64744675-64749263_HERVHF 3LTR 96.452 451 14 2 1 451 5 453 0 735 100
Pongo_abelii_CM009271.2:100985548-100990741_HERVHF 3LTR
Pongo_abelii_CM009275.2:64744675-64749263_HERVHF 5LTR 95.796 452 19 0 1 452 1 452 0 730 100
Pongo_abelii_CM009277.2:80883698-80888716_HERVHF 5LTR
Pongo_abelii_CM009283.2:13770595-13775495_HERVHF 3LTR 93.955 397 23 1 27 422 1 397 5.66E-176605 92
Pongo_abelii_CM009277.2:80883698-80888716_HERVHF 3LTR
Pongo_abelii_CM009283.2:13770595-13775495_HERVHF 5LTR 93.317 404 25 2 23 424 1 404 2.38E-174600 95
Rhinopithecus_roxellana_CM017354.1:24650719-24659249_HERVIPADP 3LTR
Rhinopithecus_roxellana_CM017354.1:24740588-24749134_HERVIPADP 3LTR 96.491 399 14 0 1 399 1 399 0 657 99
Rhinopithecus_roxellana_CM017354.1:24650719-24659249_HERVIPADP 5LTR
Rhinopithecus_roxellana_CM017354.1:24740588-24749134_HERVIPADP 5LTR 91.667 396 33 0 1 396 1 396 5.61E-165566 100
Rhinopithecus_roxellana_CM017359.1:126642581-126647888_HERVHF 3LTR
Rhinopithecus_roxellana_CM017368.1:43723812-43728715_HERVHF 3LTR 90.506 316 27 3 1 314 44 358 4.24E-123425 100
Rhinopithecus_roxellana_CM017359.1:126642581-126647888_HERVHF 5LTR
Rhinopithecus_roxellana_CM017368.1:43723812-43728715_HERVHF 5LTR 91.346 312 22 4 1 309 46 355 3.43E-124428 99

Supplementary data S8 The alignments of the 5LTR sequences of HERV in Figure 2B

>Homo_sapiens_Y:4355841-4360781_HERVHF:5LTR(-)
tcaggcctctgagcccaagccaagccatcgcatcccctgtcacttgcacatatatgcccagatggcctgaagtaactgaagaatcacaaaagaagtg
aatatgccctgccccaccttaactgatgacattccaccacaacagaagtataaatggctggtccttgccttaagtgatgacattatcttgtgaaagtcctttt
cctgctcatcatggctcaaaaagcacccccccactgagcaccttgtgacccccactcctgcccgccagagaacaacctccctttgactgtaattttccttt
acctacccaaatcctataaaacggccccacccctatctcccttcactgactctcttttcggactcagcccacctgcacccaggtgaaataaacagcctc
gttgctcacacaaagcctgtttggcggtctcttcacatggatgcgcatgaa
>Homo_sapiens_X:90773760-90778707_HERVHF:5LTR(-)
tcaggcctctgagcccaagccaagccatcgcatcccctgtgacttgcacggatacgaccagatggccggaagtaactgaaaaatcacaaaagaagt
gaatatgccctgccccaccttaactgatgacattccaccacaacagaagtataaatggctggtctttgccttaagtgatgacattatcttgtgaaagtcctt
ttcctgctcatcatggctcaaaaagca-cccccactgagcaccttgtgacccctactcctgcccgccagagaacaaccccccctttgactgtaattttccta
tacctacccaaatcctataaaacggccccacccctatcttccttcactga--ctcttttcagactcagcccacctgcacccaggtgaaataaacagcctc
gttgctcacacaaagcctgtttggtggtctcttcacacggaagtgcatga-

Supplementary data S9 The alignments of the 3LTR sequences of HERV in Figure 2B

>Homo_sapiens_X:90773760-90778707_HERVHF:3LTR(-)
tcaggcctctgagcccaagccaagccatcgcatcccctgtcacttgcacgtatacacccagatggcctgaagtaactgaagaatcacaaaagaagtg
aatacaccctgccccaccttaactgatgacattccaccacaaaagaagtgcaaatggccggtccttgccttaagtgatgacattaccttgtgaaagtcct
tttcctggctcatcctggctcaaaaagcacccccactgagcaccttgcgacccccactcctgcccttcagagaacaaccccccctttgactataattttcct
ttacctacccaaatcctataaaacagccccacccctatctcccttcactgactctcttttcggactcagcccgcctgcacccaggtgaaataaacagcca
tgttgctcacacaaagcctgtttggtggtctcttcacacagatgcgcatg--
>Homo_sapiens_Y:4355841-4360781_HERVHF:3LTR(-)
tcaggcctctgagcccaagccaagccatcgcatcccctgtcacttgcacgtatacacccagatggcctgaagtaactgaagaatcacaaaagaagtg
aatacgccctgccct-ccttaactgatgacattccaccacaaaagaagtgcaaacggccggtccttgccttaactgatgacattaccttgtgaaagtcctt
ttcctggctcatcctggctcaaaaagcacccccactgagcaccttgcaacccccactcctgccctccagagaacaaccccccctttgactataattttcctt
tacctacccaaatcctataaaacagccccacccctaactcccttcgctgactctcttttttggactcagcccgcctgcacccaggtgaaataaacagcca
cgttgctcacacaaagcctgtttggtggtctcttcacacggatgcgcatgaa

Supplementary data S10 The relationship between HERVs in human genomes and Human ncRNA

chr1	S1	E1	HERVname	O1	chr2	S2	E2	genename	O2	merged-length
1	22997488	22997912	Homo_sapiens_1_23000272-23002212-HERVHF-5LTR	-	1	22983561	23004042	NONHSAG057580.1	-	424
1	22997488	22997912	Homo_sapiens_1_23000272-23002212-HERVHF-5LTR	-	1	22983561	23004042	NONHSAT151253.1	-	424
1	22998530	23001867	Homo_sapiens_1_23000272-23002212-HERVHF-pol	-	1	22983561	23004042	NONHSAG057580.1	-	3337
1	22998530	23001867	Homo_sapiens_1_23000272-23002212-HERVHF-pol	-	1	22983561	23004042	NONHSAT151253.1	-	3337
1	23002236	23002547	Homo_sapiens_1_23000272-23002212-HERVHF-gag	-	1	22983561	23004042	NONHSAG057580.1	-	311
1	23002236	23002547	Homo_sapiens_1_23000272-23002212-HERVHF-gag	-	1	22983561	23004042	NONHSAT151253.1	-	311
1	43085533	43091095	Homo_sapiens_1_43087972-43089561-HERVHF	+	1	42958276	43196506	NONHSAG058967.2	+	5562
1	43085533	43085862	Homo_sapiens_1_43087972-43089561-HERVHF-5LTR	+	1	42958276	43196506	NONHSAG058967.2	+	329
1	43087974	43088405	Homo_sapiens_1_43087972-43089561-HERVHF-pro	+	1	42958276	43196506	NONHSAG058967.2	+	431
1	43088444	43090183	Homo_sapiens_1_43087972-43089561-HERVHF-pol	+	1	42958276	43196506	NONHSAG058967.2	+	1739
1	43090771	43091095	Homo_sapiens_1_43087972-43089561-HERVHF-3LTR	+	1	42958276	43196506	NONHSAG058967.2	+	324
1	43085533	43091095	Homo_sapiens_1_43087972-43089561-HERVHF	+	1	42959045	43196506	NONHSAT224980.1	+	5562
1	43085533	43085862	Homo_sapiens_1_43087972-43089561-HERVHF-5LTR	+	1	42959045	43196506	NONHSAT224980.1	+	329
1	43087974	43088405	Homo_sapiens_1_43087972-43089561-HERVHF-pro	+	1	42959045	43196506	NONHSAT224980.1	+	431
1	43088444	43090183	Homo_sapiens_1_43087972-43089561-HERVHF-pol	+	1	42959045	43196506	NONHSAT224980.1	+	1739
1	43090771	43091095	Homo_sapiens_1_43087972-43089561-HERVHF-3LTR	+	1	42959045	43196506	NONHSAT224980.1	+	324
1	43087974	43088405	Homo_sapiens_1_43087972-43089561-HERVHF-pro	+	1	43087944	43092069	NONHSAG056389.1	+	431
1	43087974	43088405	Homo_sapiens_1_43087972-43089561-HERVHF-pro	+	1	43087944	43092069	NONHSAT148830.1	+	431
1	43088444	43090183	Homo_sapiens_1_43087972-43089561-HERVHF-pol	+	1	43087944	43092069	NONHSAG056389.1	+	1739
1	43088444	43090183	Homo_sapiens_1_43087972-43089561-HERVHF-pol	+	1					

43087944 43092069 NONHSAT148830.1 + 1739
1 43090771 43091095 Homo_sapiens_1_43087972-43089561-HERVHF-3LTR + 1
43087944 43092069 NONHSAG056389.1 + 324
1 43090771 43091095 Homo_sapiens_1_43087972-43089561-HERVHF-3LTR + 1
43087944 43092069 NONHSAT148830.1 + 324
1 68386003 68391994 Homo_sapiens_1_68388791-68390135-HERVHF + 1 68385473
68484266 NONHSAG001773.3 + 5991
1 68386003 68386439 Homo_sapiens_1_68388791-68390135-HERVHF-5LTR + 1
68385473 68484266 NONHSAG001773.3 + 436
1 68386003 68386439 Homo_sapiens_1_68388791-68390135-HERVHF-5LTR + 1
68385473 68391984 NONHSAT225125.1 + 436
1 68386003 68391994 Homo_sapiens_1_68388791-68390135-HERVHF + 1 68385473
68451507 NONHSAT225126.1 + 5991
1 68386003 68386439 Homo_sapiens_1_68388791-68390135-HERVHF-5LTR + 1
68385473 68451507 NONHSAT225126.1 + 436
1 68386003 68391994 Homo_sapiens_1_68388791-68390135-HERVHF + 1 68385473
68468517 NONHSAT225127.1 + 5991
1 68386003 68386439 Homo_sapiens_1_68388791-68390135-HERVHF-5LTR + 1
68385473 68468517 NONHSAT225127.1 + 436
1 68386003 68391994 Homo_sapiens_1_68388791-68390135-HERVHF + 1 68385473
68484266 NONHSAT225128.1 + 5991
1 68386003 68386439 Homo_sapiens_1_68388791-68390135-HERVHF-5LTR + 1
68385473 68484266 NONHSAT225128.1 + 436
1 68388421 68388741 Homo_sapiens_1_68388791-68390135-HERVHF-gag + 1
68385473 68484266 NONHSAG001773.3 + 320
1 68388421 68388741 Homo_sapiens_1_68388791-68390135-HERVHF-gag + 1
68385473 68391984 NONHSAT225125.1 + 320
1 68388421 68388741 Homo_sapiens_1_68388791-68390135-HERVHF-gag + 1
68385473 68451507 NONHSAT225126.1 + 320
1 68388421 68388741 Homo_sapiens_1_68388791-68390135-HERVHF-gag + 1
68385473 68468517 NONHSAT225127.1 + 320
1 68388421 68388741 Homo_sapiens_1_68388791-68390135-HERVHF-gag + 1
68385473 68484266 NONHSAT225128.1 + 320
1 68389169 68391111 Homo_sapiens_1_68388791-68390135-HERVHF-pol + 1
68385473 68484266 NONHSAG001773.3 + 1942
1 68389169 68391111 Homo_sapiens_1_68388791-68390135-HERVHF-pol + 1
68385473 68391984 NONHSAT225125.1 + 1942
1 68389169 68391111 Homo_sapiens_1_68388791-68390135-HERVHF-pol + 1
68385473 68451507 NONHSAT225126.1 + 1942
1 68389169 68391111 Homo_sapiens_1_68388791-68390135-HERVHF-pol + 1
68385473 68468517 NONHSAT225127.1 + 1942
1 68389169 68391111 Homo_sapiens_1_68388791-68390135-HERVHF-pol + 1
68385473 68484266 NONHSAT225128.1 + 1942
1 68391558 68391994 Homo_sapiens_1_68388791-68390135-HERVHF-3LTR + 1

68385473 68484266 NONHSAG001773.3 + 436
1 68391558 68391994 Homo_sapiens_1_68388791-68390135-HERVHF-3LTR + 1
68385473 68451507 NONHSAT225126.1 + 436
1 68391558 68391994 Homo_sapiens_1_68388791-68390135-HERVHF-3LTR + 1
68385473 68468517 NONHSAT225127.1 + 436
1 68391558 68391994 Homo_sapiens_1_68388791-68390135-HERVHF-3LTR + 1
68385473 68484266 NONHSAT225128.1 + 436
1 82354581 82360561 Homo_sapiens_1_82356924-82357985-HERVHF - 1 82212413
82848205 ENSG00000233290 - 5980
1 82354581 82354965 Homo_sapiens_1_82356924-82357985-HERVHF-5LTR - 1
82212413 82848205 ENSG00000233290 - 384
1 82354581 82360561 Homo_sapiens_1_82356924-82357985-HERVHF - 1 82212413
82814210 ENSG00000233290 - 5980
1 82354581 82354965 Homo_sapiens_1_82356924-82357985-HERVHF-5LTR - 1
82212413 82814210 ENSG00000233290 - 384
1 82355816 82357821 Homo_sapiens_1_82356924-82357985-HERVHF-pol - 1
82212413 82848205 ENSG00000233290 - 2005
1 82355816 82357821 Homo_sapiens_1_82356924-82357985-HERVHF-pol - 1
82212413 82814210 ENSG00000233290 - 2005
1 82357279 82358037 Homo_sapiens_1_82356924-82357985-HERVHF-pro - 1
82212413 82848205 ENSG00000233290 - 758
1 82357279 82358037 Homo_sapiens_1_82356924-82357985-HERVHF-pro - 1
82212413 82814210 ENSG00000233290 - 758
1 82360176 82360561 Homo_sapiens_1_82356924-82357985-HERVHF-3LTR - 1
82212413 82848205 ENSG00000233290 - 385
1 82360176 82360561 Homo_sapiens_1_82356924-82357985-HERVHF-3LTR - 1
82212413 82814210 ENSG00000233290 - 385
1 82355816 82357821 Homo_sapiens_1_82356924-82357985-HERVHF-pol - 1
82354967 82362589 NONHSAG001922.2 - 2005
1 82355816 82357821 Homo_sapiens_1_82356924-82357985-HERVHF-pol - 1
82354967 82362589 NONHSAT004141.2 - 2005
1 82357279 82358037 Homo_sapiens_1_82356924-82357985-HERVHF-pro - 1
82354967 82362589 NONHSAG001922.2 - 758
1 82357279 82358037 Homo_sapiens_1_82356924-82357985-HERVHF-pro - 1
82354967 82362589 NONHSAT004141.2 - 758
1 82360176 82360561 Homo_sapiens_1_82356924-82357985-HERVHF-3LTR - 1
82354967 82362589 NONHSAG001922.2 - 385
1 82360176 82360561 Homo_sapiens_1_82356924-82357985-HERVHF-3LTR - 1
82354967 82362589 NONHSAT004141.2 - 385
1 82955299 82961592 Homo_sapiens_1_82956772-82959208-HERVHF + 1 82903182
83180821 NONHSAG001926.2 + 6293
1 82955299 82955725 Homo_sapiens_1_82956772-82959208-HERVHF-5LTR + 1
82903182 83180821 NONHSAG001926.2 + 426
1 82955299 82961592 Homo_sapiens_1_82956772-82959208-HERVHF + 1 82903182

83166815 NONHSAT004147.2 + 6293
1 82955299 82955725 Homo_sapiens_1_82956772-82959208-HERVHF-5LTR + 1
82903182 83166815 NONHSAT004147.2 + 426
1 82957379 82957759 Homo_sapiens_1_82956772-82959208-HERVHF-gag + 1
82903182 83180821 NONHSAG001926.2 + 380
1 82957379 82957759 Homo_sapiens_1_82956772-82959208-HERVHF-gag + 1
82903182 83166815 NONHSAT004147.2 + 380
1 82957868 82958632 Homo_sapiens_1_82956772-82959208-HERVHF-pro + 1
82903182 83180821 NONHSAG001926.2 + 764
1 82957868 82958632 Homo_sapiens_1_82956772-82959208-HERVHF-pro + 1
82903182 83166815 NONHSAT004147.2 + 764
1 82958120 82960341 Homo_sapiens_1_82956772-82959208-HERVHF-pol + 1
82903182 83180821 NONHSAG001926.2 + 2221
1 82958120 82960341 Homo_sapiens_1_82956772-82959208-HERVHF-pol + 1
82903182 83166815 NONHSAT004147.2 + 2221
1 82961176 82961592 Homo_sapiens_1_82956772-82959208-HERVHF-3LTR + 1
82903182 83180821 NONHSAG001926.2 + 416
1 82961176 82961592 Homo_sapiens_1_82956772-82959208-HERVHF-3LTR + 1
82903182 83166815 NONHSAT004147.2 + 416
1 82955299 82961592 Homo_sapiens_1_82956772-82959208-HERVHF + 1 82903183
83166815 ENSG00000230817 + 6293
1 82955299 82955725 Homo_sapiens_1_82956772-82959208-HERVHF-5LTR + 1
82903183 83166815 ENSG00000230817 + 426
1 82955299 82961592 Homo_sapiens_1_82956772-82959208-HERVHF + 1 82903183
83180821 NONHSAT149257.1 + 6293
1 82955299 82955725 Homo_sapiens_1_82956772-82959208-HERVHF-5LTR + 1
82903183 83180821 NONHSAT149257.1 + 426
1 82957379 82957759 Homo_sapiens_1_82956772-82959208-HERVHF-gag + 1
82903183 83166815 ENSG00000230817 + 380
1 82957379 82957759 Homo_sapiens_1_82956772-82959208-HERVHF-gag + 1
82903183 83180821 NONHSAT149257.1 + 380
1 82957868 82958632 Homo_sapiens_1_82956772-82959208-HERVHF-pro + 1
82903183 83166815 ENSG00000230817 + 764
1 82957868 82958632 Homo_sapiens_1_82956772-82959208-HERVHF-pro + 1
82903183 83180821 NONHSAT149257.1 + 764
1 82958120 82960341 Homo_sapiens_1_82956772-82959208-HERVHF-pol + 1
82903183 83166815 ENSG00000230817 + 2221
1 82958120 82960341 Homo_sapiens_1_82956772-82959208-HERVHF-pol + 1
82903183 83180821 NONHSAT149257.1 + 2221
1 82961176 82961592 Homo_sapiens_1_82956772-82959208-HERVHF-3LTR + 1
82903183 83166815 ENSG00000230817 + 416
1 82961176 82961592 Homo_sapiens_1_82956772-82959208-HERVHF-3LTR + 1
82903183 83180821 NONHSAT149257.1 + 416
1 82955299 82961592 Homo_sapiens_1_82956772-82959208-HERVHF + 1 82903201

83097023 ENSG00000230817 + 6293
1 82955299 82955725 Homo_sapiens_1_82956772-82959208-HERVHF-5LTR + 1
82903201 83097023 ENSG00000230817 + 426
1 82957379 82957759 Homo_sapiens_1_82956772-82959208-HERVHF-gag + 1
82903201 83097023 ENSG00000230817 + 380
1 82957868 82958632 Homo_sapiens_1_82956772-82959208-HERVHF-pro + 1
82903201 83097023 ENSG00000230817 + 764
1 82958120 82960341 Homo_sapiens_1_82956772-82959208-HERVHF-pol + 1
82903201 83097023 ENSG00000230817 + 2221
1 82961176 82961592 Homo_sapiens_1_82956772-82959208-HERVHF-3LTR + 1
82903201 83097023 ENSG00000230817 + 416
1 82955299 82961592 Homo_sapiens_1_82956772-82959208-HERVHF + 1 82903220
83064102 ENSG00000230817 + 6293
1 82955299 82955725 Homo_sapiens_1_82956772-82959208-HERVHF-5LTR + 1
82903220 83064102 ENSG00000230817 + 426
1 82957379 82957759 Homo_sapiens_1_82956772-82959208-HERVHF-gag + 1
82903220 83064102 ENSG00000230817 + 380
1 82957868 82958632 Homo_sapiens_1_82956772-82959208-HERVHF-pro + 1
82903220 83064102 ENSG00000230817 + 764
1 82958120 82960341 Homo_sapiens_1_82956772-82959208-HERVHF-pol + 1
82903220 83064102 ENSG00000230817 + 2221
1 82961176 82961592 Homo_sapiens_1_82956772-82959208-HERVHF-3LTR + 1
82903220 83064102 ENSG00000230817 + 416
1 82955299 82961592 Homo_sapiens_1_82956772-82959208-HERVHF + 1 82907560
82997561 NONHSAT004148.2 + 6293
1 82955299 82955725 Homo_sapiens_1_82956772-82959208-HERVHF-5LTR + 1
82907560 82997561 NONHSAT004148.2 + 426
1 82957379 82957759 Homo_sapiens_1_82956772-82959208-HERVHF-gag + 1
82907560 82997561 NONHSAT004148.2 + 380
1 82957868 82958632 Homo_sapiens_1_82956772-82959208-HERVHF-pro + 1
82907560 82997561 NONHSAT004148.2 + 764
1 82958120 82960341 Homo_sapiens_1_82956772-82959208-HERVHF-pol + 1
82907560 82997561 NONHSAT004148.2 + 2221
1 82961176 82961592 Homo_sapiens_1_82956772-82959208-HERVHF-3LTR + 1
82907560 82997561 NONHSAT004148.2 + 416
1 82955299 82961592 Homo_sapiens_1_82956772-82959208-HERVHF + 1 82907561
82997561 NONHSAT004149.2 + 6293
1 82955299 82955725 Homo_sapiens_1_82956772-82959208-HERVHF-5LTR + 1
82907561 82997561 NONHSAT004149.2 + 426
1 82957379 82957759 Homo_sapiens_1_82956772-82959208-HERVHF-gag + 1
82907561 82997561 NONHSAT004149.2 + 380
1 82957868 82958632 Homo_sapiens_1_82956772-82959208-HERVHF-pro + 1
82907561 82997561 NONHSAT004149.2 + 764
1 82958120 82960341 Homo_sapiens_1_82956772-82959208-HERVHF-pol + 1

82907561 82997561 NONHSAT004149.2 + 2221
1 82961176 82961592 Homo_sapiens_1_82956772-82959208-HERVHF-3LTR + 1
82907561 82997561 NONHSAT004149.2 + 416
1 82955299 82961592 Homo_sapiens_1_82956772-82959208-HERVHF + 1 82907562
82997561 ENSG00000230817 + 6293
1 82955299 82955725 Homo_sapiens_1_82956772-82959208-HERVHF-5LTR + 1
82907562 82997561 ENSG00000230817 + 426
1 82957379 82957759 Homo_sapiens_1_82956772-82959208-HERVHF-gag + 1
82907562 82997561 ENSG00000230817 + 380
1 82957868 82958632 Homo_sapiens_1_82956772-82959208-HERVHF-pro + 1
82907562 82997561 ENSG00000230817 + 764
1 82958120 82960341 Homo_sapiens_1_82956772-82959208-HERVHF-pol + 1
82907562 82997561 ENSG00000230817 + 2221
1 82961176 82961592 Homo_sapiens_1_82956772-82959208-HERVHF-3LTR + 1
82907562 82997561 ENSG00000230817 + 416
1 209451675 209453142 Homo_sapiens_1_209451456-209452546-HERVHF-pol + 1
209449432 209499156 NONHSAG057153.1 + 1467
1 209451675 209453142 Homo_sapiens_1_209451456-209452546-HERVHF-pol + 1
209449432 209499156 NONHSAT150429.1 + 1467
1 209453552 209454012 Homo_sapiens_1_209451456-209452546-HERVHF-3LTR +
1 209449432 209499156 NONHSAG057153.1 + 460
1 209453552 209454012 Homo_sapiens_1_209451456-209452546-HERVHF-3LTR +
1 209449432 209499156 NONHSAT150429.1 + 460
1 224840009 224846095 Homo_sapiens_1_224842067-224843662-HERVHF - 1
224802959 224881355 ENSG00000286719 - 6086
1 224840009 224840329 Homo_sapiens_1_224842067-224843662-HERVHF-5LTR -
1 224802959 224881355 ENSG00000286719 - 320
1 224841379 224843462 Homo_sapiens_1_224842067-224843662-HERVHF-pol - 1
224802959 224881355 ENSG00000286719 - 2083
1 224842881 224843657 Homo_sapiens_1_224842067-224843662-HERVHF-pro - 1
224802959 224881355 ENSG00000286719 - 776
1 224845764 224846095 Homo_sapiens_1_224842067-224843662-HERVHF-3LTR -
1 224802959 224881355 ENSG00000286719 - 331
1 228942542 228942868 Homo_sapiens_1_228948757-228950104-HERVHF-5LTR +
1 228941467 228945760 NONHSAG057288.1 + 326
1 228942542 228942868 Homo_sapiens_1_228948757-228950104-HERVHF-5LTR +
1 228941467 228945637 NONHSAT150689.1 + 326
1 228942542 228942868 Homo_sapiens_1_228948757-228950104-HERVHF-5LTR +
1 228941640 228945760 NONHSAT150690.1 + 326
1 232120241 232120654 Homo_sapiens_1_232120704-232121847-HERVHF-gag + 1
232118508 232186796 NONHSAG004630.2 + 413
1 232120241 232120654 Homo_sapiens_1_232120704-232121847-HERVHF-gag + 1
232118508 232186796 NONHSAT010200.2 + 413
1 232120661 232121512 Homo_sapiens_1_232120704-232121847-HERVHF-pro + 1

232118508	232186796	NONHSAG004630.2	+	851
1	232120661	232121512	Homo_sapiens_1_232120704-232121847-HERVHF-pro +	1
232118508	232186796	NONHSAT010200.2	+	851
1	232120994	232122891	Homo_sapiens_1_232120704-232121847-HERVHF-pol +	1
232118508	232186796	NONHSAG004630.2	+	1897
1	232120994	232122891	Homo_sapiens_1_232120704-232121847-HERVHF-pol +	1
232118508	232186796	NONHSAT010200.2	+	1897
1	232123477	232123943	Homo_sapiens_1_232120704-232121847-HERVHF-3LTR	+
1	232118508	232186796	NONHSAG004630.2	+	466
1	232123477	232123943	Homo_sapiens_1_232120704-232121847-HERVHF-3LTR	+
1	232118508	232186796	NONHSAT010200.2	+	466
1	241433890	241439885	Homo_sapiens_1_241436435-241438237-HERVHF	+	1
241413716	241450482	ENSG00000287516 +	5995
1	241433890	241434298	Homo_sapiens_1_241436435-241438237-HERVHF-5LTR	+
1	241413716	241450482	ENSG00000287516 +	408
1	241436142	241436447	Homo_sapiens_1_241436435-241438237-HERVHF-gag +	1
241413716	241450482	ENSG00000287516 +	305
1	241436737	241439030	Homo_sapiens_1_241436435-241438237-HERVHF-pol +	1
241413716	241450482	ENSG00000287516 +	2293
1	241439482	241439885	Homo_sapiens_1_241436435-241438237-HERVHF-3LTR	+
1	241413716	241450482	ENSG00000287516 +	403
1	241433890	241439885	Homo_sapiens_1_241436435-241438237-HERVHF	+	1
241413742	241450400	ENSG00000287516 +	5995
1	241433890	241434298	Homo_sapiens_1_241436435-241438237-HERVHF-5LTR	+
1	241413742	241450400	ENSG00000287516 +	408
1	241436142	241436447	Homo_sapiens_1_241436435-241438237-HERVHF-gag +	1
241413742	241450400	ENSG00000287516 +	305
1	241436737	241439030	Homo_sapiens_1_241436435-241438237-HERVHF-pol +	1
241413742	241450400	ENSG00000287516 +	2293
1	241439482	241439885	Homo_sapiens_1_241436435-241438237-HERVHF-3LTR	+
1	241413742	241450400	ENSG00000287516 +	403
1	241433890	241439885	Homo_sapiens_1_241436435-241438237-HERVHF	+	1
241413750	241464542	ENSG00000287516 +	5995
1	241433890	241434298	Homo_sapiens_1_241436435-241438237-HERVHF-5LTR	+
1	241413750	241464542	ENSG00000287516 +	408
1	241433890	241439885	Homo_sapiens_1_241436435-241438237-HERVHF	+	1
241413750	241464722	ENSG00000287516 +	5995
1	241433890	241434298	Homo_sapiens_1_241436435-241438237-HERVHF-5LTR	+
1	241413750	241464722	ENSG00000287516 +	408
1	241433890	241439885	Homo_sapiens_1_241436435-241438237-HERVHF	+	1
241413750	241468661	ENSG00000287516 +	5995
1	241433890	241434298	Homo_sapiens_1_241436435-241438237-HERVHF-5LTR	+
1	241413750	241468661	ENSG00000287516 +	408
1	241433890	241439885	Homo_sapiens_1_241436435-241438237-HERVHF	+	1

241413750	241450415	ENSG00000287516 +	5995
1	241433890	241434298	Homo_sapiens_1_241436435-241438237-HERVHF-5LTR	+
1	241413750	241450415	ENSG00000287516 +	408
1	241436142	241436447	Homo_sapiens_1_241436435-241438237-HERVHF-gag +	1
241413750	241464542	ENSG00000287516 +	305
1	241436142	241436447	Homo_sapiens_1_241436435-241438237-HERVHF-gag +	1
241413750	241464722	ENSG00000287516 +	305
1	241436142	241436447	Homo_sapiens_1_241436435-241438237-HERVHF-gag +	1
241413750	241468661	ENSG00000287516 +	305
1	241436142	241436447	Homo_sapiens_1_241436435-241438237-HERVHF-gag +	1
241413750	241450415	ENSG00000287516 +	305
1	241436737	241439030	Homo_sapiens_1_241436435-241438237-HERVHF-pol +	1
241413750	241464542	ENSG00000287516 +	2293
1	241436737	241439030	Homo_sapiens_1_241436435-241438237-HERVHF-pol +	1
241413750	241464722	ENSG00000287516 +	2293
1	241436737	241439030	Homo_sapiens_1_241436435-241438237-HERVHF-pol +	1
241413750	241468661	ENSG00000287516 +	2293
1	241436737	241439030	Homo_sapiens_1_241436435-241438237-HERVHF-pol +	1
241413750	241450415	ENSG00000287516 +	2293
1	241439482	241439885	Homo_sapiens_1_241436435-241438237-HERVHF-3LTR	+
1	241413750	241464542	ENSG00000287516 +	403
1	241439482	241439885	Homo_sapiens_1_241436435-241438237-HERVHF-3LTR	+
1	241413750	241464722	ENSG00000287516 +	403
1	241439482	241439885	Homo_sapiens_1_241436435-241438237-HERVHF-3LTR	+
1	241413750	241468661	ENSG00000287516 +	403
1	241439482	241439885	Homo_sapiens_1_241436435-241438237-HERVHF-3LTR	+
1	241413750	241450415	ENSG00000287516 +	403
1	241433890	241439885	Homo_sapiens_1_241436435-241438237-HERVHF	+	1
241413769	241458955	ENSG00000287516 +	5995
1	241433890	241434298	Homo_sapiens_1_241436435-241438237-HERVHF-5LTR	+
1	241413769	241458955	ENSG00000287516 +	408
1	241436142	241436447	Homo_sapiens_1_241436435-241438237-HERVHF-gag +	1
241413769	241458955	ENSG00000287516 +	305
1	241436737	241439030	Homo_sapiens_1_241436435-241438237-HERVHF-pol +	1
241413769	241458955	ENSG00000287516 +	2293
1	241439482	241439885	Homo_sapiens_1_241436435-241438237-HERVHF-3LTR	+
1	241413769	241458955	ENSG00000287516 +	403
1	241433890	241439885	Homo_sapiens_1_241436435-241438237-HERVHF	+	1
241423795	241450400	ENSG00000287516 +	5995
1	241433890	241434298	Homo_sapiens_1_241436435-241438237-HERVHF-5LTR	+
1	241423795	241450400	ENSG00000287516 +	408
1	241436142	241436447	Homo_sapiens_1_241436435-241438237-HERVHF-gag +	1
241423795	241450400	ENSG00000287516 +	305
1	241436737	241439030	Homo_sapiens_1_241436435-241438237-HERVHF-pol +	1

241423795 241450400 ENSG00000287516 + 2293
1 241439482 241439885 Homo_sapiens_1_241436435-241438237-HERVHF-3LTR +
1 241423795 241450400 ENSG00000287516 + 403
1 241433890 241439885 Homo_sapiens_1_241436435-241438237-HERVHF + 1
241424233 241450400 ENSG00000287516 + 5995
1 241433890 241434298 Homo_sapiens_1_241436435-241438237-HERVHF-5LTR +
1 241424233 241450400 ENSG00000287516 + 408
1 241436142 241436447 Homo_sapiens_1_241436435-241438237-HERVHF-gag + 1
241424233 241450400 ENSG00000287516 + 305
1 241436737 241439030 Homo_sapiens_1_241436435-241438237-HERVHF-pol + 1
241424233 241450400 ENSG00000287516 + 2293
1 241439482 241439885 Homo_sapiens_1_241436435-241438237-HERVHF-3LTR +
1 241424233 241450400 ENSG00000287516 + 403
1 241433890 241439885 Homo_sapiens_1_241436435-241438237-HERVHF + 1
241424415 241450400 ENSG00000287516 + 5995
1 241433890 241434298 Homo_sapiens_1_241436435-241438237-HERVHF-5LTR +
1 241424415 241450400 ENSG00000287516 + 408
1 241436142 241436447 Homo_sapiens_1_241436435-241438237-HERVHF-gag + 1
241424415 241450400 ENSG00000287516 + 305
1 241436737 241439030 Homo_sapiens_1_241436435-241438237-HERVHF-pol + 1
241424415 241450400 ENSG00000287516 + 2293
1 241439482 241439885 Homo_sapiens_1_241436435-241438237-HERVHF-3LTR +
1 241424415 241450400 ENSG00000287516 + 403
1 241433890 241439885 Homo_sapiens_1_241436435-241438237-HERVHF + 1
241424421 241450400 NONHSAG115278.1 + 5995
1 241433890 241434298 Homo_sapiens_1_241436435-241438237-HERVHF-5LTR +
1 241424421 241450400 NONHSAG115278.1 + 408
1 241436142 241436447 Homo_sapiens_1_241436435-241438237-HERVHF-gag + 1
241424421 241450400 NONHSAG115278.1 + 305
1 241436737 241439030 Homo_sapiens_1_241436435-241438237-HERVHF-pol + 1
241424421 241450400 NONHSAG115278.1 + 2293
1 241439482 241439885 Homo_sapiens_1_241436435-241438237-HERVHF-3LTR +
1 241424421 241450400 NONHSAG115278.1 + 403
1 241433890 241439885 Homo_sapiens_1_241436435-241438237-HERVHF + 1
241424422 241450400 ENSG00000287516 + 5995
1 241433890 241434298 Homo_sapiens_1_241436435-241438237-HERVHF-5LTR +
1 241424422 241450400 ENSG00000287516 + 408
1 241436142 241436447 Homo_sapiens_1_241436435-241438237-HERVHF-gag + 1
241424422 241450400 ENSG00000287516 + 305
1 241436737 241439030 Homo_sapiens_1_241436435-241438237-HERVHF-pol + 1
241424422 241450400 ENSG00000287516 + 2293
1 241439482 241439885 Homo_sapiens_1_241436435-241438237-HERVHF-3LTR +
1 241424422 241450400 ENSG00000287516 + 403
1 241433890 241439885 Homo_sapiens_1_241436435-241438237-HERVHF + 1

241424731 241450593 ENSG00000287516 + 5995
1 241433890 241434298 Homo_sapiens_1_241436435-241438237-HERVHF-5LTR +
1 241424731 241450593 ENSG00000287516 + 408
1 241436142 241436447 Homo_sapiens_1_241436435-241438237-HERVHF-gag + 1
241424731 241450593 ENSG00000287516 + 305
1 241436737 241439030 Homo_sapiens_1_241436435-241438237-HERVHF-pol + 1
241424731 241450593 ENSG00000287516 + 2293
1 241439482 241439885 Homo_sapiens_1_241436435-241438237-HERVHF-3LTR +
1 241424731 241450593 ENSG00000287516 + 403
1 241433890 241439885 Homo_sapiens_1_241436435-241438237-HERVHF + 1
241433492 241450400 NONHSAT259099.1 + 5995
1 241433890 241434298 Homo_sapiens_1_241436435-241438237-HERVHF-5LTR +
1 241433492 241450400 NONHSAT259099.1 + 408
1 241436142 241436447 Homo_sapiens_1_241436435-241438237-HERVHF-gag + 1
241433492 241450400 NONHSAT259099.1 + 305
1 241436737 241439030 Homo_sapiens_1_241436435-241438237-HERVHF-pol + 1
241433492 241450400 NONHSAT259099.1 + 2293
1 241439482 241439885 Homo_sapiens_1_241436435-241438237-HERVHF-3LTR +
1 241433492 241450400 NONHSAT259099.1 + 403
2 5000768 5001218 Homo_sapiens_2_5003505-5005037-HERVHF-5LTR + 2 4993143
5003638 NONHSAG077068.1 + 450
2 5000768 5001218 Homo_sapiens_2_5003505-5005037-HERVHF-5LTR + 2 4993143
5003638 NONHSAT181175.1+ 450
2 5003044 5003355 Homo_sapiens_2_5003505-5005037-HERVHF-gag + 2 4993143
5003638 NONHSAG077068.1 + 311
2 5003044 5003355 Homo_sapiens_2_5003505-5005037-HERVHF-gag + 2 4993143
5003638 NONHSAT181175.1+ 311
2 16791950 16797713 Homo_sapiens_2_16793801-16795145-HERVHF - 2 16791979
16797455 NONHSAG078497.1 - 5476
2 16791950 16797713 Homo_sapiens_2_16793801-16795145-HERVHF - 2 16791979
16797455 NONHSAT184285.1 - 5476
2 16792786 16794855 Homo_sapiens_2_16793801-16795145-HERVHF-pol - 2
16791979 16797455 NONHSAG078497.1 - 2069
2 16792786 16794855 Homo_sapiens_2_16793801-16795145-HERVHF-pol - 2
16791979 16797455 NONHSAT184285.1 - 2069
2 16794364 16795194 Homo_sapiens_2_16793801-16795145-HERVHF-pro - 2
16791979 16797455 NONHSAG078497.1 - 830
2 16794364 16795194 Homo_sapiens_2_16793801-16795145-HERVHF-pro - 2
16791979 16797455 NONHSAT184285.1 - 830
2 16795201 16795527 Homo_sapiens_2_16793801-16795145-HERVHF-gag - 2
16791979 16797455 NONHSAG078497.1 - 326
2 16795201 16795527 Homo_sapiens_2_16793801-16795145-HERVHF-gag - 2
16791979 16797455 NONHSAT184285.1 - 326
2 34789818 34796058 Homo_sapiens_2_34792231-34793755-HERVHF + 2 34653353

35016615 NONHSAG077257.1 + 6240
2 34789818 34790229 Homo_sapiens_2_34792231-34793755-HERVHF-5LTR + 2
34653353 35016615 NONHSAG077257.1 + 411
2 34789818 34796058 Homo_sapiens_2_34792231-34793755-HERVHF + 2 34653353
35016615 NONHSAT181538.1 + 6240
2 34789818 34790229 Homo_sapiens_2_34792231-34793755-HERVHF-5LTR + 2
34653353 35016615 NONHSAT181538.1 + 411
2 34791769 34792152 Homo_sapiens_2_34792231-34793755-HERVHF-gag + 2
34653353 35016615 NONHSAG077257.1 + 383
2 34791769 34792152 Homo_sapiens_2_34792231-34793755-HERVHF-gag + 2
34653353 35016615 NONHSAT181538.1 + 383
2 34792609 34793756 Homo_sapiens_2_34792231-34793755-HERVHF-pol + 2
34653353 35016615 NONHSAG077257.1 + 1147
2 34792609 34793756 Homo_sapiens_2_34792231-34793755-HERVHF-pol + 2
34653353 35016615 NONHSAT181538.1 + 1147
2 34794693 34795526 Homo_sapiens_2_34792231-34793755-HERVHF-env + 2
34653353 35016615 NONHSAG077257.1 + 833
2 34794693 34795526 Homo_sapiens_2_34792231-34793755-HERVHF-env + 2
34653353 35016615 NONHSAT181538.1 + 833
2 34795641 34796058 Homo_sapiens_2_34792231-34793755-HERVHF-3LTR + 2
34653353 35016615 NONHSAG077257.1 + 417
2 34795641 34796058 Homo_sapiens_2_34792231-34793755-HERVHF-3LTR + 2
34653353 35016615 NONHSAT181538.1 + 417
2 38080800 38086513 Homo_sapiens_2_38082627-38083973-HERVHF - 2 38070066
38109902 ENSG00000138061 - 5713
2 38080800 38081210 Homo_sapiens_2_38082627-38083973-HERVHF-5LTR - 2
38070066 38109902 ENSG00000138061 - 410
2 38081220 38081575 Homo_sapiens_2_38082627-38083973-HERVHF-env - 2
38070066 38109902 ENSG00000138061 - 355
2 38081793 38083597 Homo_sapiens_2_38082627-38083973-HERVHF-pol - 2
38070066 38109902 ENSG00000138061 - 1804
2 38083220 38083771 Homo_sapiens_2_38082627-38083973-HERVHF-pro - 2
38070066 38109902 ENSG00000138061 - 551
2 38086112 38086513 Homo_sapiens_2_38082627-38083973-HERVHF-3LTR - 2
38070066 38109902 ENSG00000138061 - 401
2 67333734 67334075 Homo_sapiens_2_67334137-67335887-HERVHF-gag + 2
67331882 67345739 NONHSAG028042.3 + 341
2 67333734 67334075 Homo_sapiens_2_67334137-67335887-HERVHF-gag + 2
67331882 67337638 NONHSAT240668.1 + 341
2 67333734 67334075 Homo_sapiens_2_67334137-67335887-HERVHF-gag + 2
67331882 67341136 NONHSAT240669.1 + 341
2 67334330 67335070 Homo_sapiens_2_67334137-67335887-HERVHF-pro + 2
67331882 67345739 NONHSAG028042.3 + 740
2 67334330 67335070 Homo_sapiens_2_67334137-67335887-HERVHF-pro + 2

67331882 67337638 NONHSAT240668.1 + 740
2 67334330 67335070 Homo_sapiens_2_67334137-67335887-HERVHF-pro + 2
67331882 67341136 NONHSAT240669.1 + 740
2 67334504 67336274 Homo_sapiens_2_67334137-67335887-HERVHF-pol + 2
67331882 67345739 NONHSAG028042.3 + 1770
2 67334504 67336274 Homo_sapiens_2_67334137-67335887-HERVHF-pol + 2
67331882 67337638 NONHSAT240668.1 + 1770
2 67334504 67336274 Homo_sapiens_2_67334137-67335887-HERVHF-pol + 2
67331882 67341136 NONHSAT240669.1 + 1770
2 67337203 67337603 Homo_sapiens_2_67334137-67335887-HERVHF-3LTR + 2
67331882 67345739 NONHSAG028042.3 + 400
2 67337203 67337603 Homo_sapiens_2_67334137-67335887-HERVHF-3LTR + 2
67331882 67337638 NONHSAT240668.1 + 400
2 67337203 67337603 Homo_sapiens_2_67334137-67335887-HERVHF-3LTR + 2
67331882 67341136 NONHSAT240669.1 + 400
2 67334330 67335070 Homo_sapiens_2_67334137-67335887-HERVHF-pro + 2
67334004 67340821 NONHSAT071280.2 + 740
2 67334504 67336274 Homo_sapiens_2_67334137-67335887-HERVHF-pol + 2
67334004 67340821 NONHSAT071280.2 + 1770
2 67337203 67337603 Homo_sapiens_2_67334137-67335887-HERVHF-3LTR + 2
67334004 67340821 NONHSAT071280.2 + 400
2 67334330 67335070 Homo_sapiens_2_67334137-67335887-HERVHF-pro + 2
67334007 67345739 NONHSAT181958.1 + 740
2 67334504 67336274 Homo_sapiens_2_67334137-67335887-HERVHF-pol + 2
67334007 67345739 NONHSAT181958.1 + 1770
2 67337203 67337603 Homo_sapiens_2_67334137-67335887-HERVHF-3LTR + 2
67334007 67345739 NONHSAT181958.1 + 400
2 69789900 69790233 Homo_sapiens_2_69792965-69796021-HUERSP-5LTR - 2
69789551 69798948 NONHSAG028084.2 - 333
2 69789900 69790233 Homo_sapiens_2_69792965-69796021-HUERSP-5LTR - 2
69789551 69798948 NONHSAT071358.2 - 333
2 69794775 69795859 Homo_sapiens_2_69792965-69796021-HUERSP-pol - 2
69789551 69798948 NONHSAG028084.2 - 1084
2 69794775 69795859 Homo_sapiens_2_69792965-69796021-HUERSP-pol - 2
69789551 69798948 NONHSAT071358.2 - 1084
2 69795440 69795853 Homo_sapiens_2_69792965-69796021-HUERSP-pro - 2
69789551 69798948 NONHSAG028084.2 - 413
2 69795440 69795853 Homo_sapiens_2_69792965-69796021-HUERSP-pro - 2
69789551 69798948 NONHSAT071358.2 - 413
2 77965137 77970868 Homo_sapiens_2_77967627-77968976-HERVHF + 2 77942237
77981314 NONHSAG077496.1 + 5731
2 77965137 77965570 Homo_sapiens_2_77967627-77968976-HERVHF-5LTR + 2
77942237 77981314 NONHSAG077496.1 + 433
2 77965137 77970868 Homo_sapiens_2_77967627-77968976-HERVHF + 2 77942237

77981314 NONHSAT182071.1 + 5731
2 77965137 77965570 Homo_sapiens_2_77967627-77968976-HERVHF-5LTR + 2
77942237 77981314 NONHSAT182071.1 + 433
2 77967584 77968414 Homo_sapiens_2_77967627-77968976-HERVHF-pro + 2
77942237 77981314 NONHSAG077496.1 + 830
2 77967584 77968414 Homo_sapiens_2_77967627-77968976-HERVHF-pro + 2
77942237 77981314 NONHSAT182071.1 + 830
2 77967872 77970012 Homo_sapiens_2_77967627-77968976-HERVHF-pol + 2
77942237 77981314 NONHSAG077496.1 + 2140
2 77967872 77970012 Homo_sapiens_2_77967627-77968976-HERVHF-pol + 2
77942237 77981314 NONHSAT182071.1 + 2140
2 77970418 77970868 Homo_sapiens_2_77967627-77968976-HERVHF-3LTR + 2
77942237 77981314 NONHSAG077496.1 + 450
2 77970418 77970868 Homo_sapiens_2_77967627-77968976-HERVHF-3LTR + 2
77942237 77981314 NONHSAT182071.1 + 450
2 192506078 192513184 Homo_sapiens_2_192509946-192511342-HERVHF+ 2
192396591 192546131 NONHSAG030125.2 + 7106
2 192506078 192506492 Homo_sapiens_2_192509946-192511342-HERVHF-5LTR +
2 192396591 192546131 NONHSAG030125.2 + 414
2 192506078 192513184 Homo_sapiens_2_192509946-192511342-HERVHF+ 2
192396591 192546131 NONHSAT183345.1 + 7106
2 192506078 192506492 Homo_sapiens_2_192509946-192511342-HERVHF-5LTR +
2 192396591 192546131 NONHSAT183345.1 + 414
2 192507423 192509865 Homo_sapiens_2_192509946-192511342-HERVHF-gag + 2
192396591 192546131 NONHSAG030125.2 + 2442
2 192507423 192509865 Homo_sapiens_2_192509946-192511342-HERVHF-gag + 2
192396591 192546131 NONHSAT183345.1 + 2442
2 192510149 192510781 Homo_sapiens_2_192509946-192511342-HERVHF-pro + 2
192396591 192546131 NONHSAG030125.2 + 632
2 192510149 192510781 Homo_sapiens_2_192509946-192511342-HERVHF-pro + 2
192396591 192546131 NONHSAT183345.1 + 632
2 192510377 192512317 Homo_sapiens_2_192509946-192511342-HERVHF-pol + 2
192396591 192546131 NONHSAG030125.2 + 1940
2 192510377 192512317 Homo_sapiens_2_192509946-192511342-HERVHF-pol + 2
192396591 192546131 NONHSAT183345.1 + 1940
2 192512768 192513184 Homo_sapiens_2_192509946-192511342-HERVHF-3LTR +
2 192396591 192546131 NONHSAG030125.2 + 416
2 192512768 192513184 Homo_sapiens_2_192509946-192511342-HERVHF-3LTR +
2 192396591 192546131 NONHSAT183345.1 + 416
2 192506078 192506492 Homo_sapiens_2_192509946-192511342-HERVHF-5LTR +
2 192500447 192506865 NONHSAG078059.1 + 414
2 192506078 192506492 Homo_sapiens_2_192509946-192511342-HERVHF-5LTR +
2 192500447 192506865 NONHSAT183346.1 + 414
2 215922303 215928129 Homo_sapiens_2_215924899-215926434-HERVHF + 2

215922571 215924919 NONHSAG078151.1 + 2348
2 215922303 215928129 Homo_sapiens_2_215924899-215926434-HERVHF + 2
215922571 215924919 NONHSAT183525.1 + 2348
2 215924520 215924849 Homo_sapiens_2_215924899-215926434-HERVHF-gag + 2
215922571 215924919 NONHSAG078151.1 + 329
2 215924520 215924849 Homo_sapiens_2_215924899-215926434-HERVHF-gag + 2
215922571 215924919 NONHSAT183525.1 + 329
2 224225331 224230988 Homo_sapiens_2_224227814-224229160-HERVHF + 2
224221224 224250090 NONHSAG078214.1 + 5657
2 224225331 224225730 Homo_sapiens_2_224227814-224229160-HERVHF-5LTR +
2 224221224 224250090 NONHSAG078214.1 + 399
2 224225331 224230988 Homo_sapiens_2_224227814-224229160-HERVHF + 2
224221224 224250090 NONHSAT183659.1 + 5657
2 224225331 224225730 Homo_sapiens_2_224227814-224229160-HERVHF-5LTR +
2 224221224 224250090 NONHSAT183659.1 + 399
2 224228022 224228600 Homo_sapiens_2_224227814-224229160-HERVHF-pro + 2
224221224 224250090 NONHSAG078214.1 + 578
2 224228022 224228600 Homo_sapiens_2_224227814-224229160-HERVHF-pro + 2
224221224 224250090 NONHSAT183659.1 + 578
2 224228193 224229742 Homo_sapiens_2_224227814-224229160-HERVHF-pol + 2
224221224 224250090 NONHSAG078214.1 + 1549
2 224228193 224229742 Homo_sapiens_2_224227814-224229160-HERVHF-pol + 2
224221224 224250090 NONHSAT183659.1 + 1549
2 224230587 224230988 Homo_sapiens_2_224227814-224229160-HERVHF-3LTR +
2 224221224 224250090 NONHSAG078214.1 + 401
2 224230587 224230988 Homo_sapiens_2_224227814-224229160-HERVHF-3LTR +
2 224221224 224250090 NONHSAT183659.1 + 401
2 237606784 237607197 Homo_sapiens_2_237609479-237610820-HERVHF-5LTR +
2 237603880 237612508 NONHSAG110040.1 + 413
2 237606784 237607197 Homo_sapiens_2_237609479-237610820-HERVHF-5LTR +
2 237603880 237607390 NONHSAT241581.1 + 413
2 237606784 237607197 Homo_sapiens_2_237609479-237610820-HERVHF-5LTR +
2 237603880 237612508 NONHSAT241582.1 + 413
2 237609020 237609400 Homo_sapiens_2_237609479-237610820-HERVHF-gag + 2
237603880 237612508 NONHSAG110040.1 + 380
2 237609020 237609400 Homo_sapiens_2_237609479-237610820-HERVHF-gag + 2
237603880 237612508 NONHSAT241582.1 + 380
2 237609517 237610233 Homo_sapiens_2_237609479-237610820-HERVHF-pro + 2
237603880 237612508 NONHSAG110040.1 + 716
2 237609517 237610233 Homo_sapiens_2_237609479-237610820-HERVHF-pro + 2
237603880 237612508 NONHSAT241582.1 + 716
2 237609622 237611630 Homo_sapiens_2_237609479-237610820-HERVHF-pol + 2
237603880 237612508 NONHSAG110040.1 + 2008
2 237609622 237611630 Homo_sapiens_2_237609479-237610820-HERVHF-pol + 2

237603880 237612508 NONHSAT241582.1 + 2008
3 21189031 21194139 Homo_sapiens_3_21190643-21192440-HERVHF - 3 21006729 21226305 NONHSAG085388.2 - 5108
3 21189031 21189353 Homo_sapiens_3_21190643-21192440-HERVHF-5LTR - 3 21006729 21226305 NONHSAG085388.2 - 322
3 21189031 21194139 Homo_sapiens_3_21190643-21192440-HERVHF - 3 21006729 21226305 NONHSAT246351.1 - 5108
3 21189031 21189353 Homo_sapiens_3_21190643-21192440-HERVHF-5LTR - 3 21006729 21226305 NONHSAT246351.1 - 322
3 21189872 21191978 Homo_sapiens_3_21190643-21192440-HERVHF-pol - 3 21006729 21226305 NONHSAG085388.2 - 2106
3 21189872 21191978 Homo_sapiens_3_21190643-21192440-HERVHF-pol - 3 21006729 21226305 NONHSAT246351.1 - 2106
3 21192518 21192895 Homo_sapiens_3_21190643-21192440-HERVHF-gag - 3 21006729 21226305 NONHSAG085388.2 - 377
3 21192518 21192895 Homo_sapiens_3_21190643-21192440-HERVHF-gag - 3 21006729 21226305 NONHSAT246351.1 - 377
3 21193803 21194139 Homo_sapiens_3_21190643-21192440-HERVHF-3LTR - 3 21006729 21226305 NONHSAG085388.2 - 336
3 21193803 21194139 Homo_sapiens_3_21190643-21192440-HERVHF-3LTR - 3 21006729 21226305 NONHSAT246351.1 - 336
3 21189031 21194139 Homo_sapiens_3_21190643-21192440-HERVHF - 3 21006730 21226305 ENSG00000282987 - 5108
3 21189031 21189353 Homo_sapiens_3_21190643-21192440-HERVHF-5LTR - 3 21006730 21226305 ENSG00000282987 - 322
3 21189872 21191978 Homo_sapiens_3_21190643-21192440-HERVHF-pol - 3 21006730 21226305 ENSG00000282987 - 2106
3 21192518 21192895 Homo_sapiens_3_21190643-21192440-HERVHF-gag - 3 21006730 21226305 ENSG00000282987 - 377
3 21193803 21194139 Homo_sapiens_3_21190643-21192440-HERVHF-3LTR - 3 21006730 21226305 ENSG00000282987 - 336
3 21189031 21194139 Homo_sapiens_3_21190643-21192440-HERVHF - 3 21171015 21224899 NONHSAT195484.1 - 5108
3 21189031 21189353 Homo_sapiens_3_21190643-21192440-HERVHF-5LTR - 3 21171015 21224899 NONHSAT195484.1 - 322
3 21189872 21191978 Homo_sapiens_3_21190643-21192440-HERVHF-pol - 3 21171015 21224899 NONHSAT195484.1 - 2106
3 21192518 21192895 Homo_sapiens_3_21190643-21192440-HERVHF-gag - 3 21171015 21224899 NONHSAT195484.1 - 377
3 21193803 21194139 Homo_sapiens_3_21190643-21192440-HERVHF-3LTR - 3 21171015 21224899 NONHSAT195484.1 - 336
3 54634482 54634938 Homo_sapiens_3_54636349-54637755-HERVHF-5LTR - 3 54632121 54639857 NONHSAG035219.2 - 456
3 54634482 54634938 Homo_sapiens_3_54636349-54637755-HERVHF-5LTR - 3

54632121 54639857 NONHSAT090016.2 - 456
3 54635330 54637456 Homo_sapiens_3_54636349-54637755-HERVHF-pol - 3
54632121 54639857 NONHSAG035219.2 - 2126
3 54635330 54637456 Homo_sapiens_3_54636349-54637755-HERVHF-pol - 3
54632121 54639857 NONHSAT090016.2 - 2126
3 54636914 54637756 Homo_sapiens_3_54636349-54637755-HERVHF-pro - 3
54632121 54639857 NONHSAG035219.2 - 842
3 54636914 54637756 Homo_sapiens_3_54636349-54637755-HERVHF-pro - 3
54632121 54639857 NONHSAT090016.2 - 842
3 54637769 54638068 Homo_sapiens_3_54636349-54637755-HERVHF-gag - 3
54632121 54639857 NONHSAG035219.2 - 299
3 54637769 54638068 Homo_sapiens_3_54636349-54637755-HERVHF-gag - 3
54632121 54639857 NONHSAT090016.2 - 299
3 54634482 54634938 Homo_sapiens_3_54636349-54637755-HERVHF-5LTR - 3
54632122 54639857 ENSG00000265992 - 456
3 54635330 54637456 Homo_sapiens_3_54636349-54637755-HERVHF-pol - 3
54632122 54639857 ENSG00000265992 - 2126
3 54636914 54637756 Homo_sapiens_3_54636349-54637755-HERVHF-pro - 3
54632122 54639857 ENSG00000265992 - 842
3 54637769 54638068 Homo_sapiens_3_54636349-54637755-HERVHF-gag - 3
54632122 54639857 ENSG00000265992 - 299
3 112418410 112423366 Homo_sapiens_3_112419312-112420768-HERVHF- 3
112411243 112460780 NONHSAG035734.2 - 4956
3 112418410 112418865 Homo_sapiens_3_112419312-112420768-HERVHF-5LTR -
3 112411243 112460780 NONHSAG035734.2 - 455
3 112418410 112423366 Homo_sapiens_3_112419312-112420768-HERVHF- 3
112411243 112460780 NONHSAT091178.2- 4956
3 112418410 112418865 Homo_sapiens_3_112419312-112420768-HERVHF-5LTR -
3 112411243 112460780 NONHSAT091178.2- 455
3 112419048 112420617 Homo_sapiens_3_112419312-112420768-HERVHF-pol - 3
112411243 112460780 NONHSAG035734.2 - 1569
3 112419048 112420617 Homo_sapiens_3_112419312-112420768-HERVHF-pol - 3
112411243 112460780 NONHSAT091178.2- 1569
3 112420824 112421368 Homo_sapiens_3_112419312-112420768-HERVHF-gag - 3
112411243 112460780 NONHSAG035734.2 - 544
3 112420824 112421368 Homo_sapiens_3_112419312-112420768-HERVHF-gag - 3
112411243 112460780 NONHSAT091178.2- 544
3 112422911 112423366 Homo_sapiens_3_112419312-112420768-HERVHF-3LTR -
3 112411243 112460780 NONHSAG035734.2 - 455
3 112422911 112423366 Homo_sapiens_3_112419312-112420768-HERVHF-3LTR -
3 112411243 112460780 NONHSAT091178.2- 455
3 115798715 115799166 Homo_sapiens_3_115795176-115796709-HERVHF-3LTR -
3 115797524 115799318 NONHSAG085690.1 - 451
3 115798715 115799166 Homo_sapiens_3_115795176-115796709-HERVHF-3LTR -

3 115797524 115799318 NONHSAT196129.1 - 451
3 155274423 155274841 Homo_sapiens_3_155276457-155278448-HERVHF-5LTR -
3 155250505 155280483 NONHSAG036456.2 - 418
3 155274423 155274841 Homo_sapiens_3_155276457-155278448-HERVHF-5LTR -
3 155250505 155280483 NONHSAT092826.2 - 418
3 155275702 155278149 Homo_sapiens_3_155276457-155278448-HERVHF-pol - 3
155250505 155280483 NONHSAG036456.2 - 2447
3 155275702 155278149 Homo_sapiens_3_155276457-155278448-HERVHF-pol - 3
155250505 155280483 NONHSAT092826.2 - 2447
3 155277628 155278386 Homo_sapiens_3_155276457-155278448-HERVHF-pro - 3
155250505 155280483 NONHSAG036456.2 - 758
3 155277628 155278386 Homo_sapiens_3_155276457-155278448-HERVHF-pro - 3
155250505 155280483 NONHSAT092826.2 - 758
3 155278439 155278762 Homo_sapiens_3_155276457-155278448-HERVHF-gag - 3
155250505 155280483 NONHSAG036456.2 - 323
3 155278439 155278762 Homo_sapiens_3_155276457-155278448-HERVHF-gag - 3
155250505 155280483 NONHSAT092826.2 - 323
3 186660747 186661298 Homo_sapiens_3_186660542-186661888-HERVHF-pro + 3
186660216 186666181 ENSG00000113905 + 551
3 186660786 186662849 Homo_sapiens_3_186660542-186661888-HERVHF-pol + 3
186660216 186666181 ENSG00000113905 + 2063
3 186663286 186663692 Homo_sapiens_3_186660542-186661888-HERVHF-3LTR +
3 186660216 186666181 ENSG00000113905 + 406
4 3927445 3927854 Homo_sapiens_4_3929901-3931242-HERVHF-5LTR + 4 3905745
3931105 NONHSAG087348.2 + 409
4 3929526 3929825 Homo_sapiens_4_3929901-3931242-HERVHF-gag + 4 3905745
3931105 NONHSAG087348.2 + 299
4 3929852 3930682 Homo_sapiens_4_3929901-3931242-HERVHF-pro + 4 3905745
3931105 NONHSAG087348.2 + 830
4 3927445 3933082 Homo_sapiens_4_3929901-3931242-HERVHF + 4 3927632
3931105 NONHSAT198208.1 + 3473
4 3929526 3929825 Homo_sapiens_4_3929901-3931242-HERVHF-gag + 4 3927632
3931105 NONHSAT198208.1 + 299
4 3929852 3930682 Homo_sapiens_4_3929901-3931242-HERVHF-pro + 4 3927632
3931105 NONHSAT198208.1 + 830
4 16997808 17003928 Homo_sapiens_4_17000127-17001778-HERVHF + 4 16997950
16999118 NONHSAT198342.1 + 1168
4 17000545 17002680 Homo_sapiens_4_17000127-17001778-HERVHF-pol + 4
16997950 17009063 NONHSAG037572.2 + 2135
4 17000545 17002680 Homo_sapiens_4_17000127-17001778-HERVHF-pol + 4
16997950 17009063 NONHSAT095697.2 + 2135
4 17003527 17003928 Homo_sapiens_4_17000127-17001778-HERVHF-3LTR + 4
16997950 17009063 NONHSAG037572.2 + 401
4 17003527 17003928 Homo_sapiens_4_17000127-17001778-HERVHF-3LTR + 4

16997950 17009063 NONHSAT095697.2 + 401
4 24500975 24501427 Homo_sapiens_4_24503534-24505060-HERVHF-5LTR + 4
24499921 24501593 NONHSAG037630.2 + 452
4 24500975 24501427 Homo_sapiens_4_24503534-24505060-HERVHF-5LTR + 4
24499921 24501593 NONHSAT095802.2 + 452
4 27974874 27981319 Homo_sapiens_4_27976550-27977552-HERVHF + 4 27902566
28003138 NONHSAG037691.2 + 6445
4 27974874 27975246 Homo_sapiens_4_27976550-27977552-HERVHF-5LTR + 4
27902566 28003138 NONHSAG037691.2 + 372
4 27974874 27981319 Homo_sapiens_4_27976550-27977552-HERVHF + 4 27902566
27993633 NONHSAT095904.2 + 6445
4 27974874 27975246 Homo_sapiens_4_27976550-27977552-HERVHF-5LTR + 4
27902566 27993633 NONHSAT095904.2 + 372
4 27975998 27976339 Homo_sapiens_4_27976550-27977552-HERVHF-gag + 4
27902566 28003138 NONHSAG037691.2 + 341
4 27975998 27976339 Homo_sapiens_4_27976550-27977552-HERVHF-gag + 4
27902566 27993633 NONHSAT095904.2 + 341
4 27976612 27977274 Homo_sapiens_4_27976550-27977552-HERVHF-pro + 4
27902566 28003138 NONHSAG037691.2 + 662
4 27976612 27977274 Homo_sapiens_4_27976550-27977552-HERVHF-pro + 4
27902566 27993633 NONHSAT095904.2 + 662
4 27976735 27980331 Homo_sapiens_4_27976550-27977552-HERVHF-pol + 4
27902566 28003138 NONHSAG037691.2 + 3596
4 27976735 27980331 Homo_sapiens_4_27976550-27977552-HERVHF-pol + 4
27902566 27993633 NONHSAT095904.2 + 3596
4 27980964 27981319 Homo_sapiens_4_27976550-27977552-HERVHF-3LTR + 4
27902566 28003138 NONHSAG037691.2 + 355
4 27980964 27981319 Homo_sapiens_4_27976550-27977552-HERVHF-3LTR + 4
27902566 27993633 NONHSAT095904.2 + 355
4 27974874 27981319 Homo_sapiens_4_27976550-27977552-HERVHF + 4 27902568
28003138 NONHSAT198438.1 + 6445
4 27974874 27975246 Homo_sapiens_4_27976550-27977552-HERVHF-5LTR + 4
27902568 28003138 NONHSAT198438.1 + 372
4 27975998 27976339 Homo_sapiens_4_27976550-27977552-HERVHF-gag + 4
27902568 28003138 NONHSAT198438.1 + 341
4 27976612 27977274 Homo_sapiens_4_27976550-27977552-HERVHF-pro + 4
27902568 28003138 NONHSAT198438.1 + 662
4 27976735 27980331 Homo_sapiens_4_27976550-27977552-HERVHF-pol + 4
27902568 28003138 NONHSAT198438.1 + 3596
4 27980964 27981319 Homo_sapiens_4_27976550-27977552-HERVHF-3LTR + 4
27902568 28003138 NONHSAT198438.1 + 355
4 92271492 92271860 Homo_sapiens_4_92273363-92274770-HERVHF-5LTR - 4
92255183 92277377 NONHSAG038435.2 - 368
4 92271492 92271860 Homo_sapiens_4_92273363-92274770-HERVHF-5LTR - 4

92255183 92277377 NONHSAT097428.2 - 368
4 92272378 92274561 Homo_sapiens_4_92273363-92274770-HERVHF-pol - 4
92255183 92277377 NONHSAG038435.2 - 2183
4 92272378 92274561 Homo_sapiens_4_92273363-92274770-HERVHF-pol - 4
92255183 92277377 NONHSAT097428.2 - 2183
4 92273926 92274771 Homo_sapiens_4_92273363-92274770-HERVHF-pro - 4
92255183 92277377 NONHSAG038435.2 - 845
4 92273926 92274771 Homo_sapiens_4_92273363-92274770-HERVHF-pro - 4
92255183 92277377 NONHSAT097428.2 - 845
4 92274781 92275299 Homo_sapiens_4_92273363-92274770-HERVHF-gag - 4
92255183 92277377 NONHSAG038435.2 - 518
4 92274781 92275299 Homo_sapiens_4_92273363-92274770-HERVHF-gag - 4
92255183 92277377 NONHSAT097428.2 - 518
4 92271492 92271860 Homo_sapiens_4_92273363-92274770-HERVHF-5LTR - 4
92262497 92277302 NONHSAT097430.2 - 368
4 92272378 92274561 Homo_sapiens_4_92273363-92274770-HERVHF-pol - 4
92262497 92277302 NONHSAT097430.2 - 2183
4 92273926 92274771 Homo_sapiens_4_92273363-92274770-HERVHF-pro - 4
92262497 92277302 NONHSAT097430.2 - 845
4 92274781 92275299 Homo_sapiens_4_92273363-92274770-HERVHF-gag - 4
92262497 92277302 NONHSAT097430.2 - 518
4 92271492 92271860 Homo_sapiens_4_92273363-92274770-HERVHF-5LTR - 4
92262498 92277076 NONHSAT200205.1 - 368
4 92272378 92274561 Homo_sapiens_4_92273363-92274770-HERVHF-pol - 4
92262498 92277076 NONHSAT200205.1 - 2183
4 92273926 92274771 Homo_sapiens_4_92273363-92274770-HERVHF-pro - 4
92262498 92277076 NONHSAT200205.1 - 845
4 92274781 92275299 Homo_sapiens_4_92273363-92274770-HERVHF-gag - 4
92262498 92277076 NONHSAT200205.1 - 518
4 92271492 92271860 Homo_sapiens_4_92273363-92274770-HERVHF-5LTR - 4
92268766 92277075 NONHSAT097431.2 - 368
4 92272378 92274561 Homo_sapiens_4_92273363-92274770-HERVHF-pol - 4
92268766 92277075 NONHSAT097431.2 - 2183
4 92273926 92274771 Homo_sapiens_4_92273363-92274770-HERVHF-pro - 4
92268766 92277075 NONHSAT097431.2 - 845
4 92274781 92275299 Homo_sapiens_4_92273363-92274770-HERVHF-gag - 4
92268766 92277075 NONHSAT097431.2 - 518
4 92271492 92271860 Homo_sapiens_4_92273363-92274770-HERVHF-5LTR - 4
92268767 92277075 ENSG00000249152 - 368
4 92272378 92274561 Homo_sapiens_4_92273363-92274770-HERVHF-pol - 4
92268767 92277075 ENSG00000249152 - 2183
4 92273926 92274771 Homo_sapiens_4_92273363-92274770-HERVHF-pro - 4
92268767 92277075 ENSG00000249152 - 845
4 92274781 92275299 Homo_sapiens_4_92273363-92274770-HERVHF-gag - 4

92268767 92277075 ENSG00000249152 - 518
4 103553770 103554203 Homo_sapiens_4_103555460-103556971-HERVHF-5LTR - 4 103550586 103559147 ENSG00000250920 - 433
4 103553770 103554203 Homo_sapiens_4_103555460-103556971-HERVHF-5LTR - 4 103550586 103559112 ENSG00000250920 - 433
4 103554640 103556729 Homo_sapiens_4_103555460-103556971-HERVHF-pol - 4 103550586 103559147 ENSG00000250920 - 2089
4 103554640 103556729 Homo_sapiens_4_103555460-103556971-HERVHF-pol - 4 103550586 103559112 ENSG00000250920 - 2089
4 103556187 103557020 Homo_sapiens_4_103555460-103556971-HERVHF-pro - 4 103550586 103559147 ENSG00000250920 - 833
4 103556187 103557020 Homo_sapiens_4_103555460-103556971-HERVHF-pro - 4 103550586 103559112 ENSG00000250920 - 833
4 103557024 103557353 Homo_sapiens_4_103555460-103556971-HERVHF-gag - 4 103550586 103559147 ENSG00000250920 - 329
4 103557024 103557353 Homo_sapiens_4_103555460-103556971-HERVHF-gag - 4 103550586 103559112 ENSG00000250920 - 329
4 103553770 103554203 Homo_sapiens_4_103555460-103556971-HERVHF-5LTR - 4 103550589 103559277 ENSG00000250920 - 433
4 103553770 103554203 Homo_sapiens_4_103555460-103556971-HERVHF-5LTR - 4 103550589 103559271 ENSG00000250920 - 433
4 103553770 103554203 Homo_sapiens_4_103555460-103556971-HERVHF-5LTR - 4 103550589 103559271 ENSG00000250920 - 433
4 103554640 103556729 Homo_sapiens_4_103555460-103556971-HERVHF-pol - 4 103550589 103559277 ENSG00000250920 - 2089
4 103554640 103556729 Homo_sapiens_4_103555460-103556971-HERVHF-pol - 4 103550589 103559271 ENSG00000250920 - 2089
4 103554640 103556729 Homo_sapiens_4_103555460-103556971-HERVHF-pol - 4 103550589 103559271 ENSG00000250920 - 2089
4 103556187 103557020 Homo_sapiens_4_103555460-103556971-HERVHF-pro - 4 103550589 103559277 ENSG00000250920 - 833
4 103556187 103557020 Homo_sapiens_4_103555460-103556971-HERVHF-pro - 4 103550589 103559271 ENSG00000250920 - 833
4 103556187 103557020 Homo_sapiens_4_103555460-103556971-HERVHF-pro - 4 103550589 103559271 ENSG00000250920 - 833
4 103557024 103557353 Homo_sapiens_4_103555460-103556971-HERVHF-gag - 4 103550589 103559277 ENSG00000250920 - 329
4 103557024 103557353 Homo_sapiens_4_103555460-103556971-HERVHF-gag - 4 103550589 103559271 ENSG00000250920 - 329
4 103557024 103557353 Homo_sapiens_4_103555460-103556971-HERVHF-gag - 4 103550589 103559271 ENSG00000250920 - 329
4 103553770 103554203 Homo_sapiens_4_103555460-103556971-HERVHF-5LTR - 4 103550592 103559147 ENSG00000250920 - 433
4 103554640 103556729 Homo_sapiens_4_103555460-103556971-HERVHF-pol - 4

103550592 103559147 ENSG00000250920 - 2089
4 103556187 103557020 Homo_sapiens_4_103555460-103556971-HERVHF-pro - 4
103550592 103559147 ENSG00000250920 - 833
4 103557024 103557353 Homo_sapiens_4_103555460-103556971-HERVHF-gag - 4
103550592 103559147 ENSG00000250920 - 329
4 103553770 103554203 Homo_sapiens_4_103555460-103556971-HERVHF-5LTR -
4 103550926 103559127 NONHSAG038544.2 - 433
4 103553770 103554203 Homo_sapiens_4_103555460-103556971-HERVHF-5LTR -
4 103550926 103559127 NONHSAT097639.2 - 433
4 103554640 103556729 Homo_sapiens_4_103555460-103556971-HERVHF-pol - 4
103550926 103559127 NONHSAG038544.2 - 2089
4 103554640 103556729 Homo_sapiens_4_103555460-103556971-HERVHF-pol - 4
103550926 103559127 NONHSAT097639.2 - 2089
4 103556187 103557020 Homo_sapiens_4_103555460-103556971-HERVHF-pro - 4
103550926 103559127 NONHSAG038544.2 - 833
4 103556187 103557020 Homo_sapiens_4_103555460-103556971-HERVHF-pro - 4
103550926 103559127 NONHSAT097639.2 - 833
4 103557024 103557353 Homo_sapiens_4_103555460-103556971-HERVHF-gag - 4
103550926 103559127 NONHSAG038544.2 - 329
4 103557024 103557353 Homo_sapiens_4_103555460-103556971-HERVHF-gag - 4
103550926 103559127 NONHSAT097639.2 - 329
4 103553770 103554203 Homo_sapiens_4_103555460-103556971-HERVHF-5LTR -
4 103550927 103559127 ENSG00000250920 - 433
4 103554640 103556729 Homo_sapiens_4_103555460-103556971-HERVHF-pol - 4
103550927 103559127 ENSG00000250920 - 2089
4 103556187 103557020 Homo_sapiens_4_103555460-103556971-HERVHF-pro - 4
103550927 103559127 ENSG00000250920 - 833
4 103557024 103557353 Homo_sapiens_4_103555460-103556971-HERVHF-gag - 4
103550927 103559127 ENSG00000250920 - 329
4 128640901 128641313 Homo_sapiens_4_128642726-128644003-HERVHF-5LTR -
4 128609245 128646352 NONHSAG088517.2 - 412
4 128641891 128643929 Homo_sapiens_4_128642726-128644003-HERVHF-pol - 4
128609245 128646352 NONHSAG088517.2 - 2038
4 128643291 128643971 Homo_sapiens_4_128642726-128644003-HERVHF-pro - 4
128609245 128646352 NONHSAG088517.2 - 680
4 128644112 128644450 Homo_sapiens_4_128642726-128644003-HERVHF-gag - 4
128609245 128646352 NONHSAG088517.2 - 338
4 128640901 128641313 Homo_sapiens_4_128642726-128644003-HERVHF-5LTR -
4 128627628 128646352 NONHSAT200384.1 - 412
4 128640901 128641313 Homo_sapiens_4_128642726-128644003-HERVHF-5LTR -
4 128627628 128646352 NONHSAT200385.1 - 412
4 128641891 128643929 Homo_sapiens_4_128642726-128644003-HERVHF-pol - 4
128627628 128646352 NONHSAT200384.1 - 2038
4 128641891 128643929 Homo_sapiens_4_128642726-128644003-HERVHF-pol - 4

128627628	128646352	NONHSAT200385.1	-	2038
4	128643291	128643971	Homo_sapiens_4_128642726-128644003-HERVHF-pro	-	4
128627628	128646352	NONHSAT200384.1	-	680
4	128643291	128643971	Homo_sapiens_4_128642726-128644003-HERVHF-pro	-	4
128627628	128646352	NONHSAT200385.1	-	680
4	128644112	128644450	Homo_sapiens_4_128642726-128644003-HERVHF-gag	-	4
128627628	128646352	NONHSAT200384.1	-	338
4	128644112	128644450	Homo_sapiens_4_128642726-128644003-HERVHF-gag	-	4
128627628	128646352	NONHSAT200385.1	-	338
4	145698823	145703505	Homo_sapiens_4_145701612-145702617-HERVHF	+	4
145680146	145732797	ENSG00000237136	+	4682
4	145698823	145699274	Homo_sapiens_4_145701612-145702617-HERVHF-5LTR	+
4	145680146	145732797	ENSG00000237136	+	451
4	145701596	145702378	Homo_sapiens_4_145701612-145702617-HERVHF-pro	+	4
145680146	145732797	ENSG00000237136	+	782
4	145701734	145702528	Homo_sapiens_4_145701612-145702617-HERVHF-pol	+	4
145680146	145732797	ENSG00000237136	+	794
4	145703056	145703505	Homo_sapiens_4_145701612-145702617-HERVHF-3LTR	+
4	145680146	145732797	ENSG00000237136	+	449
4	152741345	152747172	Homo_sapiens_4_152743196-152744540-HERVHF	-	4
152741397	152746621	NONHSAG039129.2	-	5224
4	152741345	152747172	Homo_sapiens_4_152743196-152744540-HERVHF	-	4
152741397	152746621	NONHSAT098813.2	-	5224
4	152742614	152744164	Homo_sapiens_4_152743196-152744540-HERVHF-pol	-	4
152741397	152746621	NONHSAG039129.2	-	1550
4	152742614	152744164	Homo_sapiens_4_152743196-152744540-HERVHF-pol	-	4
152741397	152746621	NONHSAT098813.2	-	1550
4	152743760	152744263	Homo_sapiens_4_152743196-152744540-HERVHF-pro	-	4
152741397	152746621	NONHSAG039129.2	-	503
4	152743760	152744263	Homo_sapiens_4_152743196-152744540-HERVHF-pro	-	4
152741397	152746621	NONHSAT098813.2	-	503
4	175461163	175467003	Homo_sapiens_4_175463047-175464647-HERVHF	-	4
175457726	175473197	ENSG00000249945	-	5840
4	175461163	175461498	Homo_sapiens_4_175463047-175464647-HERVHF-5LTR	-
4	175457726	175473197	ENSG00000249945	-	335
4	175462086	175464209	Homo_sapiens_4_175463047-175464647-HERVHF-pol	-	4
175457726	175473197	ENSG00000249945	-	2123
4	175464527	175464889	Homo_sapiens_4_175463047-175464647-HERVHF-pro	-	4
175457726	175473197	ENSG00000249945	-	362
4	175466677	175467003	Homo_sapiens_4_175463047-175464647-HERVHF-3LTR	-
4	175457726	175473197	ENSG00000249945	-	326
4	175461163	175461498	Homo_sapiens_4_175463047-175464647-HERVHF-5LTR	-
4	175458288	175466697	NONHSAG039390.2	-	335
4	175461163	175461498	Homo_sapiens_4_175463047-175464647-HERVHF-5LTR	-

4 175458288 175466697 NONHSAT099342.2 - 335
4 175462086 175464209 Homo_sapiens_4_175463047-175464647-HERVHF-pol - 4
175458288 175466697 NONHSAG039390.2 - 2123
4 175462086 175464209 Homo_sapiens_4_175463047-175464647-HERVHF-pol - 4
175458288 175466697 NONHSAT099342.2 - 2123
4 175464527 175464889 Homo_sapiens_4_175463047-175464647-HERVHF-pro - 4
175458288 175466697 NONHSAG039390.2 - 362
4 175464527 175464889 Homo_sapiens_4_175463047-175464647-HERVHF-pro - 4
175458288 175466697 NONHSAT099342.2 - 362
4 175461163 175461498 Homo_sapiens_4_175463047-175464647-HERVHF-5LTR -
4 175458289 175466697 ENSG00000249945 - 335
4 175462086 175464209 Homo_sapiens_4_175463047-175464647-HERVHF-pol - 4
175458289 175466697 ENSG00000249945 - 2123
4 175464527 175464889 Homo_sapiens_4_175463047-175464647-HERVHF-pro - 4
175458289 175466697 ENSG00000249945 - 362
5 92826033 92826407 Homo_sapiens_5_92826486-92827829-HERVHF-gag + 5
92823935 92844992 ENSG00000248588 + 374
5 92826862 92828213 Homo_sapiens_5_92826486-92827829-HERVHF-pol + 5
92823935 92844992 ENSG00000248588 + 1351
5 92829273 92829706 Homo_sapiens_5_92826486-92827829-HERVHF-3LTR + 5
92823935 92844992 ENSG00000248588 + 433
5 92826862 92828213 Homo_sapiens_5_92826486-92827829-HERVHF-pol + 5
92826254 92844004 NONHSAG041017.2 + 1351
5 92826862 92828213 Homo_sapiens_5_92826486-92827829-HERVHF-pol + 5
92826254 92844004 NONHSAT102781.2 + 1351
5 92829273 92829706 Homo_sapiens_5_92826486-92827829-HERVHF-3LTR + 5
92826254 92844004 NONHSAG041017.2 + 433
5 92829273 92829706 Homo_sapiens_5_92826486-92827829-HERVHF-3LTR + 5
92826254 92844004 NONHSAT102781.2 + 433
5 92826862 92828213 Homo_sapiens_5_92826486-92827829-HERVHF-pol + 5
92826255 92844004 ENSG00000248588 + 1351
5 92829273 92829706 Homo_sapiens_5_92826486-92827829-HERVHF-3LTR + 5
92826255 92844004 ENSG00000248588 + 433
5 92823742 92829706 Homo_sapiens_5_92826486-92827829-HERVHF + 5 92828197
92828613 NONHSAG041018.2 + 416
5 92823742 92829706 Homo_sapiens_5_92826486-92827829-HERVHF + 5 92828197
92828613 NONHSAT102782.2 + 416
5 136303790 136304620 Homo_sapiens_5_136303833-136305180-HERVHF-pro + 5
136303756 136316101 NONHSAG041632.2 + 830
5 136303790 136304620 Homo_sapiens_5_136303833-136305180-HERVHF-pro + 5
136303756 136316100 NONHSAT103961.2 + 830
5 136304078 136305769 Homo_sapiens_5_136303833-136305180-HERVHF-pol + 5
136303756 136316101 NONHSAG041632.2 + 1691
5 136304078 136305769 Homo_sapiens_5_136303833-136305180-HERVHF-pol + 5

136303756 136316100 NONHSAT103961.2 + 1691
5 136306563 136307028 Homo_sapiens_5_136303833-136305180-HERVHF-3LTR + 5 136303756 136316101 NONHSAG041632.2 + 465
5 136306563 136307028 Homo_sapiens_5_136303833-136305180-HERVHF-3LTR + 5 136303756 136316100 NONHSAT103961.2 + 465
5 136303790 136304620 Homo_sapiens_5_136303833-136305180-HERVHF-pro + 5 136303757 136316100 ENSG00000250947 + 830
5 136303790 136304620 Homo_sapiens_5_136303833-136305180-HERVHF-pro + 5 136303757 136316101 NONHSAT203263.1 + 830
5 136304078 136305769 Homo_sapiens_5_136303833-136305180-HERVHF-pol + 5 136303757 136316100 ENSG00000250947 + 1691
5 136304078 136305769 Homo_sapiens_5_136303833-136305180-HERVHF-pol + 5 136303757 136316101 NONHSAT203263.1 + 1691
5 136306563 136307028 Homo_sapiens_5_136303833-136305180-HERVHF-3LTR + 5 136303757 136316100 ENSG00000250947 + 465
5 136306563 136307028 Homo_sapiens_5_136303833-136305180-HERVHF-3LTR + 5 136303757 136316101 NONHSAT203263.1 + 465
5 161245405 161254586 Homo_sapiens_5_161251016-161252646-HERVHF + 5 161246663 161247643 NONHSAG090654.1 + 980
5 161245405 161254586 Homo_sapiens_5_161251016-161252646-HERVHF + 5 161246663 161247643 NONHSAT203511.1+ 980
6 16259010 16264893 Homo_sapiens_6_16260854-16262201-HERVHF - 6 16259100 16264553 NONHSAG113149.1 - 5453
6 16259010 16264893 Homo_sapiens_6_16260854-16262201-HERVHF - 6 16259100 16262926 NONHSAT252012.1 - 3826
6 16260019 16261731 Homo_sapiens_6_16260854-16262201-HERVHF-pol - 6 16259100 16264553 NONHSAG113149.1 - 1712
6 16260019 16261731 Homo_sapiens_6_16260854-16262201-HERVHF-pol - 6 16259100 16262926 NONHSAT252012.1 - 1712
6 16261417 16262241 Homo_sapiens_6_16260854-16262201-HERVHF-pro - 6 16259100 16264553 NONHSAG113149.1 - 824
6 16261417 16262241 Homo_sapiens_6_16260854-16262201-HERVHF-pro - 6 16259100 16262926 NONHSAT252012.1 - 824
6 16259010 16264893 Homo_sapiens_6_16260854-16262201-HERVHF - 6 16259101 16262926 ENSG00000282024 - 3825
6 16260019 16261731 Homo_sapiens_6_16260854-16262201-HERVHF-pol - 6 16259101 16262926 ENSG00000282024 - 1712
6 16261417 16262241 Homo_sapiens_6_16260854-16262201-HERVHF-pro - 6 16259101 16262926 ENSG00000282024 - 824
6 16259010 16264893 Homo_sapiens_6_16260854-16262201-HERVHF - 6 16262681 16264553 NONHSAT252013.1 - 1872
6 16259010 16264893 Homo_sapiens_6_16260854-16262201-HERVHF - 6 16262682 16264553 ENSG00000282024 - 1871
6 18754142 18754535 Homo_sapiens_6_18755932-18757277-HERVHF-5LTR - 6

18743775 18757467 NONHSAG043117.2 - 393
6 18754142 18754535 Homo_sapiens_6_18755932-18757277-HERVHF-5LTR - 6
18743775 18757467 NONHSAT107982.2 - 393
6 18754988 18756902 Homo_sapiens_6_18755932-18757277-HERVHF-pol - 6
18743775 18757467 NONHSAG043117.2 - 1914
6 18754988 18756902 Homo_sapiens_6_18755932-18757277-HERVHF-pol - 6
18743775 18757467 NONHSAT107982.2 - 1914
6 80509795 80515805 Homo_sapiens_6_80511941-80513513-HERVHF - 6 80487666
80548762 NONHSAG113295.1 - 6010
6 80509795 80510208 Homo_sapiens_6_80511941-80513513-HERVHF-5LTR - 6
80487666 80548762 NONHSAG113295.1 - 413
6 80509795 80515805 Homo_sapiens_6_80511941-80513513-HERVHF - 6 80487666
80548659 NONHSAT252454.1 - 6010
6 80509795 80510208 Homo_sapiens_6_80511941-80513513-HERVHF-5LTR - 6
80487666 80548659 NONHSAT252454.1 - 413
6 80510376 80513272 Homo_sapiens_6_80511941-80513513-HERVHF-pol - 6
80487666 80548762 NONHSAG113295.1 - 2896
6 80510376 80513272 Homo_sapiens_6_80511941-80513513-HERVHF-pol - 6
80487666 80548659 NONHSAT252454.1 - 2896
6 80512745 80513302 Homo_sapiens_6_80511941-80513513-HERVHF-pro - 6
80487666 80548762 NONHSAG113295.1 - 557
6 80512745 80513302 Homo_sapiens_6_80511941-80513513-HERVHF-pro - 6
80487666 80548659 NONHSAT252454.1 - 557
6 80513596 80513907 Homo_sapiens_6_80511941-80513513-HERVHF-gag - 6
80487666 80548762 NONHSAG113295.1 - 311
6 80513596 80513907 Homo_sapiens_6_80511941-80513513-HERVHF-gag - 6
80487666 80548659 NONHSAT252454.1 - 311
6 80515391 80515805 Homo_sapiens_6_80511941-80513513-HERVHF-3LTR - 6
80487666 80548762 NONHSAG113295.1 - 414
6 80515391 80515805 Homo_sapiens_6_80511941-80513513-HERVHF-3LTR - 6
80487666 80548659 NONHSAT252454.1 - 414
6 80509795 80515805 Homo_sapiens_6_80511941-80513513-HERVHF - 6 80508653
80548762 NONHSAT252455.1 - 6010
6 80509795 80510208 Homo_sapiens_6_80511941-80513513-HERVHF-5LTR - 6
80508653 80548762 NONHSAT252455.1 - 413
6 80510376 80513272 Homo_sapiens_6_80511941-80513513-HERVHF-pol - 6
80508653 80548762 NONHSAT252455.1 - 2896
6 80512745 80513302 Homo_sapiens_6_80511941-80513513-HERVHF-pro - 6
80508653 80548762 NONHSAT252455.1 - 557
6 80513596 80513907 Homo_sapiens_6_80511941-80513513-HERVHF-gag - 6
80508653 80548762 NONHSAT252455.1 - 311
6 80515391 80515805 Homo_sapiens_6_80511941-80513513-HERVHF-3LTR - 6
80508653 80548762 NONHSAT252455.1 - 414
6 94553917 94559610 Homo_sapiens_6_94555806-94557152-HERVHF - 6 94546196

94607932 NONHSAG044390.2 - 5693
6 94553917 94554368 Homo_sapiens_6_94555806-94557152-HERVHF-5LTR - 6
94546196 94607932 NONHSAG044390.2 - 451
6 94553917 94559610 Homo_sapiens_6_94555806-94557152-HERVHF - 6 94546196
94573623 NONHSAT209443.1 - 5693
6 94553917 94554368 Homo_sapiens_6_94555806-94557152-HERVHF-5LTR - 6
94546196 94573623 NONHSAT209443.1 - 451
6 94554823 94556910 Homo_sapiens_6_94555806-94557152-HERVHF-pol - 6
94546196 94607932 NONHSAG044390.2 - 2087
6 94554823 94556910 Homo_sapiens_6_94555806-94557152-HERVHF-pol - 6
94546196 94573623 NONHSAT209443.1 - 2087
6 94556395 94557201 Homo_sapiens_6_94555806-94557152-HERVHF-pro - 6
94546196 94607932 NONHSAG044390.2 - 806
6 94556395 94557201 Homo_sapiens_6_94555806-94557152-HERVHF-pro - 6
94546196 94573623 NONHSAT209443.1 - 806
6 94559159 94559610 Homo_sapiens_6_94555806-94557152-HERVHF-3LTR - 6
94546196 94607932 NONHSAG044390.2 - 451
6 94559159 94559610 Homo_sapiens_6_94555806-94557152-HERVHF-3LTR - 6
94546196 94573623 NONHSAT209443.1 - 451
6 94553917 94559610 Homo_sapiens_6_94555806-94557152-HERVHF - 6 94546571
94607932 NONHSAT114040.2- 5693
6 94553917 94554368 Homo_sapiens_6_94555806-94557152-HERVHF-5LTR - 6
94546571 94607932 NONHSAT114040.2- 451
6 94554823 94556910 Homo_sapiens_6_94555806-94557152-HERVHF-pol - 6
94546571 94607932 NONHSAT114040.2- 2087
6 94556395 94557201 Homo_sapiens_6_94555806-94557152-HERVHF-pro - 6
94546571 94607932 NONHSAT114040.2- 806
6 94559159 94559610 Homo_sapiens_6_94555806-94557152-HERVHF-3LTR - 6
94546571 94607932 NONHSAT114040.2- 451
6 97779489 97785327 Homo_sapiens_6_97782122-97783636-HERVHF + 6 97282325
98400189 NONHSAG044421.3 + 5838
6 97779489 97779949 Homo_sapiens_6_97782122-97783636-HERVHF-5LTR + 6
97282325 98400189 NONHSAG044421.3 + 460
6 97782076 97782870 Homo_sapiens_6_97782122-97783636-HERVHF-pro + 6
97282325 98400189 NONHSAG044421.3 + 794
6 97782271 97784463 Homo_sapiens_6_97782122-97783636-HERVHF-pol + 6
97282325 98400189 NONHSAG044421.3 + 2192
6 97784874 97785327 Homo_sapiens_6_97782122-97783636-HERVHF-3LTR + 6
97282325 98400189 NONHSAG044421.3 + 453
6 97779489 97785327 Homo_sapiens_6_97782122-97783636-HERVHF + 6 97305553
98398783 ENSG00000271860 + 5838
6 97779489 97779949 Homo_sapiens_6_97782122-97783636-HERVHF-5LTR + 6
97305553 98398783 ENSG00000271860 + 460
6 97782076 97782870 Homo_sapiens_6_97782122-97783636-HERVHF-pro + 6

97305553 98398783 ENSG00000271860 + 794
6 97782271 97784463 Homo_sapiens_6_97782122-97783636-HERVHF-pol + 6
97305553 98398783 ENSG00000271860 + 2192
6 97784874 97785327 Homo_sapiens_6_97782122-97783636-HERVHF-3LTR + 6
97305553 98398783 ENSG00000271860 + 453
6 97779489 97785327 Homo_sapiens_6_97782122-97783636-HERVHF + 6 97305577
98398863 ENSG00000271860 + 5838
6 97779489 97779949 Homo_sapiens_6_97782122-97783636-HERVHF-5LTR + 6
97305577 98398863 ENSG00000271860 + 460
6 97782076 97782870 Homo_sapiens_6_97782122-97783636-HERVHF-pro + 6
97305577 98398863 ENSG00000271860 + 794
6 97782271 97784463 Homo_sapiens_6_97782122-97783636-HERVHF-pol + 6
97305577 98398863 ENSG00000271860 + 2192
6 97784874 97785327 Homo_sapiens_6_97782122-97783636-HERVHF-3LTR + 6
97305577 98398863 ENSG00000271860 + 453
6 97779489 97785327 Homo_sapiens_6_97782122-97783636-HERVHF + 6 97305592
97828320 ENSG00000271860 + 5838
6 97779489 97779949 Homo_sapiens_6_97782122-97783636-HERVHF-5LTR + 6
97305592 97828320 ENSG00000271860 + 460
6 97782076 97782870 Homo_sapiens_6_97782122-97783636-HERVHF-pro + 6
97305592 97828320 ENSG00000271860 + 794
6 97782271 97784463 Homo_sapiens_6_97782122-97783636-HERVHF-pol + 6
97305592 97828320 ENSG00000271860 + 2192
6 97784874 97785327 Homo_sapiens_6_97782122-97783636-HERVHF-3LTR + 6
97305592 97828320 ENSG00000271860 + 453
6 97779489 97785327 Homo_sapiens_6_97782122-97783636-HERVHF + 6 97305607
98399462 NONHSAT251316.1 + 5838
6 97779489 97779949 Homo_sapiens_6_97782122-97783636-HERVHF-5LTR + 6
97305607 98399462 NONHSAT251316.1 + 460
6 97782076 97782870 Homo_sapiens_6_97782122-97783636-HERVHF-pro + 6
97305607 98399462 NONHSAT251316.1 + 794
6 97782271 97784463 Homo_sapiens_6_97782122-97783636-HERVHF-pol + 6
97305607 98399462 NONHSAT251316.1 + 2192
6 97784874 97785327 Homo_sapiens_6_97782122-97783636-HERVHF-3LTR + 6
97305607 98399462 NONHSAT251316.1 + 453
6 97779489 97785327 Homo_sapiens_6_97782122-97783636-HERVHF + 6 97305881
98133972 ENSG00000271860 + 5838
6 97779489 97779949 Homo_sapiens_6_97782122-97783636-HERVHF-5LTR + 6
97305881 98133972 ENSG00000271860 + 460
6 97782076 97782870 Homo_sapiens_6_97782122-97783636-HERVHF-pro + 6
97305881 98133972 ENSG00000271860 + 794
6 97782271 97784463 Homo_sapiens_6_97782122-97783636-HERVHF-pol + 6
97305881 98133972 ENSG00000271860 + 2192
6 97784874 97785327 Homo_sapiens_6_97782122-97783636-HERVHF-3LTR + 6

97305881 98133972 ENSG00000271860 + 453
6 97779489 97785327 Homo_sapiens_6_97782122-97783636-HERVHF + 6 97412357 97839433 NONHSAG093448.1 + 5838
6 97779489 97779949 Homo_sapiens_6_97782122-97783636-HERVHF-5LTR + 6 97412357 97839433 NONHSAG093448.1 + 460
6 97779489 97785327 Homo_sapiens_6_97782122-97783636-HERVHF + 6 97412357 97815571 NONHSAT207696.1 + 5838
6 97779489 97779949 Homo_sapiens_6_97782122-97783636-HERVHF-5LTR + 6 97412357 97815571 NONHSAT207696.1 + 460
6 97782076 97782870 Homo_sapiens_6_97782122-97783636-HERVHF-pro + 6 97412357 97839433 NONHSAG093448.1 + 794
6 97782076 97782870 Homo_sapiens_6_97782122-97783636-HERVHF-pro + 6 97412357 97815571 NONHSAT207696.1 + 794
6 97782271 97784463 Homo_sapiens_6_97782122-97783636-HERVHF-pol + 6 97412357 97839433 NONHSAG093448.1 + 2192
6 97782271 97784463 Homo_sapiens_6_97782122-97783636-HERVHF-pol + 6 97412357 97815571 NONHSAT207696.1 + 2192
6 97784874 97785327 Homo_sapiens_6_97782122-97783636-HERVHF-3LTR + 6 97412357 97839433 NONHSAG093448.1 + 453
6 97784874 97785327 Homo_sapiens_6_97782122-97783636-HERVHF-3LTR + 6 97412357 97815571 NONHSAT207696.1 + 453
6 97779489 97785327 Homo_sapiens_6_97782122-97783636-HERVHF + 6 97440833 98398863 ENSG00000271860 + 5838
6 97779489 97779949 Homo_sapiens_6_97782122-97783636-HERVHF-5LTR + 6 97440833 98398863 ENSG00000271860 + 460
6 97782076 97782870 Homo_sapiens_6_97782122-97783636-HERVHF-pro + 6 97440833 98398863 ENSG00000271860 + 794
6 97782271 97784463 Homo_sapiens_6_97782122-97783636-HERVHF-pol + 6 97440833 98398863 ENSG00000271860 + 2192
6 97784874 97785327 Homo_sapiens_6_97782122-97783636-HERVHF-3LTR + 6 97440833 98398863 ENSG00000271860 + 453
6 97779489 97785327 Homo_sapiens_6_97782122-97783636-HERVHF + 6 97440844 97828109 NONHSAT251320.1 + 5838
6 97779489 97779949 Homo_sapiens_6_97782122-97783636-HERVHF-5LTR + 6 97440844 97828109 NONHSAT251320.1 + 460
6 97779489 97785327 Homo_sapiens_6_97782122-97783636-HERVHF + 6 97440844 97998895 NONHSAT251321.1 + 5838
6 97779489 97779949 Homo_sapiens_6_97782122-97783636-HERVHF-5LTR + 6 97440844 97998895 NONHSAT251321.1 + 460
6 97782076 97782870 Homo_sapiens_6_97782122-97783636-HERVHF-pro + 6 97440844 97828109 NONHSAT251320.1 + 794
6 97782076 97782870 Homo_sapiens_6_97782122-97783636-HERVHF-pro + 6 97440844 97998895 NONHSAT251321.1 + 794
6 97782271 97784463 Homo_sapiens_6_97782122-97783636-HERVHF-pol + 6

97440844 97828109 NONHSAT251320.1 + 2192
6 97782271 97784463 Homo_sapiens_6_97782122-97783636-HERVHF-pol + 6
97440844 97998895 NONHSAT251321.1 + 2192
6 97784874 97785327 Homo_sapiens_6_97782122-97783636-HERVHF-3LTR + 6
97440844 97828109 NONHSAT251320.1 + 453
6 97784874 97785327 Homo_sapiens_6_97782122-97783636-HERVHF-3LTR + 6
97440844 97998895 NONHSAT251321.1 + 453
6 97779489 97785327 Homo_sapiens_6_97782122-97783636-HERVHF + 6 97440845
98398863 ENSG00000271860 + 5838
6 97779489 97779949 Homo_sapiens_6_97782122-97783636-HERVHF-5LTR + 6
97440845 98398863 ENSG00000271860 + 460
6 97779489 97785327 Homo_sapiens_6_97782122-97783636-HERVHF + 6 97440845
98398863 ENSG00000271860 + 5838
6 97779489 97779949 Homo_sapiens_6_97782122-97783636-HERVHF-5LTR + 6
97440845 98398863 ENSG00000271860 + 460
6 97782076 97782870 Homo_sapiens_6_97782122-97783636-HERVHF-pro + 6
97440845 98398863 ENSG00000271860 + 794
6 97782076 97782870 Homo_sapiens_6_97782122-97783636-HERVHF-pro + 6
97440845 98398863 ENSG00000271860 + 794
6 97782271 97784463 Homo_sapiens_6_97782122-97783636-HERVHF-pol + 6
97440845 98398863 ENSG00000271860 + 2192
6 97782271 97784463 Homo_sapiens_6_97782122-97783636-HERVHF-pol + 6
97440845 98398863 ENSG00000271860 + 2192
6 97784874 97785327 Homo_sapiens_6_97782122-97783636-HERVHF-3LTR + 6
97440845 98398863 ENSG00000271860 + 453
6 97784874 97785327 Homo_sapiens_6_97782122-97783636-HERVHF-3LTR + 6
97440845 98398863 ENSG00000271860 + 453
6 97779489 97785327 Homo_sapiens_6_97782122-97783636-HERVHF + 6 97440852
98133704 ENSG00000271860 + 5838
6 97779489 97779949 Homo_sapiens_6_97782122-97783636-HERVHF-5LTR + 6
97440852 98133704 ENSG00000271860 + 460
6 97782076 97782870 Homo_sapiens_6_97782122-97783636-HERVHF-pro + 6
97440852 98133704 ENSG00000271860 + 794
6 97782271 97784463 Homo_sapiens_6_97782122-97783636-HERVHF-pol + 6
97440852 98133704 ENSG00000271860 + 2192
6 97784874 97785327 Homo_sapiens_6_97782122-97783636-HERVHF-3LTR + 6
97440852 98133704 ENSG00000271860 + 453
6 97779489 97785327 Homo_sapiens_6_97782122-97783636-HERVHF + 6 97440853
98099256 ENSG00000271860 + 5838
6 97779489 97779949 Homo_sapiens_6_97782122-97783636-HERVHF-5LTR + 6
97440853 98099256 ENSG00000271860 + 460
6 97782076 97782870 Homo_sapiens_6_97782122-97783636-HERVHF-pro + 6
97440853 98099256 ENSG00000271860 + 794
6 97782271 97784463 Homo_sapiens_6_97782122-97783636-HERVHF-pol + 6

97440853 98099256 ENSG00000271860 + 2192
6 97784874 97785327 Homo_sapiens_6_97782122-97783636-HERVHF-3LTR + 6
97440853 98099256 ENSG00000271860 + 453
6 97779489 97785327 Homo_sapiens_6_97782122-97783636-HERVHF + 6 97627074
97969561 NONHSAT251327.1 + 5838
6 97779489 97779949 Homo_sapiens_6_97782122-97783636-HERVHF-5LTR + 6
97627074 97969561 NONHSAT251327.1 + 460
6 97782076 97782870 Homo_sapiens_6_97782122-97783636-HERVHF-pro + 6
97627074 97969561 NONHSAT251327.1 + 794
6 97782271 97784463 Homo_sapiens_6_97782122-97783636-HERVHF-pol + 6
97627074 97969561 NONHSAT251327.1 + 2192
6 97784874 97785327 Homo_sapiens_6_97782122-97783636-HERVHF-3LTR + 6
97627074 97969561 NONHSAT251327.1 + 453
6 97779489 97779949 Homo_sapiens_6_97782122-97783636-HERVHF-5LTR + 6
97773576 97782168 NONHSAG093449.1 + 460
6 97779489 97779949 Homo_sapiens_6_97782122-97783636-HERVHF-5LTR + 6
97773576 97782168 NONHSAT207699.1 + 460
6 123582333 123588007 Homo_sapiens_6_123584156-123585562-HERVHF - 6
123216339 123636950 ENSG00000186439 - 5674
6 123582333 123582734 Homo_sapiens_6_123584156-123585562-HERVHF-5LTR -
6 123216339 123636950 ENSG00000186439 - 401
6 123584586 123585260 Homo_sapiens_6_123584156-123585562-HERVHF-pol - 6
123216339 123636950 ENSG00000186439 - 674
6 123584721 123585290 Homo_sapiens_6_123584156-123585562-HERVHF-pro - 6
123216339 123636950 ENSG00000186439 - 569
6 123587604 123588007 Homo_sapiens_6_123584156-123585562-HERVHF-3LTR -
6 123216339 123636950 ENSG00000186439 - 403
6 123582333 123588007 Homo_sapiens_6_123584156-123585562-HERVHF - 6
123349475 123636988 ENSG00000186439 - 5674
6 123582333 123582734 Homo_sapiens_6_123584156-123585562-HERVHF-5LTR -
6 123349475 123636988 ENSG00000186439 - 401
6 123584586 123585260 Homo_sapiens_6_123584156-123585562-HERVHF-pol - 6
123349475 123636988 ENSG00000186439 - 674
6 123584721 123585290 Homo_sapiens_6_123584156-123585562-HERVHF-pro - 6
123349475 123636988 ENSG00000186439 - 569
6 123587604 123588007 Homo_sapiens_6_123584156-123585562-HERVHF-3LTR -
6 123349475 123636988 ENSG00000186439 - 403
6 123582333 123588007 Homo_sapiens_6_123584156-123585562-HERVHF - 6
123464250 123636909 ENSG00000186439 - 5674
6 123582333 123582734 Homo_sapiens_6_123584156-123585562-HERVHF-5LTR -
6 123464250 123636909 ENSG00000186439 - 401
6 123584586 123585260 Homo_sapiens_6_123584156-123585562-HERVHF-pol - 6
123464250 123636909 ENSG00000186439 - 674
6 123584721 123585290 Homo_sapiens_6_123584156-123585562-HERVHF-pro - 6

123464250	123636909	ENSG00000186439 -	569
6	123587604	123588007	Homo_sapiens_6_123584156-123585562-HERVHF-3LTR	-
6	123464250	123636909	ENSG00000186439 -	403
6	123582333	123588007	Homo_sapiens_6_123584156-123585562-HERVHF	-	6
123503651	123637093	ENSG00000186439 -	5674
6	123582333	123582734	Homo_sapiens_6_123584156-123585562-HERVHF-5LTR	-
6	123503651	123637093	ENSG00000186439 -	401
6	123584586	123585260	Homo_sapiens_6_123584156-123585562-HERVHF-pol -	6
123503651	123637093	ENSG00000186439 -	674
6	123584721	123585290	Homo_sapiens_6_123584156-123585562-HERVHF-pro -	6
123503651	123637093	ENSG00000186439 -	569
6	123587604	123588007	Homo_sapiens_6_123584156-123585562-HERVHF-3LTR	-
6	123503651	123637093	ENSG00000186439 -	403
6	123582333	123588007	Homo_sapiens_6_123584156-123585562-HERVHF	-	6
123528681	123636972	ENSG00000186439 -	5674
6	123582333	123582734	Homo_sapiens_6_123584156-123585562-HERVHF-5LTR	-
6	123528681	123636972	ENSG00000186439 -	401
6	123584586	123585260	Homo_sapiens_6_123584156-123585562-HERVHF-pol -	6
123528681	123636972	ENSG00000186439 -	674
6	123584721	123585290	Homo_sapiens_6_123584156-123585562-HERVHF-pro -	6
123528681	123636972	ENSG00000186439 -	569
6	123587604	123588007	Homo_sapiens_6_123584156-123585562-HERVHF-3LTR	-
6	123528681	123636972	ENSG00000186439 -	403
6	125701846	125707764	Homo_sapiens_6_125703727-125705069-HERVHF	-	6
125575411	125749395	NONHSAG044767.3	-	5918
6	125701846	125702300	Homo_sapiens_6_125703727-125705069-HERVHF-5LTR	-
6	125575411	125749395	NONHSAG044767.3	-	454
6	125701846	125707764	Homo_sapiens_6_125703727-125705069-HERVHF	-	6
125575411	125749395	NONHSAT252665.1	-	5918
6	125701846	125702300	Homo_sapiens_6_125703727-125705069-HERVHF-5LTR	-
6	125575411	125749395	NONHSAT252665.1	-	454
6	125701846	125707764	Homo_sapiens_6_125703727-125705069-HERVHF	-	6
125575411	125749395	NONHSAT252666.1	-	5918
6	125701846	125702300	Homo_sapiens_6_125703727-125705069-HERVHF-5LTR	-
6	125575411	125749395	NONHSAT252666.1	-	454
6	125701846	125707764	Homo_sapiens_6_125703727-125705069-HERVHF	-	6
125575411	125749395	NONHSAT252667.1	-	5918
6	125701846	125702300	Homo_sapiens_6_125703727-125705069-HERVHF-5LTR	-
6	125575411	125749395	NONHSAT252667.1	-	454
6	125702812	125704819	Homo_sapiens_6_125703727-125705069-HERVHF-pol -	6
125575411	125749395	NONHSAG044767.3	-	2007
6	125702812	125704819	Homo_sapiens_6_125703727-125705069-HERVHF-pol -	6
125575411	125749395	NONHSAT252665.1	-	2007
6	125702812	125704819	Homo_sapiens_6_125703727-125705069-HERVHF-pol -	6

125575411	125749395	NONHSAT252666.1	-	2007
6	125702812	125704819	Homo_sapiens_6_125703727-125705069-HERVHF-pol -	6
125575411	125749395	NONHSAT252667.1	-	2007
6	125705125	125705535	Homo_sapiens_6_125703727-125705069-HERVHF-gag -	6
125575411	125749395	NONHSAG044767.3	-	410
6	125705125	125705535	Homo_sapiens_6_125703727-125705069-HERVHF-gag -	6
125575411	125749395	NONHSAT252665.1	-	410
6	125705125	125705535	Homo_sapiens_6_125703727-125705069-HERVHF-gag -	6
125575411	125749395	NONHSAT252666.1	-	410
6	125705125	125705535	Homo_sapiens_6_125703727-125705069-HERVHF-gag -	6
125575411	125749395	NONHSAT252667.1	-	410
6	125707310	125707764	Homo_sapiens_6_125703727-125705069-HERVHF-3LTR	-
6	125575411	125749395	NONHSAG044767.3	-	454
6	125707310	125707764	Homo_sapiens_6_125703727-125705069-HERVHF-3LTR	-
6	125575411	125749395	NONHSAT252665.1	-	454
6	125707310	125707764	Homo_sapiens_6_125703727-125705069-HERVHF-3LTR	-
6	125575411	125749395	NONHSAT252666.1	-	454
6	125707310	125707764	Homo_sapiens_6_125703727-125705069-HERVHF-3LTR	-
6	125575411	125749395	NONHSAT252667.1	-	454
6	125701846	125707764	Homo_sapiens_6_125703727-125705069-HERVHF	-	6
125577545	125749180	ENSG00000237742 -	5918
6	125701846	125702300	Homo_sapiens_6_125703727-125705069-HERVHF-5LTR	-
6	125577545	125749180	ENSG00000237742 -	454
6	125702812	125704819	Homo_sapiens_6_125703727-125705069-HERVHF-pol -	6
125577545	125749180	ENSG00000237742 -	2007
6	125705125	125705535	Homo_sapiens_6_125703727-125705069-HERVHF-gag -	6
125577545	125749180	ENSG00000237742 -	410
6	125707310	125707764	Homo_sapiens_6_125703727-125705069-HERVHF-3LTR	-
6	125577545	125749180	ENSG00000237742 -	454
6	125701846	125707764	Homo_sapiens_6_125703727-125705069-HERVHF	-	6
125577575	125745622	NONHSAT252668.1	-	5918
6	125701846	125702300	Homo_sapiens_6_125703727-125705069-HERVHF-5LTR	-
6	125577575	125745622	NONHSAT252668.1	-	454
6	125702812	125704819	Homo_sapiens_6_125703727-125705069-HERVHF-pol -	6
125577575	125745622	NONHSAT252668.1	-	2007
6	125705125	125705535	Homo_sapiens_6_125703727-125705069-HERVHF-gag -	6
125577575	125745622	NONHSAT252668.1	-	410
6	125707310	125707764	Homo_sapiens_6_125703727-125705069-HERVHF-3LTR	-
6	125577575	125745622	NONHSAT252668.1	-	454
6	125701846	125707764	Homo_sapiens_6_125703727-125705069-HERVHF	-	6
125578557	125745622	NONHSAT209650.1	-	5918
6	125701846	125702300	Homo_sapiens_6_125703727-125705069-HERVHF-5LTR	-
6	125578557	125745622	NONHSAT209650.1	-	454
6	125702812	125704819	Homo_sapiens_6_125703727-125705069-HERVHF-pol -	6

125578557 125745622 NONHSAT209650.1 - 2007
6 125705125 125705535 Homo_sapiens_6_125703727-125705069-HERVHF-gag - 6
125578557 125745622 NONHSAT209650.1 - 410
6 125707310 125707764 Homo_sapiens_6_125703727-125705069-HERVHF-3LTR -
6 125578557 125745622 NONHSAT209650.1 - 454
6 125701846 125707764 Homo_sapiens_6_125703727-125705069-HERVHF - 6
125578558 125745610 ENSG00000237742 - 5918
6 125701846 125702300 Homo_sapiens_6_125703727-125705069-HERVHF-5LTR -
6 125578558 125745610 ENSG00000237742 - 454
6 125701846 125707764 Homo_sapiens_6_125703727-125705069-HERVHF - 6
125578558 125749097 NONHSAT209651.1 - 5918
6 125701846 125702300 Homo_sapiens_6_125703727-125705069-HERVHF-5LTR -
6 125578558 125749097 NONHSAT209651.1 - 454
6 125702812 125704819 Homo_sapiens_6_125703727-125705069-HERVHF-pol - 6
125578558 125745610 ENSG00000237742 - 2007
6 125702812 125704819 Homo_sapiens_6_125703727-125705069-HERVHF-pol - 6
125578558 125749097 NONHSAT209651.1 - 2007
6 125705125 125705535 Homo_sapiens_6_125703727-125705069-HERVHF-gag - 6
125578558 125745610 ENSG00000237742 - 410
6 125705125 125705535 Homo_sapiens_6_125703727-125705069-HERVHF-gag - 6
125578558 125749097 NONHSAT209651.1 - 410
6 125707310 125707764 Homo_sapiens_6_125703727-125705069-HERVHF-3LTR -
6 125578558 125745610 ENSG00000237742 - 454
6 125707310 125707764 Homo_sapiens_6_125703727-125705069-HERVHF-3LTR -
6 125578558 125749097 NONHSAT209651.1 - 454
6 125701846 125707764 Homo_sapiens_6_125703727-125705069-HERVHF - 6
125578566 125745028 NONHSAT114802.2- 5918
6 125701846 125702300 Homo_sapiens_6_125703727-125705069-HERVHF-5LTR -
6 125578566 125745028 NONHSAT114802.2- 454
6 125701846 125707764 Homo_sapiens_6_125703727-125705069-HERVHF - 6
125578566 125749186 NONHSAT114803.2- 5918
6 125701846 125702300 Homo_sapiens_6_125703727-125705069-HERVHF-5LTR -
6 125578566 125749186 NONHSAT114803.2- 454
6 125701846 125707764 Homo_sapiens_6_125703727-125705069-HERVHF - 6
125578566 125745622 NONHSAT209652.1 - 5918
6 125701846 125702300 Homo_sapiens_6_125703727-125705069-HERVHF-5LTR -
6 125578566 125745622 NONHSAT209652.1 - 454
6 125701846 125707764 Homo_sapiens_6_125703727-125705069-HERVHF - 6
125578566 125749186 NONHSAT209653.1 - 5918
6 125701846 125702300 Homo_sapiens_6_125703727-125705069-HERVHF-5LTR -
6 125578566 125749186 NONHSAT209653.1 - 454
6 125702812 125704819 Homo_sapiens_6_125703727-125705069-HERVHF-pol - 6
125578566 125745028 NONHSAT114802.2- 2007
6 125702812 125704819 Homo_sapiens_6_125703727-125705069-HERVHF-pol - 6

125578566 125749186 NONHSAT114803.2- 2007
6 125702812 125704819 Homo_sapiens_6_125703727-125705069-HERVHF-pol - 6
125578566 125745622 NONHSAT209652.1 - 2007
6 125702812 125704819 Homo_sapiens_6_125703727-125705069-HERVHF-pol - 6
125578566 125749186 NONHSAT209653.1 - 2007
6 125705125 125705535 Homo_sapiens_6_125703727-125705069-HERVHF-gag - 6
125578566 125745028 NONHSAT114802.2- 410
6 125705125 125705535 Homo_sapiens_6_125703727-125705069-HERVHF-gag - 6
125578566 125749186 NONHSAT114803.2- 410
6 125705125 125705535 Homo_sapiens_6_125703727-125705069-HERVHF-gag - 6
125578566 125745622 NONHSAT209652.1 - 410
6 125705125 125705535 Homo_sapiens_6_125703727-125705069-HERVHF-gag - 6
125578566 125749186 NONHSAT209653.1 - 410
6 125707310 125707764 Homo_sapiens_6_125703727-125705069-HERVHF-3LTR -
6 125578566 125745028 NONHSAT114802.2- 454
6 125707310 125707764 Homo_sapiens_6_125703727-125705069-HERVHF-3LTR -
6 125578566 125749186 NONHSAT114803.2- 454
6 125707310 125707764 Homo_sapiens_6_125703727-125705069-HERVHF-3LTR -
6 125578566 125745622 NONHSAT209652.1 - 454
6 125707310 125707764 Homo_sapiens_6_125703727-125705069-HERVHF-3LTR -
6 125578566 125749186 NONHSAT209653.1 - 454
6 125701846 125707764 Homo_sapiens_6_125703727-125705069-HERVHF - 6
125578567 125745028 ENSG00000237742 - 5918
6 125701846 125702300 Homo_sapiens_6_125703727-125705069-HERVHF-5LTR -
6 125578567 125745028 ENSG00000237742 - 454
6 125701846 125707764 Homo_sapiens_6_125703727-125705069-HERVHF - 6
125578567 125749186 ENSG00000237742 - 5918
6 125701846 125702300 Homo_sapiens_6_125703727-125705069-HERVHF-5LTR -
6 125578567 125749186 ENSG00000237742 - 454
6 125701846 125707764 Homo_sapiens_6_125703727-125705069-HERVHF - 6
125578567 125749187 NONHSAT209654.1 - 5918
6 125701846 125702300 Homo_sapiens_6_125703727-125705069-HERVHF-5LTR -
6 125578567 125749187 NONHSAT209654.1 - 454
6 125702812 125704819 Homo_sapiens_6_125703727-125705069-HERVHF-pol - 6
125578567 125745028 ENSG00000237742 - 2007
6 125702812 125704819 Homo_sapiens_6_125703727-125705069-HERVHF-pol - 6
125578567 125749186 ENSG00000237742 - 2007
6 125702812 125704819 Homo_sapiens_6_125703727-125705069-HERVHF-pol - 6
125578567 125749187 NONHSAT209654.1 - 2007
6 125705125 125705535 Homo_sapiens_6_125703727-125705069-HERVHF-gag - 6
125578567 125745028 ENSG00000237742 - 410
6 125705125 125705535 Homo_sapiens_6_125703727-125705069-HERVHF-gag - 6
125578567 125749186 ENSG00000237742 - 410
6 125705125 125705535 Homo_sapiens_6_125703727-125705069-HERVHF-gag - 6

125578567 125749187 NONHSAT209654.1 - 410
6 125707310 125707764 Homo_sapiens_6_125703727-125705069-HERVHF-3LTR -
6 125578567 125745028 ENSG00000237742 - 454
6 125707310 125707764 Homo_sapiens_6_125703727-125705069-HERVHF-3LTR -
6 125578567 125749186 ENSG00000237742 - 454
6 125707310 125707764 Homo_sapiens_6_125703727-125705069-HERVHF-3LTR -
6 125578567 125749187 NONHSAT209654.1 - 454
6 125701846 125707764 Homo_sapiens_6_125703727-125705069-HERVHF - 6
125686162 125749395 NONHSAT252669.1 - 5918
6 125701846 125702300 Homo_sapiens_6_125703727-125705069-HERVHF-5LTR -
6 125686162 125749395 NONHSAT252669.1 - 454
6 125702812 125704819 Homo_sapiens_6_125703727-125705069-HERVHF-pol - 6
125686162 125749395 NONHSAT252669.1 - 2007
6 125705125 125705535 Homo_sapiens_6_125703727-125705069-HERVHF-gag - 6
125686162 125749395 NONHSAT252669.1 - 410
6 125707310 125707764 Homo_sapiens_6_125703727-125705069-HERVHF-3LTR -
6 125686162 125749395 NONHSAT252669.1 - 454
6 126851224 126852054 Homo_sapiens_6_126851273-126852794-HERVHF-pro + 6
126851010 126882099 NONHSAG044785.3 + 830
6 126851224 126852054 Homo_sapiens_6_126851273-126852794-HERVHF-pro + 6
126851010 126882099 NONHSAT251492.1 + 830
6 126851557 126853403 Homo_sapiens_6_126851273-126852794-HERVHF-pol + 6
126851010 126882099 NONHSAG044785.3 + 1846
6 126851557 126853403 Homo_sapiens_6_126851273-126852794-HERVHF-pol + 6
126851010 126882099 NONHSAT251492.1 + 1846
6 126854026 126854456 Homo_sapiens_6_126851273-126852794-HERVHF-3LTR +
6 126851010 126882099 NONHSAG044785.3 + 430
6 126854026 126854456 Homo_sapiens_6_126851273-126852794-HERVHF-3LTR +
6 126851010 126882099 NONHSAT251492.1 + 430
6 131295347 131301206 Homo_sapiens_6_131297975-131299503-HERVHF + 6
131294420 131348082 NONHSAG093612.2 + 5859
6 131295347 131295808 Homo_sapiens_6_131297975-131299503-HERVHF-5LTR +
6 131294420 131348082 NONHSAG093612.2 + 461
6 131295347 131301206 Homo_sapiens_6_131297975-131299503-HERVHF + 6
131294420 131301790 NONHSAT251499.1 + 5859
6 131295347 131295808 Homo_sapiens_6_131297975-131299503-HERVHF-5LTR +
6 131294420 131301790 NONHSAT251499.1 + 461
6 131295347 131301206 Homo_sapiens_6_131297975-131299503-HERVHF + 6
131294420 131309129 NONHSAT251500.1 + 5859
6 131295347 131295808 Homo_sapiens_6_131297975-131299503-HERVHF-5LTR +
6 131294420 131309129 NONHSAT251500.1 + 461
6 131295347 131301206 Homo_sapiens_6_131297975-131299503-HERVHF + 6
131294420 131319640 NONHSAT251501.1 + 5859
6 131295347 131295808 Homo_sapiens_6_131297975-131299503-HERVHF-5LTR +

6 131294420 131319640 NONHSAT251501.1 + 461
6 131295347 131301206 Homo_sapiens_6_131297975-131299503-HERVHF + 6
131294420 131328725 NONHSAT251502.1 + 5859
6 131295347 131295808 Homo_sapiens_6_131297975-131299503-HERVHF-5LTR +
6 131294420 131328725 NONHSAT251502.1 + 461
6 131295347 131301206 Homo_sapiens_6_131297975-131299503-HERVHF + 6
131294420 131344687 NONHSAT251503.1 + 5859
6 131295347 131295808 Homo_sapiens_6_131297975-131299503-HERVHF-5LTR +
6 131294420 131344687 NONHSAT251503.1 + 461
6 131298228 131299742 Homo_sapiens_6_131297975-131299503-HERVHF-pol + 6
131294420 131348082 NONHSAG093612.2 + 1514
6 131298228 131299742 Homo_sapiens_6_131297975-131299503-HERVHF-pol + 6
131294420 131301790 NONHSAT251499.1 + 1514
6 131298228 131299742 Homo_sapiens_6_131297975-131299503-HERVHF-pol + 6
131294420 131309129 NONHSAT251500.1 + 1514
6 131298228 131299742 Homo_sapiens_6_131297975-131299503-HERVHF-pol + 6
131294420 131319640 NONHSAT251501.1 + 1514
6 131298228 131299742 Homo_sapiens_6_131297975-131299503-HERVHF-pol + 6
131294420 131328725 NONHSAT251502.1 + 1514
6 131298228 131299742 Homo_sapiens_6_131297975-131299503-HERVHF-pol + 6
131294420 131344687 NONHSAT251503.1 + 1514
6 131300740 131301206 Homo_sapiens_6_131297975-131299503-HERVHF-3LTR +
6 131294420 131348082 NONHSAG093612.2 + 466
6 131300740 131301206 Homo_sapiens_6_131297975-131299503-HERVHF-3LTR +
6 131294420 131301790 NONHSAT251499.1 + 466
6 131300740 131301206 Homo_sapiens_6_131297975-131299503-HERVHF-3LTR +
6 131294420 131309129 NONHSAT251500.1 + 466
6 131300740 131301206 Homo_sapiens_6_131297975-131299503-HERVHF-3LTR +
6 131294420 131319640 NONHSAT251501.1 + 466
6 131300740 131301206 Homo_sapiens_6_131297975-131299503-HERVHF-3LTR +
6 131294420 131328725 NONHSAT251502.1 + 466
6 131300740 131301206 Homo_sapiens_6_131297975-131299503-HERVHF-3LTR +
6 131294420 131344687 NONHSAT251503.1 + 466
6 131338799 131344566 Homo_sapiens_6_131340338-131342739-HERVHF + 6
131294420 131348082 NONHSAG093612.2 + 5767
6 131338799 131339252 Homo_sapiens_6_131340338-131342739-HERVHF-5LTR +
6 131294420 131348082 NONHSAG093612.2 + 453
6 131338799 131344566 Homo_sapiens_6_131340338-131342739-HERVHF + 6
131294420 131344687 NONHSAT251503.1 + 5767
6 131338799 131339252 Homo_sapiens_6_131340338-131342739-HERVHF-5LTR +
6 131294420 131344687 NONHSAT251503.1 + 453
6 131340935 131341324 Homo_sapiens_6_131340338-131342739-HERVHF-gag + 6
131294420 131348082 NONHSAG093612.2 + 389
6 131340935 131341324 Homo_sapiens_6_131340338-131342739-HERVHF-gag + 6

131294420 131344687 NONHSAT251503.1 + 389
6 131341378 131342177 Homo_sapiens_6_131340338-131342739-HERVHF-pro + 6
131294420 131348082 NONHSAG093612.2 + 799
6 131341378 131342177 Homo_sapiens_6_131340338-131342739-HERVHF-pro + 6
131294420 131344687 NONHSAT251503.1 + 799
6 131341770 131343672 Homo_sapiens_6_131340338-131342739-HERVHF-pol + 6
131294420 131348082 NONHSAG093612.2 + 1902
6 131341770 131343672 Homo_sapiens_6_131340338-131342739-HERVHF-pol + 6
131294420 131344687 NONHSAT251503.1 + 1902
6 131344112 131344566 Homo_sapiens_6_131340338-131342739-HERVHF-3LTR +
6 131294420 131348082 NONHSAG093612.2 + 454
6 131344112 131344566 Homo_sapiens_6_131340338-131342739-HERVHF-3LTR +
6 131294420 131344687 NONHSAT251503.1 + 454
6 131298228 131299742 Homo_sapiens_6_131297975-131299503-HERVHF-pol + 6
131298073 131309274 NONHSAT114915.2+ 1514
6 131300740 131301206 Homo_sapiens_6_131297975-131299503-HERVHF-3LTR +
6 131298073 131309274 NONHSAT114915.2+ 466
6 131338799 131344566 Homo_sapiens_6_131340338-131342739-HERVHF + 6
131324550 131348082 NONHSAT208028.1 + 5767
6 131338799 131339252 Homo_sapiens_6_131340338-131342739-HERVHF-5LTR +
6 131324550 131348082 NONHSAT208028.1 + 453
6 131340935 131341324 Homo_sapiens_6_131340338-131342739-HERVHF-gag + 6
131324550 131348082 NONHSAT208028.1 + 389
6 131341378 131342177 Homo_sapiens_6_131340338-131342739-HERVHF-pro + 6
131324550 131348082 NONHSAT208028.1 + 799
6 131341770 131343672 Homo_sapiens_6_131340338-131342739-HERVHF-pol + 6
131324550 131348082 NONHSAT208028.1 + 1902
6 131344112 131344566 Homo_sapiens_6_131340338-131342739-HERVHF-3LTR +
6 131324550 131348082 NONHSAT208028.1 + 454
6 131903830 131904159 Homo_sapiens_6_131904209-131905555-HERVHF-gag + 6
131901962 131920565 NONHSAG044846.2 + 329
6 131903830 131904159 Homo_sapiens_6_131904209-131905555-HERVHF-gag + 6
131901962 131920565 NONHSAT114930.2+ 329
6 131904166 131904993 Homo_sapiens_6_131904209-131905555-HERVHF-pro + 6
131901962 131920565 NONHSAG044846.2 + 827
6 131904166 131904993 Homo_sapiens_6_131904209-131905555-HERVHF-pro + 6
131901962 131920565 NONHSAT114930.2+ 827
6 131904451 131906364 Homo_sapiens_6_131904209-131905555-HERVHF-pol + 6
131901962 131920565 NONHSAG044846.2 + 1913
6 131904451 131906364 Homo_sapiens_6_131904209-131905555-HERVHF-pol + 6
131901962 131920565 NONHSAT114930.2+ 1913
6 131906969 131907420 Homo_sapiens_6_131904209-131905555-HERVHF-3LTR +
6 131901962 131920565 NONHSAG044846.2 + 451
6 131906969 131907420 Homo_sapiens_6_131904209-131905555-HERVHF-3LTR +

6	131901962	131920565	NONHSAT114930.2+	451
6	131903830	131904159	Homo_sapiens_6_131904209-131905555-HERVHF-gag +	6
131901963	131920565	ENSG00000236673 +	329
6	131903830	131904159	Homo_sapiens_6_131904209-131905555-HERVHF-gag +	6
131901963	131907530	NONHSAT114931.2+	329
6	131904166	131904993	Homo_sapiens_6_131904209-131905555-HERVHF-pro +	6
131901963	131920565	ENSG00000236673 +	827
6	131904166	131904993	Homo_sapiens_6_131904209-131905555-HERVHF-pro +	6
131901963	131907530	NONHSAT114931.2+	827
6	131904451	131906364	Homo_sapiens_6_131904209-131905555-HERVHF-pol +	6
131901963	131920565	ENSG00000236673 +	1913
6	131904451	131906364	Homo_sapiens_6_131904209-131905555-HERVHF-pol +	6
131901963	131907530	NONHSAT114931.2+	1913
6	131906969	131907420	Homo_sapiens_6_131904209-131905555-HERVHF-3LTR	+
6	131901963	131920565	ENSG00000236673 +	451
6	131906969	131907420	Homo_sapiens_6_131904209-131905555-HERVHF-3LTR	+
6	131901963	131907530	NONHSAT114931.2+	451
6	131903830	131904159	Homo_sapiens_6_131904209-131905555-HERVHF-gag +	6
131901964	131907530	ENSG00000236673 +	329
6	131904166	131904993	Homo_sapiens_6_131904209-131905555-HERVHF-pro +	6
131901964	131907530	ENSG00000236673 +	827
6	131904451	131906364	Homo_sapiens_6_131904209-131905555-HERVHF-pol +	6
131901964	131907530	ENSG00000236673 +	1913
6	131906969	131907420	Homo_sapiens_6_131904209-131905555-HERVHF-3LTR	+
6	131901964	131907530	ENSG00000236673 +	451
6	131903830	131904159	Homo_sapiens_6_131904209-131905555-HERVHF-gag +	6
131903647	131907530	NONHSAT114932.2+	329
6	131904166	131904993	Homo_sapiens_6_131904209-131905555-HERVHF-pro +	6
131903647	131907530	NONHSAT114932.2+	827
6	131904451	131906364	Homo_sapiens_6_131904209-131905555-HERVHF-pol +	6
131903647	131907530	NONHSAT114932.2+	1913
6	131906969	131907420	Homo_sapiens_6_131904209-131905555-HERVHF-3LTR	+
6	131903647	131907530	NONHSAT114932.2+	451
6	131903830	131904159	Homo_sapiens_6_131904209-131905555-HERVHF-gag +	6
131903648	131907530	ENSG00000236673 +	329
6	131904166	131904993	Homo_sapiens_6_131904209-131905555-HERVHF-pro +	6
131903648	131907530	ENSG00000236673 +	827
6	131904451	131906364	Homo_sapiens_6_131904209-131905555-HERVHF-pol +	6
131903648	131907530	ENSG00000236673 +	1913
6	131906969	131907420	Homo_sapiens_6_131904209-131905555-HERVHF-3LTR	+
6	131903648	131907530	ENSG00000236673 +	451
6	144923164	144928866	Homo_sapiens_6_144925698-144927040-HERVHF	+	6
144922305	144930077	NONHSAG095837.2	+	5702
6	144923164	144923609	Homo_sapiens_6_144925698-144927040-HERVHF-5LTR	+

6 144922305 144930077 NONHSAG095837.2 + 445
6 144925945 144927834 Homo_sapiens_6_144925698-144927040-HERVHF-pol + 6 144922305 144930077 NONHSAG095837.2 + 1889
6 144928414 144928866 Homo_sapiens_6_144925698-144927040-HERVHF-3LTR + 6 144922305 144930077 NONHSAG095837.2 + 452
6 144923164 144923609 Homo_sapiens_6_144925698-144927040-HERVHF-5LTR + 6 144922314 144926487 NONHSAT251624.1 + 445
6 144923164 144923609 Homo_sapiens_6_144925698-144927040-HERVHF-5LTR + 6 144922314 144928748 NONHSAT251625.1 + 445
6 144923164 144928866 Homo_sapiens_6_144925698-144927040-HERVHF + 6 144922314 144929082 NONHSAT251626.1 + 5702
6 144923164 144923609 Homo_sapiens_6_144925698-144927040-HERVHF-5LTR + 6 144922314 144929082 NONHSAT251626.1 + 445
6 144923164 144928866 Homo_sapiens_6_144925698-144927040-HERVHF + 6 144922314 144929082 NONHSAT251627.1 + 5702
6 144923164 144923609 Homo_sapiens_6_144925698-144927040-HERVHF-5LTR + 6 144922314 144929082 NONHSAT251627.1 + 445
6 144923164 144928866 Homo_sapiens_6_144925698-144927040-HERVHF + 6 144922314 144929880 NONHSAT251628.1 + 5702
6 144923164 144923609 Homo_sapiens_6_144925698-144927040-HERVHF-5LTR + 6 144922314 144929880 NONHSAT251628.1 + 445
6 144925945 144927834 Homo_sapiens_6_144925698-144927040-HERVHF-pol + 6 144922314 144928748 NONHSAT251625.1 + 1889
6 144925945 144927834 Homo_sapiens_6_144925698-144927040-HERVHF-pol + 6 144922314 144929082 NONHSAT251626.1 + 1889
6 144925945 144927834 Homo_sapiens_6_144925698-144927040-HERVHF-pol + 6 144922314 144929082 NONHSAT251627.1 + 1889
6 144925945 144927834 Homo_sapiens_6_144925698-144927040-HERVHF-pol + 6 144922314 144929880 NONHSAT251628.1 + 1889
6 144928414 144928866 Homo_sapiens_6_144925698-144927040-HERVHF-3LTR + 6 144922314 144929082 NONHSAT251626.1 + 452
6 144928414 144928866 Homo_sapiens_6_144925698-144927040-HERVHF-3LTR + 6 144922314 144929082 NONHSAT251627.1 + 452
6 144928414 144928866 Homo_sapiens_6_144925698-144927040-HERVHF-3LTR + 6 144922314 144929880 NONHSAT251628.1 + 452
6 144925945 144927834 Homo_sapiens_6_144925698-144927040-HERVHF-pol + 6 144923387 144930077 NONHSAT208190.1 + 1889
6 144928414 144928866 Homo_sapiens_6_144925698-144927040-HERVHF-3LTR + 6 144923387 144930077 NONHSAT208190.1 + 452
6 144923164 144928866 Homo_sapiens_6_144925698-144927040-HERVHF + 6 144923586 144928835 NONHSAT115388.2+ 5249
6 144925945 144927834 Homo_sapiens_6_144925698-144927040-HERVHF-pol + 6 144923586 144928835 NONHSAT115388.2+ 1889
7 26024199 26029809 Homo_sapiens_7_26026061-26027405-HERVHF - 7 26018880

26130859 NONHSAG047156.2 - 5610
7 26024199 26024639 Homo_sapiens_7_26026061-26027405-HERVHF-5LTR - 7
26018880 26130859 NONHSAG047156.2 - 440
7 26025479 26027163 Homo_sapiens_7_26026061-26027405-HERVHF-pol - 7
26018880 26130859 NONHSAG047156.2 - 1684
7 26026567 26027451 Homo_sapiens_7_26026061-26027405-HERVHF-pro - 7
26018880 26130859 NONHSAG047156.2 - 884
7 26029364 26029809 Homo_sapiens_7_26026061-26027405-HERVHF-3LTR - 7
26018880 26130859 NONHSAG047156.2 - 445
7 26024199 26029809 Homo_sapiens_7_26026061-26027405-HERVHF - 7 26019503
26130859 NONHSAT119611.2- 5610
7 26024199 26024639 Homo_sapiens_7_26026061-26027405-HERVHF-5LTR - 7
26019503 26130859 NONHSAT119611.2- 440
7 26024199 26029809 Homo_sapiens_7_26026061-26027405-HERVHF - 7 26019503
26130859 NONHSAT119612.2- 5610
7 26024199 26024639 Homo_sapiens_7_26026061-26027405-HERVHF-5LTR - 7
26019503 26130859 NONHSAT119612.2- 440
7 26025479 26027163 Homo_sapiens_7_26026061-26027405-HERVHF-pol - 7
26019503 26130859 NONHSAT119611.2- 1684
7 26025479 26027163 Homo_sapiens_7_26026061-26027405-HERVHF-pol - 7
26019503 26130859 NONHSAT119612.2- 1684
7 26026567 26027451 Homo_sapiens_7_26026061-26027405-HERVHF-pro - 7
26019503 26130859 NONHSAT119611.2- 884
7 26026567 26027451 Homo_sapiens_7_26026061-26027405-HERVHF-pro - 7
26019503 26130859 NONHSAT119612.2- 884
7 26029364 26029809 Homo_sapiens_7_26026061-26027405-HERVHF-3LTR - 7
26019503 26130859 NONHSAT119611.2- 445
7 26029364 26029809 Homo_sapiens_7_26026061-26027405-HERVHF-3LTR - 7
26019503 26130859 NONHSAT119612.2- 445
7 26025479 26027163 Homo_sapiens_7_26026061-26027405-HERVHF-pol - 7
26024570 26097303 NONHSAG097003.1 - 1684
7 26025479 26027163 Homo_sapiens_7_26026061-26027405-HERVHF-pol - 7
26024570 26097303 NONHSAT213299.1 - 1684
7 26026567 26027451 Homo_sapiens_7_26026061-26027405-HERVHF-pro - 7
26024570 26097303 NONHSAG097003.1 - 884
7 26026567 26027451 Homo_sapiens_7_26026061-26027405-HERVHF-pro - 7
26024570 26097303 NONHSAT213299.1 - 884
7 26029364 26029809 Homo_sapiens_7_26026061-26027405-HERVHF-3LTR - 7
26024570 26097303 NONHSAG097003.1 - 445
7 26029364 26029809 Homo_sapiens_7_26026061-26027405-HERVHF-3LTR - 7
26024570 26097303 NONHSAT213299.1 - 445
7 34300132 34300573 Homo_sapiens_7_34301985-34303122-HERVHF-5LTR - 7
34298855 34301559 NONHSAG047318.2 - 441
7 34300132 34300573 Homo_sapiens_7_34301985-34303122-HERVHF-5LTR - 7

34298855 34301559 NONHSAT120040.2 - 441
7 102975230 102975898 Homo_sapiens_7_102976263-102977254-HERVHF-gag + 7
102973509 102988857 NONHSAG048439.2 + 668
7 102975230 102975898 Homo_sapiens_7_102976263-102977254-HERVHF-gag + 7
102973509 102988844 NONHSAT212473.1 + 668
7 102976371 102977021 Homo_sapiens_7_102976263-102977254-HERVHF-pro + 7
102973509 102988857 NONHSAG048439.2 + 650
7 102976371 102977021 Homo_sapiens_7_102976263-102977254-HERVHF-pro + 7
102973509 102988844 NONHSAT212473.1 + 650
7 102976449 102977042 Homo_sapiens_7_102976263-102977254-HERVHF-pol + 7
102973509 102988857 NONHSAG048439.2 + 593
7 102976449 102977042 Homo_sapiens_7_102976263-102977254-HERVHF-pol + 7
102973509 102988844 NONHSAT212473.1 + 593
7 102977224 102977607 Homo_sapiens_7_102976263-102977254-HERVHF-env + 7
102973509 102988857 NONHSAG048439.2 + 383
7 102977224 102977607 Homo_sapiens_7_102976263-102977254-HERVHF-env + 7
102973509 102988844 NONHSAT212473.1 + 383
7 102978322 102978736 Homo_sapiens_7_102976263-102977254-HERVHF-3LTR +
7 102973509 102988857 NONHSAG048439.2 + 414
7 102978322 102978736 Homo_sapiens_7_102976263-102977254-HERVHF-3LTR +
7 102973509 102988844 NONHSAT212473.1 + 414
7 102975230 102975898 Homo_sapiens_7_102976263-102977254-HERVHF-gag + 7
102973522 102988856 ENSG00000230257 + 668
7 102975230 102975898 Homo_sapiens_7_102976263-102977254-HERVHF-gag + 7
102973522 102988857 NONHSAT212474.1 + 668
7 102976371 102977021 Homo_sapiens_7_102976263-102977254-HERVHF-pro + 7
102973522 102988856 ENSG00000230257 + 650
7 102976371 102977021 Homo_sapiens_7_102976263-102977254-HERVHF-pro + 7
102973522 102988857 NONHSAT212474.1 + 650
7 102976449 102977042 Homo_sapiens_7_102976263-102977254-HERVHF-pol + 7
102973522 102988856 ENSG00000230257 + 593
7 102976449 102977042 Homo_sapiens_7_102976263-102977254-HERVHF-pol + 7
102973522 102988857 NONHSAT212474.1 + 593
7 102977224 102977607 Homo_sapiens_7_102976263-102977254-HERVHF-env + 7
102973522 102988856 ENSG00000230257 + 383
7 102977224 102977607 Homo_sapiens_7_102976263-102977254-HERVHF-env + 7
102973522 102988857 NONHSAT212474.1 + 383
7 102978322 102978736 Homo_sapiens_7_102976263-102977254-HERVHF-3LTR +
7 102973522 102988856 ENSG00000230257 + 414
7 102978322 102978736 Homo_sapiens_7_102976263-102977254-HERVHF-3LTR +
7 102973522 102988857 NONHSAT212474.1 + 414
7 125920130 125920885 Homo_sapiens_7_125920071-125921895-HERVHF-pro + 7
125917870 125933832 NONHSAG048705.2 + 755
7 125920130 125920885 Homo_sapiens_7_125920071-125921895-HERVHF-pro + 7

125917870 125933832 NONHSAT123111.2+ 755
7 125920475 125922154 Homo_sapiens_7_125920071-125921895-HERVHF-pol + 7
125917870 125933832 NONHSAG048705.2 + 1679
7 125920475 125922154 Homo_sapiens_7_125920071-125921895-HERVHF-pol + 7
125917870 125933832 NONHSAT123111.2+ 1679
7 125923701 125924112 Homo_sapiens_7_125920071-125921895-HERVHF-3LTR +
7 125917870 125933832 NONHSAG048705.2 + 411
7 125923701 125924112 Homo_sapiens_7_125920071-125921895-HERVHF-3LTR +
7 125917870 125933832 NONHSAT123111.2+ 411
7 125920130 125920885 Homo_sapiens_7_125920071-125921895-HERVHF-pro + 7
125917871 125933832 ENSG00000197462 + 755
7 125920475 125922154 Homo_sapiens_7_125920071-125921895-HERVHF-pol + 7
125917871 125933832 ENSG00000197462 + 1679
7 125923701 125924112 Homo_sapiens_7_125920071-125921895-HERVHF-3LTR +
7 125917871 125933832 ENSG00000197462 + 411
7 155238821 155239129 Homo_sapiens_7_155240740-155241657-HERVK-env - 7
155238273 155254077 NONHSAG049243.2 - 308
7 155238821 155239129 Homo_sapiens_7_155240740-155241657-HERVK-env - 7
155238273 155251512 NONHSAT124357.2 - 308
7 155239444 155241440 Homo_sapiens_7_155240740-155241657-HERVK-pol - 7
155238273 155254077 NONHSAG049243.2 - 1996
7 155239444 155241440 Homo_sapiens_7_155240740-155241657-HERVK-pol - 7
155238273 155251512 NONHSAT124357.2 - 1996
7 155241335 155242276 Homo_sapiens_7_155240740-155241657-HERVK-pro - 7
155238273 155254077 NONHSAG049243.2 - 941
7 155241335 155242276 Homo_sapiens_7_155240740-155241657-HERVK-pro - 7
155238273 155251512 NONHSAT124357.2 - 941
7 155243682 155244070 Homo_sapiens_7_155240740-155241657-HERVK-3LTR -
7 155238273 155254077 NONHSAG049243.2 - 388
7 155243682 155244070 Homo_sapiens_7_155240740-155241657-HERVK-3LTR -
7 155238273 155251512 NONHSAT124357.2 - 388
8 71676972 71677343 Homo_sapiens_8_71677433-71678968-HERVHF-gag + 8
71675209 71706261 NONHSAG050489.2 + 371
8 71676972 71677343 Homo_sapiens_8_71677433-71678968-HERVHF-gag + 8
71675209 71706261 NONHSAT127188.2 + 371
8 71677387 71678241 Homo_sapiens_8_71677433-71678968-HERVHF-pro + 8
71675209 71706261 NONHSAG050489.2 + 854
8 71677387 71678241 Homo_sapiens_8_71677433-71678968-HERVHF-pro + 8
71675209 71706261 NONHSAT127188.2 + 854
8 71677678 71679621 Homo_sapiens_8_71677433-71678968-HERVHF-pol + 8
71675209 71706261 NONHSAG050489.2 + 1943
8 71677678 71679621 Homo_sapiens_8_71677433-71678968-HERVHF-pol + 8
71675209 71706261 NONHSAT127188.2 + 1943
8 71680214 71680514 Homo_sapiens_8_71677433-71678968-HERVHF-3LTR + 8

71675209 71706261 NONHSAG050489.2 + 300
8 71680214 71680514 Homo_sapiens_8_71677433-71678968-HERVHF-3LTR + 8
71675209 71706261 NONHSAT127188.2 + 300
8 71676972 71677343 Homo_sapiens_8_71677433-71678968-HERVHF-gag + 8
71675300 71702786 ENSG00000254277 + 371
8 71677387 71678241 Homo_sapiens_8_71677433-71678968-HERVHF-pro + 8
71675300 71702786 ENSG00000254277 + 854
8 71677678 71679621 Homo_sapiens_8_71677433-71678968-HERVHF-pol + 8
71675300 71702786 ENSG00000254277 + 1943
8 71680214 71680514 Homo_sapiens_8_71677433-71678968-HERVHF-3LTR + 8
71675300 71702786 ENSG00000254277 + 300
8 71676972 71677343 Homo_sapiens_8_71677433-71678968-HERVHF-gag + 8
71675328 71704577 NONHSAT127189.2 + 371
8 71677387 71678241 Homo_sapiens_8_71677433-71678968-HERVHF-pro + 8
71675328 71704577 NONHSAT127189.2 + 854
8 71677678 71679621 Homo_sapiens_8_71677433-71678968-HERVHF-pol + 8
71675328 71704577 NONHSAT127189.2 + 1943
8 71680214 71680514 Homo_sapiens_8_71677433-71678968-HERVHF-3LTR + 8
71675328 71704577 NONHSAT127189.2 + 300
8 71676972 71677343 Homo_sapiens_8_71677433-71678968-HERVHF-gag + 8
71675376 71702435 NONHSAT127190.2 + 371
8 71677387 71678241 Homo_sapiens_8_71677433-71678968-HERVHF-pro + 8
71675376 71702435 NONHSAT127190.2 + 854
8 71677678 71679621 Homo_sapiens_8_71677433-71678968-HERVHF-pol + 8
71675376 71702435 NONHSAT127190.2 + 1943
8 71680214 71680514 Homo_sapiens_8_71677433-71678968-HERVHF-3LTR + 8
71675376 71702435 NONHSAT127190.2 + 300
8 71676972 71677343 Homo_sapiens_8_71677433-71678968-HERVHF-gag + 8
71675377 71702435 ENSG00000254277 + 371
8 71677387 71678241 Homo_sapiens_8_71677433-71678968-HERVHF-pro + 8
71675377 71702435 ENSG00000254277 + 854
8 71677678 71679621 Homo_sapiens_8_71677433-71678968-HERVHF-pol + 8
71675377 71702435 ENSG00000254277 + 1943
8 71680214 71680514 Homo_sapiens_8_71677433-71678968-HERVHF-3LTR + 8
71675377 71702435 ENSG00000254277 + 300
8 90090224 90090661 Homo_sapiens_8_90091914-90093348-HERVHF-5LTR - 8
90063299 90095475 ENSG00000104327 - 437
8 90091063 90093283 Homo_sapiens_8_90091914-90093348-HERVHF-pol - 8
90063299 90095475 ENSG00000104327 - 2220
8 90093495 90093794 Homo_sapiens_8_90091914-90093348-HERVHF-gag - 8
90063299 90095475 ENSG00000104327 - 299
8 90090224 90090661 Homo_sapiens_8_90091914-90093348-HERVHF-5LTR - 8
90063435 90095351 ENSG00000104327 - 437
8 90091063 90093283 Homo_sapiens_8_90091914-90093348-HERVHF-pol - 8

90063435 90095351 ENSG00000104327 - 2220
8 90093495 90093794 Homo_sapiens_8_90091914-90093348-HERVHF-gag - 8
90063435 90095351 ENSG00000104327 - 299
8 90090224 90090661 Homo_sapiens_8_90091914-90093348-HERVHF-5LTR - 8
90078369 90095459 NONHSAG050711.2 - 437
8 90090224 90090661 Homo_sapiens_8_90091914-90093348-HERVHF-5LTR - 8
90078369 90095459 NONHSAT127642.2 - 437
8 90091063 90093283 Homo_sapiens_8_90091914-90093348-HERVHF-pol - 8
90078369 90095459 NONHSAG050711.2 - 2220
8 90091063 90093283 Homo_sapiens_8_90091914-90093348-HERVHF-pol - 8
90078369 90095459 NONHSAT127642.2 - 2220
8 90093495 90093794 Homo_sapiens_8_90091914-90093348-HERVHF-gag - 8
90078369 90095459 NONHSAG050711.2 - 299
8 90093495 90093794 Homo_sapiens_8_90091914-90093348-HERVHF-gag - 8
90078369 90095459 NONHSAT127642.2 - 299
8 90090224 90090661 Homo_sapiens_8_90091914-90093348-HERVHF-5LTR - 8
90078424 90095470 ENSG00000104327 - 437
8 90091063 90093283 Homo_sapiens_8_90091914-90093348-HERVHF-pol - 8
90078424 90095470 ENSG00000104327 - 2220
8 90093495 90093794 Homo_sapiens_8_90091914-90093348-HERVHF-gag - 8
90078424 90095470 ENSG00000104327 - 299
8 97200769 97206658 Homo_sapiens_8_97202388-97204973-HERVHF + 8 97192408
97208977 NONHSAG098987.1 + 5889
8 97200769 97201222 Homo_sapiens_8_97202388-97204973-HERVHF-5LTR + 8
97192408 97208977 NONHSAG098987.1 + 453
8 97200769 97206658 Homo_sapiens_8_97202388-97204973-HERVHF + 8 97192408
97208977 NONHSAT216128.1 + 5889
8 97200769 97201222 Homo_sapiens_8_97202388-97204973-HERVHF-5LTR + 8
97192408 97208977 NONHSAT216128.1 + 453
8 97202976 97203299 Homo_sapiens_8_97202388-97204973-HERVHF-gag + 8
97192408 97208977 NONHSAG098987.1 + 323
8 97202976 97203299 Homo_sapiens_8_97202388-97204973-HERVHF-gag + 8
97192408 97208977 NONHSAT216128.1 + 323
8 97203392 97204225 Homo_sapiens_8_97202388-97204973-HERVHF-pro + 8
97192408 97208977 NONHSAG098987.1 + 833
8 97203392 97204225 Homo_sapiens_8_97202388-97204973-HERVHF-pro + 8
97192408 97208977 NONHSAT216128.1 + 833
8 97203683 97205588 Homo_sapiens_8_97202388-97204973-HERVHF-pol + 8
97192408 97208977 NONHSAG098987.1 + 1905
8 97203683 97205588 Homo_sapiens_8_97202388-97204973-HERVHF-pol + 8
97192408 97208977 NONHSAT216128.1 + 1905
8 97206202 97206658 Homo_sapiens_8_97202388-97204973-HERVHF-3LTR + 8
97192408 97208977 NONHSAG098987.1 + 456
8 97206202 97206658 Homo_sapiens_8_97202388-97204973-HERVHF-3LTR + 8

97192408 97208977 NONHSAT216128.1 + 456
8 114284546 114285295 Homo_sapiens_8_114284508-114286044-HERVHF-pro + 8
114281914 114311268 NONHSAG051074.2 + 749
8 114284546 114285295 Homo_sapiens_8_114284508-114286044-HERVHF-pro + 8
114281914 114305621 NONHSAT128350.2 + 749
8 114284753 114286697 Homo_sapiens_8_114284508-114286044-HERVHF-pol + 8
114281914 114311268 NONHSAG051074.2 + 1944
8 114284753 114286697 Homo_sapiens_8_114284508-114286044-HERVHF-pol + 8
114281914 114305621 NONHSAT128350.2 + 1944
8 114287268 114287727 Homo_sapiens_8_114284508-114286044-HERVHF-3LTR +
8 114281914 114311268 NONHSAG051074.2 + 459
8 114287268 114287727 Homo_sapiens_8_114284508-114286044-HERVHF-3LTR +
8 114281914 114305621 NONHSAT128350.2 + 459
8 114284546 114285295 Homo_sapiens_8_114284508-114286044-HERVHF-pro + 8
114282066 114295807 NONHSAT128351.2 + 749
8 114284753 114286697 Homo_sapiens_8_114284508-114286044-HERVHF-pol + 8
114282066 114295807 NONHSAT128351.2 + 1944
8 114287268 114287727 Homo_sapiens_8_114284508-114286044-HERVHF-3LTR +
8 114282066 114295807 NONHSAT128351.2 + 459
8 114284546 114285295 Homo_sapiens_8_114284508-114286044-HERVHF-pro + 8
114282067 114295807 ENSG00000254339 + 749
8 114284546 114285295 Homo_sapiens_8_114284508-114286044-HERVHF-pro + 8
114282067 114295840 NONHSAT216269.1 + 749
8 114284753 114286697 Homo_sapiens_8_114284508-114286044-HERVHF-pol + 8
114282067 114295807 ENSG00000254339 + 1944
8 114284753 114286697 Homo_sapiens_8_114284508-114286044-HERVHF-pol + 8
114282067 114295840 NONHSAT216269.1 + 1944
8 114287268 114287727 Homo_sapiens_8_114284508-114286044-HERVHF-3LTR +
8 114282067 114295807 ENSG00000254339 + 459
8 114287268 114287727 Homo_sapiens_8_114284508-114286044-HERVHF-3LTR +
8 114282067 114295840 NONHSAT216269.1 + 459
8 114284546 114285295 Homo_sapiens_8_114284508-114286044-HERVHF-pro + 8
114282068 114295813 NONHSAT128352.2 + 749
8 114284753 114286697 Homo_sapiens_8_114284508-114286044-HERVHF-pol + 8
114282068 114295813 NONHSAT128352.2 + 1944
8 114287268 114287727 Homo_sapiens_8_114284508-114286044-HERVHF-3LTR +
8 114282068 114295813 NONHSAT128352.2 + 459
8 114284546 114285295 Homo_sapiens_8_114284508-114286044-HERVHF-pro + 8
114282069 114295813 ENSG00000254339 + 749
8 114284753 114286697 Homo_sapiens_8_114284508-114286044-HERVHF-pol + 8
114282069 114295813 ENSG00000254339 + 1944
8 114287268 114287727 Homo_sapiens_8_114284508-114286044-HERVHF-3LTR +
8 114282069 114295813 ENSG00000254339 + 459
8 114284546 114285295 Homo_sapiens_8_114284508-114286044-HERVHF-pro + 8

114282071 114295624 NONHSAT128353.2 + 749
8 114284753 114286697 Homo_sapiens_8_114284508-114286044-HERVHF-pol + 8
114282071 114295624 NONHSAT128353.2 + 1944
8 114287268 114287727 Homo_sapiens_8_114284508-114286044-HERVHF-3LTR +
8 114282071 114295624 NONHSAT128353.2 + 459
8 114284546 114285295 Homo_sapiens_8_114284508-114286044-HERVHF-pro + 8
114282072 114295624 ENSG00000254339 + 749
8 114284546 114285295 Homo_sapiens_8_114284508-114286044-HERVHF-pro + 8
114282072 114295839 NONHSAT128354.2 + 749
8 114284753 114286697 Homo_sapiens_8_114284508-114286044-HERVHF-pol + 8
114282072 114295624 ENSG00000254339 + 1944
8 114284753 114286697 Homo_sapiens_8_114284508-114286044-HERVHF-pol + 8
114282072 114295839 NONHSAT128354.2 + 1944
8 114287268 114287727 Homo_sapiens_8_114284508-114286044-HERVHF-3LTR +
8 114282072 114295624 ENSG00000254339 + 459
8 114287268 114287727 Homo_sapiens_8_114284508-114286044-HERVHF-3LTR +
8 114282072 114295839 NONHSAT128354.2 + 459
8 114284546 114285295 Homo_sapiens_8_114284508-114286044-HERVHF-pro + 8
114282073 114295839 ENSG00000254339 + 749
8 114284753 114286697 Homo_sapiens_8_114284508-114286044-HERVHF-pol + 8
114282073 114295839 ENSG00000254339 + 1944
8 114287268 114287727 Homo_sapiens_8_114284508-114286044-HERVHF-3LTR +
8 114282073 114295839 ENSG00000254339 + 459
8 114284546 114285295 Homo_sapiens_8_114284508-114286044-HERVHF-pro + 8
114282081 114287996 NONHSAT128355.2 + 749
8 114284753 114286697 Homo_sapiens_8_114284508-114286044-HERVHF-pol + 8
114282081 114287996 NONHSAT128355.2 + 1944
8 114287268 114287727 Homo_sapiens_8_114284508-114286044-HERVHF-3LTR +
8 114282081 114287996 NONHSAT128355.2 + 459
8 114284546 114285295 Homo_sapiens_8_114284508-114286044-HERVHF-pro + 8
114282136 114287996 ENSG00000254339 + 749
8 114284753 114286697 Homo_sapiens_8_114284508-114286044-HERVHF-pol + 8
114282136 114287996 ENSG00000254339 + 1944
8 114287268 114287727 Homo_sapiens_8_114284508-114286044-HERVHF-3LTR +
8 114282136 114287996 ENSG00000254339 + 459
8 132080235 132080688 Homo_sapiens_8_132081909-132083445-HERVHF-5LTR -
8 132024238 132085655 ENSG00000258417 - 453
8 132081082 132083067 Homo_sapiens_8_132081909-132083445-HERVHF-pol - 8
132024238 132085655 ENSG00000258417 - 1985
8 132083498 132083827 Homo_sapiens_8_132081909-132083445-HERVHF-gag - 8
132024238 132085655 ENSG00000258417 - 329
8 132080235 132086002 Homo_sapiens_8_132081909-132083445-HERVHF - 8
132061486 132105265 ENSG00000132297 - 5767
8 132080235 132080688 Homo_sapiens_8_132081909-132083445-HERVHF-5LTR -

8 132061486 132105265 ENSG00000132297 - 453
8 132081082 132083067 Homo_sapiens_8_132081909-132083445-HERVHF-pol - 8
132061486 132105265 ENSG00000132297 - 1985
8 132083498 132083827 Homo_sapiens_8_132081909-132083445-HERVHF-gag - 8
132061486 132105265 ENSG00000132297 - 329
8 132085544 132086002 Homo_sapiens_8_132081909-132083445-HERVHF-3LTR -
8 132061486 132105265 ENSG00000132297 - 458
8 132080235 132080688 Homo_sapiens_8_132081909-132083445-HERVHF-5LTR -
8 132063785 132081104 ENSG00000132297 - 453
8 132080235 132086002 Homo_sapiens_8_132081909-132083445-HERVHF - 8
132063785 132111159 ENSG00000132297 - 5767
8 132080235 132080688 Homo_sapiens_8_132081909-132083445-HERVHF-5LTR -
8 132063785 132111159 ENSG00000132297 - 453
8 132081082 132083067 Homo_sapiens_8_132081909-132083445-HERVHF-pol - 8
132063785 132111159 ENSG00000132297 - 1985
8 132083498 132083827 Homo_sapiens_8_132081909-132083445-HERVHF-gag - 8
132063785 132111159 ENSG00000132297 - 329
8 132085544 132086002 Homo_sapiens_8_132081909-132083445-HERVHF-3LTR -
8 132063785 132111159 ENSG00000132297 - 458
8 132080235 132086002 Homo_sapiens_8_132081909-132083445-HERVHF - 8
132069675 132111159 ENSG00000132297 - 5767
8 132080235 132080688 Homo_sapiens_8_132081909-132083445-HERVHF-5LTR -
8 132069675 132111159 ENSG00000132297 - 453
8 132081082 132083067 Homo_sapiens_8_132081909-132083445-HERVHF-pol - 8
132069675 132111159 ENSG00000132297 - 1985
8 132083498 132083827 Homo_sapiens_8_132081909-132083445-HERVHF-gag - 8
132069675 132111159 ENSG00000132297 - 329
8 132085544 132086002 Homo_sapiens_8_132081909-132083445-HERVHF-3LTR -
8 132069675 132111159 ENSG00000132297 - 458
9 12950832 12951653 Homo_sapiens_9_12950845-12952399-HERVHF-pro + 9
12948721 12975294 NONHSAG101172.2 + 821
9 12950832 12951653 Homo_sapiens_9_12950845-12952399-HERVHF-pro + 9
12948721 12968675 NONHSAT256494.1 + 821
9 12950832 12951653 Homo_sapiens_9_12950845-12952399-HERVHF-pro + 9
12948721 12975294 NONHSAT256495.1 + 821
9 12950832 12951653 Homo_sapiens_9_12950845-12952399-HERVHF-pro + 9
12948721 12975294 NONHSAT256496.1 + 821
9 12951135 12953200 Homo_sapiens_9_12950845-12952399-HERVHF-pol + 9
12948721 12975294 NONHSAG101172.2 + 2065
9 12951135 12953200 Homo_sapiens_9_12950845-12952399-HERVHF-pol + 9
12948721 12968675 NONHSAT256494.1 + 2065
9 12951135 12953200 Homo_sapiens_9_12950845-12952399-HERVHF-pol + 9
12948721 12975294 NONHSAT256495.1 + 2065
9 12951135 12953200 Homo_sapiens_9_12950845-12952399-HERVHF-pol + 9

12948721 12975294 NONHSAT256496.1 + 2065
9 12953648 12954130 Homo_sapiens_9_12950845-12952399-HERVHF-3LTR + 9
12948721 12975294 NONHSAG101172.2 + 482
9 12953648 12954130 Homo_sapiens_9_12950845-12952399-HERVHF-3LTR + 9
12948721 12968675 NONHSAT256494.1 + 482
9 12953648 12954130 Homo_sapiens_9_12950845-12952399-HERVHF-3LTR + 9
12948721 12975294 NONHSAT256495.1 + 482
9 12953648 12954130 Homo_sapiens_9_12950845-12952399-HERVHF-3LTR + 9
12948721 12975294 NONHSAT256496.1 + 482
9 80137297 80143055 Homo_sapiens_9_80139873-80141469-HERVHF + 9 79824529
80182097 NONHSAG052646.2 + 5758
9 80137297 80137640 Homo_sapiens_9_80139873-80141469-HERVHF-5LTR + 9
79824529 80182097 NONHSAG052646.2 + 343
9 80139559 80139885 Homo_sapiens_9_80139873-80141469-HERVHF-gag + 9
79824529 80182097 NONHSAG052646.2 + 326
9 80140314 80142260 Homo_sapiens_9_80139873-80141469-HERVHF-pol + 9
79824529 80182097 NONHSAG052646.2 + 1946
9 80142712 80143055 Homo_sapiens_9_80139873-80141469-HERVHF-3LTR + 9
79824529 80182097 NONHSAG052646.2 + 343
9 80137297 80143055 Homo_sapiens_9_80139873-80141469-HERVHF + 9 79988025
80182097 NONHSAT219669.1 + 5758
9 80137297 80137640 Homo_sapiens_9_80139873-80141469-HERVHF-5LTR + 9
79988025 80182097 NONHSAT219669.1 + 343
9 80139559 80139885 Homo_sapiens_9_80139873-80141469-HERVHF-gag + 9
79988025 80182097 NONHSAT219669.1 + 326
9 80140314 80142260 Homo_sapiens_9_80139873-80141469-HERVHF-pol + 9
79988025 80182097 NONHSAT219669.1 + 1946
9 80142712 80143055 Homo_sapiens_9_80139873-80141469-HERVHF-3LTR + 9
79988025 80182097 NONHSAT219669.1 + 343
9 80137297 80143055 Homo_sapiens_9_80139873-80141469-HERVHF + 9 79989859
80182073 NONHSAT132568.2 + 5758
9 80137297 80137640 Homo_sapiens_9_80139873-80141469-HERVHF-5LTR + 9
79989859 80182073 NONHSAT132568.2 + 343
9 80139559 80139885 Homo_sapiens_9_80139873-80141469-HERVHF-gag + 9
79989859 80182073 NONHSAT132568.2 + 326
9 80140314 80142260 Homo_sapiens_9_80139873-80141469-HERVHF-pol + 9
79989859 80182073 NONHSAT132568.2 + 1946
9 80142712 80143055 Homo_sapiens_9_80139873-80141469-HERVHF-3LTR + 9
79989859 80182073 NONHSAT132568.2 + 343
9 80137297 80143055 Homo_sapiens_9_80139873-80141469-HERVHF + 9 80030579
80181759 NONHSAT219670.1 + 5758
9 80137297 80137640 Homo_sapiens_9_80139873-80141469-HERVHF-5LTR + 9
80030579 80181759 NONHSAT219670.1 + 343
9 80139559 80139885 Homo_sapiens_9_80139873-80141469-HERVHF-gag + 9

80030579 80181759 NONHSAT219670.1 + 326
9 80140314 80142260 Homo_sapiens_9_80139873-80141469-HERVHF-pol + 9
80030579 80181759 NONHSAT219670.1 + 1946
9 80142712 80143055 Homo_sapiens_9_80139873-80141469-HERVHF-3LTR + 9
80030579 80181759 NONHSAT219670.1 + 343
9 85458329 85466955 Homo_sapiens_9_85461166-85462902-HERVHF + 9 85461082
85461878 NONHSAT219743.1 + 796
9 85461120 85461926 Homo_sapiens_9_85461166-85462902-HERVHF-pro + 9
85461082 85467386 NONHSAG052703.2 + 806
9 85461120 85461926 Homo_sapiens_9_85461166-85462902-HERVHF-pro + 9
85461082 85467386 NONHSAT132793.2 + 806
9 85461315 85463744 Homo_sapiens_9_85461166-85462902-HERVHF-pol + 9
85461082 85467386 NONHSAG052703.2 + 2429
9 85461315 85463744 Homo_sapiens_9_85461166-85462902-HERVHF-pol + 9
85461082 85467386 NONHSAT132793.2 + 2429
9 85465130 85466268 Homo_sapiens_9_85461166-85462902-HERVHF-env + 9
85461082 85467386 NONHSAG052703.2 + 1138
9 85465130 85466268 Homo_sapiens_9_85461166-85462902-HERVHF-env + 9
85461082 85467386 NONHSAT132793.2 + 1138
9 85466509 85466955 Homo_sapiens_9_85461166-85462902-HERVHF-3LTR + 9
85461082 85467386 NONHSAG052703.2 + 446
9 85466509 85466955 Homo_sapiens_9_85461166-85462902-HERVHF-3LTR + 9
85461082 85467386 NONHSAT132793.2 + 446
9 115475420 115475800 Homo_sapiens_9_115475976-115477349-HERVHF-gag + 9
115473527 115692166 NONHSAG053288.3 + 380
9 115475420 115475800 Homo_sapiens_9_115475976-115477349-HERVHF-gag + 9
115473527 115591079 NONHSAT134412.2 + 380
9 115476023 115476676 Homo_sapiens_9_115475976-115477349-HERVHF-pro + 9
115473527 115692166 NONHSAG053288.3 + 653
9 115476023 115476676 Homo_sapiens_9_115475976-115477349-HERVHF-pro + 9
115473527 115591079 NONHSAT134412.2 + 653
9 115476248 115478150 Homo_sapiens_9_115475976-115477349-HERVHF-pol + 9
115473527 115692166 NONHSAG053288.3 + 1902
9 115476248 115478150 Homo_sapiens_9_115475976-115477349-HERVHF-pol + 9
115473527 115591079 NONHSAT134412.2 + 1902
9 115478470 115478923 Homo_sapiens_9_115475976-115477349-HERVHF-3LTR +
9 115473527 115692166 NONHSAG053288.3 + 453
9 115478470 115478923 Homo_sapiens_9_115475976-115477349-HERVHF-3LTR +
9 115473527 115591079 NONHSAT134412.2 + 453
10 6797081 6802954 Homo_sapiens_10_6798770-6800364-HERVHF - 10 6738434
6842906 NONHSAG005151.3 - 5873
10 6797081 6797535 Homo_sapiens_10_6798770-6800364-HERVHF-5LTR - 10 6738434
6842906 NONHSAG005151.3 - 454
10 6797081 6802954 Homo_sapiens_10_6798770-6800364-HERVHF - 10 6738434

6842906 NONHSAT011256.2- 5873
10 6797081 6797535 Homo_sapiens_10_6798770-6800364-HERVHF-5LTR - 10 6738434
6842906 NONHSAT011256.2- 454
10 6798366 6799928 Homo_sapiens_10_6798770-6800364-HERVHF-pol - 10 6738434
6842906 NONHSAG005151.3 - 1562
10 6798366 6799928 Homo_sapiens_10_6798770-6800364-HERVHF-pol - 10 6738434
6842906 NONHSAT011256.2- 1562
10 6800444 6800767 Homo_sapiens_10_6798770-6800364-HERVHF-gag - 10 6738434
6842906 NONHSAG005151.3 - 323
10 6800444 6800767 Homo_sapiens_10_6798770-6800364-HERVHF-gag - 10 6738434
6842906 NONHSAT011256.2- 323
10 6802501 6802954 Homo_sapiens_10_6798770-6800364-HERVHF-3LTR - 10 6738434
6842906 NONHSAG005151.3 - 453
10 6802501 6802954 Homo_sapiens_10_6798770-6800364-HERVHF-3LTR - 10 6738434
6842906 NONHSAT011256.2- 453
10 6797081 6797535 Homo_sapiens_10_6798770-6800364-HERVHF-5LTR - 10 6796177
6802848 NONHSAG005154.3 - 454
10 6797081 6797535 Homo_sapiens_10_6798770-6800364-HERVHF-5LTR - 10 6796177
6802848 NONHSAT011265.2- 454
10 6798366 6799928 Homo_sapiens_10_6798770-6800364-HERVHF-pol - 10 6796177
6802848 NONHSAG005154.3 - 1562
10 6798366 6799928 Homo_sapiens_10_6798770-6800364-HERVHF-pol - 10 6796177
6802848 NONHSAT011265.2- 1562
10 6800444 6800767 Homo_sapiens_10_6798770-6800364-HERVHF-gag - 10 6796177
6802848 NONHSAG005154.3 - 323
10 6800444 6800767 Homo_sapiens_10_6798770-6800364-HERVHF-gag - 10 6796177
6802848 NONHSAT011265.2- 323
10 25716420 25722928 Homo_sapiens_10_25718978-25720776-HERVHF + 10 25651711
25732935 NONHSAG005427.3 + 6508
10 25716420 25716834 Homo_sapiens_10_25718978-25720776-HERVHF-5LTR + 10
25651711 25732935 NONHSAG005427.3 + 414
10 25716420 25722928 Homo_sapiens_10_25718978-25720776-HERVHF + 10 25651711
25732935 NONHSAT011808.2+ 6508
10 25716420 25716834 Homo_sapiens_10_25718978-25720776-HERVHF-5LTR + 10
25651711 25732935 NONHSAT011808.2+ 414
10 25716420 25722928 Homo_sapiens_10_25718978-25720776-HERVHF + 10 25651711
25732935 NONHSAT155232.1 + 6508
10 25716420 25716834 Homo_sapiens_10_25718978-25720776-HERVHF-5LTR + 10
25651711 25732935 NONHSAT155232.1 + 414
10 25718477 25718863 Homo_sapiens_10_25718978-25720776-HERVHF-gag + 10
25651711 25732935 NONHSAG005427.3 + 386
10 25718477 25718863 Homo_sapiens_10_25718978-25720776-HERVHF-gag + 10
25651711 25732935 NONHSAT011808.2+ 386
10 25718477 25718863 Homo_sapiens_10_25718978-25720776-HERVHF-gag + 10

25651711 25732935 NONHSAT155232.1 + 386
10 25718959 25719759 Homo_sapiens_10_25718978-25720776-HERVHF-pro + 10
25651711 25732935 NONHSAG005427.3 + 800
10 25718959 25719759 Homo_sapiens_10_25718978-25720776-HERVHF-pro + 10
25651711 25732935 NONHSAT011808.2+ 800
10 25718959 25719759 Homo_sapiens_10_25718978-25720776-HERVHF-pro + 10
25651711 25732935 NONHSAT155232.1 + 800
10 25719121 25721515 Homo_sapiens_10_25718978-25720776-HERVHF-pol + 10
25651711 25732935 NONHSAG005427.3 + 2394
10 25719121 25721515 Homo_sapiens_10_25718978-25720776-HERVHF-pol + 10
25651711 25732935 NONHSAT011808.2+ 2394
10 25719121 25721515 Homo_sapiens_10_25718978-25720776-HERVHF-pol + 10
25651711 25732935 NONHSAT155232.1 + 2394
10 25722171 25722503 Homo_sapiens_10_25718978-25720776-HERVHF-env + 10
25651711 25732935 NONHSAG005427.3 + 332
10 25722171 25722503 Homo_sapiens_10_25718978-25720776-HERVHF-env + 10
25651711 25732935 NONHSAT011808.2+ 332
10 25722171 25722503 Homo_sapiens_10_25718978-25720776-HERVHF-env + 10
25651711 25732935 NONHSAT155232.1 + 332
10 25722512 25722928 Homo_sapiens_10_25718978-25720776-HERVHF-3LTR + 10
25651711 25732935 NONHSAG005427.3 + 416
10 25722512 25722928 Homo_sapiens_10_25718978-25720776-HERVHF-3LTR + 10
25651711 25732935 NONHSAT011808.2+ 416
10 25722512 25722928 Homo_sapiens_10_25718978-25720776-HERVHF-3LTR + 10
25651711 25732935 NONHSAT155232.1 + 416
10 25716420 25722928 Homo_sapiens_10_25718978-25720776-HERVHF + 10 25651712
25732935 ENSG00000280809 + 6508
10 25716420 25716834 Homo_sapiens_10_25718978-25720776-HERVHF-5LTR + 10
25651712 25732935 ENSG00000280809 + 414
10 25718477 25718863 Homo_sapiens_10_25718978-25720776-HERVHF-gag + 10
25651712 25732935 ENSG00000280809 + 386
10 25718959 25719759 Homo_sapiens_10_25718978-25720776-HERVHF-pro + 10
25651712 25732935 ENSG00000280809 + 800
10 25719121 25721515 Homo_sapiens_10_25718978-25720776-HERVHF-pol + 10
25651712 25732935 ENSG00000280809 + 2394
10 25722171 25722503 Homo_sapiens_10_25718978-25720776-HERVHF-env + 10
25651712 25732935 ENSG00000280809 + 332
10 25722512 25722928 Homo_sapiens_10_25718978-25720776-HERVHF-3LTR + 10
25651712 25732935 ENSG00000280809 + 416
10 25716420 25722928 Homo_sapiens_10_25718978-25720776-HERVHF + 10 25651719
25732819 ENSG00000280809 + 6508
10 25716420 25716834 Homo_sapiens_10_25718978-25720776-HERVHF-5LTR + 10
25651719 25732819 ENSG00000280809 + 414
10 25718477 25718863 Homo_sapiens_10_25718978-25720776-HERVHF-gag + 10

25651719 25732819 ENSG00000280809 + 386
10 25718959 25719759 Homo_sapiens_10_25718978-25720776-HERVHF-pro + 10
25651719 25732819 ENSG00000280809 + 800
10 25719121 25721515 Homo_sapiens_10_25718978-25720776-HERVHF-pol + 10
25651719 25732819 ENSG00000280809 + 2394
10 25722171 25722503 Homo_sapiens_10_25718978-25720776-HERVHF-env + 10
25651719 25732819 ENSG00000280809 + 332
10 25722512 25722928 Homo_sapiens_10_25718978-25720776-HERVHF-3LTR + 10
25651719 25732819 ENSG00000280809 + 416
10 25716420 25722928 Homo_sapiens_10_25718978-25720776-HERVHF + 10 25651735
25732741 ENSG00000280809 + 6508
10 25716420 25716834 Homo_sapiens_10_25718978-25720776-HERVHF-5LTR + 10
25651735 25732741 ENSG00000280809 + 414
10 25718477 25718863 Homo_sapiens_10_25718978-25720776-HERVHF-gag + 10
25651735 25732741 ENSG00000280809 + 386
10 25718959 25719759 Homo_sapiens_10_25718978-25720776-HERVHF-pro + 10
25651735 25732741 ENSG00000280809 + 800
10 25719121 25721515 Homo_sapiens_10_25718978-25720776-HERVHF-pol + 10
25651735 25732741 ENSG00000280809 + 2394
10 25722171 25722503 Homo_sapiens_10_25718978-25720776-HERVHF-env + 10
25651735 25732741 ENSG00000280809 + 332
10 25722512 25722928 Homo_sapiens_10_25718978-25720776-HERVHF-3LTR + 10
25651735 25732741 ENSG00000280809 + 416
10 25716420 25722928 Homo_sapiens_10_25718978-25720776-HERVHF + 10 25651747
25732741 ENSG00000280809 + 6508
10 25716420 25716834 Homo_sapiens_10_25718978-25720776-HERVHF-5LTR + 10
25651747 25732741 ENSG00000280809 + 414
10 25718477 25718863 Homo_sapiens_10_25718978-25720776-HERVHF-gag + 10
25651747 25732741 ENSG00000280809 + 386
10 25718959 25719759 Homo_sapiens_10_25718978-25720776-HERVHF-pro + 10
25651747 25732741 ENSG00000280809 + 800
10 25719121 25721515 Homo_sapiens_10_25718978-25720776-HERVHF-pol + 10
25651747 25732741 ENSG00000280809 + 2394
10 25722171 25722503 Homo_sapiens_10_25718978-25720776-HERVHF-env + 10
25651747 25732741 ENSG00000280809 + 332
10 25722512 25722928 Homo_sapiens_10_25718978-25720776-HERVHF-3LTR + 10
25651747 25732741 ENSG00000280809 + 416
10 25716420 25722928 Homo_sapiens_10_25718978-25720776-HERVHF + 10 25651748
25732774 ENSG00000280809 + 6508
10 25716420 25716834 Homo_sapiens_10_25718978-25720776-HERVHF-5LTR + 10
25651748 25732774 ENSG00000280809 + 414
10 25716420 25722928 Homo_sapiens_10_25718978-25720776-HERVHF + 10 25651748
25732741 ENSG00000280809 + 6508
10 25716420 25716834 Homo_sapiens_10_25718978-25720776-HERVHF-5LTR + 10

25651748 25732741 ENSG00000280809 + 414
10 25716420 25722928 Homo_sapiens_10_25718978-25720776-HERVHF + 10 25651748 25732741 ENSG00000280809 + 6508
10 25716420 25716834 Homo_sapiens_10_25718978-25720776-HERVHF-5LTR + 10 25651748 25732741 ENSG00000280809 + 414
10 25718477 25718863 Homo_sapiens_10_25718978-25720776-HERVHF-gag + 10 25651748 25732774 ENSG00000280809 + 386
10 25718477 25718863 Homo_sapiens_10_25718978-25720776-HERVHF-gag + 10 25651748 25732741 ENSG00000280809 + 386
10 25718477 25718863 Homo_sapiens_10_25718978-25720776-HERVHF-gag + 10 25651748 25732741 ENSG00000280809 + 386
10 25718959 25719759 Homo_sapiens_10_25718978-25720776-HERVHF-pro + 10 25651748 25732774 ENSG00000280809 + 800
10 25718959 25719759 Homo_sapiens_10_25718978-25720776-HERVHF-pro + 10 25651748 25732741 ENSG00000280809 + 800
10 25718959 25719759 Homo_sapiens_10_25718978-25720776-HERVHF-pro + 10 25651748 25732741 ENSG00000280809 + 800
10 25719121 25721515 Homo_sapiens_10_25718978-25720776-HERVHF-pol + 10 25651748 25732774 ENSG00000280809 + 2394
10 25719121 25721515 Homo_sapiens_10_25718978-25720776-HERVHF-pol + 10 25651748 25732741 ENSG00000280809 + 2394
10 25719121 25721515 Homo_sapiens_10_25718978-25720776-HERVHF-pol + 10 25651748 25732741 ENSG00000280809 + 2394
10 25722171 25722503 Homo_sapiens_10_25718978-25720776-HERVHF-env + 10 25651748 25732774 ENSG00000280809 + 332
10 25722171 25722503 Homo_sapiens_10_25718978-25720776-HERVHF-env + 10 25651748 25732741 ENSG00000280809 + 332
10 25722171 25722503 Homo_sapiens_10_25718978-25720776-HERVHF-env + 10 25651748 25732741 ENSG00000280809 + 332
10 25722512 25722928 Homo_sapiens_10_25718978-25720776-HERVHF-3LTR + 10 25651748 25732774 ENSG00000280809 + 416
10 25722512 25722928 Homo_sapiens_10_25718978-25720776-HERVHF-3LTR + 10 25651748 25732741 ENSG00000280809 + 416
10 25722512 25722928 Homo_sapiens_10_25718978-25720776-HERVHF-3LTR + 10 25651748 25732741 ENSG00000280809 + 416
10 25716420 25722928 Homo_sapiens_10_25718978-25720776-HERVHF + 10 25651753 25732196 ENSG00000280809 + 6508
10 25716420 25716834 Homo_sapiens_10_25718978-25720776-HERVHF-5LTR + 10 25651753 25732196 ENSG00000280809 + 414
10 25718477 25718863 Homo_sapiens_10_25718978-25720776-HERVHF-gag + 10 25651753 25732196 ENSG00000280809 + 386
10 25718959 25719759 Homo_sapiens_10_25718978-25720776-HERVHF-pro + 10 25651753 25732196 ENSG00000280809 + 800
10 25719121 25721515 Homo_sapiens_10_25718978-25720776-HERVHF-pol + 10

25651753 25732196 ENSG00000280809 + 2394
10 25722171 25722503 Homo_sapiens_10_25718978-25720776-HERVHF-env + 10
25651753 25732196 ENSG00000280809 + 332
10 25722512 25722928 Homo_sapiens_10_25718978-25720776-HERVHF-3LTR + 10
25651753 25732196 ENSG00000280809 + 416
10 25716420 25722928 Homo_sapiens_10_25718978-25720776-HERVHF + 10 25651766
25732741 ENSG00000280809 + 6508
10 25716420 25716834 Homo_sapiens_10_25718978-25720776-HERVHF-5LTR + 10
25651766 25732741 ENSG00000280809 + 414
10 25718477 25718863 Homo_sapiens_10_25718978-25720776-HERVHF-gag + 10
25651766 25732741 ENSG00000280809 + 386
10 25718959 25719759 Homo_sapiens_10_25718978-25720776-HERVHF-pro + 10
25651766 25732741 ENSG00000280809 + 800
10 25719121 25721515 Homo_sapiens_10_25718978-25720776-HERVHF-pol + 10
25651766 25732741 ENSG00000280809 + 2394
10 25722171 25722503 Homo_sapiens_10_25718978-25720776-HERVHF-env + 10
25651766 25732741 ENSG00000280809 + 332
10 25722512 25722928 Homo_sapiens_10_25718978-25720776-HERVHF-3LTR + 10
25651766 25732741 ENSG00000280809 + 416
10 25716420 25722928 Homo_sapiens_10_25718978-25720776-HERVHF + 10 25651768
25731672 ENSG00000280809 + 6508
10 25716420 25716834 Homo_sapiens_10_25718978-25720776-HERVHF-5LTR + 10
25651768 25731672 ENSG00000280809 + 414
10 25716420 25722928 Homo_sapiens_10_25718978-25720776-HERVHF + 10 25651768
25732741 ENSG00000280809 + 6508
10 25716420 25716834 Homo_sapiens_10_25718978-25720776-HERVHF-5LTR + 10
25651768 25732741 ENSG00000280809 + 414
10 25716420 25722928 Homo_sapiens_10_25718978-25720776-HERVHF + 10 25651768
25732741 ENSG00000280809 + 6508
10 25716420 25716834 Homo_sapiens_10_25718978-25720776-HERVHF-5LTR + 10
25651768 25732741 ENSG00000280809 + 414
10 25718477 25718863 Homo_sapiens_10_25718978-25720776-HERVHF-gag + 10
25651768 25731672 ENSG00000280809 + 386
10 25718477 25718863 Homo_sapiens_10_25718978-25720776-HERVHF-gag + 10
25651768 25732741 ENSG00000280809 + 386
10 25718477 25718863 Homo_sapiens_10_25718978-25720776-HERVHF-gag + 10
25651768 25732741 ENSG00000280809 + 386
10 25718959 25719759 Homo_sapiens_10_25718978-25720776-HERVHF-pro + 10
25651768 25731672 ENSG00000280809 + 800
10 25718959 25719759 Homo_sapiens_10_25718978-25720776-HERVHF-pro + 10
25651768 25732741 ENSG00000280809 + 800
10 25718959 25719759 Homo_sapiens_10_25718978-25720776-HERVHF-pro + 10
25651768 25732741 ENSG00000280809 + 800
10 25719121 25721515 Homo_sapiens_10_25718978-25720776-HERVHF-pol + 10

25651768 25731672 ENSG00000280809 + 2394
10 25719121 25721515 Homo_sapiens_10_25718978-25720776-HERVHF-pol + 10
25651768 25732741 ENSG00000280809 + 2394
10 25719121 25721515 Homo_sapiens_10_25718978-25720776-HERVHF-pol + 10
25651768 25732741 ENSG00000280809 + 2394
10 25722171 25722503 Homo_sapiens_10_25718978-25720776-HERVHF-env + 10
25651768 25731672 ENSG00000280809 + 332
10 25722171 25722503 Homo_sapiens_10_25718978-25720776-HERVHF-env + 10
25651768 25732741 ENSG00000280809 + 332
10 25722171 25722503 Homo_sapiens_10_25718978-25720776-HERVHF-env + 10
25651768 25732741 ENSG00000280809 + 332
10 25722512 25722928 Homo_sapiens_10_25718978-25720776-HERVHF-3LTR + 10
25651768 25731672 ENSG00000280809 + 416
10 25722512 25722928 Homo_sapiens_10_25718978-25720776-HERVHF-3LTR + 10
25651768 25732741 ENSG00000280809 + 416
10 25722512 25722928 Homo_sapiens_10_25718978-25720776-HERVHF-3LTR + 10
25651768 25732741 ENSG00000280809 + 416
10 25716420 25722928 Homo_sapiens_10_25718978-25720776-HERVHF + 10 25652789
25732771 ENSG00000280809 + 6508
10 25716420 25716834 Homo_sapiens_10_25718978-25720776-HERVHF-5LTR + 10
25652789 25732771 ENSG00000280809 + 414
10 25718477 25718863 Homo_sapiens_10_25718978-25720776-HERVHF-gag + 10
25652789 25732771 ENSG00000280809 + 386
10 25718959 25719759 Homo_sapiens_10_25718978-25720776-HERVHF-pro + 10
25652789 25732771 ENSG00000280809 + 800
10 25719121 25721515 Homo_sapiens_10_25718978-25720776-HERVHF-pol + 10
25652789 25732771 ENSG00000280809 + 2394
10 25722171 25722503 Homo_sapiens_10_25718978-25720776-HERVHF-env + 10
25652789 25732771 ENSG00000280809 + 332
10 25722512 25722928 Homo_sapiens_10_25718978-25720776-HERVHF-3LTR + 10
25652789 25732771 ENSG00000280809 + 416
10 25716420 25722928 Homo_sapiens_10_25718978-25720776-HERVHF + 10 25686474
25732088 ENSG00000280809 + 6508
10 25716420 25716834 Homo_sapiens_10_25718978-25720776-HERVHF-5LTR + 10
25686474 25732088 ENSG00000280809 + 414
10 25718477 25718863 Homo_sapiens_10_25718978-25720776-HERVHF-gag + 10
25686474 25732088 ENSG00000280809 + 386
10 25718959 25719759 Homo_sapiens_10_25718978-25720776-HERVHF-pro + 10
25686474 25732088 ENSG00000280809 + 800
10 25719121 25721515 Homo_sapiens_10_25718978-25720776-HERVHF-pol + 10
25686474 25732088 ENSG00000280809 + 2394
10 25722171 25722503 Homo_sapiens_10_25718978-25720776-HERVHF-env + 10
25686474 25732088 ENSG00000280809 + 332
10 25722512 25722928 Homo_sapiens_10_25718978-25720776-HERVHF-3LTR + 10

25686474 25732088 ENSG00000280809 + 416
10 25716420 25722928 Homo_sapiens_10_25718978-25720776-HERVHF + 10 25686479 25732820 ENSG00000280809 + 6508
10 25716420 25716834 Homo_sapiens_10_25718978-25720776-HERVHF-5LTR + 10 25686479 25732820 ENSG00000280809 + 414
10 25718477 25718863 Homo_sapiens_10_25718978-25720776-HERVHF-gag + 10 25686479 25732820 ENSG00000280809 + 386
10 25718959 25719759 Homo_sapiens_10_25718978-25720776-HERVHF-pro + 10 25686479 25732820 ENSG00000280809 + 800
10 25719121 25721515 Homo_sapiens_10_25718978-25720776-HERVHF-pol + 10 25686479 25732820 ENSG00000280809 + 2394
10 25722171 25722503 Homo_sapiens_10_25718978-25720776-HERVHF-env + 10 25686479 25732820 ENSG00000280809 + 332
10 25722512 25722928 Homo_sapiens_10_25718978-25720776-HERVHF-3LTR + 10 25686479 25732820 ENSG00000280809 + 416
10 25716420 25722928 Homo_sapiens_10_25718978-25720776-HERVHF + 10 25691536 25772941 NONHSAG005428.2 + 6508
10 25716420 25716834 Homo_sapiens_10_25718978-25720776-HERVHF-5LTR + 10 25691536 25772941 NONHSAG005428.2 + 414
10 25718477 25718863 Homo_sapiens_10_25718978-25720776-HERVHF-gag + 10 25691536 25772941 NONHSAG005428.2 + 386
10 25718959 25719759 Homo_sapiens_10_25718978-25720776-HERVHF-pro + 10 25691536 25772941 NONHSAG005428.2 + 800
10 25719121 25721515 Homo_sapiens_10_25718978-25720776-HERVHF-pol + 10 25691536 25772941 NONHSAG005428.2 + 2394
10 25722171 25722503 Homo_sapiens_10_25718978-25720776-HERVHF-env + 10 25691536 25772941 NONHSAG005428.2 + 332
10 25722512 25722928 Homo_sapiens_10_25718978-25720776-HERVHF-3LTR + 10 25691536 25772941 NONHSAG005428.2 + 416
10 25716420 25722928 Homo_sapiens_10_25718978-25720776-HERVHF + 10 25691717 25772941 NONHSAT155235.1 + 6508
10 25716420 25716834 Homo_sapiens_10_25718978-25720776-HERVHF-5LTR + 10 25691717 25772941 NONHSAT155235.1 + 414
10 25718477 25718863 Homo_sapiens_10_25718978-25720776-HERVHF-gag + 10 25691717 25772941 NONHSAT155235.1 + 386
10 25718959 25719759 Homo_sapiens_10_25718978-25720776-HERVHF-pro + 10 25691717 25772941 NONHSAT155235.1 + 800
10 25719121 25721515 Homo_sapiens_10_25718978-25720776-HERVHF-pol + 10 25691717 25772941 NONHSAT155235.1 + 2394
10 25722171 25722503 Homo_sapiens_10_25718978-25720776-HERVHF-env + 10 25691717 25772941 NONHSAT155235.1 + 332
10 25722512 25722928 Homo_sapiens_10_25718978-25720776-HERVHF-3LTR + 10 25691717 25772941 NONHSAT155235.1 + 416
10 25716420 25716834 Homo_sapiens_10_25718978-25720776-HERVHF-5LTR + 10

25691780 25722651 NONHSAT155236.1 + 414
10 25718477 25718863 Homo_sapiens_10_25718978-25720776-HERVHF-gag + 10
25691780 25722651 NONHSAT155236.1 + 386
10 25718959 25719759 Homo_sapiens_10_25718978-25720776-HERVHF-pro + 10
25691780 25722651 NONHSAT155236.1 + 800
10 25719121 25721515 Homo_sapiens_10_25718978-25720776-HERVHF-pol + 10
25691780 25722651 NONHSAT155236.1 + 2394
10 25722171 25722503 Homo_sapiens_10_25718978-25720776-HERVHF-env + 10
25691780 25722651 NONHSAT155236.1 + 332
10 25716420 25722928 Homo_sapiens_10_25718978-25720776-HERVHF + 10 25715562
25732765 ENSG00000280809 + 6508
10 25716420 25716834 Homo_sapiens_10_25718978-25720776-HERVHF-5LTR + 10
25715562 25732765 ENSG00000280809 + 414
10 25718477 25718863 Homo_sapiens_10_25718978-25720776-HERVHF-gag + 10
25715562 25732765 ENSG00000280809 + 386
10 25718959 25719759 Homo_sapiens_10_25718978-25720776-HERVHF-pro + 10
25715562 25732765 ENSG00000280809 + 800
10 25719121 25721515 Homo_sapiens_10_25718978-25720776-HERVHF-pol + 10
25715562 25732765 ENSG00000280809 + 2394
10 25722171 25722503 Homo_sapiens_10_25718978-25720776-HERVHF-env + 10
25715562 25732765 ENSG00000280809 + 332
10 25722512 25722928 Homo_sapiens_10_25718978-25720776-HERVHF-3LTR + 10
25715562 25732765 ENSG00000280809 + 416
10 25718477 25718863 Homo_sapiens_10_25718978-25720776-HERVHF-gag + 10
25716739 25732931 ENSG00000280809 + 386
10 25718959 25719759 Homo_sapiens_10_25718978-25720776-HERVHF-pro + 10
25716739 25732931 ENSG00000280809 + 800
10 25719121 25721515 Homo_sapiens_10_25718978-25720776-HERVHF-pol + 10
25716739 25732931 ENSG00000280809 + 2394
10 25722171 25722503 Homo_sapiens_10_25718978-25720776-HERVHF-env + 10
25716739 25732931 ENSG00000280809 + 332
10 25722512 25722928 Homo_sapiens_10_25718978-25720776-HERVHF-3LTR + 10
25716739 25732931 ENSG00000280809 + 416
11 6366039 6371662 Homo_sapiens_11_6368276-6369885-HERVHF + 11 6366346
6371567 NONHSAG007525.2 + 5221
11 6366039 6371662 Homo_sapiens_11_6368276-6369885-HERVHF + 11 6366346
6371567 NONHSAT017712.2 + 5221
11 6367846 6368226 Homo_sapiens_11_6368276-6369885-HERVHF-gag + 11 6366346
6371567 NONHSAG007525.2 + 380
11 6367846 6368226 Homo_sapiens_11_6368276-6369885-HERVHF-gag + 11 6366346
6371567 NONHSAT017712.2 + 380
11 6368676 6370836 Homo_sapiens_11_6368276-6369885-HERVHF-pol + 11 6366346
6371567 NONHSAG007525.2 + 2160
11 6368676 6370836 Homo_sapiens_11_6368276-6369885-HERVHF-pol + 11 6366346

6371567 NONHSAT017712.2 + 2160
11 6366039 6371662 Homo_sapiens_11_6368276-6369885-HERVHF + 11 6368204
6371567 NONHSAT017713.2 + 3363
11 6368676 6370836 Homo_sapiens_11_6368276-6369885-HERVHF-pol + 11 6368204
6371567 NONHSAT017713.2 + 2160
11 27629072 27629528 Homo_sapiens_11_27630864-27632291-HERVHF-5LTR - 11
27599343 27634720 NONHSAG007904.2 - 456
11 27629072 27629528 Homo_sapiens_11_27630864-27632291-HERVHF-5LTR - 11
27599343 27634720 NONHSAT018512.2 - 456
11 27630027 27632049 Homo_sapiens_11_27630864-27632291-HERVHF-pol - 11
27599343 27634720 NONHSAG007904.2 - 2022
11 27630027 27632049 Homo_sapiens_11_27630864-27632291-HERVHF-pol - 11
27599343 27634720 NONHSAT018512.2 - 2022
11 27631507 27632340 Homo_sapiens_11_27630864-27632291-HERVHF-pro - 11
27599343 27634720 NONHSAG007904.2 - 833
11 27631507 27632340 Homo_sapiens_11_27630864-27632291-HERVHF-pro - 11
27599343 27634720 NONHSAT018512.2 - 833
11 27632416 27632889 Homo_sapiens_11_27630864-27632291-HERVHF-gag - 11
27599343 27634720 NONHSAG007904.2 - 473
11 27632416 27632889 Homo_sapiens_11_27630864-27632291-HERVHF-gag - 11
27599343 27634720 NONHSAT018512.2 - 473
11 27629072 27629528 Homo_sapiens_11_27630864-27632291-HERVHF-5LTR - 11
27617625 27634522 NONHSAT018513.2 - 456
11 27629072 27629528 Homo_sapiens_11_27630864-27632291-HERVHF-5LTR - 11
27617625 27634627 NONHSAT018514.2 - 456
11 27630027 27632049 Homo_sapiens_11_27630864-27632291-HERVHF-pol - 11
27617625 27634522 NONHSAT018513.2 - 2022
11 27630027 27632049 Homo_sapiens_11_27630864-27632291-HERVHF-pol - 11
27617625 27634627 NONHSAT018514.2 - 2022
11 27631507 27632340 Homo_sapiens_11_27630864-27632291-HERVHF-pro - 11
27617625 27634522 NONHSAT018513.2 - 833
11 27631507 27632340 Homo_sapiens_11_27630864-27632291-HERVHF-pro - 11
27617625 27634627 NONHSAT018514.2 - 833
11 27632416 27632889 Homo_sapiens_11_27630864-27632291-HERVHF-gag - 11
27617625 27634522 NONHSAT018513.2 - 473
11 27632416 27632889 Homo_sapiens_11_27630864-27632291-HERVHF-gag - 11
27617625 27634627 NONHSAT018514.2 - 473
11 27629072 27629528 Homo_sapiens_11_27630864-27632291-HERVHF-5LTR - 11
27617626 27634522 ENSG00000254934 - 456
11 27629072 27629528 Homo_sapiens_11_27630864-27632291-HERVHF-5LTR - 11
27617626 27634628 NONHSAT160176.1 - 456
11 27630027 27632049 Homo_sapiens_11_27630864-27632291-HERVHF-pol - 11
27617626 27634522 ENSG00000254934 - 2022
11 27630027 27632049 Homo_sapiens_11_27630864-27632291-HERVHF-pol - 11

27617626 27634628 NONHSAT160176.1 - 2022
11 27631507 27632340 Homo_sapiens_11_27630864-27632291-HERVHF-pro - 11
27617626 27634522 ENSG00000254934 - 833
11 27631507 27632340 Homo_sapiens_11_27630864-27632291-HERVHF-pro - 11
27617626 27634628 NONHSAT160176.1 - 833
11 27632416 27632889 Homo_sapiens_11_27630864-27632291-HERVHF-gag - 11
27617626 27634522 ENSG00000254934 - 473
11 27632416 27632889 Homo_sapiens_11_27630864-27632291-HERVHF-gag - 11
27617626 27634628 NONHSAT160176.1 - 473
11 27629072 27629528 Homo_sapiens_11_27630864-27632291-HERVHF-5LTR - 11
27617992 27634627 NONHSAT018515.2 - 456
11 27630027 27632049 Homo_sapiens_11_27630864-27632291-HERVHF-pol - 11
27617992 27634627 NONHSAT018515.2 - 2022
11 27631507 27632340 Homo_sapiens_11_27630864-27632291-HERVHF-pro - 11
27617992 27634627 NONHSAT018515.2 - 833
11 27632416 27632889 Homo_sapiens_11_27630864-27632291-HERVHF-gag - 11
27617992 27634627 NONHSAT018515.2 - 473
11 27629072 27629528 Homo_sapiens_11_27630864-27632291-HERVHF-5LTR - 11
27617993 27634627 ENSG00000254934 - 456
11 27630027 27632049 Homo_sapiens_11_27630864-27632291-HERVHF-pol - 11
27617993 27634627 ENSG00000254934 - 2022
11 27631507 27632340 Homo_sapiens_11_27630864-27632291-HERVHF-pro - 11
27617993 27634627 ENSG00000254934 - 833
11 27632416 27632889 Homo_sapiens_11_27630864-27632291-HERVHF-gag - 11
27617993 27634627 ENSG00000254934 - 473
11 27629072 27629528 Homo_sapiens_11_27630864-27632291-HERVHF-5LTR - 11
27618025 27634551 NONHSAT018516.2 - 456
11 27629072 27629528 Homo_sapiens_11_27630864-27632291-HERVHF-5LTR - 11
27618025 27634551 NONHSAT018517.2 - 456
11 27630027 27632049 Homo_sapiens_11_27630864-27632291-HERVHF-pol - 11
27618025 27634551 NONHSAT018516.2 - 2022
11 27630027 27632049 Homo_sapiens_11_27630864-27632291-HERVHF-pol - 11
27618025 27634551 NONHSAT018517.2 - 2022
11 27631507 27632340 Homo_sapiens_11_27630864-27632291-HERVHF-pro - 11
27618025 27634551 NONHSAT018516.2 - 833
11 27631507 27632340 Homo_sapiens_11_27630864-27632291-HERVHF-pro - 11
27618025 27634551 NONHSAT018517.2 - 833
11 27632416 27632889 Homo_sapiens_11_27630864-27632291-HERVHF-gag - 11
27618025 27634551 NONHSAT018516.2 - 473
11 27632416 27632889 Homo_sapiens_11_27630864-27632291-HERVHF-gag - 11
27618025 27634551 NONHSAT018517.2 - 473
11 94641661 94647315 Homo_sapiens_11_94644134-94645475-HERVHF + 11 94637790
94651631 NONHSAG009399.3 + 5654
11 94641661 94642011 Homo_sapiens_11_94644134-94645475-HERVHF-5LTR + 11

94637790 94651631 NONHSAG009399.3 + 350
11 94644136 94644915 Homo_sapiens_11_94644134-94645475-HERVHF-pro + 11
94637790 94651631 NONHSAG009399.3 + 779
11 94644373 94646313 Homo_sapiens_11_94644134-94645475-HERVHF-pol + 11
94637790 94651631 NONHSAG009399.3 + 1940
11 94646973 94647315 Homo_sapiens_11_94644134-94645475-HERVHF-3LTR + 11
94637790 94651631 NONHSAG009399.3 + 342
11 94641661 94647315 Homo_sapiens_11_94644134-94645475-HERVHF + 11 94638045
94652884 ENSG00000255666 + 5654
11 94641661 94642011 Homo_sapiens_11_94644134-94645475-HERVHF-5LTR + 11
94638045 94652884 ENSG00000255666 + 350
11 94644136 94644915 Homo_sapiens_11_94644134-94645475-HERVHF-pro + 11
94638045 94652884 ENSG00000255666 + 779
11 94644373 94646313 Homo_sapiens_11_94644134-94645475-HERVHF-pol + 11
94638045 94652884 ENSG00000255666 + 1940
11 94646973 94647315 Homo_sapiens_11_94644134-94645475-HERVHF-3LTR + 11
94638045 94652884 ENSG00000255666 + 342
11 94641661 94647315 Homo_sapiens_11_94644134-94645475-HERVHF + 11 94638070
94651631 ENSG00000255666 + 5654
11 94641661 94642011 Homo_sapiens_11_94644134-94645475-HERVHF-5LTR + 11
94638070 94651631 ENSG00000255666 + 350
11 94644136 94644915 Homo_sapiens_11_94644134-94645475-HERVHF-pro + 11
94638070 94651631 ENSG00000255666 + 779
11 94644373 94646313 Homo_sapiens_11_94644134-94645475-HERVHF-pol + 11
94638070 94651631 ENSG00000255666 + 1940
11 94646973 94647315 Homo_sapiens_11_94644134-94645475-HERVHF-3LTR + 11
94638070 94651631 ENSG00000255666 + 342
11 94641661 94642011 Homo_sapiens_11_94644134-94645475-HERVHF-5LTR + 11
94640098 94642192 NONHSAT230024.1 + 350
11 94644136 94644915 Homo_sapiens_11_94644134-94645475-HERVHF-pro + 11
94641906 94651628 NONHSAT023734.2 + 779
11 94644373 94646313 Homo_sapiens_11_94644134-94645475-HERVHF-pol + 11
94641906 94651628 NONHSAT023734.2 + 1940
11 94646973 94647315 Homo_sapiens_11_94644134-94645475-HERVHF-3LTR + 11
94641906 94651628 NONHSAT023734.2 + 342
11 94644136 94644915 Homo_sapiens_11_94644134-94645475-HERVHF-pro + 11
94641907 94651628 ENSG00000255666 + 779
11 94644373 94646313 Homo_sapiens_11_94644134-94645475-HERVHF-pol + 11
94641907 94651628 ENSG00000255666 + 1940
11 94646973 94647315 Homo_sapiens_11_94644134-94645475-HERVHF-3LTR + 11
94641907 94651628 ENSG00000255666 + 342
11 94644136 94644915 Homo_sapiens_11_94644134-94645475-HERVHF-pro + 11
94641929 94648574 NONHSAT023735.2 + 779
11 94644373 94646313 Homo_sapiens_11_94644134-94645475-HERVHF-pol + 11

94641929 94648574 NONHSAT023735.2 + 1940
11 94646973 94647315 Homo_sapiens_11_94644134-94645475-HERVHF-3LTR + 11
94641929 94648574 NONHSAT023735.2 + 342
11 94644136 94644915 Homo_sapiens_11_94644134-94645475-HERVHF-pro + 11
94641930 94648574 ENSG00000255666 + 779
11 94644373 94646313 Homo_sapiens_11_94644134-94645475-HERVHF-pol + 11
94641930 94648574 ENSG00000255666 + 1940
11 94646973 94647315 Homo_sapiens_11_94644134-94645475-HERVHF-3LTR + 11
94641930 94648574 ENSG00000255666 + 342
11 96499960 96500271 Homo_sapiens_11_96501724-96503654-HERVHF-5LTR + 11
96389989 96507574 ENSG00000183340 + 311
11 96501014 96501424 Homo_sapiens_11_96501724-96503654-HERVHF-gag + 11
96389989 96507574 ENSG00000183340 + 410
11 96501726 96502403 Homo_sapiens_11_96501724-96503654-HERVHF-pro + 11
96389989 96507574 ENSG00000183340 + 677
11 96501900 96504947 Homo_sapiens_11_96501724-96503654-HERVHF-pol + 11
96389989 96507574 ENSG00000183340 + 3047
11 96506073 96506627 Homo_sapiens_11_96501724-96503654-HERVHF-env + 11
96389989 96507574 ENSG00000183340 + 554
11 96587635 96588088 Homo_sapiens_11_96590449-96591982-HERVHF-5LTR + 11
96579577 96593188 NONHSAG009431.2 + 453
11 96587635 96588088 Homo_sapiens_11_96590449-96591982-HERVHF-5LTR + 11
96579577 96593188 NONHSAT023810.2 + 453
11 96590439 96591233 Homo_sapiens_11_96590449-96591982-HERVHF-pro + 11
96579577 96593188 NONHSAG009431.2 + 794
11 96590439 96591233 Homo_sapiens_11_96590449-96591982-HERVHF-pro + 11
96579577 96593188 NONHSAT023810.2 + 794
11 96590598 96592771 Homo_sapiens_11_96590449-96591982-HERVHF-pol + 11
96579577 96593188 NONHSAG009431.2 + 2173
11 96590598 96592771 Homo_sapiens_11_96590449-96591982-HERVHF-pol + 11
96579577 96593188 NONHSAT023810.2 + 2173
11 96587635 96593677 Homo_sapiens_11_96590449-96591982-HERVHF + 11 96587796
96588978 NONHSAT159567.1 + 1182
11 96590439 96591233 Homo_sapiens_11_96590449-96591982-HERVHF-pro + 11
96587796 96596592 NONHSAG009432.2 + 794
11 96590439 96591233 Homo_sapiens_11_96590449-96591982-HERVHF-pro + 11
96587796 96596592 NONHSAT023811.2+ 794
11 96590598 96592771 Homo_sapiens_11_96590449-96591982-HERVHF-pol + 11
96587796 96596592 NONHSAG009432.2 + 2173
11 96590598 96592771 Homo_sapiens_11_96590449-96591982-HERVHF-pol + 11
96587796 96596592 NONHSAT023811.2+ 2173
11 96593224 96593677 Homo_sapiens_11_96590449-96591982-HERVHF-3LTR + 11
96587796 96596592 NONHSAG009432.2 + 453
11 96593224 96593677 Homo_sapiens_11_96590449-96591982-HERVHF-3LTR + 11

96587796 96596592 NONHSAT023811.2+ 453
11 96590439 96591233 Homo_sapiens_11_96590449-96591982-HERVHF-pro + 11
96590316 96713822 NONHSAG009434.2 + 794
11 96590439 96591233 Homo_sapiens_11_96590449-96591982-HERVHF-pro + 11
96590316 96713822 NONHSAT023813.2 + 794
11 96590598 96592771 Homo_sapiens_11_96590449-96591982-HERVHF-pol + 11
96590316 96713822 NONHSAG009434.2 + 2173
11 96590598 96592771 Homo_sapiens_11_96590449-96591982-HERVHF-pol + 11
96590316 96713822 NONHSAT023813.2 + 2173
11 96593224 96593677 Homo_sapiens_11_96590449-96591982-HERVHF-3LTR + 11
96590316 96713822 NONHSAG009434.2 + 453
11 96593224 96593677 Homo_sapiens_11_96590449-96591982-HERVHF-3LTR + 11
96590316 96713822 NONHSAT023813.2 + 453
11 96590439 96591233 Homo_sapiens_11_96590449-96591982-HERVHF-pro + 11
96590317 96713822 ENSG00000254587 + 794
11 96590598 96592771 Homo_sapiens_11_96590449-96591982-HERVHF-pol + 11
96590317 96713822 ENSG00000254587 + 2173
11 96593224 96593677 Homo_sapiens_11_96590449-96591982-HERVHF-3LTR + 11
96590317 96713822 ENSG00000254587 + 453
11 130565609 130567330 Homo_sapiens_11_130566060-130567548-HERVHF-pol -
11 130564429 130758600 NONHSAG010050.2 - 1721
11 130565609 130567330 Homo_sapiens_11_130566060-130567548-HERVHF-pol -
11 130564429 130758600 NONHSAT025134.2 - 1721
11 130566809 130567627 Homo_sapiens_11_130566060-130567548-HERVHF-pro -
11 130564429 130758600 NONHSAG010050.2 - 818
11 130566809 130567627 Homo_sapiens_11_130566060-130567548-HERVHF-pro -
11 130564429 130758600 NONHSAT025134.2 - 818
11 130569681 130570121 Homo_sapiens_11_130566060-130567548-HERVHF-3LTR -
11 130564429 130758600 NONHSAG010050.2 - 440
11 130569681 130570121 Homo_sapiens_11_130566060-130567548-HERVHF-3LTR -
11 130564429 130758600 NONHSAT025134.2 - 440
11 130753494 130753946 Homo_sapiens_11_130755373-130756704-HERVHF-5LTR -
11 130564429 130758600 NONHSAG010050.2 - 452
11 130753494 130753946 Homo_sapiens_11_130755373-130756704-HERVHF-5LTR -
11 130564429 130758600 NONHSAT025134.2 - 452
11 130754355 130756582 Homo_sapiens_11_130755373-130756704-HERVHF-pol -
11 130564429 130758600 NONHSAG010050.2 - 2227
11 130754355 130756582 Homo_sapiens_11_130755373-130756704-HERVHF-pol -
11 130564429 130758600 NONHSAT025134.2 - 2227
11 130755941 130756702 Homo_sapiens_11_130755373-130756704-HERVHF-pro -
11 130564429 130758600 NONHSAG010050.2 - 761
11 130755941 130756702 Homo_sapiens_11_130755373-130756704-HERVHF-pro -
11 130564429 130758600 NONHSAT025134.2 - 761
12 4018623 4023691 Homo_sapiens_12_4021109-4022208-HERVHF + 12 4012902

4026658 ENSG00000256969 + 5068
12 4018623 4018993 Homo_sapiens_12_4021109-4022208-HERVHF-5LTR + 12 4012902
4026658 ENSG00000256969 + 370
12 4020593 4021006 Homo_sapiens_12_4021109-4022208-HERVHF-gag + 12 4012902
4026658 ENSG00000256969 + 413
12 4021003 4021665 Homo_sapiens_12_4021109-4022208-HERVHF-pro + 12 4012902
4026658 ENSG00000256969 + 662
12 4021511 4022836 Homo_sapiens_12_4021109-4022208-HERVHF-pol + 12 4012902
4026658 ENSG00000256969 + 1325
12 4023325 4023691 Homo_sapiens_12_4021109-4022208-HERVHF-3LTR + 12 4012902
4026658 ENSG00000256969 + 366
12 4018623 4023691 Homo_sapiens_12_4021109-4022208-HERVHF + 12 4012903
4026657 ENSG00000256969 + 5068
12 4018623 4018993 Homo_sapiens_12_4021109-4022208-HERVHF-5LTR + 12 4012903
4026657 ENSG00000256969 + 370
12 4020593 4021006 Homo_sapiens_12_4021109-4022208-HERVHF-gag + 12 4012903
4026657 ENSG00000256969 + 413
12 4021003 4021665 Homo_sapiens_12_4021109-4022208-HERVHF-pro + 12 4012903
4026657 ENSG00000256969 + 662
12 4021511 4022836 Homo_sapiens_12_4021109-4022208-HERVHF-pol + 12 4012903
4026657 ENSG00000256969 + 1325
12 4023325 4023691 Homo_sapiens_12_4021109-4022208-HERVHF-3LTR + 12 4012903
4026657 ENSG00000256969 + 366
12 4020593 4021006 Homo_sapiens_12_4021109-4022208-HERVHF-gag + 12 4019113
4026890 NONHSAG010232.2 + 413
12 4020593 4021006 Homo_sapiens_12_4021109-4022208-HERVHF-gag + 12 4019113
4026890 NONHSAT025939.2 + 413
12 4021003 4021665 Homo_sapiens_12_4021109-4022208-HERVHF-pro + 12 4019113
4026890 NONHSAG010232.2 + 662
12 4021003 4021665 Homo_sapiens_12_4021109-4022208-HERVHF-pro + 12 4019113
4026890 NONHSAT025939.2 + 662
12 4021511 4022836 Homo_sapiens_12_4021109-4022208-HERVHF-pol + 12 4019113
4026890 NONHSAG010232.2 + 1325
12 4021511 4022836 Homo_sapiens_12_4021109-4022208-HERVHF-pol + 12 4019113
4026890 NONHSAT025939.2 + 1325
12 4023325 4023691 Homo_sapiens_12_4021109-4022208-HERVHF-3LTR + 12 4019113
4026890 NONHSAG010232.2 + 366
12 4023325 4023691 Homo_sapiens_12_4021109-4022208-HERVHF-3LTR + 12 4019113
4026890 NONHSAT025939.2 + 366
12 4021003 4021665 Homo_sapiens_12_4021109-4022208-HERVHF-pro + 12 4020976
4026658 NONHSAT025940.2 + 662
12 4021511 4022836 Homo_sapiens_12_4021109-4022208-HERVHF-pol + 12 4020976
4026658 NONHSAT025940.2 + 1325
12 4023325 4023691 Homo_sapiens_12_4021109-4022208-HERVHF-3LTR + 12 4020976

4026658 NONHSAT025940.2 + 366
12 4021003 4021665 Homo_sapiens_12_4021109-4022208-HERVHF-pro + 12 4020977 4026658 ENSG00000256969 + 662
12 4021003 4021665 Homo_sapiens_12_4021109-4022208-HERVHF-pro + 12 4020977 4026659 NONHSAT161751.1 + 662
12 4021511 4022836 Homo_sapiens_12_4021109-4022208-HERVHF-pol + 12 4020977 4026658 ENSG00000256969 + 1325
12 4021511 4022836 Homo_sapiens_12_4021109-4022208-HERVHF-pol + 12 4020977 4026659 NONHSAT161751.1 + 1325
12 4023325 4023691 Homo_sapiens_12_4021109-4022208-HERVHF-3LTR + 12 4020977 4026658 ENSG00000256969 + 366
12 4023325 4023691 Homo_sapiens_12_4021109-4022208-HERVHF-3LTR + 12 4020977 4026659 NONHSAT161751.1 + 366
12 11462168 11468022 Homo_sapiens_12_11463877-11465381-HERVHF - 12 11393972 11501041 ENSG00000121335 - 5854
12 11462168 11462625 Homo_sapiens_12_11463877-11465381-HERVHF-5LTR- 12 11393972 11501041 ENSG00000121335 - 457
12 11463071 11465137 Homo_sapiens_12_11463877-11465381-HERVHF-pol - 12 11393972 11501041 ENSG00000121335 - 2066
12 11467569 11468022 Homo_sapiens_12_11463877-11465381-HERVHF-3LTR- 12 11393972 11501041 ENSG00000121335 - 453
12 11462168 11468022 Homo_sapiens_12_11463877-11465381-HERVHF - 12 11398707 11486679 NONHSAG010510.2 - 5854
12 11462168 11462625 Homo_sapiens_12_11463877-11465381-HERVHF-5LTR- 12 11398707 11486679 NONHSAG010510.2 - 457
12 11462168 11468022 Homo_sapiens_12_11463877-11465381-HERVHF - 12 11398707 11486679 NONHSAT163315.1 - 5854
12 11462168 11462625 Homo_sapiens_12_11463877-11465381-HERVHF-5LTR- 12 11398707 11486679 NONHSAT163315.1 - 457
12 11463071 11465137 Homo_sapiens_12_11463877-11465381-HERVHF-pol - 12 11398707 11486679 NONHSAG010510.2 - 2066
12 11463071 11465137 Homo_sapiens_12_11463877-11465381-HERVHF-pol - 12 11398707 11486679 NONHSAT163315.1 - 2066
12 11467569 11468022 Homo_sapiens_12_11463877-11465381-HERVHF-3LTR- 12 11398707 11486679 NONHSAG010510.2 - 453
12 11467569 11468022 Homo_sapiens_12_11463877-11465381-HERVHF-3LTR- 12 11398707 11486679 NONHSAT163315.1 - 453
12 11462168 11468022 Homo_sapiens_12_11463877-11465381-HERVHF - 12 11399380 11486678 NONHSAT026937.2 - 5854
12 11462168 11462625 Homo_sapiens_12_11463877-11465381-HERVHF-5LTR- 12 11399380 11486678 NONHSAT026937.2 - 457
12 11463071 11465137 Homo_sapiens_12_11463877-11465381-HERVHF-pol - 12 11399380 11486678 NONHSAT026937.2 - 2066
12 11467569 11468022 Homo_sapiens_12_11463877-11465381-HERVHF-3LTR- 12

11399380 11486678 NONHSAT026937.2 - 453
12 11462168 11468022 Homo_sapiens_12_11463877-11465381-HERVHF - 12 11399381
11486678 ENSG00000255790 - 5854
12 11462168 11462625 Homo_sapiens_12_11463877-11465381-HERVHF-5LTR- 12
11399381 11486678 ENSG00000255790 - 457
12 11463071 11465137 Homo_sapiens_12_11463877-11465381-HERVHF-pol - 12
11399381 11486678 ENSG00000255790 - 2066
12 11467569 11468022 Homo_sapiens_12_11463877-11465381-HERVHF-3LTR- 12
11399381 11486678 ENSG00000255790 - 453
12 11462168 11468022 Homo_sapiens_12_11463877-11465381-HERVHF - 12 11401484
11486674 NONHSAT026938.2 - 5854
12 11462168 11462625 Homo_sapiens_12_11463877-11465381-HERVHF-5LTR- 12
11401484 11486674 NONHSAT026938.2 - 457
12 11463071 11465137 Homo_sapiens_12_11463877-11465381-HERVHF-pol - 12
11401484 11486674 NONHSAT026938.2 - 2066
12 11467569 11468022 Homo_sapiens_12_11463877-11465381-HERVHF-3LTR- 12
11401484 11486674 NONHSAT026938.2 - 453
12 11462168 11468022 Homo_sapiens_12_11463877-11465381-HERVHF - 12 11401485
11486674 ENSG00000255790 - 5854
12 11462168 11462625 Homo_sapiens_12_11463877-11465381-HERVHF-5LTR- 12
11401485 11486674 ENSG00000255790 - 457
12 11463071 11465137 Homo_sapiens_12_11463877-11465381-HERVHF-pol - 12
11401485 11486674 ENSG00000255790 - 2066
12 11467569 11468022 Homo_sapiens_12_11463877-11465381-HERVHF-3LTR- 12
11401485 11486674 ENSG00000255790 - 453
12 11462168 11468022 Homo_sapiens_12_11463877-11465381-HERVHF - 12 11430215
11486708 ENSG00000255790 - 5854
12 11462168 11462625 Homo_sapiens_12_11463877-11465381-HERVHF-5LTR- 12
11430215 11486708 ENSG00000255790 - 457
12 11463071 11465137 Homo_sapiens_12_11463877-11465381-HERVHF-pol - 12
11430215 11486708 ENSG00000255790 - 2066
12 11467569 11468022 Homo_sapiens_12_11463877-11465381-HERVHF-3LTR- 12
11430215 11486708 ENSG00000255790 - 453
12 11462168 11468022 Homo_sapiens_12_11463877-11465381-HERVHF - 12 11430361
11486632 NONHSAT026942.2 - 5854
12 11462168 11462625 Homo_sapiens_12_11463877-11465381-HERVHF-5LTR- 12
11430361 11486632 NONHSAT026942.2 - 457
12 11463071 11465137 Homo_sapiens_12_11463877-11465381-HERVHF-pol - 12
11430361 11486632 NONHSAT026942.2 - 2066
12 11467569 11468022 Homo_sapiens_12_11463877-11465381-HERVHF-3LTR- 12
11430361 11486632 NONHSAT026942.2 - 453
12 11462168 11468022 Homo_sapiens_12_11463877-11465381-HERVHF - 12 11430362
11486632 ENSG00000255790 - 5854
12 11462168 11462625 Homo_sapiens_12_11463877-11465381-HERVHF-5LTR- 12

11430362 11486632 ENSG00000255790 - 457
12 11463071 11465137 Homo_sapiens_12_11463877-11465381-HERVHF-pol - 12
11430362 11486632 ENSG00000255790 - 2066
12 11467569 11468022 Homo_sapiens_12_11463877-11465381-HERVHF-3LTR- 12
11430362 11486632 ENSG00000255790 - 453
12 34269097 34271546 Homo_sapiens_12_34268101-34269869-HERVHF-pol + 12
34269048 34278082 NONHSAG010874.2 + 2449
12 34269097 34271546 Homo_sapiens_12_34268101-34269869-HERVHF-pol + 12
34269048 34278082 NONHSAT027661.2 + 2449
12 34272421 34273596 Homo_sapiens_12_34268101-34269869-HERVHF-env + 12
34269048 34278082 NONHSAG010874.2 + 1175
12 34272421 34273596 Homo_sapiens_12_34268101-34269869-HERVHF-env + 12
34269048 34278082 NONHSAT027661.2 + 1175
12 34273840 34274242 Homo_sapiens_12_34268101-34269869-HERVHF-3LTR + 12
34269048 34278082 NONHSAG010874.2 + 402
12 34273840 34274242 Homo_sapiens_12_34268101-34269869-HERVHF-3LTR + 12
34269048 34278082 NONHSAT027661.2 + 402
12 70444894 70450107 Homo_sapiens_12_70446553-70447732-HERVK - 12 70446459
70447585 NONHSAG011664.2 - 1126
12 70444894 70450107 Homo_sapiens_12_70446553-70447732-HERVK - 12 70446459
70447585 NONHSAT029412.2 - 1126
12 70445322 70447607 Homo_sapiens_12_70446553-70447732-HERVK-pol - 12
70446459 70447585 NONHSAG011664.2 - 1126
12 70445322 70447607 Homo_sapiens_12_70446553-70447732-HERVK-pol - 12
70446459 70447585 NONHSAT029412.2 - 1126
12 86941530 86943723 Homo_sapiens_12_86941432-86943069-HERVHF-pol + 12
86941138 86964891 NONHSAG064903.1 + 2193
12 86941530 86943723 Homo_sapiens_12_86941432-86943069-HERVHF-pol + 12
86941138 86964891 NONHSAT162510.1 + 2193
12 86944355 86944748 Homo_sapiens_12_86941432-86943069-HERVHF-3LTR + 12
86941138 86964891 NONHSAG064903.1 + 393
12 86944355 86944748 Homo_sapiens_12_86941432-86943069-HERVHF-3LTR + 12
86941138 86964891 NONHSAT162510.1 + 393
13 42868001 42868452 Homo_sapiens_13_42869513-42870545-HERVHF-5LTR - 13
42840531 42872900 NONHSAG013351.2 - 451
13 42868001 42868452 Homo_sapiens_13_42869513-42870545-HERVHF-5LTR - 13
42840531 42872900 NONHSAT033441.2 - 451
13 42869058 42870339 Homo_sapiens_13_42869513-42870545-HERVHF-pol - 13
42840531 42872900 NONHSAG013351.2 - 1281
13 42869058 42870339 Homo_sapiens_13_42869513-42870545-HERVHF-pol - 13
42840531 42872900 NONHSAT033441.2 - 1281
13 42869842 42870594 Homo_sapiens_13_42869513-42870545-HERVHF-pro - 13
42840531 42872900 NONHSAG013351.2 - 752
13 42869842 42870594 Homo_sapiens_13_42869513-42870545-HERVHF-pro - 13

42840531 42872900 NONHSAT033441.2 - 752
13 42870678 42871007 Homo_sapiens_13_42869513-42870545-HERVHF-gag - 13
42840531 42872900 NONHSAG013351.2 - 329
13 42870678 42871007 Homo_sapiens_13_42869513-42870545-HERVHF-gag - 13
42840531 42872900 NONHSAT033441.2 - 329
13 42868001 42868452 Homo_sapiens_13_42869513-42870545-HERVHF-5LTR - 13
42842411 42872744 NONHSAT166672.1 - 451
13 42869058 42870339 Homo_sapiens_13_42869513-42870545-HERVHF-pol - 13
42842411 42872744 NONHSAT166672.1 - 1281
13 42869842 42870594 Homo_sapiens_13_42869513-42870545-HERVHF-pro - 13
42842411 42872744 NONHSAT166672.1 - 752
13 42870678 42871007 Homo_sapiens_13_42869513-42870545-HERVHF-gag - 13
42842411 42872744 NONHSAT166672.1 - 329
13 42868001 42868452 Homo_sapiens_13_42869513-42870545-HERVHF-5LTR - 13
42850246 42872744 NONHSAT166675.1 - 451
13 42869058 42870339 Homo_sapiens_13_42869513-42870545-HERVHF-pol - 13
42850246 42872744 NONHSAT166675.1 - 1281
13 42869842 42870594 Homo_sapiens_13_42869513-42870545-HERVHF-pro - 13
42850246 42872744 NONHSAT166675.1 - 752
13 42870678 42871007 Homo_sapiens_13_42869513-42870545-HERVHF-gag - 13
42850246 42872744 NONHSAT166675.1 - 329
13 48866771 48872457 Homo_sapiens_13_48868391-48870330-HERVHF - 13 48854720
48874381 NONHSAG067525.1 - 5686
13 48866771 48867093 Homo_sapiens_13_48868391-48870330-HERVHF-5LTR - 13
48854720 48874381 NONHSAG067525.1 - 322
13 48866771 48872457 Homo_sapiens_13_48868391-48870330-HERVHF - 13 48854720
48874381 NONHSAT166751.1 - 5686
13 48866771 48867093 Homo_sapiens_13_48868391-48870330-HERVHF-5LTR - 13
48854720 48874381 NONHSAT166751.1 - 322
13 48868555 48870047 Homo_sapiens_13_48868391-48870330-HERVHF-pol - 13
48854720 48874381 NONHSAG067525.1 - 1492
13 48868555 48870047 Homo_sapiens_13_48868391-48870330-HERVHF-pol - 13
48854720 48874381 NONHSAT166751.1 - 1492
13 48869643 48870272 Homo_sapiens_13_48868391-48870330-HERVHF-pro - 13
48854720 48874381 NONHSAG067525.1 - 629
13 48869643 48870272 Homo_sapiens_13_48868391-48870330-HERVHF-pro - 13
48854720 48874381 NONHSAT166751.1 - 629
13 48872132 48872457 Homo_sapiens_13_48868391-48870330-HERVHF-3LTR - 13
48854720 48874381 NONHSAG067525.1 - 325
13 48872132 48872457 Homo_sapiens_13_48868391-48870330-HERVHF-3LTR - 13
48854720 48874381 NONHSAT166751.1 - 325
13 51169866 51175008 Homo_sapiens_13_51172521-51173517-HERVHF + 13 51169742
51187944 NONHSAG013541.3 + 5142
13 51169866 51170315 Homo_sapiens_13_51172521-51173517-HERVHF-5LTR+ 13

51169742 51187944 NONHSAG013541.3 + 449
13 51169866 51175008 Homo_sapiens_13_51172521-51173517-HERVHF + 13 51169742
51176200 NONHSAT232794.1 + 5142
13 51169866 51170315 Homo_sapiens_13_51172521-51173517-HERVHF-5LTR+ 13
51169742 51176200 NONHSAT232794.1 + 449
13 51172014 51172343 Homo_sapiens_13_51172521-51173517-HERVHF-gag + 13
51169742 51187944 NONHSAG013541.3 + 329
13 51172014 51172343 Homo_sapiens_13_51172521-51173517-HERVHF-gag + 13
51169742 51176200 NONHSAT232794.1 + 329
13 51172529 51173197 Homo_sapiens_13_51172521-51173517-HERVHF-pro + 13
51169742 51187944 NONHSAG013541.3 + 668
13 51172529 51173197 Homo_sapiens_13_51172521-51173517-HERVHF-pro + 13
51169742 51176200 NONHSAT232794.1 + 668
13 51172592 51174102 Homo_sapiens_13_51172521-51173517-HERVHF-pol + 13
51169742 51187944 NONHSAG013541.3 + 1510
13 51172592 51174102 Homo_sapiens_13_51172521-51173517-HERVHF-pol + 13
51169742 51176200 NONHSAT232794.1 + 1510
13 51174556 51175008 Homo_sapiens_13_51172521-51173517-HERVHF-3LTR+ 13
51169742 51187944 NONHSAG013541.3 + 452
13 51174556 51175008 Homo_sapiens_13_51172521-51173517-HERVHF-3LTR+ 13
51169742 51176200 NONHSAT232794.1 + 452
13 51172014 51172343 Homo_sapiens_13_51172521-51173517-HERVHF-gag + 13
51169990 51187944 NONHSAT033887.2 + 329
13 51172529 51173197 Homo_sapiens_13_51172521-51173517-HERVHF-pro + 13
51169990 51187944 NONHSAT033887.2 + 668
13 51172592 51174102 Homo_sapiens_13_51172521-51173517-HERVHF-pol + 13
51169990 51187944 NONHSAT033887.2 + 1510
13 51174556 51175008 Homo_sapiens_13_51172521-51173517-HERVHF-3LTR+ 13
51169990 51187944 NONHSAT033887.2 + 452
13 54127417 54127817 Homo_sapiens_13_54129305-54130960-HERVHF-5LTR - 13
54108833 54132891 NONHSAG013596.3 - 400
13 54127417 54127817 Homo_sapiens_13_54129305-54130960-HERVHF-5LTR - 13
54108833 54132891 NONHSAT233261.1 - 400
13 54128407 54130751 Homo_sapiens_13_54129305-54130960-HERVHF-pol - 13
54108833 54132891 NONHSAG013596.3 - 2344
13 54128407 54130751 Homo_sapiens_13_54129305-54130960-HERVHF-pol - 13
54108833 54132891 NONHSAT233261.1 - 2344
13 54130113 54130865 Homo_sapiens_13_54129305-54130960-HERVHF-pro - 13
54108833 54132891 NONHSAG013596.3 - 752
13 54130113 54130865 Homo_sapiens_13_54129305-54130960-HERVHF-pro - 13
54108833 54132891 NONHSAT233261.1 - 752
13 54130954 54131292 Homo_sapiens_13_54129305-54130960-HERVHF-gag - 13
54108833 54132891 NONHSAG013596.3 - 338
13 54130954 54131292 Homo_sapiens_13_54129305-54130960-HERVHF-gag - 13

54108833 54132891 NONHSAT233261.1 - 338
13 54127417 54127817 Homo_sapiens_13_54129305-54130960-HERVHF-5LTR - 13
54110836 54131272 NONHSAT034023.2 - 400
13 54128407 54130751 Homo_sapiens_13_54129305-54130960-HERVHF-pol - 13
54110836 54131272 NONHSAT034023.2 - 2344
13 54130113 54130865 Homo_sapiens_13_54129305-54130960-HERVHF-pro - 13
54110836 54131272 NONHSAT034023.2 - 752
13 54127417 54127817 Homo_sapiens_13_54129305-54130960-HERVHF-5LTR - 13
54113862 54132891 NONHSAT233262.1 - 400
13 54128407 54130751 Homo_sapiens_13_54129305-54130960-HERVHF-pol - 13
54113862 54132891 NONHSAT233262.1 - 2344
13 54130113 54130865 Homo_sapiens_13_54129305-54130960-HERVHF-pro - 13
54113862 54132891 NONHSAT233262.1 - 752
13 54130954 54131292 Homo_sapiens_13_54129305-54130960-HERVHF-gag - 13
54113862 54132891 NONHSAT233262.1 - 338
13 54127417 54127817 Homo_sapiens_13_54129305-54130960-HERVHF-5LTR - 13
54114978 54132891 NONHSAT233263.1 - 400
13 54128407 54130751 Homo_sapiens_13_54129305-54130960-HERVHF-pol - 13
54114978 54132891 NONHSAT233263.1 - 2344
13 54130113 54130865 Homo_sapiens_13_54129305-54130960-HERVHF-pro - 13
54114978 54132891 NONHSAT233263.1 - 752
13 54130954 54131292 Homo_sapiens_13_54129305-54130960-HERVHF-gag - 13
54114978 54132891 NONHSAT233263.1 - 338
13 54127417 54127817 Homo_sapiens_13_54129305-54130960-HERVHF-5LTR - 13
54115783 54132866 ENSG00000234787 - 400
13 54128407 54130751 Homo_sapiens_13_54129305-54130960-HERVHF-pol - 13
54115783 54132866 ENSG00000234787 - 2344
13 54130113 54130865 Homo_sapiens_13_54129305-54130960-HERVHF-pro - 13
54115783 54132866 ENSG00000234787 - 752
13 54130954 54131292 Homo_sapiens_13_54129305-54130960-HERVHF-gag - 13
54115783 54132866 ENSG00000234787 - 338
13 54127417 54127817 Homo_sapiens_13_54129305-54130960-HERVHF-5LTR - 13
54115788 54132866 NONHSAT034024.2 - 400
13 54127417 54127817 Homo_sapiens_13_54129305-54130960-HERVHF-5LTR - 13
54115788 54132891 NONHSAT034025.2 - 400
13 54128407 54130751 Homo_sapiens_13_54129305-54130960-HERVHF-pol - 13
54115788 54132866 NONHSAT034024.2 - 2344
13 54128407 54130751 Homo_sapiens_13_54129305-54130960-HERVHF-pol - 13
54115788 54132891 NONHSAT034025.2 - 2344
13 54130113 54130865 Homo_sapiens_13_54129305-54130960-HERVHF-pro - 13
54115788 54132866 NONHSAT034024.2 - 752
13 54130113 54130865 Homo_sapiens_13_54129305-54130960-HERVHF-pro - 13
54115788 54132891 NONHSAT034025.2 - 752
13 54130954 54131292 Homo_sapiens_13_54129305-54130960-HERVHF-gag - 13

54115788 54132866 NONHSAT034024.2 - 338
13 54130954 54131292 Homo_sapiens_13_54129305-54130960-HERVHF-gag - 13
54115788 54132891 NONHSAT034025.2 - 338
13 54127417 54127817 Homo_sapiens_13_54129305-54130960-HERVHF-5LTR - 13
54115789 54132867 NONHSAT166813.1 - 400
13 54128407 54130751 Homo_sapiens_13_54129305-54130960-HERVHF-pol - 13
54115789 54132867 NONHSAT166813.1 - 2344
13 54130113 54130865 Homo_sapiens_13_54129305-54130960-HERVHF-pro - 13
54115789 54132867 NONHSAT166813.1 - 752
13 54130954 54131292 Homo_sapiens_13_54129305-54130960-HERVHF-gag - 13
54115789 54132867 NONHSAT166813.1 - 338
13 54127417 54127817 Homo_sapiens_13_54129305-54130960-HERVHF-5LTR - 13
54123609 54132891 NONHSAT233264.1 - 400
13 54128407 54130751 Homo_sapiens_13_54129305-54130960-HERVHF-pol - 13
54123609 54132891 NONHSAT233264.1 - 2344
13 54130113 54130865 Homo_sapiens_13_54129305-54130960-HERVHF-pro - 13
54123609 54132891 NONHSAT233264.1 - 752
13 54130954 54131292 Homo_sapiens_13_54129305-54130960-HERVHF-gag - 13
54123609 54132891 NONHSAT233264.1 - 338
13 54127417 54127817 Homo_sapiens_13_54129305-54130960-HERVHF-5LTR - 13
54124323 54132613 NONHSAT034027.2 - 400
13 54127417 54127817 Homo_sapiens_13_54129305-54130960-HERVHF-5LTR - 13
54124323 54132891 NONHSAT034028.2 - 400
13 54128407 54130751 Homo_sapiens_13_54129305-54130960-HERVHF-pol - 13
54124323 54132613 NONHSAT034027.2 - 2344
13 54128407 54130751 Homo_sapiens_13_54129305-54130960-HERVHF-pol - 13
54124323 54132891 NONHSAT034028.2 - 2344
13 54130113 54130865 Homo_sapiens_13_54129305-54130960-HERVHF-pro - 13
54124323 54132613 NONHSAT034027.2 - 752
13 54130113 54130865 Homo_sapiens_13_54129305-54130960-HERVHF-pro - 13
54124323 54132891 NONHSAT034028.2 - 752
13 54130954 54131292 Homo_sapiens_13_54129305-54130960-HERVHF-gag - 13
54124323 54132613 NONHSAT034027.2 - 338
13 54130954 54131292 Homo_sapiens_13_54129305-54130960-HERVHF-gag - 13
54124323 54132891 NONHSAT034028.2 - 338
13 54127417 54127817 Homo_sapiens_13_54129305-54130960-HERVHF-5LTR - 13
54124324 54132862 ENSG00000234787 - 400
13 54128407 54130751 Homo_sapiens_13_54129305-54130960-HERVHF-pol - 13
54124324 54132862 ENSG00000234787 - 2344
13 54130113 54130865 Homo_sapiens_13_54129305-54130960-HERVHF-pro - 13
54124324 54132862 ENSG00000234787 - 752
13 54130954 54131292 Homo_sapiens_13_54129305-54130960-HERVHF-gag - 13
54124324 54132862 ENSG00000234787 - 338
13 54127417 54127817 Homo_sapiens_13_54129305-54130960-HERVHF-5LTR - 13

54124812 54132891 NONHSAT166815.1 - 400
13 54128407 54130751 Homo_sapiens_13_54129305-54130960-HERVHF-pol - 13
54124812 54132891 NONHSAT166815.1 - 2344
13 54130113 54130865 Homo_sapiens_13_54129305-54130960-HERVHF-pro - 13
54124812 54132891 NONHSAT166815.1 - 752
13 54130954 54131292 Homo_sapiens_13_54129305-54130960-HERVHF-gag - 13
54124812 54132891 NONHSAT166815.1 - 338
13 54127417 54127817 Homo_sapiens_13_54129305-54130960-HERVHF-5LTR - 13
54124813 54132851 ENSG00000234787 - 400
13 54128407 54130751 Homo_sapiens_13_54129305-54130960-HERVHF-pol - 13
54124813 54132851 ENSG00000234787 - 2344
13 54130113 54130865 Homo_sapiens_13_54129305-54130960-HERVHF-pro - 13
54124813 54132851 ENSG00000234787 - 752
13 54130954 54131292 Homo_sapiens_13_54129305-54130960-HERVHF-gag - 13
54124813 54132851 ENSG00000234787 - 338
13 54127417 54127817 Homo_sapiens_13_54129305-54130960-HERVHF-5LTR - 13
54124994 54132891 NONHSAT166816.1 - 400
13 54128407 54130751 Homo_sapiens_13_54129305-54130960-HERVHF-pol - 13
54124994 54132891 NONHSAT166816.1 - 2344
13 54130113 54130865 Homo_sapiens_13_54129305-54130960-HERVHF-pro - 13
54124994 54132891 NONHSAT166816.1 - 752
13 54130954 54131292 Homo_sapiens_13_54129305-54130960-HERVHF-gag - 13
54124994 54132891 NONHSAT166816.1 - 338
13 54127417 54127817 Homo_sapiens_13_54129305-54130960-HERVHF-5LTR - 13
54124995 54132819 ENSG00000234787 - 400
13 54128407 54130751 Homo_sapiens_13_54129305-54130960-HERVHF-pol - 13
54124995 54132819 ENSG00000234787 - 2344
13 54130113 54130865 Homo_sapiens_13_54129305-54130960-HERVHF-pro - 13
54124995 54132819 ENSG00000234787 - 752
13 54130954 54131292 Homo_sapiens_13_54129305-54130960-HERVHF-gag - 13
54124995 54132819 ENSG00000234787 - 338
13 54127417 54127817 Homo_sapiens_13_54129305-54130960-HERVHF-5LTR - 13
54126861 54132891 NONHSAT034029.2 - 400
13 54128407 54130751 Homo_sapiens_13_54129305-54130960-HERVHF-pol - 13
54126861 54132891 NONHSAT034029.2 - 2344
13 54130113 54130865 Homo_sapiens_13_54129305-54130960-HERVHF-pro - 13
54126861 54132891 NONHSAT034029.2 - 752
13 54130954 54131292 Homo_sapiens_13_54129305-54130960-HERVHF-gag - 13
54126861 54132891 NONHSAT034029.2 - 338
13 54127417 54127817 Homo_sapiens_13_54129305-54130960-HERVHF-5LTR - 13
54126862 54132866 ENSG00000234787 - 400
13 54128407 54130751 Homo_sapiens_13_54129305-54130960-HERVHF-pol - 13
54126862 54132866 ENSG00000234787 - 2344
13 54130113 54130865 Homo_sapiens_13_54129305-54130960-HERVHF-pro - 13

54126862 54132866 ENSG00000234787 - 752
13 54130954 54131292 Homo_sapiens_13_54129305-54130960-HERVHF-gag - 13
54126862 54132866 ENSG00000234787 - 338
13 54127417 54133159 Homo_sapiens_13_54129305-54130960-HERVHF - 13 54127361
54142319 ENSG00000234787 - 5742
13 54127417 54127817 Homo_sapiens_13_54129305-54130960-HERVHF-5LTR - 13
54127361 54142319 ENSG00000234787 - 400
13 54128407 54130751 Homo_sapiens_13_54129305-54130960-HERVHF-pol - 13
54127361 54142319 ENSG00000234787 - 2344
13 54130113 54130865 Homo_sapiens_13_54129305-54130960-HERVHF-pro - 13
54127361 54142319 ENSG00000234787 - 752
13 54130954 54131292 Homo_sapiens_13_54129305-54130960-HERVHF-gag - 13
54127361 54142319 ENSG00000234787 - 338
13 54132759 54133159 Homo_sapiens_13_54129305-54130960-HERVHF-3LTR - 13
54127361 54142319 ENSG00000234787 - 400
13 54127417 54133159 Homo_sapiens_13_54129305-54130960-HERVHF - 13 54130894
54132891 NONHSAT233265.1 - 1997
13 54130954 54131292 Homo_sapiens_13_54129305-54130960-HERVHF-gag - 13
54130894 54132891 NONHSAT233265.1 - 338
13 66141327 66147037 Homo_sapiens_13_66143157-66144503-HERVHF - 13 66086287
66205334 NONHSAG067586.1 - 5710
13 66141327 66141765 Homo_sapiens_13_66143157-66144503-HERVHF-5LTR - 13
66086287 66205334 NONHSAG067586.1 - 438
13 66141327 66147037 Homo_sapiens_13_66143157-66144503-HERVHF - 13 66086287
66205334 NONHSAT166892.1 - 5710
13 66141327 66141765 Homo_sapiens_13_66143157-66144503-HERVHF-5LTR - 13
66086287 66205334 NONHSAT166892.1 - 438
13 66142250 66144110 Homo_sapiens_13_66143157-66144503-HERVHF-pol - 13
66086287 66205334 NONHSAG067586.1 - 1860
13 66142250 66144110 Homo_sapiens_13_66143157-66144503-HERVHF-pol - 13
66086287 66205334 NONHSAT166892.1 - 1860
13 66144597 66144917 Homo_sapiens_13_66143157-66144503-HERVHF-gag - 13
66086287 66205334 NONHSAG067586.1 - 320
13 66144597 66144917 Homo_sapiens_13_66143157-66144503-HERVHF-gag - 13
66086287 66205334 NONHSAT166892.1 - 320
13 66146605 66147037 Homo_sapiens_13_66143157-66144503-HERVHF-3LTR - 13
66086287 66205334 NONHSAG067586.1 - 432
13 66146605 66147037 Homo_sapiens_13_66143157-66144503-HERVHF-3LTR - 13
66086287 66205334 NONHSAT166892.1 - 432
13 66142250 66144110 Homo_sapiens_13_66143157-66144503-HERVHF-pol - 13
66141367 66232074 NONHSAG013698.2 - 1860
13 66142250 66144110 Homo_sapiens_13_66143157-66144503-HERVHF-pol - 13
66141367 66232074 NONHSAT034205.2 - 1860
13 66144597 66144917 Homo_sapiens_13_66143157-66144503-HERVHF-gag - 13

66141367 66232074 NONHSAG013698.2 - 320
13 66144597 66144917 Homo_sapiens_13_66143157-66144503-HERVHF-gag - 13
66141367 66232074 NONHSAT034205.2 - 320
13 66146605 66147037 Homo_sapiens_13_66143157-66144503-HERVHF-3LTR - 13
66141367 66232074 NONHSAG013698.2 - 432
13 66146605 66147037 Homo_sapiens_13_66143157-66144503-HERVHF-3LTR - 13
66141367 66232074 NONHSAT034205.2 - 432
13 79276611 79277441 Homo_sapiens_13_79276654-79278001-HERVHF-pro + 13
79276453 79296131 NONHSAG067153.1 + 830
13 79276611 79277441 Homo_sapiens_13_79276654-79278001-HERVHF-pro + 13
79276453 79296131 NONHSAT165987.1 + 830
13 79276860 79278376 Homo_sapiens_13_79276654-79278001-HERVHF-pol + 13
79276453 79296131 NONHSAG067153.1 + 1516
13 79276860 79278376 Homo_sapiens_13_79276654-79278001-HERVHF-pol + 13
79276453 79296131 NONHSAT165987.1 + 1516
13 79279414 79279830 Homo_sapiens_13_79276654-79278001-HERVHF-3LTR + 13
79276453 79296131 NONHSAG067153.1 + 416
13 79279414 79279830 Homo_sapiens_13_79276654-79278001-HERVHF-3LTR + 13
79276453 79296131 NONHSAT165987.1 + 416
14 38193319 38194104 Homo_sapiens_14_38193317-38194783-HERVHF-pro + 14
38190982 38203533 NONHSAG014760.3 + 785
14 38193319 38194104 Homo_sapiens_14_38193317-38194783-HERVHF-pro + 14
38190982 38202923 NONHSAT036484.2 + 785
14 38193319 38194104 Homo_sapiens_14_38193317-38194783-HERVHF-pro + 14
38190982 38203533 NONHSAT233631.1 + 785
14 38193319 38194104 Homo_sapiens_14_38193317-38194783-HERVHF-pro + 14
38190982 38203533 NONHSAT233632.1 + 785
14 38193562 38195486 Homo_sapiens_14_38193317-38194783-HERVHF-pol + 14
38190982 38203533 NONHSAG014760.3 + 1924
14 38193562 38195486 Homo_sapiens_14_38193317-38194783-HERVHF-pol + 14
38190982 38202923 NONHSAT036484.2 + 1924
14 38193562 38195486 Homo_sapiens_14_38193317-38194783-HERVHF-pol + 14
38190982 38203533 NONHSAT233631.1 + 1924
14 38193562 38195486 Homo_sapiens_14_38193317-38194783-HERVHF-pol + 14
38190982 38203533 NONHSAT233632.1 + 1924
14 38196077 38196529 Homo_sapiens_14_38193317-38194783-HERVHF-3LTR + 14
38190982 38203533 NONHSAG014760.3 + 452
14 38196077 38196529 Homo_sapiens_14_38193317-38194783-HERVHF-3LTR + 14
38190982 38202923 NONHSAT036484.2 + 452
14 38196077 38196529 Homo_sapiens_14_38193317-38194783-HERVHF-3LTR + 14
38190982 38203533 NONHSAT233631.1 + 452
14 38196077 38196529 Homo_sapiens_14_38193317-38194783-HERVHF-3LTR + 14
38190982 38203533 NONHSAT233632.1 + 452
14 38193319 38194104 Homo_sapiens_14_38193317-38194783-HERVHF-pro + 14

38190983 38202923 ENSG00000258649 + 785
14 38193562 38195486 Homo_sapiens_14_38193317-38194783-HERVHF-pol + 14
38190983 38202923 ENSG00000258649 + 1924
14 38196077 38196529 Homo_sapiens_14_38193317-38194783-HERVHF-3LTR + 14
38190983 38202923 ENSG00000258649 + 452
14 38193319 38194104 Homo_sapiens_14_38193317-38194783-HERVHF-pro + 14
38193207 38203526 NONHSAT036485.2 + 785
14 38193562 38195486 Homo_sapiens_14_38193317-38194783-HERVHF-pol + 14
38193207 38203526 NONHSAT036485.2 + 1924
14 38196077 38196529 Homo_sapiens_14_38193317-38194783-HERVHF-3LTR + 14
38193207 38203526 NONHSAT036485.2 + 452
14 41521426 41521883 Homo_sapiens_14_41518469-41520184-HERVHF-3LTR + 14
41520522 41526746 NONHSAG014802.2 + 457
14 41521426 41521883 Homo_sapiens_14_41518469-41520184-HERVHF-3LTR + 14
41520522 41526746 NONHSAT036571.2 + 457
14 48255191 48263146 Homo_sapiens_14_48256895-48258584-HERVHF - 14 48227412
48266912 NONHSAG014863.3 - 7955
14 48255191 48255959 Homo_sapiens_14_48256895-48258584-HERVHF-5LTR - 14
48227412 48266912 NONHSAG014863.3 - 768
14 48255191 48255959 Homo_sapiens_14_48256895-48258584-HERVHF-5LTR - 14
48227412 48260643 NONHSAT234204.1 - 768
14 48255191 48255959 Homo_sapiens_14_48256895-48258584-HERVHF-5LTR - 14
48227412 48260643 NONHSAT234205.1 - 768
14 48256057 48258390 Homo_sapiens_14_48256895-48258584-HERVHF-pol - 14
48227412 48266912 NONHSAG014863.3 - 2333
14 48256057 48258390 Homo_sapiens_14_48256895-48258584-HERVHF-pol - 14
48227412 48260643 NONHSAT234204.1 - 2333
14 48256057 48258390 Homo_sapiens_14_48256895-48258584-HERVHF-pol - 14
48227412 48260643 NONHSAT234205.1 - 2333
14 48257788 48258639 Homo_sapiens_14_48256895-48258584-HERVHF-pro - 14
48227412 48266912 NONHSAG014863.3 - 851
14 48257788 48258639 Homo_sapiens_14_48256895-48258584-HERVHF-pro - 14
48227412 48260643 NONHSAT234204.1 - 851
14 48257788 48258639 Homo_sapiens_14_48256895-48258584-HERVHF-pro - 14
48227412 48260643 NONHSAT234205.1 - 851
14 48262389 48263146 Homo_sapiens_14_48256895-48258584-HERVHF-3LTR - 14
48227412 48266912 NONHSAG014863.3 - 757
14 48255191 48263146 Homo_sapiens_14_48256895-48258584-HERVHF - 14 48233461
48266912 NONHSAT036697.2 - 7955
14 48255191 48255959 Homo_sapiens_14_48256895-48258584-HERVHF-5LTR - 14
48233461 48266912 NONHSAT036697.2 - 768
14 48256057 48258390 Homo_sapiens_14_48256895-48258584-HERVHF-pol - 14
48233461 48266912 NONHSAT036697.2 - 2333
14 48257788 48258639 Homo_sapiens_14_48256895-48258584-HERVHF-pro - 14

48233461 48266912 NONHSAT036697.2 - 851
14 48262389 48263146 Homo_sapiens_14_48256895-48258584-HERVHF-3LTR - 14
48233461 48266912 NONHSAT036697.2 - 757
14 48255191 48263146 Homo_sapiens_14_48256895-48258584-HERVHF - 14 48255676
48260643 NONHSAT234206.1 - 4967
14 48256057 48258390 Homo_sapiens_14_48256895-48258584-HERVHF-pol - 14
48255676 48260643 NONHSAT234206.1 - 2333
14 48257788 48258639 Homo_sapiens_14_48256895-48258584-HERVHF-pro - 14
48255676 48260643 NONHSAT234206.1 - 851
14 48262389 48263146 Homo_sapiens_14_48256895-48258584-HERVHF-3LTR - 14
48262021 48265609 ENSG00000287492 - 757
15 74354141 74359786 Homo_sapiens_15_74355867-74357936-HERVHF + 15 74350767
74364620 NONHSAG017411.2 + 5645
15 74354141 74354539 Homo_sapiens_15_74355867-74357936-HERVHF-5LTR + 15
74350767 74364620 NONHSAG017411.2 + 398
15 74354141 74359786 Homo_sapiens_15_74355867-74357936-HERVHF + 15 74350767
74361903 NONHSAT047227.2 + 5645
15 74354141 74354539 Homo_sapiens_15_74355867-74357936-HERVHF-5LTR + 15
74350767 74361903 NONHSAT047227.2 + 398
15 74355965 74356405 Homo_sapiens_15_74355867-74357936-HERVHF-gag + 15
74350767 74364620 NONHSAG017411.2 + 440
15 74355965 74356405 Homo_sapiens_15_74355867-74357936-HERVHF-gag + 15
74350767 74361903 NONHSAT047227.2 + 440
15 74356488 74357237 Homo_sapiens_15_74355867-74357936-HERVHF-pro + 15
74350767 74364620 NONHSAG017411.2 + 749
15 74356488 74357237 Homo_sapiens_15_74355867-74357936-HERVHF-pro + 15
74350767 74361903 NONHSAT047227.2 + 749
15 74356599 74358842 Homo_sapiens_15_74355867-74357936-HERVHF-pol + 15
74350767 74364620 NONHSAG017411.2 + 2243
15 74356599 74358842 Homo_sapiens_15_74355867-74357936-HERVHF-pol + 15
74350767 74361903 NONHSAT047227.2 + 2243
15 74359383 74359786 Homo_sapiens_15_74355867-74357936-HERVHF-3LTR + 15
74350767 74364620 NONHSAG017411.2 + 403
15 74359383 74359786 Homo_sapiens_15_74355867-74357936-HERVHF-3LTR + 15
74350767 74361903 NONHSAT047227.2 + 403
15 74354141 74359786 Homo_sapiens_15_74355867-74357936-HERVHF + 15 74350768
74361903 ENSG00000260266 + 5645
15 74354141 74354539 Homo_sapiens_15_74355867-74357936-HERVHF-5LTR + 15
74350768 74361903 ENSG00000260266 + 398
15 74355965 74356405 Homo_sapiens_15_74355867-74357936-HERVHF-gag + 15
74350768 74361903 ENSG00000260266 + 440
15 74356488 74357237 Homo_sapiens_15_74355867-74357936-HERVHF-pro + 15
74350768 74361903 ENSG00000260266 + 749
15 74356599 74358842 Homo_sapiens_15_74355867-74357936-HERVHF-pol + 15

74350768 74361903 ENSG00000260266 + 2243
15 74359383 74359786 Homo_sapiens_15_74355867-74357936-HERVHF-3LTR + 15
74350768 74361903 ENSG00000260266 + 403
15 74355965 74356405 Homo_sapiens_15_74355867-74357936-HERVHF-gag + 15
74354455 74364620 NONHSAT047228.2 + 440
15 74356488 74357237 Homo_sapiens_15_74355867-74357936-HERVHF-pro + 15
74354455 74364620 NONHSAT047228.2 + 749
15 74356599 74358842 Homo_sapiens_15_74355867-74357936-HERVHF-pol + 15
74354455 74364620 NONHSAT047228.2 + 2243
15 74359383 74359786 Homo_sapiens_15_74355867-74357936-HERVHF-3LTR + 15
74354455 74364620 NONHSAT047228.2 + 403
15 74355965 74356405 Homo_sapiens_15_74355867-74357936-HERVHF-gag + 15
74354456 74364620 ENSG00000260266 + 440
15 74356488 74357237 Homo_sapiens_15_74355867-74357936-HERVHF-pro + 15
74354456 74364620 ENSG00000260266 + 749
15 74356599 74358842 Homo_sapiens_15_74355867-74357936-HERVHF-pol + 15
74354456 74364620 ENSG00000260266 + 2243
15 74359383 74359786 Homo_sapiens_15_74355867-74357936-HERVHF-3LTR + 15
74354456 74364620 ENSG00000260266 + 403
15 87831107 87837024 Homo_sapiens_15_87833731-87835137-HERVHF + 15 87835202
87836988 NONHSAG017784.2 + 1786
15 87831107 87837024 Homo_sapiens_15_87833731-87835137-HERVHF + 15 87835202
87836988 NONHSAT048152.2 + 1786
15 87831107 87837024 Homo_sapiens_15_87833731-87835137-HERVHF + 15 87835202
87836151 NONHSAT171143.1+ 949
16 60078536 60084582 Homo_sapiens_16_60081354-60082700-HERVHF + 16 60066531
60084753 NONHSAG071739.1 + 6046
16 60078536 60078989 Homo_sapiens_16_60081354-60082700-HERVHF-5LTR + 16
60066531 60084753 NONHSAG071739.1 + 453
16 60078536 60084582 Homo_sapiens_16_60081354-60082700-HERVHF + 16 60066531
60084753 NONHSAT173129.1 + 6046
16 60078536 60078989 Homo_sapiens_16_60081354-60082700-HERVHF-5LTR + 16
60066531 60084753 NONHSAT173129.1 + 453
16 60078536 60084582 Homo_sapiens_16_60081354-60082700-HERVHF + 16 60066531
60084753 NONHSAT173130.1 + 6046
16 60078536 60078989 Homo_sapiens_16_60081354-60082700-HERVHF-5LTR + 16
60066531 60084753 NONHSAT173130.1 + 453
16 60080893 60081216 Homo_sapiens_16_60081354-60082700-HERVHF-gag + 16
60066531 60084753 NONHSAG071739.1 + 323
16 60080893 60081216 Homo_sapiens_16_60081354-60082700-HERVHF-gag + 16
60066531 60084753 NONHSAT173129.1 + 323
16 60080893 60081216 Homo_sapiens_16_60081354-60082700-HERVHF-gag + 16
60066531 60084753 NONHSAT173130.1 + 323
16 60081323 60082141 Homo_sapiens_16_60081354-60082700-HERVHF-pro + 16

60066531 60084753 NONHSAG071739.1 + 818
16 60081323 60082141 Homo_sapiens_16_60081354-60082700-HERVHF-pro + 16
60066531 60084753 NONHSAT173129.1 + 818
16 60081323 60082141 Homo_sapiens_16_60081354-60082700-HERVHF-pro + 16
60066531 60084753 NONHSAT173130.1 + 818
16 60081599 60083447 Homo_sapiens_16_60081354-60082700-HERVHF-pol + 16
60066531 60084753 NONHSAG071739.1 + 1848
16 60081599 60083447 Homo_sapiens_16_60081354-60082700-HERVHF-pol + 16
60066531 60084753 NONHSAT173129.1 + 1848
16 60081599 60083447 Homo_sapiens_16_60081354-60082700-HERVHF-pol + 16
60066531 60084753 NONHSAT173130.1 + 1848
16 60084128 60084582 Homo_sapiens_16_60081354-60082700-HERVHF-3LTR + 16
60066531 60084753 NONHSAG071739.1 + 454
16 60084128 60084582 Homo_sapiens_16_60081354-60082700-HERVHF-3LTR + 16
60066531 60084753 NONHSAT173129.1 + 454
16 60084128 60084582 Homo_sapiens_16_60081354-60082700-HERVHF-3LTR + 16
60066531 60084753 NONHSAT173130.1 + 454
16 60078536 60078989 Homo_sapiens_16_60081354-60082700-HERVHF-5LTR + 16
60068880 60083061 NONHSAT173131.1 + 453
16 60080893 60081216 Homo_sapiens_16_60081354-60082700-HERVHF-gag + 16
60068880 60083061 NONHSAT173131.1 + 323
16 60081323 60082141 Homo_sapiens_16_60081354-60082700-HERVHF-pro + 16
60068880 60083061 NONHSAT173131.1 + 818
16 65229803 65235350 Homo_sapiens_16_65231504-65233039-HERVHF - 16 65190972
65276355 NONHSAG019666.2 - 5547
16 65229803 65230258 Homo_sapiens_16_65231504-65233039-HERVHF-5LTR - 16
65190972 65276355 NONHSAG019666.2 - 455
16 65229803 65230258 Homo_sapiens_16_65231504-65233039-HERVHF-5LTR - 16
65190972 65234914 NONHSAT142973.2 - 455
16 65231100 65232797 Homo_sapiens_16_65231504-65233039-HERVHF-pol - 16
65190972 65276355 NONHSAG019666.2 - 1697
16 65231100 65232797 Homo_sapiens_16_65231504-65233039-HERVHF-pol - 16
65190972 65234914 NONHSAT142973.2 - 1697
16 65232255 65233088 Homo_sapiens_16_65231504-65233039-HERVHF-pro - 16
65190972 65276355 NONHSAG019666.2 - 833
16 65232255 65233088 Homo_sapiens_16_65231504-65233039-HERVHF-pro - 16
65190972 65234914 NONHSAT142973.2 - 833
16 65233092 65233421 Homo_sapiens_16_65231504-65233039-HERVHF-gag - 16
65190972 65276355 NONHSAG019666.2 - 329
16 65233092 65233421 Homo_sapiens_16_65231504-65233039-HERVHF-gag - 16
65190972 65234914 NONHSAT142973.2 - 329
16 65234898 65235350 Homo_sapiens_16_65231504-65233039-HERVHF-3LTR - 16
65190972 65276355 NONHSAG019666.2 - 452
16 65229803 65230258 Homo_sapiens_16_65231504-65233039-HERVHF-5LTR - 16

65190973 65234914 ENSG00000260834 - 455
16 65231100 65232797 Homo_sapiens_16_65231504-65233039-HERVHF-pol - 16
65190973 65234914 ENSG00000260834 - 1697
16 65232255 65233088 Homo_sapiens_16_65231504-65233039-HERVHF-pro - 16
65190973 65234914 ENSG00000260834 - 833
16 65233092 65233421 Homo_sapiens_16_65231504-65233039-HERVHF-gag - 16
65190973 65234914 ENSG00000260834 - 329
16 65229803 65235350 Homo_sapiens_16_65231504-65233039-HERVHF - 16 65227786
65276355 NONHSAT142974.2 - 5547
16 65229803 65230258 Homo_sapiens_16_65231504-65233039-HERVHF-5LTR - 16
65227786 65276355 NONHSAT142974.2 - 455
16 65231100 65232797 Homo_sapiens_16_65231504-65233039-HERVHF-pol - 16
65227786 65276355 NONHSAT142974.2 - 1697
16 65232255 65233088 Homo_sapiens_16_65231504-65233039-HERVHF-pro - 16
65227786 65276355 NONHSAT142974.2 - 833
16 65233092 65233421 Homo_sapiens_16_65231504-65233039-HERVHF-gag - 16
65227786 65276355 NONHSAT142974.2 - 329
16 65234898 65235350 Homo_sapiens_16_65231504-65233039-HERVHF-3LTR - 16
65227786 65276355 NONHSAT142974.2 - 452
16 65229803 65230258 Homo_sapiens_16_65231504-65233039-HERVHF-5LTR - 16
65227895 65234904 ENSG00000260834 - 455
16 65231100 65232797 Homo_sapiens_16_65231504-65233039-HERVHF-pol - 16
65227895 65234904 ENSG00000260834 - 1697
16 65232255 65233088 Homo_sapiens_16_65231504-65233039-HERVHF-pro - 16
65227895 65234904 ENSG00000260834 - 833
16 65233092 65233421 Homo_sapiens_16_65231504-65233039-HERVHF-gag - 16
65227895 65234904 ENSG00000260834 - 329
18 28693028 28693465 Homo_sapiens_18_28694974-28696314-HERVHF-5LTR - 18
28688304 28696511 NONHSAG075074.1 - 437
18 28693028 28693465 Homo_sapiens_18_28694974-28696314-HERVHF-5LTR - 18
28688304 28696511 NONHSAT178313.1 - 437
18 28694048 28696068 Homo_sapiens_18_28694974-28696314-HERVHF-pol - 18
28688304 28696511 NONHSAG075074.1 - 2020
18 28694048 28696068 Homo_sapiens_18_28694974-28696314-HERVHF-pol - 18
28688304 28696511 NONHSAT178313.1 - 2020
18 56417745 56418068 Homo_sapiens_18_56418118-56419466-HERVHF-gag + 18
56415837 56425475 NONHSAG074828.1 + 323
18 56417745 56418068 Homo_sapiens_18_56418118-56419466-HERVHF-gag + 18
56415837 56425475 NONHSAT177849.1 + 323
18 56418362 56420489 Homo_sapiens_18_56418118-56419466-HERVHF-pol + 18
56415837 56425475 NONHSAG074828.1 + 2127
18 56418362 56420489 Homo_sapiens_18_56418118-56419466-HERVHF-pol + 18
56415837 56425475 NONHSAT177849.1 + 2127
18 56420893 56421344 Homo_sapiens_18_56418118-56419466-HERVHF-3LTR + 18

56415837 56425475 NONHSAG074828.1 + 451
18 56420893 56421344 Homo_sapiens_18_56418118-56419466-HERVHF-3LTR + 18
56415837 56425475 NONHSAT177849.1 + 451
18 57064647 57065099 Homo_sapiens_18_57068491-57069834-HERVHF-5LTR - 18
57054558 57072119 ENSG00000258609 - 452
18 57064647 57065099 Homo_sapiens_18_57068491-57069834-HERVHF-5LTR - 18
57054558 57072119 NONHSAG023930.2 - 452
18 57064647 57065099 Homo_sapiens_18_57068491-57069834-HERVHF-5LTR - 18
57054558 57072119 NONHSAT059436.2 - 452
18 57065267 57065866 Homo_sapiens_18_57068491-57069834-HERVHF-env - 18
57054558 57072119 ENSG00000258609 - 599
18 57065267 57065866 Homo_sapiens_18_57068491-57069834-HERVHF-env - 18
57054558 57072119 NONHSAG023930.2 - 599
18 57065267 57065866 Homo_sapiens_18_57068491-57069834-HERVHF-env - 18
57054558 57072119 NONHSAT059436.2 - 599
18 57067308 57069458 Homo_sapiens_18_57068491-57069834-HERVHF-pol - 18
57054558 57072119 ENSG00000258609 - 2150
18 57067308 57069458 Homo_sapiens_18_57068491-57069834-HERVHF-pol - 18
57054558 57072119 NONHSAG023930.2 - 2150
18 57067308 57069458 Homo_sapiens_18_57068491-57069834-HERVHF-pol - 18
57054558 57072119 NONHSAT059436.2 - 2150
18 57069033 57069632 Homo_sapiens_18_57068491-57069834-HERVHF-pro - 18
57054558 57072119 ENSG00000258609 - 599
18 57069033 57069632 Homo_sapiens_18_57068491-57069834-HERVHF-pro - 18
57054558 57072119 NONHSAG023930.2 - 599
18 57069033 57069632 Homo_sapiens_18_57068491-57069834-HERVHF-pro - 18
57054558 57072119 NONHSAT059436.2 - 599
18 57069916 57070296 Homo_sapiens_18_57068491-57069834-HERVHF-gag - 18
57054558 57072119 ENSG00000258609 - 380
18 57069916 57070296 Homo_sapiens_18_57068491-57069834-HERVHF-gag - 18
57054558 57072119 NONHSAG023930.2 - 380
18 57069916 57070296 Homo_sapiens_18_57068491-57069834-HERVHF-gag - 18
57054558 57072119 NONHSAT059436.2 - 380
18 57064647 57065099 Homo_sapiens_18_57068491-57069834-HERVHF-5LTR - 18
57054591 57072059 ENSG00000258609 - 452
18 57064647 57065099 Homo_sapiens_18_57068491-57069834-HERVHF-5LTR - 18
57054591 57072059 ENSG00000258609 - 452
18 57065267 57065866 Homo_sapiens_18_57068491-57069834-HERVHF-env - 18
57054591 57072059 ENSG00000258609 - 599
18 57065267 57065866 Homo_sapiens_18_57068491-57069834-HERVHF-env - 18
57054591 57072059 ENSG00000258609 - 599
18 57067308 57069458 Homo_sapiens_18_57068491-57069834-HERVHF-pol - 18
57054591 57072059 ENSG00000258609 - 2150
18 57067308 57069458 Homo_sapiens_18_57068491-57069834-HERVHF-pol - 18

57054591 57072059 ENSG00000258609 - 2150
18 57069033 57069632 Homo_sapiens_18_57068491-57069834-HERVHF-pro - 18
57054591 57072059 ENSG00000258609 - 599
18 57069033 57069632 Homo_sapiens_18_57068491-57069834-HERVHF-pro - 18
57054591 57072059 ENSG00000258609 - 599
18 57069916 57070296 Homo_sapiens_18_57068491-57069834-HERVHF-gag - 18
57054591 57072059 ENSG00000258609 - 380
18 57069916 57070296 Homo_sapiens_18_57068491-57069834-HERVHF-gag - 18
57054591 57072059 ENSG00000258609 - 380
18 57064647 57065099 Homo_sapiens_18_57068491-57069834-HERVHF-5LTR - 18
57057888 57070003 ENSG00000258609 - 452
18 57064647 57065099 Homo_sapiens_18_57068491-57069834-HERVHF-5LTR - 18
57057888 57070003 ENSG00000258609 - 452
18 57064647 57065099 Homo_sapiens_18_57068491-57069834-HERVHF-5LTR - 18
57057888 57072050 ENSG00000258609 - 452
18 57064647 57065099 Homo_sapiens_18_57068491-57069834-HERVHF-5LTR - 18
57057888 57070003 ENSG00000258609 - 452
18 57065267 57065866 Homo_sapiens_18_57068491-57069834-HERVHF-env - 18
57057888 57070003 ENSG00000258609 - 599
18 57065267 57065866 Homo_sapiens_18_57068491-57069834-HERVHF-env - 18
57057888 57070003 ENSG00000258609 - 599
18 57065267 57065866 Homo_sapiens_18_57068491-57069834-HERVHF-env - 18
57057888 57072050 ENSG00000258609 - 599
18 57065267 57065866 Homo_sapiens_18_57068491-57069834-HERVHF-env - 18
57057888 57070003 ENSG00000258609 - 599
18 57067308 57069458 Homo_sapiens_18_57068491-57069834-HERVHF-pol - 18
57057888 57070003 ENSG00000258609 - 2150
18 57067308 57069458 Homo_sapiens_18_57068491-57069834-HERVHF-pol - 18
57057888 57070003 ENSG00000258609 - 2150
18 57067308 57069458 Homo_sapiens_18_57068491-57069834-HERVHF-pol - 18
57057888 57072050 ENSG00000258609 - 2150
18 57067308 57069458 Homo_sapiens_18_57068491-57069834-HERVHF-pol - 18
57057888 57070003 ENSG00000258609 - 2150
18 57069033 57069632 Homo_sapiens_18_57068491-57069834-HERVHF-pro - 18
57057888 57070003 ENSG00000258609 - 599
18 57069033 57069632 Homo_sapiens_18_57068491-57069834-HERVHF-pro - 18
57057888 57070003 ENSG00000258609 - 599
18 57069033 57069632 Homo_sapiens_18_57068491-57069834-HERVHF-pro - 18
57057888 57072050 ENSG00000258609 - 599
18 57069033 57069632 Homo_sapiens_18_57068491-57069834-HERVHF-pro - 18
57057888 57070003 ENSG00000258609 - 599
18 57069916 57070296 Homo_sapiens_18_57068491-57069834-HERVHF-gag - 18
57057888 57072050 ENSG00000258609 - 380
18 57064647 57065099 Homo_sapiens_18_57068491-57069834-HERVHF-5LTR - 18

57063555 57072119 ENSG00000258609 - 452
18 57065267 57065866 Homo_sapiens_18_57068491-57069834-HERVHF-env - 18
57063555 57072119 ENSG00000258609 - 599
18 57067308 57069458 Homo_sapiens_18_57068491-57069834-HERVHF-pol - 18
57063555 57072119 ENSG00000258609 - 2150
18 57069033 57069632 Homo_sapiens_18_57068491-57069834-HERVHF-pro - 18
57063555 57072119 ENSG00000258609 - 599
18 57069916 57070296 Homo_sapiens_18_57068491-57069834-HERVHF-gag - 18
57063555 57072119 ENSG00000258609 - 380
18 73327171 73327950 Homo_sapiens_18_73327166-73328509-HERVHF-pro + 18
73324940 73354705 NONHSAG024146.2 + 779
18 73327171 73327950 Homo_sapiens_18_73327166-73328509-HERVHF-pro + 18
73324940 73349889 NONHSAT059835.2 + 779
18 73327408 73329488 Homo_sapiens_18_73327166-73328509-HERVHF-pol + 18
73324940 73354705 NONHSAG024146.2 + 2080
18 73327408 73329488 Homo_sapiens_18_73327166-73328509-HERVHF-pol + 18
73324940 73349889 NONHSAT059835.2 + 2080
18 73329931 73330369 Homo_sapiens_18_73327166-73328509-HERVHF-3LTR + 18
73324940 73354705 NONHSAG024146.2 + 438
18 73329931 73330369 Homo_sapiens_18_73327166-73328509-HERVHF-3LTR + 18
73324940 73349889 NONHSAT059835.2 + 438
18 73327171 73327950 Homo_sapiens_18_73327166-73328509-HERVHF-pro + 18
73324941 73349879 ENSG00000261780 + 779
18 73327171 73327950 Homo_sapiens_18_73327166-73328509-HERVHF-pro + 18
73324941 73349877 ENSG00000261780 + 779
18 73327171 73327950 Homo_sapiens_18_73327166-73328509-HERVHF-pro + 18
73324941 73349878 ENSG00000261780 + 779
18 73327171 73327950 Homo_sapiens_18_73327166-73328509-HERVHF-pro + 18
73324941 73349879 NONHSAT177989.1 + 779
18 73327408 73329488 Homo_sapiens_18_73327166-73328509-HERVHF-pol + 18
73324941 73349879 ENSG00000261780 + 2080
18 73327408 73329488 Homo_sapiens_18_73327166-73328509-HERVHF-pol + 18
73324941 73349877 ENSG00000261780 + 2080
18 73327408 73329488 Homo_sapiens_18_73327166-73328509-HERVHF-pol + 18
73324941 73349878 ENSG00000261780 + 2080
18 73327408 73329488 Homo_sapiens_18_73327166-73328509-HERVHF-pol + 18
73324941 73349879 NONHSAT177989.1 + 2080
18 73329931 73330369 Homo_sapiens_18_73327166-73328509-HERVHF-3LTR + 18
73324941 73349879 ENSG00000261780 + 438
18 73329931 73330369 Homo_sapiens_18_73327166-73328509-HERVHF-3LTR + 18
73324941 73349877 ENSG00000261780 + 438
18 73329931 73330369 Homo_sapiens_18_73327166-73328509-HERVHF-3LTR + 18
73324941 73349878 ENSG00000261780 + 438
18 73329931 73330369 Homo_sapiens_18_73327166-73328509-HERVHF-3LTR + 18

73324941 73349879 NONHSAT177989.1 + 438
18 73327171 73327950 Homo_sapiens_18_73327166-73328509-HERVHF-pro + 18
73324977 73349879 ENSG00000261780 + 779
18 73327408 73329488 Homo_sapiens_18_73327166-73328509-HERVHF-pol + 18
73324977 73349879 ENSG00000261780 + 2080
18 73329931 73330369 Homo_sapiens_18_73327166-73328509-HERVHF-3LTR + 18
73324977 73349879 ENSG00000261780 + 438
18 73327171 73327950 Homo_sapiens_18_73327166-73328509-HERVHF-pro + 18
73325110 73349878 ENSG00000261780 + 779
18 73327408 73329488 Homo_sapiens_18_73327166-73328509-HERVHF-pol + 18
73325110 73349878 ENSG00000261780 + 2080
18 73329931 73330369 Homo_sapiens_18_73327166-73328509-HERVHF-3LTR + 18
73325110 73349878 ENSG00000261780 + 438
18 73327171 73327950 Homo_sapiens_18_73327166-73328509-HERVHF-pro + 18
73326661 73349879 ENSG00000261780 + 779
18 73327408 73329488 Homo_sapiens_18_73327166-73328509-HERVHF-pol + 18
73326661 73349879 ENSG00000261780 + 2080
18 73329931 73330369 Homo_sapiens_18_73327166-73328509-HERVHF-3LTR + 18
73326661 73349879 ENSG00000261780 + 438
18 73327171 73327950 Homo_sapiens_18_73327166-73328509-HERVHF-pro + 18
73326740 73349363 NONHSAT059836.2 + 779
18 73327408 73329488 Homo_sapiens_18_73327166-73328509-HERVHF-pol + 18
73326740 73349363 NONHSAT059836.2 + 2080
18 73329931 73330369 Homo_sapiens_18_73327166-73328509-HERVHF-3LTR + 18
73326740 73349363 NONHSAT059836.2 + 438
19 22568269 22575022 Homo_sapiens_19_22570768-22572352-HERVHF + 19 22368572
22699685 NONHSAG025320.2 + 6753
19 22568269 22568753 Homo_sapiens_19_22570768-22572352-HERVHF-5LTR + 19
22368572 22699685 NONHSAG025320.2 + 484
19 22568269 22575022 Homo_sapiens_19_22570768-22572352-HERVHF + 19 22368572
22699682 NONHSAT179569.1 + 6753
19 22568269 22568753 Homo_sapiens_19_22570768-22572352-HERVHF-5LTR + 19
22368572 22699682 NONHSAT179569.1 + 484
19 22571012 22573853 Homo_sapiens_19_22570768-22572352-HERVHF-pol + 19
22368572 22699685 NONHSAG025320.2 + 2841
19 22571012 22573853 Homo_sapiens_19_22570768-22572352-HERVHF-pol + 19
22368572 22699682 NONHSAT179569.1 + 2841
19 22574536 22575022 Homo_sapiens_19_22570768-22572352-HERVHF-3LTR + 19
22368572 22699685 NONHSAG025320.2 + 486
19 22574536 22575022 Homo_sapiens_19_22570768-22572352-HERVHF-3LTR + 19
22368572 22699682 NONHSAT179569.1 + 486
19 22568269 22575022 Homo_sapiens_19_22570768-22572352-HERVHF + 19 22570138
22574983 NONHSAG076047.1 + 4845
19 22568269 22575022 Homo_sapiens_19_22570768-22572352-HERVHF + 19 22570138

22574983 NONHSAT179572.1 + 4845
19 22571012 22573853 Homo_sapiens_19_22570768-22572352-HERVHF-pol + 19
22570138 22574983 NONHSAG076047.1 + 2841
19 22571012 22573853 Homo_sapiens_19_22570768-22572352-HERVHF-pol + 19
22570138 22574983 NONHSAT179572.1 + 2841
20 12756027 12756350 Homo_sapiens_20_12756400-12757916-HERVHF-gag + 20
12754218 12764939 NONHSAG031288.2 + 323
20 12756027 12756350 Homo_sapiens_20_12756400-12757916-HERVHF-gag + 20
12754218 12764881 NONHSAT078656.2 + 323
20 12756779 12758770 Homo_sapiens_20_12756400-12757916-HERVHF-pol + 20
12754218 12764939 NONHSAG031288.2 + 1991
20 12756779 12758770 Homo_sapiens_20_12756400-12757916-HERVHF-pol + 20
12754218 12764881 NONHSAT078656.2 + 1991
20 12759162 12759632 Homo_sapiens_20_12756400-12757916-HERVHF-3LTR + 20
12754218 12764939 NONHSAG031288.2 + 470
20 12759162 12759632 Homo_sapiens_20_12756400-12757916-HERVHF-3LTR + 20
12754218 12764881 NONHSAT078656.2 + 470
20 40269047 40274769 Homo_sapiens_20_40271576-40272881-HERVHF + 20 40268842
40282584 NONHSAG081519.1 + 5722
20 40269047 40269508 Homo_sapiens_20_40271576-40272881-HERVHF-5LTR + 20
40268842 40282584 NONHSAG081519.1 + 461
20 40269047 40274769 Homo_sapiens_20_40271576-40272881-HERVHF + 20 40268842
40282584 NONHSAT188741.1 + 5722
20 40269047 40269508 Homo_sapiens_20_40271576-40272881-HERVHF-5LTR + 20
40268842 40282584 NONHSAT188741.1 + 461
20 40271560 40272339 Homo_sapiens_20_40271576-40272881-HERVHF-pro + 20
40268842 40282584 NONHSAG081519.1 + 779
20 40271560 40272339 Homo_sapiens_20_40271576-40272881-HERVHF-pro + 20
40268842 40282584 NONHSAT188741.1 + 779
20 40271758 40273720 Homo_sapiens_20_40271576-40272881-HERVHF-pol + 20
40268842 40282584 NONHSAG081519.1 + 1962
20 40271758 40273720 Homo_sapiens_20_40271576-40272881-HERVHF-pol + 20
40268842 40282584 NONHSAT188741.1 + 1962
20 40274312 40274769 Homo_sapiens_20_40271576-40272881-HERVHF-3LTR + 20
40268842 40282584 NONHSAG081519.1 + 457
20 40274312 40274769 Homo_sapiens_20_40271576-40272881-HERVHF-3LTR + 20
40268842 40282584 NONHSAT188741.1 + 457
21 17124024 17124968 Homo_sapiens_21_17123959-17125734-HERVHF-pro + 21
17123654 17129309 NONHSAG110806.1 + 944
21 17124024 17124968 Homo_sapiens_21_17123959-17125734-HERVHF-pro + 21
17123654 17129309 NONHSAT244149.1 + 944
21 17124222 17126293 Homo_sapiens_21_17123959-17125734-HERVHF-pol + 21
17123654 17129309 NONHSAG110806.1 + 2071
21 17124222 17126293 Homo_sapiens_21_17123959-17125734-HERVHF-pol + 21

17123654 17129309 NONHSAT244149.1 + 2071
21 17127325 17127764 Homo_sapiens_21_17123959-17125734-HERVHF-3LTR + 21
17123654 17129309 NONHSAG110806.1 + 439
21 17127325 17127764 Homo_sapiens_21_17123959-17125734-HERVHF-3LTR + 21
17123654 17129309 NONHSAT244149.1 + 439
21 26227947 26233485 Homo_sapiens_21_26229594-26231104-HERVHF - 21 26228288
26231349 NONHSAG032575.2 - 3061
21 26227947 26233485 Homo_sapiens_21_26229594-26231104-HERVHF - 21 26228288
26231349 NONHSAT081534.2 - 3061
21 26228683 26231015 Homo_sapiens_21_26229594-26231104-HERVHF-pol - 21
26228288 26231349 NONHSAG032575.2 - 2332
21 26228683 26231015 Homo_sapiens_21_26229594-26231104-HERVHF-pol - 21
26228288 26231349 NONHSAT081534.2 - 2332
21 26230473 26231054 Homo_sapiens_21_26229594-26231104-HERVHF-pro - 21
26228288 26231349 NONHSAG032575.2 - 581
21 26230473 26231054 Homo_sapiens_21_26229594-26231104-HERVHF-pro - 21
26228288 26231349 NONHSAT081534.2 - 581
21 42800845 42801294 Homo_sapiens_21_42802518-42804296-HERVHF-5LTR - 21
42784977 42806191 NONHSAG083198.1 - 449
21 42801909 42803999 Homo_sapiens_21_42802518-42804296-HERVHF-pol - 21
42784977 42806191 NONHSAG083198.1 - 2090
21 42800845 42801294 Homo_sapiens_21_42802518-42804296-HERVHF-5LTR - 21
42785883 42806191 NONHSAT191544.1 - 449
21 42801909 42803999 Homo_sapiens_21_42802518-42804296-HERVHF-pol - 21
42785883 42806191 NONHSAT191544.1 - 2090
X 71264372 71272628 Homo_sapiens_X_71266645-71268493-HERVHF + X 71254814
71301168 ENSG00000147140 + 8256
X 71264372 71264714 Homo_sapiens_X_71266645-71268493-HERVHF-5LTR + X
71254814 71301168 ENSG00000147140 + 342
X 71266244 71266657 Homo_sapiens_X_71266645-71268493-HERVHF-gag + X
71254814 71301168 ENSG00000147140 + 413
X 71266704 71267651 Homo_sapiens_X_71266645-71268493-HERVHF-pro + X
71254814 71301168 ENSG00000147140 + 947
X 71267253 71269614 Homo_sapiens_X_71266645-71268493-HERVHF-pol + X
71254814 71301168 ENSG00000147140 + 2361
X 71271762 71272124 Homo_sapiens_X_71266645-71268493-HERVHF-env + X
71254814 71301168 ENSG00000147140 + 362
X 71272292 71272628 Homo_sapiens_X_71266645-71268493-HERVHF-3LTR + X
71254814 71301168 ENSG00000147140 + 336
X 71266244 71266657 Homo_sapiens_X_71266645-71268493-HERVHF-gag + X
71266029 71301160 ENSG00000147140 + 413
X 71266704 71267651 Homo_sapiens_X_71266645-71268493-HERVHF-pro + X
71266029 71301160 ENSG00000147140 + 947
X 71267253 71269614 Homo_sapiens_X_71266645-71268493-HERVHF-pol + X

71266029 71301160 ENSG00000147140 + 2361
X 71271762 71272124 Homo_sapiens_X_71266645-71268493-HERVHF-env + X
71266029 71301160 ENSG00000147140 + 362
X 71272292 71272628 Homo_sapiens_X_71266645-71268493-HERVHF-3LTR + X
71266029 71301160 ENSG00000147140 + 336
X 94698818 94699270 Homo_sapiens_X_94700559-94702091-HERVHF-5LTR - X
94675774 94702415 NONHSAG054922.2 - 452
X 94698818 94699270 Homo_sapiens_X_94700559-94702091-HERVHF-5LTR - X
94675774 94702415 NONHSAT137842.2 - 452
X 94699849 94701688 Homo_sapiens_X_94700559-94702091-HERVHF-pol - X
94675774 94702415 NONHSAG054922.2 - 1839
X 94699849 94701688 Homo_sapiens_X_94700559-94702091-HERVHF-pol - X
94675774 94702415 NONHSAT137842.2 - 1839
X 94701248 94701832 Homo_sapiens_X_94700559-94702091-HERVHF-pro - X
94675774 94702415 NONHSAG054922.2 - 584
X 94701248 94701832 Homo_sapiens_X_94700559-94702091-HERVHF-pro - X
94675774 94702415 NONHSAT137842.2 - 584
X 111543806 111549675 Homo_sapiens_X_111546380-111547978-HERVHF + X
111511674 111621496 NONHSAG055109.3 + 5869
X 111543806 111544260 Homo_sapiens_X_111546380-111547978-HERVHF-5LTR +
X 111511674 111621496 NONHSAG055109.3 + 454
X 111546072 111546392 Homo_sapiens_X_111546380-111547978-HERVHF-gag + X
111511674 111621496 NONHSAG055109.3 + 320
X 111546382 111547230 Homo_sapiens_X_111546380-111547978-HERVHF-pro + X
111511674 111621496 NONHSAG055109.3 + 848
X 111546688 111548769 Homo_sapiens_X_111546380-111547978-HERVHF-pol + X
111511674 111621496 NONHSAG055109.3 + 2081
X 111549221 111549675 Homo_sapiens_X_111546380-111547978-HERVHF-3LTR +
X 111511674 111621496 NONHSAG055109.3 + 454
X 111543806 111549675 Homo_sapiens_X_111546380-111547978-HERVHF + X
111518074 111621496 NONHSAT138183.2 + 5869
X 111543806 111544260 Homo_sapiens_X_111546380-111547978-HERVHF-5LTR +
X 111518074 111621496 NONHSAT138183.2 + 454
X 111546072 111546392 Homo_sapiens_X_111546380-111547978-HERVHF-gag + X
111518074 111621496 NONHSAT138183.2 + 320
X 111546382 111547230 Homo_sapiens_X_111546380-111547978-HERVHF-pro + X
111518074 111621496 NONHSAT138183.2 + 848
X 111546688 111548769 Homo_sapiens_X_111546380-111547978-HERVHF-pol + X
111518074 111621496 NONHSAT138183.2 + 2081
X 111549221 111549675 Homo_sapiens_X_111546380-111547978-HERVHF-3LTR +
X 111518074 111621496 NONHSAT138183.2 + 454
X 122227333 122227787 Homo_sapiens_X_122224556-122226109-HERVHF-3LTR +
X 122227177 122248649 NONHSAG055239.2 + 454
X 122227333 122227787 Homo_sapiens_X_122224556-122226109-HERVHF-3LTR +

X	122227177	122248649	NONHSAT138411.2+	454
